# Obscured phylogeny and possible recombinational dormancy in *Escherichia coli*

**DOI:** 10.1186/1471-2148-11-183

**Published:** 2011-06-27

**Authors:** Shana R Leopold, Stanley A Sawyer, Thomas S Whittam, Phillip I Tarr

**Affiliations:** 1 Department of Pediatrics, Washington University School of Medicine, Saint Louis, USA; 2 Molecular Microbiology and Microbial Pathogenesis Program, Washington University School of Medicine, Saint Louis, USA; 3 Institute of Hygiene, University of Muenster, Muenster, Germany; 4 Department of Mathematics, Washington University, St. Louis, USA; 5 Microbial Evolution Laboratory, National Food Safety and Toxicology Center, Michigan State University, East Lansing, USA

**Keywords:** Definition of Species, Phylogeny, Recombination

## Abstract

**Background:**

*Escherichia coli *is one of the best studied organisms in all of biology, but its phylogenetic structure has been difficult to resolve with current data and analytical techniques. We analyzed single nucleotide polymorphisms in chromosomes of representative strains to reconstruct the topology of its emergence.

**Results:**

The phylogeny of *E. coli *varies according to the segment of chromosome analyzed. Recombination between extant *E. coli *groups is largely limited to only three intergroup pairings.

**Conclusions:**

Segment-dependent phylogenies most likely are legacies of a complex recombination history. However, *E. coli *are now in an epoch in which they no longer broadly share DNA. Using the definition of species as organisms that freely exchange genetic material, this recombinational dormancy could reflect either the end of *E. coli *as a species, or herald the coalescence of *E. coli *groups into new species.

## Background

For many years, our understanding of the phylogeny of *Escherichia coli*, a diverse group of pathogenic and commensal organisms, has been based on multilocus enzyme electrophoresis (MLEE) [1] patterns of the strains in the *E. coli *Reference Collection (ECOR) [2]. MLEE demonstrated subspecific clonal structure within *E. coli *[3,4] and formed the basis for parsing this collection (and by extension the broader species) into one minor (E) and four major (A, B1, B2, D) groups. These divisions often correlate with pathogenicity or niche [2].

Multilocus sequence typing, which uses allelic variations in a sample of housekeeping genes distributed around the chromosome, and whole genome sequencing have been increasingly used to study *E. coli *phylogeny. However, these circumchromosomal sequence datasets generate incongruent phylogenetic topologies. For example, MLST frequently identifies Group B2 as being the first to branch from the phylogenetic tree, and Groups A and B1 as 'sister' groups that branch most recently. In contrast, MLEE places Groups B2 and B1 in a proximal branching position and Group A branches more distally [5]. Single gene phylogenies also fail to converge on a single topology [6], place either Group D or B2 as being the first to branch, and usually do not result in monophyletic groups [7-12]. MLST additionally demonstrates paraphyly for Groups A or B1 in some analyses [13,14], or portrays strains as hybrids [15]. Different relationships can be generated from MLST data by varying the choice of outgroup, the stringency of recombination detection [16], or the phylogenetic methodology [15,16]. A thorough analysis of the core genomes of 1,878 genes in 20 *E. coli *strains indicate an early bifurcation of *E. coli *into Group B2 and a Group D subgroup on one fork, and a second subgroup of Group D and all other strains on the other, inferring paraphyly within Group D [17]. Gordon, et al [18] apply several different but unrooted MLST analyses to a large number of *E. coli*. Their unrooted analysis cannot illuminate the order of emergence, but provides multiple different portrayals of Group relatedness. These disparate approaches have failed to resolve the topology of emergence of this species.

We attempted to produce a more cogent picture of the emergence of *E. coli *by studying backbone DNA. Backbone (also termed K-loop) DNA [19] was initially defined as the regions of the chromosome of one of the first sequenced *E. coli *O157:H7 strain EDL933 that are homologous with the non-pathogenic laboratory strain K-12, thus by definition lacking pathogenicity islands and mobile elements such as prophages [20]. We selected four extended length (ca. 25 kb) backbone segments in four different quadrants of the chromosome in strains belonging to different ECOR Groups (See Additional File 1, Table S1). We chose this strategy for three reasons: First, backbone DNA is relatively uncontaminated by horizontally acquired DNA such as pathogenicity islands (encoding virulence factors), which could have evolutionary histories quite independent of their host bacteria [21]. Second, long segments of nucleotides are more likely to generate bootstrap confidence values for node placement that are higher than those produced by more limited datasets (i.e., MLST or single gene phylogenies). Third, the separation of the studied segments provides information relevant to the overall phylogenetic topology of the species.

## Results

### Phylogenetic topology of *E. coli*

**Figure 1:**
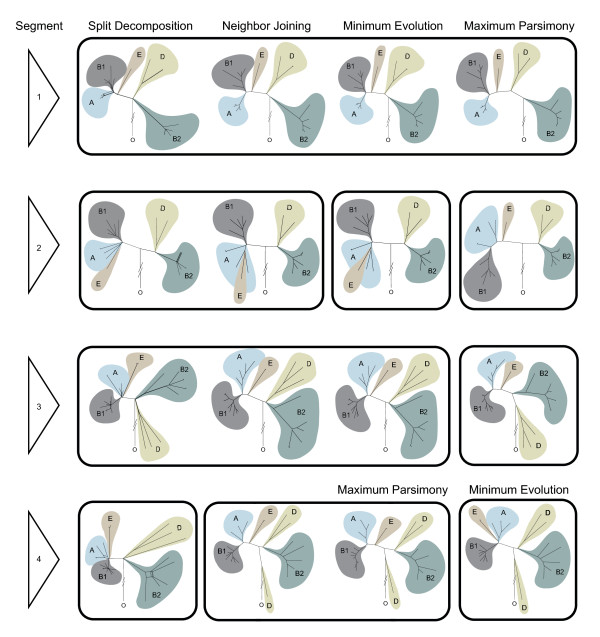
**Phylogenetic Topologies**. Various phylogenetic topologies are assigned to Segments 1, 2, 3, and 4 (rows) by SD, NJ, ME, and MP methods (columns). Congruent topologies are displayed within conjoined panels. 'O' represents the outgroup, *E. albertii*. The Segment 4 ME and MP portrayals are switched to demonstrate topologic congruency between adjacent panels.

In most topologies (Figure 1, see Additional File 2, Figure S1), SD, NJ, ME, and MP phylogenetic techniques recapitulated the major groupings of *E. coli *as have been defined by MLEE and MLST. However, in some portrayals, Group E appears as an offshoot of Group A (Additional File 2, Figure S1 Panels E, F, H) or Group D is paraphyletic (Additional File 2, Figure S1 Panels N, O, P). For Segment 1, all four methods produce a single congruent topology (topologies in which major branch points are in identical relative positions are considered congruent) (Figure 1, Row 1, Additional File 2, Figure S1 A, B, C, D). For Segments 2, 3, and 4, two or three different, i.e., incongruent, topologies emerged (Figure 1 Rows 2-4, Additional File 2, Figure S1 E, F, G, H, I, J, K, L, M, N, O, P). Most notably, we found no congruencies between the topologies ordained by the same phylogenetic methods when these analyses were applied to different Segments (Figure 1). The confidence bootstrap values (Additional File 2, Figure S1) of these phylogenies cover a spectrum of magnitude (as do their variances from congruency), but generally exceed those produced by MLST [22-25].

The choice of segment influenced the inferred topology to a greater extent than did the method used to construct the phylogeny. This is surprising, because phylogeny should be a property of organisms, and not vary as a function of the DNA segment scrutinized. Most likely, circumchromosomal datasets produce net topologies weighted by the differing evolutionary and recombination histories of components of the chromosome. In other words, the phylogenetic history of *E. coli *becomes less clear as more sequence data are entered into analysis.

### Inter-Group recombination

**Figure 2:**
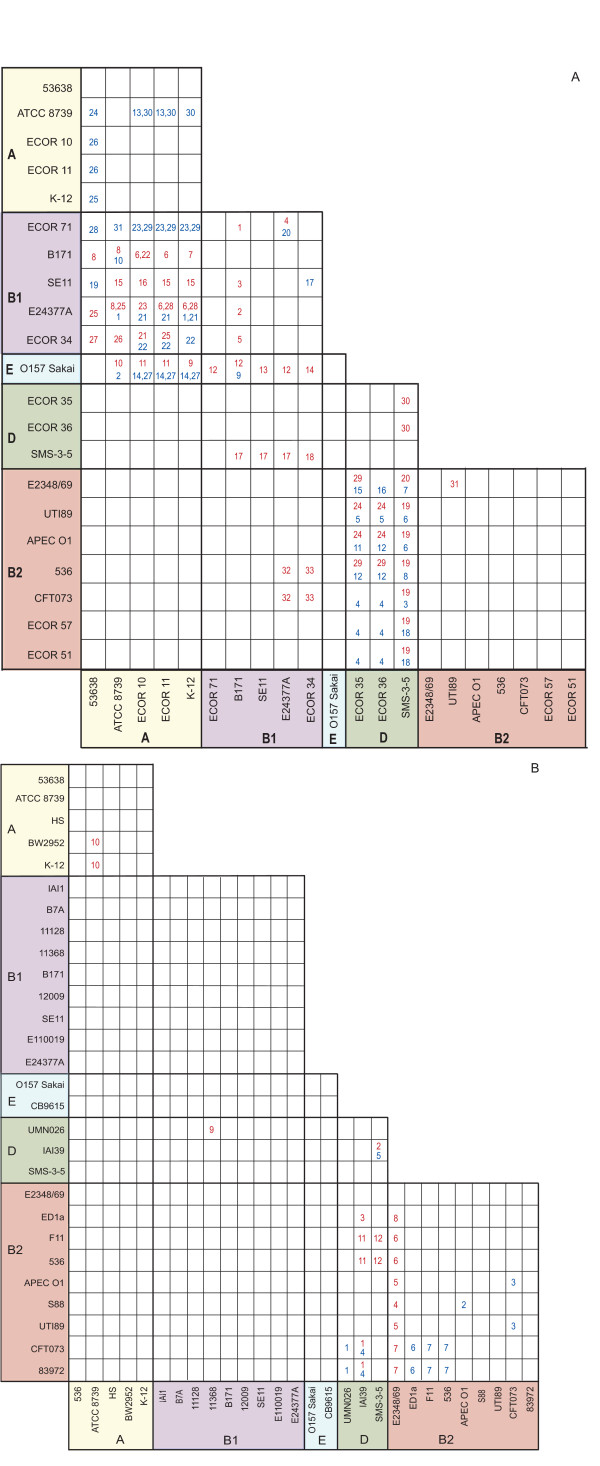
**Recombination between groups**. Strains studied in Segments 1 and 2 (Panel A) or Segment 3 and 4 (Panel B) analyses are listed along the x- and y-axes, assembled as groups. Red (Segment 1 and Segment 3) and blue (Segment 2 and Segment 4) numbers within boxes correspond to fragments identified by GENECONV as having been transferred by recombination. The Fragments from Segments 1, 2, 3, and 4 are portrayed in Figures S2A, S2B, S2C, and S2D, respectively (Additional File 3).

Next, we used GENECONV [26], a program that compares orthologous DNA and identifies regions that have been acquired by recombination, to identify among the four extended segments a total of 112 inter-group exchanges (Figure 2). Of these 112 exchanges, 41 were 'duplicates', where two or more regions identified by GENECONV had identical borders. Such conversions probably represent transfer of DNA from a single strain in one Group to a single strain in another Group before lineages diverged in the recipient Group. Of the remaining 71 converted segments, 70 overlapped partially with at least one other exchanged fragment (see Additional File 3, Figure S2).

**Figure 3:**
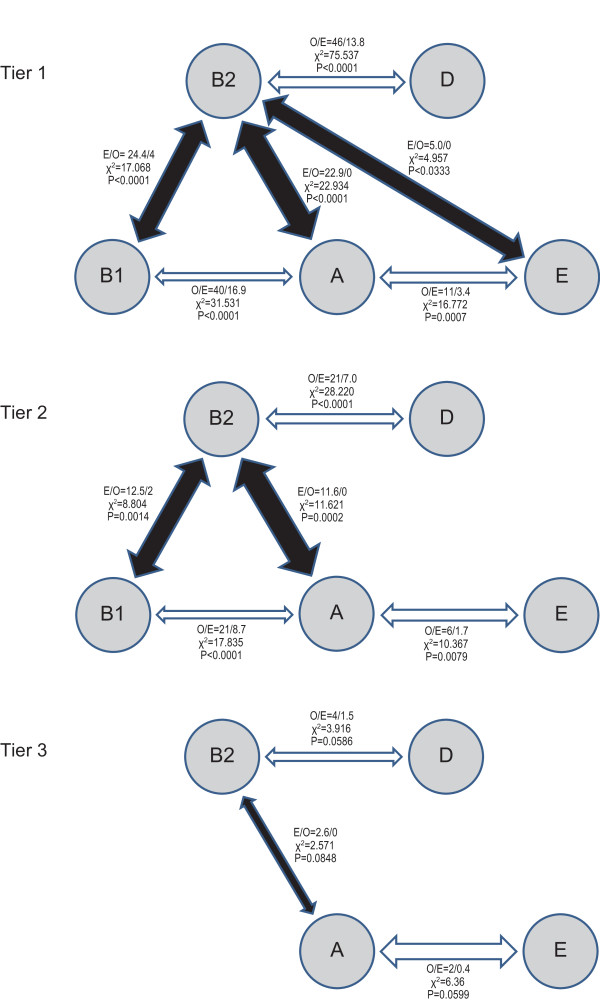
**Inter-group conversions, portrayed by tiers**. Groups are portrayed in white circles. Bidirectional arrows between groups reflect over- (white) or under- (black) represented conversions, if p-values are < 0.05 (Tiers 1 and 2) or < 0.10 (Tier 3). Each white arrow is proportional to its observed:expected ratio. Each black arrow is proportional to the expected:observed ratio, but expected values of 0 are assigned an arbitrary value of 1 and expected:observed thickness arrows are capped at thickness ratios of 7.5:1. Adjacent to arrows are observed and expected conversions, chi squared, and p values. Further details regarding expected and observed inter-group conversions are in Table S2 (Additional File 4).

We used three increasingly stringent tiers of analysis to determine if the exchanges between Groups occurred randomly (portrayed in Figure 3, see Additional File 4, Table S2). For Tier 1, we considered all 112 exchanges as independent events, and identified disproportionate (over-represented) conversions between Groups B2 and D, A and B1 (both p < 0.0001), and A and E (p < 0.001). For Tier 2, we assigned duplicate conversions of fragments with identical borders as single events, and again found statistically significant non-random associations between Groups B2 and D, A and B1 (both p < 0.0001), and A and E (p < 0.01) for the 57 such non-duplicated inter-group exchanges. For Tier 3, we counted any and all inter-group recombination events once and only once for any segment, because most recombined fragments overlap to some extent. Among the 13 such occurrences, the four B2/D and two A/E pairings were overrepresented (both p = 0.06). These different conversion enumeration strategies each suggest that DNA exchange was restricted to a subset of all possible pairings.

Intra-group recombination was more frequent than inter-group exchange. Among the 258 intra-group and 772 inter-group strain to strain opportunities for pairings, GENECONV identified 40 (expected 34), 26 (expected 18), and 10 (expected 5) intra-group and 95 (expected 101), 47 (expected 55), and 9 (expected 14) inter-group recombination events for tier 1, 2, and 3 exchanges, respectively. The chi square and two-tailed approximate P values for tier 1, 2, and 3 inter- vs. intra-group comparisons are 1.415 (P = 0.23), 4.719 (P = 0.03) and 6.786 (P = 0.009), respectively.

## Discussion

Our data prompt two questions: First, how can the robust recombination that occurred in *E. coli*'s distant past [27] be reconciled with the restricted recombination among extant groups? Second, can the restricted recombination that we demonstrated across four segments from different quadrants of the chromosome be harmonized with the concept that members of the same species readily exchange DNA [28]? In answer to these questions, we propose that for much of its existence, *E. coli *exchanged DNA freely between groups as evidenced by its convoluted phylogeny. However, as the five lineages that formed each phylogroup continued to evolve and differentiate, their abilities to receive and/or to donate DNA diminished, and the exchange of chromosomal DNA among extant *E. coli *is now largely dormant. Mechanisms for exchange restriction might include limited opportunities for interactions between groups as their members occupy particular niches (mostly in animals and humans) or organism-specific factors (e.g., phage receptors, differing DNA restriction or DNA mismatch repair capacities) hinder conversions.

The durability of the nonrandom exchange of DNA between groups could determine the fate of *E. coli*. If these conversion patterns become increasingly restricted, there could be involution of the ability to exchange chromosomal DNA across the entire species. Alternatively, if these networks are durable, Groups B2 and D, and possibly Groups A, B1, and E, could now be coalescing, (i.e., converging through recombination) as nascent species, in which case inter-group recombination will persist for these sets. This latter scenario would resemble the early fragmentation of an ancestral species into *E. coli *and *Salmonella *[29]. However, recombinational dormancy is only one explanation for our findings, and confirmation or refutation will require larger datasets, using, as we note above, optimally representative strain sets.

The disproportionately high intra-group recombination rates strengthen the case for highly restricted recombination networks between sets of organisms, as suggested by other investigators. For example, the patterns in Figure 3 resemble gene-sharing "highways" [30] between distantly related bacteria, and our intra-species analysis suggests this process applies within *E. coli*. Such networking also appears among penicillin resistant pneumococci [31].

The appropriateness of defining bacterial species based on net DNA homology has been questioned [32-34], but there remains concurrence that members of a species should exchange DNA [28,35]. Statistical comparisons of open reading frames suggest a recent and unexpected slowing of DNA exchange between enteric bacteria belonging to different species [36]. Our findings now raise the possibility that DNA exchange is also recently constrained within a single species, i.e., *E. coli*, a taxonomic rank that should, according to Mayrian theory [28], tolerate recombination.

Our study has several limitations. It is possible that the predominantly human origin of our strain set introduced biases. However, isolation of the *E. coli *from humans does not mean that these organisms are adapted to humans. Specifically, urinary and meningitis isolates occupied bladder, kidney, or meningeal niches only briefly before they were recovered, and their prior venues are unknown. *E. coli *O157:H7 infection of humans is incidental and quite ephemeral; these human enteric pathogens are much better adapted to the ruminant gut. Microbial phylogenetic studies should ideally use minimally biased, globally representative strain samples. However, until such a sample set is assembled, we remain reliant on strain sets of variable convenience, and must acknowledge that they might produce misleading interpretations regarding microbial membership in a given niche. Also, fragmentation of *E. coli *into limited recombination networks could be related to the recent entry into the species niche from which they were recovered. Such entry is soon followed by clonal expansion, and increased opportunities for recombination with other occupants, but the surge does not reflect evolutionarily-driven emergence. Indeed, Walk, et al [37], used MLST (22 loci) to study *E. coli *with "noticeably divergent sequences" and most of their phylogenetic outliers were from non-human sources. This finding lends credence to the possibility that recent expansion in human niches leads to recovery opportunity and biases, and that human-based strains might offer an incomplete picture of the broader species. However, this study of largely environmental strains could also reflect strain selection biases if they comprised only a small minority of non-human isolates in the collection. We also acknowledge that the segments chosen might be at variance from the true evolutionary history of the chromosome. However, the inclusion criteria balanced our need to select segments that were of sufficient length to identify recombination, that were widely separated on the chromosome thereby providing validity to and generalizing our findings, and that were not abundantly interrupted by non-backbone DNA, which might have introduced pro-recombinational biases. Moreover, the analysis of Segments 3 and 4 (using a somewhat different strain set) validated the data from initial data Segments 1 and 2. These measures reduce the likelihood that we inadvertently introduced a bias for certain kinds of genes that are more or less likely to have undergone recombination [27]. Additionally, it is likely that all species are gradually radiating, but our data raise the possibility that extant *E. coli*, after evolving from a set of organisms that were a species (as traditionally defined), are at or near a point where we might consider their coalescence into new species. An additional caveat when considering our findings is that we purposely focused on the core (backbone) genome, and did not enter into analysis DNA that had been clearly acquired by lateral gene transfer. It is known that such horizontally transferred DNA is readily exchanged between organisms that are quite distantly related, even belonging to different species, and we wished to retain focus on the stable portion of the chromosome. We had hoped to resolve differences in phylogeny by the reductionist approach of extended length segment analysis, but the variably discordant phylogenies suggest to us that at least at present the problem cannot be solved: the *E. coli *chromosome "chassis" has parts with too many origins to assign the emergence scenario of the whole with confidence. Our data do prompt us to propose that future phylogenetic analyses address disproportionate contributions from recently acquired, or very long, segments of chromosomes.

## Conclusion

It is currently problematic to use circumchromosomal sequence data to develop an unambiguous emergence topology for *E. coli*. Most likely, *E. coli*'s legacy of recombination [38] hinders such attempts to discern a cogent phylogeny, as predicted two decades ago by Dykhuizen and Green [6]. It will be tempting to use whole genome sequences to construct phylogenies of other microbes, but our findings from *E. coli *suggest that depending on the phylogenetic questions asked, there are optimal sizes of datasets to provide the answers. Indeed, more (i.e., total genomic) sequence might, counter-intuitively, offer less clarity in trying to discern species topology. *E. coli *might now be in an epoch of recombinational dormancy. The few non-random conversion patterns we identified could represent new species emerging, or, alternatively, vestigial recombination capabilities between existing groups, if the ability to exchange DNA is slowing, among the set of organisms we know as *E. coli*.

## Methods

### Strains

For our initial strain set, we selected 16 strains from ECOR groups A, B1, D, and B2, five fully sequenced *E. coli *deposited in GenBank as of 18 August 2006, nine additional *E. coli *in GenBank as of 4 February 2009, and *Escherichia albertii *(see Additional File 1, Table S1). In our validation strain set, we chose 28 strains in GenBank as of 29 March 2010 (Additional File 1, Table S1) that had extensive (>95%) alignment between Segments 3 and 4. We limited the analysis of Group E strains because there is negligible recombination of backbone DNA between members of this clade [39].

### Choosing, Validating, and sequencing Extended Segments

We used a subset of *E. coli *genomes (strains K-12, CFT073, UTI89, O157 Sakai, and EDL933 [19,40]) at the outset of the project for segment selection purposes. Then, we identified the conserved backbone regions that were at least 25 kb in length, and uninterrupted by O-islands. Two regions that were 25 kb in length in two different quadrants of the chromosome were selected for further analysis: 1,084,426 - 1,109,426 (Segment 1) and 2,368,611 - 2,393,611 (Segment 2) (position numbers based on nucleotide sites in the O157 Sakai chromosome) [19]. For the purposes of this study, these genes met a functional definition of backbone, as chromosomal loci common to all sequenced *E. coli *at the time we needed to choose a data set for analysis. However, it is possible that a subset of these open reading frames might not be found in subsequently sequenced strains. We then performed long range PCR across three overlapping sections of each 25 kb segment in a set of pilot ECOR strains (Additional File 1, Table S1) to ensure that these segments were likely to be intact and uninterrupted across the species.

Segments 1 and 2 were sequenced (from nucleotide positions 1,084,356 to 1,110,604 and 2,368,707 to 2,393,879, respectively) in eight ECOR strains (two each from groups A, B1, B2, and D) (Additional File 1, Table S1) based on uniform restriction patterns in these segments in these pilot strains. Orthologous sequences from 13 published *E. coli *strains (including four of the initial five-strain dataset) as well as *E. albertii *(outgroup) (Additional File 1, Table S1) were retrieved from the NCBI database using BLASTn [41], then aligned to Segments 1 and 2 of the ECOR strains. We analyzed only the nucleotides of Segments 1 and 2 that were represented in all 21 strains by concatenating these common sequences into two respective contigs for each strain (Segment 1 = 23,237 bp, Segment 2 = 23,394 bp), and then aligning them using ClustalW [42]. Validation studies used Segments 3 (3,633,818 - 3,658,818) and 4 (4,754,067 - 4,779,067), and the same alignment techniques used for Segments 1 and 2. Primers were designed to amplify ~500 bp overlapping segments of the genome in Segments 1 and 2 in eight ECOR strains (Additional File 1, Table S1). DNA was prepared by phenol chloroform extraction and ethanol precipitation, and each amplicon was Sanger sequenced.

Sequenced amplicons for each strain were assembled into contigs using the SeqMan Pro program (Lasergene v.3 DNASTAR software suite). Regions that failed to amplify and multi-nucleotide insertions or deletions were not included in the final concatenated assembly. Single nucleotide indels and SNPs occurring in only one strain were verified by visualizing the original trace data. The sequences from the amplicons that were successfully sequenced in every strain and for which there was orthologous sequence in the published genomes were concatenated using Lasergene's EditSeq program and aligned by ClustalW in Molecular Evolutionary Genetics Analysis (MEGA) software v.4.0 [43]. All analyzed sequences are provided in Table S3 (see Additional File 5), as aligned by SeaView (version 4.2.11) [44]. We chose to use *E. albertii *as an outgroup in all analyses, because, unlike *Salmonella*, it is considered a member of the *E. coli *species, and has considerably more Segment 2 orthologous sequence *E. coli *than *E. fergusonii *and evolved less rapidly (thereby diminishing the risk of long branch attraction) [37]. The ClustalW alignment of all strains (except *E. albertii*) (see Additional File 3, Figure S2) was analyzed for evidence of sequence acquired by recombination using GENECONV [26] with command-line parameter gscale = 1. Regions of sequence identified as being affected by recombination were replaced by "---". An α of 0.05 was considered statistically significant.

We constructed phylogenetic models using Neighbor Joining (NJ), Minimum Evolution (ME) and Maximum Parsimony (MP) analyses in MEGA v.4.0 software [43]. Phylogenetic analysis was performed by using Kimura-2-parameter (for NJ and ME), and complete-deletion for all trees. Bootstrapping was performed with 1,000 replicates. Split Decomposition (SD) network analysis was performed using SplitsTree v.4.10 [45].

### Statistics

We used the Pearson chi-square statistic in a permutation-like simulation test to determine the statistical significance of the differences between observed and expected inter-group recombination frequencies. For expected counts, we assume that each of the 166 (Segments 1 and 2) or 292 (Segments 3 and 4) inter-stain pairings is equally likely to be involved in a gene conversion. The relative probability of a between-group gene conversion for each segment is proportional to the number of strains in the corresponding groups. Expected and simulated counts are conditional on the total number of observed counts in segments, and observed and expected numbers are summed over segments for each pair of groups. For example, if there are 10, 20, 30, and 40 total inter-group conversions in the four segments, respectively, and if Group X has five studied strains and Group Y has six studied strains for Segments 1 and 2 and four and five respectively on Segments 3 and 4, then there would be (10+20) × (5 × 6)/166 + (30+40) × (4 × 5)/292 expected gene conversions between Groups X and Y. The Pearson chi-square statistic, which is a higher-dimensional analog of the Cochran-Mantel-Haenszel (CMH) test statistic [46] (a standard way to estimate p-values for stratified data) is summed over pairs of groups. p-values were estimated by a simulation procedure due to the large number of empty cells. The test score for the observed counts was compared with the same test score for 10^6 ^simulated count sets. In each simulation, the observed recombination events for each segment were randomly reassigned to pairs of groups according to the expected probabilities for that segment, specifically by simulating the values of a multinomial distribution for each segment. The simulated counts were summed across the four segments and the Pearson test score recomputed. The p-value for biases between-group recombination rates across segments is estimated as the proportion of simulations for which the randomized test score was greater than or equal to the observed test score.

The chi-square test was used to test the significance of the observed difference in inter- and intra-group recombination frequency. The total observed recombination events and possible recombination opportunities (inter-group and intra-group) were enumerated for each tier in each of the two categories. Group E was not included in the analysis because of the paucity of group E strains studied, as noted above.

## Authors' contributions

SRL designed analytical strategies, selected strains, performed most analyses, and wrote the text. SAS proposed mathematical techniques, and performed statistical modeling. TSW helped SRL formulate the hypothesis that phylogeny as derived from multilocus sequence typing was inaccurate. PIT reviewed and approved analytical strategies, interpreted the data, assisted in writing of the manuscript, and obtained funding for this project. TSW died before this manuscript was submitted; all other authors read and approved the final manuscript.

## Supplementary Material

Additional file 1 **Table S1**. Strains Used. Click here for file

Additional file 2 **Figure S1**. Topologies generated by various analyses from each Segment. Click here for file

Additional file 3 **Figure S2**. Fragments identified as being subjected to conversion. Click here for file

Additional file 4 **Table S2**. Conversion events identified by GENECONV. Click here for file

Additional file 5 **Table S3**. Sequence alignment. Click here for file
