# Supplementary material for: Obscured phylogeny and possible recombinational dormancy in Escherichia coli
Source: BMC Evol Biol. 2011 Jun 27;11:183. doi: 10.1186/1471-2148-11-183 (PMC3152902; doi:10.1186/1471-2148-11-183)
Supplement: Additional file 1 — Table S1. Strains Used. [file 1471-2148-11-183-S1.PDF]

A: L3E
B1: L3E
E: L2
D (All Strains): L1
B2: R1S

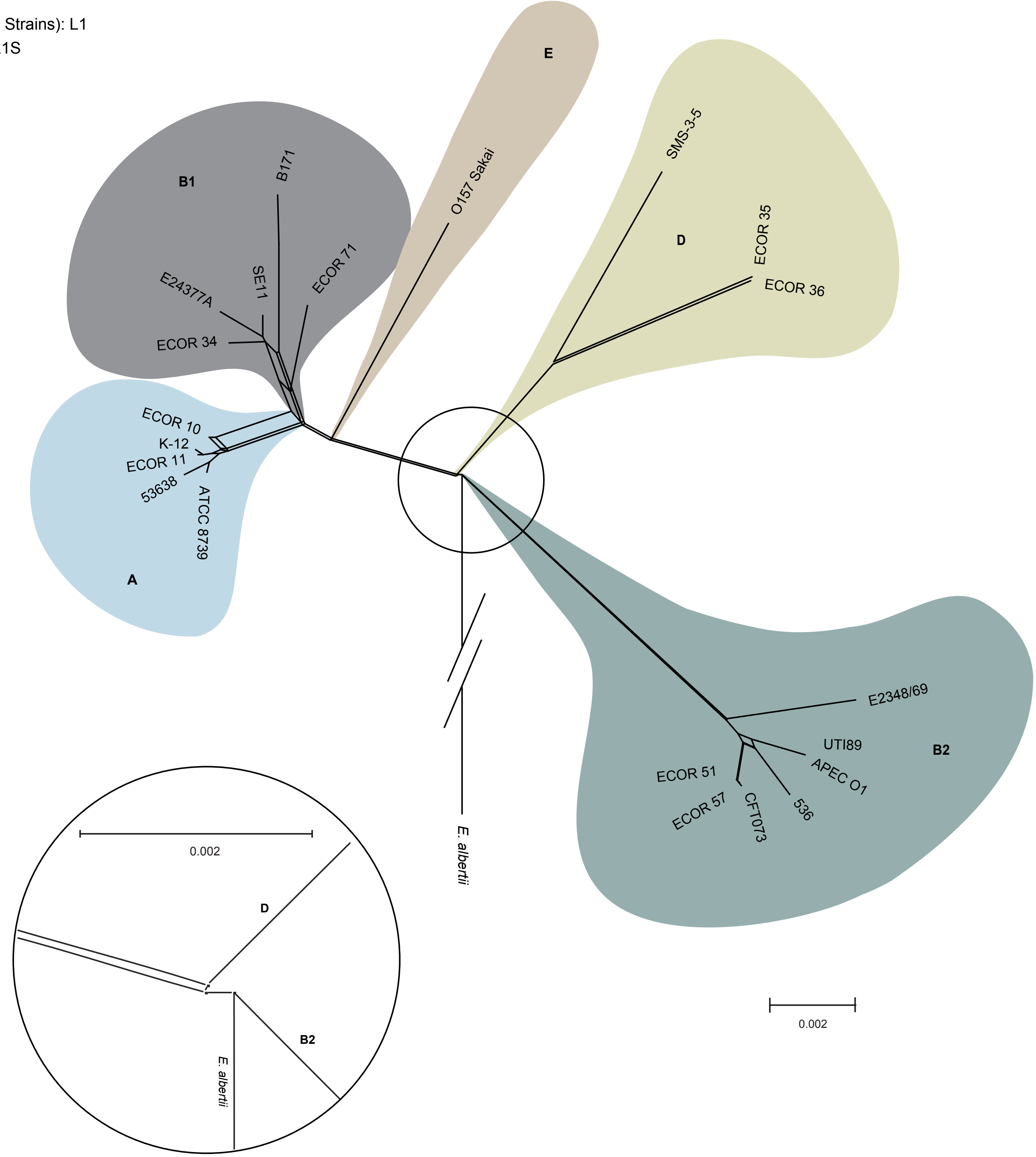

Figure S1. Panel A. Segment 1, Split Decomposition
Topologies generated by various analyses from each Segment. Individual topologies for Segment 1 (Panels A-D), 2 (Panels E-H), 3 (Panels I-L), and 4 (Panels M-P). Topologies are generated by SD (Panels A, E, I, and M), NJ (Panels B, F, J, and N), MP (Panels C, G, K, and O), and ME (Panels D, H, L, and P) phylogenetic analyses. For each node on the main branch, bootstrap confidence values are shown as numbers (except for SD portrayals). Nodes without numbers or colored dots have confidence values of 100. Color codes for all other nodes are as indicated in each panel. Each group divergence event and bifurcation in the various topologies is assigned an alpha (event descriptor) numeric (sequence of occurrence of event after species founding) and is listed in the upper left hand corner of each figure panel. In this notation, events that occur to the right or left of the founding node are prefixed by "R" or "L", respectively. E = External node, O = Offshoot from another group, S = Solitary side group, EPN = Early Paraphyletic Node.

A: L3E
B1: L3E
E: L2
D (All strains): L1
B2: R1S

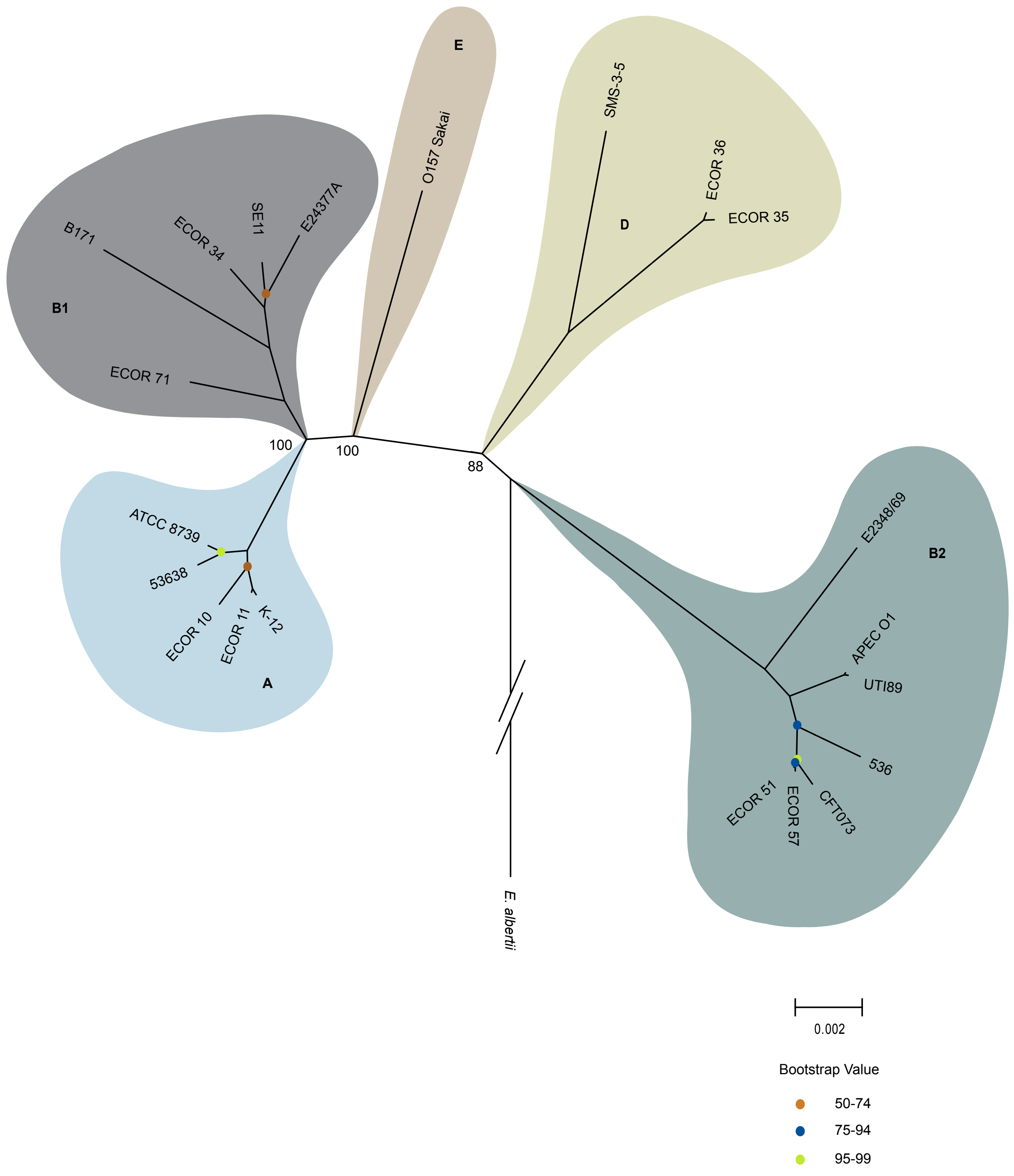

Figure S1. Panel B. Segment 1, Neighbor Joining

A: L3E
B1: L3E
E: L2
D (All strains): L1
B2: R1S

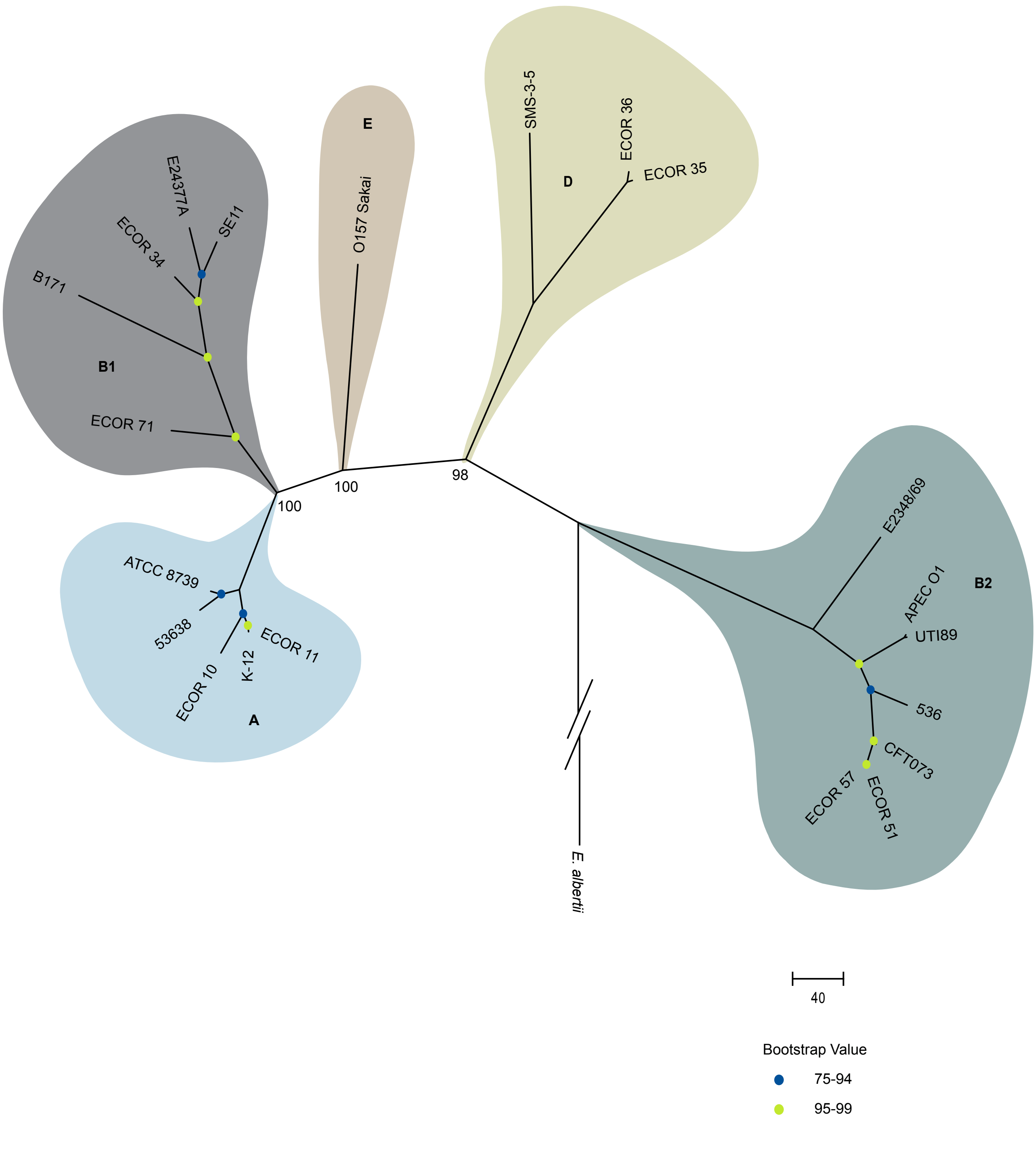

Figure S1. Panel C. Segment 1, Maximum Parsimony

A: L3E
B1: L3E
E: L2
D (All strains): L1
B2: R1S

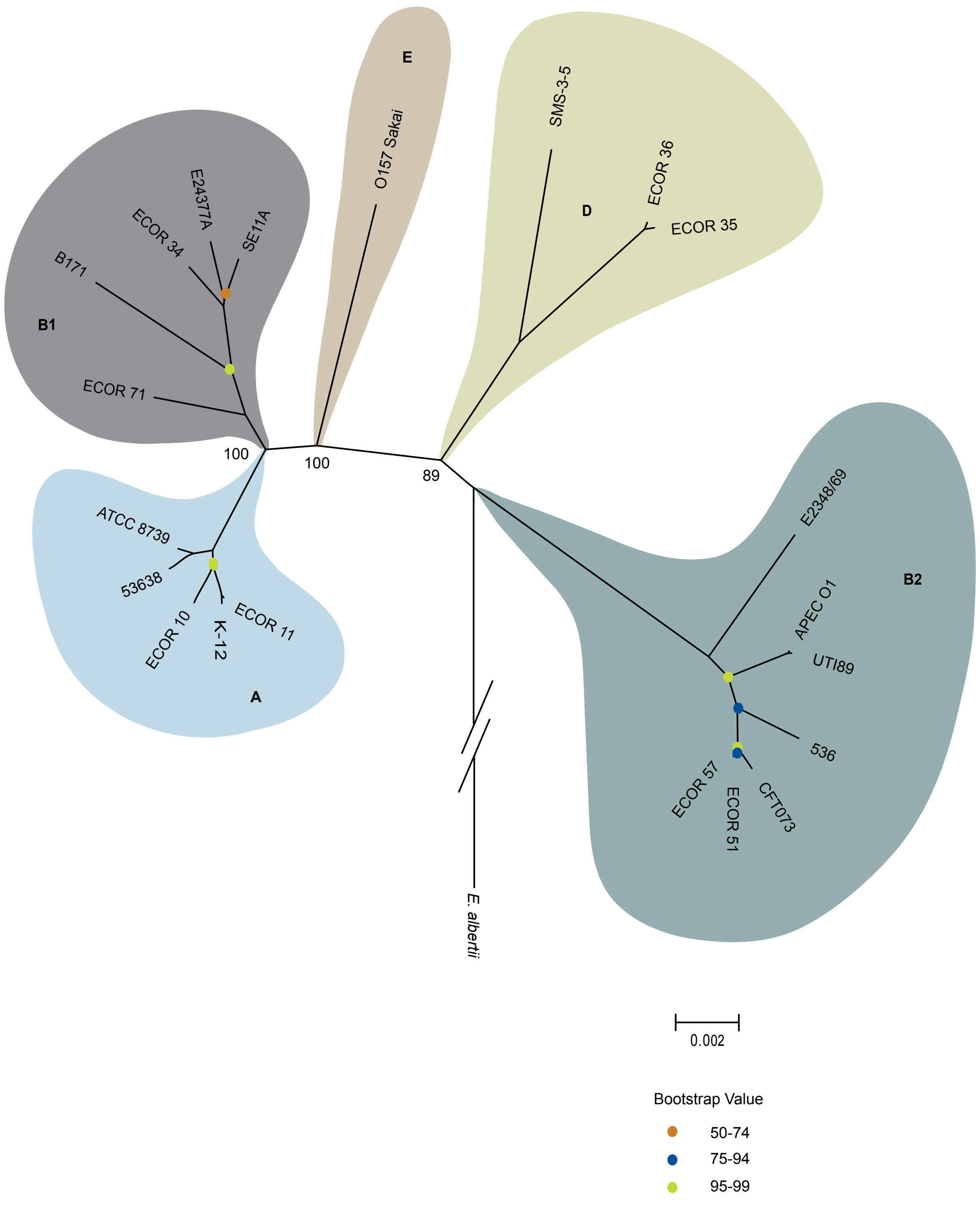

Figure S1. Panel D. Segment 1, Minimum Evolution

A: L1E
B1: L1E
E: OL1E
D (All strains): R1E
B2: R1E

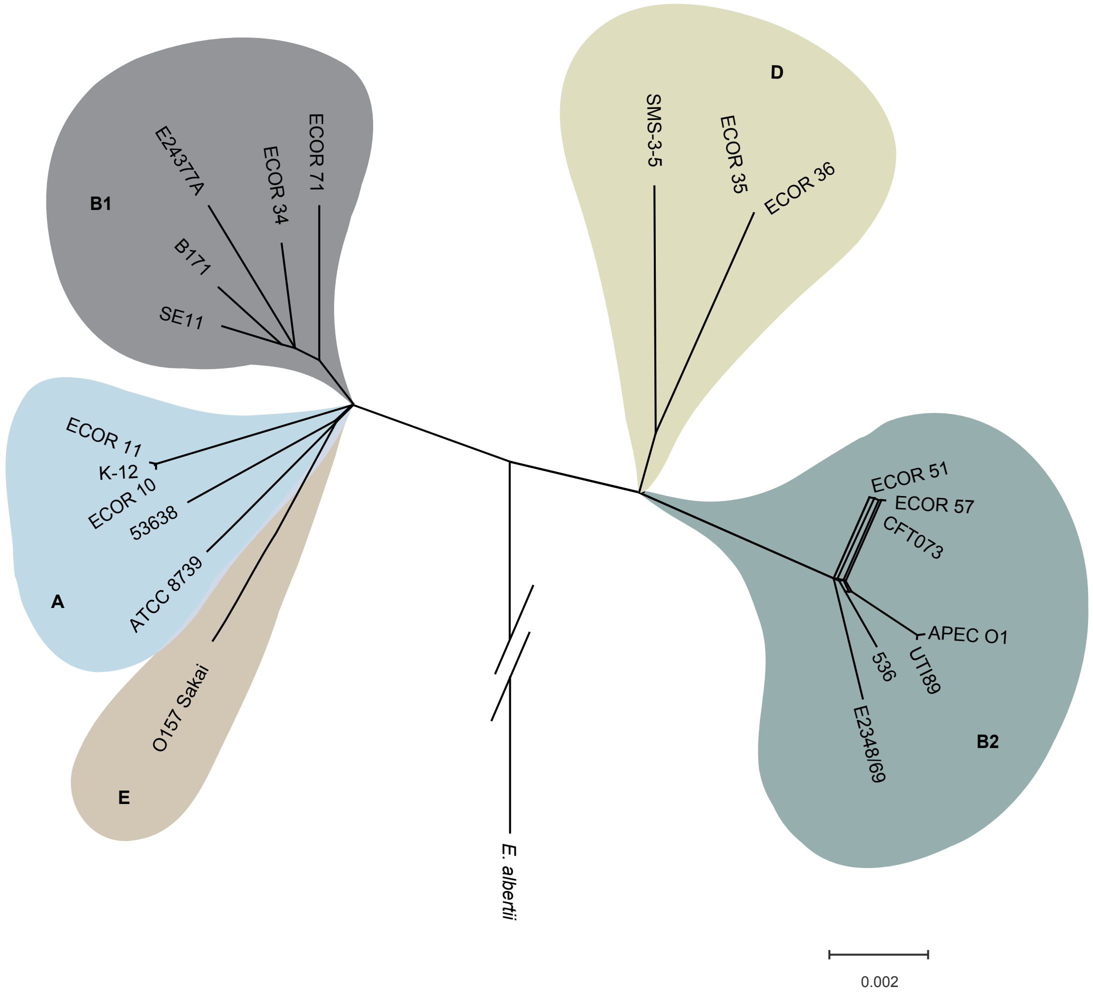

Figure S1. Panel E. Segment 2, Split Decomposition

A: L1E
B1: L1E
E: OL1E
D (All strains): R1E
B2: R1E

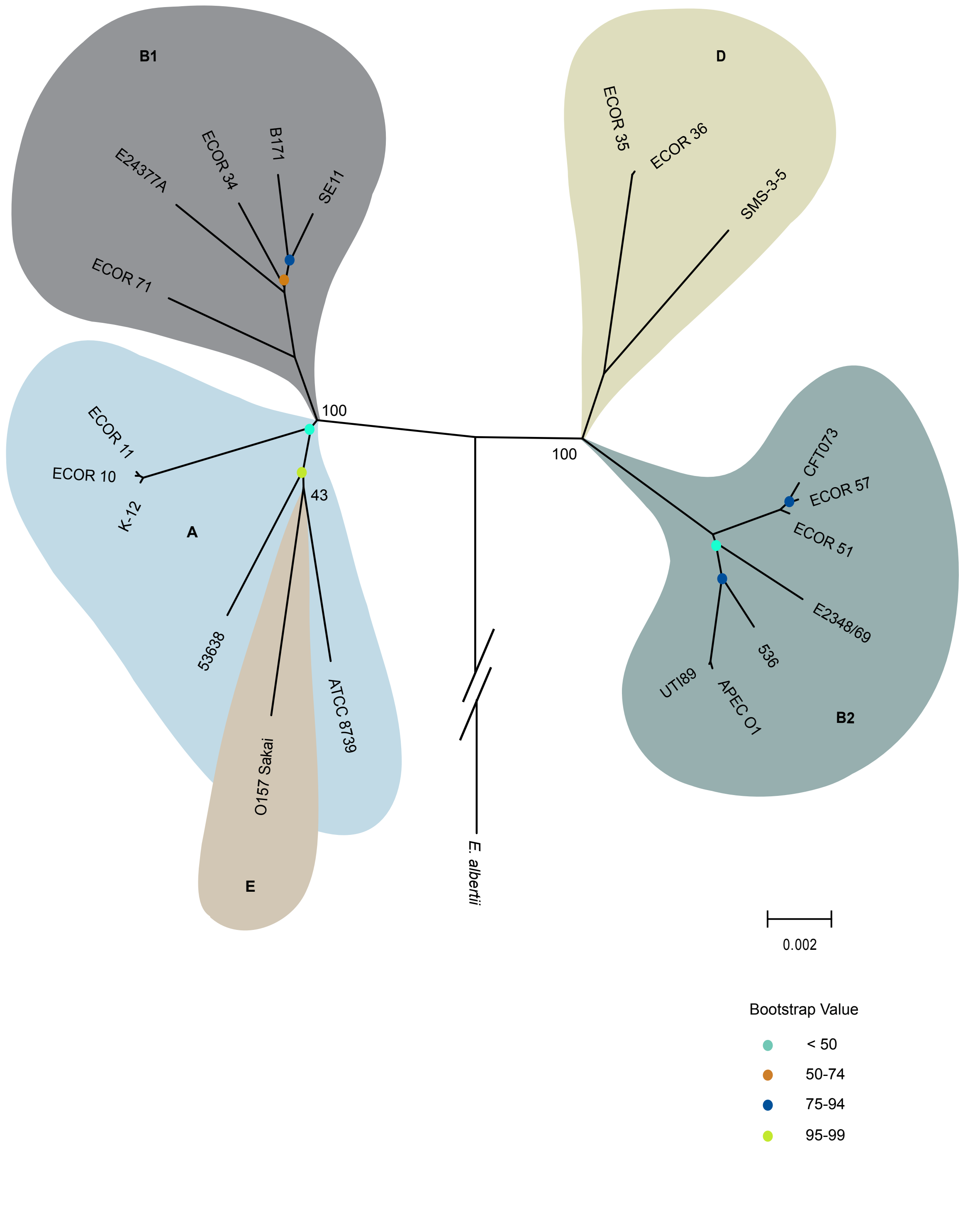

Figure S1. Panel F. Segment 2, Neighbor Joining

A: L1E
B1: OL1E
E: L1E
D (All strains): R1E
B2: R1E

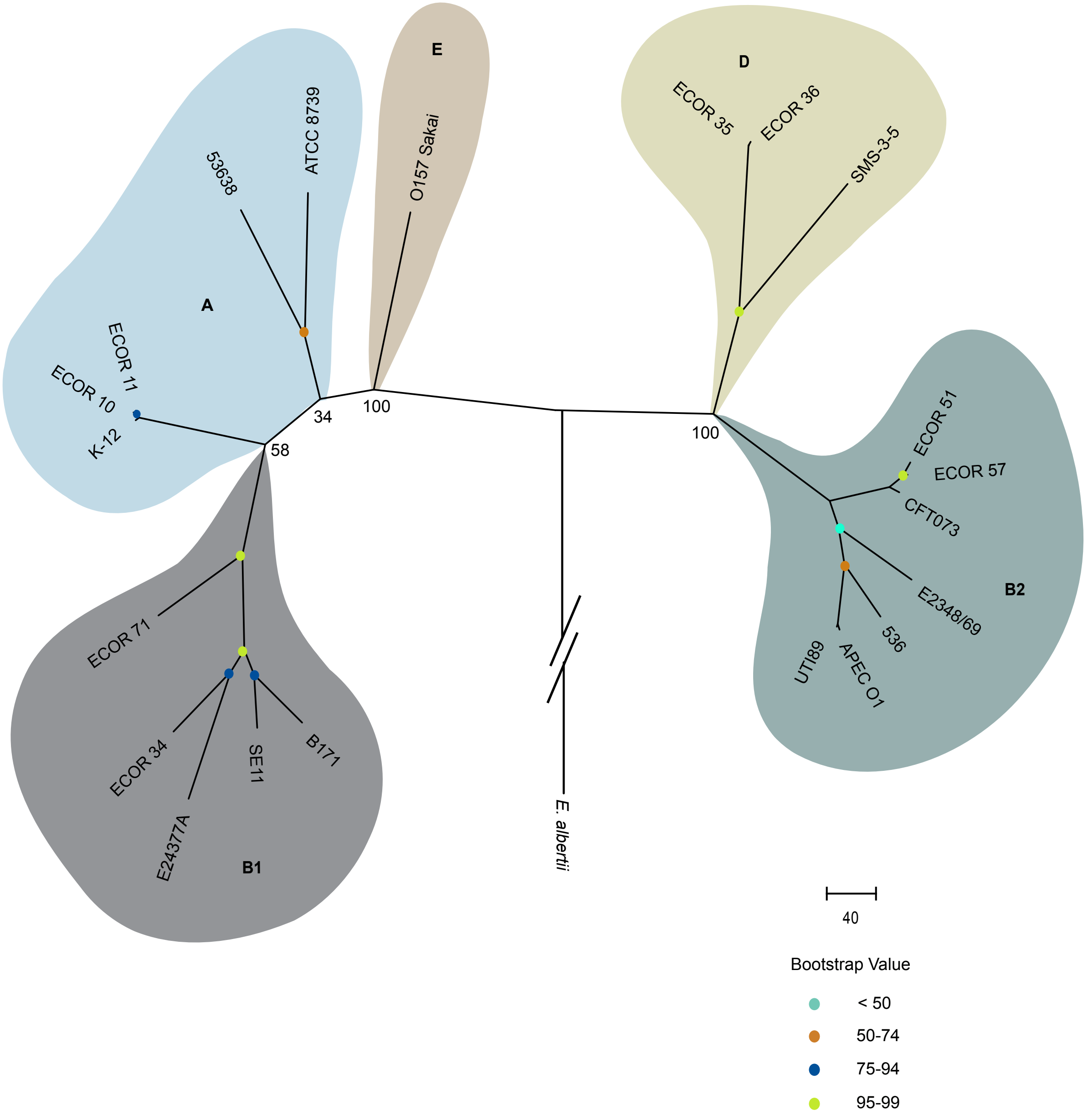

Figure S1. Panel G. Segment 2, Maximum Parsimony

A: L1E

B1: L1E

E: OL1E

D (All strains): R1E

B2: R1E

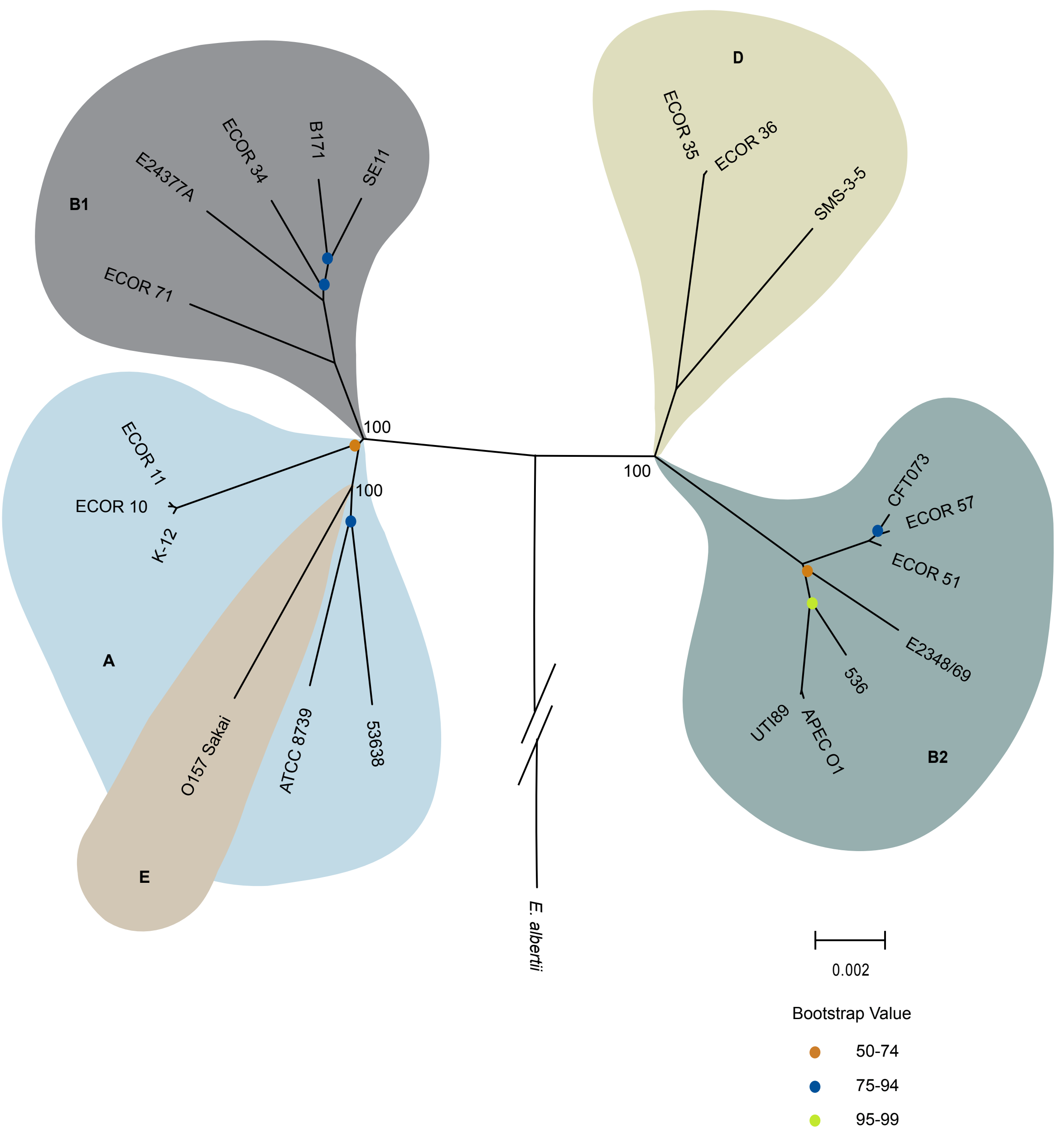

Figure S1. Panel H. Segment 2 Minimum Evolution

A: L2E
B1: L2E
E: L1
D (All strains): R1E
B2: R1E

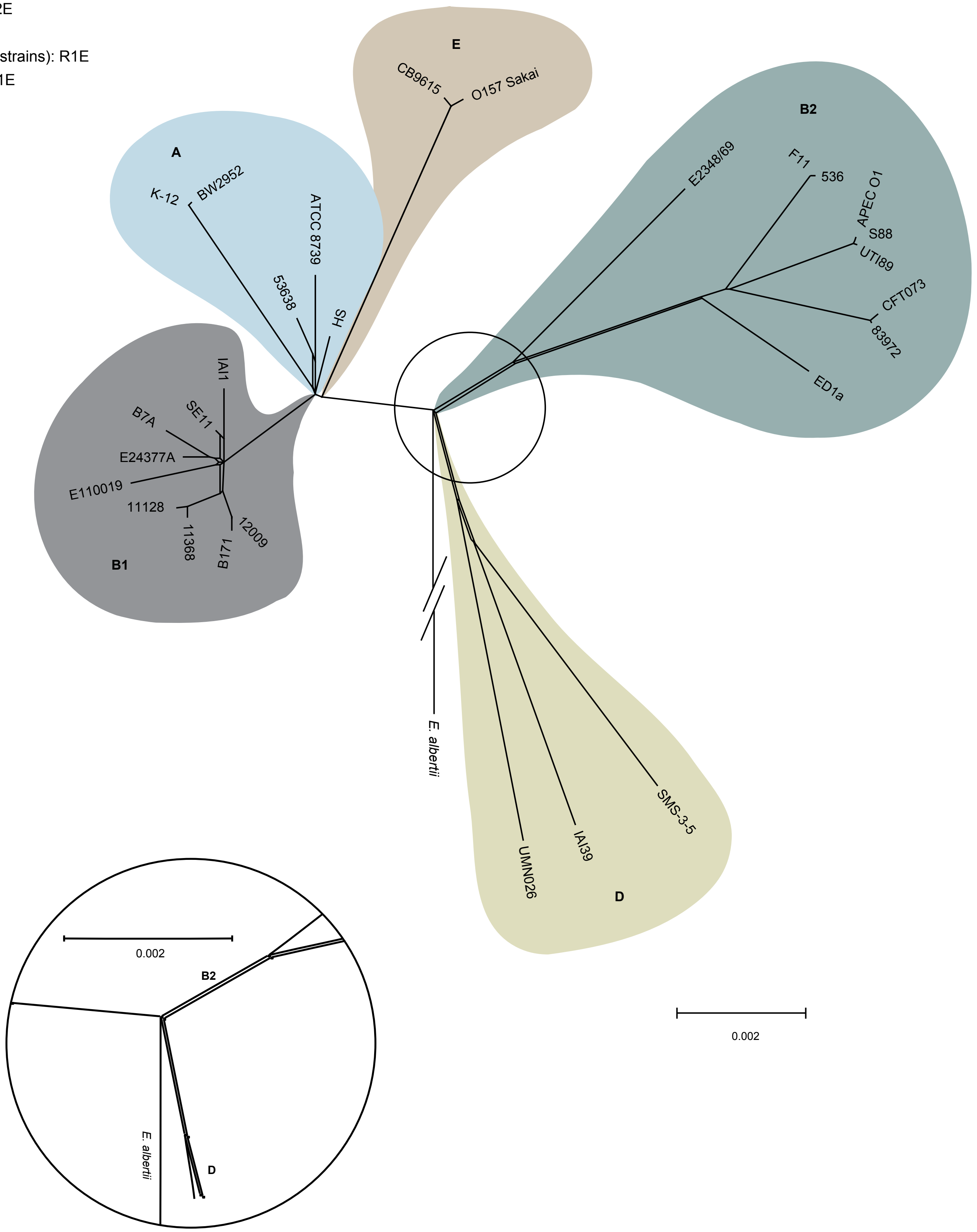

Figure S1. Panel I. Segment 3, Split Decomposition

A: L2E
B1: L2E
E: L1
D (All strains): R1E
B2: R1E

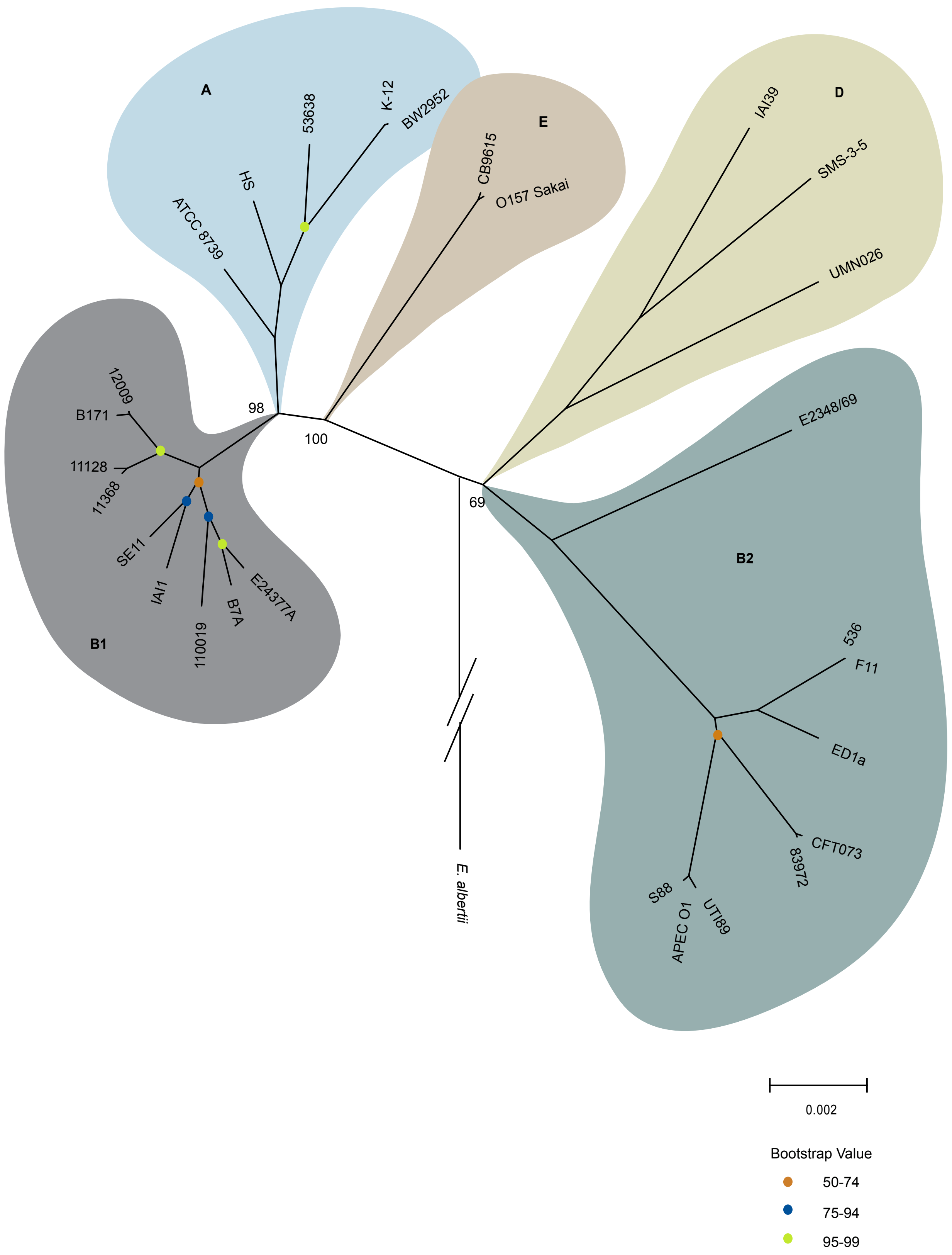

Figure S1. Panel J. Segment 3, Neighbor Joining

A: L3E
B1: L3E
E: L2
D (All strains): R1S
B2: L1

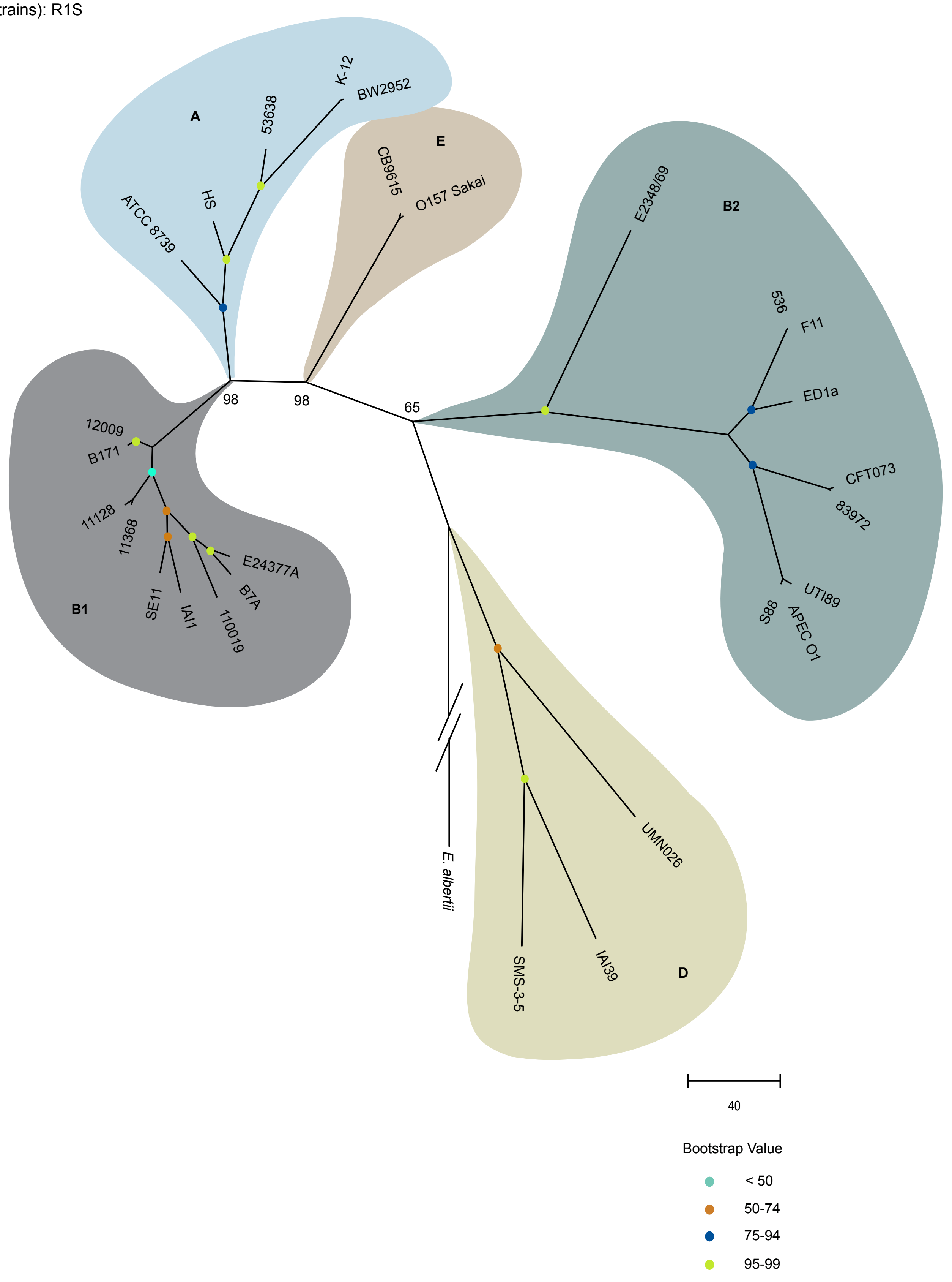

Figure S1. Panel K. Segment 3, Maximum Parsimony

A: L2E
B1: L2E
E: L1
D (All strains): R1E
B2: R1E

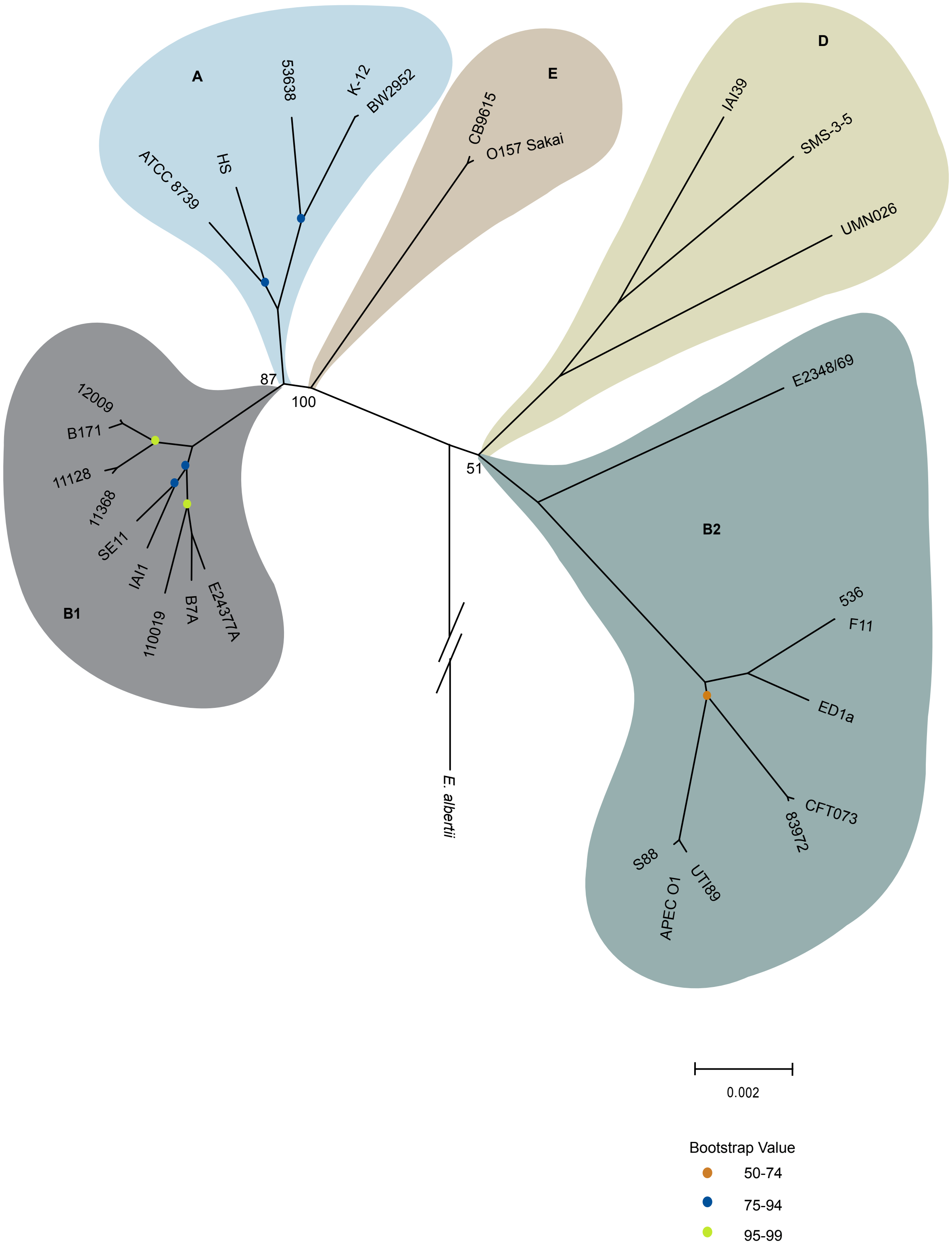

Figure S1. Panel L. Segment 3, Minimum Evolution

A: L2E
B1: L2E
E: L1
D (UMN026 Only): R1
D (SMS-3-5+IAI36 Only): R2E
B2: R2E

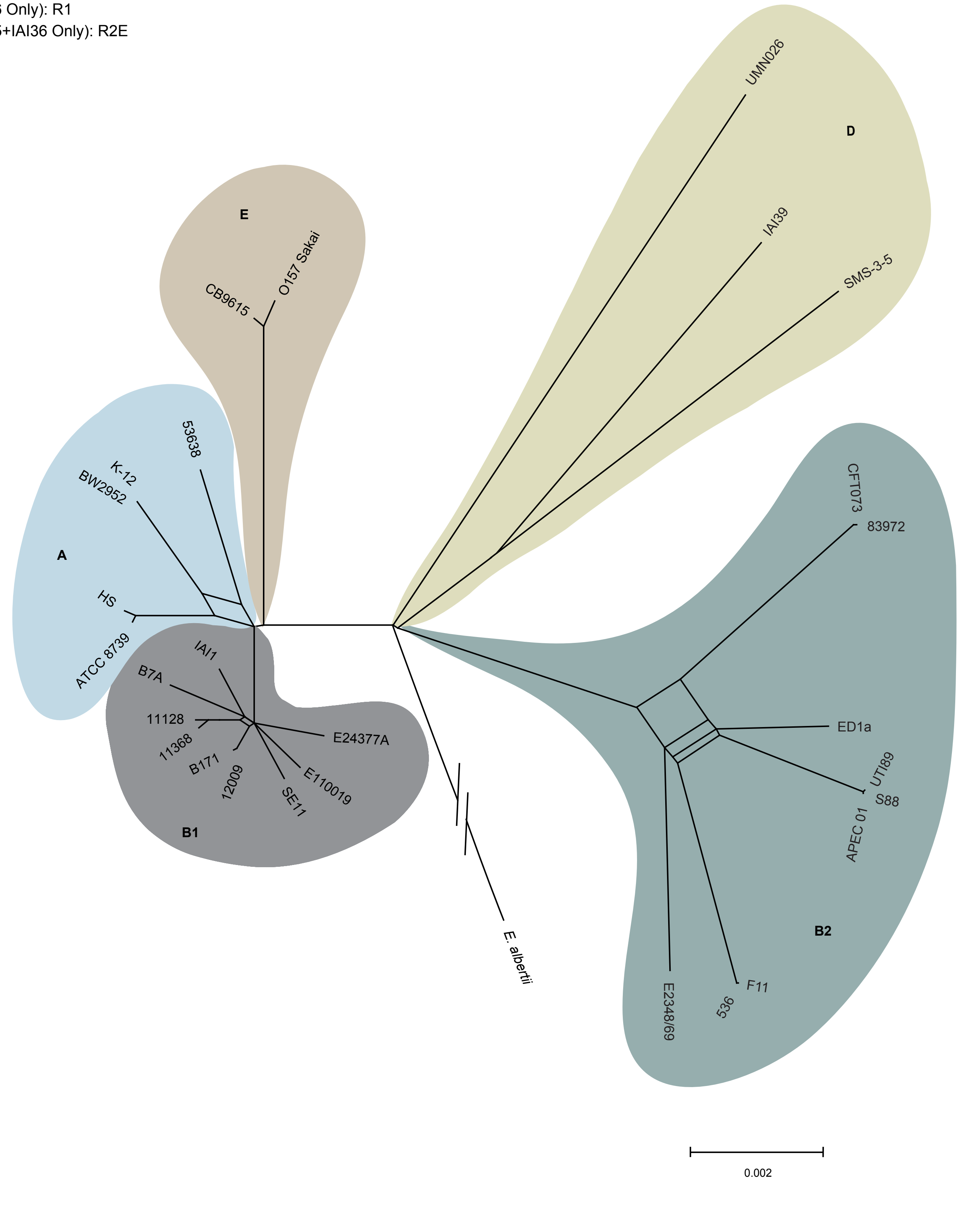

Figure S1. Panel M. Segment 4, Split Decomposition

A: L2E
B1: L2E
E: L1
D (UMN026 Only): EPN
D (SMS-3-5+IAI36 Only): R1E
B2: R1E

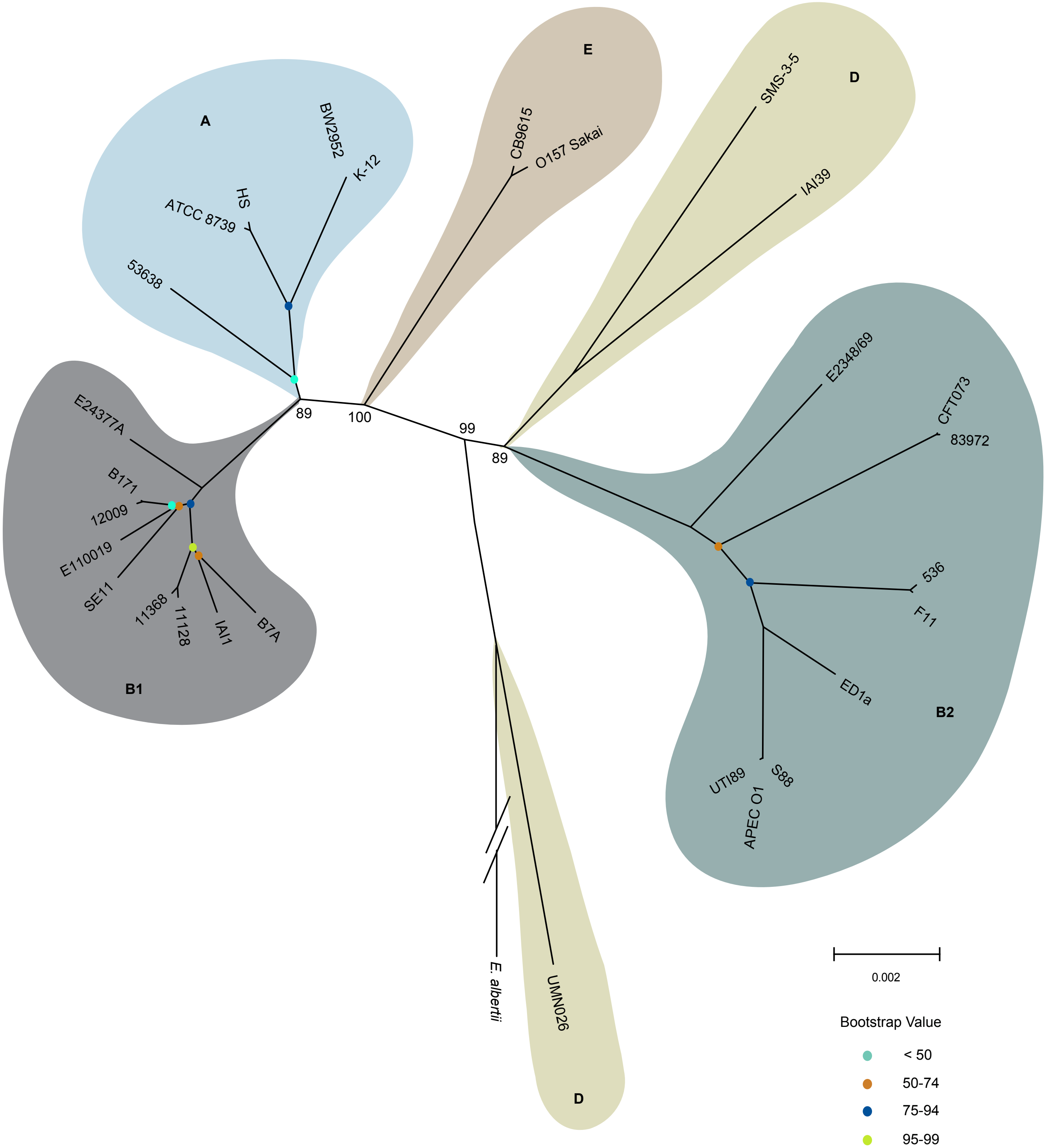

Figure S1. Panel N. Segment 4, Neighbor Joining

A: L2E
B1: L2E
E: L1
D (UMN026 Only): EPN
D (SMS-3-5+IAI36 Only): R1E
B2: R1E

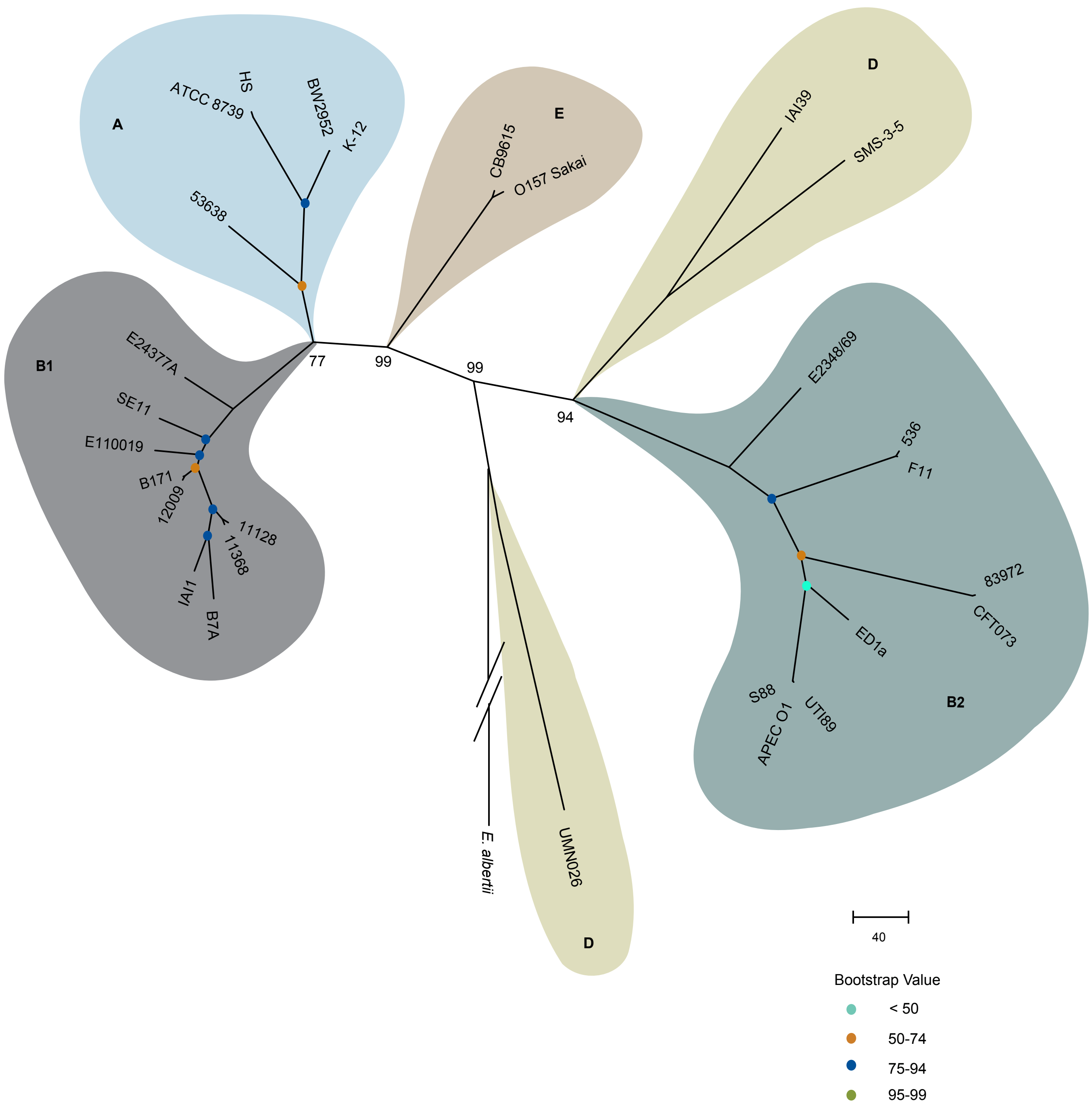

Figure S1. Panel O. Segment 4, Maximum Parsimony

A: L2E
B1: L1
E: L2E
D (UMN026 Only): EPN
D (SMS-3-5+IAI36 Only): R1E
B2: R1E

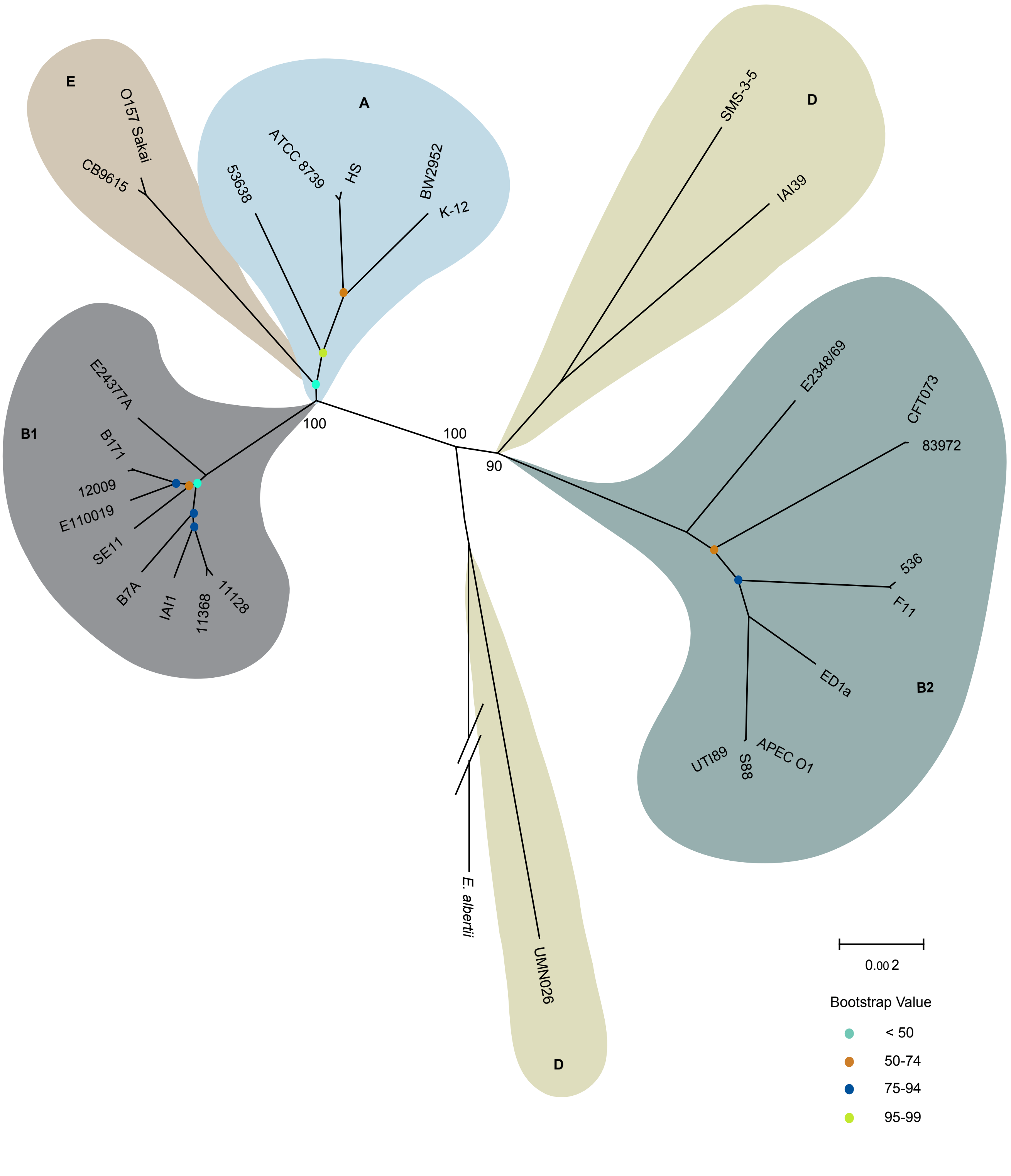

Figure S1. Panel P. Segment 4, Minimum Evolution
