# Supplementary material for: Obscured phylogeny and possible recombinational dormancy in Escherichia coli
Source: BMC Evol Biol. 2011 Jun 27;11:183. doi: 10.1186/1471-2148-11-183 (PMC3152902; doi:10.1186/1471-2148-11-183)
Supplement: Additional file 2 — Figure S1. Topologies generated by various analyses from each Segment. [file 1471-2148-11-183-S2.PDF]

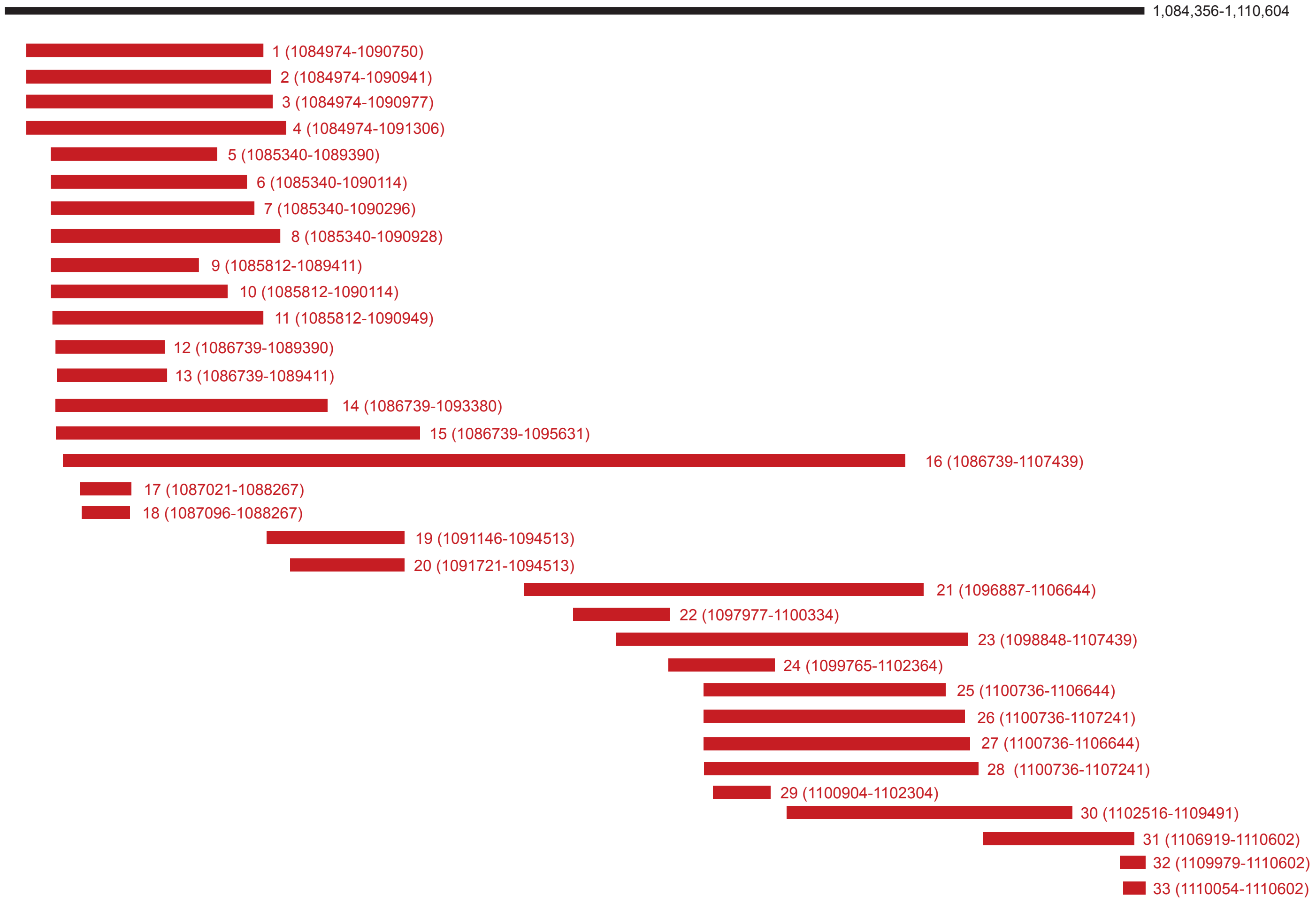

Figure S2a. Segment 1.

Figure S2A-D. Fragments identified as being subjected to conversion. Fragments of Segments 1, 2, 3, and 4 identified by GENECONV as having undergone conversions are depicted in Panels A, B, C and D respectively. Black bar at top of each figure represents nucleotides included in this analysis (coordinates relate to *E. coli* O157:H7 Sakai). Each fragment is labeled with a unique identifying number corresponding to an event portrayed in Figure 2; the borders of the recombination are also provided.

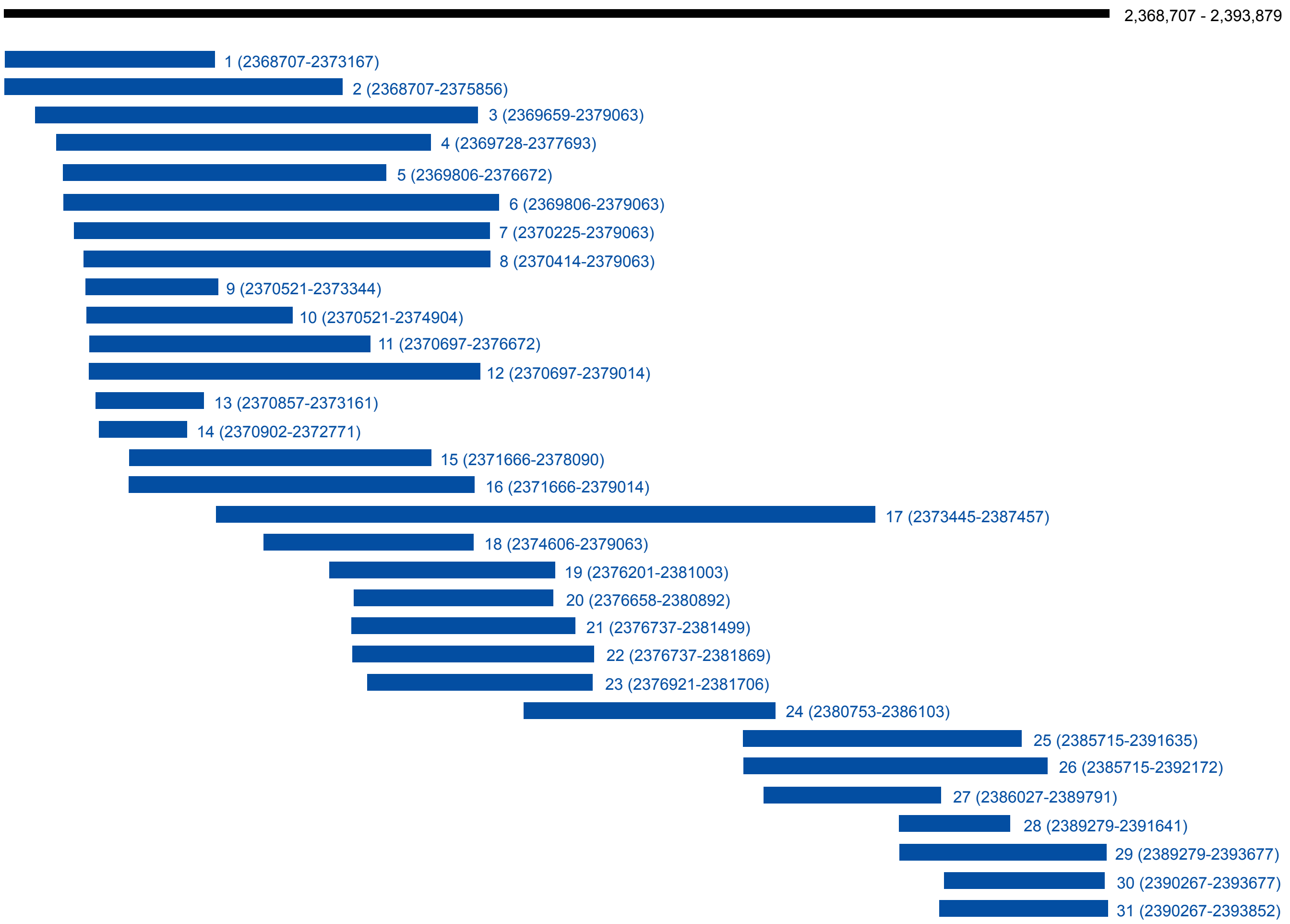

Figure S2b. Segment 2

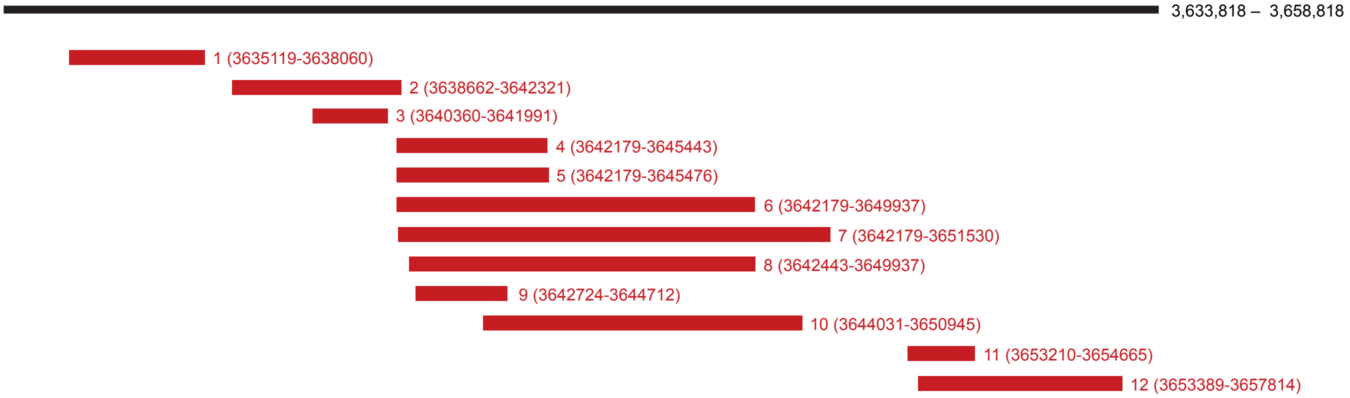

Figure S2c. Segment 3

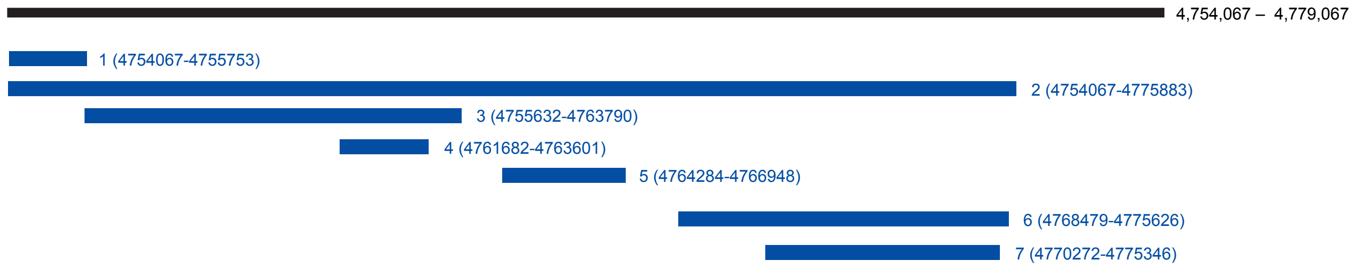

Figure S2d. Segment 4
