# Supplementary material for: Obscured phylogeny and possible recombinational dormancy in Escherichia coli
Source: BMC Evol Biol. 2011 Jun 27;11:183. doi: 10.1186/1471-2148-11-183 (PMC3152902; doi:10.1186/1471-2148-11-183)
Supplement: Additional file 4 — Table S2. Conversion events identified by GENECONV. [file 1471-2148-11-183-S4.DOC]

| **Table S3. Conversion events identified by GENECONV.** All events identified were compared (Tier 1), all conversions involving fragments with identical boundaries were counted only once (Tier 2), and any and all events between groups were counted only once for each Segment (for a maximum of four for any group pair) (Tier 3). Data correspond to Figure 3. The expected number of pairs for any one inter-group pair is calculated by dividing the product of the number of strains in each pair by all possible pairs of strains for that segment (n=916), multiplying by the number of events/occurrences for that segment (n=112, 57, or 13 for tiers 1, 2, and 3, respectively), and then summing over segments. Obs. = Observed number of events, p = p-value, Exp. = Expected number of events. | | | | | | | | | | | |
| --- | --- | --- | --- | --- | --- | --- | --- | --- | --- | --- | --- |
|  |  | | **Tier 1**  **All events identified by GENECONVcounted once** | | | **Tier 2**  **All events of different lengths identified by GENECONV counted once** | | | **Tier 3**  **Any and all exchanges identified by GENECONV counted once** | | |
| Groups  Paired | | Cells | Obs./  Exp. | χ^2^ | p | Obs./  Exp. | χ^2^ | p | Obs./  Exp. | χ^2^ | p |
| A,B1 | | 140 | 40/16.9 | 31.531 | <0.0001 | 21/8.7 | 17.835 | <0.0001 | 2/2.0 | 0.001 | 1.0000 |
| A,E | | 30 | 11/3.4 | 16.772 | 0.0007 | 6/1.7 | 10.367 | 0.0079 | 2/0.4 | 6.306 | 0.0599 |
| A,D | | 60 | 0/9.7 | 9.653 | 0.0014 | 0/4.9 | 4.878 | 0.0279 | 0/1.1 | 1.058 | 0.4145 |
| A,B2 | | 160 | 0/22.9 | 22.934 | <0.0001 | 0/11.6 | 11.621 | 0.0002 | 0/2.6 | 2.571 | 0.0848 |
| B1,E | | 46 | 6/3.8 | 1.347 | 0.2804 | 4/1.9 | 2.195 | 0.1278 | 2/0.5 | 4.714 | 0.0829 |
| B1,D | | 84 | 5/10.1 | 2.610 | 0.0987 | 3/5.2 | 0.908 | 0.3707 | 2/1.2 | 0.568 | 0.6230 |
| B1,B2 | | 232 | 4/24.4 | 17.068 | <0.0001 | 2/12.5 | 8.804 | 0.0014 | 1/2.9 | 1.281 | 0.3205 |
| E,D | | 18 | 0/2.1 | 2.054 | 0.1821 | 0/1.0 | 1.047 | 0.4350 | 0/0.2 | 0.242 | 1.0000 |
| E,B2 | | 50 | 0/5.0 | 4.957 | 0.0333 | 0/2.5 | 2.540 | 0.1149 | 0/0.6 | 0.607 | 0.6572 |
| D,B2 | | 96 | 46/13.8 | 75.537 | <0.0001 | 21/7.0 | 28.220 | <0.0001 | 4/1.5 | 3.916 | 0.0586 |
| Overall | | 916 | 112/112 | 184.462 | <0.0001 | 57/57 | 88.416 | <0.0001 | 13/13 | 21.263 | 0.0237 |
