# Supplementary material for: Obscured phylogeny and possible recombinational dormancy in Escherichia coli
Source: BMC Evol Biol. 2011 Jun 27;11:183. doi: 10.1186/1471-2148-11-183 (PMC3152902; doi:10.1186/1471-2148-11-183)
Supplement: Additional file 5 — Table S3. Sequence alignment. [file 1471-2148-11-183-S5.DOC]

**Table S1. Strains Used.**

| **Strain** | **Group** | **Source**  **Species, Description** | **Role In Study** | **GenBank Accession Numbers** | **Reference** |
| --- | --- | --- | --- | --- | --- |
| 53638 | A | EIEC | GBS | AAKB00000000 | TIGR |
| ATCC 8739 | A | Feces, Crooks Strain | GBS | CP000946 | ATCC |
| ECOR 2 | A | Human, healthy | PD |  | (1) |
| ECOR 7 | A | Orangutan, healthy | PD | |  | | --- | | (1) |
| ECOR 10 | A | Human, healthy | SSG | | Segment 1: XXXXX-A...XXXXX-B | | --- | | Segment 2: XXXXX-A...XXXXX-B | | (1) |
| ECOR 11 | A | Human, urinary tract infection | SSG | | Segment 1: XXXXX-A...XXXXX-B | | --- | | Segment 2: XXXXX-A...XXXXX-B | | (1) |
| K-12 MG1655 | A | Human, laboratory strain | GBS | U00096 | (2) |
| BW2952 | A | Human, laboratory strain | GBS | CP001396 | (3) |
| HS | A | Human, commensal | GBS | CP000802 | (4) |
| B171 | B1 | Human child, diarrhea, EPEC | GBS | AAJX00000000 | (5) |
| ECOR 28 | B1 | Human, healthy | PD | |  | | --- | | (1) |
| ECOR 32 | B1 | Giraffe, healthy | SSG | | Segment 1: XXXXX-A...XXXXX-B | | --- | | Segment 2: XXXXX-A...XXXXX-B | | (1) |
| ECOR 34 | B1 | Dog, healthy | SSG | | Segment 1: XXXXX-A...XXXXX-B | | --- | | Segment 2: XXXXX-A...XXXXX-B | | (1) |
| ECOR 71 | B1 | Human, asymptomatic bacteriuria | SSG | | Segment 1: XXXXX-A...XXXXX-B | | --- | | Segment 2: XXXXX-A...XXXXX-B | | (1) |
| E24377A | B1 | Human, diarrhea, ETEC | GBS | CP000800 | (4) |
| SE11 | B1 | Human, fecal commensal | GBS | AP009240 | (6) |
| 11128 | B1 | Human, bloody diarrhea, EHEC 2 | GBS | AP010960 | (7) |
| 11368 | B1 | Human, diarrhea, EHEC 2 | GBS | AP010953 | (7) |
| IAI1 | B1 | Human, fecal commensal | GBS | CU928160 | (8) |
| O157 Sakai | E | Human, diarrhea, EHEC 1 | GBS | BA000007 | (9) |
| CB9615 | E | Human, diarrhea, O55:H7 | GBS | CP001846 | (10) |
| ECOR 35 | D | Human, healthy | SSG | | Segment 1: XXXXX-A...XXXXX-B | | --- | | Segment 2: XXXXX-A...XXXXX-B | | (1) |
| ECOR 36 | D | Human, healthy | SSG | | Segment 1: XXXXX-A...XXXXX-B | | --- | | Segment 2: XXXXX-A...XXXXX-B | | (1) |
| ECOR 41 | D | Human, healthy | PD | |  | | --- | | (1) |
| ECOR 44 | D | Cougar, healthy | PD | |  | | --- | | (1) |
| IAI39 | D | Human, urine, ExPEC | GBS | CU928164 | (8) |
| UMN026 | D | Human, urine, ExPEC | GBS | CU928163 | (8) |
| SMS-3-5 | D | Industrial toxic metal-contaminated coastal environment | GBS | CP000970 | (11) |
| 536 | B2 | Human, acute pyelonephritis, UPEC | GBS | CP000247 | (12) |
| APEC O1 | B2 | Turkey, colisepticemia - lung, APEC | GBS | DQ381420 | (13) |
| CFT073 | B2 | Human, urine, UPEC | GBS | AE014075 | (14) |
| ECOR 51 | B2 | Infant, healthy | SSG | | Segment 1: XXXXX-A...XXXXX-B | | --- | | Segment 2: XXXXX-A...XXXXX-B | | (1) |
| ECOR 55 | B2 | Human, urinary tract infection | PD | |  | | --- | | (1) |
| ECOR 57 | B2 | Gorilla, healthy | SSG | | Segment 1: XXXXX-A...XXXXX-B | | --- | | Segment 2: XXXXX-A...XXXXX-B | | (1) |
| ECOR 60 | B2 | Human, urinary tract infection | PD | |  | | --- | | (1) |
| E2348/69 | B2 | Infant human, diarrhea, EPEC | GBS | FM180568 | (15) |
| UTI89 | B2 | human, urinary tract infection | GBS | CP000243 | (16) |
| 83972 | B2 | Human, urine, Asymptomatic bacteriuria | GBS | ACGN00000000 | (17) |
| ED1a | B2 | Human, fecal commensal | GBS | CU928162 | (8) |
| S88 | B2 | Human, cerebrospinal isolate (meningitis), ExPEC | GBS | CU928161 | (8) |
| E110019 | N/A | Human, atypical EPEC | GBS | AAJW00000000 | (18) |
| B7A | N/A | ETEC | GBS | AAJT00000000 | (4) |
| F11 | N/A | Human, urinary tract infection, ExPEC | GBS | AAJU00000000 | (4) |

**References**

1. Ochman H & Selander RK (1984) Standard reference strains of *Escherichia coli* from natural populations. *J Bacteriol* 157:690-693.

2. Blattner FR*, et al.* (1997) The complete genome sequence of *Escherichia coli* K-12. *Science* 277:1453-1462.

3. Ferenci T*, et al.* (2009) Genomic sequencing reveals regulatory mutations and recombinational events in the widely used MC4100 lineage of *Escherichia coli* K-12. *J Bacteriol* 191:4025-4029.

4. Rasko DA*, et al.* (2008) The pangenome structure of *Escherichia coli*: comparative genomic analysis of *E. coli* commensal and pathogenic isolates. *J Bacteriol* 190:6881-6893.

5. Vuopio-Varkila J & Schoolnik GK (1991) Localized adherence by enteropathogenic *Escherichia coli* is an inducible phenotype associated with the expression of new outer membrane proteins. *J Exp Med* 174:1167-1177.

6. Oshima K*, et al.* (2008) Complete genome sequence and comparative analysis of the wild-type commensal Escherichia coli strain SE11 isolated from a healthy adult. *DNA Res* 15:375-386.

7. Ogura Y*, et al.* (2009) Comparative genomics reveal the mechanism of the parallel evolution of O157 and non-O157 enterohemorrhagic *Escherichia coli*. *Proc Natl Acad Sci U S A* 106:17939-17944.

8. Touchon M*, et al.* (2009) Organised genome dynamics in the *Escherichia coli* species results in highly diverse adaptive paths. *PLoS Genet* 5:e1000344.

9. Hayashi T*, et al.* (2001) Complete genome sequence of enterohemorrhagic *Escherichia coli* O157:H7 and genomic comparison with a laboratory strain K-12. *DNA Res* 8:11-22.

10. Zhou Z*, et al.* (2010) Derivation of *Escherichia coli* O157:H7 from its O55:H7 precursor. *PLoS One* 5:e8700.

11. Fricke WF*, et al.* (2008) Insights into the environmental resistance gene pool from the genome sequence of the multidrug-resistant environmental isolate *Escherichia coli* SMS-3-5. *J Bacteriol* 190:6779-6794.

12. Hochhut B*, et al.* (2006) Role of pathogenicity island-associated integrases in the genome plasticity of uropathogenic *Escherichia coli* strain 536. *Mol Microbiol* 61:584-595.

13. Johnson TJ, Johnson SJ, & Nolan LK (2006) Complete DNA sequence of a ColBM plasmid from avian pathogenic *Escherichia coli* suggests that it evolved from closely related ColV virulence plasmids. *J Bacteriol* 188:5975-5983.

14. Welch RA*, et al.* (2002) Extensive mosaic structure revealed by the complete genome sequence of uropathogenic *Escherichia coli*. *Proc Natl Acad Sci U S A* 99:17020-17024.

15. Iguchi A*, et al.* (2009) Complete genome sequence and comparative genome analysis of enteropathogenic *Escherichia coli* O127:H6 strain E2348/69. *J Bacteriol* 191:347-354.

16. Chen SL*, et al.* (2006) Identification of genes subject to positive selection in uropathogenic strains of *Escherichia coli:* a comparative genomics approach. *Proc Natl Acad Sci U S A* 103:5977-5982.

17. Andersson P*, et al.* (1991) Persistence of *Escherichia coli* bacteriuria is not determined by bacterial adherence. *Infect Immun* 59:2915-2921.

18. Viljanen MK*, et al.* (1990) Outbreak of diarrhoea due to *Escherichia coli* O111:B4 in schoolchildren and adults: association of Vi antigen-like reactivity. *Lancet* 336:831-834.
