# Supplementary material for: Obscured phylogeny and possible recombinational dormancy in Escherichia coli
Source: BMC Evol Biol. 2011 Jun 27;11:183. doi: 10.1186/1471-2148-11-183 (PMC3152902; doi:10.1186/1471-2148-11-183)
Supplement: Additional file 3 — Figure S2. Fragments identified as being subjected to conversion. [file 1471-2148-11-183-S3.PDF]

Table S2. Sequence alignment. The complete set of sequences analyzed in Segments 1-4 are aligned, with E. coli K-12 used as a reference. Variant nucleotides are indicated by letter abbreviations. "•" and "-" represent nucleotide identity to the reference K-12 sequence, or gaps, respectively.

```
                1
K-12            CAGTTGCTCG ATGAGGTTCA GGTAATCTTC TTTTTCCCAC TCGCTGCCTG GTAGCGAAAG AATCGTTTCA
53638           .......... .......... .......... .......... .......... .......... ..........
ATCC_8739       .......... .......... .......... .......... .......... .......... ..........
ECOR_10         .......... .......... .......... .......... .......... .......... ..........
ECOR_11         .......... .......... .......... .......... .......... .......... ..........
B171            .......... .......... .......... .......... .......... .......... ..........
E24377A         .......... .......... .......... .......... .......... .......... ..........
ECOR_34         .......... .......... .......... .......... .......... .......... ..........
ECOR_71         .......... .......... .......... .......... .......... .......... ..........
SE11            .......... .......... .......... .......... .......... .......... ..........
O157_Sakai      .......... .......... .......... .......... .......... .......... ..........
ECOR_36         .......... .......... .......... C......... .....A.... .......... ..........
ECOR_35         .......... .......... .......... C......... .....A.... .......... ..........
SMS-3-5         .......... .......... .......... .......... A.A....... ......G... ...G......
536             .......... .......... .......... C......... .....A.... .......... ..........
APEC_O1         .......... .......... .......... C......... .....A.... .......... ..........
CFT073          .......... .......... .......... C......... .....A.... .......... ..........
E2348/69        .......... .......... .......... C......... .....A.... .......... ..........
ECOR_51         .......... .......... .......... C......... .....A.... .......... ..........
ECOR_57         .......... .......... .......... C......... .....A.... .......... ..........
UTI89           .......... .......... .......... C......... .....A.... .......... ..........
E._albertii     ---------- ---------- ---------- ---------- ---------- ---------- ----------

              71
K-12            CGTAAATGGC TGCCCATACT GTTATTCGCG AGCCACAGAT CTTCAGCCGG ATAAATATCG GACATGCCAG
53638           .......... .......... .......... .......... .......... .......... ..........
ATCC_8739       .......... .......... .......... .......... .......... .......... ..........
ECOR_10         .......... .......... .......... .......... .......... .......... ..........
ECOR_11         .......... .......... .......... .......... .......... .......... ..........
B171            .......... .......... .......... .......... .......... .......... ..........
E24377A         .......... .......... .......... .......... .......... .......... ..........
ECOR_34         .......... .......... .......... .......... .......... .......... ..........
ECOR_71         .......... .......... .......... .......... .......... .......... ..........
SE11            .......... .......... .......... .......... .......... .......... ..........
O157_Sakai      .......... .......... .......... .......... .......... .......... ..........
ECOR_36         .......... .......... .........C .......... .......... .......... ..........
ECOR_35         .......... .......... .........C .......... .......... .......... ..........
SMS-3-5         .......... .A........ .........T .A........ .......... .......... ..........
536             .......... .......... .........C .......... .......... .......... ..........
APEC_O1         .......... .......... .........C .......... .......... .......... ..........
CFT073          .......... .......... .........C .......... .......... .......... ..........
E2348/69        .......... .......... .........C .......... .......... .......... ..........
ECOR_51         .......... .......... .........C .......... .......... .......... ..........
ECOR_57         .......... .......... .........C .......... .......... .......... ..........
UTI89           .......... .......... .........C .......... .......... .......... ..........
E._albertii     ---------- ---------- ---------- ---------- ---------- ---------- ----------

              141
K-12            GCACGATAAT ACGGCAAGCA TAATTCTTCA TCATCGAAGG TTGGCGGAGT AAACACATCC AAATCTTTCA
53638           .......... .......... .......... .......... .......... .......... ..........
ATCC_8739       .......... .......... .......... .......... .......... .......... ..........
ECOR_10         .......... .......... .......... .......... .......... .......... ..........
ECOR_11         .......... .......... .......... .......... .......... .......... ..........
B171            .......... G........G ..G....... .......... .......... .......... ..........
E24377A         .......... G........G ..G....... .......... .......... .......... ..........
ECOR_34         .......... .......... .......... .......... .......... .......... ..........
ECOR_71         .......... .........G ..G....... .......... .......... .......... ..........
SE11            .......... G........G ..G....... .......... .......... .......... ..........
O157_Sakai      .......... .......... .......... .......... .......... .......... ..........
ECOR_36         .......... T......... .......... .......... .......... .......... ..........
ECOR_35         .......... T......... .......... .......... .......... .......... ..........
SMS-3-5         .......... .......... .......... .......... .......... .......... ..........
536             .......... .........G ..G....... .......... ....T..... .......... .G........
APEC_O1         .......... .........G ..G....... .......... ....T..... .......... .G......T.
CFT073          .......... .........G ..G....... .......... ....T..... .......... .G........
E2348/69        .......... .........G ..G....... .......... ....T..... .......... .G........
ECOR_51         .......... .........G ..G....... .......... ....T..... .......... .G........
ECOR_57         .......... .........G ..G....... .......... ....T..... .......... .G........
UTI89           .......... .........G ..G....... .......... ....T..... .......... .G......T.
E._albertii     ---------- ---------- ---------- ---------- ---------- ---------- ----------
```

```
             211
K-12           GGCCACGACC TTGCAGCAGC TCGGTCACGG TACGTTCCAG TGCTACGCCA AAATCAGGAT GCGCACCGAA
53638          .......... .......... .......... .......... .......... .......... ..........
ATCC_8739      .......... .......... .......... .......... .......... .......... ..........
ECOR_10        .......... .......... .......... .......... .......... .......... ..........
ECOR_11        .......... .......... .......... .......... .......... .......... ..........
B171           .......... .......... .......... .......... .......... .......... ..........
E24377A        .......... .......... .......... .......... .......... .......... ..........
ECOR_34        .......... .......... .......... .......... .......... .......... ..........
ECOR_71        .......... .......... .......... .......... .......... .......... ..........
SE11           .......... .......... .......... .......... .......... .......... ..........
O157_Sakai     .......... .......... .......... .......... .......... .......... ..........
ECOR_36        .......... .......... .......... .......... .......... .......... ..........
ECOR_35        .......... .......... .......... .......... .......... .......... ..........
SMS-3-5        .......... .......... .......... .......... .......... .......... ..........
536            .......... ........A. .......... .......... ...A...... .......... ..........
APEC_O1        .......... .......... .......... .......... ...A...... .......... ..........
CFT073         .......... .......... .......... .......... ...A...... .......... ..........
E2348/69       .......... .......... .......... .......... ...A...... .......... ..........
ECOR_51        .......... .......... .......... .......... ...A...... .......... ..........
ECOR_57        .......... .......... .......... .......... ...A...... .......... ..........
UTI89          .......... .......... .......... .......... ...A...... .......... ..........
E._albertii    ---------- ---------- ---------- ---------- ---------- ---------- ----------

             281
K-12           AGAGGCAAAG CAGGTACCGT TAGCAGGATT GAACAGTACC ACGCAAATCA CCGGATACTG GCCGCCAAGC
53638          .......... .......... .......... .......... .......... .......... ..........
ATCC_8739      .......... .......... .......... .......... .......... .......... ..........
ECOR_10        .......... .......... .......... .......... .......... .......... ..........
ECOR_11        .......... .......... .......... .......... .......... .......... ..........
B171           .......... .......... .......... .......... .......... .......... ..........
E24377A        .......... .......... .......... .......... .......... .......... ..........
ECOR_34        .......... .......... .......... .......... .......... .......... ..........
ECOR_71        .......... .......... .......... .......... .......... .......... ..........
SE11           .......... .......... .......... .......... .......... .......... ..........
O157_Sakai     .......... .....G.... .......... A......... .......... .......... .........G
ECOR_36        .......... .......... .......... .......... .......... .......... ..........
ECOR_35        .......... .......... .......... .......... .......... .......... ..........
SMS-3-5        .......... .....G.... .......... A....A.... .......... .......... ..........
536            T......... .......... .......... .......... .......... .......... ..........
APEC_O1        T......... .......... .......... .......... .......... .......... ..........
CFT073         T......... .......... .......... .......... .......... .......... ..........
E2348/69       T......... .......... .......... .......... .......... .......... ..........
ECOR_51        T......... .......... .......... .......... .......... .......... ..........
ECOR_57        T......... .......... .......... .......... .......... .......... ..........
UTI89          T......... .......... .......... .......... .......... .......... ..........
E._albertii    ---------- ---------- ---------- ---------- ---------- ---------- ----------

             351
K-12           GAACCGTCAT AAGCGAAGAT TGGGAAACCC TCCGCTTCCA GCGTTTCGAT GGCTTCAACT ACTGCTGGGT
53638          .......... .......... .......... .......... .......... .......... ..........
ATCC_8739      .......... .......... .......... .......... .......... .......... ..........
ECOR_10        .......... .......... .......... .......... .......... .......... ..........
ECOR_11        .......... .......... .......... .......... .......... .......... ..........
B171           .......... .......... .......... .......... .......... .......... ..........
E24377A        .......... .......... .......... .......... .......... .......... ..........
ECOR_34        .......... .......... .......... .......... .......... .......... ..........
ECOR_71        .......... .......... .......... .......... .......... .......... ..........
SE11           .......... .......... .......... .......... .......... .......... ..........
O157_Sakai     .....A.... .T........ C........T ..T....... .T........ C........C ..C.......
ECOR_36        .....A.... ....A..... .........T ..T....... .T........ .........C ..C.......
ECOR_35        .....A.... ....A..... .........T ..T....... .T........ .........C ..C.......
SMS-3-5        .....A.... ....A..... .........T ..T....... .T........ C........C ..C.......
536            .......... .......... C........T .......... .......... C......... ........A.
APEC_O1        .......... .......... C......T.T .......... .......... C......... ........A.
CFT073         .......... .......... C........T .......... .......... C......... ........A.
E2348/69       .......... .......... C........T .......... .......... C......... ........A.
ECOR_51        .......... .......... C........T .......... .......... C......... ........A.
ECOR_57        .......... .......... C........T .......... .......... C......... ........A.
UTI89          .......... .......... C......T.T .......... .......... C......... ........A.
E._albertii    ---------- ---------- ---------- ---------- ---------- ---------- ----------
```

```
             421
K-12         AACGCGCCAG CACGTCTGCC GGGATCTCTG GCAGGCTGAT GCTTTCAGCA ATAATGCGGT TTTTCACGTA
53638        .......... .......... .......... .......... .......... .......... ..........
ATCC_8739    .......... .......... .......... .......... .......... .......... ..........
ECOR_10      .......... .......... .......... .......... .......... .......... ..........
ECOR_11      .......... .......... .......... .......... .......... .......... ..........
B171         .......... .......... .......... .......... .......... .......... ..........
E24377A      .......... .......... .......... .......... .......... .......... ..........
ECOR_34      .......... .......... .......... .......... .......... .......... ..........
ECOR_71      .......... .......... .......... .......... .......... .......... ..........
SE11         .......... .......... .......... .......... .......... .......... ..........
O157_Sakai   .......... .......... .....T..C. ...A...... .......... .......... ..........
ECOR_36      .......... .......... .....T..C. ...A...... .......... .......... ..........
ECOR_35      .......... .......... .....T..C. ...A...... .......... .......... ..........
SMS-3-5      .......... .......... .....T..C. ...A...... .......... .......... ..........
536          ....T..... .......... .....T..C. ...A...... .......... .......... ..........
APEC_O1      ....T..... .......... .....T..C. ...A...... .......... .......... ..........
CFT073       ....T..... .......... .....T..C. ...A...... .......... .......... ..........
E2348/69     ....T..... .......... .....T..C. ...A...... .......... .......... ..........
ECOR_51      ....T..... .......... .....T..C. ...A...... .......... .......... ..........
ECOR_57      ....T..... .......... .....T..C. ...A...... .......... .......... ..........
UTI89        ....T..... .......... .....T..C. ...A...... .......... .......... ..........
E._albertii  ---------- ---------- ---------- ---------- ---------- ---------- ----------

             491
K-12         GCGTTCGAAA ACTTCAGACA ATCCCTGAAC GCGTGCTTCG TTGCGGGTAT TACCTGCGGA CATACCGTTG
53638        .......... .......... .......... .......... .......... .......... ..........
ATCC_8739    .......... .......... .......... .......... .......... .......... ..........
ECOR_10      .......... .......... .......... .......... .......... .......... ..........
ECOR_11      .......... .......... .......... .......... .......... .......... ..........
B171         .......... .......... .......... .......... .......... .......... ..........
E24377A      .......... .......... .......... .......... .......... .......... ..........
ECOR_34      .......... .......... .......... .......... .......... .......... ..........
ECOR_71      .......... .......... .......... .......... .......... .......... ..........
SE11         .......... .......... .......... .......... .......... .......... ..........
O157_Sakai   .......... .....G.... .C........ .......... ..A....... .G..A..A.. .........A
ECOR_36      A......... .....G.... .C........ .......... ..A....... .G..A..A.. .........A
ECOR_35      A......... .....G.... .C........ .......... ..A....... .G..A..A.. .........A
SMS-3-5      .......... .....G.... .......... .......... ..A....... .G..A..A.. .........A
536          A......... .....G.... .C........ .......... ..A....... .G..A..A.. .........A
APEC_O1      A......... .......... .......... .......... ..A....... .G..A..A.. .........A
CFT073       A......... .....G.... .C........ .......... ..A....... .G..A..A.. .........A
E2348/69     A......... .....G.... .......... .......... ..A....... .G..A..A.. .........A
ECOR_51      A......... .....G.... .C........ .......... ..A....... .G..A..A.. .........A
ECOR_57      A......... .....G.... .C........ .......... ..A....... .G..A..A.. .........A
UTI89        A......... .......... .......... .......... ..A....... .G..A..A.. .........A
E._albertii  ---------- ---------- ---------- ---------- ---------- ---------- ----------

             561
K-12         GAGACGTACA GGTTACCAAT GATATTCATC GGAATATAAA CGGTCTGATT GTCGGACTGA CGCGTAAACG
53638        .......... .......... .......... .......... .......... .......... ..........
ATCC_8739    .......... .......... .......... .......... .......... .......... ..........
ECOR_10      .......... .......... .......... .......... .......... .......... ..........
ECOR_11      .......... .......... .......... .......... .......... .......... ..........
B171         .......... .......... .......... .......... .......... .......... ..........
E24377A      .......... .......... .......... .......... .......... .......... ..........
ECOR_34      .......... .......... .......... .......... .......... .......... ..........
ECOR_71      .......... .......... .......... .......... .......... .......... ..........
SE11         .......... .......... .......... .......... .......... .......... ..........
O157_Sakai   ..A....... .......... .......... .......... .......... A..A...... ..T.......
ECOR_36      ..A....... A......... .......... .......... T......... .........G ..........
ECOR_35      ..A....... A......... .......... .......... T......... .........G ..........
SMS-3-5      ..A....... A......... .......... .......... T......G.. .........G ..........
536          ..A....... A......... .......... .......... T......... .........G ..........
APEC_O1      ..A....... A......... .......... .......... T......... .........G ..........
CFT073       ..A....... A......... .......... .......... T......... .........G ..........
E2348/69     ..A....... A......... .......... .......... T......... .........G ..........
ECOR_51      ..A....... A......... .......... .......... T......... .........G ..........
ECOR_57      ..A....... A......... .......... .......... T......... .........G ..........
UTI89        ..A....... A......... .......... .......... T......... .........G ..........
E._albertii  ---------- ---------- ---------- ---------- ---------- ---------- ----------
```

```
           631
K-12         GCAGGCCGCA AATACCACGA TCTTCGTTCC ATGCCGATGA ACTGTTGACT ATGCTTTTAG CGGATAAACC
53638        .......... .......... .......... .......... .......... .......... ..........
ATCC_8739    .......... .......... .......... .......... .......... .......... ..........
ECOR_10      .......... .......... .......... .......... .......... .......... ..........
ECOR_11      .......... .......... .......... .......... .......... .......... ..........
B171         .......... .......... .......... .......... .......... .......... ..........
E24377A      .......... .......... .......... .......... .......... .......... ..........
ECOR_34      .......... .......... .......... .......... .......... .......... ..........
ECOR_71      .......... .......... .......... .......... .......... .......... ..........
SE11         .......... .......... .......... .......... .......... .......... ..........
O157_Sakai   ....A..... .......... .......... .......... .......... .......... ..........
ECOR_36      .T..A..... .......... .......... .......... .......... .......... T.........
ECOR_35      .T..A..... .......... .......... .......... .......... .......... T.........
SMS-3-5      .T..A..A.. .......... .......... .......... .......... .......... T.........
536          .T..A..... .......... .......... .C..T..... ......AG.. .......... .....T...T
APEC_O1      .T..A..... .......... .......... .C..T..... ......AG.. .......... .....T...T
CFT073       .T..A..... .......... .......... .......... .......G.. .......... ..........
E2348/69     .T..A..... .......... .......... .......... .......G.. .......... ..........
ECOR_51      .T..A..... .......... .......... .......... .......G.. .......... ..........
ECOR_57      .T..A..... .......... .......... .......... .......G.. .......... ..........
UTI89        .T..A..... .......... .......... .C..T..... ......AG.. .......... .....T...T
E._albertii  ---------- ---------- ---------- ---------- ---------- ---------- ----------

           701
K-12         ACGTTAAGAG CATAAAAATG AAGGCGTTCG ATCTCCACCG TATGGCATTT GATAAAGTGC CTTTTGATTT
53638        .......... .......... .......... .......... .......... .......... ..........
ATCC_8739    .......... .......... .......... .......... .......... .......... ..........
ECOR_10      .......... .......... .......... .......... .......... .......... ..........
ECOR_11      .......... .......... .......... ......T... .......... .......... ..........
B171         ...A...... .......... .......... .......... .......... .......... ..........
E24377A      ...A...... .......... .......... .......... .......... .......... ..........
ECOR_34      ...A...... .......... .......... .......... .......... .......... ..........
ECOR_71      ...A...... .......... .......... .......... .......... .......... ..........
SE11         ...A...... .......... .......... .......... .......... .......... ..........
O157_Sakai   .......... .......... .......... .......... .......... .......... ..........
ECOR_36      ...A...... .......... .......... .......... .......... .......... ..........
ECOR_35      ...A...... .......... .......... .......... .......... .......... ..........
SMS-3-5      ...A...... .......... .......... .......... .......... .......... ..........
536          ...A...... .......... ........T. .......... .........C .......... ..........
APEC_O1      ...A...... .......... ........T. .......... ...A.....C .......... ..........
CFT073       ...A...... .......... .......... .......... .......... .......... ..........
E2348/69     ...A...... .......... .......... .......... .......... .......... ..........
ECOR_51      ...A...... .......... .......... .......... .......... .......... ..........
ECOR_57      ...A...... .......... .......... .......... .......... .......... ..........
UTI89        ...A...... .......... ........T. .......... ...A.....C .......... ..........
E._albertii  ---------- ---------- ---------- ---------- ---------- ---------- ----------

           771
K-12         CCTTGGCGAA GTTGCACTAC GTAGTCTTTA TACCTTTGTA CTGGTCTTTT TGTTCCTCAA AATGACCGGA
53638        .......... .......... .......... .......... .......... .......... ..........
ATCC_8739    .......... .......... .......... .......... .......... .......... ..........
ECOR_10      .......... .......... .......... .......... .......... .......... ..........
ECOR_11      .......... .......... .......... .......... .......... .......... ..........
B171         .......... .......... .......... .......... .....T.... .......... ..........
E24377A      .......... .......... .......... .......... .....T.... .......... ..........
ECOR_34      .......... .......... .......... .......... .....T...C .......... ..........
ECOR_71      .......... .......... .......... .......... .....T.... .......... ..........
SE11         .......... .......... .......... .......... .....T...C .......... ..........
O157_Sakai   .......... .......... .......... .......... .......... .......... ..........
ECOR_36      .......... ..G....... .......... .......... .....T.... .......... ..........
ECOR_35      .......... ..G....... .......... .......... .....T.... .......... ..........
SMS-3-5      .......... ..G....... .......... .......... .....T.... .......... ..........
536          .......... .......... .......... .......A.. .......... .......... ..........
APEC_O1      .......... .......... .......... .......... .......... .......... ..........
CFT073       .......... .......... .......... .......... .....T.... .......... ..........
E2348/69     .......... .......... .......... .......... .....T.... .......... ..........
ECOR_51      .......... .......... .......... .......... .....T.... .......... ..........
ECOR_57      .......... .......... .......... .......... .....T.... .......... ..........
UTI89        .......... .......... .......... .......... .......... .......... ..........
E._albertii  ---------- ---------- ---------- ---------- ---------- ---------- ----------
```

```
             841
K-12          AGACGCGGTG TGCGGCAGAT GTCGCTGTTT GAGTTGTTAT ACCGCCTGGT AATGTGGTTG ATGGCGCACA
53638         .......... .......... .......... .......... .......... .......... ..........
ATCC_8739     .......... .......... .......... .......... .......... .......... ..........
ECOR_10       .......... .......... .......... .......... .......... .......... ..........
ECOR_11       .......... .......... .......... .......... .......... .......... ..........
B171          .......... ....A..... .......... .......... .......... .......... ..........
E24377A       .......... ....A..... .......... .......... .......... .......... ..........
ECOR_34       .......... .......... .......... .......... .......... .......... ..........
ECOR_71       .......... ....A..... .......... .......... .......... .......... ..........
SE11          .......... ....A..... .......... .......... .......... .......... ..........
O157_Sakai    .......... .......... .......... .......... .......... .......... ..........
ECOR_36       .......... .......... ......T... ..T....... .......... .......... ..........
ECOR_35       .......... .......... ......T... ..T....... .......... .......... ..........
SMS-3-5       .......... .......... ......T... .......... .......... .......... ..........
536           .......... .......... .......... .......... .......... .......... ..........
APEC_O1       .......... .......... .......... .......... .......... .......... ..........
CFT073        .......... .......... .......... .......... .......... .......... ..........
E2348/69      .......... .......... .......... .......... .......... .......... ..........
ECOR_51       .......... .......... .......... .......... .......... .......... ..........
ECOR_57       .......... .......... .......... .......... .......... .......... ..........
UTI89         .......... .......... .......... .......... .......... .......... ..........
E._albertii   ---------- ---------- ---------- ---------- ---------- ---------- ----------

             911
K-12          GTGAAAAACT GGAAGATCTT CTGGAAGGCA AGCCAGTTGT CATTATTGAA GATGGCGAGC TGGCCTGGTC
53638         .......... .......... .......... .......... .......... .......... ..........
ATCC_8739     .......... .......... .......... .......... .......... .......... ..........
ECOR_10       .......... .......... .......... .......... .......... .......... ..........
ECOR_11       .......... .......... .......... .......... .......... .......... ..........
B171          .......... .........A ........T. ....G..... T......... .......... ..........
E24377A       .......... .........A ........T. ....G..... T......... .......... ..........
ECOR_34       .......... .......T.A ........T. ....G..... T......... .......... ..........
ECOR_71       .......... .......T.A ........T. ....G..... T......... .......... ..........
SE11          .......... .......T.A ........T. ....G..... T......... .......... ..........
O157_Sakai    .......... .......... .......... .......... .......... .......... ..........
ECOR_36       .......... .......... .......... ....G..... .........G .....T.... ..........
ECOR_35       .......... .......... .......... ....G..... .........G .....T.... ..........
SMS-3-5       .......... .......... .......... ....G..... .......... .......... ..........
536           .......... .......... .......... ....G..... .......... .......... ..........
APEC_O1       .......... .......... .......... ....G..... .......... .......... ..........
CFT073        .......... .......... .......... ....G..... .......... .......... ..........
E2348/69      .......... .......... .......... ....G..... .......... .......... ..........
ECOR_51       .......... .......... .......... ....G..... .......... .......... ..........
ECOR_57       .......... .......... .......... ....G..... .......... .......... ..........
UTI89         .......... .......... .......... ....G..... .......... .......... ..........
E._albertii   ---------- ---------- ---------- ---------- ---------- ---------- ----------

             981
K-12          GAAACTCAAT AACTCCAACA TGACGGAATT TGAGTTCTTT ATGGAGCTAC GATTGCGTGG CGTGGAGCAG
53638         .......... .......... .......... .......... .......... .......... ..........
ATCC_8739     .......... .......... .......... .......... .......... .......... ..........
ECOR_10       .......... .......... .......... .......... .......... .......... ..........
ECOR_11       .......... .......... .......... .......... .......... .......... ..........
B171          .......... .......... .......... .......... .......... .......... ..........
E24377A       .......... .......... .......... .......... .......... .......... ..........
ECOR_34       .......... .......... .......... .......... .......... .......... ..........
ECOR_71       .......... .......... .......... .......... .......... .......... ..........
SE11          .......... .......... .......... .......... .......... .......... ..........
O157_Sakai    .......... .......... .......... .......... .......... .......... ..........
ECOR_36       ......T... .......... .......... .......... ........G. ..C....... A.........
ECOR_35       ......T... .......... .......... .......... ........G. ..C....... A.........
SMS-3-5       A........C .......... .......... .......... .......... .......C.. T.....A...
536           .......... .......... .......... .......... .......... .......C.. T..A......
APEC_O1       .......... .......... .......... .......... .......... .......C.. T..A......
CFT073        .......... .......... .......... .......... .......... .......C.. T..A......
E2348/69      .......... .......... .......... .......... .......... .......C.. T..A......
ECOR_51       .......... .......... .......... .......... .......... .......C.. T..A......
ECOR_57       .......... .......... .......... .......... .......... .......C.. T..A......
UTI89         .......... .......... .......... .......... .......... .......C.. T..A......
E._albertii   ---------- ---------- ---------- ---------- ---------- ---------- ----------
```

```
          1051
K-12        CTGGGGCAGG TACGTCTGGC GATTCTCGAA ACCAACGGGC AAATCAGTGT CTATTTCTTT GAAGATGACA
53638       .......... .......... .......... .......... .......... .......... ..........
ATCC_8739   .......... .......... .......... .......... .......... .......... ..........
ECOR_10     .......... .......... .......... .......... .......... .......... ..........
ECOR_11     .......... .......... .......... .......... .......... .......... ..........
B171        .......... .......... .......... .......... .......... .......... ..........
E24377A     .......... .......... .......... .......... .......... .......... ..........
ECOR_34     .......... .......... .......... .......... .......... .......... ..........
ECOR_71     .......... .......... .......... .......... .......... .......... ..........
SE11        .......... .......... .......... .......... .......... .......... ..........
O157_Sakai  .......... .......... .......... .......... .......... .......... ..........
ECOR_36     T......... .......... .......... .......... .......... .......... ..........
ECOR_35     T......... .......... .......... .......... .......... .......... ..........
SMS-3-5     .......... .......... .......... .......... .......... T......... ..........
536         T......... .......... .......... .......... .......... T......... ..........
APEC_O1     T......... .......... .......... .......... .......... T......... ..........
CFT073      T......... .......... .......... .......... .......... T......... ..........
E2348/69    T......... .......... .......... .......... .......... T......... ..........
ECOR_51     T......... .......... .......... .......... .......... T......... ..........
ECOR_57     T......... .......... .......... .......... .......... T......... ..........
UTI89       T......... .......... .......... .......... .......... T......... ..........
E._albertii ---------- ---------- ---------- ---------- ---------- ---------- ----------

          1121
K-12        AGGTGAAACC GGGTTTACTT ATTTTACCCA GTGATTGTAC GCAGCGTTAC AAAGTGGTGC CGGAGTCGGC
53638       .......... .......... .......... .......... .......... .......... ..........
ATCC_8739   .......... .......... .......... .......... .......... .......... ..........
ECOR_10     .......... .......... .......... .......... .......... .......... ..........
ECOR_11     .......... .......... .......... .......... .......... .......... ..........
B171        .......... .......... .......... .......... .......... .......... ..........
E24377A     .......... .......... .......... .......... .......... .......... ..........
ECOR_34     .......... .......... .......... .......... .......... .......... ..........
ECOR_71     .......... .......... .......... .......... .......... .......... ..........
SE11        .......... .......... .......... .......... .......... .......... ..........
O157_Sakai  ........T. .......... .......... .......... .......... .......... ..........
ECOR_36     .......... .......... .......... .......... T......... .......A.. .C........
ECOR_35     .......... .......... .......... .......... T......... .......A.. .C........
SMS-3-5     .......... ...C...... .......... ....C..... T......... .......... ..........
536         .......... ...G...... .......... ....C..... T......... .......... ..........
APEC_O1     .......... ...A...... .......... ....C..... T......... .......... ..........
CFT073      .......... ...G...... .......... ....C..... T......... .......... ..........
E2348/69    .......... ...C...... .......... ....C..... T......... .......... ..........
ECOR_51     .......... ...G...... .......... ....C..... T......... .......... ..........
ECOR_57     .......... ...G...... .......... ....C..... T......... .......... ..........
UTI89       .......... ...A...... .......... ....C..... T......... .......... ..........
E._albertii ---------- ---------- ---------- ---------- ---------- ---------- ----------

          1191
K-12        GGACTATGCC TGTATTCGTT GTAGTGAAAT CATTCATATG AAAGCGGGGG AAAAACAATT ATGTCCGCGC
53638       .......... .......... .......... .......... .......... .......... ..........
ATCC_8739   .......... .......... .......... .......... .......... .......... ..........
ECOR_10     .......... .......... .......... .......... .......... .......... ..........
ECOR_11     .......... .......... .......... .......... .......... .......... ..........
B171        .......... .......... .......... .......... .......... .......... ..........
E24377A     .......... .......... .......... .......... .......... .......... ..........
ECOR_34     .......... .......... .......... .......... .......... .......... ..........
ECOR_71     .......... .......... .......... .......... .......... .......... ..........
SE11        .......... .......... .......... .......... .......... .......... ..........
O157_Sakai  .......... .......... .......... .......... .......... .......... ..........
ECOR_36     .......... ..C....... .C........ .......... ..C....... .......... ..........
ECOR_35     .......... ..C....... .C........ .......... ..C....... .......... ..........
SMS-3-5     .......... ..C..C.... .C........ .......... ..C....... .......... ..........
536         .......... ..C..C.... .C........ .......... ..C....... .......... ..........
APEC_O1     .......... ..C..C.... .C........ .......... ..C....... .......... ..........
CFT073      .......... ..C..C.... .C........ .......... ..C....... .......... ..........
E2348/69    .......... ..C..C.... .C........ .......... ..C....... ...G...... ..........
ECOR_51     .......... ..C..C.... .C........ .......... ..C....... .......... ..........
ECOR_57     .......... ..C..C.... .C........ .......... ..C....... .......... ..........
UTI89       .......... ..C..C.... .C........ .......... ..C....... .......... ..........
E._albertii ---------- ---------. ....C..... ......C... ..T....T.. .......... ......A.AT
```

```
          1261
K-12          TGTGCAAATC CAGAATGGAC GAAGGCAAGT CGGGCAAAAC GGGTGACCTG ACAGTAAAAA CATCGGCTTT
53638         .......... .......... .......... .......... .......... .......... ..........
ATCC_8739     .......... .......... .......... .......... .......... .......... ..........
ECOR_10       .......... .......... .......... .......... .......... .......... ..........
ECOR_11       .......... .......... .......... .......... .......... .......... ..........
B171          .......... .......... .......... .......... .......... .......... ..........
E24377A       .......... .......... .......... .......... .......... .......... ..........
ECOR_34       .......... .......... .......... .......... ......T... .......... ..........
ECOR_71       .......... .......... .......... .......... .......... .......... ..........
SE11          .......... .......... .......... .......... .......... .......... ..........
O157_Sakai    .......... .......... .......... .......... .......... .......... ..........
ECOR_36       .......... .......... .......... .......... .......G.. .......... ..........
ECOR_35       .......... .......... .......... .......... .......G.. .......... ..........
SMS-3-5       .......... .......... .......... .......... .......G.. .......... ..........
536           .......... .......... .......... .....T.... .......... ....CC.... ......T...
APEC_O1       .......... .......... .......... ..A..T.... .......... ....C..... ..........
CFT073        .......... .......... .......... .....T.... .......... ....CC.... ......T...
E2348/69      .......... .......... .......... .....T.... .......... ....CC.... ......T...
ECOR_51       .......... .......... .......... .....T.... .......... ....CC.... ......T...
ECOR_57       .......... .......... .......... .....T.... .......... ....CC.... ......T...
UTI89         .......... .......... .......... ..A..T.... .......... ....C..... ..........
E._albertii   ....T..... ...T..T... .....T.... T..AT..... ..A..G.... ....C..... T.....T...

          1331
K-12          TTGCTAATAA TCCGAGAGAT TCTTTTGTGT GATGCAAGCC ACATTTTTGC CCTCAACGGT TTTACTCATT
53638         .......... .......... .......... .......... .......... .......... ..........
ATCC_8739     .......... .......... .......... .......... .......... .......... ..........
ECOR_10       .......... .......... .......... .......... .......... .......... ..........
ECOR_11       .......... .......... .......... .......... .......... .......... ..........
B171          .......... .......... .......... .......... .......... .......... ..........
E24377A       .......... .......... .......... .......... .......... .......... ..........
ECOR_34       .......... .......... .......... .......... .......... .......... ..........
ECOR_71       .......... .......... .......... .......... .......... .......... ..........
SE11          .......... .......... .......... .......... .......... .......... ..........
O157_Sakai    .......... .......... .......... .......... .......... .......... ..........
ECOR_36       .......... .......... .......... .......... .......... .......... ..........
ECOR_35       .......... .......... .......... .......... .......... .......... ..........
SMS-3-5       .......... .......... .......... .......... .......... .......... ..........
536           ...AA..... .......... .......... ..C..GT... T......... .......... ..........
APEC_O1       .......... .......... .......... .......... .......... .......... ..........
CFT073        ...AA..... .......... .......... ..C..GT... T......... .......... ..........
E2348/69      ....A..... .......... .......... ..C..GT... T......... ......T... ..........
ECOR_51       ...AA..... .......... .......... ..C..GT... T......... .......... ..........
ECOR_57       ...AA..... .......... .......... ..C..GT... T......... .......... ..........
UTI89         .......... .......... .......... .......... .......... .......... ..........
E._albertii   .G.G...... .......... ...G...... ..C..GT... .......... .......... .....C....

          1401
K-12          GCGATGTGTG TCACTGAATG ATAAAACCGA TAGCCACAGG AATAATGTAT TACCTGTGGT CGCAATCGAT
53638         .......... .......... .......... .......... .......... .......... ..........
ATCC_8739     .......... .......... .......... .......... .......... .......... ..........
ECOR_10       .......... .......... .......... .......... .......... .......... ..........
ECOR_11       .......... .......... .......... .......... .......... .......... ..........
B171          .......... .......... .......... .......... .......... .......... ..........
E24377A       .......... .......... .......... .......... .......... .......... ..........
ECOR_34       .......... .......... .......... .......... .......... .......... ..........
ECOR_71       .......... .......... .......... .......... .......... .......... ..........
SE11          .......... .......... .......... .......... .......... .......... ..........
O157_Sakai    .......... .......... .......... .......... .......... .......... ..........
ECOR_36       .......... .......... .......... .......... .......... .......... ..........
ECOR_35       .......... .......... .......... .......... .......... .......... ..........
SMS-3-5       .......... .......... .......... .......... .......... .......... ..........
536           ......C... .......... .......... .......... .......... .G........ ......T...
APEC_O1       .......... .......... .......... .......T.. .......... .......... ..........
CFT073        ......C... .......... .......... .......... .......... .G........ ......T...
E2348/69      ......C... .......... .......... .......... .......... .G........ ......T...
ECOR_51       ......C... .......... .......... .......... .......... .R........ ......Y...
ECOR_57       ......C... .......... .......... .......... .......... .R........ ......Y...
UTI89         .......... .......... .......... .......T.. .......... .......... ..........
E._albertii   ...GC.C... C......... .......... ..T....... .......... CG..C.-... ..........
```

```
            1471
K-12           TGACCGCGGG TTAATAGCAA CGCAACGTGG TGAGGGGAAA TGGCTCAAAT CTTCAATTTT AGTTCTGGTC
53638          .......... .......... .......... .......... .......... .......... ..........
ATCC_8739      .......... .......... .......... .......... .......... .......... ..........
ECOR_10        .......... .......... .......... .......... .......... .......... ..........
ECOR_11        .......... .......... .......... .......... .......... .......... ..........
B171           .......... .......... .......... .......... .......... .......... ..........
E24377A        .......... .......... .......... .......... .......... .......... ..........
ECOR_34        .......... .......... .......... .......... .......... .......... ..........
ECOR_71        .......... .......... .......... .......... .......... .......... ..........
SE11           .......... .......... .......... .......... .......... .......... ..........
O157_Sakai     .......... .......... .......... .......... .......... .......... ..........
ECOR_36        .......... .......... .......... .......... .......... .......... ..........
ECOR_35        .......... .......... .......... .......... .......... .......... ..........
SMS-3-5        .......... .......... .......... .......... .......... .......... ..........
536            .......... .....G.... .......... .......... .......... .......... ..........
APEC_O1        .......... .......... .......... .......... .......... .......... ..........
CFT073         .......... .....G.... .......... .......... .......... .......... ..........
E2348/69       .......... .....G.... .......... .......... .......... .......... ..........
ECOR_51        .......... .....R.... .......... .......... .......... .......... ..........
ECOR_57        .......... .....R.... .......... .......... .......... .......... ..........
UTI89          .......... .......... .......... .......... .......... .......... ..........
E._albertii    ....T.T... .......... .......... .......... .......... .......... ..........

            1541
K-12           CGGCAATGCT ACCGGCAGAG GTGCTTAAAC AGGCTCAACA GGAACTGCGC GACTGGAACG GTCTTGGTAC
53638          .......... .......... .......... .......... .......... .......... ..........
ATCC_8739      .......... .......... .......... .......... .......... .......... ..........
ECOR_10        .......... .......... .......... .......... .......... .......... ..........
ECOR_11        .......... .......... .......... .......... .......... .......... ..........
B171           .......... .......... .......... .......... .......... .......... ..........
E24377A        .......... .......... .......... .......... .......... .......... ..........
ECOR_34        .......... .......... .......... .......... .......... .......... ..........
ECOR_71        .......... .......... .......... .......... .......... .......... ..........
SE11           .......... .......... .......... .......... .......... .......... ..........
O157_Sakai     .......... .....T.... .......... .......... .......... .......... ..........
ECOR_36        .......... .......... .......... .......... .......... .......... ..........
ECOR_35        .......... .......... .......... .......... .......... .......... ..........
SMS-3-5        .......... .......... .......... .......... .......... .......... ..........
536            .......... .......... .......... .......... .......... .......... ..........
APEC_O1        .......... .......... ......G... .......... .......... .......... ..........
CFT073         .......... .......... .......... .......... .......... .......... ..........
E2348/69       .......... .......... .......... .......... .......... .......... ..........
ECOR_51        .......... .......... .......... .......... .......... .......... ..........
ECOR_57        .......... .......... .......... .......... .......... .......... ..........
UTI89          .......... .......... ......G... .......... .......... .......... ..........
E._albertii    .......... .........A .......... .......... .......... ........T. ....G.....

            1611
K-12           GTCGGTGATG GAAGTGAGTC ACCGTGGCAA AGAGTTCATT CAGGTTGCAG AGGAAGCCGA GAAGGATTTT
53638          .......... .......... .......... .......... .......... .......... ..........
ATCC_8739      .......... .......... .......... .......... .......... .......... ..........
ECOR_10        .......... .......... .......... .......... .......... .......... ..........
ECOR_11        .......... .......... .......... .......... .......... .......... ..........
B171           .......... .......... .......... .......... .......... .......... ..........
E24377A        .......... .......... .......... .......... .......... .......... ..........
ECOR_34        .......... .......... .......... .......... .......... .......... ..........
ECOR_71        .......... .......... .......... .......... .......... .......... ..........
SE11           .......... .......... .......... .......... .......... .......... ..........
O157_Sakai     .......... .......... .......... .......... .......... .......... ..........
ECOR_36        .......... .......... ....G..... .......... .......... .......... ..........
ECOR_35        .......... .......... ....G..... .......... .......... .......... ..........
SMS-3-5        .......... .......... ....G..... .......... .......... .......... ..........
536            .......... .......... .......... .......... .......... .......... ..........
APEC_O1        .......... .......... .......... .......... .......... .......... ..........
CFT073         .......... .......... .......... .......... .......... .......... ..........
E2348/69       .......... .......... .......... .......... .......... .......... ..........
ECOR_51        .......... .......... .......... .......... .......... .......... ..........
ECOR_57        .......... .......... .......... .......... .......... .......... ..........
UTI89          .......... .......... .......... .......... .......... .......... ..........
E._albertii    A......... ........C. .......... .......... .......... .......... ..........
```

```
           1681
K-12         CGCGATCTTC TTAATGTCCC CTCCAACTAC AAGGTATTAT TCTGCCATGG CGGTGGTCGC GGTCAGTTTG
53638        .......... .......... .......... .......... .......... .......... ..........
ATCC_8739    .......... .......... .......... .......... .......... .......... ..........
ECOR_10      .......... .......... .......... .......... .......... .......... ..........
ECOR_11      .......... .......... .......... .......... .......... .......... ..........
B171         .......... .......... .......... .......... .......... .......... ..........
E24377A      .......... .......... .......... .......... .......... .......... ..........
ECOR_34      .......... .......... .......... .......... .......... .......... ..........
ECOR_71      .......... .......... .......... .......... .......... .......... ..........
SE11         .......... .......... .......... .......... .......... .......... ..........
O157_Sakai   .......... .......... .......... .......... .......... .......... ..........
ECOR_36      .......... .......... .......... ........G. .......... ...C...... ..........
ECOR_35      .......... .......... .......... ........G. .......... ...C...... ..........
SMS-3-5      .......... .......... .......... ........G. .......... ...C...... ..........
536          .......... ....C..... .......... ........G. .......... .......... .....A....
APEC_O1      .......... ....C..... .......... ........G. .......... .......... .....A....
CFT073       .......... ....C..... .......... ........G. .......... .......... .....A....
E2348/69     .......... ....C..... .......... ..A.....G. .......... .......... .....A....
ECOR_51      .......... .......... .......... .......... .......... .......... ..........
ECOR_57      .......... .......... .......... .......... .......... .......... ..........
UTI89        .......... ....C..... .......... ........G. .......... .......... .....A....
E._albertii  .......... ....C..... .......... ..A.....G. .......C.. ...C..C... ..........

           1751
K-12         CTGCGGTACC GCTGAATATT CTCGGTGATA AAACCACCGC AGATTATGTT GATGCCGGTT ACTGGGCGGC
53638        .......... .......... .......... .......... .......... .......... ..........
ATCC_8739    .......... .......... .......... .......... .......... .......... ..........
ECOR_10      .......... .......... .......... .......... .......... .......... ..........
ECOR_11      .......... .......... .......... .......... .......... .......... ..........
B171         .......... .......... .......... .......... .......... .......... ..........
E24377A      .......... .......... .......... .......... .......... .......... ..........
ECOR_34      .......... .......... .......... .......... .......... .......... ..........
ECOR_71      .......... .......... .......... .......... .......... .......... ..........
SE11         .......... .......... .......... .......... .......... .......... ..........
O157_Sakai   .......... .......... .......... .......... .......... .......... ..........
ECOR_36      ....T..T.. .......... .......... .......T.. .......... .......... ..........
ECOR_35      ....T..T.. .......... .......... .......T.. .......... .......... ..........
SMS-3-5      ....T..T.. .T........ .......... .......T.. .......... .......... ..........
536          ....T..... .......... ..T....... .......T.. .......... .....G.... .......A..
APEC_O1      ....T..... .......... ..T....... .......T.. .......... .....G.... .......A..
CFT073       ....T..... .......... ..T....... .......T.. .......... .....G.... .......A..
E2348/69     ....A..... .T........ ..T....... .......T.. .......... .....G.... .......A..
ECOR_51      .......... .......... .......... .......... .......... .......... ..........
ECOR_57      .......... .......... .......... .......... .......... .......... ..........
UTI89        ....T..... .......... ..T....... .......T.. .......... .....G.... .......A..
E._albertii  ....T..... .T........ ..T....... .......... .........C .......... ..........

           1821
K-12         AAGTGCCATT AAAGAAGCGA AAAAATACTG CACGCCTAAT GTCTTTGACG CCAAAGTGAC TGTTGATGGT
53638        .......... .......... .......... .......... .......... .......... ..........
ATCC_8739    .......... .......... .......... .......... .......... .......... ..........
ECOR_10      .......... .......... .......... .......... .......... .......... ..........
ECOR_11      .......... .......... .......... .......... .......... .......... ..........
B171         .......... .......... .......... .......... .......... .......... ..........
E24377A      .......... .......... .......... .......... .......... .......... ..........
ECOR_34      .......... .......... .......... .......... .......... .......... ..........
ECOR_71      .......... .......... .......... .......... .......... .......... ..........
SE11         .......... .......... .......... .......... .......... .......... ..........
O157_Sakai   .......... .......... .......... .......... .......... .......... ..........
ECOR_36      .......... .......... .......... .......... .......... .......... ..........
ECOR_35      .......... .......... .......... .......... .......... .......... ..........
SMS-3-5      .......... .......... .......... .......... .......... .......... ..........
536          ......T..C .......... .......... ...A..A... ..G.....T. .G........ C.........
APEC_O1      ......T..C .......... .......... ...A..A... ..G.....T. .G........ C.........
CFT073       ......T..C .......... .......... ...A..A... ..G.....T. .G........ C.........
E2348/69     ......T..C .......... .......... ...A..A... ..G.....T. .G........ C.........
ECOR_51      .......... .......... .......... .......... .......... .......... ..........
ECOR_57      .......... .......... .......... .......... .......... .......... ..........
UTI89        ......T..C .......... .......... ...A..A... ..G.....T. .G........ C.........
E._albertii  C......... ........C. .......... ......A... .......... .T........ C.....C...
```

```
             1891
K-12           C-TCGCGCGG TTAAGCCAAT GCGTGAATGG CAACTCTCTG ATAATGCTGC TTATATGCAT TATTGCCCGA
53638          .......... .......... .......... .......... .......... .......... ..........
ATCC_8739      .......... .......... .......... .......... .......G.. .......... ..C.......
ECOR_10        .......... .......... .......... .......... .......... .......... ..........
ECOR_11        .......... .......... .......... .......... .......... .......... ..........
B171           .......... .......... .......... .......... .......... .......... ..C.......
E24377A        .......... .......... .......... .......... .......... .......... ..C.......
ECOR_34        .......... .......... .......... .......... .......... .......... ..C.......
ECOR_71        .......... .......... .......... .......... .......... .......... ..C.......
SE11           .......... .......... .......... .......... .......... .......... ..C.......
O157_Sakai     .......... .......... .......... .......... .......... .......... ..C.......
ECOR_36        .......... .......... ...A...... ..G....... .......... .......... ..C.......
ECOR_35        .......... .......... ...A...... .......... .........T .......... ..C.......
SMS-3-5        .......... .......... ...C...... .......... .......... .......... ..........
536            ........A. .......... .A........ .......... .......... .......... ..C.......
APEC_O1        ........A. .......... .A........ .......... .......... .......... ..C.......
CFT073         ........A. .......... .A........ .......... .......... .......... ..C.......
E2348/69       T.......A. .......... .A........ .......... .......... .......... ..C..T....
ECOR_51        ........A. .......... .A........ .......... .......... .......... ..C.......
ECOR_57        ........A. .......... .A........ .......... .......... .......... ..C.......
UTI89          ........A. .......... .A........ .......... .......... .......... ..C.......
E._albertii    .T........ ....A..... .......... .G........ .......... .......... ..........

             1961
K-12           ATGAAACCAT CGATGGTATC GCCATCGACG AAACGCCAGA CTTCGGCGCA GATGTGGTGG TCGCCGCTGA
53638          .......... .......... .......... .......... .......... .......... ..........
ATCC_8739      .......... ...C...... .......... .G........ .......AA. .......... .......C..
ECOR_10        .......... .......... .......... .......... .......... .......... ..........
ECOR_11        .......... .......... .......... .......... .......... .......... ..........
B171           .......... ...C...... .......... .......... .......AA. .......... .......C..
E24377A        .......... ...C...... .......... .......... .......AA. .......... .......C..
ECOR_34        .......... ...C...... .......... .......... .......AA. .......... .......C..
ECOR_71        .......... ...C...... .......... .......... .......AA. .......... .......C..
SE11           .......... ...C...... .......... .......... .......AA. .......... .......C..
O157_Sakai     .......... ...C...... .......... .......... .......AA. .......... .......C..
ECOR_36        .......... ...C...... .......... .......... .......AA. .......... .......C..
ECOR_35        .......... ...C...... .......... .......... .......AA. .......... .......C..
SMS-3-5        .......... ...C...... .......... .......... .......AA. .......... .......C..
536            .C........ T..C...... ..T.....T. ....A..... ......TAA. ..C....... ..........
APEC_O1        .C........ T..C...... ..T.....T. ....A..... ......TAA. ..C....... ..........
CFT073         .C........ T..C...... ..T.....T. ....A..... ......TAA. ..C....... ..........
E2348/69       .......... T..C...... .......... ........A. ......TAA. .......... .......C..
ECOR_51        .C........ T..C...... ..T.....T. ....A..... ......TAA. ..C....... ..........
ECOR_57        .C........ T..C...... ..T.....T. ....A..... ......TAA. ..C....... ..........
UTI89          .C........ T..C...... ..T.....T. ....A..... ......TAA. ..C....... ..........
E._albertii    .......... ...C...... .....T..T. .......... .......AA. .....C.... .T..T..C..

             2031
K-12           CTTCTCTTCA ACCATTCTTT CCCGTCCGAT TGACGTCAGC CGTTATGGTG TAATTTACGC TGGCGCGCAG
53638          .......... .......... .......... .......... .......... .......... ..........
ATCC_8739      .......... .......... .......... .......... .......... .......... ..........
ECOR_10        .......... .......... .......... .......... .......... .......... ..........
ECOR_11        .......... .......... .......... .......... .......... .......... ..........
B171           .......... .......... .......... .......... ........C. .G........ ..........
E24377A        .......... .......... .......... .......... ........C. .G........ ..........
ECOR_34        .......... .......... .......... .......... ........C. .G........ ..........
ECOR_71        .......... .......... .......... .......... ........C. .G........ ..........
SE11           .......... .......... .......... .......... ........C. .G........ ..........
O157_Sakai     .......... .......... .......... .......... .......... .......... ..........
ECOR_36        .......... .......... .......... ...T...... ........G. .......... ..........
ECOR_35        .......... .......... .......... ...T...... ........G. .......... ..........
SMS-3-5        .......... .......... .......... .......... ........C. .G........ ..........
536            .......... .......... .......... ...T...... .......... .......... ..........
APEC_O1        .......... .......... .......... ...T...... .......... .......... ..........
CFT073         .......... .......... .......... ...T...... .......... .......... ..........
E2348/69       .......... .......... .......... ...T...... .......... .......... ..........
ECOR_51        .......... .......... .......... ...T...... .......... .......... ..........
ECOR_57        .......... .......... .......... ...T...... .......... .......... ..........
UTI89          .......... .......... .......... ...T...... .......... .......... ..........
E._albertii    .......... ..G....... .G........ A..T.....T ........A. .......T.. G.........
```

```
          2101
K-12         AAAAATATCG GCCCGGCTGG CCTGACAATC GTCATCGTTC GTGAAGATTT GCTGGGCAAA GCGAATATCG
53638        .......... .......A.. .......... .......... .......... .......... ..........
ATCC_8739    .......... .......... .......... .......... .......... .......... ..........
ECOR_10      .......... .......... .......... .......... .......... .......... ..........
ECOR_11      .......... .......... .......... .......... .......... .......... ..........
B171         .......... .......... .......... .......... .......... .......... ..........
E24377A      .......... .......... .......... .......... .......... .......... ..........
ECOR_34      .......... .......... .......... .......... .......... .......... ..........
ECOR_71      .......... .......... .......... .......... .......... .......... ..........
SE11         .......... .......... .......... .......... .......... .......... ..........
O157_Sakai   .......... .......A.. .......... ........C. .......... .......... ......G...
ECOR_36      .......... .......... .......... .......... .......... .......... ..........
ECOR_35      .......... .......... .......... .......... .......... .......... ..........
SMS-3-5      .......... .......A.. .......... .......... .......... .......... ..........
536          .......... .......... .......... .......... .......... .......... ..........
APEC_O1      .......... .......... .......... .......... .......... .......... ..........
CFT073       .......... .......... .......... .......... .......... .......... ..........
E2348/69     .......... .......... ...T...... .......... .......... .......... ..........
ECOR_51      .......... .......... .......... .......... .......... .......... ..........
ECOR_57      .......... .......... .......... .......... .......... .......... ..........
UTI89        .......... .......... .......... .......... .......... .......... ..........
E._albertii  .......... .T.....G.. T......... .....T.... .C.....C.. .T....T... ..A.......

          2171
K-12         CGTGTCCGTC GATTCTGGAT TATTCCATCC TCAACGATAA CGGCTCCATG TTTAACACGC CGCCGACATT
53638        .......... .......... .......... .......... ..A....... .......... ..........
ATCC_8739    .......... .......... .......... .......... .......... .......... ..........
ECOR_10      .......... .......... .......... .......... .......... .......... ..........
ECOR_11      .......... .......... .......... .......... .......... .......... ..........
B171         .......... .......... .......... .......... .......... .......... ..........
E24377A      .......... .......... .......... .......... .......... .......... ..........
ECOR_34      .......... .......... .......... .......... .......... .......... ..........
ECOR_71      .......... .......... .......... .......... ..A....... .......... ..........
SE11         .......... .......... .......... .......... .......... .......... ..........
O157_Sakai   .......... .......... .......... .......... .......... .......... ..........
ECOR_36      .C..C..A.. .......... ..C....... .......... ..AT...... ..C....... ..........
ECOR_35      .C..C..A.. .......... ..C....... .......... ..AT...... ..C....... ..........
SMS-3-5      .......... .......... .......... .......... .......... .......... ..........
536          .C..C..A.. .......... ..C....... .......... ..AT...... ..C....... ..........
APEC_O1      .C..C..A.. .......... ..C....... .......... ..AT...... ..C....... ..........
CFT073       .C..C..A.. .......... ..C....... .......... ..AT...... ..C....... ..........
E2348/69     .C..C..A.. .......... ..C....... .......... ..AT...... ..C....... ..........
ECOR_51      .C..C..A.. .......... ..C....... .......... ..AT...... ..C....... ..........
ECOR_57      .C..C..A.. .......... ..C....... .......... ..AT...... ..C....... ..........
UTI89        .C..C..A.. .......... ..C....... .......... ..AT...... ..C....... ..........
E._albertii  .T..C..A.. .......... ........T. .......... ..A....... .......... .......C..

          2241
K-12         TGCCTGGTAT CTATCTGGTC TGGTCTTTAA ATGGCTGAAA GCGAACGGCG GTGTAGCTGA AATGGATAAA
53638        .......... ..G....... .......... .......... .......... .........C ..........
ATCC_8739    .......... .......... .......... .......... .......... .......... ..........
ECOR_10      .......... .......... .......... .......... .......... .......... ..........
ECOR_11      .......... .......... .......... .......... .......... .......... ..........
B171         .......... .......... .......... .......... .......... .......... ..........
E24377A      .......... .......... .......... ........G. .......... .......... ..........
ECOR_34      .......... .......... .......... .......... .......... .......... ..........
ECOR_71      .......... .......... .......... .......... .......... .......... ..........
SE11         .......... .......... .......... .......... .......... .......... ..........
O157_Sakai   .......... .......... .......... .......... .......... .......... ..........
ECOR_36      .......... ..G....... .......... .......... .......... .........C ..........
ECOR_35      .......... ..G....... .......... .......... .......... .........C ..........
SMS-3-5      .......... ..G....... .......... .......... .......... .......... ..........
536          .........C T.......C. .......C.. .......... .......... ....G....C .........G
APEC_O1      .........C T.......C. .......C.. .......... .......... ....G....C .........G
CFT073       .........C T.......C. .......C.. .......... .......... ....G....C .........G
E2348/69     .........C T.......C. .......C.. .......... .......... ....G.T..C .........G
ECOR_51      .........C T.......C. .......C.. .......... .......... ....G....C .........G
ECOR_57      .........C T.......C. .......C.. .......... .......... ....G....C .........G
UTI89        .........C T.......C. .......C.. .......... .......... ....G....C .........G
E._albertii  .........C T.......C. ....T..C.. .........G .......... ....G..A.C G.........
```

```
           2311
K-12        ATCAATCAGC AAAAAGCAGA ACTGCTATAT GGGGTGATTG ATAACAGCGA TTTCTACCGC AATGACGTGG
53638       .......... .......... ......G... .......... .......... .......... ..........
ATCC_8739   .......... .......... .......... .......... .......... .......... ..........
ECOR_10     .......... .......... .......... .......... .......... .......... ..........
ECOR_11     .......... .......... .......... .......... .......... .......... ..........
B171        .......... .......... .......... .......... .......... .......... ..........
E24377A     .......... .......... .......... .......... .......... .......... ..........
ECOR_34     .......... .......... .......... .......... .......... .......... ..........
ECOR_71     .......... .......... .......... .......... .......... .......... ..........
SE11        .......... .......... .......... .......... .......... .......... ..........
O157_Sakai  .......... .......... .......... .......... .......... .......... ..........
ECOR_36     .......... .......... ......G... .......... .......... .......... ..........
ECOR_35     .......... .......... ......G... .......... .......... .......... ..........
SMS-3-5     .......... .......... ......G... .......... .......... .......... ..........
536         .......... .......... ......G... .......... .......... .......... .....T....
APEC_O1     .......... .......... ......G... .......... .......... .......... .....T....
CFT073      .......... .......... ......G... .......... .......... .......... .....T....
E2348/69    .......... .......... ......G... .......... .......... .......... .....T....
ECOR_51     .......... .......... ......G... .......... .......... .......... .....T....
ECOR_57     .......... .......... ......G... .......... .......... .......... .....T....
UTI89       .......... .......... ......G... .......... .......... .......... .....T....
E._albertii .......... .......C.. ......G..C ..T....... .......... .........T .....T....

           2381
K-12        CGAAAGCTAA CCGTTCGCGG ATGAACGTGC CGTTCCAGTT GGCGGACAGT GCGCTTGACA AATTGTTCCT
53638       ......T... .......... ........A. .......... ...A..T... .......... ..........
ATCC_8739   .......... .......... .......... .......... .......... .......... ..........
ECOR_10     .......... .......... .......... .......... .......... .......... ..........
ECOR_11     .......... .......... .......... .......... .......... .......... ..........
B171        .......... .......... .......... .......... .......... .......... ..........
E24377A     .......... .......... .......... .......... .......... .......... ..........
ECOR_34     .......... .......... .......... .......... .......... .......... ..........
ECOR_71     .......... .......... .......... .......... .......... .......... ..........
SE11        .......... .......... .......... .......... .......... .......... ..........
O157_Sakai  .......... .......... .......... .......... .......... .......... ..........
ECOR_36     .......... .......... .......... .......... .......... .......... ..........
ECOR_35     .......... .......... .......... .......... .......... .......... ..........
SMS-3-5     .......... .......... .......... .......... .......... .......... ..........
536         .......... .......... .......... .......... ......T..C ........T. ..........
APEC_O1     .......... .......... .......... .......... ......T..C ........T. ..........
CFT073      .......... .......... .......... .......... ......T..C ........T. ..........
E2348/69    .......... .......... .......... .......... ......T..C ........T. ..........
ECOR_51     .......... .......... .......... .......... ......T..C ........T. ..........
ECOR_57     .......... .......... .......... .......... ......T..C ........T. ..........
UTI89       .......... .......... .......... .......... ......T..C ........T. ..........
E._albertii .......... .......... ........C. .A........ A..A...... ..T..G..T. ..C.......

           2451
K-12        TGAAGAGTCT TTTGCTGCTG GCCTTCATGC ACTGAAAGGT CACCGTGTGG TCGGCGGAAT GCGCGCTTCT
53638       .......... ........A. .......... .......... .......... .......... ......G...
ATCC_8739   .......... .......... .......... .......... .......... .......... ......G...
ECOR_10     .......... .......... .......... .......... .......... .......... ..........
ECOR_11     .......... .......... .......... .......... .......... .......... ..........
B171        .......... .......... .......... .......... .......... .......... ..........
E24377A     .......... .......... .......... .......... .......... .......... ..........
ECOR_34     .......... .......... .......... .......... .......... .......... ..........
ECOR_71     .......... .......... .......... .......... .......... .......... ..........
SE11        .......... .......... .......... .......... .......... .......... ..........
O157_Sakai  .......... ........A. .......... .......... .......... .......... ......G...
ECOR_36     .......... .......... .......... G......... .......... .......... ......G...
ECOR_35     .......... .......... .......... G......... .......... .......... ......G...
SMS-3-5     .......... .......... .......... G......... .......... .......... ...T..G...
536         C......... ..C.....G. ..T.A..... G......... .....A.... .......... ...T..G...
APEC_O1     C......... ..C.....G. ..T.A..... G......... .....A.... .......... ...T..G...
CFT073      C......... ..C.....G. ..T.A..... G......... .....A.... .......... ...T..G...
E2348/69    C......... ..C.....G. ..T.A..... G......... .....A.... .......... ...T......
ECOR_51     C......... ..C.....G. ..T.A..... G......... .....A.... .......... ...T..G...
ECOR_57     C......... ..C.....G. ..T.A..... G......... .....A.... .......... ...T..G...
UTI89       C......... ..C.....G. ..T.A..... G......... .....A.... .......... ...T..G...
E._albertii C......... ..C..C..G. .......... G......... .......... ....T..... ...T......
```

```
            2521
K-12        ATTTATAACG CCATGCCGCT GGAAGGCGTT AAAGCGCTGA CAGACTTCAT GGTTGAGTTC GAACGCCGTC
53638       .......... .......... .......... .......... .......... .......... ..........
ATCC_8739   .......... .......... .......... .......... .......... .......... ..........
ECOR_10     .......... .......... .......... .......... .......... .......... ..........
ECOR_11     .......... .......... .......... .......... .......... .......... ..........
B171        .......... .......... .......... .......... .......... .......... ..........
E24377A     .......... .......... .......... .......... .......... .......... ..........
ECOR_34     .......... .......... .......... .......... .......... .......... ..........
ECOR_71     .......... .......... .......... .......... .......... .......... ..........
SE11        .......... .......... .......... .......... .......... .......... ..........
O157_Sakai  ........T. .......... .......... .......... .......... .......... ..........
ECOR_36     .......... .......... .......... .......... .......... .......... ..........
ECOR_35     .......... .......... .......... .......... .......... .......... ..........
SMS-3-5     .......... .......A.. .......... .......... .......... .......... ..........
536         .......... .......... ......T... .....A.... .......... .......... .....T....
APEC_O1     .......... .......... ......T... .....A.... .......... .......... .....T....
CFT073      .......... .......... ......T... .....A.... .......... .......... .....T....
E2348/69    .......... .......... ......T... .....A.... .......... .......... .....T....
ECOR_51     .......... .......... ......T... .....A.... .......... .......... .....T....
ECOR_57     .......... .......... ......T... .....A.... .......... .......... .....T....
UTI89       .......... .......... ......T... .....A.... .......... .......... .....T....
E._albertii .......... .......... .......... .......... .......... ......A... .....T....

            2591
K-12        ACGGTTAATG CCGAAATTTT GCTTAATCCC CACAGCCAGC CTGTGGGGTT TTTATTTCTG TTGTAGAGAG
53638       ....C..... .......... .......... .......... .......... .......... ....T.....
ATCC_8739   ....C..... .......... .......... .......... .......... .......... ..........
ECOR_10     .......... .......... .......... .......... .......... .......... ..........
ECOR_11     .......... .......... .......... .......... .......... .......... ..........
B171        .......... .......... .......... .......... .......... .......... ..........
E24377A     .......... .......... .......... .......... .......... .......... ..........
ECOR_34     .......... .......... .......... .......... .......... .......... ..........
ECOR_71     .......... .......... .......... .......... .......... .......... ..........
SE11        .......... .......... .......... .......... .......... .......... ..........
O157_Sakai  .......... .......... .......... .......... .......... .......... ..........
ECOR_36     ....C..... .......... .......... .......... .......... .......... ..........
ECOR_35     ....C..... .......... .......... .......... .......... .......... ..........
SMS-3-5     ....C..... T......... .......... .......... .......... .......... ..........
536         ....C..... T......... ..A....... .......... .......... .......... ..........
APEC_O1     ....C..... T......... ..A....... .......... .......... .......... ..........
CFT073      ....C..... T......... ..A....... .......... .......... .......... ..........
E2348/69    ....C..... T......... ..A....... .......... .......... .......... ..........
ECOR_51     ....C..... T......... ..A....... .......... .......... .......... ..........
ECOR_57     ....C..... T......... ..A....... .......... .......... .......... ..........
UTI89       ....C..... T......... ..A....... .......... .......... .......... ..........
E._albertii ....C..... .......... .......... .........A .......... .......... ..........

            2661
K-12        TTGAGTTCAT GGAATCCCTG ACGTTACAAC CCATCGCTCG TGTCGATGGC ACTATTAATC TGCCCGGTTC
53638       .......... .......... .......... .......... .......... .......... ..........
ATCC_8739   .......... .......... .......... .......... .......... .......... ..........
ECOR_10     .......... .......... .......... .......... .......... .......... ..........
ECOR_11     .......... .......... .......... .......... .......... .......... ..........
B171        .......... .......... .......... .......... .......... .......... ..........
E24377A     .......... .......... .......... .......... .......... .......... ..........
ECOR_34     .......... .......... .......... ........A. .......... .......... ..........
ECOR_71     .......... .......... .......... .......... .......... .......... ..........
SE11        .......... .......... .......... .......... .......... .......... ..........
O157_Sakai  .......... .......... .......... .......... .......... .......... ..........
ECOR_36     .......... .......... .......... .......... .......... ..C....... ....T.....
ECOR_35     .......... .......... .......... .......... .......... ..C....... ....T.....
SMS-3-5     .......... .......... .......... .......C.. .......... .......... ..........
536         .......... .........A .......... .......... .......... .......... ..........
APEC_O1     .......... .........A .......... .......... .......... .......... ..........
CFT073      .......... .........A .......... .......... .......... .......... ..........
E2348/69    .......... .........A .......... .......... .......... .......... ..........
ECOR_51     .......... .........A .......... .......... .......... .......... ..........
ECOR_57     .......... .........A .......... .......... .......... .......... ..........
UTI89       .......... .........A .......... .......... .......... .......... ..........
E._albertii .......... .......... .......... .TG....... .........T ..G....... ....T.....
```

```
            2731
K-12           CAAGAGCGTT TCTAACCGCG CTTTATTGCT GGCGGCATTA GCACACGGCA AAACAGTATT AACCAATCTG
53638          .......... .......... .......... .......... .......... .......... ..........
ATCC_8739      .......... .......... .......... .......... .......... .......... ..........
ECOR_10        .......... .......... .......... .......... .......... .......... ..........
ECOR_11        .......... .......... .......... .......... .......... .......... ..........
B171           .......... .......... .......... .......... .......... .......... ..........
E24377A        .......... .......... .......... .......... .......... .......... ..........
ECOR_34        .......... .......... .......... .......... .......... .......... ..........
ECOR_71        .......... .......... .......... .......... .......... .......... ..........
SE11           .......... .......... .......... .......... .......... .......... ..........
O157_Sakai     .......... .......... .......... .......... .......... .......... ..........
ECOR_36        .......... .......... .......... .......... .....T.... .......... ..........
ECOR_35        .......... .......... .......... .......... .....T.... .......... ..........
SMS-3-5        .......... .......... .......... .......... .....T.... .......... ..........
536            .......... .......... .......... .......... .......... .......... ..........
APEC_O1        .......... .......... .......... .......... .......... .......... ..........
CFT073         .......... .......... .......... .......... .......... .......... ..........
E2348/69       .......... .......... .......... .......... .......... .......... ..........
ECOR_51        .......... .......... .......... .......... .......... .......... ..........
ECOR_57        .......... .......... .......... .......... .......... .......... ..........
UTI89          .......... .......... .......... .......... .......... .......... ..........
E._albertii    .........A .......... ......AT.. .......... .......... .......... ..........

            2801
K-12           CTGGATAGCG ATGACGTGCG CCATATGCTG AATGCATTAA CAGCGTTAGG GGTAAGCTAT ACGCTTTCAG
53638          .......... .......... .......... .......... ...G...... .......... ..........
ATCC_8739      .......... .......... .......... .......... ...G...... .......... ..........
ECOR_10        .......... .......... .......... .......... .......... .......... ..........
ECOR_11        .......... .......... .......... .......... .......... .......... ..........
B171           .......... .......... .......... .......... .......... .......... ..........
E24377A        .......... .......... .......... .......... .......... .......... ..........
ECOR_34        .......... .......... .......... .......... .......... .......... ..........
ECOR_71        .......... .......... .......... .......... .......... .......... ..........
SE11           .......... .......... .......... .......... .......... .......... ..........
O157_Sakai     .......... .......... .......... .......... .......... .......... ..........
ECOR_36        .......... .......... .......... .........G .......... .......... ..........
ECOR_35        .......... .......... .......... .........G .......... .......... ..........
SMS-3-5        .......... ....T..T.. .T.......T .....G.... AG..A..... .......... ..........
536            .......... .......... .......... .......... .......... .......... ..........
APEC_O1        .......... .......... .......... .......... .......... .......... ..........
CFT073         .......... .......... .......... .......... .......... .......... ..........
E2348/69       .......... .......... .......... .......... .......... .......... ..........
ECOR_51        .......... .......... .......... .......... .......... .......... ..........
ECOR_57        .......... .......... .......... .......... .......... .......... ..........
UTI89          .......... .......... .......... .......... .......... .......... ..........
E._albertii    .......... ....T..T.. .........T .....G.... AG........ .......... ........T.

            2871
K-12           CCGATCGTAC GCGTTGCGAA ATTATCGGTA ACGGCGGTCC ATTACACGCA GAAGGTGCCC TGGAGTTGTT
53638          .......... .......... .......... .......... .......... .......... ..........
ATCC_8739      .......... .......... .......... .......... .......... .......... ..........
ECOR_10        .......... .......... .......... .......... .......... .......... ..........
ECOR_11        .......... .......... .......... .......... .......... .......... ..........
B171           .......... .......... .......... .......... .......... .......... ..........
E24377A        .......... .......... .......... .......... .......... .......... ..........
ECOR_34        .......... .......... .......... .......... .......... .......... ..........
ECOR_71        .......... .......... .......... .......... .......... .......... ..........
SE11           .......... .......... .......... .......... .......... .......... ..........
O157_Sakai     .......... A......... .......... .......... .......... .......... ..........
ECOR_36        .......... .......... ..C....... .......... .......... A..A...... ..........
ECOR_35        .......... .......... ..C....... .......... .......... A..A...... ..........
SMS-3-5        .......... .......... ..C....... .......... .......... A..A.C.... ..........
536            .......... .......... .......... ....T..... .......... ...A...... GT........
APEC_O1        .......... .......... .......... ....T..... .......... ...A...... G.........
CFT073         .......... .......... .......... ....T..... .......... ...A...... G.........
E2348/69       .......... .......... ..C....... .......... .......... A..A.....T ..........
ECOR_51        .......... .......... .......... ....T..... .......... ...A...... G.........
ECOR_57        .......... .......... .......... ....T..... .......... ...A...... G.........
UTI89          .......... .......... .......... ....T..... .......... ...A...... G.........
E._albertii    ....G.A... .......... ..C.....C. .T..T..C.. ......T..T .....C..GT .......A..
```

```
          2941
K-12        CCTCGGTAAC GCCGGAACGG CAATGCGTCC GCTGGCGGCA GCTCTTTGTC TGGGTAGCAA TGATATTGTG
53638       .......... .......... .......... .......... .......... .......... ..........
ATCC_8739   .......... .......... .......... .......... .......... .......... ..........
ECOR_10     .......... .......... .......... .......... .......... .......... ..........
ECOR_11     .......... .......... .......... .......... .......... .......... ..........
B171        .......... .......... .......... .......... .......... .......... ..........
E24377A     .......... .......... .......... .......... .......... .......... ..........
ECOR_34     .......... .......... .......... .......... .......... .......... ..........
ECOR_71     .......... .......... .......... .......... .......... .......... ..........
SE11        .......... .......... .......... .......... .......... .......... ..........
O157_Sakai  .......... .......... .......... .......... .......... .......... ..........
ECOR_36     .......... .......... .......... .......... .......... .......... ..........
ECOR_35     .......... .......... .......... .......... .......... .......... ..........
SMS-3-5     .......... .......... .......... .......... .......... .......... ..........
536         .......... .......... .......... .......... ..A.....C. .......... .........A
APEC_O1     .......... .......... .......... .......... ..A.....C. .......... .........A
CFT073      .......... .......... .......... .......... ..A.....C. .......... .........A
E2348/69    .......... .......... .......... .......... .......... .......... ..........
ECOR_51     .......... .......... .......... .......... ..A.....C. .......... .........A
ECOR_57     .......... .......... .......... .......... ..A.....C. .......... .........A
UTI89       .......... .......... .......... .......... ..A.....C. .......... .........A
E._albertii T........T .......... .......... A.....T..G ..A.....CT ....ACA... .......A.T

          3011
K-12        CTGACCGGTG AGCCGCGTAT GAAAGAACGC CCGATTGGTC ATCTGGTGGA TGCGCTGCGC CTGGGCGGGG
53638       .......... .......... .......... .......... .......... ...T...... ..........
ATCC_8739   .......... .......... .......... .......... .......... ...T...... ..........
ECOR_10     .......... .......... .......... .......... .......... .......... ..........
ECOR_11     .......... .......... .......... .......... .......... .......... ..........
B171        .......... .......... .......... .......... .......... .......... ..........
E24377A     .......... .......... .......... .......... .......... .......... ..........
ECOR_34     .......... .......... .......... .......... .......... .......... ..........
ECOR_71     .......... .......... .......... .......... .......... .......... ..........
SE11        .......... .......... .......... .......... .......... .......... ..........
O157_Sakai  .......... .......... ......G... .......... .......... .......... ..........
ECOR_36     .......... .A........ .......... ..C....... .......... .......... ..........
ECOR_35     .......... .A........ .......... ..C....... .......... .......... ..........
SMS-3-5     .......... .A........ .......... ..C....... .......... .......... ..........
536         .......... .A.....A.. .......... .......... .......... .......... .A........
APEC_O1     .......... .A.....A.. .......... .......... .......... .......... .A........
CFT073      .......... .A.....A.. .......... .......... .......... .......... .A........
E2348/69    .......... .A........ .......... ..C....... .......... .......... ..........
ECOR_51     .......... .A.....A.. .......... .......... .......... .......... .A........
ECOR_57     .......... .A.....A.. .......... .......... .......... .......... .A........
UTI89       .......... .A.....A.. .......... .......... .......... .......... .A........
E._albertii ........C. ....A..C.. .......... .......... .C........ .......... .A......A.

          3081
K-12        CGAAGATCAC TTACCTGGAA CAAGAAAATT ATCCGCCGTT GCGTTTACAG GGCGGCTTTA CTGGCGGCAA
53638       .......... .......... .......... .......... .......... .......... .C........
ATCC_8739   .......... .......... .......... .......... .......... .......... .C........
ECOR_10     .......... .......... .......... .......... .......... .......... ..........
ECOR_11     .......... .......... .......... .......... .......... .......... ..........
B171        .......... .......... .......... .......... .......... .......... ..........
E24377A     .......... .......... .......... .......... .......... .......... ..........
ECOR_34     .......... .......... .......... .......... .......... .......... ..........
ECOR_71     .......... .......... .......... .......... .......... .......... ..........
SE11        .......... .......... .......... .......... .......... .......... ..........
O157_Sakai  .......... .......... .......... .......... .......... .......... ..........
ECOR_36     .......... .......... .......... ....A..A.. .......... .......... ..........
ECOR_35     .......... .......... .......... ....A..A.. .......... .......... ..........
SMS-3-5     .......... .......... .......... ....A..A.. .......... .......... ..........
536         .......... .......... .......... .......A.. .......... .......... ..........
APEC_O1     .......... .......... .......... .......A.. .......... .......... ..........
CFT073      .......... .......... .......... .......A.. .......... .......... ..........
E2348/69    .......... .......... .......... ....A..A.. .......... .......... ..........
ECOR_51     .......... .......... .......... .......A.. .......... .......... ..........
ECOR_57     .......... .......... .......... .......A.. .......... .......... ..........
UTI89       .......... .......... .......... .......A.. .......... .......... ..........
E._albertii ....A..... .....A.... .....G.... ....A..... ........T. .....T..C. TC........
```

```
             3151
K-12         CGTTGACGTT GATGGCTCCG TTTCCAGCCA ATTCCTCACC GCACTGTTAA TGACTGCGCC TCTTGCGCCG
53638        .......... .......... .......... .......... .......... .......... ..........
ATCC_8739    .......... .......... .......... .......... .......... .......... ..........
ECOR_10      .......... .......... .......... .......... .......... .......... ..........
ECOR_11      .......... .......... .......... .......... .......... .......... ..........
B171         .......... .......... .......... .......... .......... .......... ..........
E24377A      .......... .......... .......... .......... .......... .......... ..........
ECOR_34      .......... .......... .......... .......... .......... .......... ..........
ECOR_71      .......... .......... .......... .......... .......... .......... ..........
SE11         .......... .......... .......... .......... .......... .......... ..........
O157_Sakai   .......... .......... .......... .......... .......... .......... ..........
ECOR_36      .......... .......... .......... G......... .......... .......A.. .......T..
ECOR_35      .......... .......... .......... G......... .......... .......A.. .......T..
SMS-3-5      .......... .......... .......... G......... .......... .......... ......A...
536          ......T... .......... .......... G......... .......... .......... ..........
APEC_O1      ......T... .......... .......... G......... .......... .......... ..........
CFT073       ......T... .......... .......... G......... .......... .......... ..........
E2348/69     .......... .......... .......... G......... .......... .......... ......A...
ECOR_51      ......T... .......... .......... G......... .......... .......... ..........
ECOR_57      ......T... .......... .......... G......... .......... .......... ..........
UTI89        ......T... .......... .......... G......... .......... .......... ..........
E._albertii  ......A... ..C.....T. .......T.. G..T...... ...T.A.... .......... G......T..

             3221
K-12         GAAGATACGG TGATTCGTAT TAAAGGCGAT CTGGTTTCTA AACCTTATAT CGACATCACA CTCAATCTGA
53638        .......... .......... .......... .......... .......... .......... ..........
ATCC_8739    .......... .......... .......... .......... .......... .......... ..........
ECOR_10      .......... .......... .......... .......... .......... .......... ..........
ECOR_11      .......... .......... .......... .......... .......... .......... ..........
B171         .......... .......... .......... .......... .......... .......... ..........
E24377A      .......... .......... .......... .......... .......... .......... ..........
ECOR_34      .......... .......... .......... .......... .......... .......... ..........
ECOR_71      .......... .......... .......... .......... .......... .......... ..........
SE11         .......... .......... .......... .......... .......... .......... ..........
O157_Sakai   .......... .......... .......... .......... .......... .......... ..........
ECOR_36      .......... .......... .......... .......... .......... .........C ..........
ECOR_35      .......... .......... .......... .......... .......... .........C ..........
SMS-3-5      .......... .......... .......... .....C.... .......... .......... ..........
536          .......... .......... .......... .......... .......... ...T.....C ..........
APEC_O1      .......... .......... .......... .......... .......... ...T.....C ..........
CFT073       .......... .......... .......... .......... .......... ...T.....C ..........
E2348/69     .......... .......... .......... .......... .......... .......... ..........
ECOR_51      .......... .......... .......... .......... .......... ...T.....C ..........
ECOR_57      .......... .......... .......... .......... .......... ...T.....C ..........
UTI89        .......... .......... .......... .......... .......... ...T.....C ..........
E._albertii  C......... .C.....C.. .......... .....C.... .G........ T..T.....G ..G..CA...

             3291
K-12         TGAAGACGTT TGGTGTTGAA ATTGAAAATC AGCACTATCA ACAATTTGTC GTAAAAGGCG GGCAGTCTTA
53638        .......... .......... .......... .......... .......... .......... ..........
ATCC_8739    .......... .......... .......... .......... .......... .......... ..........
ECOR_10      .......... .......... .......... .......... .......... .......... ..........
ECOR_11      .......... .......... .......... .......... .......... .......... ..........
B171         .......... .......... .......... .......... .......... .......... ..........
E24377A      .......... .......... .......... .......... .......... .......... ..........
ECOR_34      .......... .......... .......... .......... .......... .......... ..........
ECOR_71      .......... .......... .......... .......... .......... .......... ..........
SE11         .......... .......... .......... .......... .......... .......... ..........
O157_Sakai   .......... .......... .......... .......... .......... .......... ..........
ECOR_36      .......... .......... .......... .......... .......... ........G. ..........
ECOR_35      .......... .......... .......... .......... .......... ........G. ..........
SMS-3-5      .......... .......... .......... .......... .......... ..T....... ..........
536          .......... .......... .......... .......... .......... ........G. ..........
APEC_O1      .......... .......... .......... .......... .......... ........G. ..........
CFT073       .......... .......... .......... .......... .......... ........G. ..........
E2348/69     .......... .......... .......... .......... .......... ........G. ..........
ECOR_51      .......... .......... .......... .......... .......... ........G. ..........
ECOR_57      .......... .......... .......... .......... .......... ........G. ..........
UTI89        .......... .......... .......... .......... .......... ........G. ..........
E._albertii  ....AG.... .........G .......... .......C.. ...G.....G ..G....... ..........
```

```
            3361
K-12         TCAGTCTCCG GGTACTTATT TGGTCGAAGG CGATGCATCT TCGGCTTCTT ACTTTCTGGC AGCAGCAGCA
53638        .......... .......... .......... .......... .......... .......... ..........
ATCC_8739    .......... .......... .......... .......... .......... .......... ..........
ECOR_10      .......... .......... .......... .......... .......... .......... ..........
ECOR_11      .......... .......... .......... .......... .......... .......... ..........
B171         .......... .......... .......... .......... .......... .......... ..........
E24377A      .......... .......... .......... .......... .......... .......... ..........
ECOR_34      .......... .......... .......... .......... .......... .......... ..........
ECOR_71      .......... .......... .......... .......... .......... .......... ..........
SE11         .......... .......... .......... .......... .......... .......... ..........
O157_Sakai   .......... .......... .......... .......... .......... .......... ..........
ECOR_36      .......... .......... .......... .........C .......... .T..C..... ..........
ECOR_35      .......... .......... .......... .........C .......... .T..C..... ..........
SMS-3-5      .......... .......... .......... .......... .......... ....C..... ..........
536          .......... .......... .......... .........C .......... .T..C..... ..........
APEC_O1      C......... ..C....... ....T..... .......... .......... .T..C..... ..........
CFT073       C......... ..C....... ....T..... .......... .......... .T..C..... ..........
E2348/69     .......... .......... .......... .......... .......... ....C..... ..........
ECOR_51      C......... ..C....... ....T..... .......... .......... .T..C..... ..........
ECOR_57      C......... ..C....... ....T..... .......... .......... .T..C..... ..........
UTI89        C......... ..C....... ....T..... .......... .......... .T..C..... ..........
E._albertii  C..A...... ..G.GC.... .A..G..... T.....C..C .....C.... ....C..T.. C........G

            3431
K-12         ATCAAAGGCG GCACTGTAAA AGTGACCGGT ATTGGACGTA ACAGTATGCA GGGTGATATT CGCTTTGCTG
53638        .......... .......... .......... .......... .......... .......... ..........
ATCC_8739    .......... .......... .......... .......... .......... .......... ..........
ECOR_10      .......... .......... .......... .......... .......... .......... ..........
ECOR_11      .......... .......... .......... .......... .......... .......... ..........
B171         .......... .......... .......... .......... .......... .......... ..........
E24377A      .......... .......... .......... .......... .......... .......... ..........
ECOR_34      .......... .......... .......... .......... .......... .......... ..........
ECOR_71      .......... .......... .......... .......... .......... .......... ..........
SE11         .......... .......... .......... .......... .......... .......... ..........
O157_Sakai   .......... .......... .......... .......... .......... .......... ..........
ECOR_36      ....G..... .......... .........C ........C. .......... .......... ..........
ECOR_35      ....G..... .......... .........C ........C. .......... .......... ..........
SMS-3-5      .......... .......... .........C ........C. .......... .......... ..........
536          ....G..... .......... .........C ........C. .......... .......... ..........
APEC_O1      ....G..... .......... .........C ........C. .......... .......... ..........
CFT073       ....G..... .......... .........C ........C. .......... .......... ..........
E2348/69     .......... .......... .........C ........C. .......... .......... ..........
ECOR_51      ....G..... .......... .........C ........C. .......... .......... ..........
ECOR_57      ....G..... .......... .........C ........C. .......... .......... ..........
UTI89        ....G..... .......... .........C ........C. .......... .......... ..........
E._albertii  G.T....... .T..A..... .........C ........C. .......... A..C...... ..T.......

            3501
K-12         ATGTGCTGGA AAAAATGGGC GCGACCATTT GCTGGGGCGA TGATTATATT TCCTGCACGC GTGGTGAACT
53638        .......... .......... .......... .......... .......... .......... ..........
ATCC_8739    .......... .......... .......... .......... .......... .......... ..........
ECOR_10      .......... .......... .......... .......... .......... .......... ..........
ECOR_11      .......... .......... .......... .......... .......... .......... ..........
B171         .......... .......... .......... .......... .......... .......... ..........
E24377A      .......... .......... .......... .......... .......... .......... ..........
ECOR_34      .......... .......... .......... .......... .......... .......... ..........
ECOR_71      .......... .......... .......... .......... .......... .......... ..........
SE11         .......... .......... .......... .......... .......... .......... ..........
O157_Sakai   .......... .......... .......... .......... .......... .......... ..........
ECOR_36      .......... .........T .......... .......... .......... .......... ..........
ECOR_35      .......... .........T .......... .......... .......... .......... ..........
SMS-3-5      .......... .........T .......... .......... .......... .......... ..........
536          .......... .........T .......... .......... .......... .......... ..........
APEC_O1      ....T..... .......... .......... .......... .......... .......... ..........
CFT073       ....T..... .......... .......... .......... .......... .......... ..........
E2348/69     .......... .......... .......... .......... .......... .......T.. .G........
ECOR_51      ....T..... .......... .......... .......... .......... .......... ..........
ECOR_57      ....T..... .......... .......... .......... .......... .......... ..........
UTI89        ....T..... .......... .......... .......... .......... .......... ..........
E._albertii  .C........ .........G .........A .......G.. .......... .......... ....C.....
```

```
          3571
K-12         GAACGCTATT GATATGGATA TGAACCATAT TCCTGATGCG GCGATGACCA TTGCCAC--A AAAATCGCCA
53638        .......... .......... .......... ...C...... .......... .......... ..........
ATCC_8739    .......... .......... .......... ...C...... .......... .......... ..........
ECOR_10      .......... .......... .......... .......... .......... .......GG. ..........
ECOR_11      .......... .......... .......... .......... .......... .......GG. ..........
B171         .......... .......... .......... ...C...... .......... .......... ..........
E24377A      .......... .......... .......... ...C...... .......... .......... ..........
ECOR_34      .......... .......... .......... .......... .......... .......GG. ..........
ECOR_71      .......... .......... .......... ...C...... .......... .......GG. ..........
SE11         .......... .......... .......... .......... .......... .......... ..........
O157_Sakai   .......... .......... .......... .......... .......... ....T..GG. ..........
ECOR_36      ......C... .......... .......... .......... .......... .......GG. ..........
ECOR_35      ......C... .......... .......... .......... .......... .......GG. ..........
SMS-3-5      ......C... .......... .......... ...C...... .......... .......... ..........
536          ......C... .......... .......... ...C...... .......... .......... ..........
APEC_O1      ......C... .......... .......... ...C...... .......... .......... ..........
CFT073       ......C... .......... .......... ...C...... .......... .......... ..........
E2348/69     .......... .......... .......... .......... .......... .......... ..........
ECOR_51      ......C... .......... .......... ...C...... .......... .......... ..........
ECOR_57      ......C... .......... .......... ...C...... .......... ....T..... ..........
UTI89        ......C... .......... .......... ...C...... .......... .......... ..........
E._albertii  .......... ..C....... .......... C......... ........T. ....A..G.- ----------

          3641
K-12         AAGCATTGGG TAACAATATT AATGGCACGC CGGTCAACTA TAAGGTTTAT ATGACCAGCG ACGTCAACGC
53638        .......... .......... .......... .......... .......... .......... ..........
ATCC_8739    .......... .......... .......... .......... .......... .......... ..........
ECOR_10      .......... .......... .......... .......... .......... .......... ..........
ECOR_11      .......... .......... .......... .......... .......... .......... ..........
B171         .......... .......... .......... .......... .......... .......... ..........
E24377A      .......... .......... .......... .......... .......... .......... ..........
ECOR_34      .......... .......... .......... .......... .......... .......... ..........
ECOR_71      .......... .......... .......... .......... .......... .......... ..........
SE11         .......... .......... .......... .......... .......... .......... ..........
O157_Sakai   .......... .......... .......... .......... .......... .......... ..........
ECOR_36      .......... .......... .......... .......... .......... .......... ..........
ECOR_35      .......... .......... .......... .......... .......... .......... ..........
SMS-3-5      .......... .......... .......... .......... .......... .......... ..........
536          .......... .......... .......... .A........ .......... .......... ..........
APEC_O1      .......... .......... .......... .A........ .......... .......... ..........
CFT073       .......... .......... .......... .A........ .......... .......... ..........
E2348/69     .......... .......... .......... .......... .......... .......... ..........
ECOR_51      .......... .......... .......... .A........ .......... .......... ..........
ECOR_57      .......... .......... .......... .A........ .......... .......... ..........
UTI89        .......... .......... .......... .A........ .......... .......... ..........
E._albertii  ---------- ---------- ---------- ---------- ---------- ---------- ----------

          3711
K-12         ATGGGCGATG GCGAACGGCT GTGTTCGTGT CTACAGTGGC CTGATGGACA TGATGAATGA CAACGAAATT
53638        .......... .......... .......... .......... .......... .......... ..........
ATCC_8739    .......... .......... .......... .......... .......... .......... ..........
ECOR_10      .......... .......... .......... .......... .......... .......... ..........
ECOR_11      .......... .......... .......... .......... .......... .......... ..........
B171         .......... .......... .......... .......... .......... .......... ..........
E24377A      .......... .......... ....C..... .......... .......... .......... ..........
ECOR_34      .......... .......... ....C..... .......... .......... .......... ..........
ECOR_71      .......... .......... ....C..... .......... .......... .......... ..........
SE11         .......... .......... .......... .......... .......... .......... ..........
O157_Sakai   .......... .......... .......... T......... .......... .......C.. T.........
ECOR_36      .......... .......... ....C..... .......... .......... .......... ..........
ECOR_35      .......... .......... ....C..... .......... .......... .......... ..........
SMS-3-5      .......... .......... .......... .......... .......... .......C.. ..........
536          .......... ........T. .......... .......... .......... .......C.. ..........
APEC_O1      .......... ........T. .......... .......... .......... .......C.. ..........
CFT073       .......... ........T. .......... .......... .......... .......C.. ..........
E2348/69     .......... .......... ....C..... .......... .......... .......... ..........
ECOR_51      .......... ........T. .......... .......... .......... .......C.. ..........
ECOR_57      .......... ........T. .......... .......... .......... .......C.. ..........
UTI89        .......... ........T. .......... .......... .......... .......C.. ..........
E._albertii  ---------- ---------- ---------- ---------- ---------- ---------- ----------
```

```
           3781
K-12          GAAGGCGTTC TGGGCCATGA ACTGGGCCAC GTCGCGTTGG GTCACTCGCT GGCTGAAATG AAAGCTTCTT
53638         .......... .......... .......... .......... .G........ .......... ..........
ATCC_8739     .......... .......... .......... .......... .......... .......... ..........
ECOR_10       .......... .......... .......... .......... .......... .......... ..........
ECOR_11       .......... .......... .......... .......... .......... .......... ..........
B171          .....T.... .......... .......... .......... .G........ .......... ..........
E24377A       .....T.... .......... .......... .......... .......... .......... ..........
ECOR_34       .....T.... .......... .......... .......... .......... .......... ..........
ECOR_71       .....T.... .......... .......... .......... .......... .......... ..........
SE11          .....T.... .......... .......... .......... .......... .......... ..........
O157_Sakai    .......... .......... .......... .......... .......... .......... ..........
ECOR_36       .......... ....T..... .......... .......... .......... .......... ..........
ECOR_35       .......... ....T..... .......... .......... .......... .......... ..........
SMS-3-5       .......... ....T..... .......... .......... .......... .......... ..........
536           .......... .......... .......... .......... .......... .......... ..........
APEC_O1       .......... .......... .......... .......... .......... .......... ..........
CFT073        .......... .......... .......... .......... .......... .......... ..........
E2348/69      .....T.... .......... .......... .......... .......... .......... ..........
ECOR_51       .......... .......... .......... .......... .......... .......... ..........
ECOR_57       .......... .......... .......... .......... .......... .......... ..........
UTI89         .......... .......... .......... .......... .......... .......... ..........
E._albertii   ---------- ---------- ---------- ---------- ---------- ---------- ----------

           3851
K-12          ATGCGATCGT TGCCGCACGC GATGCCATTT CAGCTACCAA GAAACGTGGC ATCAGCACCC AGGGGCTGGT
53638         .......... .......... .......... .......... .......... .......... ..........
ATCC_8739     .......... .......... .......... .......... .......... .......... ..........
ECOR_10       .......... .......... .......... .......... .......... .......... ..........
ECOR_11       .......... .......... .......... .......... .......... .......... ..........
B171          .......... .......... .......... .......... .......... .......... ..........
E24377A       .......... .......... .......... .......... .......... .......... ..........
ECOR_34       .......... .......... .......... .......... .......... .......... ..........
ECOR_71       .......... .......... .......... .......... .......... .......... ..........
SE11          .......... .......... .......... .......... .......... .......... ..........
O157_Sakai    .......... .......... .......... .......... .......... .......... ..........
ECOR_36       .......... .........T .....G.... .C........ .......... .......... ..........
ECOR_35       .......... .........T .....G.... .C........ .......... .......... ..........
SMS-3-5       .......... .........T .....G.... .C........ .......... .......... ..........
536           .......... G......... .......... .......... .......... .....T.... ..........
APEC_O1       .......... G......... .......... .......... .......... .....T.... ..........
CFT073        .......... G......... .......... .......... .......... .....T.... ..........
E2348/69      .......... .......... .......... .......... .......... .....T.... ..........
ECOR_51       .......... G......... .......... .......... .......... .....T.... ..........
ECOR_57       .......... G......... .......... .......... .......... .....T.... ..........
UTI89         .......... G......... .......... .......... .......... .....T.... ..........
E._albertii   ---------- ---------- ---------- ---------- ---------- ---------- ----------

           3921
K-12          TGGCAGCTTT GAAACACTGG CTAGCCTGGA TGGCGGTCGC ACCCAGTCCA TGTTTGACTC TCACCCACCA
53638         .......... ....A..... .......... .......... .......... .......... ..........
ATCC_8739     .......... ....A..... .......... .......... .......... .......... ..........
ECOR_10       .......... ....A..... .......... .......... .......... .......... ..........
ECOR_11       .......... ....A..... .......... .......... .......... .......... ..........
B171          .......... ....A..... ....T..... .......... .......... .......... ..........
E24377A       .......... ....A..... ....T..... .......... .......... .......... ..........
ECOR_34       .......... ....A..... ....T..... .......... .......... .......... ..........
ECOR_71       .......... ....A..... ....T..... .......... .......... .......... ..........
SE11          .......... ....A..... ....T..... .......... .......... .......... ..........
O157_Sakai    .......... ....A..... .......... .......... .......... .......... ..........
ECOR_36       .......... ....A.T... .C........ .........T .......... .......... ..........
ECOR_35       .......... ....A.T... .C........ .........T .......... .......... ..........
SMS-3-5       .......... ....A.T... .C........ .......... .......... .......... ..........
536           ...T...... ....A..... .C........ .......... .......... .......T.. ..........
APEC_O1       ...T...... ....A..... .C........ .......... ..T....... .......T.. ..........
CFT073        ...T...... ....A..... .C........ .......... .......... .......T.. ..........
E2348/69      ...T...... ....A..... .C........ .......... .......... .......T.. ..........
ECOR_51       ...T...... ....A..... .C........ .......... .......... .......T.. ..........
ECOR_57       ...T...... ....A..... .C........ .......... .......... .......T.. ..........
UTI89         ...T...... ....A..... .C........ .......... ..T....... .......T.. ..........
E._albertii   ---------- ---------- ---------- ---------- ---------- ---------- ----------
```

```
          3991
K-12         TCAACAGAGC GTGCGCAACA CATCCGTGAT CGTATCGCCT CTGGTAAGTA AATCATTGTC ATCTTTCGGG
53638        .......... .......... .......... .......... .......... .......... ..........
ATCC_8739    .......... .......... .......... .......... .......... .......... ..........
ECOR_10      .......... .......... .......... .......... .......... .......... ..........
ECOR_11      .......... .......... .......... .......... .......... .......... ..........
B171         .......... .......... .......... .......... .......... .......... ..........
E24377A      .......... .......... .......... .......... .......... .......... ..........
ECOR_34      .......... .......... .......... .......... .......... .......... ..........
ECOR_71      .......... .......... .......... .......... .......... .......... ..........
SE11         .......... .......... .......... .......... .......... .......... ..........
O157_Sakai   .......... .......... .......... .......... .......... .......... ..........
ECOR_36      .......... .......... .......... .......... .......... .......... ..........
ECOR_35      .......... .......... .......... .......... .......... .......... ..........
SMS-3-5      .......... .......... .......... .......... .......... .......... ..........
536          .......... .......... .......... .......... .......... .......... ..........
APEC_O1      .......... .......... .......... .......... .......... .......... ..........
CFT073       .......... .......... .......... .......... .......... .......... ..........
E2348/69     .......... .......... .........G .......... .......... .......... ..........
ECOR_51      .......... .......... .......... .......... .......... .......... ..........
ECOR_57      .......... .......... .......... .......... .......... .......... ..........
UTI89        .......... .......... .......... .......... .......... .......... ..........
E._albertii  ---------- ---------- ---------- ---------- ---------- ---------- ----------

          4061
K-12         CTGGTCTTCT GCCAGCCCGC TATAATTGCG CAATAAATCC CCATCTGAAT ACAGACAAAA CTGGTTTTTG
53638        .......... .......... .......... .......... .......... .......... ..........
ATCC_8739    .......... .......... .......... .......... .......... .......... ..........
ECOR_10      .......... .......... .......... .......... .......... .......... ..........
ECOR_11      .......... .......... .......... .......... .......... .......... ..........
B171         .......... .......... .......... .......... .......... .......... ..........
E24377A      .......... .......... .......... .......... .......... .......... ..........
ECOR_34      .......... .......... .......... .......... .......... .......... ..........
ECOR_71      .......... .......... .......... .......... .......... .........C ..........
SE11         .......... .......... .......... .......... .......... .......... ..........
O157_Sakai   .......... .......... .......... .......... .......... .......... ..........
ECOR_36      .......... .......... .......... .......... .......... .......... ..........
ECOR_35      .......... .......... .......... .......... .......... .......... ..........
SMS-3-5      .......... .......... .......... .......... .......... .......... ..........
536          .......... .......... .......... .......... .......... .......... ..........
APEC_O1      .......... .......... .......... .......... .......... .......... ..........
CFT073       .......... .......... .......... .......... .......... .......... ..........
E2348/69     .......... .......... .......... .......... .......... .......... ..........
ECOR_51      .......... .......... .......... .......... .......... .......... ..........
ECOR_57      .......... .......... .......... .......... .......... .......... ..........
UTI89        .......... .......... .......... .......... .......... .......... ..........
E._albertii  ---------- ---------- ---------- ---------- ---------- ---------- -----.....

          4131
K-12         CACACAACGT TAACGATTTG TGGCGTCGGC GCGTATAATG CGCGCGG-TT ATGTTAACGG TACGCCTGTT
53638        .......... .......... .......... .......... .......... .......... ..........
ATCC_8739    .......... .......... .......... .......... .......... .......... ..........
ECOR_10      .......... .......... .......... .......... .......... .......... ..........
ECOR_11      .......... .......... .......... .......... .......... .......... ..........
B171         .......... .......... .......... .......... .......... .......... ..........
E24377A      .......... .......... .......... .......... .......... .......... ..........
ECOR_34      .......... .......... .......... .......... .......... .......... ..........
ECOR_71      .......... .......... .......... .......... .......... .......... ..........
SE11         .......... .......... .......... .......... .......... .......... ..........
O157_Sakai   .......... .......... .......... .......... .......... .......... ..........
ECOR_36      .......... .......... .......... .......... .......... .......... ..........
ECOR_35      .......... .......... .......... .......... .......... .......... ..........
SMS-3-5      .......... .......... .......... .......... .......... .......... ..........
536          .......... .......... .......... .......... .......... .......... ..........
APEC_O1      .......... .......... .......... .......... .......... .......... ..........
CFT073       .......... .......... .......... .......... .......... .......... ..........
E2348/69     .......... .......... .......... .......... .......... .......... ..........
ECOR_51      .......... .......... .......... .......... .......... .......... ..........
ECOR_57      .......... .......... .......... .......... .......... .......... ..........
UTI89        .......... .......... .......... .......... .......... .......... ..........
E._albertii  ...G..GA.. .......... ......T... .......... ....GC.A.. .......A.. .........G
```

```
           4201
K-12          TTAAGGAGAT AAAGATGACG GCAATTGCCC CGGTTATTAC CATTGATGGC CCAAGCGGTG CAGGGAAAGG
53638         .......... .......... .......... .......... .......... .......... ..........
ATCC_8739     .......... .......... .......... .......... .......... .......... ..........
ECOR_10       .......... .......... .......... .......... .......... .......... ..........
ECOR_11       .......... .......... .......... .......... .......... .......... ..........
B171          .......... .......... .......... .......... .......... .......... ..........
E24377A       .......... .......... .......... .......... .......... .......... ..........
ECOR_34       .......... .......... .......... .......... .......... .......... ..........
ECOR_71       .......... .......... .......... .......... .......... .......... ..........
SE11          .......... .......... .......... .......... .......... .......... ..........
O157_Sakai    .......... .......... .......... .......... .......... .......... ..........
ECOR_36       .......... .......... .......... .......... .......... .......... ..........
ECOR_35       .......... .......... .......... .......... .......... .......... ..........
SMS-3-5       .......... .......... .......... .......... .......... .......... ..........
536           .......... .......... .......... .......... .......... .......... ..........
APEC_O1       .......... .......... .......... .......... .......... .......... ..........
CFT073        .......... .......... .......... .......... .......... .......... ..........
E2348/69      .......... .......... .......... .......... .......... .......... ..........
ECOR_51       .......... .......... .......... .......... .......... .....T.... ..........
ECOR_57       .......... .......... .......... .......... .......... .......... ..........
UTI89         .......... .......... ........T. .......... .......... .......... ..........
E._albertii   .......... .......... .......... ....A..C.. .......... .......... ..........

           4271
K-12          CACCTTGTGT AAGGCTATGG CGGAAGCGTT GCAATGGCAT CTGCTGGACT CGGGTGCAAT TTATCGCGTA
53638         .......... .......... .......... .......... .......... .......... ..........
ATCC_8739     .......... .......... .......... .......... .......... .......... ..........
ECOR_10       .......... .......... .......... .......... .......... .......... ..........
ECOR_11       .......... .......... .......... .......... .......... .......... ..........
B171          .......... .......... .......A.. .......... ........T. .......... ..........
E24377A       .......... .......... .......A.. .......... T.......T. .......... ..........
ECOR_34       .......... .......... .......A.. .......... ........T. .......... ..........
ECOR_71       .......... .......... .......A.. .......... ........T. .......... ..........
SE11          .......... .......... .......A.. .......... ........T. .......... ..........
O157_Sakai    .......... .......... .......... .......... ........T. .......... ..........
ECOR_36       .......... .......... .......... .......... ........T. .......... ..........
ECOR_35       .......... .......... .......... .......... ........T. .......... ..........
SMS-3-5       .......... .......... .......... .......... .......... .......... ..........
536           .......... .......... .......... .......... ........T. .......... ..........
APEC_O1       .......... .......... .......... .......... ........T. .......... ..........
CFT073        .......... .......... .......... .......... ........T. .......... ..........
E2348/69      .......... .......... .......... .......... ........T. .......... ..........
ECOR_51       .......... .......... .......... .......... ........T. .......... ..........
ECOR_57       .......... .......... .......... .......... ........T. .......... ..........
UTI89         .......... .......... .......... .......... ........T. .......... ..........
E._albertii   ......A... .......... .......A.. .......... .......... .......... ..........

           4341
K-12          CTGGCATTGG CGGCATTACA TCACCATGTT GATGTTGCGT CGGAAGATGC GCTGGTACCG CTGGCATCCC
53638         .......... .......... .......... .......... .......... .......... ..........
ATCC_8739     .......... .......... .......... .......... .......... .......... ..........
ECOR_10       .......... .......... .......... .......... .......... .......... ..........
ECOR_11       .......... .......... .......... .......... .......... .......... ..........
B171          ......C.A. .......... .......... ........A. .......... .......... ..........
E24377A       .......... .......... .......... .......... .A........ .......... ..........
ECOR_34       .......... .......... .......... .......... .A........ .......... ..........
ECOR_71       .......... .......... .......... .......... .A........ .......... ..........
SE11          .......... .......... .......... .......... .A........ .......... ..........
O157_Sakai    ......C.A. .......... .......... ........A. .......... ......G... ..........
ECOR_36       ......C... .......... .......... ........T. .......... ......G... ..........
ECOR_35       ......C... .......... .......... ........T. .......... ......G... ..........
SMS-3-5       ......C... .......... .......... ........A. .......... ......G... ..........
536           ......C.A. .......... .......... ........A. .......... ......G... ..........
APEC_O1       ......C.A. .......... .......... ........A. .......... ......G... ..........
CFT073        ......C.A. .......... .......... ........A. .......... ......G... ..........
E2348/69      ......C.A. .......... .......... ........A. .......... ......G... ..........
ECOR_51       ......C.A. .......... .......... ........A. .......... ......G... ..........
ECOR_57       ......C.A. .......... .......... ........A. .......... ......G... ..........
UTI89         ......C.A. .......... .......... ........A. .......... ......G... ..........
E._albertii   .....GC... .......G.. ...T.....C ...A.C.... .......C.. C.....G... ..........
```

```
          4411
K-12        ATCTGGATGT ACGTTTTGTG TCGACCAATG GCAATCTGGA AGTGATCCTC GAAGGGGAAG ATGTCAGCGG
53638       .......... .......... .......... .......... .......... .......... ..........
ATCC_8739   .......... .......... .......... .......... .......... .......... ..........
ECOR_10     .......... .......... .......... .......... .......... .......... ..........
ECOR_11     .......... .......... .......... .......... .......... .......... ..........
B171        .C........ .......... ..A....... .......... .......... .......... ..........
E24377A     .......... .......... ..A....... .......... .......... .......... ..........
ECOR_34     .......... .......... ..A....... .......... .......... .......... ..........
ECOR_71     .......... .......... ..A....... .......... .......... .......... ..........
SE11        .......... .......... ..A....... .......... .......... .......... ..........
O157_Sakai  .......... .......... ..A....... .......... .......... .......... ..........
ECOR_36     .......... .......... .......... .......... .......... .......... ..........
ECOR_35     .......... .......... .......... .......... .......... .......... ..........
SMS-3-5     .......... .......... .......... .......... .......... .......... ..........
536         .......... .......... ..A.....C. .T........ .......... .......... ..........
APEC_O1     .......... .......... ..A.....C. .T........ .......... .......... ..........
CFT073      .......... .......... ..A.....C. .T........ .......... .......... ..........
E2348/69    .......... .......... ..A.....C. .......... .......... .......... ..........
ECOR_51     .......... .......... ..A.....C. .T........ .......... .......... ..........
ECOR_57     .......... .......... ..A.....C. .T........ .......... .......... ..........
UTI89       .......... .......... ..A.....C. .T........ .......... .......... ..........
E._albertii .......... G......... .......... .......... .......... .......... .......T..

          4481
K-12        CGAAATTCGT ACTCAGGAAG TGGCGAATGC AGCTTCACAA GTCGCGGCAT TCCCACGCGT TCGTGAAGCA
53638       .......... .......... .......... .......... .......... .......... ..........
ATCC_8739   .......... .......... .......... .......... .......... .......... ..........
ECOR_10     .......... .......... .......... .......... .......... .......... ..........
ECOR_11     .......... .......... .......... .......... .......... .......... ..........
B171        .......... .......... .......... .......... .......... .......... ..........
E24377A     .......... .......... .......... .......... .......... .......... ..........
ECOR_34     .......... .......... .......... .......... .......... .......... ..........
ECOR_71     .......... .......... .......... .......... .......... .......... ..........
SE11        .......... .......... .......... .......... .......... .......... ..........
O157_Sakai  .......... .......... .......... .......... .......... .......... ..........
ECOR_36     .......... .......... .......... T......... .......... .......... ..........
ECOR_35     .......... .......... .......... T......... .......... .......... ..........
SMS-3-5     .......... .......... .......... T..C...... .......... .......... ..........
536         .......... .......... .......... T......... .......... .......... ..........
APEC_O1     .......... .......... .......... T......... .......... .......... ..........
CFT073      .......... .......... .......... T......... .......... .......... ..........
E2348/69    .......... ........G. .......... T......... .......... .......... ..........
ECOR_51     .......... .......... .......... T......... .......... .......... ..........
ECOR_57     .......... .......... .......... T......... .......... .......... ..........
UTI89       .......... .......... .......... T......... .......... .......... ..........
E._albertii T........C ........C. .......... T..G...... ..G....... .T..T..T.. G.....G..G

          4551
K-12        TTATTGCGTC GCCAACGCGC GTTTCGCGAA TTACCAGGTC TGATTGCCGA TGGCCGCGAC ATGGGAACGG
53638       .......... .......... .......... .......... .......... .......... ..........
ATCC_8739   .......... .......... .......... .......... .......... .......... ..........
ECOR_10     .......... .......... .......... .......... .......... .......... ..........
ECOR_11     .......... .......... .......... .......... .......... .......... ..........
B171        .......... .......... .......... ........C. .......... .......... ..........
E24377A     .......... .......... .......... ........C. .......... .......... ..........
ECOR_34     .......... .......... .......... ........C. .......... .......... ..........
ECOR_71     .......... .......... .......... ........C. .......... .......... ..........
SE11        .......... .......... .......... .....C..C. .......... .......... ..........
O157_Sakai  .......... .......... .......... ........C. .......... .......... ..........
ECOR_36     .......... .......... .......... ........C. .......... .......... ..........
ECOR_35     .......... .......... .......... ........C. .......... .......... ..........
SMS-3-5     .......... .......... .......... ........C. .......... .......... ..........
536         .......... .......... .......... ........C. .......... .......... ..........
APEC_O1     .......... .......... .......... ........C. .......... .......... ..........
CFT073      .......... .......... .......... ........C. .......... .......... ..........
E2348/69    .......... .......... .......... ........C. .......... ...G...... ..........
ECOR_51     .......... .......... .......... ........C. .......... .......... ..........
ECOR_57     .......... .......... .......... ........C. .......... .......... ..........
UTI89       .......... .......... .......... ........C. .......... .......... ..........
E._albertii C.G....... .A..G..... A..C..T..G .....C..A. ....C..... ......G... ..........
```

```
            4621
K-12         TGGTATTCCC TGATGCACCA GTGAAAATTT TCCTTGACGC CTCCTCGGAA GAACGTGCGC ATCGCCGCAT
53638        .......... .......... .......... .......... .......... .......... ..........
ATCC_8739    .......... .......... .......... .......... .......... .......... ..........
ECOR_10      .......... .......... .......... .......... .......... .......... ..........
ECOR_11      .......... .......... .......... .......... .......... .......... ..........
B171         .......... ......G... .......... .......... .......... .......... ..........
E24377A      .......... ......G... .......... .......... .......... .....A.... ..........
ECOR_34      .......... ......G... .......... .......... .......... .......... ..........
ECOR_71      .......... ......G... .......... .......... .......... .......... ..........
SE11         .......... ......G... .......... .......... .......... .......... ..........
O157_Sakai   .......... ......G... .......... .......... .......... .......... ..........
ECOR_36      .......... ......G... .......... .......... .......... .......... ..........
ECOR_35      .......... ......G... .......... .......... .......... .......... ..........
SMS-3-5      .......... ......G... .......... .......... .......... .......... ..........
536          .......... ......G... .......... .......... .......... .......... ..........
APEC_O1      .......... ......G... .......... .......... .......... .......... ..........
CFT073       .......... ......G... .......... .......... .......... .......... ..........
E2348/69     .......... ......G... .......... .......... .......... .......... ..........
ECOR_51      .......... ......G... .......... .......... .......... .......... ..........
ECOR_57      .......... ......G... .......... .......... .......... .......... ..........
UTI89        .......... ......G... .......... .......... .......... .......... ..........
E._albertii  .......... ......G... .......... .......... T...C..... .......... ..........

            4691
K-12         GCTACAGTTG CAGGAGAAGG GCTTTAGTGT TAACTTTGAG CGCCTTTTGG CCGAGATCAA AGAACGCGAC
53638        .......... .......... .......... .......... .......... .......... ..........
ATCC_8739    .......... .......... .......... .......... .......... .......... ..........
ECOR_10      .......... .......... .......... .......... .......... .......... ..........
ECOR_11      .......... .......... .......... .......... .......... .......... ..........
B171         .......... .......... .......... .......... .......... .......... ..........
E24377A      .......... .......... .......... .......... .......... .......... ..........
ECOR_34      .......... .......... .......... .......... .......... .......... ..........
ECOR_71      .......... .......... .......... .......... .......... .......... ..........
SE11         .......... .......... .......... .......... .......... .......... ..........
O157_Sakai   .......... .......... .......... .......... .......... .......... ..........
ECOR_36      .......... .......... .......... .......... .......... .......... ..........
ECOR_35      .......... .......... .......... .......... .......... .......... ..........
SMS-3-5      .......... .......... .......... .......... .......... .......... ..........
536          .......... .......... .......... .......... .......... .......... ..........
APEC_O1      .......... .......... .......... .......... .......... .......... ..........
CFT073       .......... .......... .......... .......... .......... .......... ..........
E2348/69     .......... .......... .......... .......... .......... .......... ..........
ECOR_51      .......... .......... .......... .......... .......... .......... ..........
ECOR_57      .......... .......... .......... .......... .......... .......... ..........
UTI89        .......... .......... .......... .......... .......... .......... ..........
E._albertii  .......... .......... .......... .......... .......... .......A.. ..........

            4761
K-12         GACCGCGATC GTAACCGAGC GGTAGCGCCA CTGGTTCCGG CAGCCGATGC TTTAGTGTTG GATTCCACCA
53638        .......... .......... .......... .......... .......... .......... ..........
ATCC_8739    .......... .......... .......... .......... .......... .......... ..........
ECOR_10      .......... .......... .......... .......... .......... .......... ..........
ECOR_11      .......... .......... .......... .......... .......... .......... ..........
B171         .......... .......... .......... .......... .......... .......... ..........
E24377A      .......... .......... .......... .......... .......... .......... ..........
ECOR_34      .......... .......... .......... .......... .......... .......... ..........
ECOR_71      .......... .......... .......... .......... .......... .......... ..........
SE11         .......... .......... .......... .......... .......... .......... ..........
O157_Sakai   .......... .......... .......... .......... .......... .......... ..........
ECOR_36      .......... .......... .......... .......... .......... .......... ..........
ECOR_35      .......... .......... .......... .......... .......... .......... ..........
SMS-3-5      .......... .......... .......... .......... .......... .......... ..........
536          .......... .......... .......... .......... .......... .......... ..........
APEC_O1      .......... .......... .......... .......... .......... .......... ..........
CFT073       .......... .......... .......... .......... .......... .......... ..........
E2348/69     .......... .......... .......... .......... .......... .......... ..........
ECOR_51      .......... .......... .......... .......... .......... .......... ..........
ECOR_57      .......... .......... .......... .......... .......... .......... ..........
UTI89        .......... .......... .......... .......... .......... .......... ..........
E._albertii  .......... .......T.. .......... T......... .......... .C.G...... ..........
```

```
             4831
K-12         CCTTAAGCAT TGAGCAAGTG ATTGAAAAAG CGCTACAATA CGCGCGCCAG AAATTGGCTC TCGCATAAGC
53638        .......... .......... .......... .......... .......... .......... ..........
ATCC_8739    .......... .......... .......... .......... .......... .......... ..........
ECOR_10      .......... .......... .......... .......... .......... .......... ..........
ECOR_11      .......... .......... .......... .......... .......... .......... ..........
B171         .......... .......... .......... .......... .......... .......... ..........
E24377A      .......... .......... .......... .......... .......... .......... ..........
ECOR_34      .......... .......... .......... .......... .......... .......... ..........
ECOR_71      .......... .......... .......... .......... .......... .......... ..........
SE11         .......... .......... .......... .......... .......... .......... ..........
O157_Sakai   .......... .......... .......... .......... .......... .......... ..........
ECOR_36      .......... .......... .......... .......... .......... .......... ..........
ECOR_35      .......... .......... .......... .......... .......... .......... ..........
SMS-3-5      .......... .......... .......... .......... .......... .......... ..........
536          .......... .......... .......... .......... .......... .......... ..........
APEC_O1      .......... .......... .......... .......... .......... .......... ..........
CFT073       .......... .......... .......... .......... .......... .......... ..........
E2348/69     .......... .......... .......... .......... .......... .......... ..........
ECOR_51      .......... .......... .......... .......... .......... .......... ..........
ECOR_57      .......... .......... .......... .......... .......... .......... ..........
UTI89        .......... .......... .......... .......... .......... .......... ..........
E._albertii  .......... .......... .......... .......... .........A ...C...... ..........

             4901
K-12         GACCGAATTT GCAGTACCCC CGTTGCAATG GAATGACAGC GGGTATGTTA AACAACCCCA TCCGGCATGG
53638        .......... .......... .......... .......... .......... .......... ..........
ATCC_8739    .......... .......... .......... .......... .......... .......... ..........
ECOR_10      .......... .......... .......... .......... .......... .......... ..........
ECOR_11      .......... .......... .......... .......... .......... .......... ..........
B171         .......... .......... .......... .......... .......... .......... ..........
E24377A      .......... .......... .......... .......... .......... .......... ..........
ECOR_34      .......... .......... .......... .......... .......... .......... ..........
ECOR_71      .......... .......... .......... .......... .......... .......... ..........
SE11         .......... .......... .......... .......... .......... .......... ..........
O157_Sakai   .......... .......... .......... .......... .......... .......... ..........
ECOR_36      .......... .......... .......... .......... .......... .......... ..........
ECOR_35      .......... .......... .......... .......... .......... .......... ..........
SMS-3-5      .......... .......... .......... .......... .......... .......... ..........
536          .......... .......... .......... .......... .......... .......... ..........
APEC_O1      .......... .......... .......... .......... .......... .......... ..........
CFT073       .......... .......... .......... .......... .......... .......... ..........
E2348/69     .......... .......... .......... .......... .......... .......... ..........
ECOR_51      .......... .......... .......... .......... .......... .......... ..........
ECOR_57      .......... .......... .......... .......... .......... .......... ..........
UTI89        .......... .......... .......... .......... .......... .......... ..........
E._albertii  .......... .......... .......... .......... .......... .......... .......C..

             4971
K-12         AGCCAGGTGG ACGTTAAATA TAAACCTGAA GATTAAACAT GACTGAATCT TTTGCTCAAC TCTTTGAAGA
53638        .......... .......... .......... .......... .......... .......... ..........
ATCC_8739    .......... .......... .......... .......... .......... .......... ..........
ECOR_10      .......... .......... .......... .......... .......... .......... ..........
ECOR_11      .......... .......... .......... .......... .......... .......... ..........
B171         .......... .......... .......... .......... .......... .......... ..........
E24377A      .......... .......... .......... .......... .......... .......... ..........
ECOR_34      .......... .......... .......... .......... .......... .......... ..........
ECOR_71      .......... .......... .......... .......... .......... .......... ..........
SE11         .......... .......... .......... .......... .......... .......... ..........
O157_Sakai   .......... .......... .......... .......... .......... .......... ..........
ECOR_36      .......... .......... .......... .......... .......... .......... ..........
ECOR_35      .......... .......... .......... .......... .......... .......... ..........
SMS-3-5      ...T...... .......... .......... .......... .......... .......... ..........
536          .......... .......... .......... .......... .......... .......... ..........
APEC_O1      .......... .......... .......... .......... .......... .......... ..........
CFT073       .......... .......... .......... .......... .......... .......... ..........
E2348/69     .......... .......... .......... .......... .......... .......... ..........
ECOR_51      .......... .......... .......... .......... .......... .......... ..........
ECOR_57      .......... .......... .......... .......... .......... .......... ..........
UTI89        .......... .......... .......... .......... .......... .......... ..........
E._albertii  ...T...... .......... .......... .......... .......... .......... ..........
```

```
          5041
K-12         GTCCTTAAAA GAAATCGAAA CCCGCCCGGG TTCTATCGTT CGTGGCGTTG TTGTTGCTAT CGACAAAGAC
53638        .......... .......... .......... .......... .......... .......... ..........
ATCC_8739    .......... .......... .......... .......... .......... .......... ..........
ECOR_10      .......... .......... .......... .......... .......... .......... ..........
ECOR_11      .......... .......... .......... .......... .......... .......... ..........
B171         .......... .......... .......... .......... .......... .......... ..........
E24377A      .......... .......... .......... .......... .......... .......... ..........
ECOR_34      .......... .......... .......... .......... .......... .......... ..........
ECOR_71      .......... .......... .......... .......... .......... .......... ..........
SE11         .......... .......... .......... .......... .......... .......... ..........
O157_Sakai   .......... .......... .......... .......... .......... .......... ..........
ECOR_36      .......... .......... .......... .......... .......... .......... ..........
ECOR_35      .......... .......... .......... .......... .......... .......... ..........
SMS-3-5      .......... .......... .......... .......... .......... .......... ..........
536          .......... .......... .......... .......... .......... .......... ..........
APEC_O1      .......... .......... .......... .......... .......... .......... ..........
CFT073       .......... .......... .......... .......... .......... .......... ..........
E2348/69     .......... .......... .......... .......... ........A. .......... ..........
ECOR_51      .......... .......... .......... .......... .......... .......... ..........
ECOR_57      .......... .......... .......... .......... .......... .......... ..........
UTI89        .......... .......... .......... .......... .......... .......... ..........
E._albertii  .......... .......... .......... .......... .......... .......... ..........

          5111
K-12         GTAGTACTGG TTGACGCTGG TCTGAAATCT GAGTCCGCCA TCCCGGCTGA GCAGTTCAAA AACGCCCAGG
53638        .......... .......... .......... .......... .......... .......... ..........
ATCC_8739    .......... .......... .......... .......... .......... .......... ..........
ECOR_10      .......... .......... .......... .......... .......... .......... ..........
ECOR_11      .......... .......... .......... .......... .......... .......... ..........
B171         .......... .......... .......... .......... .......A.. .......... ..........
E24377A      .......... .......... .......... .......... .......... .......... ..........
ECOR_34      .......... .......... .......... .......... .......... .......... ..........
ECOR_71      .......... .......... .......... .......... .......... .......... ..........
SE11         .......... .......... .......... .......... .......... .......... ..........
O157_Sakai   .......... .......... .......... .......... .......... .......... ..........
ECOR_36      .......... .......... .......... .......... .......... .......... ..........
ECOR_35      .......... .......... .......... .......... .......... .......... ..........
SMS-3-5      .......... .......... .......... .......... .......... .......... ..........
536          .......... .......... .......... .......... .......... .......... ..........
APEC_O1      .......... .......... .......... .......... .......... .......... ..........
CFT073       .......... .......... .......... .......... .......... .......... ..........
E2348/69     .......... .......... .......... .......... .......... .......... ..........
ECOR_51      .......... .......... .......... .......... .......... .......... ..........
ECOR_57      .......... .......... .......... .......... .......... .......... ..........
UTI89        .......... .......... .......... .......... .......... .......... ..........
E._albertii  .......... .......... .......... .......... .......... .......... ..........

          5181
K-12         GCGAGCTGGA AATCCAGGTA GGTGACGAAG TTGACGTTGC TCTGGACGCA GTAGAAGACG GCTTCGGTGA
53638        .......... .......... .......... .......... .......... .......... ..........
ATCC_8739    .......... .......... .......... .......... .......... .......... ..........
ECOR_10      .......... .......... .......... .......... .......... .......... ..........
ECOR_11      .......... .......... .......... .......... .......... .......... ..........
B171         .......... .......... .......... .......... .......... .......... ..........
E24377A      .......... .......... .......... .......... .......... .......... ..........
ECOR_34      .......... .......... .......... .......... .......... .......... ..........
ECOR_71      .......... .......... .......... .......... .......... .......... ..........
SE11         .......... .......... .....T.... .......... .......... .......... ..........
O157_Sakai   .......... .......... .......... .......... .......... .......... ..........
ECOR_36      .......... .......... .......... .......... .......... .......... ..........
ECOR_35      .......... .......... .......... .......... .......... .......... ..........
SMS-3-5      .......... .......... .......... .......... .......... .......... ..........
536          .......... .......... .......... .......... .......... .......... ..........
APEC_O1      .......... .......... .......... .......... .......... .......... ..........
CFT073       .......... .......... .......... .......... .......... .......... ..........
E2348/69     .......... .......... .......... .......... .......... .......... ..........
ECOR_51      .......... .......... .......... .......... .......... .......... ..........
ECOR_57      .......... .......... .......... .......... .......... .......... ..........
UTI89        .......... .......... .......... .......... .......... .......... ..........
E._albertii  .......... .......... .......... .......... .......... .......... ..........
```

```
           5251
K-12          AACTCTGCTG TCCCGTGAGA AAGCTAAACG TCACGAAGCC TGGATCACGC TGGAAAAAGC TTACGAAGAT
53638         .......... .......... .......... .......... .......... .......... ..........
ATCC_8739     .......... .......... .......... .......... .......... .......... ..........
ECOR_10       .......... .......... .......... .......... .......... .......... ..........
ECOR_11       .......... .......... .......... .......... .......... .......... ..........
B171          .......... .......... .......... .......... .......... .......... ..........
E24377A       .......... .......... .......... .......... .......... .......... ..........
ECOR_34       .......... .......... .......... .......... .......... .......... ..........
ECOR_71       .......... .......... .......... .......... .......... .......... ..........
SE11          .......... .......... .......... .......... .......... .......... ..........
O157_Sakai    .......... .......... .......... .......... .......... .......... ..........
ECOR_36       .......... .......... .......... .......... .......... .......... ..........
ECOR_35       .......... .......... .......... .......... .......... .......... ..........
SMS-3-5       .......... .......... .......... .......... .......... .......... ..........
536           .......... .......... .......... .......... .......... .......... ..........
APEC_O1       .......... .......... .......... .......... .......... .......... ..........
CFT073        .......... .......... .......... .......... .......... .......... ..........
E2348/69      .......... .......... .......... .......... .......... .......... ..........
ECOR_51       .......... .......... .......... .......... .......... .......... ..........
ECOR_57       .......... .......... .......... .......... .......... .......... ..........
UTI89         .......... .......... .......... .......... .......... .......... ..........
E._albertii   .......... .......... .......... .......... .......... .......... ..........

           5321
K-12          GCTGAAACTG TTACCGGTGT TATCAACGGC AAAGTTAAGG GCGGCTTCAC TGTTGAGCTG AACGGTATTC
53638         .......... .......... .......... .......... .......... .......... ..........
ATCC_8739     .......... .......... .......... .......... .......... .......... ..........
ECOR_10       .......... .......... .......... .......... .......... .......... ..........
ECOR_11       .......... .......... .......... .......... .......... .......... ..........
B171          .......... .......... .......... .......... .......... .......... ..........
E24377A       .......... .......... .......... .......... .......... .......... ..........
ECOR_34       .......... .......... .......... .......... .......... .......... ..........
ECOR_71       .......... .......... .......... .......... .......... .......... ..........
SE11          .......... .......... .......... .......... .......... .......... ..........
O157_Sakai    .......... .......... .......... .......... .......... .......... ..........
ECOR_36       .......... .......... .......... .......... .......... .......... ..........
ECOR_35       .......... .......... .......... .......... .......... .......... ..........
SMS-3-5       .......... .......... .......... .......... .......... .......... ..........
536           .......... .......... .......... .......... .......... .......... ..........
APEC_O1       .......... .......... .......... .......... .......... .......... ..........
CFT073        .......... .......... .......... .......... .......... .......... ..........
E2348/69      .......... .......... .......... .......... .......... .......... ..........
ECOR_51       .......... .......... .......... .......... .......... .......... ..........
ECOR_57       .......... .......... .......... .......... .......... .......... ..........
UTI89         .......... .......... .......... .......... .......... .......... ..........
E._albertii   ........C. .......... .......... .......... .......... .......... ..........

           5391
K-12          GTGCGTTCCT GCCAGGTTCT CTGGTAGACG TTCGTCCGGT GCGTGACACT CTGCACCTGG AAGGCAAAGA
53638         .......... .......... .......... .......... .......... .......... ..........
ATCC_8739     .......... .......... .......... .......... .......... .......... ..........
ECOR_10       .......... .......... .......... .......... .......... .......... ..........
ECOR_11       .......... .......... .......... .......... .......... .......... ..........
B171          .......... .......... .......... .......... .......... .......... ..........
E24377A       .......... .......... .......... .......... .......... .......... ..........
ECOR_34       .......... .......... .......... .......... .......... .......... ..........
ECOR_71       .......... .......... .......... .......... .......... .......... ..........
SE11          .......... .......... .......... .......... .......... .......... ..........
O157_Sakai    .......... .......... .......... .......... .......... .......... ..........
ECOR_36       .......... .......... .......... .......... .......... .......... ..........
ECOR_35       .......... .......... .......... .......... .......... .......... ..........
SMS-3-5       .......... .......... .......... .......... .......... .......... ..........
536           .......... .......... .......... .......... .......... .......... ..........
APEC_O1       .......... .......... .......... .......... .......... .......... ..........
CFT073        .......... .......... .......... .......... .......... .......... ..........
E2348/69      .......... .......... .......... .......... .......... .......... ..........
ECOR_51       .......... .......... .......... .......... .......... .......... ..........
ECOR_57       .......... .......... .......... .......... .......... .......... ..........
UTI89         .......... .......... .......... .......... .......... .......... ..........
E._albertii   .......... .......... .......... .......... .......... .......... ..........
```

```
            5461
K-12         GCTTGAATTT AAAGTAATCA AGCTGGATCA GAAGCGCAAC AACGTTGTTG TTTCTCGTCG TGCCGTTATC
53638        .......... .......... .......... .......... .......... .......... ..........
ATCC_8739    .......... .......... .......... .......... .......... .......... ..........
ECOR_10      .......... .......... .......... .......... .......... .......... ..........
ECOR_11      .......... .......... .......... .......... .......... .......... ..........
B171         .........C .......... .......... .......... .......... .......... ..........
E24377A      .......... .......... .......... .......... .......... .......... ..........
ECOR_34      .......... .......... .......... .......... .......... .......... ..........
ECOR_71      .......... .......... .......... .......... .......... .......... ..........
SE11         .......... .......... .......... .......... .......... .......... ..........
O157_Sakai   .........C .......... .......... .......... .......... .......... ..........
ECOR_36      .........C .......... .......... .......... .......... .......... ..........
ECOR_35      .........C .......... .......... .......... .......... .......... ..........
SMS-3-5      .........C .......... .......... .......... .......... .......... ..........
536          .........C .......... .......... .......... .......... .......... ..........
APEC_O1      .........C .......... .......... .......... .......... .......... ..........
CFT073       .........C .......... .......... .......... .......... .......... ..........
E2348/69     .........C .......... .......... .......... .......... .......... ..........
ECOR_51      .........C .......... .......... .......... .......... .......... ..........
ECOR_57      .........C .......... .......... .......... .......... .......... ..........
UTI89        .........C .......... .......... .......... .......... .......... ..........
E._albertii  .........C .......... .......... .......... .......... .......... ..........

            5531
K-12         GAATCCGAAA ACAGCGCAGA GCGCGATCAG CTGCTGGAAA ACCTGCAGGA AGGCATGGAA GTTAAAGGTA
53638        .......... .......... .......... .......... .......... .......... ..........
ATCC_8739    .......... .......... .......... .......... .......... .......... ..........
ECOR_10      .......... .......... .......... .......... .......... .......... ..........
ECOR_11      .......... .......... .......... .......... .......... .......... ..........
B171         .......... .......... .......... .......... .......... .......... ..........
E24377A      .......... .......... .......... .......... .......... .......... ..........
ECOR_34      .......... .......... .......... .......... .......... .......... ..........
ECOR_71      .......... .......... .......... .......... .......... .......... ..........
SE11         .......... .......... .......... .......... .......... .......... ..........
O157_Sakai   .......... .......... .......... .......... .......... .......... ..........
ECOR_36      .......... .......... .......... .......... .......... .......... ..........
ECOR_35      .......... .......... .......... .......... .......... .......... ..........
SMS-3-5      .......... .......... .......... .......... .......... .......... ..........
536          .......... .......... .......... .......... ..T....... .......... ..........
APEC_O1      .......... .......... .......... .......... .......... .......... ..........
CFT073       .......... .......... .......... .......... .......... .......... ..........
E2348/69     .......... .......... .......... .......... .......... .......... ..........
ECOR_51      .......... .......... .......... .......... .......... .......... ..........
ECOR_57      .......... .......... .......... .......... .......... .......... ..........
UTI89        .......... .......... .......... .......... .......... .......... ..........
E._albertii  .......... .......... .......... .......... .......... .......... ..........

            5601
K-12         TCGTTAAGAA CCTCACTGAC TACGGTGCAT TCGTTGATCT GGGCGGCGTT GACGGCCTGC TGCACATCAC
53638        .......... .......... .......... .......... .......... .......... ..........
ATCC_8739    .......... .......... .......... .......... .......... .......... ..........
ECOR_10      .......... .......... .......... .......... .......... .......... ..........
ECOR_11      .......... .......... .......... .......... .......... .......... ..........
B171         .......... .......... .......... .......... .......... .......... ..........
E24377A      .......... .......... .......... .......... .......... .......... ..........
ECOR_34      .......... .......... .......... .......... .......... .......... ..........
ECOR_71      .......... .......... .......... .......... .......... .......... ..........
SE11         .......... .......... .......... .......... .......... .......... ..........
O157_Sakai   .......... .......... .......... .......... .......... .......... ..........
ECOR_36      .......... .......... .......... .......... .......... .......... ..........
ECOR_35      .......... .......... .......... .......... .......... .......... ..........
SMS-3-5      .......... .......... .......... .......... .......... .......... ..........
536          .......... .......... .......... .......... .......... .......... ..........
APEC_O1      .......... .......... .......... .......... .......... .......... ..........
CFT073       .......... .......... .......... .......... .......... .......... ..........
E2348/69     .......... .......... .......... .......... .......... .......... ..........
ECOR_51      .......... .......... .......... .......... .......... .......... ..........
ECOR_57      .......... .......... .......... .......... .......... .......... ..........
UTI89        .......... .......... .......... .......... .......... .......... ..........
E._albertii  .......... .......... .......... .......... .......... .......... ..........
```

```
          5671
K-12          TGACATGGCC TGGAAACGCG TTAAGCATCC GAGCGAAATC GTCAACGTGG GCGACGAAAT CACTGTTAAA
53638         .......... .......... .......... .......... .......... .......... ..........
ATCC_8739     .......... .......... .......... .......... .......... .......... ..........
ECOR_10       .......... .......... .......... .......... .......... .......... ..........
ECOR_11       .......... .......... .......... .......... .......... .......... ..........
B171          .......... .......... .......... .......... .......... .......... ..........
E24377A       .......... .......... .......... .......... .......... .......... ..........
ECOR_34       .......... .......... .......... .......... .......... .......... ..........
ECOR_71       .......... .......... .......... .......... .......... .......... ..........
SE11          .......... .......... .......... .......... .......... .......... ..........
O157_Sakai    .......... .......... .......... .......... .......... .......... ..........
ECOR_36       ...T...... .......... .......... .......... .......... .......... ..........
ECOR_35       ...T...... .......... .......... .......... .......... .......... ..........
SMS-3-5       ...T...... .......... .......... .......... .......... .......... ..........
536           .......... .......... .......... .......... .......... .......... ..........
APEC_O1       .......... .......... .......... .......... .......... .......... ..........
CFT073        .......... .......... .......... .......... .......... .......... ..........
E2348/69      .......... .......... .......... .......... .......... .......... ..........
ECOR_51       .......... .......... .......... .......... .......... .......... ..........
ECOR_57       .......... .......... .......... .......... .......... .......... ..........
UTI89         .......... .......... .......... .......... .......... .......... ..........
E._albertii   .........A .......... .......... .......... .....T.... .......... ..........

          5741
K-12          GTGCTGAAGT TCGACCGCGA ACGTACCCGT GTATCCCTGG GCCTGAAACA GCTGGGCGAA GATCCGTGGG
53638         .......... .......... .......... .......... .......... .......... ..........
ATCC_8739     .......... .......... .......... .......... .......... .......... ..........
ECOR_10       .......... .......... .......... .......... .......... .......... ..........
ECOR_11       .......... .......... .......... .......... .......... .......... ..........
B171          .......... .......... .......... .......... .......... .......... ..........
E24377A       .......... .......... .......... .......... .......... .T........ ..........
ECOR_34       .......... .......... .......... .......... .......... .T........ ..........
ECOR_71       .......... .......... .......... .......... .......... .......... ..........
SE11          .......... .......... .......... .......... .......... .T........ ..........
O157_Sakai    .......... .......... .......... .......... .......... .......... ..........
ECOR_36       .......... .......... .......... .......... .......... ......T... ..........
ECOR_35       .......... .......... .......... .......... .......... ......T... ..........
SMS-3-5       .......... .......... .......... .......... .......... .......... ..........
536           .......... .......... .......... .......... .......... .......... ..........
APEC_O1       .......... .......... .......... .......... .......... .......... ..........
CFT073        .......... .......... .......... .......... .......... .......... ..........
E2348/69      .......... .......... .......... .......... .......... .......... ..........
ECOR_51       .......... .......... .......... .......... .......... .......... ..........
ECOR_57       .......... .......... .......... .......... .......... .......... ..........
UTI89         .......... .......... .......... .......... .......... .......... ..........
E._albertii   .......... .......... .......... .......... .......... .......... ..........

          5811
K-12          TAGCTATCGC TAAACGTTAT CCGGAAGGTA CCAAACTGAC TGGTCGCGTG ACCAACCTGA CCGACTACGG
53638         .......... .......... .......... .......... .......... .......... ..........
ATCC_8739     .......... .......... .......... .......... .......... .......... ..........
ECOR_10       .......... .......... .......... .......... .......... .......... ..........
ECOR_11       .......... .......... .......... .......... .......... .......... ..........
B171          .......... .......... .......... .......... .......... .......... ..........
E24377A       .......... .......... .......... .......... .......... .......... ..........
ECOR_34       .......... .......... .......... .......... .......... .......... ..........
ECOR_71       .......... .......... .......... .......... .......... .......... ..........
SE11          .......... .......... .......... .......... .......... .......... ..........
O157_Sakai    .......... .......... .......... .......... .......... .......... ..........
ECOR_36       .......... .......... .......... .......... .......... .......... ..........
ECOR_35       .......... .......... .......... .......... .......... .......... ..........
SMS-3-5       .......... .......... .......... .......... .......... .......... ..........
536           .......... .......... .......... .......... .......... .......... ..........
APEC_O1       .......... .......... .......... .......... .......... .......... ..........
CFT073        .......... .......... .......... .......... .......... .......... ..........
E2348/69      .......... .......... ..A....... .......... .......... .......... ..........
ECOR_51       .......... .......... .......... .......... .......... .......... ..........
ECOR_57       .......... .......... .......... .......... .......... .......... ..........
UTI89         .......... .......... .......... .......... .......... .......... ..........
E._albertii   .......... .......... .......... .......... .......... .......... .T........
```

```
          5881
K-12         CTGCTTCGTT GAAATCGAAG AAGGCGTTGA AGGCCTGGTA CACGTTTCCG AAATGGACTG GACCAACAAA
53638        .......... .......... .......... .......... .......... .......T.. ..........
ATCC_8739    .......... .......... .......... .......... .......... .......T.. ..........
ECOR_10      .......... .......... .......... .......... .......... .......T.. ..........
ECOR_11      .......... .......... .......... .......... .......... .......T.. ..........
B171         .......... .......... .......... .......... .......... .......T.. ..........
E24377A      .......... .......... .......... .......... .......... .......T.. ..........
ECOR_34      .......... .......... .......... .......... .......... .......T.. ..........
ECOR_71      .......... .......... .......... .......... .......... .......T.. ..........
SE11         .......... .......... .......... .......... .......... .......T.. ..........
O157_Sakai   .......... .......... .......... .......... .......... .......T.. ..........
ECOR_36      .......... .......... ....T..... .......... .......... .......T.. ..........
ECOR_35      .......... .......... ....T..... .......... .......... .......T.. ..........
SMS-3-5      .......... .......... .......... .......... .......... .......T.. ..........
536          .......... .......... .......... .......... .......... .......T.. ..........
APEC_O1      .......... .......... .......... .......... .......... .......T.. ..........
CFT073       T......... .......... .......... .......... .......... .......T.. ..........
E2348/69     .......... .......... .......... .......... .......... .......T.. ..........
ECOR_51      .......... .......... .......... .......... .......... .......T.. ..........
ECOR_57      T......... .......... .......... .......... .......... .......T.. ..........
UTI89        .......... .......... .......... .......... .......... .......T.. ..........
E._albertii  .......... .......... .......... .......... .......... .......T.. ..........

          5951
K-12         AACATCCACC CGTCCAAAGT TGTTAACGTT GGCGATGTAG TGGAAGTTAT GGTTCTGGAT ATCGACGAAG
53638        .......... .......... .......... .......... .......... .......... ..........
ATCC_8739    .......... .......... .......... .......... .......... .......... ..........
ECOR_10      .......... .......... .......... .......... .......... .......... ..........
ECOR_11      .......... .......... .......... .......... .......... .......... ..........
B171         .......... .......... .......... .......... .......... .......... ..........
E24377A      .......... .......... .......... .......... .......... .......... ........G.
ECOR_34      .......... .......... .......... .......... .......... .......... ..........
ECOR_71      .......... .......... .......... .......... .......... .......... ..........
SE11         .......... .......... .......... .......... .......... .......... ..........
O157_Sakai   .......... .......... .......... .......... .......... .......... ..........
ECOR_36      .......... .......... .......... .......... .......... .......... ..........
ECOR_35      .......... .......... .......... .......... .......... .......... ..........
SMS-3-5      .......... .......... .......... .......... .......... .......... ..........
536          .......... .......... .......... .......... .......... .......... ..........
APEC_O1      .......... .......... .......... .......... .......... .......... ..........
CFT073       .......... .......... .......... .......... .......... .......... ..........
E2348/69     .......... .......... .......... .......... .......... .......... ..........
ECOR_51      .......... .......... .......... .......... .......... .......... ..........
ECOR_57      .......... .......... .......... .......... .......... .......... ..........
UTI89        .......... .......... .......... .......... .......... .......... ..........
E._albertii  .......... .......... .......... .......... .......... .......... ..........

          6021
K-12         AACGTCGTCG TATCTCCCTG GGTCTGAAAC AGTGCAAAGC TAACCCGTGG CAGCAGTTCG CGGAAACCCA
53638        .......... .......... .......... .......... .......... .......... ..........
ATCC_8739    .......... .......... .......... .......... .......... .......... ..........
ECOR_10      .......... .......... .......... .......... .......... .......... ..........
ECOR_11      .......... .......... .......... .......... .......... .......... ..........
B171         .......... .......... .......... .......... .......... ........T. ..........
E24377A      .......... .......... .......... .......... .......... .......... ..........
ECOR_34      .......... .......... .......... .......... .......... .......... ..........
ECOR_71      .......... .......... .......... .......... .......... .......... ..........
SE11         .......... .......... .......... .......... .......... .......... ..........
O157_Sakai   .......... .......... .......... .......... .......... .......... ..........
ECOR_36      .......... .......... .......... .......... .......... .......... ..........
ECOR_35      .......... .......... .......... .......... .......... .......... ..........
SMS-3-5      .......... .......... .......... .......... .......... .......... ..........
536          .......... .......... .......... .......... .......... .......... ..........
APEC_O1      .......... .......... .......... .......... .......... .......... ..........
CFT073       .......... .......... .......... .......... .......... .......... ..........
E2348/69     .......... .......... .......... .......... .......... .......... ..........
ECOR_51      .......... .......... .......... .......... .......... .......... ..........
ECOR_57      .......... .......... .......... .......... .......... .......... ..........
UTI89        .......... .......... .......... .......... .......... .......... ..........
E._albertii  .......... .......... .......... .......... .......... .......... ..........
```

```
           6091
K-12         CAACAAGGGC GACCGTGTTG AAGGTAAAAT CAAGTCTATC ACTGACTTCG GTATCTTCAT CGGCTTGGAC
53638        .......... .......... .......... .......... .......... .......... ....C.....
ATCC_8739    .......... .......... .......... .......... .......... .......... ....C.....
ECOR_10      .......... .......... .......... .......... .......... .......... ....C.....
ECOR_11      .......... .......... .......... .......... .......... .......... ....C.....
B171         .......... .......... .......... .......... .......... .......... ....C.....
E24377A      .......... .......... .......... .......... .......... .......... ....C.....
ECOR_34      .......... .......... .......... .......... .......... .......... ....C.....
ECOR_71      .......... .......... .......... .......... .......... .......... ....C.....
SE11         .......... .......... .......... .......... .......... .......... ....C.....
O157_Sakai   .......... .......... .......... .......... .......... .......... ....C.....
ECOR_36      .........T .......... .......... .......... .......... .......... ....C.....
ECOR_35      .........T .......... .......... .......... .......... .......... ....C.....
SMS-3-5      .........T .......... .......... .......... .......... .......... ....C.....
536          .......... .......... .......... .......... .......... .......... ....C.....
APEC_O1      .......... .......... .......... .......... .......... .......... ....C.....
CFT073       .......... .......... .......... .......... .......... .......... ....C.....
E2348/69     .......... .......... .......... .......... .......... .......... ....C.....
ECOR_51      .......... .......... .......... .......... .......... .......... ....C.....
ECOR_57      .......... .......... .......... .......... .......... .......... ....C.....
UTI89        .......... .......... .......... .......... .......... .......... ....C.....
E._albertii  .......... ..T....... .......... .......... .......... .......... ....C.....

           6161
K-12         GGCGGCATCG ACGGCCTGGT TCACCTGTCT GACATCTCCT GGAACGTTGC AGGCGAAGAA GCAGTTCGTG
53638        .......... .......... .......... .......... .......... .......... ..........
ATCC_8739    .......... .......... .......... .......... .......... .......... ..........
ECOR_10      .......... .......... .......... .......... .......... .......... ..........
ECOR_11      .......... .......... .......... .......... .......... .......... ..........
B171         .......... .......... .......... .......... .......... .......... ..........
E24377A      .......... .......... .......... .......... .......... .......... ..........
ECOR_34      .......... .......... .......... .......... .......... .......... ..........
ECOR_71      .......... .......... .......... .......... .......... .......... ..........
SE11         .......... .......... .......... .......... .......... .......... ..........
O157_Sakai   .......... .......... .......... .......... .......... .......... ..........
ECOR_36      .......... .......... .......... .......... .......... .......... ..........
ECOR_35      .......... .......... .......... .......... .......... .......... ..........
SMS-3-5      .......... .......... .......... .......... .......... .......... ..........
536          .......... .......... .......... .......... .......... .......... ..........
APEC_O1      .......... .......... .......... .......... .......... .......... ..........
CFT073       .......... .......... .......... .......... .......... .......... ..........
E2348/69     .......... .......... .......... .......... .......... .......... ..........
ECOR_51      .......... .......... .......... .......... .......... .......... ..........
ECOR_57      .......... .......... .......... .......... .......... .......... ..........
UTI89        .......... .......... .......... .......... .......... .......... ..........
E._albertii  .......... .......... .......... .......... .......... .......... ..........

           6231
K-12         AATACAAAAA AGGCGACGAA ATCGCTGCAG TTGTTCTGCA GGTTGACGCA GAACGTGAAC GTATCTCCCT
53638        .......... .......... .......... .......... .......... .......... ..........
ATCC_8739    .......... .......... .......... .......... .......... .......... ..........
ECOR_10      .......... .......... .......... .......... .......... .......... ..........
ECOR_11      .......... .......... .......... .......... .......... .......... ..........
B171         .......... .......... .......... .......... .......... .......... ..........
E24377A      .......... .......... .......... .......... .......... .......... ..........
ECOR_34      .......... .......... .......... .......... .......... .......... ..........
ECOR_71      .......... .......... .......... .......... .......... .......... ..........
SE11         .......... .......... .......... .......... .......... .......... ..........
O157_Sakai   .......... .......... .......... .......... .......... .......... ..........
ECOR_36      .......... .......... .......... .......... .......... .......... ..........
ECOR_35      .......... .......... .......... .......... .......... .......... ..........
SMS-3-5      .......... .......... .......... .......... .......... .......... ..........
536          .......... .......... .......... .......... .......... .......... ..........
APEC_O1      .......... .......... .......... .......... .......... .......... ..........
CFT073       .......... .......... .......... .......... .......... .......... ..........
E2348/69     .......... .......... .......... .......... .......... .......... ..........
ECOR_51      .......... .......... .......... .......... .......... .......... ..........
ECOR_57      .......... .......... .......... .......... .......... .......... ..........
UTI89        .......... .......... .......... .......... .......... .......... ..........
E._albertii  .......... .......... .......... .......... .......... .......... ..........
```

```
          6301
K-12        GGGCGTTAAA CAGCTCGCAG AAGATCCGTT CAACAACTGG GTTGCTCTGA ACAAGAAAGG CGCTATCGTA
53638       .......... .......... .......... .......... .......... .......... ..........
ATCC_8739   .......... .......... .......... .......... .......... .......... ..........
ECOR_10     .......... .......... .......... .......... .......... .......... ..........
ECOR_11     .......... .......... .......... .......... .......... .......... ..........
B171        .......... .......... .......... .......... .......... .......... ..........
E24377A     .......... .......... .......... .......... .......... .......... ..........
ECOR_34     .......... .......... .......... .......... .......... .......... ..........
ECOR_71     .......... .......... .......... .......... .......... .......... ..........
SE11        .......... .......... .......... .......... .......... .......... ..........
O157_Sakai  .......... .......... .......... .......... .......... .......... ..........
ECOR_36     .......... .......... .......... .......... .......... .......... ..........
ECOR_35     .......... .......... .......... .......... .......... .......... ..........
SMS-3-5     .......... .......... .......... .......... .......... .......... ..........
536         .......... .......... .......... .......... .......... .......... ..........
APEC_O1     .......... .......... .......... .......... .......... .......... ..........
CFT073      .......... .......... .......... .......... .......... .......... ..........
E2348/69    .......... .......... .......... .......... .......... .......... ..........
ECOR_51     .......... .......... .......... .......... .......... .......... ..........
ECOR_57     .......... .......... .......... .......... .......... .......... ..........
UTI89       .......... .......... .......... .......... .......... .......... ..........
E._albertii .......... .......... .......... .......... .......... .......... ..........

          6371
K-12        ACCGGTAAAG TAACTGCAGT TGACGCTAAA GGCGCAACCG TAGAACTGGC TGACGGCGTT GAAGGTTACC
53638       .......... .......... .......... .......... .......... .......... ..........
ATCC_8739   .......... .......... .......... .......... .......... .......... ..........
ECOR_10     .......... .......... .......... .......... .......... .......... ..........
ECOR_11     .......... .......... .......... .......... .......... .......... ..........
B171        .......... .......... .......... .......... .......... .......... ..........
E24377A     .......... .......... .......... .......... .......... .......... ..........
ECOR_34     .......... .......... .......... .......... .......... .......... ..........
ECOR_71     .......... .......... .......... .......... .......... .......... ..........
SE11        .......... .......... .......... .......... .......... .......... ..........
O157_Sakai  .......... .......... .......... .......... .......... .......... ..........
ECOR_36     .......... .......... .......... .......... .......... .......... ..........
ECOR_35     .......... .......... .......... .......... .......... .......... ..........
SMS-3-5     .......... .......... .......... .......... .......... .......... ..........
536         .......... .......... .......... .......... .......... .......... ..........
APEC_O1     .......... .......... .......... .......... .......... .......... ..........
CFT073      .......... .......... .......... .......... .......... .......... ..........
E2348/69    .......... .......... .......... .......... .......... .......... ..........
ECOR_51     .......... .......... .......... .......... .......... .......... ..........
ECOR_57     .......... .......... .......... .......... .......... .......... ..........
UTI89       .......... .......... .......... .......... .......... .......... ..........
E._albertii .......... .......... .......... .......... .......... .........A ..........

          6441
K-12        TGCGTGCTTC TGAAGCATCC CGTGACCGCG TTGAAGACGC TACCCTGGTT CTGAGCGTTG GCGACGAAGT
53638       .......... .......... .......... .......... .......... .......... ..........
ATCC_8739   .......... .......... .......... .......... .......... .......... ..........
ECOR_10     .......... .......... .......... .......... .......... .......... ..........
ECOR_11     .......... .......... .......... .......... .......... .......... ..........
B171        .......... .......... .......... .......... .......... .......... ..........
E24377A     .......... .......... .......... .......... .......... .......... ..........
ECOR_34     .......... .......... .......... .......... .......... .......... ..........
ECOR_71     .......... .......... .......... .......... .......... .......... ..........
SE11        .......... .......... .......... .......... .......... .......... ..........
O157_Sakai  .......... .......... .......... .......... .......... .......... ..........
ECOR_36     .......... .......... .......... .......... .......... .......... ..........
ECOR_35     .......... .......... .......... .......... .......... .......... ..........
SMS-3-5     .......... .......... .......... .......... .......... .......... ..........
536         .......... .......... .......... .......... .......... .......... ..........
APEC_O1     .......... .......... .......... .......... .......... .......... ..........
CFT073      .......... .......... .......... .......... .......... .......... ..........
E2348/69    .......... .......... .......... .......... .......... .......... ..........
ECOR_51     .......... .......... .......... .......... .......... .......... ..........
ECOR_57     .......... .......... .......... .......... .......... .......... ..........
UTI89       .......... .......... .......... .......... .......... .......... ..........
E._albertii .......... .......... .......... .......... ...T...... .......... ..........
```

```
           6511
K-12         TGAAGCTAAA TTCACCGGCG TTGATCGTAA AAACCGCGCA ATCAGCCTGT CTGTTCGTGC GAAAGACGAA
53638        .......... .......... .......... .......... .......... .......... ..........
ATCC_8739    .......... .......... .......... .......... .......... .......... ..........
ECOR_10      .......... .......... .......... .......... .......... .......... ..........
ECOR_11      .......... .......... .......... .......... .......... .......... ..........
B171         .......... .......... .......... .......... .......... .......... ..........
E24377A      .......... .......... .......... .......... .......... .......... ..........
ECOR_34      .......... .......... .......... .......... .......... .......... ..........
ECOR_71      .......... .......... .......... .......... .......... .......... ..........
SE11         .......... .......... .......... .......... .......... .......... ..........
O157_Sakai   .......... .......... .......... ......T... .......... .......... ..........
ECOR_36      .......... .......... .......... .......... .......... .......... ..........
ECOR_35      .......... .......... .......... .......... .......... .......... ..........
SMS-3-5      .......... .......... .......... .......... .......... .......... ..........
536          .......... .......... .......... .......... .......... .......... ..........
APEC_O1      .......... .......... .......... .......... .......... .......... ..........
CFT073       .......... .......... .......... .......... .......... .......... ..........
E2348/69     .......... .......... .......... .......... .......... .......... ..........
ECOR_51      .......... .......... .......... .......... .......... .......... ..........
ECOR_57      .......... .......... .......... .......... .......... .......... ..........
UTI89        .......... .......... .......... .......... .......... .......... ..........
E._albertii  .......... .....T.... .......... .......... .......... .......... ..........

           6581
K-12         GCTGACGAGA AAGATGCAAT CGCAACTGTT AACAAACAGG AAGATGCAAA CTTCTCCAAC AACGCAATGG
53638        .......... .......... .......... .......... .......... .......... ..........
ATCC_8739    .......... .......... .......... .......... .......... .......... ..........
ECOR_10      .......... .......... .......... .......... .......... .......... ..........
ECOR_11      .......... .......... .......... .......... .......... .......... ..........
B171         .......... .......... .......... .......... .......... .......... ..........
E24377A      .......... .......... .......... .......... .......... .......... ..........
ECOR_34      .......... .......... .......... .......... .......... .......... ..........
ECOR_71      .......... .......... .......... .......... .......... .......... ..........
SE11         .......... .......... .......... .......... .......... .......... ..........
O157_Sakai   .......... .......... .......... .......... .......... .......... ..........
ECOR_36      .......... .......... .......... .......... .......... .......... ..........
ECOR_35      .......... .......... .......... .......... .......... .......... ..........
SMS-3-5      .......... .......... .......... .......... .......... .......... ..........
536          .......... .......... .......... .......... .......... .......... ..........
APEC_O1      .......... .......... .......... .......... .......... .......... ..........
CFT073       .......... .......... .......... .......... .......... .......... ..........
E2348/69     .......... .......... .......... .......... .......... .......... ..........
ECOR_51      .......... .......... .......... .......... .......... .......... ..........
ECOR_57      .......... .......... .......... .......... .......... .......... ..........
UTI89        .......... .......... .......... .......... .......... .......... ..........
E._albertii  .......... .......... .......... .......... .......... .......... ..........

           6651
K-12         CTGAAGCTTT CAAAGCAGCT AAAGGCGAGT AATTCTCTGA CTCTTCGGGA TTTTTATTCC GAAGTTTGTT
53638        .......... .......... .......... .......... .......... .......... ..........
ATCC_8739    .......... .......... .......... .......... .......... .......... ..........
ECOR_10      .......... .......... .......... .......... .......... .......... ..........
ECOR_11      .......... .......... .......... .......... .......... .......... ..........
B171         .......... .......... .......... .......... .......... .......... ..........
E24377A      .......... .......... .......... .......... .......... .......... ..........
ECOR_34      .......... .......... .......... .......... .......... .......... ..........
ECOR_71      .......... .......... .......... .......... .......... .......... ..........
SE11         .......... .......... .......... .......... .......... .......... ..........
O157_Sakai   .......... .......... .......... .......... .......... .........T ..........
ECOR_36      .......... .......... .......... .......... .......... .......... ..........
ECOR_35      .......... .......... .......... .......... .......... .......... ..........
SMS-3-5      .......... .......... .......... .......... .......... .......... ..........
536          .......... .......... .......... .......... .......... .......... ..........
APEC_O1      .......... .......... .......... .......... .......... .......... ..........
CFT073       .......... .......... .......... .......... .......... .......... ..........
E2348/69     .......... .......... .......... .......... .......... .......... ..........
ECOR_51      .......... .......... .......... .......... .......... .......... ..........
ECOR_57      .......... .......... .......... .......... .......... .......... ..........
UTI89        .......... .......... .......... .......... .......... .......... ..........
E._albertii  .......... .......... .......... .......... .......... .......... .......AC.
```

```
            6721
K-12         GAGTTTACTT GACAGATTGC AGGTTTCGTC CTGTAATCAA GCACTAAGGG CGGCTACGGC CGCCCTTAAT
53638        .......... .......... .......... .......... .......... .......... ..........
ATCC_8739    .......... .......... .......... .......... .......... .......... ..........
ECOR_10      .......... .......... .......... .......... .......... .......... ..........
ECOR_11      .......... .......... .......... .......... .......... .......... ..........
B171         .......... .......... .......... .......... .......... .......... ..........
E24377A      .......... .......... .......... .......... .......... .......... ........G.
ECOR_34      .......... .......... .......... .......... .......... .......... ..........
ECOR_71      .......... .......... .......... .......... .......... .......... ..........
SE11         .......... .......... .......... .......... .......... .......... ..........
O157_Sakai   .......... .......... .......... .......... .......... .......... ..........
ECOR_36      .......... .......... .......... .......... .......... .......... ..........
ECOR_35      .......... .......... .......... .......... .......... .......... ..........
SMS-3-5      .......... .......... .......... .......... .......... .......... ..........
536          .......... .......... .......... .......... .......... .......... ..........
APEC_O1      .......... .......... .......... .......... .......... .......... ..........
CFT073       .......... .......... .......... .......... .......... .......... ..........
E2348/69     .......... .......... .......... .......... .......... .......... ..........
ECOR_51      .......... .......... .......... .......... .......... .......... ..........
ECOR_57      .......... .......... .......... .......... .......... .......... ..........
UTI89        .......... .......... .......... .......... .......... .......... ..........
E._albertii  .......... .......... G......... .......... .......... .......... ..........

            6791
K-12         CAATGCAGCA ACAGCAGCCG CTTAATTTGC CTTTAAGGAA CCGGAGGAAT CATGACCAAG TCAGAATTGA
53638        .......... .......... .......... .......... .......... .......... ..........
ATCC_8739    .......... .......... .......... .......... .......... .......... ..........
ECOR_10      .......... .......... .......... .......... .......... .......... ..........
ECOR_11      .......... .......... .......... .......... .......... .......... ..........
B171         .......... .......... .......... .......... .......... .......... ..........
E24377A      .......... .......... .......... .......... .......... .......... ..........
ECOR_34      .......... .......... .......... .......... .......... .......... ..........
ECOR_71      .......... .......... .......... .......... .......... .......... ..........
SE11         .......... .......... .......... .......... .......... .......... ..........
O157_Sakai   .......... .......... .......... .......... .......... .......... ..........
ECOR_36      .......... .......... ..A....... .......... .......... .......... ..........
ECOR_35      .......... .......... ..A....... .......... .......... .......... ..........
SMS-3-5      .......... .......... .......... .......... .......... .......... ..........
536          .......... .......... .......... .......... .......... .......... ..........
APEC_O1      .......... .......... .......... .......... .......... .......... ..........
CFT073       .......... .......... .......... .......... .......... .......... ..........
E2348/69     .......... .......... .......... .......... .......... .......... ..........
ECOR_51      .......... .......... .......... .......... .......... .......... ..........
ECOR_57      .......... .......... .......... .......... .......... .......... ..........
UTI89        .......... .......... .......... .......... .......... .......... ..........
E._albertii  .......... .......... ..A....... .......... .......... .......... ..........

            6861
K-12         TAGAAAGACT TGCCACCCAG CAATCGCACA TTCCCGCCAA GACGGTTGAA GATGCAGTAA AAGAGATGCT
53638        .......... .......... .......... .......... .......... .......... ..........
ATCC_8739    .......... .......... .......... .......... .......... .......... ..........
ECOR_10      .......... .......... .......... .......... .......... .......... ..........
ECOR_11      .......... .......... .......... .......... .......... .......... ..........
B171         .......... .......... .......... .......... .......... .......... ..........
E24377A      .......... .......... .......... .......... .......... .......... ..........
ECOR_34      .......... .......... .......... .......... .......... .......... ..........
ECOR_71      .......... .......... .......... .......... .......... .......... ..........
SE11         .......... .......... .......... .......... .......... .......... ..........
O157_Sakai   .......... .......... .......... .......... .......... .......... ..........
ECOR_36      .......... .......... .......... .......... .......... .......... ..........
ECOR_35      .......... .......... .......... .......... .......... .......... ..........
SMS-3-5      .......... .......... .......... .......... .......... .......... ..........
536          .......... .......... .......... .......... .......... .......... ..........
APEC_O1      .......... .......... .......... .......... .......... .......... ..........
CFT073       .......... .......... .......... .......... .......... .......... ..........
E2348/69     .......... .......... .......... .......... .......... .......... ....T.....
ECOR_51      .......... .......... .......... .......... .......... .......... ..........
ECOR_57      .......... .......... .......... .......... .......... .......... ..........
UTI89        .......... .......... .......... .......... .......... .......... ..........
E._albertii  .......... .......... .......... ......T... A......... .......... ..........
```

```
            6931
K-12        GGAGCATATG GCCTCGACTC TTGCGCAGGG CGAGCGTATT GAAATCCGCG GTTTCGGCAG TTTCTCTTTG
53638       .......... .......... .......... .......... .......... .......... ..........
ATCC_8739   .......... .......... .......... .......... .......... .......... ..........
ECOR_10     .......... .......... .......... .......... .......... .......... ..........
ECOR_11     .......... .......... .......... .......... .......... .......... ..........
B171        .......... .......... .......... .......... .......... .......... ..........
E24377A     .......... .......... .......... .......... .......... .......... ..........
ECOR_34     .......... .......... .......... .......... .......... .......... ..........
ECOR_71     .......... .......... .......... .......... .......... .......... ..........
SE11        .......... .......... .......... .......... .......... .......... ..........
O157_Sakai  .......... .......... .......... .......... .......... .......... ..........
ECOR_36     .......... .......... .......... .......... .......... .......... ..........
ECOR_35     .......... .......... .......... .......... .......... .......... ..........
SMS-3-5     .......... .......... .......... .......... .......... .......... ..........
536         .......... .......... .......... .......... .......... .......... ..........
APEC_O1     .......... .......... .......... .......... .......... .......... ..........
CFT073      .......... .......... .......... .......... .......... .......... ..........
E2348/69    .......... .......... .......... .......... .......... .......... ..........
ECOR_51     .......... .......... .......... .......... .......... .......... ..........
ECOR_57     .......... .......... .......... .......... .......... .......... ..........
UTI89       .......... .......... .......... .......... .......... .......... ..........
E._albertii .......... .......... .......... .......... .......... .......... ..........

            7001
K-12        CACTACCGCG CACCACGTAC CGGACGTAAT CCGAAGACTG GCGATAAAGT AGAACTGGAA GGAAAATACG
53638       .......... .......... .......... .......... .......... .......... ..........
ATCC_8739   .......... .......... .......... .......... .......... .......... ..........
ECOR_10     .......... .......... .......... .......... .......... .......... ..........
ECOR_11     .......... .......... .......... .......... .......... .......... ..........
B171        .......... .......... .......... .......... .......... .......... ..........
E24377A     .......... .......... .......... .......... .......... .......... ..........
ECOR_34     .......... .......... .......... .......... .......... .......... ..........
ECOR_71     .......... .......... .......... .......... .......... .......... ..........
SE11        .......... .......... .......... .......... .......... .......... ..........
O157_Sakai  .......... .......... .......... .......... .......... .......... ..........
ECOR_36     .......... .......... .......... .......... .......... .......... ..........
ECOR_35     .......... .......... .......... .......... .......... .......... ..........
SMS-3-5     .......... .......... .......... .......... .......... .......... ..........
536         .......... .......... .......... .......... .......... .......... ..........
APEC_O1     .......... .......... .......... .......... .......... .......... ..........
CFT073      .......... .......... .......... .......... .......... .......... ..........
E2348/69    .......... .......... .......... .......... .......... .......... ..........
ECOR_51     .......... .......... .......... .......... .......... .......... ..........
ECOR_57     .......... .......... .......... .......... .......... .......... ..........
UTI89       .......... .......... .......... .......... .......... .......... ..........
E._albertii .......... ....T..... .......... .......... .......... G......... ..........

            7071
K-12        TTCCTCACTT TAAACCTGGT AAAGAACTGC GCGATCGCGC CAATATTTAC GGTTAAGTTT TTTACTCAAA
53638       .......... .......... .......... .......... .......... .......... ..........
ATCC_8739   .......... .......... .......... .......... .......... .......... ..........
ECOR_10     .......... .......... .......... .......... .......... .......... ..........
ECOR_11     .......... .......... .......... .......... .......... .......... ..........
B171        .......... .......... .......... .......... .......... .......... ..-.......
E24377A     .......... .......... .......... .......... .......... .......... ..-.......
ECOR_34     .......... .......... .......... .......... .......... .......... ..-.......
ECOR_71     .......... ......A... .......... .......... .......... .......... ..-.......
SE11        .......... .......... .......... .......... .......... .......... ..-.......
O157_Sakai  .......... .......... .......... .......... .......... .......... ..-.......
ECOR_36     .......... ......A... .......... .......... .......... .......... ..-.......
ECOR_35     .......... ......A... .......... .......... .......... .......... ..-.......
SMS-3-5     .......... ......A... .......... .......... .......... .......... ..-.......
536         .......... ......A... .......... .......... .......... .......... ..-.......
APEC_O1     .......... ......A... .......... .......... .......... .......... ..-.......
CFT073      .......... ......A... .......... .......... .......... .......... ..-.......
E2348/69    .......... ......A... .......... .......... .......... .......... ..-.......
ECOR_51     .......... ......A... .......... .......... .......... .......... ..-.......
ECOR_57     .......... ......A... .......... .......... .......... .......... ..-.......
UTI89       .......... ......A... .......... .......... .......... .......... ..-.......
E._albertii .......... ......C... .......... .......... .......... .......... .--.AGA...
```

```
          7141
K-12         CTTGAACGAG AGAAAAGCAC CTGTCGGGTG CTTTTTTCAT TTCTCTAATC TGGAACTGGA AGCTGCCTCG
53638        .......... .......... .......... .......... .......... .......... ..........
ATCC_8739    .......... .......... .......... .......... .......... .......... ..........
ECOR_10      .......... .......... .......... .......... .......... .......... ..........
ECOR_11      .......... .......... .......... .......... .......... .......... ..........
B171         .......... .......... .......... .......... .......... .......... ......T...
E24377A      ........C. .......... .......... .......... .......... A......... ..........
ECOR_34      ........C. .......... .......... .......... .......... A......... ..........
ECOR_71      ........C. .......... .......... .......... .......... A......... ..........
SE11         ........C. .......... .......... .......... .......... A......... ..........
O157_Sakai   .......... .......... .......... .......... ..A....... .......... ..........
ECOR_36      ........C. .......... .......... .......... .......... .......... ...A.T....
ECOR_35      ........C. .......... .......... .......... ..T....... .......... ...A.T....
SMS-3-5      ........C. .......... .......... .......... .......... .......... ..........
536          ........C. .......... .......... .......... .......... .......... ..........
APEC_O1      ........C. .......... .......... .......... .......... .......... ..........
CFT073       ........C. .......... .......... .......... .......... .......... ..........
E2348/69     ........C. .......... .......... .......... .......... .......... ..........
ECOR_51      ........C. .......... .......... .......... .......... .......... ..........
ECOR_57      ........C. .......... .......... .......... .......... .......... ..........
UTI89        ........C. .......... .......... .......... .......... .......... ..........
E._albertii  .....G..C. .A........ ...G.A.... .......... ....G.C... .......... ......T...

          7211
K-12         CAGAGTTTTG AACAGTTTTC ACCCTTTCGT TAAATTCTTC TGAATATGCC TCGGGGAACG CAAAATTCCC
53638        .......... .......... .......... .......... .......... .......... ..........
ATCC_8739    .......... .......... .......... .......... .......... .......... ..........
ECOR_10      .......... .......... .......... .......... .......... .......... ..........
ECOR_11      .......... .......... .......... .......... .......... .......... ..........
B171         .......... .G........ .....C.... .......... ....CG.... .......... ..........
E24377A      .......... .G........ .......... .......... .....G.... .......... ..........
ECOR_34      .......... .G........ .......... .......... .....G.... .......... ..........
ECOR_71      .......... .G........ .....C.... .......... ....CG.... .......... ..........
SE11         .......... .G........ .......... .......... .....G.... .......... ..........
O157_Sakai   .......... .G........ .......... .......... .....G.... .......... ..........
ECOR_36      .......... .......... .....C.... .......... ....CG.... .......... .....C....
ECOR_35      .......... .......... .....C.... .......... ....CG.... .......... .....C....
SMS-3-5      .......... .G........ .....A.... .......... ....CG.... .......... .....CA...
536          .......... .G........ .....A.... .......... ....CG.... .......... .....C....
APEC_O1      .......... .G........ .....A.... .......... ....CG.... .......... .....C....
CFT073       .......... .G........ .....A.... .......... ....CG.... .......... .....C....
E2348/69     .......... .G........ .....A.... .......... ....CG.... .......... .....C....
ECOR_51      .......... .G........ .....A.... .......... ....CG.... .......... .....C....
ECOR_57      .......... .G........ .....A.... .......... ....CG.... .......... .....C....
UTI89        .......... .G........ .....A.... .......... ....CG.... .......... .....C....
E._albertii  .......... TGT....... ...T..C... .........T ....CGC... ........A. .....C....

          7281
K-12         ACCAGACAAC CGCTCAACAA AGTTGCACAC TTTCCATAAA CAGGGAGGGG TGCATGAAAA TAACGACAGT
53638        .......... .......... .......... .......... .......... .......... ..........
ATCC_8739    .......... .......... .......... .......... .......... .......... ..........
ECOR_10      .......... .......... .......... .......... .......... .......... ..........
ECOR_11      .......... .......... .......... .......... .......... .......... ..........
B171         .......... .......... .......... .......... .......... .......... ..........
E24377A      .......... .......... .......... .......... .......... .......... ..........
ECOR_34      .......... .......... .......... .......... .......... .......... ..........
ECOR_71      .......... .......... .......... .......... .......... .......... ..........
SE11         .......... .......... .......... .......... .......... .......... ..........
O157_Sakai   .......... .......... .......... .......... .......... .......... ..........
ECOR_36      .......... .......... .......... .......... .......... .......... ..........
ECOR_35      .......... .......... .......... .......... .......... .......... ..........
SMS-3-5      .......... ..T....... .......... ....T..... .........-
536          .......... ..T....... .......... ....T..... .........-
APEC_O1      .......... ..T....... .......... ....T..... .........-
CFT073       .......... ..T....... .......... ....T..... .........-
E2348/69     .......... ..T....... .......... ....T..... .........-
ECOR_51      .......... ..T....... .......... ....T..... .........-
ECOR_57      .......... ..T....... .......... ....T..... .........-
UTI89        .......... ..T....... .......... ....T..... .........-
E._albertii  G......... ....T..... .A........ ......A... ........A. .......... .......G..
```

```
           7351
K-12         CGGTGTATGC ATAATTAGCG GAATTTTTCC GTTGCTGATT TTGCCCCAAT TGCCTGGGAC ATTAACCCTT
53638        .......... .......... .......... .......... .......... .......... ..........
ATCC_8739    .......... .......... .......... .......... .......... .......... ..........
ECOR_10      .......... .......... .......... .......... .......... .......... ..........
ECOR_11      .......... .......... .......... .......... .......... .......... ..........
B171         ...C...... ......T... .......... .......... .......... .......... ..........
E24377A      ...C...... ......T... .......... .......... .......... .......... ..........
ECOR_34      ...C...... ......T... .......... .......... .......... .......... ..........
ECOR_71      ...C...... ......T... .......... .......... .......... .......... ..........
SE11         .......... .......... .......... .......... .......... .......... ..........
O157_Sakai   ...C...... ......T... .......... .......... .......... .......... ..........
ECOR_36      ...C...... ......T... .......... .......G.. .......... .......... ..........
ECOR_35      ...C...... ......T... .......... .......G.. .......... .......... ..........
SMS-3-5      ...C...... ......T... .......... .......... .......... ....A..... .G........
536          ...C...... ......T... .......... .......... .......... ....A..... .G........
APEC_O1      ...C...... ......T... .......... .......... .......... .......... .G........
CFT073       ...C...... ......T... .......... .......... .......... .......... .G........
E2348/69     ...C...... ......T... .......... .......... .......... .......... .G........
ECOR_51      ...C...... ......T... .......... .......... .......... .......... .G........
ECOR_57      ...C...... ......T... .......... .......... .......... .......... .G........
UTI89        ...C...... ......T... .......... .......... .......... .......... .G........
E._albertii  ...C...... ......T... .......... .C........ .....G.... .......... ...G......

           7421
K-12         GCGTTTCTGA CTCTCTTCGC CTGCGTACTG GCATTTATCC CTGTTAAAAC CGTCCGTTAT ATCGCGCTGA
53638        .......... .......... .......... .......... .......... .......... ..........
ATCC_8739    .......... .......... .......... .......... .......... .......... ..........
ECOR_10      .......... .......... .......... .......... .......... .......... ..........
ECOR_11      .......... .......... .......... .......... .......... .......... ..........
B171         .......... ..A....... .......... .......... .......... ...A...... ..........
E24377A      .......... .......... .......... .......... .......... .......... ..........
ECOR_34      .......... .......... .......... .......... .......... .......... ..........
ECOR_71      .......... .......... .......... .......... .......... .......... ..........
SE11         .......... .......... .......... .......... .......... .......... ..........
O157_Sakai   .......... .......... .......... .......... .......... .......... ..........
ECOR_36      .......... ..G....... .......... .T........ ..T.....G. .......... ..........
ECOR_35      .......... ..G....... .......... .T........ ..T.....G. .......... ..........
SMS-3-5      .......... .......... ...T...... .......... .......... .......... ..........
536          .......... .......... ...T...... .......... .......... .......... ..........
APEC_O1      .......... .......... ...T...... .......... .......... .......... ..........
CFT073       .......... .......... ...T...... .......... .......... .......... ....T.....
E2348/69     .......... .......... ...T...... .......... .......... .......... ....T.....
ECOR_51      .......... .......... ...T...... .......... .......... .......... ....T.....
ECOR_57      .......... .......... ...T...... .......... .......... .......... ....T.....
UTI89        .......... .......... ...T...... .......... .......... .......... ..........
E._albertii  ....G..... ..GC...... .........T ........TT ........G. .......... ..AT......

           7491
K-12         CGTTGCTGTT TTTCGTTTGG GGCATATTAT CAGCAAAGCA AATTTTGTGG GCAGGAGAAA CCTTAACTGG
53638        .......... .......... .......... .......... .......... .......... ..........
ATCC_8739    .......... .......... .......... .......... .......... .......... ..........
ECOR_10      .......... .......... .......... .......... .......... .......... ..........
ECOR_11      .......... .......... .......... .......... .......... .......... ..........
B171         .......... .......... ..T......G .G........ .......... .......... ..........
E24377A      .......... .......... .......... .......... .......... .......... ..........
ECOR_34      .......... .......... .......... .......... .......... .......... ..........
ECOR_71      .....T.... .......... .........G .......... .......... .......... ..........
SE11         .......... .......... .......... .......... .......... .......... ..........
O157_Sakai   .......... .......... .......... .......... ........T. .......... ..........
ECOR_36      .......... ...TC..... .......C.G .G........ .......... .......... ..........
ECOR_35      .......... ...TC..... .......C.G .G........ .......... .......... ..........
SMS-3-5      .......... .......... .........G .......... .......... .......... ..........
536          .......... .......... .........G .......... .......... .......... ..........
APEC_O1      .......... .......... .........G .......... .......... .......... ..........
CFT073       .......... .......... .........G .......... .......... .......... ..........
E2348/69     .......... .......... .........G .......... .......... .......... ..........
ECOR_51      .......... .......... .........G .......... .......... .......... ..........
ECOR_57      .......... .......... .........G .......... .......... .......... ..........
UTI89        .......... .......... .........G .......... .......... .......... ..........
E._albertii  .A..A..... ...TC..... ...G.....G ....G..... ......A... .......... .......CA.
```

```
          7561
K-12         CGCGACGCAG GATGCAATTG TTGAGATCAC TGCAACTGAC GGCATGACCA CTCATTACGG TCAAATTACT
53638        .......... .......... .......... .......... .......... .......... ..........
ATCC_8739    .......... .......... .......... .......... .......... .......... ..........
ECOR_10      .......... .......... .......... .......... .......... .......... ..........
ECOR_11      .......... .......... .......... .......... .......... .......... ..........
B171         T......... .......... .......... .A.T...... .......... .......... ......C...
E24377A      .......... .......... .......... .......... .......... .......... ..........
ECOR_34      .......... .......... .......... .......... .......... .......... ..........
ECOR_71      .......... .......... .......... ...T...... ..T....... .......... ..........
SE11         .......... .......... .......... .......... .......... .......... ..........
O157_Sakai   .......... .......... .......... ...T...... .......... .......... ..........
ECOR_36      .......... ........A. .......... ...T...... .......... .......... ......C...
ECOR_35      .......... ........A. .......... ...T...... .......... .......... ......C...
SMS-3-5      .......... ........A. .......... ...T...... .......... .......... ......C...
536          .......... .......... .........T ...T...... .......... .......... ..........
APEC_O1      .......... .......... .........T ...T...... .......... .......... ..........
CFT073       .......... .......... .........T ...T...... .......... .......... ..........
E2348/69     .......... .......... .........T ...T...... .......... .......... ..........
ECOR_51      .......... .......... .........T ...T...... .......... .......... ..........
ECOR_57      .......... .......... .........T ...T...... .......... .......... ..........
UTI89        .......... .......... .........T ...T...... .......... .......... ..........
E._albertii  .A........ ....T...C. .C.....T.. C..C.....T ..T....... .......T.. C........C

          7631
K-12         CATCTACAAG GTCGACGTAT ATTCCCTGCG TCAGGTCTCG TGATGTATGG CGAATATCTT CCGCAAGCGG
53638        .......... .......... .......... .......... .......... .......... ..........
ATCC_8739    .......... .......... .......... .......... .......... .......... ..........
ECOR_10      .......... .......... .......... .......... .......... .......... ..........
ECOR_11      .......... .......... .......... .......... .......... .......... ..........
B171         .......... .......... .......... C....C.... ..C....... .......... ..........
E24377A      .......... .......... .......... C....C..T. ..C....... .......... ..........
ECOR_34      .......... .......... .......... C....C..T. ..C....... .......... ..........
ECOR_71      .......... .......... .......... C..A.C..T. ..C....... .......... ..........
SE11         .......... .......... .......... .......... .......... .......... ..........
O157_Sakai   T......... ...A...... .......... C....C..T. ..C....... .......... ..........
ECOR_36      .........A .......... .......... C....C.... ..C....... .......... ..........
ECOR_35      .........A .......... .......... C....C.... ..C....... .......... ..........
SMS-3-5      .........A .......... .......... C....C.... .AC....... .......... ..........
536          .......... .......... .......... C....C.... .AC....... .......... ..........
APEC_O1      .......... .......... .......... C....C.... .AC....... .......... ..........
CFT073       .......... .......... .......... C....C.... .AC....... .......... ..........
E2348/69     .......... .......... ........T. C....C.... .AC....... .......... ..........
ECOR_51      .......... .......... .......... C....C.... .AC....... .......... ..........
ECOR_57      .......... .......... .......... C....C.... .AC....... .......... ..........
UTI89        .......... .......... .......... C....C.... .AC....... .......... ..........
E._albertii  .G...C..G. .A..G.AGC. ......GT.. C.....A..A CAC....... .......T.G ..........

          7701
K-12         TTTGTGCTGG ACAACAATGG TCAATGAAAC TCAAAGTTCG TGCAGTTCAT GGCCAACTTA ATGATGGCGG
53638        .......... .......... .......... .......... .......... .......... ..........
ATCC_8739    .......... .......... .......... .......... .......... .......... ..........
ECOR_10      .......... .......... .......... .......... .......... .......... ..........
ECOR_11      .......... .......... .......... .......... .......... .......... ..........
B171         .......... .....TG... .......... .......... ...CA..... ..T....... ..........
E24377A      .......... .......... .......... .......... .......... .......... ..........
ECOR_34      .......... .......... .......... .......... ...G...... .......... ..........
ECOR_71      .......C.. .....TG... .......... .......... ...C...... ..T....... ..........
SE11         .......... .......... .......... .......... .......... .......... ..........
O157_Sakai   .......C.. .....TG... .......... .......... ...C...... ..T....... ..........
ECOR_36      .......... .......... .......... .......... .......... .......... ..........
ECOR_35      .......... .......... .......... .......... .......... .......... ..........
SMS-3-5      .......C.. ....GT.... .......... .......... .......... ..T....... ..........
536          .......C.. ....GT.... .......... .......... .......... ..T....... ..........
APEC_O1      .......C.. ....GT.... .......... .......... .......... ..T....... ..........
CFT073       .......C.. ....GT.... .......... .......... .......... ..T....... ..........
E2348/69     .......C.. ....GT.... .......... .......... .......... ..T....... ..........
ECOR_51      .......C.. ....GT.... .......... .......... .......... ..T....... ..........
ECOR_57      .......C.. ....GT.... .......... .......... .......... ..T....... ..........
UTI89        .......C.. ....GT.... .......... .......... .......... ..T....... ..........
E._albertii  .......C.. G..G...... G.......G. .......A.. ...G.....C ..T....... ....C.....
```

```
          7771
K-12            CTTTGATAGC CAGCGTTATG CCATTGCCCA GCATCAGCCG CTCACCGGCC GTTTTCTGCA GGCAAGTGTC
53638           .......... .......... .......... .......... .......... .......... ..........
ATCC_8739       .......... .......... .......... .......... .......... .......... ..........
ECOR_10         .......... .......... .......... .......... .......... .......... ..........
ECOR_11         .......... .......... .......... .......... .......... .......... ..........
B171            .......... .......... .......... .......... .......... .C........ ..........
E24377A         .......... .......... .......... .......... .......... .C........ ..........
ECOR_34         .......... .......... .......... ......A... .......... .C........ .........T
ECOR_71         .......... .......... .......... .......... .......... .C........ .........T
SE11            .......... .......... .......... .......... .......... .......... ..........
O157_Sakai      .......... .......... .......... .......... .......... .C........ .........T
ECOR_36         .......... .......... .......... .......... .......... .C........ ..........
ECOR_35         .......... .......... .......... .......... .......... .C........ ..........
SMS-3-5         .......... .......... .......... ......A... .......... .......... ..........
536             .......... .......... .......... ......A... .......... .......... ..........
APEC_O1         .......... .......... .......... ......A... .......... .......... ..........
CFT073          .......... .......... .......... ......A... .......... .......... ..........
E2348/69        .......... .......... .......... ......A... .......... .......... ..........
ECOR_51         .......... .......... .......... ......A... .......... .......... ..........
ECOR_57         .......... .......... .......... ......A... .......... .......... ..........
UTI89           .......... .......... .......... ......A... .......... .......... ..........
E._albertii     T.....C... ..A....C.. .TG....... ...C...... ..A.....G. ACA....T.. ...G......

          7841
K-12            ATTGAACCGA ATTGTAGCCT GCGTGCACAG TATCTGGCGT CACTACAAAC AACGCTGCAA CCCTATCCGT
53638           .......... .......... .......... .......... .......... .......... ..........
ATCC_8739       .......... .......... .......... .......... .......... .......... ..........
ECOR_10         .......... .......... .......... .......... .......... .......... ..........
ECOR_11         .......... .......... .......... .......... .......... .......... ..........
B171            .......... .......... ...G...... .......... .......... .......... ......AT..
E24377A         .......... .......... .......... .......... .......... .......... ......AT..
ECOR_34         .......... .......... .......... .......... .......... .......... ..........
ECOR_71         .......... .......... .......... .......... .......... .......... ..........
SE11            .......... .......... .......... .......... .......... .......... ..........
O157_Sakai      .......... .......... .......... .......... .......... .......... ..........
ECOR_36         .......... ........T. .......... .......... ..T....... .......... ..........
ECOR_35         .......... ........T. .......... .......... ..T....... .......... ..........
SMS-3-5         .......... .......... .......... .......... ..T....... .......... ..........
536             .......... .......... .......... .......... .......... .......... ......AT..
APEC_O1         .......... .......... .......... .......... .......... .......... ......AT..
CFT073          .......... .......... .......... .......... .......... .......... ......AT..
E2348/69        .......... .......... .......... .......... .......... .......... ......AT..
ECOR_51         .......... .......... .......... .......... .......... .......... ......AT..
ECOR_57         .......... .......... .......... .......... .......... .......... ......AT..
UTI89           .......... .......... .......... .......... .......... .......... ......AT..
E._albertii     ..C......G G....C.T.. ...A..G..A .......... .GT......T G..T...... ..........

          7911
K-12            GGAATGCGGT TATTCTTGGT TTAGGTATGG GGGAACGGTT ATCCGTCCCC AAAGAAATCA AAAATATCAT
53638           .......... .......... .......... .......... .......... .......... ..........
ATCC_8739       .......... .......... .......... .......... .......... .......... ..........
ECOR_10         .......... .......... .......... .......... .......... .......... ..........
ECOR_11         .......... .......... .......... .......... .......... .......... ..........
B171            .......... .......... .......... ...T...... .........T .......... ..........
E24377A         .......... .......... .......... .......... .......... .......... ..........
ECOR_34         .......... .......... .......... .......... .......... .......... ..........
ECOR_71         .......... .......... .......... .......... .........T .......... ..........
SE11            .......... .......... .......... .......... .......... .......... ..........
O157_Sakai      .......... .......... .......... .......... .........T .......... ..........
ECOR_36         .......A.. .......... .......... .......... .........T .......... ..........
ECOR_35         .......A.. .......... .......... .......... .........T .......... ..........
SMS-3-5         .......... .......... .......... .......... ......T... .......... ..........
536             ....C..... .........A .......... .......... .......... .......... ..........
APEC_O1         ....C..... .........A ........A. .......... .......... .......... ..........
CFT073          ....C..... .........A .......... .......... .......T.. .......... ..........
E2348/69        ....C..... .........A .......... .......... .......... .......... ..........
ECOR_51         ....C..... .........A .......... .......... .......T.. .......... ..........
ECOR_57         ....C..... .........A .......... .......... .......T.. .......... ..........
UTI89           ....C..... .........A ........A. .......... .......... .......... ..........
E._albertii     ....C..... .......... .......... .C........ ...T..G..T .......... .......T..
```

```
           7981
K-12       GCGCGATACT GGAACGGCGC ATTTAATGGC GATATCGGGA TTGCACATCG CTTTTGCGGC GTTGCTGGCT
53638      .......... .......... .......... .......... .......... .......... ..........
ATCC_8739  .......... .......... .......... .......... .......... .......... ..........
ECOR_10    .......... .......... .......... .......... .......... .......... ..........
ECOR_11    .......... .......... .......... .......... .......... .......... ..........
B171       ...T...... .......... .......... .......... .......... .......... ..........
E24377A    ...T...... .......... .......... .......... .......... .......... ....T.....
ECOR_34    .......... .......... .......... .......... .......... .......... ..........
ECOR_71    ...T...... .......... .......... .......... .......... .......... ..........
SE11       .......... .......... .......... .......... .......... .......... ..........
O157_Sakai ...T...... .......... .......... .......... .......... .......... ..........
ECOR_36    .......... .......... .......... .......... .......... .......... ..........
ECOR_35    .......... .......... .......... .......... .......... .......... ..........
SMS-3-5    .......... .......... .......... .......... .....T.... .......... ..........
536        ...T...... .......... .......... ......A... .......... .......... ..........
APEC_O1    ...T...... .......... .......... ......A... .......... .......... ..........
CFT073     ...T...... .......... .......... ......A... .......... .......... ..........
E2348/69   ...T...... .......... .......... A.....A... .......... .......... ..........
ECOR_51    ...T...... .......... .......... ......A... .......... .......... ..........
ECOR_57    ...T...... .......... .......... ......A... .......... .......... ..........
UTI89      ...T...... .......... .......... ......A... .......... .......... ..........
E._albertii ....A....C ..T....... ....G..... .........C ..A..T.... .C........ A........A

           8051
K-12       GCCGGACTCA TTCGCAGTGG ACAAATTTTT CTGCCTGGGC GCTGGATCCA CTGGCAAATA CCATTAATTG
53638      .......... .......... .......... .......... .......... .......... ..........
ATCC_8739  .......... .......... .......... .......... .......... .......... ..........
ECOR_10    .......... .......... .......... .......... .......... .......... ..........
ECOR_11    .......... .......... .......... .......... .......... .......... ..........
B171       .......... .......... .......... .......... .......... .......... ..........
E24377A    .......... .....G.... G...G...A. .......... .......... .........G ..........
ECOR_34    .......... .......... .......... .......... .......... .......... ..........
ECOR_71    .......... .......... .......... .......... .......... .........G ..........
SE11       .......... .......... .......... .......... .......... .......... ..........
O157_Sakai .......... .......... .......... .......... .......... .........G ..........
ECOR_36    .......... .......... .......... .......... .......... .........G ..........
ECOR_35    .......... .......... .......... .......... .......... .........G ..........
SMS-3-5    .......... .....G.... G...G..... .......... .......... .........G ..........
536        ..A....... .....G.... G...G..... .........T .......... .........G ..........
APEC_O1    ..A....... .....G.... G...G..... .........T .......... .........G ..........
CFT073     ..A....... .....G.... G...G..... .........T .......... .........G ..........
E2348/69   ..A....... .....G.... G...G..... .........T .......... .........G ..........
ECOR_51    ..A....... .....G.... G...G..... .........T .......... .........G ..........
ECOR_57    ..A....... .....G.... G...G..... .........T .......... .........G ..........
UTI89      ..A....... .....G.... G...G..... .........T .......... .........G ..........
E._albertii ..G..G..A. .....G.G.. G......... ..T...TC.. .T.....TT. ......G..G .....G....

           8121
K-12       GCGGAATCTG CTGTGCTGCT TTTTATGCCT GGTTGACGGG AATGCAACCT CCTGCATTGC GTACCATGGT
53638      .......... .......... .......... .......... .......... .......... ..........
ATCC_8739  .......... .......... .......... .......... .......... .......... ..........
ECOR_10    .......... .......... .......... .......... .......... .......... ..........
ECOR_11    .......... .......... .......... .......... .......... .......... ..........
B171       ..A....... .......... .......... .......... .......... .......... .....G....
E24377A    .T........ .......... .......... .......... .......... .......... .....G....
ECOR_34    .T..G..... .......... .......... .......... .......... .......... .....G....
ECOR_71    .......... .......... .......... ..C....... .......... .......... .....G.T..
SE11       .......... .......... .......... .......... .......... .......... ..........
O157_Sakai .......... .......... .......... ..C....... .......... .......... .....G.T..
ECOR_36    .......... .......... .......... ..C....... .......... .......... .....G....
ECOR_35    .......... .......... .......... ..C....... .......... .......... .....G....
SMS-3-5    .......... .......... .......... ..C....T.. G......... .......... .....G....
536        .T........ .......... .......... ..C.T..... .......... .......... .....G....
APEC_O1    .T........ .......... .......... ..C.T..... .......... .......... .....G....
CFT073     .T........ .......... .......... ..C.T..... .......... .......... .....G....
E2348/69   .T........ .......... .......... ..C.T..... .......... .......... ....TG....
ECOR_51    .T........ .......... .......... ..C.T..... .......... .......... .....G....
ECOR_57    .T........ .......... .......... ..C.T..... .......... .......... .....G....
UTI89      .T........ .......... .......... ..C.T..... .......... .......... .....G....
E._albertii .......T.. ......C... .......... ..C....C.. ......G..G ..A...C... ....TG.A..
```

```
            8191
K-12            GGCGCTTGCT ACGTGGGGAA TGCTTAAGTT AAGTGGGCGA CAGTGGAGTG GCTGGGATGT ATGGATATGT
53638           .......... .......... .......... .......... .......... .......... ..........
ATCC_8739       .......... .......... .......... .......... .......... .......... ..........
ECOR_10         .......... .......... .......... .......... .......... .......... ..........
ECOR_11         .......... .......... .......... .......... .......... .......... ..........
B171            .......... .......... .......... .......... .......... .......... ..........
E24377A         .......... .......... .......... .......... .......... .......... ..........
ECOR_34         .......... .......... .......... .......... .......... .......... ..........
ECOR_71         .......... .......... .......... .......... .......... .......... ..........
SE11            .......... .......... .......... .......... .......... .......... ..........
O157_Sakai      .......... ........T. .......... .......... .......... .......... ..........
ECOR_36         .......... .......... .......... .......... .......... .......... ..........
ECOR_35         .......... .......... .......... .......... .......... .......... ..........
SMS-3-5         .......... .......... .......... .......... ..A....... .......... ..........
536             .........C .TT....... .......... ......A... ..A....... .......... ..........
APEC_O1         .........C .TT....... .......... ......A... ..A....... .......... ..........
CFT073          .........C .TT....... .......... ......A... ..A....... .......... ..........
E2348/69        .........C .TT....... .......... ......A... ..A....... .......... ..........
ECOR_51         .........C .TT....... .......... ......A... ..A....... .......... ..........
ECOR_57         .........C .TT....... .......... ......A... ..A....... .......... ..........
UTI89           .........C .TT....... .......... ......A... ..A....... .......... ..........
E._albertii     ......C..G ..C....... .......... G..C...A.G ..A.....CA .T........ .........C

            8261
K-12            TGTCTGGCGG CAATTTTGCT GATGGATCCT GTTGCCATTC TCTCGCAAAG TTTATGGCTC TCTGCCGCTG
53638           .......... .......... .......... .......... .......... .......... ..........
ATCC_8739       .......... .......... .......... .......... .......... .......... ..........
ECOR_10         .......... .......... .......... .......... .......... .......... ..........
ECOR_11         .......... .......... .......... .......... .......... .......... ..........
B171            .......... .......... .......... .......... .......... .......... ..........
E24377A         .......... .......... .......... .......... .......... .......... ..........
ECOR_34         .......... .......... .......... .......... .......... .......... ..........
ECOR_71         .......... .......... .......... .......... .......... .......... ..........
SE11            .......... .......... .......... .......... .......... .......... ..........
O157_Sakai      .......... .......... .......... ....T..... .......... .......... ..........
ECOR_36         .......... .......... .......... .......... .......... .......... ..........
ECOR_35         .......... .......... .......... .......... .......... .......... ..........
SMS-3-5         .......... .......... .......... .......... .......... .......... ..........
536             .......... .......... .......... .......... .......... .......... ..........
APEC_O1         .......... .......... .......... .......... .......... .........A ..........
CFT073          .......... .......... .......... .......... .......... .........A ..........
E2348/69        .......... .......... .......... .......... .......... .......... ..........
ECOR_51         .......... .......... .......... .......... .......... .........A ..........
ECOR_57         .......... .......... .......... .......... .......... .........A ..........
UTI89           .......... .......... .......... .......... .......... .........A ..........
E._albertii     ..C....... .......A.. .........C .....T.... .T........ C..G...... ..........

            8331
K-12            CGGTCGCGGC ATTGATATTT TGGTATCAGT GGTTTCCCTG TCCTGAGTGG CAACTGCCGC CGGTATTGCG
53638           .......... .......... .......... .......... .......... .......... ..........
ATCC_8739       .......... .......... .......... .......... .......... .......... ..........
ECOR_10         .......... .......... .......... .......... .......... .......... ..........
ECOR_11         .......... .......... .......... .......... .......... .......... ..........
B171            .......... .C....T... .....C.... .......... .......... .......... ..........
E24377A         .......... .C....T... .....C.... .......... .......... .......... ..........
ECOR_34         .......... .C....T... .....C.... .......... .......... .......... ..........
ECOR_71         .......... .......... .......... .......... .......... .......... ..........
SE11            .......... .......... .......... .......... .......... .......... ..........
O157_Sakai      .......... .......... .......... .......... .......... .......... ..........
ECOR_36         .......... .C....T... .......... ........G. .......... .......... ..........
ECOR_35         .......... .C....T... .......... ........G. .......... .......... ..........
SMS-3-5         .......... .C....T... .......... ........G. .......... .......... ..........
536             .......... .C....T... .......... .......... ...G...... ..G....... ..........
APEC_O1         .......... .C....T... .......... .......... ...G...... ..G....... ..........
CFT073          .......... .C....T... .......... .......... ...G...... ..G....... ..........
E2348/69        .......... .C....T... .......... .......... ...G...... ..G....... ..........
ECOR_51         .......... .C....T... .......... .......... ...G...... ..G....... ..........
ECOR_57         .......... .C....T... .......... .......... ...G...... ..G....... ..........
UTI89           .......... .C....T... .......... .......... ...G...... ..G....... ..........
E._albertii     .A..G..T.. GC.A..T..C .......... .......... .....TC... TCG....... .CACC.....
```

```
          8401
K-12         TGCAGTTGTT TCCCTCATCC ATCTGCAACT GGGAATCACA CTTCTGCTTA TGCCCGTGCA AATCGTCATA
53638        .......... .......... .......... .......... .......... .......... ..........
ATCC_8739    .......... .......... .......... .......... .......... .......... ..........
ECOR_10      .......... .......... .......... .......... .......... .......... ..........
ECOR_11      .......... .......... .......... .......... .......... .......... ..........
B171         .......... ........T. ..T....... .......... ..C....... .......... ..........
E24377A      .......... ........T. ..T....... .......... ..C....... ....T..... ..........
ECOR_34      .......... ........T. ..T....... .......... ..C....... ....T..... ..........
ECOR_71      .......... .......... .......... .......... .......... .......... ..........
SE11         .......... .......... .......... .......... .......... .......... ..........
O157_Sakai   .......... .......... .......... .......... .......... .......... .........T
ECOR_36      .......... .......... .......... .......... ..C....... .......... ..........
ECOR_35      .......... .......... .......... .......... ..C....... .......... ..........
SMS-3-5      ....C..... .......... .......... .......... ..C....... .......... ..........
536          C..G...... .......... .......... .........C ..C....... .......... ...T......
APEC_O1      C..G...... .......... .......... .........C ..C....... .......... ...T......
CFT073       C..G...... .......... .......... .........C ..C....... .......... ...T......
E2348/69     C..G...... .......... .......... .........C ..C....... .......... ...T......
ECOR_51      C..G...... .......... .......... .........C ..C....... .......... ...T......
ECOR_57      C..G...... .......... .......... .........C ..C....... .......... ...T......
UTI89        C..G...... .......... .......... .........C ..C....... .......... ...T......
E._albertii  CA...CC... ..A....... .......GT. ......A..G ..C...T.AC .A..AA.A.. .........G

          8471
K-12         TTTCATGGCA TTAGTCTGAC CTCGTTTATT GCAAATCTAT TAGCAATTCC CTTGGTGACA TTTATCACGG
53638        .......... .......... .......... .......... .......... .......... ..........
ATCC_8739    .......... .......... .......... .......... .......... .......... ..........
ECOR_10      .......... .......... .......... .......... .......... .......... ..........
ECOR_11      .......... .......... .......... .......... .......... .......... ..........
B171         .......... .......... .......... .......... .T........ .......... ..........
E24377A      .......... .......... .......... .......... .......... .......... ..........
ECOR_34      .......... .......... .......... .......... .......... .......... ..........
ECOR_71      .......... .......... .......... .......... .......... .C........ ..........
SE11         .......... .......... .......... .......... .......... .......... ..........
O157_Sakai   .......... .......... .......... .......... .......... .......... ..........
ECOR_36      .......... .......... .......... .......... .T........ .C........ ..........
ECOR_35      .......... .......... .......... .......... .T........ .C........ ..........
SMS-3-5      .......... .......... .......... .......... .T........ .C........ ..........
536          .......... .......... .......... ........T. .T........ .C........ ..........
APEC_O1      .......... .......... .......... ........T. .T........ .......... ..........
CFT073       .......... .......... .......... ........T. .T........ .C........ ..........
E2348/69     .......... .......... ...A...... ........T. .T........ .C........ ..........
ECOR_51      .......... .......... .......... ........T. .T........ .C........ ..........
ECOR_57      .......... .......... .......... ........T. .T........ .C........ ..........
UTI89        .......... .......... .......... ........T. .T........ .C........ ..........
E._albertii  .....C..T. .......... ...A...C.G ..G...T.G. .T........ .......... .....TT...

          8541
K-12         TTCCGTTGAT CCTCGCCGCG ATGGTTGTGC ATTTAAGCGG GCCGTTAATC CTGGAGCAAG GGTTATGGTT
53638        .......... .......... .......... .......... .......... .......... ..........
ATCC_8739    .......... .......... .......... .......... .......... .......... ..........
ECOR_10      .......... .......... .......... .......... .......... .......... ..........
ECOR_11      .......... .......... .......... .......... .......... .......... ..........
B171         .......... .......... .......... .......... .......... .......... ..........
E24377A      .......... .......... .......... .......... .......... .......... ..........
ECOR_34      .......... .......... .......... .......... .......... .......... ..........
ECOR_71      .......... .......... .......... .......... .......... .......... ..........
SE11         .......... .......... .......... .......... .......... .......... ..........
O157_Sakai   .......... .......... .......... .......... .......... .....T.... ..........
ECOR_36      .......... .........T .......... .......... .......... .....AG.G. .A........
ECOR_35      .......... .........T .......... .......... .......... .....AG.G. .A........
SMS-3-5      .......... .........T .......... .......... .......... .....AG.G. .A........
536          .......... T......... .......... .......... ......T..T .....A.... ..........
APEC_O1      .......... T......... .......... .......... ......T..T .....A.... ..........
CFT073       .......... T......... .......... .......... ......T..T .....A.... ..........
E2348/69     .......... T......... .......... .......... ......T..T .....A.... ..G.......
ECOR_51      .......... T......... .......... .......... ......T..T .....A.... ..........
ECOR_57      .......... T......... .......... .......... ......T..T .....A.... ..........
UTI89        .......... T......... .......... .......... ......T..T .....A.... ..........
E._albertii  .C..A..... ...T...... ...A...... .......... A...A.C... G....A..GT ..C.T...C.
```

```
           8611
K-12          TCTTGCCGAC CGGTCTTTGG CTTTACTTTT CTGGGGGTTA AAGAGTTTGC CGGAAGGGTG GATCAACATT
53638         .......... .......... .......... .......... .......... .......... ..........
ATCC_8739     .......... .......... .......... .......... .......... .......... ..........
ECOR_10       .......... .......... .......... .......... .......... .......... ..........
ECOR_11       .......... .......... .......... .......... .......... .......... ..........
B171          .......... .......... .......... .......... .......... .......... ..........
E24377A       .......... .......... .......... .......... .......... .A........ ..........
ECOR_34       .......... .......... .......... .......... .......... .A........ ..........
ECOR_71       .......... .......... .......... .......... .......... .......... ..........
SE11          .......... .......... .......... .......... .......... .......... ..........
O157_Sakai    .......... .......... .......... ..T....... .......... .A........ ..........
ECOR_36       .......... .......... .......... .......... .......... .......... ..........
ECOR_35       .......... .......... .......... .......... .......... .......... ..........
SMS-3-5       .......... .......... .......... .......... .......... .......... ..........
536           .......... .......... .......... .......... .......... .......... ..........
APEC_O1       .......... .......... .......... .......... .......... .......... ..........
CFT073        .......... .......... .......... .......... .......... .......... ..........
E2348/69      .......... .......... .......... .......... .......... .......... ..........
ECOR_51       .......... .......... .......... .......... .......... .......... ..........
ECOR_57       .......... .......... .......... .......... .......... .......... ..........
UTI89         .......... .......... .......... .......... .......... .......... ..........
E._albertii   .A....T... ..T..G..A. .C.....C.. T....C.... ..A.A...A. .C.....A.. ......T...

           8681
K-12          GCTGAATGTT GGCAATGGCT ATCATTTTCC CCATGGTTCC AACAACTGAT TATCCCCTGG CTCCACTGGC
53638         .......... .......... .......... .......... .......... .......... ..........
ATCC_8739     .......... .......... .......... .......... .......... .......... ..........
ECOR_10       .......... .......... .......... .......... .......... .......... ..........
ECOR_11       .......... .......... .......... .......... .......... .......... ..........
B171          ......C... .......... .......... .......... .......... .......... ..........
E24377A       ......C... .......... .......... .......... .......... .......... ..........
ECOR_34       ......C... .......... .......... .......... .......... .......... ..........
ECOR_71       .......... .......... .......... .......... .......... .......... ..........
SE11          .......... .......... .......... .......... .......... .......... ..........
O157_Sakai    ......C... .......... .......... .......... .......... .......... ..........
ECOR_36       ......C... .......... .......... .......... .......... .......... ..........
ECOR_35       ......C... .......... .......... .......... .......... .......... ..........
SMS-3-5       ......C... .......... .......... .......... .......... .......... ..........
536           ......C... .......... .A........ .......... .......... .......... ..........
APEC_O1       ......C... .......... .A........ .......... .......... .......... ..........
CFT073        ......C... .......... .A........ .......... .......... .......... ..........
E2348/69      ......C... .......... .A........ .......... .......... .......... ..........
ECOR_51       ......C... .......... .A........ .......... .......... .......... ..........
ECOR_57       ......C... .......... .A........ .......... .......... .......... ..........
UTI89         ......C... .......... .A........ .......... .......... .......... ..........
E._albertii   ......C.C. .......... G........T ..G...---. .......... .......... ..G.GT....

           8751
K-12          ATAATCTTGA ACCGGAAGGC GTTATTCTGA GCCATGAACA TCTGGATCAC CGGGGAGGGC TGGATTCAAT
53638         .......... .......... .......... .......... .......... .......... ..........
ATCC_8739     .......... .......... .......... .......... .......... .......... ..........
ECOR_10       .......... .......... .......... .......... .......... .......... ..........
ECOR_11       .......... .......... .......... .......... .......... .......... ..........
B171          .......... .......... .......... .T........ .......... .......... ....C.....
E24377A       .......... .......... .......... .T........ .......... .......... ....C.....
ECOR_34       .......... .......... .......... .T........ .......... .......... ....C.....
ECOR_71       .......... .......... .......... .T........ .......... .......... ....C.....
SE11          .......... .......... .......... .T........ .......... .......... ....C.....
O157_Sakai    .......... .......... .....C.... .T........ .......... .......... ....C.....
ECOR_36       .......... .......... .......... .T........ .......... .......... ....C.....
ECOR_35       .......... .......... .......... .T........ .......... .......... ....C.....
SMS-3-5       .......... .......... .......... .T........ .......... .......... ....C.....
536           .......... .......... ......T... .T........ .......... .......... ....C.....
APEC_O1       .......... .......... ......T... .T........ .......... .......... ....C.....
CFT073        .......... .......... ......T... .T........ .......... .......... ....C.....
E2348/69      .......... .......... ......T... .T........ .......... .......... ....C.....
ECOR_51       .......... .......... ......T... .T........ .......... .......... ....C.....
ECOR_57       .......... .......... ......T... .T........ .......... .......... ....C.....
UTI89         .......... .......... ......T... .T........ .......... .......... ....C.....
E._albertii   .....A.... .......... A.C.....T. ....C..G.. .T.......T A......... ....C.....
```

```
           8821
K-12         ATTGCATATA TGGCCGATGT TATGGATCAG AAGTCCGTTA AACTGGGAAC ATCATCAGCC CTGTGTGCGT
53638        .......... .......... .......... .......... .......... .......... ..........
ATCC_8739    .......... .......... .......... .......... .......... .......... ..........
ECOR_10      .......... .......... .......... .......... .......... .......... ..........
ECOR_11      .......... .......... .......... .......... .......... .......... ..........
B171         ........C. .......... .......... .......... .......... .......... ..........
E24377A      ......C.C. .......... .......... .......... .......... .......... ..........
ECOR_34      ......C.C. .......... .......... .......... .......... .......... ..........
ECOR_71      ......C.C. .......... .......... .......... .......... .......... ..........
SE11         ......C.C. .......... .......... .......... .......... .......... ..........
O157_Sakai   ........C. .......... .......... .......... .......... .......... ..........
ECOR_36      ........C. .......... .......... .......... .......... .C........ ..........
ECOR_35      ........C. .......... .......... .......... .......... .C........ ..........
SMS-3-5      ........C. .......... .......... .......... .......... .C........ ..........
536          ......C.C. .......... ........C. .......... .......... .......... ..........
APEC_O1      ......C.C. .......... ........C. .......... .......... .......... ..........
CFT073       ......C.C. .......... ........C. .......... .......... .......... ..........
E2348/69     ......C.C. .......... ........C. .......... .......... .......... ....A.....
ECOR_51      ......C.C. .......... ........C. .......... .......... .......... ..........
ECOR_57      ......C.C. .......... ........C. .......... .......... .......... ..........
UTI89        ......C.C. .......... ........C. .......... .......... .......... ..........
E._albertii  ........CC .......C.. .....G.... ...C..A... ........G. ....C..... .....CA...

           8891
K-12         GGCGAAGCGT GGCAATGGCA AGGATTGCGT TTCAGCGCGC ACTGGCCTTT ACAAGGTAGC AACGATAAAG
53638        .......... .......... .......... .......... .......... .......... ..........
ATCC_8739    .......... .......... .......... .......... .......... .......... ..........
ECOR_10      .......... .......... .......... .......... .......... .......... ..........
ECOR_11      .......... .......... .......... .......... .......... .......... ..........
B171         .......... .......... .......... .......T.. .......... .....C.... ..........
E24377A      .......... .......... .......... .......T.. .......... .....C.... ..........
ECOR_34      .......... .......... .......... .......T.. .......... .....C.... ..........
ECOR_71      .......... .......... .......... .......T.. .......... .....C.... ..........
SE11         .......... .......... .......... .......T.. .......... .....C.... ..........
O157_Sakai   .......... .......... .......... .......... .......... .......C.. ..........
ECOR_36      .......... .......... .......... .......T.. .......... .....C.... ..........
ECOR_35      .......... .......... .......... .......T.. .......... .....C.... ..........
SMS-3-5      .......... .......... .......... .......T.. .......... .....C.... ..........
536          .......... .......... .......... .......... .......... .......... ..T.......
APEC_O1      .......... .......... .......... .......... .......... .......... ..........
CFT073       .......... .......... .......... .......... .......... .......... ..........
E2348/69     .......... .......... .......... .......... .......... ......A... ..........
ECOR_51      .......... .......... .......... .......... .......... .......... ..........
ECOR_57      .......... .......... .......... .......... .......... .......... ..........
UTI89        .......... .......... .......... .......... .......... .......... ..........
E._albertii  ..T....... .......... .........C ..T..T..T. .T.....C.. G.CT.....T G.T..C....

           8961
K-12         GAAATAACCA TTCCTGTGTG GTTAAGGTTG ATGACGGGAC GAATAGCATT CTTCTAACCG GTGATATTGA
53638        .......... .......... .......... .......... .......... .......... ..........
ATCC_8739    .......... .......... .......... .......... .......... .......... ..........
ECOR_10      .......... .......... .......... .......... .......... .......... ..........
ECOR_11      .......... .......... .......... .......... .......... .......... ..........
B171         .......... .......... .......... .........T .......... .......... ..........
E24377A      .......... .......... .......... .......... .......... .......... ..........
ECOR_34      .......... .......... .......... .......... .......... .......... ..........
ECOR_71      .......... .......... .......... .......... .......... .......... ..........
SE11         .......... .......... .......... .......... .......... .......... ..........
O157_Sakai   .......... .......... .......... .......... .......... .......... ..........
ECOR_36      .......... .......... .......... .......... .......... .......... ..........
ECOR_35      .......... .......... .......... .......... .......... .......... ..........
SMS-3-5      .......... .......... .......... .......... .......... .......... ..........
536          .......... .......... ......A... .......... .......... .......... ..........
APEC_O1      .......... ....-----. ......A... .......... .......... .......... ..........
CFT073       .......... .......... ......A... .......... .......... .......... ..........
E2348/69     .......... .......... ......A... .......... .......... .......... ..........
ECOR_51      .......... .......... ......A... .......... .......... .......... ..........
ECOR_57      .......... .......... ......A... .......... .......... .......... ..........
UTI89        .......... .......... ......A... .......... .......... .......... ..........
E._albertii  .......... ......C... ......A... ....T..A.. AT........ ...T.G..T. .C........
```

```
            9031
K-12        AGCCCCAGCT GAACAAAAGA TGCTAAGCCG TTACTGGCAG CAAGTGCAGG CAACATTGCT TCAGGTACCT
53638       .......... .......... .......... .......... .......... .......... ..........
ATCC_8739   .......... .......... .......... .......... .......... .......... ..........
ECOR_10     .......... .......... .......... .......... .......... .......... ..........
ECOR_11     .......... .......... .......... .......... .......... .......... ..........
B171        ..T...C... .......... .......... .......... .......... .......... ..........
E24377A     ..T...C... .......... .......... .......... .........A .......... ..........
ECOR_34     ..T...C... .......... .......... .......... .........A .......... ..........
ECOR_71     ..T...C... .......... .......... .......... .........A .......... ..........
SE11        ..T...C... .......... .......... .......... .........A .......... ..........
O157_Sakai  .......... .......... .......... .......... .......... .......... ..........
ECOR_36     ......C... .......... .......... .......... .......... .......... ..........
ECOR_35     ......C... .......... .......... .......... .......... .......... ..........
SMS-3-5     ......C... .......... .......... .......... .......... .......... ..........
536         ......C... .......... .......... .......... ...A...... .......A.. ..........
APEC_O1     ......C... .......... .......... .......... ...A...... .......A.. ..........
CFT073      ......C... .......... .......... .......... ...A...... .......A.. ..........
E2348/69    ......C... .......... .......... .......... ...A...... .......A.. ..........
ECOR_51     ......C... .......... .......... .......... ...A...... .......A.. ..........
ECOR_57     ......C... .......... .......... .......... ...A...... .......A.. ..........
UTI89       ......C... .......... .......... .......... ...A...... .......A.. ..........
E._albertii ...AT.G... ........A. .......T.. .......AC. ..GT...... .......A.. C.....G...

            9101
K-12        CACCATGGCA GTAATACCTC ATCATCGTTG CCATTAATTC AGCGAGTGAA TGGAAAAGTG GCACTCGCAT
53638       .......... .......... .......... .......... .......... .......... ..........
ATCC_8739   .......... .......... .......... .......... .......... .......... ..........
ECOR_10     .......... .......... .......... .......... .......... .......... ..........
ECOR_11     .......... .......... .......... .......... .......... .......... ..........
B171        .......... .......... .......... .......... .......... .......... ..........
E24377A     .......... .......... .......... .......... .......... .......... ..........
ECOR_34     .......... .......... .......... .......... .......... .......... ..........
ECOR_71     .......... .......... .......... .......... .......... .......... ..........
SE11        .......... .......... .......... .......... .......... .......... ..........
O157_Sakai  .......... .......... ......A... .......... .......... .......... ..........
ECOR_36     .......... .......... .......... .......... .......... .......... ..........
ECOR_35     .......... .......... .......... .......... .......... .......... ..........
SMS-3-5     .......... .......... .......... .......... .......... .......... ..........
536         .......... .......... .......... .......... .......... .......... ..........
APEC_O1     .......... .......... .......... ......-... .......... .......... ..........
CFT073      .......... .......... .......... .......... .......... .......... ..........
E2348/69    .......... .......... .......... .......... .......... .......... ..........
ECOR_51     .......... .......... .......... .......... .......... .......... ..........
ECOR_57     .......... .......... .......... .......... .......... .......... ..........
UTI89       .......... .......... .......... .......... .......... .......... ..........
E._albertii ..T..C.... .......G.. ......A... .....C.... .A........ ...C..G... ..G..G..G.

            9171
K-12        CGGCATCGCG CTATAACGCA TGGCGACTGC CCTCTAACAA AGTTAAGCAT CGCTATCAAC TGCAAGGATA
53638       .......... .......... .......... .......... .......... .......... ..........
ATCC_8739   .......... .......... .......... .......... .......... .......... ..........
ECOR_10     .......... .......... .......... .......... .......... .......... ..........
ECOR_11     .......... .......... .......... .......... .......... .......... ..........
B171        .......... .......... .......... .......... .......... .......... AA..G..T..
E24377A     .......... .......... ......T... .......T.. .......... .......... A.........
ECOR_34     .......... .......... ......T... .......T.. .......... .......... A.........
ECOR_71     .......... .......... ......T... .......T.. .......... .......... A.........
SE11        .......... .......... ......T... .......T.. .......... .......... A.........
O157_Sakai  .......... .......... .......... .......... .......... .......... AA.....T..
ECOR_36     .......... .......... ......T... .......... .......... .......... A.........
ECOR_35     .......... .......... ......T... .......... .......... .......... A.........
SMS-3-5     .......... .......... .......... ....A.G... ......A... .......... AA..G..T..
536         .......... .......... .......... ....A.G... ......A... .......... AA..G..C..
APEC_O1     .......... .......... .......... ....A.G... ......A... .......... AA..G..C..
CFT073      .......... .......... .......... ....A.G... ......A... .......... AA..G..T..
E2348/69    .......... .......... .......... ....A.G... ......A... .......... AA..G..T..
ECOR_51     .......... .......... .......... ....A.G... ......A... .......... AA..G..T..
ECOR_57     .......... .......... .......... ....A.G... ......A... .......... AA..G..T..
UTI89       .......... .......... .......... ....A.G... ......A... .......... AA..G..C..
E._albertii .A.....A.. .......... ......T... .T..G..... .......... .......... AA..G..T..
```

```
             9241
K-12            TCAATGGATT GATACTCCAC ATCAAGGTCA AACAACGGTC AATTTTTCAG CGCAAGGCTG GCGGATTAGC
53638           .......... .......... .......... .......... .......... .......... ..........
ATCC_8739       .......... .......... .......... .......... .......... .......... ..........
ECOR_10         .......... .......... .......... .......... .......... .......... ..........
ECOR_11         .......... .......... .......... .......... .......... .......... ..........
B171            .ACG...C.C ........T. ....G..G.. .GT....... .......... .......... ..........
E24377A         .......C.. .......... ....G..... .GTG...... .......... .......... ..........
ECOR_34         .......C.. .......... ....G..... .GTG...... .......... .......... ..........
ECOR_71         .......C.. .......... ....G..... .GTG...... .......... .......... ..........
SE11            .......C.. .......... ....G..... .GTG...... .......... .......... ..........
O157_Sakai      .ACG...C.. ........T. ....G..G.. .GT....... G......... .......... ..........
ECOR_36         .......GG. .......... ....G..... .GTG...... .......... .......... ..........
ECOR_35         .......GG. .......... ....G..... .GTG...... .......... .......... ..........
SMS-3-5         .A.G...C.. .......... ....G..... ..T....... G......... .......... ..........
536             .A.G...C.. .......... ....G..... ..T....... .......... .......... ..........
APEC_O1         .A.G...C.. .......... ....G..... ..T....... .......... .......... ..........
CFT073          .A.G...C.. .......... ....G..... ..T....... .......... .......... ..........
E2348/69        .A.G...C.. .......... ....G..... ..T....... .......... .......... .T........
ECOR_51         .A.G...C.. .......... ....G..... ..T....... .......... .......... ..........
ECOR_57         .A.G...C.. .......... ....G..... ..T....... .......... .......... ..........
UTI89           .A.G...C.. .......... ....G..... ..T....... .......... .......... ..........
E._albertii     .......CCG .....C.... ....G..G.. ..T..A.... ........T. ...G...T.. .A.AG.A...

             9311
K-12            AGCCTCAGGG AGCAAATTTT ACCTCGTTGG TATCATCAGT GGTTTGGCGT GCCAGTGGAT AACGGGTAGA
53638           .......... .......... .......... .......... .......... .......... ..........
ATCC_8739       .......... .......... .......... .......... .......... .......... ..........
ECOR_10         .......... .......... .......... .......... .......... .......... ..........
ECOR_11         .......... .......... .......... .......... .......... .......... ..........
B171            ..T.....A. .......... .......... .......... .......... .......... ..........
E24377A         ........A. .......... .......... .......... .......... .......... ..........
ECOR_34         ........A. .......... .......... .......... .......... .......... ..........
ECOR_71         ........A. .......... .......... .......... .......... .......... ..........
SE11            ........A. .......... .......... .......... .......... .......... ..........
O157_Sakai      .......... .......... .......... .......... .......... .......... ..........
ECOR_36         ........A. .......... .......... .......... .......... .......... ..........
ECOR_35         ........A. .......... .......... .......... .......... .......... ..........
SMS-3-5         ........A. .......... .......... .......... .......... .......... ..........
536             ........A. .......... .......... .......... .......... .......... ..........
APEC_O1         ........A. .......... .......... .......... .......... .......... ..........
CFT073          ........A. .......... .......... .......... .......... .......... ..........
E2348/69        ........A. .......... .......... .......... .......... .......... ..........
ECOR_51         ........A. .......... .......... .......... .......... .......... ..........
ECOR_57         ........A. .......... .......... .......... .......... .......... ..........
UTI89           ........A. .......... .......... .......... .......... .......... ..........
E._albertii     .....A.... .A........ .......... .......... .......... .T........ ..........

             9381
K-12            ATATGCGGCT ATTTCAACAA ATGCTGGTTT TTTGAATGCA TAACGACAAA GATCTCTCTA CGTGGCAGAC
53638           .......... .......... .......... .......... .......... .......... ..........
ATCC_8739       .......... .......... .......... .......... .......... .......... ..........
ECOR_10         .......... .......... .......... .......... .......... .......... ..........
ECOR_11         .......... .......... .......... .......... .......... .......... ..........
B171            .......... .......... .......... .......... .......... .......... ..........
E24377A         .......... .......... .......... .......... .......... .......... ..........
ECOR_34         .......... .......... .......... .......... .......... .......... ..........
ECOR_71         .......... .......... .......... .......... .......... .......... ..........
SE11            .......... .......... .......... .......... .......... .......... ..........
O157_Sakai      .......... .......... .......... .......... .......... .......... ..........
ECOR_36         .......... .......... .......... .......... .......... .......... ..........
ECOR_35         .......... .......... .......... .......... .......... .......... ..........
SMS-3-5         .......... .......... .......... .......... .......... .......... ..........
536             .......... .......... .......... .......... .......... .......... ..........
APEC_O1         .......... .......... .......... .......... .......... .......... ..........
CFT073          .......... .......... .......... .......... .......... .......... ..........
E2348/69        .....T.... .......... .......... .......... .......... .......... ..........
ECOR_51         .......... .......... .......... .......... .......... .......... ..........
ECOR_57         .......... .......... .......... .......... .......... .......... ..........
UTI89           .......... .......... .......... .......... .......... .......... ..........
E._albertii     .......... .......... .......... .......... .......... .......... ..........
```

```
            9451
K-12         ATTCCGCCGA CTGTGGCCAA CCATTGCGCC TTTCAAAGCG GGTCTGATCG TGGCGGGCGT AGCGTTAATC
53638        .......... .......... .......... .......... .......... .......... ..........
ATCC_8739    .......... .......... .......... .......... .......... .......... ..........
ECOR_10      .......... .......... .......... .......... .......... .......... ..........
ECOR_11      .......... .......... .......... .......... .......... .......... ..........
B171         G......... .......... .......... ...T...... .......... .......... ..........
E24377A      G......... .......... .......... ...T...... .......... .......... ..........
ECOR_34      G......... .......... .......... ...T...... .......... .......... ..........
ECOR_71      G......... .......... .......... ...T...... .......... .......... ..........
SE11         G......... .......... .......... ...T...... .......... .......... ..........
O157_Sakai   .......... .......... .......... ...T...... .......... .......... ..........
ECOR_36      .......... .......... .......... ...T...... .......... .......... ..........
ECOR_35      G......... .......... .......... ...T...... .......... .......... ..........
SMS-3-5      G......... .......... .......... ...T...... .......... .......... ..........
536          G......... .......... .......... ...T...... .......... .......... ..........
APEC_O1      G......... .......... .......... ...T...... .......... .......... ..........
CFT073       G......... .......... .......... ...T...... .......... .......... ..........
E2348/69     G......... .......... .......... ...T...... .......... .......... ..........
ECOR_51      G......... .......... .......... ...T...... .......... .......... ..........
ECOR_57      G......... .......... .......... ...T...... .......... .......... ..........
UTI89        G......... .......... .......... ...T...... .......... .......... ..........
E._albertii  .......... .......... .......... C..T...... .......... .......... ..........

            9521
K-12         CTCAACGCAG CCAGCGATAC CTTCATGTTA TCGCTCCTTA AGCCACTTCT TGATGATGGC TTTGGTAAAA
53638        .......... .......... .......... .......... .......... .......... ..........
ATCC_8739    .......... .......... .......... .......... .......... .......... ..........
ECOR_10      .......... .......... .......... .......... .......... .......... ..........
ECOR_11      .......... .......... .......... .......... .......... .......... ..........
B171         .......... .......... .......... .......... .......... .......... ..........
E24377A      .......... .......... .......... .......... .......... .......... ..........
ECOR_34      .......... .......... .......... .......... .......... .......... ..........
ECOR_71      .......... .......... .......... .......... .......... .......... ..........
SE11         .......... .......... .......... .......... .......... .......... ..........
O157_Sakai   .......... .......... .......... .......... .......... .......... ..........
ECOR_36      .......... .......... .......... .......... .......... .......... ..........
ECOR_35      .......... .......... .......... .......... .......... .......... ..........
SMS-3-5      .......... .......... .......... .......... .......... .......... ..........
536          .......... .......... .......... .......... .......... .......... ..........
APEC_O1      .......... .......... .......... .......... .......... .......... ..........
CFT073       .......... .......... .......... .......... .......... .......... ..........
E2348/69     .......... .......... .......... .......... .......... .......... ..........
ECOR_51      .......... .......... .......... .......... .......... .......... ..........
ECOR_57      .......... .......... .......... .......... .......... .......... ..........
UTI89        .......... .......... .......... .......... .......... .......... ..........
E._albertii  .......... .......... .......... .......... .......... .......... ..........

            9591
K-12         CAGATCGCTC CGTGCTGGTG TGGATGCCGC TGGTGGTGAT CGGGCTGATG ATTTTACGTG GTATCACCAG
53638        .......... .......... .......... .......... .......... .......... ..........
ATCC_8739    .......... .......... .......... .......... .......... .......... ..........
ECOR_10      .......... .......... .......... .......... .......... .......... ..........
ECOR_11      .......... .......... .......... .......... .......... .......... ..........
B171         .......... .......... .......... .......... .......... .......... ..........
E24377A      .......... .......... .......... .......... .......... .......... ..........
ECOR_34      .......... .......... .......... .......... .......... .......... ..........
ECOR_71      .......... .......... .......... .......... .......... .......... ..........
SE11         .......... .......... .......... .......... .......... .......... ..........
O157_Sakai   .......... .......... .......... .......... .......... ....G..... ..........
ECOR_36      .......... .......... .......... .......... .......... ....G..... ..........
ECOR_35      .......... .......... .......... .......... .......... ....G..... ..........
SMS-3-5      .......... .......... .......... .......... .......... ....G..... ..........
536          .......... .......A.. .......... .......... .......... .......... ..........
APEC_O1      .......... .......A.. .......... .......... .......... .......... ..........
CFT073       .......... .......A.. .......... .......... .......... .......... ..........
E2348/69     .......... .......A.. .......... .......... .......... .......... ..........
ECOR_51      .......... .......A.. .......... .......... .......... .......... ..........
ECOR_57      .......... .......A.. .......... .......... .......... .......... ..........
UTI89        .......... .......A.. .......... .......... .......... .......... ..........
E._albertii  .......... .......A.. .......... .......T.. T..T...... .......... ..........
```

```
           9661
K-12         CTATGTCTCC AGCTACTGTA TCTCCTGGGT ATCAGGAAAG GTGGTAATGA CCATGCGTCG CCGCCTGTTT
53638        .......... .......... .......... .......... .......... .......... ..........
ATCC_8739    .......... .......... .......... .......... .......... .......... ..........
ECOR_10      .......... .......... .......... .......... .......... .......... ..........
ECOR_11      .......... .......... .......... .......... .......... .......... ..........
B171         .......... .......... .......... .......... .......... .......... ..........
E24377A      .......... .......... .......... .......... .......... .......... ..........
ECOR_34      .......... .......... .......... .......... .......... .......... ..........
ECOR_71      .......... .......... .......... .......... .......... .......... ..........
SE11         .......... .......... .......... .......... .......... .......... ..........
O157_Sakai   .......... .......... .......... .......... .......... .......... ..........
ECOR_36      .......... .......... .......... .......... .......... .......... ..........
ECOR_35      .......... .......... .......... .......... .......... .......... ..........
SMS-3-5      .......... .......... .......... .......... .......... .......... ..........
536          ....A..... .......... .......... .......... .......... .T........ ..........
APEC_O1      ....A..... .......... .......... .......... .......... .T........ ..........
CFT073       ....A..... .......... .......... .......... .......... .T........ ..........
E2348/69     ....A..... .......... .......... .......... .......... .T........ ..........
ECOR_51      ....A..... .......... .......... .......... .......... .T........ ..........
ECOR_57      ....A..... .......... .......... .......... .......... .T........ ..........
UTI89        ....A..... .......... .......... .......... .......... .T........ ..........
E._albertii  ....A..... ........C. .......... ...G..C..A .......... .......... ...T......

           9731
K-12         GGTCACATGA TGGGAATGCC AGTTTCATTC TTTGACAAAC AGTCAACGGG TACGCTGTTG TCACGTATTA
53638        .......... .......... .......... .......... .......... .......... ..........
ATCC_8739    .......... .......... .......... .......... .......A.. .......... ..........
ECOR_10      .......... .......... .......... .......... .......... .......... ..........
ECOR_11      .......... .......... .......... .......... .......... .......... ..........
B171         .A........ .......... G.....C..T .......... .......... .......... ........C.
E24377A      .......... .......... G........T .......... .......... G......... ........C.
ECOR_34      .......... .......... G........T .......... .......... G......... ........C.
ECOR_71      .......... .......... G........T .......... .......... G......... ........C.
SE11         .......... .......... G........T .......... .......... G......... ........C.
O157_Sakai   .......... .......... G......... .......... .......... .......... ........C.
ECOR_36      .......... .......... G......... .......... .......... .......... ........C.
ECOR_35      .......... .......... G......... .......... .......... .......... ........C.
SMS-3-5      .......... .......... G......... .......... .......... .......... ........C.
536          .......... .......... G......... .......... .......... .......... ........C.
APEC_O1      .......... .......... G......... .......... .......... .......... ........C.
CFT073       .......... .......... G......... .......... .......... .......... ........C.
E2348/69     .......... .......... G......... .......... .......... .......... ........C.
ECOR_51      .......... .......... G......... .......... .......... .......... ........C.
ECOR_57      .......... .......... G......... .......... .......... .......... ........C.
UTI89        .......... .......... G......... .......... .......... .......... ........C.
E._albertii  ..C..T.... .......... G..G.....T .......... .......C.. A..A...... ........C.

           9801
K-12         CCTACGATTC CGAACAGGTT GCTTCTTCTT CTTCCGGCGC ACTGATTACT GTTGTGCGTG AAGGTGCGTC
53638        .......... .......... .......... .......... .......... .......... ..........
ATCC_8739    .......... .......... .......... .......... .......... .......... ..........
ECOR_10      .......... .......... .......... .......... .......... .......... ..........
ECOR_11      .......... .......... .......... .......... .......... .......... ..........
B171         .......... .......... .......... .......... ......A..G .......... ..........
E24377A      .......... .......... .......... .......... ......A..G .......... ..........
ECOR_34      .......... .......... .......... .......... ......A..G .......... ..........
ECOR_71      .......... .......... .......... .......... ......A..G .......... ..........
SE11         .......... .......... .......... .......... ......A..G .......... ..........
O157_Sakai   .......... .......... ........C. .......... .......... .......... ..........
ECOR_36      .......... .......... ........C. .......... .......... .......... ..........
ECOR_35      .......... .......... ........C. .......... .......... .......... ..........
SMS-3-5      .......... .......... ........C. .......... .......... .......... ..........
536          .......... .........C .......... .......... .......... .......... ..........
APEC_O1      .......... .........C .......... .......... .......... .......... ..........
CFT073       .......... .........C .......... .......... .......... .......... ..........
E2348/69     .......... .........C .......... .......... .......... .......... ..........
ECOR_51      .......... .........C .......... .......... .......... .......... ..........
ECOR_57      .......... .........C .......... .......... .......... .......... ..........
UTI89        .......... .........C .......... .......... .......... .......... ..........
E._albertii  .A..T..... .......... .....G..A. ....T..T.. G........A ..G....... ....C..A..
```

```
            9871
K-12         GATCATCGGC CTGTTCATCA TGATGTTCTA TTACAGTTGG CAACTGTCGA TCATTTTGAT TGTGCTGGCA
53638        .......... .......... .......... .......... .......... .......... ..........
ATCC_8739    .......... .......... .......... .......... .......... .......... ..........
ECOR_10      .......... .......... .......... .......... .......... .......... ..........
ECOR_11      .......... .......... .......... .......... .......... .......... ..........
B171         A......... .......... .......... .......... .......... .......... ..........
E24377A      A......... .......... .......... .......... .......... .......... ..........
ECOR_34      A......... .......... .......... .......... .......... .......... ..........
ECOR_71      A......... .......... .......... .......... .......... .......... ..........
SE11         A......... .......... .......... .......... .......... .......... ..........
O157_Sakai   .......... .......... .......... .......... ...T...... .......... ..........
ECOR_36      .......... .......... .......... .......... .......... .......... ..........
ECOR_35      .......... .......... .......... .......... .......... .......... ..........
SMS-3-5      .......... .......... .......... .......... .......... .......... ..........
536          .......... .......... .......... .......... .......... .......... ..........
APEC_O1      .......... .......... .......... .......... .......... .......... ..........
CFT073       .......... .......... .......... .......... .......... .......... ..........
E2348/69     .......... .......... .......... .......... .......... .......... ..........
ECOR_51      .......... .......... .......... .......... .......... .......... ..........
ECOR_57      .......... .......... .......... .......... .......... .......... ..........
UTI89        .......... .......... .......... .......... .......... .......... ..........
E._albertii  C..T..T... ......GC.. .......... ......C... ..G.....A. .T........ .........G

            9941
K-12         CCGATTGTTT CGATTGCGAT TCGCGTTGTA TCGAAGCGTT TTCGCAACAT CAGTAAAAAC ATGCAGAACA
53638        .......... .......... .......... .......... .......... .......... ..........
ATCC_8739    .......... .......... .......... .......... .......... .......... ..........
ECOR_10      .......... .......... .......... .......... .......... .......... ..........
ECOR_11      .......... .......... .......... .......... .......... .......... ..........
B171         .......... .......... .......... .......... .......... .......... ..........
E24377A      .......... .......... ......A... .......... .......... .......... ..........
ECOR_34      .......... .......... ......A... .......... .......... .......... ..........
ECOR_71      .......... .......... ......A... .......... .......... .......... ..........
SE11         .......... .......... ......A... .......... .......... .......... ..........
O157_Sakai   .......... .......... ......A... .......... .......... .......... ..........
ECOR_36      .......... .......... .......... .......... .......... .......... ..........
ECOR_35      .......... .......... .......... .......... .......... .......... ..........
SMS-3-5      .......... .......... .......... .......... .......T.. .......... ..........
536          .......... .......... .......... .......... .......... .......... ..........
APEC_O1      .......... .......... .......... .......... .......... .......... ..........
CFT073       .......... .......... .......... .......... .......... .......... ..........
E2348/69     .......... .......... .......... .......... .......... .......... ..........
ECOR_51      .......... .......... .......... .......... .......... .......... ..........
ECOR_57      .......... .......... .......... .......... .......... .......... ..........
UTI89        .......... .......... .......... .......... .......... .......... ..........
E._albertii  ........C. .......A.. C..TA....T ..T....... .C....G... T.....G... ..........

           10011
K-12         CCATGGGGCA GGTGACCACC AGCGCAGAAC AAATGCTGAA GGGCCACAAA GAAGTATTGA TTTTCGGTGG
53638        .......... .......... .......... .......... .......... .......... ..........
ATCC_8739    .......... .......... .......... .......... .......... .......... ..........
ECOR_10      .......... .......... .......... .......... .......... .......... ..........
ECOR_11      .......... .......... .......... .......... .......... .......... ..........
B171         .......... .......... .....C.... .......A.. .......... .......... ..........
E24377A      .......... .......... .....C.... .......... .......... .......... ..........
ECOR_34      .......... .......... .....C.... .......... .......... .......... ..........
ECOR_71      .......... .......... .....C.... .......... .......... .......... ..........
SE11         .......... .......... .....C.... .......... .......... .......... ..........
O157_Sakai   .......... .......... .....T.... .......... A.....T... .......... ..........
ECOR_36      .......A.. .......... .....T.... .......... .......... .......... ..........
ECOR_35      .......A.. .......... .....T.... .......... .......... .......... ..........
SMS-3-5      .......... .......... .....C.... .......... A.....T... .......... ..........
536          .......... .......... .....C.... .......... A.....T... .......... ..........
APEC_O1      .......... .......... .....C.... .......... A.....T... .......... ..........
CFT073       .......... .......... .....C.... .......... A.....T... .......... ..........
E2348/69     .......... .......... .....C.... .......... A.....T... .......... ..........
ECOR_51      .......... .......... .....C.... .......... A.....T... .......... ..........
ECOR_57      .......... .......... .....C.... .......... A.....T... .......... ..........
UTI89        .......... .......... .....C.... .......... A.....T... .......... ..........
E._albertii  .......... ......T... ..T..G.... .G........ A......... ..G..G.... ....T.....
```

```
              10081
K-12          TCAGGAAGTG GAAACGAAAC GCTTTGATAA AGTCAGCAAC CGAATGCGTC TTCAGGGGAT GAAAATGGTT
53638         .......... .......... .......... .......... .......... .......... ..........
ATCC_8739     .......... .......... .......... .......... .......... .......... ..........
ECOR_10       .......... .......... .......... .......... .......... .......... ..........
ECOR_11       .......... .......... .......... .......... .......... .......... ..........
B171          .......... .......... ....C..... .......... .......... .......... ..........
E24377A       .......... .......... .......... .......... .......... .......... ..........
ECOR_34       .......... .......... .......... .......... .......... .......... ..........
ECOR_71       .......... .......... .......... .......... .......... .......... ..........
SE11          .......... .......... .......... .......... .......... .......... ..........
O157_Sakai    .......... .......... ....C..... .......... .......... .......... ..........
ECOR_36       .......... .......... ....C..... ......T... .......... .......... ..........
ECOR_35       .......... .......... ....C..... ......T... .......... .......... ..........
SMS-3-5       .......... .......... ....C..... .......... .......... .......... ..........
536           .......... .......... ....C..... .......... .......... .......... ..........
APEC_O1       .......... .......... ....C..... .......... .......... .......... ..........
CFT073        .......... .......... ....C..... .......... .......... .......... ..........
E2348/69      .......... .......... ....C..... ......T... .......... .......... ..........
ECOR_51       .......... .......... ....C..... .......... .......... .......... ..........
ECOR_57       .......... .......... ....C..... .......... .......... .......... ..........
UTI89         .......... .......... ....C..... .......... .......... .......... ..........
E._albertii   .........A ........G. .T........ .......... AAG....... .G........ ...G.....A

              10151
K-12          TCAGCCTCTT CCATCTCTGA TCCGATCATT CAGCTGATCG CCTCTTTGGC GCTGGCGTTT GTTCTGTATG
53638         .......... .......... .......... .......... .......... .......... ..........
ATCC_8739     .......... .......... .......... .......... .......... .......... ..........
ECOR_10       .......... .......... .......... .......... .......... .......... ..........
ECOR_11       .......... .......... .......... .......... .......... .......... ..........
B171          .......... .......... .......... .......... .......... .......... ..........
E24377A       .......... .......... .......... .......... .......... .......... ..........
ECOR_34       .......... .......... .......... .......... .......... .......... ..........
ECOR_71       .......... .......... .......... .......... .......... .......... ..........
SE11          .......... .......... .......... .......... .......... .......... ..........
O157_Sakai    .......... .......... .......... .......... .......... .......... ..........
ECOR_36       .......... .......... .......... .......... .......... .......... ..........
ECOR_35       .......... .......... .......... .......... .......... .......... ..........
SMS-3-5       .......... .......... .......... .......... .......... .......... ..........
536           .......... .......... .......... .......... .......... .......... ..........
APEC_O1       .......... .......... .......... .......... .......... .......... ..........
CFT073        .......... .T........ .......... .......... .......... .......... ..........
E2348/69      .......... .T........ .......... .......... .......... .......... ..........
ECOR_51       .......... .T........ .......... .......... .......... .......... ..........
ECOR_57       .......... .T........ .......... .......... .......... .......... ..........
UTI89         .......... .......... .......... .......... .......... .......... ..........
E._albertii   ..T..A..G. .T........ .......... .......... .......... ......C... ..........

              10221
K-12          CGGCGAGCTT CCCAAGTGTC ATGGATAGCC TGACTGCCGG TACGATTACC GTTGTTTTCT CTTCAATGAT
53638         .......... .......... .......... .......... .......... .......... ..........
ATCC_8739     .......... .......... .......... .......... .......... .......... ..........
ECOR_10       .......... .......... .......... .......... .......... .......... ..........
ECOR_11       .......... .......... .......... .......... .......... .......... ..........
B171          ....A..... .......... .......... .......... .......... .......... ..........
E24377A       .......... .......... .......... .......... .......... .......... ..........
ECOR_34       .......... .......... .......... .......... .......... .......... ..........
ECOR_71       .......... .......... .......... .......... .......... .......... ..........
SE11          .......... .......... .......... .......... .......... .......... ..........
O157_Sakai    .......... .......... .......... .......... .......... .......... ..........
ECOR_36       ....A..... .......... .......... .......... .......... .......... ..........
ECOR_35       ....A..... .......... .......... .......... .......... .......... ..........
SMS-3-5       ....A..... .......... .......... .......... .......... .......... ..........
536           ....A..... .......... .......... .......... .......... .......... ..........
APEC_O1       ....A..... .......... .......... .......... .......... .......... ..........
CFT073        ....A..... .......... .......... .......... .......... .......... ..........
E2348/69      .A..A..... .......... .......... .......... .......... .......... ..........
ECOR_51       ....A..... .......... .......... .......... .......... .......... ..........
ECOR_57       ....A..... .......... .......... .......... .......... .......... ..........
UTI89         ....A..... .......... .......... .......... .......... .......... ..........
E._albertii   .......... ......C... .......... .......... .........T ..C....... ....G.....
```

```
        10291
K-12          TGCACTGATG CGTCCGCTGA AATCGCTGAC CAACGTTAAC GCCCAGTTCC AGCGCGGTAT GGCGGCTTGT
53638         .......... .......... .......... .......... .......... .......... ..........
ATCC_8739     .......... .......... .......... .......... .......... .......... ..........
ECOR_10       .......... .......... .......... .......... .......... .......... ..........
ECOR_11       .......... .......... .......... .......... .......... .......... ..........
B171          ......T... ..C....... .......... .......... .......... .......... ..........
E24377A       .......... .......... ....A..... .......... .......... .......... ..........
ECOR_34       .......... .......... .......... .......... .......... .......... ..........
ECOR_71       .......... .......... .......... .......... .......... .......... ..........
SE11          .......... .......... ....A..... .......... .......... .......... ..........
O157_Sakai    .......... .......... .......... T......... .......... .......... ..........
ECOR_36       .......... .....A.... .......... .......... .......... .......... ..........
ECOR_35       .......... .....A.... .......... .......... .......... .......... ..........
SMS-3-5       ......T... .......... .......... .......... .......... .......... ..........
536           ......T... .......... .......... .......... .......... .......... ..........
APEC_O1       ......T... .......... .......... .......... .......... .......... ..........
CFT073        ......T... .......... .......... .......... .......... ....T..... ..........
E2348/69      ......T... .......... .......... .......... .......... ....T..... ..........
ECOR_51       ......T... .......... .......... .......... .......... ....T..... ..........
ECOR_57       ......T... .......... .......... .......... .......... ....T..... ..........
UTI89         ......T... .......... .......... .......... .......... .......... ..........
E._albertii   ...GT.A... .......... .....T.... .......... ..T....... .......... ...C......

        10361
K-12          CAGACGCTGT TTACCATTCT GGACAGTGAG CAGGAGAAAG ATGAAGGTAA GCGCGTGATC GAGCGTGTGC
53638         .......... .......... .......... .......... .......... .......... ..........
ATCC_8739     .......... .......... .......... .......... .......... .......... ..........
ECOR_10       .......... .......... .......... .......... .......... .......... ..........
ECOR_11       .......... .......... .......... .......... .......... .......... ..........
B171          .......... .......... .......... .......... .C........ .......... ..........
E24377A       .......... .......... .......... .......... .C........ .......... ..........
ECOR_34       .......... .......... .......... .......... .......... .......... ..........
ECOR_71       .......... .......... .......... .......... .......... .......... ..........
SE11          .......... .......... .......... .......... .C........ .......... ..........
O157_Sakai    .....A.... .......... .......... .......... .C........ .......... ..........
ECOR_36       .......... .......... .......... .......... .C........ .......... ..........
ECOR_35       .......... .......... .......... .......... .C........ .......... ..........
SMS-3-5       .......... .......... .......... .......... .C........ .......... ..........
536           .......... .......... .......... .......... .C........ .......... ..........
APEC_O1       .......... .......... .......... .......... .C........ .......... ..........
CFT073        .......... .......... .......... .......... .C........ .......... ..........
E2348/69      .......... .......... .......... .......... .C........ .......... ..........
ECOR_51       .......... .......... .......... .......... .C........ .......... ..........
ECOR_57       .......... .......... .......... .......... .C........ .......... ..........
UTI89         .......... .......... .......... .......... .C........ .......... ..........
E._albertii   ......T... .C........ ...T.....A ..A....... .C........ A..A.A...T ..A....C..

        10431
K-12          TCTGGTGTCG CAGAATGTCC ATCTGTTTAA CGATACGGTT GCTAACAACA TTGCTTACGC ACGGACTGAA
53638         .......... .......... .......... .......... .......... .......... ..........
ATCC_8739     .......... .......... .......... .......... .......... .......... ..........
ECOR_10       .......... .......... .......... .......... .......... .......... ..........
ECOR_11       .......... .......... .......... .......... .......... .......... ..........
B171          .......... .......... .......... .......... .......... .......... ...A......
E24377A       .......... .......... .......... .......... .......... .......... ..........
ECOR_34       .......... .......... .......... .......... .......... .......... ..........
ECOR_71       .......... .......... .......... .......... .......... .......... ..........
SE11          .......... .......... .......... .......... .......... .......... ..........
O157_Sakai    .......... .......... .......... .......... .......... .......... ...A......
ECOR_36       .......... .......... .......... .......... ..C....... .......... .A........
ECOR_35       .......... .......... .......... .......... ..C....... .......... .A........
SMS-3-5       .......... .......... .......... .......... ..C....... .......... ..........
536           .......... .......... .......... .......... ..C....... .......... GA........
APEC_O1       .......... .......... .......... .......... ..C....... .......... .A........
CFT073        .......... .......... .......... .......... ..C....... .......... .A........
E2348/69      .......... .......... .......... .......... ..C....... .......... .A........
ECOR_51       .......... .......... .......... .......... ..C....... .......... .A........
ECOR_57       .......... .......... .......... .......... ..C....... .......... .A........
UTI89         .......... .......... .......... .......... ..C....... .......... .A........
E._albertii   .--....... ........T. .......... .......... ..C....... .......T.. G..T......
```

```
         10501
K-12          CAGTACAGCC GTGAGCAAAT TGAAGAAGCG GCGCGTATGG CCTACGCCAT GGACTTCATC AATAAGATGG
53638         .......... .......... .......... .......... .......... .......... ..........
ATCC_8739     .......... .......... .......... .......... .......... .......... ..........
ECOR_10       .......... .......... .......... .......... .......... .......... ..........
ECOR_11       .......... .......... .......... .......... .......... .......... ..........
B171          .......... .......... .......... .......... .......T.. .......... ..........
E24377A       .......... .......... .......... .......... .......... .......... ..........
ECOR_34       .......... .......... .......... .......... .......... .......... ..........
ECOR_71       .......... .......... .......... .......... .......... .......... ..........
SE11          .......... .......... .......... .......... .......... .......... ..........
O157_Sakai    .......... .......... .......... .......... .......... .......... ..........
ECOR_36       .......... .......... .......... .......... .......... .......... ..........
ECOR_35       .......... .......... .......... .......... .......... .......... ..........
SMS-3-5       .......... .......... .......... .......... .......... .......... ..........
536           .......... .......... .........T .......... .......... .......... ..........
APEC_O1       .......... .......... .........T .......... .......... .......... ..........
CFT073        .......... .......... .........T .......... .......... .......... ..........
E2348/69      .......... .......... .........T .......... .......... .......... ..........
ECOR_51       .......... .......... .........T .......... .......... .......... ..........
ECOR_57       .......... .......... .........T .......... .......... .......... ..........
UTI89         .......... .......... .........T .......... .......... .......... ..........
E._albertii   .....T.... .......... .......... .......... ....T..... ......T... ..........

         10571
K-12          ATAACGGTCT CGATACAGTG ATTGGTGAAA ACGGCGTGCT GCTCTCTGGC GGTCAGCGTC AGCGTATTGC
53638         .......... .......... .......... .......... .......... .......... ..........
ATCC_8739     .......... .......... .......... .......... .......... .......... ..........
ECOR_10       .......... .......... .......... .......... .......... .......... ..........
ECOR_11       .......... .......... .......... .......... .......... .......... ..........
B171          .......... .......... .......... .......... .......... .......... ..........
E24377A       .......... .......... .......... .......... .......... .......... ..........
ECOR_34       .......... .......... .......... .......... .......... .......... ..........
ECOR_71       .......... .......... .......... .......... .......... .......... ..........
SE11          .......... .......... .......... .......... .......... .......... ..........
O157_Sakai    .......... .......... .......... .......A.. .......... .......... ..........
ECOR_36       .......... .........C .......... .......... .......... .......... ..........
ECOR_35       .......... .........C .......... .......... .......... .......... ..........
SMS-3-5       .......... .......... .......... .......... .......... .......... ..........
536           .......... .........C .......... ........T. .......... .......... ..........
APEC_O1       .......... .........C .......... ........T. .......... .......... ..........
CFT073        .......... .........C .......... .......... .......... .......... ..........
E2348/69      .......... .........C .......... .......... .......... .......... ..........
ECOR_51       .......... .........C .......... .......... .......... .......... ..........
ECOR_57       .......... .........C .......... .......... .......... .......... ..........
UTI89         .......... .........C .......... ........T. .......... .......... ..........
E._albertii   ....A..... T.....G..A .......... ....T...T. ......C..T .......... ..........

         10641
K-12          TATCGCTCGA GCCTTGTTGC GTGATAGCCC GATTCTGATT CTGGACGAAG CTACCTCGGC TCTGGATACC
53638         .......... .......... .......... .......... .......... .......... ..........
ATCC_8739     .......... .......... .......... .......... .......... .......... ..........
ECOR_10       .......... ......C... .......... .......... .....T.... .......... ..........
ECOR_11       .......... .......... .......... .......... .......... .......... ..........
B171          .......... .......... .......... .......... .......... .......... ..........
E24377A       .......... ......C... .......... .......... .....T.... .......... ..........
ECOR_34       .......... ......C... .......... .......... .....T.... .......... ..........
ECOR_71       .......... .......... .......... .......... .......... .......... ..........
SE11          .......... ......C... .......... .......... .....T.... .......... ..........
O157_Sakai    .......... .......... .......... .......... .....T.... .......... ..........
ECOR_36       .......... .......... .......... ...A...... .......... .......... GT........
ECOR_35       .......... .......... .......... ...A...... .......... .......... GT........
SMS-3-5       .......... .......... .......... .......... .......... .......... GT........
536           .......... .......... .......... ...A...... .......... .......... GT........
APEC_O1       .......... .......... .......... ...A...... .......... .......... GT........
CFT073        .......... .......... .......... ...A...... .......... .......... GT........
E2348/69      .......... .......... .......... ...A...... .......... .......... GT........
ECOR_51       .......... .......... .......... ...A...... .......... .......... GT........
ECOR_57       .......... .......... .......... ...A...... .......... .......... GT........
UTI89         .......... .......... .......... ...A...... .......... .......... GT........
E._albertii   C.....C..G ..T....... ....C..... ...AT....C ..T..T.... .C..T..... G.........
```

```
         10711
K-12         GAATCCGAAC GTGCGATTCA GGCGGCACTG GATGAGTTGC AGAAAAACCG TACCTCTCTG GTGATTGCCC
53638        .......... .......... .......... .......... .......... .......... ..........
ATCC_8739    .......... .......... .......... .......... .......... .......... ..........
ECOR_10      .......... .......... .......... .......... .......... .......... ..........
ECOR_11      .......... .......... .......... .......... .......... .......... ..........
B171         .......... .......... .......... .......... .......... .......... ..........
E24377A      .......... .......... .......... .......... .......... .......... ..........
ECOR_34      .......... .......... .......... .......... .......... .......... ..........
ECOR_71      .......... .......... .......... .......... .......... .......... ..........
SE11         .......... .......... .......... .......... .......... .......... ..........
O157_Sakai   .......... .......... .......... .......... .......... .......... ..........
ECOR_36      .......... .......... .......... .......... .......... .......... ..........
ECOR_35      .......... .......... .......... .......... .......... .......... ..........
SMS-3-5      .......... .......... .......... .......... .......... .......... ..........
536          .......... .......... .......... .......... .......... .......... ..........
APEC_O1      .......... .......... .......... .......... .......... .......... ..........
CFT073       .......... .......... .......... .......... .......... .......... ..........
E2348/69     .......... .......... .......... .......... .......... .......... ..........
ECOR_51      .......... .......... .......... .......... .......... .......... ..........
ECOR_57      .......... .......... .......... .......... .......... .......... ..........
UTI89        .......... .......... .......... .......... .......... .......... ..........
E._albertii  .....T.... ....A..... ......GT.. ......C... .......... ...T..AT.A ..........

         10781
K-12         ACCGCTTGTC TACCATTGAA AAGGCAGACG AAATCGTGGT CGTCGAGGAT GGTGTCATTG TGGAACGCGG
53638        .......... .......... .......... .......... .......... .......... ..........
ATCC_8739    .......... .......... .......... .......... .......... .......... ..........
ECOR_10      .......... .......... .......... .......... .......... .......... ..........
ECOR_11      .......... .......... .......... .......... .......... .......... ..........
B171         .......... .......... .......... .......... .......... .......... ..........
E24377A      .......... .......... .......... .......... .......... .......... ..........
ECOR_34      .......... .......... .......... .......... .......... .......... ..........
ECOR_71      .......... .......... .......... .......... .......... .......... ..........
SE11         .......... .......... .......... .......... .......... .......... ..........
O157_Sakai   .......... .......... .......... .......... .......... .......... ..........
ECOR_36      .......... .......... .......... .......... .......... .......... ..........
ECOR_35      .......... .......... .......... .......... .......... .......... ..........
SMS-3-5      .......... .......... .......... .......... .......... .......... ..........
536          .......... .......... .......... .......... T......... .......... ..........
APEC_O1      .......... .......... .......... .......... T......... .......... ..........
CFT073       .......... .......... .......... .......... T......... .......... ..........
E2348/69     .......... .......... .......... .......... T......... .......... ..........
ECOR_51      .......... .......... .......... .......... T......... .......... ..........
ECOR_57      .......... .......... .......... .......... T......... .......... ..........
UTI89        .......... .......... .......... .......... T......... .......... ..........
E._albertii  .......... ......C..G ..A....... .......... ...T.....C ..G..T.... ....G..T..

         10851
K-12         TACGCATAAC GATTTGCTTG AGCACCGCGG CGTTTACGCG CAACTTCACA AAATGCAGTT TGGCCAATGA
53638        .......... .......... .......... .......... .......... .......... ..........
ATCC_8739    .......... .......... .......... .......... .......... .......... ..........
ECOR_10      .......... .......... .......... .......... .......... .......... ..........
ECOR_11      .......... .......... .......... .......... .......... .......... ..........
B171         .......... .....A.... .......T.. .......... .......... .......... ..........
E24377A      .......... .......... .......... .......... .......... .......... ..........
ECOR_34      .......... .......... .......... .......... .......... .......... ..........
ECOR_71      .......... .......... .......... .......... .......... .......... ..........
SE11         .......... .......... .......... .......... .......... .......... ..........
O157_Sakai   .......... .......... .......... .......... .......... .......... ..........
ECOR_36      .......... .......... .......T.. .......... .......... .......... ..........
ECOR_35      .......... .......... .......T.. .......... .......... .......... ..........
SMS-3-5      .......... .......... .......T.. .......... .......... .......... ..........
536          .......... .......... .......T.. .......... .......... .......... ..........
APEC_O1      .......... .......... .......T.. .......... .......... .......... ..........
CFT073       .......... .......... .......T.. .......... .......... .......... ..........
E2348/69     .......... .......... ...G...T.. .......... .......... .......... ..........
ECOR_51      .......... .......... .......T.. .......... .......... .......... ..........
ECOR_57      .......... .......... .......T.. .......... .......... .......... ..........
UTI89        .......... .......... .......T.. .......... .......... .......... ..........
E._albertii  ......C... ........G. .......T.. ...C...... .......... .......... ..........
```

```
        10921
K-12          TCGAAAAAAT CTGGTCTGGT GAATCCCCTT TGTGGCGGCT ATTGCTGCCA CTCTCCTGGT TGTATGGCCT
53638         .......... .......... .......... .......... .......... .......... ..........
ATCC_8739     .......... .......... .......... .......... .......... .......... ..........
ECOR_10       .......... .......... .......... .......... .......... .......... ..........
ECOR_11       .......... .......... .......... .......... .......... .......... ..........
B171          .......... .......... .......... .......... .......... .......... ..........
E24377A       .......... .......... .......... .......... .......... .......... ..........
ECOR_34       .......... .......... .......... .......... .......... .......... ..........
ECOR_71       .......... .......... .......... .......... .......... .......... ..........
SE11          .......... .......... .......... .......... .......... .......... ..........
O157_Sakai    .......... .......... .......... .......... .......... .......... ..........
ECOR_36       .......... .......... .......... .......... .......... .......... ..........
ECOR_35       .......... .......... .......... .......... .......... .......... ..........
SMS-3-5       .......... .......... .......... .......... .......... .......... ..........
536           .......... .......... .......... .A........ .......... .......... ..........
APEC_O1       .......... .......... .......... .A........ .......... .......... ..........
CFT073        .......... .......... .......... .A...T.... .......... .......... ..........
E2348/69      .......... .......... .......... .A........ .......... .......... ..........
ECOR_51       .......... .......... .......... .A...T.... .......... .......... ..........
ECOR_57       .......... .......... .......... .A...T.... .......... .......... ..........
UTI89         .......... .......... .......... .A........ .......... .......... ..........
E._albertii   .......... .......... .....T...C .......... G......... .........C ..........

        10991
K-12          GGTGAGTGGC GCGATCCGTC TTTGCTATAA ACTAAAACTG AAGCGCGCCT GGCGTGCCCC CGTACCGGTT
53638         .......... .......... .......... .......... .......... .......... ..........
ATCC_8739     .......... .......... .......... .......... .......... .......... ..........
ECOR_10       ...A...... .......... .......... .......... .......... .......... ..........
ECOR_11       .......... .......... .......... .......... .......... .......... ..........
B171          ...A...... .......... .......... .......... .......... .......... ..........
E24377A       .......... .......... .......... .......... .......... .......... ..........
ECOR_34       ...A...... .......... .......... .......... .......... .......... ..........
ECOR_71       .......... .......... .......... .......... .......... .......... ..........
SE11          ...A...... .......... .......... .......... .......... .......... ..........
O157_Sakai    .......... .......... .......... .......... .......... .......... ..........
ECOR_36       .......... .......... .......... .......... .......... .......... ..........
ECOR_35       .......... .......... .......... .......... .......... .......... ..........
SMS-3-5       .......... .......... .......... .......... .......... .......... ..........
536           .......... .......... .......... .......... .......... .......... .A........
APEC_O1       .......... .......... .......... .......... .......... .......... ..........
CFT073        .......... .......... .......... .......... .......... .......... ..........
E2348/69      .......... .......... .......... .......... .......... .......... ..........
ECOR_51       .......... .......... .......... .......... .......... .......... ..........
ECOR_57       .......... .......... .......... .......... .......... .......... ..........
UTI89         .......... .......... .......... .......... .......... .......... ..........
E._albertii   .......... ..T.....C. .......... .T........ ..A....... ....C..T.. G..G..T..G

        11061
K-12          GTCGTGGTTG GTAATCTCAC CGCAGGCGGC AACGGAAAAA CCCCGGTCGT TGTCTGGCTG GTGGAACAGT
53638         .......... .......... .......... .......... .......... .......... ..........
ATCC_8739     .......... .......... .......... .......... .......... .......... ..........
ECOR_10       .......... .......... .......... .......... .......... .......... ..........
ECOR_11       .......... .......... .......... .......... .......... .......... ..........
B171          .......... .......... .......... .......... .......... .......... ..........
E24377A       .......... .......... .......... .......... .......... .......... ..........
ECOR_34       .......... .......... .......... .......... .......... .......... ..........
ECOR_71       .......... .......... .......... .......... .......... .......... ..........
SE11          .......... .......... .......... .......... .......... .......... ..........
O157_Sakai    .......... .......... .......... .......... .......... .......... ..........
ECOR_36       .......... .......... .......... .......... .......... .......... ..........
ECOR_35       .......... .......... .......... .......... .......... .......... ..........
SMS-3-5       .......... .......... .......... .......... .......... .......... ..........
536           .......... .......... .......... .......... .......... .......... .........C
APEC_O1       .......... .......... .......... .......... .......... .......... .........C
CFT073        .......... .......... .......... .......... .......... .......... .........C
E2348/69      .......... .......... .......... .......... .......... .......... .........C
ECOR_51       .......... .......... .......... .......... .......... .......... .........C
ECOR_57       .......... .......... .......... .......... .......... .......... .........C
UTI89         .......... .......... .......... .......... .......... .......... .........C
E._albertii   .......... .A..C..G.. T......... .......... .G.....T.. .A........ ........AC
```

```
        11131
K-12          TGCAACAGCG CGGTATTCGC GTGGGGGTC- GTATCGCGGG GATATGGTGG TAAGGCTGAA TCTTATCCGC
53638         .......... .......... .......... .......... .......... .......... ..........
ATCC_8739     .......... .......... .......... .......... .......... .......... ..........
ECOR_10       .......... .......... .......... .......... .......... .......... ..........
ECOR_11       .......... .......... .......... .......... .......... .......... ..........
B171          .......... .......... .......... .......... .....A.... .......... ..........
E24377A       .......... ...A.....A .......... .......... .......... .......... ..........
ECOR_34       .......... .......... .......... .......... .......... .......... ..........
ECOR_71       .......... .......... .......... .......... .......... .......... ..........
SE11          .......... .......... .......... .......... .......... .......... ..........
O157_Sakai    .......... .......... .......... .......... .......... .......... ..........
ECOR_36       .......... .......... .......... .......... .......... .......... ..........
ECOR_35       .......... .......... .......... .......... .......... .......... ..........
SMS-3-5       .......... .......T.. .......... .......... .......... .......... ..........
536           .......... .......... ........T. .......... .......... .......... ..........
APEC_O1       .......... .......... ........T. .......... .......... .......... ..........
CFT073        .......... .......... .........A .......... .......... ....N..... ..........
E2348/69      .......... .......... ........T. .......... .......... .......... ..........
ECOR_51       .......... .......... .......... .......... .......... .......... ..........
ECOR_57       .......... .......... .......... .......... .......... .......... ..........
UTI89         .......... .......... ........T. .......... .......... .......... ..........
E._albertii   .......A.. T.......AT ..T..T.... ..G..C..C. .T........ ...A.....G .G.......T

        11201
K-12          TGTTATTGTC GGCAGATACC ACAACAGCAC AGGCGGGTGA TGAACCTGTG TTGATTTATC AACGCACTGA
53638         .......... .......... .......... .......... .......... .......... ..........
ATCC_8739     .......... .......... .......... .......... .......... .......... ..........
ECOR_10       .......... .......... .......... .......... .......... .......... ..........
ECOR_11       .......... .......... .......... .......... .......... .......... ..........
B171          .......... .......... .......... .......... .......... .......... ..........
E24377A       .......... .......... .......... .......... .......... .......... ..........
ECOR_34       .......... .......... .......... .......... .......... .......... ..........
ECOR_71       .......... .......... .......... .......... .......... .......... ..........
SE11          .......... .......... .......... .......... .......... .......... ..........
O157_Sakai    .......... .......... ..C....... .......... .......... .......... ..........
ECOR_36       .......... .......... ..C....... .......... .......... .......... ..........
ECOR_35       .......... .......... ..C....... .......... .......... .......... ..........
SMS-3-5       .......... .......... ..C....... .......... .......... .......... ..........
536           .......... .......... ..C....... .......... .......... .......... .........G
APEC_O1       .......... .......... ..C....... .......... .......... .......... .........G
CFT073        .......... .......... ..C....... .......... .......... ......N... .........G
E2348/69      .......... .......... ..C....... .......... .......... .......... .........G
ECOR_51       .......... .......... ..C....... .......... .......... .......... .........G
ECOR_57       .......... .......... ..C....... .......... .......... .......... .........G
UTI89         .......... .......... ..C....... .......... .......... .......... .........G
E._albertii   ....GC.... .........T ..C..CA... ....A..C.. .........A C......... ..G..T...G

        11271
K-12          TGCGCCTGTT GCGGTTTCTC CCGTTCGTTC TGATGCGGTA AAAGCCATTC TGGCGCAACA CCCTGATGTG
53638         .......... .......... .......... .......... .......... .......... ..........
ATCC_8739     .......... .......... .......... .......... .......... .......... ..........
ECOR_10       .......... .......... .......... .......... .......... .......... ..........
ECOR_11       .......... .......... .......... .......... .......... .......... ..........
B171          .......... .......... .......... .......... .......... .......... ..........
E24377A       .......... .......... .......... .......... .......... .......... ..........
ECOR_34       .......... .......... .......... .......... .......... .......... ..........
ECOR_71       .......... .......... .......... .......... .......... .......... ..........
SE11          ......C... .......... .......... .......... .......... .......... ..........
O157_Sakai    .......... .......... .......... .......... .......... .......... ..........
ECOR_36       .......... .......... ....G..... .......A.. .......... .......... ..........
ECOR_35       .......... .......... ....G..... .......A.. .......... .......... ..........
SMS-3-5       .......... .......... ....G..... .......A.. .......... .......... ..........
536           C..A...... .......... ....G..... ...C...... .......... .......... ..........
APEC_O1       C..A...... .......... ....G..... ...C...... .......... .......... ..........
CFT073        C......... .......... ....G..... ...C...... .......... .......... ..........
E2348/69      C..A...... .......... ....G..... ...C...... .......... .......... ..........
ECOR_51       C......... .......... ....G..... ...C...... .......... .......... ..........
ECOR_57       C......... .......... ....G..... ...C...... .......... .......... ..........
UTI89         C..A...... .......... ....G..... ...C...... .......... .......... ..........
E._albertii   ......C... .......... .T..A..... .......... .....G.... .......... T..C......
```

```
        11341
K-12          CAGATCATCG TAACCGACGA CGGTTTACAG CAT-ACCGTC TGGCGCGTGA TGTGGAAATT GTCGTTATTG
53638         .......... .......... .......... .......... .......... .......... ..........
ATCC_8739     .......... .......... .......... .......... .......... .......... ..........
ECOR_10       .......... .......... .......... .......... .......... .......... ..........
ECOR_11       .......... .......... .......... .......... .......... .......... ..........
B171          .......... .......... .......... .......... .......... .......... ..........
E24377A       .......... .......... .......... .......... .......... .......... ..........
ECOR_34       .......... .......... .......... .......... .......... .......... ..........
ECOR_71       .......... .......... .......... .......... .......... .......... ..........
SE11          .......... .......... .......... .......... .......... .......... ..........
O157_Sakai    .......... .......... .......... .......... .......... .......... ..........
ECOR_36       .......... .......... .......... .......... .......... .......... ..........
ECOR_35       .......... .......... .......... .......... .......... .......... ..........
SMS-3-5       .......... .......... .......... .......... .......... .......... ..........
536           .......... .......... T......... .....T.... .......... .......... ....C.....
APEC_O1       .......... .......... T......... .....T.... .......... .......... ..........
CFT073        .......... .......... T......... .....T.... .......... .......... ..........
E2348/69      .......... .......... T......... .....T.... .......... .......... ..........
ECOR_51       .......... .......... T......... .....T.... .......... .......... ..........
ECOR_57       .......... .......... T......... .....T.... .......... .......... ..........
UTI89         .......... .......... T......... .....T.... .......... .......... ..........
E._albertii   ..A..T.... .G..T..... .......... ...T.T.... .......G.. ......G... ..........

        11411
K-12          ATGGTGTGCG TCGCTTTGGC AATGGCTGGT GGTTGCCGGC GGGGCCAATG CGTGAGCGAG CGGGGCGCTT
53638         .......... .......... .......... .......... .......... .......... ..........
ATCC_8739     .......... .......... .......... .......... .......... .......... ..........
ECOR_10       .......... .......... .......... .......... .......... .......... ..........
ECOR_11       .......... .......... .......... .......... .......... .......... ..........
B171          .......... .......... .......... .......... .......... .......... ..........
E24377A       .......... .......... .......... .......... .......... .......... ..........
ECOR_34       .......... .......... .......... .......... .......... .......... ..........
ECOR_71       .......... .......... .......... .......... .......... .......... ..........
SE11          .......... .......... .......... .......... .......... .......... ..........
O157_Sakai    .......... .......... .......... .......... .......... .......... ..........
ECOR_36       .......... .......... .......... .......... .......... .......... ..........
ECOR_35       .......... .......... .......... .......... .......... .......... ..........
SMS-3-5       .......... .......... .......... .......... .......... .......... ..........
536           .......... .......... .......... .......... .......... .......... ..........
APEC_O1       .......... .......... .......... .......... .......... .......... ..........
CFT073        .......... .......... .......... .......... .......... .......... ..........
E2348/69      .......... .......... .......... .......... ....T..... .......... ..........
ECOR_51       .......... .......... .......... .......... .......... .......... ..........
ECOR_57       .......... .......... .......... .......... .......... .......... ..........
UTI89         .......... .......... .......... .......... .......... .......... ..........
E._albertii   .C..C..... .........T .....T.... .......T.. .......... .......... .CA.T.....

        11481
K-12          AAAGTCGGTT GATGCGGTAA TCGTCAACGG CGGTGTCCCT CGCAGCGGTG AAATCCCCAT GCATCTGCTG
53638         .......... .......... .......... .......... .......... .......... ..........
ATCC_8739     .......... .......... .......... .......... .......... .......... ..........
ECOR_10       .......... .......... .......... T......... .......... .......... ..........
ECOR_11       .......... .......... .......... .......... .......... .......... ..........
B171          .......... .......... .......... .......... .......... .......... ..........
E24377A       ......A... .......... .......... .........C .......... .......... ..........
ECOR_34       .......... .......... .......... T......... .......... .......... ..........
ECOR_71       .......... .......... .......... .......... .......... .......... ..........
SE11          .......... .......... .......... .......... .......... .......... ..........
O157_Sakai    ......A... .......... .......... .........C .......... .......... ..........
ECOR_36       ......C... .......... .......... T........C .......... .......... ..........
ECOR_35       ......C... .......... .......... T........C .......... .......... ..........
SMS-3-5       .......... .......... .......... T........C .......... .......... ..........
536           .......... .......... .......... .........C .......... .......... ..........
APEC_O1       .......... .......... .......... .........C .......... .......... ..........
CFT073        .......... .......... .......... .........C .......... .......... ..........
E2348/69      .......... .......... .......... .........C .......... .......... ..........
ECOR_51       .......... .......... .......... .........C .......... .......... ..........
ECOR_57       .......... .......... .......... .........C .......... .......... ..........
UTI89         .......... .......... .......... .........C .......... .......... ..........
E._albertii   ...A...... ........T. .T........ T..A..A..G .....T.... ....T..A.. .T....TA.A
```

```
         11551
K-12          CCGGGTCAGG CGGTGAATTT ACGTACCGGT ACGCGTTGTG ACGTTGCTCA GCTTGAACAT GTAGTGGCGA
53638         .......... .......... .......... .......... .......... .......... ..........
ATCC_8739     .......... .......... .......... .......... .......... .......... ..........
ECOR_10       .......... .......... .......... .......... .......... .......... ..........
ECOR_11       .......... .......... .......... .......... .......... .......... ..........
B171          .......... .......... .......... .......... .......... .......... ..........
E24377A       .......... .......... .......... .......... .T........ .......... ..G.......
ECOR_34       .......... .......... .......... .......... .......... .......... ..........
ECOR_71       .......... .......... .......... .......... .......... .......... ..........
SE11          .......... .......... .......... .......... .......... .......... ..........
O157_Sakai    .......... .......... .......... .......... .......... .......... ..G.......
ECOR_36       .......... .......... .......... .......... .......... .......... ..G.......
ECOR_35       .......... .......... .......... .......... .......... .......... ..G.......
SMS-3-5       .......... .......... .......... .......... .......... .......... ..G.......
536           .......... .......... .......... .......... .......... .......... ..G.......
APEC_O1       .......... .......... .......... .......... .......... .......... ..G.......
CFT073        .......... .......... .......... .......... .......... .......... ..G.......
E2348/69      .......... .......... .......... .......... .......... .......... ..G.......
ECOR_51       .......... .......... .......... .......... .......... .......... ..G.......
ECOR_57       .......... .......... .......... .......... .......... .......... ..G.......
UTI89         .......... .......... .......... .......... .......... .......... ..G.......
E._albertii   ..C....... ..A.A..... G......... G.T..A.... .T..C..A.. A..G...... ..G.......

         11621
K-12          TGGCGGGGAT TGGGCATCCG CCGCGCTTTT TTGCCACGCT GAAGATGTGT GGCGTACAAC CGGAAAAATG
53638         .......... .......... .......... .......... .......... .......... ..........
ATCC_8739     .......... .......... .......... .......... .......... .......... ..........
ECOR_10       .......... .......... .......... .......... .......... .......... ..........
ECOR_11       .......... .......... .......... .......... .......... .......... ..........
B171          .......... .......... .......... .......... .......... .......... ..........
E24377A       ....A..... .......... .......... .......... .........C .......... ..........
ECOR_34       .......... .......... .......... .......... .......... .......... ..........
ECOR_71       .......... .......... .......... .......... .......... .......... ..........
SE11          .......... .......... .......... .......... .......... .......... ..........
O157_Sakai    ....A..... .......... .......... .......... .........C .......... ..........
ECOR_36       ....A..... ...T...... .......... .......... .......... .......... ..........
ECOR_35       ....A..... ...T...... .......... .......... .......... .......... ..........
SMS-3-5       ....A..... ...T...... .......... .......... .......... .......... ..........
536           .T........ .......... .......... .......... .........C .......... ..........
APEC_O1       .T........ .......... .......... .......... .........C .......... ..........
CFT073        .T........ .......... .......... .......... .........C .......... ..........
E2348/69      .T........ .......... .......... .......... .........C .......... ..........
ECOR_51       .T........ .......... .......... .......... .........C .......... ..........
ECOR_57       .T........ .......... .......... .......... .........C .......... ..........
UTI89         .T........ .......... .......... .......... .........C .......... ..........
E._albertii   ....T..A.. ...T..C..A .......... .C..T..... .........C ..G..T.... ....G.....

         11691
K-12          TGTACCGCTG GCCGATCATC AGTCTTTGAA CCATGCGGAT GTCAGTGCGT TGGTAAGCGC CGGGCAAACG
53638         .......... .......... .......... .......... .......... .......... ..........
ATCC_8739     .......... .......... .......... .......... .......... .......... ..........
ECOR_10       .......... .......... .......... .......... .......... .......... ..........
ECOR_11       .......... .......... .......... .......... .......... .......... ..........
B171          .......... .......... .......... .......... .......... .......... ..........
E24377A       .......... .......... .......... .......... ....C..... ........A. ..........
ECOR_34       .......... .......... .......... .......... .......... .......... ..........
ECOR_71       .......... .......... .......... .......... .......... .......... ..........
SE11          .......... .......... .......... .......... .......... .......... ..........
O157_Sakai    .......... .......... .......... .......... ....C..... ........A. ..........
ECOR_36       .......... .......... .......... T......... ....C..... ....C..... T.........
ECOR_35       .......... .......... .......... T......... ....C..... ....C..... T.........
SMS-3-5       .......... .......... .......... T......... ....C..... ....C..... ..........
536           .......... .......... .......... T......... ....C..... .......... ...A......
APEC_O1       .......... .......... .......... T......... ....C..... .......... ...A......
CFT073        .......... .......... .......... T......... ....C..... .......... ...A......
E2348/69      .......... .......... .......... T......... ....C..... .......... ...A......
ECOR_51       .......... .......... .......... T......... ....C..... .......... ...A......
ECOR_57       .......... .......... .......... T......... ....C..... .......... ...A......
UTI89         .......... .......... .......... T......... ....C..... .......... ...A......
E._albertii   ...CG..... .......... ...TCC.TG. T.......GG ..T....... ..T....... ......G...
```

```
         11761
K-12          CTGGTAATGA CTGAAAAAGA TGCGGTGAAA TGCCGGGCCT TTGCAGAAGA AAATTGGTGG TATTTGCCTG
53638         .......... .......... .......... .......... .......... .......... ..........
ATCC_8739     .......... .......... .......... .......... .......... .......... ..........
ECOR_10       .......... .......... .......... .......... .......... .......... ..........
ECOR_11       .......... .......... .......... .......... .......... .......... ..........
B171          .......... .......... .......... .......... .......... .......... ..........
E24377A       .......... .......... .......... .......... .......... .......... ..........
ECOR_34       .......... .......... .......... .......... .......... .......... ..........
ECOR_71       .......... .......... .......... .......... .......... .......... ..........
SE11          .......... .......... .......... .......... .......... .......... ..........
O157_Sakai    .......... .......... .......... .......... .......... .......... ........C.
ECOR_36       .......... .......... .......... .......... .......... .......... ..........
ECOR_35       .......... .......... .......... .......... .......... .......... ..........
SMS-3-5       .......... .A........ .......... .......... .......... .......... ..........
536           .......... .......... .......... .......... .......... .......... ..........
APEC_O1       .......... .......... .......... .......... .......... .......... ..........
CFT073        .......... .......... .......... .......... .......... .......... ..........
E2348/69      .......... .......... .......... .......... .......... .......... ..........
ECOR_51       .......... .......... .......... .......... .......... .......... ..........
ECOR_57       .......... .......... .......... .......... .......... .......... ..........
UTI89         .......... .......... .......... .......... .......... .......... ..........
E._albertii   .....C.... .G........ .......... .....T..T. ....G..... T..C...... ........C.

         11831
K-12          TAGACGCACA GCTTTCAGGT GATGAACCAG CGAAACTGCT TACGCAACTA ACCTTGCTGG CTTCTGGCAA
53638         .......... .......... .......... .......... .......... ....C..... ..........
ATCC_8739     .......... .......... .......... .......... .......... ....C..... ..........
ECOR_10       .......... .......... .......... .......... .......... ....C..... ..........
ECOR_11       .......... .......... .......... .......... .......... .......... ..........
B171          .......... .......... .......... .......... .......... ....C..... ..........
E24377A       .......... .......... .......... .......... .......... ....C..... ..........
ECOR_34       .......... .......... .......... .......... .......... ....C..... ..........
ECOR_71       .......... .......... .......... .......... .......... ....C..... ..........
SE11          .......... .......... .......... .......... .......... ....C..... ..........
O157_Sakai    .T........ .......... .......... .......... .G........ ....C..... ..........
ECOR_36       .......... .......... .......... .......... .G........ ....C..... ..........
ECOR_35       .......... .......... .......... .......... .G........ ....C..... ..........
SMS-3-5       .......... .......... .......... .......... .G........ ....C..... ..........
536           .......... .......... .......... .......... .G........ ....C..... ........C.
APEC_O1       .......... .......... .......... .......... .G........ ....C..... ........C.
CFT073        .......... .......... .......... .......... .G........ ....C..... ........C.
E2348/69      .......... .......... .......... .......... .G........ ....C..... ........C.
ECOR_51       .......... .......... .......... .......... .G........ ....C..... ........C.
ECOR_57       .......... .......... .......... .......... .G........ ....C..... ........C.
UTI89         .......... .......... .......... .......... .G........ ....C..... ........C.
E._albertii   .T..T..G.G ....A.G..C .......... ACG....... CG.......T T.T.C..... ..........

         11901
K-12          CTAGTTACGC CGCGGCAGCG TTCGATTGAT GGAGTCATGA ATGTCGCTGC CGCACCTCTC CCTTGCTGAT
53638         .......... .......... .......... .......... .......... .......... ..........
ATCC_8739     .......... .......... .......... .......... .......... .......... ..........
ECOR_10       .......... .......... .......... .......... .......... .......... ..........
ECOR_11       .......... .......... .......... .......... .......... .......... ..........
B171          .......... .......... .......... .......... .......... .......... ..........
E24377A       .......... .......... .......... .......... .......... .......... ..........
ECOR_34       .......... .......... .......... .......... .......... .......... ..........
ECOR_71       .......... .......... .......... .......... .......... .......... ..........
SE11          .......... .......... .......... .......... .......... .......... ..........
O157_Sakai    .......... .......... .......... .......... .......... .......... ..........
ECOR_36       .......... .......... .......... .......... .......... .......... T.........
ECOR_35       .......... .......... .......... .......... .......... .......... T.........
SMS-3-5       .......... .......... .......... .......... .......... .......... T.........
536           .......... .......... .......... .......... .......... .......... ..........
APEC_O1       .......... .......... .......... .......... .......... .......... ..........
CFT073        .......... .......... .......... .......... .......... .......... ..........
E2348/69      .......... .......... .......... .......... .......... .......... ..........
ECOR_51       .......... .......... .......... .......... .......... .......... ..........
ECOR_57       .......... .......... .......... .......... .......... .......... ..........
UTI89         .......... .......... .......... .......... .......... .......... ..........
E._albertii   ...A..T... .A........ ..A....... ....C..... .......... .......T.. ....A.C...
```

```
          11971
K-12         GCGCGTAATC TTCACCTTGC CGCACAAGGC CTGTTAAACA AACCCCGCCG TCGAGCGTCG TTGGAGGATA
53638        .......... .......... .......... .......... .......... .......... ..........
ATCC_8739    .......... .......... .......... .......... .......... .......... ..........
ECOR_10      .......... .......... .......... .......... .......... ..A....... ..........
ECOR_11      .......... .......... .......... .......... .......... .......... ..........
B171         .......... .......... .......... .......... .......... ..A....... ..........
E24377A      .......... .......... .......... .......... .......... .......... ..........
ECOR_34      .......... .......... .......... .......... .......... ..A....... ..........
ECOR_71      .......... .......... .......... .......... .......... .......... ..........
SE11         .......... .......... .......... .......... .......... ..A....... ..........
O157_Sakai   .......... .......... ...G...... .......... .......... .......... ..........
ECOR_36      .......... .......C.. ...G...... .......... .......... .......... ..........
ECOR_35      .......... .......C.. ...G...... .......... .......... .......... ..........
SMS-3-5      .......... .......C.. ...G...... .......... .......... .......... ..........
536          .......... .......... ...G...... .......... .......... .......... ..........
APEC_O1      .......... .......... ...G...... .......... .......... .......... ..........
CFT073       .......... .......... ...G...... .......... .......... ........T. ..........
E2348/69     .......... .......... ...G...... .......... .......... ......C... ..........
ECOR_51      .......... .......... ...G...... .......... .......... .......... ..........
ECOR_57      .......... .......... ...G...... .......... .......... .......... ..........
UTI89        .......... .......... ...G...... .......... .......... .......... ..........
E._albertii  ..A....... .G........ ...G...... T......CA. ..T.....A. C..T....T. CC..G.....

          12041
K-12         TTCCGGCAAC GATCTCCCGC ATGTCCTTGC TGCAAATCGA TACCATCAAT ATTGTTGCCC GTAGTCCATA
53638        .......... .......... .......... .......... .......... .......... ..........
ATCC_8739    .......... .......... .......... .......... .......... .......... ..........
ECOR_10      .......... .......... .......... .......... .......... .......... ..........
ECOR_11      .......... .......... .......... .......... .......... .......... ..........
B171         .......... .......... .......... C......... .......... .......... ..........
E24377A      .......... .......... .......... .......... .......... .......... ..........
ECOR_34      .......... .......... .......... .......... .......... .......... ..........
ECOR_71      .......... .......... .......... .......... .......... .......... ..........
SE11         .......... .......... .......... .......... .......... .......... ..........
O157_Sakai   .......... .......... .......... .......... .......... .......... ....C.....
ECOR_36      .......... .......... .......... .......... ......T... .......... ....C.....
ECOR_35      .......... .......... .......... .......... ......T... .......... ....C.....
SMS-3-5      .......... .......... .......... .......... .......... .......... ....C.....
536          .......... .......... .......... .......... .......... .......T.T ....C.....
APEC_O1      .......... .......... .......... .......... .......... .......T.. ....C.....
CFT073       ..T....... .......... ....N..... .......... .......... .......T.. ....C.....
E2348/69     .......... .......... .......... .......... .......... .......... ..........
ECOR_51      .......... .......... .......... .......... .......... .......T.. ....C.....
ECOR_57      .......... .......... .......... .......... .......... .......T.. ....C.....
UTI89        .......... .......... .......... .......... .......... .......T.. ....C.....
E._albertii  .C.T...G.. ...T..T... .....TC..T .......... C.....A... ..C..G..G. .......C..

          12111
K-12         TCTGGTGCTT TTCAGTCGTC TGGGAAATTA TCCTGCCCAG TGGCTGGATG AGTCTCTGGC GCGTGGCGAA
53638        .......... .......... .......... .......... .......... .......... ..........
ATCC_8739    .......... .......... .......... .......... .......... .......... ..........
ECOR_10      .......... .......... .......... .......... .......... .......... ..........
ECOR_11      .......... .......... .......... .......... .......... .......... ..........
B171         .......... .......... .......... .......... .......... .......... ..........
E24377A      .......... .......... .......... .......... .......... .......... ..........
ECOR_34      .......... .......... .......... .......... .......... .......... ..........
ECOR_71      .......... .......... .......... .......... .......... .......... ..........
SE11         .......... .......... .......... .......... .......... .......... ..........
O157_Sakai   .......... .......... .......... .......... .......... .......... ..........
ECOR_36      .......... .......... .......... .......... .......... .......... ..........
ECOR_35      .......... .......... .......... .......... .......... .......... ..........
SMS-3-5      .......... .......... .......... .......... .......... .......... ..........
536          .......... .....C.... .....G.... ......G... .......... .......... ......A...
APEC_O1      .......... .....C.... .....G.... ......G... .......... .......... ......A...
CFT073       .......... .....C.... .....G.... ......G... .......... .......... ......A...
E2348/69     .......... .......... .......... .......... .......... .......... ..........
ECOR_51      .......... .....C.... .....G.... ......G... .......... .......... ......A...
ECOR_57      .......... .....C.... .....G.... ......G... .......... .......... ......A...
UTI89        .......... .....C.... .....G.... ......G... .......... .......... ......A...
E._albertii  .......... .....C.... ....GC.... ...CC.G... ...T...... ....G....T A.....T...
```

```
         12181
K-12          TTAATGGAAT ACTGGGCGCA TGAAGCCTGC TTTATGCCGC GTAGCGACTT TCGTCTTATT CGCCACCGCA
53638         .......... .......... .......... .......... .......... .......... ..........
ATCC_8739     .......... .......... .......... .......... .......... .......... ..........
ECOR_10       .......... .......... .......... .......... .......... .......... ..........
ECOR_11       .......... .......... .......... .......... .......... .......... ..........
B171          .......... .......... .......... .......... .......... .......... ..........
E24377A       .......... .......... .......... ........A. .C........ .......... ..........
ECOR_34       .......... .......... .......... .......... .......... .......... ..........
ECOR_71       .......... .......... .......... .......... .......... .......... ..........
SE11          .......... .......... .......... .......... .......... .......... ..........
O157_Sakai    .......... .......... .......... ........A. .C..T..... .......... ..........
ECOR_36       .......... .......... .......... .......... .......... .......... ..........
ECOR_35       .......... .......... .......... .......... .......... .......... ..........
SMS-3-5       .......... .......... .......... ........A. .......T.. .......... ..........
536           .......... .......... .......... .......... .......T.. .......... ..........
APEC_O1       .......... .......... .......... .......... .......T.. .......... ..........
CFT073        .......... .......... .......... .......... .......T.. .......... ..........
E2348/69      .......... .......... .......... .......... .......T.. .......... ..........
ECOR_51       .......... .......... .......... .......... .......T.. .......... ..........
ECOR_57       .......... .......... .......... .......... .......T.. .......... ..........
UTI89         .......... .......... .......... .......... .......T.. .......... ..........
E._albertii   .......... .......... .......... .......... .......T.. C.A...C... .....T..A.

         12251
K-12          TGCTGGCACC TGAAAAAATG GGCTGGAAAT ACAAAGACGC CTGGATGCAG GAACATGAGG CGGAAATTGC
53638         .......... .......... .......... .......... .......... .......... ..........
ATCC_8739     .......... .......... .......... .......... .......... .......... ..........
ECOR_10       .......... .......... .......... .......... .......... .......... ..........
ECOR_11       .......... .......... .......... .......... .......... .......... ..........
B171          .......... .......... .......... .......... .......... .......... ..........
E24377A       .......... .......... .......... .......... .......... .......... ..........
ECOR_34       .......... .......... .......... .......... .......... .......... ..........
ECOR_71       .......... .......... .......... .......... .......... .......... ..........
SE11          .......... .......... .......... .......... .......... .......... ..........
O157_Sakai    .......... .......... .......... .......... .......... .......... ..........
ECOR_36       .......... .......... .......... .......... .......... .......C.. ..........
ECOR_35       .......... .......... .......... .......... .......... .......C.. ..........
SMS-3-5       .......... .......... .......... .......... .......... .......... T.........
536           .......... .......... .......... .T....G... .......... .......C.. ..........
APEC_O1       .......... .......... .......... .T........ .......... .......C.. ..........
CFT073        .......... .......... .......... .T........ .......... .......C.. ..........
E2348/69      .......... .......... .......... .T........ .......... .......C.. ..........
ECOR_51       .......... .......... .......... .T........ .......... .......C.. ..........
ECOR_57       .......... .......... .......... .T........ .......... .......C.. ..........
UTI89         .......... .......... .......... .T........ .......... .......C.. ..........
E._albertii   ..T....... ...G...... .......... ........A. .......... .......T.. .A.....A.A

         12321
K-12          ACAGTTAATT CAGCATATTC ATGATAAGGG GCCGGTACGT TCAGCCGATT TTGAGCATCC TCGTAAAGGT
53638         .......... .......... .......... .......... .......... .......... ..........
ATCC_8739     .......... .......... .......... .......... .......... .......... ..........
ECOR_10       .......... .......... .......... .......... .......... .......... ..........
ECOR_11       .......... .......... .......... .......... .......... .......... ..........
B171          .......... .......... .......... .......... .......... .......... ..........
E24377A       .......... .......... .......... .......... .......... .......... ..........
ECOR_34       .......... .......... .......... .......... .......... .......... ..........
ECOR_71       .......... .......... .......... .......... .......... .......... ..........
SE11          .......... .......... .......... .......... .......... .......... ..........
O157_Sakai    .......... .......... .......... .......... .......... .....T.... ..........
ECOR_36       .......... .......... .......... .......... .......... .C......T. ..........
ECOR_35       .......... .......... .......... .......... .......... .C......T. ..........
SMS-3-5       .......... .......... .......... .......... .......... .......... .........G
536           ..T....... .......... .......... .......... .......... .......... .........G
APEC_O1       ..T....... .......... .......... .......... .......... .......... .........G
CFT073        ..T....... .......... .......... .......... .......... .......... .........G
E2348/69      .......... .......... .......... ...A...... .......... .......... .........G
ECOR_51       ..T....... .......... .......... .......... .......... .......... .........G
ECOR_57       ..T....... .......... .......... .......... .......... .......... .........G
UTI89         ..T....... .......... .......... .......... .......... .......... .........G
E._albertii   ....C..... .......... .G.....C.. A.....T..C .......... ....A..... G....C...A
```

```
         12391
K-12        GCAAGCGGCT GGTGGGAATG GAAGCCGCAT AAACGGCATC TGGAAGGTTT ATTTACTGCC GGAAAGGTGA
53638       .......... .......... .......... .......... .......... .......... ..........
ATCC_8739   .......... .......... .......... .......... .......... .......... ..........
ECOR_10     .......... .......... .......... .......... .......... .......... ..........
ECOR_11     .......... .......... .......... .......... .......... .......... ..........
B171        .......... .......... .......... .......... .......... .......... ..........
E24377A     .......... .......... .......... .......... .......... .......... ..........
ECOR_34     .......... .......... .......... .......... .......... .......... ..........
ECOR_71     .......... .......... .......... .......... .......... .......... ..........
SE11        .......... .......... .......... .......... .......... .......... ..........
O157_Sakai  .......... .......... .......... .......... .......... .......... ..........
ECOR_36     .......... .......... .......... .......... .......... .......... ..........
ECOR_35     .......... .......... .......... .......... .......... .......... ..........
SMS-3-5     .......... .......G.. .......... .....A.... .......... .......... ..........
536         ..G....... .......G.. .......... .....A.... .......... .......... ..........
APEC_O1     ..G....... .......G.. .......... .....A.... .......... .......... ..........
CFT073      ..G....... .......G.. .......... .....A.... .......... .......... ..........
E2348/69    ..G....... .......G.. .......... .....A.... .......... .......... ..........
ECOR_51     ..G....... .......G.. .......... .....A.... .......... .......... ..........
ECOR_57     ..G....... .......G.. .......... .....A.... .......... .......... ..........
UTI89       ..G....... .......G.. .......... .....A.... .......... .......... ..........
E._albertii ..G....... .......G.. .......... .......... .......AC. ......C..T ..G.......

         12461
K-12        TGGTGATTGA ACGGCGCAAC TTCCAGCGCG TTTATGATTT AACCCACCGT GTCATGCCTG ACTGGGATGA
53638       .......... .......... .......... .......... .......... .......... ..........
ATCC_8739   .......... .......... .......... .......... .......... .......... ..........
ECOR_10     .......... .......... .......... .......... .......... .......... ..........
ECOR_11     .......... .......... .......... .......... .......... .......... ..........
B171        .......... .......... .......... .......... .......... .......... ..........
E24377A     .......... .......... .......... .......... .......... .......... ..........
ECOR_34     .......... .......... .......... .......... .......... .......... ..........
ECOR_71     .......... .......... .......... .......... .......... .......... ..........
SE11        .......... .......... .......... .......... .......... .......... ..........
O157_Sakai  .......... .......... .......... .......... .......... .......... ..........
ECOR_36     .......... .......... .......... .......... .......... .......... ..........
ECOR_35     .......... .......... .......... .......... .......... .......... ..........
SMS-3-5     .......... .......... .......... .......... .......... .......... ..........
536         .......... .......... .......... .......... ...T...... .......... ..........
APEC_O1     .......... .......... .......... .......... ...T...... .......... ..........
CFT073      .......... .......... .......... .......... ...T...... .......... .G...A....
E2348/69    .......... .......... .......... .......... ...T...... .......... .G...A....
ECOR_51     .......... .......... .......... .......... ...T...... .......... .G...A....
ECOR_57     .......... .......... .......... .......... ...T...... .......... .G...A....
UTI89       .......... .......... .......... .......... ...T...... .......... ..........
E._albertii .....G.... .........T ........T. .C........ ......T..C ........GC .T........

         12531
K-12        TGAGCGCGAT CTCGTTTCGC AAACAGAAGC AGAAATCATC ATGCTGGATA ACAGTGCGCG TAGCCTGGGA
53638       .......... .......... .......... .......... .......... .......... ..........
ATCC_8739   .......... .......... .......... .......... .......... .......... ..........
ECOR_10     .......... .......... .......... .......... .......... .......... ..........
ECOR_11     .......... .......... .......... .......... .......... .......... ..........
B171        .......... .......... .......... .......... .......... .......... ..........
E24377A     .......... .......... .......... .......... .......... .......... ..........
ECOR_34     .......... .......... .......... .......... .......... .......... ..........
ECOR_71     .......... .......... .......... .......... .......... .......... ..........
SE11        .......... .......... .......... .......... .......... .......... ..........
O157_Sakai  .......... .......... .......... .......... .......... .......... .....A....
ECOR_36     .......... .......... .......... .......... .......... .......... .........C
ECOR_35     .......... .......... .......... .......... .......... .......... .........C
SMS-3-5     .......... .......... .......... .......... .......... .......... .........C
536         .......... .......... ..G....... .......... .......... .......... ..........
APEC_O1     .......... .......... ..G....... .......... .......... .......... ..........
CFT073      .......... .......... ..G....... .......... .......... .......... ..........
E2348/69    .......... .......... ..G....... .......... .......... .......... ..........
ECOR_51     .......... .......... ..G....... .......... .......... .......... ..........
ECOR_57     .......... .......... ..G....... .......... .......... .......... ..........
UTI89       .......... .......... ..G....... .......... .......... .......... ..........
E._albertii C..A..T... ...A...... ..G.G..... T.....TGC. .......... .T........ ...T......
```

```
          12601
K-12         ATATTCCGCG AACAGTGGCT GGCAGATTAC TATCGGCTGA AACGTCCGGC ACTGGCGGCG TGGCGCGAAG
53638        .......... .......... .......... .......... .......... .......... ..........
ATCC_8739    .......... .......... .......... .......... .......... .......... ..........
ECOR_10      .......... .......... .......... .......... .......... .......... ..........
ECOR_11      .......... .......... .......... .......... .......... .......... ..........
B171         .......... .......... .......... .......... .......... .......... ..........
E24377A      .......... .......... .......... .......... .......... .......... ..........
ECOR_34      .......... .......... .......... .......... .......... .......... ..........
ECOR_71      .......... .......... .......... .......... .......... .......... ..........
SE11         .......... .......... .......... .......... .......... .......... ..........
O157_Sakai   .......... .......... .......... .......... .......... ......A... ..........
ECOR_36      .......... .......... .......... .......... .......... .........T ..........
ECOR_35      .......... .......... .......... .......... .......... .........T ..........
SMS-3-5      .......... .......... .......... .......... .......... .........T ..........
536          .....T.... .......... .......... ..C.....A. .......... .......... .....T....
APEC_O1      .....T.... .......... .......... ..C.....A. .......... .......... .....T....
CFT073       .....T.... .......... .......... ..C.....A. .......... .......... .....T....
E2348/69     .....T.... .......... .......... ..C.....A. .......... .......... .....T....
ECOR_51      .....T.... .......... .......... ..C.....A. .......... .......... .....T....
ECOR_57      .....T.... .......... .......... ..C.....A. .......... .......... .....T....
UTI89        .....T.... .......... .......... ..C.....A. .......... .......... .....T....
E._albertii  ..C..T..A. .......... ..TC...... .....A.... .G..C..... GT...G.AAT ........TA

          12671
K-12         CGAGGGCTGA ACAGCAGCAA ATCATTGCTG TGCATGTTGA AAAATTGGGC AATCTTTGGC TGCATGATGA
53638        .......... .......... .......... .......... .......... .......... ..........
ATCC_8739    .......... .......... .......... .......... .......... .......... ..........
ECOR_10      .......... .......... .......... .......... .......... .......... ......C...
ECOR_11      .......... .......... .......... .......... .......... .......... ..........
B171         .......... .......... .......... .......... .......... .......... ......C...
E24377A      .......... .......... .......... .......... .......... .......... ......C...
ECOR_34      .......... .......... .......... .......... .......... .......... ......C...
ECOR_71      .......... .......... .......... .......... .......... .......... ......C...
SE11         .......... .......... .......... .......... .......... .......... ......C...
O157_Sakai   .......... .....G.... .......... .......... .......... .......... ......C...
ECOR_36      .......... .......... .......... .......... .......... .......... ......C...
ECOR_35      .......... .......... .......... .......... .......... .......... ......C...
SMS-3-5      .......... ..T....... .......... .......... .......... .........T ......C...
536          .......... .......... .......... .......... .........T .......... ......C...
APEC_O1      .......... .......... .......... .......... .......... .......... ......C...
CFT073       .......... .......... .......... .......... .........T .......... ......C...
E2348/69     .......... .......... .......... .......... .......... .......... ......C...
ECOR_51      .......... .......... .......... .......... .........T .......... ......C...
ECOR_57      .......... .......... .......... .......... .........T .......... ......C...
UTI89        .......... .......... .......... .......... .......... .......... ......C...
E._albertii  T.C....... .....G.... .........C .......... ...G.....A .......... ......CC..

          12741
K-12         TTTGCTGCCG CTACTCGAGC GAGCGCTGGC CGGAAAGCTC ACTGCAACGC ACAGCGCGGT ACTTTCGCCT
53638        .......... .......... .......... .......... .......... .......... ..........
ATCC_8739    .......... .......... .......... .......... .......... .......... ..........
ECOR_10      .......... .......... .T........ .......... .......... .......... ..........
ECOR_11      .......... .......... .......... .......... .......... .......... ..........
B171         .......... .......... .T........ .......... .......... .......... ..........
E24377A      .......... .......... .T........ .......... .......... .......... ..........
ECOR_34      .......... .......... .T........ .......... .......... .......... ..........
ECOR_71      .......... .......... .T........ .......... .......... .......... ..........
SE11         .......... .......... .T........ .......... .......... .......... ..........
O157_Sakai   .......... .......... .......... .......... .......... .......... ..........
ECOR_36      .......... .......... .T.....C.. .......... .......... .......... ..........
ECOR_35      .......... .......... .T.....C.. .......... .......... .......... ..........
SMS-3-5      .......... .......... .T........ .......... .......... .......... ..........
536          .......... .......... .T.....C.. .......... .........T .......... ..........
APEC_O1      .......... .......... .T.....C.. .......... .......... .......... ..........
CFT073       .......... .......... .T.....C.. .......... .......... .......... ..........
E2348/69     .......... .......... .T.....C.. .......... .......... .......... ..........
ECOR_51      .......... .......... .T.....C.. .......... .......... .......... ..........
ECOR_57      .......... .......... .T.....C.. .......... .......... .......... ..........
UTI89        .......... .......... .T.....C.. .......... .......... .......... ..........
E._albertii  ...AT..... ..G..G..A. .C.....C.. A.....T... .T........ ......T... GT.G..C...
```

```
          12811
K-12           TTTGATCCTG TTGTCTGGGA TCGCAAACGC GCAGAGCAGC TTTTTGATTT TAGCTACCGG CTGGAGTGCT
53638          .......... .......... .......... .......... .......... .......... ..........
ATCC_8739      .......... .......... .......... .......... .......... .......... ..........
ECOR_10        .......... .......... .......... .......... .......... .......... ..........
ECOR_11        .......... .......... .......... .......... .......... .......... ..........
B171           .......... .......... .......... ......T... .......... .......... ..........
E24377A        .......... .......... .......... .......... .......... .......... ..........
ECOR_34        .......... .......... .......... .......... .......... .......... ..........
ECOR_71        .......... .......... .......... .......... .......... .......... ..........
SE11           .......... .......... .......... .......... .......... .......... ..........
O157_Sakai     .......... .......... .......... .......... .......... .......... ..........
ECOR_36        .......... .......... .......... .......... .......... .......... ..........
ECOR_35        .......... .......... .......... .......... .......... .......... ..........
SMS-3-5        .......... .......... .......... .......... .......... .......... ..........
536            .......... .......... .......... ..G....... .......... .........A ..........
APEC_O1        .......... .......... .......... ..G....... .......... .......... ..........
CFT073         .......... .......... .......... ..G....... .......... .......... ..........
E2348/69       .......... .......... .......... ..G....... .......... .......... ..........
ECOR_51        .......... .......... .......... ..G....... .......... .......... ..........
ECOR_57        .......... .......... .......... ..G....... .......... .......... ..........
UTI89          .......... .......... .......... ..G....... .......... .......... ..........
E._albertii    .......... .......... .......... ........A. .C........ ......T... ..C.......

          12881
K-12           ATACCCCAGC GCCGAAACGC CAGTATGGCT ATTTTGTTCT GCCGTTATTA CATCGTGGGC AATTAGTTGG
53638          .......... .......... .......... .......... .......... .......... ..........
ATCC_8739      .......... .......... .......... .......... .......... .......... ..........
ECOR_10        .......... .......... .......... .......... ...A...... .......... ..........
ECOR_11        .......... .......... .......... .......... .......... .......... ..........
B171           .......... .......... .......... .......... ...A...... .......... ..........
E24377A        .......... .......... .......... .......... ...A...... .......... ..........
ECOR_34        .......... .......... .......... .......... ...A...... .......... ..........
ECOR_71        .......... .......... .......... .......... ...A...... .......... ..........
SE11           .......... .......... .......... .......... ...A...... .......... ..........
O157_Sakai     .......... .......... .......... .......... .......... .......... ..........
ECOR_36        .......... .......... .......... .......... .......... .......... ..........
ECOR_35        .......... .......... .......... .......... .......... .......... ..........
SMS-3-5        .......T.. .......... .......... .......... .......... .......... ..........
536            ...T...... .......... .......... .......... .......... .......... ..........
APEC_O1        ...T...... .......... ........A. .......... .......... .......... ..........
CFT073         ...T...... .......... .......... .......... .......... .......... ..........
E2348/69       ...T...... .......... .......... .......... .......... .......... ..........
ECOR_51        ...T...... .......... .......... .......... .......... .......... ..........
ECOR_57        ...T...... .......... .......... .......... .......... .......... ..........
UTI89          ...T...... .......... ........A. .......... .......... .......... ..........
E._albertii    .C..G..... ...C.....T .......... ....C..... A......... ..C....... .......C..

          12951
K-12           GCGAATGGAT GCCAAAATGC ATCGCCAGAC AGGCATCCTT GAAGTTATCT CTCTGTGGTT ACAGGAAGGT
53638          .......... .......... .......... .......... .......... .......... ..........
ATCC_8739      .......... .......... .......... .......... .......... .......... ..........
ECOR_10        .......... .......... .......... .......... .......... .......... .........C
ECOR_11        .......... .......... .......... .......... .......... .......... ..........
B171           .......... .......... .......... .......... .......... .......... .........C
E24377A        .......... .......... .......... .......... .......... .......... .........C
ECOR_34        .......... .......... .......... .......... .......... .......... .........C
ECOR_71        .......... .......... .......... .......... .......... .......... .........C
SE11           .......... .......... .......... .......... .......... .......... .........C
O157_Sakai     .......... .......... .......... .......... .......... .......... ..........
ECOR_36        .......... .......... .......... .......... .......... .......... ..........
ECOR_35        .......... .......... .......... .......... .......... .......... ..........
SMS-3-5        .......... .......... .......... .......... .......... .......... .........C
536            ..A....... .......... .......... .......... .......... .......... .........C
APEC_O1        .......... .......... .......... .......... .......... .......... .........C
CFT073         .......... .......... .......... .......... .......... .......... .........C
E2348/69       .......... .......... .......... .........G .......... .......... .........C
ECOR_51        .......... .......... .......... .......... .......... .......... .........C
ECOR_57        .......... .......... .......... .......... .......... .......... .........C
UTI89          .......... .......... .......... .......... .......... .......... .........C
E._albertii    .......... .......... ....T...G. G..TG.T... ...A...... .C..C..... ......T..C
```

```
         13021
K-12          ATTAAACCAA CGACAACGCT GCAAAAAGGG CTACGTCAGG CGATTACTGA TTTCGCTAAC TGGCAGCAGG
53638         .......... .......... .......... .......... .......... .......... ..........
ATCC_8739     .......... .......... .......... .......... .......... .......... ..........
ECOR_10       .......... ......T... .......... T......... .......... ......G.G. ..........
ECOR_11       .......... .......... .......... .......... .......... .......... ..........
B171          .......... ......T... .......... T......... .......... ......G.G. ..........
E24377A       .......... ......T... .......... T......... .......... ......G.G. ..........
ECOR_34       .......... ......T... .......... T......... .......... ......G.G. ..........
ECOR_71       .......... ......T... .......... T......... .......... ......G.G. ..........
SE11          .......... ......T... .......... T......... .......... ......G.G. ..........
O157_Sakai    .......... .......... .......... T......... .......... .......... ..........
ECOR_36       .......... .......... .......... T......... .......... .......... ..........
ECOR_35       .......... .......... .......... T......... .......... .......... ..........
SMS-3-5       .......... .......... .......... T......... .......... .......... ..........
536           .......... .......... .......... T......... .......... .......... ..........
APEC_O1       .......... .......... .......... T......... .......... .......... ..........
CFT073        ........G. .......... .......... T......... .......... .......... ..........
E2348/69      .....G.... .......... .......... T......... .......... .......... ..........
ECOR_51       ........G. .......... .......... T......... .......... .......... ..........
ECOR_57       ........G. .......... .......... T......... .......... .......... ..........
UTI89         .......... .......... .......... T......... .......... .......... ..........
E._albertii   ..C....AG. ..G.TC.T.. T........A ..G..C.... .......... ........G. ..........

         13091
K-12          CAACGCGGGT GACATTAGGA TGCTGCCCGC AAGGCCTCTT TACTGATTGC CGCACCGGCT GGGAAATAGA
53638         .......... .......... .......... .......... .......... .......... ..........
ATCC_8739     .......... .......... .......... .......... .......... .......... ..........
ECOR_10       .......... .......... C......... ....T..... .......... .....T.... ..........
ECOR_11       .......... .......... .......... .......... .......... .......... ..........
B171          .......... .......... C......... ....T..... .......... .....T.... ..........
E24377A       .......... .......... C......... ....T..... .......... .....T.... ..........
ECOR_34       .......... .......... C......... ....T..... .......... .....T.... ..........
ECOR_71       .......... .......... C......... ....T..... .......... .....T.... ..........
SE11          .......... .......... C......... ....T..... .......... .....T.... ..........
O157_Sakai    .......... .......... CA........ ....T..... ...G...... .......... ..........
ECOR_36       .......... .......... C......... .......... .......... .......... ..........
ECOR_35       .......... .......... C......... .......... .......... .......... ..........
SMS-3-5       .......... .......... C......... .......... .......... .......... ..........
536           .......... .......... C......... .......... .......... ...G...... ..........
APEC_O1       .......... .......... C......... .......... .......... ...G...... ..........
CFT073        .......... .......... C.....T... .......... .........T ...G...... ..........
E2348/69      .......... .......C.. C......... .......... .......... ...G...... ..........
ECOR_51       .......... .......... C.....T... .......... .........T ...G...... ..........
ECOR_57       .......... .......... C.....T... .......... .........T ...G...... ..........
UTI89         .......... .......... C......... .......... .......... ...G...... ..........
E._albertii   .G........ .......... C....T..A. ....T..... ...C...... ..T..A.... .........C

         13161
K-12          CCCCGTCGCA TAAATGATTA TGATAAGATT TATGCATTCA TAACCCGATC CATCTGGAGG AACTATGGAT
53638         .......... .......... .......... .......... .......... .......... ..........
ATCC_8739     .......... .......... .......... .......... .......... .......... ..........
ECOR_10       .......... .......... .......... .......... .......... .......... ..........
ECOR_11       .......... .......... .......... .......... .......... .......... ..........
B171          .......... .......... .......... .......... .......... .......... ..........
E24377A       .......... .......... .......... .......... .......... .......... ..........
ECOR_34       .......... .......... .......... .......... .......... .......... ..........
ECOR_71       .......... .......... .......... .......... .......... .......... ..........
SE11          .......... .......... .......... .......... .......... .......... ..........
O157_Sakai    .......... .......... .......... .......... .......... .......... ..........
ECOR_36       .......... .......... .......... .......... .......... .......... ..........
ECOR_35       .......... .......... .......... .......... .......... .......... ..........
SMS-3-5       .......... .......... .......... .......... .......... .......... ..........
536           .......... .......... .......... .......... .......... .......... ..........
APEC_O1       .......... .......... .......... .......... .......... .......... ..........
CFT073        .......... .......... .......... .......... .......... .......... ..........
E2348/69      .......... .......... .......... .......... .......... .......... ..........
ECOR_51       .......... .......... .......... .......... .......... .......... ..........
ECOR_57       .......... .......... .......... .......... .......... .......... ..........
UTI89         .......... .......... .......... .......... .......... .......... ..........
E._albertii   ....TA..T. .......... .......... .......... .......... ....A..... ..........
```

```
         13231
K-12          CATCGTCTGC TTGAAATCAT TGCCTGCCCG GTTTGCAACG GAAAACTTTG GTATAACCAG GAAAAACAAG
53638         .......... .......... .......... .......... .......... .......... ..........
ATCC_8739     .......... .......... .......... .......... .......... .......... ..........
ECOR_10       .......... .......... .......... .......... .......... .........A ..........
ECOR_11       .......... .......... .......... .......... .......... .......... ..........
B171          ......T... .......... .......... .......... .......... .........A ..........
E24377A       .......... .......... .......... .......... .......... .........A ..........
ECOR_34       .......... .......... .......... .......... .......... .........A ..........
ECOR_71       ......T... .......... .......... .......... .......... .......... ..........
SE11          .......... .......... .......... .......... .......... .........A ..........
O157_Sakai    .......... .......... .......... .......... .......... .......... ..........
ECOR_36       .......... .......... .......... .......... .......... .......... ..........
ECOR_35       .......... .......... .......... .......... .......... .......... ..........
SMS-3-5       .......... .......... .......... .......... .......... .......... ..........
536           .......... .......... .......... .......... .......... .......... ..........
APEC_O1       .......... .......... .......... .......... .......... .......... ..........
CFT073        .......... .......... .......... .......... .......... .......... ..........
E2348/69      .......... .......... .......... .......... .......... .......... ..........
ECOR_51       .......... .......... .......... .......... .......... .......... ..........
ECOR_57       .......... .......... .......... .......... .......... .......... ..........
UTI89         .......... .......... .......... .......... .......... .......... ..........
E._albertii   .......... .......... ......T... .......... .C........ .......... ..........

         13301
K-12          AGCTTATTTG CAAACTGGAC AACCTCGCTT TCCCGCTGCG TGATGGCATT CCAGTGTTGC TGGAAACGGA
53638         .......... .......... .......... .......... .......... .......... ..........
ATCC_8739     .......... .......... .......... .......... .......... .......... ..........
ECOR_10       .A........ .......... .....G.... .......... .......... .......... ..........
ECOR_11       .......... .......... .......... .......... .......... .......... ..........
B171          .......... .........T .....G.... .......C.. C......... .......... ..........
E24377A       .A........ .......... .....G.... .......... .......... .......... ..........
ECOR_34       .A........ .......... .....G.... .......... .......... .......... ..........
ECOR_71       .......... .........T .....G.... .......C.. C......... .......... ..........
SE11          .A........ .......... .....G.... .......... .......... .......... ..........
O157_Sakai    .A........ .......... .....G.... .......... C......... .......... ..........
ECOR_36       .A........ .......... .....G.... .......... C......... .......... ..........
ECOR_35       .A........ .......... .....G.... .......... C......... .......... ..........
SMS-3-5       .A........ .......... .....G.... .......... C......... .......... ..........
536           .......... .......... .....G.... .......... C......... .......... ..........
APEC_O1       .......... .......... .....G.... .......... C......... .......... ..........
CFT073        .......... .......... .....G.... .......... C......... .......... ..........
E2348/69      .......... .......... .....G.... .......... C......... .......... ..........
ECOR_51       .......... .......... .....G.... .......... C......... .......... ..........
ECOR_57       .......... .......... .....G.... .......... C......... .......... ..........
UTI89         .......... .......... .....G.... .......... C......... .......... ..........
E._albertii   .A..G..... .......... .....G.... .......... C......... ......C.A. ..........

         13371
K-12          AGCCCGCGTG CTGACTGCTG ATGAGAGTAA ATCATGAGTT TTGTGGTCAT TATTCCCGCG CGCTACGCGT
53638         .......... .......... .......... .......... .......... .......... ..........
ATCC_8739     .......... .......... .......... .......... .......... .......... ..........
ECOR_10       .......... .......... .......... .......... .......... .......... ..........
ECOR_11       .......... .......... .......... .......... .......... .......... ..........
B171          .......... .......... .......... .......... .......... .......... ..........
E24377A       .......... .......... .......... .......... .......... .......... ..........
ECOR_34       .......... .......... .......... .......... .......... .......... ..........
ECOR_71       .......... .......... .......... .......... .......... .......... ..........
SE11          .......... .......... .......... .......... .......... .......... ..........
O157_Sakai    .......... .......... .......... .......... .......... .......... ..........
ECOR_36       .......... .......... .......... .......... .......... .......... ..........
ECOR_35       .......... .......... .......... .......... .......... .......... ..........
SMS-3-5       .......... .......... .......... .......... .......... .......... ..........
536           .......... .......... .......... .......... .......... .......... ..T.......
APEC_O1       .......... .......... .......... .......... .......... .......... ..T.......
CFT073        .......... T......... .......... .......... .......... .......... ..T.......
E2348/69      .......... .......... .......... .......... .......... .......... ..T.......
ECOR_51       .......... T......... .......... .......... .......... .......... ..T.......
ECOR_57       .......... T......... .......... .......... .......... .......... ..T.......
UTI89         .......... .......... .......... .......... .......... .......... ..T.......
E._albertii   ......T... .......... .......... .......... ....T..... ......T..T ..T...T.T.
```

```
        13441
K-12         CCACGCGTCT GCCCGGTAAA CCATTGGTTG ATATTAACGG CAAACCCATG ATTGTTCATG TTCTTGAACG
53638        .......... .......... .......... .......... .......... .......... ..........
ATCC_8739    .......... .......... .......... .......... .......... .......... ..........
ECOR_10      .A........ .......... .......... .......T.. .......... .......... ..........
ECOR_11      .......... .......... .......... .......... .......... .......... ..........
B171         .......... .......... .......... .......... .......... .......... ..........
E24377A      .A........ .......... .......... .......T.. .......... .......... ..........
ECOR_34      .A........ .......... .......... .......T.. .......... .......... ..........
ECOR_71      .......... .......... .......... .......... .......... .......... ..........
SE11         .A........ .......... .......... .......T.. .......... .......... ..........
O157_Sakai   .......... .......... .......... .......... .......... .......... ..........
ECOR_36      .......... .......... .......... .......... .......... .......... ..........
ECOR_35      .......... .......... .......... .......... .......... .......... ..........
SMS-3-5      .......... .......... .......... .......... T......... .......... ..........
536          .......... .......... .......... .......... .......... .......... ..........
APEC_O1      .......... .......... .......... .......... .......... .......... ..........
CFT073       .......... .......... .......... .......... .......... .......... ..........
E2348/69     .......... .......... .......... .......... .......... .......... ..........
ECOR_51      .......... .......... .......... .......... .......... .......... ..........
ECOR_57      .......... .......... .......... .......... .......... .......... ..........
UTI89        .......... .......... .......... .......... .......... .......... ..........
E._albertii  .T.....CT. .......... ..GC.T.... ....C..... ...GT..... .......... .G.....G..

        13511
K-12         CGCGCGTGAA TCAGGTGCCG AGCGCATCAT CGTGGCAACC GATCATGAGG ATGT-GCCCG CGCCGTTGAA
53638        .......... .......... .......... .......... .......... .......... ..........
ATCC_8739    .......... .......... .......... .......... .......... .......... ..........
ECOR_10      T..T..C... .......... .T........ .......... .......... .......... ..........
ECOR_11      .......... .......... .......... .......... .......... .......... ..........
B171         .......... .......... .......... .......... .......... .......... ..........
E24377A      T..T..C... .......... .T........ .......... .......... .......... ..........
ECOR_34      T..T..C... .......... .T........ .......... .......... .......... ..........
ECOR_71      .......... .......... .......... .......... .......... .......... ..........
SE11         T..T..C... .......... .T........ .......... .......... .......... ..........
O157_Sakai   ......C... .......... .......... .......... .......... .......... ..........
ECOR_36      ......C... .......... .......... .......... .......... .......... ...T......
ECOR_35      ......C... .......... .......... .......... .......... .......... ...T......
SMS-3-5      ......C... .......... .......... .......... .......... .......... ...T......
536          ......C... .......... .......... .......... .......... .......... ...T......
APEC_O1      ......C... .......... .......... .......... .......... .......... ...T......
CFT073       ......C... .......... .......... .......... .......... .......... ...T......
E2348/69     ......C... .......... .......... .......... .......... .......... ...T......
ECOR_51      ......C... .......... .......... .......... .......... .......... ...T......
ECOR_57      ......C... .......... .......... .......... .......... .......... ...T......
UTI89        ......C... .......... .......... .......... .......... .......... ...T......
E._albertii  ...C..A... ..G..A..T. .......... T..T...... ........A. ....C..T.. ..........

        13581
K-12         GCCGCTGGCG GTGAAGTATG TATGACGCGC GCCGATCATC AGTCAGGAAC AGAACGTCTG GCGGAAGTTG
53638        .......... .......... .......... .......... .......... .......... ..........
ATCC_8739    .......... .......... .......... .......... .......... .......... ..........
ECOR_10      .......... .......... .......... ........C. .......G.. ......C... ..........
ECOR_11      .......... .......... .......... .......... .......... .......... ..........
B171         .......... .......... .......... .......... .......... .......... ..........
E24377A      .......... .......... .......... ........C. .......G.. ......C... ..........
ECOR_34      .......... .......... .......... ........C. .......G.. ......C... ..........
ECOR_71      .......... .......... .......... .......... .......... .......... ..........
SE11         .......... .......... .......... ........C. .......G.. ......C... ..........
O157_Sakai   .......... .......... .......... ........C. .......G.. ......CT.. ..........
ECOR_36      .......... .......... .......... .......... .......... .......... ..........
ECOR_35      .......... .......... .......... .......... .......... .......... ..........
SMS-3-5      .......... .......... .......... .......... .......... .......... ..........
536          .......... .C........ .......... .......... .......T.. ......AT.. ..........
APEC_O1      .......... .C........ .......... .......... .......T.. ......AT.. ..........
CFT073       .T........ .C........ .......... .......... .......T.. .......... ..........
E2348/69     .......... .C........ .......... .......... .......T.. ......AT.. ..........
ECOR_51      .T........ .C........ .......... .......... .......T.. .......... ..........
ECOR_57      .T........ .C........ .......... .......... .......T.. .......... ..........
UTI89        .......... .C........ .......... .......... .......T.. ......AT.. ..........
E._albertii  .....G..G. .G........ C........A .......... .......C.. T..G...... ..........
```

```
        13651
K-12          TCGAAAAATG CGCATTCAGC GACGACACGG TGATCGTTAA TGTGCAGGGT GATGAACCGA TGATCCCTGC
53638         .......... .......... .......... .......... .......... .......... ..........
ATCC_8739     .......... .......... .......... .......... .......... .......... ..........
ECOR_10       .......... .......... .......... .......... .......... .......... ..........
ECOR_11       .......... .......... .......... .......... .......... .......... ..........
B171          .......... .......... .......... .......... .......... .......... ..........
E24377A       .......... .......... .......... .......... .......... .......... ..........
ECOR_34       .......... .......... .......... .......... .......... .......... ..........
ECOR_71       .......... .......... .......... .......... .......... .......... ..........
SE11          .......... .......... .......... .......... .......... .......... ..........
O157_Sakai    .......... ...G...... .......... .......... .......... .......... ..........
ECOR_36       .......... .......... .......... .......... .........G .......... ..........
ECOR_35       .......... .......... .......... .......... .........G .......... ..........
SMS-3-5       .......... .......... .......... .......... .........G .......... ..........
536           .......... .......... ........T. .......... .......... .......... ..........
APEC_O1       .......... .......... ........T. .......... .......... .......... ..........
CFT073        .......... .......... .......... .......... .......... .......... ..........
E2348/69      .......... .......... ........T. .......... .......... .......... ..........
ECOR_51       .......... .......... .......... .......... .......... .......... ..........
ECOR_57       .......... .......... .......... .......... .......... .......... ..........
UTI89         .......... .......... ........T. .......... .......... .......... ..........
E._albertii   .......... T..G..T... .........A .C.....G.. ...A...... ........C. .......C..

        13721
K-12          GACAATCATT CGTCAGGTTG CTGATAACCT CGCTCAGCGT CAGGTGGGTA TGGCGACTCT GGCGGTGCCA
53638         .......... .......... .......... .......... .......... .......... ..........
ATCC_8739     .......... .......... .......... .......... .......... .......... ..........
ECOR_10       .......... .......... .......... .......... .......... .......... ..........
ECOR_11       .......... .......... .......... .......... .......... .......... ..........
B171          ...C...... .......... .......... .......... .......... .......... .....C....
E24377A       .......... .......... .......... .......... .......... .......... ..........
ECOR_34       .......... .......... .......... .......... .......... .......... ..........
ECOR_71       .......... .......... .......... .......... .......... .......... ..........
SE11          .......... .......... .......... .......... .......... .......... ..........
O157_Sakai    .......... .......... .......... .......... .......... .......... ..........
ECOR_36       .......... .......... .......T.. ...C...... .......... .......... ..........
ECOR_35       .......... .......... .......T.. ...C...... .......... .......... ..........
SMS-3-5       .......... .......... .......T.. ...C...... .......... .......... ..........
536           .......... .......... .......... .......... .......... .......... ..........
APEC_O1       .......... .......... .......... .......... .......... .......... ..........
CFT073        .......... .......... .......... .......... .......... .......... ..........
E2348/69      .......... .......... .......... .......... .......... .......... ..........
ECOR_51       .......... .......... .......... .......... .......... .......... ..........
ECOR_57       .......... .......... .......... .......... .......... .......... ..........
UTI89         .......... .......... .......... .......... .......... .......... ..........
E._albertii   .......... .......... .C........ ...C...... ........A. .......... .........T

        13791
K-12          ATCCACAATG CGGAAGAAGC GTTTAACCCG AATGCGGTGA AAGTGGTTCT CGACGCTGAA GGGTATGCAC
53638         .......... .......... .......... .......... .......... .......... ..........
ATCC_8739     .......... .......... .......... .......... .......... .......... ..........
ECOR_10       .......... .......... .......... .......... .......... .......... ..........
ECOR_11       .......... .......... .......... .......... .......... .......... ..........
B171          .......... .......... .......... .......... ....C..... .......... ........T.
E24377A       .......... .......... .......... .......... .......... .......... ..........
ECOR_34       .......... .......... .......... .......... .......... .......... ..........
ECOR_71       ....G..... .......... .......... .......... .......... .......... ..........
SE11          .......... .......... .......... .......... .......... .......... ..........
O157_Sakai    .......... .......... .......... .......... .......... .......... ..........
ECOR_36       .......... ....G..... .......... .......... .......... ......C... .....C....
ECOR_35       .......... ....G..... .......... .......... .......... ......C... .....C....
SMS-3-5       .......... ....G..... .......... .......... .......C.. .......... ..........
536           .......... .......... .......... .......... .......... ......C... .....C....
APEC_O1       .......... .......... .......... .......... .......... .......... .....C....
CFT073        .......... .......... .......... .......... .......... ......C... .....C....
E2348/69      .......... .......... .......... .......... .......... ......C... .....C....
ECOR_51       .......... .......... .......... .......... .......... ......C... .....C....
ECOR_57       .......... .......... .......... .......... .......... ......C... .....C....
UTI89         .......... .......... .......... .......... .......... .......... .....C....
E._albertii   ..T....GC. .......... C........A .......... .......... ...T..C... ..T.......
```

```
         13861
K-12          TGTACTTCTC TCGCGCCACC ATTCCTTGGG ATCGTGATCG TTTTGCAGAA GGCCTTGAAA CCGTTGGCGA
53638         .......... .......... .......... .......... .......... .......... ..........
ATCC_8739     .......... .......... .......... .......... .......... .......... ..........
ECOR_10       .......... .......... .......... .......... .......... .......... ..........
ECOR_11       .......... .......... .......... .......... .......... .......... ..........
B171          .......... A......... .....G.... .......C.. .......A.. .A........ ..........
E24377A       .......... .......... .......... .......... .......... .......... ..........
ECOR_34       .......... .......... .......... .......... .......... .......... ..........
ECOR_71       .......... A......... .......... .......... .......... .......... ..........
SE11          .......... .......... .......... .......... .......... .......... ..........
O157_Sakai    .......... A.....T... .....G.... .......... .......A.. .......... ..........
ECOR_36       .......... .......... .....G.... .......... .......A.. .A........ ....A.....
ECOR_35       .......... .......... .....G.... .......... .......A.. .A........ ....A.....
SMS-3-5       .......... .......... .......... .......... .......A.. .......... ..........
536           .......... .......... .....G.... .......... .......A.. .A........ ....A.....
APEC_O1       .......... .......... .....G.... .......... .......A.. .A........ ....A.....
CFT073        .......... .......... .....G.... .......... .......A.. .A........ ....A.....
E2348/69      .......... .......... .....G.... .......... .....T.A.. .A........ ....A.....
ECOR_51       .......... .......... .....G.... .......... .......A.. .A........ ....A.....
ECOR_57       .......... .......... .....G.... .......... .......A.. .A........ ....A.....
UTI89         .......... .......... .....G.... .......... .......A.. .A........ ....A.....
E._albertii   .......T.. G..T...... .....G.... .......C.. ...C...A.. A.T.AGC... ..........

         13931
K-12          TAACTTCCTG CGTCATCTTG GTATTTATGG CTACCGTGCA GGCTTTATCC GTCGTTACGT CAACTGGCAG
53638         .......... .......... .......... .......... .......... .......... ..........
ATCC_8739     .......... .......... .......... .......... .......... .......... ..........
ECOR_10       .......... .......... .......... .......... .......... .......... ..........
ECOR_11       .......... .......... .......... .......... .......... .......... ..........
B171          .......... .......... .......... .......... .......... .......... ..C.......
E24377A       .......... .......... .......... .......... .......... .......... ..........
ECOR_34       .......... .......... .......... .......... .......... .......... ..........
ECOR_71       .......... .......... .......... .......... .......... .......... ..........
SE11          .......... .......... .......... .......... .......... .......... ..........
O157_Sakai    .......... .......... .......... .......... .......... .......... ..C.......
ECOR_36       .......... .......... .......... ......A... .......... .......T.. T.C.......
ECOR_35       .......... .......... .......... ......A... .......... .......T.. T.C.......
SMS-3-5       .......... .......... .......... .......... ..A....... .......... ..........
536           .......... .......... .......... ......A... .......... .......T.. T.C.......
APEC_O1       .......... .......... .......... ......A... .......... .......T.. T.C.......
CFT073        .......... .......... .......... ......A... .......... .......T.. T.C.......
E2348/69      .......... .......... .......... ......A... .......... .......T.. T.C.......
ECOR_51       .......... .......... .......... ......A... .......... .......T.. T.C.......
ECOR_57       .......... .......... .......... ......A... .......... .......T.. T.C.......
UTI89         .......... .......... .......... ......A... .......... .......T.. T.C.......
E._albertii   .T.T...... ......T.G. .G........ ......C..G ........T. .......T.. T........A

         14001
K-12          CCAAGTCCGT TAGAACACAT CGAAATGTTA GAGCAGCTTC GTGTTCTGTG GTACGGCGAA AAAATCCATG
53638         .......... .......... .......... .......... .......... .......... ..........
ATCC_8739     .......... .......... .......... .......... .......... .......... ..........
ECOR_10       .......... .......... .......... .......... .......... .......... ..........
ECOR_11       .......... .......... .......... .......... .......... ...T...... ..........
B171          .....C.... .......... .......... .......... .......... .......... ..........
E24377A       .......... .......... .......... .......... .......... .......... ..........
ECOR_34       .......... .......... .......... .......... .......... .......... ..........
ECOR_71       G......... .......... .......... .......... .......... .......... ..........
SE11          .......... .......... .......... .......... .......... .......... ..........
O157_Sakai    .....C.... .......... .......... .......... .....T.... .......... ..........
ECOR_36       .......... .......T.. .......... .......... .......... .......... ........C.
ECOR_35       .......... .......T.. .......... .......... .......... .......... ........C.
SMS-3-5       .......... .......... .......... .......... .......... .......... ..........
536           .......... .......T.. .......... .......... .......... .......... ........C.
APEC_O1       .......... .......T.. ......A... .......... .......... .......... ........C.
CFT073        .......... .......T.. .......... .......... .......... .......... ........C.
E2348/69      .......... .......T.. .......... .......... .......... .......... ........C.
ECOR_51       .......... .......T.. .......... .......... .......... .......... ........C.
ECOR_57       .......... .......T.. .......... .......... .......... .......... ........C.
UTI89         .......... .......T.. .......... .......... .......... .......... ........C.
E._albertii   ..G..C.... .......... .......C.G .....A.... .C..C..... .......... ........C.
```

```
        14071
K-12         TTGCTGTTGC TCAGGAAGTT CCTGGCACAG GTGTGGATAC CCCTGAAGAT CTTGAGCGCG TTCGCGCTGA
53638        .......... .......... .......... .......... .......... .......... ..........
ATCC_8739    .......... .......... .......... .......... .......... .......... ..........
ECOR_10      .......... .......... .......... .......... .......... .......... ..........
ECOR_11      .......... .......... .......... .......... .......... .......... ..........
B171         .......... .......... .......... .......... .......... .......... ..........
E24377A      .......... .......... .......... .......... .......... .......... ..........
ECOR_34      .......... .......... .......... .......... .......... .......... ..........
ECOR_71      .......... G..T...... .......... .......... .......... ..C....... ..........
SE11         .......... .......... .......... .......... .......... .......... ..........
O157_Sakai   .......C.. G..T...... .......... .......... .......... ..C....... ..........
ECOR_36      .......C.. C......... .......... .......... .......... ..C......A ..........
ECOR_35      .......C.. C......... .......... .......... .......... ..C......A ..........
SMS-3-5      .......C.. C......... .......... .......... .......... ..C....... ..........
536          .......C.. C......... .......... .......... .......... ..CA...... .......C..
APEC_O1      .......C.. C......... .......... .......... .......... ..CA...... .......C..
CFT073       .......C.. C......... .......... .......... .......... ..CA...... .......C..
E2348/69     .......C.. C......... .......... .......... .......... ..CA...... .......C..
ECOR_51      .......C.. C......... .......... .......... .......... ..CA...... .......C..
ECOR_57      .......C.. C......... .......... .......... .......... ..CA...... .......C..
UTI89        .......C.. C......... .......... .......... .......... ..CA...... .......C..
E._albertii  .......A.. C........A ..G..A..G. .C..C..... T..C...... ..C..A.... ....T.....

        14141
K-12         AATGCGCTAA TTTCACTTCA CGACACTTCA GCCAATTTTG GGAGGAGTGT CGTACCGTTA CGATTTTCCT
53638        .......... .......... .......... .......... .......... .......... ..........
ATCC_8739    .......... .......... .......... .......... .......... .......... ..........
ECOR_10      .......... .......... .......... .......... .......... .......... ..........
ECOR_11      .......... .......... .......... .......... .......... .......... ..........
B171         .......... .......... .......... .......... .......... .......... ..........
E24377A      .......... .......... .......... .......... .......... .......... ..........
ECOR_34      .......... .......... .......... .......... .......... .......... ..........
ECOR_71      .......... .......... .......... .......... .......... .......... ..........
SE11         .......... .......... .......... .......... .......... .......... ..........
O157_Sakai   .......... .......... .......... .......... .......... .......... ..........
ECOR_36      .......... .......... .......C.. .......... .......... .......... ..........
ECOR_35      .......... .......... .......C.. .......... .......... .......... ..........
SMS-3-5      .......... .......... .......C.. .......... .......... .......... ..........
536          .......... .......... .......C.. .......... .......... .......... ..........
APEC_O1      .......... .......... .......C.. .......... .......... .......... ..........
CFT073       .......... .......... .......C.. .......... .......... .......... ..........
E2348/69     .......... .......... .......C.. .......... .......... .......... ..........
ECOR_51      .......... .......... .......C.. .......... .......... .......... ..........
ECOR_57      .......... .......... .......C.. .......... .......... .......... ..........
UTI89        .......... .......... .......C.. .......... .......... .......... ..........
E._albertii  ......T... C.CATT.C.. GA.....C.T C.....AC.. .......... .......... ..........

        14211
K-12         CAATTTTTCT TTTCAACAAT TGATCTCATT CAGGTGACAT CTTTTATATT GGCGCTCATT ATGAAAGCAG
53638        .......... .......... .......... .......... .......... .......... ..........
ATCC_8739    .......... .......... .......... .......... .......... .......... ..........
ECOR_10      .......... .......... .......... .......... .......... .......... ..........
ECOR_11      .......... .......... .......... .......... .......... .......... ..........
B171         .......... .......... .......... .......... .......... .......... ..........
E24377A      .......... .......... .......... .......... .......... .......... ..........
ECOR_34      .......... .......... .......... .......... .......... .......... ..........
ECOR_71      .......... .......... .......... .......... .......... .......... ..........
SE11         .......... .......... .......... .......... .......... .......... ..........
O157_Sakai   .......... .......... .......... .......... .......... .......... ..........
ECOR_36      .......... .......... .......... .......... .......... .......... ..........
ECOR_35      .......... .......... .......... .......... .......... .......... ..........
SMS-3-5      .......... .......... .......... .......... .......... .......... ..........
536          .......... .......... .......... .......... .......... .......... ..........
APEC_O1      .......... .......... .......... .......... .......... .......... ..........
CFT073       .......... .......... .......... .......... .......... .......... ..........
E2348/69     .......... .......... .......... .......... .......... .......... ..........
ECOR_51      .......... .......... .......... .......... .......... .......... ..........
ECOR_57      .......... .......... .......... .......... .......... .......... ..........
UTI89        .......... .......... .......... .......... .......... .......... ..........
E._albertii  .......... .......... .......... .......... .......... .A........ ..........
```

```
        14281
K-12          TAGCTTTTAT GAGGGTAATC TGAATGGAAC AGCTGCGTGC CGAATTAAGC CATTTACTGG GCGAAAAACT
53638         .......... .......... .......... .......... .......... .......... ..........
ATCC_8739     .......... .......... .......... .......... .......... .......... ..........
ECOR_10       .......... .......... .......... .......... .......... .......... ..........
ECOR_11       .......... .......... .......... .......... .......... .......... ..........
B171          .......... .......... .......... .......... .......... .......... ..........
E24377A       .......... .......... .......... .......... .......... .......... ..........
ECOR_34       .......... .......... .......... .......... .......... .......... ..........
ECOR_71       .......... .......... .......... .......... .......... .......... ..........
SE11          .......... .......... .......... .......... .......... .......... ..........
O157_Sakai    .......... .......... .......... .......... .......... .......... ..........
ECOR_36       .......... .......... .......... .......... .......... .......... ..........
ECOR_35       .......... .......... .......... .......... .......... .......... ..........
SMS-3-5       .......... .......... .......... .......... .......... .......... ..........
536           .......... .......... .......... .......... .......... .......... ..........
APEC_O1       .......... .......... .......... .......... .......... .......... ..........
CFT073        .......... .......... .......... .......... .......... .......... ..........
E2348/69      .......... .......... .......... .......... .......... .......... ..........
ECOR_51       .......... .......... .......... .......... .......... .......... ..........
ECOR_57       .......... .......... .......... .......... .......... .......... ..........
UTI89         .......... .......... .......... .......... .......... .......... ..........
E._albertii   .......... .......... .......... .......... .......... ...C.G.... ..........

        14351
K-12          CAGTCGTATT GAGTGCGTCA ATGAAAAAGC GGATACGGCG TTGTGGGCTT TGTATGACAG CCAGGGAAAC
53638         .......... .......... .......... .......... .......... .......... ..........
ATCC_8739     .......... .......... .......... .......... .......... .......... ..........
ECOR_10       .......... .......... .......... .......... .......... .......... ..........
ECOR_11       .......... .......... .......... .......... .......... .......... ..........
B171          .......... .......... .......... .......... .......... .......... ..........
E24377A       .......... .......... .......... .......... .......... .......... ..........
ECOR_34       .......... .......... .......... .......... .......... .......... ..........
ECOR_71       .......... .......... .......... .......... .......... .......... ..........
SE11          .......... .......... .......... .......... .......... .......... ..........
O157_Sakai    .......... .......... .......... .......... .......... .......... ..........
ECOR_36       .......... .......... .......... .......... .......... .......... ..........
ECOR_35       .......... .......... .......... .......... .......... .......... ..........
SMS-3-5       .......... .......... .......... .......... .......... .......... ..........
536           .......... .......... .......... .......... .......... .......... ..........
APEC_O1       .......... .......... .......... .......... .......... .......... ..........
CFT073        .......... .......... .......... .......... .......... .......... ..........
E2348/69      .......... .......... .......... .......... .......... .......... ..........
ECOR_51       .......... .......... .......... .......... .......... .......... ..........
ECOR_57       .......... .......... .......... .......... .......... .......... ..........
UTI89         .......... .......... .......... .......... .......... .......... ..........
E._albertii   .......... .....T..T. .......... .A...A..A. .......... ....C..... .A........

        14421
K-12          CCAATGCCGT TAATGGCAAG AAGCTTTAGT ACGCCCGGAA AAGCCCGACA ACTGGCATGG AAAACCACCA
53638         .......... .......... .......... .......... .......... .......... .......T..
ATCC_8739     .......... .......... .......... .......... .......... .......... ..........
ECOR_10       .......... .......... .......... .......... .......... .......... ..........
ECOR_11       .......... .......... .......... .......... .......... .......... ..........
B171          .......... .......... .......... .......... .......... .......... ..........
E24377A       .......... .......... .......... .......... .......... .......... ..........
ECOR_34       .......... .......... .......... .......... .......... .......... ..........
ECOR_71       .......... .......... .......... .......... .......... .......... ..........
SE11          .......... .......... .......... .......... .......... .......... ..........
O157_Sakai    .........C .......... .......... .......... .......... .......... ..........
ECOR_36       .......... .......... .......... G......... .......... .......... ..........
ECOR_35       .......... .......... .......... G......... .......... .......... ..........
SMS-3-5       .......... .......... .......... .......... .......... .......... ..........
536           .......... .......... .......... G......... .......... .......... ..........
APEC_O1       .......... .......... .......... G......... .......... .......... ..........
CFT073        .......... .......... .......... G......... .......... .......... ..........
E2348/69      .......... .......... .......... G......... .......... .......... ..........
ECOR_51       .......... .......... .......... G......... .......... .......... ..........
ECOR_57       .......... .......... .......... G......... .......... .......... ..........
UTI89         .......... .......... .......... G......... .......... .......... ..........
E._albertii   .......... .G........ .......... ..T..A..GC .G.....G.. GT........ .....G....
```

```
         14491
K-12         TGCTGGCAAG AAGTGGGACT GTCCGTATGC CGACTATTTA TGGTGTGATG ACGCATGAAG AACACCCCGG
53638        .......... .......... .......... .......... .......... .......... ..........
ATCC_8739    .......... .......... .......... .......... .......... .......... ..........
ECOR_10      .......... .......... .......... .......... .......... .......... ..........
ECOR_11      .......... .......... .......... .......... .......... .......... ..........
B171         .......... .......... .......... .......... .......... .......... ..........
E24377A      .......... .......... .......... .......... .......... .......... ..........
ECOR_34      .......... .......... .......... .......... .......... .......... ..........
ECOR_71      .......... .......... .......... .......... .......... .......... ..........
SE11         .......... .......... .......... .......... .......... .......... ..........
O157_Sakai   .......... .......... .......... .......... .......... .......... ..........
ECOR_36      .......... .......... .......... .......... .......... .......... ..........
ECOR_35      .......... .......... .......... .......... .......... .......... ..........
SMS-3-5      .......... .......... .......... .......... .......... .......... ..........
536          .......... .......... .......... .......... .......... .......... ..........
APEC_O1      .......... .......... .......... .......... .......... .......... ..........
CFT073       .......... .......... .......... .......... .......... .....C.... ..........
E2348/69     .......... .......... .......... .......... .......... .......... ..........
ECOR_51      .......... .......... .......... .......... .......... .....C.... ..........
ECOR_57      .......... .......... .......... .......... .......... .....C.... ..........
UTI89        .......... .......... .......... .......... .......... .......... ..........
E._albertii  ..T.....C. .......... ..T..G.... ....A..C.. ...C...... ........G. .G..T..T..

         14561
K-12         CCCCGATGTC CTGCTACTGG AGCGGATGCG TGGTGTTTCG GTGGAGGCAC CAGCCCGAAC ACCAGAACGC
53638        T......... .......... .......... .......... .......... .......... ..........
ATCC_8739    .......... .......... .......... .......... .......... .......... ..........
ECOR_10      .......... .......... .......... .......... .......... .......... ..........
ECOR_11      .......... .......... .......... .......... .......... .......... ..........
B171         ...T...... .......... .......... .......... .......... .......... ..........
E24377A      .......... .......... .......... .......... .......... .......... ..........
ECOR_34      .......... .......... .......... .......... .......... .......... ..........
ECOR_71      .......... .......... .......... .......... .......... .......... ..........
SE11         .......... .......... .......... .......... .......... .......... ..........
O157_Sakai   ...T...... .......... .......... .......... ..A....... .......... .........T
ECOR_36      .......... .......... ....A..... .......... .......... .......... ..........
ECOR_35      .......... .......... ....A..... .......... .......... .......... ..........
SMS-3-5      .......... .......... .......... .......... .......... .......... ..........
536          .......... .......... ....A..... .......... .......... .......... ..........
APEC_O1      .......... .......... ....A..... .......... .......... .......... ..........
CFT073       .......... .......... ....A..... .......... .......... .......... ..........
E2348/69     .......... .......... ....A..... .......... .......... .......... ..........
ECOR_51      .......... .......... ....A..... .......... .......... .......... ..........
ECOR_57      .......... .......... ....A..... .......... .......... .......... ..........
UTI89        .......... .......... ....A..... .......... .......... .......... ..........
E._albertii  A..T...... T..T.G.... ....A..... ...C.....T ..C..A..G. .C..A..T.. G.........

         14631
K-12         TGGGAACAAC TCAAAGACCA AATCGTTGAA GCCTTACTGG CCTGGCACCG TCAGGACAGT CGCGGTTGCG
53638        .......... .......... .......... .......... .......... .......... ..........
ATCC_8739    .......... .......... .......... .......... .......... .......... ..........
ECOR_10      .......... .......... .......... .......... .......... .......... ..........
ECOR_11      .......... .......... .......... .......... .......... .......... ..........
B171         ........G. .......... .......... .......... .......... .......... ..........
E24377A      .......... .......... .......... .......... .......... .......... ..........
ECOR_34      .......... .......... .......... .......... .......... .......... ..........
ECOR_71      .......... .......... .......... .......... .......... .......... ..........
SE11         .......... .......... .......... .......... .......... .......... ..........
O157_Sakai   ........G. .......... .......... .......... .......... .......... ..........
ECOR_36      .......... .......T.. .......... .......... .......... .......... ..T.......
ECOR_35      .......... .......T.. .......... .......... .......... .......... ..T.......
SMS-3-5      .......... .......T.. .......... .......... .......... .......... ..........
536          .......... .......T.. .......... .......... .......... .......... ..T.......
APEC_O1      .......... .......T.. .......... .......... .......... .......... ..T.......
CFT073       .......... .......T.. .......... .......... .......... .......... ..T.......
E2348/69     .......... .......T.. .......... .......... .......... .......... ..T.......
ECOR_51      .......... .......T.. .......... .......... .......... .......... ..T.......
ECOR_57      .......... .......T.. .......... .......... .......... .......... ..T.......
UTI89        .......... .......T.. .......... .......... .......... .......... ..T.......
E._albertii  ....GG..T. .A.....T.. ......G... ..G..G.... .......... .......... .....C....
```

```
        14701
K-12           TCGGCGCGGT CGACAATACT CAGGAAAATT TCTGGCCCTC ATGGTACCGG CAACATGTTG AAGTGCTATG
53638          .......... .......... .......... .......... .......... .......... ..........
ATCC_8739      .T........ .......... .......... .......... .......... .......... ..........
ECOR_10        .......... .......... .......... .......... .......... .......... ..........
ECOR_11        .......... .......... .......... .......... .......... .......... ..........
B171           .......... .......... .......... .......... ........T. .......... ..........
E24377A        .......... .......... .......... .......... .......... .......... ..........
ECOR_34        .......... .......... .......... .......... .......... .......... ..........
ECOR_71        .......... .......... .......... .......... .......... .......... ..........
SE11           .......... .......... .......... .......... .......... .......... ..........
O157_Sakai     .......... .......... .......... .......... .......... .......... ..........
ECOR_36        .......... .......... .......... .......... .......... .......... ..........
ECOR_35        .......... .......... .......... .......... .......... .......... ..........
SMS-3-5        .......... .......... .......... .......... .......... .......... ..........
536            .......... .......... .......... .......... .......... .......... ..........
APEC_O1        .......... .......... .......... .......... .......... .......... ..........
CFT073         .......... .......... .......... .......... .......... .......... ..........
E2348/69       .......... .......... .......... .......... .......... .......... ..........
ECOR_51        .......... .......... .......... .......... .......... .......... ..........
ECOR_57        .......... .......... .......... .......... .......... .......... ..........
UTI89          .......... .......... .......... .......... .......... .......... ..........
E._albertii    ....TC.... A..T...... .......... .......T.. C.....T.A. .......... ......A...

        14771
K-12           GACCACGCTC AATCAGTTCA ATAACACCGG TCTGACGATG CAGGATAAGC GGATCCTGTT TCGCACTCGC
53638          .......... .......... .......... .......... .......... .......... ..........
ATCC_8739      .......... .......... .......... .......... .......... .......... ..........
ECOR_10        .......... .......... .......... .......... .......... .......... ..........
ECOR_11        .......... .......... .......... .......... .......... .......... ..........
B171           .......... .......... ....T..... .......... .......... .......... ..........
E24377A        .......... .......... .......... .......... .......... .......... ..........
ECOR_34        .......... .......... .......... .......... .......... .......... ..........
ECOR_71        .......... .......... .......... .......... .......... .......... ..........
SE11           .......... .......... .......... .......... .......... .......... ..........
O157_Sakai     .......... .......... .......... .......... .......... .......... ..........
ECOR_36        .......... .......... .......... .......... .......... .......... ..........
ECOR_35        .......... .......... .......... .......... .......... .......... ..........
SMS-3-5        .......... .......... .......... .......... .......... .......... ..........
536            .......... .......... .......... ...A...... .......... .......... ..........
APEC_O1        .......... .......... .......... ...A...... .......... .......... ..........
CFT073         .......... .......... .......... ...A...... .......... .......... ..........
E2348/69       .......... .......... .......... ...A...... .......... .......... ..........
ECOR_51        .......... .......... .......... ...A...... .......... .......... ..........
ECOR_57        .......... .......... .......... ...A...... .......... .......... ..........
UTI89          .......... .......... .......... ...A...... .......... .......... ..........
E._albertii    .G.G..A... .......... ....T..T.. CT.A..C... ........A. .......... C..T..A..T

        14841
K-12           GAATGTCTCC CGGCATTATT TGAAGGCTTT AACGACAATT GTGTGCTGAT TCACGGTAAC TTCTGTTTAC
53638          .......... .......... .......... .......... .......... .......... ..........
ATCC_8739      .......... .......... .......... .......... .......... .......... ..........
ECOR_10        .......... .......... .......... .......... .......... .......... ..........
ECOR_11        .......... .......... .......... .......... .......... .......... ..........
B171           .......... .......... .......... .......... .....T.... .......... ..........
E24377A        .......... .......... .......... .......... .......... .......... ..........
ECOR_34        .......... .......... .......... .......... .......... .......... ..........
ECOR_71        .......... .......... .......... .......... .....T.... .......... ..........
SE11           .......... .......... .......... .......... .....T.... .......... ..........
O157_Sakai     .......... .......... ......T... .......... .......... .......... ..........
ECOR_36        .......... .......... .......... .......... ....A..... .......... ..........
ECOR_35        .......... .......... .......... .......... ....A..... .......... ..........
SMS-3-5        .......... .......... .......... .......... ....A..... .......... ..........
536            .......... .......... .......... .......... ....A..... .......... ..........
APEC_O1        .......... .......... .......... .......... ....A..... .......... ..........
CFT073         .......... .......... .......... .......... ....A..... .......... ..........
E2348/69       .......... .......... .......... .......... ....A..... .......... ..........
ECOR_51        .......... .......... .......... .......... ....A..... .......... ..........
ECOR_57        .......... .......... .......... .......... ....A..... .......... ..........
UTI89          .......... .......... .......... .......... ....A..... .......... ..........
E._albertii    ........G. .....C.G.. ......A... .....T.... .C........ C.....C..T ..T..C..G.
```

```
        14911
K-12         GCAGCATGTT GAAAGATTCG CGCAGCGATC AGTTACTGGC GATGGTCGGG CCGGGACTAA TGCTTTGGGC
53638        .......... .......... .......... .......... .......... .......... ..........
ATCC_8739    .......... .......... .......... .......... .......... .......... ..........
ECOR_10      .......... .......... .......... .......... .......... .......... ..........
ECOR_11      .......... .......... .......... .......... .......... .......... ..........
B171         ........C. .......... .......... .......... .......... .......... ..........
E24377A      .......... .......... .......... .......... .......... .......... ..........
ECOR_34      .......... .......... .......... .......... .......... .......... ..........
ECOR_71      ........C. .......... .......... .......... .......... .......... .........T
SE11         ........C. .......... .......... .......... .......... .......... ..........
O157_Sakai   .......... .......... .......... .......... .......... .......... ..........
ECOR_36      ........C. .......... .......... .......... .........A .......... ..........
ECOR_35      ........C. .......... .......... .......... .........A .......... ..........
SMS-3-5      ........C. .......... .......... .......... .........A .......... ..........
536          .T......C. .......... .......... .......... .........A .......... ..........
APEC_O1      .T......C. .......... .......... .......... .........A .......... ..........
CFT073       .T......C. .......... .......... .......... .........A .......... ..........
E2348/69     .T......C. .......... .......... .......... .........A .......... ..........
ECOR_51      .T......C. .......... .......... .......... .........A .......... ..........
ECOR_57      .T......C. .......... .......... .......... .........A .......... ..........
UTI89        .T......C. .......... .......... .......... .........A .......... ..........
E._albertii  ....T..... .........C .......... ....G..... .......... ..T..C..C. ....C.....

        14981
K-12         ACCGCGAGAA TACGAACTGT TCCGACTAAT GGATAATTCT CTGGCGGAAG ATTTGCTCTG GAGTTACCTG
53638        .......... .......... .......... .......... .......... .......... ..........
ATCC_8739    .......... .......... .......... .......... .......... .......... ..........
ECOR_10      .......... .......... .......... .......... .......... .......... ..........
ECOR_11      .......... .......... .......... .......... .......... .......... ..........
B171         .......... .......... .......... .......... .......... .......... ..........
E24377A      .......... .......... .......... .......... .......... .......... ..........
ECOR_34      .......... .......... .......... .......... .......... .......... ..........
ECOR_71      .......... .......... .......... .......... .......... .......... ..........
SE11         .......... .......... .......... .......... .......... .......... ..........
O157_Sakai   .......... .......... .......... .......... .......... .......... ..........
ECOR_36      .......... .......... .......... .......... .......... .......... ..........
ECOR_35      .......... .......... .......... .......... .......... .......... ..........
SMS-3-5      .......... .......... .......... .......... .......... .......... ......T...
536          .......... .......... .......... .......... .......... .......... ......T...
APEC_O1      .......... .......... .......... .......... .......... .......... ......T...
CFT073       .......... .......... .......... .......... .......... .......... ......T...
E2348/69     .......... .......... .......... .......... .......... .......... ......T...
ECOR_51      .......... .......... .......... .......... .......... .......... ......T...
ECOR_57      .......... .......... .......... .......... .......... .......... ......T...
UTI89        .......... .......... .......... .......... .......... .......... ......T...
E._albertii  G.....C..G ..T..G.... .T..CT.G.. .......C.C .......... .......... ......T...

        15051
K-12         CAACGCGCGC CAGTGGCGGA GTCGTTCATC TGGCGGCGTT GGTTGTATGT GTTATGGGAT GAAGTTGCGC
53638        .......... .......... .......... .......... .......... .......... ..........
ATCC_8739    .......... .......... .......... .......... .......... .......... ..........
ECOR_10      .......... .......... .......... .......... .......... .......... ..........
ECOR_11      .......... .......... .......... .......... .......... .......... ..........
B171         .......... .......... .......... .......... .......... .......... ..........
E24377A      .......... .......... .......... .......... .......... .......... ..........
ECOR_34      .......... .......... .......... .......... .......... .......... ..........
ECOR_71      .......... .......... .......... .......... .......... .......... ..........
SE11         .......... .......... .......... .......... .......... .......... ..........
O157_Sakai   .......... .......... .......... .......... .......... .......... ..........
ECOR_36      .......... .......... .......... .......... .......... .......... ..........
ECOR_35      .......... .......... .......... .......... .......... .......... ..........
SMS-3-5      .......... .......... .......... .......... .......... .......... ..........
536          .......... .......... .......... .......... .......... .......... ..........
APEC_O1      .......... .......... .......... .......... .......... .......... ..........
CFT073       .......... .......... .......... .......... .......... .......... ..........
E2348/69     .......... .......... .......... .......... .......... .......... ..........
ECOR_51      .......... .......... .......... .......... .......... .......... ..........
ECOR_57      .......... .......... .......... .......... .......... .......... ..........
UTI89        .......... .......... .......... .......... .......... .......... ..........
E._albertii  .......... .......A.. A..C...... .......... ..C.A..... T......A.. ..G.......
```

```
        15121
K-12          AACTGGTTAA TACCGGACGA TTTAGTCGGC GCAACTTCGA TCTGGCATCA AAATCACTCT TGCCGTGGCT
53638         .......... .......... .......... ...C...... .......... .......... ..........
ATCC_8739     .......... .......... .......... .......... .......... .......... ..........
ECOR_10       .......... .......... .......... .......... .......... .......... ..........
ECOR_11       .......... .......... .......... .......... .......... .......... ..........
B171          .......... .......... .......... .......... .......... .......... ..........
E24377A       .......... .......... .......... .......... .......... .......... ..........
ECOR_34       .......... .......... .......... .......... .......... .......... ..........
ECOR_71       .......... .......... .......... .......... .......... .......... ..........
SE11          .......... .......... .......... .......... .......... .......... ..........
O157_Sakai    .......... .......... .......... .......... .......... .......... ..........
ECOR_36       .......C.. C......... .......... .T........ .......... .......... ..........
ECOR_35       .......C.. C......... .......... .T........ .......... .......... ..........
SMS-3-5       .......C.. C......... .......... .T........ .......... .......... ..........
536           .......C.. C......... .......... .T........ .......... .......... ..........
APEC_O1       .......C.. C......... .......... .T........ .......... .......... ..........
CFT073        .......... .......... .......... .......... .......... .......... ..........
E2348/69      .......C.. C......... .......... .T........ .......... .......... ..........
ECOR_51       .......... .......... .......... .......... .......... .......... ..........
ECOR_57       .......... .......... .......... .......... .......... .......... ..........
UTI89         .......C.. C......... .......... .T........ .......... .......... ..........
E._albertii   .......... ......G..C ....AC..CA .T........ C.....GG.. ........G. ....C.....

        15191
K-12          CGCCTGACGA ACCTTTTAGC CACTGCCAGA TACGGCCAAG CGTTTCATAG CCAACACGAT CGCTATGCAT
53638         .......... .......... .......... .......... .......... .......... ..........
ATCC_8739     .......... .......... .......... .......... .......... .......... ..........
ECOR_10       .......... .......... .......... .......... .......... .......... ..........
ECOR_11       .......... .......... .......... .......... .......... .......... ..........
B171          .........G ......C... .......... .G........ .......... .......... ..........
E24377A       .......... .......... .......... .......... .......... .......... ..........
ECOR_34       .......... .......... .......... .......... .......... .......... ..........
ECOR_71       .........G ......C... .......... .G........ .......... .......... ..........
SE11          .........G ......C... .......... .G........ .......... .......... ..........
O157_Sakai    .......... .......... .......... .G........ .......... .......... ..........
ECOR_36       .......... .......... .......... .G........ .......... .......... ..........
ECOR_35       .......... .......... .......... .G........ .......... .......... ..........
SMS-3-5       .......... ......C... ..T....... .C........ .......... .....G..G. ..........
536           .......... ......C... .......... .T........ .......... .....G..G. ..........
APEC_O1       .......... .......... .......... .G........ .......... .......... ..........
CFT073        .......... .......... .......... .G........ .......... .......... ..........
E2348/69      .......... ......C... .......... .C........ .......... .....G..G. ..........
ECOR_51       .......... .......... .......... .G........ .......... .......... ..........
ECOR_57       .......... .......... .......... .G........ .......... .......... ..........
UTI89         .......... .......... .......... .G........ .......... .......... ..........
E._albertii   T.T......G .G........ ..T....... G...A..G.. ......G..A ..C....... ..........

        15261
K-12          CAACCAGACC GGAGAAGGGA TTGCCCGTTC CCACGGGTTA AGCGGCGAGT CGATGGCCAA CTGATTCGCC
53638         .......... .......... .......... .......... .......... .......... ..........
ATCC_8739     .......... .......... .......... .......... .......... .......... ..........
ECOR_10       .......... .......... .......... .......... .......... .......... ..........
ECOR_11       .......... .......... .......... .......... .......... .......... ..........
B171          ..G....... .......... .......... .......... .......... .......... ..........
E24377A       ..G....... .......... .......... .......... .......... .......... ..........
ECOR_34       .......... .......... .......... .......... .......... .......... ..........
ECOR_71       ..G....... .......... .......... ......A... .......... .......... ..........
SE11          ..G....... .......... .......... ......A... .......... .......... ..........
O157_Sakai    .......... .......... .......... .......... .......... .......... ..........
ECOR_36       .......... .......... .......... .......... .......... .......... ..........
ECOR_35       .......... .......... .......... .......... .......... .......... ..........
SMS-3-5       ......A... .......... .......... ......A... .......... .......... ..........
536           ......A... .......... .......... ......A... .......... .......... ..........
APEC_O1       ......A... .......... .......... ......A... .......... .......... ..........
CFT073        .......... .......... .......... ......A... .......... .......... ..........
E2348/69      ......A... .......... .......... ......A... .......... .......... ..........
ECOR_51       .......... .......... .......... ......A... .......... .......... ..........
ECOR_57       .......... .......... .......... ......A... .......... .......... ..........
UTI89         ......A... .......... .......... ......A... .......... .......... ..........
E._albertii   .......... .....C..A. ....T..... ...T..A... .....T.... ....A..... ......T...
```

```
         15331
K-12          GGGGCTGGTA GCGGATTTAA CCCTTCCTGC TGAAAAAAGA TCATCGCCCG CGGCAGATGT GATGCGGAGG
53638         .......... .......... .......... .......... .......... .......... ..........
ATCC_8739     .......... .......... .......... .......... .......... .......... ..........
ECOR_10       .......... .......... .......... .......... .......... .......... ..........
ECOR_11       .......... .......... .......... .......... .......... .......... ..........
B171          .......... .......... .......... .......... .......... .......... ..........
E24377A       .......... .......... .......... .......... .......... .......... ..........
ECOR_34       .......... .......... .......... .......... .......... .......... ..........
ECOR_71       .......... .......... .......... .......... .......... .......... ..........
SE11          .......... .......... .......... .......... .......... .......... ..........
O157_Sakai    .......... .T........ .......... .......... .......... .......... ..........
ECOR_36       .......... .......... .......... .......... .......... .....A...A ..C..A..T.
ECOR_35       .......... .......... .......... .......... .......... .....A...A ..C..A..T.
SMS-3-5       .......... .......... .......... .......... .......... .......... ..........
536           .......... .......... .......... .......... .......... .......... ..........
APEC_O1       ........C. .........G .......... .......... .......... .......... ..........
CFT073        .......... .......... .......... .......... .......... .......... ..........
E2348/69      .......... .......... .......... .......... .......... .......... ..........
ECOR_51       .......... .......... .......... .......... .......... .......... ..........
ECOR_57       .......... .......... .......... .......... .......... .......... ..........
UTI89         ........C. .........G .......... .......... .......... .......... ..........
E._albertii   ..C..C..C. .T..T..C.G ....G.T..T .......... .......G.. G..T.A...A .....A..A.

         15401
K-12          TCACCAGCAG GAAAGGGGCG TCACCAATCG CCTGTTTCAC TGCTGCAGCT TCTTCTTCGG TATCTTTTGG
53638         .......... .......... .......... .......... ...C...... .......... ..........
ATCC_8739     .......... .......... .......... .......... ...C...... .......... ..........
ECOR_10       .......... .......... .......... .......... ...C...... .......... ..........
ECOR_11       .......... .......... .......... .......... .......... .......... ..........
B171          .......... .......... .......... .......... ...C...... .......... ..........
E24377A       .......... .......... .......... .......... ...C...... .......... ..........
ECOR_34       .......... .......... .......... .......... ...C...... .......... ..........
ECOR_71       .......... .......... .......... .......... ...C...... .......... ..........
SE11          .......... .......... .......... .......... ...C...... .......... ..........
O157_Sakai    .......... .......... .......... .......... ...C...... .......... ..........
ECOR_36       .......... ......T... .......... .......... ...C...... .......... ..........
ECOR_35       .......... ......T... .......... .......... ...C...... .......... ..........
SMS-3-5       .......... .......... .......... .......... ...C...... .......... ..........
536           .......... ......T... .......... .......... .......... .......... ..........
APEC_O1       .......... .......... ........T. .......... ...C...... .......... ..........
CFT073        .......... ......T... .......... .......... .......... .......... ..........
E2348/69      .......... ......T... .......... .......... .......... .......... ..........
ECOR_51       .......... ......T... .......... .......... .......... .......... ..........
ECOR_57       .......... ......T... .......... .......... .......... .......... ..........
UTI89         .......... .......... ........T. .......... ...C...... .......... ..........
E._albertii   .......... .......... .TG.....T. .......... C......... .......... ....C.....

         15471
K-12          CAAATCCAGG GTGATAATTT GCTCGCGCGG CACGCCCAGC GATTGCGCAA CTCTGGCACC TACTTCCGCT
53638         .......... .......... .......... .......... .......... .......... ..........
ATCC_8739     .......... .......... .......... .......... .......... .......... ..........
ECOR_10       .......... .......... .....T.... .......... .......... .......... ..........
ECOR_11       .......... .......... .......... .......... .......... .......... ..........
B171          .......... .......... .......... .......... .......... .......... ..........
E24377A       .......... .......... .......... .......... .......... .......... ..........
ECOR_34       .......... .......... .....T.... .......... .......... .......... ..........
ECOR_71       .......... .......... .......... .......... .......... .......... ..........
SE11          .......... .......... .......... .......... .......... .......... ..........
O157_Sakai    .......... .......... .......... T......... .......... .......... ..........
ECOR_36       .......... .......... .......... T......... .......... .......... ..........
ECOR_35       .......... .......... .......... T......... .......... .......... ..........
SMS-3-5       .......... .......... .......... T......... .......... .......... ..........
536           .......... .......... .......... .......... .......... .......... ......G...
APEC_O1       .........A .......... .......... .......... .......... ....T..... ......G...
CFT073        .......... .......... .......... .......... .......... .......... ..........
E2348/69      .......... .......... .......... .......... .......... .......... ......G...
ECOR_51       .......... .......... .......... .......... .......... .......... ..........
ECOR_57       .......... .......... .......... .......... .......... .......... ..........
UTI89         .........A .......... .......... .......... .......... ....T..... ......G...
E._albertii   T......... .......... .......... G.....G... .....T.... .......T.. C.........
```

```
          15541
K-12         GTACTCACCG TATTGGTTTT TGCTACGCCT CCCGTGAAGA TCAGTTTTGA TCCCGGATTT TCGCGCCATA
53638        .......... .......... .......... .......... .......... .......... ..........
ATCC_8739    .......... .......... .......... .......... .......... .......... ..........
ECOR_10      .......... .......... .......... .......... .......... .......... ..........
ECOR_11      .......... .......... .......... .......... .......... .......... ..........
B171         .......... .......... .......... .......... .......... .......... ..........
E24377A      .......... .......... .......... .......... .......... .......... ..........
ECOR_34      .......... .......... .......... .......... .......... .......... ..........
ECOR_71      .......... .......... .......... .......... .......... .......... ..........
SE11         .......... .......... .......... .......... .......... .......... ..........
O157_Sakai   .......... .......... .......... .......... .......... A......... ..........
ECOR_36      .......... .......... .......... .......... .......... .......... ..........
ECOR_35      .......... .......... .......... .......... .......... .......... ..........
SMS-3-5      .......... .......... .......... .......... .......... .......... ..........
536          ..G....... .......... A......... .....A.... .......... .......... ..........
APEC_O1      .......... .......... .......... .......... .......... .......... ..........
CFT073       ..G....... .......... A......... .....A.... .......... .......... ..........
E2348/69     ..G....... .......... A......... .....A.... .......... .......... ..........
ECOR_51      ..G....... .......... A......... .....A.... .......... .......... ..........
ECOR_57      ..G....... .......... A......... .....A.... .......... .......... ..........
UTI89        .......... .......... .......... .......... .......... .......... ..........
E._albertii  .CG..G.... .........G C..C..C..A ..G..A.... .T.AC....C G..A.....G G.......C.

          15611
K-12         AGCGAATACC TTCATTCAGG CGTGGCAGAC TGTTATTGAT TAAATTAGAG CTCGGTGCCC ACTGCGGGTT
53638        .......... .......... .......... .......... .......... .......... ..........
ATCC_8739    .......... .......... .......... .......... .......... .......... ..........
ECOR_10      .......... .......... .......... .......... .......... .......... ....T.....
ECOR_11      .......... .......... .......... .......... .......... .......... ..........
B171         .......... .......... .......... .......... .......... .......... ..........
E24377A      .......... .......... .......... .......... .......... .......... ..........
ECOR_34      .......... .......... .......... .......... .......... .......... ....T.....
ECOR_71      .......... .......... .......... .......... .......... .......... ....T.....
SE11         .......... .......... .......... .......... .......... .......... ..........
O157_Sakai   .A..G..... C......... .......... .......... .......... .......... ..........
ECOR_36      .......... .......... .......... .......... ......C..A .......... ....T.....
ECOR_35      .......... .......... .......... .......... ......C..A .......... ....T.....
SMS-3-5      .......... .......... .......... .......... ......C..A .......... ....T.....
536          .......... .......... .......... .......... .......... .....C.... ..........
APEC_O1      .......... .......... ..G....... .......... ......C... ..T....... ..........
CFT073       .......... .......... .......... .......... .......... .....C.... ..........
E2348/69     .......... .......... .......... .......... .......... .....C.... ..........
ECOR_51      .......... .......... .......... .......... .......... .....C.... ..........
ECOR_57      .......... .......... .......... .......... .......... .....C.... ..........
UTI89        .......... .......... ..G....... .......... ......C... ..T....... ..........
E._albertii  GA....CC.. C..G...... ..C....A.. .......A.. ......C... .CT....... ..........

          15681
K-12         CCAGGTATAA CCACCTCCGA GCACCACGAT ATAGTCCACT TTTTGCGAAT TATTCCATGT CGGATAGGTG
53638        .......... .......... .......... .......... .......... .......... ..........
ATCC_8739    .......... .......... .......... .......... .......... .......... ..........
ECOR_10      .......... .......... .......... .......... .......... .......... ..........
ECOR_11      .......... .......... .......... .......... .......... .......... ..........
B171         .......... .......... .......... .......... .......... .......... ..........
E24377A      .......... .......... .......... .......... .......... .......... ..........
ECOR_34      .......... .......... .......... .......... .......... .......... ..........
ECOR_71      .......... .......... .......... .......... .......... .......... ..........
SE11         .......... .......... .......... .......... .......... .......... ..........
O157_Sakai   .......... .......... .......... .......... .......... .......... ..........
ECOR_36      .......... ..G....... .......... .......... .......... .......... ..........
ECOR_35      .......... ..G....... .......... .......... .......... .......... ..........
SMS-3-5      .......... ..G....... .......... .......... .......... .......... ..........
536          .......... .......... .......... .......... .......... .......... ......A...
APEC_O1      .......... .......... .......... .......... .......... .......... ..........
CFT073       .......... .......... .......... .......... .......... .......... ......A...
E2348/69     ...A...... .......... .......... .......... .......... .......... ......A...
ECOR_51      .......... .......... .......... .......... .......... .......... ......A...
ECOR_57      .......... .......... .......... .......... .......... .......... ......A...
UTI89        .......... .......... .......... .......... .......... .......... ..........
E._albertii  .T.......G ..G..A..C. AT........ ........T. .......... .......... ..........
```

```
         15751
K-12          CTTTCGATGG GACGCAGCAG GCGGTCGGCT ACCGGTTGCA AACTCAATAA CAAGAGCGCC AGCCACCCAA
53638         .......... .......... .......... .......... .......... ......T... ..........
ATCC_8739     .......... .......... .......... .......... .......... ......T... ..........
ECOR_10       .......... .......... .......... .......... .......... ......T... ..........
ECOR_11       .......... .......... .......... .......... .......... .......... ..........
B171          .......... .......... .......... .......... .......... ......T... ..........
E24377A       .......... .......... .......... .......... .......... ......T... ..........
ECOR_34       .......... .......... .......... .......... .......... ......T... ..........
ECOR_71       .......... .......... .......... .......... .......... ......T... ..........
SE11          .......... .......... .......... .......... .......... ......T... ..........
O157_Sakai    .......... .......... .......... .......... .......... ......T... ..........
ECOR_36       .......... .......... .......... .......... .......... .......... ..........
ECOR_35       .......... .......... .......... .......... .......... .......... ..........
SMS-3-5       .......... .......... .......... .......... .......... .......... ..........
536           .......... ......A... .......... .T........ .......... .......... ..........
APEC_O1       .......... .......... .......... .......... .......... .......... ..........
CFT073        .......... ......A... .......... .T........ .......... .......... ..........
E2348/69      .......... ......A... .......... .T........ .......... .......... ..........
ECOR_51       .......... ......A... .......... .T........ .......... .......... ..........
ECOR_57       .......... ......A... .......... .T........ .......... .......... ..........
UTI89         .......... .......... .......... .......... .......... .......... ..........
E._albertii   .....T.... .G........ .TA....... .TG..C.... ....T..... G..A...... .....T....

         15821
K-12          TGCTAATAAA AATCTTCCCG GTTTTTTGAA AACGGCTAAA CCACAGCAGC GCCAGGCC-A GGCCAATAAT
53638         .......... .......... .......... .......... .......... .......... ..........
ATCC_8739     .......... .......... .......... .......... .......... .......... ..........
ECOR_10       .......... .......... .......... .......... .......... .......... ..........
ECOR_11       .......... .......... .......... .......... .......... .......... ..........
B171          .......... .......... .......... .......... .......... .......... ..........
E24377A       .......... .......... .......... .......... .......... .......... ..........
ECOR_34       .......... .......... .......... .......... .......... .......... ..........
ECOR_71       .......... .......... .......... .......... .......... .......... ..........
SE11          .......... .......... .......... .......... .......... .......... ..........
O157_Sakai    .......... .......... .......... .......... ......T... .......... ..........
ECOR_36       .......... .......... .......... .......... .......... .......... ..........
ECOR_35       .......... .......... .......... .......... .......... .......... ..........
SMS-3-5       .......... .......... .......... .......... .......... .......... ..........
536           .......... .......... .......... .......... .......... .......... ..........
APEC_O1       .......... .......... .......... .......... .......... .......... ..........
CFT073        .......... .......... .......... .......... .......... .......... ..........
E2348/69      .......... .......... .......... .......... .......... .......... ..........
ECOR_51       .......... .......... .......... .......... .......... .......... ..........
ECOR_57       .......... .......... .......... .......... .......... .......... ..........
UTI89         .......... .......... .......... .......... .......... .......... ..........
E._albertii   .......... T..T..T..A .......... ....A..... ...G..T... .....A..C. C...G.....

         15891
K-12          AA-CAACATC AGCGGAAGTG GCAGCAACAT ATTGCCAATC ACTTTTTTCA GTGTAAAAAG CATCCTTTCT
53638         .......... .......... .......... .......... .......... .......... ..........
ATCC_8739     .......... .......... .......... .......... .......... .......... ..........
ECOR_10       .......... .......... .......... .......... .......... .......... ..........
ECOR_11       .......... .......... .......... .......... .......... .......... ..........
B171          .......... .......... .......... .......... .......... .......... ..........
E24377A       .......... .......... .......... .......... .......... .......... ..........
ECOR_34       .......... .......... .......... .......... .......... .......... ..........
ECOR_71       .......... .......... .......... .......... .......... .......... ..........
SE11          .......... .......... .......... .......... .......... .......... ..........
O157_Sakai    .......... .......... .......... .......... .......... .......... ..........
ECOR_36       .......... .......... .......... .......... .......... .......... ..........
ECOR_35       .......... .......... .......... .......... .......... .......... ..........
SMS-3-5       .......... .......... .......... .......... .......... .......... ..........
536           .......... .......... .......... .......... .......... .......... ..........
APEC_O1       .......... .......... .......... .......... .......... .......... ..........
CFT073        .......... .......... .......... .......... .......... .......... ..........
E2348/69      .......... .......... .......... .......... .......... .......... ..........
ECOR_51       .......... .......... .......... .......... .......... .......... ..........
ECOR_57       .......... .......... .......... .......... .......... .......... ..........
UTI89         .......... .......... .......... .......... .......... .......... ..........
E._albertii   T.T..G.... .AT.....C. ....T.G... .CC....... .......... .......... .......C..
```

```
         15961
K-12            GGTTCCTTTT TTAACCATAT AGCAAGGGAT CGCCAGCGAT ATTACACCAG ACGGGTTCAT TCTCGCCGTG
53638           .......... .......... .......... .......... .......... .......... ..........
ATCC_8739       .......... .......... .......... .......... .......... .......... ..........
ECOR_10         .......... .......... .......... .......... .......... .......... ..........
ECOR_11         .......... .......... .......... .......... .......... .......... ..........
B171            .......... .......... .......... .......... .......... .......... ..........
E24377A         .......... .......... .......... .......... .......... .......... ..........
ECOR_34         .......... .......... .......... .......... .......... .......... ..........
ECOR_71         .......... .......... .......... .......... .......... .......... ..........
SE11            .......... .......... .......... .......... .......... .......... ..........
O157_Sakai      .......... .......... .......... .......... .......... .......... ..........
ECOR_36         .......... .......... .......... .......... .......... .......... ..........
ECOR_35         .......... .......... .......... .......... .......... .......... ..........
SMS-3-5         .......... .......... .......... .......... .......... .......... ..........
536             .......... ..G....... .......... .......... .......... .......... ..........
APEC_O1         .......... ..G....... .......... .......... .......... .......... ..........
CFT073          .......... ..G....... .......... .......... .......... .......... ..........
E2348/69        .......... ..G....... .......... .......... .......... .......... ..........
ECOR_51         .......... ..G....... .......... .......... .......... .......... ..........
ECOR_57         .......... ..G....... .......... .......... .......... .......... ..........
UTI89           .......... ..G....... .......... .......... .......... .......... ..........
E._albertii     .......... .......... ...G..A... ..A.C.T... .......... .......... ....AT..C.

         16031
K-12            GCTGTGACAA AATAGCGTCT TTATCAGAAA ACGTCTTAGC CAACCGGTGG AGAAAGCAAT GCAGGATCGC
53638           .......... .......... .......... .......... .......... .......... ..........
ATCC_8739       .......... .......... .......... .......... .......... .......... ..........
ECOR_10         .......... .......... .......... .......... .......... .......... ..........
ECOR_11         .......... .......... .......... .......... .......... .......... ..........
B171            .......... .......... .......... .......... .......... .......... ..........
E24377A         .......... .......... .......... .......... .......... .......... ..........
ECOR_34         .......... .......... .......... .......... .......... .......... ..........
ECOR_71         .......... .......... .......... .......... .......... .......... ..........
SE11            .......... .......... .......... .......... .......... .......... ..........
O157_Sakai      .......... .......... .......... .......... .......... .......... ..........
ECOR_36         .......... .......... .......... .......... .......... .......... ..........
ECOR_35         .......... .......... .......... .......... .......... .......... ..........
SMS-3-5         .......... .......... .......... .......... .......... .......... ..........
536             .......... .......... .......... .......... .......... .......... ..........
APEC_O1         .......... .......... .......... .......... .......... .......... ..........
CFT073          .......... .......... .......... .......... .......... .......... ..........
E2348/69        .......... .......... .......... .......... .......... .......... ..........
ECOR_51         .......... .......... .......... .......... .......... .......... ..........
ECOR_57         .......... .......... .......... .......... .......... .......... ..........
UTI89           .......... .......... .......... .......... .......... .......... ..........
E._albertii     .......... .......C.. .......... .......... .......... .......... ..........

         16101
K-12            AATTTTGATG ATATTGCGGA AAAGTTTTCC CGTAACATTT ACGGCACCAC CAAAGGGCAG CTTCGACAGG
53638           .......... .......... .......... .......... .......... .......... ..........
ATCC_8739       .......... .......... .......... .......... .......... .......... ..........
ECOR_10         .......... .......... .......... .......... .......... .......... ..........
ECOR_11         .......... .......... .......... .......... .......... .......... ..........
B171            .......... .......... .......... .......... .......... .......... ..........
E24377A         .......... .......... .......... .......... .......... .......... ..........
ECOR_34         .......... .......... .......... .......... .......... .......... ..........
ECOR_71         .....C.... .......... .......... .......... .......... .......... ..........
SE11            .......... .......... .......... .......... .......... .......... ..........
O157_Sakai      .......... .......... .......... .......... .......... .......... ..........
ECOR_36         .......... .......... .......... .......... .......... .......... ..........
ECOR_35         .......... .......... .......... .......... .......... .......... ..........
SMS-3-5         .......... .......... .......... .......... .......... .......... ..........
536             .......... .......... .......... .......... .......... .......... ..........
APEC_O1         .......... .......... .......... .......... .......... .......... ..........
CFT073          .......... .......... .......... .......... .......... .......... ..........
E2348/69        .......... .......... .......... .......... .......... .......... ..........
ECOR_51         .......... .......... .......... .......... .......... .......... ..........
ECOR_57         .......... .......... .......... .......... .......... .......... ..........
UTI89           .......... .......... .......... .......... .......... .......... ..........
E._albertii     ..C....... .......... .......... .......... .......... .......T.. ..........
```

```
         16171
K-12          CTATTCTGTG GCAGGATCTC GATCGCGTGC TGGCGGAAAT GGGCCCGCAA AAACTGCGTG TGCTGGATGC
53638         .......... .......... .......... .......... .......... .......... ..........
ATCC_8739     .......... .......... .......... .......... .......... .......... ..........
ECOR_10       .......... .......... ....A..... .......... .......... .......... ..........
ECOR_11       .......... .......... .......... .......... .......... .......... ..........
B171          .......... .......... .......... .......... .......... .......... ..........
E24377A       .......... .......... .......... .......... .......... .......... ..........
ECOR_34       .......... .......... ....A..... .......... .......... .......... ..........
ECOR_71       .......... .......... ....A..... .......... .......... .......... ..........
SE11          .......... .......... .......... .......... .......... .......... ..........
O157_Sakai    .......... .......... .......... .......... ...T...... .......... ..........
ECOR_36       .......... .......... .......... .......... .......... .......... ..........
ECOR_35       .......... .......... .......... .......... .......... .......... ..........
SMS-3-5       .......... .......... .......... .......... .......... .......... ..........
536           .......... .......... .......... .......... .......... .......... ..........
APEC_O1       .......... .......... .......... .......... .......... .......... ..........
CFT073        .......... .......... .......... .......... .......... .......... ..........
E2348/69      .......... .......... .......... .......... .......... .......... ..........
ECOR_51       .......... .......... .......... .......... .......... .......... ..........
ECOR_57       .......... .......... .......... .......... .......... .......... ..........
UTI89         .......... .......... .......... .......... .......... .......... ..........
E._albertii   .......... ...A...... ........A. ....A..T.. A..T.A.... .......... ..T.A.....

         16241
K-12          TGGCGGTGGA GAAGGGCAGA CCGCAATCAA AATGGCCGAG CGTGGGCATC AGGTCATTTT ATGCGATCTT
53638         .......... .......... .......... .......... .......... .......... ..........
ATCC_8739     .......... .......... .......... .......... .......... .......... ..........
ECOR_10       .........T .......... .......... .......... .......... .......... ..........
ECOR_11       .......... .......... .......... .......... .......... .......... ..........
B171          .......... .......... .......... .......... .......... .......... ..........
E24377A       .......... .......... .......... .......... .......... .......... ..........
ECOR_34       .........T .......... .......... .......... .......... .......... ..........
ECOR_71       .......... .......... .......... .......... .......... .......... ..........
SE11          .......... .......... .......... .......... .......... .......... ..........
O157_Sakai    G......... .......... .......... .......... ..C....... .......... ..........
ECOR_36       .......... .......... .......... ......T... ..C....... .......... ..........
ECOR_35       .......... .......... .......... ......T... ..C....... .......... ..........
SMS-3-5       .......... .......... .......... ......T... ..C....... .......... ..........
536           ......C... .......... .......... .......... .......... .......... ..........
APEC_O1       .......... .......... .......... .......... ..C....... .......... ..........
CFT073        ......C... .......... .......... .......... ..C....... .......... ..........
E2348/69      .......... .......... .......... .......... .......... .......... ..........
ECOR_51       ......C... .......... .......... .......... ..C....... .......... ..........
ECOR_57       ......C... .......... .......... .......... ..C....... .......... ..........
UTI89         .......... .......... .......... .......... ..C....... .......... ..........
E._albertii   C........T .....A.... ....G..... ......T... T.C..A.... .......... .........C

         16311
K-12          TCTGCGCAGA TGATCGACCG CGCAAAACAG GCGGCAGAAG CAAAAGGTGT GAGCGACAAC ATGCAATTTA
53638         .......... .......... .......... .......... .......... .......... ..........
ATCC_8739     .......... .......... .......... .......... .......... .......... ..........
ECOR_10       .......... .......... .......... .......... .......... .......... ..........
ECOR_11       .......... .......... .......... .......... .......... .......... ..........
B171          .......... .......... .......... .......... .......... .......... ..........
E24377A       .......... .......... .......... .......... .......... .......... ..........
ECOR_34       .......... .......... .......... .......... .......... .......... ..........
ECOR_71       .......... .......... .......... .......... .......... .......... ..........
SE11          .......... .......... .......... .......... .......... .......... ..........
O157_Sakai    .......... .......... .......... .......... .......... .......... ..........
ECOR_36       .......... .......... .......... .......... .......... .......... ..........
ECOR_35       .......... .......... .......... .......... .......... .......... ..........
SMS-3-5       .......... .......... .......... .......... .......... .......... ..........
536           .......... .......... .......... .......... .......... .......... ..........
APEC_O1       ....T..... .......... .......... .......... .......... .......... ..........
CFT073        .......... .......... .......... .......... .......... .......... ..........
E2348/69      .......... .......... .......... .......... .......... .......... ..........
ECOR_51       .......... .......... .......... .......... .......... .......... ..........
ECOR_57       .......... .......... .......... .......... .......... .......... ..........
UTI89         ....T..... .......... .......... .......... .......... .......... ..........
E._albertii   ..G.A..... ....T.GT.. .......... .....T..G. .......... .......... ..........
```

```
         16381
K-12          TACATTGCGC CGCTCAGGAT GTTGCTTCGC ATTTGGAAAC GCCCGTTGAT CTGATATTGT TCCATGCGGT
53638         .......... .......... .......... .......... .......... .......... ..........
ATCC_8739     .......... .......... .......... .......... .......... .......... ..........
ECOR_10       .......... .......... .......... .......... .......... .......... ..........
ECOR_11       .......... .......... .......... .......... .......... .......... ..........
B171          .......... .......... .......... .......... .......... .......... ..........
E24377A       .......... .......... .......... .......... .......... .......... ..........
ECOR_34       .......... .......... .......... .......... .......... .......... ..........
ECOR_71       .......... .......... .......... .......... .......... .......... ..........
SE11          .......... .......... .......... .......... .......... .......... ..........
O157_Sakai    .......... .......... .......... .......... .......... .......... ..........
ECOR_36       .......... .......... .......... .......... .......... .......... ..........
ECOR_35       .......... .......... .......... .......... .......... .......... ..........
SMS-3-5       .......... .......... .......... .......... .......... .......... ..........
536           .......... .......... .......... .......... .......... .......... ..........
APEC_O1       .......... .......... .......... .......... .......... .......... ..........
CFT073        .......... .......... .......... .......... .......... .......... ..........
E2348/69      .......... .......... .......... .......... .......... .......... ..........
ECOR_51       .......... .......... .......... .......... .......... .......... ..........
ECOR_57       .......... .......... .......... .......... .......... .......... ..........
UTI89         .......... .......... .......... .......... .......... .......... ..........
E._albertii   .......... .........C ........A. .......... .......... .......... ..........

         16451
K-12          GCTCGAGTGG GTGGCTGATC CCCGCAGCGT ATTGCAGACC CTCTGGTCAG TGTTGCGTCC AGGCGGCGTG
53638         .......... .....C.... .......T.. .......... ..G....... .......... ..........
ATCC_8739     .......... .....C.... .......T.. .......... ..G....... .......... ..........
ECOR_10       .......... .....C.... .......T.. .......... ..G....... .......... ..........
ECOR_11       .......... .......... .......... .......... ..G....... .......... ..........
B171          .......... .....C.... .......T.. .......... ..G....... .......... ..........
E24377A       .......... .....C.... .......T.. .......... ..G....... .......... ..........
ECOR_34       .......... .....C.... .......T.. .......... ..G....... .......... ..........
ECOR_71       .......... .....C.... .......T.. .......... ..G....... .......... ......A...
SE11          .......... .....C.... .......T.. .......... ..G....... .......... ..........
O157_Sakai    .......... .......... .......... .......... ..G....... .......... ..........
ECOR_36       .......... .....C.... .......... ...A...... ..G....... .......... ..........
ECOR_35       .......... .....C.... .......... ...A...... ..G....... .......... ......T...
SMS-3-5       .......... .....C.... .......... ...A...... ..G....... .......... ..........
536           .......... .....C.... .......... ...A...... ..G....... .......... .........A
APEC_O1       .......... .....C.... .......... ...A...... ..G....... .......... ..........
CFT073        .......... .....C.... .......... ...A...... ..G....... .......... .........A
E2348/69      ...G...... .....C.... .......... .......... ..G....... .......... ..........
ECOR_51       .......... .....C.... .......... ...A...... ..G....... .......... .........A
ECOR_57       .......... .....C.... .......... ...A...... ..G....... .......... .........A
UTI89         .......... .....C.... .......... ...A...... ..G....... .......... ..........
E._albertii   ...G..A... ..A..C.... ....TG.... ...A..A..A ..G....... ....A..C.. G.........

         16521
K-12          TTGTCGTTAA TGTTCTACAA TGCGCATGGT TTGTTGATGC ATAACATGGT CGCCGGGAAT TTTGATTACG
53638         .......... .......... .......... .......... .......... .......... ..........
ATCC_8739     .......... .......... .......... .......... .......... .......... ..........
ECOR_10       .......... .......... .......... .......... .......... .......... ..........
ECOR_11       .......... .......... .......... .......... .......... .......... ..........
B171          .......... .......... .......... .......... .......... .......... ..........
E24377A       .......... .......... .......... .......... .......... .......... ..........
ECOR_34       .......... .......... .......... .......... .......... .......... ..........
ECOR_71       .......... .......... .......... .......... .......... .......... ..........
SE11          .......... .......... .......... .......... .......... .......... ..........
O157_Sakai    .......... .......... .......... .......... .......... .......... ..........
ECOR_36       .......... .......... ......C..C ...C...... .......... .......... ..........
ECOR_35       .......... .......... ......C..C ...C...... .......... .......... ..........
SMS-3-5       .......... .......... ......C..C ...C...... .......... .......... ..........
536           .......... .......... .......... .......... .......... .......... ..........
APEC_O1       .......... .......... .......... .......... .......... .......... ..........
CFT073        .......... .......... .......... .......... .......... .......... ..........
E2348/69      .......... .......... .......... .......... .......... .......... ..........
ECOR_51       .......... .......... .......... .......... .......... .......... ..........
ECOR_57       .......... .......... .......... .......... .......... .......... ..........
UTI89         .......... .......... .......... .......... .......... .......... ..........
E._albertii   .......... .......... .........C ...C...... .C........ T..A...... ..........
```

```
        16591
K-12          TGCAGGCGGG AATGCCGAAA AAGAAAAAAC GGACGCTTTC GCCAGATTAT CCACGCGACC CGGCGCAGGT
53638         .......... .......... .......... .......... .......... .......... ..........
ATCC_8739     .......... .......... .......... .......... .......... .......... ..........
ECOR_10       .......... .......... .......... .......... .......... .......... ..........
ECOR_11       .......... .......... .......... .......... .......... .......... ..........
B171          .......... .......... .......... .......... .......... .......... ..........
E24377A       .......... .......... .......... .......... .......... .......... ..........
ECOR_34       .......... .......... .......... .......... .......... .......... ..........
ECOR_71       .......... .......... .......... .......... .......... .......... ..........
SE11          .......... .......... .......... .......... .......... .......... ..........
O157_Sakai    .......... .......... .......... .......... .......... .......... ..A.......
ECOR_36       .......... .......... .......... .......... .......... .......... ..........
ECOR_35       .......... .......... .......... .......... .......... .......... ..........
SMS-3-5       .......... .......... .......... .......... .......... .......... ..........
536           .......... .......... .......... .......... .......... .......... ..........
APEC_O1       .......... .......... .......... .......... .......... .......... ..........
CFT073        .......... .......... .......... .......... .......... .......... ..........
E2348/69      .......... .......... .......... .......... .......... .......... ..........
ECOR_51       .......... .......... .......... .......... .......... .......... ..........
ECOR_57       .......... .......... .......... .......... .......... .......... ..........
UTI89         .......... .......... .......... ...T...... .......... .......... ..........
E._albertii   .......... .......... .......... .......... ......C... ........T. ..........

        16661
K-12          TTATCTGTGG CTGGAAGAAG CTGGTTGGCA AATTATGGGT AAGACAGGCG TTCGCGTGTT TCATGATTAT
53638         .......... .......... .......... .......... .......... .......... ..........
ATCC_8739     .......... .......... .......... .......... .......... .......... ..........
ECOR_10       .......... .......... .......... .......... .......... .......... ..........
ECOR_11       .......... .......... G......... .......... .......... .......... ..........
B171          .......... .......... .......... .......... .......... .......... ..........
E24377A       .......... .......... .......... .......... .......... .......... ..........
ECOR_34       .......... .......... .......... .......... .......... .......... ..........
ECOR_71       .......... .......... .......... .......... .......... .......... ..........
SE11          .......... .......... .......... .......... .......... .......... ..........
O157_Sakai    .......... .......... .......... .......... .......... .......... ..........
ECOR_36       .......... .......... .......... .......... .......... ....T..... ..........
ECOR_35       .......... .......... .......... .......... .......... ....T..... ..........
SMS-3-5       .......... .......... .......... .......... .......... ....T..... ..........
536           .......... .......... .......... .......... .......... .......... ..........
APEC_O1       .......... .......... .......... .......... .......... .......... ..........
CFT073        .......... .......... .......... ....C..... .......... .......... ..........
E2348/69      .......... .......... .......... ....C..... .......... .......... ..........
ECOR_51       .......... .......... .......... ....C..... .......... .......... ..........
ECOR_57       .......... .......... .......... ....C..... .......... .......... ..........
UTI89         .......... .......... .......... .......... .......... .......... ..........
E._albertii   .......... .......... ....C..... .....CA... ..A....... .......... ..........

        16731
K-12          CTGCGCGAGA AACACCAGCA GCGCGACTGC TATGAAGCAT TACTTGAATT AGAAACGCGT TATTGCCGTC
53638         .......... .......... .......... .......... .......... .......... ..........
ATCC_8739     .......... .......... .......... .......... .......... .......... ..........
ECOR_10       .......... .G........ .......... .......... .......... .......... ..........
ECOR_11       .......... .......... .......... .......... .......... .......... ..........
B171          .......... .G........ .......... .......... .......... .......... ..........
E24377A       .......... .G........ .......... .......... .......... .......... ..........
ECOR_34       .......... .G........ .......... .......... .......... .......... ..........
ECOR_71       .......... .G........ .......... .......... .......... .......... ..........
SE11          .......... .G........ .......... .......... .......... .......... ..........
O157_Sakai    .......... .G........ .......... .......... .......... .......... ..........
ECOR_36       .......... .G........ .......... .......... .......... .......... ..........
ECOR_35       .......... .G........ .......... .......... .......... .......... ..........
SMS-3-5       .......... .G........ .......... .......... .......... .......... ..........
536           .......... .G........ .......... .......... .......... .......... ..........
APEC_O1       .......... .G........ .......... .......... .......... .......... ..........
CFT073        .......... .G........ .......... .......... .......... .......... ..........
E2348/69      .......... .G..T..... .......... .......... .......... .......... ..........
ECOR_51       .......... .G........ .......... .......... .......... .......... ..........
ECOR_57       .......... .G........ .......... .......... .......... .......... ..........
UTI89         .......... .G........ .......... .......... .......... .......... ..........
E._albertii   .....T..A. .G.....A.. ......T... ..C....... .......... ........A. ........C.
```

```
         16801
K-12          AGGAACCGTA TATTACCCTG GGGCGTTATA TTCATGTCAC CGCGCGCAAA CCGCAGAGCA AGGATAAAGT
53638         .......... .......... .......... .......... .......... .......... ..........
ATCC_8739     .......... .......... .......... .......... .......... .......... ..........
ECOR_10       .......... .......... .......... .......... .......... .......... ..........
ECOR_11       .......... .......... .......... .......... .......... .......... ..........
B171          .......... .......... .......... .......... .......... .......... ..........
E24377A       .......... .......... .......... .......... .......... .......... ..........
ECOR_34       .......... .......... .......... .......... .......... .......... ..........
ECOR_71       .......... .......... .......... .......... .......... .......... ..........
SE11          .......... .......... .......... .......... .......... .......... ..........
O157_Sakai    .......... .......... .......... .......... .......... .......... ..........
ECOR_36       .......... .......... .......... .......... .......... .......... ..........
ECOR_35       .......... .......... .......... .......... .......... .......... ..........
SMS-3-5       .......... .......... .......... .......... .......... .......... ..........
536           .......... .......... .......... .......... .......... .......... ..........
APEC_O1       .......... .......... .......... .......... .......... .......... ..........
CFT073        .......... .......T.. .......... .......... .......... .......... ..........
E2348/69      .......... .......... .......... .......... .......... .......... ..........
ECOR_51       .......... .......... .......... .......... .......... .......... ..........
ECOR_57       .......... .......T.. .......... .......... .......... .......... ..........
UTI89         .......... .......... .......... .......... .......... .......... ..........
E._albertii   .......... .....A.... .......... .......... .......... .......... ..........

         16871
K-12          ATGAGTGAAT TTTCCCAGAC AGTCCCCGAA CTGGTTGCCT GGGCCAGAAA AAATGACTTC TCCATCTCGC
53638         .......... .......... .......... .......... .......... .......... ..........
ATCC_8739     .......... .......... .......... .......... .......... .......... ..........
ECOR_10       .......... .......... .......... .......... .......... .......... ..........
ECOR_11       .......... .......... .......... .......... .......... .......... ..........
B171          .......... .......... .......... .......... .......... .......... ..........
E24377A       .......... .......... .......... .......... .......... .......... ..........
ECOR_34       .......... .......... .......... .......... .......... .......... ..........
ECOR_71       .......... .......... .......... .......... .......... .......... ..........
SE11          .......... .......... .......... .......... .......... .......... ..........
O157_Sakai    .......... .......... .......... .......... .......... .......... .....T....
ECOR_36       .......... .......... .......... .......... .......... .......... ..........
ECOR_35       .......... .......... .......... .......... .......... .......... ..........
SMS-3-5       .......... .......... .......... .......... .......... .......... ..........
536           .......... .......... .......... .......... .......... .......... ..........
APEC_O1       .......... .......... .......... .......... .......... .......... ..........
CFT073        .......... .......... .......... .......... .......... .......... ..........
E2348/69      .......... .......... .......... .......... .......... .......... ..........
ECOR_51       .......... .......... .......... .......... .......... .......... ..........
ECOR_57       .......... .......... .......... .......... .......... .......... ..........
UTI89         .......... .......... .......... .......... .......... .......... ..........
E._albertii   .......... .......... .......... .......... .......... .......... .........T

         16941
K-12          TGCCGGTAGA CCGACTCTCT TTTCTGCTGG CGGTTGCCAC GCTGAACGGC GAGCGTCTGG ATGGTGAGAT
53638         .......... .......... .......... .......... .......... .......... ..........
ATCC_8739     .......... .......... .......... .......... .......... .......... ..........
ECOR_10       .......... .......... .......... .......... .......... .......... ..........
ECOR_11       .......... .......... .......... .......... .......... .......... ..........
B171          .......... .......... .......... .......... .......... .......... ..........
E24377A       .......... .......... .......... .......... .......... .......... ..........
ECOR_34       .......... .......... .......... .......... .......... .......... ..........
ECOR_71       .......... .......... .......... .......... .......... .......... ..........
SE11          .......... .......... .......... .......... .......... .......... ..........
O157_Sakai    .......... .......... .......... .A........ .......... .......... ..........
ECOR_36       .......... .......... .......... .......... .........A .......... ..........
ECOR_35       .......... .......... .......... .......... .........A .......... ..........
SMS-3-5       .......... .......... .......... .......... .......... .......... ..........
536           .......... .......... .......... .......... .......... .......... ..........
APEC_O1       .......... .......... .......... .......... .......... .......... ..........
CFT073        .......... .......... .......... .......... .......... .......... ..........
E2348/69      .......... .......... .......... .......... .......... .......... ..........
ECOR_51       .......... .......... .......... .......... .......... .......... ..........
ECOR_57       .......... .......... .......... .......... .......... .......... ..........
UTI89         .......... .......... .......... .......... .......... .......... ..........
E._albertii   .......... .........C .......... .......... .......... .......... .C........
```

```
          17011
K-12          GAGTGAAGGC GAGCTGGTGG ATGCATTCCG CCATGTGAGT GATGCGTTTG AGCAAACCAG CGAAACCATC
53638         .......... .......... .......... .......... .......... .......... ..........
ATCC_8739     .......... .......... .......... .......... .......... .......... ..........
ECOR_10       .......... .......... .......... .......... .......... .......... ..........
ECOR_11       .......... .......... .......... .......... .......... .......... ..........
B171          .......... .......... .......... .......... .......... .......... ..........
E24377A       .......... .......... .......... .......... .......... .......... ..........
ECOR_34       .......... .......... .......... .......... .......... .......... ..........
ECOR_71       .......... .......... .......... .......... .......... .......... ..........
SE11          .......... .......... .......... .......... .......... .......... ..........
O157_Sakai    .......... .......... .......... .......... .......... .......... ..........
ECOR_36       .......... .......... .......... .......... .......... .......... ..........
ECOR_35       .......... .......... .......... .......... .......... .......... ..........
SMS-3-5       .......... .......... .......... .......... .......... .......... ..........
536           .......... .......... .......... .......... .......... .......... ..........
APEC_O1       .......... .......... .......... .......... .......... .......... ..........
CFT073        .......... .......... .......... .......... .......... .......... ..........
E2348/69      .......... .......... .......... .......... .......... .......... ..........
ECOR_51       .......... .......... .......... .......... .......... .......... ..........
ECOR_57       .......... .......... .......... .......... .......... .......... ..........
UTI89         .......... .......... .......... .......... .......... .......... ..........
E._albertii   ...C...... .......... .......... .......... .......... .......... ..........

          17081
K-12          GGCGTGCGCG CCAATAACGC GATCAACGAC ATGGTGCGTC AACGTCTGCT GAACCGCTTT ACCAGCGAGC
53638         .......... .......... .......... .......... .......... .......... ..........
ATCC_8739     .......... .......... .......... .......... .......... .......... ..........
ECOR_10       .......... .......... .......... .......... .......... .......... ..........
ECOR_11       .......... .......... .......... .......... .......... .......... ..........
B171          .......... .......... .......... .......... .......... .......... ..........
E24377A       .......... .......... .......... .......... .......... .......... ..........
ECOR_34       .......... .......... .......... .......... .......... .......... ..........
ECOR_71       .......... ....C..... .......... .......... .......... .......... ..........
SE11          .......... .......... .......... .......... .......... .......... ..........
O157_Sakai    .......... ....C..... .......... .......... .......... .......... ..........
ECOR_36       ........T. ....C..... .......... .......... .......... .......... ..........
ECOR_35       ........T. ....C..... .......... .......... .......... .......... ..........
SMS-3-5       ........T. ....C..... .......... .......... .......... .......... ..........
536           .......... ....C..... .......... .......... .......... .......... ..........
APEC_O1       .......... ....C..... .......... .......... .......... .......... ..........
CFT073        .......... ....C..... .......... .......... .......... .......... ..........
E2348/69      .......... ....C..... .......... .......... .......... .......... ..........
ECOR_51       .......... ....C..... .......... .......... .......... .......... ..........
ECOR_57       .......... ....C..... .......... .......... .......... .......... ..........
UTI89         .......... ....C..... .......... .......... .......... .......... ..........
E._albertii   ..T....... ....C..... .......... .......... .......... .......... ........A.

          17151
K-12          AGGCGGAAGG GAACGCAATT TACCGTCTGA CGCCGCTCGG CATCGGCATT ACTGACTACT ACATCCGTCA
53638         .......... .......... .......... .......... .......... .......... ..........
ATCC_8739     .......... .......... .......... .......... .......... .......... ..........
ECOR_10       .......... .......... .......... .......... .......... .......... ..........
ECOR_11       .......... .......... .......... .......... .......... .......... ..........
B171          .......... .......... .......... .......... .......... .......... ..........
E24377A       .......... .......... .......... .......... .......... .......... ..........
ECOR_34       .......... .......... .......... .......... .......... .......... ..........
ECOR_71       .......... .......... .......... .......... .........C ........T. ....T.....
SE11          .......... .......... .......... .......... .......... .......... ..........
O157_Sakai    ....A..... .......... .......... .......... .......... ........T. ..........
ECOR_36       .......... .......... .......... .......... .......... ........T. ..........
ECOR_35       .......... .......... .......... .......... .......... ........T. ..........
SMS-3-5       .......... .......... .......... .......... .......... ........T. ..........
536           .......... ......G... .......... .......... .........C ........T. ....T.....
APEC_O1       .......... .......... .......... .......... .........C ........T. ....T.....
CFT073        .......... .......... .......... .......... .........C ........T. ....T.....
E2348/69      .......... .......... .......... .......... .........C ........T. ....T.....
ECOR_51       .......... .......... .......... .......... .........C ........T. ....T.....
ECOR_57       .......... .......... .......... .......... .........C ........T. ....T.....
UTI89         .......... .......... .......... .......... .........C ........T. ....T.....
E._albertii   .......... ......C... .......... .......... ...T...... ..G..T.... ....T.....
```

```
          17221
K-12         GCGCGAGTTT TCTACGCTGC GTCTTTCTAT GCAGTTGTCG ATTGTGGCGG GTGAGCTCAA ACGCGCAGCA
53638        .......... .......... .......... .......... .......... .......... ..........
ATCC_8739    .......... .......... .......... .......... .......... .......... ..........
ECOR_10      .......... .......... .......... .......... .......... .......... ..........
ECOR_11      .......... .......... .......... .......... .......... .......... ..........
B171         .......... .......... .......... .......... .......... ....A..... .........T
E24377A      .......... .......... .......... .......... .......... .......... ..........
ECOR_34      .......... .......... .......... .......... .......... .......... ..........
ECOR_71      .......... .......... .C........ .......... .......... .......... ..........
SE11         .......... .......... .......... .......... .......... .......... ..........
O157_Sakai   .......... .......... .C........ .......... .......... .......... ..........
ECOR_36      .......... .......... .C........ .......... .......... .......... ......G...
ECOR_35      .......... .......... .C........ .......... .......... .......... ......G...
SMS-3-5      .......... .......... .C........ .......... .......... .......... ......G...
536          .......... .......... .C........ ...A...... .......... .......... G.........
APEC_O1      .......... .......... .C........ ...A...... .......... .......... G.........
CFT073       .......... .......... .C........ ...A...... .......... .......... G.........
E2348/69     .......... .......... .C........ .......... .......... .......... ..........
ECOR_51      .......... .......... .C........ ...A...... .......... .......... G.........
ECOR_57      .......... .......... .C........ ...A...... .......... .......... G.........
UTI89        .......... .......... .C........ ...A...... .......... .......... G.........
E._albertii  .......... .......... .C........ .......... ..C..T..A. .C..A..G.. G..T.....G

          17291
K-12         GATGCCGCCG AAGAGGGCGG TGATGAATTT CACTGGCACC GTAATGTCTA TGCGCCACTG AAATATTCGG
53638        .......... .......... .......... .......... .......... .......... ..........
ATCC_8739    .......... .......... .......... .......... .......... .......... ..........
ECOR_10      .......... .......... .......... .......... .......... .......... ..........
ECOR_11      .......... .......... .......... .......... .......... .......... ..........
B171         .......... .......... .......... .......... .......... .......... ..........
E24377A      .......... .......... .......... .......... .......... .......... ..........
ECOR_34      .......... .......... .......... .......... .......... .......... ..........
ECOR_71      .......... .......... .......... .......... .......T.. ...T...... ..........
SE11         .......... .......... .......... .......... .......... .......... ..........
O157_Sakai   .......... .......... .......... .......... .......... .......... ..........
ECOR_36      .......... .......... .......... .......... .......T.. .......... ..........
ECOR_35      .......... .......... .......... .......... .......T.. .......... ..........
SMS-3-5      .......... .......... .......... .......... .......T.. .......... ..........
536          .....T.... .......... .......... .......... .......T.. .......... ..........
APEC_O1      .......... .......... .......... .......... .......T.. .......... ..........
CFT073       .......... .......... .......... .......... .......T.. .......... ..........
E2348/69     .......... .......... .......... .......... .......T.. .......... ..........
ECOR_51      .......... .......... .......... .......... .......T.. .......... ..........
ECOR_57      .......... .......... .......... .......... .......T.. .......... ..........
UTI89        .......... .......... .......... .......... .......T.. .......... ..........
E._albertii  ........A. .......... ......G... ........T. ....C..T.. ......G... ..........

          17361
K-12         TAGCAGAAAT TTTCGACAGT ATCGACCTGA CGCAACGTCT GATGGACGAA CAGCAGCAGC AGGTGAAGGA
53638        .......... .......... .......... .......... .......... .......... ..........
ATCC_8739    .......... .......... .......... .......... .......... .......... ..........
ECOR_10      .......... .......... .......... .......... .......... .......... ..........
ECOR_11      .......... .......... .......... .......... .......... .......... ..........
B171         .......... .......... .......... .......... .......... .......... ..........
E24377A      .......... .......... .......... .......... .......... .......... ..........
ECOR_34      .......... .......... .......... .......... .......... .......... ..........
ECOR_71      .......... .......... .......... .......... ......T... .......... ..........
SE11         .......... .......... .......... .......... .......... .......... ..........
O157_Sakai   .......... .......... .......... .......... .......... .......... ..........
ECOR_36      .......... .......... .......... .......... .......... .......... ..........
ECOR_35      .......... .......... .......... .......... .......... .......... ..........
SMS-3-5      .......... .......... .......... .......... .......... .......... ..........
536          .......... .......... .......... .......... ......T... .......... ..........
APEC_O1      .......... .......... .......... .......... ......T... .......... ..........
CFT073       .......... .......... .......... .......... ......T... .......... ..........
E2348/69     .......... .......... .......... .......... ......T... .......... ..........
ECOR_51      .......... .......... .......... .......... ......T... .......... ..........
ECOR_57      .......... .......... .......... .......... ......T... .......... ..........
UTI89        .......... .......... .......... .......... ......T... .......... ..........
E._albertii  .G..G..... .......... .......... .......... .......... .......... .......A..
```

```
        17431
K-12         CGATATCGCC CAGTTGCTGA ACAAAGACTG GCGGGCGGCG ATTTCCAGCT GTGAATTGTT GCTTTCGGAA
53638        .......... .......... .......... .......... .......... .......... ..........
ATCC_8739    .......... .......... .......... .......... .......... .......... ..........
ECOR_10      .......... .......... .......... .......... .......... .......... ..........
ECOR_11      .......... .......... .......... .......... .......... .......... ..........
B171         ......T... .......... .......... .......... .......... .......... ......A...
E24377A      .......... .......... .......... .......... .......... .......... ..........
ECOR_34      .......... .......... .......... .......... .......... .......... ..........
ECOR_71      T......... .......... .......... .......... .......... .......... ..........
SE11         .......... .......... .......... .......... .......... .......... ..........
O157_Sakai   .......... .......... .......... .......... .......... .......... ..........
ECOR_36      .......... .......... .......... .......... ..C....... .......... ..........
ECOR_35      .......... .......... .......... .......... ..C....... .......... ..........
SMS-3-5      .......... .......... .......... .......... ..C....... .......... ..........
536          T......... ......T... .......... ...T...... .......... .......... ......A...
APEC_O1      T......... ......T... .......... .......... .......... .......... ......A...
CFT073       T......... ......T... .......... .......... .......... .......... ......A...
E2348/69     T......... ......T... .......... .......... .......... .......... ......A...
ECOR_51      T......... ......T... .......... .......... .......... .......... ......A...
ECOR_57      T......... ......T... .......... .......... .......... .......... ......A...
UTI89        T......... ......T... .......... .......... .......... .......... ......A...
E._albertii  ...C.....T .......... .T........ ...A..A... .......... .......... ..........

        17501
K-12         ACTTCCGGAA CGCTGCGTGA ATTGCAGGAT ACGCTGGAAG CGGCAGGCGA CAAATTGCAG GCTAATCTGT
53638        .......... .......... .......... .......... .......... .......... ..........
ATCC_8739    .......... .......... .......... .......... .......... .......... ..........
ECOR_10      ........G. .......... .......... .......... .......... .......... ..........
ECOR_11      .......... .......... .......... .......... .......... .......... ..........
B171         .......... .......... .......... .......... .......... ...G...... ..........
E24377A      .......... .......... .......... .......... .......... .......... ..........
ECOR_34      ........G. .......... .......... .......... .......... .......... ..........
ECOR_71      .......... .......... .......... .......... .......... .......... ..........
SE11         .......... .......... .......... .......... .......... .......... ..........
O157_Sakai   .......... .......... .......... ..A....... .......... .......... ..........
ECOR_36      .......... .......... .......... .......... .......... .......... ..........
ECOR_35      .......... .......... .......... .......... .......... .......... ..........
SMS-3-5      .......... .......... .......... .......... .......... .......... ..........
536          .......... .......... .......... .......... .......... T......... ..C..C....
APEC_O1      .......... .......... .......... .......... .......... T......... ..C..C....
CFT073       .......... .......... .......... .......... .......... T......... ..C..C....
E2348/69     ........C. .......... .......... .......... .......... T......... ..C..C....
ECOR_51      .......... .......... .......... .......... .......... T......... ..C..C....
ECOR_57      .......... .......... .......... .......... .......... T......... ..C..C....
UTI89        .......... .......... .......... .......... .......... T......... ..C..C....
E._albertii  ..C..A.... .......... .......... ..A....... .......... .......... ..C..C....

        17571
K-12         TGCGCATTCA GGATGCGACG ATGACCCATG ACGATCTGCA TTTCGTCGAT CGTCTGGTGT TCGATCTGCA
53638        .......... .......... .......... .......... .......... .......... ..........
ATCC_8739    .......... .......... .......... .......... .......... .......... ..........
ECOR_10      .......... .......... .......... .......... .......... .......... ..........
ECOR_11      .......... .......... .......... .......... .......... .......... ..........
B171         .......... .......... .......... ....C..... ...T...... .......... ..........
E24377A      .......... .......... .......... .......... .......... .......... ..........
ECOR_34      .......... .......... .......... .......... .......... .......... ..........
ECOR_71      .......... .......... .......... .......... .......... .......... ..........
SE11         .......... .......... .......... .......... .......... .......... ..........
O157_Sakai   .......... .......... .....T.... .......... .......... .......... ..........
ECOR_36      .......... .......... .......... .......... ...T...... .......... ..........
ECOR_35      .......... .......... .......... .......... ...T...... .......... ..........
SMS-3-5      .......... .......... .......... .......... ...T...... .......... ..........
536          .A........ .......... .......... ....C..... ...T...... .......... .......T..
APEC_O1      .A........ .......... .......... ....C..... ...T...... .......... .......T..
CFT073       .A........ .......... .......... ....C..... ...T...... .......... .......T..
E2348/69     .A........ .......... ....T..... .......... ...T...... ........A. ..........
ECOR_51      .A........ .......... .......... ....C..... ...T...... .......... .......T..
ECOR_57      .A........ .......... .......... ....C..... ...T...... .......... .......T..
UTI89        .A........ .......... .......... ....C..... ...T...... .......... .......T..
E._albertii  .A........ ...A..C..C .......... .T.....A.. ...T...... ........A. ..........
```

```
        17641
K-12         GAGCAAACTC GATCGTATTA TCAGTTGGGG CCAGCAATCC ATCGACTTGT GGATTGGCTA CGACCGCCAC
53638        .......... .......... .......... .......... .......... .......... ..........
ATCC_8739    .......... .......... ..T....... .......... .......... .......... ..........
ECOR_10      .......... .......... .......... .......... .......... .......... ..........
ECOR_11      .......... .......... .......... .......... .......... .......... ..........
B171         .......... ..C....... ....C..... .......... .......... .......... ...T......
E24377A      .......... .......... .......... .......... .......... .......... ..........
ECOR_34      .......... .......... .......... .......... .......... .......... ..........
ECOR_71      .......... .......... .......... .......... .......... .......... ..........
SE11         .......... .......... .......... .......... .......... .......... ..........
O157_Sakai   .......... ..C....... .......... .......... .....T.... .......... ..........
ECOR_36      .........T ..C....... .......... .......... .......... .......... ..........
ECOR_35      .........T ..C....... .......... .......... .......... .......... ..........
SMS-3-5      .........T ..C....... .......... .......... .......... .......... ..........
536          .......... ..C....... .......... .......... .......... .......... ..........
APEC_O1      .......... ..C....... .......... .......... .......... .......... ..........
CFT073       .......... ..C....... .......... .......... .......... .......... ..........
E2348/69     .........T ..C....... ....C..... .......... .......... .......... ..........
ECOR_51      .......... ..C....... .......... .......... .......... .......... ..........
ECOR_57      .......... ..C....... .......... .......... .......... .......... ..........
UTI89        .......... ..C....... .......... .......... .......... .......... ..........
E._albertii  A.....G... .......... .......... .......... .......... ....C..... T.........
```

```
        17711
K-12         GTACACAAAT TTATTCGTAC CGCGATCGAT ATGGATAAAA ACCGCGTCTT TGCTCAGCGG TTACGTCAGT
53638        .......... .......... .......... .......... .......... .......... ..........
ATCC_8739    .......... .......... .......... .......... .......... .......... ..........
ECOR_10      .......... .......... .......... .......... .......... .......... ..........
ECOR_11      .......... .......... .......... .......... .......... .......... ..........
B171         .......... .......... T......... .......... .......... ...C.....T ..........
E24377A      .......... .......... .......... .......... .......... .......... ..........
ECOR_34      .......... .......... .......... .......... .......... .......... ..........
ECOR_71      .......... .......... .......... .......... .......... .......... ..........
SE11         .......... .......... .......... .......... .......... .......... ..........
O157_Sakai   .....T.... .......... .......... .......... .......... .......... ..........
ECOR_36      .......... ....C..... ...A...... .......... .......... .......... ..........
ECOR_35      .......... ....C..... ...A...... .......... .......... .......... ..........
SMS-3-5      .......... ....C..... ...A...... .......... .......... .......... ..........
536          .......... .......... T......... .......... .......... .......... ..........
APEC_O1      .......... .......... T......... .......... .......... .......... ..........
CFT073       .......... .......... T......... .......... .......... .......... ..........
E2348/69     .......... .......... T......... .......... .......... .......... ..........
ECOR_51      .......... .......... T......... .......... .......... .......... ..........
ECOR_57      .......... .......... T......... .......... .......... .......... ..........
UTI89        .......... .......... T......... .......... .......... .......... ..........
E._albertii  .....T.... ....C..... T..T...... .......... .......... ...C.....A ..G.......
```

```
        17781
K-12         CGGTACAAAC CTATTTTGAT GAGCCGTGGG CGCTAACTTA TGCCAATGCC GATCGTCTGC TGGATATGCG
53638        .......... .......... .......... .......... .......... .......... ..........
ATCC_8739    .......... .......... .......... .......... .......... .......... ..........
ECOR_10      .......... .......... .......... .......... .......... .......... ..........
ECOR_11      .......... .......... .......... .......... .......... .......... ..........
B171         .......... .......... .......... .......... .......... .......... ..........
E24377A      .......... .......... .......... .......... .......... .......... ..........
ECOR_34      .......... .......... .......... .......... .......... .......... ..........
ECOR_71      .......... .......... .......... .......... .......... .......... ..........
SE11         .......... .......... .......... .......... .......... .......... ..........
O157_Sakai   .......... .......... .......... ......G... .......... .......... ..........
ECOR_36      .......... .......... .......... .......... .......... .......... ..........
ECOR_35      .......... .......... .......... .......... .......... .......... ..........
SMS-3-5      .......... .......... .......... .......... .......... .......... ..........
536          .......... .......... .......... .......... .......... .......... ..........
APEC_O1      .......... .......... .......... .......... .......... .......... ..........
CFT073       .......... .......... .......... .......... .......... .......... ..........
E2348/69     .......... .......... .......... .......... .......... .......... ..........
ECOR_51      .......... .......... .......... .......... .......... .......... ..........
ECOR_57      .......... .......... .......... .......... .......... .......... ..........
UTI89        .......... .......... .......... .......... .......... .......... ..........
E._albertii  .......... ...C...... .CT....... ....G..... C......... .......... ..........
```

```
        17851
K-12          TGACGAAGAG ATGGCACTGC GCGATGAAGA AGTGACTGGG GAACTTCCTG AGGATCTGGA ATACGAAGAG
53638         .......... .......... .......... .......... .......... .......... ..........
ATCC_8739     .......... .......... .......... .......... .......... .......... ..........
ECOR_10       .......... .......... .......... .......... .......... .......... ..........
ECOR_11       .......... .......... .......... .......... .......... .......... ..........
B171          .......... .......... .......... .......... ........GC CA...T.A.. ..........
E24377A       .......... .......... .......... .......... .......... .......... ..........
ECOR_34       .......... .......... .......... .......... .......... .......... ..........
ECOR_71       .......... .......... .......... .......... .......... .......... ..........
SE11          .......... .......... .......... .......... .......... .......... ..........
O157_Sakai    .......... .......... .......... .......... ........GC CA...T.... ..........
ECOR_36       .......... .......... .......... .......... .......... .......... G.........
ECOR_35       .......... .......... .......... .......... .......... .......... G.........
SMS-3-5       .......... .......... .......... .......... .......... .......... G.........
536           .......... ....T..... .......... .......... .......... .......... G.........
APEC_O1       .......... ....T..... .......... .......... .......... .......... G.........
CFT073        .......... ....T..... .......... .......... .......... .......... G.........
E2348/69      ...T...... ....T..... .......... .......... .......... .......... G.........
ECOR_51       .......... ....T..... .......... .......... .......... .......... G.........
ECOR_57       .......... ....T..... .......... .......... .......... .......... G.........
UTI89         .......... ....T..... .......... .......... .......... .......... G.........
E._albertii   .......... ......T... .T..C..C.. ......C... ........GC CA...T.A.. G.........

        17921
K-12          TTTAACGAGA TCCGCGAACA GCTGGCGGCG ATCATCGAAG AACAACTTGC CGTGTACAAA ACCAGACAAG
53638         .......... .......... .......... .......... .......... .......... ..........
ATCC_8739     .......... .......... .......... .......... .......... .......... ..........
ECOR_10       .......... .......... .......... .......... .......... .......... ..........
ECOR_11       .......... .......... .......... .......... .......... .......... ..........
B171          .......... .......... .......... .......... .......... T......... ..........
E24377A       .......... .......... .......... .......... .......... .......... ..........
ECOR_34       .......... .......... .......... .......... .......... .......... ..........
ECOR_71       .......... .......... .......... .......... .......... .......... ..........
SE11          .......... .......... .......... .......... .......... .......... ..........
O157_Sakai    .......... .......... .......... .......... .......... .......... ..........
ECOR_36       .......... .......... .......... .....T.... .......... .......... ..........
ECOR_35       .......... .......... .......... .....T.... .......... .......... ..........
SMS-3-5       .......... .......... .......... .....T.... .......... .......... ..........
536           .......... .......... .......... .......... .......... .......... ..........
APEC_O1       .......... .......... .......... .......... .......... .......... ..........
CFT073        .......... .......... .......... .......... .......... .......... ..........
E2348/69      .......... .......... .......... .......... .......... .......... ..........
ECOR_51       .......... .......... .......... .......... .......... .......... ..........
ECOR_57       .......... .......... .......... .......... .......... .......... ..........
UTI89         .......... .......... .......... .......... .......... .......... ..........
E._albertii   .......... ....T..G.. A......... .......... .G..G..... T..A..T... ..........

        17991
K-12          TGCCGCTGGA TCTTGGTCTG GTGGTACGCG AATATCTGTC ACAGTATCCG CGTGCACGTC ACTTTGACGT
53638         .......... .......... .......... .......... .......... .......... ..........
ATCC_8739     .......... .......... .......... .......... .......... .......... ..........
ECOR_10       .......... .......... .......... .......... .......... .......... ..........
ECOR_11       .......... .......... .......... .......... .......... .......... ..........
B171          .......... .......... .......... .......... .......... .......... ..........
E24377A       .......... .......... .......... .......... .......... .......... ..........
ECOR_34       .......... .......... .......... .......... .......... .......... ..........
ECOR_71       .......... .......... .......... .......... .......... .......... ..........
SE11          .......... .......... .......... .......... .......... .......... ..........
O157_Sakai    .......... .......... .......... .......... .......... .......... ..........
ECOR_36       .......... .......... .......... .......... .......... .......... ..........
ECOR_35       .......... .......... .......... .......... .......... .......... ..........
SMS-3-5       .......... .......... .......... .......... .......... .......... ..........
536           .....T.... .......... .......... .......... .......... .......... ..........
APEC_O1       .......... .......... .......... .......... .......... .......... ..........
CFT073        .....T.... .......... .......... .......... .......... .......... ..........
E2348/69      .....T.... .......... .......... .......... .......... .......... ..........
ECOR_51       .....T.... .......... .......... .......... .......... .......... ..........
ECOR_57       .....T.... .......... .......... .......... .......... .......... ..........
UTI89         .......... .......... .......... .......... .......... .......... ..........
E._albertii   .A........ .......... .......... .......... G........A ....G..... ........T.
```

```
         18061
K-12          TGCGCGTATT GTTATTGATC AGGCGGTACG TCTTGGCGTA GCGCATCATG CATTCCTGAT GGATTTTCAG
53638         .......... .......... .......... .......... .......... .......... ..........
ATCC_8739     .......... .......... .......... .......... .......... .......... ..........
ECOR_10       .......... .......... .......... .......... .......... .......... ..........
ECOR_11       .......... .......... .......... .......... .......... .......... ..........
B171          ......C... .......... .......... .......... .......... .......... ..........
E24377A       ......C... .......... .......... .......... .......... .......... ..........
ECOR_34       .......... .......... .......... .......... .......... .......... ..........
ECOR_71       .......... .......... .......... .......... .......... .......... ..........
SE11          ......C... .......... .......... .......... .......... .......... ..........
O157_Sakai    ......C... .......... .......... .......... .......... .......... ..........
ECOR_36       ......C... .......... .......... .......... .......... .......... ..........
ECOR_35       ......C... .......... .......... .......... .......... .......... ..........
SMS-3-5       ......C... .......... .......... .......... .......... .......... ..........
536           ......C... .......... .......... .......... .......... .......... ..........
APEC_O1       ......C... .......... .......... .......... .......... .......... ..........
CFT073        ......C... .......... .......... .......... .......... .......... ..........
E2348/69      ......C... .......... .......... .......... .......... .......... ..........
ECOR_51       ......C... .......... .......... .......... .......... .......... ..........
ECOR_57       ......C... .......... .......... .......... .......... .......... ..........
UTI89         ......C... .......... .......... .......... .......... .......... ..........
E._albertii   ......C..C .......... .......... C..C...... .......... .T........ ..........

         18131
K-12          GAATATCTGG AAGAGTTTTA CGCGCGTTAT AACGTTGAGC TTATTCGCGC ACCAGAAGGG TTCTTCTATT
53638         .......... .......... .......... .......... .......... ...G...... ..........
ATCC_8739     .......... .......... .......... .......... .......... ...G...... ..........
ECOR_10       .......... .......... .......... .......... .......... .......... ..........
ECOR_11       .......... .......... .......... .......... .......... .......... ..........
B171          .......... .......... .......... .......... .......... .......... ..........
E24377A       .......... .......... .......... .......... .......... ...G...... ..........
ECOR_34       .......... .......... .......... .......... .......... .......... ..........
ECOR_71       .......... .......... .......... .......... .......... .......... ..........
SE11          .......... .......... .......... .......... .......... ...G...... ..........
O157_Sakai    .......... .......... .......... .......... .......T.. ...G...... ..........
ECOR_36       .......... .......... .......... .......... .......... ...G...... ..........
ECOR_35       .......... .......... .......... .......... .......... ...G...... ..........
SMS-3-5       .......... .......... .......... .......... .......... ...G...... ..........
536           .......... .......... .......... .......... .......T.. ...G...... .........C
APEC_O1       .......... .......... .......... .......... .......... ...G...... .........C
CFT073        .......... .......... .......... .......... .......T.. ...G...... .........C
E2348/69      .......... .......... .......... .......... .......T.. ...G...... .........C
ECOR_51       .......... .......... .......... .......... .......T.. ...G...... .........C
ECOR_57       .......... .......... .......... .......... .......T.. ...G...... .........C
UTI89         .......... .......... .......... .......... .......... ...G...... .........C
E._albertii   .......... .......... .......... .......... .......... G..G.....T ..........

         18201
K-12          TACGCCCACG TTCCACCACG CTGATCCCTC GTTCCGTCTT GTCGGAACTG GATATGATGG TCGGGAAAAT
53638         .......... .........T ..T.....G. .......... .......... .......... ..........
ATCC_8739     .......... .........T ..T.....G. .......... .......... .......... ..........
ECOR_10       .......... .......... .......... .......... .......... .......... ..........
ECOR_11       .......... .......... .......... .......... .......... .......... ..........
B171          .......... .......... .......... .......... .......... .......... ..........
E24377A       .......... .........T ..T.....G. .......... .......... .......... ..........
ECOR_34       .......... .......... .......... .......... .......... .......... ..........
ECOR_71       .......... .......... .......... .......... .......... .......... ..........
SE11          .......... .........T ..T.....G. .......... .......... .......... ..........
O157_Sakai    ....T..... .......... .......... .......... .......... .......... ..........
ECOR_36       .......... .........T ..T.....G. .......... .......... .......... ..........
ECOR_35       .......... .........T ..T.....G. .......... .......... .......... ..........
SMS-3-5       .......... .........T ..T.....G. .......... .......... .......... ..........
536           .......... .......... .......... .......... .......... .......... ..........
APEC_O1       .......... .......... .......... .......... .......... .......... ..........
CFT073        .......... .......... .......... .......... .......... .......... ..........
E2348/69      .......... .......... .......... .......... .......... .......... ..........
ECOR_51       .......... .......... .......... .......... .......... .......... ..........
ECOR_57       .......... .......... .......... .......... .......... .......... ..........
UTI89         .......... .......... .......... .......... .......... .......... ..........
E._albertii   ....T..... .......... T.......G. .......G.. A..A..G... ..C....... ..........
```

```
        18271
K-12          CCTCTGTTAT CTCTATCTCA GCCCGGAACG GCTGGCGAAT GAGGGGATTT TCACCCAGCA GGAACTGTAC
53638         .......... .......... .......... .......... .......... .......... ..........
ATCC_8739     .......... .......... .......... .......... .......... .......... ..........
ECOR_10       .......... .......... .......... .......... .......... .......... ..........
ECOR_11       .......... .......... .......... .......... .......... .......... ..........
B171          .......... .......... .T........ .......... .......... .......... ..........
E24377A       .......... .......... .......... .......... .......... .......... ..........
ECOR_34       .......... .......... .......... .......... .......... .......... ..........
ECOR_71       .......... .......... .......... .......... .......... .......... ..........
SE11          .......... .......... .......... .......... .......... .......... ..........
O157_Sakai    .......... .......... .......... .......... .......... .......... ..........
ECOR_36       .......... .......... .......... .........C .......... .......... ..........
ECOR_35       .......... .......... .......... .........C .......... .......... ..........
SMS-3-5       .......... .......... .......... .........C .......... .......... ..........
536           .......... .......... .......... .......... .......... .T..T..A.. ..........
APEC_O1       .......... .......... .......... .......... .......... .T..T..A.. ..........
CFT073        .......... .......... .......... .......... .......... .T..T..A.. ..........
E2348/69      .......... .......... .......... .......... .......... .T..T..A.. ..........
ECOR_51       .......... .......... .......... .......... .......... .T..T..A.. ..........
ECOR_57       .......... .......... .......... .......... .......... .T..T..A.. ..........
UTI89         .......... .......... .......... .......... .......... .T..T..A.. ..........
E._albertii   ...T...... ..T.....T. .......G.. .........C .....A..C. .T........ ....T....T

        18341
K-12          GACGAACTGC TCACCCTGGC CGATGAAGCA AAACTGCTGA AACTGGTGAA CAACCGTTCA ACCGGTTCAG
53638         .......... .......... .......... .......... .......... .......... ..........
ATCC_8739     .......... .......... .......... .......... .......... .......... ..........
ECOR_10       .......... .......... .......... .......... .......... .......... ..........
ECOR_11       .......... .......... .......... .......... .......... .......... ..........
B171          .......... .......... .........G .......... .......... .......... ..........
E24377A       .......... .......... .......... .......... .......... .......... ..........
ECOR_34       .......... .......... .......... .......... .......... .......... ..........
ECOR_71       .......... .......... .......... .......... .......... .......... ..........
SE11          .......... .......... .......... .......... .......... .......... ..........
O157_Sakai    .......... .......... .........G .......... .......... .......... ..........
ECOR_36       .......... .......... .........G .......... .......... .......... ..........
ECOR_35       .......... .......... .........G .......... .......... .......... ..........
SMS-3-5       .......... .......... .........G .......... .......... .......... ..........
536           ..T....... .......... .........G .......... .......... .......... ..........
APEC_O1       .......... .......... .........G .......... .......... .......... ..........
CFT073        .......... .......... .........G .......... .......... .......... ..........
E2348/69      .......... .......... .........G .......... .......... .......... ..........
ECOR_51       .......... .......... .........G .......... .......... .......... ..........
ECOR_57       .......... .......... .........G .......... .......... .......... ..........
UTI89         .......... .......... .........G .......... .......... .......... ..........
E._albertii   .......... .......... .........G .......... .......A.. .........T ..........

        18411
K-12          ACGTTGACCG TCAGAAGTTG CAGGAGAAAG TACGTTCTTC GCTCAACCGT CTGCGTCGTT TAGGCATGGT
53638         .......... .......... .......... .......... .......... .......... ..........
ATCC_8739     .......... .......... .......... .......... .......... .......... ..........
ECOR_10       .......... .......... .......... .......... .......... .......... ..........
ECOR_11       .......... .......... .......... .......... .......... .......... ..........
B171          .......... .......... .......... .......... .......... .......... ..........
E24377A       .......... .......... .......... .......... .......... .......... ..........
ECOR_34       .......... .......... .......... .......... .......... .......... ..........
ECOR_71       .......... .......... .......... .......... .......... .......... ..........
SE11          .......... .......... .......... .......... .......... .......... ..........
O157_Sakai    ..A....... .......... .....A.... .......... .......... .......... ....T.....
ECOR_36       .......... C......... .......... .......... .......... .......... .G..T.....
ECOR_35       .......... C......... .......... .......... .......... .......... .G..T.....
SMS-3-5       .......... C......... .......... .......... .......... .......... .G..T.....
536           .......... .......... .....A.... .......... .......... .......... ..........
APEC_O1       .......... .......... .....A.... .......... .......... .......... ..........
CFT073        .......... .......... .....A.... .......... .......... .......... ..........
E2348/69      .......... .......... .....A.... .......... .......... T......... ..........
ECOR_51       .......... .......... .....A.... .......... .......... .......... ..........
ECOR_57       .......... .......... .....A.... .......... .......... .......... ..........
UTI89         .......... .......... .....A.... .......... .......... .......... ..........
E._albertii   ....C..T.. C......... .....A.... .......C.. ...T...... ........C. ....T.....
```

```
          18481
K-12          GTGGTTTATG GGCCACGACA GCAGCAAGTT TCGCATTACC GAATCGGTGT TCCGCTTCGG AGCCGATGTG
53638         .......... .......... .......... C......... .......... .......... ..........
ATCC_8739     .......... .......... .......... .......... .......... .......... ..........
ECOR_10       .......... .......... .......... .......... .......... .......... ..........
ECOR_11       .......... .......... .......... .......... .......... .......... ..........
B171          .......... .......... .......... C......... .......... .......... ..........
E24377A       .......... .......... .......... .......... .......... .......... ..........
ECOR_34       .......... .......... .......... .......... .......... .......... ..........
ECOR_71       .......... .......... .......... .......... .......... .......... ..........
SE11          .......... .......... .......... .......... .......... .......... ..........
O157_Sakai    .......... .......... .......... C......... .......... .......... ..........
ECOR_36       .......... .......... .......... C......... .......... .......... ..........
ECOR_35       .......... .......... .......... C......... .......... .......... ..........
SMS-3-5       .......... .......... .......... C......... .......... .......... ..........
536           .......... .......... .......... C......... .......... .......... C.........
APEC_O1       .......... .......... .......... C......... .......... .......... C.........
CFT073        .......... .......... .......... C......... .......... .......... ..........
E2348/69      .......... .......... .......... C......... .......... .......... ..........
ECOR_51       .......... .......... .......... C......... .......... .......... ..........
ECOR_57       .......... .......... .......... C......... .......... .......... ..........
UTI89         .......... .......... .......... C......... .......... .......... C.........
E._albertii   .......... ..G..T.... .......A.. C........G .......... .T..T..T.. T..G......

          18551
K-12          CGTGCTGGCG ACGATCCCCG TGAAGCACAG CGTCGCCTGA TTCGTGATGG CGAAGCAATG CCGATTGAAA
53638         .......... .......... .......... .......... .......... .......... ..........
ATCC_8739     .......... .......... .......... .......... .......... .......... ..........
ECOR_10       .......... .......... .......... .......... .......... .......... ..........
ECOR_11       .......... .......... .......... .......... .......... .......... ..........
B171          .......... .......... .......... .......... .......... .......... ..........
E24377A       .......... .......... .......... .......... .......... .......... ..........
ECOR_34       .......... .......... .......... .......... .......... .......... ..........
ECOR_71       .......... .......... .......... .......... .......... .......... ..........
SE11          .......... .......... .......... .......... .......... .......... ..........
O157_Sakai    .......... .......... .......... .......... .......... .......... ..........
ECOR_36       .......... .......... .......... .......... .......... .......... ..........
ECOR_35       .......... .......... .......... .......... .......... .......... ..........
SMS-3-5       .......... .......... .......... .......... .......... .......... ..........
536           .......... .......... .......... .......... .......... T......... ..........
APEC_O1       .......... .......... .......... .......... .......... T......... ..........
CFT073        .......... .......... .......... .......... .......... T......... ..........
E2348/69      .......... .......... .......... .......... .......... T......... ..........
ECOR_51       .......... .......... .......... .......... .......... T......... ..........
ECOR_57       .......... .......... .......... .......... .......... T......... ..........
UTI89         .......... .......... .......... .......... .......... T......... ..........
E._albertii   .......... .......G.. C..G..C... .......... ....C..... T......... T..C.G....

          18621
K-12          ATCATCTGCA ACTCAACGAT GAAACCGAAG AGAATCAGCC AGATAGCGGA GAGGAAGAAT AATGATTGAA
53638         .......... .......... .......... ...GC..... .......... .......... ..........
ATCC_8739     .......... .......... .......... .......... .......... .......... ..........
ECOR_10       .......... .......... .......... .......... .......... .......... ..........
ECOR_11       .......... .......... .......... .......... .......... .......... ..........
B171          .......... .......... ....A..... .......... .......... .......... ..........
E24377A       .......... .......... .......... .......... .......... .......... ..........
ECOR_34       .......... .......... .......... .A........ .......... .......... ..........
ECOR_71       .......... .......... .......... .......... .......... .......... ..........
SE11          .......... .......... .......... .......... .......... .......... ..........
O157_Sakai    .......... .......... .......... .......... .......... .......... ..........
ECOR_36       .......... .......... .......... .......... .......... .......... ..........
ECOR_35       .......... .......... .......... .......... .......... .......... ..........
SMS-3-5       .......... .......... .......... .......... .......... .......... ..........
536           .......... .......... .......... ...G...... .......... .......... ..........
APEC_O1       .......... .......... .......... ...G...... .......... .......... ..........
CFT073        .......... .......... .......... ...G...... .......... .......... ..........
E2348/69      .......... .......... .......... ...G...... .......... .......... ..........
ECOR_51       .......... .......... .......... ...G...... .......... .......... ..........
ECOR_57       .......... .......... .......... ...G...... .......... .......... ..........
UTI89         .......... .......... .......... ...G...... .......... .......... ..........
E._albertii   ....C..... ......T... .......... .......... .......... .......... ..........
```

```
          18691
K-12          CGCGGTAAAT TTCGCTCACT GACGCTGATT AACTGGAACG GCTTTTTTGC CCGAACTTTT GACCTTGACG
53638         .......... .......... .......... .......... .......... .......... ..........
ATCC_8739     .......... .......... .......... .......... .......... .......... ..........
ECOR_10       .......... .......... .......... .......... .......... .......... ..........
ECOR_11       .......... .......... .......... .......... .......... .......... ..........
B171          .......... .......... .......... .......... .......... .......... ..........
E24377A       .......... .......... .......... .......... .......... .......... ..........
ECOR_34       .......... .......... .......... .......... .......... .......... ..........
ECOR_71       .......... .......... .......... .......... .......... .......... ..........
SE11          .......... .......... .......... .......... .......... .......... ..........
O157_Sakai    .......... .......... .......... .......... ....C..... .......... ..........
ECOR_36       .......... .......... .......... .......... .......... .......... ..........
ECOR_35       .......... .......... .......... .......... .......... .......... ..........
SMS-3-5       .......... .......... .......... .......... .......... .......... ..........
536           .......... .......... .......... .......... .......... .......... ..........
APEC_O1       .......... .......... .......... .......... .......... .......... ..........
CFT073        .......... .......... .......... .......... .......... .......... ..........
E2348/69      .......... .......... .......... .......... .......... .......... ..........
ECOR_51       .......... .......... .......... .......... .......... .......... ..........
ECOR_57       .......... .......... .......... .......... .......... .......... ..........
UTI89         .......... .......... .......... .......... .......... .......... ..........
E._albertii   .......... .......... .......... .......... .......... ...T..C... ........T.

          18761
K-12          AGCTGGTCAC GACGCTTTCT GGCGGTAACG GGGCGGGTAA ATCCACCACC ATGGCGGCGT TCGTTACGGC
53638         .......... .......... .......... .......... .......... .......... ..........
ATCC_8739     .......... .......... .......... .......... .......... .......... ..........
ECOR_10       .......... .......... .......... .......... .......... .......... ..........
ECOR_11       .......... .......... .......... .......... .......... .......... ..........
B171          .......... .......... .......... .......... .........T .......... ..........
E24377A       .......... .......... .......... .......... .......... .......... ..........
ECOR_34       .......... .......... .......... .......... .......... .......... ..........
ECOR_71       .......... .......... .......... .......... .......... .......... ..........
SE11          .......... .......... .......... .......... .......... .......... ..........
O157_Sakai    .......... .......... .......... .......... .......... .......... ..........
ECOR_36       .......... .......... .......... .......... .......... .......... ..........
ECOR_35       .......... .......... .......... .......... .......... .......... ..........
SMS-3-5       .......... .......... .......... .......... .......... .......... ..........
536           .......... .......... .......... .......... .......... .......... ..........
APEC_O1       .......... .......... .......... .......... .......... .......... ..........
CFT073        .......... .......... .......... .......... .......... .......... ..........
E2348/69      .......... .......... .......... .......... .......... .......... ..........
ECOR_51       .......... .......... .......... .......... .......... .......... ..........
ECOR_57       .......... .......... .......... .......... .......... .......... ..........
UTI89         .......... .......... .......... .......... .......... .......... ..........
E._albertii   .......... .........C .......... ....T..... .......... .......... .T........

          18831
K-12          GCTGATCCCC GACCTGACCC TGCTGCATTT CCGTAACACT ACGGAAGCCG GGGCCACCAG CGGTTCGCGC
53638         .......... .......... .......... .......... .......... .......... ..........
ATCC_8739     .......... .......... .......... .......... .......... .......... ..........
ECOR_10       .......... .......... .......... .......... .......... .......... ..........
ECOR_11       .......... .......... .......... .......... .......... .......... ..........
B171          .......... .......... .......... .......... .......... .......... ..........
E24377A       .......... .......... .......... .......... .......... .......... ..........
ECOR_34       .......... .......... .......... .......... .......... .......... ..........
ECOR_71       .......... .......... .......... .......... .......... .......... ..........
SE11          .......... .......... .......... .......... .......... .......... ..........
O157_Sakai    .......... .......... .......... .......... .......... .......... ..........
ECOR_36       .......... .......... .......... .........C .......... .......... ..........
ECOR_35       .......... .......... .......... .........C .......... .......... ..........
SMS-3-5       .......... .......... .......... .........C .......... .......... ..........
536           ...T...... ..T....... .......... .......... .......... .......... ..........
APEC_O1       ...T...... .......... .......... .......... .......... .......... ..........
CFT073        ...T...... .......... .......... .......... .......... .......... ..........
E2348/69      ...T...... ..T....... .......... .......... .......... .......... ..........
ECOR_51       ...T...... .......... .......... .......... .......... .......... ..........
ECOR_57       ...T...... .......... .......... .......... .......... .......... ..........
UTI89         ...T...... .......... .......... .......... .......... .......... ..........
E._albertii   ......T... .......... .......... .........C ........A. .......A.. T.....A...
```

```
         18901
K-12         GATAAAGGTC TGCACGGTAA GCTGAAAGCG GGTGTCTGTT ATTCGATGCT CGACACCATT AACTCGCGCC
53638        .......... .......... .......... .......... .......... .......... ..........
ATCC_8739    .......... .......... .......... .......... .......... .......... ..........
ECOR_10      .......... .......... .......... .......... .......... .......... ..........
ECOR_11      .......... .......... .......... .......... .......... .......... ..........
B171         .......... .......... .......... .......... .......... .......... ..........
E24377A      .......... .......... .......... .......... .......... .......... ..........
ECOR_34      .......... .......... .......... .......... .......... .......... ..........
ECOR_71      .......... .......... .......... .......... .......... .......... ..........
SE11         .......... .......... .......... .......... .......... .......... ..........
O157_Sakai   .......... .......... .......... .......... .......... .......... ..........
ECOR_36      .......... .......... .......... .......... .......... .......... ..........
ECOR_35      .......... .......... .......... .......... .......... .......... ..........
SMS-3-5      .......... .......... .......... .......... .......... .......... ..........
536          .......... .......... .......... .......... .......... T......... ..........
APEC_O1      .......... .......... .......... .......... .......... .......... .......A..
CFT073       .......... .......... .......... .......... .......... .......... ..........
E2348/69     .......... .......... .......... .......... .......... .......... ..........
ECOR_51      .......... .......... .......... .......... .......... .......... ..........
ECOR_57      .......... .......... .......... .......... .......... .......... ..........
UTI89        .......... .......... .......... .......... .......... .......... .......A..
E._albertii  ........CT .......G.. .T.......C ..G....... .......T.. .......... ..T..A....

         18971
K-12         ACCAGCGCGT GGTGGTCGGT GTGCGTCTGC AACAGGTTGC CGGACGCGAT CGTAAAGTGG ATATCAAGCC
53638        .......... .......... .......... .......... .......... .......... .......A..
ATCC_8739    .......... .......... .......... .......... .......... .......... .......A..
ECOR_10      .......... .......... .......... .......... .......... .......... ..........
ECOR_11      .......... .......... .......... .......... .......... .......... ..........
B171         .......... .......... .......... .......... .......... .......... .......A..
E24377A      .......... .......... .......... .......... .......... .......... ..........
ECOR_34      .......... .......... .......... .......... .......... .......... ..........
ECOR_71      .......... .......... .......... .......... .......... .......... .......A..
SE11         .......... .......... .......... .......... .......... .......... ..........
O157_Sakai   .......... .......... .......... .......... .......... .......... .......A..
ECOR_36      .......... .......... .......... .......... .......... .......... .......A..
ECOR_35      .......... .......... .......... .......... .......... .......... .......A..
SMS-3-5      .......... .......... .......... .......... .......... .......... .......A..
536          .......T.. A......... .......... .......... .......... .......... .......A..
APEC_O1      .......... A......... .....C.... .......... .......... .......... .......A..
CFT073       .......T.. A......... .......... .......... .......... .......... .......A..
E2348/69     .......... A......... .......... .......... .......... .......... .......A..
ECOR_51      .......T.. A......... .......... .......... .......... .......... .......A..
ECOR_57      .......T.. A......... .......... .......... .......... .......... .......A..
UTI89        .......... A......... .....C.... .......... .......... .......... .......A..
E._albertii  .T.....T.. AC....T... ......T.A. .......C.. ...G...... ........C. .......A..

         19041
K-12         GTTTGCCATT CAGGGACTGC CGATGTCGGT GCAGCCGACA CAGCTGGTGA CCGAAACCCT GAACGAACGC
53638        A......... .......... .......... .......... .......... .......... ..........
ATCC_8739    A......... .......... .......... .......... .......... .......... ..........
ECOR_10      .......... .......... .......... .......... .......... .......... ..........
ECOR_11      .......... .......... .......... .......... .......... .......... ..........
B171         A......... .......... .......... ..T......G .......... .......... ..........
E24377A      .......... .......... .......... .......... .......... .......... ..........
ECOR_34      .......... .......... .......... .......... .......... .......... ..........
ECOR_71      A......... .......... .......... .......... .......... .......... ..........
SE11         .......... .......... .......... .......... .......... .......... ..........
O157_Sakai   A......... .......... .......... .......... .......... .......T.. ..........
ECOR_36      .......... .....TT.A. .......... .......... ..A....... .......... ..G......T
ECOR_35      .......... .....TT.A. .......... .......... ..A....... .......... ..G......T
SMS-3-5      .......... .....TT.A. .......... .......... ..A....... .......... ..G......T
536          A......... .....C.... .......... .......... ..A....... .......T.. ..........
APEC_O1      A......... .....C.... .......... .......... ..A....... .......T.. ..........
CFT073       A......... .....C.... .......... .......... ..A....... .......T.. ..........
E2348/69     A......... .....C.... .......... .......... ..A....... .......T.. ..........
ECOR_51      A......... .....C.... .......... .......... ..A....... .......T.. ..........
ECOR_57      A......... .....C.... .......... .......... ..A....... .......T.. ..........
UTI89        A......... .....C.... .......... .......... ..A....... .......T.. ..........
E._albertii  ......G... .....CT... .......... .TT......G ..A....... .......... ...T......
```

```
        19111
K-12         CAGGCGCGCG TGCTGCCGCT TAACGAGCTG AAAGACAAGC TCGAGGCGAT GGAAGGCGTG CAGTTTAAAC
53638        .......... .......... .......... .......... .......... .......... ..........
ATCC_8739    .......... .......... .......... .......... .......... .......... ..........
ECOR_10      .......... .......... .......... .......... .......... .......... ..........
ECOR_11      .......... .......... .......... .......... .......... .......... ..........
B171         .......... .......... .......... .......... .......... .......... ..........
E24377A      .......... .......... .......... .......... .......... .......... ..........
ECOR_34      .......... .......... .......... .......... .......... .......... ..........
ECOR_71      .......... .......... .......... .......... .......... .......... ..........
SE11         .......... .......... .......... .......... .......... .......... ..........
O157_Sakai   .......... .......... .......... .......... .......... .......... ..........
ECOR_36      .......... .......... .......... .......... .......... .......... ..........
ECOR_35      .......... .......... .......... .......... .......... .......... ..........
SMS-3-5      .......... .......... .......... .......... .......... .......... ..........
536          .......... .......... ...T...... .......... .......... .......... ........G.
APEC_O1      .......... .......... ...T...... .......... .......... .......... ........G.
CFT073       .......... .......... .......... .......... .......... .......... ..........
E2348/69     .......... .......... .......... .......... .......... .......... ........G.
ECOR_51      .......... .......... .......... .......... .......... .......... ..........
ECOR_57      .......... .......... .......... .......... .......... .......... ..........
UTI89        .......... .......... ...T...... .......... .......... .......... ........G.
E._albertii  ........T. .......... G......... .....G.... .......... .......... ........G.

        19181
K-12         AGTTCAACTC CATTACTGAT TACCACTCGC TGATGTTCGA TCTGGGCATC ATCGCGCGTC GTCTGCGCTC
53638        .......... ......C... .......... .......... .......... .......... ..........
ATCC_8739    .......... ......C... .......... .......... .......... .......... ..........
ECOR_10      .......... .......... .......... .......... .......... .......... ..........
ECOR_11      .......... .......... .......... .......... .......... .......... ..........
B171         .......... ......C... .......... .......... .......... .......... ..........
E24377A      .......... .......... .......... .......... .......... .......... ..........
ECOR_34      .......... .......... .......... .......... .......... .......... ..........
ECOR_71      .......... ......C... .......... .......... ...T...... .......... ..........
SE11         .......... .......... .......... .......... .......... .......... ..........
O157_Sakai   .......... ......C... .......... .......... .......... ..T....... ..........
ECOR_36      .......... ......C... .......... .......... .......... .......... ..........
ECOR_35      .......... ......C... .......... .......... .......... .......... ..........
SMS-3-5      .......... ......C... .......... .......... .......... .......... ..........
536          .......... ......C... .......... .......... .......... .......... ..........
APEC_O1      .......... ......C... .......... .......... .......... .......... ..........
CFT073       .......... ......C... .......... .......... G......... .......... ..........
E2348/69     .......... ......C... .......... .......... .......... .......... .C.....T..
ECOR_51      .......... ......C... .......... .......... G......... .......... ..........
ECOR_57      .......... ......C... .......... .......... G......... .......... ..........
UTI89        .......... ......C... .......... .......... .......... .......... ..........
E._albertii  .......... ......C... .....T.... .......... .......... ..T....... ..T.......

        19251
K-12         CGCATCTGAC CGTAGCAAAT TCTATCGTCT GATCGAAGCT TCGCTGTATG GCGGGATCTC CAGTGCCATT
53638        .......... .......... .......... .......... .......... .......... ..........
ATCC_8739    .......... .......... .......... .......... .......... .......... ..........
ECOR_10      .......... .......... .......... .......... .......... .......... ..........
ECOR_11      .......... .......... .......... .......... .......... .......... ..........
B171         .......... .......... .......... .......... .......... .......... ..........
E24377A      .......... .......... .......... .......... .......... .......... ..........
ECOR_34      .......... .......... .......... .......... .......... .......... ..........
ECOR_71      .......... .......... .......... .......... .......... .......... ..........
SE11         .......... .......... .......... .......... .......... .......... ..........
O157_Sakai   .......... .......... .......... .......... .......... .......... ..........
ECOR_36      A..G...... .....T.... .......... .......... ........C. .......... ...C......
ECOR_35      A..G...... .....T.... .......... .......... ........C. .......... ...C......
SMS-3-5      A..G...... .....T.... .......... .......... ........C. .......... ...C......
536          A..G...... .....T.... .......... .........C .......... .......... ...C......
APEC_O1      A..G...... .....T.... .......... .........C .......... .......... ...C......
CFT073       A..G...... .....T.... .......... .........C .......... .......... ...C......
E2348/69     A..G.....T .....T.... .......... .........C .......... .......... ...C......
ECOR_51      A..G...... .....T.... .......... .........C .......... .......... ...C......
ECOR_57      A..G...... .....T.... .......... .........C .......... .......... ...C......
UTI89        A..G...... .....T.... .......... .........C .......... .......... ...C......
E._albertii  G..C...... .......... ....C..... A......... ........C. .T..T..T.. ...C......
```

```
          19321
K-12          ACCCGTTCTC TGCGCGACTA CCTGTTGCCA GAAAACAGCG GCGTGCGTAA AGCGTTCCAG GACATGGAAG
53638         .......... .......... .........G .......... .......... .......... ..........
ATCC_8739     .......... .......... .........G .......... .......... .......... ..........
ECOR_10       .......... .......... .......... .......... .......... .......... ..........
ECOR_11       .......... .......... .......... .......... .......... .......... ..........
B171          .......... .......... .........G .......... .......... .......... ..........
E24377A       .......... .......... .......... .......... .......... .......... ..........
ECOR_34       .......... .......... .........G .......... .......... .......... ..........
ECOR_71       .......... .......... .........G .......... .......... .......... ..........
SE11          .......... .......... .......... .......... .......... .......... ..........
O157_Sakai    .......... .......... .........G .......... .......... .......... ..........
ECOR_36       ..G..C.... .......... .........G .......... .......G.. .......... ..........
ECOR_35       ..G..C.... .......... .........G .......... .......G.. .......... ..........
SMS-3-5       ..G..C.... .......... .........G .......... .......G.. .......... ..........
536           ..T....... .......... .........G .......... .G.....G.. .......... ..........
APEC_O1       ..T....... .......... .........G .......... .G.....G.. .......... ..........
CFT073        ..T....... .......... .........G ........T. .G.....G.. .......... ..........
E2348/69      ..T....... .......... .........G .......... .G.....G.. .......... ..........
ECOR_51       ..T....... .......... .........G ........T. .G.....G.. .......... ..........
ECOR_57       ..T....... .......... .........G ........T. .G.....G.. .......... ..........
UTI89         ..T....... .......... .........G .......... .G.....G.. .......... ..........
E._albertii   .......... .......... .........G .......... .T........ ......T... ..T.......

          19391
K-12          CGGCGCTGCG TGAAAACCGT ATGACGCTGG AAGCGATTCG TGTCACCCAG TCGGACCGCG ACCTGTTTAA
53638         .......... .........C .......... .......... .......... ........T. ..........
ATCC_8739     .......... .........C .......... .......... .......... ........T. ..........
ECOR_10       .......... .......... .......... .......... .......... .......... ..........
ECOR_11       .......... .......... .......... .......... .......... .......... ..........
B171          .......... .........C .......... .......... .......... ........T. ..........
E24377A       .......... .......... .......... .......... .......... .......... ..........
ECOR_34       .......... .......... .......... .......... .......... ........T. ..........
ECOR_71       .......... .........C .......... .......... .......... ........T. ..........
SE11          .......... .......... .......... .......... .......... .......... ..........
O157_Sakai    .......... .........C .......... .......... .......... ........T. ..........
ECOR_36       .......... C........C .......... .......... .......... .......... ..........
ECOR_35       .......... C........C .......... .......... .......... .......... ..........
SMS-3-5       .......... C........C .......... .......... .......... .......... ..........
536           .......... .........C .......... .......... .......... ........T. ..........
APEC_O1       .......... .........C .......... .......... .......... ........T. ..........
CFT073        .......... .........C .......... .......... .......... ........T. ..........
E2348/69      .......... .........C .......... .......... .......... ........T. ..........
ECOR_51       .......... .........C .......... .......... .......... ........T. ..........
ECOR_57       .......... .........C .......... .......... .......... ........T. ..........
UTI89         .......... .........C .......... .......... .......... ........T. ..........
E._albertii   .......... .......... .......... .......... C.....T... ........T. ..........

          19461
K-12          GCATCTGATC AGCGAAGCCA CCAACTACGT GGCGGCGGAC TACATGCGTC ACGCCAACGA GCGCCGTGTC
53638         .......... ........T. .......... .......... .......... .......... ..........
ATCC_8739     .......... ........T. .......... .......... .......... .......... ..........
ECOR_10       .......... .......... .......... .......... .......... .......... ..........
ECOR_11       .......... .......... .......... .......... .......... .......... ..........
B171          .......... ........T. .......... .......... .......... .......... ..........
E24377A       .......... .......... .......... .......... .......... .......... ..........
ECOR_34       .......... ........T. .......... .......... .......... .......... ..........
ECOR_71       .......... ........T. .......... .......... .......... .......... ..........
SE11          .......... .......... .......... .......... .......... .......... ..........
O157_Sakai    .......... ........T. .......... .......... .......... .......... ..........
ECOR_36       .........T .......... .......... .......... .......... .......... ..........
ECOR_35       .........T .......... .......... .......... .......... .......... ..........
SMS-3-5       .........T .......... .......... .......... .......... .......... ..........
536           .......... .......... .......... .......... .......... .......... .........T
APEC_O1       .......... .......... .......... .......... .......... .......... .........T
CFT073        .......... .......... .......... .......... .......... .......... .........T
E2348/69      .........T .......... ....T..... .......... .......... .......... .........T
ECOR_51       .......... .......... .......... .......... .......... .......... .........T
ECOR_57       .......... .......... .......... .......... .......... .......... .........T
UTI89         .......... .......... .......... .......... .......... .......... .........T
E._albertii   .........T ........T. .......... ......A... .......... .......... .........T
```

```
        19531
K-12          CATCTCGACA AAGCCCTGGA GTTTCGTCGC GAGCTACATA CTTCGCGTCA GCAACTGGCG GCTGAGCAGT
53638         .......... .......... .......... ...T...... .......... .......... ..........
ATCC_8739     .......... .......... .......... ...T...... .......... .......... ..........
ECOR_10       .......... .......... .......... .......... .......... .......... ..........
ECOR_11       .......... .......... .......... .......... .......... .......... ..........
B171          .......... .......... .......... .......... .......... .......... ..........
E24377A       .......... .......... .......... .......... .......... .......... ..........
ECOR_34       .......... .......... .......... ...T...... .......... .......... ..........
ECOR_71       .......... .......... .......... ...T...... .......... .......... ..........
SE11          .......... .......... .......... .......... .......... .......... ..........
O157_Sakai    ........T. .G........ .......... ..A....... .......... .......... ..G.......
ECOR_36       .......... .......... .......... ........C. .......... .......... ........T.
ECOR_35       .......... .......... .......... ........C. .......... .......... ........T.
SMS-3-5       .......... .......... .......... ........C. .......... .......... ..........
536           ........T. .G........ .......... .......... ........A. .......... ..........
APEC_O1       ........T. .G........ .......... .......... ........A. .......... ..........
CFT073        ........T. .G........ .......... .......... .......... .......... ..........
E2348/69      ........T. .G........ .......... .......... .......... .......... ..........
ECOR_51       ........T. .G........ .......... .......... .......... .......... ..........
ECOR_57       ........T. .G........ .......... .......... .......... .......... ..........
UTI89         ........T. .G........ .......... .......... ........A. .......... ..........
E._albertii   .....T..T. ....G..... .........T .......... T......... .......... ..G.......

        19601
K-12          ACAAACACGT CGATATGGCG CGTGAGCTGG CGAG-CACAA CGGTGCCGAA GGCGATCTGG AAGCGGATTA
53638         .......... .......... .......... .......... .......... .......... ..........
ATCC_8739     .......... .......... .......... .......... .......... .......... ..........
ECOR_10       .......... .......... .......... .......... .......... .......... ..........
ECOR_11       .......... .......... .......... .......... .......... .......... ..........
B171          .......... .......... .......... .......... .......... .......... ..........
E24377A       .......... .......... .......... .......... .......... .......... ..........
ECOR_34       .......... .......... .......... .......... .......... .......... ..........
ECOR_71       .......... .......... .......... .......... .......... .......... ..........
SE11          .......... .......... .......... .......... .......... .......... ..........
O157_Sakai    .......... .......... .......... .......... .......... .......... ..........
ECOR_36       .......... .......... .......... .......... .......... .......... ....A.....
ECOR_35       .......... .......... .......... .......... .......... .......... ....A.....
SMS-3-5       .......... .......... .......... .......... .......... .......... ....A.....
536           .......... ...C...... ..A....... .......... .......... .......... ..........
APEC_O1       .......... ...C...... ..A....... .......... .......... .......... ..........
CFT073        .......... ...C...... ..A....... .......... .......... .......... ..........
E2348/69      .......... .......... .......... .......... .......... .......... ..........
ECOR_51       .......... ...C...... ..A....... .......... .......... .......... ..........
ECOR_57       .......... ...C...... ..A....... .......... .......... .......... ..........
UTI89         .......... ...C...... ..A....... .......... .......... .......... ..........
E._albertii   .......... .........A .....A.... ..GAA..... ...C...... .......... ....A.....

        19671
K-12          TCAGGCGGCC AGTGATCACC TGAACCTGGT GCAAACCGCA CTGCGTCAGC AGGAGAAGAT CGAACGCTAC
53638         .......... ..C....... .......... .......... .......... .......... ..........
ATCC_8739     .......... ..C....... .......... .......... .......... .......... ..........
ECOR_10       .......... .......... .......... .......... .......... .......... ..........
ECOR_11       .......... .......... .......... .......... .......... .......... ..........
B171          .......... ..C....... .......... .......... .......... .......... ..........
E24377A       .......... .......... .......... .......... .......... .......... ..........
ECOR_34       .......... ..C....... .......... .......... .......... .......... ..........
ECOR_71       .......... ..C....... .......... .......... .......... .......... ..........
SE11          .......... .......... .......... .......... .......... .......... ..........
O157_Sakai    .......... ..C....... .......... .......... .......... .......... ..........
ECOR_36       .......... ..C....... .......... .......... .......... .......... ..........
ECOR_35       .......... ..C....... .......... .......... .......... .......... ..........
SMS-3-5       .......... ..C....... .......... .......... .......... .......... ..........
536           .......... ..C....... .......... .......... .......... .......... ..........
APEC_O1       .......... ..C....... .......... .......... .......... .......... ..........
CFT073        .......... ..C....... .......... .......... .......... .......... ..........
E2348/69      .......... ..C....... .......... .......... .......... .......... ..........
ECOR_51       .......... ..C....... .......... .......... .......... .......... ..........
ECOR_57       .......... ..C....... .......... .......... .......... .......... ..........
UTI89         .......... ..C....... .......... .......... .......... .......... ..........
E._albertii   ......T... ..C....... ....T..... ...G..A... ........A. .......... ..........
```

```
         19741
K-12          GAAGCGGATC TCGATGAGCT GCAGATCCGT CTGGAAGAGC AAAATGAAGT GGTGGCAGAA GCCATCGAAC
53638         .......... .......... .......... .......... ....C..... .......... ..........
ATCC_8739     .......... .......... .......... .......... ....C..... .......... ..........
ECOR_10       .......... .......... .......... .......... .......... .......... ..........
ECOR_11       .......... .......... .......... .......... .......... .......... ..........
B171          .......... .......... .......... .......... ....C..... .......... ..........
E24377A       .......... .......... .......... .......... .......... .......... ..........
ECOR_34       .......... .......... .......... .......... ....C..... .......... ..........
ECOR_71       .......... .......... .......... .......... ....C..... .......... ..........
SE11          .......... .......... .......... .......... .......... .......... ..........
O157_Sakai    .......... .......... .......... .......... ....C..... .......... ..........
ECOR_36       .......... .......... .......... .......... ....C..... .......... ..........
ECOR_35       .......... .......... .......... .......... ....C..... .......... ..........
SMS-3-5       .......... .......... .......... .......... ....C..... .......... ..........
536           .......... .......... .......... .......... ....C..... .......... ..........
APEC_O1       .......... .......... .......... .......... ....C..... .......... ..........
CFT073        .......... .......... .......... .......... ....C..... .......... ..........
E2348/69      .......... .......... .......... .......... ....C..... .......... ..........
ECOR_51       .......... .......... .......... .......... ....C..... .......... ..........
ECOR_57       .......... .......... .......... .......... ....C..... .......... ..........
UTI89         .......... .......... .......... .......... ....C..... .......... ..........
E._albertii   ........C. .T........ .......... .......... ....C..... A..A..G... .....T..G.

         19811
K-12          GCCAGCAAGA GAATGAGGCT CGTGCGGAAG CTGCCGAACT GGAAGTGGAC GAGCTGAAAA GCCAGCTTGC
53638         .....G.... ...C...... ..C....... .......... .......... .......... ..........
ATCC_8739     .....G.... A..C...... ..C....... .......... .......... .......... ..........
ECOR_10       .....G.... .......... .......... .......... .......... .......... ..........
ECOR_11       .....G.... .......... .......... .......... .......... .......... ..........
B171          .....G.... ...C...... .......... .......... .......... .......... ..........
E24377A       .....G.... .......... .......... .......... .......... .......... ..........
ECOR_34       .....G.... ...C...... ..C....... .......... .......... .......... ..........
ECOR_71       .....G.... ...C...... ..C....... .......... .......... .......... ..........
SE11          .....G.... .......... .......... .......... .......... .......... ..........
O157_Sakai    .....G.... ...C...... ..C....... .......... .......... .......... ..........
ECOR_36       .....G.... ...C...... .......... .......... .......... .......... ..........
ECOR_35       .....G.... ...C...... .......... .......... .......... .......... ..........
SMS-3-5       .....G.... ...C...... .......... .......... .......... .......... ..........
536           .....G.... ...C...... .......... .......... .......... .......... ..........
APEC_O1       .....G.... ...C...... .......... .......... .......... .......... ..........
CFT073        .....G.... ...C...... .......... .......... .......... .......... ..........
E2348/69      .....G.... ...C...... .......... .......... .......... .......... ..........
ECOR_51       .....G.... ...C...... .......... .......... .......... .......... ..........
ECOR_57       .....G.... ...C...... .......... .......... .......... .......... ..........
UTI89         .....G.... ...C...... .......... .......... .......... .......... ..........
E._albertii   .....G.... A..C..A..G .....A.... .......G.. .........T ..A....... ..........

         19881
K-12          TGACTACCAG CAGGCGCTGG ACGTCCAGCA AACGCGCGCG ATCCAGTATA ACCAGGCGAT TGCTGCGCTT
53638         .......... .......... .......... .......... .......... .......... ......A...
ATCC_8739     .......... .......... .......... .......... .......... .......... ......A...
ECOR_10       .......... .......... .......... .......... .......... .......... ..........
ECOR_11       .......... .......... .......... .......... .......... .......... ..........
B171          .......... .......... .......... .......... .......... .......... ..........
E24377A       .......... .......... .......... .......... .......... .......... ..........
ECOR_34       .......... .......... .......... .......... .......... .......... ..........
ECOR_71       .......... .......... .......... .......... .......... .......... ..........
SE11          .......... .......... .......... .......... .......... .......... ..........
O157_Sakai    .......... .......... .......... .......... ........C. .......... ..........
ECOR_36       .......... .......... .......... .......... .......... .......... ..........
ECOR_35       .......... .......... .......... .......... .......... .......... ..........
SMS-3-5       .......... .......... .......... .......... .......... .......... ..........
536           .......... .......... .......... .......... .......... .......... ..........
APEC_O1       .......... .......... .......... .......... .......... .......... ..........
CFT073        .......... .......... .......... .......... .......... .......... ..........
E2348/69      .......... .......... .......... .......... .......... .......... ..........
ECOR_51       .......... .......... .......... .......... .......... .......... ..........
ECOR_57       .......... .......... .......... .......... .......... .......... ..........
UTI89         .......... .......... .......... .......... .......... .......... ..........
E._albertii   .......... .......... .......... ......T..A ..T.....C. .......... ...C......
```

```
        19951
K-12         AATCGTGCCA AAGAACTGTG CCATCTGCCG GACTTAACCG CCGACTGCGC CGCCGAATGG CTGGAAACCT
53638        .......... .......... .......... .......... .....A.... .......... ..........
ATCC_8739    .......... .......... .......... .......... .....A.... .......... ..........
ECOR_10      .......... .......... .......... .......... .......... .......... ..........
ECOR_11      .......... .......... .......... .......... .......... .......... ..........
B171         .....C.... .......... .......... .......... .....A.... .......... ..........
E24377A      .......... .......... .......... .......... .......... .......... ..........
ECOR_34      .....C.... .......... .......... .......... .....A.... .......... ..........
ECOR_71      .....C.... .......... .......... .........T .....A.... .......... ..........
SE11         .......... .......... .......... .......... .......... .......... ..........
O157_Sakai   .......... .......... .......... .......... .....A.... .......... ..........
ECOR_36      .......... .......... .......... .......... .....A.... .......... ..........
ECOR_35      .......... .......... .......... .......... .....A.... .......... ..........
SMS-3-5      .......... .......... .......... .......... .....A.... .......... ..........
536          .......... .......... .......... .......... .....A.... T......... ..........
APEC_O1      .......... .......... .......... .......... .....A.... T......... ..........
CFT073       .......... .......... .......... .......... .....A.... T......... ..........
E2348/69     .......... .......... .......... .......... .....A.... T......... ..........
ECOR_51      .......... .......... .......... .......... .....A.... T......... ..........
ECOR_57      .......... .......... .......... .......... .....A.... T......... ..........
UTI89        .......... .......... .......... .......... .....A.... T......... ..........
E._albertii  .......... ....GT.... T......... .......... .....A.... ...A...... ..........

        20021
K-12         TCCAGGCGAA AGAGCTGGAA GCGACTGAAA AAATGCTCTC TCTTGAGCAG AAAATGAGCA TGGCGCAAAC
53638        .......... .......... .......... .......... .......... .......... ..........
ATCC_8739    .......... .......... .......... .......... .......... .......... ..........
ECOR_10      .......... .......... .......... .......... .......... .......... ..........
ECOR_11      .......... .......... .......... .......... .......... .......... ..........
B171         .......... .......... .......... .......... .......... .......... ..........
E24377A      .......... .......... .......... .......... .......... .......... ..........
ECOR_34      .......... .......... .......... .......... .......... .......... ..........
ECOR_71      .......... .......... .......... .......... .......... .......... ..........
SE11         .......... .......... .......... .......... .......... .......... ..........
O157_Sakai   .......... .......... .......... .......... .......... .......... ..........
ECOR_36      .......... .......... .....C.... .......... .......... .......... ..........
ECOR_35      .......... .......... .....C.... .......... .......... .......... ..........
SMS-3-5      .......... .......... .....C.... .......... .......... .......... ..........
536          .......... .......... .....C.... .......... .......... .......... ..........
APEC_O1      .......... .......... .....C.... .......... .......... .......... ..........
CFT073       .......... .......... .....C.... .......... .......... .......... ..........
E2348/69     .......... .......... .....C.... .......... .......... .......... ..........
ECOR_51      .......... .......... .....C.... .......... .......... .......... ..........
ECOR_57      .......... .......... .....C.... .......... .......... .......... ..........
UTI89        .......... .......... .....C.... .......... .......... .......... ..........
E._albertii  .......... .......... .....C.... .......... .......... .......... ..........

        20091
K-12         CGCGCACAGC CAGTTTGAGC AGGCTTATCA GCTGGTGGTG GCAATCAACG GCCCACTGGC GCGTAACGAG
53638        .......... .......... .......... .......... .......... .......... ..........
ATCC_8739    .......... .......... .......... .......... .......... .......... ..........
ECOR_10      .......... .......... .......... .......... .......... .......... ..........
ECOR_11      .......... .......... .......... .......... .......... .......... ..........
B171         .......... .......... .......... .......... .......... ....G..... ..........
E24377A      .......... .......... .......... .......... .......... ....G..... ..........
ECOR_34      .......... .......... .......... .......... .......... ....G..... ..........
ECOR_71      .......... .......... .......... .......... .......... ....G..... ..........
SE11         .......... .......... .......... .......... .......... ....G..... ..........
O157_Sakai   .......... .......... .......... .......... .......... ....G..... ..........
ECOR_36      .......... .......... .......... .......... .......... ....G..... ..........
ECOR_35      .......... .......... .......... .......... .......... ....G..... ..........
SMS-3-5      .......... .......... .......... .......... .......... ....G..... ..........
536          .......... .......... .......... .......... .......... ....G..... ..........
APEC_O1      .......... .......... .......... .......... .......... ....G..... ..........
CFT073       .......... .......... .......... .......... .......... ....G..... ..........
E2348/69     .......... .......... .......... .......... .......... ....G..... ..........
ECOR_51      .......... .......... .......... .......... .......... ....G..... ..........
ECOR_57      .......... .......... .......... .......... .......... ....G..... ..........
UTI89        .......... .......... .......... .......... .......... ....G..... ..........
E._albertii  .......... ........A. .......C.. AT.A..C..A .......... ....G..... .........A
```

```
        20161
K-12         GCGTGGGATG TCGCTCGCGA ACTATTGCGC GAAGGGGTCG ATCAGCGTCA CCTGGCAGAG CAGGTTCAGC
53638        .......... .......... .......... .......... .......... .......... ..........
ATCC_8739    .......... .......... .......... .......... .......... .......... ..........
ECOR_10      .......... .......... .......... .......... .......... .......... ..........
ECOR_11      .......... .......... .......... .......... .......... .......... ..........
B171         ..T....... ....C..... ...GC..... .......... .......... .......... ........A.
E24377A      .......... .......... .......... .......... .......... .......... ........A.
ECOR_34      .......... .......... .......... .......... .......... .......... ........A.
ECOR_71      .......... .......... .......... .......... .......... .......... ..........
SE11         .......... .......... .......... .......... .......... .......... ........A.
O157_Sakai   .......... .......... .......... .......... .......... .......... ..........
ECOR_36      .......... .......... .......... .......... .......... .......... ..........
ECOR_35      .......... .......... .......... .......... .......... .......... ..........
SMS-3-5      .......... .......... .......... .......... .......... .......... ..........
536          .......... .......... .......... .......... .......... .......... ..........
APEC_O1      .......... .......... .......... .......... .......... .......... ..........
CFT073       .......... .......... .......... .......... .......... .......... ..........
E2348/69     .......... .......... .......... .......... .......... .......... ..........
ECOR_51      .......... .......... .......... .......... .......... .......... ..........
ECOR_57      .......... .......... .......... .......... .......... .......... ..........
UTI89        .......... .......... .......... .......... .......... .......... ..........
E._albertii  ..A....... .......... .T.GC....T .......... .......C.. .........A ..........

        20231
K-12         CGCTGCGGAT GCGATTAAGC GAACTGGAAC AGCGTCTACG CGAGCAGCAA GAAGCTGAGC GTCTGCTGGC
53638        .......... .......... .......... .......... .......... .......... ..........
ATCC_8739    .......... .......... .......... .......... .......... .......... ..........
ECOR_10      .......... .......... .......... .......... .......... .......... ..........
ECOR_11      .......... .......... .......... .......... .......... .......... ..........
B171         ..T....... ...C.....T .......... .......G.. .......... .....C..A. .C........
E24377A      ..T....... ...C.....T .......... .......G.. .......... .....C..A. .C........
ECOR_34      ..T....... ...C.....T .......... .......G.. .......... .....C..A. .C........
ECOR_71      ..T....... ...C...... .......... .......G.. ...A...... .......... ..........
SE11         ..T....... ...C.....T .......... .......G.. .......... .....C..A. .C........
O157_Sakai   ..T....... ...C...... .......... .......G.. ...A...... .......... ..........
ECOR_36      ..T....... ...C...... .......... .......G.. T......... .......... ..........
ECOR_35      ..T....... ...C...... .......... .......G.. T......... .......... ..........
SMS-3-5      ..T....... ...C...... .......... .......G.. T......... .......... ..........
536          ..T....... ...C...... .......... .......G.. T......... .......... ..........
APEC_O1      ..T....... ...C...... .......... .......G.. T......... .......... ..........
CFT073       ..T....... ...C...... .......... .......G.. T......... .......... ..........
E2348/69     ..T....... ...C...... .......... .......G.. T......... .......... ..........
ECOR_51      ..T....... ...C...... .......... .......G.. T......... .......... ..........
ECOR_57      ..T....... ...C...... .......... .......G.. T......... .......... ..........
UTI89        ..T....... ...C...... .......... .......G.. T......... .......... ..........
E._albertii  .......... ...C...... .......... ....C..G.. .........G .....C.... .C........

        20301
K-12         AGATTTCTGC AAACGTCAGG GCAAGAATTT TGATATCGAC GAACTGGAAG CCCTGCATCA GGAACTGGAA
53638        .......... .......... .......... .......... .......... .......... ..........
ATCC_8739    .......... .......... .......... .......... .......... .......... ..........
ECOR_10      .......... .......... .......... .......... .......... .......... ..........
ECOR_11      .......... .......... .......... .......... .......... .......... ..........
B171         ...C...... .......... .......... ......T..T .......... .T........ ...G......
E24377A      ...C...... .......... .......... ......T..T .......... .T........ ...G......
ECOR_34      ...C...... .......... .......... ......T..T .......... .T........ ...G......
ECOR_71      .......... .......... .......... ...C..T... ..G....... .T........ ...G......
SE11         ...C...... .......... .......... ......T..T .......... .T........ ...G......
O157_Sakai   .......... .......... .......... ...C..T... ..G....... .......... ...G......
ECOR_36      .......... .......... .......... ...C..T... ..G....... .T........ ...G......
ECOR_35      .......... .......... .......... ...C..T... ..G....... .T........ ...G......
SMS-3-5      .......... .......... .......... ...C..T... ..G....... .T........ ...G......
536          .......... .......... .......... ......T..T .......... .T........ ...G......
APEC_O1      .......... .......... .......... ......T..T .......... .T........ ...G......
CFT073       .......... .......... .......... ......T..T .......... .T........ ...G......
E2348/69     .......... .......... .......... ......T..T .......... .T........ ...G......
ECOR_51      .......... .......... .......... ......T..T .......... .T........ ...G......
ECOR_57      .......... .......... .......... ......T..T .......... .T........ ...G......
UTI89        .......... .......... .......... ......T..T .......... .T........ ...G......
E._albertii  .......... .......... .......... .......... ..G....... .......... ......C...
```

```
        20371
K-12          GCACGCATTG CCTCTCTTTC CGATAGCGTG TCTAACGCCC GTGAAGAGCG CATGGCACTG CGCCAGGAGC
53638         .......... .......... .......... .......... .......... .......... ..........
ATCC_8739     .......... .......... .......... .......... .......... .......... ..........
ECOR_10       .......... .......... .......... .......... .......... .......... ..........
ECOR_11       .......... .......... .......... .......... .......... .......... ..........
B171          ..G....... .......... .......... .......... .......... .......... ........A.
E24377A       ..G....... .......... .......... .......... .......... .......... ........A.
ECOR_34       ..G....... .......... .......... .......... .......... .......... ........A.
ECOR_71       ..G....... .......... .......... .......... .......... .......... ........A.
SE11          ..G....... .......... .......... .......... .......... .......... ........A.
O157_Sakai    .......... .......... .......... .......... .......... G......... ..........
ECOR_36       .......... .......... .......... .......... .......... G...A..... ..........
ECOR_35       .......... .......... .......... .......... .......... G...A..... ..........
SMS-3-5       .......... .......... .......... .......... .......... G...A..... ..........
536           .......... .......... .........C .......... .A........ G......... ..........
APEC_O1       .......... .......... .........C .......... .A........ G......... ..........
CFT073        .......... .......... .........C .......... .A........ G......... ..........
E2348/69      .......... .......... .........C .......... .A........ G......... ..........
ECOR_51       .......... .......... .........C .......... .A........ G......... ..........
ECOR_57       .......... .......... .........C .......... .A........ G......... ..........
UTI89         .......... .......... .........C .......... .A........ G......... ..........
E._albertii   .....T..A. .T..A..... T........T ..C.GT.... .......... G.....G... ..T.....A.

        20441
K-12          AGGAACAGCT GCAGTCTCGC ATTCAGAGTT TGATGCAGCG TGCGCCGGTT TGGCTGGCAG CGCAAAACAG
53638         .......... .......... .......... .......... .......... .......... ..........
ATCC_8739     .......... .......... .......... .......... .......... .......... ..........
ECOR_10       .......... .......... .......... .......... .......... .......... ..........
ECOR_11       .......... .......... .......... .......... .......... .......... ..........
B171          .......... .......... .......... .......... .......... .......... ..........
E24377A       .......... .......... .......... .......... .......... .......... ..........
ECOR_34       .......... .......... .......... .......... .......... .......... ..........
ECOR_71       .......... .......... .......... .......... .......... .......... ..........
SE11          .......... .......... .......... .......... .......... .......... ..........
O157_Sakai    .......... .......... .......... .......... ...A...... .......... ..........
ECOR_36       .......... .......... .......... ......G... .......... ........T. ..........
ECOR_35       .......... .......... .......... ......G... .......... ........T. ..........
SMS-3-5       .......... .......... .......... ......G... .......... ........T. ..........
536           .......... .......... .......... .......... .......... .......... ..........
APEC_O1       .......... .......... .......... .......... .......... .......... ..........
CFT073        .......... .......... .......... .......... .......... .......... ..........
E2348/69      .......... T......... .......... .......... .......... .......... ..........
ECOR_51       .......... .......... .......... .......... .......... .......... ..........
ECOR_57       .......... .......... .......... .......... .......... .......... ..........
UTI89         .......... .......... .......... .......... .......... .......... ..........
E._albertii   .......A.. .......... .........C .......... .......A.. ........C. ....G.....

        20511
K-12          TCTCAACCAG TTGAGCGAAC AGTGCGGCGA AGAGTTTACC TCCAGCCAGG ACGTCACCGA ATATCTGCAA
53638         .......... .......... .......... .......... .......... .......... ..........
ATCC_8739     .......... .......... .......... .......... .......... .......... ..........
ECOR_10       .......... .......... .......... .......... .......... .......... ..........
ECOR_11       .......... .......... .......... .......... .......... .......... ..........
B171          .......... ........G. .......T.. .......... .......... .......... ..........
E24377A       .......... ........G. .......T.. .......... .......... .......... ..........
ECOR_34       .......... ........G. .......T.. .......... .......... .......... ..........
ECOR_71       .......... .......... .......... .......... .......... .......... ..........
SE11          .......... ........G. .......T.. .......... .......... .......... ..........
O157_Sakai    .......... .......... .......... ......C... .......... .......T.. ..........
ECOR_36       .......... .......... .......... ......CT.. .......... .T........ ..........
ECOR_35       .......... .......... .......... ......CT.. .......... .T........ ..........
SMS-3-5       .......... .......... .......... ......CT.. .......... .T........ ..........
536           .......... .......... .......... ......C... .......... .T........ ..T.......
APEC_O1       .......... .......... .......... ......C... .......... .T........ ..T.......
CFT073        .......... .......... .......... ......C... .......... .T........ ..T.......
E2348/69      .......... .......... .......... ......C... .......... .T........ ..T.......
ECOR_51       .......... .......... .......... ......C... .......... .T........ ..T.......
ECOR_57       .......... .......... .......... ......C... .......... .T........ ..T.......
UTI89         .......... .......... .......... ......C... .......... .T........ ..T.......
E._albertii   .........A .......... .......T.. ...A..C... ........A. ....T..... ..........
```

```
         20581
K-12          CAGTTGCTGG AGCGTGAGCG AGAGGCGATT GTTGAACGCG ATGAAGTGGG CGCGCGCAAA AACGCCGTCG
53638         .......... .......... .......... .......... .......... .......... ..........
ATCC_8739     .......... .......... .......... .......... .......... .......... ..........
ECOR_10       .......... .......... .......... .......... .......... .......... ..........
ECOR_11       .......... .......... .......... .......... .......... .......... ..........
B171          .......... .......... .......... .......... .......... .......... .....T....
E24377A       .......... .......... .......... .......... .......... .......... .....T....
ECOR_34       .......... .......... .......... .......... .......... .......... .....T....
ECOR_71       .......... .......... .......... .......... .......... .......... ..........
SE11          .......... .......... .......... .......... .......... .......... .....T....
O157_Sakai    .......... .......... C.....C... .......... .......A.. .......... ..........
ECOR_36       .......... .......... C..A..C... .......... .......A.. .......... ..........
ECOR_35       .......... .......... C..A..C... .......... .......A.. .......... ..........
SMS-3-5       .......... .......... C..A..C... .......... .......A.. .......... ..........
536           .......... ....C..... C.....A... .......... .......A.. .......... ..........
APEC_O1       .......... .......... C.....A... .......... .......A.. .......... ..........
CFT073        .......... ....C..... C.....A... .......... .......A.. .......... ..........
E2348/69      .......... .......... C.....A... .......... .......A.. .......... ..........
ECOR_51       .......... ....C..... C.....A... .......... .......A.. .......... ..........
ECOR_57       .......... ....C..... C.....A... .......... .......A.. .......... ..........
UTI89         .......... .......... C.....A... .......... .......A.. .......... ..........
E._albertii   .......... ....C..... C.....C... ........T. .......... .......... ..........

         20651
K-12          ATGAAGAGAT CGAACGTTTA AGCCAGCCTG GCGGCTCTGA AGATCAGCGT CTGAACGCGC TGGCGGAGCG
53638         .......... .......... .......... .......... .......... .......... ..........
ATCC_8739     .......... .......... .......... .......... .......... .......... ..........
ECOR_10       .......... .......... .......... .......... .......... .......... ..........
ECOR_11       .......... .......... .......... .......... .......... .......... ..........
B171          .......... .......... .......... .......... .......... .......... ..........
E24377A       .......... .......... .......... .......... .......... .......... ..........
ECOR_34       .......... .......... .......... .......... .......... .......... ..........
ECOR_71       .......... .......... .......... .......... .......... .......... ..........
SE11          .......... .......... .......... .......... .......... .......... ..........
O157_Sakai    .......... .......... .......... .......... .......... .......... .A........
ECOR_36       .......... .......... ........A. .T..T..... ...C...... .....T.... ..........
ECOR_35       .......... .......... ........A. .T..T..... ...C...... .....T.... ..........
SMS-3-5       .......... .......... ........A. .T..T..... ...C...... .....T.... ..........
536           .......... .......... ........A. .T..T..... .......... .......... ..........
APEC_O1       .......... .......... ........A. .T..T..... .......... .......... ..........
CFT073        .......... .......... ........A. .T..T..... .......... .......... ..........
E2348/69      .......... .......... ........A. .T..T..... .......... .......... ..........
ECOR_51       .......... .......... ........A. .T..T..... .......... .......... ..........
ECOR_57       .......... .......... ........A. .T..T..... .......... .......... ..........
UTI89         .......... .......... ........A. .T..T..... .......... .......... ..........
E._albertii   .......... T..G...... ........A. ....T..... .......... .....T..T. ....A..A..

         20721
K-12          TTTTGGTGGT GTGCTGCTGT CAGAAATTTA TGACGACGTT AGCCTGGAAG ATGCGCCGTA CTTCTCAGCG
53638         .......... .......... .......... .......... .......... .......... ..........
ATCC_8739     .......... .......... .......... .......... .......... .......... ..........
ECOR_10       .......... .......... .......... .......... .......... .......... ..........
ECOR_11       .......... .......... .......... .......... .......... .......... ..........
B171          ......C..C .......... .......... .......... .......... .......... ..........
E24377A       ......C..C .......... .......... .......... .......... .......... ..........
ECOR_34       ......C..C .......... .......... .......... .......... .......... ..........
ECOR_71       .......... .......... .......... .......... .......... .......... ..........
SE11          ......C..C .......... .......... .......... .......... .......... ..........
O157_Sakai    .......... .......... .......... .......... .......... .......... ..........
ECOR_36       ......C..C .......... .......... .......... .......... .......... ..........
ECOR_35       ......C..C .......... .......... .......... .......... .......... ..........
SMS-3-5       ......C..C .......... .......... .......... .......... .......... ..........
536           .......... .......... .......... .......... .......... .......... .........A
APEC_O1       .......... .......... .......... .......... .......... .......... ..........
CFT073        .......... .......... .......... .......... .......... .......... .........A
E2348/69      .......... .......... .......... .......... .......... .......... ..........
ECOR_51       .......... .......... .......... .......... .......... .......... .........A
ECOR_57       .......... .......... .......... .......... .......... .......... .........A
UTI89         .......... .......... .......... .......... .......... .......... ..........
E._albertii   G......... ...T...... .......... ...T..T..C .......... ....A..A.. T.....G...
```

```
         20791
K-12          CTGTATGGCC CGTCACGCCA CGCCATCGTG GTGCCAGATC TGTCACAGGT AACTGAACAC CTGGAAGGCT
53638         .......... .......... .......... .......... .......... .......... ..........
ATCC_8739     .......... .......... .......... .......... .......... .......... ..........
ECOR_10       .......... .......... .......... .......... .......... .......... ..........
ECOR_11       .......... .......... .......... .......... .......... .......... ..........
B171          .......... .......... .......... .......... .......... .......... ..........
E24377A       .......... .......... .......... .......... .......... .......... ..........
ECOR_34       .......... .......... .......... .......... .......... .......... ..........
ECOR_71       .......... .......... .......... .......... .......... .......... ..........
SE11          .......... .......... .......... .......... .......... .......... ..........
O157_Sakai    .......... .......... .......... .......... .......... ...C...... ........T.
ECOR_36       .......... .......... .......... .......... .......... ...C...... ..........
ECOR_35       .......... .......... .......... .......... .......... ...C...... ..........
SMS-3-5       .......... .......... .......... .......... .......... ...C...... ..........
536           .......... .......... .......... .......... .......... ...C...... ..........
APEC_O1       .......... .......... .......... .......... .......... ...C...... ..........
CFT073        .......... .......... .......... .......... .......... ...C...... ..........
E2348/69      .......... .......... .......... .......... .......... ...C...... ..........
ECOR_51       .......... .......... .......... .......... .......... ...C...... ..........
ECOR_57       .......... .......... .......... .......... .......... ...C...... ..........
UTI89         .......... .......... .......... .......... .......... ...C...... ..........
E._albertii   .......... ....T..... ......T... ..A..G.... ....G..A.. TG.......A ........T.

         20861
K-12          TGACCGATTG CCCGGAAGAT CTCTATCTGA TCGAAGGAGA TCCGCAGTCA TTCGATGACA GCGTGTTCAG
53638         .......... .......... .......... .......... .......... .......... ..........
ATCC_8739     .......... .......... .......... .......... .......... .......... ..........
ECOR_10       .......... .......... .......... .......... .......... .......... ..........
ECOR_11       .......... .......... .......... .......... .......... .......... ..........
B171          .......... .......... ......T... .......G.. .......... .......... ..........
E24377A       .......... .......... ......T... .......G.. .......... .......... ..........
ECOR_34       .......... .......... .......... .......... .......... .......... ..........
ECOR_71       .......... .......... .......... .......... .......... .......... ..........
SE11          .......... .......... ......T... .......G.. .......... .......... ..........
O157_Sakai    .......... .......... ......T... .......G.. .......... .......... ..........
ECOR_36       .......... .......... .......... .......G.. .......... .......... ..........
ECOR_35       .......... .......... .......... .......G.. .......... .......... ..........
SMS-3-5       .......... .......... .......... .......G.. .......... .......... ..........
536           .......... .......... ......T... .......G.. .......... .......... ..........
APEC_O1       .......... .......... .......... .......G.. .......... .......... ..........
CFT073        .......... .......... ......T... .......G.. .......... .......... ..........
E2348/69      .......... .......... ......T... .......G.. .......... .......... ..........
ECOR_51       .......... .......... ......T... .......G.. .......... .......... ..........
ECOR_57       .......... .......... ......T... .......G.. .......... .......... ..........
UTI89         .......... .......... .......... .......G.. .......... .......... ..........
E._albertii   .A........ .......... .....CT... .......G.. .........G ..T....... ..........

         20931
K-12          CGTTGATGAG CTGGAAAAAG CGGTAGTGGT GAAAATCGCC GATCGTCAGT GGCGTTATTC ACGTTTCCCG
53638         .......... .......... .......... .......... .......... .......... ..........
ATCC_8739     .......... .......... .......... .......... .......... .......... ..........
ECOR_10       .......... .......... .......... .......... .......... .......... ..........
ECOR_11       .......... .......... .......... .......... .......... .......... ..........
B171          .......... .......... .......... T......... .......... .......... ..........
E24377A       .......... .......... .......... .......... .......... .......... ..........
ECOR_34       .......... .......... .......... .......... .......... .......... ..........
ECOR_71       .......... .......... .......... .......... .......... .......... ..........
SE11          .......... .......... .......... .......... .......... .......... ..........
O157_Sakai    .......... .......... .......... .......... .......... .......... ..........
ECOR_36       .......... .......... ....G..... .......... .......... .......... ..........
ECOR_35       .......... .......... ....G..... .......... .......... .......... ..........
SMS-3-5       .......... .......... ....G..... .......... .......... .......... ..........
536           .......... .......... ....G..... .......... .......... .......... ..........
APEC_O1       .......... .......... ....G..... .......... .......... .......... ..........
CFT073        .......... .......... ....G..... .......... .......... .......... ..........
E2348/69      .......... .......... ....G..... .......... .......... .......... ..........
ECOR_51       .......... .......... ....G..... .......... .......... .......... ..........
ECOR_57       .......... .......... ....G..... .......... .......... .......... ..........
UTI89         .......... .......... ....G..... .......... .......... .......... ..........
E._albertii   .......... .......... .......... .......... .....C.... .......C.. C..C......
```

```
          21001
K-12          GAAGTGCCGC TGTTTGGTCG TGCTGCGCGT GAAAGCCGTA TTGAAAGCCT CCATGCCGAG CGTGAAGTGC
53638         .......... .......... .......... .......... .......... .......... ..........
ATCC_8739     .......... .......... .......... .......... .......... .......... ..........
ECOR_10       .......... .......... .......... .......... .......... .......... ..........
ECOR_11       .......... .......... .......... .......... .......... .......... ..........
B171          .......... .......... .......... .......... .........G .......... ..........
E24377A       .......... .......... .......... .......... .......... .......... ..........
ECOR_34       .......... .......... .......... .......... .......... .......... ..........
ECOR_71       .......... .......... .......... .......... .......... .......... ..........
SE11          .......... .......... .......... .......... .......... .......... ..........
O157_Sakai    .......... .......... .......... .......... .......... .......... ..........
ECOR_36       .......... .......... .......... .......... .......... .......... ..........
ECOR_35       .......... .......... .......... .......... .......... .......... ..........
SMS-3-5       .......... .......... .......... .......... .......... .......... ..........
536           .......... .......... .......... .......... .......... .......... ..G.......
APEC_O1       .......... .......... .......... .......... .......... .......... ..G.......
CFT073        .......... .......... .......... .......... .......... .......... ..G.......
E2348/69      .......... .......... .......... .......... .......... .......... ..G.......
ECOR_51       .......... .......... .......... .......... .......... .......... ..G.......
ECOR_57       .......... .......... .......... .......... .......... .......... ..G.......
UTI89         .......... .......... .......... .......... .......... .......... ..G.......
E._albertii   .........T .......... ...G...... .....T.... .C.....T.. T......... ..........

          21071
K-12          TTTCCGAACG CTTCGCCACG CTCTCCTTTG ATGTACAGAA AACTCAGCGT CTGCATCAGG CGTTCAGCCG
53638         .......... .......... .......... .......... .......... .......... ..........
ATCC_8739     .......... .......... .......... .......... .......... .......... ..........
ECOR_10       .......... .......... .......... .......... .......... .......... ..........
ECOR_11       .......... .......... .......... .......... .......... .......... ..........
B171          .......... .......... .......... .......... .......... .......... ..........
E24377A       .......... .......... .......... .......... .......... .......... ..........
ECOR_34       .......... .......... .......... .......... .......... .......... ..........
ECOR_71       .......... .......... .......... .......... .......... .......... ..........
SE11          .......... .......... .......... .......... .......... .......... ..........
O157_Sakai    .......... .......... .......... .......... ...C...... .......... ..........
ECOR_36       .......... .......... .......... .......... ...C...... .......... ..........
ECOR_35       .......... .......... .......... .......... ...C...... .......... ..........
SMS-3-5       .......... .......... .......... .......... ...C...... .......... ..........
536           .......... .......... .......... .......... ...C...... .......... ..........
APEC_O1       .......... .......... .......... .......... ...C...... .......... ..........
CFT073        .......... .......... .......... .......... ...C...... .......... ..........
E2348/69      .......... .......... .......... .......... ..TC...... .......... ..........
ECOR_51       .......... .......... .......... .......... ...C...... .......... ..........
ECOR_57       .......... .......... .......... .......... ...C...... .......... ..........
UTI89         .......... .......... .......... .......... ...C...... .......... ..........
E._albertii   .......... T.....T... .......... .......... ...C...... .......... ..........

          21141
K-12          CTTTATCGGC AGTCATCTGG CGGTTGCGTT TGAGTCTGAC CCGGAAGCAG AAATCCGTCA ACTGAACAGC
53638         .......... .......... .......... .......... .......... .......... ..........
ATCC_8739     .......... .......... .......... .......... .......... .......... ..........
ECOR_10       .......... .......... .......... .......... .......... .......... ..........
ECOR_11       .......... .......... .......... .......... .......... .......... ..........
B171          .......... .......... .......... .......... .......... .......... ..........
E24377A       .......... .......... .......... .........T .......... .......... ..........
ECOR_34       .......... .......... .......... .......... .......... .......... ..........
ECOR_71       .......... .......... .......... .......... .......... .......... ..........
SE11          .......... .......... .......... .......... .......... .......... ..........
O157_Sakai    .......... .......... ....G..... .......... .......... .......... ..........
ECOR_36       .......... ..C..C.... ....G..... .........T ........C. .......... ..........
ECOR_35       .......... ..C..C.... ....G..... .........T ........C. .......... ..........
SMS-3-5       .......... ..C..C.... ....G..... .........T ........C. .......... ..........
536           .......... .......... ....G..... .......... .......... .......... ..........
APEC_O1       .......... .......... ....G..... .......... .......... .......... ..........
CFT073        .......... .......... ....G..... .......... .......... .......... ..........
E2348/69      .......... .......... ....G..... .......... .......... .......... ..........
ECOR_51       .......... .......... ....G..... .......... .......... .......... ..........
ECOR_57       .......... .......... ....G..... .......... .......... .......... ..........
UTI89         .......... .......... ....G..... .......... .......... .......... ..........
E._albertii   .......... ..C....... .A..G..... .......... ........C. .......... ...A......
```

```
        21211
K-12        CGTCGCGTCG AACTGGAGCG GGCGTTAAGT AATCATGAAA ATGATAACCA GCAGCAGCGT ATTCAGTTTG
53638       .......... .......... .......... .......... .......... .......... ..........
ATCC_8739   .......... .......... .......... .......... .......... .......... ..........
ECOR_10     .......... .......... .......... .......... .......... .......... ..........
ECOR_11     .......... .......... .......... .......... .......... .......... ..........
B171        .......... .......... ......G... .......... .......... .......... ..........
E24377A     .......... .......... ......G... .......... .......... .......... ..........
ECOR_34     .......... .......... ......G... .......... .......... .......... ..........
ECOR_71     .......... .......... ......G... .......... .......... .......... ..........
SE11        .......... .......... ......G... .......... .......... .......... ..........
O157_Sakai  .......... .......... ......G... .......... .......... .......... ..........
ECOR_36     .......... .......... ......G... .......... .......... .......... ..........
ECOR_35     .......... .......... ......G... .......... .......... .......... ..........
SMS-3-5     .......... .......... ......G... .......... .......... .......... ..........
536         .......... .......A.. ......G... .......... .......... .......... ..........
APEC_O1     .......... .......A.. ......G... .......... .......... .......... ..........
CFT073      .......... .......A.. ......G... .......... .......... .......... ..........
E2348/69    .......... .......A.. ......G... .......... .......... .......... ..........
ECOR_51     .......... .......A.. ......G... .......... .......... .......... ..........
ECOR_57     .......... .......A.. ......G... .......... .......... .......... ..........
UTI89       .......... .......A.. ......G... .......... .......... .......... ..........
E._albertii .......... .......... ......G... .......... .......... .......... ..........

        21281
K-12        AGCAGGCGAA AGAGGGCGTT ACGGCGCTGA ACCGCATTCT GCCGCGTCTC AACCTGTTGG CTGATGACAG
53638       .......... .......... .......... .......... .......... .......... ..........
ATCC_8739   .......... .......... .......... .......... .......... .......... ..........
ECOR_10     .......... .......... .......... .......... .......... .......... ..........
ECOR_11     .......... .......... .......... .......... .......... .......... ..........
B171        .......... ...A...... .......... .......... .......... .......... ..........
E24377A     .......... ...A...... .......... .......... .......... .......... ..........
ECOR_34     .......... ...A...... .......... .......... .......... .......... ..........
ECOR_71     .......... ...A...... .......... .......... .......... .......... ..........
SE11        .......... ...A...... .......... .......... .......... .......... ..........
O157_Sakai  .......... ...A...... .......... .......... .......... .......... ..........
ECOR_36     .......... ......T... .......... .......... .......... ...T...... ..........
ECOR_35     .......... ......T... .......... .......... .......... ...T...... ..........
SMS-3-5     .......... ......T... .......... .......... .......... ...T...... ..........
536         .......... .......... .......... .......... .......... .......... ..........
APEC_O1     .......... .......... .......... .......... .......... .......... ..........
CFT073      .......... .......... .......... .......... .......... .......... ..........
E2348/69    .......... .......... .......... .......... .......... .......... ..........
ECOR_51     .......... .......... .......... .......... .......... .......... ..........
ECOR_57     .......... .......... .......... .......... .......... .......... ..........
UTI89       .......... .......... .......... .......... .......... .......... ..........
E._albertii .......... .......... .......... .......... .......... .......... ..........

        21351
K-12        CCTGGCGGAT CGCGTCGATG AAATCCGCGA ACGTCTGGAT GAAGCCCAGG AAGCCGCGCG TTTTGTTCAG
53638       .......... .......... .......... .......... .......... .......... ..........
ATCC_8739   .......... .......... .......... .......... .......... .......... ..........
ECOR_10     .......... .......... .......... .......... .......... .......... ..........
ECOR_11     .......... .......... .......... .......... .......... .......... ..........
B171        .......... .......... .......... .......... .......... .......... ..........
E24377A     .......... .......... .......... .......... .......... .......... ..........
ECOR_34     .......... .......... .......... .......... .......... .......... ..........
ECOR_71     .......... .......... .......... .......... .......... .......... ..........
SE11        .......... .......... .......... .......... .......... .......... ..........
O157_Sakai  .......... .......... .......... .......... .......... .......... ..........
ECOR_36     .......... .......... .......... .......... .......... .......... ..........
ECOR_35     .......... .......... .......... .......... .......... .......... ..........
SMS-3-5     .......... .......... .......... .......... .......... .......... ..........
536         .......... .......... .......... .......... .......... .......... ..........
APEC_O1     .......... .......... .......... .......... .......... .......... ..........
CFT073      .......... .......... .......... .......... .......... .......... ..........
E2348/69    .......... .......... .......... .......... .......... .......... ..........
ECOR_51     .......... .......... .......... .......... .......... .......... ..........
ECOR_57     .......... .......... .......... .......... .......... .......... ..........
UTI89       .......... .......... .......... .......... .......... .......... ..........
E._albertii .......... .......... .......... .......... .......... ....G..... ..........
```

```
          21421
K-12         CAGTTTGGCA ATCAACTGGC GAAACTGGAA CCGATTGTTT CGGTATTGCA GAGCGACCCG GAACAGTTCG
53638        .......... .......... .......... .......... .......... .......... ..........
ATCC_8739    .......... .......... .......... .......... .......... .......... ..........
ECOR_10      .......... .......... .......... .......... .......... .......... ..........
ECOR_11      .......... .......... .......... .......... .......... .......... ..........
B171         .......... .......... .......... .....C.... .......... .......... ..........
E24377A      .......... .......... .......... .....C.... .......... .......... ..........
ECOR_34      .......... .......... .......... .....C.... .......... .......... ..........
ECOR_71      .......... .......... .......... .....C.... .......... .......... ..........
SE11         .......... .......... .......... .....C.... .......... .......... ..........
O157_Sakai   .......... .......... .......... .....C.... .......... .......... ..........
ECOR_36      .....C..T. .......... .......... .....C.... .......... ...T...... ..........
ECOR_35      .....C..T. .......... .......... .....C.... .......... ...T...... ..........
SMS-3-5      .....C..T. .......... .......... .....C.... .......... ...T...... ..........
536          .......... .......... .......... .....C.... .......... .......... ..........
APEC_O1      .......... .......... .......... .....C.... .......... .......... ..........
CFT073       .......... .......... .......... .....C.... .......... .......... ..........
E2348/69     .......... .......... .......... .....C.... .......... .......... ..........
ECOR_51      .......... .......... .......... .....C.... .......... .......... ..........
ECOR_57      .......... .......... .......... .....C.... .......... .......... ..........
UTI89        .......... .......... .......... .....C.... .......... .......... ..........
E._albertii  .......... .......... .......... .....C.... .......... .......... ..........

          21491
K-12         AACAGTTAAA AGAAGATTAC GCGTACTCTC AGCAGATGCA GCGTTCTGAC TCGGCAGAAA TGCTTAGCGG
53638        .......... .......... .......... .......... .......... .......... ..........
ATCC_8739    .......... .......... .......... .......... .......... .......... ..........
ECOR_10      .......... .......... .......... .......... .......... .......... ..........
ECOR_11      .......... .......... .......... .......... .......... .......... ..........
B171         .....C.... .......... .......... .......... .......... .......... ....G.....
E24377A      .....C.... .......... .......... .......... .......... .......... ....G.....
ECOR_34      .....C.... .......... .......... .......... .......... .......... ....G.....
ECOR_71      .....C.... .......... .......... .......... .......... .......... ....G.....
SE11         .....C.... .......... .......... .......... .......... .......... ....G.....
O157_Sakai   .......... .......... .......... .......... ...C...... .......... ..........
ECOR_36      .......... .......... .......... .......... ...C...... .......... ..........
ECOR_35      ....A..... .......... .......... .......... ...C...... .......... ..........
SMS-3-5      ....A..... .......... .......... .......... ...C...... .......... ..........
536          .......... .......... .......... .......... ...C...... .......... ..........
APEC_O1      .......... .......... .......... .......... ...C...... .......... ..........
CFT073       .......... .......... .......... .......... ...C...... .......... ..........
E2348/69     .......... .......... .......... .......... ...C...... .......... ..........
ECOR_51      .......... .......... .......... .......... ...C...... .......... ..........
ECOR_57      .......... .......... .......... .......... ...C...... .......... ..........
UTI89        .......... .......... .......... .......... ...C...... .......... ..........
E._albertii  .......... .......... .......... .......... ...C...... .......... ....C.....

          21561
K-12         TAACAGCGAT CTCAACGAAA AACTGCGTGA ACGTCTGGAA CAGGCGGAAG CGGAGCGTAC CCGCGCTCGC
53638        .......... .......... .......... .......... .......... .......... ..........
ATCC_8739    .......... .......... .......... .......... .......... .......... ..........
ECOR_10      .......... .......... .......... .......... .......... .......... ..........
ECOR_11      .......... .......... .......... .......... .......... .......... ..........
B171         ...T...... .......... .G........ .......... .......... .......... T........T
E24377A      ...T...... .......... .G........ .......... .......... .......... T........T
ECOR_34      ...T...... .......... .G........ .......... .......... .......... T........T
ECOR_71      ...T...... .......... .G........ .......... .......... .......... T........T
SE11         ...T...... .......... .G........ .......... .......... .......... T........T
O157_Sakai   .......... .......... .......... .......... .......... .......... ..........
ECOR_36      .......... .......... .......... .......... .......... .......... .........T
ECOR_35      .......... .......... .......... .......... .......... .......... ......G..T
SMS-3-5      .......... .......... .......... .......... .......... .......... ......G..T
536          .......... .......... .......... .......... .......... .......... .........T
APEC_O1      .......... .......... .......... .......... .......... .......... .........T
CFT073       .......... .......... .......... .......... .......... .......... .........T
E2348/69     .......... .......... .......... .......... .......... .......... .........T
ECOR_51      .......... .......... .......... .......... .......... .......... .........T
ECOR_57      .......... .......... .......... .......... .......... .......... .........T
UTI89        .......... .......... .......... .......... .......... .......... .........T
E._albertii  ...T...... .......... .......... G......... ..T..A.... .A........ T.....C..T
```

```
         21631
K-12          GAAGCGTTGC GCGGTCACGC AGCGCAGTTG AGTCAGTACA ACCAGGTGCT GGCTTCGCTG AAAAGTTCTT
53638         .......... .......... .......... .......... .......... .......... ..........
ATCC_8739     .......... .......... .......... .......... .......... .......... ..........
ECOR_10       .......... .......... .......... .......... .......... .......... ..........
ECOR_11       .......... .......... .......... .......... .......... .......... ..........
B171          .......... .T........ .......... .......... .......... .......... ..........
E24377A       .......... .T........ .......... .......... .......... .......... ..........
ECOR_34       .......... .T........ .......... .......... .......... .......... ..........
ECOR_71       .......... .T........ .......... .......... .......... .......... ..........
SE11          .......... .T........ .......... .......... .......... .......... ..........
O157_Sakai    .......... .......... .......... .......... .......... .......... ..........
ECOR_36       .......... ....A..... .......... .A........ .......... .......... ........G.
ECOR_35       .......... ....A..... .......... .......... .......... .......... ..........
SMS-3-5       .......... ....A..... .......... .......... .......... .......... ..........
536           .......... ....A..... .......... .A........ .......... .......... ........G.
APEC_O1       .......... ....A..... .......... .A........ .......... .......... ........G.
CFT073        .......... ....A..... .......... .A........ .......... .......... ........G.
E2348/69      .......... ....A..... .......... .A........ .......... .......... ........G.
ECOR_51       .......... ....A..... .......... .A........ .......... .......... ........G.
ECOR_57       .......... ....A..... .......... .A........ .......... .......... ........G.
UTI89         .......... ....A..... .......... .A........ .......... .......... ........G.
E._albertii   ......C... .......T.. CA.T...... .AC....... .......... ...A..A..A ..........

         21701
K-12          ACGACACCAA AAAAGAGCTA CTCAACGATC TGCAACGTGA ATTGCAGGAT ATCGGCGTGC GTGCTGATAG
53638         .......... .......... .......... .......... .......... .......... ..........
ATCC_8739     .......... .......... .......... .......... .......... .......... ..........
ECOR_10       .......... .......... .......... .......... .......... .......... ..........
ECOR_11       .......... .......... .......... .......... .......... .......... ..........
B171          .......... .......... .......... .......... .......... .......... .C..C.....
E24377A       .......... .......... .......... .......... .......... .......... .C..C.....
ECOR_34       .......... .......... .......... .......... .......... .......... .C..C.....
ECOR_71       .......... .......... .......... .......... .......... .......... ..........
SE11          .......... .......... .......... .......... .......... .......... .C..C.....
O157_Sakai    .......... .......... .......... .......... .......... .......... ..........
ECOR_36       .T........ .......... ..T....... .......... .......... .......... ..........
ECOR_35       .......... .......... .......... .......... .......... .......... ..........
SMS-3-5       .......... .......... .......... .......... .......... .......... ..........
536           .T........ .......... ..T....... .......... .......... .......... ..........
APEC_O1       .T........ .......... ..T....... .......... .......... .......... ..........
CFT073        .T........ .......... ..T....... .......... .......... .......... ..........
E2348/69      .T........ .......... ..T....... .......... .......... .......... ..........
ECOR_51       .T........ .......... ..T....... .......... .......... .......... ..........
ECOR_57       .T........ .......... ..T....... .......... .......... .......... ..........
UTI89         .T........ .......... ..T....... .......... .......... .......... ..........
E._albertii   .T........ .........G ..T....... .......C.. .C........ ..T....... ....C.....

         21771
K-12          CGGGGCAGAA GAGCGGGCGC GTATTCGCCG TGACGAGCTG CATGCGCAAC TGAGCAATAA CCGTTCACGC
53638         .......... .......... .......... .......... .......... .......... ..........
ATCC_8739     .......... .......... .......... .......... .......... .......... ..........
ECOR_10       .......... .......... .......... .......... .......... .......... ..........
ECOR_11       .......... .......... .......... .......... .......... .......... ..........
B171          .......... .......... .A........ .......... .......... ....T..... ...G..C...
E24377A       .......... .......... .A........ .......... .......... ....T..... ...G..C...
ECOR_34       .......... .......... .A........ .......... .......... ....T..... ...G..C...
ECOR_71       .......... .......... .......... .......... .......... .......... ..........
SE11          .......... .......... .A........ .......... .......... ....T..... ...G..C...
O157_Sakai    .......... ........A. .......... .......... .....A.... .......... ......G...
ECOR_36       .......... .......... .......... .......T.. ..C....... .......... ..........
ECOR_35       .......... .......... .......... .......T.. ..C....... .......... ..........
SMS-3-5       .......... .......... .......... .......T.. ..C....... .......... ..........
536           .......... ........A. .......... .......T.. ..C....... .......... .........A
APEC_O1       .......... ........A. .......... .......T.. ..C....... .......... .........A
CFT073        .......... ........A. .......... .......T.. ..C....... .......... .........A
E2348/69      .......... ........A. .......... .......T.. ..C....... .......... .........A
ECOR_51       .......... ........A. .......... .......T.. ..C....... .......... .........A
ECOR_57       .......... ........A. .......... .......T.. ..C....... .......... .........A
UTI89         .......... ........A. .......... .......T.. ..C....... .......... .........A
E._albertii   ...T...... .....C.... .......T.. ...T.....T ..C....... .......... ......T..T
```

```
        21841
K-12         CGCAATCAAC TGGAAAAAGC GCTTACCTTC TGCGAAGCGG AGATGGACAA CCTGACCCGC AAACTGCGCA
53638        .......... .......... .......... .......... .......... .......... ..........
ATCC_8739    .......... .......... .......... .......... .......... .......... ..........
ECOR_10      .......... .......... .......... .......... .......... .......... ..........
ECOR_11      .......... .......... .......... .......... .......... .......... ..........
B171         .....C..G. .......... A..G..T... .......... .......... T......... ..........
E24377A      .....C..G. .......... A..G..T... .......... .......... T......... ..........
ECOR_34      .....C..G. .......... A..G..T... .......... .......... T......... ..........
ECOR_71      .......... .......... .......... .......... .......... .......... ..........
SE11         .....C..G. .......... A..G..T... .......... .......... T......... ..........
O157_Sakai   .......... .......... ...C...... .......... .......T.. .......... ..........
ECOR_36      .......... .......... .......... ........A. .......... .......... ..........
ECOR_35      .......... .......... .......... ........A. .......... .......... ..........
SMS-3-5      .......... .......... .......... ........A. .......... .......... ..........
536          .......... .......... ......A... .......... .......... .........T ..........
APEC_O1      .......... .......... ......A... .......... .......... .........T ..........
CFT073       .......... .......... ......A... .......... .......... .........T ..........
E2348/69     .......... .......... .......... .......... .......... .........T ..........
ECOR_51      .......... .......... ......A... .......... .......... .........T ..........
ECOR_57      .......... .......... ......A... .......... .......... .........T ..........
UTI89        .......... .......... ......A... .......... .......... .........T ..........
E._albertii  .....C.... ....G..... ......T..T .......... .......... .........G ..........

        21911
K-12         AGCTGGAGCG GGATTACTTT GAGATGCGCG AGCAGGTAGT GACCGCCAAA GCGGGCTGGT GTGCGGTGAT
53638        .......... .......... .......... .......... .......... .......... ..........
ATCC_8739    .......... .......... .......... .......... .......... .......... ..........
ECOR_10      .......... .......... .......... .......... .......... .......... ..........
ECOR_11      .......... .......... .......... .......... .......... .......... ..........
B171         .......... .......... .......... .......... .......... .......... ....A.....
E24377A      .......... .......... .......... .......... .......... .......... ....A.....
ECOR_34      .......... .......... .......... .......... .......... .......... ....A.....
ECOR_71      .......... .......... .......... .......... .......... .......... ..........
SE11         .......... .......... .......... .......... .......... .......... ....A.....
O157_Sakai   .......... T......... .......... .......... ......G... .......... ....A..C..
ECOR_36      .......... .......... .......... .......... ......G... .......... ....A..C..
ECOR_35      .......... .......... .......... .......... ......G... .......... ....A..C..
SMS-3-5      .......... .......... .......... .......... ......G... .......... ....A..C..
536          .......... .......... .......... .......... .......... .......... ..........
APEC_O1      .......... .......... .......... .......... .......... .......... ..........
CFT073       .......... .......... .......... .......... .......... .......... ..........
E2348/69     .......... .......... .......... .......... .......... .......... ..........
ECOR_51      .......... .......... .......... .......... .......... .......... ..........
ECOR_57      .......... .......... .......... .......... .......... .......... ..........
UTI89        .......... .......... .......... .......... .......... .......... ..........
E._albertii  .......... T..C...CA. ..A....... .......G.. .......... .......... .......T..

        21981
K-12         GCGCATGGTG AAAGATAACG GCGTTGAGCG CCGCTTACAC CGTCGTGAGC TGGCTTATCT CTCCGCTGAT
53638        .......... .......... .......... .......... .......... .......... ..........
ATCC_8739    .......... .......... .......... .......... .......... .......... ..........
ECOR_10      .......... .......... .......... .......... .......... .......... ..........
ECOR_11      .......... .......... .......... .......... .......... .......... ..........
B171         .......... .......... .......A.. G......... .....C.... .......... ..........
E24377A      .......... .......... .......A.. G......... .....C.... .......... ..........
ECOR_34      .......... .......... .......A.. G......... .....C.... .......... ..........
ECOR_71      .......... .......... .......... .......... .......... .......... ..........
SE11         .......... .......... .......A.. G......... .....C.... .......... ..........
O157_Sakai   .......... .......... .......... .......... .......... .......... ..........
ECOR_36      .......... .......... .......... .......... .......... .......... ..........
ECOR_35      .......... .......... .......... .......... .......... .......... ..........
SMS-3-5      .......... .......... .T........ .......... .......... .......... ..........
536          .......... ........T. .......... T......... .......... .......... ..........
APEC_O1      .......... ........T. .......... T......... .......... .......... ..........
CFT073       .......... ........T. .......... T......... .......... .......... ..........
E2348/69     .......... ........T. .......... T......... .......... .......... ..........
ECOR_51      .......... ........T. .......... T......... .......... .......... ..........
ECOR_57      .......... ........T. .......... T......... .......... .......... ..........
UTI89        .......... ........T. .......... T......... .......... .......... ..........
E._albertii  .......... .......... .......... T........T .......... .......... ...T.....C
```

```
         22051
K-12           GATTTGCGTT CCATGTCGGA TAAGGCGTTA GGTGCGCTGC GTCTGGCGGT GGCGGATAAC GAACATCTGC
53638          .......... .......... .......... .......... .......... .......... ..........
ATCC_8739      .......... .......... .......... .......... .......... .......... ..........
ECOR_10        .......... .......... .......... .......... .......... .......... ..........
ECOR_11        .......... .......... .......... .......... .......... .......... ..........
B171           ........C. .T........ ......A..G .......... .......... .......... ..........
E24377A        ........C. .T........ ......A..G .......... .......... .......... ..........
ECOR_34        ........C. .T........ ......A..G .......... .......... .......... ..........
ECOR_71        ........C. .T........ ......A..G .......... .......... .......... ..........
SE11           ........C. .T........ ......A..G .......... .......... .......... ..........
O157_Sakai     .......... .......... .......... .......... .......... .......... ..........
ECOR_36        .......... .......... .......... .......... .......... .......... ..........
ECOR_35        .......... .......... .......... .......... .......... .......... ..........
SMS-3-5        .......... .......... .......... .......... .......... .......... ..........
536            .......... .......... .......... .......... .......... ...A...... ..........
APEC_O1        .......... .......... .......... .......... .......... ...A...... ..........
CFT073         .......... .......... .......... .......... .......... ...A...... ..........
E2348/69       .......... .......... .......... .......... .......... ...A...... ..........
ECOR_51        .......... .......... .......... .......... .......... ...A...... ..........
ECOR_57        .......... .......... .......... .......... .......... ...A...... ..........
UTI89          .......... .......... .......... .......... .......... ...A...... ..........
E._albertii    ...C...... .......... .........G ......T... .......... .........T ..........

         22121
K-12           GCGACGTGCT GCGCATGTCG GAAGATCCGA AACGTCCGGA GCGTAAAATT CAGTTCTTCG TGGCGGTTTA
53638          .......... .......... .......... .......... .......... .......... ..........
ATCC_8739      .......... .......... .......... .......... .......... .......... ..........
ECOR_10        .......... .......... .......... .......... .......... .......... ..........
ECOR_11        .......... .......... .......... .......... .......... .......... ..........
B171           .......... .......... ........A. .......... A......... .......... ..........
E24377A        .......... .......... ........A. .......... A......... .......... ..........
ECOR_34        .......... .......... ........A. .......... A......... .......... ..........
ECOR_71        .......... .......... .......... .......... .......... .......... ..........
SE11           .......... .......... ........A. .......... A......... .......... ..........
O157_Sakai     .......... .......... ........C. .......... .......... .......... ..........
ECOR_36        .......... ...T...... ........A. .......... A......... .......... ..........
ECOR_35        .......... ...T...... ........A. .......... A......... .......... ..........
SMS-3-5        .......... ...G...... .......... .......... .......... .......... ..........
536            .......... ...G...... ........A. .......... A......... .......... ..........
APEC_O1        .......... ...G...... ........A. .......... A......... .......... ..........
CFT073         .......... ...G...... ........A. .......... A......... .......... ..........
E2348/69       .......... ...G...... ........A. .......... A......... .......... ..........
ECOR_51        .......... ...G...... ........A. .......... A......... .......... ..........
ECOR_57        .......... ...G...... ........A. .......... A......... .......... ..........
UTI89          .......... ...G...... ........A. .......... A......... .......... ..........
E._albertii    .......A.. ...T...... ........A. ....C..... A......... .......... ..........

         22191
K-12           TCAGCATCTG CGTGAACGTA TTCGTCAGGA TATTATTCGT ACCGATGATC CGGTGGAAGC TATCGAACAG
53638          .......... .......... .......... .......... .......... .......... ..........
ATCC_8739      .......... .......... .......... .......... .......... .......... ..........
ECOR_10        .......... .......... .......... .......... .......... .......... ..........
ECOR_11        .......... .......... .......... .......... .......... .......... ..........
B171           .......... .......... .......... .......... .......... .......... C.....G...
E24377A        .......... .......... .......... .......... .......... .......... C.....G...
ECOR_34        .......... .......... .......... .......... .......... .......... C.....G...
ECOR_71        .......... .......... .......... .......... .......... .......... ..........
SE11           .......... .......... .......... .......... .......... .......... C.....G...
O157_Sakai     .......... .......... .......... .......... .......... .......... ..........
ECOR_36        .......... .......... .......... .......... .......... .......... ..........
ECOR_35        .......... .......... .......... .......... .......... .......... ..........
SMS-3-5        .......... ..C..G.... .......... .......... .......... ....T..... ......G...
536            .......... .......... .......... .......... .......... .......... ..........
APEC_O1        .......... .......... .......... .......... .......... .......... ..........
CFT073         .......... .......... .......... .......... .......... .......... ..........
E2348/69       .......... .......... .......... .......... .......... .......... ..........
ECOR_51        .......... .......... .......... .......... .......... .......... ..........
ECOR_57        .......... .......... .......... .......... .......... .......... ..........
UTI89          .......... .......... .......... .......... .......... .......... ..........
E._albertii    .......... ..C..G.... .......... .......... ..T....... .......... ......G...
```

```
        22261
K-12         ATGGAGATTG AACTTAGCCG TCTGACCGAA GAATTAACCT CCCGTGAACA GAAACTGGCG ATCAGTTCCC
53638        .......... .......... .......... .......... .......... .......... ..........
ATCC_8739    .......... .......... .......... .......... .......... .......... ..........
ECOR_10      .......... .......... .......... .......... .......... .......... ..........
ECOR_11      .......... .......... .......... .......... .......... .......... ..........
B171         .....A..C. .......... A........G ..G....... ....C..... .......... ..........
E24377A      .....A..C. .......... A........G ..G....... ....C..... .......... ..........
ECOR_34      .....A..C. .......... A........G ..G....... ....C..... .......... ..........
ECOR_71      ........C. .......... .......... .......... .......... .......... ..........
SE11         .....A..C. .......... A........G ..G....... ....C..... .......... ..........
O157_Sakai   ........C. .......... .......... ..G....... .......... .......... ..........
ECOR_36      ........C. .......... .......... ..G....... ....C..... .......... ..........
ECOR_35      ........C. .......... .......... ..G....... ....C..... .......... ..........
SMS-3-5      ........C. .......T.. ......T... ..G.....G. ....C..... .......... ..........
536          ........C. .G........ .......... .......... .......... .......... ..........
APEC_O1      ........C. .G........ .......... .......... .......... .......... ..........
CFT073       ........C. .G........ .......... .......... .......... .......... ..........
E2348/69     ........C. .G........ .......... .......... .......... .......... ..........
ECOR_51      ........C. .G........ .......... .......... .......... .......... ..........
ECOR_57      ........C. .G........ .......... .......... .......... .......... ..........
UTI89        ........C. .G........ .......... .......... .......... .......... ..........
E._albertii  ........C. .......... .......... ..GC....T. ....C..G.. .......... .....C..T.

        22331
K-12         GCAGCGTGGC GAACATCATT CGCAAAACCA TTCAGCGCGA GCAGAACCGT ATCCGTATGC TCAACCAGGG
53638        .......... .......... .......... .......... .......... .......... ..........
ATCC_8739    .......... .......... .......... .......... .......... .......... ..........
ECOR_10      .......... .......... .......... .......... .......... .......... ..........
ECOR_11      .......... .......... .......... .......... .......... .......... ..........
B171         .......... .......... .......... .......... .......... .......... ..........
E24377A      .......... .......... .......... .......... .......... .......... ..........
ECOR_34      .......... .......... .......... .......... .......... .......... ..........
ECOR_71      .......... .......... .......... .......... .......... .......... ..........
SE11         .......... .......... .......... .......... .......... .......... ..........
O157_Sakai   .......... .......... .......... .......... .......... .......... ..........
ECOR_36      .......... ......T... .......... .......... .......... .......... ..........
ECOR_35      .......... ......T... .......... .......... .......... .......... ..........
SMS-3-5      .......... .........C ..T....... .......... .......... .......... ..........
536          .......... .......... .......... .......... .......... .......... ..........
APEC_O1      .......... .......... .......... .......... .......... .......... ..........
CFT073       .......... .......... .......... .......... .......... .......... ..........
E2348/69     .......... .......... .......... .......... .......... .......... ..........
ECOR_51      .......... .......... .......... .......... .......... .......... ..........
ECOR_57      .......... .......... .......... .......... .......... .......... ..........
UTI89        .......... .......... .......... .......... .......... .......... ..........
E._albertii  .......... ......T... .......... .......... .......... ..T..C.... .T........

        22401
K-12         GTTGCAGAAC GTATCGTTTG GTCAGGTGAA CAGCGTGCGT CTCAACGTGA ACGTGCGTGA AACGCACGCC
53638        .......... .......... .......... .......... .......... .......... ..........
ATCC_8739    .......... .......... .......... .......... .......... .......... ..........
ECOR_10      .......... .......... .......... .......... .......... .......... ..........
ECOR_11      .......... .......... .......... .......... .......... .......... ..........
B171         .......... .......... .......... ......A... ..G....... .......... ..........
E24377A      .......... .......... .......... ......A... ..G....... .......... ..........
ECOR_34      .......... .......... .......... ......A... ..G....... .......... ..........
ECOR_71      .......... .......... .......... .........C .......... .......... ..........
SE11         .......... .......... .......... ......A... ..G....... .......... ..........
O157_Sakai   .......... .......... .......... .........C .......... .......... ..........
ECOR_36      C.......G. .......... .C........ .......... .......... .......... ..........
ECOR_35      C.......G. .......... .C........ .......... .......... .......... ..........
SMS-3-5      C......... ........C. .......... .........C .......... .......... ......T...
536          .......... .......... .C........ ......A... ..G....... ....A..... ..........
APEC_O1      .......... .......... .C........ ......A... ..G....... ....A..... ..........
CFT073       .......... .......... .C........ ......A... ..G....... ....A..... ..........
E2348/69     .......... .......... .C........ ......A... ..G....... ....A..... ..........
ECOR_51      .......... .......... .C........ ......A... ..G....... ....A..... ..........
ECOR_57      .......... .......... .C........ ......A... ..G....... ....A..... ..........
UTI89        .......... .......... .C........ ......A... ..G....... ....A..... ..........
E._albertii  C.......GT .......... .......T.. .......... .......... .......... ...T......
```

```
        22471
K-12          ATGCTACTGG ATGTGCTCTC TGAACAGCAC GAGCAGCATC AGGATCTGTT TAACAGCAAC CGTTTGACCT
53638         .......... .......... .......... .......... .......... .......... ..........
ATCC_8739     .......... .......... .......... .......... .......... .......... ..........
ECOR_10       .......... .......... .......... .......... .......... .......... ..........
ECOR_11       .......... .......... .......... .......... .......... .......... ..........
B171          .....G.... .......... .......... .......... .......... .......... ...C......
E24377A       .....G.... .......... .......... .......... .......... .......... ...C......
ECOR_34       .....G.... .......... .......... .......... .......... .......... ...C......
ECOR_71       .....G.... .......... .......... .......... .......... .......... ...C......
SE11          .....G.... .......... .......... .......... .......... .......... ...C......
O157_Sakai    .....G.... .......... .........T .......... .......... .......... ...C......
ECOR_36       .....G.... .......... .........T .....A.... .......... .......... ...C......
ECOR_35       .....G.... .......... .........T .....A.... .......... .......... ...C......
SMS-3-5       .....G.... .......... .......... .....A.... .......... .......... ...C......
536           .....G.... .......... .......... .......... .......... .......... ..CC......
APEC_O1       .....G.... .......... .......... .......... .......... .......... ..CC......
CFT073        .....G.... .......... .......... .......... .......... .......... ..CC......
E2348/69      .....G.... .......... .......... .......... .......... .......... ..CC......
ECOR_51       .....G.... .......... .......... .......... .......... .......... ..CC......
ECOR_57       .....G.... .......... .......... .......... .......... .......... ..CC......
UTI89         .....G.... .......... .......... .......... .......... .......... ..CC......
E._albertii   ...T.G.... .......T.. .........T ..A..A..C. .....T.... .......... ...C......

        22541
K-12          TCTCGGAAGC GCTGGCGAAA CTGTATCAAC GTCTTAACCC GCAGATTGAT ATGGGGCAGC GCACGCCGCA
53638         .......... .......... .......... .......... .......... .......... ..........
ATCC_8739     .......... .......... .......... .......... .......... .......... ..........
ECOR_10       .......... .......... .......... .......... .......... .......... ..........
ECOR_11       .......... .......... .......... .......... .......... .......... ..........
B171          .......... .......... T.......G. .......T.. .......... .......... ..G.......
E24377A       .......... .......... T.......G. .......T.. .......... .......... ..G.......
ECOR_34       .......... .......... T.......G. .......T.. .......... .......... ..........
ECOR_71       .......... .T........ T.......G. .......T.. .......... .....A.... ..........
SE11          .......... .......... T.......G. .......T.. .......... .......... ..G.......
O157_Sakai    .......... .......... T.......G. .......T.. .......... .......... ..........
ECOR_36       ....A..... .......... ........G. .......T.. .......... ........A. ..........
ECOR_35       ....A..... .......... ........G. .......T.. .......... ........A. ..........
SMS-3-5       .......... .......... .......... .......... .......... .......... ..........
536           ....A..... .......... .......... .......... .......... .......... ..........
APEC_O1       ....A..... .......... .......... .......... .......... .......... ..........
CFT073        ....A..... .......... .......... .......... .......... .......... ..........
E2348/69      ....A..... .......... .......... .......... .......... .......... ..........
ECOR_51       ....A..... .......... .......... .......... .......... .......... ..........
ECOR_57       ....A..... .......... .......... .......... .......... .......... ..........
UTI89         ....A..... .......... .......... .......... .......... .......... ..........
E._albertii   .......... .......... T.......G. .......T.. ...A...... .....C.... .T........

        22611
K-12          GACCATCGGT GAAGAACTGC TGGATTACCG CAACTATCTG GAAATGGAAG TTGAGGTTAA CCGTGGTTCC
53638         .......... .......... .......... .......... .......... .......... ..........
ATCC_8739     .......... .......... .......... .......... .......... .......... ..........
ECOR_10       .......... .......... .......... .......... .......... .......... ..........
ECOR_11       .......... .......... .......... .......... .......... .......... ..........
B171          ......T..C .......... .......... .......... .......... .......... ..........
E24377A       ......T..C .......... .......... .......... .......... .......... ..........
ECOR_34       ......T..C .......... .......... .......... .......... .......... T.........
ECOR_71       ......T..C .......... .......... .......... .......... .......... ..........
SE11          ......T..C .......... .......... .......... .......... .......... ..........
O157_Sakai    ......T..C .......... .......... .......... .......... .......... ..........
ECOR_36       ......T..C .......... .......... .......... .......... .......... ..........
ECOR_35       ......T..C .......... .......... .......... .......... .......... ..........
SMS-3-5       .......... .......... .......... .......... .......... .......... ..........
536           .......... .......... .......... .......... .......... .......... ..........
APEC_O1       .......... .......... .......... .......... .......... .......... ..........
CFT073        .......... .......... .......... .......... .......... .......... ..........
E2348/69      .......... .......... .......... .......... .......... .......... ..........
ECOR_51       .......... .......... .......... .......... .......... .......... ..........
ECOR_57       .......... .......... .......... .......... .......... .......... ..........
UTI89         .......... .......... .......... .......... .......... .......... ..........
E._albertii   ...T..T... .......... .......... T.....C... ..G....... .......... ..........
```

```
        22681
K-12         GATGGCTGGC TGCGCGCAGA GTCTGGTGCA TTGTCGACCG GTGAGGCGAT TGGTACCGGT ATGTCGATTC
53638        .......... .......... .......... .......... .......... .......... ..........
ATCC_8739    .......... .......... .......... .......... .......... .......... ..........
ECOR_10      .......... .......... .......... .......... .......... .......... ..........
ECOR_11      .......... .......... .......... .......... .......... .......... ..........
B171         .........T .......... .......... .......... .......... .......... ..........
E24377A      .........T .......... .......... .......... .......... .......... ..........
ECOR_34      .........T .......... .......... .......... .......... .......... ..........
ECOR_71      .....T...T .......... .......... .......... .......... .......... ..........
SE11         .........T .......... .......... .......... .......... .......... ..........
O157_Sakai   .........T .......... .......... .......... .......... .......... ..........
ECOR_36      .........T .......G.. .......... .......... .......T.. .......... ..........
ECOR_35      .........T .......G.. .......... .......... .......T.. .......... ..........
SMS-3-5      .......... .......... A.....C..T C......... .......... .......... ..........
536          .........T ....T..... .......... .......... .......... .......... ..........
APEC_O1      .........T ....T..... .......... .......... .......... .......... ..........
CFT073       .........T ....T..... .......... .......... .......... .......... ..........
E2348/69     .........T ....T..... .......... .......... .......... .......... ..........
ECOR_51      .........T ....T..... .......... .......... .......... .......... ..........
ECOR_57      .........T ....T..... .......... .......... .......... .......... ..........
UTI89        .........T ....T..... .......... .......... .......... .......... ..........
E._albertii  .........T ....A..G.. ......C... .......... .......C.. C.....T... ..........

        22751
K-12         TGGTGATGGT GGTACAAAGC TGGGAAGATG AATCTCGCCG CCTGCGCGGT AAAGATATCT CTCCTTGCCG
53638        .......... .......... .......... .......... .......... .......... ..........
ATCC_8739    .......... .......... .......... .......... .......... .......... ..........
ECOR_10      .......... .......... .......... .......... .......... .......... ..........
ECOR_11      .......... .......... .......... .......... .......... .......... ..........
B171         .......... .......... .......... .......... .......... .......... ..........
E24377A      .......... .......... .......... .......... .......... .......... ..........
ECOR_34      .......... .......... .......... .......... .......... .......... ..........
ECOR_71      .......... .......... .......... .......... .......... .......... ..........
SE11         .......... .......... .......... .......... .......... .......... ..........
O157_Sakai   .......... .......... .......... .......... .......... .......... ..........
ECOR_36      .......... ......G..T .......... .......... .T........ .......... ..........
ECOR_35      .......... ......G..T .......... .......... .T........ .......... ..........
SMS-3-5      .......... .......... .......... .......... .......... .......... ..........
536          .......... .......... .......... .......... .......... .......... ..........
APEC_O1      .......... .......... .......... .......... .......... .......... ..........
CFT073       .......... .......... .......... .......... .......... .......... ..........
E2348/69     .......... .......... .......... .......... .......... .......... ..........
ECOR_51      .......... .......... .......... .......... .......... .......... ..........
ECOR_57      .......... .......... .......... .......... .......... .......... ..........
UTI89        .......... .......... .......... .......... .......... .......... ..........
E._albertii  .......... ...G..G... .....G.... ....A..... T......... .......... ..........

        22821
K-12         CCTGCTGTTC CTCGATGAAG CAGCGCGACT GGATGCTCGT TCTATCGCCA CGCTGTTTGA ATTGTGTGAG
53638        .......... .......... .......... .......... .......... .......... ..........
ATCC_8739    .......... .......... .......... .......... .......... .......... ..........
ECOR_10      .......... .......... .......... .......... .......... .......... ..........
ECOR_11      .......... .......... .......... .......... .......... .......... ..........
B171         .......... .......... .......... .......... .......... .......... .........A
E24377A      .......... .......... .......... .......... .......... .......... .........A
ECOR_34      .......... .......... .......... .......... .......... .......... .........A
ECOR_71      .......... .......... .......... .......... .......... .......... ..........
SE11         .......... .......... .......... .......... .......... .......... .........A
O157_Sakai   .......... .......... .......... .......... .......... .......... ..........
ECOR_36      T......... ..T....... .......... .......... ..C....... .......... ..........
ECOR_35      T......... ..T....... .......... .......... ..C....... .......... ..........
SMS-3-5      .......... .......... .......... .......... ..C....... .......... ..........
536          .......... .......... .......... .......... .......... .......... .........A
APEC_O1      .......... .......... .......... ...C..C... ..C....... .......... .........A
CFT073       .......... .......... .......... .......... .......... .......... .........A
E2348/69     .......... .......... .......... ...C..C... ..C....... .......... .........A
ECOR_51      .......... .......... .......... .......... .......... .......... .........A
ECOR_57      .......... .......... .......... .......... .......... .......... .........A
UTI89        .......... .......... .......... ...C..C... ..C....... .......... .........A
E._albertii  T..A...... ..T.....G. .......... .......... ..G....... .......... .........A
```

```
        22891
K-12         CGTTTGCAAA TGCAACTCAT CATCGCAGCG CCGGAAAATA TCAGCCCGGA GAAAGGCACC ACCTATAAAC
53638        .......... .......... .......... .......... .......... .......... ..........
ATCC_8739    .......... .......... .......... .......... .......... .......... ..........
ECOR_10      .......... .......... .......... .......... .......... .......... ..........
ECOR_11      .......... .......... .......... .......... .......... .......... ..........
B171         .......... ....G..... .......... .......... .......... .......... ..........
E24377A      .......... ....G..... .......... .......... .......... .......... ..........
ECOR_34      .......... ....G..... .......... .......... .......... .......... ..........
ECOR_71      .......... ....G..... .......... .......... .......... .......... ..........
SE11         .......... ....G..... .......... .......... .......... .......... ..........
O157_Sakai   .......... ....G..... .......... .......... .......... .......... ..........
ECOR_36      .......... ....G..... .......... .......... ....T..... A......... ..........
ECOR_35      .......... ....G..... .......... .......... ....T..... A......... ..........
SMS-3-5      .......... ....G..... .......... .......... .......... A......... ..........
536          .......... ....G..... .......... .......... ....T..... A......... ..........
APEC_O1      .......... ....G..... T......... .......... .......... .......... ..........
CFT073       .......... ....G..... .......... .......... ....T..... A......... ..........
E2348/69     .......... ....G..... T......... .......... .......... .......... ..........
ECOR_51      .......... ....G..... .......... .......... ....T..... A......... ..........
ECOR_57      .......... ....G..... .......... .......... ....T..... A......... ..........
UTI89        .......... ....G..... T......... .......... .......... .......... ..........
E._albertii  .......... ....G..... ......G..A ..A....... .......... .......... ..........

        22961
K-12         TGGTGCGTAA AGTCTTCCAG AATACCGAAC ACGTTCATGT CGTCGGCCTG CGAGGATTTG CGCCGCAACT
53638        .......... .......... .......... .......... .......... .......... ..........
ATCC_8739    .......... .......... .......... .......... .......... .......... ..........
ECOR_10      .......... .......... .......... .......... .......... .......... ..........
ECOR_11      .......... .......... .......... .......... .......... .......... ..........
B171         ....A..... .......... .......... .......C.. .......... .......... ..........
E24377A      .......... .......... .......... .......C.. .......... .......... ..........
ECOR_34      .......... .......... .......... .......C.. .......... .......... ..........
ECOR_71      .......... .......... .......... .......C.. .......... .......... ..........
SE11         .......... .......... .......... .......C.. .......... .......... ..........
O157_Sakai   .......... .......... .......... .T........ .......... .......... ..........
ECOR_36      ....A..... .......... .......... .......C.. .......... ..T..C.... .A........
ECOR_35      ....A..... .......... .......... .......C.. .......... ..T..C.... .A........
SMS-3-5      .......... .......... .......... .......C.. A.....T... ..T..T.... .A........
536          .......... .......... .......... .......C.. .......... .......... ..........
APEC_O1      .......... .......... .......... .......C.. .......... .......... ..........
CFT073       .......... .......... .......... .......C.. .......... .......... ..........
E2348/69     .......... .......... .......... .......C.. .......... .......... ..........
ECOR_51      .......... .......... .......... .......C.. .......... .......... ..........
ECOR_57      .......... .......... .......... .......C.. .......... .......... ..........
UTI89        .......... .......... .......... .......C.. .......... .......... ..........
E._albertii  ....A..... .......... .......... .......C.. .......... .....C.... .A........

        23031
K-12         CCCTGAAACG CTTCCAGGAA CTGACGAAGC GCCTTCTCAG GCGAGTTAAA ATTAAGGCGG CAGCAATGCC
53638        .......... .......... .......... .......... .......... .......... ..........
ATCC_8739    .......... .......... .......... .......... .......... .......... ..........
ECOR_10      .......... .......... .......... .......... .......... .......... ..........
ECOR_11      .......... .......... .......... .......... .......... .......... ..........
B171         .......... .......... G......... .......... .......... .......... ..........
E24377A      .......... .......... G......... .......... .......... .......... ..........
ECOR_34      .......... .......... G......... .......... .......... .......... ..........
ECOR_71      .......... .......... G......... .......... .......... .......... ..........
SE11         .......... .......... G......... .......... .......... .......... ..........
O157_Sakai   .......... .......... G......... .......... .......... .......... ..........
ECOR_36      .........A .......... .......... ......C... .....C...G ...G...... ....G.....
ECOR_35      .........A .......... .......... ......C... .....C...G ...G...... ....G.....
SMS-3-5      .......... .......... G......... ......C... .....C...G ...G...... ....G.....
536          .......... .......... G......... ......C... .......... ..G....... ..........
APEC_O1      T......... .......... G......... .......... .......... .......... ..........
CFT073       .......... .......... G......... ......C... .......... ..G....... ..........
E2348/69     T......... .......... G......... .......... ..T....... .......... ..........
ECOR_51      .......... .......... G......... ......C... .......... ..G....... ..........
ECOR_57      .......... .......... G......... ......C... .......... ..G....... ..........
UTI89        T......... .......... G......... .......... .......... .......... ..........
E._albertii  ......G... .......... .......... ...C..C... .....C...G ....G..... ..........
```

```
         23101
K-12         GCCTTTTCTT TTTCCGAAAA CTCCGTTTCT GCACTAAAAA AGTGGCACAT TACGGCGCGC TTTTCTTTAA
53638        .......... .......... .......... .......... .......... .......... ..........
ATCC_8739    .......... .......... .......... .......... .......... .......... ..........
ECOR_10      .......... .......... .......... .......... .......... .......... ..........
ECOR_11      .......... .......... .......... .......... .......... .......... ..........
B171         .......... .......... .......... .......... .......... .......... ..........
E24377A      .......... .......... .......... .......... .......... .......... ..........
ECOR_34      .......... .......... .......... .......... .......... .......... ..........
ECOR_71      .......... .......... .........C .......... .......... .......... ..........
SE11         .......... .......... .........C .......... .......... .......... ..........
O157_Sakai   .......... .......... .......... .......... .......... .......... ..........
ECOR_36      ......C..G ....T..... .......... .......... ....C..... .CT....... ..........
ECOR_35      ......C..G ....T..... .......... .......... ....C..... .CT....... ..........
SMS-3-5      .........A ....T..... .......... .......... .A..C..... .CT....... ..........
536          .......... .......... .......... .......... .......... .......... ..........
APEC_O1      .......... .......... .......... .......... .......... .......... ..........
CFT073       .......... .......... .......... .......... .......... .......... ..........
E2348/69     .......... .......... .......... .......... .......... .......... ..........
ECOR_51      .......... .......... .......... .......... .......... .......... ..........
ECOR_57      .......... .......... .......... .......... .......... .......... ..........
UTI89        .......... .......... .......... .......... .......... .......... ..........
E._albertii  ........C. ....G..... .......... .........T .A..C.G... ...A....A. ..........

         23171
K-12         ACTTCTTTAC ATTAGGTTAT GTAAAAACGT ATCGGCGTTT ATATACTGAA GATAAGCCTG ATGAGTAACA
53638        .......... .......... .......... .......... .......... .......... ..........
ATCC_8739    .......... .......... .......... .......... .......... .......... ..........
ECOR_10      .......... .......... .......... .......... .......... .......... ..........
ECOR_11      .......... .......... .......... .......... .......... .......... ..........
B171         .......... .......... .......... .......... .......... .......... ..........
E24377A      .......... .......... .......... .......... .......... .......... ..........
ECOR_34      .......... .......... .......... .......... .......... .......... ..........
ECOR_71      .......... .......... .......... .......... .......... .......... ..........
SE11         .......... .......... .......... .......... .......... .......... ..........
O157_Sakai   .......... .......... .......... .......... .......... .......... ..........
ECOR_36      .......... .......... .......... .......... .......... .......... ..........
ECOR_35      .......... .......... .......... .......... .......... .......... ..........
SMS-3-5      .......... .......... .......... .......... .......... .......... ..........
536          .......... .......... .......... .......... .......... .......... ..........
APEC_O1      .......... .......... .......... .......... .......... .......... ..........
CFT073       .......... .......... .......... .......... .......... .......... ..........
E2348/69     .......... .......... .......... .......... .......... .......... ..........
ECOR_51      .......... .......... .......... .......... .......... .......... ..........
ECOR_57      .......... .......... .......... .......... .......... .......... ..........
UTI89        .......... .......... .......... .......... .......... .......... ..........
E._albertii  .......... .........G .......... .......... .......... .G....T... ..........

         23241
K-12         GGCT
53638        ....
ATCC_8739    ....
ECOR_10      ....
ECOR_11      ....
B171         ....
E24377A      ....
ECOR_34      ....
ECOR_71      ....
SE11         ....
O157_Sakai   ....
ECOR_36      ....
ECOR_35      ....
SMS-3-5      ....
536          ....
APEC_O1      ....
CFT073       ....
E2348/69     ....
ECOR_51      ....
ECOR_57      ....
UTI89        ....
E._albertii  ....
```

Table S2. Sequences alignment. Segment 2

```
               1
K-12           TGCTCATCAT TATGCAGGTG AGTTTCGCGT GTTCACGTCG CGTCGACGAT TTGACGCACA AAAAAGGTGA
53638          .......... .......... .......... A......... .......... .......... ..........
ATCC_8739      .......... .......... .......... .......... .......... .......... ..........
ECOR_10        .......... .......... .......... .......... .......... .......... ..........
ECOR_11        .......... .......... .......... .......... .......... .......... ..........
B171           .......... .......... .......... .......... .......... .......... ..........
E24377A        .......... .......... .......... .......... .......... .......... ..........
ECOR_34        .......... .......... .......... .......... .......... .......... ..........
ECOR_71        .......... .......... .......... .......... .......... .......... ..........
SE11           .......... .......... .......... .......... .......... .......... ..........
O157_Sakai     .......... .......... .......... .......... .......... .......... ..........
ECOR_35        .......... .......... .......... .......... .......... .......... ..........
ECOR_36        .......... .......... .......... .......... .......... .......... ..........
SMS-3-5        .......... .......... .......... .......... .......... .......... ..........
536            .......... .......... .......... .......... .......... .......... ..........
APEC_O1        .......... .......... .......... .......... .......... .......... ..........
CFT073         .......... .......... .......... .......... .......... .......... ..........
E2348/69       .......... .......... .......... .......... .......... .......... ..........
ECOR_51        .......... .......... .......... .......... .......... .......... ..........
ECOR_57        .......... .......... .......... .......... .......... .......... ..........
UTI89          .......... .......... .......... .......... .......... .......... ..........
E._albertii    .......... ...AT..... .....T.... A......... .......... .......... ..........

              71
K-12           AAAGTAGTTA TGGTAAATGT TCGTCAGCCC AGGGATGTCG CACAAATTCT GCTTTCGGTG CTGTTTTTAG
53638          .......... .......... .......... .......... .......... .......... ..........
ATCC_8739      .......... .......... .......... .......... .......... .......... ..........
ECOR_10        .......... .......... .......... .......... .......... .......... ..........
ECOR_11        .......... .......... .......... .......... .......... .......... ..........
B171           .......... .......... .......... .......... .......... .......... ..........
E24377A        .......... .......... .......... .......... .......... .......... ..........
ECOR_34        .......... .......... .......... .......... .......... .......... ..........
ECOR_71        .......... .......... .......... .......... .......... .......... ..........
SE11           .......... .......... .......... .......... .......... .......... ..........
O157_Sakai     .......... .......... .......... .......... .......... .......... ..........
ECOR_35        .......... .......... .......... .......... .......... .......... ..........
ECOR_36        .......... .......... .......... .......... .......... .......... ..........
SMS-3-5        .......... .......... .......... .......... .......... .......... ..C.......
536            .......... .......... .......... .......... .......... .......... ..........
APEC_O1        .......... .......... .......... .......... .......... .......... ..........
CFT073         .......... .......... .......... .......... .......... .......... ..........
E2348/69       .......... .......... .......... .A........ .......... .......... ..........
ECOR_51        .......... .......... .......... .......... .......... .......... ..........
ECOR_57        .......... .......... .......... .......... .......... .......... ..........
UTI89          .......... .......... .......... .......... .......... .......... ..........
E._albertii    .......... ........A. .......... ........T. .......... .......... ..........

             141
K-12           CCATCATGAT TGTGGCATGT CTGTGGATTG TTCAACCCTT TATTCTCGGC TTTGCATGGG CCGGTACGGT
53638          .......... .......... .......... .......... .......... .......... ..........
ATCC_8739      .......... .......... .......... .......... .......... .......... ..........
ECOR_10        .......... .......... .......... .......... .......... .......... ..........
ECOR_11        .......... .......... .......... .......... .......... .......... ..........
B171           .......... .......... .......... .......... .......... .......... ..........
E24377A        .......... .......... .......... .......... .......... .......... ..........
ECOR_34        .......... .......... .......... .......... .......... .......... ..........
ECOR_71        .......... .......... .......... .......... .......... .......... ..........
SE11           .......... .......... .......... .......... .......... .......... ..........
O157_Sakai     .......... .......... .......... .......... .......... .......... ..........
ECOR_35        .......... .......... .......... .......... .......... .......... .......A..
ECOR_36        .......... .......... .......... .......... .......... .......... .......A..
SMS-3-5        .......... .......... .......... .A........ .......... .......... .T........
536            .......... .......... .......... .A........ .......... .......... .......A..
APEC_O1        .......... .......... .......... .A........ .......... .......... .......A..
CFT073         .......... .......... .......... .A........ .......... .......... .......A..
E2348/69       .......... .......... .......... .A........ .......... .......... .......A..
ECOR_51        .......... .......... .......... .A........ .......... .......... .......A..
ECOR_57        .......... .......... .......... .A........ .......... .......... .......A..
UTI89          .......... .......... .......... .A........ .......... .......... .......A..
E._albertii    .......... .......... .......... .......... .......... .......... ..........
```

```
            211
K-12         GGTTATCGCC ACCTGGCCGG TATTGTTACG TTTGCAAAAG ATCATGTTTG GCCGCCGCTC TCTCGCCGTT
53638        .......... .......... .......... .......... .......... .......... ..........
ATCC_8739    .......... .......... .......... .......... .......... .......... ..........
ECOR_10      .......... .......... .......... .......... .......... .......... ..........
ECOR_11      .......... .......... .......... .......... .......... .......... ..........
B171         .......... .......... .......... .......... .......... .......... ..........
E24377A      .......... .......... .......... .......... .......... .......... ..........
ECOR_34      .......... .......... .......... .......... .......... .......... ..........
ECOR_71      .......... .......... .......... .......... .......... .......... ..........
SE11         .......... .......... .......... .......... .......... .......... ..........
O157_Sakai   .......... .......... .......... .......... .......... .......... ..........
ECOR_35      .......... .......... .......... .......... .......... .......... ..........
ECOR_36      .......... .......... .......... .......... .......... .......... ..........
SMS-3-5      .......... .......... .......... .......... T......... .T........ ..........
536          .......... .......... .......... .......... .......... .T........ ..........
APEC_O1      .......... .......... .......... .......... .......... .T........ ..........
CFT073       .......... .......... .......... .......... .......... .T........ ..........
E2348/69     .......... .......... .......... .......... .......... .T........ ..........
ECOR_51      .......... .......... .......... .......... .......... .T........ ..........
ECOR_57      .......... .......... .......... .......... .......... .T........ ..........
UTI89        .......... .......... .......... .......... .......... .T........ ..........
E._albertii  .......... ..A....... .......... C..A...... .......... ....T..... .........G

            281
K-12         CTGGTGATGA CGCTGTTATT AGTGATGGTG TTTATCATCC CTATTGCTTT GCTGGTTAAC AGTATCGTCG
53638        .......... .......... .......... .......... .......... .......... ..........
ATCC_8739    .......... .......... .......... .......... .......... .......... ..........
ECOR_10      .......... .......... .......... .......... .......... .......... ..........
ECOR_11      .......... .......... .......... .......... .......... .......... ..........
B171         .......... .......... .......... .......... .......... .......... ..........
E24377A      .......... .......... .......... .......... .......... .......... ..........
ECOR_34      .......... .......... .......... .......... .......... .......... ..........
ECOR_71      .......... .......... .......... .......... .......... .......... ..........
SE11         .......... .......... .......... .......... .......... .......... ..........
O157_Sakai   .......... .......... .......... .......... .......... .......... ..........
ECOR_35      .......... .......... .......... .......... .......C.. .T........ .....T....
ECOR_36      .......... .......... .......... .......... .......C.. .T........ .....T....
SMS-3-5      .......... .......... .......... .......... .......C.. .......... ..........
536          .......... .......... .......... .......... .......... .......... ..........
APEC_O1      .......... .......... .......... .......... .......... .......... ..........
CFT073       .......... .......... .......... .......... .......... .......... ..........
E2348/69     .......... .......... .......... .......... .......... .......... ..........
ECOR_51      .......... .......... .......... .......... .......... .......... ..........
ECOR_57      .......... .......... .......... .......... .......... .......... ..........
UTI89        .......... .......... .......... .......... .......... .......... ..........
E._albertii  .......... .AT....... .......... .......... .......... A........T ..C..T..T.

            351
K-12         ACGGCAGCGG CCCGCTAATT AAAGCCATTT CCAGCGGTGA CATGACGTTA CCCGATCTGG CGTGGCTTAA
53638        .......... .......... .......... .......... .......... .......... ..........
ATCC_8739    .......... .......... .......... .......... .......... .......... ..........
ECOR_10      .......... .......... .......... .......... .......... .......... ..........
ECOR_11      .......... .......... .......... .......... .......... .......... ..........
B171         .......... .......... .......... .......C.. .......... .......... ..........
E24377A      .......... .......... .......... .......C.. .......... .......... ..........
ECOR_34      .......... .......... .......... .......C.. .......... .......... ..........
ECOR_71      .......... .......... .......... .......C.. .......... .......... ..........
SE11         .......... .......... .......... .......C.. .......... .......... ..........
O157_Sakai   .......... .......... .......... .......... .......... .......... ..........
ECOR_35      .T........ T......... .......... .......... .......... ........A. ..........
ECOR_36      .T........ T......... .......... .......... .......... ........A. ..........
SMS-3-5      .......... .......... .......... .......... .......... .......... ..........
536          .......... .......... .......... ....T..... .......... .......... ..........
APEC_O1      .......... .......... .......... ....T..... .......... .......... ..........
CFT073       .......... .......... .......... ....T..... .......... .......... ..........
E2348/69     .......... .......... .......... ....T..... ......A... .......... ..........
ECOR_51      .......... .......... .......... ....T..... .......... .......... ..........
ECOR_57      .......... .......... .......... ....T..... .......... .......... ..........
UTI89        .......... .......... .......... ....T..... .......... .......... ..........
E._albertii  .......... ......G..C ........C. ....T..... .......C.. ........C. .C........
```

```
             421
K-12          TACCATTCCG GTGATTGGCG CGAAGCTGTA TGCAGGCTGG CACAACTTGC TGGATATGGG GGGGACGGCG
53638         .......... .......... .A........ .......... .......... .......... ..........
ATCC_8739     .......... .......... .......... .......... .......... .......... ..........
ECOR_10       .......... .......... .......... .......... .......... .......... ..........
ECOR_11       .......... .......... .......... .......... .......... .......... ..........
B171          .......... .......... .......... .......... .......... .......... ..........
E24377A       .......... .......... .......... .......... .......... .......... ..........
ECOR_34       .......... .......... .......... .......... .......... .......... ..........
ECOR_71       .......... .......... .......... .......... .......... .......... ..........
SE11          .......... .......... .......... .......... .......... .......... ..........
O157_Sakai    .......... .......... .......... .......... .......... .......... ..........
ECOR_35       .......... .......... .......A.. .......... .......... .......... ...T......
ECOR_36       .......... ........T. .......A.. .......... .......... .......... ...T......
SMS-3-5       .......... .......... .......A.. .......... .......... .......... ..........
536           .......... .......... .......A.. .......... .....T.... .......... ..........
APEC_O1       .......... .......... .......A.. .......... .....T.... .......... ..........
CFT073        .......... .......... .......A.. .......... .....T.... .......... ..........
E2348/69      A......... .........A .......A.. .......... .....T.... .......... ..........
ECOR_51       .......... .......... .......A.. .......... .....T.... .......... ..........
ECOR_57       .......... .......... .......A.. .......... .....T.... .......... ..........
UTI89         .......... .......... .......A.. .......... .....T.... .......... ..........
E._albertii   ......C... ..A....... .......T.. C..T..T... ..T..T.... .......... C..C......

             491
K-12          ATCATGGCGA AAGTCCGCCC TTATATTGGC ACCACCACCA CCTGGTTCGT TGGGCAGGCG GCGCATATCG
53638         .......... .......... .......... .......... .......... .......... ..........
ATCC_8739     .......... .......... .......... .......... .......... ......A... ..........
ECOR_10       .......... .......... .......... .......... .......... .......... ..........
ECOR_11       .......... .......... .......... .......... .......... .......... ..........
B171          .......... .......... .......... .......... .......... .......... ..........
E24377A       .......... .......... .......... .......... .......... .......... ..........
ECOR_34       .......... .......... .......... .......... .......... .......... ..........
ECOR_71       .......... .......... .......... .......... .......... .......... ..........
SE11          .......... .......... .......... .......... .......... .......... ..........
O157_Sakai    .......... .......... .......... ........T. .......... ......A... ..........
ECOR_35       .......... .......T.. .......... .......... .......... .......... ..........
ECOR_36       .......... .......T.. .......... .......... .......... .......... ..........
SMS-3-5       ........A. .......T.. .......... .......... .......... .......... ..........
536           .......... .......T.. .......... .......... .......... .......... ..........
APEC_O1       .......... .......T.. .......... .......... .......... .......... ..........
CFT073        .......... .......T.. .......... .......... .......... .......... ..........
E2348/69      .......... .......T.. .......... .......... .......... .......... ..........
ECOR_51       .......... .......T.. .......... .......... .......... .......... ..........
ECOR_57       .......... .......T.. .......... .......... .......... .......... ..........
UTI89         .......... .......T.. .......... .......... .......... .......... ..........
E._albertii   .......... .......T.. .......... .....T.... .......T.. ...A.....T .....C..T.

             561
K-12          GGCGCTTTAT GGTGCATTGT GCGCTGATGC TTCTCTTCAG TGCCCTGCTG TACTGGCGCG GTGAACAGGT
53638         .......... .......... .......... .......... .......... .......... ..........
ATCC_8739     .......... .......... .......... .......... .......... .......... ..........
ECOR_10       .......... .......... .......... .......... .......... .......... ..........
ECOR_11       .......... .......... .......... .......... .......... .......... ..........
B171          .......... .......... .......... .......... .......... .......... ..........
E24377A       .......... .......... .......... .......... .......... .......... ..........
ECOR_34       .......... .......... .......... .......... .......... .......... ..........
ECOR_71       .......... .......... .......... .......... .......... .......... ..........
SE11          .......... .......... .......... .......... .......... .......... ..........
O157_Sakai    .......... .......... .......... .......... .......... .......... ..........
ECOR_35       .......... .......... .......... .......... .......... .......... ..........
ECOR_36       .......... .......... .......... .......... .......... .......... ..........
SMS-3-5       .......... .......... .......... .......... C......... .......... ..........
536           .......... .......... .......... .......... C......... .......... ..........
APEC_O1       .......... .......... .......... .......... C......... .......... ..........
CFT073        .......... .......... .......... .......... C......... .......... ..........
E2348/69      .......... .......... .......... .......... C......... .......... ..........
ECOR_51       .......... .......... .......... .......... C......... .......... ..........
ECOR_57       .......... .......... .......... .......... C......... .......... ..........
UTI89         .......... .......... .......... .......... C......... .......... ..........
E._albertii   .A..T..... .A........ .......... .G..G..... C...T..... .......... .C........
```

```
             631
K-12         GGCACAAGGC ATTCGCCATT TTGCAACCCG TCTGGCAGGC GTTCGCGGAG ATGCCGCCGT GCTGCTGGCG
53638        T......... .......... .......... .......... .......... .......... A.........
ATCC_8739    .......... .......... ....G..... .......... .......... .......... A.........
ECOR_10      .......... .......... .......... .......... .......... .......... ..........
ECOR_11      .......... .......... .......... .......... .......... .......... ..........
B171         .......... .......... .......... .......... .......... .......... A.........
E24377A      .......... .......... .......... .......... .......... .......... A.........
ECOR_34      .......... .......... .......... .......... .......... .......... A.........
ECOR_71      .......... .......... .......... .......... .......... .......... A.........
SE11         .......... .......... .......... .......... .......... .......... ..........
O157_Sakai   .......... .......... ....G..... .......... .......... .......... A.........
ECOR_35      .......... .......... .......... .......... ........T. ....T..T.. ...A......
ECOR_36      .......... .......... .......... .......... ........T. ....T..T.. ...A......
SMS-3-5      .......... .......... .......... .......... .....T.... .......... ..........
536          .......... .......... .......... .......... .......... .......... ..........
APEC_O1      .......... .......... .......... .......... .......... .......... ..........
CFT073       .......... .......... .......... .......... .......... .......... ..........
E2348/69     .......... .......... .......... .......... .......... .......... ..........
ECOR_51      .......... .......... .......... .......... .......... .......... ..........
ECOR_57      .......... .......... .......... .......... .......... .......... ..........
UTI89        .......... .......... .......... .......... .......... .......... ..........
E._albertii  A..G...... ..C....... ....G..... ......G... ........T. ....G..T.. .T.A......

             701
K-12         GCACAGGCTA TCCGCGCGGT GGCGCTGGGT GTGGTGGTGA CGGCGTTAGT ACAGGCAGTG CTTGGCGGTA
53638        .......... .......... .......... .......... .......... .......... ..........
ATCC_8739    .......... .......... .......... .......... .......... .......... ..........
ECOR_10      .......... .......... .......... .......... .......... .......... ..........
ECOR_11      .......... .......... .......... .......... .......... .......... ..........
B171         .......... .......... .......... .......... .......... .......... ..........
E24377A      .......... .......... .......... .......... .......... .......... ..........
ECOR_34      .......... .......... .......... .......... .......... .......... ..........
ECOR_71      .......... .......... .......... .......... .......... .......... ..........
SE11         .......... .......... .......... .......... .......... .......... ..........
O157_Sakai   .......... .......... .......... .......... .......... .......... ..........
ECOR_35      .......... .......... .......... .......... .......... .......... ..........
ECOR_36      .......... .......... .......... .......... .......... .......... ..........
SMS-3-5      .......... .......... .......... ..T....... .A........ .......... ..........
536          .......... .......... .......... .......... .......... .......... ..........
APEC_O1      .......... .......... .......... .......... .......... .......... ..........
CFT073       .......... .......... .......... .......... .......... .......... ..........
E2348/69     .......... .......... .......... .......... .......... .......... ..........
ECOR_51      .......... .......... .......... .......... .......... .......... ..........
ECOR_57      .......... .......... .......... .......... .......... .......... ..........
UTI89        .......... .......... .......... .......... .......... .......... ..........
E._albertii  ..G..A..C. .T..T..C.. A...T....G ..A..A..C. .C..TC.G.. T.....G... ........C.

             771
K-12         TCGGCCTCGC CGTATCCGGC GTACCTTATG CAACGTTGCT AACGGTGTTA ATGATCCTCT CCTGCCTTGT
53638        .......... ...T...... .......... ....T..... .......... .......... ..........
ATCC_8739    .......... ...T...... .......... ....T..... .......... .......... ..........
ECOR_10      .......... .......... .......... .......... .......... .......... ..........
ECOR_11      .......... .......... .......... .......... .......... .......... ..........
B171         .......... .......... .......... .......... .......... .......... ..........
E24377A      .......... .......... .......... .......... .......... .......... ..........
ECOR_34      .......... .......... .......... .......... .......... .......... ..........
ECOR_71      .......... .......... .......... .......... .......... .......... ..........
SE11         .......... .......... .......... .......... .......... .......... ..........
O157_Sakai   .......... .......... .......... ....T..... .......... ........T. ..........
ECOR_35      .......... .......... .......... ....T..... C......... .......... ..........
ECOR_36      .......... .......... .......... ....T..... C......... .......... ..........
SMS-3-5      .......... .......... .......... ....T..... C......... .......... ..........
536          .......... .......... .......... ....T..... .......... .......... ..........
APEC_O1      .......... .......... .......... ....T..... .......... .......... ..........
CFT073       .......... .......... .......... ....T..... .......... .......... ..........
E2348/69     .......... .......... .......... ....T..... .......... .......... ..........
ECOR_51      .......... .......... .......... ....T..... .......... .......... ..........
ECOR_57      .......... .......... .......... ....T..... .......... .......... ..........
UTI89        .......... .......... .......... ....T..... .......... .......... ..........
E._albertii  .T..T..G.. G..TG..... ..T..C..C. .......AT. G.....A... .......... ..........
```

```
             841
K-12          CCAGCTTGGC CCGTTGCCGG TACTGATTCC GGCGATTATC TGGCTCTACT GGACTGGCGA TACCACCTGG
53638         .......... .......... .......... .......... .......... ....C..... ..........
ATCC_8739     .......... .......... .......... .......... .......... ....C..... ..........
ECOR_10       .......... .......... .......... .......... .......... .......... ..........
ECOR_11       .......... .......... .......... .......... .......... .......... ..........
B171          .......... .......... .......... .......... .......... .......... ..........
E24377A       .......... .......... .......... .......... .......... .......... ..........
ECOR_34       .......... .......... .......... .......... .......... .......... ..........
ECOR_71       .......... .......... .......... .......... .......... .......... ..........
SE11          .......... .......... .......... .......... .......... ....C..... ..........
O157_Sakai    .......... .......... .......... .......... .......... .......... ..........
ECOR_35       .........A .......... .......... .......... .......... ....C..T.. ..........
ECOR_36       .........A .......... .......... .......... .......... ....C..T.. ..........
SMS-3-5       .........A .......... .......... .......... .......... .......... ..........
536           .........A .......... .......... .......... .......... .......... ..........
APEC_O1       .........A .......... .......... .......... .......... .......... ..........
CFT073        .........A .......... .......... .......... .......... .......... ..........
E2348/69      .........A .......... .......... .......... .......... .......... ..........
ECOR_51       .........A .......... .......... .......... .......... .......... ..........
ECOR_57       .........A .......... .......... .......... .......... .......... ..........
UTI89         .........A .......... .......... .......... .......... .......... ..........
E._albertii   .......... ........A. ..T....... .......... .......... ....A..T.. C.........

             911
K-12          GGAACGGTAT TGTTAGTGTG GAGCGGTGTG GTTGGCACGC TGGATAACGT CATCCGCCCA ATGTTAATTC
53638         .......... .......... .......... .......... .......... .......... ..........
ATCC_8739     .......... .......... .......... .......... .......... .......... ..........
ECOR_10       .......... .......... .......... .......... .......... .......... ..........
ECOR_11       .......... .......... .......... .......... .......... .......... ..........
B171          .......... .......... .......... .......... .......... .......... ..........
E24377A       .......... .......... .......... .......... .......... .......... ..........
ECOR_34       .......... .......... .......... .......... .......... .......... ..........
ECOR_71       .......... .......... .......... .......... .......... .......... ..........
SE11          .......... .......... .......... .......... .......... .......... ..........
O157_Sakai    .......... .......... .......... .......... .......... .......... ..........
ECOR_35       .......... .......... .......... .......... .......... .......... ..........
ECOR_36       .......... .......... .......... .......... .......... .......... ..........
SMS-3-5       .......... .......... .......... .......... .......... .......... ..........
536           .......... .......T.. ......G... .......... .C........ .......... ..........
APEC_O1       .......... .......T.. .......... .......... .C........ .......... ..........
CFT073        .........C .......T.. .......... .......... .C........ .......... ..........
E2348/69      .......... .......T.. .......... .......... .C........ .......... ..........
ECOR_51       .........C .......T.. .......... .......... .C........ .......... ..........
ECOR_57       .........C .......T.. .......... .......... .C........ .......... ..........
UTI89         .......... .......T.. .......... .......... .C........ .......... ..........
E._albertii   ..C..T..GC ..C.G..... ......C..A .....G.... .......TA. ......T..T ...C.T....

             981
K-12          GCATGGGTGC CGATTTACCG CTGATCCTGA TTCTCTCTGG CGTTATTGGT GGTTTGATTG CTTTCGGGAT
53638         .......... .......... .......... .C........ .......... .......... ..........
ATCC_8739     .......... .......... .......... .C........ .......... .......... ..........
ECOR_10       .......... .......... .......... .......... .......... .......... ..........
ECOR_11       .......... .......... .......... .......... .......... .......... ..........
B171          .......... .......... .......... .C........ T......... .......... ..........
E24377A       .......... .......... .......... .C........ T......... .......... ..........
ECOR_34       .......... .......... .......... .C........ T......... .......... ..........
ECOR_71       .......... .......... .......... .C........ T......... .......... ..........
SE11          .......... .......... .......... .C........ .......... .......... ..........
O157_Sakai    .......... .......... .......... .C........ .......... .......... ..........
ECOR_35       .......... .......... .......... .C........ T......... .......... ..........
ECOR_36       .......... .......... .......... .C........ T......... .......... ..........
SMS-3-5       .......... .......... .......... .......... .......... .......... ..........
536           .......... .......... .......... .C........ .......... .......... ..........
APEC_O1       .......... .......... .......... .C........ .......... .......... ..........
CFT073        .......... .......... .......... .......... .......... .......... ..........
E2348/69      .......... .......... .......... .C........ .......... .......... ..........
ECOR_51       .......... .......... .......... .......... .......... .......... ..........
ECOR_57       .......... .......... .......... .......... .......... .......... ..........
UTI89         .......... .......... .......... .C........ .......... .......... ..........
E._albertii   .......C.. .......... .....T..A. .C.....C.. G.....C..G ..C..A.... .C..T..T..
```

```
          1051
K-12          GATCGGTCTG TTTATTGGTC CGGTTCTGTT AGCCGTTTCC TGGCGTCTGT TTGCCGCGTG GGTGGAAGAA
53638         .......... .......... .......... .......... .......... .......... ..........
ATCC_8739     .......... .......... .......... .......... .......... .......... ..........
ECOR_10       .......... .......... .......... .......... .......... .......... ..........
ECOR_11       .......... .......... .......... .......... .......... .......... ..........
B171          .......... .......... .......... .......... .......... .......... ..........
E24377A       .......... .......... .......... .......... .......... .......... ..........
ECOR_34       .......... .......... .......... .......... .......... .......... ..........
ECOR_71       .......... .......... .......... .......... .......... .......... ..........
SE11          .......... .......... .......... .......... .......... .......... ..........
O157_Sakai    .......... .......... .......... .......... .......... .......... .........G
ECOR_35       ......C... .......... .......... .......... ........T. .......... ..........
ECOR_36       ......C... .......... .......... .......... ........T. .......... ..........
SMS-3-5       ......C... .......... .......... .......... ........T. .......... ..........
536           .......... .......... .......... .......... .......... .......... ..........
APEC_O1       .......... .......... .......... .......... .......... .......... ..........
CFT073        ......C... .......... .......... .......... ........T. .......... ..........
E2348/69      .......... .......... .......... .......... .......... .......... ..........
ECOR_51       ......C... .......... .......... .......... ........T. .......... ..........
ECOR_57       ......C... .......... .......... .......... ........T. .......... ..........
UTI89         .......... .......... .......... .......... .......... .......... ..........
E._albertii   ......C... .......... .T.....C.. G....A.... ........T. ....A..C.. ...C......

          1121
K-12          GTCCCGCCGC CGACGGACCA ACCGGAAGAA ATTCTCGAAG AACTTGGCGA AATCGAGAAA CCGAATAAGT
53638         .......... .......... .......... .......... .......... .......... ..........
ATCC_8739     .......... .......... .......... .......... .......... .......... ..........
ECOR_10       .......... .......... .......... .......... .......... .......... ..........
ECOR_11       .......... .......... .......... .......... .......... .......... ..........
B171          .......... .......... .......... .......... .......... .......... ..........
E24377A       .......... .......... .......... .......... .......... .......... ..........
ECOR_34       .......... .......... .......... .......... .......... .......... ..........
ECOR_71       .......... .......... .......... .......... .......... .......... ..........
SE11          .......... .......... .......... .......... .......... .......... ..........
O157_Sakai    .....A.... .......T.. .......... .......... .......... ....C..... ..........
ECOR_35       .....A.... .......... .......... .......... .......... .......... T.........
ECOR_36       .....A.... .......... .......... .......... .......... .......... T.........
SMS-3-5       .....A.... .......... .......... .......... .......... .......... T.........
536           .....A.... .......... .......... .......... .......... .......... T.........
APEC_O1       .....A.... .......... .......... .......... .......... .......... ..........
CFT073        .....A.... .......... .......... .......... .......... .......... T.........
E2348/69      .....A..A. .......... .......... .......... .......... .......... ..........
ECOR_51       .....A.... .......... .......... .......... .......... .......... T.........
ECOR_57       .....A.... .......... .......... .......... .......... .......... T.........
UTI89         .....A.... .......... .......... .......... .......... .......... ..........
E._albertii   ..T..A..A. .A..T..... ..AT..G... .......... ....G..G.. ...A..A... A.......A.

          1191
K-12          AATTTCTCAT CAGGCGGCTC TGCCGCCTGA TTGTTAACCA CCGCTAATTG ATATTTGCTT GCTCTTCCCC
53638         .......... .......... .......... .......... .......... .......... ..........
ATCC_8739     .......... .......... .......... .......... .......... .......... ..........
ECOR_10       .......... .......... .......... .......... .......... .......... ..........
ECOR_11       .......... .......... .......... .......... .......... .......... ..........
B171          .......... .......... .......... .........C .......... .......... A.........
E24377A       .......... .......... .......... .........C .......... .......... ..........
ECOR_34       .......... .......... .......... .........C .......... .......A.. ..........
ECOR_71       .......... .......... .......... .........C .......... .......... ..........
SE11          .......... .......... .......... .......... .......... .......... ..........
O157_Sakai    .......... .......... .......... .......... .......... .......... ..........
ECOR_35       .......... .......... .......... .........C .T........ .......... ..........
ECOR_36       .......... .......... .......... .........C .T........ .......... ..........
SMS-3-5       .......... .......... .......... .........C .T........ .......... ..........
536           .......... .......... ..T....... .........C .T........ .......... ..........
APEC_O1       .......... .......... .......... .........C .T........ .......... ..........
CFT073        .......... .......... .......... .........C .T........ .......... ..........
E2348/69      .......... .......... .......... .........C TG........ .......... ..........
ECOR_51       .......... .......... .......... .........C .T........ .......... ..........
ECOR_57       .......... .......... .......... .........C .T........ .......... ..........
UTI89         .......... .......... .......... .........C .T........ .......... ..........
E._albertii   ...C...... .........T .......... ..C....... T.AT.T...A .CTA.CCA.. TT.GC..TTT
```

```
         1261
K-12         ATATTTTCCT GCTTACCGCC ATTCAGCTGG TAGTACCTGT CGCAAATTCT TTACAGTTTT TAAACTAATG
53638        .......... .......... .......... .......... .......... .......... ..........
ATCC_8739    .......... .......... .......... .......... .......... .......... ..........
ECOR_10      .......... .......... .......... .......... .......... .......... ..........
ECOR_11      .......... .......... .......... .......... .......... .......... ..........
B171         .......... .......... .......... .......... .......... .......... ..........
E24377A      .......... .......... .......... .......... .......... .......... ..........
ECOR_34      .......... .......... .......... .......... .......... .......... ..........
ECOR_71      .......... .......... .......... .......... .......... .......... C.........
SE11         .......... .......... .......... .......... .......... .......... ..........
O157_Sakai   .......... .......... .......... .......... .......... .......... ..........
ECOR_35      .......... .......... .......... .......... .......... .......... ..........
ECOR_36      .......... .......... .......... .......... .......... .......... ..........
SMS-3-5      .......... .......... .......... .......... .......... .......... ..........
536          .......... .......A.T .......... .......... .......... .......... ..........
APEC_O1      .......... .........T .......... .......... .......... .......... ..........
CFT073       .......... .......... .......... .......... .......... .......... ..........
E2348/69     .......... .........T .......... .......... .......... .......... ..........
ECOR_51      .......... .......... .......... .......... .......... .......... ..........
ECOR_57      .......... .......... .......... .......... .......... .......... ..........
UTI89        .......... .........T .......... .......... .......... .......... ..........
E._albertii  -C...C...G A.G...TTT. .....A.C.T C.T.T..C.. ......A... ......C..C .........A

         1331
K-12         AGACGAATCT GATCGACGCA AAAAGTCCGT ATGCCTACTA TTAGCTCACG GTTATAAATC AACATATTGA
53638        .......... .......... .......... .......... .......... .......... ..........
ATCC_8739    .......... .......... .......... .......... .......... .......... ..........
ECOR_10      .......... .......... .......... .......... .......... .......... ..........
ECOR_11      .......... .......... .......... .......... .......... .......... ..........
B171         .......... .......... .......... .......... ......T... .......... ..........
E24377A      .......... .......... .......... .......... .......... .......... ..........
ECOR_34      .......... .......... .....C.... .......... ......T... .......... ..........
ECOR_71      .......... .......... .......... .......... ......T... .......... ..........
SE11         .......... .......... .......... .......... .......... .......... ..........
O157_Sakai   .......... .......... .......... .......... .......... .......... ..........
ECOR_35      .......... .......... .......... .......... .......... .......... ..........
ECOR_36      .......... .......... .......... .......... .......... .......... ..........
SMS-3-5      .......... .......... .......... .......... .......... .......... ..........
536          .......... .......... .......... .......... .......... .......... ..........
APEC_O1      .......... .......... .......... .......... .......... .......... ..........
CFT073       .......... .......... .......... .......... .......... .......... ..........
E2348/69     .......... .......... .......... .......... .......... .......... ..........
ECOR_51      .......... .......... .......... .......... .......... .......... ..........
ECOR_57      .......... .......... .......... .......... .......... .......... ..........
UTI89        .......... .......... .......... .......... .......... .......... ..........
E._albertii  .......... .......... .......... .......... .......... A.....G... ...G......

         1401
K-12         TTTATAAGCA TGGAAATCCC CTGAGTGAAA CAACGAATTG CTGTGTGTAG TCTTTGCCCA TCTCCCACGA
53638        .......... .......... .......... .......... .......... .......... ..........
ATCC_8739    .......... .......... .......... .......... .......... .......... ..........
ECOR_10      .......... .......... .......... .......... .......... .......... ..........
ECOR_11      .......... .......... .......... .......... .......... .......... ..........
B171         .......... .......... .......... .......... .......... .......... ..........
E24377A      .......... .......... .......... .......... .......... .......... ..........
ECOR_34      .......... .......... .......... .......... .......... .......... ..........
ECOR_71      .......... .......... .......... .......... .......... .......... ..........
SE11         .......... .......... .......... .......... .......... .......... ..........
O157_Sakai   .......... .......... .......... .......... .......... .......... ..........
ECOR_35      .......... .......... .......... .......... .......... .......... ..........
ECOR_36      .......... .......... .......... .......... .......... .......... ..........
SMS-3-5      .......... .......... .......... .......... .......... .......... ..........
536          .......... .......... .......... .......... .......... .......... ..........
APEC_O1      .......... .......... .......... .......... .......... .......... ..........
CFT073       .......... .......... .......... .......... .......... .......... ..........
E2348/69     .......... .......... .......... .......... .......... .......... ..........
ECOR_51      .......... .......... .......... .......... .......... .......... ..........
ECOR_57      .......... .......... .......... .......... .......... .......... ..........
UTI89        .......... .......... .......... .......... .......... .......... ..........
E._albertii  ........T. .......... .......... T......... .......... .......... ..........
```

```
            1471
K-12        TGGGCTTTTT TTTAACATTT TTCCGCTT-C GCTACCTCGC CCCTCACTCT TTCCAATAAT GTTGCACAAG
53638       .......... .......... ......A... .......... .......... .......... ..........
ATCC_8739   .......... .......... ......A... .......... .......... .......... ..........
ECOR_10     .......... .......... .......... .......... .......... .......... ..........
ECOR_11     .......... .......... .......... .......... .......... .......... ..........
B171        .......... .......... .......... .......... .......... .......... ..........
E24377A     .......... .......... .......... .......... .......... .......... ..........
ECOR_34     .......... .......... .......... .......... .......... .......... ..........
ECOR_71     .......... .......... .......... .......... .......... .......... ..........
SE11        .......... .......... .......... .......... .......... .......... ..........
O157_Sakai  .......... .......... .......... .......... .......... .......... ..........
ECOR_35     .......... .......... .......... .......... .......... .......... ..........
ECOR_36     .......... .......... .......... .......... .......... .......... ..........
SMS-3-5     .......... .......... .......... ..C....... .......G.. .......... ..........
536         .......... .......... ...T...... .......... .......... .......... ..........
APEC_O1     .......... .......... .......... .......... .......... .......... ..........
CFT073      .......... .......... .......... .......... .......... .......... ..........
E2348/69    .......... .......... .......... .......... .......... .......... ..........
ECOR_51     .......... .......... .......... .......... .......... .......... ..........
ECOR_57     .......... .......... .......... .......... .......... .......... ..........
UTI89       .......... .......... .......... .......... .......... .......... ..........
E._albertii .......... ..-....... .......CA. T.AT..-..T .T...GT... C......... ..........

            1541
K-12        ATACTGTGAC TCTCTTCAAG CCGTCAGCAG GCTGATAATG TTATTTATAT TTT-ATTGTT TATTTCATGC
53638       .......... .......... .......... .......... .......... .......... ..........
ATCC_8739   .......... .......... .......... .......... .......... .......... ..........
ECOR_10     .......... .......... .......... .......... .......... .......... ..........
ECOR_11     .......... .......... .......... .......... .......... .......... ..........
B171        .......... .......... .......... .......... .......... .......... ..........
E24377A     .......... .......... .......... .......... .......... .......... ..........
ECOR_34     .......... .......... .......... .......... .......... .......... ..........
ECOR_71     .......... .......... .......... .......... .......... .......... ..........
SE11        .......... .......... .......... .......... .......... .......... ..........
O157_Sakai  .......... .......... .......... .......... .......... .......... ..........
ECOR_35     .......... .......... .......... .......... .......... .......... ..........
ECOR_36     .......... .......... .......... .......... .......... .......... ..........
SMS-3-5     .......... .......... .......... .......... .......... .......... ..........
536         .......... .......... .......... .......... .......... .......... ..........
APEC_O1     .......... .......... .......... .......... .......... .......... ..........
CFT073      .......... .......... .......... .......... .......... .......... ..........
E2348/69    .......... .......... .......... .......... .......... .......... ..........
ECOR_51     .......... .......... .......... .......... .......... .......... ..........
ECOR_57     .......... .......... .......... .......... .......... .......... ..........
UTI89       .......... .......... .......... .......... .......... .......... ..........
E._albertii .......... .......... T...T..... .......G.. .......... ...T...C.. .T........

            1611
K-12        AGCCATTACA CGATGAACGC TTATGAACTC CAGGCACTCC GCCATATTTT TGCCATGACT ATTGATGAAT
53638       .......... .......... .......... .......... .......... .......... ..........
ATCC_8739   .......... .......... .......... .......... .......... .......... ..........
ECOR_10     .......... .......... .......... .......... .......... .......... ..........
ECOR_11     .......... .......... .......... .......... .......... .......... ..........
B171        .......... .......... .......... ........T. .......... .......... ..........
E24377A     .......... .......... .......... .......... .......... .......... ..........
ECOR_34     .......... .......... .......... ........T. .......... .......... ..........
ECOR_71     .......... .......... .......... ........T. .......... .......... ..........
SE11        .......... .......... .......... .......... .......... .......... ..........
O157_Sakai  .......... .......... .......... .......... .......... .......... ..........
ECOR_35     .......... .......... .......... ........T. .......... .......... ..........
ECOR_36     .......... .......... .......... ........T. .......... .......... ..........
SMS-3-5     .......... .......... .......... ........T. .......... .......... ..........
536         .......... .......... .......... ........T. .......... .......... ..........
APEC_O1     .......... .......... .......... ........T. .......... .......... ..........
CFT073      .......... .......... .......... ........T. .......... .......... ..........
E2348/69    .......... .......... .......... ........T. .......... .......... ..........
ECOR_51     .......... .......... .......... ........T. .......... .......... ..........
ECOR_57     .......... .......... .......... ........T. .......... .......... ..........
UTI89       .......... .......... .......... ........T. .......... .......... ..........
E._albertii ......A... .......... .......... ........A. .......... .A.T.....C ..........
```

```
           1681
K-12        GCGCGACCTG GATTGCCCAG ACCGGTGACA GTGAAAGCTG GCGTCAGTGG GAAAATGGCA AGTGTGCCAT
53638       .......... .......... .......... .......... ......A... .......... ..........
ATCC_8739   .......... .......... .T........ .......... ......A... .......... ..........
ECOR_10     .......... .......... .......... .......... .......... .......... ..........
ECOR_11     .......... .......... .......... .......... .......... .......... ..........
B171        .......... .......... .......... .......... .......... .......... .A..C.....
E24377A     .......... .......... .......... .......... .......... .......... ..........
ECOR_34     .......... .......... .......... .......... .......... .......... .A..C.....
ECOR_71     .......... .......... .......... .......... .......... .......... .A..C.....
SE11        .......... .......... .......... .......... ......A... .......... ..........
O157_Sakai  .......... .......... .......... .......... ......A... .......... G.........
ECOR_35     .......... .......... ....A..... .......... .......... .......... .A..C.....
ECOR_36     .......... .......... ....A..... .......... .......... .......... .A..C.....
SMS-3-5     .......... .......... .......... .......... .......... .......... .A..C.....
536         .......... .......... ......A... .......... .......... .......... .A..C.....
APEC_O1     .......... .......... ......A... .......... .......... .......... .A..C.....
CFT073      .......... .......... ......A... .......... .......... .......... .A..C.....
E2348/69    .......... .......... .......... .......... .......... .......... .A..C.....
ECOR_51     .......... .......... ......A... .......... .......... .......... .A..C.....
ECOR_57     .......... .......... ......A... .......... .......... .......... .A..C.....
UTI89       .......... .......... ......A... .......... .......... .......... .A..C.....
E._albertii .......... ........T. ..G....... .....G.T.. ..AG...... ........A. .AACA..T..

           1751
K-12        TCCTGATCGT GTAGTCGAGC AACTGTTGGC TATGCGTCAG CAAAGAAAAA AACATCTTCA TGCCATCATA
53638       .......T.. .......... .......... .......... .......... .......... ...G......
ATCC_8739   .......T.. .......... .......... .......... .......... .......... ...G......
ECOR_10     .......... .......... .......... .......... .......... .......... ..........
ECOR_11     .......... .......... .......... .......... .......... .......... ..........
B171        .......... .......... .......... .......... ...C...... .......... ..........
E24377A     ......G... .......... .......... .......... .......... .......... ..........
ECOR_34     .......... .......... .......... .......... ...C...... .......... ..........
ECOR_71     .......... .......... .......... .......... ...C...... .......... ..........
SE11        .......... .......... .......... .......... .......... .......... ..........
O157_Sakai  .......T.. .......... .......... .......... .......... .......... ...G......
ECOR_35     .......T.. .......... .......... .......... ...C...... .......... ..........
ECOR_36     .......T.. .......... .......... .......... ...C...... .......... ..........
SMS-3-5     .......T.. .......... .......... .......... ...C...... .......... ..........
536         .......T.. .......... .......... .......... ...C...... .......... ..........
APEC_O1     .......T.. .......... .......... .......... T..C...... .......... ..........
CFT073      .......T.. .......... .......... .......... ...C...... .......... ..........
E2348/69    .......T.. .....A.... .......... .......... ...C...... .......... ..........
ECOR_51     .......T.. .......... .......... .......... ...C...... .......... ..........
ECOR_57     .......T.. .......... .......... .......... ...C...... .......... ..........
UTI89       .......T.. .......... .......... .......... ...C...... .......... ..........
E._albertii ...C..CGAA ..C..T..AA ..T....... A......... .G........ ....C..... ..........

           1821
K-12        GAGAAAATAA ATAACCGCAT CGGCAACAAC ACCATGCGTT TTTTCCCCGA CTTAACCGCA TTTCAACGAG
53638       .......... .......... .......... .......... .......... .........G .......A..
ATCC_8739   .......... .......... .......... .......... .......... .........G .......A..
ECOR_10     .......... .......... .......... .......... .......... .......... ..........
ECOR_11     .......... .......... .......... .......... .......... .......... ..........
B171        .......... .......... .......... .......... .......... .........G .......A..
E24377A     .......... .......... .......... .......... .......... .......... ..........
ECOR_34     .......... .......... .......... .......... .......... .........G .......A..
ECOR_71     .......... .......... .......... .......... .......... .........G .......A..
SE11        .......... .......... .......... .......... .......... .......... ..........
O157_Sakai  .......... .......... .......... .......... .......... .........G .......A..
ECOR_35     .......... .......... .......... .......... .......... .........G .......A..
ECOR_36     .......... .......... .......... .......... .......... .........G .......A..
SMS-3-5     .......... .......... .......... .......... .......... .........G .......A..
536         .......... .......... .......... .......... .......... .........G .......A..
APEC_O1     .......... .......... .......... .......... .......... .........G .......A..
CFT073      .......... .......... .......... .......... .......... .........G .......A..
E2348/69    .......... .......... .......... .......... .......... .........G .......A..
ECOR_51     .......... .......... .......... .......... .......... .........G .......A..
ECOR_57     .......... .......... .......... .......... .......... .........G .......A..
UTI89       .......... .......... .......... .......... .......... .........G .......A..
E._albertii ..A....... .......T.. T..G..T... .......... .......T.. T...G.T... .......AG.
```

```
           1891
K-12         TCTACCCTGA TGGCAATTTT ATTGACTGGA AGATCTATCA ATCGGTAGCT GCTGAATTAT ATGCACACGA
53638        .......... .......... .......... .......... .........C .......... ..........
ATCC_8739    .......... .......... .......... .......... .........C .......... ..........
ECOR_10      .......... .......... .......... .......... .......... .......... ..........
ECOR_11      .......... .......... .......... .......... .......... .......... ..........
B171         .......... .......... .......... .......... .........C .......... ..........
E24377A      .......... .......... .......... .......... .........C .......... ..........
ECOR_34      .......... .......... .......... .......... .........C .......... ..........
ECOR_71      .......... .......... .......... .......... .........C .......... ..........
SE11         .......... .......... .......... .......... .........C .......... ..........
O157_Sakai   .......... .......... .......... .......... .........C .......... ..........
ECOR_35      .......... .......... .......... .......... .........C .......... ..........
ECOR_36      .......... .......... .......... .......... .........C .......... ..........
SMS-3-5      .......... .......... .......... .......... .........C .......... ..........
536          .......... .......... .......... .......... .........C .......... ..........
APEC_O1      .......... .......... .......... .......... .........C .......... ..........
CFT073       .......... .......... .......... .......... .........C .......... ..........
E2348/69     .......... .......... .......... .......... .........C .......... ..........
ECOR_51      .......... .......... .......... .......... .........C .......... ..........
ECOR_57      .......... .......... .......... .......... .........C .......... ..........
UTI89        .......... .......... .......... .......... .........C .......... ..........
E._albertii  .......... A..T...... .......... ....T..C.. ...C..C..A A.C....... .C..T..T..

           1961
K-12         TCTGGAGCGG CTTTGTTAAT TTTTCCACAG AAAGGAATTG TCGT--TGTT ACAACAATAA TGAACGGATG
53638        .......... .......... ..C......A .......... .......... .......... .........A
ATCC_8739    .......... .......... ..C......A .......... .......... .......... .........A
ECOR_10      .......... .....CC.G. ..C......A .......... .......... .......... ..........
ECOR_11      .......... .....CC.G. ..C......A .......... .......... .......... ..........
B171         .......... .......... ..C......A .......... .......... .......... .........A
E24377A      .......... .......... .......... .......... .......... .......... ....T....A
ECOR_34      .......... .....CC.G. ..C......A .......... .......... .......... .........A
ECOR_71      .......... .....CC.G. ..C......A .......... .......... .......... .........A
SE11         .......... .....C.... .......... .......... .......... .......... ....T....A
O157_Sakai   .......... .......... ..C......A .......... .......... .......... .........A
ECOR_35      .......... .....CC.G. ..C......A .......... .......... .......... .........A
ECOR_36      .......... .....CC.G. ..C......A .......... .......... .......... .........A
SMS-3-5      .......... .......... ..CC...... .......... .......... .......... .........A
536          .......... .......... ..C....... .......... .......... .......... .........A
APEC_O1      .......... .......... ..C....... .......... .......... .......... .........A
CFT073       .......... .......... ..C....... .......... .......... .......... .........A
E2348/69     .......... .......... ..C....... .......... .......... .......... .........A
ECOR_51      .......... .....CC.G. ..C......A .......... .......... .......... .........A
ECOR_57      .......... .....CC.G. ..C......A .......... .......... .......... .........A
UTI89        .......... .......... ..C....... .......... .......... .......... .........A
E._albertii  .........T .......G.. CCCCGGT..A .G.T.CTC.C ..ACCA.C.G G...A....G .....A...A

           2031
K-12         CTGACACAAC ATCGCTTCAC TTTTTAAAGC ACCTTTGCTA AGTAGAACCT ATGAAAAATC CCTATTTCCC
53638        .......... ......A... .......... .......... .......... .......... ......A...
ATCC_8739    .......... ......A... .......... .......... .......... .......... ......A...
ECOR_10      .......... .......... .......... .......... .......... .......... ..........
ECOR_11      .......... .......... .......... .......... .......... .......... ..........
B171         .......... ......A... .......... .......... .......... .......... ......A...
E24377A      .......... ......A... .......... .......... .......... .......... ......A...
ECOR_34      .......... ......A... .......... .......... .......... .......... ......A...
ECOR_71      .......... ......A... .......... .......... .......... .......... ......A...
SE11         .......... ......A... .......... .......... .......... .......... ......A...
O157_Sakai   .......... ......A... .......... .......... .......... .......... ......A...
ECOR_35      .......... ......A... .......... .......... .......... .......... ......A...
ECOR_36      .......... ......A... .......... .......... .......... .......... ......A...
SMS-3-5      .......... ......A... .......... .......... .......... .......... ......A...
536          .......... ......A... .......... .......... .......... .......... ......A...
APEC_O1      .......... ......A... .......... .......... .......... .......... ......A...
CFT073       .......... ......A... .......... .......... .......... .......... ......A...
E2348/69     .......... ......A... .......... .......... .......... .......... ......A...
ECOR_51      .......... ......A... .......... .......... .......... .......... ......A...
ECOR_57      .......... ......A... .......... .......... .......... .......... ......A...
UTI89        .......... ......A... .......... .......... .......... .......... ......A...
E._albertii  ....T.T... .....GA..- ......C... .AA....... ...G..T.T. .......... ..........
```

```
            2101
K-12        TACCGCACTT GGGTTGTATT TTAATTACCT GGTGCATGGT ATGGGCGTCC TTTTGATGAG CCTGAATATG
53638       .......... .......... .......... .......... .........A .......... ..........
ATCC_8739   .......... .......... .......... .......... .........A .......... ..........
ECOR_10     .......... .......... .......... .......... .........  .......... ..........
ECOR_11     .......... .......... .......... .......... .........  .......... ..........
B171        .......... .......... .......... .......... .........A .......... ..........
E24377A     .......... .......... .......... .......... .........A .......... ..........
ECOR_34     .......... .......... .......... .......... .........A .......... ..........
ECOR_71     .......... .......... .......... .......... .........A .......... ..........
SE11        .......... .......... .......... .......... .........A .......... ..........
O157_Sakai  .......... .......... .......... .......... .........A .......... ..........
ECOR_35     .......... .......... .......... .......... .........A .......... ..........
ECOR_36     .......... .......... .......... .......... .........A .......... ..........
SMS-3-5     .........G .......... .......T.. .......... .........A .......... ..........
536         .......... .......... .......... .......... .........A .......... ..........
APEC_O1     .......... .......... .......... .......... .........A .......... ..........
CFT073      .......... .......... .......... .......... .........A .......... ..........
E2348/69    .......... .......... .......... .......... .........A .......... ..........
ECOR_51     .......... .......... .......... .......... .........A .......... ..........
ECOR_57     .......... .......... .......... .......... .........A .......... ..........
UTI89       .......... .......... .......... .......... .........A .......... ..........
E._albertii ......G..G .......... .C........ .......... .........A .......... ..........

            2171
K-12        GCCTCGCTGG AGACACTTTG GCAGACTAAT GCCGCGGGTG TCTCGATAGT TATCTCATCG CTGGGCATTG
53638       .......... .......... .......... .......... .......... .......... ..........
ATCC_8739   .......... .......... .......... .......... .......... .......... ..........
ECOR_10     .......... .......... .......... .......... .......... .......... ..........
ECOR_11     .......... .......... .......... .......... .......... .......... ..........
B171        .......... .......... .......... .......... .......... .......... ..........
E24377A     .......... .......... .......... .......... .......... .......... ..........
ECOR_34     .......... .......... .......... .......... .......... .......... ..........
ECOR_71     .......... .......... .......... .......... .......... .......... ..........
SE11        .......... .......... .......... .......... .......... .......... ..........
O157_Sakai  .......... .......... ....T..... .......... .......... .......... ..........
ECOR_35     .......... ...TG..... .......... ........C. .......... .........A ..........
ECOR_36     .......... ...TG..... .......... ........C. .......... .........A ..........
SMS-3-5     .......... ....G..... .......... ........C. .......... .........A ..........
536         .......... ....G..... .......... ........C. .......... .........A ..........
APEC_O1     .......... ....G..... .......... ........C. .......... .........A ..........
CFT073      .......... ....G..... .......... ........C. .......... .........A ..........
E2348/69    .......... ....G..... .......... ........C. .......... .........A ..........
ECOR_51     .......... ....G..... .......... ........C. .......... .........A ..........
ECOR_57     .......... ....G..... .......... ........C. .......... .........A ..........
UTI89       .......... ....G..... .......... ........C. .......... .........A ..........
E._albertii .......... .A..G..G.. ...T..C... ..A.....A. ....CG.C.. .........A ..A..T....

            2241
K-12        GTCGATTAAG TGTCTTGCTT TTTGCAGGAT TATTATCCGA TCGCTTTGGT CGCCGCCCTT TTATCATGCT
53638       .......... .......... .......... .......... .......... .......... ..........
ATCC_8739   .......... .......... .......... .......... .......... .......... ..........
ECOR_10     .......... .......... .......... .......... .......... .......... ..........
ECOR_11     .......... .......... .......... .......... .......... .......... ..........
B171        .......... .......... .......... .......... .......... .......... ..........
E24377A     .......... .......... .......... .......... .......... .......... ..........
ECOR_34     .......... .......... .......... .......... .......... .......... ..........
ECOR_71     .......... .......... .......... .......... .......... .......... ..........
SE11        .......... .......... .......... .......... .......... .......... ..........
O157_Sakai  .......... .......... .......... .......... .......... .......... ..........
ECOR_35     .......... .......... .......... .......... .......... .......... ..........
ECOR_36     .......... .......... .......... .......... .......... .......... ..........
SMS-3-5     .......... .......... .......... .......... .......... .......... ..........
536         .......... .......... .......... .......... .......... .......... ..........
APEC_O1     .......... .......... .......... .......... .......... .......... ..........
CFT073      .......... .......... .......... .......... .......... .......... ..........
E2348/69    .......... .......... .......... .......... .......... .......... ..........
ECOR_51     .......... .......... .......... .......... .......... .......... ..........
ECOR_57     .......... .......... .......... .......... .......... .......... ..........
UTI89       .......... .......... .......... .......... .......... .......... ..........
E._albertii .C........ C..TC..T.. C.G..C.... .......... .......... ..T..A..A. .......T..
```

```
          2311
K-12          CGGGATGTGC TGCTATATGG CCTTCTTTTT TGGCATCCTG CAGACCAATA ACATCATTAT CGCTTATGTT
53638         .......... .......... .......... .......... .......... .......... ..........
ATCC_8739     .......... .......... .......... .......... .......... .......... ..........
ECOR_10       .......... .......... .......... .......... .......... .......... ..........
ECOR_11       .......... .......... .......... .......... .......... .......... ..........
B171          .......... .......... .......... .......... ..C....... .......... ...C......
E24377A       .......... .......... .......... .......... ..C....... .......... ..........
ECOR_34       .......... .......... .......... .......... ..C....... .......... ..........
ECOR_71       .......... .......... .......... .......... ..C....... .......... ..........
SE11          .........T .......... .......... .......... .......... .......... ..........
O157_Sakai    .......... .......... .......... .......... .......... .......... ..........
ECOR_35       .......... .......... .......... .......... ..C....... .......... ..........
ECOR_36       .......... .......... .......... .......... ..C....... .......... ..........
SMS-3-5       .......... .......... .......... .......... ..C....... .......... ..........
536           .......... .......... .......... .......... ..C....... .......... ..........
APEC_O1       .......... .......... .......... .......... ..C....... .......... ..........
CFT073        .......... .......... .......... .......... ..C....... .......... ..........
E2348/69      .......... .......... .......... .......... ..C....... .......... ..........
ECOR_51       .......... .......... .......... .......... ..C....... .......... ..........
ECOR_57       .......... .......... .......... .......... ..C....... .......... ..........
UTI89         .......... .......... .......... .......... ..C....... .......... ..........
E._albertii   ...T..AA.. .....CC... .A........ ...T..T... ..CG.G.... .........C ..........

          2381
K-12          TTTGGCTTTC TGGCGGGAAT GGCAAACAGT TTTCTCGATG CAGGCACTTA TCCCAGTTTG ATGGAAGCTT
53638         .......... .......... .......... .......... .......... .......... ..........
ATCC_8739     .......... .......... .......... .......... .......... .......... ..........
ECOR_10       .......... .......... .......... .......... .......... .......... ..........
ECOR_11       .......... .......... .......... .......... .......... .......... ..........
B171          .......... .......... .......... .......... .......... .......... ..........
E24377A       .......... ....A..... .......... .......... .......... .......... ..........
ECOR_34       .......... ....A..... .......... .......... .......... .......... ..........
ECOR_71       .......... ....A..... .......... .......... .......... .........A ..........
SE11          .......... .......... .......... .......... .......... .......... ..........
O157_Sakai    .......... .......... .......... .......... .......... .......... ..........
ECOR_35       .......... .......... .......... .......... .......C.. .......... ..........
ECOR_36       .......... .......... .......... .......... .......C.. .......... ..........
SMS-3-5       .......... .......... .......... .......... .......C.. .......... ..........
536           .......... .......... .......... .......... .......C.. .......... ..........
APEC_O1       .......... .......... .......... .......... .......C.. .......... ..........
CFT073        .......... .......... .......... .......... .......C.. .......... ..........
E2348/69      .......... .......... .......... .......... .......C.. .......... ..........
ECOR_51       .......... .......... .......... .......... .......C.. .......... ..........
ECOR_57       .......... .......... .......... .......... .......C.. .......... ..........
UTI89         .......... .......... .......... .......... .......C.. .......... ..........
E._albertii   .....T.... ....T..... ......T... .....T.... .C..A..C.. ...A..CC.. .....G..A.

          2451
K-12          TTCCACGCTC ACCTGGGACA GCCAATATTT TAATTAAAGC ATTTGTTTCC AGCGGACAAT TTTTATTACC
53638         .......... .......... .......... .......... .......... .......... ..........
ATCC_8739     .......... .......... .......... .......... .......... .......... ..........
ECOR_10       .......... .......... .......... .......... .......... .......... ..........
ECOR_11       .......... .......... .......... .......... .......... .......... ..........
B171          .......... .......... .......... .......... .......... .......... ..........
E24377A       .......... .......... .......... .......... .......... .......... ..........
ECOR_34       .......... .......... .......... .......... .......... .......... ..........
ECOR_71       .......... .......... .......... .......... .......... .......... ..........
SE11          .......... .......... .......... .......... .......... .......... ..........
O157_Sakai    .......... .......... .......... .......... .......... .......... ..........
ECOR_35       .......... ...G..T... .......... .......... .......... .......... ..........
ECOR_36       .......... ...G..T... .......... .......... .......... .......... ..........
SMS-3-5       .......... ...G..T... .......... .......... .......... .......... ..........
536           .......... ...G..T... .......... .......... .......... .......... ..........
APEC_O1       .......... ...G..T... .......... .......... .......... .......... ..........
CFT073        .......... ...G..T... .......... .......... .......... ........G. ..........
E2348/69      .......... ...G..T... .......... .......... .......... ..T....... ..........
ECOR_51       .......... ...G..T... .......... .......... .......... ........G. ..........
ECOR_57       .......... ...G..T... .......... .......... .......... ........G. ..........
UTI89         .......... ...G..T... .......... .......... .......... .......... ..........
E._albertii   ....T..T.. G..A..C..G ..A....... ....C..... G......... ..T....... ..........
```

```
          2521
K-12         GCTAATCATT AGCCTGTTAG TGTGGGCTGA ACTGTGGTTC GGTTGGTCCT TTATGATTGC TGCAGGCATT
53638        .......... .......... .......... .......... .......... .......... ..........
ATCC_8739    .......... .......... .......... .......... .......... .......... ..........
ECOR_10      .......... .......... .......... .......... .......... .......... ..........
ECOR_11      .......... .......... .......... .......... .......... .......... ..........
B171         .......... .......... .......... .......... .......... .......... ..........
E24377A      .......... .......... .......... .......... .......... .......... ..........
ECOR_34      .......... .......... .......... .......... .......... .......... ..........
ECOR_71      .......... .......... .......... .......... .......... .......... ..........
SE11         .......... .......... .......... .......... .......... .......... ..........
O157_Sakai   .......... .......... .......... .......... .......... .......... ..........
ECOR_35      ......T..C .......... .......... .......... .......... .......... C.........
ECOR_36      ......T..C .......... .......... .......... .......... .......... C.........
SMS-3-5      ......T..C .......... .......... .......... .......... .......... C.........
536          ......T..C .......... .......... .......... .......... .......... C.........
APEC_O1      ......T..C .......... .......... .......... .......... .......... C.........
CFT073       ......T..C .......... .......... .......... .......... .......... C.........
E2348/69     .........C ......C... .......... .......... .......... .......... C.........
ECOR_51      ......T..C .......... .......... .......... .......... .......... C.........
ECOR_57      ......T..C .......... .......... .......... .......... .......... C.........
UTI89        ......T..C .......... .......... .......... .......... .......... C.........
E._albertii  .T....T... .......... .C........ GT........ ........A. .......C.. C.........

          2591
K-12         ATGTTTATTA ACGCTCTGTT TTTATACCGT TGTACGTTCC CACCCCATCC GGGTCGTCGC TTACCTGTCA
53638        .......... .......... .......... .......... .......... .......... .C........
ATCC_8739    .......... .......... .......... .......... .......... .......... ..........
ECOR_10      .......... .......... .......... .......... .......... .......... ..........
ECOR_11      .......... .......... .......... .......... .......... .......... ..........
B171         .......... .......... .......... .......... .......... .......... ..........
E24377A      .......... .......... .......... .......... .......... .....A.... ..........
ECOR_34      .......... .......... .......... .......... .......... .....A.... ..........
ECOR_71      .......... .......... ........T. .......... .......... .....A.... ..........
SE11         ...C...... .......... .......... .......... .......... .......... ..........
O157_Sakai   .......... .......... .......... .......... .......... .......... ..........
ECOR_35      .......... .......... .......... .......... .......... ........A. ..........
ECOR_36      .......... .......... .......... .......... .......... ........A. ..........
SMS-3-5      .......... .......... .......... .......... .......... .......... ..........
536          .......... .......... .......... .......... .......... ........A. ..........
APEC_O1      .......... .......... .......... .......... .......... ........A. ..........
CFT073       .......... .......... .......... .......... .......... ........A. ..........
E2348/69     .......... .......... .......... .......... .......... .......... ..........
ECOR_51      .......... .......... .......... .......... .......... ........A. ..........
ECOR_57      .......... .......... .......... .......... .......... ........A. ..........
UTI89        .......... .......... .......... .......... .......... ........A. ..........
E._albertii  ...A.C.... .T.....C.. .....GT..C .....C..T. .G..A..... ...A..G.AA G.CGTCACGC

          2661
K-12         TAAAGAAAAC CACCAGCTCT ACGGAACATC GCTGTTCAAT TATCGATTTA GCCAGTTATA CCTTATATGG
53638        .......... .......... .......... .......... .......... .......... ..........
ATCC_8739    .......... .......... .......... .......... .......... .......... ..........
ECOR_10      .......... .......... .......... .......... .......... .......... ..........
ECOR_11      .......... .......... .......... .......... .......... .......... ..........
B171         .......... .......... .......... .......... .......... .......... ..........
E24377A      .......... .......... .......... .......... .......... .......... ..........
ECOR_34      .......... .......... .......... .......... .......... .......... ..........
ECOR_71      .......... .......... .......... .......... .......... .......... ..........
SE11         .......... ....C..... .......... .......... .......... .......... ..........
O157_Sakai   .......... .......... .......... .......... .......... .......... ..........
ECOR_35      .......... .......... .......... .......... .......... .........T ..C.......
ECOR_36      .......... .......... .......... .......... .......... .........T ..C.......
SMS-3-5      .......... .......... .......... .......... .......... .......... ..........
536          .......... .......... .......... .......... .......... .........T ..C.......
APEC_O1      .......... .......... .......... .......... .......... .........T ..C.......
CFT073       .......... .......... .......... .......... .......... .........T ..C.......
E2348/69     .......... .......... .......... .......... .......... .......... ..........
ECOR_51      .......... .......... .......... .......... .......... .........T ..C.......
ECOR_57      .......... .......... .......... .......... .......... .........T ..C.......
UTI89        .......... .......... .......... .......... .......... .........T ..C.......
E._albertii  C.G.A...CT ..AA.CGC.. T...CG.... .......... .......C.C A....C..C. ..........
```

```
           2731
K-12         CTATATCTCA ATGGCAACGT TTTATCTGGT TAGCCAGTGG CTGGCACAGT ACGGACAATT TGTTGCAGGC
53638        .......... .......... .......... .......... .......... .......... ..........
ATCC_8739    .......... .......... .......... .......... .......... .......... ..........
ECOR_10      .......... .......... .......... .......... .......... .......... ..........
ECOR_11      .......... .......... .......... .......... .......... .......... ..........
B171         .......... .......... .......... .......... .......... .......... ..........
E24377A      .......... .......... .......... .......... .......... .......... ..........
ECOR_34      .......... .......... .......... .......... .......... .......... ..........
ECOR_71      .......... .......... .......... .......... .......... .......... ..........
SE11         .......... .......... .......... .......... ...T...... .......... ..........
O157_Sakai   .......... .......... .......... .......... .......... .......... ..........
ECOR_35      .......... .......... .......... .......... ........A. .......G.. ...C..T..A
ECOR_36      .......... .......... .......... .......... ........A. .......G.. ...C..T..A
SMS-3-5      .......... .......... .......... .......... ........A. .......... ..........
536          .......... .......... .......... .......... ........A. .......... ...C..T...
APEC_O1      .......... .......... .......... .......... ........A. .......... ...C..T...
CFT073       .......... .......... .......... .......... ........A. .......... ...C..T...
E2348/69     .......... .......... .......... .......... .......... .......... ..........
ECOR_51      .......... .......... .......... .......... .......... .......... ..........
ECOR_57      .......... .......... .......... .......... ........A. .......... ...C..T...
UTI89        .......... .......... .......... .......... ........A. .......... ...C..T...
E._albertii  ......T... ........A. .C......A. A.....A... .....G..A. .T..G..... ......C..A

           2801
K-12         ATGTCATACA CTATGTCGAT CAAACTACTC AGTATCTACA CCGTGGGTTC GCTGCTTTGT GTATTTATTA
53638        .......... .......... .......... .......... .......... .......... ..........
ATCC_8739    .......... .......... .......... .......... .......... .......... ..........
ECOR_10      .......... .......... .......... .......... .......... .......... ..........
ECOR_11      .......... .......... .......... .......... .......... .......... ..........
B171         .......... .......... T......... .......... .......... .......... ..........
E24377A      .......... .......... T......... .......... .......... .......... ..........
ECOR_34      .......... .......... .......... .......... .......... .......... ..........
ECOR_71      .......... .......... T......... .......... .......... .......... ..........
SE11         .......... .......... .......... .......... .......... .......... ..........
O157_Sakai   .......... .......... .......... .......... .......... .......... ..........
ECOR_35      .......... .......... .......... .......... .......... .......... ..........
ECOR_36      .......... .......... .......... .......... .......... .......... ..........
SMS-3-5      .......... .......... .......... .......... .......... .......... ..........
536          .......... .C........ ....T..... .......... .......... ......G... ..........
APEC_O1      .......... .C........ ....T..... .......... .......... ......G... ..........
CFT073       .......... .C........ ....T..... .......... .......... ......G... ..........
E2348/69     .......... .......... .........T .......... .......... .......... ..........
ECOR_51      .......... .......... .......... .......... .......... .......... ..........
ECOR_57      .......... .......... .......... .......... .......... .......... ..........
UTI89        .......... .C........ ....T..... .......... .......... ......G... ..........
E._albertii  .....C.... .......A.. A...T....A ..C..T..T. .G..A..A.. .........C ........C.

           2871
K-12         CCGCTCCACT CATTCGTAAT ACCGTTCGCC CAACAACATT ACTGATGCTG TACACCTTTA TCTCATTTAT
53638        .......... .......... .......... .......... .......... .......... ..........
ATCC_8739    .......... .......... .......... .......... .......... .......... ..........
ECOR_10      .......... .......... .......... .......... .......... .......... ..........
ECOR_11      .......... .......... .......... .......... .......... .......... ..........
B171         .......... .......... .......... .......... .......... .........T ..........
E24377A      .......... .......... .......... .......... .......... .......... ..........
ECOR_34      .......... .......... .......... .......... .......... .......... ..........
ECOR_71      .......... .......... .......... .......... .......... .......... ..........
SE11         .......... ......A... .......... .......... .......... .......... ..........
O157_Sakai   .......... .......... .......... .......... .......... .......... ..........
ECOR_35      .......... .......... .......... .......... .......... .......... ..........
ECOR_36      .......... .......... .......... .......... .......... .......... ..........
SMS-3-5      .......... .......... .......... .......... .......... .......... ..........
536          .T........ .......... .......... .......... .......... .......... ..........
APEC_O1      .T........ .......... .......... .......... .......... .......... ..........
CFT073       .T........ T......... .......... .......... .......... .......... ..........
E2348/69     .......... .......... .......... .......... .......... .......... .........C
ECOR_51      .......... .......... .......... .......... .......... .......... ..........
ECOR_57      .......... .......... .......... .......... .......... .......... ..........
UTI89        .T........ .......... .......... .......... .......... .......... ..........
E._albertii  .T..G..... G..A.....A G....A..TG .T...T.C.. .T.......T ..T..T.... .T..G.....
```

```
          2941
K-12         TGCTCTGTTT ACCGTCTGCC TGCATCCCAC ATTTTATGTG GTGATAATAT TTGCTTTTGT CATTGGTTTT
53638        .......... .......... .......... .......... .......... .......... ..........
ATCC_8739    .......... .......... .......... .......... .......... .......... ..........
ECOR_10      .......... .......... .......... .......... .......... .......... ..........
ECOR_11      .......... .......... .......... .......... .......... .......... ..........
B171         .......... .......... .......... .......... .......... .......... ..........
E24377A      .......... .......... .......... .......... .......... .......... ..........
ECOR_34      .......... .......... .......... .......... .......... ..T....... ..........
ECOR_71      .......... .......... .......... .......... .......... .......... ..........
SE11         .......... .......... .......... .......... .......... .......... ..........
O157_Sakai   .......... .......... .......... .......... .......... ..T....... ..........
ECOR_35      C..C...C.C .......... .......... .......... .......... .......C.. ...C......
ECOR_36      C..C...C.C .......... .......... .......... .......... .......C.. ...C......
SMS-3-5      C..C...C.C .......... .......... .......... .......... .......C.. ...C......
536          C..C...C.C .......... .......... .......... .......... .......C.. ...C......
APEC_O1      C..C...C.C .......... .......... .......... .......... .......C.. ...C......
CFT073       C..C...C.C .......... .......... .......... .......... .......C.. ...C......
E2348/69     .......... ........T. .......... .......... .......... .......C.. ...C......
ECOR_51      .......... .......... .......... .......... .......... .......... ..........
ECOR_57      .......... .......... .......... .......... .......... ..T....... ..........
UTI89        C..C...C.C .......... .......... .......... .......... .......C.. ...C......
E._albertii  ......AC.C .T........ .A..A..... .......... .....CGCT. .......C.. ...C......

          3011
K-12         ACCTCTGCTG GAGGTGTTGT GCAAATTGGC CTGACGTTAA TGGCTGAACG TTTCCCTTAC GCTAAAGGTA
53638        .......... .......... .......... .......... ........T. .......... ..........
ATCC_8739    .......... .......... .......... .......... .......... .......... ..........
ECOR_10      .......... .......... .......... .......... .......... .......... ..........
ECOR_11      .......... .......... .......... .......... .......... .......... ..........
B171         .......... .......... .......... .......... .......... .......... ..........
E24377A      .......... .......... .......... .......... .......... .......... ..........
ECOR_34      ..T....... .......... .......... .......... .......... .......... ..........
ECOR_71      .......... .......... .......... .......... .......... .......... ..........
SE11         .......... .......... .......... .......... .......... ......C... ..........
O157_Sakai   ..T....... .......... .......... .......... .......... .......... ..........
ECOR_35      .....C..A. .C........ A......... .......... ....G..... ...T..C... ........A.
ECOR_36      .....C..A. .C........ A......... .......... ....G..... ...T..C... ........A.
SMS-3-5      .....C..A. .C........ A......... .......... ....G..... ...T..C... ........A.
536          .....C..A. .C........ A......... .......... ....G..... ...T..C... ........A.
APEC_O1      .....C..A. .C........ A......... .......... ....G..... ...T..C... ........A.
CFT073       .....C..A. .C........ A......... .......... ....G..... ...T..C... ........A.
E2348/69     .....C..A. .C........ A......... .......... ....G..... ...T..C... ........A.
ECOR_51      .......... .......... .......... .......... ....G..... ...T..C... ........A.
ECOR_57      ..T....... .......... A......... .......... ....G..... ...T..C... ........A.
UTI89        .....C..A. .C........ A......... .......... ....G..... ...T..C... ........A.
E._albertii  .....C..A. .C........ A......... .......... ....C..... ...T..CC.. ..C.....C.

          3081
K-12         AAGCTACAGG GATCTATTAC AGTGCGGGCA GTATTGCGAC CTTTACTATT CCGTTGATTA CGGCTCATCT
53638        .......... .......... .......... .......... .......... .......... ..........
ATCC_8739    .......... .......... .......... .......... .......... .......... ..........
ECOR_10      .......... .......... .......... .......... .......... .......... ..........
ECOR_11      .......... .......... .......... .......... .......... .......... ..........
B171         .......... .......... .......... .......... .......... .......... ..........
E24377A      .......... .......... .......... .......... .......... .......... ..........
ECOR_34      .......... .......... .......... .......... .......... .......... ..........
ECOR_71      .......... .......... .......... .......... .......... .......... ..........
SE11         .......... .......... .......... .......... .......... .......... ..........
O157_Sakai   .......... .......... .......... .......... .......... .......... ..........
ECOR_35      ....A..G.. .......... .......... .C.....A.. A.....C... .......... ..........
ECOR_36      ....A..G.. .......... .......... .C.....A.. A.....C... .......... ..........
SMS-3-5      ....A..G.. .......... .......... .C.....A.. A.....C... .......... ..........
536          ....A..G.. .......... .......... .C.....A.. A.....C... .......... ..........
APEC_O1      ....A..G.. .......... .......... .C.....A.. A.....C... .......... ..........
CFT073       ....A..G.. .......... .....T.... .C.....A.. A.....C... .......... ..........
E2348/69     ....A..G.. .......... .......... .C.....A.. A.....C... .......... ..........
ECOR_51      ....A..G.. .......... .....T.... .C.....A.. A.....C... .......... ..........
ECOR_57      ....A..G.. .......... .....T.... .C.....A.. A.....C... .......... ..........
UTI89        ....A..G.. .......... .......... .C.....A.. A.....C... .......... ..........
E._albertii  ....A..... .........T ........T. .C.....A.. A.....C... .......... .C..G.....
```

```
         3151
K-12         GTCCCAAAGA AGTATTGCCG ATATTATGTG GTTCGATACC GCCATCGCTG CCATCGGTTT TTTACTGGCA
53638        .......... .......... .......... .......... .......... .......... ..........
ATCC_8739    .......... .......... .......... .......... .......... .......... ..........
ECOR_10      .......... .......... .......... .......... .......... .......... ..........
ECOR_11      .......... .......... .......... .......... .......... .......... ..........
B171         .......... .......... .......... .......... .......... .......... ..........
E24377A      .......... .......... .......... .......... .......... .......... ..........
ECOR_34      .......... .......... .......... .......... A......... .......... ..........
ECOR_71      .......... .......... .......... .......... .......... .......... ..........
SE11         .......... .......... .......... .......... .......... .......C.. ..........
O157_Sakai   .......... .......... .......... .......... .......... .......... ..........
ECOR_35      T..G...... .......... .......... .......... .......... .......C.. .A........
ECOR_36      T..G...... .......... .......... .......... .......... .......C.. .A........
SMS-3-5      T..G...... .......... .......... .......... .......... .......C.. .A........
536          T..G...... .......... .......... .......... .......... .T.....C.. .A........
APEC_O1      T..G...... .......... .......... .......... .......... .T.....C.. .A........
CFT073       T..G...... .......... .......... .......... .......... .T.....C.. .A........
E2348/69     T..G...... .......... .......... .......... .......... .T.....C.. .A........
ECOR_51      T..G...... .......... .......... .......... .......... .T.....C.. .A........
ECOR_57      T..G...... .......... .......... .......... .......... .T.....C.. .A........
UTI89        T..G...... .......... .......... .......... .......... .T.....C.. .A........
E._albertii  T..AG..... .......... .......... .........A T.T..T..C. ..G....C.. ....T....G

         3221
K-12         CTGTTTATCG GCTTACGCAG CCGCAAAAAA ACGCGGCATC ACTCGCTAAA GGAAAATGTC GCTCCGGGTG
53638        .......... .......... .......... .......... .......... .......... ..........
ATCC_8739    .......... .......... .......... .......... .......... .......... ..........
ECOR_10      .......... .......... .......... .......... .......... .......... ..........
ECOR_11      .......... .......... .......... .......... .......... .......... ..........
B171         .......... .......... .......... .......... .......... .......... ..........
E24377A      .......... .......... .......... .......... .......... .......... ..........
ECOR_34      .......... .......... .......... .......... .G........ .......... ..........
ECOR_71      .......... .......... .......... .......... .......... .......... ..........
SE11         .......... .......... .......... .......... .......... .......... ..........
O157_Sakai   .......... .......... .......... .......... .......... .......... ..........
ECOR_35      ........T. .......... T......G.. .......... .......... .......... ..........
ECOR_36      ........T. .......... T......G.. .......... .......... .......... ..........
SMS-3-5      ........T. .......... T......G.. .......... .......... .......... ..........
536          ........T. .......... T......G.. ......A... ..A....... .......A.. ..........
APEC_O1      ........T. .......... T......G.. .......... ..A....... .......A.. ..........
CFT073       ........T. .......... T......G.. .......... ..A....... .......A.. ..........
E2348/69     ........T. .......... T......G.. .......... ..A....... .......A.. ..........
ECOR_51      ........T. .......... T......G.. .......... ..A....... .......A.. ..........
ECOR_57      ........T. .......... T......G.. .......... ..A....... .......A.. ..........
UTI89        ........T. .......... T......G.. .......... ..A....... .......A.. ..........
E._albertii  ........T. .TC.G..... .A.A...G.. G.A.AA.... .G..AG..C. ......C..T ..CA.A...C

         3291
K-12         GGTAATGCAA TATTCTTTTC AGGTCATGCA AGATCTTACG -GATAAATAA CTCTTTCTGC ---GCTAACT
53638        .......... .......... .T........ .......... .......... .......... ..........
ATCC_8739    .......... .......... .......... .......... .......... .......... ..........
ECOR_10      .......... .......... .......... .......... .......... .......... ..........
ECOR_11      .......... .......... .......... .......... .......... .......... ..........
B171         .......... .......... .......... .......... .......... .......... ..........
E24377A      .......... .......... .......... .......... .......... .......... ..........
ECOR_34      .......... .......... .......... .......... .......... .......... ..........
ECOR_71      .......... .......... .......... .......... .......... .......... ..........
SE11         .......... .......... .......... .......... .......... .......... ..........
O157_Sakai   .......... .......... .......... .......... .......... .......... ..........
ECOR_35      .......... .......... .......... .......... .......... .......... ..........
ECOR_36      .......... .......... .......... .......... .......... .......... ..........
SMS-3-5      .......... .......... ....T..... .......... .......... .......... ..........
536          .......... .......... ....T..... .......... .......... .......... ..........
APEC_O1      .......... .......... ....T..... .......... .......... .......... ..........
CFT073       .......... .......... ....T..... .......... .......... .......... ..........
E2348/69     .......... .......... ....T..... .......... .......... .......... ..........
ECOR_51      .......... .......... ....T..... .......... .......... .......... ..........
ECOR_57      .......... .......... ....T..... .......... .......... .......... ..........
UTI89        .......... .......... ....T..... .......... .......... .......... ..........
E._albertii  TC.G...... C...A....T GTTA.G.... .......... A..A...GC. A.TA...A.T CAT...C.GA
```

```
           3361
K-12          AAGGAAAATC GCGATCAAAA ACAA-ACT-- ----ATGACA TGCAATATTC CTGGAAACAT AAACTTTATG
53638         .......... .......... .......... .......... .......... .......... ..........
ATCC_8739     .......... .......... .......... .......... .......... .......... ..........
ECOR_10       .......... .......... .......... .......... .......... .......... ..........
ECOR_11       .......... .......... .......... .......... .......... .......... ..........
B171          .......... .......... .......... .......... .......... .......... ..........
E24377A       .......... .......... .......... .......... .......... .......... ..........
ECOR_34       .......... .......... .......... .......... .......... .......... ..........
ECOR_71       .......... .......... .......... .......... .......... .......... ..........
SE11          .......... .......... .......... .......... .......... .......... ..........
O157_Sakai    .......... .......... .......... .......... .......... .......... ..........
ECOR_35       .......... .......... .......... .......... .......... .......... ..........
ECOR_36       .......... .......... .......... .......... .......... .......... ..........
SMS-3-5       .......... .......... .......... .......... .......... .......... ..........
536           .......... .......... .......... .......... .......... .......... ..........
APEC_O1       .......... .......... .......... .......... .......... .......... ..........
CFT073        .......... .......... .......... .......... .......... .......... ..........
E2348/69      .......... .......... .......... .......... .......... .......... ..........
ECOR_51       .......... .......... .......... .......... .......... .......... ..........
ECOR_57       .......... .......... .......... .......... .......... .......... ..........
UTI89         .......... .......... .......... .......... .......... .......... ..........
E._albertii   G......GC. CGC.AA..C. G...G...CG CAAT..T..G ........C. .......G.. ..........

           3431
K-12          CCATGTACCC AGGGAAAATC ATCTTCAGTA TAGTAATTAT GTAAACCGTC GGAGAACAAT ACGTACGGTA
53638         .......... .....T.... .......... .......... .......... .......... ..........
ATCC_8739     .......... .......... .......... .......... .......... .......... ..........
ECOR_10       .......... .......... .......... .......... .......... .......... ..........
ECOR_11       .......... .......... .......... .......... .......... .......... ..........
B171          .......... .......... .......... .......... .......... .......... ..........
E24377A       .......... .......... .......... .......... .......... .......... ..........
ECOR_34       .......... .......... .......... .......... .......... .......... ..........
ECOR_71       .......... ...A...... .......... .......... .......... .A........ ..........
SE11          .......... .......... .......... .......... .......... .A........ ..........
O157_Sakai    .......... .......... .......... .......... .......... .......... ..........
ECOR_35       .......... .......... .......... .......... .......... .......... ..........
ECOR_36       .......... .......... .......... .......... .......... .......... ..........
SMS-3-5       .......A.. .......... .......... .......... .......... .......... ..........
536           .......... .......... .......... .......... .......... .......... ..........
APEC_O1       .......... .......... .......... .......... .......... .......... ..........
CFT073        .......... .......... .......... .......... .......... .......... ..........
E2348/69      .......... .......... .......... .......... .......... .......... ..........
ECOR_51       .......... .......... .......... .......... .......... .......... ..........
ECOR_57       .......... .......... .......... .......... .......... .......... ..........
UTI89         .......... .......... .......... .......... .......... .......... ..........
E._albertii   .......... ..AA.G..A. .A........ ..T..C...A ........G. AA.TG...-. ...C......

           3501
K-12          ACGAAATTA- -TCT----TT CAGCAAGGAG CTGTGAAAAT GTCTCAAAAT AAGGCTTTCA GCACGCCATT
53638         .......A.. .......... .......... .......... .......... .......... ..........
ATCC_8739     .......... .......... .......... .......... .......... .......... ..........
ECOR_10       .......... .......... .......... .......... .......... .......... ..........
ECOR_11       .......... .......... .......... .......... .......... .......... ..........
B171          .......... .......... .......... .......... .......... .......... ..........
E24377A       .......... .......... .......... .......... .......... .......... ..........
ECOR_34       .......... .......... .......... .......... .......... .......... ..........
ECOR_71       .......... .......... .......... .......... .......... .......... ..........
SE11          .......... .......... .......... .......... .......... .......... ..........
O157_Sakai    .......... .......... .......... .......... .......... .......... ..........
ECOR_35       .......... .......... .......... .......... .......... .......... ..........
ECOR_36       .......... .......... .......... .......... .......... .......... ..........
SMS-3-5       .......... .......... .......... .......... .......... .......... ..........
536           .......... .......... .......... .......... .......... .......... ..........
APEC_O1       .......... .......... .......... .......... .......... .......... ..........
CFT073        .......... .......... .......... .......... .......... .......... ..........
E2348/69      .......... .......... .......... .......... .......... .......... ..........
ECOR_51       .......... .......... .......... .......... .......... .......... ..........
ECOR_57       .......... .......... .......... .......... .......... .......... ..........
UTI89         .......... .......... .......... .......... .......... .......... ..........
E._albertii   .TA....ATT T...CATT.. .......... .......... .......... .......... ..........
```

```
          3571
K-12        TATCCTGGCT GTTCTTTGTA TTTACTTCAG CTACTTCCTG CACGGCATTA GTGTTATTAC GCTTGCCCAA
53638       .......... .......... .......... .......... .......... .......... ..........
ATCC_8739   .......... .......... .......... .......... .......... .......... ..........
ECOR_10     .......... .......... .......... .......... .......... .......... ..........
ECOR_11     .......... .......... .......... .......... .......... .......... ..........
B171        .......... .......... .......... ...T...... .......... .......... ..........
E24377A     .......... .......... .......... .......... .......... .......... ..........
ECOR_34     .......... .......... .......... .......... .......... .......... ..........
ECOR_71     .......... .......... .......... .......... .......... .......... ..........
SE11        .......... .......... .......... .......... .......... .......... ..........
O157_Sakai  .......... .......... .......... .......... .......... .......... ..........
ECOR_35     .......... .......... .......... ...T...... .....T.... .......... ..........
ECOR_36     .......... .......... .......... ...T...... .....T.... .......... ..........
SMS-3-5     .......... .......... .......... ...T...... .......... .......... ..........
536         .......... .......... .......... ...T...... .......... .......... ..........
APEC_O1     .......... .......... .......... ...T...... .......... .......... ..........
CFT073      .......... .......... .......... ...T...... .......... .......... ..........
E2348/69    .......... .......... .......... ...T...... .......... .......... ..........
ECOR_51     .......... .......... .......... ...T...... .......... .......... ..........
ECOR_57     .......... .......... .......... ...T...... .......... .......... ..........
UTI89       .......... .......... .......... ...T...... .......... .......... ..........
E._albertii .......... .......... .C..T..... ...T..T..A ..T.....C. .C........ ......A...

          3641
K-12        AATATGTCAT CTCTGGCGGA AAAGTTTTCC ACTGACAACG CGGGCATTGC CTACTTAATT TCCGGTATCG
53638       .......... .......... .......... .......... .......... .......... ..........
ATCC_8739   .......... .......... .......... .......... .......... .......... ..........
ECOR_10     .......... .......... .......... .......... .......... .......... ..........
ECOR_11     .......... .......... .......... .......... .......... .......... ..........
B171        .......... .......A.. .......... .......... .......... .......... ..........
E24377A     ..C....... .......... .......... .......... .......... .......... ........T.
ECOR_34     ..C....... .......... .......... .......... .......... ...T...... ..........
ECOR_71     ..C....... .......... .......... .......... .......... .......... ........T.
SE11        ..C....... .......... .......... .......... .......... .......... ........T.
O157_Sakai  .......... .......... .......... .......... .......... .......... ..........
ECOR_35     ..C...A... ..T....A.. .......... .......... .......... ...T...... ..........
ECOR_36     ..C...A... ..T....A.. .......... .......... .......... ...T...... ..........
SMS-3-5     ..C...A... .......A.. .......... .......... .......... ...T...... ..........
536         ..C...A... ..T....A.. .......... .......... .......... ...T...... ..........
APEC_O1     ..C...A... ..T....A.. .......... .......... .......... ...T...... ..........
CFT073      ..C...A... ..T....A.. .......... .......... .......... ...T...... ..........
E2348/69    ..C...A... ..T....A.. .......... .......... .......... ...T...... ..........
ECOR_51     ..C...A... ..T....A.. .......... .......... .......... ...T...... ..........
ECOR_57     ..C...A... ..T....A.. .......... .......... .......... ...T...... ..........
UTI89       ..C...A... ..T....A.. .......... .......... .......... ...T...... ..........
E._albertii ..C....... .......T.. G........T ..C.....T. .T........ ...T...... .....C..T.

          3711
K-12        GTTTGGGGCG ATTGATCAGT ATTTTATTCT TCGGTGTGAT CTCCGATAAG TTTGGTCGTC GGGCGGTGAT
53638       .......... .......... .......... .......... .......... .......... ..........
ATCC_8739   .......... .......... .......... .......... .......... .......... ..........
ECOR_10     .......... .......... .......... .......... .......... .......... ..........
ECOR_11     .......... .......... .......... .......... .......... .......... ..........
B171        .......... .......... .......... .......... .......... .......... ..........
E24377A     .......... .......... .......... .......... .......... .......... ..........
ECOR_34     .......... .......... .......... .......... .......... .......... ..........
ECOR_71     .......... .......... .......... .......... .......... .......... ..........
SE11        .......... .......... .......... .......... .......... .......... ..........
O157_Sakai  .......... .......... .......... .......... .......... .......... ..........
ECOR_35     .A........ .......... .......... ....A..... .......... .......... ..........
ECOR_36     .A........ .......... .......... ....A..... .......... .......... ..........
SMS-3-5     .......... .......... .......... .......... .......... .......... ..........
536         .A........ .......... .......... ....A..... .......... .......... ..........
APEC_O1     .A........ .......... .......... ....A..... .......... .......... ..........
CFT073      .A........ .......... .......... ....A..... .......... .......... ..........
E2348/69    .A........ .......... .......... ....A..... .......... .......... ..........
ECOR_51     .A........ .......... .......... ....A..... .......... .......... ..........
ECOR_57     .A........ .......... .......... ....A..... .......... .......... ..........
UTI89       .A........ .......... .......... ....A..... .......... .......... ..........
E._albertii ..C....T.. CC....A..C .......... ....C..... A........A ........G. ..A....A..
```

```
             3781
K-12          ATTAATGGCA GTAATAATGT ATCTGCTATT CTTCTTTGGT ATTCCCGCTT GCCCGAATTT AACTCTCGCC
53638         .......... .......... .......... .......... .......... .......... ..........
ATCC_8739     .......... .......... .......... .......... .......... .......... ..........
ECOR_10       .......... .......... .......... .......... .......... .......... ..........
ECOR_11       .......... .......... .......... .......... .......... .......... ..........
B171          .......... .......... .......... .......... .......... .......... ..........
E24377A       .......... .......... .......... .......... .......... .......... ..........
ECOR_34       .......... .......... .......... .......... .......... .......... ..........
ECOR_71       .......... .......... .......... .......... .......... .......... ..........
SE11          .......... .......... .......... .......... .......... .......... ..........
O157_Sakai    .......... .......... .......... .......... .......... .......... ..........
ECOR_35       .......... .......... .......... .......... .....T.... .......... ..........
ECOR_36       .......... .......... .......... .......... .....T.... .......... ..........
SMS-3-5       .......... .......... .......... .......... .....T.... .......... ..........
536           .......... .......... .......... .......... .....T.... .......... ..........
APEC_O1       .......... .......... .......... .......... .....T.... .......... ..........
CFT073        .......... .......... .......... .......... .....T.... .......... ..........
E2348/69      .......... .......... .......... .......... .....T.... .......... ..........
ECOR_51       .......... .......... .......... .......... .....T.... .......... ..........
ECOR_57       .......... .......... .......... .......... .....T.... .......... ..........
UTI89         .......... .......... .......... .......... .....T.... .......... ..........
E._albertii   .........G ..GG.C.... ..A....G.. T......... ........C. .T..A..... .........T

             3851
K-12          TACGGTCTGG CAGTGTGCGT AGGTATCGCT AACTCAGCGC TGGATACGGG TGGCTACCCC GCGCTCATGG
53638         .......... .......... .......... .......... .......... .......... ..........
ATCC_8739     .......... .......... .......... .......... .......... .......... ..........
ECOR_10       .......... .......... .......... .......... .......... .......... ..........
ECOR_11       .......... .......... .......... .......... .......... .......... ..........
B171          .......... .......... .......... .......... .......... .......... ..........
E24377A       .......... .......... .......... .......... .......... .......... ..........
ECOR_34       .......... .......... .......... .......... .......... .......... ..........
ECOR_71       .......... .......... .......... .......... .......... .......... ..........
SE11          .......... .......... .......... .......... .......... .......... ..........
O157_Sakai    .......... .......... .......... .......... .......... .......... ..........
ECOR_35       ...T...... .......... .......... .......... .......... .......... .....T....
ECOR_36       ...T...... .......... .......... .......... .......... .......... .....T....
SMS-3-5       ...T...... .......... .......... .......... .......... .......... ..........
536           ...T...... .......... .......... .......... .......... .......... .....T....
APEC_O1       ...T...... .......... .......... .......... .......... .......... ..........
CFT073        ...T...... .......... .......... .......... .......... .......... ..........
E2348/69      ...T...... .......... .......... .......... .......... .......... ..........
ECOR_51       ...T...... .......... .......... .......... .......... .......... ..........
ECOR_57       ...T...... .......... .......... .......... .......... .......... ..........
UTI89         ...T...... .......... .......... .......... .......... .......... ..........
E._albertii   ...T.C.... .G........ C........C ........T. .T.....T.. C..T.....G ..T.......

             3921
K-12          AATGCTTTCC GAAAGCCTCT GGTTCGGCGG TCATACTGGT TAAAGCGATG GTGTCATTTG GGCAAATGTT
53638         .......... .......... .......... .......... .......... ........C. ..........
ATCC_8739     .......... .......... .......... .......... .......... .......... ..........
ECOR_10       .......... .......... .......... .......... .......... .......... ..........
ECOR_11       .......... .......... .......... .......... .......... .......... ..........
B171          .......... .......... .......... .......... .......... .......... ..........
E24377A       .......... .......... .......... .......... .......... .......... ..........
ECOR_34       .......... .......... .......... .......... .......... ........C. .A........
ECOR_71       .......... .......... .......... .......... .......... ........C. .A........
SE11          .......... .......... .......... .......... .......... .......... ..........
O157_Sakai    .......... .......... .......... .......... .......... .......... ..........
ECOR_35       .......... .......... .......... .......... .......... ........C. ..........
ECOR_36       .......... .......... .......... .......... .......... ........C. ..........
SMS-3-5       .......... .......... .......... .......... .......... ........C. ..........
536           .......... .......... .......... .......... .......... ........C. ..........
APEC_O1       .......... .......... .......... .......... .......... ........C. ..........
CFT073        .......... .......... .......... .......... .......... ........C. ..........
E2348/69      .......... .......... .......... .......... .......... .......... ..........
ECOR_51       .......... .......... .......... .......... .......... ........C. ..........
ECOR_57       .......... .......... .......... .......... .......... ........C. ..........
UTI89         .......... .......... .......... .......... .......... ........C. ..........
E._albertii   .G..T..C.. .........C .......... .A..T..... ......A... A.C.....C. .T......C.
```

```
            3991
K-12         CTACCCAATG CTGGTGAGCT ATATGTTGCT CAATAATATC TGGTACGGCT ATGGGCTGAT TATTCCGGGT
53638        .......... .......... .......... .......... .......... .......... ..........
ATCC_8739    .......... .......... .......... .......... .......... .......... ..........
ECOR_10      .......... .......... .......... .......... .......... .......... ..........
ECOR_11      .......... .......... .......... .......... .......... .......... ..........
B171         .......... .......... .......... .......... .......... .......... ..........
E24377A      .......... .......... .......... .......... .......... .......... ..........
ECOR_34      .......... .......... .......... ......C... .......... .......... ......A..C
ECOR_71      .......... .......... .......... ......C... .......... .......... ......A..C
SE11         .......... .......... .......... .......... .......... .......... ..........
O157_Sakai   .......... .......... .......... .......... .......... .......... ..........
ECOR_35      .......... .......... .......... ......C... .......... .......... ..........
ECOR_36      .......... .......... .......... ......C... .......... .......... ..........
SMS-3-5      .......... .......... .......... ......C... .......... .......... ..........
536          .......... .......... .......... ......C... .......... .......... ..........
APEC_O1      .......... .......... .......... ......C... .......... .......... ..........
CFT073       .......... .......... .......... ......C... .......... .......... ..........
E2348/69     .......... .......... .......... ......C... .......... .......... ..........
ECOR_51      .......... .......... .......... ......C... .......... .......... ..........
ECOR_57      .......... .......... .......... ......C... .......... .......... ..........
UTI89        .......... .......... .......... ......C... .......... .......... ..........
E._albertii  ...T..G... .......... .....C..T. A......... .......... .C.....A.. C.....A..C

            4061
K-12         ATTCTATTTG TACTGATCAC GCTGATGCTG TTGAAAAGCA AATTCCCCAG CCAGTTGGTG GACGCCAGCG
53638        .....G.... .......... .......... .......... .......... .......... ..........
ATCC_8739    .......... .......... .......... .......... .......... .......... ..........
ECOR_10      .......... .......... .......... .......... .......... .......... ..........
ECOR_11      .......... .......... .......... .......... .......... .......... ..........
B171         .....G.... .......... .......... .......... .......... .......... ..........
E24377A      .....G.... .......... .......... .......... .......... .......... ..........
ECOR_34      ..C....... .......... .......... .......... .......... .......... ..........
ECOR_71      ..C....... .......... .......... .......... .......... .......... ..........
SE11         .....G.... .......... .......... .......... .......... .......... ..........
O157_Sakai   .....G.... .......... .......... .......... .......... .......... ..........
ECOR_35      .....G.... .......... .......... .......... .......... .......... ..........
ECOR_36      .....G.... .......... .......... .......... .......... .......... ..........
SMS-3-5      .....G.... .......... .......... .......... .......... .......... ..........
536          .....G.... .......... .......... .......... .......... .......... ..........
APEC_O1      .....G.... .......... .......... .......... .......... .......... ..........
CFT073       .....G.... .......... .......... .......... .......... .......... ..........
E2348/69     .....G.... .......... .......... .......... .......... .......... ..........
ECOR_51      .....G.... .......... .......... .......... .......... .......... ..........
ECOR_57      .....G.... .......... .......... .......... .......... .......... ..........
UTI89        .....G.... .......... .......... .......... .......... .......... ..........
E._albertii  ...T...... .C.....T.. .........A .......... .......... T......... ..T.......

            4131
K-12         TAACTAATGA ATTACCGCAA ATGAACAGCA AACCGTTAGT CTGGCTGGAA GGTGTTTCAT CGGTACTGTT
53638        ..G....... .......... .......... .......... .......... .......... ..........
ATCC_8739    .......... .......... .......... .......... .......... .......... ..........
ECOR_10      .......... .......... .......... .......... .......... .......... ..........
ECOR_11      .......... .......... .......... .......... .......... .......... ..........
B171         ..G....... .......... .......... .......... .......... .......... ..........
E24377A      ..G....... .......... .......... .......... .......... .......... ..........
ECOR_34      ..G....... .......... .......... .......... .......... .......... ..........
ECOR_71      ..G....... .......... .......... .......... .......... .......... ..........
SE11         ..G....... .......... .......... .......... .......... .......... ..........
O157_Sakai   ..G....... .......... .......... .......... .......... .......... ..........
ECOR_35      ..G....... .......... .......... .......... .......... .......... .A........
ECOR_36      ..G....... .......... .......... .......... .......... .......... .A........
SMS-3-5      ..G....... .......... .......... .......... .......... .......... .A........
536          ..G....... .......... .......... .......... .......... .......... .A........
APEC_O1      ..G....... .......... .......... .......... .......... .......... .A........
CFT073       ..G....... .......... .......... .......... .......... .......... .A........
E2348/69     ..G....... .......... .......... .......... .......... .......... .A........
ECOR_51      ..G....... .......... .......... .......... .......... .......... .A........
ECOR_57      ..G....... .......... .......... .......... .......... .......... .A........
UTI89        ..G....... .......... .......... .......... .......... .......... .A........
E._albertii  .TG.C..... ...G...... ........T. .....C.... .........G ..G....... .T........
```

```
             4201
K-12         CGGTGTAGCC GCATTCTCGA CCTTTTATGT GATTGTGGTG TGGATGCCCA AATATGCGAT GGCTTTTGCT
53638        .........T .......... .......... .......... .......... .......... ..........
ATCC_8739    .......... .......... .......... .......... .......... .......... ..........
ECOR_10      .......... .......... .......... .......... .......... .......... ..........
ECOR_11      .......... .......... .......... .......... .......... .......... ..........
B171         .......... .......... .......... .......... .......... .......... ..........
E24377A      .......... .......... .......... .......... .......... .......... ..........
ECOR_34      .......... .......... .......... .......... .......... .......... ..........
ECOR_71      .......... .......... .......... .......... .......... .......... ..........
SE11         .......... .......... .......... .......... .......... .......... ..........
O157_Sakai   .......... .......... .......... .......... .......... .......... ..........
ECOR_35      .......... .......... .......... .......... .......... .......... ..........
ECOR_36      .......... .......... .......... .......... .......... .......... ..........
SMS-3-5      .......... .......... .......... .......... .......... .......... ..........
536          .......... .......... .......... .......... .......... .......... ..........
APEC_O1      .......... .......... .......... .......... .......... .......... ..........
CFT073       .......... .......... .......... .......... .......... .......... ..........
E2348/69     .......... .......... .......... .......... .......... .......... ..........
ECOR_51      .......... .......... .......... .......... .......... .......... ..........
ECOR_57      .......... .......... .......... .......... .......... .......... ..........
UTI89        .......... .......... .......... .......... .......... .......... ..........
E._albertii  ...C..T... ........A. .......... ...A...... ........A. .......... ...G.....C

             4271
K-12         GGTATGTCAG AAGCTGAGGC ATTAAAAACC ATCTCTTATT ACAGTATGGG CTCGTTGGTC TGTGTCTTTA
53638        .......... .......... .......... .......... .......... .......... ..........
ATCC_8739    .......... .......... .......... .......... .......... .......... ..........
ECOR_10      .......... .......... .......... .......... .......... .......... ..........
ECOR_11      .......... .......... .......... .......... .......... .......... ..........
B171         .......... .......... .......... .......... .......... .......... ..........
E24377A      .......... .......... .......... .......... .......... .......... ..........
ECOR_34      .......... .......... .......... .......... .......... .......... ..........
ECOR_71      .......... .......... .......... .......... .......... .......... ..........
SE11         .......... .......... .......... .......... .......... .......... ..........
O157_Sakai   .......... .......... .......... .......... .......... ....C..... ..........
ECOR_35      .......... .......... .......... .......... .......... T...C..... ..........
ECOR_36      .......... .......... .......... .......... .......... T...C..... ..........
SMS-3-5      .......... .......... .......... .......... .......... T...C..... ..........
536          .......... .......... .......... .......... .......... T...C..... ..........
APEC_O1      .......... .......... .......... .......... .......... T...C..... ..........
CFT073       .......... .......... .......... .......... .......... T...C..... ..........
E2348/69     .......... .......... .......... .......... .......... T...C..... ..........
ECOR_51      .......... .......... .......... .......... .......... T...C..... ..........
ECOR_57      .......... .......... .......... .......... .......... T...C..... ..........
UTI89        .......... .......... .......... .......... .......... T...C..... ..........
E._albertii  ........C. ....C..... T......... ..T....... .......... T..AC..... ..C.......

             4341
K-12         TTTTTGCCGC ACTACTGAAA AAAATGGTCC GGCCCATCTG GGCTAATGTA TTTAACTCTG CACTGGCAAC
53638        .......... .......... .......... .......... .......... .......... ..........
ATCC_8739    .......... .......... .......... .......... .......... .......... ..........
ECOR_10      .......... .......... .......... .......... .......... .......... ..........
ECOR_11      .......... .......... .......... .......... .......... .......... ..........
B171         .......... .......... .......... .......... .......... .......... ..........
E24377A      .......... .......... .......... .......... .......... .......... ..........
ECOR_34      .......... .......... .......... .......... .......... .........A ..........
ECOR_71      .......... .......... .......... .......... .......... .......... ..........
SE11         .......... .......... .......... .......... .......... .......... ..........
O157_Sakai   .......... .......... .......... .......... .......... .......... ..........
ECOR_35      .......... C..G...... .......... .......... .......... .......... ..........
ECOR_36      .......... C..G...... .......... .......... .......... .......... ..........
SMS-3-5      .......... ...G...... .......... .......... ........C. .......... ..........
536          .......... .......... .......... .T........ .......... .......... ..........
APEC_O1      .......... ...G...... .......... .......... .......... .......... ..........
CFT073       .......... .......... .......... .T........ .......... .......... ..........
E2348/69     .......... ...G...... .......... .......... .......... .......... ..........
ECOR_51      .......... .......... .......... .T........ .......... .......... ..........
ECOR_57      .......... .......... .......... .T........ .......... .......... ..........
UTI89        .......... ...G...... .......... .......... .......... .......... ..........
E._albertii  ....C..... .T........ .......... ....T..... .........T .......... .G........
```

```
          4411
K-12         AATAACAGCA GCCATTATCT ACCTGTACCC TTCTCCACTG GTGTGCAATG CCGGAGCCTT TGTTATCGGT
53638        .......... .......... .......... .......... .....T.... .......... ..........
ATCC_8739    .......... .......... .......... .......... .....T.... ....C..... ..........
ECOR_10      .......... .......... .......... .......... .......... .......... ..........
ECOR_11      .......... .......... .......... .......... .......... .......... ..........
B171         .......... .......... .......... .......... .....T.... ....C..... ..........
E24377A      .......... .......... .......... .......... .....T.... .T..C..... ..........
ECOR_34      .......... .......... .......... .......... .....T.... ....C..... ..........
ECOR_71      .......... .......... .......... .......... .....T.... .......... ..........
SE11         .......... .......... .......... .......... .....T.... ....C..... ..........
O157_Sakai   .......... .......... .......... .......... .....T.... ....C..... ..........
ECOR_35      .........T .......... .......... .......T.. .....T.... .......... ..........
ECOR_36      .........T .......... .......... .......T.. .....T.... .......... ..........
SMS-3-5      .........T .......... .......... .......T.. .....T.... .......... ..........
536          .........T .......... .......... .......T.. .....T.... .......... ..........
APEC_O1      .........T .......... .......... A......T.. .....T.... .......... ..........
CFT073       .........T .......... .......... .......T.. .....T.... .......... ..........
E2348/69     .........T .......... .......... .......T.. .....T.... .......... ..........
ECOR_51      .........T .......... .......... .......T.. .....T.... .......... ..........
ECOR_57      .........T .......... .......... .......T.. .....T.... .......... ..........
UTI89        .........T .......... .......... A......T.. .....T.... .......... ..........
E._albertii  GG.......C .....C..T. .T.....T.. ......G... ..C..T.... .T........ ..........

          4481
K-12         TTCTCAGCAG CTGGCGGCAT TTTACAGCTC GGCGTTTCGG TCATGTCAGA GTTTTTTCCC AAAAGCAAAG
53638        .......... .......... ......A... ..T....... .......... ......C..A ..........
ATCC_8739    .......... .......... .......... ..T....... .......... ......C..A ..........
ECOR_10      .......... .......... .......... .......... .......... .......... ..........
ECOR_11      .......... .......... .......... .......... .......... .......... ..........
B171         .......... .......... .......... ..T....... .......... ......C..A ..........
E24377A      .......... ....T..... .......... .......... .......... ......C... ..........
ECOR_34      .......... .......... .......... .......... .......... ......C... ..........
ECOR_71      .......... ....T..... .......... .......... .......... ......C... ..........
SE11         .......... .......... .......... ..T....... .......... ......C..A ..........
O157_Sakai   .......... .......... .......... ..T....... .......... ......C..A ..........
ECOR_35      .......... .......... .......... .......... .......... ......C..A ..........
ECOR_36      .......... .......... .......... .......... .......... ......C..A ..........
SMS-3-5      .......... .......... .......... .......... .......... ......C..A ..........
536          .......... .......... .......... .......... .......... ......C... ..........
APEC_O1      .......... .......... .......... .......... .......... ......C..A ..........
CFT073       .......... .......... .......... .......... .......... ......C... ..........
E2348/69     .......... .......... .......... .......... .......... ......C..A ..........
ECOR_51      .......... .......... .......... .......... .......... ......C... ..........
ECOR_57      .......... .......... .......... .......... .......... ......C... ..........
UTI89        .......... .......... .......... .......... .......... ......C..A ..........
E._albertii  .....T..T. .......... ......A..T .....G.... .......G.. ......C... ..........

          4551
K-12         CCAAAGTCAC CAGTATTTAT ATGATGATGG GTGGACTGGC TAACTTTGTT ATTCCACTGA TTACCGGTTA
53638        .......... .......... .......... .......... .......... .....G.... ..........
ATCC_8739    .......... .......... .......... .......... .......... .....G.... ..........
ECOR_10      .......... .......... .......... .......... .......... .......... ..........
ECOR_11      .......... .......... .......... .......... .......... .......... ..........
B171         .......... .......... .......... .......... .......... .....G.... ..........
E24377A      .......... .......... .......... .C...T.... ...T...... .....G.... ..........
ECOR_34      .......... ......A... .......... .......... .......... .....G.... ..........
ECOR_71      .......... .......... .......... .....T.... ...T...... .....G.... ..........
SE11         .......... .......... .......... .......... .......... .....G.... ..........
O157_Sakai   .......... .......... .......... .......... .......... .....G.... ..........
ECOR_35      .......... ......A... .......... .......... .......... .....G.... ..........
ECOR_36      .......... ......A... .......... .......... .......... .....G.... ..........
SMS-3-5      .......... ......A... .......... .......... .......... .....G.... ..........
536          .......... ......A... .......... .......... .......... .....G.... ..........
APEC_O1      .......... ......A... .......... .......... .......... .....G.... ..........
CFT073       .......... ......A... .......... .......... .......... .....G.... ..........
E2348/69     .......... ......A... .......... .......... .......A.. .....G.... ..........
ECOR_51      .......... ......A... .......... .......... .......... .....G.... ..........
ECOR_57      .......... ......A... .......... .......... .......... .....G.... ..........
UTI89        .......... ......A... .......... .......... .......... .....G.... ..........
E._albertii  .......... .......... .......... ....G..... A......... .....G.... .......A..
```

```
             4621
K-12         TCTGTCGAAC ATCGGCCTGC AATATATCAT TGTTCTCGAT TTTACTTTCG CGCTGCTGGC CCTGATTACC
53638        .......... .......... .......... .......... .......... .......... ..........
ATCC_8739    .......... .......... .......... .......... .......... .......... ..........
ECOR_10      .......... .......... .......... .......... .......... .......... ..........
ECOR_11      .......... .......... .......... .......... .......... .......... ..........
B171         .......... ........T. .......... .......... .......... .......... ..........
E24377A      .......... .....T.... .......... .A........ .......... .......... ..........
ECOR_34      .......... .......... .......... .......... .......... .......... ..........
ECOR_71      .......... .......... .......... .......... .......... .......... ..........
SE11         .......... ........T. .......... .......... .......... .......... ..........
O157_Sakai   .......... .......... .......... .......... .......... .......... ..........
ECOR_35      .......... .......... .......... .......... .......... .......... ..........
ECOR_36      .......... .......... .......... .......... .......... .......... ..........
SMS-3-5      .......... .......... .......... .......... .......... .......... ..........
536          .......... .......... .......... .......... .......... .......... ..........
APEC_O1      .......... .......... .......... .......... ....A..... .......... ..........
CFT073       .......... .......... .......... .......... .......... .......... ..........
E2348/69     .......... .......... .......... .......... .......... .......... ..........
ECOR_51      .......... .......... .......... .......... .......... .......... ..........
ECOR_57      .......... .......... .......... .......... .......... .......... ..........
UTI89        .......... .......... .......... .......... .......... .......... ..........
E._albertii  ...T.....T .......... .......... ......T... ....T..... ..T.AT.A.. T.........

             4691
K-12         GCAATTATTG TTTTTATCCG CTATTACCGC GTTTTCATTA TTCCTGAAAA TGATGTGCGG TTTGGCGAGC
53638        .......... .......... .......... .....T.... .......G.. .......... ..........
ATCC_8739    .......... .......... .......... .....T.... .......G.. .......... ..........
ECOR_10      .......... .......... .......... .......... .......... .......... ..........
ECOR_11      .......... .......... .......... .......... .......... .......... ..........
B171         .......... .......... .......... .....T.... .......G.. .......... ..........
E24377A      .......... .C........ .......... .....T.... .......G.. .......... ..........
ECOR_34      .......... .......... .......... .......... .......... .......... ..........
ECOR_71      .......... .C........ .......... .......... .......G.. .........A ..........
SE11         .......... .......... .......... .....T.... .......G.. .......... ..........
O157_Sakai   .......... .......... .......... .....T.... .....A.G.. .......... ..........
ECOR_35      .......... .......... .......... .......... .......... .......... ..........
ECOR_36      .......... .......... .......... .......... .....A.G.. .......... ..........
SMS-3-5      .......... .......... .......... .......... .......... .......... ..........
536          .......... .......... .......... .......... .......... .......... ..........
APEC_O1      .......... .......... .......... .......... .......... .......... ..........
CFT073       .......... .......... .......... .......... .......... .......... ..........
E2348/69     .......... .......... .......... .......... .......... .......... ..........
ECOR_51      .......... .......... .......... .......... .......... .......... ..........
ECOR_57      .......... .......... .......... .......... .......... .......... ..........
UTI89        .......... .......... .......... .......... .......... .......... ..........
E._albertii  ..G....... ....C..T.. .........T ..C..T.... .......... A...T.A... ..A.....A.

             4761
K-12         GTAAATTTTG CACC-CGGTT AAACACAATT AAGCATAGAG GTTAAAGGAG TTAATTATGG ATGTTACCGC
53638        .C......A. .......... .......... .......... .......... .......... ..........
ATCC_8739    .C......A. .......... .......... .......... .......... .......... ..........
ECOR_10      .......... .......... .......... .......... .......... .......... ..........
ECOR_11      .......... .......... .......... .......... .......... .......... ..........
B171         ........A. .......... .......... .......... .......... .......... ..........
E24377A      ........A. .......... .......... .......... .......... .......... ..........
ECOR_34      ........A. .......... .......... .......... .......... .......... ..........
ECOR_71      ........A. ........C. .......... .......... .......... .......... ..........
SE11         ........A. .......... .......... .......... .......... .......... ..........
O157_Sakai   .C......A. .......... .......... .......... .......... .......... ..........
ECOR_35      A.......A. .......... ......G... ........G. .......... .......... ..........
ECOR_36      A.......A. .......... ......G... ........G. .......... .......... ..........
SMS-3-5      A.......A. .......... .......... .......... .......... .........A ..........
536          A.......A. .......... .......... .......... .......... .......... ..........
APEC_O1      A.......A. .......... .......... .......... .......... .......... ..........
CFT073       A.......A. .......... ......G... .......... .......... .......... ..........
E2348/69     A.......A. ......A... .......... .......... .......... .......... ..........
ECOR_51      A.......A. .......... ......G... .......... .......... .......... ..........
ECOR_57      A.......A. .......... ......G... .......... .......... .......... ..........
UTI89        A.......A. .......... .......... .......... .......... .......... ..........
E._albertii  ACT.T..CC. ....T.A... ..G...C... ...A...A.. .......... .......... .......G..
```

```
         4831
K-12        AAAATACGAA TTGATTGGGT TGATGGCCTA TCCTATCCGC CACAGTTTAT CGCCCGAAAT GCAGAATAAA
53638       .......... .......... .......T.. .......... .......... ....T..... ..........
ATCC_8739   .......... .......... .......T.. .......... .......... ....T..... ..........
ECOR_10     .......... .......... .......... .......... .......... .......... ..........
ECOR_11     .......... .......... .......... .......... .......... .......... ..........
B171        .......... .........C .......... .......... .......... ....T..... ..........
E24377A     .......... .........C .......... .......... .......... ....TA.... ..........
ECOR_34     .......... .........C .......... .......... .......... ....T..... ..........
ECOR_71     .......... .......... .......T.. .......... .......... ....T..... ..........
SE11        .......... .........C .......... .......... .......... ....T..... ..........
O157_Sakai  .......... .......... .......T.. .......... .......... ....T..... ..........
ECOR_35     .......... .......... .......... ...C...... .......... .......... ..........
ECOR_36     .......... .......... .......... ...C...... .......... .......... ..........
SMS-3-5     .......... .......... .......... ...C...... .......... .......... ..........
536         .......... .......... .......... ...C...... .......... .......... ..........
APEC_O1     .......... .......... .......... ...C...... .......... .......... ..........
CFT073      .......... .......... .......... ...C...... .......... .......... ..........
E2348/69    .......... .......... .......... ...C...... .......... .......... ..........
ECOR_51     .......... .......... .......... ...C...... .......... .......... ..........
ECOR_57     .......... .......... .......... ...C...... .......... .......... ..........
UTI89       .......... .......... .......... ...C...... .......... .......... ..........
E._albertii T.....T... ........CC .......T.. .......... .......... ....T..... ...A......

         4901
K-12        GCCTTAGAAA AAGCGGGATT GCCATTTACC TATATGGCCT TCGAAGTGGA TAACGATAGC TTTCCTGGAG
53638       .......... .......... .......... .......... .......... .......... .....C....
ATCC_8739   .......... .......... .......... .......... .......... .......... .....C....
ECOR_10     .......... .......... .......... .......... .......... .......... ..........
ECOR_11     .......... .......... .......... .......... .......... .......... ..........
B171        .......... .......... .......... .......... .T........ .........T .....C....
E24377A     .......... .......... .......... .......... .T........ .........T .....C....
ECOR_34     .......... .......... .......... .......... .T........ .........T .....C....
ECOR_71     .......... .......... .......... .......... .......... .......... .....C....
SE11        .......... .......... .......... .......... .T........ .........T .....C....
O157_Sakai  .......... .......... .......... .......... .......... .......... .....C....
ECOR_35     .......... .......... .......... .......... .T........ .........T .....C....
ECOR_36     .......... .......... .......... .......... .T........ .........T .....C....
SMS-3-5     .......... .......... .......... .......... .T........ .........T .....C.C..
536         .......... .......... .......... .......... .T........ .........T .....C.C..
APEC_O1     .......... .......... .......... .......... .T........ .........T .....C.C..
CFT073      .......... .......... .......... .......... .T........ .........T .....C....
E2348/69    .......... .......... .......... .......... .T........ .........T .....C.C..
ECOR_51     .......... .......... .......... .......... .T........ .........T .....C....
ECOR_57     .......... .......... .......... .......... .T........ .........T .....C....
UTI89       .......... .......... .......... .......... .T........ .........T .....C.C..
E._albertii ..G....... .......... A......... .......... .......... ......C... ...G.C..T.

         4971
K-12        CAATTGAAGG ATTAAAAGCC CTCAAAATGC GCGGAACTGG TGTATCGATG CCGAACAAAC AACTGGCGTG
53638       .......... .........T ..A....... .......... .A....A... .......... ..........
ATCC_8739   .......... .........T ..A....... .......... ......A... .......... ..........
ECOR_10     .......... .......... .......... .......... .......... .......... ..........
ECOR_11     .......... .......... .......... .......... .......... .......... ..........
B171        .......... .......... .......... .......... .A....A... .......... ..........
E24377A     .......... .......... .......... .......... .A....A... .......... ..........
ECOR_34     .......... .......... .......... .......... .A....A... .......... ..........
ECOR_71     .......... .........T ..A....... .......... ......A... .......... ..........
SE11        .......... .......... .......... .......... .A....A... .......... ..........
O157_Sakai  .......... .........T ..A....... .......... ......A... .......... ..........
ECOR_35     .......... .......... .......... .......... ......A... .......... ..........
ECOR_36     .......... .......... .......... .......... ......A... .......... ..........
SMS-3-5     .......... .......... .......... .T........ ...G...... .......... ..........
536         .......... .......... .......... .......... ......A... .......... ..........
APEC_O1     .......... .......... .......... .......... ......A... .......... ..........
CFT073      .......... .......... .......... .......... ......A... .......... ..........
E2348/69    .......... .......... .......... .......... ......A... .......... ..........
ECOR_51     .......... .......... .......... .......... ......A... .......... ..........
ECOR_57     .......... .......... .......... .......... ......A... .......... ..........
UTI89       .......... .......... .......... .......... ......A... .......... ..........
E._albertii .C........ .C........ T.A....... .T.C...A.. C......... ..A..T.... .......T..
```

```
             5041
K-12         TGAATATGTT GATGAATTAA CACCAGCTGC CAAACTGGTG GGGGCCATCA ACACCATCGT TAATGATGAT
53638        .......... .......... .G.....A.. .......... ..T....... .......... ..........
ATCC_8739    .......... .......... .G.....A.. .......... ..T....... .......... ..........
ECOR_10      .......... .......... .......... .......... .......... .......... ..........
ECOR_11      .......... .......... .......... .......... .......... .......... ..........
B171         .......... .......... .G.....A.. .......... ..T....... .......... ..........
E24377A      .......... .......... .G.....A.. .......... ..T....... .......... ..........
ECOR_34      .......... .......... .G.....A.. .......... ..T....... .......... ..........
ECOR_71      .......... .......... .G.....A.. .......... ..T....... .......... ..........
SE11         .......... .......... .G.....A.. .......... ..T....... .......... ..........
O157_Sakai   .......... .......... .G.....A.. .......... ..T....... .......... ..........
ECOR_35      .......... .......... .G.....A.. .......... ..T....... .......... A.........
ECOR_36      .......... .......... .G.....A.. .......... ..T....... .......... A.........
SMS-3-5      .......... .......... .G.....G.. .......... ..T....... .......... ..........
536          .......... .......... .G.....A.. .......... ..T....... .......... A.........
APEC_O1      .......... .......... .G.....G.. .......... ..A....... .......... ..........
CFT073       .......... .......... .G.....A.. .......... ..T....... .......... A.........
E2348/69     .......... .......... .G.....A.. .......... ..T....... .......... A.........
ECOR_51      .......... .......... .G.....A.. .......... ..T....... .......... A.........
ECOR_57      .......... .......... .G.....A.. .......... ..T....... .......... A.........
UTI89        .......... .......... .G.....G.. .......... ..A....... .......... ..........
E._albertii  .......... .......... .G.....G.. A........A ..C..T.... .T........ C.........

             5111
K-12         GGCTATCTGC GTGGCTATAA CACCGACGGC ACGGGCCATA TTCGCGCCAT TAAAGAGAGC GGTTTTGATA
53638        .....C.... .C........ .......... .......... .......... .......... ..........
ATCC_8739    .....C.... .C........ .......... .......... ....A..... .......... ..........
ECOR_10      .......... .......... .......... .......... .......... .......... ..........
ECOR_11      .......... .......... .......... .......... .......... .......... ..........
B171         .....C.... .C........ .......... .......... .......... .......... ..........
E24377A      .....C.... .C........ .......... .......... .......... .......... ..........
ECOR_34      ..T..C.... .C........ .......... .......... ....A..... .......... ..........
ECOR_71      .....C.... .C........ .......... .......... .......... .......... ..........
SE11         .....C.... .C........ .......... .......... .......... .......... ..........
O157_Sakai   .....C.... .C........ .......... .......... .......... .......... ..........
ECOR_35      .......... .......... .......... .......... .......... .......... ..........
ECOR_36      .......... .......... .......... .......... .......... .......... ..........
SMS-3-5      .......... .......... .......... .....T.... .......... .......... ..........
536          .......... .......... .......... .......... .......... .......... ..........
APEC_O1      .......... .......... .......... .....T.... .......... .......... ..........
CFT073       .......... .......... .......... .......... .......... .......... ..........
E2348/69     .......... .......... .......... .......... .......... .......... ..........
ECOR_51      .......... .......... .......... .......... .......... .......... ..........
ECOR_57      .......... .......... .......... .......... .......... .......... ..........
UTI89        .......... .......... .......... .....T.... .......... .......... ..........
E._albertii  ..T..C.... .......... ......T..T ..A....... .......... .......... ..........

             5181
K-12         TCAAAGGCAA AACGATGGTG CTGTTAGGGG CCGGTGGTGC CTCAACGGCA ATTGGCGCGC AGGGGGCAAT
53638        .......... .......... .......... ....A..... .........G .......... ..........
ATCC_8739    .......... ...A...... .......... ....A..... .........G .......... ..........
ECOR_10      .......... .......... .......... .......... .......... .......... ..........
ECOR_11      .......... .......... .......... .......... .......... .......... ..........
B171         .......... ...A...... .......... ....A..... .........G .......... ..........
E24377A      .......... ...A...... .......... ....A..... .........G .......... ..........
ECOR_34      .......... ...A...... .......... ....A..... .........G .......... ..........
ECOR_71      .......... ...A...... .......... ....A..... .........G .......... ..........
SE11         .......... ...A...... .......... ....A..... .........G .......... ..........
O157_Sakai   .......... ...A...... .......... ....A..... .........G .......... ..........
ECOR_35      .......... .......... .......... ....A..... .......... .......... ..........
ECOR_36      .......... .......... .......... ....A..... .......... .......... ..........
SMS-3-5      .......... .......... ..A....... .......... .......... .......... ..........
536          .......... .......... .......... ....A..... .......... .......... ..........
APEC_O1      .......... .......... ..A....... .......... .......... .......... ..........
CFT073       .......... .......... .......... ....A..... .......... .......... ..........
E2348/69     .......... .......... .......... ....A..... .......... .......... ..........
ECOR_51      .......... .......... .......... ....A..... .......... .......... ..........
ECOR_57      .......... .......... .......... ....A..... .......... .......... ..........
UTI89        .......... .......... ..A....... .......... .......... .......... ..........
E._albertii  ...C...... .........C ........C. .T..C..C.. A.....A..G ........C. .......G..
```

```
             5251
K-12         TGAAGGTTTA AAAGAAATTA AACTCTTTAA CCGTCGGGAT GAGTTCTTCG ATAAAGCCCT CGCCTTCGCG
53638        .......... .......... .......... .......... .......... .......... ..........
ATCC_8739    .......... .......... .......... .......... .......... .......... ..........
ECOR_10      .......... .......... .......... .......... .......... .......... ..........
ECOR_11      .......... .......... .......... .......... .......... .......... ..........
B171         .......... .......... .......... .......... .......... .......... ..........
E24377A      .......... .......... .......... .......... .......... .......... ..........
ECOR_34      .......... .......... .......... .......... .......... .......... ..........
ECOR_71      .......... .......... .......... .......... .......... .......... ..........
SE11         .......... .......... .......... .......... .......... .......... ..........
O157_Sakai   .......... .......... .......... .......... A......... .......... ..........
ECOR_35      .......... .......... .......... .......... .......... .......... ..........
ECOR_36      .......... .......... .......... .......... .......... .......... ..........
SMS-3-5      .......... .......... .......... .......... .......... .......... ..........
536          .......... .......... .......... .......... .......... .......... ..........
APEC_O1      .......... .......... .......... .......... .......... .......T.. ..........
CFT073       .......... .......... .......... .......... .......... .......... ..........
E2348/69     .......... .......... .......... .......... .......... .......... ..........
ECOR_51      .......... .......... .......... .......... .......... .......... ..........
ECOR_57      .......... .......... .......... .......... .......... .......... ..........
UTI89        .......... .......... .......... .......... .......... .......T.. ..........
E._albertii  .......... .......... .......C.. ...CAAA... .....T..T. .......... .........T

             5321
K-12         CAGCGGGTTA ATGAAAACAC CGATTGTGTC GTCACGGTCA CCGATCTCGC CGATCAGCAA GCCTTTGCTG
53638        .......... .C........ .......... ........T. .......... .......... ..........
ATCC_8739    .......... .C........ .......... ........T. .......... .......... ..........
ECOR_10      .......... .......... .......... .......... .......... .......... ..........
ECOR_11      .......... .......... .......... .......... .......... .......... ..........
B171         .......... .C........ .......... ........T. .......... .......... ..........
E24377A      .......... .C........ .......... ........T. .......... .......... ..........
ECOR_34      .......... .C........ .......... ........T. .......... .......... ..........
ECOR_71      .......... .C........ .......... ........T. .......... ......A... ..........
SE11         .......... .C........ .......... ........T. .......... .......... ..........
O157_Sakai   .......... .C........ .......... ........T. .......... .......... ..........
ECOR_35      .......... .C........ ......C... ..G..A.... .......... .......... ..........
ECOR_36      .......... .C........ ......C... ..G..A.... .......... .......... ..........
SMS-3-5      .......... .C........ .......... ........T. .......... .......... ..........
536          .......... .C........ ......C... ..G..A.... .......... .......... ........A.
APEC_O1      .......... .C........ ......C... ..G..A.... .......... .......... ........A.
CFT073       .......... .C........ ......C... ..G..A.... .......... .......... ..........
E2348/69     .......... .C........ ......C... ..G..A.... .......... .......... ..........
ECOR_51      .......... .C........ ......C... ..G..A.... .......... .......... ..........
ECOR_57      .......... .C........ ......C... ..G..A.... .......... .......... ..........
UTI89        .......... .C........ ......C... ..G..A.... .......... .......... ........A.
E._albertii  ........C. .C........ .......... .......... .G........ .......... ..........

             5391
K-12         AAGCCCTGGC TTCCGCCGAC ATTTTAACCA ATGGCACAAA AGTGGGTATG AAACCCCTTG AGAATGAATC
53638        .......... .......... .......... .......... .......... .......... ..........
ATCC_8739    .......... .......... .......... .......... .......... .......... ..........
ECOR_10      .......... .......... .......... .......... .......... .......... ..........
ECOR_11      .......... .......... .......... .......... .......... .......... ..........
B171         .......... .......... .......... .......... .......... .......... ..........
E24377A      .......... .......... .......... .......... .......... .......... ..........
ECOR_34      .......... .......... .......... .......... .......... .......... ..........
ECOR_71      .......... .......... .......... .......... .......... .......... ..........
SE11         .......... .......... .......... .......... .......... .......... ..........
O157_Sakai   .......... .......... .......... .......... .......... .......... .....A....
ECOR_35      .......... .......... .......... .......... .......... .......... ..........
ECOR_36      .......... .......... .......... .......... .......... .......... ..........
SMS-3-5      .......... .......... .......... .......... .......... .......... ..........
536          .......... .......... .......... .......C.. .......... .......... ..........
APEC_O1      .......... .......... .......... .......C.. .......... .......... ..........
CFT073       .......... .......... .......... .......C.. .......... .......... ..........
E2348/69     .......... .......... .......... .......C.. .......... .......... ..........
ECOR_51      .......... .......... .......... .......C.. .......... .......... ..........
ECOR_57      .......... .......... .......... .......C.. .......... .......... ..........
UTI89        .......... .......... .......... .......C.. .......... .......... ..........
E._albertii  ....T..... .........T .......... .C.....C.. ......A... .....G.... .A........
```

```
           5461
K-12         ATTGGTTAAT GATATCAGTC TGTTACATCC GGGACTTCTG GTCACTGAAT GCGTGTATAA CCCGCATATG
53638        .......T.. .......... .......... ......G... .......... .......... ..........
ATCC_8739    .......... .......... .......... ......G... .......... .......... ..........
ECOR_10      .......... .......... .......... .......... .......... .......... ..........
ECOR_11      .......... .......... .......... .......... .......... .......... ..........
B171         ...A...... .......... .......... ......G... .......... .......... ..........
E24377A      ...A...... .......... .......... ......G... .......... .......... ..........
ECOR_34      ...A...... .......... .......... ......G... .......... .......... ..........
ECOR_71      ...A...... .......... .......... ......G... .......... .......... ..........
SE11         ...A...... .......... .......... ......G... .......... .......... ..........
O157_Sakai   .......... .......... .......... ......G... .......... .......... ..........
ECOR_35      .......... .......... .......... .......... .......... .......... ..........
ECOR_36      .......... .......... .......... .......... .......... .......... ..........
SMS-3-5      .......C.. .......... .......... .......... .......... .......... ..........
536          .......... .......... .......... .......... .......... .......... ..........
APEC_O1      .......... .......... .......... .......... .......... .......... ..........
CFT073       .......... .......... .......... .......... .......... .......... ..........
E2348/69     .......... .......... .......... .......... .......... .......... ..........
ECOR_51      .......... .......... .......... .......... .......... .......... ..........
ECOR_57      .......... .......... .......... .......... .......... .......... ..........
UTI89        .......... .......... .......... .......... .......... .......... ..........
E._albertii  ...AA.C... .......... .A........ C......T.. .....A.... .T..C..C.. T.........

           5531
K-12         ACGAAGTTAT TGCAGCAGGC GCAACAAGCT GGTTGCAAAA CGATTGATGG ATACGGCATG TTGTTGTGGC
53638        .......... .......... A......... .......... .......... .......... ..........
ATCC_8739    .......... .......... .......... .......... .......... .......... ..........
ECOR_10      .......... .......... .......... .......... .......... .......... ..........
ECOR_11      .......... .......... .......... .......... .......... .......... ..........
B171         .......... .......... .......... .......... .......... .......... ..........
E24377A      .......... .......... .......... .......... .......... .......... ..........
ECOR_34      .......... .......... .......... .......... .......... .......... ..........
ECOR_71      .......... .......... .......... .......... .......... .......... ..........
SE11         .......... .......... .......... .......... .......... .......... ..........
O157_Sakai   .......... .......... .......... .......... .......... .......... ..........
ECOR_35      .......... .......... .........G .......... .......... .......... ..........
ECOR_36      .......... .......... .........G .......... .......... .......... ..........
SMS-3-5      .......... .......... .........G .......... .......... .......... ..........
536          .......... .......... .........G .......... .......... .......... ..........
APEC_O1      .......... .......... .......... .......... .......... .......... ..........
CFT073       .......... .......... .........G .......... .......... .......... ..........
E2348/69     .......... .......... .........G .......... .......... .......... ..........
ECOR_51      .......... .......... .........G .......... .......... .......... ..........
ECOR_57      .......... .......... .........G .......... .......... .......... ..........
UTI89        .......... .......... .......... .......... .......... .......... ..........
E._albertii  .......... ....A..... .........C .......... .......... .......... ..........

           5601
K-12         AAGGGGCTGA ACAGTTCACA TTATGGACTG GCAAAGATTT CCCTCTGGAA TATGTTAAAC AGGTCATGGG
53638        .......... .........G .......... .......... .......... .......... ..........
ATCC_8739    .......... .........G .......... .......... .......... .......... ..........
ECOR_10      .......... .......... .......... .......... .......... .......... ..........
ECOR_11      .......... .......... .......... .......... .......... .......... ..........
B171         .......... .........G .......... .......... .......... .......... ..........
E24377A      .......... .........G .......... .......... .......... .......... ..........
ECOR_34      .......... .........G .......... .......... .......... .......... ..........
ECOR_71      .......... .........G .......... .......... .......... .......... ..........
SE11         .......... .........G .......... .......... .......... .......... ..........
O157_Sakai   .......... .........G .......... .......... .......... .......... ..........
ECOR_35      .......... .........G .......... .......... .......... .......... ..........
ECOR_36      .......... .........G .......... .......... .......... .......... ..........
SMS-3-5      .......... .........G .......... .......... .......... .......... ..........
536          .......... .........G .......... .......... .......... .......... ..........
APEC_O1      .......... .........G .......... .......... .......... .......... ..........
CFT073       .......... .........G .......... .......... .......... .......... ..........
E2348/69     .......... .........G .......... .......... .......... .......... ..........
ECOR_51      .......... .........G .......... .......... .......... .......... ..........
ECOR_57      .......... .........G .......... .......... .......... .......... ..........
UTI89        .......... .........G .......... .......... .......... .......... ..........
E._albertii  .......... ........AG .......... .T........ .......... ..C....... ..........
```

```
             5671
K-12         GTTCGGTGCC TGACAGGCTG ACCGCGTGCA GAAAGGGTAA AAAATGAAAA CCGTAACTGT AAAAGATCTC
53638        .......... .......... .......... .......... .......... .......... ..........
ATCC_8739    .......... .......... .......... .......... .......... .......... ..........
ECOR_10      .......... .......... .......... .......... .......... .......... ..........
ECOR_11      .......... .......... .......... .......... .......... .......... ..........
B171         .......... .......... .......... .......... .......... .......... ..........
E24377A      .......... .......... .......... .......... .......... .......... ..........
ECOR_34      .......... .......... .......... .......... .......... .......... ..........
ECOR_71      .......... .......... .......... .......... .......... .......... ..........
SE11         .......... .......... .......... .......... .......... .......... ..........
O157_Sakai   .......... .......... .......... .......... .......... .......... ..........
ECOR_35      .......... .......... .......... .......... .......... .......... ..........
ECOR_36      .......... .......... .......... .......... .......... .......... ..........
SMS-3-5      .......... .......... .......... .......... .......... .......... ..........
536          .......... .......... .......... .......... .......... .......... ..........
APEC_O1      .......... .......... .......... .......... .......... .......... ..........
CFT073       .......... .......... .......... .......... .......... .......... ..........
E2348/69     .......... .......... .......... .......... .......... .......... ..........
ECOR_51      .......... .......... .......... .......... .......... .......... ..........
ECOR_57      .......... .......... .......... .......... .......... .......... ..........
UTI89        .......... .......... .......... .......... .......... .......... ..........
E._albertii  ......C... .......... .......... .......... .......... .......C.. ..........

             5741
K-12         GTCATTGGTA CGGGCGCACC TAAAATCATC GTCTCGCTGA TGGCGAAAGA TATCGCCAGC GTGAAATCCG
53638        .........G .......... .......... .......... .......... .......... ..........
ATCC_8739    .........G .......... .......... .......... .......... .......C.. ..........
ECOR_10      .......... .......... .......... .......... .......... .......... ..........
ECOR_11      .......... .......... .......... .......... .......... .......... ..........
B171         .......... .......... .......... .......... .......... .......C.. ..........
E24377A      .......... .......... .......... .......... .......... .......C.. ..........
ECOR_34      .......... .......... .......... .......... .......... .......C.. ..........
ECOR_71      .......... .......... .......... .......... .......... .......C.. ..........
SE11         .......... .......... .......... .......... .......... .......C.. ..........
O157_Sakai   .......... .......... .......... .......... .......... .......C.A ..........
ECOR_35      .......... .......... .......... .......... .......... .......... ..........
ECOR_36      .......... .......... .......... .......... .......... .......... ..........
SMS-3-5      .......... .......... .......... .......... .......... .......... ..........
536          .......... .......... .......... .......... .......... .......... ..........
APEC_O1      .......... .......... .......... .......... .......... .......... ..........
CFT073       .......... .......... .......... .......... .......... .......... ..........
E2348/69     .......... .......... .......... .......... .......... .......... ..........
ECOR_51      .......... .......... .......... .......... .......... .......... ..........
ECOR_57      .......... .......... .......... .......... .......... .......... ..........
UTI89        .......... .......... .......... .......... .......... .......... ..........
E._albertii  ........CG .......... A..G..T... ..G....... .......... .......... ........A.

             5811
K-12         AAGCTCTCGC CTATCGTGAA GCGGACTTTG ATATTCTGGA ATGGCGTGTG GACCACTATG CCGACCTCTC
53638        .......... .......... .......... .......... .......... .......... ..........
ATCC_8739    .......... .......... .......... .......... .......... .......T.. ..........
ECOR_10      .......... .......... .......... .......... .......... .......... ..........
ECOR_11      .......... .......... .......... .......... .......... .......... ..........
B171         .......... .......... .......... .......... .......... .......T.. ..........
E24377A      .......... .......... .......... .......... .........A .......... ....T.....
ECOR_34      .......... .......... .......... .......... .......... .......T.. ..........
ECOR_71      .......... .......... .......... .......... .......... .......T.. ..........
SE11         .......... .......... .......... .......... .......... .......T.. ..........
O157_Sakai   .......... .......... .......... .......... .......... .......T.. ..........
ECOR_35      .......... .......... .......... .......... .......... .......... ..........
ECOR_36      .......... .......... .......... .......... .......... .......... ..........
SMS-3-5      .......... .......... .......... .......... .........T .......... ..........
536          .......... .......... .......... .......... .......... .......... ..........
APEC_O1      .......... .......... .......... .......... .......... .......... ....T.....
CFT073       .......... .......... .......... .......... .......... .......... ....T.....
E2348/69     .......... .......... .......... .......... .......... .......... ....T.....
ECOR_51      .......... .......... .......... .......... .......... .......... ....T.....
ECOR_57      .......... .......... .......... .......... .......... .......... ....T.....
UTI89        .......... .......... .......... .......... .......... .......... ....T.....
E._albertii  ...T...... ......C... .......... .......C.. ......C..T .......T.. ..........
```

```
            5881
K-12         CAATGTGGAG TCTGTCATGG CGGCAGCAAA AATTCTCCGT GAGACCATGC CAGAAAAACC GCTGCTGTTT
53638        .......... .......... .......... .......... ..A....... .......... ..........
ATCC_8739    .......... .......... ....G..... .......... ..A....... .......... ..........
ECOR_10      .......... .......... .......... .......... .......... .......... ..........
ECOR_11      .......... .......... .......... .......... .......... .......... ..........
B171         .......... .......... ....G..... .......... ..A....... .......... ..........
E24377A      .......... .......... .......... .......... ..A....... .......... ..........
ECOR_34      .......... .......... ....G..... .......... ..A....... .......... ..........
ECOR_71      .......... .......... ....G..... .......... ..A....... .......... ..........
SE11         .......... .......... ....G..... .......... ..A....... .......... ..........
O157_Sakai   .......... .......... ....G..... .......... ..A....... .......... ..........
ECOR_35      .......... ........A. .......... .......... ..A....... .......... ..........
ECOR_36      .......... ........A. .......... .......... ..A....... .......... ..........
SMS-3-5      .......... .......... .......... .......... ..A....... .......... ..........
536          .......... .......... .......... .......... ..A....... .......... ..........
APEC_O1      .......... ........A. .......C.. .......... ..A....... .......... ..........
CFT073       .......... ........A. .......... .......... ..A....... .......... ..........
E2348/69     .......... ........A. .......... .......... ..A....... .......... ..........
ECOR_51      .......... ........A. .......... .......... ..A....... .......... ..........
ECOR_57      .......... ........A. .......... .......... ..A....... .......... ..........
UTI89        .......... ........A. .......C.. .......... ..A....... .......... ..........
E._albertii  ..G.A..... .......... .A..G..C.. G..C...... ..AGT..... .G........ A.........

            5951
K-12         ACCTTCCGCA GTGCCAAAGA AGGCGGCGAG CAGGCGATTT CCACCGAGGC TTATATTGCA CTCAATCGTG
53638        .......... .......... .......... .......... .......... .......... ..........
ATCC_8739    .......... .......... .......... .......... .......... .........T ..........
ECOR_10      .......... .......... .......... .......... .......... .......... ..........
ECOR_11      .......... .......... .......... .......... .......... .......... ..........
B171         .......... .......... .......... .......... .......... ......C..T ..........
E24377A      .......... .......... ......T... .......... .......... .........T ..........
ECOR_34      .......... .......... .......... .......... .......... .........T ..........
ECOR_71      .......... .......... .......... .......... .......... .........T ..........
SE11         .......... .......... .......... .......... .......... ......C..T ..........
O157_Sakai   .......... .......... .......... .......... .......... .........T ..........
ECOR_35      .......... .......... .......... .......... .......... .......... ..........
ECOR_36      .......... .......... .......... .......... .......... .......... ..........
SMS-3-5      .......... .......... .......... .......... .......... .......... ..........
536          ....A..... .......... .......... .......... .......... .......... ..........
APEC_O1      .......... .......... .......... .......... .......... .......... ..........
CFT073       .......... .......... .......... .......... .......... .......... ..........
E2348/69     .......... .......... .......... .......... .......... .......... ..........
ECOR_51      .......... .......... .......... .......... .......... .......... ..........
ECOR_57      .......... .......... .......... .......... .......... .......... ..........
UTI89        .......... .......... .......... .......... .......... .......... ..........
E._albertii  .......... .C........ ......T..A .....TC... ....T..... .........G ........C.

            6021
K-12         CAGCCATCGA CAGCGGCCTG GTTGATATGA TCGATCTGGA GTTATTTACC GGTGATGATC AGGTTAAAGA
53638        .......... .......... .......... .......... .......... ..C....... ..........
ATCC_8739    .......... .......... .......... .......... .......... ..C....... ..........
ECOR_10      .......... .......... .......... .......... .......... .......... ..........
ECOR_11      .......... .......... .......... .......... .......... .......... ..........
B171         .......... .......... .......... .......... .......... ..C....... ..........
E24377A      .......... .......... .......... .......... .......... ..C....... ..........
ECOR_34      .......... .......... .......... .......... .......... ..C....... ..........
ECOR_71      .......... .......... .......... .......... .......... ..C....... ..........
SE11         .......... .......... .......... .......... .......... ..C....... ..........
O157_Sakai   .......... .......... .......... .......... .......... ..C....... ..........
ECOR_35      .......... .......... .......... .......... .......... ..C....... ..........
ECOR_36      .......... .......... .......... .......... .......... ..C....... ..........
SMS-3-5      .......... ...T...... .......... .......... .......... ..C....... ..........
536          .......... .......... .......... .......... .......... ..C....... ..........
APEC_O1      .......... ...T...... .......... .......... .......... ..C....... ..........
CFT073       .......... ...T...... .......... .......... .......... ..C....... ..........
E2348/69     .......... ...T...... .......... .......... .......... ..C....... ..........
ECOR_51      .......... ...T...... .......... .......... .......... ..C....... ..........
ECOR_57      .......... ...T...... .......... .......... .......... ..C....... ..........
UTI89        .......... ...T...... .......... .......... .......... ..C....... ..........
E._albertii  .C........ ...T..T... ..C....... .......... A......... .......CA. ..........
```

```
          6091
K-12         AACCGTCGCC TACGCCCACG CGCATGATGT GAAAGTAGTC ATGTCCAACC ATGACTTCCA TAAAACGCCG
53638        .......... .......... .......... ......T... .......... .......... ..........
ATCC_8739    .......... .......... .......... ......T... .......... .......... ..........
ECOR_10      .......... .......... .......... .......... .......... .......... ..........
ECOR_11      .......... .......... .......... .......... .......... .......... ..........
B171         .......... .......... .......... ......T... .......... .......... ..........
E24377A      .......... .......... .......... ......T... .......... .......... ..........
ECOR_34      .......... .......... .......... ......T... .......... .......... ..........
ECOR_71      .......... .......... .......... ......T... .......... .......... ..........
SE11         .......... .......... .......... ......T... .......... .......... ..........
O157_Sakai   .......... .......... .......... ......T... .......... .......... ..........
ECOR_35      .......... .......... .......... ......T... .......... .......... ..........
ECOR_36      .......... .......... .......... ......T... .......... .......... ..........
SMS-3-5      .......... .......... .......... ......T... .....T.... .......... ..........
536          .......... .......... .......... ......T... .......... .......... ..........
APEC_O1      .......... .......... .......... ......T... .......... .......... ..........
CFT073       .......... .......... .......... ......T... .......... .......... ..........
E2348/69     .......... .......... .......... ......T... .......... .......... ..........
ECOR_51      .......... .......... .......... ......T... .......... .......... ..........
ECOR_57      .......... .......... .......... ......T... .......... .......... ..........
UTI89        .......... .......... .......... ......T... .......... .......... ..........
E._albertii  ...T...... .......... A......... ......T..A .......... .......... ..........

          6161
K-12         GAAGCCGAAG AAATCATTGC CCGTCTGCGC AAAATGCAAT CCTTCGACGC CGATATTCCT AAGATTGCGC
53638        .......... .......... .......... ........G. .......... .......... ..........
ATCC_8739    .......... .......... .......... .......... .......... .......... ..........
ECOR_10      .......... .......... .......... .......... .......... .......... ..........
ECOR_11      .......... .......... .......... .......... .......... .......... ..........
B171         .......... .......... .......... ........G. .......... .......... ..........
E24377A      .......... .......... .......... ........G. .......... .......... ..........
ECOR_34      .......... .......... .......... ........G. .......... .......... ..........
ECOR_71      .......... .......... .......... ........G. .......... .......... ..........
SE11         .......... .......... .......... ........G. .......... .......... ..........
O157_Sakai   .......... .......... .......... ........G. .......... .......... ..........
ECOR_35      .......... .......... .......... ........G. .......... .......... ..........
ECOR_36      .......... .......... .......... ........G. .......... .......... ..........
SMS-3-5      .......... .......... .......... ........G. .......... .......... ..........
536          .......... .......... .......... ........G. .......... .......... ..........
APEC_O1      .......... .......... .......... ........G. .......... .......... ..........
CFT073       .......... .......... .......... ........G. .......... .......... ..........
E2348/69     .......... .......... .......... ........G. .......... .......... ..........
ECOR_51      .......... .......... .......... ........G. .......... .......... ..........
ECOR_57      .......... .......... .......... ........G. .......... .......... ..........
UTI89        .......... .......... .......... ........G. .......... .......... ..........
E._albertii  .......... .......... ...C...... ........G. .....A.T.. .......... ..A.......

          6231
K-12         TGATGCCGCA AAGTACCAGC GATGTGCTGA CGTTGCTTGC CGCGACCCTG GAGATGCAGG AGCAGTATGC
53638        .......... .......... .......... .......... .......... .......... ..........
ATCC_8739    .......... .......... .......... .......... .......... .......... ..........
ECOR_10      .......... .......... .......... .......... .......... .......... ..........
ECOR_11      .......... .......... .......... .......... .......... .......... ..........
B171         .......... .......... .......... ........A. .......... .......... ..........
E24377A      .......... .......... .......... ........A. .......... .......... ..........
ECOR_34      .......... .......... .......... ........A. .......... .......... ..........
ECOR_71      .......... .......... .......... .......... .......... .......... ..........
SE11         .......... .......... .......... ........A. .......... .......... ..........
O157_Sakai   .......... .......... .........G .......... .......... .......... ..........
ECOR_35      .......... .......... .......... .......... .......... .......... ..........
ECOR_36      .......... .......... .......... .......... .......... .......... ..........
SMS-3-5      .......... .......... .......... .......... .......... .......... ..........
536          .......... .......... .......... .......... .......... .......... ..........
APEC_O1      .......... .......... .......... .......... .......... .......... ..........
CFT073       .......... .......... .......... .......... .......... .......... .A........
E2348/69     .......... .......... .......... .......... .......... .......... ..........
ECOR_51      .......... .......... .......... .......... .......... .......... .A........
ECOR_57      .......... .......... .......... .......... .......... .......... .A........
UTI89        .......... .......... .......... .......... .......... .......... ..........
E._albertii  .......... ..AC...... .......... .T........ ...A...... ........C. .A........
```

```
               6301
K-12           CGATCGTCCA ATTATCACGA TGTCGATGGC AAAAACTGGC GTAATTTCTC GTCTGGCTGG TGAAGTATTT
53638          .......... ..C....... .......... .......... .......... .......... ..........
ATCC_8739      .......... ..C....... .......... .......... .......... .......... ..........
ECOR_10        .......... .......... .......... .......... .......... .......... ..........
ECOR_11        .......... .......... .......... .......... .......... .......... ..........
B171           .......... ..C....... .......... .......... .......... .......... ..........
E24377A        .......... ..C....... .......... .......... .......... .......... ..........
ECOR_34        .......... ..C....... .......... .......... .......... .......... ..........
ECOR_71        .......... ..C....... ....A..... .......... .......... .......... ..........
SE11           .......... ..C....... .......... .......... .......... .......... ..........
O157_Sakai     .......... ..C....... ....A..... .......... .......... .......... ..........
ECOR_35        .......... ..C....... .......... .......... .......... .......... ..........
ECOR_36        .......... ..C....... .......... .......... .......... .......... ..........
SMS-3-5        .......... ..C....... .......... .......... .......... .......... ..........
536            .......... ..C....... .......... .......... .......... .......... ..........
APEC_O1        .......... ..C....... .......... .......... .......... .......... ..........
CFT073         .......... ..C....... .......... .......... .......... .......... ..........
E2348/69       .......... ..C....... .......... .......... .......... .......... ..........
ECOR_51        .......... ..C....... .......... .......... .......... .......... ..........
ECOR_57        .......... ..C....... .......... .......... .......... .......... ..........
UTI89          .......... ..C....... .......... .......... .......... .......... ..........
E._albertii    ......C..G ..C.....C. .......... ...G...... ..C.....C. .......A.. ..........

               6371
K-12           GGCTCGGCGG CAACTTTTGG TGCGGTAAAA AAAGCGTCTG CGCCAGGGCA AATCTCGGTA AATGATTTGC
53638          .......... .......... .......... .....C.... .......... .......... ..........
ATCC_8739      ..G....... .......... .......... .....C.... .......... .......... .C......A.
ECOR_10        .......... .......... .......... .......... .......... .......... ..........
ECOR_11        .......... .......... .......... .......... .......... .......... ..........
B171           .......... .......... .......... .....C.... .A........ .......... ........A.
E24377A        .......... .......... .......... .....C.... .A........ .......... ........A.
ECOR_34        .......... .......... .......... .....C.... .A........ .......... ........A.
ECOR_71        .......... .......... .......... .....C.... .......... .......... ..........
SE11           .......... .......... .......... .....C.... .A........ .......... ........A.
O157_Sakai     .......... .......... .......... .....C.... .......... .......... ..........
ECOR_35        ..G....... .......... .......... .....C.... .......... .......... ..........
ECOR_36        ..G....... .......... .......... .....C.... .......... .......... ..........
SMS-3-5        ..G....... .......... .......... .....C.... .......... .......... ..........
536            ..G....... .......... .......... .....C.... .......... .......... ..........
APEC_O1        .......... .......... .......... .....C.... .......... .......... ..........
CFT073         ..G....... .......... .......... .....C.... .......... .......... ..........
E2348/69       ..G....... .......... .......... .....C.... .......... .......... ..........
ECOR_51        ..G....... .......... .......... .....C.... .......... .......... ..........
ECOR_57        ..G....... .......... .......... .....C.... .......... .......... ..........
UTI89          .......... .......... .......... .....C.... .......... .......... ..........
E._albertii    ..T..A.... ....C..... C..A...... .....C.... .C..C..... ...A.....T ........A.

               6441
K-12           GCACGGTATT AACTATTTTA CACCAGGCAT AAGCAATAAT ATTTCGGCGG GAACACCCTC -CCCGCCGAA
53638          ....A..... .......... ..T....... .......... .......... A..T...... C.........
ATCC_8739      ....A..... .......... ..T....... .......... .......... ...T.T.... C.........
ECOR_10        .......... .......... .......... .......... .......... .......... ..........
ECOR_11        .......... .......... .......... .......... .......... .......... ..........
B171           .......... .......... .......... .......... .......... .......... ..........
E24377A        .......... .......... .......... .......... .......... .......... ..........
ECOR_34        .......... .......... .......... .......... .......... .......... ..........
ECOR_71        ....A..... .......... ..T....... .......... .......... ...T.T.... C.........
SE11           .......... .......... .......... .......... .......... .......... ..........
O157_Sakai     ....A..... .......... ..T....... .......... .......... ...T.T.... C.........
ECOR_35        .T........ .......... ..T....... .......... .......... ...T...... C.........
ECOR_36        .T........ .......... ..T....... .......... .......... ...T...... C.........
SMS-3-5        .T........ .......... ..T....... .......... .......... ...T...... C.........
536            .T........ .......... ..T....... .......... .......... ...T...... C.........
APEC_O1        .T........ .......... ..T....... .......... .......... ...T...... C.........
CFT073         .T........ .......... ..T....... .......... .......... ...T...... T.........
E2348/69       .T........ .......... ..T....... .......... .......... ...T...... C.........
ECOR_51        .T........ .......... ..T....... .......... .......... ...T...... T.........
ECOR_57        .T........ .......... ..T....... .......... .......... ...T...... T.........
UTI89          .T........ .......... ..T....... .......... .......... ...T...... C.........
E._albertii    ....A..... ..G......G .......... ..TA...... CGC-...... ...T.ATAA. G.........
```

```
          6511
K-12         CTAAAAAA-- TATATTCAAT CGTATTTAAT AAAAATATTT CGTGAGTCTC TGTGCGCTAA TTCTCCATTT
53638        A.......AG ...T..T..C .......T.. .......... .......... .......... ........C.
ATCC_8739    ........A. .........C .......... .......... .......... .......... ..A.....C.
ECOR_10      .......... .......... .......... .......... .......... .......... ..........
ECOR_11      .......... .......... .......... .......... .......... .......... ..........
B171         ..T.....A. ......T..C .......... .......... .......... .......... ........C.
E24377A      ..T.....A. ......T..C .......... .......... .......... .......... ........C.
ECOR_34      ..T.....A. ......T..C .......... .......... .......... .......... ........C.
ECOR_71      ........A. ........GC .......... .......... .......... .......... ..A.....C.
SE11         ..T.....A. ......T..C .......... .......... .......... .......... ........C.
O157_Sakai   ........A. .........C .......... .......... .......... .......... ..A.....C.
ECOR_35      ........AG ...T..T..C .......... .......... .......... .......... ........C.
ECOR_36      ........AG ...T..T..C .......... .......... .......... .......... ........C.
SMS-3-5      ........AG ...T..T..C .......... .......... .......... .......... ........C.
536          .........G ...T..T..C .......... .......... .......... .......... ........C.
APEC_O1      ........AG ...T..T..C .......... .......... .......... .......... ........C.
CFT073       .......GAG ...T..T..C .......... .......... .......... .......... ........C.
E2348/69     .........G ...T..T..C .......... .......... .......... .......... ........C.
ECOR_51      .......GAG ...T..T..C .......... .......... .......... .......... ........C.
ECOR_57      .......GAG ...T..T..C .......... .......... .......... .......... ........C.
UTI89        ........AG ...T..T..C .......... .......... .......... .......... ........C.
E._albertii  TA..G.C.CT AT.T.....G A........C .........C T...G..A.. .........T ..T.--..CA

          6581
K-12         GGCGTAGGGA AAATCACATC TGAATCAGGA ATTAACAATG AAACCTGTAA AACCACCTCG TATTAATGGA
53638        .......... ........C. .......... .......... .......... ......T... ..........
ATCC_8739    .......... ........C. .......... .......... .......... .......... ..........
ECOR_10      .......... .......... .......... .......... .......... .......... ..........
ECOR_11      .A........ .......... .......... .......... .......... .......... ..........
B171         .......... .......... .......... .......... .......... .......... ..........
E24377A      .......... .......... .......... .......... .......... .......... ..........
ECOR_34      .......... .......... .......... .......... .......... .......... ..........
ECOR_71      .......... ........C. .......... .......... .......... .......... ..........
SE11         .......... .......... .......... .......... .......... .......... ..........
O157_Sakai   .......... ........C. .......... .......... .......... .......... ..........
ECOR_35      ...A...... ........C. .......... .......... .......... .......... ..........
ECOR_36      ...A...... ........C. .......... .......... .......... .......... ..........
SMS-3-5      .......... ........C. .......... .......... .......... .......... ..........
536          .......... ........C. .......... .......... .......... .......... ..........
APEC_O1      .......... ........C. .......... .......... .......... .......... ..........
CFT073       .......... ........C. .......... .......... .......... .......... ..........
E2348/69     .......... ........C. .......... .......... .......... .......... ..........
ECOR_51      .......... ........C. .......... .......... .......... .......... ..........
ECOR_57      .......... ........C. .......... .......... .......... .......... ..........
UTI89        .......... ........C. .......... .......... .......... .......... ..........
E._albertii  T...C...T. ..T.....C. .......... .AA....... .......... .......A.. .........G

          6651
K-12         CGAGTGCCGG TCCTGTCGGC ACAGGAAGCG GTGAATTATA TTCCCGACGA AGCAACACTT TGTGTGTTAG
53638        .......... .......... .......... .......... .......... .......... ........G.
ATCC_8739    .......... .......... .......... .......... .......... .......... ..........
ECOR_10      .......... .......... .......... .......... .......... .......... ..........
ECOR_11      .......... .......... .......... .......... .......... .......... ..........
B171         .......... .......... .......... .......... .......... .......... ..........
E24377A      .......... .......... .......... .......... .......... .......... ..........
ECOR_34      .......... .......... .......... .......... .......... .......... ..........
ECOR_71      .......... .......... .......... .......... .......... .......... ..........
SE11         .......... .......... .......... .......... .......... .......... ..........
O157_Sakai   .......... .......... .......... .......... .......... .......... ..........
ECOR_35      .......... .......... .......... .......... .......... .......... ..........
ECOR_36      .......... .......... .......... .......... .......... .......... ..........
SMS-3-5      .......... .......... .......... .......... .......... .......... ..........
536          .......... .......... .......... .......... .......... .......... ..........
APEC_O1      .......... .......... .......... .......... .......... .......... ..........
CFT073       .......... .......... .......... .......... .......... .......... ..........
E2348/69     .......... .......... .......... .......... .......... .......... ..........
ECOR_51      .......... .......... .......... .......... .......... .......... ..........
ECOR_57      .......... .......... .......... .......... .......... .......... ..........
UTI89        .......... .......... .......... .......... .......... .......... ..........
E._albertii  T.T..A.... .......A.. .......... ..A....... .......... ......G... .....A....
```

```
             6721
K-12         GCGCTGGCGG CGGTATTCTG GAAGCCACCA CGTTAATTAC TGCTCTTGCT GATAAATATA AACAGACTCA
53638        .......... .......... .......... .......... .......... .......... ..........
ATCC_8739    .......... .......... .......... .......... .......... .......... ..........
ECOR_10      .......... .......... .......... .......... .......... .......... ..........
ECOR_11      .......... .......... .......... .......... .......... .......... ..........
B171         .......... .......... .......... .......... A......... .......... ..........
E24377A      .......... .......... .......... .......... A......... .......... ..........
ECOR_34      .......... .......... .......... .......... .......... .......... ..........
ECOR_71      .......... .......... .......... .......... .......... .......... ..........
SE11         .......... .......... .......... .......... A......... .......... ..........
O157_Sakai   .......... .......... .......... .......... .......... .......... ..........
ECOR_35      .......... .......... .......... .......... .......... .......... ..........
ECOR_36      .......... .......... .......... .......... .......... .......... ..........
SMS-3-5      .......... .......... .......... .......... .......... .......... ..........
536          .......... .......... .......... .......... .......... .......... ..........
APEC_O1      .......... .......... .......... .......... .......... .......... ..........
CFT073       .......... .......... .......... .......... .......... .......... ..........
E2348/69     .......... .......... .......... .......... .......... .......... ..........
ECOR_51      .......... .......... .......... .......... .......... .......... ..........
ECOR_57      .......... .......... .......... .......... .......... .......... ..........
UTI89        .......... .......... .......... .......... .......... .......... ..........
E._albertii  ....A..... .......... ........A. .A........ C..C.....C ..G....... ....A..A..

             6791
K-12         AACACCACGT AATTTATCGA TTATTAGTCC AACAGGGCTT GGCGATCGCG CCGACCGTGG TATTAGTCCT
53638        ...G...... .......... .......... .......... .......... .......C.. .........G
ATCC_8739    ...G...... .......... .......... .......... .......... .......C.. .........G
ECOR_10      .......... .......... .......... .......... .......... .......... ..........
ECOR_11      .......... .......... .......... .......... .......... .......... ..........
B171         ...G...... .......... .......... G......... .......... .......... ..........
E24377A      ...G...... .......... .......... G......... .......... .......... ..........
ECOR_34      ...G...... .......... .......... .......... .......... .......C.. ..........
ECOR_71      ...G...... .......... .......... G......... .......... .......... ..........
SE11         ...G...... .......... .......... G......... .......... .......... ..........
O157_Sakai   ...G...... .......... .......... .......... .......... .......C.. .........G
ECOR_35      .......... .......... .......... .......... .......... .......... .........G
ECOR_36      .......... .......... .......... .......... .......... .......... .........G
SMS-3-5      .......... .......... .......... .......... .......... .......... C........G
536          .......... .......... .......... .......... .......... .......... .........G
APEC_O1      .......... .......... .......... .......... .......... .......... .........G
CFT073       .......... .......... .......... .......... .......... .......... .........G
E2348/69     ...G...... .......... ....A..... .......... .......... .......... .........G
ECOR_51      .......... .......... .......... .......... .......... .......... .........G
ECOR_57      .......... .......... .......... .......... .......... .......... .........G
UTI89        .......... .......... .......... .......... .......... .......... .........G
E._albertii  G..G...... ........A. .......C.. C......... .......... .......... .........G

             6861
K-12         CTGGCGCAAG AAGGTCTGGT GAAATGGGCA TTATGTGGTC ACTGGGGACA ATCGCCGCGT ATTTCTGAAC
53638        .......... .......... .......... .....C.... .......... .......... ........T.
ATCC_8739    .......... ........C. .......... .....C.... .......... .......... ........T.
ECOR_10      .......... .......... .......... .......... .......... .......... ..........
ECOR_11      .......... .......... .......... .......... .......... .......... ..........
B171         .......... .......... .......... .......... .......... .......... ..........
E24377A      .......... .......... .......... .......... .......... .......... ..........
ECOR_34      .......... .......... .......... .......... .......... .......... ..........
ECOR_71      .......... .......... .......... .......... .......... .......... ..........
SE11         .......... .......... .......... .......... .......... .......... ..........
O157_Sakai   .......... .......... .......... .....C.... .......... .......... ........T.
ECOR_35      .......... .......... .......... .....C.... .......... .......... ........T.
ECOR_36      .......... .......... .......... .....C.... .......... .......... ........T.
SMS-3-5      .....A.... .......... .......... .....C.... .......... .......... ........T.
536          .......... .......... .......... .....C.... .......... .......... ........T.
APEC_O1      .......... .......... .......... .....C.... .......... .......... ........T.
CFT073       .......... .......... .......... .....C.... .......... .......... ..........
E2348/69     .......... .......... .......... .....C.... .......... .......... ........T.
ECOR_51      .......... .......... .......... .....C.... .......... .......... ..........
ECOR_57      .......... .......... .......... .....C.... .......... .......... ..........
UTI89        .......... .......... .......... .....C.... .......... .......... ........T.
E._albertii  ..A....... .G......A. A......... .....C..A. .......... ...A...... ........T.
```

```
          6931
K-12         TCGCAGAACA AAATAAAATT ATTGCTTATA ACTACCCACA AGGTGTACTT ACACAAACCT TACGCGCCGC
53638        .....A.... .......... .......... ....T..... .......... .......... ..........
ATCC_8739    .......... .......... .......... ....T..... .......... .......... ..........
ECOR_10      .......... .......... .......... .......... .......... .......... ..........
ECOR_11      .......... .......... .......... .......... .......... .......... ..........
B171         .......... .......... .......... ....T..... .......... .......... ..........
E24377A      .......... .......... .......... ....T..... .......... .......... ..........
ECOR_34      .......... .......... .......... ....T..... .......... .......... ..........
ECOR_71      .......... .......... .......... ....T..... .......... .......... ..........
SE11         .......... .......... .......... ....T..... .......... .......... ..........
O157_Sakai   .......... .......... .......... ....T..... .......... .......... ..........
ECOR_35      .......... .......... .......... ....T..... .......... .......... ..........
ECOR_36      .......... .......... .......... ....T..... .......... .......... ..........
SMS-3-5      .......... .......... .......... ....T..... .......... .......... ..........
536          .......... .......... .......... ....T..... .......... .......... ..........
APEC_O1      .......... .......... .......... ....T..... .......... .......... ..........
CFT073       .......... .......... .......... ....T..... .......... .......... ..........
E2348/69     .......... .......... .......... ....T..... .......... .......... ..........
ECOR_51      .......... .......... .......... ....T..... .......... .......... ..........
ECOR_57      .......... .......... .......... ....T..... .......... .......... ..........
UTI89        .......... .......... .......... ....T..... .......... .......... ..........
E._albertii  .T........ .......... GCC....... ....T..G.. ...C..G... ..T.....AC .G..T.....

          7001
K-12         CGCAGCCCAC CAGCCTGGTA TTATTAGTGA TATTGGCATC GGGACATTTG TCGATCCACG CCAGCAAGGC
53638        .......... .......... .......... .......... .......... .......... ..........
ATCC_8739    .......... .......... .......... .......... .......... .......... ..........
ECOR_10      .......... .......... .......... .......... .......... .......... ..........
ECOR_11      .......... .......... .......... .......... .......... .......... ..........
B171         .......... .......... .......... .......... .......... .......... ..........
E24377A      .......... .......... .......... .......... .......... .......... ..........
ECOR_34      .......... .......... .......... .......... .......... .......... ..........
ECOR_71      .......... .......... .......... .......... .......... .......... ..........
SE11         .......... .......... .......... .......... .......... .......... ..........
O157_Sakai   .......... .......... .......... .......... .......... .......... ..........
ECOR_35      .......... .......... .......... .......... .......... .......... ..........
ECOR_36      .......... .......... .......... .......... .......... .......... ..........
SMS-3-5      .......... .......... .......... .......... ..A....... .......... ..........
536          .......... .......... .......... .......... ..A....... .......... ..........
APEC_O1      .......... .......... .......... .......... .......... .......... ..........
CFT073       .......... .......... .......... .......... ..A....... .......... ..........
E2348/69     .......... .......... .......... .......... ..A....... .......... ..........
ECOR_51      .......... .......... .......... .......... ..A....... .......... ..........
ECOR_57      .......... .......... .......... .......... ..A....... .......... ..........
UTI89        .......... .......... .......... .......... .......... .......... ..........
E._albertii  .......... .....A..A. .......C.. .......... .......... ....C..... ...A......

          7071
K-12         GGCAAACTGA ATGAAGTCAC TAAAGAAGAC CTGATTAAAC TGGTCGAGTT TGATAACAAA GAATATCTCT
53638        .......... .......... .......... .......... .......... .......... ..........
ATCC_8739    .......... .......... .......... .......... .......... .......... ..........
ECOR_10      .......... .......... .......... .......... .......... .......... ..........
ECOR_11      .......... .......... .......... .......... .......... .......... ..........
B171         .......... .......... G......... .......... .......... .......... ..........
E24377A      .......... .......... G......... .......... .......... .......... ..........
ECOR_34      .......... .......... G......... .......... .......... .......... ..........
ECOR_71      .......... .......... G......... .......... .......... .......... ..........
SE11         .......... .......... G......... .......... .......... .......... ..........
O157_Sakai   .......... .......... .......... .......... .......... .......... ..........
ECOR_35      .......... .......... .......... .......... .......... .......... ..........
ECOR_36      .......... .......... .......... .......... .......... .......... ..........
SMS-3-5      .......... .......... .......... .......... .......... .......... ..........
536          .......... .......... .......... .......... .......... .......... ..........
APEC_O1      .......... .......... .........A .......... .......... .......... ..........
CFT073       .......... .......... .........A .......... .......... .......... ..........
E2348/69     .......... .......... .......... .......... .......... .......... ..........
ECOR_51      .......... .......... .........A .......... .......... .......... ..........
ECOR_57      .......... .......... .........A .......... .......... .......... ..........
UTI89        .......... .......... .........A .......... .......... .......... ..........
E._albertii  ..A...T... ....T..G.. C......... .......... .......A.. C.....T... ..........
```

```
             7141
K-12         ATTACAAAGC GATTGCGCCA GATATTGCCT TCATTCGCGC TACCACCTGC GACAGTGAAG GCTACGCCAC
53638        .......... .......... .....C.... .......... .......... .....C.... ..........
ATCC_8739    .......... .......... .....C.... .......... .......... .....C.... ..........
ECOR_10      .......... .......... .......... .......... .......... .......... ..........
ECOR_11      .......... .......... .......... .......... .......... .......... ..........
B171         .......... .......... .....C.... .......... .......... .......... ..........
E24377A      ........C. .......... .....C.... .......... .......... .......... ..........
ECOR_34      .......... .......... .....C.... .......... .......... .......... ..........
ECOR_71      .......... .......... .....C.... .......... .......... .......... ..........
SE11         .......... .......... .....C.... .......... .......... .......... ..........
O157_Sakai   .......... A......... ........T. .......... .......... .......... ..........
ECOR_35      .......... ...A...... .....C.... .......... .......... .....C.... ..........
ECOR_36      .......... ...A...... .....C.... .......... .......... .....C.... ..........
SMS-3-5      ........T. .......... .....C.... .......... .......... .....C.... ..........
536          .......... .......... .....C.... .......... .......... .....C.... ..........
APEC_O1      .......... ...A...... .....C.... .......... .......... .....C.... ..........
CFT073       .......... ...A...... .....C.... .......... .......... .....C.... ..........
E2348/69     .......... .......... .....C.... .......... .......... .....C.... ..........
ECOR_51      .......... ...A...... .....C.... .......... .......... .....C.... ..........
ECOR_57      .......... ...A...... .....C.... .......... .......... .....C.... ..........
UTI89        .......... ...A...... .....C.... .......... .......... .....C.... ..........
E._albertii  .......... ...A..C..G ........T. .T........ C......... ..T....... ........T.

             7211
K-12         TTTTGAAGAT GAGGTGATGT ATCTCGACGC ATTGGTTATT GCCCAGGCGG TGCACAATAA CGGCGGTATT
53638        .......... .......... .......... .......... .......... .......... ..........
ATCC_8739    .......... .......... .......... .......... .......... .......... ..........
ECOR_10      .......... .......... .......... .......... .......... .......... ..........
ECOR_11      .......... .......... .......... .......... .......... .......... ..........
B171         .......... .......... .......... .......... .......... .......... ..........
E24377A      .......... .......... .......... .......... .......... .......... ..........
ECOR_34      .......... .......... .......... .......... .......... .......... ..........
ECOR_71      .......... .......... .......... .......... .......... .......... ..........
SE11         .......... .......... .......... .......... .......... .......... ..........
O157_Sakai   .......... .......... .......... .......... .......... .......... ..........
ECOR_35      .......... .......... .......... .......... ..T....... .......... ..........
ECOR_36      .......... .......... .......... .......... ..T....... .......... ..........
SMS-3-5      .......... .......... .......... .......... ..T....... .......... ..........
536          .......... .......... .......... .......... .......... .......... ..........
APEC_O1      .......... .......... .......... .......... ..T....... .......... ..........
CFT073       .......... .......... .......... .......... ..T....... .......... ..........
E2348/69     .......... .......... .......... .......... .......... .......... ..........
ECOR_51      .......... .......... .......... .......... ..T....... .......... ..........
ECOR_57      .......... .......... .......... .......... ..T....... .......... ..........
UTI89        .......... .......... .......... .......... ..T....... .......... ..........
E._albertii  A........C ..A..C.... .......... .C....G... .......... ....T..... ......A..C

             7281
K-12         GTGATGATGC AGGTGCAGAA AATGGTTAAG AAAGCCACGC TGCATCCTAA ATCTGTCCGT ATTCCGGGTT
53638        .......... .......... .......... G......... .......... .......... ..........
ATCC_8739    .......... .......... .......... .......... .......... .......... ..........
ECOR_10      .......... .......... .......... .......... .......... .......... ..........
ECOR_11      .......... .......... .......... .......... .......... .......... ..........
B171         .......... .......... .......... .......... .......... .......... ..........
E24377A      .......... .......... .......... .......... .......... .......... ..........
ECOR_34      .......... .......... .......... .......... .......... .......... ..........
ECOR_71      .......... .......... .......... .......... .......... .......... ..........
SE11         .......... .......... .......... .......... .......... .......... ..........
O157_Sakai   .......... .......... .......... ........A. .......... .......... ..........
ECOR_35      .......... .......... .......... .......... .......... .......... ..........
ECOR_36      .......... .......... .......... .......... .......... .......... ..........
SMS-3-5      .......... .......... .......... .......... .......... .......... ..........
536          .......... .......... .......... .......... .......... .......... ..........
APEC_O1      .......... .......... .......... .......... .......... .......... ..........
CFT073       .......... .......... .......... .......... .......... .......... ..........
E2348/69     .......... .......... .......... .......... .......... .......... ..........
ECOR_51      .......... .......... .......... .......... .......... .......... ..........
ECOR_57      .......... .......... .......... .......... .......... .......... ..........
UTI89        .......... .......... .......... .......... .......... .......... ..........
E._albertii  .......... .......A.. .......... ........AT .......A.. .G.G...... ........G.
```

```
         7351
K-12         ATCTGGTGGA TATTGTGGTG GTCGATCCGG ATCAAACCCA ACTGTATGGC GGTGCACCGG TTAACCGCTT
53638        .......... .......... .......... .......... .......... .......... ..........
ATCC_8739    .......... .......... .......... .......... .......... .......... ..........
ECOR_10      .......... .......... .......... .......... .......... .......... ..........
ECOR_11      .......... .......... .......... .......... .......... .......... ..........
B171         .......... .......... .......... ......G... .......... .....G.... .......T..
E24377A      .......... .......... .......... ......G... .......... .....G.... ..........
ECOR_34      .......... .......... .......... ......G... .......... .....G.... .......T..
ECOR_71      .......... .......... .......... ......G... .......... .....G.... .......T..
SE11         .......... .......... .......... ......G... .......... .....G.... .......T..
O157_Sakai   .......... .......... .......... ......G... ...A...... .....G.... ..........
ECOR_35      .......... .......... .......... .......... .......... .....G.... ..........
ECOR_36      .......... .......... .......... .......... .......... .....G.... ..........
SMS-3-5      .......... .......... .......... .......... .......... .......... ..........
536          .......... .......... .......... .......... .......... .....G.... ..........
APEC_O1      .......... .......... .......... .......... .......... .......... ..........
CFT073       .......... .......... .......... .......... .......... .......... ..........
E2348/69     .......... .......... .......... .......... .......... .....G.... ..........
ECOR_51      .......... .......... .......... .......... .......... .......... ..........
ECOR_57      .......... .......... .......... .......... .......... .......... ..........
UTI89        .......... .......... .......... .......... .......... .......... ..........
E._albertii  .......A.. .........C .....C..C. .G.....G.. G..A...... .....G.... .C..T.....

         7421
K-12         TATTTCTGGT GACTTCACCC TTGATGACAG TACCAAACTT AGCCTGCCCC TAAACCAACG TAAATTAGTT
53638        .......... .......... .......... .......... .......... .......... ..........
ATCC_8739    .......... .......... .......... .......... .......... .......... ..........
ECOR_10      .......... .......... .......... .......... .......... .......... ..........
ECOR_11      .......... .......... .......... .......... .......... .......... ..........
B171         .......... .......... .......... C......... .......... .......... ..........
E24377A      .......... .......... .......... C......T.. .......... .......... ..........
ECOR_34      .......... .......... .......... C......... .......... .......... ..........
ECOR_71      .......... .......... .......... C......... .......... .......... ..........
SE11         .......... .......... .......... C......... .......... .......... ..........
O157_Sakai   .......... .......... .......... C......... .......... .......... ..........
ECOR_35      .......... ........T. .......... C......... .......... .......... ..........
ECOR_36      .......... ........T. .......... C......... .......... .......... ..........
SMS-3-5      .......... .......... .......... .......... .......... .......... ..........
536          .......... ........T. .......... C......... .......... .......... ..........
APEC_O1      .......... .......... .......... .......... .......... .......... ..........
CFT073       .......... .......... .......... .......... .......... .......... ..........
E2348/69     .......... ........T. .......... C......... .......... .......... ..........
ECOR_51      .......... .......... .......... .......... .......... .......... ..........
ECOR_57      .......... .......... .......... .......... .......... .......... ..........
UTI89        .......... .......... .......... .......... .......... .......... ..........
E._albertii  ......C..C ..T.....G. .C........ C........G .C...C.... .C.....G.. ....C.G...

         7491
K-12         GCGCGGCGCG CATTATTCGA AATGCGTAAA GGCGCGGTGG GGAATGTCGG CGTCGGTATT GCTGACGGCA
53638        ...T....T. .......... .......... .......... .......... .......... ........T.
ATCC_8739    .......... .......... .......... .......... .......... .......... ..........
ECOR_10      .......... .......... .......... .......... .......... .......... ..........
ECOR_11      .......... .......... .......... .......... .......... .......... ..........
B171         ........T. .......... .......... .......... .......... .......... ........T.
E24377A      ...A....T. .......... .......... .......... .......... .......... ........T.
ECOR_34      ........T. .......... .......... .......... .......... .......... ........T.
ECOR_71      ........T. .......... .......... .......... .......... .......... ........T.
SE11         ........T. .......... .......... .......... .......... .......... ........T.
O157_Sakai   ..A.....T. .......... .......... .......... .......... .......... ..........
ECOR_35      ..A.....T. .......... ......C... .....A.... .......... .......... ..........
ECOR_36      ..A.....T. .......... ......C... .....A.... .......... .......... ..........
SMS-3-5      .......... .......... ......C... .....A.... .......... .......... ..........
536          ..A.....T. .......... ......C... .....A.... .......... .......... ..........
APEC_O1      ..A.....T. .......... ......C... .....A.... .......... .......... ..........
CFT073       ..A.....T. .......... ......C... .....A.... .......... .......... ..........
E2348/69     ..A.....T. .......... ......C... .....A.... .......... .......... ..........
ECOR_51      ..A.....T. .......... ......C... .....A.... .......... .......... ..........
ECOR_57      ..A.....T. .......... ......C... .....A.... .......... .......... ..........
UTI89        ..A.....T. .......... ......C... .....A.... .......... .......... ..........
E._albertii  ........G. .G....A... ......C.C. .....A..T. .T........ ...A..C... ..C.......
```

```
          7561
K-12          TTGGCCTGGT CGCCCGAGAA GAAGGTTGTG CTGATGACTT TATTCTGACG GTAGAAACAG GTCCGATTGG
53638         .......... ......G... .......... .......... .......... .......... ..........
ATCC_8739     .......... .......... .......... .......... .......... .......... ..........
ECOR_10       .......... .......... .......... .......... .......... .......... ..........
ECOR_11       .......... .......... .......... .......... .......... .......... ..........
B171          .......... ......G... .......... .......... .......... .......... ..........
E24377A       .......... ......G... .......... .......... .......... .......... ..........
ECOR_34       .......... ......G... .......... .......... .......... .......... ..........
ECOR_71       .......... ......G... .......... .......... .......... .......... ..........
SE11          .......... ......G... .......... .......... .......... .......... ..........
O157_Sakai    .......... ......G... .......... .......... .......... .......... ..........
ECOR_35       .......... ......G... .......... .......... .......... .......... ..........
ECOR_36       .......... ......G... .......... .......... .......... .......... ..........
SMS-3-5       .......... ......G... .......... .......... .......... .......... ..........
536           .......... ......G... .......... .......... .......... .......... ..........
APEC_O1       .......... ......G... .......... .......... .......... .......... ..........
CFT073        .......... ......G... .......... .......... .......... .......... ..........
E2348/69      .......... ......G... .......... A......... .......... .......... ..........
ECOR_51       .......... ......G... .......... .......... .......... .......... ..........
ECOR_57       .......... ......G... .......... .......... .......... .......... ..........
UTI89         .......... ......G... .......... .......... .......... .......... ..........
E._albertii   ....T..... ......G... .......... .......... .........A ..C.....C. ..........

          7631
K-12          CGGAATTACT TCACAGGGGA TCGCCTTTGG CGCGAACGTG AATACCCGTG CCATTCTGGA TATGACGTCC
53638         .......... ........T. .......... .......... ........C. .......... ..........
ATCC_8739     .......... .......... .......... .......... .......... .......... ..........
ECOR_10       .......... .......... .......... .......... .......... .......... ..........
ECOR_11       .......... .......... .......... .......... .......... .......... ..........
B171          .......... ........T. .......... .......... ........C. .......... ..........
E24377A       .......... ........T. .......... .......... ........C. .......... ..........
ECOR_34       .......... ........T. .......... .......... ........C. .......... ..........
ECOR_71       .......... ........T. .......... .......... ........C. .......... ..........
SE11          .......... ........T. .......... .......... ........C. .......... ..........
O157_Sakai    T......... ........T. .......... .......... .......... .......... ..........
ECOR_35       ...T...... .......... .......... .......... ........C. .......... ..........
ECOR_36       ...T...... .......... .......... .......... ........C. .......... ..........
SMS-3-5       ...T...... .......... .......... .......... ........C. .......... ..........
536           ...T...... .......... .......... .......... ........C. .......... ..........
APEC_O1       ...T...... .......... .......... .......... ........C. .......... ..........
CFT073        ...T...... .......... .......... .......... ........C. .......... ..........
E2348/69      ...T...... .......... .......... .......... ........C. .......... ..........
ECOR_51       ...T...... .......... .......... .......... ........C. .......... ..........
ECOR_57       ...T...... .......... .......... .......... ........C. .......... ..........
UTI89         ...T...... .......... .......... .......... ........C. .......... ..........
E._albertii   ...C.....C .....A..A. .T........ ...A.....T ........C. .......... C.........

          7701
K-12          CAGTTTGATT TTTATCACGG TGGCGGTCTG GATGTTTGTT ATTTGAGTTT TGCTGAAGTC GACCAGCACG
53638         .......... .......... ...T...... .......... .......... .......... ..........
ATCC_8739     .......... .......... .......... .......... .......... .......... ..........
ECOR_10       .......... .......... .......... .......... .......... .......... ..........
ECOR_11       .......... .......... .......... .......... .......... .......... ..........
B171          .......... .......... ...T...... .......... .......... .......... ..........
E24377A       .......... .......... ...T...... .......... .......... .......... ..........
ECOR_34       .......... .......... ...T...... .......... .......... .......... ..........
ECOR_71       .......... .......... ...T...... .......... .......... .......... ..........
SE11          .......... .......... ...T...... .......... .......... .......... ..........
O157_Sakai    .......... .......... ...T...... .......... .......... .......... ..........
ECOR_35       .......... .......... ...T...... .......... .......... .......... ..........
ECOR_36       .......... .......... ...T...... .......... .......... .......... ..........
SMS-3-5       .......... .......... ...T...... .......... .......... .......... ..........
536           .......... .......... ...T...... .......... .......... .......... ..........
APEC_O1       .......... .......... ...T...... .......... .......... .......... ..........
CFT073        .......... .......... ...T...... .......... .......... .......... ..........
E2348/69      .......... .......... C..T...... .......... .......... .......... ..........
ECOR_51       .......... .......... ...T...... .......... .......... .......... ..........
ECOR_57       .......... .......... ...T...... .......... .......... .......... ..........
UTI89         .......... .......... ...T...... .......... .......... .......... ..........
E._albertii   .....C.... ....C..... C..T...... .......... ....A..... ...C...... ..T.......
```

```
             7771
K-12         GTAACGTCGG CGTGCATAAA TTCAATGGTA AAATCATGGG CACCGGTGGA TTTATTGATA TCAGTGCCAC
53638        .......... .......... ........C. .......... T......... .......... ..........
ATCC_8739    .......... .......... .......... .......... .......... .......... ..........
ECOR_10      .......... .......... .......... .......... .......... .......... ..........
ECOR_11      .......... .......... .......... .......... .......... .......... ..........
B171         .......... .......... ........C. .......... T......... .......... ..........
E24377A      .......... .......... ........C. .......... T......... .......... ..........
ECOR_34      .......... .......... ........C. .......... T......... .......... ..........
ECOR_71      .......... .......... ........C. .......... T......... .......... ..........
SE11         .......... .......... ........C. .......... T......... .......... ..........
O157_Sakai   .......... .......... ........C. .......... T......... .......... ..........
ECOR_35      .......... .......... .......... .......... .......... .......... ..........
ECOR_36      .......... .......... .......... .......... .......... .......... ..........
SMS-3-5      .......... .......... .......... .......... .......... .......... ..........
536          .......... .......... .......... .......... .......... .......... ..........
APEC_O1      .......... .......... .......... .......... .......... .......... ..........
CFT073       .......... .......... .......... .......... .......... .......... ..........
E2348/69     .......... .......... .......... .......... .......... .......... ..........
ECOR_51      .......... .......... .......... .......... .......... .......... ..........
ECOR_57      .......... .......... .......... .......... .......... .......... ..........
UTI89        .......... .......... .......... .......... .......... .......... ..........
E._albertii  .......T.. .......... ........C. ....T..... ...T....T. .......... ....C.....

             7841
K-12         TTCGAAGAAA ATCATTTTCT GCGGCACATT AACTGCGGGC AGTTTAAAAA CAGAAATTAC CGACGGCAAA
53638        C......... ..T....... .......G.. .......... .......... ........G. ...T......
ATCC_8739    .......... .......... .......... .......... .......... .......... ..........
ECOR_10      .......... .......... .......... .......... .......... .......... ..........
ECOR_11      .......... .......... .......... .......... .......... .......... ..........
B171         C......... ..T....... .......G.. .......... .......... ........G. ...T......
E24377A      C......... ..T....... .......G.. ...C...... .......... ........G. ...T......
ECOR_34      C......... ..T....... .......G.. .......... .......... ........G. ...T......
ECOR_71      C......... ..T....... .......G.. .......... .......... ........G. ...T......
SE11         C......... ..T....... .......G.. .......... .......... ........G. ...T......
O157_Sakai   C......... ..T....... .......G.. .......... .......... ........G. ...T......
ECOR_35      .......... ...G.G.... .......... .......... .......... .......... ..........
ECOR_36      .......... ...G.G.... .......... .......... .......... .......... ..........
SMS-3-5      .......... ...G.G.... .......... .......... .......... .......... ..........
536          .......... ...G...... .......... .......... .......... .......... ..........
APEC_O1      .......... ...G...... .......... .......... .......... .......... ..........
CFT073       .......... ...G.G.... .......... .......... .......... .......... ..........
E2348/69     .......... ...G.G.... .......... .......... .......... .......... ..........
ECOR_51      .......... ...G.G.... .......... .......... .......... .......... ..........
ECOR_57      .......... ...G.G.... .......... .......... .......... .......... ..........
UTI89        .......... ...G...... .......... .......... .......... .......... ..........
E._albertii  C......... ..T....... .......T.. ...C..A... .......... .G........ ......T...

             7911
K-12         TTAAATATCG TCCAGGAAGG ACGGGTGAAG AAATTTATTC GGGAACTACC GGAAATTACT TTCAGCGGAA
53638        ..GC...... .......... ......A..T .......... .......... .......... ..T.......
ATCC_8739    .......... .......... .......... .......... .......... .......... ..........
ECOR_10      .......... .......... .......... .......... .......... .......... ..........
ECOR_11      .......... .......... .......... .......... .......... .......... ..........
B171         ..GC...... .......... ......A..T .......... .......... .......... ..T.......
E24377A      .......... .......... .......... .......... A......... .......... ..T.......
ECOR_34      ..GC...... .......... ......A..T .......... .......... .......... ..T.......
ECOR_71      ..GC...... .......... ......A..T .......... .......... .......... ..T.......
SE11         ..GC...... .......... ......A..T .......... .......... .......... ..T.......
O157_Sakai   ..G....... .......... .......... .......... .......... .......... ..T.......
ECOR_35      .......... .......... .......... .......... .......... ......A... ..........
ECOR_36      .......... .......... .......... .......... .......... ......A... ..........
SMS-3-5      .......... .......... .......... .......... .......... ......A... ..........
536          .......... .......... .......... .......... .......... .......... .....T....
APEC_O1      .......... .......... .......... .......... .......... .......... .....T....
CFT073       .......... .......... .......... .......... .......... ......A... ..........
E2348/69     .......... .......... .......... .......... .......... ......A... ..........
ECOR_51      .......... .......... .......... .......... .......... ......A... ..........
ECOR_57      .......... .......... .......... .......... .......... ......A... ..........
UTI89        .......... .......... .......... .......... .......... .......... .....T....
E._albertii  ........T. .......... ...A...... ........C. .T........ ......C... ..........
```

```
              7981
K-12          AAATCGCTCT CGAGCGAGGG CTGGATGTTC GTTATATCAC TGAGCGCGCA GTATTCACGC TGAAAGAAGA
53638         .......G.. ...A..G..A .......... ....C..... C..A..T..T .......... ..........
ATCC_8739     .......... .......... .......... .......... .......... .......... ..........
ECOR_10       .......... .......... .......... .......... .......... .......... ..........
ECOR_11       .......... .......... .......... .......... .......... .......... ..........
B171          .......G.. ...A..G..A .......... ....C..... C..A..T..T .......... ..........
E24377A       .......G.. ...A..G.AA .......... ....C..... C..A..T..T .......... ..........
ECOR_34       .......G.. ...A..G..A .......... ....C..... C..A..T..T .......... ..........
ECOR_71       .......G.. ...A..G..A .......... ....C..... C..A..T..T .......... ..........
SE11          .......G.. ...A..G..A .......... ....C..... C..A..T..T .......... ..........
O157_Sakai    .......... .......... .......... .......... .........C .......... ..........
ECOR_35       .......... .......... .......... .......... .........T .........T .A........
ECOR_36       .......... .......... .......... .......... .........T .........T .A........
SMS-3-5       .......... .......... .......... .......... .........C .........T .A........
536           .......... .A........ .......... .......... .........C .......... .A........
APEC_O1       .......... .A........ .......... .......... .........C .......... .A........
CFT073        .......... .......... .......... .......... .........C .......... .A........
E2348/69      .......... .......... .......... .......... .........C .........T .A........
ECOR_51       .......... .......... .......... .......... .........C .......... .A........
ECOR_57       .......... .......... .......... .......... .........C .......... .A........
UTI89         .......... .A........ .......... .......... .........C .......... .A........
E._albertii   .......... ...A..G..A .......... .......... .........C .....T.... ..........

              8051
K-12          CGGCCTGCAT TTAATCGAAA TCGCCCCTGG CGTCGATTTA CAAAAAGATA TTCTCGACAA AATGGATTTC
53638         .......... .......... .......... .......... .......... .......... ......C...
ATCC_8739     .......... .......... .......... .......... .......... .......... ..........
ECOR_10       .......... .......... .......... .......... .......... .......... ..........
ECOR_11       .......... .......... .......... .......... .......... .......... ..........
B171          .......... .......... .......... .......... .......... .......... ......C...
E24377A       .......... .......... .......... .......... .......... .......... ......C...
ECOR_34       .......... .......... .......... .......... .......... .......... ......C...
ECOR_71       .......... .......... .......... .......... .......... .......... ......C...
SE11          .......... .......... .......... .......... .......... .......... ......C...
O157_Sakai    ...A...... .......... .......... .......... .......... .......... ..........
ECOR_35       .......... .......... ....T..... .......... .......... .......... ..........
ECOR_36       .......... .......... ....T..... .......... .......... .......... ..........
SMS-3-5       .......... .......... ....T..... .......... .......... ..T....... ..........
536           .......... .......... ....T..... .......... .......... .......... ..........
APEC_O1       .......... .......... ....T..... .......... .......... .......... ..........
CFT073        .......... .......... ....T..... .......... .......... .......... ..........
E2348/69      .......... .......... ....T..... .......... .......... .......... ..........
ECOR_51       .......... .......... ....T..... .......... .......... .......... ..........
ECOR_57       .......... .......... ....T..... .......... .......... .......... ..........
UTI89         .......... .......... ....T..... .......... .......... .......... ..........
E._albertii   .......... .......... .T..T..C.. ...T.....G .......... .........G ..........

              8121
K-12          ACCCCAGTGA TTTCGCCAGA ACTCAAACTG ATGGACGAAA GATTATTTAT CGATGCGGCG ATGGGTTTTG
53638         .......... .......... .......... .......... .......... .......... ..........
ATCC_8739     .......... .......... .......... .......... .......... .......... ..........
ECOR_10       .......... .......... .......... .......... .......... .......... ..........
ECOR_11       .......... .......... .......... .......... .......... .......... ..........
B171          .......... .......... .......... .......... .......... .......... ..........
E24377A       .......... .......... .......... .......... .......... .......... ..........
ECOR_34       .......... .......... .......... .......... .......... .......... ..........
ECOR_71       .......... .......... .......... .......... .......... .......... ..........
SE11          .......... .......... .......... .......... .......... .......... ..........
O157_Sakai    .......... .......... .......... .......... .......... .......... ..........
ECOR_35       .......... .......... .......... .......... .......... .......... ..........
ECOR_36       .......... .......... .......... .......... .......... .......... ..........
SMS-3-5       .......... .......... .......... .......... .......... .......... ..........
536           .......... ....A..... ...G...... .......... .......... .......... ..........
APEC_O1       .......... ....A..... ...G...... .......... .......... .......... ..........
CFT073        .......... .......... .......... .......... .......... .......... ..........
E2348/69      .......... .......... .......... .......... .......... .......... ..........
ECOR_51       .......... .......... .......... .......... .......... .......... ..........
ECOR_57       .......... .......... .......... .......... .......... .......... ..........
UTI89         .........C ....A..... ...G...... .......... .......... .......... ..........
E._albertii   ........T. .......T.. G..T..G... .......... .G........ .......... ..........
```

```
           8191
K-12          TCCTGCCTGA AGCGGCTCAT TAACAGGAGT ATAATGATGG ATTTTTCTTT AACTGAAGAA CAAGAACTGC
53638         .......... .......... .......... .......... .......... .......... ..........
ATCC_8739     .......... .......... .......... .......... .......... .......... ..........
ECOR_10       .......... .......... .......... .......... .......... .......... ..........
ECOR_11       .......... .......... .......... .......... .......... .......... ..........
B171          .......... .......... .......... .......... .......... .......... ..........
E24377A       .......... .......... .......... .......... .......... .......... ..........
ECOR_34       .......... .......... .......... .......... .......... .......... ..........
ECOR_71       .......... .......... ...T...... .......... .......... .......... ..........
SE11          .......... .......... .......... .......... .......... .......... ..........
O157_Sakai    .......... .......... .......... ..C.C..... .......... .......... ..........
ECOR_35       .......... .......... .......... .......... .......... .......... ..........
ECOR_36       .......... .......... .......... .......... .......... .......... ..........
SMS-3-5       .......... .......... .......... .......... .......... .......... ..........
536           .......... .......... .......... .......... .......... .......... ..........
APEC_O1       .......... .......... .......... .......... ....C..... .......... ..........
CFT073        .......... .......... .......... .......... ....C..... .......... ..........
E2348/69      .......... .......... .......... .......... .......... .......... ..........
ECOR_51       .......... .......... .......... .......... ....C..... .......... ..........
ECOR_57       .......... .......... .......... .......... ....C..... .......... ..........
UTI89         .......... .......... .......... .......... ....C..... .......... ..........
E._albertii   .......... .......... .......... .....A.... .......... .......... ........A.

           8261
K-12          TGCTGGCCAG TATTCGCGAA CTGATTACGA CTAACTTTCC GGAAGAGTAT TTCCGCACCT GCGATCAAAA
53638         .......... .......... .......... .......... .......... .......... ..........
ATCC_8739     .......... .......... .......... .......... .......... .......... ..........
ECOR_10       .......... .......... .......... .......... .......... .......... ..........
ECOR_11       .......... .......... .......... .......... .......... .......... ..........
B171          .......... .......... .......... .......... .......... .......... ..........
E24377A       .......... .......... .......... .......... .......... .......... ..........
ECOR_34       .......... .......... .......... .......... .......... .......... ..........
ECOR_71       .......... .......... .......... .......... .......... .......... ..........
SE11          .......... .......... .......... .......... .......... .......... ..........
O157_Sakai    .......... .......... .......... .......... .......... .......... ..........
ECOR_35       .......... .......... .......... .......... .......... .......... ..........
ECOR_36       .......... .......... .......... .......... .......... .......... ..........
SMS-3-5       .......... .......... .......... .......... .......... .......... ..........
536           .......... .......... .......... .......... .......... .......... ..........
APEC_O1       .......... ......T... .......... .......... .......... .......... ..........
CFT073        .......... .......... .......... .......... .......... .......... ..........
E2348/69      .......... .......... .......... .......... .......... .......... ..........
ECOR_51       .......... .......... .......... .......... .......... .......... ..........
ECOR_57       .......... .......... .......... .......... .......... .......... ..........
UTI89         .......... ......T... .......... .......... .......... .......... ..........
E._albertii   .......... .........T ........A. .C.....C.. ......A... ..T..T.... ..........

           8331
K-12          CGGGACATAT CCGCGTGAGT TTATGCGGGG GCTCAGATAA CAACAGTGCC ACTACCACTT ACACGCGTAA
53638         .......... .......... .......... .......... .......... .......... ..........
ATCC_8739     .......... .......... .......... .......... .......... ..C....... ..........
ECOR_10       .......... .......... .......... .......... .......... .......... ..........
ECOR_11       .......... .......... .......... .......... .......... .......... ..........
B171          .......... .......... .......... .......... .......... .......... ..........
E24377A       .......... .......... .......... .......... .......... .......... ..........
ECOR_34       .......... .......... .......... .......... .......... .......... ..........
ECOR_71       .......... .......... .......... .......... .......... .......... ..........
SE11          .......... .......... .......... .......... .......... .......... ..........
O157_Sakai    .......... .......... .......... .......... .......... .......... ..........
ECOR_35       .........C .......... .......... .......... .......... .......... ..........
ECOR_36       .........C .......... .......... .......... .......... .......... ..........
SMS-3-5       .........C .......... .......... .......C.. .......... .......... ..........
536           .........C .......... .......... .......C.. .......... .......... ..........
APEC_O1       .........C .......... .......... .......C.. .......... .......... ..........
CFT073        .........C .......... .......... .......C.. .......... .......... ..........
E2348/69      .........C .......... .......... .......C.. .......... .......... ..........
ECOR_51       .........C .......... .......... .......... .......... .......... ..........
ECOR_57       .........C .......... .......... .......... .......... .......... ..........
UTI89         .........C .......... .......... .......C.. .......... .......... ..........
E._albertii   ...T..G... ........A. ......AA.. .......C.. ......C... ..C.....G. ..........
```

```
         8401
K-12            AAACGGCAAG GTCTACATCA ACGGACAAAA AACCTTTATT ACCGGTGCGA AAGAGTACCC GTATATGCTG
53638           .......... .......... .......... .......... .......... .......... ..........
ATCC_8739       .......... ..T....... .......G.. .......... .......... .......... ..........
ECOR_10         .......... .......... .......... .......... .......... .......... ..........
ECOR_11         .......... .......... .......... .......... .......... .......... ..........
B171            .......... .......... .......... .......... .......... .......... ..........
E24377A         .......... .......... .......... .......... .......... .......... ..........
ECOR_34         .......... .......... .......... .......... .......... .......... ..........
ECOR_71         .......... .......... .......... .......... .......... .......... ..........
SE11            .......... .......... .......... .......... .......... .......... ..........
O157_Sakai      .......... ..T....... .......G.. .......... .......... .......... ..........
ECOR_35         .......... ..T....... .......G.. .......... .......... .......T.. A.........
ECOR_36         .......... ..T....... .......G.. .......... .......... .......T.. A.........
SMS-3-5         .......... ..T....... .......G.. .......... .......... .......T.. A.........
536             .......... ..T....... .......G.. .......... .......... .......... A.........
APEC_O1         .......... ..T....... .......G.. .......... .......... .......... A.........
CFT073          .......... ..T....... .......G.. .......... .......... .......T.. A.........
E2348/69        .......... ..T....... .......G.. .......... .......... .......T.. A.........
ECOR_51         .......... ..T....... .......G.. .......... .......... .......T.. A.........
ECOR_57         .......... ..T....... .......G.. .......... .......... .......T.. A.........
UTI89           .......... ..T....... .......G.. .......... .......... .......... A.........
E._albertii     ...T...... ..T....... .......G.. .......... .....C.... .......... ..........

         8471
K-12            GTGCTGGCGC GCGATCCGCA ACCGAAAGAT CCCAAAAAAG CCTTCACCCT GTGGTGGGTC GACTCCAGTA
53638           .......... .......... .......... .......... .......... .......... ..........
ATCC_8739       ...T...... .......... .......... .......... .......... .......... ..........
ECOR_10         .......... .......... .......... .......... .......... .......... ..........
ECOR_11         .......... .......... .......... .......... .......... .......... ..........
B171            .......... .......... .......... .......... .......... .......... ..........
E24377A         ...T...... .......... .......... .......... .......... .......... ..........
ECOR_34         ...T...... .......... .......... .......... .......... .......... ..........
ECOR_71         ...T...... .......... .......... .......... .......... .......... ..........
SE11            .......... .......... .......... .......... .......... .......... ..........
O157_Sakai      .......... .......... .......... .......... .......... .......... ..........
ECOR_35         ...T...... .......... .......... ..G.....G. ....T..T.. .......... ..........
ECOR_36         ...T...... .......... .......... ..G.....G. ....T..T.. .......... ..........
SMS-3-5         ...T...... .......... .......... ..G.....G. ....T..T.. .......... ..........
536             ...T...... .......... .......... ..G.....G. ....T..T.. .......... ..........
APEC_O1         ...T...... .......... .......... ..G.....G. ....T..G.. .......... ..........
CFT073          ...T...... .......... .......... ..G.....G. ....T..T.. .......... ..........
E2348/69        ...T...... .......... .......... ..G.....G. ....T..T.. .......... ..........
ECOR_51         ...T...... .......... .......... ..G.....G. ....T..T.. .......... ..........
ECOR_57         ...T...... .......... .......... ..G.....G. ....T..T.. .......... ..........
UTI89           ...T...... .......... .......... ..G.....G. ....T..G.. .......... ..........
E._albertii     ..A.....C. .T.....AG. ...T.....C ..G....... .......AT. .......... ..G.....C.

         8541
K-12            AGCCCGGCAT TAAGATTAAC CCGCTGCATA AAATCGGCTG GCATATGCTC AGCACCTGCG AAGTCTATCT
53638           .......... .......... .......... .......... .......... .......... ..........
ATCC_8739       .......... .......... .......... .......... .......... .......... ..........
ECOR_10         .......... .......... .......... .......... .......... .......... ..........
ECOR_11         .......... .......... .......... .......... .......... .......... ..........
B171            .......... .......... .......... .......... .......... .......... ..........
E24377A         .......... .......... .......... .......... .......... .......... ..........
ECOR_34         .......... .......... .......... .......... .......... .......... ..........
ECOR_71         .......... .......... .......... .......... .......... .......... ..........
SE11            .......... .......... .......... .......... .......... .......... ..........
O157_Sakai      .......... .......... .......... .......... .......... .......... ..........
ECOR_35         .......... ...A..C..T ..A....... .......... .......... .......... ..........
ECOR_36         .......... ...A..C..T ..A....... .......... .......... .......... ..........
SMS-3-5         .A........ ...A..C..T ..A....... .......... .......... .......... ..........
536             .A........ ...A..C..T ..A....... .......... .......... .......... ..........
APEC_O1         .......... ...A..C..T ..A....... .......... .......... .......... ..........
CFT073          .......... ...A..C..T ..A....... .......... .......... .......... ..........
E2348/69        .......... ...A..C..T ..A....... .......... .......... .......... ..........
ECOR_51         .......... ...A..C..T ..A....... .......... .......... .......... ..........
ECOR_57         .......... ...A..C..T ..A....... .......... .......... .......... ..........
UTI89           .......... ...A..C..T ..A....... .......... .......... .......... ..........
E._albertii     .A........ C.....C... ...T...... ....T..... .........G .......... ..........
```

```
          8611
K-12          CGACAACGTG GAGGTTGAAG AGAGCGACAT GGTGGGCGAA GAAGGAATGG GTTTCCTCAA TGTGATGTAC
53638         .......... .......... .......... .......... .......... .......... ..........
ATCC_8739     .......... ..A....... .......... .......... .......... .......... ..........
ECOR_10       .......... .......... .......... .......... .......... .......... ..........
ECOR_11       .......... .......... .......... .......... .......... .......... ..........
B171          .......... .......... .......... .......... .......... .......... ..........
E24377A       .......... .......... .......... .......... .......... .......... ..........
ECOR_34       .......... .......... .......... .......... .......... .......... ..........
ECOR_71       .......... .......... .......... .......... .......... .......... ..........
SE11          .......... .......... .......... .......... .......... .......... ..........
O157_Sakai    .......... ..A....... .......... .......... .......... .......... ..........
ECOR_35       .......... ..A....... .......... .......... .......... .......... ..........
ECOR_36       .......... ..A....... .......... .......... .......... .......... ..........
SMS-3-5       .......... ..A....... .......... .......... .......... .......... ..........
536           .......... ..A....... .......... .......... .......... .......... ..........
APEC_O1       .......... ..A....... .......... .......... .......... .......... ..........
CFT073        .......... ..A....... .......... .......... .......... .......... ..........
E2348/69      .......... ..A....... .......... .......... .......... .......... ..........
ECOR_51       .......... ..A....... .......... .......... .......... .......... ..........
ECOR_57       .......... ..A....... .......... .......... .......... .......... ..........
UTI89         .......... ..A....... .......... .......... .......... .......... ..........
E._albertii   .........T ..A.....T. ....T..... ...T...... .....T.... .......... C.........

          8681
K-12          AACTTTGAGA TGGAGCGCCT GATCAACGCC GCGCGCAGCA CCGGCTTTGC CGAATGCGCC TTTGAAGATG
53638         .......... .......... .......... .......... .......... .......... ..........
ATCC_8739     .......... .......... .......... .......... .......... .......... ..........
ECOR_10       .......... .......... .......... .......... .......... .......... ..........
ECOR_11       .......... .......... .......... .......... .......... .......... ..........
B171          .......... .......... .......... .......... .......... .......... ..........
E24377A       .......... .......... .......... .......... .......... .......... ..........
ECOR_34       .......... .......... .......... .......... .......... .......... ..........
ECOR_71       .......... .......... .......... .......... .......... .......... ..........
SE11          .......... .......... .......... .......... .......... .......... ..........
O157_Sakai    .......... .......... .......... .......... .......... ...G..T... ..........
ECOR_35       .......... .......... .......... .......... .......... .........A ..C.......
ECOR_36       .......... .......... .......... .......... .......... .........A ..C.......
SMS-3-5       .......... .......... .......... .......... .......... .........A ..C.......
536           .......... .......... .......... .......... .......... .........A ..C.......
APEC_O1       .......... .......... .......... .......... .......... .........A ..C.......
CFT073        .......... .......... .......... .......... .......... ...G..T... ..........
E2348/69      .......... .......... .......... .......... .......... .........A ..C.......
ECOR_51       .......... .......... .......... .......... .......... ...G..T... ..........
ECOR_57       .......... .......... .......... .......... .......... ...G..T... ..........
UTI89         .......... .......... .......... .......... .......... .........A ..C.......
E._albertii   .......... ....A..... .......... ..C....... .......... .........A ..........

          8751
K-12          CCGCCCGCTA TGCCAACCAA CGTATCGCTT TTGGTAAGCC CATTGGTCAT AACCAGATGA TCCAGGAAAA
53638         .......... .......... .......... .......... .......... .......... ..........
ATCC_8739     .......... .......... .......... .......... .......... .......... ..........
ECOR_10       .......... .......... .......... .......... .......... .......... ..........
ECOR_11       .......... .......... .......... .......... .......... .......... ..........
B171          .......... .......... .......... .......... .......... .......... ..........
E24377A       .......... .......... .......... .......... .......... .......... ..........
ECOR_34       .......... .......... .......... .......... .......... .......... ..........
ECOR_71       .......... .......... .......... .......... .......... .......... ..........
SE11          .......... .......... .......... .......... .......... .......... ..........
O157_Sakai    ....T..... .........G .......... .......A.. .......... .......... ..........
ECOR_35       .......... .........G .......... .......... .......... .......... ..........
ECOR_36       .......... .........G .......... .......... .......... .......... ..........
SMS-3-5       .......... .........G .......... .......... .......... .......... ..........
536           .......... .........G .......... .......... .......... .......... ..........
APEC_O1       .......... .........G .......... .......... .......... .......... ..........
CFT073        .......... .........G .......... .......... .......... .......... ..........
E2348/69      .......... .........G .......... .......... .......... .......... .A........
ECOR_51       .......... .........G .......... .......... .......... .......... ..........
ECOR_57       .......... .........G .......... .......... .......... .......... ..........
UTI89         .......... .........G .......... .......... .......... .......... ..........
E._albertii   .......T.. ...T..T..G ........G. .......A.. G......... .......... .......G..
```

```
           8821
K-12         ACTGGCGAAC AAACTGCGCC GCCAGATTTA TCCAACAAGA CTTACCGGTT GAGGAAATTC AGATAGGATT
53638        .......... .......... .......... C..GG..... .......... .......... ..........
ATCC_8739    .......... .......... .......... .......... .......... .......... ..........
ECOR_10      .......... .......... .......... .......... .......... .......... ..........
ECOR_11      .......... .......... .......... .......... .......... .......... ..........
B171         .......... .......... .......... C..GG..... .......... .......... ..........
E24377A      .......... .......... .......... .......... .......... .......... ..........
ECOR_34      .......... .......... .......... .......... .......... .......... ..........
ECOR_71      .......... .......... .......... .......... .......... .......... ..........
SE11         .......... .......... .......... C..GG..... .......... .......... ..........
O157_Sakai   .......... .......... .......... ...GG..... .......... .......... ..........
ECOR_35      .......... .......... ...T...... ...GG..... .......... .......... .....A....
ECOR_36      .......... .......... .......... ...GG..... .......... .......... .....A....
SMS-3-5      .......... .......... .......... ...GG..... .....T.... .......... ..........
536          .......... .......... .......... ...GG..... .....T.... .......... ..........
APEC_O1      .......... .......... .......... ...GG..... .......... .......... ..........
CFT073       .......... .......... .......... ...GG..... .......... .......... ..........
E2348/69     ...T...... .......... .......... ...GG..... .......... .......... ..........
ECOR_51      .......... .......... .......... ...GG..... .......... .......... ..........
ECOR_57      .......... .......... .......... ...GG..... .......... .......... ..........
UTI89        .......... .......... .......... ...GG..... .......... .......... ..........
E._albertii  .......--- ---------- ---------- ---------- ---------- ---------- ----------

           8891
K-12         TCTTGTTCCG CAAAACAGTT TTTAAATTGT CTACGGTAAT CGCTGGGCGA ACAACCGACA TGGCGCAAAA
53638        .......... .......... .........C ..G....... .......... .......... ..........
ATCC_8739    .......... .......... .........C ..G....... .......T.. ...G...... ..........
ECOR_10      .......... .......... .......... .......... .......... .......... ..........
ECOR_11      .......... .......... .......... .......... .......... .......... ..........
B171         .......... .......... .........C ..G....... .......... .......... ..........
E24377A      .......... .......... .......... .......... .......... .......... ..........
ECOR_34      .......... .......... .......... .......... .......... .......... ..........
ECOR_71      .......... .......... .......... .......... .......... .......... ..........
SE11         .......... .......... .........C ..G....... .......... .......... ..........
O157_Sakai   .......... .......... .........C ..G....... .......... ...G...... ..........
ECOR_35      .......... .......... .........C ..G....... .......... ...G...... ..........
ECOR_36      .......... .......... .........C ..G....... .......... ...G...... ..........
SMS-3-5      .......... .......... .........C ..G....... .......... ...G...... ..........
536          .......... .......... .........C ..G....... .......... ...G...... ..........
APEC_O1      .......... .......... .........C ..G....... .......... ...G...... ..........
CFT073       .......... .......... .........C ..G....... .......... ...G...... ..........
E2348/69     .......... .......... .........C ..G....... .......... ...G...... ..........
ECOR_51      .......... .......... .........C ..G....... .......... ...G...... ..........
ECOR_57      .......... .......... .........C ..G....... .......... ...G...... ..........
UTI89        .......... .......... .........C ..G....... .......... ...G...... ..........
E._albertii  ---------- ---------- ---------- ---------- ---------- ---------- ----------

           8961
K-12         ACAGTTTGGC AAAGTGATCG ACATTTTCAT AACCCACACG CCAGGAGATC TCTGCCTGTG ATAATTCAGT
53638        .......... .......... .......... .......... .......... .......... ..........
ATCC_8739    ........C. .......... .......... .......... .......... .......... ..........
ECOR_10      .......... .......... .......... .......... .......... .......... ..........
ECOR_11      .......... .......... .......... .......... .......... .......... ..........
B171         .......... .......... .......... .......... .......... .......... ..........
E24377A      .......... .......... .......... .......... .......... .......... ..........
ECOR_34      .......... .......... .......... .......... .......... .......... ..........
ECOR_71      .......... .......... .......... .......... .......... .......... ..........
SE11         .......... .......... .......... .......... .......... .......... ..........
O157_Sakai   .......... .......... .......... .......... .......... .......... ..........
ECOR_35      .......... .......... .......... .......... .......... ....T..... ..........
ECOR_36      .......... .......... .......... .......... .......... ....T..... ..........
SMS-3-5      .......... .......... .......... .......... ......A... .......... ..........
536          .......... .......... .......... .......... ......A... .......... ..........
APEC_O1      .......... .......... .......... .......... ......A... .......... ..........
CFT073       .......... .......... .......... .......... .......... ........A. ..........
E2348/69     .......... .......... .......... .......... .......... .......... ..........
ECOR_51      .......... .......... .......... .......... .......... ........A. ..........
ECOR_57      .......... .......... .......... .......... .......... ........A. ..........
UTI89        .......... .......... .......... .......... ......A... .......... ..........
E._albertii  ---------- ---------- ---------- ---------- ---------- ---------- ----------
```

```
          9031
K-12         ATTAGTGAGT GACCATTTTG CTTCCGTCAT ACGCCGTTGA ATAACATAGT TAATAGGGGA AATACGATAC
53638        .......... ........C. .......... ..A....... .......... .......... ..........
ATCC_8739    .......... A.......C. .......... .......... .......... ....T..... ..........
ECOR_10      .......... .......... .......... .......... .......... .......... ..........
ECOR_11      .......... .......... .......... .......... .......... .......... ..........
B171         .......... ........C. .......... .......... .......... .......... ..........
E24377A      .......... .......... .......... .......... .......... .......... ..........
ECOR_34      .......... .......... .......... .......... .......... .......... ..........
ECOR_71      .......... .......... .......... .......... .......... .......... ..........
SE11         .......... ........C. .......... .......... .......... .......... ..........
O157_Sakai   .......... .......... .......... .......... .......... .......... ..........
ECOR_35      .......... ........C. .......... .......... .......... .......... ...G......
ECOR_36      .......... ........C. .......... .......... .......... .......... ...G......
SMS-3-5      .......... ........C. .......... .......... .......... .......... ..........
536          .......... ........C. .......... .......... .......... .......... ..........
APEC_O1      .......... ........C. .......... .......... .......... .......... ..........
CFT073       .......... ........C. .......... .......... .......... .......... ..........
E2348/69     .......... ........C. .......... .......... .......... .......... ..........
ECOR_51      .......... ........C. .......... .......... .......... .......... ..........
ECOR_57      .......... ........C. .......... .......... .......... .......... ..........
UTI89        .......... ........C. .......... .......... .......... .......... ..........
E._albertii  ---------- ---------- ---------- ---------- ---------- ---------- ----------

          9101
K-12         TCTTTGGTAA ATTCATGGCA AATATAACTG ACGCTGGCAC GAAATTTTTT CGATAACTGT TCTAAAGTGA
53638        .......... .......... .......... .......... .......... .......... ..........
ATCC_8739    .......... .......... .......... .......... .......... .......... ..........
ECOR_10      .......... .......... .......... .......... .......... .......... ..........
ECOR_11      .......... .......... .......... .......... .......... .......... ..........
B171         .......... .......... .......... .......... .......... .......... ..........
E24377A      .......... .......... .......... .......... .......... .......... ..........
ECOR_34      .......... .......... .......... .......... .......... .......... ..........
ECOR_71      .......... .......... .......... .......... .......... .......... ..........
SE11         .......... .......... .......... .......... .......... .......... ..........
O157_Sakai   .......... .......... .......... .......... .......... .......... ..........
ECOR_35      .......... .......... .......... .......... ....C..... .........C ..........
ECOR_36      .......... .......... .......... .......... ....C..... .........C ..........
SMS-3-5      .......... .......... G......... .......... .......... .........C ..........
536          .......... .......... G......... .......... .......... .........C ..........
APEC_O1      .......... .......... G......... .......... .......... .........C ..........
CFT073       .......... .......... G......... .......... .......... .........C ..........
E2348/69     .......... .......... G......... .......... .......... .........C ..........
ECOR_51      .......... .......... G......... .......... .......... .........C ..........
ECOR_57      .......... .......... G......... .......... .......... .........C ..........
UTI89        .......... .......... G......... .......... .......... .........C ..........
E._albertii  ---------- ---------- ---------- ---------- ---------- ---------- ----------

          9171
K-12         TTTTTTCGCG ATAATTATTA TTCAGATAGA AAAGAATATC TTTTATCAGA ACATCTTTTT TAATATACCC
53638        .......... .......... .......... .......... .......... .......... ..........
ATCC_8739    .......... .......... .......... .......... .......... .......... ..........
ECOR_10      .......... .......... .......... .......... .......... .......... ..........
ECOR_11      .......... .......... .......... .......... .......... .......... ..........
B171         .......... .......... .......... .......... .......... .......... ..........
E24377A      .......... .......... .......... .......... .......... .......... ..........
ECOR_34      .......... .......... .......... .......... .......... .......... ..........
ECOR_71      .......... .......... .......... .......... .......... .......... ..........
SE11         .......... .......... .......... .......... .......... .......... ..........
O157_Sakai   .......... .......... .......... .......... .......... .......... ..........
ECOR_35      .......... ...G...... .......... .......... .......... .......... .......T..
ECOR_36      .......... ...G...... .......... .......... .......... .......... .......T..
SMS-3-5      .......... ...G...... .......... .......... .......... .......... .......T..
536          .......... ...G...... .......... .......... .......... .......... .......T..
APEC_O1      .......... ...G...... .......... .......... .......... .......... .......T..
CFT073       .......... ...G...... .......... .......... .......... .......... .......T..
E2348/69     .......... ...G...... .......... .......... .......... .......... ..........
ECOR_51      .......... ...G...... .......... .......... .......... .......... .......T..
ECOR_57      .......... ...G...... .......... .......... .......... .......... .......T..
UTI89        .......... ...G...... .......... .......... .......... .......... .......T..
E._albertii  --------.. .......... ..A.....A. ......C... .......... .T........ .......T..
```

```
           9241
K-12          CTGCTCCGAA CGATAAGCAT TTTTAAAATT TTCGTAGTAA AGAATGGCTA ACGTATAGGC AAATGCATCC
53638         .......... .......... .......... .......... .......... .......... ..........
ATCC_8739     .......... .......... .......G.. .......... .....T.... .T........ ..........
ECOR_10       .......... .......... .......... .......... .......... .......... ..........
ECOR_11       .......... .......... .......... .......... .......... .......... ..........
B171          .......... .......... .......... .......... .......... .......... ..........
E24377A       .......... .......... .......... .......... .......... .......... ..........
ECOR_34       .......... .......... .......... .......... .......... .......... ..........
ECOR_71       .......... .......... .......... .......... .......... .......... ..........
SE11          .......... .......... .......... .......... .......... .......... ..........
O157_Sakai    .......... .......... .......G.. .......... .....T.... .T........ ..........
ECOR_35       .......... .......... .......G.. ...A...... .....T.... .......... ......G...
ECOR_36       .......... .......... .......G.. ...A...... .....T.... .......... ......G...
SMS-3-5       .......... .......... .......G.. .......... .....T.... .......... ......G...
536           .......... .......... .......G.. .......... .....T.... .......... ......G...
APEC_O1       .......... .......... .......G.. .......... .....T.... .......... ......G...
CFT073        .......... .......... .......G.. .......... .....T.... .......... ......G...
E2348/69      .......... .......... .......G.. .......... .....T.... .......... ......G...
ECOR_51       .......... .......... .......G.. .......... .....T.... .......... ......G...
ECOR_57       .......... .......... .......G.. .......... .....T.... .......... ......G...
UTI89         .......... .......... .......G.. .......... .....T.... .......... ......G...
E._albertii   ...T...... .....G.... .......G.. C..A..A... .....C.... .T.....C.. ...A..G...

           9311
K-12          CATAACGAAG ATGTTTGGCT ATTTTTACTT TGCGGCAAAA TCACACTTAG CTCATTAAAT AAGGTTTTTA
53638         .......... .......... .......... .......... .......... .......... ..........
ATCC_8739     .......... .......... .......... .......... .......... .......... ........A.
ECOR_10       .......... .......... .......... .......... .......... .......... ..........
ECOR_11       .......... .......... .......... .......... .......... .......... ..........
B171          .......... .......... .......... .......... .......... .......... ..........
E24377A       .......... .......... .......... .......... .......... .......... ..........
ECOR_34       .......... .......... .......... .......... .......... .......... ..........
ECOR_71       .......... .......... .......... .......... .......... .......... .....G....
SE11          .......... .......... .......... .......... .......... .......... ..........
O157_Sakai    .......... .......... .......... .......... .......... .......... ........A.
ECOR_35       .......... .......... .......... .......... .......... .......... ........A.
ECOR_36       .......... .......... .......... .......... .......... .......... ........A.
SMS-3-5       .......... .......... .......... .......... .......... .......... ........A.
536           .......... .......... .......... .......... .......... .......... ........A.
APEC_O1       .......... .......... .......... .......... .......... .......... ........A.
CFT073        .......... .......... .......... .......... .......... .......... ........A.
E2348/69      .......... .......... .......... .......... .......... .......... ........A.
ECOR_51       .......... .......... .......... .......... .......... .......... ........A.
ECOR_57       .......... .......... .......... .......... .......... .......... ........A.
UTI89         .......... .......... .......... .......... .......... .......... ........A.
E._albertii   ..CGTT.... ....C....C GC........ ........T. .A..G....A .........A .GA.....G.

           9381
K-12          TGACTTCTTT TCCCTGCCCC GCGGCAATTA CCGGACAAGA ATGCGGTTGC AGTAGCTGAT TTTCCTCAGC
53638         .......... .......... .......... .......... ....A..... .......... ..........
ATCC_8739     .......... .......... .......... .......... .......... .....A.... ..........
ECOR_10       .......... .......... .......... .......... .......... .......... ..........
ECOR_11       .......... .......... .......... .......... .......... .......... ..........
B171          .......... .......... .......... .......... .......... .......... ..........
E24377A       .......... .......... .......... .......... .......... .......... ..........
ECOR_34       .......... .......... .......... .......... .......... .......... ..........
ECOR_71       .......... .......... .......... .......... .......... .......... ..........
SE11          .......... .......... .......... .......... .......... .......... ..........
O157_Sakai    .......... .......... .......... .......... .......... .....A.... ..........
ECOR_35       .......... .........T .......... ....G..... .......... .......... ..........
ECOR_36       .......... .........T .......... ....G..... .......... .......... ..........
SMS-3-5       .......... .........T .......... ....G..... .......... .......... ..........
536           .......... .........T .......... ....G..... .......... .......... ..........
APEC_O1       .......... .........T .......... ....G..... .......... .......... ........A.
CFT073        .......... ......T..T .......... ....G..... .......... .......... ..........
E2348/69      .......... .........T .......... ....G..... .......... .......... ..........
ECOR_51       .......... ......T..T .......... ....G..... .......... .......... ..........
ECOR_57       .......... ......T..T .......... ....G..... .......... .......... ..........
UTI89         .......... .........T .......... ....G..... .......... .......... ........A.
E._albertii   ....C..... .........T ........C. .......G.. G..G...... ....A..... .......T..
```

```
          9451
K-12          CCCCTGAAAC TGAAAGCCGT ACAGCGCACA GGTACACGTC GTTGCCGGAT CGTTAACGTC AGAGGCCACC
53638         .......... .......... .......... .......... .......... .......... ..........
ATCC_8739     .......... .......... .......... .......... .......... .......... ..........
ECOR_10       .......... .......... .......... .......... .......... .......... ..........
ECOR_11       .......... .......... .......... .......... .......... .......... ..........
B171          .......... .......... .......... .......... .......... .......... ..........
E24377A       .......... .......... .......... .......... .......... .......... ..........
ECOR_34       .......... .......... .......... .......... .......... .......... ..........
ECOR_71       .......... .......... .......... .......... .......... .......... ..........
SE11          .......... .......... .......... .......... .......... .......... ..........
O157_Sakai    .......... .......... .......... A......... .......... .......... ..........
ECOR_35       .......... .......... .......... .......... .......... .......... ..........
ECOR_36       .......... .......... .......... .......... .......... .......... ..........
SMS-3-5       .......... .......... .......... .......... .......... .......... ..........
536           .......... .......... .......... .......... .......... .......... ..........
APEC_O1       .......... .......... .......... .......... .......... .......... ..........
CFT073        .......... .......... .......... .......... .......... .......... ..........
E2348/69      .......... .......... .......... .......... .......... .......... ..........
ECOR_51       .......... .......... .......... .......... .......... .......... ..........
ECOR_57       .......... .......... .......... .......... .......... .......... ..........
UTI89         .......... .......... .......... .......... .......... .......... ..........
E._albertii   G......... .....A.... .......... ......G... ..C.....G. .A........ ...A......

          9521
K-12          GCATGCAGCC TGCCGCGTTC TATCACCACA ATGTCATCCG CATGTGCGAC ATATAACGAA GAATCGATGG
53638         .......... .......... .........T ..A.....G. .......... .........T ..........
ATCC_8739     .......... .......... .........T ..A.....T. ....C..... .......... ..........
ECOR_10       .......... .......... .......... .......... .......... .......... ..........
ECOR_11       .......... .......... .......... .......... .......... .......... ..........
B171          .......... .......... .......... .......... .......... .......... ..........
E24377A       .......... .......... .......... .......... .......... .......... ..........
ECOR_34       .......... .......... .........T ..A.....G. .......... .........T ..........
ECOR_71       .......... .......... .......... .......... .......... .......... ..........
SE11          .......... .......... .........T ..A.....G. .......... .........T ..........
O157_Sakai    .......... .......... .........T ..A.....T. ....C..... .......... ..........
ECOR_35       .......... ....A..... .......... ........G. ....C..... .......... ..........
ECOR_36       .......... ....A..... .......... ........G. ....C..... .......... ..........
SMS-3-5       .......... ....A..... .......... ........G. ....C..... .......... ..........
536           .......... ....A..... .......... ........G. ....C..... .......... ..........
APEC_O1       .......... ....A..... .......... ........G. ....C..... .......... ..........
CFT073        .......A.. ....A..... .......... ........G. ....C..... .......... ..........
E2348/69      .......... ....A..... .......... ........G. ....C..... .......... ..........
ECOR_51       .......A.. ....A..... .......... ........G. ....C..... .......... ..........
ECOR_57       .......A.. ....A..... .......... ........G. ....C..... .......... ..........
UTI89         .......... ....A..... .......... ........G. ....C..... .......... ..........
E._albertii   .....T.... ......A... ...T...... ........G. ....G..... ......T... ..G..A....

          9591
K-12          TAAACCTTGC AACCCCTTTC TTGACGTAAA TCAACTCGGT TTCATTGTCA TGAACGTGAT GTCCGGACTC
53638         .......... .......... .......... ...G...... .......... .......... ....A.....
ATCC_8739     .......... C......... .......... ...G...... .......... .......... ....A.....
ECOR_10       .......... .......... .......... .......... .......... .......... ..........
ECOR_11       .......... .......... .......... .......... .......... .......... ..........
B171          .......... T......... .......... ...G...... .......... .......... ....A.....
E24377A       .......... .......... .......... .......... .......... .......... ..........
ECOR_34       .......... T......... .......... ...G...... .......... .......... ....A.....
ECOR_71       .......... .......... .......... .......... .......... .......... ..........
SE11          .......... .......... .......... ...G...... .......... .......... ....A.....
O157_Sakai    .......... C......... .......... ...G...... .......... .......... ....A.....
ECOR_35       .......... T......... .......... ...G...... .......... .......... ..........
ECOR_36       .......... T......... .......... ...G...... .......... .......... ..........
SMS-3-5       .......... T......... .......... ...G...... .......... .......... ..........
536           .......... T......... .......... ...G...... .......... .......... ....A.....
APEC_O1       .......... T......... .......... ...G...... .......... .......... ....A.....
CFT073        .......... T......... .......... ...G...... .......... .......... ....A.....
E2348/69      .......... T......... .......... ...G...... .......... .......... ....A.....
ECOR_51       .......... T......... .......... ...G...... .......... .......... ....A.....
ECOR_57       .......... T......... .......... ...G...... .......... .......... ....A.....
UTI89         .......... T......... .......... ...G...... .......... .......... ....A.....
E._albertii   .......... C..T..C..T .......... .T.G...... ......A... ........G. .C..C.....
```

```
         9661
K-12        CCATTTTGGA TCATCGCTAA ATGCAAAACG TGAAAGCCGT GGCGTTTTAC CGGCGACAAA CAGCGTTTCA
53638       .......... .......... .......... .......... .......... .......... ..........
ATCC_8739   .......... .......... .......... .......... .......... .A........ ..........
ECOR_10     .......... .......... .......... .......... .......... .......... ..........
ECOR_11     .......... .......... .......... .......... .......... .......... ..........
B171        .......... .......... T......... .......... .......... .......... ..........
E24377A     .......... .......... .......... .......... ..T....... .......... ..........
ECOR_34     .......... .......... T......... .......... .......... .......... ..........
ECOR_71     .......... .......... .......... .......... .......... .......... ..........
SE11        .......... .......... .......... .......... .......... .......... ..........
O157_Sakai  .......... .......... .......... .......... .......... .A........ ..........
ECOR_35     .......... .......... ....G..... .......... .......... .......... ..........
ECOR_36     .......... .......... ....G..... .......... .......... .......... ..........
SMS-3-5     .......... .......... ....G..... .......... .......... .......... ..........
536         .......... .......... ....G..... .......... .......... .......... ..........
APEC_O1     .......... .......... ....G..... .......... .......... .......... ..........
CFT073      .......... .......... ....G..... .......... .......... .......... ..........
E2348/69    .......... .......... ....G..... .......... .......... .......... ..........
ECOR_51     .......... .......... ....G..... .......... .......... .......... ..........
ECOR_57     .......... .......... ....G..... .......... .......... .......... ..........
UTI89       .......... .......... ....G..... .......... .......... .......... ..........
E._albertii ......G... .....A.... .G.....G.. ......T... .......... ...GC..... ...T.....G

         9731
K-12        CTGGCGTTAT CAAAACAGCG TTGATACATG ACAACCTCCC TATTCCATGA GCAAGCAAAA ACAATATATG
53638       .....A.... .......... .......... .......... .......... .......... ..........
ATCC_8739   .....A.... .......... .......... .......... .......... .......... ..........
ECOR_10     .......... .......... .......... .......... .......... .......... ..........
ECOR_11     .......... .......... .......... .......... .......... .......... ..........
B171        .....A.... .......... .......... .......... .......... .......... ..........
E24377A     .......... .......... .......... .......... .......... .......... ..........
ECOR_34     .....A.... .......... .......... .......... .......... .......... ..........
ECOR_71     .......... .......... .......... .......... .......... .......... ..........
SE11        .....A.... .......... .......... .......... .......... .......... ..........
O157_Sakai  .....A.... .......... .......... .......... .......... .......... ..........
ECOR_35     .......... .......... .......... .......... .......... .......... ..........
ECOR_36     .......... .......... .......... .......... .......... .......... ..........
SMS-3-5     .....A.... .......... .......... .......... .......... .......... ..........
536         .....A.... .......... .......... .......... .......... ....A..... ..........
APEC_O1     .....A.... .......... .......... .......... .......... ....A..... ..........
CFT073      .....A.... .......... .......... .......... .......... ....A..... ..........
E2348/69    .....A.... .......... .......... .......... .......... ....A..... ..........
ECOR_51     .....A.... .......... .......... .......... .......... ....A..... ..........
ECOR_57     .....A.... .......... .......... .......... .......... ....A..... ..........
UTI89       .....A.... .......... .......... .......... .......... ....A..... ..........
E._albertii .....A.... ......T.T. .......... .......... .......... .......... ..........

         9801
K-12        CCGGATAAAA TACGGCGTGT ATTGCGGTTA TACAACCGCG TTTAGCGAGA GCTAAACAAG ATTATTTACA
53638       .......... .......... .......... .......... .......... .......... ..........
ATCC_8739   .......... .......... .......... .......... .......... .......... ..........
ECOR_10     .......... .......... .......... .......... .......... .......... ..........
ECOR_11     .......... .......... .......... .......... .......... .......... ..........
B171        .......... .......... .......... .......... .......... .......... ..........
E24377A     .......... .......... .......... .......... .......... .......... ..........
ECOR_34     .......... .......... .......... .......... .......... .......... ..........
ECOR_71     .......... .......... .......... .......... .......... .......... ..........
SE11        .......... .......... .......... .......... .......... .......... ..........
O157_Sakai  .......... .......... .......... .......... .......... .......... ..........
ECOR_35     .......... .......... .......... .......... .......... .......... ..........
ECOR_36     .......... .......... .......... .......... .......... .......... ..........
SMS-3-5     .......... .......... .......... .......... .......... .......... ..........
536         .......... .......... .......... .......... .......... .......... ..........
APEC_O1     .......... .......... .......... .......... .......... .......... ..........
CFT073      .......... .......... .......... .......... .......... .......... ..........
E2348/69    .......... .......... .......... .......... .......... .......... ..........
ECOR_51     .......... .......... .......... .......... .......... .......... ..........
ECOR_57     .......... .......... .......... .......... .......... .......... ..........
UTI89       .......... .......... .......... .......... .......... .......... ..........
E._albertii .A........ .......... .CG....... ......T... .......... .......... ..........
```

```
           9871
K-12         CTGAATGCAA GATTGTACGG TCATGGAATA ACTATATTGC CGCAAATTGC ACTTTTGTTT TAGCGATCGC
53638        .......... .......... .......... .......... .......... .......... ..........
ATCC_8739    .......... .......T.. .......... .......... .......... .......... ..........
ECOR_10      .......... .......... .......... .......... .......... .......... ..........
ECOR_11      .......... .......... .......... .......... .......... .......... ..........
B171         .......... .......... .......... .......... .......... .......... ..........
E24377A      .......... .......... .......... .......... .......... .......... ..........
ECOR_34      .......... .......... .......... .......... .......... .......... ..........
ECOR_71      .......... .......... .......... .......... .......... .......... ..........
SE11         .......... .......... .......... .......... .......... .......... ..........
O157_Sakai   .......... .......... .......... .......... .......... .......... ..........
ECOR_35      .......... .......... .......... .......... .......... .......... ..........
ECOR_36      .......... .......... .......... .......... .......... .......... ..........
SMS-3-5      .......... .......... .......... .......... .......... .......... ..........
536          .......... .....C.... .......... .......... .......... .......... ..........
APEC_O1      .......... .....C.... .......... .......... .......... .......... ..........
CFT073       .......... .....C.... .......... .......... .......... .......... ..........
E2348/69     .......... .....C.... .......... .......... .......... .......... ..........
ECOR_51      .......... .....C.... .......... .......... .......... .......... ..........
ECOR_57      .......... .....C.... .......... .......... .......... .......... ..........
UTI89        .......... .....C.... .......... .......... .......... .......... ..........
E._albertii  G......... ...A.....T C..GCAC.G. .TA.....T. T.T.T...T. G......... .CA.......

           9941
K-12         ATTTTTTTTG CAAGATTGTT GGCAAGGAAA ACAGCTTGCT CCGTCGAAAA CCCCGCACCG CTATCGCACA
53638        .......... .......... .......... .......... .......... .......... ..........
ATCC_8739    .......... .......... .......... .......... .......... .......... ..........
ECOR_10      .......... .......... .......... .......... .......... .......... ..........
ECOR_11      .......... .......... .......... .......... .......... .......... ..........
B171         .......... .......... .......... .......... .......... .......... ..........
E24377A      .......... .......... .......... .......... .......... .......... ..........
ECOR_34      .......... .......... .......... .......... .......... .......... ..........
ECOR_71      .......... .......... .......... .......... .......... .......... ..........
SE11         .......... .......... .......... .......... .......... .......... ..........
O157_Sakai   .......... .......... .......... .......... .......... .......... ..........
ECOR_35      .......... .......... .......... .......... .......... .......... ..........
ECOR_36      .......... .......... .......... .......... .......... .......... ..........
SMS-3-5      .......... .......... .......... .......... .......... .......... ..........
536          .......... .......... .......... .......... .......... .......... ..........
APEC_O1      .......... .......... A......... .......... .......... .......... ..........
CFT073       .......... .......... .......... .......... .......... .......... ..........
E2348/69     .......... .......... .......... .......... .......... .......... ..........
ECOR_51      .......... .......... .......... .......... .......... .......... ..........
ECOR_57      .......... .......... .......... .......... .......... .......... ..........
UTI89        .......... .......... A......... .......... .......... .......... ..........
E._albertii  .......... .......... .......... .......... .......... ....TTC.T. ..........

          10011
K-12         CTATTTTCAG GCCATTTTTA CCTTCCATCG GAGATGGTTC CGTATGCGAC TCACAGGAGA AATCATGAAA
53638        .......... .......... .......... .......... .......... .......... ..........
ATCC_8739    .......... .......... .......... .......... .......... .......... ..........
ECOR_10      .......... .......... .......... .......... .......... .......... ..........
ECOR_11      .......... .......... .......... .......... .......... .......... ..........
B171         .......... .......... .......... .......... .......... .......... ..........
E24377A      .......... .......... .......... .......... .......... .......... ..........
ECOR_34      .......... .......... .......... .......... .......... .......... ..........
ECOR_71      .......... .......... .......... .......... .......... .......... ..........
SE11         .......... .......... .......... .......... .......... .......... ..........
O157_Sakai   .......... .......... .......... .......... .......... .......... ..........
ECOR_35      .......... .......... .......... .......... .......... .......... ..........
ECOR_36      .......... .......... .......... .......... .......... .......... ..........
SMS-3-5      .......... .....A.... .......... .......... .......... .......... ..........
536          .......... .......... .......... .......... .......... .......... ..........
APEC_O1      .......... .......... .......... .......... .......... .......... ..........
CFT073       .......... .......... .........A .......... .......... .......... ..........
E2348/69     .......... .......... .......... .......... .......... .......... ..........
ECOR_51      .......... .......... .........A .......... .......... .......... ..........
ECOR_57      .......... .......... .........A .......... .......... .......... ..........
UTI89        .......... .......... .......... .......... .......... .......... ..........
E._albertii  .......... .......... .......... .......... .......... .......... ..........
```

```
            10081
K-12         ATAATAACCT GCTTTAAGCT GGTGCCTGAA GAACAGGACA TTGTTGTCAC TCCAGAATAC ACCCTGAATT
53638        .......... .......... .......... .......... .......... .......... ..........
ATCC_8739    .......... .......... .......... .......... .......... .......... ..........
ECOR_10      .......... .......... .......... .......... .......... .......... ..........
ECOR_11      .......... .......... .......... .......... .......... .......... ..........
B171         .......... .......... .......... .......... .......... .......... ..........
E24377A      .......... .......... .......... .......... .......... .......... ..........
ECOR_34      .......... .......... .......... .......... .......... .......... ..........
ECOR_71      .......... .......... .......... .......... .......... .......... ..........
SE11         .......... .......... .......... .......... .......... .......... ..........
O157_Sakai   .......... .......... .......... .......... .......... .......... ..........
ECOR_35      .......... .......... .......... .......... .......... .........T ..........
ECOR_36      .......... .......... .......... .......... .......... .........T ..........
SMS-3-5      .......... .......... .......... .......... .......... .........T ..........
536          .......... .......... .......... .......... .......... .......... ..........
APEC_O1      .......... .......... .......... .......... .......... .......... ..........
CFT073       .......... .......... .......... .......... .......... .......... ..........
E2348/69     .......... .......... .......... .......... .......... .......... ..........
ECOR_51      .......... .......... .......... .......... .......... .......... ..........
ECOR_57      .......... .......... .......... .......... .......... .......... ..........
UTI89        .......... .......... .......... .......... .......... .......... ..........
E._albertii  .......... .......... .......... .......... ....G..... ...C..C... .....T....

            10151
K-12         TCGACAATGC CGACGCCAAA ATCAGCCAGT TCGATCTCAA TGCCATTGAA GCTGCAAGCC AGCTCGCAAC
53638        .......... .......... .......... .......... .......... .......... ....T.....
ATCC_8739    .......... .......... .......... .......... .......... .......... ....T.....
ECOR_10      .......... .......... .......... .......... .......... .......... ..........
ECOR_11      .......... .......... .......... .......... .......... .......... ..........
B171         .......... .......... .......... .......... .......... .......... ....T.....
E24377A      .......... .......... .......... .......... .......... .......... ..........
ECOR_34      .......... .......... .......... .......... .......... .......... ..........
ECOR_71      .......... .......... .......... .......... .......... .......... .....A....
SE11         .......... .......... .......... .......... .......... .......... ....T.....
O157_Sakai   .......... .......... .......... .......... .......... .......... ....T.....
ECOR_35      .......... .......... .......... .......... .......... .......... ..A.T.....
ECOR_36      .......... .......... .......... .......... .......... .......... ..A.T.....
SMS-3-5      .......... .......... .......... .......... .......... .......... ....T...T.
536          .......... .......... .......... .......... .......... .......... ....T.....
APEC_O1      .......... .......... .......... .......... .......... .T........ ....T.....
CFT073       .......... .......... .......... .......... .......... .......... ....T.....
E2348/69     .......... .......... .......... .......... .......... .......... ....T.....
ECOR_51      .......... .......... .......... .......... .......... .......... ....T.....
ECOR_57      .......... .......... .......... .......... .......... .......... ....T.....
UTI89        .......... .......... .......... .......... .......... .T........ ....T.....
E._albertii  ....T...A. .......... .......... .T........ .......... ..C..C.A.. T.......GG

            10221
K-12         AGATGACGAT GAGATAGCCG CGCTGACCGT TGGCGGCTCA TTGTTGCAGA ACTCGAAAGT GCGCAAAGAC
53638        .......... .........T .......... .......... .......... .......... ..........
ATCC_8739    .......... .......... .......... .......... .......... .......... ..........
ECOR_10      .......... .......... .......... .......... .......... .......... ..........
ECOR_11      .......... .......... .......... .......... .......... .......... ..........
B171         .......... .......... .......... .......... .......... .......... ..........
E24377A      .......... .......... .......... .......... .......... .......... ..........
ECOR_34      .......... A......... .......... .......... .......... .......... ..........
ECOR_71      .......... .......... .......... .......... .......... .......... ..........
SE11         .......... .......... .......... .......... .......... .......... ..........
O157_Sakai   .......... .......... .......... .......... .......... .......... ..........
ECOR_35      .......... .......... ........A. .......... .......... ...T...... ..........
ECOR_36      .......... .......... ........A. .......... .......... ...T...... ..........
SMS-3-5      .......... .......... ........A. .......... .......... ...A...... ..........
536          .......... .......... .......... C........T .......... .......... ..........
APEC_O1      .......... .......... .......... C........T .......... .......... ..........
CFT073       .......... .......... .......... C........T .......... .......... ..........
E2348/69     .......... .......... ..T....... C........T .......... .......... ..........
ECOR_51      .......... .......... .......... C........T .......... .......... ..........
ECOR_57      .......... .......... .......... C........T .......... .......... ..........
UTI89        .......... .......... .......... C........T .......... .......... ..........
E._albertii  CA...G.... .....T.T.. .......... C.....T... .....A..A. ....A..... ..........
```

```
        10291
K-12          GTGCTATCCC GCGGGCCGCA CAGCCTGTAT TTGGTGCAGG ATGCGCAACT TGAACATGCA CTGCCTCTCG
53638         .......... .......... .......... .......... .......... .......... ..........
ATCC_8739     .......... .......... .......... A......... ....A..... ...G...... ..........
ECOR_10       .......... .......... .......... .......... .......... .......... ..........
ECOR_11       .......... .......... .......... .......... .......... .......... ..........
B171          .......... .......... .......... .......... .......... .......... ..........
E24377A       .......... .......... .......... .......... .......... .......... ..........
ECOR_34       .......... .......... .......... .......... .......... .......... ..........
ECOR_71       .......... .......... .......... .......... .......... .......... ..........
SE11          .......... .......... .......... .......... .......... .......... ..........
O157_Sakai    .......... .......... .......... A......... ....A..... ...G...... ..........
ECOR_35       .......... .......... T......... .......... .......... .......... ..........
ECOR_36       .......... .......... T......... .......... .......... .......... ..........
SMS-3-5       .......... .......... T......... .......... .......... .......... ..........
536           .......... .......... .......... A......... ....A..... ...G...... ..........
APEC_O1       .......... .......... .......... A......... ....A..... ...G...... ..........
CFT073        .......... .......... .......... A......... ....A..... ...G...... ..........
E2348/69      .......... .......... .......... A........A ....A..... ...G...... ..........
ECOR_51       .......... .......... .......... A......... ....A..... ...G...... ..........
ECOR_57       .......... .......... .......... A......... ....A..... ...G...... ..........
UTI89         .......... .......... .......... A......... ....A..... ...G...... ..........
E._albertii   ...T....T. .T.....A.. .......... A......... .C..C..G.. ...G..C..G ..C..CG...

        10361
K-12          ATACCGCAAA AGCGCTGGCG GCAGCAATTG AAAAGATCGG CTTCGATTTA CTGATCTTTG GTGAAGGTTC
53638         .......... .......... ......G... .......... .......... .......... ..........
ATCC_8739     .......... .......... ......G... .......... .......... .......... ..........
ECOR_10       .......... .......... .......... .......... .......... .......... ..........
ECOR_11       .......... .......... .......... .......... .......... .......... ..........
B171          .......... .........A ......G... .......... .......... .......... ..........
E24377A       .......... .......... .......... .......... .......... .......... ..........
ECOR_34       .......... .......... ......G... .......... .......... .......... ..........
ECOR_71       .......... .......... .......... .......... .......... .......... ..........
SE11          .......... .......... ......G... .......... .......... .......... ..........
O157_Sakai    .......... .......... ......G... .......... .......... .......... ..A.......
ECOR_35       .......... G......... ......G... .......... .......... .......... ..........
ECOR_36       .......... G......... ......G... .......... .......... .......... ..........
SMS-3-5       .......... G......... ......G... .......... .......... .......... ..........
536           .......... G......... A.....G... .......... .......... .......... ..........
APEC_O1       .......... G......... A.....G... .......... .......... .......... ..........
CFT073        .......... G......... A.....G... .......... .......... .......... ..........
E2348/69      .......... G......... A.....G... .......... .......... .......... ..........
ECOR_51       .......... .......... A......... .......... .......... .......... ..........
ECOR_57       .......... G......... A.....G... .......... .......... .......... ..........
UTI89         .......... G......... A.....G... .......... .......... .......... ..........
E._albertii   .......... G..A.....A ..G...GCA. .......... T......... .......... ..........

        10431
K-12          CGGCGACCTT TATGCCCAGC AGGTTGGCTT GCTGGTCGGA GAAATTCTGC AACTTCCGGT GATTAATGCA
53638         .......... .......... ...C...... .......... .......... .......... ..........
ATCC_8739     .......... .......... .......... .......... .......... .......... ..........
ECOR_10       .......... .......... .......... .......... .......... .......... ..........
ECOR_11       .......... .......... .......... .......... .......... .......... ..........
B171          .......... .......... ...C...... .......... .......... .......... ..........
E24377A       .......... .......... .......... .......... .......... .......... ..........
ECOR_34       .......... .......... ...C...... .......... .......... .......... ..........
ECOR_71       .......... .......... .......... .......... .......... .......... ..........
SE11          .......... .......... .......... .......... .......... .......... ..........
O157_Sakai    .......... .......... .......... .......... .......... .......... ..........
ECOR_35       .......... .......... .......... .......... .......... .......... ..........
ECOR_36       .......... .......... .......... .......... .......... .......... ..........
SMS-3-5       .......... .......... .......... .......... .......... .......... ..........
536           .......... .......... .......... .......... .......... .......... ..........
APEC_O1       .......... .......... .......... .......... ...C...... .......... ..........
CFT073        .......... .......... .......... .......... ...C...... .......... ..........
E2348/69      .......... .......... .......... .......... .......... .......... ..........
ECOR_51       .......... .......... .......... .......... .......... .......... ..........
ECOR_57       .......... .......... .......... .......... ...C...... .......... ..........
UTI89         .......... .......... .......... .......... ...C...... .......... ..........
E._albertii   ......T..C .......... ....C...C. ......G... .....GT... ....C..T.. ...C.....G
```

```
            10501
K-12         GTGAGTGCTA TTCAGCGTCA GGGCAATACA CTGGTGATTG AACGCACGCT TGAAGATGAT GTTGAAGTTA
53638        .......... .......... .......... .......... .......... .......... ..........
ATCC_8739    .......... .......... .......... .......... .......... .........C ..........
ECOR_10      .......... .......... .......... .......... .......... .......... ..........
ECOR_11      .......... .......... .......... .......... .......... .......... ..........
B171         .......... .......... .......... .......... .......... .......... ..........
E24377A      .......... .......... .......... .......... .......... .......... ..........
ECOR_34      .......... .......... .......... .......... .......... .......... ..........
ECOR_71      .......... .......... .......... .......... .......... .......... ..........
SE11         .......... .......... .......... .......... .......... .......... ..........
O157_Sakai   .......... .......... .......... .......... .......... .........C ..........
ECOR_35      .......... .......... .......... .......... .......... .......... ..........
ECOR_36      .......... .......... .......... .......... .......... .......... ..........
SMS-3-5      .......... .......... .......... .......... .......... .......... ..........
536          ........C. .......... .......... .......... .......... .......... ..........
APEC_O1      .......... .......... .......... .......... .......... .......... ..........
CFT073       .......... .......... .......... .......... .......... .......... ..........
E2348/69     ........C. .......... .......... .......... .......... .......... ..........
ECOR_51      .......... .......... .......... .......... .......... .......... ..........
ECOR_57      .......... .......... .......... .......... .......... .......... ..........
UTI89        .......... .......... .......... .......... .......... .......... ..........
E._albertii  .......... .......... ...G.....G ......G... .......T.. .........A ........G.

            10571
K-12         TTGAACTCTC TGTTCCAGCC GTGCTCTGCG TCACCTCCGA TATTAACGTG CCACGCATTC CTTCGATGAA
53638        .......... .......... .......... .......... .......... .......... ..........
ATCC_8739    .......... .......... .......... .......... .......... .......... ..........
ECOR_10      .......... .......... .......... .......... .......... .......... ..........
ECOR_11      .......... .......... .......... .......... .......... .......... ..........
B171         .......... .......... .......... .......... .......... .......... ..........
E24377A      .......... .......... .......... .......... .......... .......... ..........
ECOR_34      .......... .......... .......... .......... .......... .......... ..........
ECOR_71      .......... .......... .......... .......... .......... .......... ..........
SE11         .......... .......... .......... .......... .......... .......... ..........
O157_Sakai   .......... .......... .......... .......... .......... .......... ..........
ECOR_35      ....G..... .......... .......... .......... .......A.. .......... ..........
ECOR_36      ....G..... .......... .......... .......... .......A.. .......... ..........
SMS-3-5      ....G..... .......... .......... .......... .......A.. .......... ..........
536          .......... .......... .......... .......... .......... .....T.... ..........
APEC_O1      .......... .......... .......... .......... .........A .....T.... ..........
CFT073       .......... .......... .......... .......... .........A .....T.... ..........
E2348/69     .......... .......... .......... .......... .........A .....T.... ..........
ECOR_51      .......... .......... .......... .......... .........A .......... ..........
ECOR_57      .......... .......... .......... .......... .........A .....T.... ..........
UTI89        .......... .......... .......... .......... .........A .....T.... ..........
E._albertii  .......... ......T... .......... ....A..A.. .......... .....T.... .A........

            10641
K-12         AGCCATTCTC GGCGCGGGTA AAAAACCGGT AAATCAGTGG CAGGCAAGTG ATATTGACTG GAGCCAGAGC
53638        .........T .......... .......... .......... .......... ......G... ..........
ATCC_8739    .......... .......... .......... .......... .......... ......G... ..........
ECOR_10      .......... .......... .......... .......... .......... .......... ..........
ECOR_11      .......... .......... .......... .......... .......... .......... ..........
B171         .......... .......... .......... .......... .......... G......... ..........
E24377A      .......... .......... .......... .......... .......... .......... ..........
ECOR_34      .......... .......... .......... .......... .......... .......... ..........
ECOR_71      .......... .......... .......... .......... .......... .......... ..........
SE11         .......... .......... .......... .......... .......... .......... ..........
O157_Sakai   .......... .......... .......... .......... .......... ......G... ..........
ECOR_35      .......... .......... .......... .......... .......... .C....G... ..........
ECOR_36      .......... .......... .......... .......... .......... .C....G... ..........
SMS-3-5      .......... .......... .......... .......... .......... .C....G... ..........
536          .......... .......... .......... .......... .......... ......G... ..........
APEC_O1      ......C... .......... .......... .......... .......... ......G... ..........
CFT073       ......C... .......... .......... .......... .......... ......G... ..........
E2348/69     ......C... .......... .......... .......... .......... ......G... ..........
ECOR_51      .......... .......... .......... .......... .......... ......G... ..........
ECOR_57      ......C... .......... .......... .......... .......... ......G... ..........
UTI89        ......C... .......... .......... .......... .......... ......G... ..........
E._albertii  .......... .......... .......... G..C...... .....C.... ......G... ..........
```

```
        10711
K-12         GCGCCACTTG CTGAACTGGT CGGCATTCGC GTACCGCCGC AAACAGAACG TAAGCACATC ATTATCGATA
53638        .......... .......... .......... .......... .......... .......... ...C......
ATCC_8739    ..A....... ........AC .......... .......... .......... .......... ...C......
ECOR_10      .......... .......... .......... .......... .......... .......... ..........
ECOR_11      .......... .......... .......... .......... .......... .......... ..........
B171         .......... .......... .......... .......... .......... .......... ..........
E24377A      .......... .......... .......... .......... .......... .......... ..........
ECOR_34      .......... .......... .......... .......... .......... .......... ..........
ECOR_71      .......... .......... .......... .......... .......... .......... ..........
SE11         .......... .......... .......... .......... .......... .......... ..........
O157_Sakai   ..A....... ........AC .......... .......... .......... .......... ...C......
ECOR_35      .......... ........AC .......... .......... .......... .......... ...C......
ECOR_36      .......... ........AC .......... .......... .......... .......... ...C......
SMS-3-5      ..A....... ........AC ...T...... .......... .......... .......... ...C......
536          ..A....... ........AC .......... .......... .......... .......... ...C......
APEC_O1      ..A....... ........AC ...T...... .......... .......... .......... ...C......
CFT073       ..A....... ........AC ...T...... .......... .....A.... .......... ...C......
E2348/69     ..A....... ........AC ...T...... .......... .......... .......... ...C......
ECOR_51      ..A....... ........AC ...T...... .......... .....A.... .......... ...C......
ECOR_57      ..A....... ........AC ...T...... .......... .....A.... .......... ...C......
UTI89        ..A....... ........AC ...T...... .......... .......... .......... ...C......
E._albertii  .......... .C...T.AAC .......... .......... ....C..... C..A...... ...T....A.

        10781
K-12         ACGATTCGCC GGAGGCCATT GCCGAGCTGG CGGAACATCT GAAGAAAGCC CTGAACTGAA GCCTACGGAG
53638        .......... .......... .......... .......... .......... .......... ..........
ATCC_8739    .......... .......... .......... .......... .......... .......... ..........
ECOR_10      .......... .......... .......... .......... .......... .......... ..........
ECOR_11      .......... .......... .......... .......... .......... .......... ..........
B171         .......... .......... .......... .......... .......... .......... ..........
E24377A      .......... .......... .......... .......... .......... .......... ..........
ECOR_34      .......... .......... .......... .......... .......... .......... ..........
ECOR_71      .......... .......... .......... .......... .......... .......... ..........
SE11         .......... .......... .......... .......... .......... .......... ..........
O157_Sakai   .......... .......... .......... .......... .......... .......... ..........
ECOR_35      .......... .......... .......... T......... .......... .......... ..........
ECOR_36      .......... .......... .......... T......... .......... .......... ..........
SMS-3-5      .......... .......... .......... .......... .......... .......... .T........
536          .......... .......... .......... .......... .......... .......... ..........
APEC_O1      .......... .......... .......... .......... ...A...... .......... ..........
CFT073       .......... .......... .......... .......... ...A...... .......... ..........
E2348/69     .......... .......... .......... .......... ...A...... .......... ..........
ECOR_51      .......... .......... .......... .......... ...A...... .......... ..........
ECOR_57      .......... .......... .......... .......... ...A...... .......... ..........
UTI89        .......... .......... .......... .......... ...A...... .......... ..........
E._albertii  .......... ...A...... .......... .......... ...A...... .......A.. ...A......

        10851
K-12         AAGAAACGAT GAGTCAATTA AACAGCGTCT GGGTCTTTAG CGATAATCCT GAACGTTATG CTGAACTGTT
53638        .......... .......... .......... .......... .......... .......... .......A..
ATCC_8739    .......... .......... .......... .......... .......... .......... ..........
ECOR_10      .......... .......... .......... .......... .......... .......... ..........
ECOR_11      .......... .......... .......... .......... .......... .......... ..........
B171         .......... .......... .......... .......... .......... .......... ..........
E24377A      .......... .......... .......... .......... .......... .......... ..........
ECOR_34      .......... .......... .......... .......... .......... .......... ..........
ECOR_71      .......... .......... .......... .......... .......... .......... ..........
SE11         .......... .......... .......... .......... .......... .......... ..........
O157_Sakai   .......... .......... .......... .......... .......... .......... ..........
ECOR_35      .......... .......... .......... .......... .......... .......... ..........
ECOR_36      .......... .......... .......... .......... .......... .......... ..........
SMS-3-5      .......... .......... .......... .......... .......... .......... ..........
536          .......... .......... .......... .......... .......... .......... ..........
APEC_O1      .......... .......... .......... .......... .......... ....A..... ..........
CFT073       .......... .......... .......... .......... .......... .......... ..........
E2348/69     .......... .......... .......... .......... .......... .......... ..........
ECOR_51      .......... .......... .......... .......... .......... .......... ..........
ECOR_57      .......... .......... .......... .......... .......... .......... ..........
UTI89        .......... .......... .......... .......... .......... ....A..... ..........
E._albertii  .TT.C..A.. ...CA..... .C..A..... .......... ......C... .......... .C........
```

```
        10921
K-12         TGGCGGCGCT CAGCAATGGG GCCAACAGGT GTATGCCATT GTACAAAATA CCGACCAGGC GCAGGCAGTT
53638        .......... .......... .......... .......... .......... .......... ..........
ATCC_8739    .......... .......... .......... .......... .......... .......... ..........
ECOR_10      .......... .......... .......... .......... .......... .......... ..........
ECOR_11      .......... .......... .......... .......... .......... .......... ..........
B171         .......... .......... .......... .......... .......... .......... ..........
E24377A      .......... .......... .......... .......... .......... .......... ..........
ECOR_34      .......... .......... .......... .......... .......... .......... ..........
ECOR_71      .......... .......... .......... .......... .......... .......... ..........
SE11         .......... .......... .......... .......... .......... .......... ..........
O157_Sakai   .......... .......... .......... .......... .......... .......... ..........
ECOR_35      .......... .......... .......... .......... .......... .......... ....A.....
ECOR_36      .......... .......... .......... .......... .......... .......... ....A.....
SMS-3-5      .......... .......... .......... .......... .......... ....G..... ..........
536          .......... .......... .......... .......... .......... ....G..... ..........
APEC_O1      .......... .......... .......... .......... .......... ....G..... ......G...
CFT073       .......... .......... .......... .......... .......... ....G..... ......G...
E2348/69     .......... .......... .......... .......... .......... .......... ....A.....
ECOR_51      .......... .......... .......... .......... .......... ....G..... ......G...
ECOR_57      .......... .......... .......... .......... .......... ....G..... ......G...
UTI89        .......... .......... .......... .......... .......... ....G..... ......G...
E._albertii  ......T..G ..A....... .T........ A......... .........G .......... ......G...

        10991
K-12         ATGCCTTATG GTCCAAAATG TCTTTATGTT CTTGCGCAAA ACGACGCGCT GCAACGCACT GAAAATTACG
53638        .......... .......... .A........ .......... .......... .......... ..........
ATCC_8739    .......... .......... .A........ ....A..... .......... .......... ..........
ECOR_10      .......... .......... .......... .......... .......... .......... ..........
ECOR_11      .......... .......... .......... .......... .......... .......... ..........
B171         .......... .......... .A........ ....A..... .......... .......... ..........
E24377A      .......... .......... .A........ ....A..... .......... .......... ..........
ECOR_34      .......... .......... .A........ ....A..... .......... .......... ..........
ECOR_71      .......... .......... .A........ ....A..... .......... .......... ..........
SE11         .......... .......... .A........ ....A..... .......... .......... ..........
O157_Sakai   .......... .......... .A........ ....A..... .......... .......... ..........
ECOR_35      .......... .G........ CA........ ....A..... .......... ......T... ..........
ECOR_36      .......... .G........ CA........ ....A..... .......... ......T... ..........
SMS-3-5      .......... .G........ CA........ ....A..... .......... ......T... ..........
536          .......... .G........ CA........ ....A..... .......... ......T... ..........
APEC_O1      .......... .G........ CA........ ....A..... .......... ......T... ..........
CFT073       .......... .G........ CA........ ....A..... .......... ......T... ..........
E2348/69     .......... .G........ CA........ ....A..... .......... ......T... ..........
ECOR_51      .......... .G........ CA........ ....A..... .......... ......T... ..........
ECOR_57      .......... .G........ CA........ ....A..... .......... ......T... ..........
UTI89        .......... .G........ CA........ ....A..... .......... ......T... ..........
E._albertii  ....A..... ....TCTCG. CA.......G ..G.A..... .TTCAT.A.. ........T. ..........

        11061
K-12         CCGAAAGCAT TGCTGCCCTG CTGAAAGATA AACACCCCGC TATGTTGCTG TTGGCCGCGA CGAAACGTGG
53638        .......... .......... .......... .......... .......... .......... ..........
ATCC_8739    .......... ......T... .......... .......... .........A C......... ..........
ECOR_10      .......... .......... .......... .......... .......... .......... ..........
ECOR_11      .......... .......... .......... .......... .......... .......... ..........
B171         .......... .......... .......... .......... .......... .......... ..........
E24377A      .......... .......... .......... .......... .......... .......... ..........
ECOR_34      .......... .......... .......... .......... .......... .......... ..........
ECOR_71      .......... .......... .......... .......... .......... .......... ..........
SE11         .......... .......... .......... .......... .......... .......... ..........
O157_Sakai   .......... ......T... .......... .......... .........A C......... ..........
ECOR_35      .......... .......... .......... .......TT. .......... C......... ..........
ECOR_36      .......... .......... .......... .......TT. .......... C......... ..........
SMS-3-5      .......... .......... .......... .......... .......... .......... ..........
536          .......... .......... .......... .......... .......... .......... ..........
APEC_O1      .......... .......... .......... .......... .......... .......... ..........
CFT073       .......... .......... .......... .......... .......... .......... ..........
E2348/69     .......... .......... .......... .......... .........A .......... ..........
ECOR_51      .......... .......... .......... .......... .......... .......... ..........
ECOR_57      .......... .......... .......... .......... .......... .......... ..........
UTI89        .......... .......... .......... .......... .......... .......... ..........
E._albertii  .T........ C..CT..... ..T....... ..GC....T. A.....A..C A.AT....C. .T.....C..
```

```
        11131
K-12         TAAAGCGCTG GCAGCACGGT TAAGTGTGCA ACTGAATGCG GCGCTGGTGA ACGATGCCAC GGCGGTGGAT
53638        .......... .......... .......... .......... .......... .......... ..........
ATCC_8739    ......T... .......... .......... .......... .......... ........G. ..........
ECOR_10      .......... .......... .......... .......... .......... .......... ..........
ECOR_11      .......... .......... .......... .......... .......... .......... ..........
B171         ......T... .......... .......... .......... .......... .......... ..........
E24377A      ......T... .......... .......... .......... .......... .......... ..........
ECOR_34      ......T... .......... .......... .......... .......... .......... ..........
ECOR_71      ......T... .......... .......... .......... .......... .......... ..........
SE11         ......T... .......... .......... .......... .......... .......... ..........
O157_Sakai   .......... .......... .......... .......... .......... .......... ..........
ECOR_35      ......T... .......... .......... .......... .......... .......... T.........
ECOR_36      ......T... .......... .......... .......... .......... .......... T.........
SMS-3-5      ......T... .......... .......... .......... .......... .......... ..........
536          ......T... .......... .......... .......... .......... .......... ..........
APEC_O1      ......T... ..C....... .......... .......... .......... .......... ..........
CFT073       ......T... ..C....... .......... .......... .......... .......... ..........
E2348/69     ......T... .......... .......... .......... .......... .......... ..........
ECOR_51      ......T... ..C....... .......... .......... .......... .......... ..........
ECOR_57      ......T... ..C....... .......... .......... .......... .......... ..........
UTI89        ......T... ..C....... .......... .......... .......... .......... ..........
E._albertii  ......T... ..G..G.... .G..C..... .........A ..C.....A. .......T.. A.AA..T..C

        11201
K-12         ATTGTCGATG GTCACATTTG CGCCGAACAC CGGATGTATG GCGGGTTAGC GTTCGCTCAG GAAAAGATCA
53638        .......... .......... .......... .......... .......... .......... ..........
ATCC_8739    .......... .......... .......... .......... .......... .......... ..........
ECOR_10      .......... .......... .......... .......... .......... .......... ..........
ECOR_11      .......... .......... .......... .......... .......... .......... ..........
B171         ...A...... .......... .......... .......... .......... .......... ..........
E24377A      .......... .......... .......... .......... .......... .......... ..........
ECOR_34      ...A...... .......... .......... .......... .......... .......... ..........
ECOR_71      .......... .......... .......... .......... .......... .......... ..........
SE11         .......... .......... .......... .......... .......... .......... ..........
O157_Sakai   .......... .......... .......... .......... .......... .......... ..........
ECOR_35      .......... .......... .......... .......... .......... .......... ..........
ECOR_36      .......... .......... .......... .......... .......... .......... ..........
SMS-3-5      .......... .......... .......... .......... .......... ......A... ..........
536          .......... .......... .......... .......... .......... .......... ..........
APEC_O1      .......... .......... .......... .......... .......... .......... ..........
CFT073       .......... .......... .......... .......... .......... .......... ..........
E2348/69     .......... .......... .......... .......... .......... .......... ..........
ECOR_51      .......... .......... .......... .......... .......... .......... ..........
ECOR_57      .......... .......... .......... .......... .......... .......... ..........
UTI89        .......... .......... .......... .......... .......... .......... ..........
E._albertii  G.G..T...A AC...G.... T..T...... ..T....... ....AC.G.. A..T..C... .....A....

        11271
K-12         ACAGCCCGCT GGCGATCATT ACCCTTGCAC CCGGTGTTCA GGAACCGTGC ACCAGTGATA CCTCTCATCA
53638        .....A.... A......... .......... .......... .......... .......... ..........
ATCC_8739    .....T.... .......... .......... .......... .......... G......... A.........
ECOR_10      .......... .......... .......... .......... .......... .......... ..........
ECOR_11      .......... .......... .......... .......... .......... .......... ..........
B171         .......... .......... .......... .......... .......... .......... ..........
E24377A      .......... .......... .......... .......... .......... .......... ..........
ECOR_34      .......... .......... .......... .......... .......... .......... ..........
ECOR_71      .......... .......... .......... .......... .......... .......... ..........
SE11         .......... .......... .......... .......... .......... .......... ..........
O157_Sakai   .....T.... .......... .......... .......... .......... .......... ..........
ECOR_35      .......... ...A...... .......... .......... .......... .......... T.........
ECOR_36      .......... ...A...... .......... .......... .......... .......... T.........
SMS-3-5      .......... .......... .......... .......... .......... ...C...... ..........
536          .......... .......... .......... .......... ...G...... .......... T.........
APEC_O1      .......... .......... .......... .......... ...G...... ...C...... T.........
CFT073       .......... .......... .......... .......... ...G...... ...C...... T.........
E2348/69     .......... .......... G.......G. .......... .......... ...C...... T.........
ECOR_51      .......... .......... .......... .......... ...G...... ...C...... T.........
ECOR_57      .......... .......... .......... .......... ...G...... ...C...... T.........
UTI89        .......... .......... .......... .......... ...G...... ...C...... T.........
E._albertii  ....T..AT. ...A..A..C .......... .......C.. ......A... G..CA...CG ....C.....
```

```
         11341
K-12         GTGCCCGACA GAAACGGTAC CTTATGTTGC TCCGCGTCAT GAAATTCTCT GTCGCGAACG CCGTGCGAAA
53638        .......... .......... .......... .......... .......... .......... ..........
ATCC_8739    .......... .......... .......... .......... .......... .......G.. ..........
ECOR_10      .......... .......... .......... .......... .......... .......... ..........
ECOR_11      .......... .......... .......... .......... .......... .......... ..........
B171         .......... .......... .......... .......... .......... .......... ..........
E24377A      .......... .......... .......... .......... .......... .......... ..........
ECOR_34      .......... .......... .......... ...A...... .......... .......... ..........
ECOR_71      .......... .......... .......... .......... .......... .......... ..........
SE11         .......... .......... .......... .......... .......... .......... ..........
O157_Sakai   .......... .......... ....C..... .......... .......... .......... ..........
ECOR_35      .......... .......... .......... C......... .......... .......... ..........
ECOR_36      .......... .......... .......... C......... .......... .......... ..........
SMS-3-5      .......... .......... .......... C......... .......... .......... ..........
536          .......... .......... .......... C......... .......... .......... ..........
APEC_O1      .......... .......... .......... C......... .......... .......... ..........
CFT073       .......... .......... .......... C......... .......... .......... ..........
E2348/69     .......... .......... .......... C......... .......... .......... ..........
ECOR_51      .......... .......... .......... C......... .......... .......... ..........
ECOR_57      .......... .......... .......... C......... .......... .......... ..........
UTI89        .......... .......... .......... C......... .......... .......... ..........
E._albertii  ...T....TT ...G.T..G. ....C..... C......... .......... .C........ ..........

         11411
K-12         GCCGCAAGCA GCGTGGACCT GAGCAAAGCA AAACGTGTGG TTGGCGTCGG TCGTGGACTG GCGGCGCAGG
53638        .......... .......... .......... .......... .......... ......T... ..........
ATCC_8739    ..G....... .......... .......... .......... .......... ......T... ..A.......
ECOR_10      .......... .......... .......... .......... .......... .......... ..........
ECOR_11      .......... .......... .......... .......... .......... .......... ..........
B171         .......... .......... .......... .......... .......... ......T... ..........
E24377A      .......... .......... .......... .......... .......... ......T... ..........
ECOR_34      .......... .......... .......... .......... .......... ......T... ..........
ECOR_71      .......... .......... .......... .......... .......... ......T..T ..........
SE11         .......... .......... .......... .......... .......... ......T... ..........
O157_Sakai   .......... .......... .......... .......... .......... ......T... ..A.......
ECOR_35      ..G....... .......... .......... .......... .......... ......T... ..........
ECOR_36      ..G....... .......... .......... .......... .......... ......T... ..........
SMS-3-5      ..G....... .......... .......... .......... .......... ......T... ..........
536          ..G....... .......... .......... .......... .......... ......T... ..........
APEC_O1      ..G....... .......... .......... .......... .......... ......T... ..........
CFT073       ..G....... .......... .......... .......... .......... ......T... ..........
E2348/69     ..G....... .......... .......... .......... .......... ......T... ..........
ECOR_51      ..G....... .......... .......... .......... .......... ......T... ..........
ECOR_57      ..G....... .......... .......... .......... .......... ......T... ..........
UTI89        ..G....... .......... .......... .......... .......... ......T... ..........
E._albertii  ..G....... .......... ...T...... .....C.... .C..T..... ......T... ..A..A....

         11481
K-12         ATGACCTAAA AATGGTCCAC GAACTGGCGG CGGTGCTGAA TGCTGAAGTC GGCTGTTCAC GTCCAATTGC
53638        .......G.. .......... .......... .......... .......... .......... ..........
ATCC_8739    .......G.. .......... .......... .......... .......... .......... ..........
ECOR_10      .......... .......... .......... .......... .......... .......... ..........
ECOR_11      .......... .......... .......... .......... .......... .......... ..........
B171         .......G.. .......... .......... .......... .......... .......... ..........
E24377A      .......G.. .......... .......... .......... .......... .......... ..........
ECOR_34      .......G.. .......... .......... .......... .......... .......... ..........
ECOR_71      .......G.. .......... .......... .......... .......... .......... ..........
SE11         .......G.. .......... .......... .......... .......... .......... ..........
O157_Sakai   .......G.. .......... .......... .......... .......... .......... ..........
ECOR_35      .......G.. .......... .......... .A........ .......... .......... ..........
ECOR_36      .......G.. .......... .......... .A........ .......... .......... ..........
SMS-3-5      .......G.. .......... .......... .A........ .......... .......... ..........
536          .......G.. .......... .......... ....A..... .......... .......... ..........
APEC_O1      .......G.. .......... .......... .A........ .......... .......... ..........
CFT073       .......G.. .......... .......... .A........ .......... .......... ..........
E2348/69     .......G.. .......... .......... .A........ .........T .......... ..........
ECOR_51      .......G.. .......... .......... .A........ .......... .......... ..........
ECOR_57      .......G.. .......... .......... .A........ .......... .......... ..........
UTI89        .......G.. .......... .......... .A........ .......... .......... ..........
E._albertii  ....T..G.. .......... ........A. ...C...... .......... .......... ..........
```

```
           11551
K-12         CGAAGGCGAG AACTGGATGG AGCGTGAACG TTATATCGGT GTCTCCGGCG TGTTGCTGAA ATCCGATCTC
53638        .......... .......... .......... .......... .......... .......... ..........
ATCC_8739    .......... .......... .......... .......... .......... .......... ..........
ECOR_10      .......... .......... .......... .......... .......... .......... ..........
ECOR_11      .......... .......... .......... .......... .......... .......... ..........
B171         .......... .......... .......... .......... .......... .......... ..........
E24377A      .......... .......... .......... .......... .......... .......... ..........
ECOR_34      .......... .......... .......... .......... .......... .......... ..........
ECOR_71      .......... .......... .......... .......... .......... .......... ...T......
SE11         .......... .......... .......... .......... .......... .......... ..........
O157_Sakai   .......... .......... .......... .......... .......... .......... ..........
ECOR_35      .......... .......... .......... .......... .......... .......... ..........
ECOR_36      .......... .......... .......... .......... .......... .......... ..........
SMS-3-5      .......... .......... .......... .......... .......... .......... ..........
536          .......... .......... .......... .......... .......... .......... ..........
APEC_O1      .......... .......... .......... .......... .......... .......... ..........
CFT073       .......... .......... .......... .......... .......... .......... ..........
E2348/69     .......... .......... .......... .......... ........T. .......... ..........
ECOR_51      .......... .......... .......... .......... .......... .......... ..........
ECOR_57      .......... .......... .......... .......... .......... .......... ..........
UTI89        .......... .......... .......... .......... .......... .......... ..........
E._albertii  T......... .......... ...AC..... C..C..T... ..T....... ..C....... ..........

           11621
K-12         TACCTGACGC TGGGGATCTC CGGGCAGATC CAGCATATGG TTGGCGGCAA CGGCGCAAAA GTGATTGTCG
53638        .......... .......... .......... .......... .......... .......... ..........
ATCC_8739    .......... .......... .......... ..A....... .......... .......... ..........
ECOR_10      .......... .......... .......... .......... .......... .......... ..........
ECOR_11      .......... .......... .......... .......... .......... .......... ..........
B171         .......... .......... .......... .......... .......... .......... ..........
E24377A      .......... .......... .......... .......... .......... .......... .........A
ECOR_34      .......... .......... .......... .......... .......... .......... ..........
ECOR_71      .......... .......... .......... ..A....... .......... .......... ..........
SE11         .......... .......... .......... .......... .......... .......... ..........
O157_Sakai   .......... .A........ .......... .......... .......... .......... ..........
ECOR_35      .......... .......... .......... ..A....... .......... .......... ..........
ECOR_36      .......... .......... .......... ..A....... .......... .......... ..........
SMS-3-5      .......... .......... .......... .......... .......... .......... ..........
536          .......... .......... .......... .......... .......... .......... ..........
APEC_O1      .......... .......... .......... .......... .......... .......... ..........
CFT073       .......... .......... .......... .......... .......... .......... ..........
E2348/69     .......... .......... .......... .......... .......... .......... ..........
ECOR_51      .......... .......... .......... .......... .......... .......... ..........
ECOR_57      .......... .......... .......... .......... .......... .......... ..........
UTI89        .......... .......... .......... .......... .......... .......... ..........
E._albertii  .........T .......T.. .......... .......... .C........ ......G... ........T.

           11691
K-12         CCATCAATAA AGATAAAAAT GCGCCAATCT TCAACTATGC CGACTACGGT CTGGTGGGCG ATATCTACAA
53638        .......... .......... .......... .......... .......... .......... ..........
ATCC_8739    .......... .......... .......... .......... .......... .......... ..........
ECOR_10      .......... .......... .......... .......... .......... .......... ..........
ECOR_11      .......... .......... .......... .......... .......... .......... ..........
B171         .......... .......... .......... ....T..... .......... .......... ..........
E24377A      .......... .......... .......... ....T..... .......... .......... ..........
ECOR_34      .......... .......... .......... ....T..... .......... .......... ..........
ECOR_71      .......... .......... .......... .......... .......... .......... ..........
SE11         .......... .......... .......... ....T..... .......... .......... ..........
O157_Sakai   .......... .......... .......... .......... .......... .......... ..........
ECOR_35      .......... .......... .......... .......... .......... .......... ..........
ECOR_36      .......... .......... .......... .......... .......... .......... ..........
SMS-3-5      .......... .......... .......... .......... .......... .......... ..........
536          ....T..... .......... .......... .......... .......... .......... ..........
APEC_O1      ....T..... .......... .......... .......... .......... .......... ..........
CFT073       ....T..... .......... .......... .......... .......... .......... ..........
E2348/69     ....T..... .......... .......... .......... .......... .......... ..........
ECOR_51      ....T..... .......... .......... .......... .......... .......... ..........
ECOR_57      ....T..... .......... .......... .......... .......... .......... ..........
UTI89        ....T..... .......... .......... .......... .......... .......... ..........
E._albertii  .......... .........C .......... .......... .......... .....T.... ..........
```

```
         11761
K-12          AGTCGTCCCT GCCCTGATTA GCCAGTTGAG CCGCTAATTC CCCTTTCAAC CACATCTCCC GCTGTAAACG
53638         .......... .......... .......... .......... .......... .......... ..........
ATCC_8739     .......... .......... .......... .......... .......... .......... ..........
ECOR_10       .......... .......... .......... .......... .......... .......... ..........
ECOR_11       .......... .......... .......... .......... .......... .......... ..........
B171          .......... .......... .......... .......... .......... .......... ..........
E24377A       .......... .......... .......... .......... .......... .......... ..........
ECOR_34       .......... .......... .......... .......... .......... .......... ..........
ECOR_71       .......... .......... .......... .......... .......... .......... ..........
SE11          .......... .......... .......... .......... .......... .......... ..........
O157_Sakai    .......... .......... .......... .......... .......... .......... ..........
ECOR_35       .......... .......... .......... .......... .......... .......... ..........
ECOR_36       .......... .......... .......... .......... .......... .......... ..........
SMS-3-5       .......... .......... .......... T......... ..-....... .......... ..........
536           .......... .......... .......... T......... ..-....... .......... ..........
APEC_O1       .......... .......... .........A T......... ..-....... .......... ..........
CFT073        .......... .......... .........A T......... ..-....... .......... ..........
E2348/69      .......... .......... .......... T......... ..-....... .......... ..........
ECOR_51       .......... .......... .........A T......... ..-....... .......... ..........
ECOR_57       .......... .......... .........A T......... ..-....... .......... ..........
UTI89         .......... .......... .........A T......... ..-....... .......... ..........
E._albertii   ...G...... .......... A.......G. ..A.....C. T.T....... .......... .....TT...

         11831
K-12          CAGCGGGAAG GGAGCATAAC GCATGTCGGA TGACAAATTT GATGCCATTG TGGTCGGTGC GGGCGTTGCT
53638         .......... .......... .......... .......... .......... .......... ..........
ATCC_8739     .......... .......... .......... .......... .......... .......... ..........
ECOR_10       .......... .......... .......... .......... .......... .......... ..........
ECOR_11       .......... .......... .......... .......... .......... .......... ..........
B171          .......... .......... .......... A......... .......... .......... ..........
E24377A       .......... .......... .......... .......... .......... .......... ..........
ECOR_34       .......... .......... .......... A......... .......... .......... ..........
ECOR_71       .......... .......... .......... .......... .......... .......... ..........
SE11          .......... .......... .......... A......... .......... .......... ..........
O157_Sakai    .......... .......... .......... .......... .......... .......... ..........
ECOR_35       .......... .......... .......... .......... .......... .......... .........C
ECOR_36       .......... .......... .......... .......... .......... .......... .........C
SMS-3-5       .......... .......... .......... .......... .......... .......... .........C
536           .......... .......... .......... .......... .......... .......... .........C
APEC_O1       .......... .......... .......... .......... .......... .......... .........C
CFT073        .......... .......... .......... .......... .......... .......... .........C
E2348/69      .......... .......... .......... .......... .......... .......... .........C
ECOR_51       .......... .......... .......... .......... .......... .......... .........C
ECOR_57       .......... .......... .......... .......... .......... .......... .........C
UTI89         .......... .......... .......... .......... .......... .......... .........C
E._albertii   .......... .......... .......... ...T...... .......... .......... ...T.....G

         11901
K-12          GGTAGCGTTG CCGCACTGGT CATGGCACGA GCCGGGCTGG ATGTCCTGGT GATAGAACGC GGCGACAGTG
53638         .......... .......... .......... ..A....... .......... .......... ..........
ATCC_8739     .......... .......... .......... .......... .......... .......... ..........
ECOR_10       .......... .......... .......... .......... .......... .......... ..........
ECOR_11       .......... .......... .......... .......... .......... .......... ..........
B171          .......... .......... ......G... .......... .......... .......... ..........
E24377A       .......... .......... ......G... .......... .......... .......... ..........
ECOR_34       .......... .......... ......G... .......... .......... .......... ..........
ECOR_71       .......... .......... .......... .......... .......... .......... ..........
SE11          .......... .......... ......G... .......... .......... .......... ..........
O157_Sakai    .......... .......... ......G... .......... ..A....... .......... ..........
ECOR_35       .......... .......... ......G... .......... .......... .......... ..........
ECOR_36       .......... .......... ......G... .......... .......... .......... ..........
SMS-3-5       .......... .......... ......G.A. .......... .......... .......... ..........
536           .......... .......... ......G... .......... .......... .......... ..........
APEC_O1       .......... .......... ......G... .......... .......... .......... ..........
CFT073        .......... .......... ......G... .......... .......... .......... ..........
E2348/69      .......... .......... ......G... .......... .......... .......... ..........
ECOR_51       .......... .......... ......G... .......... .......... .......... ..........
ECOR_57       .......... .......... ......G... .......... .......... .......... ..........
UTI89         .......... .......... ......G... .......... .......... .......... ..........
E._albertii   ....C..... .T........ ......G... .......... ....G..... A......... ........C.
```

```
         11971
K-12         CCGGATGTAA AAACATGACC GGCGGGCGTC TTTATGCCCA CACACTTGAA GCAATCATTC CAGGCTTTGC
53638        .......... .......... ........C. .......... .......... .......... ..........
ATCC_8739    .......... .......... ........C. .......... .......... .......... ..........
ECOR_10      .......... .......... .......... .......... .......... .......... ..........
ECOR_11      .......... .......... .......... .......... .......... .......... ..........
B171         .......... .......... .......... .......... .......... .......... ..........
E24377A      .......... .......... .......... .......... .......... .......... ..........
ECOR_34      .......... .......... .......... .......... .......... .......... ..........
ECOR_71      .......... .......... .......... .......... .......... .......... ..........
SE11         .......... .......... .......... .......... .......... .......... ..........
O157_Sakai   .......... .......... .......... .......... .......... .......... ..........
ECOR_35      .......... .......... .......... .......... .......... .......... ..........
ECOR_36      .......... .......... .......... .......... .......... .......... ..........
SMS-3-5      .......... .......... .......... .......... .......... ..G..T.... ..........
536          .......... .......... .......... .......... ......C... .....T.... ..........
APEC_O1      .......... .......... .......... .......... ......C... .....T.... ..........
CFT073       .......... .......... .......... .......... .......... .....T.... ..........
E2348/69     .......... .......... .......... .......... ......C... .....T.... ..........
ECOR_51      .......... .......... .......... .......... .......... .....T.... ..........
ECOR_57      .......... .......... .......... .......... .......... .....T.... ..........
UTI89        .......... .......... .......... .......... ......C... .....T.... ..........
E._albertii  .......C.. ...T...... ........A. .C........ .T.G...... .......... ..........

         12041
K-12         AGTATCAGCG CCGGTAGAAC GCAAGGTCAC ACGCGAGAAA ATCTCCTTCT TAACCGAAGA AAGCGCCGTT
53638        ..C....... .......... .......... .......... .......... .......... G.........
ATCC_8739    ..C....... .......... .......... .......... .......... .......... G.........
ECOR_10      .......... .......... .......... .......... .......... .......... ..........
ECOR_11      .......... .......... .......... .......... .......... .......... ..........
B171         .......... .......... .......... .......... .......... .......... ..........
E24377A      .......... .......... .......... .......... .......... .......... ..........
ECOR_34      .......... .......... .......... .......... .......... .......... ..........
ECOR_71      ..C....... .......... .......... .......... .......... .......... ..........
SE11         .......... .......... .......... .......... .......... .......... ..........
O157_Sakai   ..C....... .......... .......... .......... .......... .......... G.........
ECOR_35      ..C....... ..A....... .......... .......... .......... .....A.... G..T......
ECOR_36      ..C....... ..A....... .......... .......... .......... .....A.... G..T......
SMS-3-5      ..C....... .......... .......... .......... .......... .......T.. G..T......
536          ..C....... .......... .......... .......... .......... .......... ..........
APEC_O1      ..C....... ...A...... .......... .......... .......... .......... ..........
CFT073       ..C....... .......... .......... .......... .......... .......... ..........
E2348/69     ..C....... ...A...... .......... .......... .......... .......... ..........
ECOR_51      ..C....... .......... .......... .......... .......... .......... ..........
ECOR_57      ..C....... .......... .......... .......... .......... .......... ..........
UTI89        ..C....... ...A...... .......... .......... .......... .......... ..........
E._albertii  C.C......A ...A.T.... ..G.A..... G......... .......... .G..T..... GC.......A

         12111
K-12         ACCCTCGATT TTCACCGCGA GCAGCCAGAT GTTCCGCAAC ACGCATCTTA TACCGTATTG CGTAATCGTC
53638        .......... .......... ...A...... .......... .......... .......... ..........
ATCC_8739    .......... .......... ...A...... .......... .......... .......... ..........
ECOR_10      .......... .......... .......... .......... .......... .......... ..........
ECOR_11      .......... .......... .......... .......... .......... .......... ..........
B171         .......... .......... ...A...... .......... .......... .......... ..........
E24377A      .......... .......... ...A...... .......... .......... .......... ..........
ECOR_34      .......... .......... ...A...... .......... .......... .......... ..........
ECOR_71      .......... .......... .......... .......... .......... .......... ..........
SE11         .......... .......... ...A...... .......... .......... .......... ..........
O157_Sakai   .......... .......... ...A...... .......... .......... .......... ..........
ECOR_35      .......... .......... ...AT..... .......... ....G..... .......... ..........
ECOR_36      .......... .......... ...AT..... .......... ....G..... .......... ..........
SMS-3-5      .......... .......... ...A...... .......... ....G..... .......... ..........
536          .......... .......... ...A...... .......... ....G..... .......... ..........
APEC_O1      .......... .......... ...A...... .......... ....G..... .......... ..........
CFT073       .......... .......... ...A...... .......... ....G..... .......... ..........
E2348/69     .......... .......... ...A...... .......... ....G..... .......... ..........
ECOR_51      .......... .......... ...A...... .......... ....G..... .......... ..........
ECOR_57      .......... .......... ...A...... .......... ....G..... .......... ..........
UTI89        .......... .......... ...A...... .......... ....G..... .......... ..........
E._albertii  .......... .......T.. ...A..C... ...G...... ....G..... .......... ..C.......
```

```
         12181
K-12         TGGACCCGTG GTTGATGGAA CAAGCCGAGC AGGCGGGCGC GCAGTTTATC CCAGGCGTTC GCGTCGACGC
53638        .......... .......... ..T....... .......... A......... ..G..A.... .......T..
ATCC_8739    .......... .......... ..T....... .......... A......... ..G..A.... .......T..
ECOR_10      .......... .......... .......... .......... .......... .......... ..........
ECOR_11      .......... .......... .......... .......... .......... .......... ..........
B171         .......... .......... .......... .......... .......... .......... .......T..
E24377A      .......... .......... .......... .......... A......... ..G....... .......T..
ECOR_34      .......... .......... .......... .......... .......... .......... .......T..
ECOR_71      .......... .......... .......... .......... .......... .......... .......T..
SE11         .......... .......... .......... .......... .......... .......... .......T..
O157_Sakai   .......... .......... .......... .......... A......... ..G..A.... .......T..
ECOR_35      .......... .......... ........A. ....T..... .......... ..G....... .T.....T..
ECOR_36      .......... .......... ........A. ....T..... .......... ..G....... .T.....T..
SMS-3-5      .......... .......... ........A. ....T..... .......... ..G....... .T.....T..
536          .......... .......... .......... .......... .......... ..G.....A. .......T..
APEC_O1      .......... .......... .......... .......... .......... ..G.....A. .......T..
CFT073       .......... .......... .......... .......... .......... ..G.....A. .......T..
E2348/69     .......... .......... .......... .......... .......... ..G.....A. .......T..
ECOR_51      .......... .......... .......... .......... .......... ..G.....A. .......T..
ECOR_57      .......... .......... .......... .......... .......... ..G.....A. .......T..
UTI89        .......... .......... .......... .......... .......... ..G.....A. .......T..
E._albertii  .C........ ....G..... ........A. ....A..... A........T ..G....... ..........

         12251
K-12         GCTGGTTCGT GAAGGAAACA AGGTCACTGG CGTGCAGGCT GGGGATGATA TTCTCGAAGC GAATGTGGTG
53638        .......... .......... .......... ...C...... .......... ........A. ..........
ATCC_8739    .......... .......... .......... ...C...... .......... ........A. ..........
ECOR_10      .......... .......... .......... .......... .......... .......... ..........
ECOR_11      .......... .......... .......... .......... .......... .......... ..........
B171         .......... .......... .......... .......... .......... .......... ..........
E24377A      .T........ .......... .......... .........C .......... .....C.... ..........
ECOR_34      .......... .......... .......... .......... .......... .......... ..........
ECOR_71      .......... .......... ....A..... .......... .......... .......... ..........
SE11         .......... .......... .......... .......... .......... .......... ..........
O157_Sakai   .T........ .......... .......... A..C.....C .......... .......... C.........
ECOR_35      .......... .......... .......... ...T.....C .........G ....T..... ..........
ECOR_36      .......... .......... .......... ...T.....C .........G ....T..... ..........
SMS-3-5      .......... ........T. .......... ...C.....C .......... .......... ..........
536          .......... .......... .......... ...C.....C .......... .......... ..........
APEC_O1      .......... .......... .......... ...C.....C .......... .......... ..........
CFT073       .......... .......... .......... ...C.....C .......... .......... ..........
E2348/69     A......... .......... .......... ...C.....C .......... .......... ..........
ECOR_51      .......... .......... .......... ...C.....C .......... .......... ..........
ECOR_57      .......... .......... .......... ...C.....C .......... .......... ..........
UTI89        .......... .......... .......... ...C.....C .......... .......... ..........
E._albertii  .......... .....C.... .......A.. ...A.....C .......... .......... A........C

         12321
K-12         ATTCTGGCTG ATGGCGTTAA CTCGATGCTT GGCCGCTCGC TGGGAATGGT TCCCGCTTCC GATCCGCATC
53638        .......... .......... .......... .......... .......... .......... ..........
ATCC_8739    .......... .......... .......... .......... .......... .......... ..........
ECOR_10      .......... .......... .......... .......... .......... .......... ..........
ECOR_11      .......... .......... .......... .......... .......... .......... ..........
B171         .......... .......... .......... .......... .......... .......... ..........
E24377A      G....A.... .......... .......... .......... .......... .......... ..........
ECOR_34      .......... .......... .......... .......... .......... .......... ..........
ECOR_71      .......... .......... .......... .......... .......... .......... ..........
SE11         .......... .......... .......... .......... .......... .......... ..........
O157_Sakai   ...T.A..C. .......... .......... .......... .......... .......... ..........
ECOR_35      .......... .......... .......... .......... .......... .......... ..........
ECOR_36      .......... .......... .......... .......... .......... .......... ..........
SMS-3-5      .......... .......... .......... .......... .......... .......... ..........
536          .......... .......... .......... .......... .......... .T........ ..........
APEC_O1      .......... .......... .......... .......... .......... .T........ ..........
CFT073       .......... .......... .......... .......... .......... .T........ ..........
E2348/69     .......... .......... .......... .......... .......... .T........ ..........
ECOR_51      .......... .......... .......... .......... .......... .T........ ..........
ECOR_57      .......... .......... .......... .......... .......... .T........ ..........
UTI89        .......... .......... .......... .......... .......... .T........ ..........
E._albertii  .......... .......C.. ...A..A... ..T..T..T. .......... ......C... .....A....
```

```
        12391
K-12          ATTACGCTGT TGGTGTTAAA GAGGTTATTG GCCTCACACC AGAACAGATC AACGATCGCT TTAATATTAC
53638         .......... .......... .......... .......... .......... .......... ..........
ATCC_8739     .......... .......... .......... .......... .......... .......... ..........
ECOR_10       .......... .......... .......... .......... .......... .......... ..........
ECOR_11       .......... .......... .......... .......... .......... .......... ..........
B171          .......... .......... .......... .......... .......... .......... ..........
E24377A       .......... .......... .......... .......... .......... .......... ..........
ECOR_34       .......... .......... .......... .......... .......... .......... ..........
ECOR_71       .......... .......... .......... .......G.. .......... .......... ..........
SE11          .......... .......... .......... .......... .......... .......... ..........
O157_Sakai    .......... .......... .......... .......... .......... .......... ..........
ECOR_35       .......... .......... .......... .......... .......... .......... ..........
ECOR_36       .......... .......... .......... .......... .......... .......... ..........
SMS-3-5       .......... .......... .......... .......... .......... ..G....... ..........
536           .......... .......... ........A. ....G..... .......... .......... .....G....
APEC_O1       .......... .......... ........A. ....G..... .......... .......... .....G....
CFT073        .......... .......... ........A. .T..G..... .......... .......... .....G....
E2348/69      .......... .......... ........A. ....G..... .......... .......... .....G....
ECOR_51       .......... .......... ........A. .T..G..... .......... .......... .....G....
ECOR_57       .......... .......... ........A. .T..G..... .......... .......... .....G....
UTI89         .......... .......... ........A. ....G..... .......... .......... .....G....
E._albertii   .......CA. C..C...... ..A..C.... .T.....G.. .C.G..A... ........T. .....G....

        12461
K-12          GGGCGAGGAA GGTGCCGCCT GGCTGTTTGC CGGTTCCCCT TCTGACGGCC TGATGGGCGG GGGATTTCTC
53638         .......... .......... .......... .......... .......... .......T.. C.....C...
ATCC_8739     .......... .......... .......... .......... .......... .......T.. C.....C...
ECOR_10       .......... .......... .......... .......... .......... .......... ..........
ECOR_11       .......... .......... .......... .......... .......... .......... ..........
B171          .......... .......... .......... .......... .......... .......... ..........
E24377A       .......... .......... .......... .......... .......... .......... ..........
ECOR_34       .......... .......... .......... .......... .......... .......... ..........
ECOR_71       .......... .......... .......... .......... .......... .......T.. C.....C...
SE11          .......... .......... .......... .......... .......... .......... ..........
O157_Sakai    .......... .......... .......... .......... .......... .......... ..........
ECOR_35       .......... .......... .......... .......... ..G....... .......T.. C.........
ECOR_36       .......... .......... .......... .......... ..G....... .......T.. C.........
SMS-3-5       .......... .......... .......... .......... .......... .......... C.....C...
536           .......... .......... .......... .......... .......... .......... C.....C...
APEC_O1       .......... .......... .......... .......... .......... .......... C.....C...
CFT073        .......... .......... .......... .......... .......... .......... C.....C...
E2348/69      .......... .......... .......... .......... .......... .......... C.....C...
ECOR_51       .......... .......... .......... .......... .......... .......... C.....C...
ECOR_57       .......... .......... .......... .......... .......... .......... C.....C...
UTI89         .......... .......... .......... .......... .......... .......... C.....C...
E._albertii   ..A.A.T... .....T..A. .......... T.....G... .......... .......... T.........

        12531
K-12          TATACCAACA AGGATTCCAT ATCCTTGGGG CTGGTTTGTG GATTGGGTGA TATCGCCCAT GCGCAAAAAA
53638         ........T. .A........ ...G...... .......... .......... .......... ..........
ATCC_8739     ........T. .......... ...G...... .......... .......... .......... ..........
ECOR_10       .......... .......... .......... .......... .......... .......... ..........
ECOR_11       .......... .......... .......... .......... .......... .......... ..........
B171          .......... .......... .......... .......... .......... .......... ..........
E24377A       .......... .......... .......... .......... .......... .......... ..........
ECOR_34       .......... .......... .......... .......... .......... .......... A.........
ECOR_71       .......... .C......G. ...G...... .......... .......... .......... ..........
SE11          .......... .......... .......... .......... .......... .......... A.........
O157_Sakai    .......... .......... .......... .......... ......C... .......... ..........
ECOR_35       .......... ........G. ...G...... .......... .......... .......... ..........
ECOR_36       .......... ........G. ...G...... .......... .......... .......... ..........
SMS-3-5       ........T. .......... ...G...... .......... .......... .......... ..........
536           .......... ........G. ...G...... .......... .......... .......... ..........
APEC_O1       .......... ........G. ...G...... .......... .....A.... .......... ..........
CFT073        .......... .A......G. ...G...... .......... .....A.... .......... ..........
E2348/69      .......... ........G. ...G...... .......... .......... .......... ..........
ECOR_51       .......... .A......G. ...G...... .......... .....A.... .......... ..........
ECOR_57       .......... .A......G. ...G...... .......... .....A.... .......... ..........
UTI89         .......... ........G. ...G...... .......... .....A.... .......... ..........
E._albertii   ........TC GC..C...G. G..G.....T .......... .......C.. ......T... ..........
```

```
        12601
K-12          GCGTGCCGCA AATGCTGGAA GATTTTAAAC AACACCCCGC CATTCGCCCG CTGATTAGCG GCGGCAAACT
53638         .......... .......... .......... .......... .......... .......... ..........
ATCC_8739     .......... .......... .......... .......... .......... .......... ..........
ECOR_10       .......... .......... .......... .......... .......... .......... ..........
ECOR_11       .......... .......... .......... .......... .......... .......... ..........
B171          .......... .......... .......... .......... .........A ..A....... ..........
E24377A       .......... .......... .......... .......... .........A ..A....... ..........
ECOR_34       .......... .......... .......... .......... .........A ..A....... ..........
ECOR_71       .......... .......... .......... .......... .......... .......... ..........
SE11          .......... .......... .......... .......... .........A ..A....... ..........
O157_Sakai    .......... .......... .......... .......... .......... .......... ..........
ECOR_35       .T........ .......... .......... .......... .......... .......... ..........
ECOR_36       .T........ .......... .......... .......... .......... .......... ..........
SMS-3-5       .......... .......... .......... .......... .....A.... .......... ..........
536           .......... .......... .......... .......... .......... .......... ..........
APEC_O1       .......... .......... .......... .......... .......... .......... ..........
CFT073        .......... .......... .......... .......... .......... .......... ..........
E2348/69      .......... .......... .......... .......... .......... .......... ..........
ECOR_51       .......... .......... .......... .......... .......... .......... ..........
ECOR_57       .......... .......... .......... .......... .......... .......... ..........
UTI89         .......... .......... .......... .......... .......... .......... ..........
E._albertii   ....A..... G......... .......... .G..T...A. .......... .....C..T. ....G...T.

        12671
K-12          GCTTGAATAT TCCGCGCATA TGGTGCCGGA AGGCGGTCTG GCAATGGTGC CGCAACTGGT TAACGAGGGC
53638         .......... .......... .......... .......... .......... .......... ..........
ATCC_8739     .......... .......... .......... .......... .......... .......... ..........
ECOR_10       .......... .......... .......... .......... .......... .......... ..........
ECOR_11       .......... .......... .......... .......... .......... .......... ..........
B171          .......... ....G..... .......A.. .......... .......... ....GA.... ......T...
E24377A       .......... .......... .......A.. .......... .......... ....GA.... ......T...
ECOR_34       .......... .......... .......A.. .......... .......... ....GA.... ......T...
ECOR_71       .......... .......... .......A.. .......... ..G....... .......... ......T...
SE11          .......... .......... .......A.. .......... .......... ....GA.... ......T...
O157_Sakai    .......... .......... .......A.. .......... ..G.....A. .......... ......T...
ECOR_35       .........C .......... ....A..... .......... ..G....... ....G..... ......T...
ECOR_36       .........C .......... ....A..... .......... ..G....... ....G..... ......T...
SMS-3-5       .......... .......... .......... G......... .......... ....G..... ......T...
536           .......... .......... .......... G......... .......... ....G..... ......T...
APEC_O1       .......... .......... .......... G......... .......... ....G..... ......T...
CFT073        .......... .......... .......... G......... .......... ....G..... ......T...
E2348/69      .......... .......... .......... G......... .......... ....G..... ......T...
ECOR_51       .......... .......... .......... G......... .......... ....G..... ......T...
ECOR_57       .......... .......... .......... G......... .......... ....G..... ......T...
UTI89         .......... .......... .......... G......... .......... ....G..... ......T...
E._albertii   ...C..G..C .....A.... .......... ...T...... ..G....... ........A. ......T...

        12741
K-12          GTGATGATCG TTGGTGACGC CGCAGGCTTC TGCCTGAATT TGGGTTTTAC GGTCCGCGGC ATGGATTTAG
53638         .......... .......... .......... .......... .......... .......... ..........
ATCC_8739     .......... .......... .......... .......... .......... .......... ..........
ECOR_10       .......... .......... .......... .......... .......... .......... ..........
ECOR_11       .......... .......... .......... .......... .......... .......... ..........
B171          .......... .......... .......... .......... .......... A..T..T... ..........
E24377A       .......... .......... .......... .......... .......... A..T..T... ..........
ECOR_34       .......... .......... .......... .......... .......... A..T..T... ..........
ECOR_71       .......... .......... .......... .......... .......... ...T...... ..........
SE11          .......... .......... .......... .......... .......... A..T..T... ..........
O157_Sakai    .......... .......... .......... .......... .......... .......... ..........
ECOR_35       .....T.... .......... .......... .......... .......... A..T...... ..........
ECOR_36       .....T.... .......... .......... .......... .......... A..T...... ..........
SMS-3-5       .......... .......... .......... .......... .......... A..T...... ..........
536           .......... .......... .......... .......... .......... A..T...... ..........
APEC_O1       .......... .......... .......... .......... .......... A..T...... ..........
CFT073        .......... .......... .......... .......... .......... A..T...... ..........
E2348/69      .......... .......... .......... .......... .......... A..T...... ..........
ECOR_51       .......... .......... .......... .......... .......... A..T...... ..........
ECOR_57       .......... .......... .......... .......... .......... A..T...... ..........
UTI89         .......... .......... .......... .......... .......... A..T...... ..........
E._albertii   ..A....... .C........ A..C..T... .....T..CC ....C..... ...T..T..A ..........
```

```
        12811
K-12         CCATTGCATC GGCTCAGGCT GCCGCCACAA CGGTGATCGC CGCCAAAGAA CGCGCGGATT TCTCCGCCAG
53638        .......... .......... .......... .......... .......... .......... .........A
ATCC_8739    .......... ......A... .......... .......... .......... .......... ..........
ECOR_10      .......... .......... .......... .......... .......... .......... ..........
ECOR_11      .......... .......... .......... .......... .......... .......... ..........
B171         .......... .......... .......... .......... .......... ....A..... ..........
E24377A      .......... .......... .......... .......... .......... ....A..... ..........
ECOR_34      .......... .......... .......... .......... .......... ....A..... ..........
ECOR_71      .......... .......... .......... .A........ .......... ....A..... ..........
SE11         .......... .......... .......... .......... .......... ....A..... ..........
O157_Sakai   .......... .......... .......... .A........ .......... .......... ..........
ECOR_35      .......... A......... .......... .A.....T.. .......... .......... ..........
ECOR_36      .......... A......... .......... .A.....T.. .......... .......... ..........
SMS-3-5      .......... A......... .......... .A.....T.. A......... .......... ..........
536          .......... A......... .......... .......... .......... .......... ..........
APEC_O1      .......... A......... .......... .......... .......... .......... ..........
CFT073       .......... A......... .......... .......... .......... .......... ..........
E2348/69     .......... A......... .......... .......... .......... .......... ..........
ECOR_51      .......... A......... .......... .......... .......... .......... ..........
ECOR_57      .......... A......... .......... .......... .......... .......... ..........
UTI89        .......... A......... .......... .......... .......... .......... ..........
E._albertii  ....C..T.. ...A.....C .......... ...CT..T.. .........G ..T..T.... ....T....A

        12881
K-12         CAGTCTGGCG CAATACAAAC GTGAGCTGGA ACAAAGCTGC GTCATGCGTG ATATGCAGCA TTTTCGCAAG
53638        ......T... .......... .......... .......... .......... .......... ..........
ATCC_8739    ......T... .......... .......... .......... .......... .......... ..........
ECOR_10      .......... .......... .......... .......... .......... .......... ..........
ECOR_11      .......... .......... .......... .......... .......... .......... ..........
B171         .......... .......... .......... .......... ........C. .......... ...C......
E24377A      .......... .......... .......... .......... ........C. .......... ...C......
ECOR_34      .......... .......... .......... .......... ........C. .......... ...C......
ECOR_71      .......... .......... .......... .......... ........C. .......... ...C......
SE11         .......... .......... .......... .......... ........C. .......... ...C......
O157_Sakai   .......... .......... .......... .......... .......... .......... ..........
ECOR_35      ......T... .......... .......... ......T..T ..T.....C. .......... ...C......
ECOR_36      ......T... .......... .......... ......T..T ..T.....C. .......... ...C......
SMS-3-5      .......... .......... .......... .......... .......... .......... ..........
536          .......... .......... .......... ......T..T ..T.....C. .......... ...C......
APEC_O1      .......... .......... .......... ......T..T ..T.....C. .......... ...C......
CFT073       .......... .......... .......... ......T..T ..T.....C. .......... ...C......
E2348/69     .......... .......... .......... ......T..T ..T.....C. .......... ...C......
ECOR_51      .......... .......... .......... ......T..T ..T.....C. .......... ...C......
ECOR_57      .......... .......... .......... ......T..T ..T.....C. .......... ...C......
UTI89        .......... .......... .......... ......T..T ..T.....C. .......... ...C......
E._albertii  .......... .......... .C..A..... ......T... .....C..C. .......... ..........

        12951
K-12         ATCCCGGCGC TGATGGAAAA CCCGCGCCTG TTTAGCCAAT ACCCACGAAT GGTAGCCGAC ATCATGAACG
53638        .......... .......... .......... .......... .......... .......... ..........
ATCC_8739    .......... .......... .......... .......... .......... .......... ..........
ECOR_10      .......... .......... .......... .......... .......... .......... ..........
ECOR_11      .......... .......... .......... .......... .......... .......... ..........
B171         .......... .......... .......... .......... .......... ...C...... ..........
E24377A      .......... .......... .......... .......... .......... ...C...... ..........
ECOR_34      .......... .......... .......... .......... .......... ...C...... ..........
ECOR_71      .......... .......... .......... .......... .......... ...C...... ..........
SE11         .......... .......... .......... .......... .......... ...C...... ..........
O157_Sakai   .......... .......... .......... .......... .......... .......... ..........
ECOR_35      .......... .......... .......... .......... .......... .......... ..........
ECOR_36      .......... .......... .......... .......... .......... .......... ..........
SMS-3-5      .......... .......... T......... .....T..G. .T..G..C.. ...C...... ..........
536          C......... .......... .......... .......... .......... ...C...... ..........
APEC_O1      .......... .......... .......... .......... .......... ...C...... ..........
CFT073       .......... .......... .......... .......... .......... ...C...... ..........
E2348/69     .......... .......... .......... .......... .......... ...C...... ..........
ECOR_51      .......... .......... .......... .......... .......... ...C...... ..........
ECOR_57      .......... .......... .......... .......... .......... ...C...... ..........
UTI89        .......... .......... .......... .......... .......... ...C...... ..........
E._albertii  .......... .......... ...A...... .......... .......C.. ...C...... ..........
```

```
        13021
K-12         AGATGTTCAC CATCGACGGC AAACCAAACC AGCCGGTACG AAAAATGATC ATGGGACATG CGAAGAAAAT
53638        .......... ...T...... .......... .......... C......... .......... ..........
ATCC_8739    .......... ...T...... .......... .......... C......... .......... ..........
ECOR_10      .......... .......... .......... .......... .......... .......... ..........
ECOR_11      .......... .......... .......... .......... .......... .......... ..........
B171         .......... ...T...... .......... .......... C......... .......... ..........
E24377A      .......... ...T...... .......... .......... C......... .......... ..........
ECOR_34      .......... ...T...... .......... .......... C......... .......... ..........
ECOR_71      .......... ...T...... .......... .......... C......... .......... ..........
SE11         .......... ...T...... .......... .......... C......... .......... ..........
O157_Sakai   .......... ...T.....T ..G..T.... .......... C......... ........C. ..........
ECOR_35      .......... ...T...... .......... .......... C......... ........C. ..........
ECOR_36      .......... ...T...... .......... .......... C......... ........C. ..........
SMS-3-5      .......... ...T...... .......... .......... C......... ........C. ..........
536          .T........ ...T...... .......... .......... C......... ........C. ..........
APEC_O1      .T........ ...T...... .......... .......... C......... ........C. ..........
CFT073       .T........ ...T...... .......... .......... C......... ........C. ..........
E2348/69     .T........ ...T...... .......... .......... C......... ........C. ..........
ECOR_51      .T........ ...T...... .......... .......... C......... ........C. ..........
ECOR_57      .T........ ...T...... .......... .......... C......... ........C. ..........
UTI89        .T........ ...T...... .......... .......... C......... ........C. ..........
E._albertii  .T........ ...T.....T .GG.....T. ....A..... C........A C.C.....C. ..........

        13091
K-12         TGGCTGATCA AACTTGCTGA AAGATGGCAT TAAGGGAGCA ACCGCGCTAT GAGCCAGAAC GCTACGGTTA
53638        C......... .......... .......... .......... .......... .......... ..........
ATCC_8739    C......... .......... .......... .......... .......... .......... ..........
ECOR_10      .......... .......... .......... .......... .......... .......... ..........
ECOR_11      .......... .......... .......... .......... .......... .......... ..........
B171         .......... .......... .......... .......... .......... .......... ..........
E24377A      .......... .......... .......... .......... .......... .......... ..........
ECOR_34      .......... .......... .......... .......... .......... .......... ..........
ECOR_71      .......... .......... .......... .......... .......... .......... ..........
SE11         .......... .......... .......... .......... .......... .......... ..........
O157_Sakai   .......... .......... .......... .......... .......... .......... ..........
ECOR_35      .......... .......... .......... .......... .......... .......... ..........
ECOR_36      .......... .......... .......... .......... .......... .......... ..........
SMS-3-5      .......... .......... .......... .......... .......... .......... ..........
536          .......... .......... .......... .......... .......... .......... ..........
APEC_O1      .......... .......... .......... .......... .......... .......... ..........
CFT073       .......... .......... .......... .......... .......... .......... ..........
E2348/69     .......... .......... .......... .......... .......... .......... ..........
ECOR_51      .......... .......... .......... .......... .......... .......... ..........
ECOR_57      .......... .......... .......... .......... .......... .......... ..........
UTI89        .......... .......... .......... .......... .......... .......... ..........
E._albertii  C..C...... ...C...... .......... .......... .......... ...T...... .....A..A.

        13161
K-12         ACGTCGACAT CAAATTAGGC GTCAATAAAT TCCATGTTGA TGAGGGCCAC CCGCATATCA TTCTGGCGGA
53638        .......... .......... .......... .......... .......... .......... ..T......C
ATCC_8739    .......... .......... .......... .......... .......... .......... ..T......C
ECOR_10      .......... .......... .......... .......... .......... .......... ..........
ECOR_11      .......... .......... .......... .......... .......... .......... ..........
B171         .......... .......... .......... .......... .......... .......... .........C
E24377A      .......... .......... .......... .......... .......... .......... .........C
ECOR_34      .......... .......... .......... .......... .......... .......... .........C
ECOR_71      .......... .......... .......... .......... .......... .......... .........C
SE11         .......... .......... .......... .......... .......... .......... .........C
O157_Sakai   .......... .......... .......... .......... .......... .......... ..T......C
ECOR_35      .......... .......... .......... .......... .......... .......... ..T......C
ECOR_36      .......... .......... .......... .......... .......... .......... ..T......C
SMS-3-5      .......... .......... .......... .......... ......A... .......... ..T......C
536          .......... .......... .......... .......... .......... .......... ..T......C
APEC_O1      .......... .......... .......... .......... .......... .......... ..T......C
CFT073       .......... .......... .......... .......... .......... .......... ..T......C
E2348/69     .......... .......... .......... .......... .......... .......... ..T......C
ECOR_51      .......... .......... .......... .......... .......... .......... ..T......C
ECOR_57      .......... .......... .......... .......... .......... .......... ..T......C
UTI89        .......... .......... .......... .......... .......... .......... ..T......C
E._albertii  ....T...G. T......... ..T..C.... .......... ......GA.T .....C.... .C.....A.C
```

```
             13231
K-12         AAATCCCGAT ATCAATGAAT TCCATAAATT AATGAAAGCC TGCCCTGCCG GACTTTATAA GCAGGATGAC
53638        .......... .......... ...G...... .......... .......... .......... ..........
ATCC_8739    .......... .......... ...G...... .......... .......... .......... ..........
ECOR_10      .......... .......... .......... .......... .......... .......... ..........
ECOR_11      .......... .......... .......... .......... .......... .......... ..........
B171         .......... .......... ...G...... .......... .......... .......... ..........
E24377A      .......... .......... ...G...... .......... .......... .......... ..........
ECOR_34      .......... .......... ...G...... .......... .......... .......... ..........
ECOR_71      .......... .......... ...G...... .......... .......... .......... ..........
SE11         .......... .......... ...G...... .......... .......... .......... ..........
O157_Sakai   .......... .......... ...G...... .......... .......... .......... ..........
ECOR_35      .......... .......... ...G...... .......... .......... .......... ..........
ECOR_36      .......... .......... ...G...... .......... .......... .......... ..........
SMS-3-5      .......... .......... ...G...... .........T .......... .......... ..........
536          .......... .......... ...G...... .........T .......... .......... ..........
APEC_O1      .......... .......... ...G...... .........T .......... .......... ..........
CFT073       .......... .......... ...G...... .........T .......... .......... ..........
E2348/69     .......... .......... ...G...... .........T .......... .......... ..........
ECOR_51      .......... .......... ...G...... .........T .......... .......... ..........
ECOR_57      .......... .......... ...G...... .........T .......... .......... ..........
UTI89        .......... .......... ...G...... .........T .......... .......... ..........
E._albertii  ......T... .......... .T.G...... .......... ........G. .......... A........A

             13301
K-12         GCAGGAAACA TTCATTTTGA TTCCGCCGGT TGTCTGGAGT GCGGCACCTG TCGGGTGCTG TGCGGTAACA
53638        ..C..C.... .......... ...T...... .......... .......... .......T.. ..........
ATCC_8739    ..C..C.... .......... ...T...... .......... .......... .....G.T.. ..........
ECOR_10      .......... .......... .......... .......... .......... .......... ..........
ECOR_11      .......... .......... .......... .......... .......... .......... ..........
B171         ..C..C.... .......... ...T...... .......... .......... .......... ..........
E24377A      ..C..C.... .......... ...T...... .......... .......... .......... ..........
ECOR_34      ..C..C.... .......... ...T...... .......... .......... .......... ..........
ECOR_71      ..C..C.... .......... ...T...... .......... .......... .......... ..........
SE11         ..C..C.... .......... ...T...... .......... .......... .......... ..........
O157_Sakai   .......... .......... .......... .......... .......... .......... ..........
ECOR_35      .......... .......C.. .......... .......... .......... .......... ..........
ECOR_36      .......... .......C.. .......... .......... .......... .......... ..........
SMS-3-5      .......... .......C.. .......... .......... .......... .......... .....C....
536          .......... .......C.. .......... .......... .......... .......... .....C....
APEC_O1      .......... .......C.. .......... .......... .......... .......... .....C....
CFT073       .......... .......C.. .......... .......... .......... .......... .....C....
E2348/69     .......... .......C.. .......... .......... .......... .......... .....C....
ECOR_51      .......... .......C.. .......... .......... .......... .......... .....C....
ECOR_57      .......... .......C.. .......... .......... .......... .......... .....C....
UTI89        .......... .......C.. .......... .......... .......... .......... .....C....
E._albertii  ..C.....T. .C........ ......T..C ........A. .......... CA........ .....C....

             13371
K-12         CTATTCTCGA ACAGTGGCAA TATCCCGCAG GCACCTTCGG TATTGATTTT CGCTACGGCT AATCATGCAT
53638        .......... .......A.. .......... .T........ .........C ..T....... .....C....
ATCC_8739    .......... .......A.. .......... .T........ .........C ..T....... .....C....
ECOR_10      .......... .......... .......... .......... .......... .......... ..........
ECOR_11      .......... .......... .......... .......... .......... .......... ..........
B171         .......... .......... .......... .......... .......... .......... ..........
E24377A      .......... .......... .......... .......... .......... .......... ..........
ECOR_34      .......... .......... .......... .......... .......... .......... ..........
ECOR_71      .......... .......... .......... .......... .......... .......... ..........
SE11         .......... .......... .......... .......... .......... .......... ..........
O157_Sakai   .......... .......... .......... .......... .......... .......... ..........
ECOR_35      .......... .......... .......... .......... .......... .......... ..........
ECOR_36      .......... .......... .......... .......... .......... .......... ..........
SMS-3-5      .......... .......... .......... .......... .......... .......... ..........
536          .......... .......... .......... .......... ......A... .......... ..........
APEC_O1      .......... .......... .......... .......... ......A... .......... ..........
CFT073       .......... .......... .......... .......... ......A... .......... ..........
E2348/69     .......... .......... .......... .......... ......A... .......... ..........
ECOR_51      .......... .......... .......... .......... ......A... .......... ..........
ECOR_57      .......... .......... .......... .......... ......A... .......... ..........
UTI89        .......... .......... .......... .......... ......A... .......... ..........
E._albertii  .C........ .......... ..C....... .T........ .........C ..T....... ...T.C..C.
```

```
        13441
K-12          CCCACAGGCC CGCATCTCGG GCCTGATGTT CTGTTTCGAG AGTCCAACAT GAAAGTGACA TTAACGTTTA
53638         .......... ....CA.... .......... .......... .......A.. .......... ..........
ATCC_8739     .......... ....CA.... .......... .......... .......A.. .......... ..........
ECOR_10       .......... .......... .......... .......... .......... .......... ..........
ECOR_11       .......... .......... .......... .......... .......... .......... ..........
B171          .......... .......... .......... .......... .......... .......... ........C.
E24377A       .......... .......... .......... .......... .......... .......... ........C.
ECOR_34       .......... .......... .......... .......... .......... .......... ........C.
ECOR_71       .......... .......... .......... .......... .......... .......... ........C.
SE11          .......... .......... .......... .......... .......... .......... ........C.
O157_Sakai    .......... .......... .......... .......... .......A.. .......... ..........
ECOR_35       .......... .......... .......... .......... .......A.. .......... ..........
ECOR_36       .......... .......... .......... .......... .......A.. .......... ..........
SMS-3-5       .......... .......... .......... .......... .......A.. .......... ..........
536           .......... .......... .......... .......... .......A.. .......... ........C.
APEC_O1       .......... .......... .......... .......... .......A.. .......... ........C.
CFT073        .......... .......... .......... .......... .......A.. .......... ........C.
E2348/69      .......... .......... .......... .......... .......A.. .......... ........C.
ECOR_51       .......... .......... .......... .......... .......A.. .......... ........C.
ECOR_57       .......... .......... .......... .......... .......A.. .......... ........C.
UTI89         .......... .......... .......... .......... .......A.. .......... ........C.
E._albertii   .......... ..G.CA.... ......C... .A......G. .A.GAC.A.. .......... .....A....

        13511
K-12          ACGAACAACG TCGTGCGGCG TATCGTCAGC AAGGGTTATG GGGCGATGCT TCGCTGGCCG ATTACTGGCA
53638         .......... .......... .......... .....C.... .......... .......... ..........
ATCC_8739     .......... .......... .......... .....C.... .......... .......... ..........
ECOR_10       .......... .......... .......... .......... .......... .......... ..........
ECOR_11       .......... .......... .......... .......... .......... .......... ..........
B171          .......... .......... .......... .....C.... .......... .......... ..........
E24377A       .......... .......... .......... .....C.... .......... .......... ..........
ECOR_34       .......... .......... .......... .....C.... .......... .......... ..........
ECOR_71       .......... .......... .......... .....C.... .......... .......... ..........
SE11          .......... .......... .......... .....C.... .......... .......... ..........
O157_Sakai    .......... .......... .......... .....C.... .......... .......... ..........
ECOR_35       .......... .......... .......... .......... .......... ..A....... ..........
ECOR_36       .......... .......... .......... .......... .......... ..A....... ..........
SMS-3-5       .......... .......... .......... .....C.... .......... .......... ..........
536           .......... .......... .......... .......... .......... ..A....... ..........
APEC_O1       .......... .......... .......... .......... .......... .......... ..........
CFT073        .......... .......... .......... .......... .......... .......... ..........
E2348/69      .......... .......... .......... .......... .......... .......... ..........
ECOR_51       .......... .......... .......... .......... .......... .......... ..........
ECOR_57       .......... .......... .......... .......... .......... .......... ..........
UTI89         .......... .......... .......... .......... .......... .......... ..........
E._albertii   ...CG..... ......A..A .......... ....A..... .......... ...T....G. ..........

        13581
K-12          GCAGACCGCT CGTGCGATGC CAGACAAAAT TGCCGTGGTC GATAATCATG GTGCATCGTA CACCTATAGC
53638         .......... .......... .......... .......... .......... .A........ ..........
ATCC_8739     .......... .......... .......... .......... .......... .A........ ..........
ECOR_10       .......... .......... .......... .......... .......... .......... ..........
ECOR_11       .......... .......... .......... .......... .......... .......... ..........
B171          .......... .......... .......... .......... .......... .......... ..........
E24377A       .......... .......... .......... .......... .......... .......... ..........
ECOR_34       .......... .......... .......... .......... .......... .......... ..........
ECOR_71       .......... .......... .......... .......... .......... .A........ ..........
SE11          .......... .......... .......... .......... .......... .......... ..........
O157_Sakai    .......... .......... .......... .......... .......... .A........ ..........
ECOR_35       .......... .......... .......... .......... ..C....... .....A.... ..........
ECOR_36       .......... .......... .......... .......... ..C....... .....A.... ..........
SMS-3-5       .......... .......... .......... .......... .......... .......A.. ..........
536           .......... .......... .......... .......... ..C....... .....A.... ..........
APEC_O1       .......... .......... .......... .......... ..C....... .....A.... ..........
CFT073        .......... .......... .......... .......... .......... .....A.... ..........
E2348/69      .......... .......... .......... .......... .......... .....A.... ..........
ECOR_51       .......... .......... .......... .......... .......... .....A.... ..........
ECOR_57       .......... .......... .......... .......... .......... .....A.... ..........
UTI89         .......... .......... .......... .......... ..C....... .....A.... ..........
E._albertii   C.......TA ..G..A.... .G........ A..T...... .......... .......... ..........
```

```
         13651
K-12         GCGCTCGATC ACGCCGCGAG CTGTCTGGCA AACTGGATGT TAGCGAAGGG TATTGAATCA GGCGATCGCA
53638        ..A....... .......... .......... .......... .......... .......... ..........
ATCC_8739    ..A....... .......... .......... .......... .......... .......... ..........
ECOR_10      .......... .........A .......... .......... .......... .......... ..........
ECOR_11      .......... .......... .......... .......... .......... .......... ..........
B171         .......... .......... .......... .......... .......... .......... ..........
E24377A      .......... .......... .......... .......... .......... .......... ..........
ECOR_34      .......... .......... .......... .......... .......... .......... ..........
ECOR_71      .......... .......... .......... .......... .......... .......... ..........
SE11         .......... .......... .......... .......... .......... .......... ..........
O157_Sakai   .......... .......... .......... .......... .......... .......... ..........
ECOR_35      .......... .......... .......... .......... .......... .......... ..........
ECOR_36      .......... .......... .......... .......... .......... .......... ..........
SMS-3-5      .......... .......... .......... .......... .GA....... .......... ..........
536          .......... .......... .......... .......... .......... .......... ..........
APEC_O1      .......... .......... .......... .......... .......... .......... ..........
CFT073       .......... .......... .......... .......... .......... .......... ..........
E2348/69     .......... .......... .......... .......... .......... .......... ..........
ECOR_51      .......... .......... .......... .......... .......... .......... ..........
ECOR_57      .......... .......... .......... .......... .......... .......... ..........
UTI89        .......... .......... .......... .......... .......... .......... ..........
E._albertii  T......... .T........ .......... .G.......C .G.A...C.. .........C ......T..G

         13721
K-12         TCGCATTTCA ACTGCCTGGC TGGTGTGAAT TTACCGTTAT CTATCTTGCC TGCCTGAAAA TCGGTGCAGT
53638        .......... ......A... .......... .......... .......... .......... .......G..
ATCC_8739    .......... ......A... .......... .......... .......... .......... .......G..
ECOR_10      .......... .......... .......... .......... .......... .......... ..........
ECOR_11      .......... .......... .......... .......... .......... .......... ..........
B171         .......... .......... .......... .......... .......... .......... .......G..
E24377A      .......... .......... .......... .......... .......... .......... .......G..
ECOR_34      .......... .......... .......... .......... .......... .......... .......G..
ECOR_71      .......... ......A... .......... .......... .......... .......... C......G..
SE11         .......... .......... .......... .......... .......... .......... .......G..
O157_Sakai   .A........ ......A... .......... .......... .......... .......... .......G..
ECOR_35      .......... ......A... .......... .......... .......... .......... C......G..
ECOR_36      .......... ......A... .......... .......... .......... .......... C......G..
SMS-3-5      .......... .......... .......... .......... .......... .......... .......G..
536          .......... ......A... .......... .......... .......... .......... C......G..
APEC_O1      .......... ......A... .......... .......... .......... .......... C......G..
CFT073       .......... ......A... .......... .......... .......... .......... C......G..
E2348/69     .......... ......A... .......... .......... .......... .......... C......G..
ECOR_51      .......... ......A... .......... .......... .......... .......... C......G..
ECOR_57      .......... ......A... .......... .......... .......... .......... C......G..
UTI89        .......... ......A... .......... .......... .......... .......... C......G..
E._albertii  ....T..... ......C... .......... .......... ......C... .......... C......G..

         13791
K-12         TTCCGTGCCG CTGTTGCCTT CCTGGCGGGA AGCAGAACTG GTGTGGGTGC TCAATAAGTG TCAGGCAAAA
53638        ......A... .......... .......... .......... .......... .T........ ..........
ATCC_8739    ......A... .......... .......... .......... .......... .T........ ..........
ECOR_10      .......... .......... .......... .......... .......... .......... ..........
ECOR_11      .......... .......... .......... .......... .......... .......... ..........
B171         .......... .......... .......... .......... .......... .......... ..........
E24377A      .......... .......... .......... .......... .......... .......... ..........
ECOR_34      .......... .......... .......... .......... .......... .......... ..........
ECOR_71      .......... .......... .......... .......... .......... .......... ..........
SE11         .......... .......... .......... .......... .......... .......... ..........
O157_Sakai   ......A... .......... .......... .......... ..A....... .T........ ..........
ECOR_35      .......... .....A.... .......... .......... .......... .......... ..........
ECOR_36      .......... .....A.... .......... .......... .......... .......... ..........
SMS-3-5      ......A... .......... .......... .........A ..A.....A. .......... ..........
536          .......... .....A.... .......... .......... .......... .......... ..........
APEC_O1      .......... .....A.... .......... .......... ..A....... .......... ..........
CFT073       .......... .....A.... .......... .......... .......... .......... ..........
E2348/69     .......... .....A.... .......... .......... .......... .......... ..........
ECOR_51      .......... .....A.... .......... .......... .......... .......... ..........
ECOR_57      .......... .....A.... .......... .......... .......... .......... ..........
UTI89        .......... .....A.... .......... .......... ..A....... .......... ..........
E._albertii  C......... ..A....... .......A.. ...G...... TGT....... .......... C.....G...
```

```
        13861
K-12         ATGTTCTTTG CACCGACGTT GTTTAAACAA ACGCGTCCGG TAGATTTAAT CCTGCCGCTG CAAAATCAGC
53638        .......... .......... .......... .......... .......... .......... ..........
ATCC_8739    .......... .......... .......... .......... .......... .......... ..........
ECOR_10      .......... .......... .......... .......... .......... .......... ..........
ECOR_11      .......... .......... .......... .......... .......... .......... ..........
B171         .......... .......... .......... .....C.... .......... .......... ..........
E24377A      .......... .......... .......... .....C.... .......... .......... ..........
ECOR_34      .......... .......... .......... .....C.... .......... .......... ..........
ECOR_71      .......... .......... .......... .......... .......... .......... ..........
SE11         .......... .......... .......... .....C.... .......... .......... ..........
O157_Sakai   .........A .......... .......... .......... .......... .......... ..........
ECOR_35      .......... .......... .......... .......... .......... ......A... ..........
ECOR_36      .......... .......... .......... .......... .......... ......A... ..........
SMS-3-5      .......... .......... .......... .......... .......... .......... ..........
536          .......... .......... .......... .......... .......... .......... ..........
APEC_O1      .......... .......... .......... ........A. .......... .......... ..........
CFT073       .......... .......... .......... .......... .......... .......... ..........
E2348/69     .......... .......... .......... ........A. .......... .......... ..........
ECOR_51      .......... .......... .......... .......... .......... .......... ..........
ECOR_57      .......... .......... .......... .......... .......... .......... ..........
UTI89        .......... .......... .......... ........A. .......... .......... ..........
E._albertii  .......... .G..A..... A......... ..A....... .......... .......... ..........

        13931
K-12         TTCCACAACT ACAACAAATT GTCGGCGTGG ACAAACTGGC TCCCGCCACC TCTTCCCTCT CATTAAGTCA
53638        .......... .......... .......... .......... .......... .......... ..........
ATCC_8739    .......... .......... .......... .......... .......... .......... ..........
ECOR_10      .......... .......... .......... .......... .......... .......... ..........
ECOR_11      .......... .......... .......... .......... .......... .......... ..........
B171         .......... .......... .......... .......... .......... .......... ..........
E24377A      .......... .......... .......... .......... .......... .......... ..........
ECOR_34      .......... .......... .......... .......... .......... .......... ..........
ECOR_71      .......... .......... .....T.... .......... .......... .......... ..........
SE11         .......... .......... .......... .......... .......... .......... ..........
O157_Sakai   .......... .......... .......... .......... .......... .......... ..........
ECOR_35      .......... .......... .......... .......... .......... .......... ..........
ECOR_36      .......... .......... .......... .......... .......... .......... ..........
SMS-3-5      .......... .......... .......... .......... .......... .......... ..........
536          .......... .......... .......... .......... .......... .......... ..........
APEC_O1      .......... .......... .......... .......... .......... .......... ..........
CFT073       .......... .......... .......... .......... .......... .......... ..........
E2348/69     .......... .......... .......... ........T. .......... .......... ..........
ECOR_51      .......... .......... .......... .......... .......... .......... ..........
ECOR_57      .......... .......... .......... .......... .......... .......... ..........
UTI89        .......... .......... .......... .......... .......... .......... ..........
E._albertii  .A........ G......... ....CT..A. .......... .......... .....TA... .G........

        14001
K-12         GATTATCGCC GACAATACCT CACTGACCAC GGCGATAACG ACCCACGGCG ATGAATTAGC TGCGGTGCTG
53638        .......... .........C .......... .......... .......... .......... ..........
ATCC_8739    .......... .........C .......... .......... .......... .......... ..........
ECOR_10      .......... .......... .......... .......... .......... .......... ..........
ECOR_11      .......... .......... .......... .......... .......... .......... ..........
B171         .......... .......... .......... .......... .......... .......... ..........
E24377A      .......... .........C .......... .......... .......... .......... ..........
ECOR_34      .......... .........C .......... .......... .......... .......... ..........
ECOR_71      .......... .......... .......... .......... .......... .......... ..........
SE11         .......... .........C .......... .......... .......... .......... ..........
O157_Sakai   .......... .........C .......... .......... .......... .......... ..........
ECOR_35      .......... ........TC .T........ .......... GT........ .......... C.........
ECOR_36      .......... ........TC .T........ .......... GT........ .......... C.........
SMS-3-5      ....C..... .........C .......T.. .......... .......... .......... C..T.....A
536          ....C..... ........TC .T........ .......... GT........ .......... C..T.....A
APEC_O1      ....C..... .........C .T........ .......... .......... .......... C..T.....A
CFT073       ....C..... .........C .T........ .......... .......... .......... C..T.....A
E2348/69     ....C..... .........C .T........ .......... .......... .......... C..T.....A
ECOR_51      ....C..... .........C .T........ .......... .......... .......... C..T.....A
ECOR_57      ....C..... .........C .T........ .......... .......... .......... C..T.....A
UTI89        ....C..... .........C .T........ .......... .......... .......... C..T.....A
E._albertii  ......TA.. .....CG.TC .G....GT.. .A.A...T.A .G......A. .......... G..A..A..A
```

```
          14071
K-12          TTTACCTCCG GAACCGAGGG TCTGCCAAAG GGCGTGATGC TAACGCATAA CAATATTCTC GCCAGTGAGC
53638         .......... .......... .......... .......... .......... .......... ..........
ATCC_8739     .......... .......... .......... .......... .......... .......... ..........
ECOR_10       .......... .......... .......... .......... .......... .......... ..........
ECOR_11       .......... .......... .......... .......... .......... .......... ..........
B171          .......... .......... .......... .....A.... .......... .......... ..........
E24377A       .......... .......... .......... .......... .......... .......... ..........
ECOR_34       .......... .......... .......... .......... .......... .......... ..........
ECOR_71       .......... .......... .......... .......... .......... .......... ..........
SE11          .......... .......... .......... .......... .......... .......... ..........
O157_Sakai    .......... .......... .......... .......... .T........ .......... ..........
ECOR_35       .......... .......... .......... .......... .......... .......... ..........
ECOR_36       .......... .......... .......... .......... .......... .......... ..........
SMS-3-5       .......... .......... .......... .......... .......... .......... ..........
536           .......... .......... .......... ..T....... .......... .......... ..........
APEC_O1       .......... .......... .......... ..T....... .......... .......... ..........
CFT073        .......... .......... .......... ..T....... .......... .......... ..........
E2348/69      .......... .......... .......... .......... .......... .......... ..........
ECOR_51       .......... .......... .......... ..T....... .......... .......... ..........
ECOR_57       .......... .......... .......... ..T....... .......... .......... ..........
UTI89         .......... .......... .......... ..T....... .......... .......... ..........
E._albertii   .......... .G..A..A.. .A........ .....A.... .G........ ......C..G .....C....

          14141
K-12          GGGCTTATTG CGCGCGACTG AATCTGACCT GGCAGGATGT CTTTATGATG CCTGCGCCAC TTGGTCACGC
53638         .......... ......G... .......... .......... .......... .......... .C..C.....
ATCC_8739     .......... ......G... .......... .......... .......... .......... .C..C.....
ECOR_10       .......... .......... .......... .......... .......... .......... ..........
ECOR_11       .......... .......... .......... .......... .......... .......... ..........
B171          .......... ..T...G... .......... .......... .......... .......... ..........
E24377A       .......... ......G... .......... .......... .......... .......... .C..C.....
ECOR_34       .......... ......G... .......... .......... .......... .......... .C..C.....
ECOR_71       .......... .......... .......... .......... .......... .......... ..........
SE11          .......... ......G... .......... .......... .......... .......... .C..C.....
O157_Sakai    .......... ......G... .....A.... .......... .......... .......... ..........
ECOR_35       .......... ......G... .......... .......... .......... .......... ..........
ECOR_36       .......... ......G... .......... .......... .......... .......... ..........
SMS-3-5       .......... ......G... .......... .......... .......... .......... ..........
536           .......... ......G..A .......... .......... .......... .......... ..........
APEC_O1       .......... ......G..A .......... .......... .......... .......... ..........
CFT073        .......... ......G..A .......... .......... .......... .......... ..........
E2348/69      .......... ......G..A .......... .......... .......... .......... ..........
ECOR_51       .......... ......G..A .......... .......... .......... .......... ..........
ECOR_57       .......... ......G..A .......... .......... .......... .......... ..........
UTI89         .......... ......G..A .......... .......... .......... .......... ..........
E._albertii   .A........ T..A.....A .......... ...T...... T......... ..C....... .C..C.....

          14211
K-12          AACGGGCTTT CTGCATGGCG TAACGGCACC ATTCTTAATT GGCGCTCGCA GCGTGTTGTT AGATATTTTC
53638         .......... .......... ....A..... ...T..G... .......... .......... ..........
ATCC_8739     .......... .......... ....A..... ...T..G... .......... .......... ..........
ECOR_10       .......... .......... .......... .......... .......... .......... ..........
ECOR_11       .......... .......... .......... .......... .......... .......... ..........
B171          ......A... .......... ....A..... ...T...... ..T....... .......... ..........
E24377A       .......... .......... .......... .......... .......... .......... ..........
ECOR_34       .......... .......... .......... .......... .......... .......... ..........
ECOR_71       .......... .......... .......... .......... .......... .......... ..........
SE11          .......... .......... .......... .......... .......... .......... ..........
O157_Sakai    .......... .......... .......... .......... .......... .......... ..........
ECOR_35       .......... .......... ....A..... ...T...... ..T....... .......... ..........
ECOR_36       .......... .......... ....A..... ...T...... ..T....... .......... ..........
SMS-3-5       .......... .......... ....A..... ...T...... ..T....... .......... ..........
536           .......... .......... ....A..... T..T..G... ..T....... .......... ..........
APEC_O1       .......... .......... ....A..... T..T..G... ..T....... .......... ..........
CFT073        .......... .......... ....A..... T..T..G... ..T....... .......... ..........
E2348/69      .......... .......... ....A..... T..T..G... ..T....... .......... ..........
ECOR_51       .......... .......... ....A..... T..T..G... ..T....... .......... ..........
ECOR_57       .......... .......... ....A..... T..T..G... ..T....... .......... ..........
UTI89         .......... .......... ....A..... T..T..G... ..T....... .......... ..........
E._albertii   G..A..T... .......... .......G.. ...T...C.. .....A.AG. ..C....... ......C...
```

```
        14281
K-12         ACTCCTGATG CGTGTCTCGC GCTGCTTGAG CAGCAGCGTT GCACCTGTAT GCTCGGCGCA ACGCCGTTTG
53638        .......... .......... .......... .......... .......... .......... ..........
ATCC_8739    .......... .......... .......... .......... .......... .......... ..........
ECOR_10      .......... .......... .......... .......... .......... .......... ..........
ECOR_11      .......... .......... .......... .......... .......... .......... ..........
B171         .......... .......... .......... .......... .......... ...A...... ..........
E24377A      .......... .......... .......... .......... .......... ...A...... ..........
ECOR_34      .......... .......... .......... .......... .......... ...A...... ..........
ECOR_71      .......... .......... .......... .......... .......... ...A...... ..........
SE11         .......... .......... .......... .......... .......... ...A...... ..........
O157_Sakai   .......... .......... .......... .......... ..T....... .......... ..........
ECOR_35      .......C.. .......... .......... .......... .......... .......... ..........
ECOR_36      .......C.. .......... .......... .......... .......... .......... ..........
SMS-3-5      .......C.. .......... .......... .......... .......... .......... ..........
536          .......... .......... .......... .......... .......... .......... ..........
APEC_O1      .......... .......... .......... .......... .......... .......... ..........
CFT073       .......... .......... .......... .......... .......... .......... ..........
E2348/69     .......... .......... .......... .......... .......... .......... ..........
ECOR_51      .......... .......... .......... .......... .......... .......... ..........
ECOR_57      .......... .......... .......... .......... .......... .......... ..........
UTI89        .......... .......... .......... .......... .......... .......... ..........
E._albertii  ..G.....A. TC......T. ...A...... ..A....... .T........ ...T...... ........C.

        14351
K-12         TCTATGATCT TTTGAATGTA CTAGAGAAAC AACCCGCGGA CCTTTCAGCG CTGCGTTTCT TTCTTTGCGG
53638        .......... .......T.. .......... .......A.. .......... .....C.... ..........
ATCC_8739    .......... .......T.. .......... .......A.. .......... .....C.... ..........
ECOR_10      .......... .......... .......... .......... .......... .......... ..........
ECOR_11      .......... .......... .......... .......... .......... .......... ..........
B171         .......... .......... .......... .......... ...G...... .......... ..........
E24377A      .......... .......... .......... .......... ...G...... .......... ..........
ECOR_34      .......... .......... .......... .......... ...G...... .......... ..........
ECOR_71      .......... .......... .......... .......... .......... .......... ..........
SE11         .......... .......... .......... .......... ...G...... .......... ..........
O157_Sakai   .......... .......T.. .......... .......A.. .......... .....C.... ..........
ECOR_35      .......... .......T.. .......... .G.....A.. .......... .......... ..........
ECOR_36      .......... .......T.. .......... .G.....A.. .......... .......... ..........
SMS-3-5      .......... .......T.. .......... .G.....A.. .......... .......... .......T..
536          .......... .......T.. .......... .......A.. .......... .....C.... ..........
APEC_O1      .......... .......T.. .......... .......A.. .......... .....C.... ..........
CFT073       .......... .......T.. .......... .......A.. .......... .....C.... ..........
E2348/69     .......... .......T.. .......... .......A.. .......... .....C.... ..........
ECOR_51      .......... .......T.. .......... .......A.. .......... .....C.... ..........
ECOR_57      .......... .......T.. .......... .......A.. .......... .....C.... ..........
UTI89        .......... .......T.. .......... .......A.. .......... .....C.... ..........
E._albertii  .G........ AC.....T.G ..G..A.... .G...A.A.. ......T..T ..A..C.... .C........

        14421
K-12         CGGAACCACA ATCCCCAAAA AAGTGGCGCG TGAATGCCAG CAGCGCGGCA TTAAATTATT AAGTGTTTAT
53638        ...T...... .......... .......A.. .......... .......... .......... ..........
ATCC_8739    ...T...... .......... .......A.. .......... .......... .......... ..........
ECOR_10      .......... .......... .......... .......... .......... .......... ..........
ECOR_11      .......... .......... .......... .......... .......... .......... ..........
B171         .......... .......... .......... .......... ....T..... .......... ..........
E24377A      .......... .......... .......... .......... ....T..... .......... ..........
ECOR_34      .......... .......... .......... .......... ....T..... .......... ..........
ECOR_71      ...T...... .......... .......A.. .......... .......... .......... ..........
SE11         .......... .......... .......... .......... ....T..... .......... ..........
O157_Sakai   ...T...... .......... .......... .......... .......... .......... ..........
ECOR_35      ...T...... .......... .......... .......... .......... .......... ..........
ECOR_36      ...T...... .......... .......... .......... .......... .......... ..........
SMS-3-5      ...T...... .......... .......... .......... .......... .......... ..........
536          A..T...... .......... .......... A......... .......... .......... ..........
APEC_O1      A..T...... .......... .......... A......... .......... .......... ..........
CFT073       A..T...... .......... .......... A......... .......... .......... ..........
E2348/69     A..T...... .......... .......... A......... .......... .......... ..........
ECOR_51      A..T...... .......... .......... A......... .......... .......... ..........
ECOR_57      A..T...... .......... .......... A......... .......... .......... ..........
UTI89        A..T...... .......... .......... A......... .......... .......... ..........
E._albertii  ...G.....G ......C... .....AT... .........A ..A....... .C...C.G.. ...C......
```

```
         14491
K-12         GGTTCCACAG AAAGTTCGCC GCATGCGGTG GTGAATCTCG ATGATCCTTT GTCGCGCTTT ATGCACACCG
53638        .......... .......... .......... .......... .......... .......... ..........
ATCC_8739    .......... .......... .......... .......... .......... .......... ..........
ECOR_10      .......... .......... .......... .......... .......... .......... ..........
ECOR_11      .......... .......... .......... .......... .......... .......... ..........
B171         .......... .......... .......... .......... .......... .......... ..........
E24377A      .......... .......... ........A. .......... .......... .......... ..........
ECOR_34      .......... .......... .......... .......... .......... .......... ..........
ECOR_71      .......... .......... .......... .......... .......... .......... ..........
SE11         .......... .......... .......... .......... .......... .......... ..........
O157_Sakai   .......... .......... .......... .......... .......... .......... ..........
ECOR_35      .......... .......... .......... .......... .......... .......... ..........
ECOR_36      .......... .......... .......... .......... .......... .......... ..........
SMS-3-5      .......... .......... .......... .......... .......... .......... ..........
536          .......... .......... .......... .......... .C........ .C........ ..........
APEC_O1      .......... .......... .......... .......... .C........ .C........ ..........
CFT073       .......... .......... .......... .......... .C........ .C........ ..........
E2348/69     .......... .......... .......... .......... .C........ .C........ ..........
ECOR_51      .......... .......... .......... .......... .C........ .C........ ..........
ECOR_57      .......... .......... .......... .......... .C........ .C........ ..........
UTI89        .......... .......... .......... .......... .C........ .C........ ..........
E._albertii  ..C..A.... .......... A........A .......... .......G.. ...A...... .....T....

         14561
K-12         ATGGTTACGC TGCCGCAGGT GTAGAGATTA AAGTGGTCGA TGACGCACGC AAGACCTTAC CGCCAGGTTG
53638        .......... .......... .......... .......... .......... .......... ..........
ATCC_8739    .......... .......... .......... .......... .......... .......... ..........
ECOR_10      .......... .......... .......... .......... .......... .......... ..........
ECOR_11      .......... .......... .......... .......... .......... ..A....... ..........
B171         .......... .......... .......... .......... .......... .......... ..........
E24377A      .......... .......... .......... .......... .......... .......... ..........
ECOR_34      .......... .......... .......... .......... .......... .......... ..........
ECOR_71      .......... .......... .......... .......... .......... .......... ..........
SE11         .......... .......... .......... .......... .......... .......... ..........
O157_Sakai   .......... .......... .......... .......... .......... .......... ..........
ECOR_35      .......... .......... .......... .......... .......... .......... ..........
ECOR_36      .......... .......... .......... .......... .......... .......... ..........
SMS-3-5      .......... .......... .......... .......... ..G....... .......... ..........
536          .C........ .......... .......... .......... .......... .....T.... ..........
APEC_O1      .C........ .......... .......... .......... .......... .....T.... ..........
CFT073       .C........ .......... .......... .......... .......... .....T.... ..........
E2348/69     .C........ .......... .......... .......... .......... .....T.... ..........
ECOR_51      .C........ .......... .......... .......... .......... .....T.... ..........
ECOR_57      .C........ .......... .......... .......... .......... .....T.... ..........
UTI89        .C........ .......... .......... .......... .......... .....T.... ..........
E._albertii  .......... G..T..T..C ..G.....C. .......... C..T.AG..T .......... ..........

         14631
K-12         CGAAGGTGAA GAAGCCTCGC GTGGCCCCAA TGTGTTTATG GGGTATTTTG ATGAACCTGA ATTAACCGCC
53638        .......... .......... .......... .......... ..A....... .......... ..........
ATCC_8739    .......... .......... .......... .......... ..A....... .......... ..........
ECOR_10      .......... .......... .......... .......... .......... .......... ..........
ECOR_11      .......... .......... .......... .......... .......... .......... ..........
B171         .......... .......... .......... .......... ..A....... .......... ..........
E24377A      .......... .......... .......... .......... ..A....... .......... ..........
ECOR_34      .......... .......... .......... .......... ..A....... .......... ..........
ECOR_71      .......... .......... .......... .......... .......... .......... ..........
SE11         .......... .......... .......... .......... ..A....... .......... ..........
O157_Sakai   .......... .......... .......... .......... ..A....... .......... ..........
ECOR_35      .......... .......... .......... .......... .......... .......... ..........
ECOR_36      .......... .......... .......... .......... .......... .......... ..........
SMS-3-5      .......... .......... .......... .......... .......... .......... ..........
536          .......... .......... .......... .......... .......... .......... ..........
APEC_O1      .......... .......... .......... .......... .......... .......... ..........
CFT073       .......... .......... .......... .......... .......... .......... ......T...
E2348/69     .......... .......... .......... .......... .......... .......... ..........
ECOR_51      .......... .......... .......... .......... .......... .......... ......T...
ECOR_57      .......... .......... .......... .......... .......... .......... ......T...
UTI89        .......... .......... .......... .......... .......... .......... ..........
E._albertii  T.....C... ..G..T.... .C.....G.. .......... ..C...C... .......A.. ......T...
```

```
        14701
K-12         CGTGCCCTGG ATGAAGAAGG CTGGTATTAC AGCGGCGATC TCTGCCGTAT GGATGAGGCT GGCTATATAA
53638        .....G.... .......... .......... .......... .......... .......... ..........
ATCC_8739    .....G.... .......... .......... .......... .......... .......... ..........
ECOR_10      .......... .......... .......... .......... .......... .......... ..........
ECOR_11      .......... .......... .......... .......... .......... .......... ..........
B171         .....G.... .......... .......... .......... .......... .......... ..........
E24377A      .....G.... .......... .......... .......... .......... .......... ..........
ECOR_34      .....G.... .......... .......... .......... .......... .......... ..........
ECOR_71      .....G.... .C........ .......... .......... .......C.. .......... ..........
SE11         .....G.... .......... .......... .......... .......... .......... ..........
O157_Sakai   .......... .......... .......... .......... .......... .......... ..........
ECOR_35      .....G.... .......... .......... .......... .......C.. .......... ..........
ECOR_36      .....G.... .......... .......... .......... .......C.. .......... ..........
SMS-3-5      .A........ .......... .......... .......... .......C.. .......... ..........
536          .......... .......... .......... .......... .......C.. .......... ..........
APEC_O1      .....G.... .......... .......... .......... .......C.. .......... ..........
CFT073       .....G.... .......... .......... .......... .......C.. .......... ..........
E2348/69     .....G.... .......... .......... .......... .......C.. .......... ..........
ECOR_51      .....G.... .......... .......... .......... .......C.. .......... ..........
ECOR_57      .....G.... .......... .......... .......... .......C.. .......... ..........
UTI89        .....G.... .......... .......... .......... .......C.. .......... ..........
E._albertii  ...A.G.... ..A.C..T.. .......... ..T....... .......C.. .........G ........C.

        14771
K-12         AAATTACCGG ACGCAAAAAA GATATTATTG TCCGCGGCGG CGAAAATATT AGCAGCCGTG A-CCACGCCG
53638        .......... .......... .......... .......... .......... .......... ..........
ATCC_8739    .......... .......... .......... .......... .......... .......... ..........
ECOR_10      .......... .......... .......... .......... .......... .......... ..........
ECOR_11      .......... .......... .......... .......... .......... .......... ..........
B171         .......... .......... .......... .......... .......... .......... ..........
E24377A      .......... .......... .......... .......... .......... .......... ..........
ECOR_34      .......... .......... .......... .......... .......... .......... ..........
ECOR_71      .......... .......... .......... .......... .......... .......... ..........
SE11         .......... .......... .......... .......... .......... .......... ..........
O157_Sakai   .......... .......... .......... .......... .......... .......... ..........
ECOR_35      ....A..... G.....G... .......... .......... .......... .......... -....GT...
ECOR_36      ....A..... G.....G... .......... .......... .......... .......... -....GT...
SMS-3-5      ....A..... G.....G... .......... .......... .......... .......... ..........
536          ....A..... G.....G... .......... .......... .......... .......... ..........
APEC_O1      ....A..... G.....G... .......... .......... .......... .......... ..........
CFT073       ....A..... G.....G... .......... .......... .......... .......... ..........
E2348/69     ....A..... G.....G... .......... .......... .......... .......... ..........
ECOR_51      ....A..... G.....G... .......... .......... .......... .......... ..........
ECOR_57      ....A..... G.....G... .......... .......... .......... .......... ..........
UTI89        ....A..... G.....T... .......... .......... .......... .......... ..........
E._albertii  ....A..... G......... .......... .T........ A......... .....T.... .A.......A

        14841
K-12         GAGTCACGGT CCAGACCGAG CGCCAGCTGC GTCATATCGT TTGAGCCAAT TGAGAAGCCG TCGAAATATT
53638        .......... .......... .......... .......... .......... .......... ..........
ATCC_8739    .......... .......... .......... .......... .......... .......... ..........
ECOR_10      .......... .......... .......... .......... .......... .......... ..........
ECOR_11      .......... .......... .......... .......... .......... .......... ..........
B171         ........A. .......... .........T .......... .......... .......... ..........
E24377A      ........A. .......... .........T .......... .......... .......... ..........
ECOR_34      ........A. .......... .........T .......... .......... .......... ..........
ECOR_71      ........A. .......... .........T .......... .......... .......... ..........
SE11         ........A. .......... .........T .......... .......... .......... ..........
O157_Sakai   ........A. .......... .......... .....G.... .......... .......... ..........
ECOR_35      ........A. .......... .......... .....G.... .......... .......... ..........
ECOR_36      ........A. .......... .......... .....G.... .......... .......... ..........
SMS-3-5      ........A. .......... .......... .....G.... .......... .......... ..........
536          ........A. .......... .......... .....G.... .......... .......... ..........
APEC_O1      ........A. .......... .......... .....G.... .......... .......... ..........
CFT073       ........A. .......... .......... .....G.... .......... .......... ..........
E2348/69     ........A. .......... .......... .....G.... .......... .......... ..........
ECOR_51      ........A. .......... .......... .....G.... .......... .......... ..........
ECOR_57      ........A. .......... .......... .....G.... .......... .......... ..........
UTI89        ........A. .......... .......... .....G.... .......... .......... ..........
E._albertii  ........A. .......... ..T..A.... .....G.... .A.....G.. ......A... .....G....
```

```
        14911
K-12          CGAGGAACTG CTCGGCCAGC AAGGCGTTGG ACGGGATTTC ACACATCATG ATGATTTTCA GCCCGTTCTC
53638         .......... .......... .......... .......... .......... .......... ..........
ATCC_8739     .......... .......... .......... .......... .......... .......... ..........
ECOR_10       .......... .......... .......... .......... .......... .......... ..........
ECOR_11       .......... .......... .......... .......... .......... .......... ..........
B171          .......... ...T...... .......... ....A..... .......... .......... ..........
E24377A       .......... ...T...... .......... ....A..... .......... .......... ..........
ECOR_34       .......... ...T...... .......... ....A..... .......... .......... ..........
ECOR_71       .......... ...T...... .......... ....A..... .......... .......... ..........
SE11          .......... ...T...... .......... ....A..... .......... .......... ..........
O157_Sakai    .......... .......... .......... .......... .......... .......... ..........
ECOR_35       .......... .......... .......... .......... .......... .......... ..........
ECOR_36       .......... .......... .......... .......... .......... .......... ..........
SMS-3-5       .......... .......... .......... .......... .......... .......... ..........
536           .C........ .......... .......... .......... .......... .......... ..........
APEC_O1       .C........ ...T...... .......... .......... .......... .......... ..........
CFT073        .C........ ...T...... .......... ....A..... .......... .......... ..........
E2348/69      .C........ ...T...... .......... ....A..... .......... .......... ..........
ECOR_51       .C........ ...T...... .......... ....A..... .......... .......... ..........
ECOR_57       .C........ ...T...... .......... ....A..... .......... .......... ..........
UTI89         .C........ ...T...... .......... .......... .......... .......... ..........
E._albertii   .......... T..A.....T ..A....... .......C.. .......... .......... .T..A..T..

        14981
K-12          GCCACGTTTC AGCCCCTGAC GCGCCAGTTC TTCAACCACC GCTTTCGCCT GATCTACGGT ACGCACGAAC
53638         A.....C... .......... .......... C......... .......... .......... ..........
ATCC_8739     A.....C... .......... .......... C......... .......... .......... ..........
ECOR_10       .......... .......... .......... .......... .......... .......... ..........
ECOR_11       .......... .......... .......... .......... .......... .......... ..........
B171          .......... .......... .......... .......... .......... .......... ..........
E24377A       .......... .......... .......... .......... .......... .......... ..........
ECOR_34       .......... .......... .......... .......... .......... .......... ..........
ECOR_71       .......... .......... .......... .......... .......... .......... ..........
SE11          .......... .......... .......... .......... .......... .......... ..........
O157_Sakai    A.....C... .......... .......... C......... .......... .......... ..........
ECOR_35       A......... .......... .......... C......... .......... .......... ..........
ECOR_36       A......... .......... .......... C......... .......... .......... ..........
SMS-3-5       A......... .......... .......... C......... .......... .......... ..........
536           A......... .........T .......... C......... .......... ....A..... ..........
APEC_O1       A......... .......... .......... C......... .......... ....A..... ..........
CFT073        A......... .........T .......... C......... .......... ....A..... ..........
E2348/69      .......... .......... .......... C......... .......... ....A..... ..........
ECOR_51       A......... .........T .......... C......... .......... ....A..... ..........
ECOR_57       A......... .........T .......... C......... .......... ....A..... ..........
UTI89         A......... .......... .......... C......... .......... ....A..... ..........
E._albertii   A......... .AG....... .......C.. .........A .......... .G..A..A.. ..........

        15051
K-12          GGGATCATGA TCTCAACGTT GGTCAGTCCC ATGTCGTTGC GCACACGTTT CACTGCTTCA CACTCCAGCG
53638         .......... .......... ......C... .......... .......... .........G ..........
ATCC_8739     .......... .......... ......C... .......... .......... .........G ..........
ECOR_10       .......... .......... .......... .......... .......... .......... ..........
ECOR_11       .......... .......... .......... .......... .......... .......... ..........
B171          .......... .......... ......C... .......... ....T..... .......... ..........
E24377A       .......... .......... ......C... .......... ....T..... .......... .....T....
ECOR_34       .......... .......... ......C... .......... ....T..... .......... ..........
ECOR_71       .......... .......... ......C... .......... ....T..... .......... ..........
SE11          .......... .......... ......C... .......... ....T..... .......... ..........
O157_Sakai    .......... .......... ......C... .......... .......... .......... ..........
ECOR_35       .......... .......... ......C... .......... .......... .......... ..........
ECOR_36       .......... .......... ......C... .......... .......... .......... ..........
SMS-3-5       .......... .......... ......C... .......... .......... .......... ..........
536           .......... .......... A.....C... .......... .......... .......... ..........
APEC_O1       .......... .......... A.....C... .......... .......... .......... ..........
CFT073        .......... .......... A.....C... .......... .......... .......... ..........
E2348/69      .......... .......... A.....C... .......... .......... .......... ..........
ECOR_51       .......... .......... A.....C... .......... .......... .......... ..........
ECOR_57       .......... .......... A.....C... .......... .......... .......... ..........
UTI89         .......... .......... A.....C... .......... .......... .......... ..........
E._albertii   .......... .T........ ......C... .......... ....G..... .......... ..........
```

```
         15121
K-12         CGAAACAGTC GCGGAAGCTG TCGGAAACAT AGCGGCCCGC GCCACGGAAG CCGAGCATCG GGTTCTCTTC
53638        .......... .......... .......... ....T..... .......... .......... ..........
ATCC_8739    .......... .......... .......... ....T..... .......... .......... ..........
ECOR_10      .......... .......... .......... .......... .......... .......... ..........
ECOR_11      .......... .......... .......... .......... .......... .......... ..........
B171         ....G..... .......... .......... .......... .......... .......... ..........
E24377A      ....G..... .......... .......... .......... .......... ........A. ..........
ECOR_34      ....G..... .......... .......... .......... .......... .......... ..........
ECOR_71      ....G..... .......... .......... .......... .......... .......... ..........
SE11         ....G..... .......... .......... .......... .......... .......... ..........
O157_Sakai   .......... .......... .......... ....T..... .......... .......... ..........
ECOR_35      ....G..... .......... .......... .......... .......... .......... ..........
ECOR_36      ....G..... .......... .......... .......... .......... .......... ..........
SMS-3-5      ....G..... .......... .......... .......... .......... .......... ..........
536          ....G..... .......... .......... .......... .......... .......... ..........
APEC_O1      ....G..... .......... .......... .......... .......... .......... ..........
CFT073       ....G..... .......... .......... .......... .......... .......... ..........
E2348/69     ....G..... .......... .......... .......... .......... .......... ..........
ECOR_51      ....G..... .......... .......... .......... .......... .......... ..........
ECOR_57      ....G..... .......... .......... .......... .......... .......... ..........
UTI89        ....G..... .......... .......... .......... .......... .......... ..........
E._albertii  ....G..A.. .......... ..A.....G. .A..C..G.. .........A ..C....... ....T.....

         15191
K-12         ATCTGGCTCG TAACGCTCAC CACCGACCAG GTTGGCATAT TCGTTCGATT TAAAATCAGA GAGACGGACA
53638        .......... .......... .......... .......... .......... .......... ..........
ATCC_8739    .......... .......... .......... .......... .......... .......... ..........
ECOR_10      .......... .......... .......... .......... .......... .......... ..........
ECOR_11      .......... .......... .......... .......... .......... .......... ..........
B171         .......... .......... .......... .......... .......... .......... ..........
E24377A      .........A .......... .......... .......... .......... .......... ..........
ECOR_34      .......... .......... .......... .......... .......... .......... ..........
ECOR_71      .......... .......... .......... .......... .......... .......... ..........
SE11         .......... .......... .......... .......... .......... .......... ..........
O157_Sakai   .........A .......... .......... .......... .......... .......... ..........
ECOR_35      .......... .......... .......... .......... .......... .......... ..........
ECOR_36      .......... .......... .......... .......... .......... .......... ..........
SMS-3-5      .......... .......... .......... .......... .......... .......... ..........
536          .......... .......... .......... A......... .......... .......... ..........
APEC_O1      .......... .......... .......... A......... .......... .......... A.........
CFT073       .......... .......... .......... A......... .......... .......... ..........
E2348/69     .......... .......... .......... A......... .......... .......... ..........
ECOR_51      .......... .......... .......... A......... .......... .......... ..........
ECOR_57      .......... .......... .......... A......... .......... .......... ..........
UTI89        .......... .......... .......... A......... .......... .......... A.........
E._albertii  ...C..T... ........G. .G.....G.. ......G... ..A..A.... ....G..... ......A...

         15261
K-12         ATGACGCGCT TCGGATAAAA CGCGGCACCC AGCGTCGCGA TCCCTTCAGT CAGACGACCA ACGTAAAATT
53638        .......... .......... .......... .......... .......... .......... ..........
ATCC_8739    .......... .......... .......... .......... .......... .......... ..........
ECOR_10      .......... .......... .......... .......... .......... .......... ..........
ECOR_11      .......... .......... .......... .......... .......... .......... ..........
B171         .......... .......... .......... .......... .......... .......... ..........
E24377A      .......... .......... .......... .......... .......... .......... ..........
ECOR_34      .......... .......... .......... .......... .......... .......... ..........
ECOR_71      .......... .......... .......... .......... .......... .......... ..........
SE11         .......... .......... .......... .......... .......... .......... ..........
O157_Sakai   .......... .......... .......... .......... .......... .......... ..........
ECOR_35      .......... .......... .......... .......... .......... ..A...C... ..........
ECOR_36      .......... .......... .......... .......... .......... ..A...C... ..........
SMS-3-5      .......... .......... .......... .......... .......... .......... ..........
536          .......... .......... .......... .......... .......... .......... ..........
APEC_O1      .......... .......... .......... .......... .......... .......... ..........
CFT073       ......T... .......... .......... .......... .......... .......... ..........
E2348/69     .......... .......... .......... .......... .......... ..A...C... ..........
ECOR_51      .......... .......... .......... .......... .......... .......... ..........
ECOR_57      .......... .......... .......... .......... .......... .......... ..........
UTI89        .......... .......... .......... .......... .......... .......... ..........
E._albertii  .......T.. .......... ...C..C... .......... .......C.. ......G..T ..A.....C.
```

```
        15331
K-12         CACGCGGAGA ATCAAAACCT TTCATCATCT CGCGGATTTC GTTTTGCAAC TGCGGTTCCT GATCGTCAAA
53638        .......... .......... .......... .......... .......... .......... ..........
ATCC_8739    .......... .......... .......... .......... .......... .......... ..........
ECOR_10      .......... .......... .......... .......... .......... .......... ..........
ECOR_11      .......... .......... .......... .......... .......... .......... ..........
B171         .......... .......... .......... .......... .......... .......... ..........
E24377A      .......... .......... .......... .......... .......... .......... ..........
ECOR_34      .......... .......... .......... .......... .......... .......... ..........
ECOR_71      .......... .......... .......... .......... .......... .......... ..........
SE11         .......... .......... .......... .......... .......... .......... ..........
O157_Sakai   .......... .......... .......... .......... .......... .......... ..........
ECOR_35      .......... .......... .......... .......... .......... .......... ..........
ECOR_36      .......... .......... .......... .......... .......... .......... ..........
SMS-3-5      .......... .......... .......... .......... .......... ........T. ..........
536          .......... .......... .......... .......... .......... .......... ..........
APEC_O1      .......... .......... .......... .......... .......... .......... ..........
CFT073       .......... .......... .......... .......... .......... .......... ..........
E2348/69     .......... .......... .......... .......... .......... .......... ..........
ECOR_51      .......... .......... .......... .......... .......... .......... ..........
ECOR_57      .......... .......... .......... .......... .......... .......... ..........
UTI89        .......... .......... .......... .......... .......... .......... ..........
E._albertii  .......... ...G...... .......... G...A..... .......... .......... .......G..

        15401
K-12         CTCAAGCAGT GCGCGTGGGT GGACGCCAAT CATACGGTTG ATGATAAATT CCAGACGCGC AAGGCCCACG
53638        .......... .......... .......... .......... .......... .......... ...T......
ATCC_8739    .......... .......... .......... .......... .......... .......... ...T......
ECOR_10      .......... .......... .......... .......... .......... .......... ..........
ECOR_11      .......... .......... .......... .......... .......... .......... ..........
B171         .......... .......... .......... .......... .......... .......... ..........
E24377A      .......... .......... .......... .......... .......... .......... ..........
ECOR_34      .......... .......... .......... .......... .......... .......... ..........
ECOR_71      .......... .......... .......... .......... .......... .......... ..........
SE11         .......... .......... .......... .......... .......... .......... ..........
O157_Sakai   .......... .......... .......... .......... .......... .......T.. ..........
ECOR_35      .......... .......... .......... .......... .......... .......... ..........
ECOR_36      .......... .......... .......... .......... .......... .......... ..........
SMS-3-5      .........C
536          .......... .......... .......... .......... .......... .......... ..........
APEC_O1      .......... .......... .......... .......... .......... .......... ..........
CFT073       .......... .......... .......... .......... .......... .......... ..........
E2348/69     .......... .......... .......... .......... .......... .......... ..........
ECOR_51      .......... .......... .......... .......... .......... .......... ..........
ECOR_57      .......... .......... .......... .......... .......... .......... ..........
UTI89        .......... .......... .......... .......... .......... .......... ..........
E._albertii  ......T.A. ..A..C.... .......... ...G...... .......... .......T.. C..C......

        15471
K-12         CCTTCGTTCG GTAGGCAGGC GAAGTCGAAA GCACGGTCCG GGTTACCGAC GTTCATCATC ACTTTCAACG
53638        .......... .C..A..... .......... ..T....... .......... .......... ..........
ATCC_8739    .......... .C..A..... .......... ..T....... .......... .......... ..........
ECOR_10      .......... .......... .......... .......... .......... .......... ..........
ECOR_11      .......... .......... .......... .......... .......... .......... ..........
B171         .......... .C........ .......... .......... .......... .......... ..........
E24377A      .......... .C........ .......... .......... .......... .......... ..........
ECOR_34      .......... .C........ .......... .......... .......... .......... ..........
ECOR_71      .......... .C........ .......... .......... .......... .......... ..........
SE11         .......... .C........ .......... .......... .......... .......... ..........
O157_Sakai   .......... .C........ .......... .......... .......... .......... ..........
ECOR_35      .......... .C........ .......... .....A.... .......... .......... ..C.......
ECOR_36      .......... .C........ .......... .....A.... .......... .......... ..C.......
SMS-3-5      .......... .C........ .......... .....A.... .......... .......... ..C.......
536          .......... .C........ .......... .......... .......... .......... ..C.......
APEC_O1      .......... .C........ .......... .......... .......... .......... ..C.......
CFT073       .......... .C........ .......... .......... .......... .......... ..C.......
E2348/69     .......... .C........ .......... .......... .A........ .......... ..C.......
ECOR_51      .......... .C........ .......... .......... .......... .......... ..C.......
ECOR_57      .......... .C........ .......... .......... .......... .......... ..C.......
UTI89        .......... .C........ .......... .......... .......... .......... ..C.......
E._albertii  .......... .C........ .......... .......... .......A.. .......... .......G..
```

```
        15541
K-12          GCAGATCCGG CATCGTTTCT ACGCTGGAGC TTTTCACGCT AAATTCCAGC AACTCCGCAT AGACGTAACC
53638         .......... .......... .......... .......... .......... .......... ..........
ATCC_8739     .......... .......... .......... .......... .......... .......... ..........
ECOR_10       .......... .......... .......... .......... .......... .......... ..........
ECOR_11       .......... .......... .......... .......... .......... .......... ..........
B171          .......... .......... .......... .......... .......... .......... ..........
E24377A       .......... .......... .......... .......... .......... .......... ..........
ECOR_34       .......... .......... .......... .......... .......... .......... ..........
ECOR_71       .......... .......... .......... .......... .......... .......... ..........
SE11          .......... .......... .......... .......... .......... .......... ..........
O157_Sakai    .......... .......... .......... .......... .......... .......... ..........
ECOR_35       ....G..... .......... .......... .......... .......... .......... ....A.....
ECOR_36       ....G..... .......... .......... .......... .......... .......... ....A.....
SMS-3-5       ....G..... .......... .......... .......... .......... .......... ....A.....
536           ....G..... .......... .......... .......... .......... .......... ..........
APEC_O1       ....G..... .......... .......... .......... .......... .......... ..........
CFT073        ....G..... .......... .......... .......... .......... .......... ..........
E2348/69      ....G..... .......... .......... .......... .......... .......... ..........
ECOR_51       ....G..... .......... .......... .......... .......... .......... ..........
ECOR_57       ....G..... .......... .......... .......... .......... .......... ..........
UTI89         ....G..... .......... .......... .......... .......... .......... ..........
E._albertii   .......... ...G.....C .......... .......... G..C...... ....A..... .A..A..G..

        15611
K-12          GGTATCACCT TCGGCACAAG AAACAGTGAC GTTCTCACCG TCTTTCATCC GTTCTGTTGC ATCTCCACAG
53638         .......... .......... .......... .......... .......... .......... ...A......
ATCC_8739     .......... .......... .......... .......... .......... .......... ...A......
ECOR_10       .......... .......... .......... .......... .......... .......... ..........
ECOR_11       .......... .......... .......... .......... .......... .......... ..........
B171          .......... .......... .......... T......... .......... .......... ..........
E24377A       .......... .......... .......T.. .......... .......... .......... ...A......
ECOR_34       .......... .......... .......... .......... .......... .......... ...A......
ECOR_71       .......... .......... .......... .......... .......... .......... ...A......
SE11          .......... .......... .......... .......... .......... .......... ...A......
O157_Sakai    .......... .......... .......... .......... .......... .......... ...A......
ECOR_35       .......... .......... .......... .......... .......... .......... ...A......
ECOR_36       .......... .......... .......... .......... .......... .......... ...A......
SMS-3-5       .......... .......... .......... .......... .......... .......... ...A......
536           .......... .......... .......... ......G... .......... .......... ...A......
APEC_O1       .......... .......... .......... ......G... .......... .......... ...A......
CFT073        .......... .......... .......... ......G... .......... .......... ...A......
E2348/69      .......... .......... .......... ......G... .......... .......... ...A......
ECOR_51       .......... .......... .......... ......G... .......... .......... ...A......
ECOR_57       .......... .......... .......... ......G... .......... .......... ...A......
UTI89         .......... .......... .......... ......G... .......... .......... ...A......
E._albertii   .......... .......... ....G..... .......... .......... ....G..C.. ...A......

        15681
K-12          CCCACTACCG CCGGAATGCC CAGTTCACGA GCGATGATCG CCGCGTGACA GGTACGACCG CCACGGTTGG
53638         .......... .......... .......... .......... .......... .......... ..........
ATCC_8739     .......... .......... .......... .......... .......... .......... ..........
ECOR_10       .......... .......... .......... .......... .......... .......... ..........
ECOR_11       .......... .......... .......... .......... .......... .......... ..........
B171          .......... .......... .......... .......... .......... .......... ..........
E24377A       .......... .......... .......... .......... .......... .......... ..........
ECOR_34       .......... .......... .......... .......... .......... .......... ..........
ECOR_71       .......... .......... .......... .......... .......... .......... ..........
SE11          .......... .......... .......... .......... .......... .......... ..........
O157_Sakai    .......... .......... .......... .......... .......... .......... ..........
ECOR_35       .......... .......... G......... .......... .......... .......... ..........
ECOR_36       .......... .......... G......... .......... .......... .......... ..........
SMS-3-5       .......... .......... G......... .......... .......... .......... ..........
536           .......... .......... G......... .......... .......... .......... ..........
APEC_O1       .......... .......... G......... .......... .......... .......... ..........
CFT073        .......... .......... G......... .......... .......... .......... ..........
E2348/69      .......... .......... G......... .......... .......... .......... .....A....
ECOR_51       .......... .......... G......... .......... .......... .......... ..........
ECOR_57       .......... .......... G......... .......... .......... .......... ..........
UTI89         .......... .......... G......... .......... .......... .......... ..........
E._albertii   ..A..C.... .......A.. .........C .......... ....A..... .......... .....A....
```

```
         15751
K-12          TGACGATGGC AGATGCTTTC TTCATGATCG GTTCCCAGTC CGGGTCGGTC ATGTCAGTAA CCAGCACGTC
53638         .......... .......... .......... .......... .......... ........G. ..........
ATCC_8739     .......... .......... .......... .......... .......... ........G. ..........
ECOR_10       .......... .......... .......... .......... .......... .......... ..........
ECOR_11       .......... .......... .......... .......... .......... .......... ..........
B171          .......... .......... .......... .......... .......... .......... ..........
E24377A       .......... .......... .......... .......... .......... ........G. ..........
ECOR_34       .......... .......... .......... .......... .......... ........G. ..........
ECOR_71       .......... .......... .......... .......... .......... ........G. ..........
SE11          .......... .......... .......... .......... .......... ........G. ..........
O157_Sakai    .......... .......... .......... .......... .......... ........G. ..........
ECOR_35       .......... .......... .......... .......... .......... .......... ..........
ECOR_36       .......... .......... .......... .......... .......... .......... ..........
SMS-3-5       .......... .......... .......... .......... .......... .......... ..........
536           .......... .......... .......... .......... .......... .......... ..........
APEC_O1       .......... .......... .......... .......... .......... .......... ..........
CFT073        .......... .......... .......... .......... .......... .......... ..........
E2348/69      .......... .......... .......... .......... .......... .......... ..........
ECOR_51       .......... .......... .......... .......... .......... .......... ..........
ECOR_57       .......... .......... .......... .......... .......... .......... ..........
UTI89         .......... .......... .......... .......... .......... .......... ..........
E._albertii   ....A..... .......... .......... .......... .......... .......... ..........

         15821
K-12          GCCAGGTTCG ATGCGGTTCA TTTCGCTGAT GTCATGGATG ACTTTCACCG GACCCGCACC GATGCGATGA
53638         A......... .......... .......... A......... .......... .......... ..........
ATCC_8739     A......... .......... .......... A......... .......... .......... ..........
ECOR_10       .......... .......... .......... .......... .......... .......... ..........
ECOR_11       .......... .......... .......... .......... .......... .......... ..........
B171          .......... .......... .......... .......... .......... .......G.. ..........
E24377A       .......... .......... .......... .......... .......... .......... ..........
ECOR_34       .......... .......... .......... .......... .......... .......G.. ..........
ECOR_71       .......... .......... .......... .......... .......... .......G.. ..........
SE11          .......... .......... .......... .......... .......... .......G.. ..........
O157_Sakai    A......... .......... .......... A......... .......... .......... ..........
ECOR_35       A......... .......... .......... .......... .......... .......G.. ..........
ECOR_36       A......... .......... .......... .......... .......... .......G.. ..........
SMS-3-5       A......... .......... .......... .......... .......... .......G.. ..........
536           .......... .......... .......... .......... .......... .......G.. ..........
APEC_O1       .......... .......... .......... .......... .......... .......G.. ..........
CFT073        .......... .......... .......... .......... .......... .......G.. ..........
E2348/69      .......... .......... .......... .......... .......... .......G.. ..........
ECOR_51       .......... .......... .......... .......... .......... .......G.. ..........
ECOR_57       .......... .......... .......... .......... .......... .......G.. ..........
UTI89         .......... .......... .......... .......... .......... .......G.. ..........
E._albertii   ...T...... .......... .......... .......... .......... .......G.. A.........

         15891
K-12          CCGATAGCAC GGCCTTCGGC GATAATCTTA CCCTGTGAAT GCAGCGTATA ACGCTCCATG ACCTGACCGC
53638         .......... .......... .......... .......... .......... .......... ..........
ATCC_8739     .......... .......... .......... .......... .......... .......... ..........
ECOR_10       .......... .......... .......... .......... .......... .......... ..........
ECOR_11       .......... .......... .......... .......... .......... .......... ..........
B171          .......... .......... .......... .......... .......... .......... ..........
E24377A       .......... .......... .......... .......... .......... .......... ..........
ECOR_34       .......... .......... .......... .......... .......... .......... ..........
ECOR_71       .......... .......... .......... .......... .......... .......... ..........
SE11          .......... .......... .......... .......... .......... .......... ..........
O157_Sakai    .......... .......... .......... .......... .......... .......... ..........
ECOR_35       .......... .......... .......... .......... .......... .......... ..........
ECOR_36       .......... .......... .......... .......... .......... .......... ..........
SMS-3-5       .......... .......... .......... .......... .......... .......... ..........
536           .......... .......... .......... .......... .......... .......... ..........
APEC_O1       .......... .......... .......... .......... .......... .......... ..........
CFT073        .......... .......... .......... .......... .......... .......... ..........
E2348/69      .......... .......... .......... .......... .......... .......... ..........
ECOR_51       .......... .......... .......... .......... .......... .......... ..........
ECOR_57       .......... .......... .......... .......... .......... .......... ..........
UTI89         .......... .......... .......... .......... .......... .......... ..........
E._albertii   ........G. .A.....A.. .......... .......... ....G..... ...T.....A .....G....
```

```
         15961
K-12           GTGAGCGCAC GGTTTCCGGA CGCGCCTGCA CAATGAACAG TTTACCGGTG TGGCCATCTT TCGCCCACTC
53638          .......... .......... .......... .......... .......... .......... ..........
ATCC_8739      .......... .......... .......... .......... .......... .......... ..........
ECOR_10        .......... .......... .......... .......... .......... .......... ..........
ECOR_11        .......... .......... .......... .......... .......... .......... ..........
B171           .......... .......... .......... .......... .......... .......... ..........
E24377A        .......... .......... .......... .......... ...G...... .......... ..........
ECOR_34        .......... .......... .......... .......... ...G...... .......... ..........
ECOR_71        .......... .......... .......... .......... ...G...... .......... ..........
SE11           .......... .......... .......... .......... ...G...... .......... ..........
O157_Sakai     .......... .......... .......... .......... ......A... .......... ..........
ECOR_35        .......... .......... .......... .......... ...G...... .......... ..........
ECOR_36        .......... .......... .......... .......... ...G...... .......... ..........
SMS-3-5        .......... .......... .......... .......... ...G...... .......... ..........
536            .......... .......... .......... .......... ...G...... .......... ..........
APEC_O1        .......... .......... .......... .......... ...G...... .......... ..........
CFT073         .......... .......... .......... .......... ...G...... .......... ..........
E2348/69       .......... .......... .......... .......... ...G...... .......... ..........
ECOR_51        .......... .......... .......... .......... ...G...... .......... ..........
ECOR_57        .......... .......... .......... .......... ...G...... .......... ..........
UTI89          .......... .......... .......... .......... ...G...... .......... ..........
E._albertii    ....A..... .........G ........A. ....A..... ...G.....A ..A....... ..........

         16031
K-12           AATATCCATC GGGCGACCGT AGTGTTTCTC AATTTGTACG GCCTGTTTTG CCAGTTCCTG CACTTCTTCG
53638          .......... .......... .......... .......... .......... .......... ..........
ATCC_8739      .......... .......... .......... .......... .......... .......... ..........
ECOR_10        .......... .......... .......... .......... .......... .......... ..........
ECOR_11        .......... .......... .......... .......... .......... .......... ..........
B171           .........T .......... .......... .......... .......... .......... ..........
E24377A        .......... .......... .......... .......... .......... .......... ..........
ECOR_34        .......... .......... .......... .......... .......... .......... ..........
ECOR_71        .......... .......... .......... .......... .......... .......... ..........
SE11           .......... .......... .......... .......... .......... .......... ..........
O157_Sakai     .......... .......... .......... .......... .......... .......... ..........
ECOR_35        .......... .......... .......... .......... .......... .......... ..........
ECOR_36        .......... .......... .......... .......... .......... .......... ..........
SMS-3-5        .......... .......... .......... .......... .......... .......... ..........
536            .......... .......... .......... .......... .......... .......... ..........
APEC_O1        .......... .......... .......... .......... .......... .......... ..........
CFT073         .......... .......... .......... .......... .......... .......... ..........
E2348/69       .......... .......... .......... .......... .......... .......... ..........
ECOR_51        .......... .......... .......... .......... .......... .......... ..........
ECOR_57        .......... .......... .......... .......... .......... .......... ..........
UTI89          .......... .......... .......... .......... .......... .......... ..........
E._albertii    .......... ..A....... .......... .......... .......... ....C..... ......A...

         16101
K-12           TTGGTCAGCG AGAAGATGTC ACGCTGTTCC TGCGGTACGT CTTCGATTTT AACCTGCTTG CCGTGCTCCT
53638          .......... .......... .......... .......... .......... .......... ..........
ATCC_8739      .......... .......... .......... .......... .......... .......... ..........
ECOR_10        .......... .......... .......... .......... .......... .......... ..........
ECOR_11        .......... .......... .......... .......... .......... .......... ..........
B171           .......... .......... .......... .......... .......... .......... ..........
E24377A        .......... .......... .......... .......... .......... .......... ..........
ECOR_34        .......... .......... .......... .......... .......... .......... ..........
ECOR_71        .......... .......... .......... .......... .......... .......... ..........
SE11           .......... .......... .......... .......... .......... .......... ..........
O157_Sakai     .......... .......... .......... .......... .......... .......... ..A.......
ECOR_35        .......... .......... .......... .......... .......... .......... ..........
ECOR_36        .......... .......... .......... .......... .......... .......... ..........
SMS-3-5        .......... .......... .......... .......... .......... .......... ..........
536            .......... .......... .......... .......... .......... .......... ..........
APEC_O1        .......... .......... .......... .......... .......... .......... ..........
CFT073         .......... .......... .......... .......... .......... .......... ..........
E2348/69       .......... .......... .......... .......... .......... .......... ..........
ECOR_51        .......... .......... .......... .......... .......... .......... ..........
ECOR_57        .......... .......... .......... .......... .......... .......... ..........
UTI89          .......... .......... .......... .......... .......... .......... ..........
E._albertii    ........T. .......A.. G......... .....C.... .......... .......... ..A..T....
```

```
             16171
K-12         GGGTCGGCGC GTAAACCATG CGGATTTTTT TCGACCCCAT GGTGCGGCGC ACGATAGCCG GGCGATTCGC
53638        .......... .......... .......... .......... .......... .......... ..........
ATCC_8739    .......... .......... .......... .......... .......... .......... ..........
ECOR_10      .......... .......... .......... .......... .......... .......... ..........
ECOR_11      .......... .......... .......... .......... .......... .......... ..........
B171         .......... .......... .......... .......... .......... .......... ..........
E24377A      .......... .......... .......... .......... ......A... .......... ..........
ECOR_34      .......... .......... .......... .......... ......A... .......... ..........
ECOR_71      .......... .......... .......... .......... ......A... .......... ..........
SE11         .......... .......... .......... .......... ......A... .......... ..........
O157_Sakai   .......... .......... .......... .......... .......... .......... ..........
ECOR_35      .......... .......... .......... .......... .......... .......... ..........
ECOR_36      .......... .......... .......... .......... .......... .......... ..........
SMS-3-5      .......... .......... .......... .......... .......... .......... ..........
536          .......... .......... .......... .......... .......... .......... ..........
APEC_O1      .......... .......... .......... .......... .......... .......... ..........
CFT073       .......... .......... .......... .......... .......... ..A....... ..........
E2348/69     .......... .......... .......... .......... .......... .......... ..........
ECOR_51      .......... .......... .......... .......... .......... ..A....... ..........
ECOR_57      .......... .......... .......... .......... .......... ..A....... ..........
UTI89        .......... .......... .......... .......... .......... .......... ..........
E._albertii  .......... A..T.....A .......... .......... ......A... .......... ..........

             16241
K-12         CGCCAGTGTC GGTTTATGCA CGTAAAACTC ATCCGGGTTA ACCGCACCCT GCACGACCAT CTCACCAAGG
53638        .......... .......... .......... .......... .......... .......... ..........
ATCC_8739    T..A...... ........G. .......... .......... .....G.... .......... T.........
ECOR_10      .......... .......... .......... .......... .......... .......... ..........
ECOR_11      .......... .......... .......... .......... .......... .......... ..........
B171         .......... ........G. .......... .......... .......... .......... T..C......
E24377A      .T........ ........G. .......... .......... .....G.... .......... T.........
ECOR_34      T..A...... ........G. .......... .......... .....G.... .......... T.........
ECOR_71      T..A...... ........G. .......... .......... .....G.... .......... T.........
SE11         T..A...... ........G. .......... .......... .....G.... .......... T.........
O157_Sakai   .......... .......... .......... .......... .......... .......... ..........
ECOR_35      .......... .....G.... .......... .......... .......... .......... T.........
ECOR_36      .......... .....G.... .......... .......... .......... .......... T.........
SMS-3-5      .......... .....G.... .......... .......... .......... .......... T.........
536          .......... .....G.... .......... .......... .......... .......... T.........
APEC_O1      ...A...... T......... .A........ ......A... .......... .......... T.........
CFT073       ...A...... .......... .A........ .......... .......... .......... T.........
E2348/69     ...A...... .......... .A........ .......... .......... .......... T.........
ECOR_51      ...A...... .......... .A........ .......... .......... .......... T.........
ECOR_57      ...A...... .......... .A........ .......... .......... .......... T.........
UTI89        ...A...... T......... .A........ ......A... .......... .......... T.........
E._albertii  ......C... ........G. ....G..T.. .......... ..A..C.... ....C..... T........C

             16311
K-12         CCCCATGCGG AAGTGATAAA CACCACCTGG TCAAAGCCGG ATTCGGTATC AATGGAGAAC ATCACGCCAG
53638        .......... .......... .......... .......... .......... .......... ..........
ATCC_8739    .......... .......... .......... .......... .......... .......... ..........
ECOR_10      .......... .......... .......... .......... .......... .......... ..........
ECOR_11      .......... .......... .......... .......... .......... .......... ..........
B171         .......... .......... .......... .......... .......... .......... ..A.......
E24377A      .......... .......... .......... .......... .......... .......... ..........
ECOR_34      .......... .......... .......... .......... .......... .......... ..........
ECOR_71      .......... .......... .......... .......... .......... .......... ..........
SE11         .......... .......... .......... .......... .......... .......... ..........
O157_Sakai   .......... .......... .......... .......... .......... .......... ..........
ECOR_35      .......... .......... .......... .......... .......... .......... ..........
ECOR_36      .......... .......... .......... .......... .......... .......... ..........
SMS-3-5      .......... .......... .......... .......... .......... .......... ..........
536          .......... .......... .......... .......... .......... .......... ..........
APEC_O1      .......... .......... .......... .......... .......... .......... ..........
CFT073       .......... .......... .......... ........A. .......... .......... ..........
E2348/69     .......... .......... .......... .......... .......... .......... ..........
ECOR_51      .......... .......... .......... ........A. .......... .......... ..........
ECOR_57      .......... .......... .......... ........A. .......... .......... ..........
UTI89        .......... .......... .......... .......... .......... .......... ..........
E._albertii  .......... .......... .......... ..G....... .......... .......... ..........
```

```
         16381
K-12         ATGATGCGAG GTCAGAGCGC ACCATCCGTT GAACACCGGC GGAGAGCGCC ACACCACGGT GATCGTAACC
53638        .......... .......... .......... .......... .......... .......... ..........
ATCC_8739    .A........ .......... .......... .......... .......... .......... ..........
ECOR_10      .......... .......... .......... .......... .......... .......... ..........
ECOR_11      .......... .......... .......... .......... .......... .......... ..........
B171         .......... .......... .......... .......... .......... ..G....... ..........
E24377A      .A........ .......... .......... .......... .......... .......... ..........
ECOR_34      .A........ .......... .......... .......... .......... ..G....... ..........
ECOR_71      .A........ .......... .......... .......... .......... ..G....... ..........
SE11         .A........ .......... .......... .......... .......... .......... ..........
O157_Sakai   .A........ .......... .......... .......... .......... .......... ..........
ECOR_35      .A........ ...G...... ........C. .......... .......... ..G....... ..........
ECOR_36      .A........ ...G...... ........C. .......... .......... ..G....... ..........
SMS-3-5      .A........ ...G...... ........C. .......... .......... ..G....... ..........
536          .A........ ...G...... ........C. .......... .......... ..G....... ..........
APEC_O1      .A........ ...G...... ........C. .......... .........T ..G.....A. .G........
CFT073       .A........ ...G...... ........C. .......... .........T ..G.....A. .G........
E2348/69     .A........ ...G...... ........C. .......... .........T ..G.....A. .G........
ECOR_51      .A........ ...G...... ........C. .......... .........T ..G.....A. .G........
ECOR_57      .A........ ...G...... ........C. .......... .........T ..G.....A. .G........
UTI89        .A........ ...G...... ........C. .......... .........T ..G.....A. .G........
E._albertii  .A..C..A.. A..G..A..T .......AC. .......C.. A......... ..G..G..A. .G........

         16451
K-12         CTGGTGCACA CGATAAGAGA TGGCGCGATC GTTAAACAGA GAAGCAAATA CATGTTTCAC TGCCACGAGA
53638        .......... .......... .......... .......... .......... .......... ..........
ATCC_8739    .......... .......... .......... .......... .......... .......... ..........
ECOR_10      .......... .......... .......... .......... .......... .......... ..........
ECOR_11      .......... .......... .......... .......... .......... .......... ..........
B171         .......... .......... .......... .......... .......... .......... ..........
E24377A      .......... .......... .......... .......... .......... .......... ..........
ECOR_34      .......... .......... .......... .......... .......... .......... ..........
ECOR_71      .......... .......... .......... .......... .......... .......... ..........
SE11         .......... .......... .......... .......... .......... .......... ..........
O157_Sakai   .......... .......... .......... .......... .......... .......... ..........
ECOR_35      .......... .......... .......... .......... .......... .......... ..........
ECOR_36      .......... .......... .......... .......... .......... .......... ..........
SMS-3-5      .......... .......... .......... .......... .......... .......... ..........
536          .......... .......... .......... .......... .......... .......... ..........
APEC_O1      .......... .......... .......... .......... .......... ....C..... ..........
CFT073       .......... .......... .......... .......... .......... ....C..... ..........
E2348/69     .......... .......... .......... .......... .......... ....C..... ..........
ECOR_51      .......... .......... .......... .......... .......... ....C..... ..........
ECOR_57      .......... .......... .......... .......... .......... ....C..... ..........
UTI89        .......... .......... .......... .......... .......... ....C..... ..........
E._albertii  ......A..G .......... .......... .........C ........G. .G........ C.........

         16521
K-12         ACGGCGTCAA AACCCTGAAC GTTGAGGAAG GTTTCCTGCT GACCGGCAAA AGAAGCGTCC GGCATATCTT
53638        .......... .......... .......... .......... .......... .......... ..........
ATCC_8739    .......... .......... .......... .......... .......... G......... ..........
ECOR_10      .......... .......... .......... .......... .......... .......... ..........
ECOR_11      .......... .......... .......... .......... .......... .......... ..........
B171         .......... .C........ .......... .......... .......... G......... ..........
E24377A      .......... .......... .......... .......... .......... .......... .....G....
ECOR_34      .......... .......... .......... .......... .......... G......... ..........
ECOR_71      .......... .......... .......... .......... .......... G......... ..........
SE11         .......... .......... .......... .......... .......... G......... ..........
O157_Sakai   .......... .......... .......... .......... .......... .......... ..........
ECOR_35      .......... .......... .......... .......... .......... .......... ..........
ECOR_36      .......... .......... .......... .......... .......... .......... ..........
SMS-3-5      .......... .......... .......... .......... .......... .......... ..........
536          .......... .......... .........A .......... .......... .......... ..........
APEC_O1      .......... .......... .........A .......... .......... .......... ..........
CFT073       .......... .......... .........A .......... .......... .......... ..........
E2348/69     .......... .......... .........A .......... .......... .......... ..........
ECOR_51      .......... .......... .........A .......... .......... .......... ..........
ECOR_57      .......... .......... .........A .......... .......... .......... ..........
UTI89        .......... .......... .........A .......... .......... .......... ..........
E._albertii  ..A..A.... .......C.. .......... .......... .......... G......... .....G....
```

```
         16591
K-12         CTGCGGTGGC GGAGGAGCGC ACCGCAAAAG AGGCGTTTTC GTCATCGGCG GAAAGCTGTG CATAGGCTTC
53638        .......... .......... .......... .......... .......... .......... ..........
ATCC_8739    ....A..A.. .......... .......... .......... .......... .......... ....A.....
ECOR_10      .......... .......... .......... .......... .......... .......... ..........
ECOR_11      .......... .......... .......... .......... .......... .......... ..........
B171         .......... .......... .......... .......... .......... .......... ..........
E24377A      .......... .........T .......... .......... .......... .......... ....A.....
ECOR_34      ....A..A.. .......... .......... .......... .......... .......... ....A.....
ECOR_71      ....A..A.. .......... .......... .......... .......... .......... ....A.....
SE11         ....A..A.. .......... .......... .......... .......... .......... ....A.....
O157_Sakai   .......... .......... .......... .......... .......... .......... ..........
ECOR_35      ....A..A.. ......A... ........C. .......... .......... .......... ..........
ECOR_36      ....A..A.. ......A... ........C. .......... .......... .......... ..........
SMS-3-5      ....A..A.. ......A... ........C. .......... .......... .......... ..........
536          ....A..A.. ......A... ........C. .A........ .......... .......... ..........
APEC_O1      ....A..A.. ......A... ........C. .A........ .......... .........C ..........
CFT073       ....A..A.. ......A... ........C. .A........ .......... .......... ..........
E2348/69     ....A..A.. ......A... ........C. .A........ .......... .......... ..........
ECOR_51      ....A..A.. ......A... ........C. .A........ .......... .......... ..........
ECOR_57      ....A..A.. ......A... ........C. .A........ .......... .......... ..........
UTI89        ....A..A.. ......A... ........C. .A........ .......... .........C ..........
E._albertii  .C..A..... A.....C... .......... .......... ......A... .....T.... ..........

         16661
K-12         GCGGATGGCG TTTTCCAGCT CAGGCTGGAA GGGAGTGTCG ATAATCCACT GGCGGATTTG CGCGCCCGCT
53638        .......... .......... .......... .......... .......... .......... ..........
ATCC_8739    .........A .......... .......... .......... .......... .......... ..........
ECOR_10      .......... .......... .......... .......... .......... .......... ..........
ECOR_11      .......... .......... .......... .......... .......... .......... ..........
B171         .......... .......... .......... .......... .......... .......... ..........
E24377A      ...T...... .......... .......... .......... .......... .......... ..........
ECOR_34      .........A .......... .......... .......... .......... .......... ..........
ECOR_71      .........A .......... .......... .......... .......... .......... ..........
SE11         .........A .......... .......... .......... .......... .......... ..........
O157_Sakai   .......... .......... .......... .......... .......... .......... ..........
ECOR_35      .......... .......... .......... .......... .......... .......... ..........
ECOR_36      .......... .......... .......... .......... .......... .......... ..........
SMS-3-5      .......... .......... .......... .......... .......... .......... ..........
536          .......... .......... .......... .......... .......... .......... ..........
APEC_O1      .......... .......... .......... .......... .......... .......... ..........
CFT073       .......... .......... .......... .......... .......... .......... ..........
E2348/69     .......... .......... .......... .......... .......... .......... ..........
ECOR_51      .......... .......... .......... .......... .......... .......... ..........
ECOR_57      .......... .......... .......... .......... .......... .......... ..........
UTI89        .......... .......... .......... .......... .......... .......... ..........
E._albertii  .........A .......... .......... .......... .......... .......... ...C......

         16731
K-12         TTCGCAAGCT GAGTAACATC GTCAATATCC GTTTTATCCA GCAGTTCATA AATGCGCTGG TTTACGCCGC
53638        .......... .......... .......... .......... .......... .......... ..........
ATCC_8739    .......... .G........ .......... .......... .......... .......... ..........
ECOR_10      .......... .......... .......... .......... .......... .......... ..........
ECOR_11      .......... .......... .......... .......... .......... .......... ..........
B171         .......... .G........ .......... .......... .......... .......... ..........
E24377A      .......... .G........ .......... .......... .......... .......... ..........
ECOR_34      .......... .G........ .......... .......... .......... .......... ..........
ECOR_71      .......... .G........ .......... .......... .......... .......... ..........
SE11         .......... .G........ .......... .......... .......... .......... ..........
O157_Sakai   .......... .......... .......... .......... .......... .......... ..........
ECOR_35      .......... .G........ .......... .......... .......... .......... ..........
ECOR_36      .......... .G........ .......... .......... .......... .......... ..........
SMS-3-5      .......... .G........ .......... .......... .......... .......... ..........
536          .......... .G........ .......... .......... .......... .......... ..........
APEC_O1      .......... .G........ .......... .......... .......... .......... ..........
CFT073       .......... .G........ .......... .......... .......... .......... ..........
E2348/69     .......... .G........ .......... .......... .......... .......... ..........
ECOR_51      .......... .G........ .......... .......... .......... .......... ..........
ECOR_57      .......... .G........ .......... .......... .......... .......... ..........
UTI89        .......... .G........ .......... .......... .......... .......... ..........
E._albertii  .......... .G..T..... .......... .......... .......... .......... ..........
```

```
         16801
K-12          TTTGGTCCAG AAACTGGTTA AACGCGTCGG CGGTTGTGGC GAAACCATTC GGAACGGAAA TTGCGCAAAT
53638         .......... .......... .......... .......... .......... .......... ..........
ATCC_8739     .......... .......... .......... .......... .......... .......... ..........
ECOR_10       .......... .......... .......... .......... .......... .......... ..........
ECOR_11       .......... .......... .......... .......... .......... .......... ..........
B171          .......... .......... .......... .......... .......... .......... .........C
E24377A       .......... .......... .......... .......... A......... .......... .........C
ECOR_34       .......... .......... .......... .......... .......... .......... .........C
ECOR_71       .......... .......... .......... .......... .......... .......... .........C
SE11          .......... .......... .......... .......... .......... .......... .........C
O157_Sakai    .......... .......... .......... .......... .......... .......... ..........
ECOR_35       .......... .......... .......... .......... .......... .......... ..........
ECOR_36       .......... .......... .......... .......... .......... .......... ..........
SMS-3-5       .......... .......... .......... .......... .......... .......... ..........
536           .......... .......... .......... .......... .......... .......... ..........
APEC_O1       .......... .......... .......... .......... .......... .......... ..........
CFT073        .......... .......... .......... .......... .......... .......... ..........
E2348/69      .......... .......... .......... .......... .......... .......... ..........
ECOR_51       .......... .......... .......... .......... .......... .......... ..........
ECOR_57       .......... .......... .......... .......... .......... .......... ..........
UTI89         .......... .......... .......... .......... .......... .......... ..........
E._albertii   ....A..... .........G ..T..A.... .......... A......... .......... ----------

         16871
K-12          CTGCTCTCTT CGACAATTTC CACATAAACC ATCGGTATAA ACGAAACCAT AAAAATGAAA TGCTGTTTTC
53638         .......... .......... .......... .......... .......... .......... ..........
ATCC_8739     .......... .......... .......... .......... .......... .......... ..........
ECOR_10       .......... .......... .......... .......... .......... .......... ..........
ECOR_11       .......... .......... .......... .......... .......... .......... ..........
B171          .......A.. .......... .......... .......... .......... .......... ..........
E24377A       .......A.. ......A... .......... .......... .......... .......... ..........
ECOR_34       .......A.. .......... .......... .......... .......... .......... ..........
ECOR_71       .......A.. .......... .......... .......... .......... .......... ..........
SE11          .......A.. .......... .......... .......... .......... .......... ..........
O157_Sakai    .......T.. .......... .......... .......... .......... .......... ..........
ECOR_35       .......T.. .......... .......... .......... .......... .......... ..........
ECOR_36       .......T.. .......... .......... .......... .......... .......... ..........
SMS-3-5       .......... .......... .......... .......... .......... .......... ..........
536           .......... .......... .......... .......... .......... .......... ..........
APEC_O1       .......... .......... .......... .......... .......... .......... ..........
CFT073        .......... .......... .......... .......... .......... .......... ..........
E2348/69      .......... .......... .......... .......... .......... .......... .A........
ECOR_51       .......... .......... .......... .......... .......... .......... ..........
ECOR_57       .......T.. .......... .......... .......... .......... .......... ..........
UTI89         .......... .......... .......... .......... .......... .......... ..........
E._albertii   -----..... ...TG...C. .......... .......... .T.G.GTG.- .......... ...A......

         16941
K-12          ATAAAAAATA AAATTGAAGG TTCATTTTAT AAACCAGACA TAACGTTCTA CGCTTCTGTG CGTTTTTAAT
53638         .......... .......... .......... .......... .......... .......... ..........
ATCC_8739     .......... .......... .......... .......... ......C... .......... ..........
ECOR_10       .......... .......... .......... .......... .......... .......... ..........
ECOR_11       .......... .......... .......... .......... .......... .......... ..........
B171          .......... .......... .......... .......... .T....C... .......... ..........
E24377A       .......... .......... .......... .......... .T....C... .......... ..........
ECOR_34       .......... .......... .......... .......... .T....C... .......... ..........
ECOR_71       .......... .......... .......... .......... .T....C... .......... ..........
SE11          .......... .......... .......... .......... .T....C... .......... ..........
O157_Sakai    .......... .......... .......... .......... .......... .......... ..........
ECOR_35       .......... .....T...T .......... .......... .......... .......... ..........
ECOR_36       .......... .....T...T .......... .......... .......... .......... ..........
SMS-3-5       .......... .......... .......-.. .......... .......... .......... ..........
536           .......... .....T...T .......... .......... .......... .......... ..........
APEC_O1       .......... .....T...T .......... .......... .......... .......... ..........
CFT073        .......... .....T...T .......... .......... .......... .........A ..........
E2348/69      .......... .....T...T .......... .......... .......... .......... ..........
ECOR_51       .......... .....T...T .......... .......... .......... .........A ..........
ECOR_57       .......... .....T...T .......... .......... .......... .........A ..........
UTI89         .......... .....T...T .......... .......... .......... .......... ..........
E._albertii   .......... .....AC... .......... ...AT.AG.. .CT.CA.A.. .......TCA .A........
```

```
        17011
K-12          TTATGCTTTC ATAGAATTAT GTCTGCATCA CGGGAAGAAC AAAATGGATA ATGCTGTTGA TCGCCACGTT
53638         .......... .......... .......... .......... .......... .......... ..........
ATCC_8739     .......... .......... .......... .......... .......... .......... ..........
ECOR_10       .......... .......... .......... .......... .......... .......... ..........
ECOR_11       .......... .......... .......... .......... .......... .......... ..........
B171          .......... .......... .......... .......... .......... .......... ..........
E24377A       .......... .......... .......... .......... .......... .......... ..........
ECOR_34       .......... .......... .......... .......... .......... .......... ..........
ECOR_71       .......... .......... .......... .......... ........A. .......... ..........
SE11          .......... .......... .......... .......... .......... .......... ..........
O157_Sakai    .......... .......... .......... .......... .......... .......... ..........
ECOR_35       .......... .......... .......... .......... .......... .......... ..........
ECOR_36       .......... .......... .......... .......... .......... .......... ..........
SMS-3-5       .......... .......... .......... .......... .......... .......... ..........
536           .......... .......... .......... .......... .......... .......... ..........
APEC_O1       .......... .......... .......... .......... .......... .......... ..........
CFT073        .......... .......... .......... .......... .......... .......... ..........
E2348/69      .......... .......... .......... .......... .......... .......... ..........
ECOR_51       .......... .......... .......... .......... .......... .......... ..........
ECOR_57       .......... .......... .......... .......... .......... .......... ..........
UTI89         .......... .......... .......... .......... .......... .......... ..........
E._albertii   .......... .......... .......... .......... .......... .......... .........A

        17081
K-12          TTTTATATTT CTGATGGTAC GGCAATAACT GCGGAGGTAT TAGGACACGC AGTAATGTCA CAATTTCCCG
53638         .......... .......... .......... .......... .......... .......... ..........
ATCC_8739     .......... .......... .......... .......... .......... .......... ..........
ECOR_10       .......... .......... .......... .......... .......... .......... ..........
ECOR_11       .......... .......... .......... .......... .......... .......... ..........
B171          .......... .......... .......... .......... .......... .......... ..........
E24377A       .......... .......... .......... .......... .......... .......... ..........
ECOR_34       .......... .......... .......... .......... .......... .......... ..........
ECOR_71       .......... .......... .......... .......... .......... .......... ..........
SE11          .......... .......... .......... .......... .......... .......... ..........
O157_Sakai    .......... .......... .......... .......... .......... .......... ..........
ECOR_35       .......... .......... .......... .......... .......... .......... ..........
ECOR_36       .......... .......... .......... .......... .......... .......... ..........
SMS-3-5       .......... .......... .......... .......... .......... .......... ..........
536           .......... .......... .......... .......... .......... .......... ..........
APEC_O1       .......... .......... .......... .......... .......... .......... ..........
CFT073        .......... .......... .......... .......... .......... .......... ..........
E2348/69      .......... .......... .......... .......... .......... .......... ..........
ECOR_51       .......... .......... .......... .......... .......... .......... ..........
ECOR_57       .......... .......... .......... .......... .......... .......... ..........
UTI89         .......... .......... .......... .......... .......... .......... ..........
E._albertii   .......... .......... ......T... ........T. .G..T..T.. ...G.....G ..........

        17151
K-12          TCACTATCAG CAGCATCACG CTGCCGTTTG TCGAAAATGA GAGCCGTGCA CGGGCAGTGA AGGATCAGAT
53638         .......... .......... .......... .......... .......... .......... ..........
ATCC_8739     .......... ......T... .......... .......... .......... .......... ..........
ECOR_10       .......... .......... .......... .......... .......... .......... ..........
ECOR_11       .......... .......... .......... .......... .......... .......... ..........
B171          .......... ......T... .......... .......... .......... .......... ..........
E24377A       .......... ......T... .......... .......... .......... .......... ..........
ECOR_34       .......... ......T... .......... .......... .......... .......... ..........
ECOR_71       .......... ......T... .......... .......... .......... .......... ..........
SE11          .......... ......T... .......... .......... .......... .......... ..........
O157_Sakai    .......... .......... .......... .......... .......... .......... ..........
ECOR_35       .......... ......T... .......... .......... .......... .......... ..........
ECOR_36       .......... ......T... .......... .......... .......... .......... ..........
SMS-3-5       .......... ......T... .......... .......... .......... .......... ..........
536           .......... ......T... .......... .......... .......... .......... ..........
APEC_O1       .......... ......T... .......... .......... .......... .......... .......A..
CFT073        .......... ......T... .......... .......... .......... .......... .A........
E2348/69      .......... ......T... .......... .......... .......... .......... ..........
ECOR_51       .......... ......T... .......... .......... .......... .......... .A........
ECOR_57       .......... ......T... .......... .......... .......... .......... .A........
UTI89         .......... ......T... .......... .......... .......... .......... ..........
E._albertii   .......... ......T... .......... .......... A........T ..A..G..A. .A........
```

```
        17221
K-12          TGACGCAATT TATCACCAGA CAGGCGTGCG CCCGCTGGTC TTCTACTCCA TCGTGTTGCC GGAGATTCGC
53638         .......... .......... .......... .......... .......... .......... ..........
ATCC_8739     .......... .......... .......... .......... .......... .......... ..........
ECOR_10       .......... .......... .......... .......... .......... .......... ..........
ECOR_11       .......... .......... .......... .......... .......... .......... ..........
B171          .......... .......... ....T..... .......... .......... .......... ..........
E24377A       ......C... .......... ....T..... .......... ........T. .......... ..........
ECOR_34       .......... .......... ....T..... .......... .......... .......... ..........
ECOR_71       .......... .......... ....T..... .......... .......... .......... ..........
SE11          .......... .......... ....T..... .......... .......... .......... ..........
O157_Sakai    .......... .......... .......... T......... .......... .......... ...A......
ECOR_35       .......... .......... ....T..... .......... .......... .......... ..........
ECOR_36       .......... .......... ....T..... .......... .......... .......... ..........
SMS-3-5       .......... .......... .......... .......... .......... .......... ..........
536           .......... .......... ....T..... .......... .......... .......... ..........
APEC_O1       .......... .......... ....T..... .......... .......... .......... ..........
CFT073        .......... .......... ....T..... .......... .......... .......... ..........
E2348/69      .......... .......... ....T..... .......... .......... .......... ..........
ECOR_51       .......... .......... ....T..... .......... .......... .......... ..........
ECOR_57       .......... .......... ....T..... .......... .......... .......... ..........
UTI89         .......... .......... ....T..... .......... .......... .......... ..........
E._albertii   C.....C... ..C..G.... .G.....A.. T......... ........T. .T..TC.... ...A......

        17291
K-12          GCCATCATCT TGCAAAGTGA AGGCTTTTGC CAGGATATCG TTCAGGCGCT GGTTGCCCCG CTACAACAAG
53638         .......... .......... .......... .......... .......... .......... ..........
ATCC_8739     .......... .......... .......... .......... .......... .......... ..........
ECOR_10       .......... .......... .......... .......... .......... .......... ..........
ECOR_11       .......... .......... .......... .......... .......... .......... ..........
B171          .......... .......... .......... ........T. .......... .......... ..........
E24377A       .......... .......... ...T...... .......... .......... .......... ..........
ECOR_34       .......... .......... .......... .......... .......... .......... ..........
ECOR_71       .......... .......... ...T...... .......... .......... .......... ..........
SE11          .......... .......... .......... ........T. .......... .......... ..........
O157_Sakai    .......... .......... ...T...... .......... .......... .......... ..........
ECOR_35       .......... .......... G......... .......... .......... .......... ..........
ECOR_36       .......... .......... G......... .......... .......... .......... ..........
SMS-3-5       .......... .......... G......... .......... .......... .......... ..........
536           ..T....... ...G...... .......... .......... .......... T......... ..........
APEC_O1       ..T....... .......... .......... .......... .......... .......... ..........
CFT073        ..T....... .......... .......... .......... .......... .......... ..........
E2348/69      ..T....... .......... .......... .......... .......... .......... ..........
ECOR_51       ..T....... .......... .......... .......... .......... .......... ..........
ECOR_57       ..T....... .......... .......... .......... .......... .......... ..........
UTI89         ..T....... .......... .......... .......... .......... .......... ..........
E._albertii   ........T. .......C.. .......... ........T. .......... ......A..A T.........

        17361
K-12          AGATGAAACT GGATCCAACG CCGATTGCTC ATCGTACCCA TGGCCTTAAC CCTAATAATC TCAATAAATA
53638         .......... .......... .......... .......... .......... .......... ..........
ATCC_8739     .......... .......... .......... .......... .......... .......... ..........
ECOR_10       .......... .......... .......... .......... .......... .......... ..........
ECOR_11       .......... .......... .......... .......... .......... .......... ..........
B171          .......... .......... .......... .......... .......... .......... ..........
E24377A       .......... .......... .......... .......... .......... .......... ..........
ECOR_34       .......... .......... .......... .......... .......... .......... ..........
ECOR_71       .......... .......... .......... .......... .......... .......... ..........
SE11          .......... .......... .......... .......... .......... .......... ..........
O157_Sakai    .......... .......... .......... .......... .......... .......... ..........
ECOR_35       .......... ......C... .......... .......... ......G... ........C. ..........
ECOR_36       .......... ......C... .......... .......... ......G... ........C. ..........
SMS-3-5       .......... ......C... .......... .......... ......G... ........C. ..........
536           .......... .......... .......... .......... .......... .......... ..........
APEC_O1       .......... .......... .......... .......... .......... .......... ..........
CFT073        .......... .......... .......... .......... .......... .......... ..........
E2348/69      .......... .......... .......... .......... .......... .......... ..........
ECOR_51       .......... .......... .......... .......... .......... .......... ..........
ECOR_57       .......... .......... .......... .......... .......... .......... ..........
UTI89         .......... .......... .......... .......... .......... .......... ..........
E._albertii   .......... ...C..G... ..TG.C..C. .C........ C..T..G..T ..C.....C. ..........
```

```
         17431
K-12           TGATGCGCGC ATTGCGGCGA TTGATTACAC CCTCGCCCAC GATGACGGCA TTTCGTTGCG CAATCTGGAC
53638          .......... .......... .......... .......... .......... .......... ..........
ATCC_8739      .......... .......... .......... .......... .......... .......... .........T
ECOR_10        .......... .......... .......... .......... .......... .......... ..........
ECOR_11        .......... .......... .......... .......... .......... .......... ..........
B171           .......... .......... .......... .......... .......... .......... ..........
E24377A        .......... .......... .......... ...T...... ........T. .......... ..........
ECOR_34        .......... .......... .......... ...T...... ........T. .......... ..........
ECOR_71        .......... .......... .......... .......... .......... .......... ..........
SE11           .......... .......... .......... .......... .......... .......... ..........
O157_Sakai     .......... .......... .......... .......... .......... .......... .........T
ECOR_35        .........T .......... .......... ...T.....T ..C....... .......... ..........
ECOR_36        .........T .......... .......... ...T.....T ..C....... .......... ..........
SMS-3-5        .........T .......... .......... ...T.....T ..C....... .......... ..........
536            .......... .......... .......... ...T...... ........T. .......... ..........
APEC_O1        .......... .......... .......... ...T...... ........T. .......... ..........
CFT073         .......... .......... .......... ...T...... ........T. .......... ..........
E2348/69       .......... .......... .......... ...T...... ........T. .......... ..........
ECOR_51        .......... .......... .......... ...T...... ........T. .......... ..........
ECOR_57        .......... .......... .......... ...T...... ........T. .......... ..........
UTI89          .......... .......... .......... ...T...... ........T. .......... ..........
E._albertii    .......... ..A.....A. .......T.. .........T .....T..A. .......... ...C..C..T

         17501
K-12           CAGGCTCAGG TGATCCTGCT CGGTGTTTCT CGCTGTGGTA AAACCCCCAC CAGTCTGTAT CTGGCAATGC
53638          .......... .......... .......... .......... .......... .......... ..........
ATCC_8739      .......... .......... .......... .......... .......... .......... ..........
ECOR_10        .......... .......... .......... .......... .......... .......... ..........
ECOR_11        .......... .......... .......... .......... .......... .......... ..........
B171           .......... .......... .......... .......... .......... .......... ..........
E24377A        .......... .......... .......... .......... .......... ......A... ..........
ECOR_34        .......... .......... ...C...... .......... .......... .......... ..........
ECOR_71        .......... .......... .......... .......... .......... .......... ..........
SE11           .......... .......... .......... .......... .......... .......... ..........
O157_Sakai     .......... .......... .......... .......... .......... .......... ..........
ECOR_35        .......... .......... .......... .......... .......... .......... ..........
ECOR_36        .......... .......... .......... .......... .......... .......... ..........
SMS-3-5        .......... .......... .......... .......... .......... .......... ..........
536            .......... .......... .......... .......... .......... .......... ..........
APEC_O1        .......... .......... .......... .......... .......... .......... ..........
CFT073         .......... .......... .......... .......... .......... .......... ..........
E2348/69       .......... .......... .......... .......... .......... .......... ..........
ECOR_51        .......... .......... .......... .......... .......... .......... ..........
ECOR_57        .......... .......... .......... .......... .......... .......... ..........
UTI89          .......... .......... .......... .......... .......... .......... ..........
E._albertii    .......... .......... .........C ........A. ....G..T.. ....T..... .....G....

         17571
K-12           AGTTTGGTAT CCGCGCGGCA AACTACCCCT TTATTGCCGA CGATATGGAT AATCTGGTGC TACCCGCGTC
53638          .......... .......... .......... .......... .......... .......... ..........
ATCC_8739      .A........ .......... .......... .......... .......... .......... ..........
ECOR_10        .......... .......... .......... .......... .......... .......... ..........
ECOR_11        .......... .......... .......... .......... .......... .......... ..........
B171           .......... .......... .......... .......... .........C .......... ..........
E24377A        .A........ .......... .......... .......... .........C .......... ..........
ECOR_34        .A........ .......... .......... .......... .......... .......... ..........
ECOR_71        .......... .......... .......... .......... .......... .......... ..........
SE11           .......... .......... .......... .......... .........C .......... ..........
O157_Sakai     .A........ .......... .......... .......... .......... .......... ..........
ECOR_35        .A........ .......... .......... .......... .......... .......... ..........
ECOR_36        .A........ .......... .......... .......... .......... .......... ..........
SMS-3-5        .A........ .......... .......... .......... .......... .......... ..........
536            .A........ .......... ..T....... .......... .......... .......... ..........
APEC_O1        .A........ .......... ..T....... .......... .......... .......... .G........
CFT073         .A........ .......... ..T....... .......... .......... .......... ..........
E2348/69       .A........ .......... ..T....... .......... .......... .......... ..........
ECOR_51        .A........ .......... ..T....... .......... .......... .......... ..........
ECOR_57        .A........ .......... ..T....... .......... .......... .......... ..........
UTI89          .A........ .......... ..T....... .......... .......... .......... .G........
E._albertii    .......C.. ...G...... .......... .......... .........C ........C. .G..T.....
```

```
         17641
K-12         GCTCAAACCG CTTCAGCATA AATTGTTCGG CCTGACTATC GACCCGGAAC GTCTGGCGGC GATTCGCGAG
53638        .......... .......... .......... .......... .......... .......... ..........
ATCC_8739    .......... .......... .......... .........A .......... .......... ..........
ECOR_10      .......... .......... .......... .......... .......... .......... ..........
ECOR_11      .......... .......... .......... .......... .......... .......... ..........
B171         .......... .......... .......... .......... .......... .......... ..........
E24377A      .......... .......... .......... .......... .......... .......... ......T..A
ECOR_34      .......... ........C. .......... .......... .......... .......... ..........
ECOR_71      .......... .......... .......... .......... .......... .A........ ..........
SE11         .......... .......... .......... .......... .......... .......... ..........
O157_Sakai   .......... .......... .......... .......... .......... .......... ..........
ECOR_35      .......... ........C. .......... .......... .......... .......... ..........
ECOR_36      .......... ........C. .......... .......... .......... .......... ..........
SMS-3-5      .......... ........C. .......... .......... .......... .......... ..........
536          .........A ........C. .......... .......... .......... .......... ..........
APEC_O1      .........A ........C. .......... .......... .......... .......... ..........
CFT073       .........A ........C. .......... .......... .......... .......... ..........
E2348/69     .......... ........C. .......... .......... .......... .......... ..........
ECOR_51      .........A ........C. .......... .......... .......... .......... ..........
ECOR_57      .........A ........C. .......... .......... .......... .......... ..........
UTI89        .........A ........C. .......... .......... .......... .......... ..........
E._albertii  A......... ..G..A.... ..C....... .......... .....A..G. .G........ C.....T...

         17711
K-12         GAACGTCGGG AGAACAGTCG CTATGCCTCG CTTCGTCAGT GCAGGATGGA AGTCGCGGAA GTGGAAGCCT
53638        .......... .......... .......... .......... .......... .......... ..........
ATCC_8739    .......... .......C.. .......... .......... .......... .......... ..A.....G.
ECOR_10      .......... .......... .......... .......... .......... .......... ..........
ECOR_11      .......... .......... .......... .......... .......... .......... ..........
B171         .......... .......C.. .......... .......... .......... .......... ..A.....G.
E24377A      ..G....... .......... .......... .......... .......... .......... ........G.
ECOR_34      .......... .......... .......... .......... .......... .......... ........G.
ECOR_71      .......... .......... .......... .......... .......... .......... .......TG.
SE11         .......... .......C.. .......... .......... .......... .......... ..A.....G.
O157_Sakai   .......... .......... .......... .......... .......... .......... ..........
ECOR_35      .......... .......... .......... .......... .......... .......... ........G.
ECOR_36      .......... .......... .......... .......... .......... .......... ........G.
SMS-3-5      .......... .......... .......... .......... .......... .......... ........G.
536          .......... .......... .......... .......... .......... .......... ........G.
APEC_O1      .......... .......C.. .......... .......... .......... .......... ........G.
CFT073       .......... .......... .......... .......... .......... .......... ........G.
E2348/69     .......... .......... .......... .......... .......... .......... ........G.
ECOR_51      .......... .......... .......... .......... .......... .......... ........G.
ECOR_57      .......... .......... .......... .......... .......... .......... ........G.
UTI89        .......... .......C.. .......... .......... .......... .......... ........G.
E._albertii  .....CA... .......C.. .......... ..G..C.... ....A..... ...A..T... ..A.....GC

         17781
K-12         TGTACCGTAA AAATCAGATC CCGTGGATTA ACAGTACCAA TTATTCGGTA GAAGAGATTG CCACCAAGAT
53638        .......... .......... .......... .......... .......... .......... ..........
ATCC_8739    .......... .......... .......... .......... .......... .......... ..........
ECOR_10      .......... .......... .......... .......... .......... .......... ..........
ECOR_11      .......... .......... .......... .......... .......... .......... ..........
B171         .......... .......... .......... .......... .......... .......... ..........
E24377A      .......... .......... .......... .......... .......... .......... ..........
ECOR_34      .......... .......... .......... .......... .......... .......... ..........
ECOR_71      .......... .......... .......... .......... .......... .......... ..........
SE11         .......... .......... .......... .......... .......... .......... ..........
O157_Sakai   .......... .......... .......... .......... .......... .......... ..........
ECOR_35      .......... .......... .......... .......... .......... .......... ..........
ECOR_36      .......... .......... .......... .......... .......... .......... ..........
SMS-3-5      .......... .......... .......... .......... .......... .......... ..........
536          .......... .......... .......... .......... .......... .......... ..........
APEC_O1      .......... .......... .......... .......... .......... .......... ..........
CFT073       .......... .......... .......... .......... .......... .......... ..........
E2348/69     .......... .......... .......... .......... .......... .......... ..........
ECOR_51      .......... .......... .......... .......... .......... .......... ..........
ECOR_57      .......... .......... .......... .......... .......... .......... ..........
UTI89        .......... .......... .......... .......... .......... .......... ..........
E._albertii  .......C.. ......A... ........C. .......... .......... .......... .T........
```

```
        17851
K-12         CCTCGATATC ATGGGCCTTA GTCGCCGAAT GTACTAGAGA ACTAGTGCAT TAGCTTATTT TTTTGTTATC
53638        .......... .......... .......... .......... .......... .......... ..........
ATCC_8739    .......... .....G.... .C........ .......... .......... .......... ..........
ECOR_10      .......... .......... .......... .......... .......... .......... ..........
ECOR_11      .......... .......... .......... .......... .......... .......... ..........
B171         .......... .....G.... .C........ .......... G......... .......... ..........
E24377A      .......... .......... .C........ .......... .......... .......... ..........
ECOR_34      .......... .......... .......... .......... ......T... .......... ..........
ECOR_71      .......... .......... .C..T..... .......... .......... ..A...G... ..........
SE11         .......... .....G.... .C........ .......... .......... .......... ...-......
O157_Sakai   .......... .......... .......... .......... .......... .......... ..........
ECOR_35      .......... .......... .C..T..... ........A. .......... .G....GC.. ..........
ECOR_36      .......... .......... .C..T..... ........A. .......... .G....GC.. ..........
SMS-3-5      .......... .......... .C..T..... .......... .......... ..A....... ..........
536          .......... .......... .C........ .......... ........G. ..A...G... ..........
APEC_O1      .......... .......... .C........ .......... .......... .G.....C.. ..........
CFT073       .......... .......... .C........ .......... .......... ..A...G... ..........
E2348/69     .......... .......... .C........ .......... .......... .G....G... ..........
ECOR_51      .......... .......... .C........ .......... .......... ..A...G... ..........
ECOR_57      .......... .......... .C........ .......... .......... ..A...G... ..........
UTI89        .......... .......... .C........ .......... .......... .G.....C.. ..........
E._albertii  .........T .....G..G. A...AA.... ........T. ......AT.A GG.T.CG.C. .........T

        17921
K-12         ATGCTAACCA CCCGGCGAGG TGTGACACAC CTCGCACTTG AAATCAGCAG CGATTGGTTT ATCGTGATGC
53638        .......... .......... .......... .......... .......... .......... ..........
ATCC_8739    .......... .......... .......... .......... ........T. .......... ..........
ECOR_10      .......... .......... .......... .......... .......... .......... ..........
ECOR_11      .......... .......... .......... .......... .......... .......... ..........
B171         .......... .......... .......... .......... ........T. .......... ..........
E24377A      .......... .......... .......... .......... ........T. .......... ..........
ECOR_34      .......... .......... .......... .......... .......... .......... ..........
ECOR_71      .......... .......... .......... .......... ........T. .......... ..........
SE11         .......... .......... .......... .......... ........T. .......... ..........
O157_Sakai   .......... .......... .......... .......... .......... .......... ..........
ECOR_35      .......... .......... .......... .......... ........T. .......... ..........
ECOR_36      .......... .......... .......... .......... ........T. .......... ..........
SMS-3-5      .......... .......... .......... .......... ........T. .......... ..........
536          .......... .......... .......... .......... ........T. .......... ..........
APEC_O1      .......... .......... .......... .......... ........T. .......... ..........
CFT073       .......... .......... .......... .......... ........T. .......... ..........
E2348/69     .......... .......... .......... .......... ........T. .......... ..........
ECOR_51      .......... .......... .......... .......... ........T. .......... ..........
ECOR_57      .......... .......... .......... .......... ........T. .......... ..........
UTI89        .......... .......... .......... .......... ........T. .......... ..........
E._albertii  ....CGC.A. TTGC..A... C..TG.CTG. .C...T.... .......... G......... ..........

        17991
K-12         GCATCACTTC CCGGCAGTCC TGCCGTAGAA GCAACAAATT TCTGAGACTT GTAATGAACA GAACTGACGA
53638        .......... .......... .......... .......... .......... .......... ..........
ATCC_8739    .......... ....T..... .......... .......... .......... .......... ..........
ECOR_10      .......... .......... .......... .......... .......... .......... ..........
ECOR_11      .......... .......... .......... .......... .......... .......... ..........
B171         .......... ....T..... .......... .......... .......... .......... ..........
E24377A      .......... ....T..... .......... ......T... .......... .......... ..........
ECOR_34      .......... .......... .......... .......... .......... .......... ..........
ECOR_71      .......... ....T..... .......... .......... .......... .......... ..........
SE11         .......... ....T..... .......... .......... .......... .......... ..........
O157_Sakai   .......... .......... .......... .......... .......... .......... ..........
ECOR_35      .......... ....T..... .......... .......... .......... .......... ..........
ECOR_36      .......... ....T..... .......... .......... .......... .......... ..........
SMS-3-5      .......... ....T..... .......... .......... .......... .......... ..........
536          .......... ....T..... .......... .......... .......... .......... ..........
APEC_O1      .......... ....T..... .......... .......... .......... .......... ..........
CFT073       .......... ....T..... .......... .......... .......... .......... ..........
E2348/69     .......... ....T..... .......... .......... .......... .......... ..........
ECOR_51      .......... ....T..... .......... .......... .......... .......... ..........
ECOR_57      .......... ....T..... .......... .......... .......... .......... ..........
UTI89        .......... ....T..... .......... .......... .......... .......... ..........
E._albertii  .......... .T....AC.. ....A.T... .......... .......G.. .......... ..........
```

```
         18061
K-12         ACTCCGTACT GCGCGTATTG AGAGCCTGGT AACGCCCGCC GAACTCGCGC TACGGTATCC CGTAACGCCT
53638        .......... .......... .......... .......... .......... .......... ..........
ATCC_8739    .......... .......... .......... .......... .......... .......... ..........
ECOR_10      .......... .......... .....T.... .......... .......... .......... ..........
ECOR_11      .......... .......... .......... .......... .......... .......... ..........
B171         .......... .......... .......... .......... .......... .......... ..........
E24377A      .......... .......... .....T.... .......... .......... .......... ..........
ECOR_34      .......... .......... .......... .......... .......... .......... ..........
ECOR_71      .......... .......... .......... .......... .......... .......... ..........
SE11         .......... .......... .......... .......... .......... .......... ..........
O157_Sakai   .......... .......... .......... .......... .......... .......... ..........
ECOR_35      .......... .......... .......... .......... .......... .......... .......A..
ECOR_36      .......... .......... .......... .......... .......... .......... .......A..
SMS-3-5      .......... .......... .......... .......... .......... .......... ..........
536          .......... .......... .......... .......... .....T.... .......... ..........
APEC_O1      .......... .......... .......... .......... .......... .......... ..........
CFT073       .......... .......... .......... .......... .......... .......... ..........
E2348/69     .......... .......... .......... .......... .......... .......... ..........
ECOR_51      .......... .......... .......... .......... .......... .......... ..........
ECOR_57      .......... .......... .......... .......... .......... .......... ..........
UTI89        .......... .......... .......... .......... .......... .......... ..........
E._albertii  .......... ........C. ....T..... .......... .......... .......... ..........

         18131
K-12         GGCGTCGCCA CCCATGTCAC CGACTCCCGC CGCAGAATTG AAAAAATACT GAATGGTGAA GATAAGCGAC
53638        .......... .......... .......... .......... .......... .......... ..........
ATCC_8739    .......... .......... .......... .......... .......... .......... ..........
ECOR_10      .......... .......... .......... .......... .......... .......... ..........
ECOR_11      .......... .......... .......... .......... .......... .......... ..........
B171         .......... .......... .......... .......... .......... .......... ..........
E24377A      .......... .......... .......... .......... .......... .......... ..........
ECOR_34      .........G .......... .......... .......... .......... .......... ..........
ECOR_71      .......... .......... .......... .......... .......... .......... ..........
SE11         .......... .......... .......... .......... .......... .......... ..........
O157_Sakai   .......... .......... .......... .......... .......... C......... ..........
ECOR_35      .......... .......... .......... .......... .......... C......... ..........
ECOR_36      .......... .......... .......... .......... .......... C......... ..........
SMS-3-5      .......... .......... .......... .......... .......... .......... ..........
536          .......... .......... .......... ..T....... .......... .......... ..........
APEC_O1      .......... .......... .......... ..T....... .......... .......... ..........
CFT073       .......... .......... .......... ..T....... .......... .......... ..........
E2348/69     .........C .......... .......... ..T....... .......... .......... ..........
ECOR_51      .......... .......... .......... ..T....... .......... .......... ..........
ECOR_57      .......... .......... .......... ..T....... .......... .......... ..........
UTI89        .......... .......... .......... ..T....... .......... .......... ..........
E._albertii  .....T.... GG.......T ...T.....A .......... .......... .......... ..........

         18201
K-12         TGTTGGTCAT TATTGGCCCC TGCTCGATCC ACGATCTCAC CGCTGCAATG GAGTACGCCA CCCGTCTGCA
53638        .......... .......... .......... .......... .......... .......... ..........
ATCC_8739    .......... .......... .......... .......... .......... .......... ..........
ECOR_10      .......... .......... .......... .......... .......... .......... ..........
ECOR_11      .......... .......... .......... .......... .......... .......... ..........
B171         .......... .......... .......... .......... .......... .......... ..........
E24377A      .......... .......... .......... .......... .......... .......... ..........
ECOR_34      .......T.. .......... .......... .......... .......... .......... ..........
ECOR_71      .......... .......... .......... .......... .......... ..A....... ..........
SE11         .......... .......... .......... .......... .......... .......... ..........
O157_Sakai   .......... .......... .......... .......... .......... .......... ..........
ECOR_35      .......... .......... .......... .......... .......... .......... ..........
ECOR_36      .......... .......... .......... .......... .......... .......... ..........
SMS-3-5      .......... .......... .......... .......... .......... .......... ..........
536          .......... .......... .......... .......... ......G... .......... ..........
APEC_O1      .......... .......... .......... .......... ......G... .......... ..........
CFT073       .......... .......... .......... .......... ......G... .......... ..........
E2348/69     .......... .......... .......... .......... ......G... .......... ..........
ECOR_51      .......... .......... .......... .......... ......G... .......... ..........
ECOR_57      .......... .......... .......... .......... ......G... .......... ..........
UTI89        .......... .......... .......... .......... ......G... .......... ..........
E._albertii  .......... .......... .......... .......A.. ...C...... .......... G.........
```

```
        18271
K-12         GTCGCTGCGC AACCAGTACC AGTCACGGCT GGAAATCGTA ATGCGCACCT ATTTTGAAAA ACCACGAACT
53638        .......... .......... .......... .......... .......... .......... ..........
ATCC_8739    .......... .......... .......... .......... .......... .......... ..........
ECOR_10      .......... .......... .......... .......... .......... .......... ..........
ECOR_11      .......... .......... .......... .......... .......... .......... ..........
B171         .......... .......... .......... .......... .......... .......... ..........
E24377A      .......... .......... .......... .......... .......... .......... ..........
ECOR_34      .......... .......... .......... .......... .......... .......... ..........
ECOR_71      .......... .......... .......... T......... .......... ...A...... ...C......
SE11         .......... .......... .......... .......... .......... .......... ..........
O157_Sakai   .......... .......... .......... .......... .......... .......... ..........
ECOR_35      .......... .......... .......... .......... .......... .......... ..........
ECOR_36      .......... .......... .......... .......... .......... .......... ..........
SMS-3-5      .......... .......... .......... .......... .......... .......... ..........
536          .......... .......... .......... .......... .......... .......... .........G
APEC_O1      .......... .......... .......... .......... .......... .......... .........G
CFT073       .......... .......... .......... .......... .......... .......... .........G
E2348/69     ......A... .......... ..C....... .......... .......... .......... .........G
ECOR_51      .......... .......... .......... .......... .......... .......... .........G
ECOR_57      .......... .......... .......... .......... .......... .......... .........G
UTI89        .......... .......... .......... .......... .......... .......... .........G
E._albertii  .A.......A ........T. .......A.. .........C .....T.... .......... ......T..C

        18341
K-12         GTTGTCGGCT GGAAAGGACT AATCTCCGAT CCAGATTTAA ACGGCAGCTA TCGGGTAAAT CACGGTCTGG
53638        .......... .......... .......... .......... .......... .......... ..........
ATCC_8739    .......... .......G.. .......... ..G....... .......... .......... ..........
ECOR_10      .......... .......... .......... .......... .......... .......... ..........
ECOR_11      .......... .......... .......... .......... .......... .......... ..........
B171         .......... .......G.. .......... ..G....... .......... .......... ..........
E24377A      .......... .......G.. ...T...... ..G....... .......... .......... ..........
ECOR_34      .......... .......G.. .......... ..G....... .......... .......... ..........
ECOR_71      .......... .......G.. .......... .......... .......... .......... ..........
SE11         .......... .......G.. .......... ..G....... .......... .......... ..........
O157_Sakai   .......... .......... .......... .......... .......... ....A..... ..........
ECOR_35      .......... .......... .......... .......... ....T..... .......... ..........
ECOR_36      .......... .......... .......... .......... ....T..... .......... ..........
SMS-3-5      .......... .......... C......... .......... ....T..... .......... ..........
536          .......... .......... C......... .......... ....T..... .......... ..........
APEC_O1      .......... .......... C......... .......... ....T..... .......... ..........
CFT073       .......... .......... C......... .......... ....T..... .......... ..........
E2348/69     .......... .......... C......... .......... ....T..... .......... ..........
ECOR_51      .......... .......... C......... .......... ....T..... .......... ..........
ECOR_57      .......... .......... C......... .......... ....T..... .......... ..........
UTI89        .......... .......... C......... .......... ....T..... .......... ..........
E._albertii  .......... .......TT. ...T.....C ..G....... .......... ...C...... ..........

        18411
K-12         AGCTGGCGCG CAAATTACTT TTACAGGTAA ATGAGCTGGG CGTCCCAACC GCGACCGAGT TCCTCGATAT
53638        .......... .......... .......... .......... .......... .......... ..........
ATCC_8739    .......... .......... .......... .......... T.....T... .....T.... .T........
ECOR_10      .......... .......... .......... .......... .......... .......... ..........
ECOR_11      .......... .......... .......... .......... .......... .......... ..........
B171         .......... .......... .......... .......... T.....T... .....T.... ..........
E24377A      .......... .......... .......... .......... ......T... .....T.... ..........
ECOR_34      .......... .......... .......... .......... T.....T... .....T.... ..........
ECOR_71      .......... .......... .......... .......... T.....T... .....T.... ..........
SE11         .......... .......... .......... .......... T.....T... .....T.... ..........
O157_Sakai   .......... .......... .......... .......... .......... .....T.... ..........
ECOR_35      .......... .......... .......... .......... ......T... .....T.... ..........
ECOR_36      .......... .......... .......... .......... ......T... .....T.... ..........
SMS-3-5      .......... .......... .......... .......... ......T... .....T.... ..........
536          .......... .......... .......... .......... ......T... .....T.... ..........
APEC_O1      .......... .......... .......... .......... ......T... .....T.... ..........
CFT073       .......... .......... .......... .......... ......T... .....T.... ..........
E2348/69     .......... .......... .......... .......... ......T... .....T.... ..........
ECOR_51      .......... .......... .......... .......... ......T... .....T.... ..........
ECOR_57      .......... .......... .......... .......... ......T... .....T.... ..........
UTI89        .......... .......... .......... .......... ......T... .....T.... ..........
E._albertii  .A........ T..G.....G ..G.....G. .......... ...G..C... .....T.... .T........
```

```
             18481
K-12         GGTGACCGGT CAGTTTATTG CTGATTTAAT CAGTTGGGGC GCGATTGGCG CACGTACTAC CGAAAGTCAG
53638        .......... .......... .......... .......... .......... .......... ..........
ATCC_8739    .......... .......... .......... .......... .......... .......... ..........
ECOR_10      .......... .......... .......... .......... .......... .......... ..........
ECOR_11      .......... .......... .......... .......... .......... .......... ..........
B171         .......... .......... .......... .......... .......... .......... ..........
E24377A      .......... .......... .......... .......... .......... .......... ..........
ECOR_34      .......... .......... .......... .......... .......... .......... ..........
ECOR_71      .......... .......... .......... .........A .......... .......... ..........
SE11         .........C .......... .......... .......... .......... .......... ..........
O157_Sakai   .......... .......... .......... .......... .......... .......... ..........
ECOR_35      .......... .......... .......... .......... .......... .......... ..........
ECOR_36      .......... .......... .......... .......... .......... .......... ..........
SMS-3-5      .......... .......... .......... .......... .......... .T........ ..........
536          .......... .......... .......... .........T .......... .......... ..........
APEC_O1      .......... .......... .......... .......... .......... .......... ..........
CFT073       .......... .......... .......... .........T .......... .......... ..........
E2348/69     .......... .......... .......... .......... .......... .......... ..........
ECOR_51      .......... .......... .......... .........T .......... .......... ..........
ECOR_57      .......... .......... .......... .........T .......... .......... ..........
UTI89        .......... .......... .......... .......... .......... .......... ..........
E._albertii  .......... .......... .C...C.... T........A .......... .G.....A.. ......C...

             18551
K-12         ATCCACCGCG AAATGGCTTC GGCACTCTCC TGTCCGGTAG GTTTTAAAAA TGGTACCGAT GGCAATACGC
53638        .......... .......... .......... .......... .......... .......... ..........
ATCC_8739    .......... .......... A......... .......... .......... .......... .....C....
ECOR_10      .......... .......... .......... .......... .......... .......... ..........
ECOR_11      .......... .......... .......... .......... .......... .......... ..........
B171         ........T. .......... .......... .......... .......... .......... .....C....
E24377A      ........T. .......... .......... .......... .......... .......... .....C....
ECOR_34      ........T. .......... .......... .......... .......... .......... .....C....
ECOR_71      ........T. .......... .......... .......... .......... .......... .....C....
SE11         ........T. .......... .......... .......... .......... .......... .....C....
O157_Sakai   ........T. .......... .......... .......... .......... .......... ..........
ECOR_35      ........T. .......... .......... .......... .......... .......... .....C....
ECOR_36      ........T. .......... .......... .......... .......... .......... .....C....
SMS-3-5      .......... .......... A......... .......... .......... .......... .....C....
536          .......... .......... .......... ........G. .......... .......... .....C....
APEC_O1      .......... .......... .......... ........G. .......... .......... .....C....
CFT073       .......... .......... .......... ........G. .......... .......... .....C....
E2348/69     .......... .......... .......... ........G. .......... .......... .....C....
ECOR_51      .......... .......... .......... ........G. .......... .......... .....C....
ECOR_57      .......... .......... .......... ........G. .......... .......... .....C....
UTI89        .......... .......... .......... ........G. .......... .......... .....C....
E._albertii  ........T. .G........ ...G...... ..C....... .......... .........C .....C..A.

             18621
K-12         GGATTGCTGT GGATGCTATC CGCGCAGCCC GCGCCAGCCA TATGTTCCTC TCGCCAGACA AAAATGGTCA
53638        .......... .......... .......... .......... .......... .......... ..........
ATCC_8739    .......C.. .......... .......... .......... .......... .......... ..........
ECOR_10      .......... .......... .......... .......... .......... .......... ..........
ECOR_11      .......... .......... .......... .......... .......... .......... ..........
B171         .......C.. .......... .......... .......... ......T... .......... ..........
E24377A      .......C.. .......... .......... .......... ......T... .......... ..........
ECOR_34      .......C.. .......... .......... .......... .......... .......... ..........
ECOR_71      .......C.. .......... .......... .......... ......T... .......... ..........
SE11         .......C.. .......... .......... .......... ......T... .......... ..........
O157_Sakai   .......... .......... .......... .......... .......... .......... ..........
ECOR_35      .......... .......... .......... .......... .......... .......... ..........
ECOR_36      .......... .......... .......... .......... .......... .......... ..........
SMS-3-5      .......C.. .......... .......... .......... .......... ..A....... ....C.....
536          .......C.. .......... .......... .......... .......... .......... ..........
APEC_O1      .......C.. .......... .......... .......... .......... .......... ..........
CFT073       .......C.. .......... .......... .......... .......... .......... ..........
E2348/69     .......C.. .......... .......... .......... .......... .......... ..........
ECOR_51      .......C.. .......... .......... .......... .......... .......... ..........
ECOR_57      .......C.. .......... .......... .......... .......... .......... ..........
UTI89        .......C.. .......... .......... .......... .......... .......... ..........
E._albertii  .......C.. .......... .....T.... .......... .........T .....G..T. ....C..C..
```

```
        18691
K-12         GATGACCATC TATCAGACCA GCGGCAACCC GTATGGCCAC ATTATTATGC GTGGCGGCAA AAAACCGAAT
53638        .......... .......... .......... .......... .......... .......... ..........
ATCC_8739    .......... .......... .......... .......... .......... .......... ..........
ECOR_10      .......... .......... .......... .......... .......... .......... ..........
ECOR_11      .......... .......... .......... .......... .......... .......... ..........
B171         .......... .......... .......... .......... .......... .......... ..........
E24377A      .......... .......... .......... .......... .......... .......... ..........
ECOR_34      .......... .......... .......... .......... .......... .......... ..........
ECOR_71      .......... .......... .......... .......... .......... .......... ..........
SE11         .......... .......... .......... .......... .......... .......... ..........
O157_Sakai   .......... .......... .......... .......... .......... .......... ..........
ECOR_35      .......... .......... .......... .......... .......... .......... ..........
ECOR_36      .......... .......... .......... .......... .......... .......... ..........
SMS-3-5      .......... .......... .T........ .........T .......... .......... ..........
536          .......... .......... .......... .......... .......... ....T..... ..........
APEC_O1      .......... .......... .......... .......... .......... ....T..... ..........
CFT073       .......... .......... .......... .......... .......... ....T..... ..........
E2348/69     .......... .......... .......... .......... .......... ....T..... ..........
ECOR_51      .......... .......... .......... .......... .......... ....T..... ..........
ECOR_57      .......... .......... .......... .......... .......... ....T..... ..........
UTI89        .......... .......... .......... .......... .......... ....T..... ..........
E._albertii  .......... ..C....... .......... ......T... .......... .......... ......A..C

        18761
K-12         TATCATGCCG ATGATATCGC CGCAGCCTGC GATACGCTGC ACGAGTTTGA TTTACCTGAA CATCTGGTGG
53638        .......... .......... .......... .......... .......... .......... ..........
ATCC_8739    .......... .......... .......... .......... .......... .......... ..........
ECOR_10      .......... .......... .......... .......... .......... .......... ..........
ECOR_11      .......... .......... .......... .......... .......... .......... ..........
B171         .......... .......... .......... .......... .......... .......... ..........
E24377A      .......... .......... .......... .......... .......... .......... ........A.
ECOR_34      .......... .......... .......... .......... .......... .......... ..........
ECOR_71      .......... .......... .......... .......... .......... .......... ..........
SE11         .......... .......... .......... .......... .......... .......... ..........
O157_Sakai   .......... .......... .......... .......... .......... .......... ..........
ECOR_35      .......... .......... .......... .......... .......... .......... ..........
ECOR_36      .......... .......... .......... .......... .......... .......... ..........
SMS-3-5      ..C....... .......... .......... .......... .......... .......... ..........
536          .......... .......... .......... .......... .......... .......... ..........
APEC_O1      .......... .......... .......... .......... .......... .......... ..........
CFT073       .......... .......... .......... .......... .......... .......... ..........
E2348/69     .......... .......... .......... .......... .......... .......... ..........
ECOR_51      .......... .......... .......... .......... .......... .......... ..........
ECOR_57      .......... .......... .......... .......... .......... .......... ..........
UTI89        .......... .......... .......... .......... .......... .......... ..........
E._albertii  ..C..C.... .......... ...T.....T ......T... .T..A..... .......... ........A.

        18831
K-12         TGGATTTCAG CCACGGTAAC TGCCAGAAGC AGCACCGTCG CCAGTTAGAA GTTTGTGAGG ATATTTGTCA
53638        .......... .......... .......... .......... .......... .......... ..........
ATCC_8739    .......... .......... .......... .......... .......... .......... ..........
ECOR_10      .......... .......... .......... .......... .......... .......... ..........
ECOR_11      .......... .......... .......... .......... .......... .......... ..........
B171         .......... .......... .......... .......... .......... .......... ..........
E24377A      .......... .......... .......... .......... .......... .......... ..........
ECOR_34      .......... .......... .......... .......... .......... .......... ..........
ECOR_71      .......... .......... .......... .......... .......... .......... ..........
SE11         .......... .......... .......... .......... .......... .......... ..........
O157_Sakai   .......... .......... .......... .......... .......... .......... ..........
ECOR_35      .......... .......... .......... .......... .......... .......... ..........
ECOR_36      .......... .......... .......... .......... .......... .......... ..........
SMS-3-5      .......... .......... .......... .......... .......... .......... ..........
536          .......... .......... .......... .......... .......... .......... ..........
APEC_O1      .......... .......... .......... .......... .......... .......... ..........
CFT073       .......... .......... .......... .......... .......... .......... ..........
E2348/69     .......... .......... .......... .......... .......... .......... ..........
ECOR_51      .......... .......... .......... .......... .......... .......... ..........
ECOR_57      .......... .......... .......... .......... .......... .......... ..........
UTI89        .......... .......... .......... .......... .......... .......... ..........
E._albertii  .......... ......C... .......... ....T..... T......... ..C.....A. ..G....C..
```

```
       18901
K-12        GCAAATCCGC AATGGCTCTA CGGCGATTGC TGGAATTATG GCGGAAAGTT TCCTGCGCGA AGGAACGCAA
53638       .......... .......... .......... .......... .......... .......... ..........
ATCC_8739   .......... .......... .......... .......... .......... .......... ..........
ECOR_10     .......... .......... .......... .......... .......... .......... ..........
ECOR_11     .......... .......... .......... .......... .......... .......... ..........
B171        .......... .......... .......... .......... .......... .......... ..........
E24377A     .......... .......... .......... .......... .......... .......... ..........
ECOR_34     .......... .......... .......... .......... .......... .......... ..........
ECOR_71     .......... .......... .......... .......... .......... .......... ..........
SE11        .......... .......... .......... .......... .......... .......... ..........
O157_Sakai  .......... .......... .......... .......... .......... .......... ..........
ECOR_35     .......... .......... .......... .......... .......... .......... ..........
ECOR_36     .......... .......... .......... .......... .......... .......... ..........
SMS-3-5     .......... .......... .......... .......... .......... .......... ..........
536         .......... .......... .......... .......... .......... .......... ..........
APEC_O1     .......... .......... .......... .......... .......... .......... ..........
CFT073      .......... .......... .......... .......... .......... .......... ..........
E2348/69    .......... .....T.... .......... .......... .......... .......... ..........
ECOR_51     .......... .......... .......... .......... .......... .......... ..........
ECOR_57     .......... .......... .......... .......... .......... .......... ..........
UTI89       .......... .......... .......... .......... .......... .......... ..........
E._albertii A......... ........C. .......... C......... .......... .......... ...C......

       18971
K-12        AAAATCGTCG GCAGTCAGCC GCTCACTTAC GGTCAATCCA TTACCGACCC GTGTCTGGGC TGGGAGGATA
53638       .......... .......... .......... .......... .......... .......... ..........
ATCC_8739   .......... ..G....... .......... .......... .......... .......... .....A....
ECOR_10     .......... .......... .......... .......... .......... .......... ..........
ECOR_11     .......... .......... .......... .......... .......... .......... ..........
B171        .......... ..G....... .......... .......... .......... .......... .....A....
E24377A     .......... ..G....... .......... .......... .......... .......... .....A....
ECOR_34     .......... .......... .......... .......... .......... .......... .....A....
ECOR_71     .......... ..G....... .......... .......... .......... .......... .....A....
SE11        .......... ..G....... .......... .......... .......... .......... .....A....
O157_Sakai  .......... ..G....... .......... .......... .......... .......... .....A....
ECOR_35     .......... ..G....... .......... .......... .......... .......... .....A....
ECOR_36     .......... ..G....... .......... .......... .......... .......... .....A....
SMS-3-5     .......... ..G....... .......... ........G. .......... .......... .....A....
536         .......... ..G....... .......... .......... .......... .......... .....A....
APEC_O1     .......... ..G....... .......... .......... .......... .......... .....A....
CFT073      .......... ..G....... .......... .......... .......... .......... .....A....
E2348/69    .......... ..G....... .......... .......... .......... .......... .....A....
ECOR_51     .......... ..G....... .......... .......... .......... .......... .....A....
ECOR_57     .......... ..G....... .......... .......... .......... .......... .....A....
UTI89       .......... ..G....... .......... .......... .......... .......... .....A....
E._albertii .........A ..G.AACC.. ......C..T .....G..G. .C.....T.. ...CT.A..T .....A....

       19041
K-12        CCGAACGCCT GGTCGAAAAA CTCGCCTCTG CGGTAGATAC CCGCTTCTGA ATGCGTGCCC ATTCCTGACG
53638       .......... .......... .......... .......... .......... .......... ..........
ATCC_8739   .......... .......... .......... .......... .......... .......... ........T.
ECOR_10     .......... .......... .......... .......... .......... .......... ..........
ECOR_11     .......... .......... .......... .......... .......... .......... ..........
B171        .......... .......... ..T....... .......... .......... .......... ........T.
E24377A     .......... .......... .......... .......... .......... .......... ........T.
ECOR_34     .......... .......... .......... .......... .......... .......... ........T.
ECOR_71     .......... .......... .......... .......... .......... .......... ........T.
SE11        .......... .......... ..T....... .......... .......... .......... ........T.
O157_Sakai  .......... .......... ..A....... .......... .......... .......... ........T.
ECOR_35     .......... .......... .......... .......... .......... .......... ........T.
ECOR_36     .......... .......... .......... .......... .......... .......... ........T.
SMS-3-5     .......... .......... .......... ....T..... .......... .......... ..........
536         .......... .......... .......... .......... T......... .......... ..........
APEC_O1     .......... .......... .......... .......... T......... .......... ..........
CFT073      .......... .......... .......... .......... T......... .......... ..........
E2348/69    .......... .......... .......... .......... T......... .......... ..........
ECOR_51     .......... .......... .......... .......... T......... .......... ..........
ECOR_57     .......... .......... .......... .......... T......... .......... ..........
UTI89       .......... .......... .......... .......... T......... .......... ..........
E._albertii .T...A.... .......... ......G... ....G..... T..T....AG G..AT..... .....CACA.
```

```
        19111
K-12         GAATGGGCAT TTCTGCGCAA CTTGTTGTCT TCTCAACAAA TTACTGCTTG CTCTGGTCAG CCATAATATT
53638        .......... .......... .......... .......... .......... .......... ..........
ATCC_8739    .G........ .......... .......... ........C. .......... ......C.T. ..........
ECOR_10      .......... .......... .......... .......... .......... .......... ..........
ECOR_11      .......... .......... .......... .......... .......... .......... ..........
B171         .G........ .......... .......... .....G..C. .......... .......... ..........
E24377A      .G........ .......... .......... ........C. .......... .......... ..........
ECOR_34      .G........ .......... .......... .....G..C. .......... .......T.. ..........
ECOR_71      .G........ .......... .......... .....G..C. .......... .......... ..........
SE11         .G........ .......... .......... .....G..C. .......... .......... ..........
O157_Sakai   .G........ .......... .......... ........C. .......... .......... ..........
ECOR_35      .G........ ...C...... .......... ........C. .......... ......C.C. ...G......
ECOR_36      .G........ ...C...... .......... ........C. .......... ......C.C. ...G......
SMS-3-5      .......... .......... .......... ........C. .......... ......C.C. ...G......
536          .G........ .......... .......... .......... .......... ......C.C. ..........
APEC_O1      .G........ .......... .......... .......... .....A.... ......C.C. ..........
CFT073       .G........ .......... .......... .......... .....A.... ......C.C. ..........
E2348/69     .G........ .......... .......... .......... .....A.... ......CTC. ..........
ECOR_51      .G........ .......... .......... .......... .....A.... ......C.C. ..........
ECOR_57      .G........ .......... .......... .......... .....A.... ......C.C. ..........
UTI89        .G........ .......... .......... .......... .....A.... ......C.C. ..........
E._albertii  .........- ---------- ---------- ---------- ---------- ---------- ----------

        19181
K-12         GATAATAAGA ATCATTGTTA TATCAATTAT TATTAATTTT TATGCGTTAT ACGGATAGCA GAAAACTCAC
53638        ...G...... .......... .......... .......... .......... .......... ..........
ATCC_8739    .......... .......... .......... .......... .......... .......... ..........
ECOR_10      .......... .......... .......... .......... .......... .......... ..........
ECOR_11      .......... .......... .......... .......... .......... .......... ..........
B171         .......... .......... .......... .......... .......... .......... ..........
E24377A      .......... .......... .......... .......... .......... .......... ..........
ECOR_34      .......... .......... .......... .......... .......... .......... ..........
ECOR_71      .......... .......... .......... .......... .......... .......... ..........
SE11         .......... .......... .......... .......... .......... .......... ..........
O157_Sakai   .......... .......... .......... .......... .......... .......... ..........
ECOR_35      .......... ..T....... .......... .......... .......... .......... ..........
ECOR_36      .......... ..T....... .......... .......... .......... .......... ..........
SMS-3-5      .......... .......... .......... .......... .......... .......... .....T....
536          .......... .......... ...A...... .......... .......... .......... ..........
APEC_O1      .......... .......... ...A...... .......... .......... .......... ..........
CFT073       .......... .......... ...A...... .......... .......... .......... ..........
E2348/69     .......... .......... ...A...... .......... .......... .......... ..........
ECOR_51      .......... .......... ...A...... .......... .......... .......... ..........
ECOR_57      .......... .......... ...A...... .......... .......... .......... ..........
UTI89        .......... .......... ...A...... .......... .......... .......... ..........
E._albertii  ---------- ---------- ---------- ---------- ---------- ---------- ----------

        19251
K-12         GCCTGAAACG GATGCCAATC ACAAGACCGC TTCCCCGCAG CCTATTCGGC GAATTTCCAG CCAGACACTG
53638        .......... .......... .......... .......... .......... .......... ..........
ATCC_8739    .......... .......... .......... .......... ..C..C.... .....A.... ..........
ECOR_10      .......... .......... .......... .......... .......... .......... ..........
ECOR_11      .......... .......... .......... .......... .......... .......... ..........
B171         .......... .......... .......... .......... .......... .......... ..........
E24377A      .......... .......... .......... .......... .......... .......... ..........
ECOR_34      .......... .......... .......... .......... .......... .......... ..........
ECOR_71      .......... .......... .......... .......... .......... .......... ..........
SE11         .......... .......... .......... .......... .......... .......... ..........
O157_Sakai   .......... .......... .......... .......... .......... .......... ..........
ECOR_35      .......... .......... .......... .......... ..C....... .....A.... ..........
ECOR_36      .......... .......... .......... .......... ..C....... .....A.... ..........
SMS-3-5      .......... .......... .......... .......... ..C....... .....A.... ..........
536          .......... .......... .......... .......... ..C..C.... .....A.... ..........
APEC_O1      .......... .......G.. .......... .......... ..C..C.... .....A.... ..........
CFT073       .......... .......G.. .......... .......... ..C..C.... .....A.... ..........
E2348/69     .......... .......... .......... .......... ..C..C.... .....A.... ..........
ECOR_51      .......... .......G.. .......... .......... ..C..C.... .....A.... ..........
ECOR_57      .......... .......G.. .......... .......... ..C..C.... .....A.... ..........
UTI89        .......... .......G.. .......... .......... ..C..C.... .....A.... ..........
E._albertii  ---------- ---------- ---------- ---------- ---------- ---------- ----------
```

```
         19321
K-12        TTAGGTCCGG ATGGCAAACT GATTATCGAT CATGACGGGC AAGAATATCT GCTCCGTAAA ACTCAGGCTG
53638       .......... .......... .......... .......... .......... .......... ..........
ATCC_8739   .......... .......... .......... .......... .......... .......... ..........
ECOR_10     .......... .......... .......... .......... .......... .......... ..........
ECOR_11     .......... .......... .......... .......... .......... .......... ..........
B171        .......... .......... .......... .......... .......... .......... ..........
E24377A     .......... .......... .......... .......... .......... .......... ..........
ECOR_34     .......... .......... .......... .......... .......... .......... ..........
ECOR_71     .......... .......... .......... .......... .......... .......... ..........
SE11        .......... .......... .......... .......... .......... .......... ..........
O157_Sakai  .......... .......... .......... .......... .......... .......... ..........
ECOR_35     .......... .......... .......... .......... .......... .......... ..........
ECOR_36     .......... .......... .......... .......... .......... .......... ..........
SMS-3-5     .......... .......... .......... .......... .......... .......... ..........
536         .......... .......... .......... .......... .......... .......... ..........
APEC_O1     .......... .......... .......... .......... .......... .......... ..........
CFT073      .......... .......... .......... .......... .......... .......... ..........
E2348/69    .......... .......... .......... .......... .......... .......... ..........
ECOR_51     .......... .......... .......... .......... .......... .......... ..........
ECOR_57     .......... .......... .......... .......... .......... .......... ..........
UTI89       .......... .......... .......... .......... .......... .......... ..........
E._albertii ---------- ---------- ---------- ---------- ---------- ---------- ----------

         19391
K-12        GCAAGCTGCT GTTGACCAAG TAGCCTTTAA CTCGAGCAGC TGACTTCCAG CCGTTTACCC CAGTCAGGTG
53638       .......... .......... .......... .......... .......... .......... ..........
ATCC_8739   .......... ...A..T... .......... .......... .......... .......... ..........
ECOR_10     .......... .......... .......... .......... .......... .......... ..........
ECOR_11     .......... .......... .......... .......... .......... .......... ..........
B171        .......... .......... .......... .......... .......... .......... ..........
E24377A     .......... .......... .......... .......... .......... .......... ..........
ECOR_34     .......... ...A..T... .......... .......... .......... .......... ..........
ECOR_71     .......... .......... .......... .......... .......... .......... ..........
SE11        .......... .......... .......... .......... .......... .......... ..........
O157_Sakai  .......... .......... .......... .......... .......... .......... ..........
ECOR_35     .......... .......... .......... .......... .......... .......... ..........
ECOR_36     .......... .......... .......... .......... .......... .......... ..........
SMS-3-5     .......... .......... .......... .......... .......... .......... ..........
536         .......... .......... ..A....... .......... .......... .......... .....G....
APEC_O1     .......... .......... .......... .......... .......... .......... .....G....
CFT073      .......... .......... .......... .......... .......... .......... .....G....
E2348/69    .......... .......... .......... .......... .......... .......... .....G....
ECOR_51     .......... .......... .......... .......... .......... .......... .....G....
ECOR_57     .......... .......... .......... .......... .......... .......... .....G....
UTI89       .......... .......... .......... .......... .......... .......... .....G....
E._albertii ---------- ---------- ---------- ---------- ---------- ---------- ----------

         19461
K-12        GACGACTGAC ATAGTCATCA TCTCTGTCGC TGAAAGGATT TCGCAACGCC TCATGCAGGC GGTGCAATTC
53638       .......... .......... .......... .......... .......... .......... ..........
ATCC_8739   .......... .......... .......... .......... .......... .......... ..........
ECOR_10     .......... .......... .......... .......... .......... .......... ..........
ECOR_11     .......... .......... .......... .......... .......... .......... ..........
B171        .......... .......... G......... .......... .......... .......... ..........
E24377A     .......... .......... .......... .......... .......... .......... ..........
ECOR_34     .......... .......... .......... .......... .......... .......... ..........
ECOR_71     .......... .......... G......... .......... .......... .......... ..........
SE11        .......... .......... G......... .......... .......... .......... ..........
O157_Sakai  .......... .......... .......... .......... .......... .......... ..........
ECOR_35     .......... .......... .......... .......... .......... .......... ..........
ECOR_36     .......... .......... .......... .......... .......... .......... ..........
SMS-3-5     .......... .......... .......... .......... .......... .......... ..........
536         .......... .......... .......... .......... .......... .......... ..........
APEC_O1     .......... .......... .......... .......... .......... .......... ..........
CFT073      .......... .......... .......... .......... .......... .......... ..........
E2348/69    .......... .......... .......... .......... .......... .......... ..........
ECOR_51     .......... .......... .......... .......... .......... .......... ..........
ECOR_57     .......... .......... .......... .......... .......... .......... ..........
UTI89       .......... .......... .......... .......... .......... .......... ..........
E._albertii ---------- ---------- ---------- ---------- ---------- ---------- ----------
```

```
        19531
K-12         CGTCATATCA CCCTTTCAGT TGCTGACGCT CACTATCGCT AACCTCGTCT TGTTGCAAAC GCCCCCGATA
53638        .......... .......... .......... .......... .......... .......... ..........
ATCC_8739    .A........ .......... .......... .......... .......... .......... ..........
ECOR_10      .......... .......... .......... .......... .......... .......... ..........
ECOR_11      .......... .......... .......... .......... .......... .......... ..........
B171         .......... .........C .......... .......... .......... .......... ..........
E24377A      .......... .........C .......... .......... .......... .......... ..........
ECOR_34      .......... .......... .......... ..A....... .......... .......... ...T......
ECOR_71      .......... .........C .......... .......... .......... .......... ..........
SE11         .......... .........C .......... .......... .......... .......... ..........
O157_Sakai   .......... .......... .......... .......... .......... .......... ....T.....
ECOR_35      .......... .......... .......... ........G. .......... .......... .TA.......
ECOR_36      .......... .......... .......... ........G. .......... .......... .TA.......
SMS-3-5      .......... .......... .......... ..A....... .......... .......... ...T......
536          .......... .......... .......... ........G. ..T....... .......... ..........
APEC_O1      .......... T......... .......... ........G. ..T....... .......... ..........
CFT073       .......... T......... .......... ........G. ..T....... .......... ..........
E2348/69     .......... .........C .......... ........G. .......... .......... .TA.......
ECOR_51      .......... .........C .......... .......... .......... .......... ..........
ECOR_57      .......... .......... .......... ........G. ..T....... .......... ..........
UTI89        .......... T......... .......... ........G. ..T....... .......... ..........
E._albertii  ---------- --------.. ...C.T..T. .........C ...G..A..C ..C..T.... ..G.......

        19601
K-12         ACGGGCAAAC CAGTCATCAA ATGCCGCACG ATCAATAAAC TCATCACGTA GCGGTGACGC CGCGCTGTGC
53638        .......... .......... .......... .......... .......... .......... ..........
ATCC_8739    .......... .......... .......... .......... .......... .......... ..........
ECOR_10      .......... .......... .......... .......... .......... .......... ..........
ECOR_11      .......... .......... .......... .......... .......... .......... ..........
B171         .......... .......... .......... .......... .......... .......... ..........
E24377A      .......... .......... .......... .......... .......... .......... ..........
ECOR_34      .......... .......... .......... .......... .......... .......... ..........
ECOR_71      .......... .......... .......... .......... .......... .......... ..........
SE11         .......... .......... .......... .......... .......... .......... ..........
O157_Sakai   .......... .......... .......... .......... .......... .......... ..........
ECOR_35      .......... .......... .......... ...G...... .......... .......... ........A.
ECOR_36      .......... .......... .......... ...G...... .......... .......... ........A.
SMS-3-5      .......... .......... .......... .......... .......... .......... ..........
536          .......... .......... .......... .......... .......... .......... ..........
APEC_O1      .......... .......... .......... .......... .......... .......... ..........
CFT073       .......... .......... .......... .......... .......... .......... ..........
E2348/69     .......... .......... .......... .......... .......... .......... ..........
ECOR_51      .......... .......... .......... .......... .......... .......... ..........
ECOR_57      .......... .......... .......... .......... .......... .......... ..........
UTI89        .......... .......... .......... .......... .......... .......... ..........
E._albertii  ...T...... ....T..... .C..T..G.. G......... .......... A......A.. .......C..

        19671
K-12         TGCTCGGTCA GACTCAGCAT GCGGAATGTG CGGGTATAAT CGCTGCGCTC TCGCGCCATC AGACTGAATA
53638        .......... .......... .......... .......... .......... .......... ..........
ATCC_8739    .......... .......... .......... .......... .......... .......... ..........
ECOR_10      .......... .......... .......... .......... .......... .......... ..........
ECOR_11      .......... .......... .......... .......... .......... .......... ..........
B171         .......... .......... .......... .......... .......... .......... ..........
E24377A      .......... .......... .......... .......... .......... .......... ..........
ECOR_34      .......... .......... .......... .......... .......... .......... ..........
ECOR_71      .......... .......... .......... .......... .......... .......... ..........
SE11         .......... .......... .......... .......... .......... .......... ..........
O157_Sakai   .......... .......... ...A...... .......... .......... .......... ..........
ECOR_35      .......... .......... .......... .......... .......... .......... ..........
ECOR_36      .......... .......... .......... .......... .......... .......... ..........
SMS-3-5      .......... .......... .......... .......... .......... .......... ..........
536          .......... .......... .......... .......... .......... .......... ..........
APEC_O1      .......... .......... .......... .......... .......... .......... ..........
CFT073       .......... .......... .......... .......... .......... .......... ..........
E2348/69     .......... .......... .......... .......... .......... .......... ..........
ECOR_51      .......... .......... .......... .......... .......... .......... ..........
ECOR_57      .......... .......... .......... .......... .......... .......... ..........
UTI89        .......... .......... .......... .......... .......... .......... ..........
E._albertii  ..T..A...T .......... A..A..C... ........G. ....A..T.. G......... .AG.......
```

```
        19741
K-12         ATTCATTCAG TAGCGCGTTA TCCTCTTTTT GCTCCGTCAT GAAGCCCAGT TTCTGCCGCA TCCGTTCTCC
53638        .......... .......... .......... .......... .......... .......... ..........
ATCC_8739    .......... .......... .......... .......... .......... .......... ......G...
ECOR_10      .......... .......... .......... .......... .......... .......... ..........
ECOR_11      .......... .......... .......... .......... .......... .......... ..........
B171         .......... .......... .......... .......... .......... .......... ......G...
E24377A      .......... .......... .......... .......... .......... .......... ......G...
ECOR_34      .......... .......... .......... .......... .......... .......... ......G...
ECOR_71      .......... .......... .......... .......... .......... .......... ..........
SE11         .......... .......... .......... .......... .......... .......... ......G...
O157_Sakai   .......... .......... .......... .......... A......... .......... ......G...
ECOR_35      .......... .......... .......... .......... .......... .......... ......G...
ECOR_36      .......... .......... .......... .......... .......... .......... ......G...
SMS-3-5      .......... .......... .......... .......... .........C .......... ......G...
536          .......A.. .......... .......... .......... .......... .......... ......G...
APEC_O1      .......A.. .......... .......... .......... .......... .......... ......G...
CFT073       .......A.. .......... .......... .......... .......... .......... ......G...
E2348/69     .......... .......... .......... .......... .......... .......... ......G...
ECOR_51      .......... .......... .......... .......... .......... .......... ......G...
ECOR_57      .......A.. .......... .......... .......... .......... .......... ......G...
UTI89        .......A.. .......... .......... .......... .......... .......... ......G...
E._albertii  .......A.. C....TA..G .......... .TG....... ...C.....C .....G.... .A....GC..

        19811
K-12         ATAATGCGTC AACAAAACCT GCTGATAGCT GTCCAGGGCC TCATTCAGGG CATCTACGGC AACAAATGGC
53638        .......... .......... .......... .......... .......... .......... ..........
ATCC_8739    .......... .......... .......... .......... .......... .......... .........T
ECOR_10      .......... .......... .......... .......... .......... .......... ..........
ECOR_11      .......... .......... .......... .......... .......... .......... ..........
B171         .......... .......... .......... .......... .......... .......... .........T
E24377A      .......... .......... .......... .......... .......... .......... .........T
ECOR_34      .......... .......... .......... .......... .......... .......... .........T
ECOR_71      .......... .......... .......... .......... .......... .......... ..........
SE11         .......... .......... .......... .......... .......... .......... .........T
O157_Sakai   .......... .......... .......... .......... .......... .......... .........T
ECOR_35      .......... .......... .......... .......... .......... .......... .........T
ECOR_36      .......... .......... .......... .......... .......... .......... .........T
SMS-3-5      .......... .......... .......... .......... .......... .......... .........T
536          .......... .......... .......... .......... ........C. ....A..... .........T
APEC_O1      .......... .......... .......... .......... ........C. ....A..... .........T
CFT073       .......... .......... .......... .......... ........C. ....A..... .........T
E2348/69     .......... .......... .......... .......... .......... ....A..... .........T
ECOR_51      .......... .......... .......... .......... ........C. ....A..... .........T
ECOR_57      .......... .......... .......... .......... ........C. ....A..... .........T
UTI89        .......... .......... .......... .......... ........C. ....A..... .........T
E._albertii  ......GC.T .....G..T. .T........ ......C... .......AC. ....G..... ..T...C...

        19881
K-12         GACAATGTCT GCGCCAGACG CTGTAAATTC CACAACGCGA CGGCAGGTTG ATTATCAAAG CTGTAACGCC
53638        .......... .......... .......... .......... .......... .......... ..........
ATCC_8739    .......... .......... .......... .......... .A........ .......... ..A.......
ECOR_10      .......... .......... .......... .......... .......... .......... ..........
ECOR_11      .......... .......... .......... .......... .......... .......... ..........
B171         .......... .......... .......... .......... .......... .......... ..........
E24377A      .......... .......... A......... .......... .A........ .......... ..A.......
ECOR_34      .......... .......... .......... .......... .A........ .......... ..A.......
ECOR_71      .......... .......... .......... .......... .......... .......... ..........
SE11         .......... .......... .......... .......... .......... .......... ..........
O157_Sakai   .......... .......... .......A.. .......... .A........ ...G...... ........T.
ECOR_35      .......... .......... .......... .......... .A........ .......... ..A.......
ECOR_36      .......... .......... .......... .......... .A........ .......... ..A.......
SMS-3-5      .......... .......... .......... .......... .A........ .......... ..A.......
536          .......... .......... .......... .......... .......... .......... ..........
APEC_O1      .......... .......... .......... .......... .......... .......... ..........
CFT073       .......... .......... .......... .......... .......... .......... ..........
E2348/69     .......... .......... .......... .......... .......... .......... ..........
ECOR_51      .......... .......... .......... .......... .......... .......... ..........
ECOR_57      .......... .......... .......... .......... .......... .......... ..........
UTI89        .......... .......... .......... .......... .......... .......... ..........
E._albertii  .......... .......... .......... ........T. .......... .......... ..A.......
```

```
             19951
K-12            CTTGATGATC CGAGTGATTA CAAATAAAAC CGGGTTCGTA ATCATCAAGA AAACCAAACG GCCCGTAATC
53638           .......... .......... .......... .......... .......... .......... ..........
ATCC_8739       .......... .......... .......... .......... .......... .......... ..........
ECOR_10         .......... .......... .......... .......... .......... .......... ..........
ECOR_11         .......... .......... .......... .......... .......... .......... ..........
B171            .......... .......... .......... .......... .......... .......... ..........
E24377A         .......... .......... .......... .......... ......C... .......... ..........
ECOR_34         .......... .......... .......... .......... .......... .......... ..........
ECOR_71         .......... .......... .......... .......... .......... .......... ..........
SE11            .......... .......... .......... .......... .......... .......... ..........
O157_Sakai      .......... .......... .......... .......... .....T.... .......... ..........
ECOR_35         .......... .......... .......... .......... .......... .......... ..........
ECOR_36         .......... .......... .......... .......... .......... .......... ..........
SMS-3-5         .......... .......... .......... .......... .......... .......... ..........
536             .......... .......... .......... .......... .......... .......... ..........
APEC_O1         .......... .......... .......... .......... .......... .......... ..........
CFT073          .......... .......... .......... .......... .......... .......... ..........
E2348/69        .......... .......... .......... .......... .......... .......... ..........
ECOR_51         .......... .......... .......... .......... .......... .......... ..........
ECOR_57         .......... .......... .......... .......... .......... .......... ..........
UTI89           .......... .......... .......... .......... .......... .......... ..........
E._albertii     .C..G..... A......... .......... .C.AC..A.. .......... .......... ..........

             20021
K-12            AAGCGTCAGC CCCAGCAGCG ACATGTTGTC GGTATTCATC ACCCCATGAG CAAAGCCGAC CGTCTGCCAT
53638           ........A. .......... .......... .......... .......... .......... ..........
ATCC_8739       .........T .......... .......... .......... ........T. .......... ..........
ECOR_10         .......... .......... .......... .......... .......... .......... ..........
ECOR_11         .......... .......... .......... .......... .......... .......... ..........
B171            .......... .......... .......... .......... .......... .......... ..........
E24377A         .......... .......... .......A.. .......... .......... .......... ..........
ECOR_34         .......... .......... .......... .......... .......... .......... ..........
ECOR_71         .......... .......... .......... .......... .......... .......... ..........
SE11            .......... .......... .......... .......... .......... .......... ..........
O157_Sakai      .........T .......... .......... .......... ........T. .......... ..........
ECOR_35         .......... .......... .......... .......... .......... .......... ..........
ECOR_36         .......... .......... .......... .......... .......... .......... ..........
SMS-3-5         .......... .......... .......... .......... .......... .......... ..........
536             .......... .......... .......... .......... .......... .......... ..........
APEC_O1         .......... .......... .......... .......... .......... .......... ..........
CFT073          .......... .......... .......... .......... .......... .......... ..........
E2348/69        .......... .......... .......... .......... ..T....... .......... ..........
ECOR_51         .......... .......... .......... .......... .......... .......... ..........
ECOR_57         .......... .......... .......... .......... .......... .......... ..........
UTI89           .......... .......... .......... .......... .......... .......... ..........
E._albertii     G......... ..G..A..A. .......... .........T .....G.... .......... .........G

             20091
K-12            TGGGCAATTA ACGACGCGGT ACGTGCGACA ACATCGCTAA ACCAGAGACG GTATTTGTCC TCATCATCTG
53638           .......... .......... .......... .......... .......... .......... .........T
ATCC_8739       .......... .......... .......... .......... .......... .......... .........T
ECOR_10         .......... .......... .......... .......... .......... .......... ..........
ECOR_11         .......... .......... .......... .......... .......... .......... ..........
B171            .......... ....T..... .......... ......T... .......... .......... .........T
E24377A         .......... ....T..... .......... .......... .......G.. .......... .........T
ECOR_34         .......... ....T..... .......... ......G... .......... .......... ..........
ECOR_71         .......... .......... .......... .......... .......... .......... .........T
SE11            .......... ....T..... .......... ......T... .......... .......... .........T
O157_Sakai      .......... ....T..... .......... ......T... .......... .......... .........T
ECOR_35         .......... G...T..... .......... .......... .......... .......... .........T
ECOR_36         .......... G...T..... .......... .......... .......... .......... .........T
SMS-3-5         .......... ....T..... .......... .......... .......... .......... ..........
536             .......... ....G..... .......... ......G... .......... .......... ..C.....AT
APEC_O1         .......... ....G..... .......... ......G... .......... .......... ..C.....AT
CFT073          .......... ....G..... .......... ......G... .......... .......... ..C.....AT
E2348/69        .......... ....G..... .......... ......G... .......... .......... ..C.....AT
ECOR_51         .......... ....G..... .......... ......G... .......... .......... ..C.....AT
ECOR_57         .......... ....G..... .......... ......G... .......... .......... ..C.....AT
UTI89           .......... ....G..... .......... ......G... .......... .......... ..C.....AT
E._albertii     C......... ....A..C.. ...C...... .....TG... .......G.. A.....A... .......TCA
```

```
        20161
K-12         CAAGATGTGA CCAGTAATGA CGAATAGCAA AGTCAGCTAA CTGACGAACC TTTTCCGACT CGCGGCGGTA
53638        .......... .......... .......... .......... .......... .......... ..........
ATCC_8739    .......... .......... .......... .......... .......... .......... ..........
ECOR_10      .......... .......... .......... .......... .......... .......... ..........
ECOR_11      .......... .......... .......... .......... .......... .......... ..........
B171         .......... .......... .....G.... .......... .......... .......G.. .......A..
E24377A      ......A... .......... .....G.... .......... .......... .......G.. .......A..
ECOR_34      .......... .......... .....G.... .......... .......... .......G.. .......A..
ECOR_71      .......... .......... .......... .......... .......... .......... ..........
SE11         .......... .......... .....G.... .......... .......... .......G.. .......A..
O157_Sakai   .......... .......... .....G.... .......C.. .......... ..A....G.. .......A..
ECOR_35      .......... .......... .....G.... .......C.. .......... .......G.. .......A..
ECOR_36      .......... .......... .....G.... .......C.. .......... .......G.. .......A..
SMS-3-5      .......... .......... .....G.... .......C.. .......... .......G.. .......A..
536          .......... .......... .....G.... .......... .......... .......G.. .......A..
APEC_O1      .......... .......... .....G.... .......... .......... .......G.. .......A..
CFT073       .......... .......... .....G.... .......... .......... .......G.. .......A..
E2348/69     .......... .......... .....G.... .......... .......... .......G.. .......A..
ECOR_51      .......... .......... .....G.... .......... .......... .......G.. .......A..
ECOR_57      .......... .......... .....G.... .......... .......... .......G.. .......A..
UTI89        .......... .......... .....G.... .......... .......... .......G.. .......A..
E._albertii  G......C.G .........G .....G..G. ......TCC. ...G.....T .......G.. .C.....A..

        20231
K-12         GTAAAAATGT TCGAAATGAC CAAAGCGCAG ATGACTTGGT GCCACACGCA TCAGCATCGC GCCTGGCTCC
53638        .......... .......... .......... .......... .......... .......... ..........
ATCC_8739    .......... .......... .......... .......... .......... .......... ..........
ECOR_10      .......... .......... .......... .......... .......... .......... ..........
ECOR_11      ........A. .......... .......... .......... .......... .......... ..........
B171         .......... .......... .......... .......... ........T. .......... ..........
E24377A      .......... .......... .......... .......... .......... .......... ..........
ECOR_34      .......... .......... .......... .......... ........T. .......... ..........
ECOR_71      .......... .......... .......... .......... .......... .......... ..........
SE11         .......... .......... .......... .......... ........T. .......... ..........
O157_Sakai   .......... .......... .......... .......... .......... .......... ..........
ECOR_35      .......... .......... .......... .......A.. .......... .......... ..........
ECOR_36      .......... .......... .......... .......A.. .......... .......... ..........
SMS-3-5      .......... .......... .......... .......... .......... .......... ..........
536          .......... .......... .......... .......... .......... .......... .....A....
APEC_O1      .......... .......... .......... .......... .......... .......... .....A....
CFT073       .......... .......... .......... .......... .......... .......... .....A....
E2348/69     .......... .......... .......... .......... .......... .......... .....A....
ECOR_51      .......... .......... .......... .......... .......... .......... .....A....
ECOR_57      .......... .......... .......... .......... .......... .......... .....A....
UTI89        .......... .......... .......... .......... .......... .......... .....A....
E._albertii  .......... ..A....... ....T..T.A ...G...T.C .....G.... .......... .....AT..G

        20301
K-12         GCCGTTTCCC GATACACTGG CGAATCGCTG GTGACGATAC TTAACGCGCG GGTCGTCGGA ATGCCCAGAT
53638        .......... .......... .......... .......... .......... .......... ..........
ATCC_8739    .......... .......... .......... .......... .......... .......... ..........
ECOR_10      .......... .......... .......... .......... .......... .......... ..........
ECOR_11      .......... .......... .......... .......... .......... .......... ..........
B171         A......... .......... .......... .......... .......... .......... ..........
E24377A      A......... .......... .......... .......... .......... .......... ..........
ECOR_34      A......... .......... .......... .......... .......... .......... ..........
ECOR_71      .......... .......... .......... .......... .......... .......... ..........
SE11         A......... .......... .......... .......... .......... .......... ..........
O157_Sakai   A......... .......... .......... .......... .......... .......... ..........
ECOR_35      AT........ .......... .......... .......... .......... .......... ..........
ECOR_36      AT........ .......... .......... .......... .......... .......... ..........
SMS-3-5      A......... .......... .......... .A........ .......... .......... ..........
536          A......... .......... .......... .......... ....A..... .......... ..........
APEC_O1      A......... .......... .......... .......... ....A..... .......... ..........
CFT073       A......... .......... .......... .......... ....A..... .......... ..........
E2348/69     A......... .......... .......... .......... ....A..... .......... ..........
ECOR_51      A......... .......... .......... .......... ....A..... .......... ..........
ECOR_57      A......... .......... .......... .......... ....A..... .......... ..........
UTI89        A......... .......... .......... .......... ....A..... .......... ..........
E._albertii  A....C.... .G..G..C.. ..T...A... ..A..A..G. .C..T..... C......... ........G.
```

```
        20371
K-12         AATGCATCGC CTCACTGGCG AGACTTTCTC GTATCGTCGA ACGTAAAACC GCCCGTCCAT CACCCATTCG
53638        .......... .......... .......... .......... .......... .......... ..........
ATCC_8739    .......... .......... .......... .......... .......... .......... ..........
ECOR_10      .......... .......... .......... .......... .......... .......... ..........
ECOR_11      .......... .......... .......... .......... .......... .......... ..........
B171         .......... .......... .......... .......... .......... .......... ..........
E24377A      .......... .......... .......... .......... .......... .......... ..........
ECOR_34      .......... .......... .......... .......... .......... .......... ..........
ECOR_71      .......... .......... .......... .......... .......... .......... ..........
SE11         .......... .......... .......... .......... .......... .......... ..........
O157_Sakai   .......... .......... .......... .......... ......T... .......... ..........
ECOR_35      .......... .......... .......... .......... .......... .......... ..........
ECOR_36      .......... .......... .......... .......... .......... .......... ..........
SMS-3-5      .......... .......... .......... .......... .........T .......... ..........
536          .......... .......... .......... .......... .......... .......... .C........
APEC_O1      .......... .......... .......... .......... .......... .......... .C........
CFT073       .......... .......... .......... .......... .......... .......... .C........
E2348/69     .......... .......... .......... .......... .......... .......... .C........
ECOR_51      .......... .......... .......... .......... .......... .......... .C........
ECOR_57      .......... .......... .......... .......... .......... .......... .C........
UTI89        .......... .......... .......... .......... .......... .......... .C........
E._albertii  G......T.. .........A ........G. .G..T..... T..C..T... .......... .T.....C..

        20441
K-12         CGAATAAGGC GTCAGGCCAG CACCTTTCAG ATGCCAGTCC ATTGTAGTGC CATCAGCAAG CAGTTGTTCG
53638        .......... .......... .......... .......... .......... .......... ..........
ATCC_8739    .......... .......... .......... .......... .......... .......... ..........
ECOR_10      .......... .......... .......... .......... .......... .......... ..........
ECOR_11      .......... .......... .......... .......... .......... .......... ..........
B171         .......... .......... .......... .......... .......... .......... ..........
E24377A      .......... .......... .......... .......... .......... .......... ..........
ECOR_34      .......... .......... .......... .......... .......... .......... ..........
ECOR_71      .......... .......... .......... .......... .......... .......... ..........
SE11         .......... .......... .......... .......... .......... .......... ..........
O157_Sakai   .......... .......... .......... .......... .......... .......... ..........
ECOR_35      .......... .......... .......... .......... .......... .......... .C........
ECOR_36      .......... .......... .......... .......... .......... .......... .C........
SMS-3-5      .......... .......... .......... .......... .......... ....G..C.. .T........
536          .......... .......... .......... .......... .......... .......... ..........
APEC_O1      .......... .......... .......... .......... .......... .......... ..........
CFT073       .......... .......... .......... .......... .......... .......... ..........
E2348/69     .......... .......... .......... .......... .......... .......... ..........
ECOR_51      .......... .......... .......... .......... .......... .......... ..........
ECOR_57      .......... .......... .......... .......... .......... .......... ..........
UTI89        .......... .......... .......... .......... .......... .......... ..........
E._albertii  T.....C..T ..T..C..C. .G........ .......... .....GC... ....T..... .T.C......

        20511
K-12         CCGAGTAAAA TGCCGCGCCC ATCACCCAGT TGGCCCGCCC AGACGCCGAA CTGATGACCA CTGTAAACCT
53638        .......... .......... .......... .......... .......... .......... ..........
ATCC_8739    .......... .......... .......... .......... .......... .......... ..........
ECOR_10      .......... .......... .......... .......... .......... .......... ..........
ECOR_11      .......... .......... .......... .......... .......... .......... ..........
B171         ..A....... .......... .......... .......... .......... .......... ..........
E24377A      .......... .A........ .......... .......... .......... .......... ..........
ECOR_34      ..A....... .......... .......... .......... .......... .......... ..........
ECOR_71      .......... .......... .......... .......... .......... .......... ..........
SE11         ..A....... .......... .......... .......... .......... .......... ..........
O157_Sakai   ..A....... .......... .......... .......... .......... .......... ..........
ECOR_35      .......... .......... .......... .......... .......... .......... ..........
ECOR_36      .......... .......... .......... .......... .......... .......... ..........
SMS-3-5      .......... .......... .......... .......... .......... .......... ..........
536          .......... .......... .......... .......... .......A.. .......... ..........
APEC_O1      .......... .......... .......... .......... .......A.. .......... ..........
CFT073       .......... .......... .......... .......... .......A.. .......... ..........
E2348/69     .......... .......... .......... .......... .......A.. .......... ..........
ECOR_51      .......... .......... .......... .......... .......A.. .......... ..........
ECOR_57      .......... .......... .......... .......... .......A.. .......... ..........
UTI89        .......... .......... .......... .......... .......A.. .......... ..........
E._albertii  .......... .......... G..C...... ..T....... ...T...A.. .........G .....C....
```

```
        20581
K-12         GCGCCAGTGG TGACATACCA GGCAGTAGGG CTTCGCCGCC CTGAACCAAC GTCGTTATCT GATTTATGGG
53638        .......... .......... .......... .......... .......... .......... ..........
ATCC_8739    .......... .......... .......... .......... .......... .......... ..........
ECOR_10      .......... .......... .......... T......... .......... .......... ..........
ECOR_11      .......... .......... .......... .......... .......... .......... ..........
B171         .......... .......... .......... T......... .......... .......... ..........
E24377A      .......... .......... .......... T......... .......... .......... .......A..
ECOR_34      .......... .......... .......... T......... .......... .......... .......A..
ECOR_71      .......... .......... .......... .......... .......... .......... ..........
SE11         .......... .......... .......... T......... .......... .......... ..........
O157_Sakai   .......... .......... .......... T......... .......... .......... ..........
ECOR_35      .......... .......... .......... T......A.. .......... .......... ......CA..
ECOR_36      .......... .......... .......... T......A.. .......... .......... ......CA..
SMS-3-5      .......... .......... .......... T......... .......... .......... ......CA..
536          ....A..... .........C .........T T......... .......... .......... .......A..
APEC_O1      ....A..... .........C .........T T......... .......... .......... .......A..
CFT073       ....A..... .........C .........T T......... .......... .......... .......A..
E2348/69     ....A..... .........  .........T T......... ......T... .......... .......A..
ECOR_51      ....A..... .........C .........T T......... .......... .......... .......A..
ECOR_57      ....A..... .........C .........T T......... .......... .......... .......A..
UTI89        ....A..... .........C .........T T......... .......... .......... .......A..
E._albertii  .G....AC.. .........T .......--- ---------- ...C....GT ..T....C.. ..C...C...

        20651
K-12         AACGGCAGGC CACAGGCATC CCTGGAGAGC ATGGAAATCG AAGGGGGTAA TCTGTGCCAA TATTTCTGCC
53638        .......... .......... .......... .......... .......... .......... ..........
ATCC_8739    .......... .......... .......... .......... .......... .......... ..........
ECOR_10      .......... .......... .......... .......... .......... .......... ..........
ECOR_11      .......... .......... .......... .......... .......... .......... ..........
B171         .......... .......... .......... .......... .......... .......... ..........
E24377A      .......... .......... .......... .......... .......... .......... ........T.
ECOR_34      .......... .......... .......... .......... .......... .......... ........T.
ECOR_71      .......... .......... .......... .......... .......... .......... ..........
SE11         .......... .......... .......... .......... .......... .......... ..........
O157_Sakai   .......... .......... .......... .........  .......... .......T.. ........G.
ECOR_35      .......... .......... .......... .........A .......... .........T ........G.
ECOR_36      .......... .......... .......... .........A .......... .........T ........G.
SMS-3-5      .......... .......... .......... .........A .......... .........T ........G.
536          ...C...... .......... .......... .........A ......A... .......... ........G.
APEC_O1      ...C...... .......... .......... .........A ......A... .......... ........G.
CFT073       ...C...... .......... .......... .........A ......A... .......... ........G.
E2348/69     ...C...... .......... .......... .........A ......A... .......... ........G.
ECOR_51      ...C...... .......... .......... .........A ......A... .......... ........G.
ECOR_57      ...C...... .......... .......... .........A ......A... .......... ........G.
UTI89        ...C...... .......... .......... .........A ......A... .......... ........G.
E._albertii  ......G... .......CC. .T..A.A... G..A....TA ..C..T.CT. C.....T... C..C....GT

        20721
K-12         GTATCAATGC CTCCCGCAAT GATGCAATTG CAACAAGGGG AAATTTGCGC CTGGATCGCA CGAATAAACG
53638        .......... .......... .......... .......... .......... .......... ..........
ATCC_8739    .......... .......... .......... .......... .......... .......... ..........
ECOR_10      .......... .......... .......... .......... .......... .......... ..........
ECOR_11      .......... .......... .......... .......... .......... .......... ..........
B171         .......... .......... .......... .......... .........A .......... ..........
E24377A      .......... .C.....G.. .......... .......... .......... .......... ..........
ECOR_34      .......... .C.....G.. .......... .......... .......... .......... ..........
ECOR_71      .......... .......... .......... .......... .......... .......... ..........
SE11         .......... .......... .......... .......... .........A .......... ..........
O157_Sakai   .......... .C.....G.. .......... .......... .......... ...A...... ..........
ECOR_35      .......... .C.....G.. .......... .....T.... .......... .......... ..........
ECOR_36      .......... .C.....G.. .......... .....T.... .......... .......... ..........
SMS-3-5      .......... .C.....G.. .......... .....T.... .......... .......... ..........
536          .......... .C.....G.. .......... .......... .......... .......... ..........
APEC_O1      .......... .C.....G.. .......... .......... .......... .......... ..........
CFT073       .......... .C.....G.. .......... .......... .......... .......... ..........
E2348/69     .......... .C.....G.. .......... .......... .......... .......... ..........
ECOR_51      .......... .C.....G.. .......... .......... .......... .......... ..........
ECOR_57      .......... .C.....G.. .......... .......... .......... .......... ..........
UTI89        .......... .C.....G.. .......... .......... .......... .......... ..........
E._albertii  ..G....... .AGA.A.... ...A.T.... ..G..G..T. .......... ACA..GT... ........T.
```

```
               20791
K-12           GTTCGAAGGA GCGGTGCGTG ATTTGTTGCT GAATAAAACT TTTATCCAGC ATGACGCGGG TGAAAAGACC
53638          .......... .......... .......... .......... .......... .......... ..........
ATCC_8739      .......... .......... .......... .......... .......... .......... ..........
ECOR_10        .......... .......... .......... .......... .......... .......... ..........
ECOR_11        .......... .......... .......... .......... .......... .......... ..........
B171           .......... .......... .......... .......... .......... .......... ..........
E24377A        .......... ...A...... .......... .......... .......... .......... ..........
ECOR_34        .......... ...A...... .......... .......... .......... .......... ..........
ECOR_71        .......... .......... .......... .......... .......... .......... ..........
SE11           .......... .......... .......... .......... .......... .......... ..........
O157_Sakai     .......... ...A...... .......... .......... .......... ..T....... ..........
ECOR_35        .......... ...A...... .......... .......... .......... .......... ..........
ECOR_36        .......... ...A...... .......... .......... .......... .......... ..........
SMS-3-5        .......... ...A...... .......... .......... .......... .......... ..........
536            .......... A..A...... .......... .......... .......... .......... ..........
APEC_O1        .......... A..A...... .......... .......... .......... .......... ..........
CFT073         .......... ...A...... .......... .......... .......... .......... ..........
E2348/69       .......... A..A...... .......... .......... .......... .......... ..........
ECOR_51        .......... ...A...... .......... .......... .......... .......... ..........
ECOR_57        .......... ...A...... .......... .......... .......... .......... ..........
UTI89          .......... A..A...... .......... .......... .......... .......... ..........
E._albertii    .GG.A..... TGAA..T.AT .....CC.T. ...C...GT. C......... ..A......C .A.....C..

               20861
K-12           ATCAAAAACC GCTTTCATTG TGCTATTGCC TGCTCCCAAA TTACCTAACA CAAGGGGATA CACTTGCGAT
53638          .......... .......... .......... .......... .......... .......... ..........
ATCC_8739      .......... .......... .......... .......... .........G .......... ..........
ECOR_10        .......... .......... .......... .......... .......... .......... ..........
ECOR_11        .......... .......... .......... .......... .......... .......... ..........
B171           .......... .......... .......... .......... .......... .......... ..........
E24377A        .......... .......... .......A.. .......... ..G....... .......... ..T.......
ECOR_34        .......... .......... .......A.. .......... ..G....... .......... ..T.......
ECOR_71        .......... .......... .......... .......... .......... .......... ..........
SE11           .......... .......... .......... .......... .......... .......... ..........
O157_Sakai     .......... .......... ....G..... .......... .......... .......... ..........
ECOR_35        .......... ...C...... .......... ...G...... .......... .......... ..T.......
ECOR_36        .......... ...C...... .......... ...G...... .......... .......... ..T.......
SMS-3-5        .......... .......... .......... ...G...... .......... .......... ..T.......
536            .......... .......... .......A.. .......... .......... .......... ..T.......
APEC_O1        .......... .......... .......A.. .......... .......... .......... ..T.......
CFT073         ......C... .......... .......A.. .......... .......... .......... ..T.......
E2348/69       .......... .......... .......A.. .......... .......... .......... ..T.......
ECOR_51        ......C... .......... .......A.. .......... .......... .......... ..T.......
ECOR_57        ......C... .......... .......A.. .......... .......... .......... ..T.......
UTI89          .......... .......... .......A.. .......... .......... .......... ..T.......
E._albertii    ......G.T. ..G.....C. .A..G..... C..G...... ..G..C..A. ....T.A... A.T.......

               20931
K-12           AAGGATAAAA GACCGCGATT ATCTTTTCCT TCATTGAGAT GCGGGTAATT CTCATTAATC AACAGTTCAA
53638          .......... .......... .......... .......... .......... .......... ..........
ATCC_8739      .......... .......... .......... .......... .......... .......... ..........
ECOR_10        .......... .......... .......... .......... .......... .......... ..........
ECOR_11        .......... .......... .......... .......... .......... .......... ..........
B171           .......... .......... .......... .......... .......... .......... ..........
E24377A        .......... ..T....... .......... .......... .......... .......... ..........
ECOR_34        .......... ..T....... .......... .......... .......... .......... ..........
ECOR_71        .......... .......... .......... .......... .......... .......... ..........
SE11           .......... .......... .......... .......... .......... .......... ..........
O157_Sakai     .......... .......... .......... .......... .......... .......... ..........
ECOR_35        .....C.... ..T....... .......... ....C..... .......... .......... ..........
ECOR_36        .....C.... ..T....... .......... ....C..... .......... .......... ..........
SMS-3-5        .....A.... ..T....... .......... ....C..... .......... .......... ..........
536            .......... ..T....... .......... .......A.. .......... .......... ..........
APEC_O1        .......... ..T....... .......... .......A.. .......... .......... ..........
CFT073         .......... ..T....... .......... .......A.. .......... .......... ..........
E2348/69       .......... ..T....... .......... .......A.. .......... .......... ..........
ECOR_51        .......... ..T....... .......... .......A.. .......... .......... ..........
ECOR_57        .......... ..T....... .......... .......A.. .......... .......... ..........
UTI89          .......... ..T....... .......... .......A.. .......... .......... ..........
E._albertii    ...TGC.... .CT...A... .........C G....A..T. .T..A..... T...G.T... .G..A.....
```

```
        21001
K-12          TAAACGGATA TTTTAATAGT TCGCCTGCGT AATTATCCCT CTCTAATAAT AATGTAGCCA CTTGTGGCGT
53638         .......... .......... .......... .......... .......... .......... ..........
ATCC_8739     .......... .......... .......... .....C.... .......... .......... ..........
ECOR_10       .......... .......... .......... .......... .......... .......... ..........
ECOR_11       .......... .......... .......... .......... .......... .......... ..........
B171          .......... .......... .......... .......... .......... .......... ..........
E24377A       .......... .......... .......... .......... .......... .......... ..........
ECOR_34       .......... .......... .......... .......... .......... .......... ..........
ECOR_71       .......... .......... .......... .......... .......... .......... ..........
SE11          .......... .......... .......... .......... .......... .......... ..........
O157_Sakai    .......... .......... .......... .......... .......... .......... ..........
ECOR_35       .......... .......... .....C.... .......... G......... ..A....... .......G..
ECOR_36       .......... .......... .....C.... .......... G......... ..A....... .......G..
SMS-3-5       .......... .......... .....C.... .......... G......... ..A....... .......G..
536           .......... .......... .........A .......... G......... .......... .G........
APEC_O1       .......... .......... .........A .......... G......... .......... .G........
CFT073        .......... .......... .........A .......... G......... .......... ..........
E2348/69      .......... .......... .........A .......... G......... .......... .G........
ECOR_51       .......... .......... .........A .......... G......... .......... ..........
ECOR_57       .......... .......... .........A .......... G......... .......... ..........
UTI89         .......... .......... .........A .......... G......... .......... .G........
E._albertii   ....A...A. ....G...A. .......T.. T......A.C G.T......G CG.T.T.TT. .......T..

        21071
K-12          TAAATTAAGC CAGGCAAAGA GTTTATGTTG AATAAAAAAA TGTTGGCATG ATTTTAGTAA TTCCAACTGC
53638         .......... .......... .......... .......... .......... .......... ..........
ATCC_8739     .......... .......... .......... .......... .......... .......... ..........
ECOR_10       .......... .......... .......... .......... .......... .......... ..........
ECOR_11       .......... .......... .......... .......... .......... .......... ..........
B171          .......... .......... .......... .......... .......... .......... ..........
E24377A       .......... .......... .......... .......... .......... .......... ..........
ECOR_34       .......... .......... .......... .......... .......... .......... ..........
ECOR_71       .......... .......... .......... .......... .......... .......... ..........
SE11          .......... .......... .......... .......... .......... .......... ..........
O157_Sakai    .......... .......... .......... .......... .......... .......... ..........
ECOR_35       .......... .......... .......... .........G .......... .......C.. ..........
ECOR_36       .......... .......... .......... .........G .......... .......C.. ..........
SMS-3-5       .......... .......... .......... .........G .......... .......C.. ..........
536           .......... .......... .......... .........G .......... .......... ..........
APEC_O1       .......... .......... .......... .........G .......... .......... ..........
CFT073        .......... .......... .......... .........G .......... .......... .........T
E2348/69      .......... .......... .......... .........G .......... .......... ..........
ECOR_51       .......... .......... .......... .........G .......... .......... .........T
ECOR_57       .......... .......... .......... .........G .......... .......... .........T
UTI89         .......... .......... .......... .........G .......... .......... ..........
E._albertii   ........T. ........T. T...G..C.. .........G ..C....... ...CG..... ..T...GT..

        21141
K-12          TCTGAAAATA ATTGCCAATG TTGCTCCTCG GTAAGTTGCG CTATAACCCG ACTCGTCGGG ATACGAACCG
53638         .......... .......... .......... .......... .......... .......... .....C....
ATCC_8739     .......... .......... .......... .......... .......... .......... .....C....
ECOR_10       .......... .......... .......... .......... .......... .......... ..........
ECOR_11       .......... .......... .......... .......... .......... .......... ..........
B171          .......... .......... .......... .......... .......... .......... .....C....
E24377A       .......... .......... .......... .......... .......... .......... ..........
ECOR_34       .......... .......... .......... .......... .......... .......... .....C....
ECOR_71       .......... .......... .......... .......... .......... .......... .....C....
SE11          .......... .......... .......... .......... .......... .......... .....C....
O157_Sakai    .......... .......... .......... .......... .......... .......... .....C....
ECOR_35       ..A....... .......... .......... .......... .......... .........T ..........
ECOR_36       ..A....... .......... .......... .......... .......... .........T ..........
SMS-3-5       ..A....... .......... .......... .......... .......... .........T ..........
536           .......... .......... .......... .......... .......... .......... ..........
APEC_O1       .......... .......... .......... .......... .......... .......... ..........
CFT073        ..A....... .......... .......A.. .......... .......... .......... ..........
E2348/69      .......... .......... .......... .......... .......... .......... ..........
ECOR_51       ..A....... .......... .......A.. .......... .......... .......... ..........
ECOR_57       ..A....... .......... .......A.. .......... .......... .......... ..........
UTI89         .......... .......... .......... .......... .......... .......... ..........
E._albertii   ..G.TG.... GC........ C.....T..T .AGCA..... .C.GC..T.. CG.T.CA..A .........T
```

```
         21211
K-12          TACCATCTTC ACTGGAGAAA TGGGTAATTA ACTCAACGCC GACAAGGACT TGCTGGTTGT CTCTTATCGG
53638         .......... .......... .......... .......... .......... ........A. ..........
ATCC_8739     .G........ .......... .......... .......... .......... .......... ..........
ECOR_10       .......... .......... .......... .......... .......... .......... ..........
ECOR_11       .......... .......... .......... .......... .......... .......... ..........
B171          .G........ .......... .......... .......... ........G. ........A. ..........
E24377A       .......... .......... .......... .......... .......T.. ........A. ..........
ECOR_34       .G........ .......... .......... .......... ........G. ........A. ..........
ECOR_71       .G........ .......... .......... .......... .......... .......... ..........
SE11          .G........ .......... .......... .......... ........G. ........A. ..........
O157_Sakai    .G........ .......... .......... .......... .......... ........A. ..........
ECOR_35       .......... .......... .......... .......... .......T.. ........A. ..........
ECOR_36       .......... .......... .......... .......... .......T.. ........A. ..........
SMS-3-5       .......... .......... .......... .......... .......T.. ........A. ..........
536           .......... .......... .......... .......... .......T.. ........A. ..........
APEC_O1       .......... .......... .......... .......... .......T.. ........A. ..........
CFT073        .......... .......... .......... .......... .......T.. ........A. ..........
E2348/69      .......... .......... .......... .......... .......T.. ........A. ..........
ECOR_51       .......... .......... .......... .......... .......T.. ........A. ..........
ECOR_57       .......... .......... .......... .......... .......T.. ........A. ..........
UTI89         .......... .......... .......... .......... .......T.. ........A. ..........
E._albertii   .G.TG...GA G.....A... .....T.... T...T..T.. A.....ATG. .....AC.A. .......T..

         21281
K-12          CAGGAAATAG CAATCAGAAT GATAAAGATT CTCCAAAAAA ATCTTCATGG TAACCGTCCC TCTCGAAGGA
53638         .......... .......... .......... .......... .......... .......... ..........
ATCC_8739     .......... .......... .......... .......... .......... .......... ..........
ECOR_10       .......... .......... .......... .......... .......... .......... ..........
ECOR_11       .......... .......... .......... .......... .......... .......... ..........
B171          .......... .......... .......... .......... .......... .......... ..........
E24377A       .......... .......... .......... .......... .......... .......... ..........
ECOR_34       .......... .......... .......... .......... .......... .......... ..........
ECOR_71       .......... .......... .......... .......... .......... .......... ..........
SE11          .......... .......... .......... .......... .......... .......... ..........
O157_Sakai    .......... .......... .......... .......... .......... .......... ..........
ECOR_35       .......... .......... .......... .......... .......... .......... ..........
ECOR_36       .......... .......... .......... .......... .......... .......... ..........
SMS-3-5       .......... .......... .......... .......... .......... .......... ..........
536           .......... .......... .......... .......... .......... .......... ..........
APEC_O1       .......... .......... .......... .......... .......... .......... ..........
CFT073        .......... .......... .......... .......... .......... .....T.... ..........
E2348/69      .......... .......... .......... .......... .......... .......... ..........
ECOR_51       .......... .......... .......... .......... .......... .....T.... ..........
ECOR_57       .......... .......... .......... .......... .......... .....T.... ..........
UTI89         .......... .......... .......... .......... .......... .......... ..........
E._albertii   .T.A...... T......... ........C. .....GT... .C........ ...T..C.TT .T.AT.....

         21351
K-12          TGTTTTTCAG TATCCGGCTA AAAGGGATGA GGCCATAAGA CATAACAATT ACAGAAGGAG TAACTTTCAT
53638         .......... .......... ........A. .......... .......... .......... ..........
ATCC_8739     .......... .......... .......... .......... .......... .......... ..........
ECOR_10       .......... .......... .......... .......... .......... .......... ..........
ECOR_11       .......... .......... .......... .......... .......... .......... ..........
B171          .......... .......... .......... .......... .......... .......... ..........
E24377A       .......... .......... .......... .......... .......... ...T...... ..........
ECOR_34       .......... .......... .......... .......... .......... .......... ..........
ECOR_71       .......... .......... .......... .......... .....T.... .......... ..........
SE11          .......... .......... .......... .......... .......... .......... ..........
O157_Sakai    .......... .......... .......... .......... .......... .......... ..........
ECOR_35       .......... .......... .......... .......... .......... .......... ..........
ECOR_36       .......... .......... .......... .......... .......... .......... ..........
SMS-3-5       .......... .......... .......... .......... .......... .......... ..........
536           .......... .......... .......... .......... .......... .......... ..........
APEC_O1       .......... .......... .......... .......... .......... .......... ..........
CFT073        .......... .......... .......... .......... .......... .......... ..........
E2348/69      .......... .......... .......... .......... .......... .......... ..........
ECOR_51       .......... .......... .......... .......... .......... .......... ..........
ECOR_57       .......... .......... .......... .......... .......... .......... ..........
UTI89         .......... .......... .......... .......... .......... .......... ..........
E._albertii   .-.....T.C .....T..A. ...A....AT C.T.....AT ........AG G.....T.GA ...T......
```

```
        21421
K-12          TTGTTCCATG TTAACCACTT TTTCAGGGGT CCTTTTTTTA GATTATCCTG ATTATAAACG AATAATCCAT
53638         .......... .......... .......... .......... .......... .......... ..........
ATCC_8739     .......... .......... .......... .......... .......... .......... ..........
ECOR_10       .......... .......... .......... .......... .......... .......... ..........
ECOR_11       .......... .......... .......... .......... .......... .......... ..........
B171          .......... .......... .......... .......... .......... .......... ..........
E24377A       .......... .......... .......... .......... .......... .......... ..........
ECOR_34       .......... .......... .......... .......... .......... .......... ..........
ECOR_71       .......... .......... .......... .......... .......... .......... ..........
SE11          .......... .......... .......... .......... .......... .......... ..........
O157_Sakai    .......... .......... .......... .......... .......... .......... ..........
ECOR_35       .......... .......... .......... .......... .......... .......... ..........
ECOR_36       .......... .......... .......... .......... .......... .......... ..........
SMS-3-5       .......... .......... .......... ......A... .......... .......... ..........
536           .......... .......... .......... .......... .......... .......... ..G.......
APEC_O1       .......... .......... .........G .......... .......... .......... ..G.......
CFT073        .......... .......... .......... .......... .......... .......... ..........
E2348/69      .......... .......... .......... .......... .......... .......... ..G.......
ECOR_51       .......... .......... .......... .......... .......... .......... ..........
ECOR_57       .......... .......... .......... .......... .......... .......... ..........
UTI89         .......... .......... .......... .......... .......... .......... ..G.......
E._albertii   ..T....T.. ...C..C... ........CG T.C.....C. .........T .........C ....T.ATG.

        21491
K-12          TTACGGAATT TTTGTCTGCA AAATACTACT GTATTCAGGA GTAAATGGAC GGCGATGTAT CATCGCCGTC
53638         .......... .......... .......... .......... .......... .......... ....A.....
ATCC_8739     .......... .......... .......... .......... .......... .......... ..........
ECOR_10       .......... .......... .......... .......... .......... .......... ..........
ECOR_11       .......... .......... .......... .......... .......... .......... ..........
B171          .......... .......... .......... .......... .......... .......... ..........
E24377A       .......... .......... .......... .......... .......... .......... ..........
ECOR_34       .......... .......... .......... .......... .......... .......... ..........
ECOR_71       .......... .......... .......... .......... .......... .......... ..........
SE11          .......... .......... .......... .......... .......... .......... ..........
O157_Sakai    .......... .......... .......... ......T... .......... .......... .....A....
ECOR_35       .......... .......... .......... ......T... ......T... .......... ..........
ECOR_36       .......... .......... .......... ......T... ......T... .......... ..........
SMS-3-5       .......... ........T. .......... .......... .......... ..A....... ..........
536           .......... .......... .......... .......... .......... .......... ..........
APEC_O1       .......... .......... .......... .......... .......... .......... ..........
CFT073        .......... .......... .......... .......... .......... .......... ..........
E2348/69      .......... .......... .......... .......... .......... .......... ..........
ECOR_51       .......... .......... .......... .......... .......... .......... ..........
ECOR_57       .......... .......... .......... .......... .......... .......... ..........
UTI89         .......... .......... .......... .......... .......... .......... ..........
E._albertii   ..T...-T.. ...A...AT. ...ATT.G.C T.......A. .A......G. T......... ....A.G.C.

        21561
K-12          CATTTTTCGA CATACTAGAT TCGTCTCGCC TGCCAGAAAT TTTTCTGCCA ATAGACATTA TCAAGTGAGG
53638         .......... .......... .......... .......... .......... .......... ..........
ATCC_8739     .......... .......... .......... .......... .......... .......... ..........
ECOR_10       .......... .......... .......... .......... .......... .......... ..........
ECOR_11       .......... .......... .......... .......... .......... .......... ..........
B171          .......... .......A.. .......... .......... .......... ....C..... ..........
E24377A       .......... .......A.. .......... .......... .......... .......... ..........
ECOR_34       .......... .......... .......... .......... .......... .......... ..........
ECOR_71       .......... .......... .......... .......... .......... .......... ..........
SE11          .......... .......... .......... .......... .......... .......... ..........
O157_Sakai    ..A....... .......... .......... .......... .......... .......... ..........
ECOR_35       A......... .......... .......... .......... .......... ...A...... ..G.......
ECOR_36       A......... .......... .......... .......... .......... ...A...... ..G.......
SMS-3-5       .......... .......... .......... .......... .......... ...A...... ..G.......
536           .......... .......... .......... .......... .......... .......... ..........
APEC_O1       .......... .......... .......... .......... .......... .......... ..........
CFT073        .......... .......... .......... .......... .......... .......... ..........
E2348/69      .......... .......... .......... .......... .......... .......... ..........
ECOR_51       .......... .......... .......... .......... .......... .......... ..........
ECOR_57       .......... .......... .......... .......... .......... .......... ..........
UTI89         .......... .......... .......... .......... .......... .......... ..........
E._albertii   .....CCAA. GGA....T.. ......T..T ........G. .......... G..A..G... ..G..A....
```

```
         21631
K-12         AACGCATCAC TCCCTTGCTG GTAGAGGCGT GGATAAATTG GTTGTTGGTA TCGTAAATAC CTACATGCAA
53638        .......... .......... .......... .......... .......... .......... ..........
ATCC_8739    .......... .......... .......... .......... .......... .......... ..........
ECOR_10      .......... .......... .......... .......... .......... .......... ..........
ECOR_11      .......... .......... .......... .......... .......... .......... ..........
B171         .......... .......... .......... .......... .......... .......... ..........
E24377A      .......... .......... .......... .......... .......... .......... ..........
ECOR_34      .......... .......... .......... .......... .......... .......... ..........
ECOR_71      .......... .......... .......... .......... .......... .......... ..........
SE11         .......... .......... .......... .......... .......... .......... ..........
O157_Sakai   .......... .......... .......... .......... .......... .......... ..........
ECOR_35      .......... .......... .......... .......... .......... .......... ..........
ECOR_36      .......... .......... .......... .......... .......... .......... ..........
SMS-3-5      .......... .......... .......... .......... .......... .......... ..........
536          .......... .......... .......... .......... .......... .......... ..........
APEC_O1      .......... .......... .......... .......... .......... .......... ..........
CFT073       .......... .......... .......... .......... .......... .......... ..........
E2348/69     .......... .......... .......... .......... .......... .......... ..........
ECOR_51      .......... .......... .......... .......... .......... .......... ..........
ECOR_57      .......... .......... .......... .......... .......... .......... ..........
UTI89        .......... .......... .......... .......... .......... .......... ..........
E._albertii  .G........ C..T...... ..T..T..A. .......... ......A..G ........C. .C........

         21701
K-12         ACCATTTTGT CCGGACCCCG TTTTGAAAAA GACCAGGTCA CCTGGCAGCA ACTCGTCTTT ATCAATTTGC
53638        .......... .......... .......... .......... ..AA...... .......... ..........
ATCC_8739    .......... .......... .......... .......... .......... .......... ..........
ECOR_10      .......... .......... .......... .......... .......... .......... ..........
ECOR_11      .......... .......... .......... .......... .......... .......... ..........
B171         .......... .....A.... .......... .......... ..A....... .......... ..........
E24377A      .......... .....A.... .......... .......... ..A....... .......... ..........
ECOR_34      .......... .......... .......... .......... ..A....... .......... ..........
ECOR_71      .......... .......... .......... .......... .......... .......... ..........
SE11         .......... .......... .......... .......... ..A....... .......... ..........
O157_Sakai   .......... .....A.... .......... .......... ..A....... .......... ..........
ECOR_35      .......... .....G.... .......... .......... ..A....... .......... ..........
ECOR_36      .......... .....G.... .......... .......... ..A....... .......... ..........
SMS-3-5      .......... .....G.... .......... .......... ..A....... .......... ..........
536          .......... .....A.... .......... .......... .......... .......... ..........
APEC_O1      .......... .....A.... .......... .......... .......... .......... ..........
CFT073       .......... .....A.... .......... .......... .......... .......... ..........
E2348/69     .......... .....A.... .......... .......... .......... .......... ..........
ECOR_51      .......... .....A.... .......... .......... .......... .......... ..........
ECOR_57      .......... .....A.... .......... .......... .......... .......... ..........
UTI89        .......... .....A.... .......... .......... .......... .......... ..........
E._albertii  C........A ..A..A..G. .C........ .........G ..G..A.... GA........ ........CA

         21771
K-12         GTGCCAATAG AGGCTTGTTG TTTGGTTTCA CGGGGCAGCT GCAAATCGAA ACGATCGCGC ATCGTCACAA
53638        .....G.... .........C .........T .......... .......... .......... .....T....
ATCC_8739    .......... .......... .......... .......... .......... .......... ..........
ECOR_10      .......... .......... .......... .......... .......... .......... ..........
ECOR_11      .......... .......... .......... .......... .......... .......... ..........
B171         .....G.... .........C .........T .......... .......... .......... .....T....
E24377A      .....G.... .........C .........T .......... .......... .......... .....T....
ECOR_34      .....G.... .........C .........T .......... .......... .......... .....T....
ECOR_71      .......... .......... .......... .......... .......... .......... ..........
SE11         .....G.... .........C .........T .......... .......... .......... .....T....
O157_Sakai   .....G.... .........C .........T .......... .......... .......... ..........
ECOR_35      .....G.... .........C .........T .......... .......... .......... ..........
ECOR_36      .....G.... .........C .........T .......... .......... .......... ..........
SMS-3-5      .....G.... .........C .........T .......... .......... .......... ..........
536          .......... C......... .......... .......... .......... ......A... ..........
APEC_O1      .......... C......... .......... .......... .......... ......A... ..........
CFT073       .......... .......... C......... .......... .......... .......... ..........
E2348/69     .......... C......... .......... .......... .......... ......A... ..........
ECOR_51      .......... .......... C......... .......... .......... .......... ..........
ECOR_57      .......... .......... C......... .......... .......... .......... ..........
UTI89        .......... C......... .......... .......... .......... ......A... ..........
E._albertii  .......... .T..C..... ...T.....T ..C..G.... .T.....A.. ......T... ..........
```

```
         21841
K-12         CCACAAATCC CGAACAGTCC ACACCGCGCC GCGTCATGCC ACCATAACGA TACGGCGTGC CATGCCAGCT
53638        .......... .......... .......... .......... .......... .......... ..........
ATCC_8739    .......... .......... .......... .......... .......... .......... ..........
ECOR_10      .......... .......... .......... .......... .......... .......... ..........
ECOR_11      .......... .......... .......... .......... .......... .......... ..........
B171         .......... .......... .......... .......... .......... .......... ..........
E24377A      .......... .......... .......... .......... .......... .......... ..........
ECOR_34      .......... .......... .......... .......... .......... .......... ..........
ECOR_71      .......... .......... .......... .......... .......... .......... ..........
SE11         .......... .......... .......... .......... .......... .......... ..........
O157_Sakai   .......... .......... .......... .......... .......... .......... ..........
ECOR_35      .......C.. .......... .......... .......... .......... .......... ..........
ECOR_36      .......C.. .......... .......... .......... .......... .......... ..........
SMS-3-5      .......C.. .......... .......... .......... .......... .......... ..........
536          .......... .......... .......... .......... .......... .......... ..........
APEC_O1      .......... .......... .......... .......... .......... .......... ..........
CFT073       .......... .......... .......... .......... .......... .......... ..........
E2348/69     .......... .......... .......... .......... .......... .......... ..........
ECOR_51      .......... .......... .......... .......... .......... .......... ..........
ECOR_57      .......... .......... .......... .......... .......... .......... ..........
UTI89        .......... .......... .......... .......... .......... .......... ..........
E._albertii  .A.....C.. .......... ..C....TA. .AC....T.. G......... ..A..T..A. ..........

         21911
K-12         TTGTAGCTGG TCGTTCAAAC CGGCAATAAC GGTAATCGAA TCAGAAAGTC TGGCATTTGG CGGCGGTGCT
53638        .......... .......... .......... .......... .......... .......... ..........
ATCC_8739    .......... .......... .......... .......... .......... .......... ..........
ECOR_10      .......... .......... .......... .......... .......... .......... ..........
ECOR_11      .......... .......... .......... .......... .......... .......... ..........
B171         .......... .......... .......... .......... .......... .......... ..........
E24377A      .......... .......... .......... .......... .......... .......... ..........
ECOR_34      .......... .......... .......... .......... .......... .......... ..........
ECOR_71      .......... .......... .......... .......... .......... .......... ..........
SE11         .......... .......... .......... .......... .......... .......... ..........
O157_Sakai   .......... .......... .......... .......... .......... .......... ..........
ECOR_35      C......... .......... .......... .......... .......... .......... ..........
ECOR_36      C......... .......... .......... .......... .......... .......... ..........
SMS-3-5      C......... .......... .......... .......... .......... .......... ..........
536          .......... .......... .......... ...G...... .......... .......... ..........
APEC_O1      .......... .......... .......... ...G...... .......... .......... ..........
CFT073       .......... .......... .......... .......... .......... .......... ..........
E2348/69     .......... .......... .......... ...G...... .......... .......... ..........
ECOR_51      .......... .......... .......... .......... .......... .......... ..........
ECOR_57      .......... .......... .......... .......... .......... .......... ..........
UTI89        .......... .......... .......... ...G...... .......... .......... ..........
E._albertii  ......T..A .......... .......G.. A.....T..G ........C. ....G..... ...T.....C

         21981
K-12         TTATGGTGGC TACACCCGGC CAGAAGCAGT GCTGTGATCA AAATAAGGCA GAAACGCATT CCGTACGGTT
53638        .......... ........A. .....A.... .......... .......... .......... ..........
ATCC_8739    .......... .......... .......... .......... .......... .......... ..........
ECOR_10      .......... .......... .......... .......... .......... .......... ..........
ECOR_11      .......... .......... .......... .......... .......... .......... ..........
B171         .......... .......... .....A.... .......... .......... .......... ..........
E24377A      .......... .......... .....A.... .......... .......... .......... ..........
ECOR_34      .......... .......... .....A.... .......... .......... .......... ..........
ECOR_71      .......... .......... .......... .......... .......... .......... ..........
SE11         .......... .......... .....A.... .......... .......... .......... ..........
O157_Sakai   .......... .......... .....A.... .......... .......... .......... ..........
ECOR_35      .......... .......... .....A.... .......... .......... .......... ..........
ECOR_36      .......... .......... .....A.... .......... .......... .......... ..........
SMS-3-5      .......... .......... .....A.... .......... .......... .......... ..........
536          .......... .......... .....A.... .......... .......... .......... ..........
APEC_O1      .......... .......... .....A.... .......... .......... .......... ..........
CFT073       .......... .......... .....A.... .......... .......... .......... ..........
E2348/69     .......... .......... .....A.... .......... .......... .......... ..........
ECOR_51      .......... .......... .....A.... .......... .......... .......... ..........
ECOR_57      .......... .......... .....A.... .......... .......... .......... ..........
UTI89        .......... .......... .....A.... .......... .......... .......... ..........
E._albertii  .....A.... .......T.. .....A.... ........T. .......... .......... .......A..
```

```
        22051
K-12         CCTCTGTTT- ----TTTATT CTTGCATTAA TTTAGCGTCG TAATTACCCG ATTTTCAAGA TACTAATGAA
53638        .......... .......... .......... .......... .......... .......... ..........
ATCC_8739    .......... .......... .......... .......... .......... .......... ..........
ECOR_10      .......... .......... .......... .......... .......... .......... ..........
ECOR_11      .......... .......... .......... .......... .......... .......... ..........
B171         .......... .......... .......... .......... .......... .......... ..........
E24377A      .......... .......... .......... .......... .......... .......... ..........
ECOR_34      .......... .......... .......... .......... .......... .......... ..........
ECOR_71      .......... .......... .......... .......... .......... .......... ..........
SE11         .......... .......... ...A...... .......... .......... .......... ..........
O157_Sakai   .......... .......... .......... .......... .......... .......... ..........
ECOR_35      .......... .......... .......... .......... .......... .......... ..........
ECOR_36      .......... .......... .......... .......... .......... .......... ..........
SMS-3-5      .......... .......... .......... .......... .......... .......... ..........
536          .......... .......... .......... .......... .......... .......... ..........
APEC_O1      .......... .......... .......... .......... .......... .......... ..........
CFT073       .......... .......... .......... .......... .......... .......... ..........
E2348/69     .......... .......... .......... .......... .......... .......... ..........
ECOR_51      .......... .......... .......... .......... .......... .......... ..........
ECOR_57      .......... .......... .......... .......... .......... .......... ..........
UTI89        .......... .......... .......... .......... .......... .......... ..........
E._albertii  .........G TTTT...... ......C... .......... A.......T. .......... .G...T...T

        22121
K-12         ATCAGATGGT CGAAATCAGC ATTCTGTGAC CTTCGATATC CAGACGGCGA AAATTCATCC CATAGGCCTG
53638        .......... .......... .......... .......... .......... .......... ..........
ATCC_8739    .......... .......... .......... .......... .......... .......... ..........
ECOR_10      .......... .......... .......... .......... .......... .......... ..........
ECOR_11      .......... .......... .......... .......... .......... .......... ..........
B171         .......... .......... .......... .......... .......... .......... ..........
E24377A      .......... .......... .......... .......... .......... .......... ..........
ECOR_34      .......... .......... .......... .......... .......... .......... ..........
ECOR_71      .......... .......... .......... .......... .......... .......... ..........
SE11         .......... .......... .......... .......... .......... .......... ..........
O157_Sakai   .......... .......... .......... .......... .......... .......... ..........
ECOR_35      .......... .......... .......... .......... .......... .......... ..........
ECOR_36      .......... .......... .......... .......... .......... .......... ..........
SMS-3-5      .......... .......... .......... .......... .......... .......... ..........
536          .......... .......... .......... .......... .......... .......... ..........
APEC_O1      .......... .......... .......... .......... .......... .......... ..........
CFT073       .......... .......... .......... .......... .......... .......... ..........
E2348/69     .......... .......... .......... .......... .......... .......... ..........
ECOR_51      .......... .......... .......... .......... .......... .......... ..........
ECOR_57      .......... .......... .......... .......... .......... .......... ..........
UTI89        .......... .......... .......... .......... .......... .......... ..........
E._albertii  G....G.C.. ..G....... .....A.... ....A..G.. ......A..G ..G....... ....A...A.

        22191
K-12         CGCCAGATTT GGCGGCGTGA GCACCTCTTC CCTGCGTCCA CTGGCCAGCA TTTTTCCACC TTTTAGCAAC
53638        .......... .......... .......... .......... .......... .......... ..........
ATCC_8739    .......... .......... .......... .......... .......... .......... ..........
ECOR_10      .......... .......... .......... .......... .......... .......... ..........
ECOR_11      .......... .......... .......... .......... .......... .......... ..........
B171         .......... .......... .......... T......... .......... .......... ..........
E24377A      .......... .......... .......... T......... .......... .......... ..........
ECOR_34      .......... .......... .......... T......... .......... .......... ..........
ECOR_71      .......... .......... .......... .......... .......... .......... ..........
SE11         .......... .......... .......... T......... .......... .......... ..........
O157_Sakai   .......... .......... ....A..... T......... .......... .......... ..........
ECOR_35      .......... .......... .......... .......... .......... .......... ..........
ECOR_36      .......... .......... .......... .......... .......... .......... ..........
SMS-3-5      .......... .......... .......... .......... .......... .......... ..........
536          .......... .C........ .......... T......... .......... .......... ..........
APEC_O1      .......... .C........ .......... T......... .......... .......... ..........
CFT073       .......... .C........ .......... T......... .......... .......... ..........
E2348/69     .......... .C........ .......... T......... .......... .......... ..........
ECOR_51      .......... .C........ .......... T......... .......... .......... ..........
ECOR_57      .......... .C........ .......... T......... .......... .......... ..........
UTI89        .......... .C........ .......... T......... .......... .......... ..........
E._albertii  G........C .AT.....C. .T.....A.. ..GA..C..G ...A.T.... G......TTG ...A..T.G.
```

```
         22261
K-12         CACGCCCGAT GCGCATGACG CAATGTGTGG TTGAGATCGT GACTGCTCAT CACAATCGCC AGTCCTTGCT
53638        .......... .......... .......... .......... .......... .......... ..........
ATCC_8739    .......... .......... .......... .......... .......... .......... ..........
ECOR_10      .......... .......... .......... .......... .......... .......... ..........
ECOR_11      .......... .......... .......... .......... .......... .......... ..........
B171         .......... .......... .......... .......... .......... .......... ..........
E24377A      .......... .......... .......... .......... .......... .......... ..........
ECOR_34      .......... .......... .......... .......... .......... .......... ..........
ECOR_71      .......... .......... .......... .......... .......... .......... ..........
SE11         .......... .......... .......... .......... .......... .......... ..........
O157_Sakai   .......... .......... .......... .......... .......... .......... ..........
ECOR_35      .......... .......... ......A... .......... .......... .......... ..........
ECOR_36      .......... .......... ......A... .......... .......... .......... ..........
SMS-3-5      .......... .......... ......A... .......... .......... .......... ..........
536          .......... .......... .......... .......... .......... .......... ..........
APEC_O1      .......... .......... .......... .......... .......... .......... ..........
CFT073       .......... .......... .......... .......... .......... .......... ..........
E2348/69     .......... .......... .......... .......... .......... .......... ..........
ECOR_51      .......... .......... .......... .......... .......... .......... ..........
ECOR_57      .......... .......... .......... .......... .......... .......... ..........
UTI89        .......... .......... .......... .......... .......... .......... ..........
E._albertii  ..G.....G. .......G.. ..GC..A..A ..C..G.... .G........ ......A..G ..........

         22331
K-12         GACACAGCGC GCTCAGAATT TTGTCTAACG CACTTTGTTG CGCAACATCA AGACTGTTCA TCGGCTCATC
53638        .......... .......... .......... .......... .......... .......... ..........
ATCC_8739    .......... .......... .......... .......... .......... .......... ..........
ECOR_10      .......... .......... .......... .......... .......... .......... ..........
ECOR_11      .......... .......... .......... .......... .......... .......... ..........
B171         .......A.. .......... .......... .......... .......... .......... ..........
E24377A      .......A.. .......... .......... .......... .......... .......... ..........
ECOR_34      .......A.. .......... .......... .......... .......... .......... ..........
ECOR_71      .......... .......... .......... .......... .......... .......... ..........
SE11         .......A.. .......... .......... .......... .......... .......... ..........
O157_Sakai   .......... .......... .......... .......... .......... .......... ..........
ECOR_35      .......... .......... ........T. .......... .......... .......... .A........
ECOR_36      .......... .......... ........T. .......... .......... .......... .A........
SMS-3-5      .......... .......... .......... .......... .......... .......... .A........
536          .......... .......... ........G. .......... .......... .......... ..........
APEC_O1      .......... .......... ........G. .......... .......... .......... ..........
CFT073       .......... .......... ........G. .G........ .......... .......... ..........
E2348/69     .......... .......... ........G. .G........ .......... .......... ..........
ECOR_51      .......... .......... ........G. .G........ .......... .......... ..........
ECOR_57      .......... .......... ........G. .G........ .......... .......... ..........
UTI89        .......... .......... ........G. .......... .......... .......... ..........
E._albertii  ....A..... .....AT..C ..A.....T. .GT....... G......... ..G..A.... ..........

         22401
K-12         AAGAAGCAGC AATTGGCCTG CGGGATTGGC TTGTGGTGTG ATTTGCAACA CCACCGCAGC AAGACGTACG
53638        .......... .......... .......... .......... .......G.. .......... ..........
ATCC_8739    .......... .......... .......... .......... .......... .......... ..........
ECOR_10      .......... .......... .......... .......... .......... .......... ..........
ECOR_11      .......... .......... .......... .......... .......... .......... ..........
B171         .......... .....A.... .......... .......... .......G.. .......... ..........
E24377A      .......... .....A.... .......... .......... .......G.. .......... ..........
ECOR_34      .......... .....A.... .......... .......... .......G.. .......... ..........
ECOR_71      .......... .......... .......... .......... .......... .......... ..........
SE11         .......... .....A.... .......... .......... .......G.. .......... ..........
O157_Sakai   .......... .......... .......... .......... .......G.. .......... ..........
ECOR_35      ......T..T .......... .......... .......... .......G.. .......... ..........
ECOR_36      ......T..T .......... .......... .......... .......G.. .......... ..........
SMS-3-5      ......T..T .......... .......... .......... .......G.. .......... ..........
536          .......... .......... .......... .......... .......G.. .......... ..........
APEC_O1      .......... .......... .......... .......... .......G.. .......... ..........
CFT073       .......... .......... .......... .......... .......G.. .......... ..........
E2348/69     .......... .......... .......... .......... .......G.. .......... ..........
ECOR_51      .......... .......... .......... .......... .......G.. .......... ..........
ECOR_57      .......... .......... .......... .......... .......G.. .......... ..........
UTI89        .......... .......... .......... .......... .......G.. .......... ..........
E._albertii  C..C.A.... .....T..C. A......T.. ...C..CA.. .....T.... .......C.. ...G.....C
```

```
        22471
K-12          CGTTGCCATT CACCGCCGGA AAGTTGATTG GTGCTACGTC CGAGTTTGTC ATCAAGAGCC AGCGCCCCTG
53638         .......... .......... .......... .......... .......... .......... ..........
ATCC_8739     .......... .......... .......... .......... .......... .......... ..........
ECOR_10       .......... .......... .......... .......... .......... .......... ..........
ECOR_11       .......... .......... .......... .......... .......... .......... ..........
B171          .......... .......... .......... .......... .......... .......... ..........
E24377A       .......... .......... .......... ........A. .......... .......... ..........
ECOR_34       .......... .......... .......... .......... .......... .......... ..........
ECOR_71       .......... .......... .......... .......... .......... .......... ..........
SE11          .......... .......... .......... .......... .......... .......... ..........
O157_Sakai    .......... .......... .......... ........A. .......... .......... ..........
ECOR_35       .......... .G........ .......... .......... .......... .......... ..........
ECOR_36       .......... .G........ .......... .......... .......... .......... ..........
SMS-3-5       .......... .G........ .......... .......... .......... .......... ..........
536           .......... .......... .......... .......... .......... .......... ..........
APEC_O1       .......... .......... .......... .......... .......... .......... ..........
CFT073        .......... .......... .......... .......... .......... .......... ..........
E2348/69      .......... .......... .......... .......... .......... .......... ..........
ECOR_51       .......... .......... .......... .......... .......... .......... ..........
ECOR_57       .......... .......... .......... .......... .......... .......... ..........
UTI89         .......... .......... .......... .......... .......... .......... ..........
E._albertii   ........C. .G..A..... G........A ..A....... .C..C..... GC.G..G... .A....T.C.

        22541
K-12          CGACATCATT CAGTAGTTCG GTACGCGTTT TATCGTGCTG ATGCAGTGTC AGGTAGTGCC AGACCGGCGT
53638         .......... .......... .......... .......... .......... .......... ........A.
ATCC_8739     .......... .......... .......... .......... .......... .......... ........A.
ECOR_10       .......... .......... .......... .......... .......... .......... ........A.
ECOR_11       .......... .......... .......... .......... .......... .......... ........A.
B171          .......... .......... .......... .......... .......... .......... ........A.
E24377A       .......... .......... .......... .......... .......... .......... ........A.
ECOR_34       .......... .......... .......... .......... .......... .......... ........A.
ECOR_71       .......... .......... .......... .......... .......... .......... ........A.
SE11          .......... .......... .......... .......... .......... .......... ........A.
O157_Sakai    .......... .......... .......... .......... .......... .......... ........A.
ECOR_35       .......... .......... .......... .......... .......... .......... ........A.
ECOR_36       .......... .......... .......... .......... .......... .......... ........A.
SMS-3-5       .......... .......... .......... .......... .......... .......... ........A.
536           .......... .......... .......... .......... .......... .......... ........A.
APEC_O1       .......... .......... .......... .......... .......... .......... ........A.
CFT073        .......... .......... .......... .......... .......... .......... ........A.
E2348/69      .......... .......... .......... .......... .......... .......... ........A.
ECOR_51       .......... .......... .......... .......... .......... .......... ........A.
ECOR_57       .......... .......... .......... .......... .......... .......... ........A.
UTI89         .......... .......... .......... .......... .......... .......... ........A.
E._albertii   .T........ T.AC.AA... .......C.. ....A..... .....A..A. ..A....... .T......A.

        22611
K-12          TGCAAACGGC GGCGTCTGCT GTTGTGAAAG ATAGGCGCGA TGCAGCGCGA GTTTTGTTGC GGACCATGCT
53638         .......... .......... .......... G......... .......... .......... ..........
ATCC_8739     .......... .......... .......... .......... .......... .......... ..........
ECOR_10       .......... .......... .......... .......... .......... .......... ..........
ECOR_11       .......... .......... .......... .......... .......... .......... ..........
B171          .......... .......... .......... G......... .......... .......... ..........
E24377A       .......... .......... .......... .......... .......... .......... ..........
ECOR_34       .......... .......... .......... .......... .......... .......... ..........
ECOR_71       .......... .......... .......... .......... .......... .......... ..........
SE11          .......... .......... .......... .......... .......... .......... ..........
O157_Sakai    .......... .......... .......... ......A... .......... .......... ..........
ECOR_35       .......... .......... .......... .......... .......... .......... ..........
ECOR_36       .......... .......... .......... .......... .......... .......... ..........
SMS-3-5       .......... .......... .......... .......... .......... .......... ..........
536           .......... .......... .......... .......... .......... .C........ A.........
APEC_O1       .......... .......... .......... .......... .......... .C........ A.........
CFT073        .......... .......... .......... .......... .......... .C........ A.........
E2348/69      .......... .......... .......... .......... .......... .C........ A.........
ECOR_51       .......... .......... .......... .......... .......... .C........ A.........
ECOR_57       .......... .......... .......... .......... .......... .C........ A.........
UTI89         .......... .......... .......... .......... .......... .C........ A.........
E._albertii   A........A ..AA.T.... .......... ...A...... ........A. ....C..... ..G...G.TA
```

```
         22681
K-12          TCCAGTGGTT GCCCCGCGAA CTGAATGCTT CCCTTACCGC TGGTCATTCC GGCCATTCGC GCCAGTAAGG
53638         .......... .......... .......... .......... .......... .........T ..A..C....
ATCC_8739     .......... .......... .......... .......... .......... .......... ..........
ECOR_10       .......... ......A... .......... .......... .......... .......... ..........
ECOR_11       .......... .......... .......... .......... .......... .......... ..........
B171          .......... .......... .......... .......... .......... .........T ..A..C....
E24377A       .....G.... .......... .......... .......... .......... .........T ..A..C....
ECOR_34       .....G.... .......... .......... .......... .......... .........T ..A..C....
ECOR_71       .......... .......... .......... .......... .......... .......... ..........
SE11          .....G.... .......... .......... .......... .......... .........T ..A..C....
O157_Sakai    .......... .......... .......... .......... .......... .......... ..........
ECOR_35       .......... .......... .......... .......... .......... .........T ..A..C....
ECOR_36       .......... .......... .......... .......... .......... .........T ..A..C....
SMS-3-5       .......... .......... .......... .......... .......... .........T ..A..C....
536           .......... .......... .......... .......... .......... .......... ..........
APEC_O1       .......... .......... .......... .......... .......... .......... ..........
CFT073        .......... .......... .......... .......... .......... .......... ..........
E2348/69      .......... .......... .......... .......... .......... .......... ..........
ECOR_51       .......... .......... .......... .......... .......... .......... ..........
ECOR_57       .......... .......... .......... .......... .......... .......... ..........
UTI89         .......... .......... .......... .......... .......... .......... ..........
E._albertii   .....A..C. .A..A..A.. T.....A.GC ........A. .......... .......... ........C.

         22751
K-12          TACTCTTACC CGCGCCATTC GGCCCCACCA GGTGCAGGAT CTCCCCAGCC CGAACCTCGC CAGAAAGCGG
53638         .......... .......... .......... .......... .......... .......... ......A...
ATCC_8739     .......... .......... .......... .......... .......... .......... ..........
ECOR_10       .......... .......... .......... .......... .......... .......... ..........
ECOR_11       .......... .......... .......... .......... .......... .......... ..........
B171          .......... .......... .......... .......... .......... .......... ..........
E24377A       .......... .......... .......... .......... .......... .......... .......T..
ECOR_34       .......... .......... .......... .A........ .......... .......... .......T..
ECOR_71       .......... .......... .......... .......... .......... .......... ..........
SE11          .......... .......... .......... .......... .......... .......... .......T..
O157_Sakai    .......... .......... .......... .......... .......... .......... ..........
ECOR_35       .......... ......G... .......... .......... .......... .......... ..........
ECOR_36       .......... ......G... .......... .......... .......... .......... ..........
SMS-3-5       .......... ......G... .......... .......... .......... .......... ..........
536           .......... .......... .......... .......... .......... .......... ..........
APEC_O1       .......... .......... .......... .......... .......... .......... ..........
CFT073        .......... .......... .......... .......... .......... .......... ..........
E2348/69      .......... .......... .......... .......... .......... .......... ..........
ECOR_51       .......... .......... .......... .......... .......... .......... ..........
ECOR_57       .......... .......... .......... .......... .......... .......... ..........
UTI89         .......... .......... .......... .......... .......... .......... ..........
E._albertii   .G..T..G.. ......G... ........A. .A..T..... ......T... .......... ..........

         22821
K-12          CCCCAGGCGG GTAGATTCCG CAACATCTTG TAACTGCATC ACAATAGACA TTATTTTGCC AACGCCAGTT
53638         .......... .......... .......C.. .......... .......... .......... ..........
ATCC_8739     .......... .......... .......... .......... .......... .......... ..........
ECOR_10       .......... .......... .......... .......... .......... .......... ..........
ECOR_11       .......... .......... .......... .......... .......... .......... ..........
B171          .......... ....T..... .......C.. .......... .......... .......... ..........
E24377A       .......... .......... .......C.. .......... .......... .......... ..........
ECOR_34       .......... .......... .......C.. .......... .......... .......... ..........
ECOR_71       .......... .......... .......... .......... .......... .......... ..........
SE11          .......... .......... .......C.. .......... .......... .......... ..........
O157_Sakai    .......... .......... .......C.. .......... .......... .......... ..........
ECOR_35       .......... .......... .......C.. .......... .......... .......... ..........
ECOR_36       .......... .......... .......C.. .......... .......... .......... ..........
SMS-3-5       .......... .......... .......C.. .......... .......... .......... ..........
536           .......... .......... .......C.. .......... .......... .......... ..........
APEC_O1       .......... .......... .......C.. .......... .......... .......... ..........
CFT073        .......... ..G....... .......C.. .......... .......... .......... ..........
E2348/69      .......... .......... .......C.. .......... .......... .......... ..........
ECOR_51       .......... ..G....... .......C.. .......... .......... .......... ..........
ECOR_57       .......... ..G....... .......C.. .......... .......... .......... ..........
UTI89         .......... .......... .......C.. .......... .......... .......... ..........
E._albertii   ......A... ..T....... .C.....C.. .......... .......... ......C... ..........
```

```
        22891
K-12          TAATGCTTTC CATCACAATG GGATCTTCCG GCGTCATATC CGGGGAAAAA CGCTGGATGA CTTTTCCGTC
53638         .......... .......... .......... .......... .......... .......... .CAA......
ATCC_8739     .......... .......... .......... .......... .......... .......... ..........
ECOR_10       .......... .......... .......... .......... .......... .......... ..........
ECOR_11       .......... .......... .......... .......... .......... .......... ..........
B171          .......... .......... ........A. .......... .......... .......... .CAAC.....
E24377A       .......... .......... ..G....... .......... .......... .......... .CAA......
ECOR_34       .......... .......... ..G....... .......... .......... .......... .CAA......
ECOR_71       .......... .......... .......... .......... .......... .......... ..........
SE11          .......... .......... ..G....... .......... .......... .......... .CAA......
O157_Sakai    .......... .......... .......... .......... .......... .......... .CAA......
ECOR_35       .......... .......... .......... .......... .......... .......... .CAA......
ECOR_36       .......... .......... .......... .......... .......... .......... .CAA......
SMS-3-5       .......... .......... .......... .......... .......... .......... .CAA......
536           .......... .......... .......... .......... .......... .......... .CAA......
APEC_O1       .......... .......... .......... .......... .......... .......... .CAA......
CFT073        .......... .......... .......... .......... .......... .......... .C........
E2348/69      .......... .......... .......... .......... .......... .......... .C........
ECOR_51       .......... .......... .......... .......... .......... .......... .C........
ECOR_57       .......... .......... .......... .......... .......... .......... .C........
UTI89         .......... .......... .......... .......... .......... .......... .CAA......
E._albertii   .......... T..T..G... ........T. .T........ .......... .....A.... .C..C.....

        22961
K-12          CCTGCCAACC AGGAATTTTT CAAAATTCCA TAAAATATCA TCCGGGTACA GCGGTGCACG GCCTTTGCTG
53638         .........T .AA.....C. .......... .......... ..A....... .......... A.........
ATCC_8739     .A........ .......... .......... .......... .......... .......... ..........
ECOR_10       .......... .......... .......... .......... .......... .......... ..........
ECOR_11       .......... .......... .......... .......... .......... .......... ..........
B171          .........T .AA.....C. .......... .......... ..A....... .......... A.........
E24377A       .........T .AA.....C. .......... .......... ..A....... .......... A.........
ECOR_34       .........T .AA.....C. .......... .......... ..A....... .......... A.........
ECOR_71       .......... .......... .......... .......... .......... .......... ..........
SE11          .........T .AA.....C. .......... .......... ..A....... .......... A.........
O157_Sakai    .........T .AA.....C. .......... .......... ..A....... .......... A.........
ECOR_35       .........T .AA.....C. .......... .......... ..A....... .......... A.........
ECOR_36       .........T .AA.....C. .......... .......... ..A....... .......... A.........
SMS-3-5       .........T .AA.....C. .......... .......... ..A....... .......... A.........
536           .........T .AA.....C. .......... .......... ..A....... .......... A.........
APEC_O1       .........T .AA.....C. .......... .......... ..A....... .......... A.........
CFT073        .........T .AA.....C. .......... .......... ..A....... .......... A.........
E2348/69      .........T .AA.....C. .......... .......... ..A....... .......... A.........
ECOR_51       .........T .AA.....C. .......... .......... ..A....... .......... A.........
ECOR_57       .........T .AA.....C. .......... .......... ..A....... .......... A.........
UTI89         .........T .AA.....C. .......... .......... ..A....... .......... A.........
E._albertii   T..C..C... ..A....... .......... C......... C.A..A.... A...C..... .........A

        23031
K-12          ACCATACGGG CATAGAATCC GCTCTCTTCC GGCGCGACTG CGGTCGGCGC TGCGGCAATC AATTTTTGAT
53638         .......... .......... .......... .......... .......... .......... ..........
ATCC_8739     ......A... .......... .......... .......... .......... .......... ..........
ECOR_10       .......... .......... .......... .......... .......... .......... ..........
ECOR_11       .......... .......... .......... .......... .......... .......... ..........
B171          .......... .......... .......... .......... .......... .......... ..........
E24377A       .......... .......... .......... .......... .......... .......... ..........
ECOR_34       .......... .......... .......... .......... .......... .......... ..........
ECOR_71       .......... .......... .......... .......... .......... .......... ..........
SE11          .......... .......... .......... .......... .......... .......... ..........
O157_Sakai    .......... .......... .......... .......... .......... .....A.... ..........
ECOR_35       .......... .......... .......... .......... .......... .......... ..........
ECOR_36       .......... .......... .......... .......... .......... .......... ..........
SMS-3-5       .......... .......... .......... .......... .......... .......... ..........
536           .....T.... .......... .......... .......... .A........ .......... ..........
APEC_O1       .....T.... .......... .......... .......... .A........ .......... ..........
CFT073        .......... .......... .......... .......... .A........ .......... ..........
E2348/69      .....T.... .......... .......... .......... .A........ .......... ..........
ECOR_51       .......... .......... .......... .......... .A........ .......... ..........
ECOR_57       .......... .......... .......... .......... .A........ .......... ..........
UTI89         .....T.... .......... .......... .......... .A........ .......... ..........
E._albertii   .....T.TT. .......C.. ........T. .....A..G. .C........ A......... .G......G.
```

```
        23101
K-12         ACAGCGGATG GCGTCCTTCG CCATTAACTT CAATCTTACT GAACATCGGG AACGTCACCC CCCATGTGGT
53638        .......... .......... .......... .......... .......... .......... ..........
ATCC_8739    .......... .......... .......... .......... .......... .......... ..........
ECOR_10      .......... .......... .......... .......... .......... .......... ..........
ECOR_11      .......... .......... .......... .......... .......... .......... ..........
B171         .......... .......... .......... .......... .......... .......... ..........
E24377A      .......... .......... .......... .......... .......... .......... ..........
ECOR_34      .......... .......... .......... .......... .......... .......... ..........
ECOR_71      .......... .......... .......... .......... .......... .......... ..........
SE11         .......... .......... .......... .......... .......... .......... ..........
O157_Sakai   .......... .......... .......... .......... .......... .......... ..........
ECOR_35      .......... .......... .......... .......... .......... .......... ..........
ECOR_36      .......... .......... .......... .......... .......... .......... ..........
SMS-3-5      .......... .......... .......... .......... .......... .......... ..........
536          .......... A......... .......... .......... .......... .......... ..........
APEC_O1      .......... A......... .......... .......... .......... .......... ..........
CFT073       .......... .......... .......... .......... .......... .......... ..........
E2348/69     .......... A......... .......... .......... .......... .......... ..........
ECOR_51      .......... .......... .......... .......... .......... .......... ..........
ECOR_57      .......... .......... .......... .......... .......... .......... ..........
UTI89        .......... A......... .......... .......... .......... .......... ..........
E._albertii  ...A...... C..C.T.... .....G.... .......... .......... ..T.....T. ....G..T..

        23171
K-12         GGTACAGTAA GTTTTAATCT CTTCATCGCT GCCCGGTTCT TGTTCCAGAA ACTGGTTGCA CGGGAATCCC
53638        .......... .......... .......... .......... .......... .......... ..........
ATCC_8739    .......... .......... .......... .......... .......... .......... ..........
ECOR_10      .......... .......... .......... .......... .......... .......... ..........
ECOR_11      .......... .......... .......... .......... .......... .......... ..........
B171         .......... .......... .......... .......... .......... .......... ..........
E24377A      .......... .......... .......... .......... .......... .......... ..........
ECOR_34      .......... .......... .......... .......... .......... .......... ..........
ECOR_71      .......... .......... .......... .......... .......... .......... ..........
SE11         .......... .......... .......... .......... .......... .......... ..........
O157_Sakai   .......... .......... .......... .......... .......... .......... ..........
ECOR_35      .......... .......... .......... .......... .......... .......... ..........
ECOR_36      .......... .......... .......... .......... .......... .......... ..........
SMS-3-5      .......... .......... .......... .......... .......... .......... ..........
536          ...G..A... .......... .......... A......... ..C....... .......A.. ..........
APEC_O1      ...G..A... .......... .......... A......... ..C....... .......A.. ..........
CFT073       ...G...... .......... .......... A......... ..C....... .......A.. ..........
E2348/69     ...G..A... .......... .......... A......... ..C....... .......A.. ..........
ECOR_51      ...G...... .......... .......... A......... ..C....... .......A.. ..........
ECOR_57      ...G...... .......... .......... A......... ..C....... .......A.. ..........
UTI89        ...G..A... .......... .......... A......... ..C....... .......A.. ..........
E._albertii  ...G..A... .....T.... .......... ...A...... .....A.... .......... T..A......

        23241
K-12         AGCACCATAA AACCTCGATC GACCCAGGCT TTCTGAATAT TCTCCAACTG CTCATATTGC GGCGTTAAGC
53638        .......C.. .......... .G........ .......... .......... .......... ..........
ATCC_8739    .......C.. .......... .G........ .......... .......... .......... ..........
ECOR_10      .......... .......... .......... .......... .......... .......... ..........
ECOR_11      .......... .......... .......... .......... .......... .......... ..........
B171         .......C.. .......... .G........ .......... .......... .......... ..........
E24377A      .......C.. .......... .G........ .......... .......... .......... ..........
ECOR_34      .......C.. .......... .G........ .......... .......... .......... ..........
ECOR_71      .......C.. .......... .G........ .......... .......... .......... ..........
SE11         .......C.. .......... .G........ .......... .......... .......... ..........
O157_Sakai   .......C.. .......... .G........ .......... .......... .......... ..........
ECOR_35      .......C.. ....A..... .G........ .......... .......... .......... ..........
ECOR_36      .......C.. ....A..... .G........ .......... .......... .......... ..........
SMS-3-5      .......C.. ....A..... AG........ .......... .......... .......... ..........
536          .......C.. ....A..... AG........ .......... .......... .......... ..........
APEC_O1      .......C.. ....A..... AG........ .......... .......... .......... ..........
CFT073       .......C.. ....A..... AG........ .......... .......... .......... ..........
E2348/69     .......C.. ....A..... AG....A... .......... .......... .......... ..........
ECOR_51      .......C.. ....A..... AG........ .......... .......... .......... ..........
ECOR_57      .......C.. ....A..... AG........ .......... .......... .......... ..........
UTI89        .......C.. ....A..... AG........ .......... .......... .......... ..........
E._albertii  .A...G.C.. .C..G..... ACT.....T. .....G.... ....T..... T......... .....C.GA.
```

```
          23311
K-12         CACACTTTGA GGCGACATTG ACAATCAACA GCACATTACC GGCGAACTTC TCCAGCGTGG TCACTTCACC
53638        .......... .......... .......... .......... .......... .......... ..........
ATCC_8739    .......... .......... .......... .......... ....T..... .......... ..........
ECOR_10      .......... .......... .......... .......... .......... .......... ..........
ECOR_11      .......... .......... .......... .......... .......... .......... ..........
B171         .......... .......... .......... .......... A...T..... .......... ..........
E24377A      .......... .......... .......... .......... ....T..... .......... ..........
ECOR_34      .......... .......... .......... .......... ....T..... .......... ..........
ECOR_71      .......... .......... .......... .......... ....T..... .......... ..........
SE11         .......... .......... .......... .......... ....T..... .......... ..........
O157_Sakai   .......... .......... .......... .......... ....T..... .......... ..........
ECOR_35      .......... .......... .......... .......... .......... .......... ..........
ECOR_36      .......... .......... .......... .......... .......... .......... ..........
SMS-3-5      .......... .......... .......... .......... ....T..... .......... ..........
536          .......... .......... .......... .......... ....T..... .......... ......T...
APEC_O1      .......... .......... .......... .......... ....T..... .......... ......T...
CFT073       .......... .......... .......... .......... ....T..... .......... ..........
E2348/69     .......... .......... .......... .......... ....T..... ..T....... ..........
ECOR_51      .......... .......... .......... .......... ....T..... .......... ..........
ECOR_57      .......... .......... .......... .......... ....T..... .......... ..........
UTI89        .......... .......... .......... .......... ....T..... .......... ......T...
E._albertii  .......C.. A..A...... ..G....G.. A......... A..AT.T... .......... ..G....G..

          23381
K-12         GTCGATATCT TTCACTACGG TCGTCAGAAT GGAATCTTGC AT
53638        .......... .......... .......... .......... ..
ATCC_8739    .......... .......... .......... .......... ..
ECOR_10      .......... .......... .......... .......... ..
ECOR_11      .......... .......... .......... .......... ..
B171         .......... .......... .......... .......... ..
E24377A      .......... .......... .......... .......... ..
ECOR_34      .......... .......... .......... .......... ..
ECOR_71      .......... .......... .......... .......... ..
SE11         .......... .......... .......... .......... ..
O157_Sakai   .......... .......... .......... .......... ..
ECOR_35      .......... .......... .......... .......... ..
ECOR_36      .......... .......... .......... .......... ..
SMS-3-5      .......... .......... .......... .......... ..
536          ...A...... .......... .......... .......... ..
APEC_O1      ...A...... .......... .......... .......... ..
CFT073       .......... .......... .......... .......... ..
E2348/69     .......... .......... .......... .......... ..
ECOR_51      .......... .......... .......... .......... ..
ECOR_57      .......... .......... .......... .......... ..
UTI89        ...A...... .......... .......... .......... ..
E._albertii  ...A..TGTC .......... .T........ T......... ..
```

Table S2. Sequences alignment. Segment 3

```
               1
K-12           AACTCCCGTG CAACCGACGC GCGTCGATAA CATCCGGCAC CTGGTTGAGT TTACCCAGCA CGCGCCCCAG
53638          .......... .......... .......... .......... .......... .......... ..........
ATCC_8739      .......... .......... .......... .......... .......... .......... ..........
HS             .......... .......... .......... .......... .......... .......... ..........
BW2952         .......... .......... .......... .......... .......... .......... ..........
12009          .......... .......... .......... .......... .......... .......... ..........
B171           .......... .......... .......... .......... .......... .......... ..........
E24377A        .......... .......... .......... .......... .......... .......... ..........
SE11           .......... .......... .......... .......... .......... .......... ..........
11128          .......... .......... .......... .......... .......... .......... ..........
11368          .......... .......... .......... .......... .......... .......... ..........
IAI1           .......... .......... .......... .......... .......... .......... ..........
E110019        .......... .......... .......... .G........ .......... .......... ....G.....
B7A            .......... .......... .......... .......... .......... .......... ..........
CB9615         .......... .......... .......... .......... .......... .......... ..........
O157_Sakai     .......... .......... .......... .......... .......... .......... ..........
IAI39          .......... .........T ..A....... .......... .......... .......... .........A
UMN026         .......... .......... .......... .......... .......... .......... ....G.....
SMS-3-5        .......... .......... .......... .......... .......... .......... ....G.....
536            .......... .......... .......... .......T.. .......... .......... ....A.....
APEC_O1        .......... .......... .......... .......T.. .......... .......... ..........
CFT073         .......... .......... .......... .......T.. .......... .......... ....G.....
E2348/69       .......... .......... .......... .......... .......... .......... ....A.....
UTI89          .......... .......... .......... .......T.. .......... .......... ..........
F11            .......... .......... .......... .......T.. .......... .......... ....A.....
ED1a           .......... .......... .......... .......... .......... .......... ....A.....
83972          .......... .......... .......... .......T.. .......... .......... ....G.....
S88            .......... .......... .......... .......T.. .......... .......... ..........
E._albertii    --........ .......... .......... ....T..... .......... .......... ....G.....

              71
K-12           CACTTGCAGG TTGTAAATCT CAATGGTCAT GTCGATGGTC GCCAGTTGCT GTTTGGTGTC GCTACGGCTG
53638          .......... .......... .......... .......... .......... .......... ..........
ATCC_8739      .......... .......... .......... .......... .......... .......... ..........
HS             .......... .......... .......... .......... .......... .......... ..........
BW2952         .......... .......... .......... .......... .......... .......... ..........
12009          .......... .......... .......... .......... .......... .......... ..........
B171           .......... .......... .......... .......... .......... .......... ..........
E24377A        .......... .......... .......... .......... .......... .......... ..........
SE11           .......... .......... .......... .......... .......... .......... ..........
11128          .......... .......... .......... .......... .......... .......... ..........
11368          .......... .......... .......... .......... .......... .......... ..........
IAI1           .......... .......... .......... .......... .......... .......... ..........
E110019        ...C...... .......... .......... .........T .......... .......... ..........
B7A            .......... .......... ....A..... .......... .......... .......... ..........
CB9615         .......... .......... .......... .......... .......... .......... ..........
O157_Sakai     .......... .......... .......... .......... .......... .......... ..........
IAI39          ...C...... ..A....... ....A..... .........T .......... .......... ..........
UMN026         .......... .......... .......... .......... .......... .......... ..........
SMS-3-5        .......... .......... .......... .......... .......... .......... ..........
536            .......... .......... .......... .......... .......... .......... ..........
APEC_O1        .......... .......... .......... .......... .......... .......... ..........
CFT073         .......... .......... .......... .......... .......... .......... ..........
E2348/69       .......... .......... .......... .......... .......... .......... ..........
UTI89          .......... .......... .......... .......... .......... .......... ..........
F11            .......... .......... .......... .......... .......... .......... ..........
ED1a           .......... .......... .......... .......... .......... .......... ..........
83972          .......... .......... .......... .......... .......... .......... ..........
S88            .......... .......... .......... .......... .......... .......... ..........
E._albertii    .........A .......... ....A..... .......... .......... .C..A..... ..........
```

```
           141
K-12         GCAACGCCAA GCACGTTCAC CTTCTCGTTG GCGAGAATGG TCGTGATATC ACGTAACAAC CCACTACGAT
53638        .......... .......... .......... .......... .......... .......... ..........
ATCC_8739    .......... .......... .......... .......... .......... .......... ..........
HS           .......... .......... .......... .......... .......... .......... ..........
BW2952       .......... .......... .......... .......... .......... .......... ..........
12009        .......... .......... .......... .......... .......... .......... ..........
B171         .......... .......... .......... .......... .......... .......... ..........
E24377A      .......... .......... .......... .......... .......... .......... ..........
SE11         .......... .......... .......... .......... .......... .......... ..........
11128        .......... .......... .......... .......... .......... .......... ..........
11368        .......... .......... .......... .......... .......... .......... ..........
IAI1         .......... .......... .......... .......... .......... .......... ..........
E110019      .......... .......... .......... .......... .......... .......... ..........
B7A          .......... .......... .......... .......... .......... .......... ..........
CB9615       .......... .......... .......... .......... .......... .......... ..........
O157_Sakai   .......... .......... .......... .......... .......... .......... ..........
IAI39        .......... .T........ .......... .......... .......... .......... ..........
UMN026       .......... .T........ .......... .......... .......... .......... ..........
SMS-3-5      .......... .T........ .......... .......... .......... .......... ..........
536          .......... .T........ .......... .......... .......... .......... ..........
APEC_O1      .......... .T........ .......... .......... .......... .......... ..........
CFT073       .......... .......... .......... .......... .......... .......... ..........
E2348/69     .......... .T........ .......... .......... .......... .......... ..........
UTI89        .......... .T........ .......... .......... .......... .......... ..........
F11          .......... .T........ .......... .......... .......... .......... ..........
ED1a         .......... .T........ .......... .......... .......... .......... ..........
83972        .......... .......... .......... .......... .......... .......... ..........
S88          .......... .T........ .......... .......... .......... .......... ..........
E._albertii  .......... .......... .........A .....G..A. .G........ ...C..T... .....G....

           211
K-12         CATTAGCTAC CACGCGGACC ACCAGCGAAT ATCCGGCGGA GTAGCTCTCA CCCCATACCG CGTCAACAAT
53638        .......... .......... .......... .......... .......... .......... ..........
ATCC_8739    .......C.. .......... .......... .......... .......... .......... ..........
HS           .......C.. .......... .......... .......... .......... .......... ..........
BW2952       .......... .......... .......... .......... .......... .......... ..........
12009        .......C.. ...A...... .......... .......... .......... .......... ..........
B171         .......C.. ...A...... .......... .......... .......... .......... ..........
E24377A      .......C.. .......... .......... .......... .......... .......... ..........
SE11         .......C.. .......... .......... .......... .......... .......... ..........
11128        .......C.. .......... .......... .......... .......... .......... ..........
11368        .......C.. .......... .......... .......... .......... .......... ..........
IAI1         .......C.. .......... .......... .......... .......... .......... ..........
E110019      .......C.. .......... .......... .......... .......... .......... ..........
B7A          .......C.. .......... .......... .......... .......... .......... ..........
CB9615       .......C.. .......... .......... .......... .......... .......... ..........
O157_Sakai   .......C.. .......... .......... .......... .......... .......... ..........
IAI39        .......C.. .......... .......... .......... .........G .......... ..........
UMN026       .......C.. .......... .......... .......... .........G .......... .A........
SMS-3-5      .......C.. .......... .......... .......... .........G .......... ..........
536          .......C.. .......... .......... .......... .........G .......... ..........
APEC_O1      .......C.. .......... .......... .......... .........G .......... ..........
CFT073       .......C.. .......... .......... .......... .......... .......... ..........
E2348/69     .......C.. .......... .......... .......... .......... .......... ..........
UTI89        .......C.. .......... .......... .......... .........G .......... ..........
F11          .......C.. .......... .......... .......... .........G .......... ..........
ED1a         .......C.. .......... .......... .......... .........G .......... ..........
83972        .......C.. .......... .......... .......... .......... .......... ..........
S88          .......C.. .......... .......... .......... .........G .......... ..........
E._albertii  ....G..C.T T..A...... .......... .C....T... .........T .......... ..........
```

```
             281
K-12          GCGTTCTGGC GCATGGGAGC GCAGTTCCGC CAGTTGTTCG CAATCGGCGC GGTGTACTGA AATACCGCGC
53638         .......... .......... .......... .......... .......... .......... ..........
ATCC_8739     .......... .......... .......... .......... .......... .......... ..........
HS            .......... .......... .......... .......... .......... .......... ..........
BW2952        .......... .......... .......... .......... .......... .......... ..........
12009         .......... .......... .......... .......... .......... .......... ..........
B171          .......... .......... .......... .......... .......... .......... ..........
E24377A       .......... .......... .......... .......... .......... .......... ..........
SE11          .......... .......... .......... .......... .......... .......... ..........
11128         .......... .......... .......... .......... .......... .......... ..........
11368         .......... .......... .......... .......... .......... .......... ..........
IAI1          .......... .......... .......... .......... .......... .......... ..........
E110019       .......... .......... .......... .......... .......... .......... ..........
B7A           .......... .......... .......... .......... .......... .......... ..........
CB9615        .......... .......... .......... .......... .......... .......... .........T
O157_Sakai    .......... .......... .......... .......... .......... .......... .........T
IAI39         .......... .......... .......... .......... .......... .......... .........T
UMN026        A......... .......... .......... .......... .......... .......... .........T
SMS-3-5       .......... .......... .......... .......... .......... .......... .........T
536           .......... .......... .......... ..A....... .......... .......... ..........
APEC_O1       .......... .......... .......... ..A....... .......... .......... ..........
CFT073        .......... .......... .......... .......... .......... .......... .........T
E2348/69      .......... .......... .......... ..A....... .......... .......... ..........
UTI89         .......... .......... .......... ..A....... .......... .......... ..........
F11           .......... .......... .......... ..A....... .......... .......... ..........
ED1a          .......... .......... .......... ..A....... .......... .......... ..........
83972         .......... .......... .......... .......... .......... .......... .........T
S88           .......... .......... .......... ..A....... .......... .......... ..........
E._albertii   A.....C... ..G..T..A. .......T.. ......C..A ..G..T..A. .A..C..... .........T

             351
K-12          CCCTGGGTAA TGAAGCCGAC AATCTCATCT CCAGGAATCG GCTGGCAGCA GCGCGCGATG TGGTGCATCA
53638         .......... .......... .......... .......... .......... .......... ..........
ATCC_8739     .......... .......... .......... .......... .......... .......... ..........
HS            .......... .......... .......... .......... .......... .......... ..........
BW2952        .......... .......... .......... .......... .......... .......... ..........
12009         .......... .......... .......... .......... .......... .......... ..........
B171          .......... .......... .......... .......... .......... .......... ..........
E24377A       .......... .......... .......... .......... .......... .......... ..........
SE11          .......... .......... .......... .......... .......... .......... ..........
11128         .......... .......... .......... .......... .......... .......... ..........
11368         .......... .......... .......... .......... .......... .......... ..........
IAI1          .......... .......... .......... .......... .......... .......... ..........
E110019       .......... .......... .......... .......... .......... .......... ..........
B7A           .......... .......... .......... .......... .......... .......... ..........
CB9615        .......... .......... .......... .......... .......... .......... ..........
O157_Sakai    .......... .......... .......... .......... .......... .......... ..........
IAI39         .......... .......... .......... .......... .......... .......... ..........
UMN026        .......... .......... .......... .......... .......... .......... ..........
SMS-3-5       .......... .......... .......... .......... .......... .......... ..........
536           ..T....... .......... .......... .......... .......... .......... ..........
APEC_O1       .......... .......... .......... .......... .......... .......... ..........
CFT073        .....A.... ....A..A.. T..T.....A ........A. ....A..... .......... ..........
E2348/69      .......... .......... .......... .......... .......... .......... ..........
UTI89         .......... .......... .......... .......... .......... .......... ..........
F11           ..T....... .......... .......... .......... .......... .......... ..........
ED1a          ..T....... .......... .......... .......... .......... .......... ..........
83972         .....A.... ....A..A.. T..T.....A ........A. ....A..... .......... ..........
S88           .......... .......... .......... .......... .......... .......... ..........
E._albertii   .......... ....A..A.. .........A ..T....... .......... A........A ..........
```

```
             421
K-12          GGTTGCCAAC ACCTTCGACT ACCACGCGAC CGTTATCTTT ACTGCGGTTT TGCGGCGTGT AGCTTTTTTG
53638         .......... .......... .......... .......... .......... .......... ..........
ATCC_8739     .......... .......... .......... .......... .......... .......... ..........
HS            .......... .......... .......... .......... .......... .......... ..........
BW2952        .......... .......... .......... .......... .......... .......... ..........
12009         .......... .........C .......... .......... .......... .......... ..........
B171          .......... .........C .......... .......... .......... .......... ..........
E24377A       ....A..G.. ......A..C .......... .......... .......... .......... ..........
SE11          .......... .........C .......... .......... .......... .......... ..........
11128         .......... .........C .......... .......... .......... .......... ..........
11368         .......... .........C .......... .......... .......... .......... ..........
IAI1          .......... .........C .......... .......... .......... .......... ..........
E110019       .......... .......... .......... .......... .......... .......... ..........
B7A           .......... .......... .......... .......... .......... .......... ..........
CB9615        ....A..... ......A..C ..T..A.... .......... .......... .......... ..........
O157_Sakai    ....A..... ......A..C ..T..A.... .......... .......... .......... ..........
IAI39         ....A..G.. ......T..C .....A.... .......... .......... .......... ..........
UMN026        ....A..G.. ......A..C .....A.... .......... .......... ........A. ..........
SMS-3-5       ....A..G.. ......T..C .....A.... .......... .......... .......... ..........
536           ....A..G.. C.....T..C .....A.... .......... .......... .......... ..........
APEC_O1       ....A..G.. C.....T..C .....A.... .......... .......... .......... ..........
CFT073        ....A..G.. ......A..C .....A.... .......... .......... .......... ..........
E2348/69      ....A..... ......T..C .....A.... .......... .......... ........A. ..........
UTI89         ....A..G.. C.....T..C .....A.... .......... .......... .......... ..........
F11           ....A..G.. C.....T..C .....A.... .......... .......... .......... ..........
ED1a          ....A..G.. C.....T..C .....A.... .......... .......... .......... ..........
83972         ....A..G.. ......A..C .....A.... .......... .......... .......... ..........
S88           ....A..G.. C.....T..C .....A.... .......... .......... .......... ..........
E._albertii   .......G.. G.....T... .......... .......... ......A... ........A. ..T.......

             491
K-12          CTGAAGTTGC TTCAGCGCGG CGGCGTCCTG CTCTTCGGCA CTCGGCTTAT TAAATTGCGA TTGCAGGAAG
53638         .......... .......... .......... .......... .......... .......... ..........
ATCC_8739     .......... .......... .......... .......... .......... .......... ..........
HS            .......... .......... .......... .......... .......... .......... ..........
BW2952        .......... .......... .......... .......... .......... .......... ..........
12009         .......... .......... .......... .......... .......... .......... ..........
B171          .......... .......... .......... .......... .......... .......... ..........
E24377A       .......... .......... .......... .......... .......... .......... ..........
SE11          .......... .......... .......... .......... .......... .......... ..........
11128         .......... .......... .......... .......... .......... .......... ..........
11368         .......... .......... .......... .......... .......... .......... ..........
IAI1          .......... .......... .......... .......... .......... .......... ..........
E110019       .......... .......... .......... .......... .......... .......... ..........
B7A           .......... .......... .......... .......... .......... .......... ..........
CB9615        ......C..T .......... .......... .......... .......... .......... ..........
O157_Sakai    ......C..T .......... .......... .......... .......... .......... ..........
IAI39         ......C..T .......... ....A..... .......... .......... .......... ..........
UMN026        .......... .......... ....A..... .......... .......... .......... ..........
SMS-3-5       ......C..T .......... ....A..... .......... .......... .......... ..........
536           ......C..T .......... .......... .......... .......... .......... ..........
APEC_O1       ......C..T .......... .......... .......... .......... .......... ..........
CFT073        .......... .......... .......... .......... .......... .......... ..........
E2348/69      .......... .......... ....A..... .......... .......... .......... ..........
UTI89         ......C..T .......... .......... .......... .......... .......... ..........
F11           ......C..T .......... .......... .......... .......... .......... ..........
ED1a          ......C..T .......... .......... .......... .......... .......... ..........
83972         .......... .......... .......... .......... .......... .......... ..........
S88           ......C..T .......... .......... .......... .......... .......... ..........
E._albertii   ...C.AC..T .......... .A..A..... T........G .......... .......... ..........
```

```
             561
K-12          TTCACCATCT GATTGAGACG GATATCCCCG CCACCAATCG CCGCCAGCAA CTCGTCGACA TCATTGAAGT
53638         .......... .......... .......... .......... .......... .......... ..........
ATCC_8739     .......... .......... .......... .......... .......... .......... ..........
HS            .......... .......... .......... .......... .......... .......... ..........
BW2952        .......... .......... .......... .......... .......... .......... ..........
12009         .......... .......... .......... .......... .......... .......... .....A....
B171          .......... .......... .......... .......... .......... .......... .....A....
E24377A       .......... .......... .......... .......... .......... .......... .....A....
SE11          .......... .......... .......... .......... .......... .......... .....A....
11128         .......... .......... .......... .......... .......... .......... .....A....
11368         .......... .......... .......... .......... .......... .......... .....A....
IAI1          .......... .......... .......... .......... .......... .......... .....A....
E110019       .......... .......... .......... .......... .......... .......... .....A....
B7A           .......... .......... .......... .......... .......... .......... .....A....
CB9615        .......... .......... .......... .......... .......... .......... ..........
O157_Sakai    .......... .......... .......... .......... .......... .......... ..........
IAI39         .......... .......... .......... .......... .......... .......... ..........
UMN026        .......... .......... .......... .......... .......... .......... ..........
SMS-3-5       .......... .......... .......... .......... .......... .......... ..........
536           .......... .......... .......... .......... .......... .......... ..........
APEC_O1       .......... .......... .......... .......... .......... .......... ..........
CFT073        .......... .......... .......... .......... .......... .......... ..........
E2348/69      .......... .......... .......... ........T. .......... .......... ..........
UTI89         .......... .......... .......... .......... .......... .......... ..........
F11           .......... .......... .......... .......... .......... .......... ..........
ED1a          .......... .......... .......... .......... .......... .......... ..........
83972         .......... .......... .......... .......... .......... .......... ..........
S88           .......... .......... .......... .......... .......... .......... ..........
E._albertii   .......... .......... ......T... ..G....... .......... .......... ..........

             631
K-12          TGTAACGCGG CAGCAGATGT TTTTCTGCTT CTTTCAGGCT GATCCCCAGA TGTTCCAGCT CGTCGTCAAG
53638         .......... .......... .......... .......... .......... .......... ..........
ATCC_8739     .......... .......... .......... .......... .......... .......... ..........
HS            .......... .......... .......... .......... .......... .......... ..........
BW2952        .......... .......... .......... .......... .......... .......... ..........
12009         .......... .......... .......... .......... .......... .......... ..........
B171          .......... .......... .......... .......... .......... .......... ..........
E24377A       .......... .......... .......... .......... .......... .......... ..........
SE11          .......... .......... .......... .......... .......... .......... ..........
11128         .......... .......... .......... .......... .......... .......... ..........
11368         .......... .......... .......... .......... .......... .......... ..........
IAI1          .......... .......... .......... .......... .......... .......... ..........
E110019       .......... .......... .......... .......... .......... .......... ..........
B7A           .......... .......... .......... .......... .......... .......... ..........
CB9615        .......... .......... .......... .......... .......... .......... ..........
O157_Sakai    .......... .......... .......... .......... .......... .......... ..........
IAI39         .......... ......G... .......... .......... .......... .......... ....A.....
UMN026        .......... .......... .......... .......... .......... .......... ..........
SMS-3-5       .......... ......G... .......... .......... .......... .......... ....A.....
536           .......... .......... .......... .......... .......... .......... ..........
APEC_O1       .......... .......... .......... .......... .......... .......... ..........
CFT073        .......... .......... .......... .......... .......... .......... ..........
E2348/69      .......... .......... .......... .......... .......... .......... ..........
UTI89         .......... .......... .......... .......... .......... .......... ..........
F11           .......... .......... .......... .......... .......... .......... ..........
ED1a          .......... .......... .......... .......... .......... .......... ..........
83972         .......... .......... .......... .......... .......... .......... ..........
S88           .......... .......... .......... .......... .......... .......... ..........
E._albertii   ....G..A.. .......... .....C.... .......... .......... ........T. .......C..
```

```
           701
K-12          GATTTGCCGC CCAGCCAGAA TGTTTTTGTC ACGGTCCTGT TTACGGAACC AGGCGTGAAT TTTCGAACGC
53638         .......... .......... .......... .......... .......... .......... ..........
ATCC_8739     .......... .......... .......... .......... .......... .......... ..........
HS            .......... .......... .......... .......... .......... .......... ..........
BW2952        .......... .......... .......... .......... .......... .......... ..........
12009         .......... .......... .......... .......... .......... .......... ..........
B171          .......... .......... .......... .......... .......... .......... ..........
E24377A       .......... .......... .......... .......... .......... .......... ..........
SE11          .......... .......... .......... .......... .......... .......... ..........
11128         .......... .......... .......... .......... .......... .......... ..........
11368         .......... .......... .......... .......... .......... .......... ..........
IAI1          .......... .......... .......... .......... .......... .......... ..........
E110019       .......... .......... .......... .......... .......... .......... ..........
B7A           .......... .......... .......... .......... .......... .......... ..........
CB9615        .......... .......... .......... .......... .......... .......... ..........
O157_Sakai    .......... .......... .......... .......... .......... .......... ..........
IAI39         .........T .......... .......... ...A...... .......... .......... ......G...
UMN026        .......... .......... .......... .......... .......... .......... ..........
SMS-3-5       .........T .......... .......... ...A...... .......... .......... ......G...
536           .......... .......... .......... .......... .......... .......... ..........
APEC_O1       A......... .......... .......... .......... .......... .......... ..........
CFT073        .......... .......... .......... .......... .......... .......... ..........
E2348/69      .......... .......... .......... .......... .......... .......... ..........
UTI89         A......... .......... .......... .......... .......... .......... ..........
F11           .......... .......... .......... .......... .......... .......... ..........
ED1a          A......... .......... .......... .......... .......... .......... ..........
83972         .......... .......... .......... .......... .......... .......... ..........
S88           A......... .......... .......... .......... .......... .......... ..........
E._albertii   .......... ..G....... .......... G..A...... .......... .T........ ......G..T

           771
K-12          CCACGGCTGG TTGTGACGTA ACCGAGGTTT GGGTTTAACC AGTCACGGCT GGGGTTCGGC TGTTTCTGGG
53638         .......... .......... .......... .......... .......... .......... ..........
ATCC_8739     .......... .......... .......... .......... .......... .......... ..........
HS            .......... .......... .......... .......... .......... .......... ..........
BW2952        .......... .......... .......... .......... .......... .......... ..........
12009         .......... .......... .......... .......... .......... .......... ..........
B171          .......... .......... .......... .......... .......... .......... ..........
E24377A       .......... .......... .......... .......... .......... .......... ..........
SE11          .......... .......... .......... .......... .......... .......... ..........
11128         .......... .......... .......... .......... .......... .......... ..........
11368         .......... .......... .......... .......... .......... .......... ..........
IAI1          .......... .......... .......... .......... .......... .......... ..........
E110019       .......... .......... .......... .......... .......... .......... ..........
B7A           .......... .......... .......... .......... .......... .......... ..........
CB9615        .......... .......... .......... .......... .......... .......... ..........
O157_Sakai    .......... .......... .......... .......... .......... .......... ..........
IAI39         .....A.... .......... ......A... .......... .......... .......... ..........
UMN026        .......... .......... .......... .......... .......... .......... ..........
SMS-3-5       .....A.... .......... ......A... .......... .......... .......... ..........
536           .......... .......... .......... .......... .......... .......... ..........
APEC_O1       .......... .......... .......... .......... .......... .......... ..........
CFT073        .......... .......... .......... .......... .......... .......... ..........
E2348/69      .......... .......... .......... .......... .......... .......... ..........
UTI89         .......... .......... .......... .......... .......... .......... ..........
F11           .......... .......... .......... .......... .......... .......... ..........
ED1a          .......... .......... .......... .......... .......... .......... ..........
83972         .......... .......... .......... .......... .......... .......... ..........
S88           .......... .......... .......... .......... .......... .......... ..........
E._albertii   .......... ....A..A.. T..C...G.. ..A....... ....G..A.. A.....T... ........A.
```

```
                841
K-12            TGATAATTTC AATCTGGTCG CCCATCTGCA GCTGGTAGGT GAACGGCACA ATGCGCCCGC CAATTTTTGC
53638           .......... .......... .......... .......... .......... .......... ..........
ATCC_8739       .......... .......... .......... .......... .......... .......... ..........
HS              .......... .......... .......... .......... .......... ........A. ..........
BW2952          .......... .......... .......... .......... .......... .......... ..........
12009           .......... .......... .......... .......... .......... .......... ..........
B171            .......... .......... .......... .......... .......... .......... ..........
E24377A         .......... .......... .......... .......... .......... .......... ..........
SE11            .......... .......... .......... .......... .......... .......... ..........
11128           .......... .......... .......... .......... .......... .......... ..........
11368           .......... .......... .......... .......... .......... .......... ..........
IAI1            .......... .......... .......... .......... .......... .......... ..........
E110019         .......... .......... .......... .......... .......... .......... ..........
B7A             .......... .......... .......... .......... .......... .......... ..........
CB9615          .......... .......... .....T.... .......... .......... .......... ..........
O157_Sakai      .......... .......... .....T.... .......... .......... .......... ..........
IAI39           .......... .......... .......... .......... .......... .......... ..........
UMN026          .......... .......... .......... .......... .......... .......... ..........
SMS-3-5         .......... .......... .......... .......... .......... .......... ..........
536             .......... .......... .......... .......... .......... .......... ..........
APEC_O1         .......... .......... .......... .......... .......... .......... ..........
CFT073          .......... .......... .......... .......... .......... .......... ..........
E2348/69        .......... .......... .......... .......... .......... .......... ..........
UTI89           .......... .......... .......... .......... .......... .......... ..........
F11             .......... .......... .......... .......... .......... .......... ..........
ED1a            .......... .......... .......... .......... .......... .......... ..........
83972           .......... .......... .......... .......... .......... .......... ..........
S88             .......... .......... .......... .......... .......... .......... ..........
E._albertii     .......... .........C .......... A...A..... A......... ..A..T.... .......G..

                911
K-12            CCCGATGCAG CGGTGTCCGA CATCACTGTG GATGTGGTAA GCGAAGTCCA GCGGCGTTGA TCCCGCAGGC
53638           .......... .......... .......... .......... .......... .......... ..........
ATCC_8739       ...A...... .......... .......... .......... .......... .......... ..........
HS              .......... .......... .......... .......... .......... .......... ..........
BW2952          .......... .......... .......... .......... .......... .......... ..........
12009           .......... .......... .......... .......... .......... .......... ..........
B171            .......... .......... .......... .......... .......... .......... ..........
E24377A         .......... .......... .......... .......... .......... .......... ..........
SE11            .......... .......... .......... .......... .......... .......... ..........
11128           .......... .......... .......... .......... .......... .......... ..........
11368           .......... .......... .......... .......... .......... .......... ..........
IAI1            .......... .......... .......... .......... .......... .......... ..........
E110019         .......... .......... .......... .......... .......... .......... ..........
B7A             .......... .......... .......... .......... .......... .......... ..........
CB9615          ......A... .......... .......... .......... .......... .......... ..........
O157_Sakai      ......A... .......... .......... .......... .......... .......... ..........
IAI39           .......... .......... .......... .......... .......... ....T..... ..........
UMN026          .......... .....C.... .......... .......... .......... .......... ..........
SMS-3-5         .......... .......... .......... .........C .......... ....T..... ..........
536             .......... .......... .......... .......... .......... .......... ..........
APEC_O1         .......... .......... .......... .......... .......... .......... ..........
CFT073          .......... .......... .......... .......... .......... .......... ..........
E2348/69        .......... .......... .......... .......... .......... ....T..... ..........
UTI89           .......... .......... .......... .......... .......... .......... ..........
F11             .......... .......... .......... .......... .......... .......... ..........
ED1a            .......... .......... .......... .......... .......... .......... ..........
83972           .......... .......... .......... .......... .......... .......... ..........
S88             .......... .......... .......... .......... .......... .......... ..........
E._albertii     ...T..A... ........A. .G..G..... .......... ..A....... .......... ....A.....
```

```
             981
K-12         AAATCAACGA CATCACCTTT CGGCGTAAAG ACGTACACCC GGTCGTCAAA GACCTGACTA CGTACTTCGT
53638        .......... .......... .......... .......... .......... .......... ..........
ATCC_8739    .......... .......... .......... ........T. .......... ......G..G ..........
HS           .......... .......... .......... .......... .......... .......... ..........
BW2952       .......... .......... .......... .......... .......... .......... ..........
12009        .......... .......... .......... ..A....... .......... ......G..G ..........
B171         .......... .......... .......... ..A....... .......... ......G..G ..........
E24377A      .......... .......... .......... ..A....... .......... ......G..G ..........
SE11         .......... .......... .......... ..A....... .......... ......G..G ..........
11128        .......... .......... .......... ..A....... .......... ......G..G ..........
11368        .......... .......... .......... ..A....... .......... ......G..G ..........
IAI1         .......... .......... .......... ..A....... .......... ......G..G ..........
E110019      .......... .......... .......... ..A....... .......... ......G..G ..........
B7A          .......... .......... .......... ..A....... .......... ......G..G ..........
CB9615       .......... .......... A......... .......... .......... ......G..G ..........
O157_Sakai   .......... .......... A......... .......... .......... ......G..G ..........
IAI39        .......... .......... .......... .......... .......... ......G... ..........
UMN026       .......... .G........ .......... .......... .......... ......G..G ..........
SMS-3-5      .......... .......... .......... .......... .......... ......G... ..........
536          .......... .......... .......... ........T. .......... .......... ..........
APEC_O1      .......... .......... .......... ........T. .......... .......... ..........
CFT073       .......... .......... .......... ........T. .......... .......... ..........
E2348/69     .......... .......... .......... .......... .......... ......G... ..........
UTI89        .......... .......... .......... ........T. .......... .......... ..........
F11          .......... .......... .......... ........T. .......... .......... ..........
ED1a         .......... .......... .......... ........T. .......... .......... ..........
83972        .......... .......... .......... ........T. .......... .......... ..........
S88          .......... .......... .......... ........T. .......... .......... ..........
E._albertii  .......... .......... .......... .......... ....A..... .........G ..C.......

            1051
K-12         CGAGCATTTC GCCGGAATCA GCCATCTCTT CCTGCCACGC AATCAGTTTA CGCAGCCAGG CAATCCGGTC
53638        .......... .......... .......... .......... .......... .......... ..........
ATCC_8739    .......... .......... .......... .......T.. .......... .......... ..........
HS           .......... .......... .......... .......... ...T...... .......... ..........
BW2952       .......... .......... .......... .......... .......... .......... ..........
12009        .......... .......... .......... .......... .......... .......... ..........
B171         .......... .......... .......... .......... .......... .......... ..........
E24377A      .......... .......... .......... .......... .......... .......... ..........
SE11         .......... .......... .......... .......... .......... .......... ..........
11128        .......... .......... .......... .......... .......... .......... ..........
11368        .......... .......... .......... .......... .......... .......... ..........
IAI1         .......... .......... .......... .......... .......... .......... ..........
E110019      .......... .......... .......... .......... .......... .......... ..........
B7A          .......... .......... .......... .......... .......... .......... ..........
CB9615       .......... .......... .......... .......... .......... .......... ..........
O157_Sakai   .......... .......... .......... .......... .......... .......... ..........
IAI39        .......... .......... .......... .......... .......... .......... ..........
UMN026       .......... .......... .......... .......... .......... .......... ....T.....
SMS-3-5      .......... .......... .......... .......... .......... .......... ..........
536          .......... .......... .......... .......... .......... .......... ..........
APEC_O1      .......... .......... .......... .......... .......... .......... ..........
CFT073       .......... .......... .......... .......... .......... .......... ..........
E2348/69     .......... .......... .......... .......... .......... .......... ..........
UTI89        .......... .......... .......... .......... .......... .......... ..........
F11          .......... .......... .......... .......... .......... .......... ..........
ED1a         .......... .......... .......... .......... .......... .......... ..........
83972        .......... .......... .......... .......... .......... .......... ..........
S88          .......... .......... .......... .......... .......... .......... ..........
E._albertii  .......... .........C .......... .......... T........T .......... ....A.....
```

```
             1121
K-12         TTCATGTCCC GAACGTGCGC CGCCAGCAGC CGCGCCCTCT TTATATTTCC AGTGCGCAGC AACACCCAAC
53638        .......... .......... .......... .......... .......... .......... ..........
ATCC_8739    .......... .......... .......... .......... .......... .......... ..........
HS           .......... .......... .......... .......... .......... .......... ..........
BW2952       .......... .......... .......... .......... .......... .......... ..........
12009        .......... .......... .......T.. .......... .......... .......... ..........
B171         .......... .......... .......T.. .......... .......... .......... ..........
E24377A      .......... .......... .......T.. .......... .......... .......... ..........
SE11         .......... .......... .......T.. .......... .......... .......... ..........
11128        .......... .......... .......T.. .......... .......... .......... ..........
11368        .......... .......... .......T.. .......... .......... .......... ..........
IAI1         .......... .......... .......T.. .......... .......... .......... ..........
E110019      .......... .......... .......T.. .......... .......... .......... ..........
B7A          .......... .......... .......T.. .......... .......... .......... ..........
CB9615       .......... .......... .......... .......... .......... .......... ..........
O157_Sakai   .......... .......... .......... .......... .......... .......... ..........
IAI39        .......... .......... .......... .......... .......... .......... ........G.
UMN026       .......... .......... .......T.. ......T... .......... .......... ...C......
SMS-3-5      .......... .......... .......T.. .......... .......... .......... ..........
536          .......... .......... .......... .......... .......... .......... ..........
APEC_O1      .......... .......... .......... .......... .......... .......... ..........
CFT073       .......... .......... .......... .......... .......... .......... ..........
E2348/69     .......... .......... .......T.. .......... .......... ....T..... ..........
UTI89        .......... .......... .......... .......... .......... .......... ..........
F11          .......... .......... .......... .......... .......... .......... ..........
ED1a         .......... .......... .......... .......... .......... .......... ..........
83972        .......... .......... .......... .......... .......... .......... ..........
S88          .......... .......... .......... .......... .......... .......... ..........
E._albertii  .......... .....A..C. ......A... ..T...T... ..G....... .......... ...G....GT

             1191
K-12         TCTGCATCTT CATGCATCTG TTTGGTGCGG ATTTGGATCT CAACGGTTTT TCCACCCGGC CCCAGAACCA
53638        .......... .......... .......... .......... .......... .......... ..........
ATCC_8739    .......... .......... .......... .......... .......... .......... ..........
HS           .......... .......... .......... .......... .......... .......... ..........
BW2952       .......... .......... .......... .......... .......... .......... ..........
12009        .......... .......... .......... .......... .......... G.....T... ..........
B171         .......... .......... .......... .......... .......... G.....T... ..........
E24377A      .......... .......... .......... .......... .......... G.....T... ..........
SE11         .......... .......... .......... .......... .......... G.....T... ..........
11128        .......... .......... .......... .......... .......... G.....T... ..........
11368        .......... .......... .......... .......... .......... G.....T... ..........
IAI1         .......... .......... .......... .......... .......... G.....T... ..........
E110019      .......... .......... .......... .......... .......... G.....T... ..........
B7A          .......... .......... .......... .......... .......... G.....T... ..........
CB9615       .......... .......... .......... .......... .......... .......... ..........
O157_Sakai   .......... .......... .......... .......... .......... .......... ..........
IAI39        .......... .......... .......... .......... .......... .......... ..........
UMN026       .......... .......... .......... .......... .......... .......... ..........
SMS-3-5      .......... .......... ...A...... .......... .......... G.....T..T ..........
536          .......... .......... .......... .......... .......... G......... ..........
APEC_O1      .......... .......... .......... .......... .......... G......... ..........
CFT073       .......... .......... .......... .......... .......... G......... ..........
E2348/69     .......... .......... .......... .......... .......... G......... ..........
UTI89        .......... .......... .......... .......... .......... G......... ..........
F11          .......... .......... .......... .......... .......... G......... ..........
ED1a         .......... .......... .......... .......... .......... G......... ..........
83972        .......... .......... .......... .......... .......... G......... ..........
S88          .......... .......... .......... .......... .......... G......... ..........
E._albertii  ..G....... .......... .......... ..C....... .G..C..C.. C..G...... ....AT..A.
```

```
          1261
K-12         CGGTATGAAT AGACTGATAA CCGTTTGGTT TCGGGTTAGC GACGTAATCG TCAAACTCAT CCGGCAGGTG
53638        .......... .......... .......... .......... .......... .......... ..........
ATCC_8739    .......... .......... .......... .......... .......... .......... ..........
HS           .......... .......... .......... .......... .......... .......... ..........
BW2952       .......... .......... .......... .......... .......... .......... ..........
12009        .......... .......... .......... .......... ...A...... .......... ..........
B171         .......... .......... .......... .......... ...A...... .......... ..........
E24377A      .......... .......... .......... .......... ...A...... .......... ..........
SE11         .......... .......... .......... .......... ...A...... .......... ..........
11128        .......... .......... .......... .......... ...A...... .......... ..........
11368        .......... .......... .......... .......... ...A...... .......... ..........
IAI1         .......... .......... .......... .......... ...A...... .......... ..........
E110019      .......... .......... .......... .......... ...A...... .......... ..........
B7A          .......... .......... .......... .......... ...A...... .......... ..........
CB9615       .......... .......... .......... .......... .......... .......... ..........
O157_Sakai   .......... .......... .......... .......... .......... .......... ..........
IAI39        .......... .......... .......... .......... A......... .......... ..........
UMN026       .......... .......... .......... .......... .......... .......... ..........
SMS-3-5      .......... .......... .......... .......... .......... .......... ..........
536          .......... G......... .......... .......... .......... .......... ..........
APEC_O1      .......... G......... .......... .......... .......... .......... ..........
CFT073       .......... G......... .......... .......... .......... .......... ..........
E2348/69     .......... .......... .......... .......... .......... .......... ..........
UTI89        .......... G......... .......... .......... .......... .......... ..........
F11          .......... G......... .......... .......... .......... .......... ..........
ED1a         .......... G......... .......... .......... .......... .......... ..........
83972        .......... G......... .......... .......... .......... .......... ..........
S88          .......... G......... .......... .......... .......... .......... ..........
E._albertii  .......... C......... .....A.... .T.....T.. ...A...... .......... .A.....A..

          1331
K-12         GCGATAGTGA GTGTGCACTA TCCCCAGTGC GGCATAGCAA TCCTGTAAAC GCTCGGCGAC AATACGTACC
53638        .......... .......... .......... .......... .......... .......... ..........
ATCC_8739    .......... .......... .......... ......A... .......... .......... ..........
HS           .......... .......... .......... ......A... .......... .......... ..........
BW2952       .......... .......... .......... .......... .......... .......... ..........
12009        .......... .......... .......... ......A... .......... .......... ..........
B171         .......... .......... .......... ......A... .......... .......... ..........
E24377A      .......... .......... .......... ......A... .......... .......... ..........
SE11         .......... .......... .......... ......A... .......... .......... ..........
11128        .......... .......... .......... ......A... .......... .......... ..........
11368        .......... .......... .......... ......A... .......... .......... ..........
IAI1         .......... .......... .......... ......A... .......... .......... ..........
E110019      .......... .......... .......... .......... .......... .......... ..........
B7A          .......... .......... .......... ......A... .......... .......... ..........
CB9615       .......... .......... .......... ......A... .......... .......... ..........
O157_Sakai   .......... .......... .......... ......A... .......... .......... ..........
IAI39        .......... .......... .......... ......A... .......... .......... ..........
UMN026       .......... .......... .......... ......A... .......... .......... ..........
SMS-3-5      .......... .......... .......... ......A... .......... .......... ..........
536          .......... .......... .......... ......A... .......... .......... ..........
APEC_O1      .......... .......... .......... ......A... .......... .......... ..........
CFT073       .......... .......... .......... ......A... .......... .......... ..........
E2348/69     .......... .......... .......... ......A... .......... .......... ..........
UTI89        .......... .......... .......... ......A... .......... .......... ..........
F11          .......... .......... .......... ......A... .......... .......... ..........
ED1a         .......... .......... .......... ......A... .......... .......... ..........
83972        .......... .......... .......... ......A... .......... .......... ..........
S88          .......... .......... .......... ......A... .......... .......... ..........
E._albertii  A......... .......... ......A... A..G...... .......G.. .T..C..... G.........
```

```
           1401
K-12        GCACGCACAT CAAACAGCTC ATCAAAGGCG AGGTTCTTTT TCTGCATTTT ACGCCAGATG CTGTAGATGT
53638       .......... .......... .......... .......... .......... .......... ..........
ATCC_8739   .......... .......... ...G...... .......... .......... G........A ..........
HS          .......... .......... ...G...... .......... .......... .......... ..........
BW2952      .......... .......... .......... .......... .......... .......... ..........
12009       .......... .......... ...G...... .......... .......... G........A ..........
B171        .......... .......... ...G...... .......... .......... G........A ..........
E24377A     .......... .......... .......... .......... .......... .......... ..........
SE11        .......... .......... ...G...... .......... .......... G........A ..........
11128       .......... .......... ...G...... .......... .......... G........A ..........
11368       .......... .......... ...G...... .......... .......... G........A ..........
IAI1        .......... .......... ...G...... .......... .......... G........A ..........
E110019     .......... .......... ...G...... .......... .......... G......... ..........
B7A         .......... .......... .......... .......... .......... .......... ..........
CB9615      .......... .......... ...G...... .......... .......... G......... ..........
O157_Sakai  .......... .......... ...G...... .......... .......... G......... ..........
IAI39       .......... .......... ...G...... .......... .......... G......... ..........
UMN026      .......... .......... ...G...... .......... .......... G......... ..........
SMS-3-5     .......... .......... ...G...... .......... .......... G......... ..........
536         .......... .......... ...G...... .......... .......... G........A ..........
APEC_O1     .......... .......... ...G...... .......... .......... G........A ..........
CFT073      .......... .......... ...G...... .......... .......... G......... ..........
E2348/69    .......... .......... ...G...... .......... .......... G......... ..........
UTI89       .......... .......... ...G...... .......... .......... G........A ..........
F11         .......... .......... ...G...... .......... .......... G........A ..........
ED1a        .......... .......... ...G...... .......... .......... G........A ..........
83972       .......... .......... ...G...... .......... .......... G......... ..........
S88         .......... .......... ...G...... .......... .......... G........A ..........
E._albertii .......... ....T.A... .......... ..A....... .......... G......... ..........

           1471
K-12        GTTTCGGACG ACCATACACT TCCGCTTTAA CGCCTTCAGC TTTCATCTCA GCGCGCAGAT GACCAACGAA
53638       .......... .......... .......... .......... .......... .......... ..........
ATCC_8739   .......... .......... ..A....... .......... ......T... .......... .G..G..A..
HS          .......... .......... ..A....... .......... .......... .......... ....G..A..
BW2952      .......... .......... .......... .......... .......... .......... ..........
12009       .......... .......... ..A....... .......... .......... .......... .G..G..A..
B171        .......... .......... ..A....... .......... .......... .......... .G..G..A..
E24377A     .......... .......... ..A....... .......... .......... .......... ....G..A..
SE11        .......... .......... ..A....... .......... .......... .......... .G..G..A..
11128       .......... .......... ..A....... .......... .......... .......... .G..G..A..
11368       .......... .......... ..A....... .......... .......... .......... .G..G..A..
IAI1        .......... .......... ..A....... .......... .......... .......... .G..G..A..
E110019     .......T.. .......... ..A....... .......... .......... .......... .G..G..A..
B7A         .......... .......... ..A....... .......... .......... .......... ....G..A..
CB9615      .......... .......... ..A....... .......... .......... .......... ....G..A..
O157_Sakai  .......... .......... ..A....... .......... .......... .......... ....G..A..
IAI39       .......... .......... ..A....... .......... .......... .......... .G..G..A..
UMN026      .......T.. .......... ..A....... .......... .......... .......... .G..G..A..
SMS-3-5     .......... .......... ..A....... .......... .......... ........G. .G..G..A..
536         .......... G......... ..A....... .......... .......... .......... .G..G..A..
APEC_O1     .......... G......... ..A....... .......... .......... .......... .G..G..A..
CFT073      .......T.. .......... ..A....... .A........ .......... .......... .G..G..A..
E2348/69    .......... .......... ..A....... .......... .......... .......... .G..G..A..
UTI89       .......... G......... ..A....... .......... .......... .......... .G..G..A..
F11         .......... G......... ..A....... .......... .......... .......... .G..G..A..
ED1a        .......... G......... ..A....... .......... .......... .......... .G..G..A..
83972       .......T.. .......... ..A....... .A........ .......... .......... .G..G..A..
S88         .......... G......... ..A....... .......... .......... .......... .G..G..A..
E._albertii .......G.. G.....T..C ..A....... .A.....G.. .........T .......... ....G..A..
```

```
            1541
K-12          CTCTTCGATG TAGTGTTCGC GGTCGAGACG CCGTTCATGC AGCAGTTTGG CAATTCGTTT GTATTCGGTT
53638         .......... .......... .......... .......... .......... .......... ..........
ATCC_8739     .........A .......... .......... G......... ........T. .G..G..... .........C
HS            .........A .......... .......... G......... .A......T. .G..G..... .........C
BW2952        .......... .......... .......... .......... .......... .......... ..........
12009         .........A .......... .......... G......... ........T. .G..G..... .........C
B171          .........A .......... .......... G......... ........T. .G..G..... .........C
E24377A       .........A .......... .......... G......... ........T. .G..G..... .........C
SE11          .........A .......... .......... G......... ........T. .G..G..... .........C
11128         .........A .......... .......... G......... ........T. .G..G..... .........C
11368         .........A .......... .......... G......... ........T. .G..G..... .........C
IAI1          .........A .......... .......... G......... ........T. .G..G..... .........C
E110019       .........A .......... .......... G......... ........T. .G..G..... .........C
B7A           .........A .......... .......... G......... ........T. .G..G..... .........C
CB9615        .........A .......... .......... G......... .A......T. .G..G..... .........C
O157_Sakai    .........A .......... .......... G......... .A......T. .G..G..... .........C
IAI39         .........A .......... .......... G......... ........T. .G..G..... .........C
UMN026        .........A .......... .......... G......... ........T. .G..G..... .........C
SMS-3-5       .........A .......... .......... G......... ........T. .G..G..... .........C
536           .........A .......... .......... G......... ........T. .G..G..... .........C
APEC_O1       .........A .......... .......... G......... ........T. .G..G..... .........C
CFT073        .........A .......... .......... G......... ........T. .G..G..... .........C
E2348/69      .........A .......... ....A..... G......... ........T. .G..G..... .........C
UTI89         .........A .......... .......... G......... ........T. .G..G..... .........C
F11           .........A .......... .......... G......... ........T. .G..G..... .........C
ED1a          .........A .......... .......... G......... ........T. .G..G..... .........C
83972         .........A .......... .......... G......... ........T. .G..G..... .........C
S88           .........A .......... .......... G......... ........T. .G..G..... .........C
E._albertii   ......A... .......... .A........ G..C...... .......... .G..G..C.. ......C..C

            1611
K-12          GGATGGAGGT AACGGAAGCA GTAATCTTCC AGTTCCCATT TCAGTTGTCC GATTCCGAGA CGGTTAGCCA
53638         .......... .......... .......... .......... .......... .......... ..........
ATCC_8739     ..G.....A. .......... .......... ..C....... .......C.. A..C..A... .....C....
HS            ..G..C..A. .......... .......... ..C....... .......C.. A..C..A... .....T....
BW2952        .......... .......... .......... .......... .......... .......... ..........
12009         ..G.....A. .......... .......... ..C....... .......C.. A..C..A... .....C....
B171          ..G.....A. .......... .......... ..C....... .......C.. A..C..A... .....C....
E24377A       ..G..C..A. .......... .......... ..C....... .......C.. A..C..A... .....C....
SE11          ..G.....A. .......... .......... ..C....... .......C.. A..C..A... .....C....
11128         ..G.....A. .......... .......... ..C....... .......C.. A..C..A... .....C....
11368         ..G.....A. .......... .......... ..C....... .......C.. A..C..A... .....C....
IAI1          ..G.....A. .......... .......... ..C....... .......C.. A..C..A... .....C....
E110019       ..G.....A. .......... .......... ..C....... .......C.. A..C..A... .....C....
B7A           ..G..C..A. .......... .......... ..C....... .......C.. A..C..A... .....C....
CB9615        ..G.....A. .......... .......... ..C....... .......C.. A..C..A... .....T....
O157_Sakai    ..G.....A. .......... .......... ..C....... .......C.. A..C..A... .....T....
IAI39         ..G..C..A. .......... .......... ..C....... .......C.. A..C..A... .....C....
UMN026        ..G.....A. .......... .......... ..C....... .......... A..C..A... .....C....
SMS-3-5       ..G.....A. .......... .......... ..C....... .......C.. A..C..A... .....C....
536           ..G.....A. .......... .......... ..C....... .......C.. A..C..A... .....C....
APEC_O1       ..G.....A. .......... .......... ..C....... .......C.. A..C..A... .....C....
CFT073        ..G..C..A. .......... .......... ..C....... .......C.. A..C..A... .....C....
E2348/69      ..G..C..A. .......... .......... ..C....... .......C.. A..C..A... .....C....
UTI89         ..G.....A. .......... .......... ..C....... .......C.. A..C..A... .....C....
F11           ..G.....A. .......... .......... ..C....... .......C.. A..C..A... .....C....
ED1a          ..G.....A. .......... .......... ..C....... .......C.. A..C..A... .....C....
83972         ..G..C..A. .......... .......... ..C....... .......C.. A..C..A... .....C....
S88           ..G.....A. .......... .......... ..C....... .......C.. A..C..A... .....C....
E._albertii   ..G.....A. .......... .......... .......... .......... ...C..A... .....C....
```

```
            1681
K-12          GCGGTGCGTA GATGTTGGTA CACTCTTTTG CCGCCAGTAC ACGTTCATCT TCCGGCGCAT CTTTTACTTC
53638         .......... .......... .......... .......... .......... .......... ..........
ATCC_8739     A...C..... ...A.....G .......... .......... G......... .......... ..........
HS            A...C..... ...A.....G .......... .......... G......... .......... ..........
BW2952        .......... .......... .......... .......... .......... .......... ..........
12009         A...C..... ...A.....G .......... .......... G......... .......... ..........
B171          A...C..... ...A.....G .......... .......... G......... .......... ..........
E24377A       A...C..... ...A.....G .......... .......... G......... .......... ..........
SE11          A...C..... ...A.....G .......... .......... G......... .......... ..........
11128         A...C..... ...A.....G .......... .......... G......... .......... ..........
11368         A...C..... ...A.....G .......... .......... G......... .......... ..........
IAI1          A...C..... ...A.....G .......... .......... G......... .......... ..........
E110019       A...C..... ...A.....G .......... .......... G......... .......... ..........
B7A           A...C..... ...A.....G .......... .......... G......... .......... ..........
CB9615        A...C..... ...A.....G .......... .......... G......... .......... ..........
O157_Sakai    A...C..... ...A.....G .......... .......... G......... .......... ..........
IAI39         A...C..... ...A.....G .......... .......... G......... .......... ..........
UMN026        A...C..... ...A.....G .......... .......... G......... ........G. ..........
SMS-3-5       A...C..... ...A.....G .......... .......... G......... ........G. ..........
536           A...C..... ...A.....G .......... .......... .......... .......... ..........
APEC_O1       A...C..... ...A.....G .......... .......... G......... .......... ..........
CFT073        A...C..... ...A.....G .......... .......... G......... .......... ..........
E2348/69      A...C..... ...A.....G .......... .......... G......... .......... ..........
UTI89         A...C..... ...A.....G .......... .......... G......... .......... ..........
F11           A...C..... ...A.....G .......... .......... .......... .......... ..........
ED1a          A...C..... ...A.....G .......... .......... G......... .......... ..........
83972         A...C..... ...A.....G .......... .......... G......... .......... ..........
S88           A...C..... ...A.....G .......... .......... G......... .......... ..........
E._albertii   ....C..... ...A.....G .......... .......C.. G.....G... ........G. ....C.....

            1751
K-12          GCGCAGATGA GCAATACGCT CCGCCAGTTT GATGACTACG CAGCGAAAAT CATCGACCAT CGCCAATAAC
53638         .......... .......... .......... .......... .......... .......... ..........
ATCC_8739     .......... .......... .......... ......C... .......... .......... ..........
HS            C......... .......... .......... ......C... .......... .......... ..........
BW2952        .......... .......... .......... .......... .......... .......... ..........
12009         .......... .......... .......... ......C... .......... .......... ..........
B171          .......... .......... .......... ......C... .......... .......... ..........
E24377A       .......... .......... .......... ......C... .......... .......... ..........
SE11          .......... .......... .......... ......C... .......... .......... ..........
11128         .......... .......... .......... ......C... .......... .......... ..........
11368         .......... .......... .......... ......C... .......... .......... ..........
IAI1          .......... .......... .......... ......C... .......... .......... ..........
E110019       .......... .......... .......... ......C... .......... .......... ..........
B7A           .......... .......... .......... ......C... .......... .......... ..........
CB9615        A......... .......... .......... ......C... .......... .......... ..........
O157_Sakai    A......... .......... .......... ......C... .......... .......... ..........
IAI39         ......G... .......... .......... ......C... .......... .......... ..........
UMN026        .......... .......... .T........ ......C... .......... .......... ..........
SMS-3-5       .......... .......... .T........ ......C... .....G.... .......... ..........
536           .......... .......... .......... .......... .......... .......... ..........
APEC_O1       ......G... .......... .......... ......C... .......... .......... ..........
CFT073        ......G... .......... .......... ......C... .......... .......... ..........
E2348/69      ......G... .......... .......... ......C... .......... .......... ..........
UTI89         ......G... .......... .......... ......C... .......... .......... ..........
F11           .......... .......... .......... .......... .......... .......... ..........
ED1a          ......G... .......... .......... ......C... .......... .......... ..........
83972         ......G... .......... .......... ......C... .......... .......... ..........
S88           ......G... .......... .......... ......C... .......... .......... ..........
E._albertii   .......... .....T.... .......... ......C... ..A..G..G. .......... .....GC...
```

```
          1821
K-12         ATCCGGCGAA CGTTATCGAC CTGTTCGGAG GAAACAGAAT CAGTGTGCGT CGCTTTCAGC TGGCGGATCG
53638        .......... .......... .......... .......... .......... .......... ..........
ATCC_8739    .......... .......... .......... .......... .......... .......... ..........
HS           .......... .......... .......... .......... .......... .......... ..........
BW2952       .......... .......... .......... .......... .......... .......... ..........
12009        .......... .......... .......... .......... .......... .......... ..........
B171         .......... .......... .......... .......... .......... T......... ..........
E24377A      .......... .......... .......... .......... .......... .......... ..........
SE11         .......... .......... .......... .......... .......... .......... ..........
11128        .......... .......... .......... .......... .......... .......... ..........
11368        .......... .......... .......... .......... .......... .......... ..........
IAI1         .......... .......... .......... .......... .......... .......... ..........
E110019      .......... .......... .......... .......... .......... .......... ..........
B7A          .......... .......... .......... .......... .......... .......... ..........
CB9615       .......... .......... .......... .......... .......... .......... ..........
O157_Sakai   .......... .......... .......... .......... .......... .......... ..........
IAI39        .......... .......... .......... .......... .......... .......... ..........
UMN026       .......... .......... ...C...... .......... .G..A..... .......... ..........
SMS-3-5      .......... .......... .......... .......... .G..A..... .......... ..........
536          .......... .......... .......... .......... .......... .......... ..........
APEC_O1      .......... .......... .......... .......... .......... .......... ..........
CFT073       .......... .......... .......... .......... .......... .......... ..........
E2348/69     .......... .......... .......... .......... .G..A..... .......... ..........
UTI89        .......... .......... .......... .......... .......... .......... ..........
F11          .......... .......... .......... .......... .......... .......... ..........
ED1a         .......... .......... .......... .......... .......... .......... ..........
83972        .......... .......... .......... .......... .......... .......... ..........
S88          .......... .......... .......... .......... .......... .......... ..........
E._albertii  .......... .......A.. .......... .....G.... .G........ G.......A. ........T.

          1891
K-12         CCGCCATATC ACGCACGCCG TGAATAAGGT TAACGACCGA CTTACCGACG CTCTCACGCA GCACATCTTC
53638        .......... .......... .......... .......... .......... .......... ..........
ATCC_8739    .......... .......... .......... .......... .......... .......... ..........
HS           .......... .......... .......... .......... .......... .......... ..........
BW2952       .......... .......... .......... .......... .......... .......... ..........
12009        .......... .......... .......... .......... .......... .......... ..........
B171         .......... .......... .......... .......... .......... .......... ..........
E24377A      .......... .......... .......... ....A..... .......... .......... ..........
SE11         .......... .......... .......... .......... .......... .......... ..........
11128        .......... .......... .......... .......... .......... .......... ..........
11368        .......... .......... .......... .......... .......... .......... ..........
IAI1         .......... .......... .......... .......... .......... .......... ..........
E110019      .......... .......... .......... ....A..... .......... .......... ..........
B7A          .......... .......... .......... ....A..... .......... .......... ..........
CB9615       .......... .......... .......... .......... .......... .......... ..........
O157_Sakai   .......... .......... .......... .......... .......... .......... ..........
IAI39        .......... .......... .......... .......... .......... .......... ..........
UMN026       .......... .......... .......... .......... .......... .......... ..........
SMS-3-5      .......... .......... .......... .......... .......... .......... ..........
536          .......... .......... .......... .......... .......... ..A....... ..........
APEC_O1      .......... .......... .......... .......... .......... .......... ..........
CFT073       .......... .......... .......... .......... .......... .......... ..........
E2348/69     .......... .......... .......... .......... .......... .......... ..........
UTI89        .......... .......... .......... .......... .......... .......... ..........
F11          .......... .......... .......... .......... .......... ..A....... ..........
ED1a         .......... .......... .......... .......... .......... .......... ..........
83972        .......... .......... .......... .......... .......... .......... ..........
S88          .......... .......... .......... .......... .......... .......... ..........
E._albertii  .......... C......... .......... .G..C..... .......... ..T....... .T........
```

```
         1961
K-12         GCTGACTACG TTGGCATCCG CCAGAGGGAA AAGCAGCGCC GCCCGCAGCG TGTCAATGTC CATACTTAAT
53638        .......... .......... .......... .......... .......... .......... ..........
ATCC_8739    .......... ........A. .......... C......... .......... .......... ..........
HS           .......... .......... .......... .......... .......... .......... ..........
BW2952       .......... .......... .......... .......... .......... .......... ..........
12009        .......... ........A. .......... C......... .......... .......... ..........
B171         .......... ........A. .......... C......... .......... .......... ..........
E24377A      ...A...... ........A. .......... C......... .......... .......... ..........
SE11         .......... ........A. .......... C......... .......... .......... ..........
11128        .......... ........A. .......... C......... .......... .......... ..........
11368        .......... ........A. .......... C......... .......... .......... ..........
IAI1         .......... ........A. .......... C......... .......... .......... ..........
E110019      ...A...... ........A. .......... C......... .......... .......... ..........
B7A          ...A...... ........A. .......... C......... .......... .......... ..........
CB9615       .......... ........A. .......... C......... .......... .......... ..........
O157_Sakai   .......... ........A. .......... C......... .......... .......... ..........
IAI39        .......... ........A. .......... C......... .......... .......... ..........
UMN026       .......... .......... .......... C......... .......... .......... ..........
SMS-3-5      .......... ........A. .......... C......... .......... .......... ..........
536          .......... ........G. .......... C......... .......... .......... ..........
APEC_O1      .......... ........A. .......... C......... .......... .......... ..........
CFT073       .......... ........A. .......... C......... .......... .......... ..........
E2348/69     .......... ........G. .......... C......... .......... .......... ..........
UTI89        .......... ........A. .......... C......... .......... .......... ..........
F11          .......... ........G. .......... C......... .......... .......... ..........
ED1a         .......... ........A. .......... C......... .......... .......... ..........
83972        .......... ........A. .......... C......... .......... .......... ..........
S88          .......... ........A. .......... C......... .......... .......... ..........
E._albertii  A........A ........A. .......... G......... ..T..T.... .......A.. ..........

         2031
K-12         GTCGAGAGGA TCTCCACCAT CTCAACACCA CGCCACAATA ACAGACTGGC ATCCGGATGC CCCTGCGTCT
53638        .......... .......... .......... .......... .......... .......... ..........
ATCC_8739    .......... .......... .......... .......... .......... .......... ..........
HS           .......... .......... .......... .......... .......... .......... ..........
BW2952       .......... .......... .......... .......... .......... .......... ..........
12009        .......... .......... .......... .......... .......... .......... ..........
B171         .......... .......... .......... .......... .......... .......... ..........
E24377A      .......... .......... .......... .......... .......... .......... ..........
SE11         .......... .......... .......... .......... .......... .......... ..........
11128        .......... .......... .......... .......... .......... .......... ..........
11368        .......... .......... .......... .......... .......... .......... ..........
IAI1         .......... .......... .......... .......... .......... .......... ..........
E110019      .......... .......... .......... .......... .......... .......... ..........
B7A          .......... .......... .......... .......... .......... .......... ..........
CB9615       .......... .......... .......... .......... .......... .......... ..........
O157_Sakai   .......... .......... .......... .......... .......... .......... ..........
IAI39        .......... .......... .......... .......... ...A...... .......... ..........
UMN026       .......... .......... .......... .......... .......... .......... ..........
SMS-3-5      .......... .......... .......... .......... .......... .......... ........A.
536          .......... .......... .......... .......... .......... .......... ..........
APEC_O1      .......... .......... .......... .......... .......... .......... ..........
CFT073       .......... .......... .......... .......... .......... .......... ..........
E2348/69     .......... .......... .......... .......... .......... .......... ..........
UTI89        .......... .......... .......... .......... .......... .......... ..........
F11          .......... .......... .......... .......... .......... .......... ..........
ED1a         .......... .......... .......... .......... .......... .......... ..........
83972        .......... .......... .......... .......... .......... .......... ..........
S88          .......... .......... .......... .......... .......... .......... ..........
E._albertii  ........T. ....T..... ...G...... ........C. G......... ...T...... ..........
```

```
            2101
K-12         GTTGCAGACA ATACGCCCAG GTTTCGGCTA AGCACTCACA CGACTTCTGG CTGGTAATAC CCAGACTTGC
53638        .......... .......... .......... .......... .......... .......... ..........
ATCC_8739    .......... .......... .......... .......... .......... .......... ..........
HS           .......... .......... .......... .......... ...A...... .......... ..........
BW2952       .......... .......... .......... .......... .......... .......... ..........
12009        .......... .......... .......... .......... .......... .......... ..........
B171         .......... .......... .......... .......... .......... .......... ..........
E24377A      .......... .......... .......... .......... .......... .......... ..........
SE11         .......... .......... .......... .......... .......... .......... ..........
11128        .......... .......... .......... .......... .......... .......... ..........
11368        .......... .......... .......... .......... .......... .......... ..........
IAI1         .......... ....A..... .......... .......... .......... .......... ..........
E110019      .......... .......... .......... .......... .......... .......... ..........
B7A          .......... .......... .......... .......... .......... .......... ..........
CB9615       .......... .......... .......... .......... .......... .......... ..........
O157_Sakai   .......... .......... .......... .......... .......... .......... ..........
IAI39        .......... .......... .......... .......... .......... .......... ..........
UMN026       .......... .......... .......... .......... .......... .......... ..........
SMS-3-5      .......... .......... .......... .......... .......... .......... ..........
536          .......... .......... .......... .......... .......... .......... ..........
APEC_O1      .......... .......... .......... .......... .......... .......... ..........
CFT073       .......... .......... .......... .......... .......... .......... ..........
E2348/69     .......... .......... .......... .......... .......... .......... ..........
UTI89        .......... .......... .......... .......... .......... .......... ..........
F11          .......... .......... .......... .......... .......... .......... ..........
ED1a         .......... .......... .......... .......... .......... .......... ..........
83972        .......... .......... .......... .......... .......... .......... ..........
S88          .......... .......... .......... .......... .......... .......... ..........
E._albertii  .C........ .......... .......... .......... ...T...... ....C..... ..........

            2171
K-12         GATCCATTTT TCCGGATCAA ATTCACCAGC CTTATTGATA TGTGCACTTC TTACCGCAAC CATCGTCCTC
53638        .......... .......... .......... .......... .......... .......... ..........
ATCC_8739    .......... .......... .......... .......... .......... .......... ..........
HS           .......... .......... .......... .......... .......... .......... ..........
BW2952       .......... .......... .......... .......... .......... .......... ..........
12009        .......... .......... .......... .......... .......... .......... ...T......
B171         .......... .......... .......... .......... .......... .......... ...T......
E24377A      .......... .......... .......... .......... .......... .......... ..........
SE11         .......... .......... .......... .......... .......... .......... ...T......
11128        .......... .......... .......... .......... .......... .......... ...T......
11368        .......... .......... .......... .......... .......... .......... ...T......
IAI1         .......... .......... .......... .......... .......... .......... ...T......
E110019      .......... .......... .......... .......... .......... .......... ..........
B7A          .......... .......... .......... .......... .......... .......... ..........
CB9615       .......... .......... .......... .......... .......... .......... ..........
O157_Sakai   .......... .......... .......... .......... .......... .......... ..........
IAI39        .......... .......... .......... .......... .......... .......... ..........
UMN026       .......... .......... .......... .......... .......... .......... ..........
SMS-3-5      .......... .......... .......... .......... .......... .......... ..........
536          .......... .......... .......... .......... .......... .......... ..........
APEC_O1      .......... .......... .......... .......... .......... .......... ..........
CFT073       .......... .......... .......... .......... .......... .......... ..........
E2348/69     .......... .......... .......... .......... .......... .......... ..........
UTI89        .......... .......... .......... .......... .......... .......... ..........
F11          .......... .......... .......... .......... .......... .......... ..........
ED1a         .......... .......... .......... .......... .......... .......... ..........
83972        .......... .......... .......... .......... .......... .......... ..........
S88          .......... .......... .......... .......... .......... .......... ..........
E._albertii  .......... .G........ .......... .......... .......... .......... ..........
```

```
             2241
K-12            TCCTTTAGGG ACCAGACCTG CCGAAATCGG CAAATCGCAA CTATTTAACG CGCGAGAAAA GTACCATCGA
53638           .......... .......... .......... .......... .......... .......... ..........
ATCC_8739       .......... .......... .......... .......T.. .......... .......... ..........
HS              .......... .......... .......... .......... .......... .......... ..........
BW2952          .......... .......... .......... .......... .......... .......... ..........
12009           .......... .......... .......... .......T.. .......... .......... ..........
B171            .......... .......... .......... .......T.. .......... .......... ..........
E24377A         .......... .......... .......... .......T.. .......... .......... ..........
SE11            .......... .......... .......... .......T.. .......... .......... ..........
11128           .......... .......... .......... .......T.. .......... .......... ..........
11368           .......... .......... .......... .......T.. .......... .......... ..........
IAI1            .......... .......... .......... .......T.. .......... .......... ..........
E110019         .......... .......... .......... .......T.. .......... .......... ..........
B7A             .......... .......... .......... .......T.. .......... .......... ..........
CB9615          .......... .......... .......... .......... .......... .......... ..........
O157_Sakai      .......... .......... .......... .......... .......... .......... ..........
IAI39           .......... .......... .......... .......... .......... .......... ..........
UMN026          .......... .......... .......... .......T.. .......... .......... ..........
SMS-3-5         .......... .......... .......... .......T.. .......... .......... ..........
536             .......... .......... .......... .......T.. .......... .......... ..........
APEC_O1         .......... .......... .......... .......T.. .......... .......... ..........
CFT073          .......... .......... .......... .......T.. .......... .......... ..........
E2348/69        .......... .......... .......... .......T.. .......... .......... ..........
UTI89           .......... .......... .......... .......T.. .......... .......... ..........
F11             .......... .......... .......... .......T.. .......... .......... ..........
ED1a            .......... .......... .......... .......T.. .......... .......... ..........
83972           .......... .......... .......... .......T.. .......... .......... ..........
S88             .......... .......... .......... .......T.. .......... .......... ..........
E._albertii     .......... .......... .......... ...G...... .......... .......... .C........

             2311
K-12            TTCCAGATGT CCCGTGTGTG GGAACATATC CAGCATCGCC AGTCGCGCAA TGGTATATCC TGCTTTTAAT
53638           .......... .......... .......... .......... .......... .......... ..........
ATCC_8739       .......... .......... .......... .......... .......... .......... ..........
HS              .......... .......... .......... .......... .......... .......... ..........
BW2952          .......... .......... .......... .......... .......... .......... ..........
12009           .......... .......... .......... .......... .......... .......... ..........
B171            .......... .......... .......... .......... .......... .......... ..........
E24377A         .......... .......... .......... .......... .......... .......... ..........
SE11            .......... .......... .......... .......... .......... .......... ..........
11128           .......... .......... .......... .......... .......... .......... ..........
11368           .......... .......... .......... .......... .......... .......... ..........
IAI1            .......... .......... .......... .......... .......... .......... ..........
E110019         .......... .......... .......... .......... .......... .......... ..........
B7A             .......... .......... .......... .......... .......... .......... ..........
CB9615          .......... .......... .......... .......... .......... .......... ..........
O157_Sakai      .......... .......... .......... .......... .......... .......... ..........
IAI39           .......... .......... .......... .......... .......... .......... ..........
UMN026          .......... .......... .......... .......... .......... .......... ..........
SMS-3-5         .......... .......... .......... .......... .......... .......... ..........
536             .......... .......... .......... .......... .......... .......... ..........
APEC_O1         .......... .......... .......... .......... .......... .......... ..........
CFT073          .......... .......... .......... .......... .......... .......... ..........
E2348/69        .......... .......... .......... .......... .......... .......... ..........
UTI89           .......... .......... .......... .......... .......... .......... ..........
F11             .......... .......... .......... .......... .......... .......... ..........
ED1a            .......... .......... .......... .......... .......... .......... ..........
83972           .......... .......... .......... .......... .......... .......... ..........
S88             .......... .......... .......... .......... .......... .......... ..........
E._albertii     .........C ........C. .......... .......... .......... .......... ..........
```

```
             2381
K-12         AACGCTTCGC TATCCCGAGC CAGCGTTGCA GGGTTACAGG ATACATAAAC TATACGAATA GGTTCCAGTT
53638        .......... .......... .......... .......... .......... .......... ..........
ATCC_8739    .......... .......... .......... ..A....... .......... .......... ..........
HS           .......... .......... .......... .......... .......... .......... ..........
BW2952       .......... .......... .......... .......... .......... .......... ..........
12009        .......... .......... .......... .......... .......... .......... ..........
B171         .......... .......... .......... .......... .......... .......... ..........
E24377A      .......... .......... .......... ..A....... .......... .......... ..........
SE11         .......... .......... .......... .......... .......... .......... ..........
11128        .......... .......... .......... .......... .......... .......... ..........
11368        .......... .......... .......... .......... .......... .......... ..........
IAI1         .......... .......... .......... .......... .......... .......... ..........
E110019      .......... .......... .......... ..A....... .......... .......... ..........
B7A          .......... .......... .......... ..A....... .......... .......... ..........
CB9615       .......... .......... .......... .......... .......... .......... ..........
O157_Sakai   .......... .......... .......... .......... .......... .......... ..........
IAI39        .......... .......... .......... .......... .......... .......... ..........
UMN026       .......... .......... .......... .......... .......... .......... ..........
SMS-3-5      .......... .......... .......... ..A....... .......... .......... ..........
536          .......... .......... .......... .......... .......... .......... ..........
APEC_O1      .......... .......... .......... .......... .......... .......... ..........
CFT073       .......... .......... .......... .......... .......... .......... ..........
E2348/69     .......... .......... .......... .......... .......... .......... ..........
UTI89        .......... .......... .......... .......... .......... .......... ..........
F11          .......... .......... .......... .......... .......... .......... ..........
ED1a         .......... .......... .......... .......... .......... .......... ..........
83972        .......... .......... .......... .......... .......... .......... ..........
S88          .......... .......... .......... .......... .......... .......... ..........
E._albertii  .......... .......... .......... .......... .......... A........G ..........

             2451
K-12         TTATAATTTG CTGCATAACA CCTGCGGCAC CTGCTCGCGC CGGGTCCAGC AACACTTTAT CGAAGCCGTT
53638        .......... .......... .......... .......... .......... .......... ..........
ATCC_8739    .......... .......... .......... .......... .......... .......... ..........
HS           .......... .......... .......... .......... .......... .......... ..........
BW2952       .......... .......... .......... .......... .......... .......... ..........
12009        .......... .......... .......... .......... .......... .......... ..........
B171         .......... .......... .......... .......... .......... .......... ..........
E24377A      .......... .......... .......... .......... G......... .......... ..........
SE11         .......... .......... .......... .......... .......... .......... ..........
11128        .......... .......... .......... .......... .......... .......... ..........
11368        .......... .......... .......... .......... .......... .......... ..........
IAI1         .......... .......... .......... .......... .......... .......... ..........
E110019      .......... .......... .......... .......... G......... .......... ..........
B7A          .......... .......... .......... .......... G......... .......... ..........
CB9615       .......... .......... .......... .......... .......... .......... ..........
O157_Sakai   .......... .......... .......... .......... .......... .......... ..........
IAI39        .......... .......... .......... .......... .......... .......... ..........
UMN026       .......... .........T .......... .......... .......... .......... ..........
SMS-3-5      .......... .......... .......... .......... .......... .......... ..........
536          .......... .......... .......... .......... ........A. .......... ..........
APEC_O1      .......... .......... .......... .......... .......... .......... ..........
CFT073       .......... .......... .......... .......... ........A. .......... ..........
E2348/69     .......... .......... .......... .......... .......... .......... ..........
UTI89        .......... .......... .......... .......... .......... .......... ..........
F11          .......... .......... .......... .......... ........A. .......... ..........
ED1a         .......... .......... .......... .......... ........A. .......... ..........
83972        .......... .......... .......... .......... ........A. .......... ..........
S88          .......... .......... .......... .......... .......... .......... ..........
E._albertii  .......... T......... .......... ....A..... .........T ........G. .A..A.....
```

```
              2521
K-12          TTTCGCCCAC GGCTGCTTTG TGACATCTTC TTCAAGATTT TCGTGATAAA ACGTCACATT CTGTAAGCCA
53638         .......... .......... .......... .......... .......... .......... ..........
ATCC_8739     .......... .......... .......... .......... ..A....... .......... ..........
HS            .......... .......... .......... .......... .......... .......... ..........
BW2952        .......... .......... .......... .......... .......... .......... ..........
12009         .......... .......... .......... .......... .......... .......... ......A...
B171          .......... .......... .......... .......... .......... .......... ......A...
E24377A       .......... .......... .......... .......... ..A....... .......... ..........
SE11          .......... .......... .......... .......... .......... .......... ......A...
11128         .......... .......... .......... .......... .......... .......... ......A...
11368         .......... .......... .......... .......... .......... .......... ......A...
IAI1          .......... .......... .......... .......... .......... .......... ......A...
E110019       .......... .......... .......... .......... ..A....... .......... ..........
B7A           .......... .......... .......... .......... ..A....... .......... ..........
CB9615        .......... .......... .......... .......... .......... .......... ........AG
O157_Sakai    .......... .......... .......... .......... .......... .......... ........AG
IAI39         .......... .......... .......... .......... .......... .......... G.........
UMN026        .......... .......... .......... .......... .......... .......... G.........
SMS-3-5       .......... .......... .......... .......... .......... .......... G.........
536           .......... .......... .......... .......... .......... .......... ..........
APEC_O1       .......... .......... .......... .......... .......... .......... ..........
CFT073        .......... .......... .......... .......... .......... .......... ..........
E2348/69      .......... .......... .......... .......... .......... .......... ..........
UTI89         .......... .......... .......... .......... .......... .......... ..........
F11           .......... .......... .......... .......... .......... .......... ..........
ED1a          .......... .......... .......... .......... .......... .......... ..........
83972         .......... .......... .......... .......... .......... .......... ..........
S88           .......... .......... .......... .......... .......... .......... ..........
E._albertii   .........T .......... .......A.. .......... .......... .......... A.........

              2591
K-12          TTAAGACGCG CATTCTGCTG GCCTTTTTCC ACCAGCGCCG GAACACCTTC TACACCGACC ACACTGGCAG
53638         .......... .......... .......... .......... .......... .......... ..........
ATCC_8739     .......... .......... .......... .......... .......... ......C... ..........
HS            .......... .......... .......... .......... .......... .......... ..........
BW2952        .......... .......... .......... .......... .......... .......... ..........
12009         .......... .......... .......... .......... .......... .......... ........T.
B171          .......... .......... .......... .......... .......... .......... ........T.
E24377A       .......... .......... .......... .......... .......... ......C... ..........
SE11          .......... .......... .......... .......... .......... .......... ........T.
11128         .......... .......... .......... .......... .......... .......... ........T.
11368         .......... .......... .......... .......... .......... .......... ........T.
IAI1          .......... .......... .......... .......... .......... .......... ........T.
E110019       .......... .......... .......... .......... .......... ......C... ..........
B7A           .......... .......... .......... .......... .......... ......C... ..........
CB9615        .......... .......... .......... .......... .......... ......C... ..........
O157_Sakai    .......... .......... .......... .......... .......... ......C... ..........
IAI39         .......... .......... .......... .......... .......... ......C... ..........
UMN026        .......... .......... .......... .......... .......... ......C... ..........
SMS-3-5       .......... .......... .......... .......... ....G..... ......C... ..........
536           .......... .......... .......... .......... .......... ......C... ..........
APEC_O1       .......... .......... .......... .......... .......... ......C... ..........
CFT073        .......... .......... .......... .......... .......... ......C... ..........
E2348/69      .......... .......... .......... .......... .......... .......... ..........
UTI89         .......... .......... .......... .......... .......... ......C... ..........
F11           .......... .......... .......... .......... .......... ......C... ..........
ED1a          .......... .......... .......... .......... .......... ......C... ..........
83972         .......... .......... .......... .......... .......... ......C... ..........
S88           .......... .......... .......... .......... .......... ......C... ..........
E._albertii   .......... .G....C... A......... ........A. .C........ C..G.....G ..G..T..C.
```

```
             2661
K-12         CTTGTGTCGC CAATGGCAGT GTAAAGTTGC CCATACCGCA GAACAGATCC AGTACGCGAT CTTCAGGTTG
53638        .......... .......... .......... .......... .......... .......... ..........
ATCC_8739    .......... .......... .......... .......... .......... .......... ....T.....
HS           .......... .......... .......... .......... .......... .......... ..........
BW2952       .......... .......... .......... .......... .......... .......... ..........
12009        .....T.... .......... .......... .......... .......... .......... ..........
B171         .....T.... .......... .......... .......... .......... .......... ..........
E24377A      .......... .......... .......... .......... .......... .......... ..........
SE11         .....T.... .......... .......... .......... .......... .......... ..........
11128        .....T.... .......... .......... .......... .......... .......... ..........
11368        .....T.... .......... .......... .......... .......... .......... ..........
IAI1         .....T.... .......... .......... .......... .......... .......... ..........
E110019      .......... .......... .......... .......... .......... .......... ..........
B7A          .......... .......... .......... .......... .......... .......... ..........
CB9615       .......... ......T... .......... .......... .......... .......... .........C
O157_Sakai   .......... ......T... .......... .......... .......... .......... .........C
IAI39        .......... .......... .......... .......... .......... .......... ..........
UMN026       .......... .......... .......... .......... .......... .......... ..........
SMS-3-5      .......... .......... .......... .......... .......... .......... ..........
536          .......... .......... .......... .......... .......... .......... .........C
APEC_O1      .......... .......... .......... .......... .......... .......A.. .........C
CFT073       .......... .......... .......... .......... .......... .......... .........C
E2348/69     .......... .......... .......... .......... .......... .......... ..........
UTI89        .......... .......... .......... .......... .......... .......A.. .........C
F11          .......... .......... .......... .......... .......... .......... .........C
ED1a         .......... .......... .......... .......... .......... .......... .........C
83972        .......... .......... .......... .......... .......... .......... .........C
S88          .......... .......... .......... .......... .......... .......A.. .........C
E._albertii  .AC.A..A.. G.CA..A..A .....A..C. ....G..... .......... ..C..A.... .G..T..C..

             2731
K-12         CACATCCAGC CATTCCAACG CACGCGCTAC CATTTTTTGG TTCACACCCG CATTGACCTG AATAAAATCG
53638        .......... .......... .......... .......... .......... .......... ..........
ATCC_8739    .......... .......... .......... .......... .......... .......... ..........
HS           .......... .......... .......... .......... .......... .......... ..........
BW2952       .......... .......... .......... .......... .......... .......... ..........
12009        .......... .......... .......... .......... .......... .......... ..........
B171         .......... .......... .......... .......... .......... .......... ..........
E24377A      .......... .......... .......... .......... .......... .......... ..........
SE11         .......... .......... .......... .......... .......... .......... ..........
11128        .......... .......... .......... .......-.. .......... .......... ..........
11368        .......... .......... .......... .......... .......... .......... ..........
IAI1         .......... .......... .......... .......... .......... .......... ..........
E110019      .......... .......... .......... .......... .......... .......... ..........
B7A          .......... .......... .......... .......... .......... .......... ..........
CB9615       ...C...... .......... .......... .......... .......... ....A..... ..........
O157_Sakai   ...C...... .......... .......... .......... .......... ....A..... ..........
IAI39        .......... .......... .......... .......... .......... .......... ..........
UMN026       .......... .......... .......... .......... .......... .......... ..........
SMS-3-5      T......... .......... .......... .......... .......... .......... ..........
536          .......... .......... .......... ......C... .......... .......... ..........
APEC_O1      .......... .......... .......... ......C... .......... .......... ..........
CFT073       .......... .......... .......... ......C... .......... .......... ..........
E2348/69     T......... .......... .......... .......... .......... .......... ..........
UTI89        .......... .......... .......... ......C... .......... .......... ..........
F11          .......... .......... .......... ......C... .......... .......... ..........
ED1a         .......... .......... .......... ......C... .......... .......... ..........
83972        .......... .......... .......... ......C... .......... .......... ..........
S88          .......... .......... .......... ......C... .......... .......... ..........
E._albertii  T......... .......G.. .CT....C.. .......... .....G..T. .G........ .........A
```

```
            2801
K-12         CGCGGGCTAA AAGTTAAGCG CAACCCGTTT GAGTCATACC AGGGCATCTC ACCAGAGACG GTTTCGAGTA
53638        .A........ .......... .......... .......... .......... .......... ..........
ATCC_8739    .......... .......... .......... .......... .......... .......... ..........
HS           .......... .......... .......... .......... .......... .......... ..........
BW2952       .......... .......... .......... .......... .......... .......... ..........
12009        .......... .......... .......... .......... .......... .......... ..........
B171         .......... .......... .......... .......... .......... .......... ..........
E24377A      .......... .......... .......... .......... .......... .......... ..........
SE11         .......... .......... .......... .......... .......... .......... ..........
11128        .......... .......... .......... .......... .......... .......... ..........
11368        .......... .......... .......... .......... .......... .......... ..........
IAI1         .......... .......... .......... .......... .......... .......... ..........
E110019      .......... .......... .......... .......... .......... .......... ..........
B7A          .......... .......... .......... .......... .......... .......... ..........
CB9615       .......... .......... .......... .......... .......... .......... ..........
O157_Sakai   .......... .......... .......... .......... .......... .......... ..........
IAI39        .......... .......... ......A... .......... .......... .......... ..........
UMN026       .......... .......... .......... .......... .......... .......... ..........
SMS-3-5      .......... .......... .......... .......... .......... .......... ..........
536          .......... .......... ......A... .......... .......... .......... ..........
APEC_O1      .......... .......... ......A... .......... .......... .......... ..........
CFT073       .......... .......... ......A... .......... .......... .......... ..........
E2348/69     .......... .......... .......... .......... .......... .......... ..........
UTI89        .......... .......... ......A... .......... .......... .......... ..........
F11          .......... .......... ......A... .......... .......... .......... ..........
ED1a         .......... .......... ......A... .......... .......... .......... ..........
83972        .......... .......... ......A... .......... .......... .......... ..........
S88          .......... .......... ......A... .......... .......... .......... ..........
E._albertii  .......... .G........ ......A... .CA....... .......... C.....T... ..........

            2871
K-12         TCTCACTATC GGGGGCGAGA TACAGATCCA GGCCTTCAGA ATGCGAAAAG CGTTCCAGTT TTTCGCGATC
53638        .......... .......... .......... .......... .......... .......... ..........
ATCC_8739    .......... .......... .......... .......... .......... .......... ..........
HS           .......... .......... .......... .......... .......... .......... ..........
BW2952       .......... .......... .......... .......... .......... .......... ..........
12009        .......... .......... .......... .......... .......... .......... ..........
B171         .......... .......... .......... .......... .......... .......... ..........
E24377A      .......... .......... .......... .......... .......... .......... ..........
SE11         .......... .......... .......... .......... .......... .......... ..........
11128        .......... .......... .......... .......... .......... .......... ..........
11368        .......... .......... .......... .......... .......... .......... ..........
IAI1         .......... .......... .......... .......... .......... .......... ..........
E110019      .......... .......... .......... .......... .......... .......... ..........
B7A          .......... .......... ......G... .......... .......... .......... ..........
CB9615       .......... .......... .......... .......... .......... .......... ..........
O157_Sakai   .......... .......... .......... .......... .......... .......... ..........
IAI39        .......... .......... .......... .......... .......... .A........ ..........
UMN026       .......... .......... .......... .......... .......... .......... ..........
SMS-3-5      .......... .......... .......... .......... .......... .......... ..........
536          .......... .......... .......... .......... .......... .A........ ..........
APEC_O1      .......... .......... .......... .......... .......... .A........ ..........
CFT073       .......... .......... .......... .......... .......... .A........ ..........
E2348/69     .......... .......... .......... .......... .......... .......... ..........
UTI89        .......... .......... .......... .......... .......... .A........ ..........
F11          .......... .......... .......... .......... .......... .A........ ..........
ED1a         .......... .......... .......... .......... .......... .A........ ..........
83972        .......... .......... .......... .......... .......... .A........ ..........
S88          .......... .......... .......... .......... .......... .A........ ..........
E._albertii  .......... A......... .......... .......... .......... .......... ..........
```

```
          2941
K-12         TGCCGAACTT AGCGGTGCGG TATGGCGCAA AATCATCAGC GTGCCGCTGG TTGCCTGTAC CAGTTCAACA
53638        .......... .......... .......... .......... .......... .......... ..........
ATCC_8739    .......... .......... .......... .......... .......... .......... ..........
HS           .......... .......... .......... .......... .......... .......... ..........
BW2952       .......... .......... .......... .......... .......... .......... ..........
12009        .......... .......... .......... .......... .......... .......... ..........
B171         .......... .......... .......... .......... .......... .......... ..........
E24377A      A......... .......... .......... .......... .......... .......... ..........
SE11         .......... .......... .......... .......... .......... .......... ..........
11128        .......... .......... .......... .......... .......... .......... ..........
11368        .......... .......... .......... .......... .......... .......... ..........
IAI1         .......... .......... .......... .......... .......... .......... ..........
E110019      A......... .......... .......... .......... .......... .......... ..........
B7A          A......... .......... .......... .......... .......... .......... ..........
CB9615       .......... .......... .......... .......... .......... .......... ..........
O157_Sakai   .......... .......... .......... .......... .......... .......... ..........
IAI39        .......... .......... .......... ......T... .......... .......... ..........
UMN026       .......... .......... .......... .......... .......... .......... ..........
SMS-3-5      .A........ .......... .......... .......... .......... .......... ..........
536          .......... .......... .......... ......T... .......... .......... ..........
APEC_O1      .......... .......... .......... ......T... .......... .......... ..........
CFT073       .......... .......... .......... ......T... .......... .......... ..........
E2348/69     A......... .......... .......... .......... .......... .......... ..........
UTI89        .......... .......... .......... ......T... .......... .......... ..........
F11          .......... .......... .......... ......T... .......... .......... ..........
ED1a         .......... .......... .......... ......T... .......... .......... ..........
83972        .......... .......... .......... ......T... .......... .......... ..........
S88          .......... .......... .......... ......T... .......... .......... ..........
E._albertii  A......T.G ........A. .......T.. .......... .......... .......C.. ...C......

          3011
K-12         TGACCAAGAT GGCGCATAGC TTGTAAGCTG CCCAGACATG CCCTGACTTT GGGCAGCAAT GCTTCAAGTT
53638        .......... .......... .......... .......... .......... .......... ..........
ATCC_8739    .......... ....T..... .......... .....G.... .......... .......... ..........
HS           .......... .......... .......... .......... .......... .......... ..........
BW2952       .......... .......... .......... .......... .......... .......... ..........
12009        .......... ........A. .......... .....G.... .......... .......... ..........
B171         .......... ........A. .......... .....G.... .......... .......... ..........
E24377A      .......... ....T..... .......... .....G.... .......... .......... ..........
SE11         .......... ........A. .......... .....G.... .......... .......... ..........
11128        .......... ........A. .......... .....G.... .......... .......... ..........
11368        .......... ........A. .......... .....G.... .......... .......... ..........
IAI1         .......... ........A. .......... .....G.... .......... .......... ..........
E110019      .......... ....T..... .......... .....G.... .......... .......... ..........
B7A          .......... ....T..... .......... .....G.... .......... .......... ..........
CB9615       .......... .......... .......... .....G.... .......... .......... ..........
O157_Sakai   .......... .......... .......... .....G.... .......... .......... ..........
IAI39        .......... .......... .......... .....G.... .......... .......... ..........
UMN026       .......... .......... .......... .....G.... .......... .......... ..........
SMS-3-5      .......... .......... .......... .....G.... .......... .......... ..........
536          .......... .......... .......... .....G.... .......... .......... ..........
APEC_O1      .......... .......... .......... .....G.... .......... .......... ..........
CFT073       .......... .......... .......... .....G.... .......... .......... ..........
E2348/69     .......... .......... .......... .....G.... .......... .......... ..........
UTI89        .......... .......... .......... .....G.... .......... .......... ..........
F11          .......... .......... .......... .....G.... .......... .......... ..........
ED1a         .......... .......... .......... .....G.... .......... .......... ..........
83972        .......... .......... .......... .....G.... .......... .......... ..........
S88          .......... .......... .......... .....G.... .......... .......... ..........
E._albertii  ..C....... .A..T..T.. ...C..A..C AT........ .......... .......... ..........
```

```
          3081
K-12         GGGGCGCTAA AATGGGGCAT TGTTTGACGT CGACAATGTC ACTGGAGCCC GCTTTGCGAA ACCCCATCTG
53638        .......... .......... .......... .......... .......... .......... ..........
ATCC_8739    .....A.... .......... .....A.... .......... .......... .......... .G........
HS           .......... .......... .......... .......... .......... .......... ..........
BW2952       .......... .......... .......... .......... .......... .......... ..........
12009        .....A.... .......... .....A.... .......... .......... .......... ..........
B171         .....A.... .......... .....A.... .......... .......... .......... ..........
E24377A      .....A.... .......... .....A.... .......... .......... .......... .G........
SE11         T....A.... .......... .....A.... .......... .......... .......... .G........
11128        .....A.... .......... .....A.... .......... .......... .......... ..........
11368        .....A.... .......... .....A.... .......... .......... .......... ..........
IAI1         T....A.... .......... .....A.... .......... .......... .......... .G........
E110019      .....A.... .......... .....A.... .......... .......... .......... .G........
B7A          .....A.... .......... .....A.... .......... .......... .......... .G........
CB9615       .....A.... ......A... .....A.... .......... .......... .......... ..........
O157_Sakai   .....A.... ......A... .....A.... .......... .......... .......... ..........
IAI39        .....A.... .......... .....A.... .......... .......... .......... ..........
UMN026       .....A.... ......A... .....A.... .......... .......... .......... ..........
SMS-3-5      .....A.... .......... .....A.... .......... .......... .......... ..........
536          .....A.... .......... .....C.... .......... .......... .......... ..........
APEC_O1      .....A.... .......... .....A.... .......... .......... .......... ..........
CFT073       .....A.... .......... .....C.... .......... .......... .......... ..........
E2348/69     .....A.... ......A... .....A.... .......... .......... .......... ..........
UTI89        .....A.... .......... .....A.... .......... .......... .......... ..........
F11          .....A.... .......... .....C.... .......... .......... .......... ..........
ED1a         .....A.... .......... .....C.... .......... .......... .......... ..........
83972        .....A.... .......... .....C.... .......... .......... .......... ..........
S88          .....A.... .......... .....A.... .......... .......... .......... ..........
E._albertii  .....A.... ...A...... .......TA. .A........ .....T...G .A...A.... .T......A.

          3151
K-12         AAGTTGCTGT GTTTTCGGTA AGTAGTTCAG ACTTAAACGC GCGCGACGGC GATAGCCCCA GGGAACATCG
53638        .......... .......... .......... .......... .......... .......... ..........
ATCC_8739    .......... .......... .........A .......... .......... .......... ..........
HS           .......... .......... .......... .......... .......... .......... ..........
BW2952       .......... .......... .......... .......... .......... .......... ..........
12009        .......... .......... .......... .......... .....G.... .......... ..........
B171         .......... .......... .......... .......... .....G.... .......... ..........
E24377A      .......... .......... .........A .......... .......... .......... ..........
SE11         .......... .......... .........A .......... .......... .......... ..........
11128        .......... .......... .......... .......... .....G.... .......... ..........
11368        .......... .......... .......... .......... .....G.... .......... ..........
IAI1         .......... .......... .........A .......... .......... .......... ..........
E110019      .......... .......... .........A .......... .......... .......... ..........
B7A          .......... .......... .........A .......... .......... .......... ..........
CB9615       .......... .......... .......... .......... .......... .......... ..........
O157_Sakai   .......... .......... .......... .......... .......... .......... ..........
IAI39        .......... .......... .......... .......... .......... .......... ..........
UMN026       .......... .......... .........A .......... .......... .......... ..........
SMS-3-5      .......... .......... .........A .......... .......... .......... ..........
536          .......... .......... .......... .......... .......... .......... ..........
APEC_O1      .......... .......... .......... .......... .......... .......... ..........
CFT073       .......... .......... .......... .......... .......... .......... ..........
E2348/69     .......... .......... .......... .......... .......... .......... ..........
UTI89        .......... .......... .......... .......... .......... .......... ..........
F11          .......... .......... .......... .......... .......... .......... ..........
ED1a         .......... .......... .......... .......... .......... .......... ..........
83972        .......... .......... .......... .......... .......... .......... ..........
S88          .......... .......... .......... .......... .......... .......... ..........
E._albertii  .........C ........C. GA..A..... .......... ..A..G..T. .G........ ...GGT...C
```

```
          3221
K-12         GCGATCACTT CAGAGACATC GTGTTTCATT AATCGGGCGA GTGCCGCACT TTTGCTTCGC TGCTGTAAAT
53638        .......... .......... .......... .......... .......... .......... ..........
ATCC_8739    .......... .......T.. .......... .......... .......... .......... ..........
HS           .......... .......... .......... .......... .......... .......... ..........
BW2952       .......... .......... .......... .......... .......... .......... ..........
12009        .......... .......... .......... .......... .......... .......... ..........
B171         .......... .......... .......... .......... .......... .......... ..........
E24377A      .......... .......T.. .......... .......... .......... .......... ..........
SE11         .......... .......T.. .......... .......... .......... .......... ..........
11128        .......... .......... .......... .......... .......... .......... ..........
11368        .......... .......... .......... .......... .......... .......... ..........
IAI1         .......... .......T.. .......... .......... .......... .......... ..........
E110019      .......... .......T.. .......... .......... .......... .......... ..........
B7A          .......... .......T.. .......... .......... .......... .......... ..........
CB9615       .......... .......T.. .......... .......... .......... .......... ..........
O157_Sakai   .......... .......T.. .......... .......... .......... .......... ..........
IAI39        .......... .......T.. .......... .......... .......... .......... ..........
UMN026       .......... .......C.. .......... .......... .......... .......... ..........
SMS-3-5      .......... .......T.. .......... .......... .......... .......... ..........
536          .......... .......T.. .......... .......... .......... .......... ..........
APEC_O1      .......... .......T.. .......... .......... .......... .......... ..........
CFT073       .......... .......T.. .......... .......... .......... .......... ..........
E2348/69     .......... .......T.. .......... .......... .......... .......... ..........
UTI89        .......... .......T.. .......... .......... .......... .......... ..........
F11          .......... .......T.. .......... .......... .......... .......... ..........
ED1a         .......... .......T.. .......... .......... .......... .......... ..........
83972        .......... .......T.. .......... .......... .......... .......... ..........
S88          .......... .......T.. .......... .......... .......... .......... ..........
E._albertii  .....A..A. ....A..G.. .......... ........A. .C.....G.. ......A..T ........C.

          3291
K-12         CCACGCTGGC GTGTTGTTGC TGACAGCCAC CGCATACGCC AAAATGAGGA CAGCGTGGCG TTTCGCGTTC
53638        .......... .......... .......... .......... .......... .......... ..........
ATCC_8739    .......... .......... .......... .......... .........G .......... ..........
HS           .......... .......... .......... .......... .......... .......... ..........
BW2952       .......... .......... .......... .......... .......... .......... ..........
12009        .......... .......... ........G. .......... .......... ........T. ..........
B171         .......... .......... ........G. .......... .......... ........T. ..........
E24377A      .......... .......... .......... .......... .........G .......... ..........
SE11         .......... .......... .......... .......... .....A...G .......... ..........
11128        .T........ .......... ........G. .......... .......... ........T. ..........
11368        .......... .......... ........G. .......... .......... ........T. ..........
IAI1         .......... .......... .......... .......... .........G .......... ..........
E110019      .......... .......... .......... .......... .........G .......... ..........
B7A          .......... .......... .......... .......... .........G .......... ..........
CB9615       .......... .......... ........G. .......... .......... .......... ..........
O157_Sakai   .......... .......... .......TG. .......... .......... .......... ..........
IAI39        ..T....... .......... .......... .......... .........G .......... ..........
UMN026       ..T....... .......... ........G. .......... .........G .......... ..........
SMS-3-5      ..T....... .......... .......... .......... .........G .......... ..........
536          ....A..... .......... ........G. .......... .......... .......... ..........
APEC_O1      ....A..... .......... ........G. .......... .......... .......... ..........
CFT073       ....A..... .......... ........G. .......... .......... .......... ..........
E2348/69     .......... .......... ........G. .......... .......... .......... ..........
UTI89        ....A..... .......... ........G. .......... .......... .......... ..........
F11          ....A..... .......... ........G. .......... .......... .......... ..........
ED1a         ....A..... .......... ........G. .......... .......... .......... ..........
83972        ....A..... .......... ........G. .......... .......... .......... ..........
S88          ....A..... .......... ........G. .......... .......... .......... ..........
E._albertii  .A........ .......... ..G.....G. .......... .......... .....C.... ....A..C..
```

```
           3361
K-12         CGGGCTATCG CTTAACCGGC GTACGACTTT AGCGCGGGCA TACTGTTTTT TATCTTCAGT AACAGTAACT
53638        .......... .......... .......... .......... .......... .......... ..........
ATCC_8739    .......... .......... .C........ .........G .......... .......... ..........
HS           .......... .......... .......... .......... .......... .......... ..........
BW2952       .......... .......... .......... .......... .......... .......... ..........
12009        .......... .......... .......... .......... .......... .......... .....C....
B171         .......... .......... .......... .......... .......... .......... .....C....
E24377A      .......... .......... .C........ .........G .......... .......... ..........
SE11         .......... .......... .C........ .........G .......... .......... ..........
11128        .......... .......... .......... .......... .......... .......... .....C....
11368        .......... .......... .......... .......... .......... .......... .....C....
IAI1         .......... .......... .C........ .........G .......... .......... ..........
E110019      .......... .......... .C........ .........G .......... .......... ..........
B7A          .......... .......... .C........ .........G .......... .......... ..........
CB9615       .......... .......... .......... .......... .......... ....C..... ..........
O157_Sakai   .......... .......... .......... .......... .......... ....C..... ..........
IAI39        .......... .......... .......... .......... .......... .......... ..........
UMN026       .......... .......... .......... .........G ..T....... .......... ..........
SMS-3-5      .......... .......... .......... .........G .......... .......... ..........
536          .......... ........A. .......... .......... .......... .......... ..........
APEC_O1      .......... .....T.... .......... .......... .......... .......... ..........
CFT073       .......... .......... .......... .......... .......... .......... ..........
E2348/69     .......... .......... .C........ .........G ..T....... .......... ..........
UTI89        .......... .....T.... .......... .......... .......... .......... ..........
F11          .......... ........A. .......... .......... .......... .......... ..........
ED1a         .......... ........A. .......... .......... .......... .......... ..........
83972        .......... .......... .......... .......... .......... .......... ..........
S88          .......... .....T.... .......... .......... .......... .......... ..........
E._albertii  T.....G... .......... .C........ ......A... .......... .......T.. ..........

           3431
K-12         TCCGCGTTTT CCTGCGGCAA TAATCCGGGG ATAAATAGCG TTTTGCCGTT ATGTCGCGCC ACGCCCTGAC
53638        .......... .......... .......... .......... .......... .......... ..........
ATCC_8739    .......... ....T....G ......A..A .......A.. C......... .......... ..........
HS           .......... .......... .......... .......... .......... .......... ..........
BW2952       .......... .......... .......... .......... .......... .......... ..........
12009        .......... .......... .......... .......A.. .......... .......... ..........
B171         .......... .......... .......... .......A.. .......... .......... ..........
E24377A      .......... ....T....G ......A..A .......A.. C......... .......... ..........
SE11         .......... ....T....G ......A..A .......A.. C......... .......... ..........
11128        .......... .......... .......... .......A.. .......... .......... ..........
11368        .......... .......... .......... .......A.. .......... .......... ..........
IAI1         .......... ....T....G ......A..A .......A.. C......... .......... ..........
E110019      .......... ....T....G ......A..A .......A.. C......... .......... ..........
B7A          .......... ....T....G ......A..A .......A.. C......... .......... ..........
CB9615       .......... .......... .......... .......... .......... .......... ..........
O157_Sakai   .......... .......... .......... .......... .......... .......... ..........
IAI39        .......... .......... .......... .....C.A.. .......... .......... ..........
UMN026       .......... ....T....G ......A..A .......A.. C......... ......T... ..........
SMS-3-5      .......... ....T..... ......A..A .......A.. .......... .......... ..........
536          .......... .......... .......... .......AT. .......... .......... ..A.......
APEC_O1      .......... .......... .......... .......AT. .......... .......... ..A.......
CFT073       .......... .......... .......... .......AT. .......... .......... ..A.......
E2348/69     .......... ....T..... ......A..A .......A.. C......... ......T... ..........
UTI89        .......... .......... .......... .......AT. .......... .......... ..A.......
F11          .......... .......... .......... .......AT. .......... .......... ..A.......
ED1a         .......... .......... .......... .....C.A.. .......... .......... ..........
83972        .......... .......... .......... .......AT. .......... .......... ..A.......
S88          .......... .......... .......... .......AT. .......... .......... ..A.......
E._albertii  .......... .T.....T.. ......A... .......A.. ....T..... ...C...... ..A.......
```

```
            3501
K-12         CAAAAGAGTC GAGGTCGTTG ACTGAAACGG TTATGATCTG ACGCGTCGTC GTGCGTCGTT TTGCAGAGTA
53638        .......... .......... .......... .......... .......... .......... ..........
ATCC_8739    .......... .......... .......... .......... .......... .......... ..........
HS           .......... .......... .......... .......... .......... .......... ..........
BW2952       .......... .......... .......... .......... .......... .......... ..........
12009        .......... .......... .......... .......... .......... .......... ..........
B171         .......... .......... .......... .......... .......... .......... ..........
E24377A      .......... .......... .......... .......... .......... .......... ..........
SE11         .......... .......... .......... .......... .......... .......... ..........
11128        .......... .......... .......... .......... .......... .......... ..........
11368        .......... .......... .......... .......... .......... .......... ..........
IAI1         .......... .......... .......... .......... .......... .......... ..........
E110019      .......... .......... .......... .......... .......... .......... ..........
B7A          .......... .......... .......... .......... .......... .......... ..........
CB9615       .......... .......... .......... .......... .......... .......... ..........
O157_Sakai   .......... .......... .......... .......... .......... .......... ..........
IAI39        .......... .......... .......... .......... .......... .......... ..........
UMN026       .......... .......... ........A. .......... .......... .......... ..........
SMS-3-5      .......... ......A... .......... .......... .......... .......... ..........
536          .......... .......... .......... .......... .......... .......... ..........
APEC_O1      .......... .......... .......... .......... .......... .......... ..........
CFT073       .......... .......... .......... .......... .......... .......... ..........
E2348/69     .......... .......... ........A. .......... .......... .......... ..........
UTI89        .......... .......... .......... .......... .......... .......... ..........
F11          .......... .......... .......... .......... .......... .......... ..........
ED1a         .......... .......... .......... .......... .......... .......... ..........
83972        .......... .......... .......... .......... .......... .......... ..........
S88          .......... .......... .......... .......... .......... .......... ..........
E._albertii  .......... ......A... .......... .......... .......... AC...C.... ..........

            3571
K-12         GAATTGCGCC ATTGGCGAGA CTTTCTCAAT TTAACAGTGT GACCTTAATT GTCCCATAAC GGAACTCCAT
53638        .......... .......... .......... .......... .......... .......... ..........
ATCC_8739    .......... .......... .......... .......... .......... .......... ..........
HS           .......... .......... .......... .......... .......... .......... ..........
BW2952       .......... .......... .......... .......... .......... .......... ..........
12009        .......... .......... .......... .......... .......... .......... ..........
B171         .......... .......... .......... .......... .......... .......... ..........
E24377A      .......... .......... .......... .......... .......... .......... ..........
SE11         .......... .......... .......... .......... .......... .......... ..........
11128        .......... .......... .......... .......... .......... .......... ..........
11368        .......... .......... .......... .......... .......... .......... ..........
IAI1         .......... .......... .......... .......... .......... .......... ..........
E110019      .......... .......... .......... .......... .......... .......... ..........
B7A          .......... .......... .......... .......... .......... .......... ..........
CB9615       .......... .......... .......... .......... .......... .......... ..........
O157_Sakai   .......... .......... .......... .......... .......... .......... ..........
IAI39        .......... .......... .......... .......... .......... .......... ..........
UMN026       .......... .......... .......... .......... .......... .......... ..........
SMS-3-5      .......... .......... .......... .......... .......... .......... ..........
536          .......... .......... .......... .......... .......... .......... ..........
APEC_O1      .......... .......... .......... .......... .......... .......... ..........
CFT073       .......... .......... .......... .......... .......... .......... ..........
E2348/69     .......... .......... .......... .......... .......... .......... ..........
UTI89        .......... .......... .......... .......... .......... .......... ..........
F11          .......... .......... .......... .......... .......... .......... ..........
ED1a         .......... .......... .......... .......... .......... .......... ..........
83972        .......... .......... .......... .......... .......... .......... ..........
S88          .......... .......... .......... .......... .......... .......... ..........
E._albertii  .......... .......... .......... .......... .......... .......... ..........
```

```
           3641
K-12           GACCAACTAC AGCCTGCGCG CACGCATGAT GATTCTGATC CTGGCACCGA CCGTCCTTAT TGGTTTATTG
53638          .......... .......... .......... .......... .......... .......... ..........
ATCC_8739      .......... .......... .......... .......... .......... .......... ..........
HS             .......... .......... .......... .......... .......... .......... ..........
BW2952         .......... .......... .......... .......... .......... .......... ..........
12009          .......... .......... .......... .......... .......... .......... ..........
B171           .......... .......... .......... .......... .......... .......... ..........
E24377A        .......... .......... .......... .......... .......... .......... ..........
SE11           .......... .......... .......... .......... .......... .......... ..........
11128          .......... .......... .......... .......... .......... .......... ..........
11368          .......... .......... .......... .......... .......... .......... ..........
IAI1           .......... .......... .......... .......... .......... .......... ..........
E110019        .......... .......... .......... .......... .......... .......... ..........
B7A            .......... .......... .......... .......... .....G.... .......... ..........
CB9615         .......... .......... .......... .......... .......... .......... ..........
O157_Sakai     .......... .......... .......... .......... .......... .......... ..........
IAI39          .......... .......... .......... .......... .......... .......... ..........
UMN026         .......... .......... .......... .......... .......... .......... ..........
SMS-3-5        .......... .......... .......... .......... .......... .......... ..........
536            .......... .......... .......... .......... .......... .......... ..........
APEC_O1        .......... .......... .......... .......... .......... .......... ..........
CFT073         .......... .......... .......... .......... .......... .......... ..........
E2348/69       .......... .......... .......... .......... .......... .......... ..........
UTI89          .......... .......... .......... .......... .......... .......... ..........
F11            .......... .......... .......... .......... .......... .......... ..........
ED1a           .......... .......... .......... .......... .......... .......... ..........
83972          .......... .......... .......... .......... .......... .......... ..........
S88            .......... .......... .......... .......... .......... .......... ..........
E._albertii    .......... .......... .......... .......... .....G.... .......... ...G......

           3711
K-12           CTGAGTATCT TTTTCGTCGT GCATCGCTAT AACGACTTGC AGCGTCAACT GGAAGATGCC GGTGCCAGCA
53638          .......... .......... .......... .......... .......... .......... ..........
ATCC_8739      .......... .......... .......... .......... .......... .......... ..........
HS             .......... .......... .......... .......... .......... .......... ..........
BW2952         .......... .......... .......... .......... .......... .......... ..........
12009          .......... .......... .......... .......... .......... .......... ..........
B171           .......... .......... .......... .......... .......... .......... ..........
E24377A        .......... .......... .......... .......... .......... .......... ..........
SE11           .......... .......... .......... .......... .......... .......... ..........
11128          .......... .......... .......... .......... .......... .......... ..........
11368          .......... .......... .......... .......... .......... .......... ..........
IAI1           .......... .......... .......... .......... .......... .......... ..........
E110019        .......... .......... .......... .......... .......... .......... ..........
B7A            .......... .......... .......... .......... .......... .......... ..........
CB9615         .......... .......... .......... .......... .......... .......... ..........
O157_Sakai     .......... .......... .......... .......... .......... .......... ..........
IAI39          .......... .......... .......... .......... .......... .......... ..........
UMN026         .......... .......T.. .......... .......... .......... .......... ..A.......
SMS-3-5        .......... .......... .......... .......... .......... .......... ..........
536            .......... .......... .......... .......... .......... .......... ........T.
APEC_O1        .......... .......... .......... .......... .......... .......... ..........
CFT073         .......... .......... .......... .......... .......... .......... ..........
E2348/69       .......... .......T.. .......... .......... .......... .......... ..A.......
UTI89          .......... .......... .......... .......... .......... .......... ..........
F11            .......... .......... .......... .......... .......... .......... ........T.
ED1a           .......... .......... .......... .......... .......... .......... ........T.
83972          .......... .......... .......... .......... .......... .......... ..........
S88            .......... .......... .......... .......... .......... .......... ..........
E._albertii    ..T....... .......... .......... .......... .......... .......... ..C.....T.
```

```
            3781
K-12         TTATTGAGCC GCTTGCAGTT TCTACTGAAT ATGGCATGAG CCTGCAAAAT CGCGAATCTA TCGGTCAGTT
53638        .......... .......... .......... .......... .......... .......... ..........
ATCC_8739    .......... .......... .......... .......... .......... .......... ..........
HS           .......... .......... .......... .......... .......... .......... ..........
BW2952       .......... .......... .......... .......... .......... .......... ..........
12009        .......... .......... .......... .......... .......... .......... ..........
B171         .......... .......... .......... .......... .......... .......... ..........
E24377A      .......... .......... .......... .......... .......... .......... ..........
SE11         .......... .......... .......... .......... .......... .......... ..........
11128        .......... .......... .......... .......... .......... .......... ..........
11368        .......... .......... .......... .......... .......... .......... ..........
IAI1         .......... .......... .......... .......... .......... .......... ..........
E110019      .......... .......... .......... .......... .......... .......... ..........
B7A          .......... .......... .......... .......... .......... .......... ..........
CB9615       .......... .......... .......... .......... .......... .......... ..........
O157_Sakai   .......... .......... .......... .......... .......... .......... ..........
IAI39        .......... .......... .......... .......... T......... .......... ..........
UMN026       .......... .......... .......... .......... .......... .......... ..........
SMS-3-5      .......... .......... .......... .......... T......... .......... ..........
536          .......... .......... .......... .......... .......... .......... ..........
APEC_O1      .......... .......... .......... .......... T......... .......... ..........
CFT073       .......... .......... .......... .......... T......... .......... ..........
E2348/69     .......... .......... .......... .......... .........C .......... ..........
UTI89        .......... .......... .......... .......... T......... .......... ..........
F11          .......... .......... .......... .......... .......... .......... ..........
ED1a         .......... .......... .......... .......... .......... .......... ..........
83972        .......... .......... .......... .......... T......... .......... ..........
S88          .......... .......... .......... .......... T......... .......... ..........
E._albertii  .......A.. ...G..G... .....C.... .......... T......... .......... ....G..A..

            3851
K-12         AATAAGCGTA CTGCATCGTC GCCATTCCGA TATTGTTCGC GCGATTTCGG TTTATGATGA AAATAACCGA
53638        .......... .......... .......... .......... .......... .......... ..........
ATCC_8739    .......... .......... .......... .......... .......... .......... ..........
HS           .......... .......... .......... .......... .......... .......... ..........
BW2952       .......... .......... .......... .......... .......... .......... ..........
12009        .......... .......... .......... .......... .......... .......... ..........
B171         .......... .......... .......... .......... .......... .......... ..........
E24377A      .......... .......... .......... .......... .......... .......... ..........
SE11         .......... .......... .......... .......... .......... .......... ..........
11128        .......... .......... .......... .......... .......... .......... ..........
11368        .......... .......... .......... .......... .......... .......... ..........
IAI1         .......... .......... .......... .......... .......... .......... ..........
E110019      .......... .......... .......... .......... .......... .......... ..........
B7A          .......... .......... .......... .......... .......... .......... ..........
CB9615       .......... .......... .......... .......... .......... .......... ..........
O157_Sakai   .......... .......... .......... .......... .......... .......... ..........
IAI39        .......... .......... .......... .......... .......... .......... ..........
UMN026       .......... .......... .......... .......... .......... .......... ..........
SMS-3-5      .......... .......... .......T.. .......... .......... .......... ..........
536          .......... .......... .......... .......... .......... .......... ..........
APEC_O1      .......... .......... .......T.. .......... .......... .......... ..........
CFT073       .......... .......... .......T.. .......... .......... .......... ..........
E2348/69     .......... .......... .......... .......... .......... .......... ..........
UTI89        .......... .......... .......T.. .......... .......... .......... ..........
F11          .......... .......... .......... .......... .......... .......... ..........
ED1a         .......... .......... .......... .......... ..A....... .......... ..........
83972        .......... .......... .......T.. .......... .......... .......... ..........
S88          .......... .......... .......T.. .......... .......... .......... ..........
E._albertii  G........T .......... .......... ......G... ..T....... .......C.. ........AC
```

```
             3921
K-12         CTCTTTGTCA CCTCCAATTT TCATCTTGAT CCCTCATCAA TGCAGCTCGG CAGCAACGTG CCGTTTCCTC
53638        .......... .......... .......... .......... .......... .......... ..........
ATCC_8739    .......... .......... .......... .......... .......... .......... ..........
HS           .......... .......... .......... .......... .......... .......... ..........
BW2952       .......... .......... .......... .......... .......... .......... ..........
12009        .......... .......... .......... .......... .......... .......... ..........
B171         .......... .......... .......... .......... .......... .......... ..........
E24377A      .......... .......... .......... .......... .......... .......... ..........
SE11         .......... .......... .......... .......... .......... .......... ..........
11128        .......... .......... .......... .......... .......... .......... ..........
11368        .......... .......... .......... .......... .......... .......... ..........
IAI1         .......... .......... .......... .......... .......... .......... ..........
E110019      .......... .......... .......... .......... .......... .......... ..........
B7A          .......... .......... .......... .......... .......... .......... ..........
CB9615       .......... .......... .......... .......... .......... .......... ..........
O157_Sakai   .......... .......... .......... .......... .......... .......... ..........
IAI39        .......... .......... .......... .......... .......... .......... ..........
UMN026       .......... .T........ .......... .......... .......... .......... ..........
SMS-3-5      .......... .......... .......... .......... .......... .......... ..........
536          .......... .......... .......... .......... .......... .......... ..........
APEC_O1      .......... .......... .......... .......... .......... .......... ..........
CFT073       .......... .......... .......... .......... .......... .......... ..........
E2348/69     .......... .......... .......... .......... .......... .......... ..........
UTI89        .......... .......... .......... .......... .......... .......... ..........
F11          .......... .......... .......... .......... .......... .......... ..........
ED1a         .......... .......... .......... .......... .......... .......... ..........
83972        .......... .......... .......... .......... .......... .......... ..........
S88          .......... .......... .......... .......... .......... .......... ..........
E._albertii  ..G....... .T........ ...A.....C ..T..GGT.. .........A ......T..A .....C..A.

             3991
K-12         GCCAGCTCAC TGTCACTCGT GACGGCGATA TTATGATCCT CCGCACGCCG ATTATTTCTG AGAGTTACTC
53638        .......... .......... .......... .......... .......... .......... ..........
ATCC_8739    .......... .......... .......... .......... .......... .......... ..........
HS           .......... .......... .......... .......... .......... .......... ..........
BW2952       .......... .......... .......... .......... .......... .......... ..........
12009        .......... .......... .......... .......... .......... .......... ..........
B171         .......... .......... .......... .......... .......... .......... ..........
E24377A      .......... .......... .......... .......... .......... .......... ..........
SE11         .......... .......... .......... .......... .......... .......... ..........
11128        .......... .......... .......... .......... .......... .......... ..........
11368        .......... .......... .......... .......... .......... .......... ..........
IAI1         .......... .......... .......... .......... .......... .......... ..........
E110019      .......... .......... .......... .......... .......... .......... ..........
B7A          .......... .......... .......... .......... .......... .......... ..........
CB9615       .......... .......... .......... .......... .......... .......... ..........
O157_Sakai   .......... .......... .......... .......... .......... .......... ..........
IAI39        .......... .......... .......... .A........ .......... .......... .A........
UMN026       .......... .......... .......... .......... .......... .......... ..........
SMS-3-5      .......... .......... .......... .A........ .......... .......... .A........
536          .......... .......... .......... .......... .......... .......... .A........
APEC_O1      .......... .......... .......... .A........ .......... .......... .A........
CFT073       .......... .......... .......... .A........ .......... .......... .A........
E2348/69     .......... ......C... .......... .......... .......... .......... ..........
UTI89        .......... .......... .......... .A........ .......... .......... .A........
F11          .......... .......... .......... .......... .......... .......... .A........
ED1a         .......... .......... .......... .......... .......... .......... .A........
83972        .......... .......... .......... .A........ .......... .......... .A........
S88          .......... .......... .......... .A........ .......... .......... .A........
E._albertii  .......... C.....A..C .......... .......... G........A .......... .......T..
```

```
             4061
K-12         CCCCGACGAA TCGCCCAGTA GCGATGCCAA AAATAGTCAA AATATGTTGG GATATATTGC GCTGGAGCTG
53638        .......... .......... .......... .......... .......... .......... ..........
ATCC_8739    .......... .......... .......... .......... .......... .......... ..........
HS           .......... .......... .......... .......... .......... .......... ..........
BW2952       .......... .......... .......... .......... .......... .......... ..........
12009        .......... .......... .......... .......... .......... .......... ..........
B171         .......... .......... .......... .......... .......... .......... ..........
E24377A      .......... .......... .......... .......... .......... .......... ...T......
SE11         .......... .......... .......... .......... .......... .......... ...T......
11128        .......... .......... .......... .......... .......... .......... ..........
11368        .......... .......... .......... .......... .......... .......... ..........
IAI1         .......... .......... .......... .......... .......... .......... ...T......
E110019      .......... .......... .......... .......... .......... .......... ...T......
B7A          .......... .......... .......... .......... .......... .......... ...T......
CB9615       .......... .......... .......... .......... .......... .......... ...T......
O157_Sakai   .......... .......... .......... .......... .......... .......... ...T......
IAI39        .......... .......... .......... .......... ......C... .......... ..........
UMN026       .......... .......... .......... .......... ......C... .......... ..........
SMS-3-5      .......... .......... .......... .......... ......C... .......... ..........
536          .......... .......... .......... .......... ......C... .......... ..........
APEC_O1      .......... .......... .......... .......... ......C... .......... ..........
CFT073       .......... .......... .......... .......... ......C... .......... ..........
E2348/69     .......... .......... .......... .......... ......C... .......... ..........
UTI89        .......... .......... .......... .......... ......C... .......... ..........
F11          .......... .......... .......... .......... ......C... .......... ..........
ED1a         .......... .......... .......... .......... ......C... .......... ..........
83972        .......... .......... .......... .......... ......C... .......... ..........
S88          .......... .......... .......... .......... ......C... .......... ..........
E._albertii  T......... ..C.....C. .......T.. .........G .......... .......... C..T..A...

             4131
K-12         GATCTTAAAT CGGTTCGCTT GCAGCAATAT AAAGAGATCT TTATTTCCAG CGTGATGATG CTGTTTTGTA
53638        .......... .......... .......... .......... .......... .......... ..........
ATCC_8739    .......... .......... .......... .......... .......... .......... ..........
HS           .......... .......... .......... .......... .......... .......... ..........
BW2952       .......... .......... .......... .......... .......... .......... ..........
12009        .......... .......... .......... .......... .......... .......... ..........
B171         .......... .......... .......... .......... .......... .......... ..........
E24377A      .......... .......... .......... .......... .......... .......... ..........
SE11         .......... .......... .......... .......... .......... .......... ..........
11128        .......... .......... .......... .......... .......... .......... ..........
11368        .......... .......... .......... .......... .......... .......... ..........
IAI1         .......... .......... .......... .......... .......... .......... ..........
E110019      .......... .......... .......... .......... .......... .......... ..........
B7A          .......... .......... .......... .......... .......... .......... ..........
CB9615       .......... .......... .......... .......... .......... .......... ..........
O157_Sakai   .......... .......... .......... .......... .......... .......... ..........
IAI39        .......... .......... .......... .......... .......... .......... ..........
UMN026       .......... .......... .......... .......... .......... .......... ..........
SMS-3-5      .......... .......... .......... .......... .......... .......... ..........
536          .......... .......... .......... .......... .......... .......... ..........
APEC_O1      .......... .......... .......... .......... .......... .......... ..........
CFT073       .......... .......... .......... .......... .......... .......... ..........
E2348/69     .......... .......... .......... .......... .......... .......... ..........
UTI89        .......... .......... .......... .......... .......... .......... ..........
F11          .......... .......... .......... .......... .......... .......... ..........
ED1a         .......... .......... .......... .......... .......... .......... ..........
83972        .......... .......... .......... .......... .......... .......... ..........
S88          .......... .......... .......... .......... .......... .......... ..........
E._albertii  .....G.... ....A..GC. A......... .......... .C........ .......... ..........
```

```
          4201
K-12         TCGGTATTGC GCTTATTTTT GGCTGGCGCT TAATGCGCGA TGTAACCGGT CCGATTCGCA ACATGGTGAA
53638        .......... .......... .......... .......... .......... .......... ..........
ATCC_8739    .......... .......... .......... .......... .......... .......... ..........
HS           .......... .......... .......... .......... .......... .......... ..........
BW2952       .......... .......... .......... .......... .......... .......... ..........
12009        .......... .......... .......... .......... .......... .......... ..........
B171         .......... .......... .......... .......... .......... .......... ..........
E24377A      .......... .......... .......... .......... .......... .......... ..........
SE11         .......... .......... .......... .......... .......... .......... ..........
11128        .......... .......... .......... .......... .......... .......... ..........
11368        .......... .......... .......... .......... .......... .......... ..........
IAI1         .......... .......... .......... .......... .......... .......... ..........
E110019      .......... .......... .......... .......... .......... .......... ..........
B7A          .......... .......... .......... .......... .......... .......... ..........
CB9615       .......... .......... .......... .......... .......... .......... ..........
O157_Sakai   .......... .......... .......... .......... .......... .......... ..........
IAI39        .......... .......... .......... .......... ...T.....C .......... ..........
UMN026       .......... .......... .......... .......... .......... .......... ..........
SMS-3-5      .......... .......... .......... .......... ...T.....C .......... ..........
536          .......... .......... .......... .......... .......... .......... ..........
APEC_O1      .......... .......... .......... .......... .......... .......... ..........
CFT073       .......... .......... .......... .......... .......... .......... ..........
E2348/69     .......... .......... .......... .......... .......... .......... .T........
UTI89        .......... .......... .......... .......... .......... .......... ..........
F11          .......... .......... .......... .......... .......... .......... ..........
ED1a         .......... .......... .......... .......... .......... .......... ..........
83972        .......... .......... .......... .......... .......... .......... ..........
S88          .......... .......... .......... .......... .......... .......... ..........
E._albertii  .T........ .......... .......... .......T.. .........C .......... ..........

          4271
K-12         TACCGTCGAC CGCATCCGTC GCGGGCAACT CGACAGCCGG GTGGAAGGAT TTATGCTCGG CGAGCTGGAT
53638        .......... .......... .......... .......... .......... .......... ..........
ATCC_8739    .......... .......... .......... .......... .......... .......... ..........
HS           .......... .......... .......... .......... .......... .......... T.........
BW2952       .......... .......... .......... .......... .......... .......... ..........
12009        .......... .......... .......... .......... .......... .......... ..........
B171         .......... .......... .......... .......... .......... .......... ..........
E24377A      .......... .......... .......... .......... .......... .......... ..........
SE11         .......... .......... .......... .......... .......... .......... ..........
11128        .......... .......... .......... .......... .......... .......... ..........
11368        .......... .......... .......... .......... .......... .......... ..........
IAI1         .......... .......... .......... .......... .......... .......... ..........
E110019      .......... .......... .......... .......... .......... .......... ..........
B7A          .......... .......... .......... .......... .......... .......... ..........
CB9615       ...T...... .......... .......... .........A .......... .......... ..........
O157_Sakai   ...T...... .......... .......... .........A .......... .......... ..........
IAI39        .......... .......... .T........ ...T...... .......... .......T.. T.........
UMN026       .......... .......... .......... .......... .......... .......T.. T.........
SMS-3-5      .......... .......... .......... .......... .......... .......... ..........
536          .......... .......... .......... .......... .......... .......... ..........
APEC_O1      .......... .......... .......... .......... ........T. .......... ...A......
CFT073       .......... .......... .......... .......... .......... .......... ..........
E2348/69     .......... .......... .......... .........T .......... .......... ..........
UTI89        .......... .......... .......... .......... ........T. .......... ...A......
F11          .......... .......... .......... .......... .......... .......... ..........
ED1a         .......... .......... .......... .......... .......... .......... ..........
83972        .......... .......... .......... .......... .......... .......... ..........
S88          .......... .......... .......... .......... ........T. .......... ...A......
E._albertii  .......... .....T..C. .T........ ...T.....C ..A.....T. .......... ...A......
```

```
          4341
K-12         ATGCTGAAAA ACGGTATCAA CTCGATGGCA ATGTCGCTGG CTGCTTATCA CGAAGAGATG CAGCACAATA
53638        .......... .......... .......... .......... .......... .......... ..........
ATCC_8739    .......... .......... .......... .......... .......... .......... ..........
HS           .......... .......... .......... .......... .......... .......... ..........
BW2952       .......... .......... .......... .......... .......... .......... ..........
12009        .......... .......... .......... .......... .......... .......... ..........
B171         .......... .......... .......... .......... .......... .......... ..........
E24377A      .......... .......... .......... .......... .......... .......... ..........
SE11         .......... .......... .......... .......... .......... .......... ..........
11128        .......... .......... .......... .......... .......... .......... ..........
11368        .......... .......... .......... .......... .......... .......... ..........
IAI1         .......... .......... .......... .......... .......... .......... ..........
E110019      .......... .......... .......... .......... .......... .......... ..........
B7A          .......... .......... .......... .......... .......... .......... ..........
CB9615       .......... .......... .......... .......... .......... .......... ..........
O157_Sakai   .......... .......... .......... .......... .......... .......... ..........
IAI39        .......... .......... .......... .......... .G........ .......... ..A.......
UMN026       .......... .......... .......... .......... .......... .......... ..........
SMS-3-5      .......... .......... .......... .......... .G........ .......... ..A.......
536          .......... .......... .......... .......... .G........ .......... ..A.......
APEC_O1      .......... .......... .......... .......... .G........ .......... ..A.......
CFT073       .......... .......... .......... .......... .G........ .......... ..A.......
E2348/69     .......... .......... .......... .......... .......... .......... ..........
UTI89        .......... .......... .......... .......... .G........ .......... ..A.......
F11          .......... .......... .......... .......... .G........ .......... ..A.......
ED1a         .......... .......... .......... .......... .G........ .......... ..A.......
83972        .......... .......... .......... .......... .G........ .......... ..A.......
S88          .......... .......... .......... .......... .G........ .......... ..A.......
E._albertii  .......... ....C..T.. T..A...... .......... .G........ .......... ..........

          4411
K-12         TCGACCAGGC GACGTCCGAT CTGCGTGAAA CGCTGGAGCA GATGGAAATT CAGAACGTTG AGTTAGATCT
53638        .......... .......... .......... .......... .......... .......... ..........
ATCC_8739    .......... .......... .......... .......... .......... .......... ....G.....
HS           .......... .......... .......... .......... .......... .......... ....G.....
BW2952       .......... .......... .......... .......... .......... .......... ..........
12009        .......... .......... .......... .......... .......... .......... ..........
B171         .......... .......... .......... .......... .......... .......... ..........
E24377A      .......... .......... .......... .......... .......... .......... ..........
SE11         .......... .......... .......... .......... .......... .......... ....G.....
11128        .......... .......... .......... .......... .......... .......... ..........
11368        .......... .......... .......... .......... .......... .......... ..........
IAI1         .......... .......... .......... .......... .......... .......... ....G.....
E110019      .......... .......... .......... .......... .......... .......... ..........
B7A          .......... .......... .......... .......... .......... .......... ..........
CB9615       .......... .......... .......... .......... .......... .......... ....G.....
O157_Sakai   .......... .......... .......... .......... .......... .......... ....G.....
IAI39        .......... .......... .......... .......... ......G... .......... ....G.....
UMN026       .......... .......... .......... .......... .......... .......... ....G.....
SMS-3-5      .......... .......... ........G. .......... .......... .......... ....G.....
536          .......... .......... ........G. .......... A.....G... .......... ....G.....
APEC_O1      .......... .......... ........G. .......... A.....G... .......... ....G.....
CFT073       .......... .......... ........G. .......... A.....G... .......... ....G.....
E2348/69     .......... .......... .......... .......... .......... .......... ....G.....
UTI89        .......... .......... ........G. .......... A.....G... .......... ....G.....
F11          .......... .......... ........G. .......... A.....G... .......... ....G.....
ED1a         .......... .......... ........G. .......... A.....G... .......... ....G.....
83972        .......... .......... ........G. .......... A.....G... .......... ....G.....
S88          .......... .......... ........G. .......... A.....G... .......... ....G.....
E._albertii  .......... ...A...... .......... .......... .......... .......... ..........
```

```
            4481
K-12          GGCGAAAAAG CGCGCCCAGG AAGCGGCGCG TATTAAATCC GAGTTTCTGG CAAATATGTC ACACGAGCTG
53638         .......... .......... .......... .......... .......... .......... ..........
ATCC_8739     .......... .......... .......... .......... .......... .......... ..........
HS            .......... .......... .......... .......... .......... .......... ..........
BW2952        .......... .......... .......... .......... .......... .......... ..........
12009         .......... .......... .......... .......... .......... .......... ..........
B171          .......... .......... .......... .......... .......... .......... ..........
E24377A       .......... .......... .......... .......... .......... .......... ..........
SE11          .......... .......... .......... .......... .......... .......... ..........
11128         .......... .......... .......... .......... .......... .......... ..........
11368         .......... .......... .......... .......... .......... .......... ..........
IAI1          .......... .......... .......... .......... .......... .......... ..........
E110019       .......... .......... .......... .......... .......... .......... ..........
B7A           .......... .......... .......... .......... .......... .......... ..........
CB9615        .......... .......... .......... .......... .......... .......... ..........
O157_Sakai    .......... .......... .......... .......... .......... .......... ..........
IAI39         .......... .......... .......... .......... .......... .......... ..........
UMN026        .......... .......... .......... .......... .......... .......... ......A...
SMS-3-5       .......... .......... .......... .......... .......... .......... ..........
536           .......... .......... .......... .......... .......... .......... ..........
APEC_O1       .......... .......... .......... .......... .......... .......... ..........
CFT073        .......... .......... .......... .......... .......... .......... ..........
E2348/69      .......... .......... .......... .......... ........T. .......... ..........
UTI89         .......... .......... .......... .......... .......... .......... ..........
F11           .......... .......... .......... .......... .......... .......... ..........
ED1a          .......... .......... .......... .......... .......... .......... ..........
83972         .......... .......... .......... .......... .......... .......... ..........
S88           .......... .......... .......... .......... .......... .......... ..........
E._albertii   A........A .......... .......... A.....G..A .....C.... .......... G..T...T.A

            4551
K-12          CGTACACCAC TGAATGGTGT TATTGGCTTT ACCCGCCTGA CGCTGAAAAC AGAATTAACA CCAACGCAGC
53638         .......... .......... .......... .......... .......... .......... ..........
ATCC_8739     .......... .......... .......... .......... .......... .......... ..........
HS            .......... .......... .......... .......... .......... .......... ..........
BW2952        .......... .......... .......... .......... .......... .......... ..........
12009         .......... .......... .......... .......... .......... .......... ..........
B171          .......... .......... .......... .......... .......... .......... ..........
E24377A       .......... .......... .......... .......... .......... .......... ..........
SE11          .......... .......... .......... .......... .......... .......... ..........
11128         .......... .......... .......... .......... .......... .......... ..........
11368         .......... .......... .......... .......... .......... .......... ..........
IAI1          .......... .......... .......... .......... .......... .......... ..........
E110019       .......... .......... .......... .......... .......... .......... ..........
B7A           .......... .......... .......... .......... .......... .......... ..........
CB9615        .......... .......... ......T... .......... .......... .......... ..........
O157_Sakai    .......... .......... ......T... .......... .......... .......... ..........
IAI39         .......... .......... .......... .......... .......... .......... ..........
UMN026        .......... .......... .......... .......... .......... .......... ..........
SMS-3-5       .......... .......... .......... .......... .......... .......... ..........
536           .......... .......... .......... .......... .......... .......... ..........
APEC_O1       .......... .......... .......... .......... .......... .......... ..........
CFT073        .......... .......... .......... .......... .......... .......... ..........
E2348/69      .......... .......... .......... .......... .......... .......... ..........
UTI89         .......... .......... .......... .......... .......... .......... ..........
F11           .......... .......... .......... .......... .......... .......... ..........
ED1a          .......... .......... .......... .......... .......... .......... ..........
83972         .......... .......... .......... .......... .......... .......... ..........
S88           .......... .......... .......... .......... .......... .......... ..........
E._albertii   .....G..GT .......... ...C...... .......... .C..A..... ....C.G..G ..G..A....
```

```
            4621
K-12         GCGATCACCT GAATACGATT GAACGTTCGG CAAATAATTT GCTGGCAATT ATTAATGATG TTCTCGACTT
53638        .......... .......... .......... .......... .......... .......... ..........
ATCC_8739    .......... .......... .......... .......... .......... .......... ..........
HS           .......... .......... .......... .......... .......... .......... ..........
BW2952       .......... .......... .......... .......... .......... .......... ..........
12009        .......... .......... .......... .......... .......... .......... ..........
B171         .......... .......... .......... .......... .......... .......... ..........
E24377A      .......... .......... .......... .......... .......... .......... ..........
SE11         .......... .......... .......... .......... .......... .......... ..........
11128        .......... .......... .......... .......... .......... .......... ..........
11368        .......... .......... .......... .......... .......... .......... ..........
IAI1         .......... .......... .......... .......... .......... .......... ..........
E110019      .......... .......... .......... .......... .......... .......... ..........
B7A          .......... .......... .......... .......... .......... .......... ..........
CB9615       .......... .......... .......... .......... .......... .......... ..........
O157_Sakai   .......... .......... .......... .......... .......... .......... ..........
IAI39        .......... .......... .......... .......... .......... .......... ..........
UMN026       .......... .......... .......... .......... .......... .......... ..........
SMS-3-5      .......... .......... .......... .......... .......... .......... ..........
536          .......... .......... .......... .......... .......... .......... ..........
APEC_O1      .......... .......... .......... .......... .......... .......... ..........
CFT073       .......... .......... .......... .......... .......... .......... ..........
E2348/69     .......... .......... .......... .......... .......... .......... ..........
UTI89        .......... .......... .......... .......... .......... .......... ..........
F11          .......... .......... .......... .......... .......... .......... ..........
ED1a         .......... .......... .......... .......... .......... .......... ..........
83972        .......... .......... .......... .......... .......... .......... ..........
S88          .......... .......... .......... .......... .......... .......... ..........
E._albertii  .A........ ......C... .......... .......C.. A........C .....C.... .C........

            4691
K-12         CTCGAAACTG GAAGCAGGTA AGCTGATTCT GGAAAGTATT CCATTCCCAC TACGCAGCAC GCTGGATGAA
53638        .......... .......... .......... .......... .......... .......... ..........
ATCC_8739    .......... .......... .......... .......... .......... .......... ..........
HS           .......... .......... .......... .......... .......... .......... ..........
BW2952       .......... .......... .......... .......... .......... .......... ..........
12009        .......... .......... .......... .......... .......... .......... ..........
B171         .......... .......... .......... .......... .......... .......... ..........
E24377A      .......... .......... .......... .......... .......... .......... ..........
SE11         .......... .......... .......... .......... .......... .......... ..........
11128        .......... .......... .......... .......... .......... .......... ..........
11368        .......... .......... .......... .......... .......... .......... ..........
IAI1         .......... .......... .......... .......... .......... .......... ..........
E110019      .......... .......... .......... .......... .......... .......... ..........
B7A          .......... .......... .......... .......... .......... .......... ..........
CB9615       .......... .......... .......... .......... .......... .......... ..........
O157_Sakai   .......... .......... .......... .......... .......... .......... ..........
IAI39        .......... .....C.... .......... .......... .......... .......... ..........
UMN026       .......... .......... .......... .......... .......... .......... ..........
SMS-3-5      .......... .......... .......... .......... .......... .......... ..........
536          .......... .....C.... .......... .......... .......... .......... ..........
APEC_O1      .......... .....C.... .......... .......... .......... .......... ..........
CFT073       .......... .....C.... .......... .......... .......... .......... ..........
E2348/69     .......... .....C.... .......... .......... .......... .......... ..........
UTI89        .......... .....C.... .......... .......... .......... .......... ..........
F11          .......... .....C.... .......... .......... .......... .......... ..........
ED1a         .......... .....C.... .......... .......... .......... .......... ..........
83972        .......... .....C.... .......... .......... .......... .......... ..........
S88          .......... .....C.... .......... .......... .......... .......... ..........
E._albertii  ...A...... .....T..C. .A........ ......C... .......... .G..T..... ..........
```

```
          4761
K-12         GTCGTTACTC TGCTGGCACA TTCTTCTCAC GATAAAGGGT TAGAACTGAC GCTCAATATT AAAAGCGACG
53638        .......... .......... .......... .......... .......... .......... ..........
ATCC_8739    .......... .......... .......... .......... .G........ .......... ..........
HS           .......... .......... .......... .......... .G........ .......... ..........
BW2952       .......... .......... .......... .......... .......... .......... ..........
12009        .......... .......... .......... .......... .G........ .......... ..........
B171         .......... .......... .......... .......... .G........ .......... ..........
E24377A      .......... .......... .......... .......... .G........ .......... ..........
SE11         .......... .......... .......... .......... .G........ .......... ..........
11128        .......... .......... .......... .......... .G........ .......... ..........
11368        .......... .......... .......... .......... .G........ .......... ..........
IAI1         .......... .......... .......... .......... .G........ .......... ..........
E110019      .......... .......... .......... .......... .G........ .......... ..........
B7A          .......... .......... .......... .......... .G........ .......... ..........
CB9615       .......... .......... .......... .......... .G........ .......... ..........
O157_Sakai   .......... .......... .......... .......... .G........ .......... ..........
IAI39        ........C. .A........ .......... .........C .G........ .......... ..........
UMN026       ........C. .......... .......... .........C .G........ .......... ..........
SMS-3-5      ........C. .......... .......... .........C .G........ .......... ..........
536          ........C. .......... .......... .........C .G........ .......... ..........
APEC_O1      ........C. .......... .......... .........C .G........ .......... ..........
CFT073       ........C. .......... .......... .........C .G........ .......... ..........
E2348/69     ........C. .......... .......... .........C .G........ .......... ..........
UTI89        ........C. .......... .......... .........C .G........ .......... ..........
F11          ........C. .......... .......... .........C .G........ .......... ..........
ED1a         ........C. .......... .......... .........C .G........ .......... ..........
83972        ........C. .......... .......... .........C .G........ .......... ..........
S88          ........C. .......... .......... .........C .G........ .......... ..........
E._albertii  ..G..G..A. .......C.. C..A..G... .....G...C .G........ .......... ..G.......

          4831
K-12         TGCCTGATAA CGTGATCGGC GACCCGCTGC GATTACAGCA AATCATCACT AACCTGGTGG GGAATGCAAT
53638        .......... .......... .......... .......... .......... .....A.... ..........
ATCC_8739    .......... .......... .......... .......... .......... .......... ..........
HS           .......... .......... .......... .......... .......... .......... ..........
BW2952       .......... .......... .......... .......... .......... .......... ..........
12009        .......... .......... .....A.... .......... .......... .......... ..........
B171         .......... .......... .....A.... .......... .......... .......... ..........
E24377A      .......... .......... .....A.... .......... .......... .......... ..........
SE11         .......... .......... .......... .......... .......... .......... ..........
11128        .......... .......... .....A.... .......... .......... .......... ..........
11368        .......... .......... .....A.... .......... .......... .......... ..........
IAI1         .......... .......... .......... .......... .......... .......... ..........
E110019      .......... .......... .....A.... .......... .......... .......... ..........
B7A          .......... .......... .....A.... .......... .......... .......... ..........
CB9615       .......... .......... .....A.... .......... .......... .......... ..........
O157_Sakai   .......... .......... .....A.... .......... .......... .......... ..........
IAI39        .......... .......... .....A..A. .......... .......... .......... ..........
UMN026       .......... ...T...... .....A..A. .......... .......... ........A. ..........
SMS-3-5      .......... ...T...... .....A..A. .......... .......... .......... ..........
536          .......... .......... .....A..A. .......... .......... ........A. ..........
APEC_O1      .......... .......... .....A..A. .......... .......... ........A. ..........
CFT073       .......... .......... .....A..A. .......... .......... ........A. ..........
E2348/69     .......... ...T...... .....A..A. .......... .......... .......... ..........
UTI89        .......... .......... .....A..A. .......... .......... ........A. ..........
F11          .......... .......... .....A..A. .......... .......... ........A. ..........
ED1a         .......... .......... .....A..A. .......... .......... ........A. ..........
83972        .......... .......... .....A..A. .......... .......... ........A. ..........
S88          .......... .......... .....A..A. .......... .......... ........A. ..........
E._albertii  ....C..... ......T... .....A.... .T........ .........A .......... .......G..
```

```
          4901
K-12         TAAATTCACC GAGAATGGCA ACATTGATAT TCTGGTAGAA AAACGTGCGC TGAGTAATAC CAAAGTGCAG
53638        .......... .......... .......... .......... .......... .......... ..........
ATCC_8739    .......... .......... .......... .......... .......... .......... ..........
HS           .......... .......... .......... .......... .......... .......... ..........
BW2952       .......... .......... .......... .......... .......... .......... ..........
12009        .......... .......... .......... .......... .......... .......... ..........
B171         .......... .......... .......... .......... .......... .......... ..........
E24377A      .......... .......... .......... .......... .......... .......... ..........
SE11         .......... .......... .......... .......... .......... .......... ..........
11128        .......... .......... .......... .......... .......... .......... ..........
11368        .......... .......... .......... .......... .......... .......... ..........
IAI1         .......... .......... .......... .......... .......... .......... ..........
E110019      .......... .......... .......... .......... .......... .......... ..........
B7A          .......... .......... .......... .......... .......... .......... ..........
CB9615       .......... .......... .......... .......... .......... .......... ..........
O157_Sakai   .......... .......... .......... .......... .......... .......... ..........
IAI39        .......... .......... .......... .......... .......... .......... .........A
UMN026       .......... .......... .......... .......... .......... .......... ..........
SMS-3-5      .......... .......... .......... .......... .......... .......... .........A
536          .......... .......... .......... .......... .......... .......... .........A
APEC_O1      .......... .......... .......... .......... .......... .......... .........A
CFT073       .......... .......... .......... .......... .......... .......... .........A
E2348/69     .......... .......... .......... .......... .......... .......... ..........
UTI89        .......... .......... .......... .......... .......... .......... .........A
F11          .......... .......... .......... .......... .......... .......... .........A
ED1a         .......... .......... ....C..... .......... .......... .......... ..........
83972        .......... .......... .......... .......... .......... .......... .........A
S88          .......... .......... .......... .......... .......... .......... .........A
E._albertii  .......... .......... ....C..... ......G... .........T .......... ..........

          4971
K-12         ATTGAAGTGC AGATTCGGGA TACCGGCATT GGTATTCCTG AACGCGATCA ATCGCGCTTA TTCCAGGCCT
53638        .......... .......... .......... .......... .......... .......... ..........
ATCC_8739    .......... .......... .......... .......... .......... .......... ..........
HS           .......... .......... .......... .......... .......... .......... ..........
BW2952       .......... .......... .......... .......... .......... .......... ..........
12009        .......... .......... .......... .......... .......... .......... ..........
B171         .......... .......... .......... .......... .......... .......... ..........
E24377A      .......... .......... .......... .......... .......... .......... ..........
SE11         .......... .......... .......... .......... .......... .......... ..........
11128        .......... .......... .......... .......... .......... .......... ..........
11368        .......... .......... .......... .......... .......... .......... ..........
IAI1         .......... .......... .......... .......... .......... .......... ..........
E110019      .......... .......... .......... .......... .......... .......... ..........
B7A          .......... .......... .......... .......... .......... .......... ..........
CB9615       .......... .......... .......... .......... .......... .......... ..........
O157_Sakai   .......... .......... .......... .......... .......... .......... ..........
IAI39        .......... .......... .......... .......... .......... .......... ..........
UMN026       .......... .......... .......... ........G. .......... .......... ..........
SMS-3-5      .......... .......... .......... .......... .......C.. .......... ..........
536          .......... .......... .......... .......... .......... .......... ..........
APEC_O1      .......... .......... .......... .......... .......... .......... ..........
CFT073       .......... .......... .......... .......... .......... .......... ..........
E2348/69     .......... .......... .......... .......... .......... .......... ..........
UTI89        .......... .......... .......... .......... .......... .......... ..........
F11          .......... .......... .......... .......... .......... .......... ..........
ED1a         .......... .......... .......... .......... .......... .......... ..........
83972        .......... .......... .......... .......... .......... .......... ..........
S88          .......... .......... .......... .......... .......... .......... ..........
E._albertii  .......... ....C..C.. ......T..C ..C.....C. .......... ...A.....G ..........
```

```
            5041
K-12           TCCGACAGGC TGATGCCAGT ATTTCCCGCC GTCATGGTGG CACCGGGCTG GGGCTGGTGA TTACACAAAA
53638          .......... .......... .......... .......... .......... .......... ..........
ATCC_8739      .......... .......... .......... .......... .......... .......... ..........
HS             .......... .......... .......... .......... .......... .......... ..........
BW2952         .......... .......... .......... .......... .......... .......... ..........
12009          .......... .......... .......... .......... ......T... .......... ..........
B171           .......... .......... .......... .......... ......T... .......... ..........
E24377A        .......... .......... .......... .......... ......T... .......... ..........
SE11           .......... .......... .......... .......... .......... .......... ..........
11128          .......... .......... .......... .......... ......T... .......... ..........
11368          .......... .......... .......... .......... ......T... .......... ..........
IAI1           .......... .......... .......... .......... .......... .......... ..........
E110019        .......... .......... .......... .......... ......T... .......... ..........
B7A            .......... .......... .......... .......... ......T... .......... ..........
CB9615         .......... .......... .......... .......... .......... .......... ..........
O157_Sakai     .......... .......... .......... .......... .......... .......... ..........
IAI39          ..A....... .......... .......... .......... .......... .......... ..........
UMN026         .......... .......... .......... .......... .......... .......... ..........
SMS-3-5        ..A....... .......... .......... .......... .......... .......... ..........
536            ..A....... .......... .......... .......... .......... .......... ..........
APEC_O1        ..A....... .......... .......... .......... .......... .......... ..........
CFT073         ..A....... .......... .......... .......... .......... .......... ..........
E2348/69       .......... .......... .......... .......... .......... .......... ..........
UTI89          ..A....... .......... .......... .......... .......... .......... ..........
F11            ..A....... .......... .......... .......... .......... .......... ..........
ED1a           ..A....... .......... .......... .......... .......... .......... ..........
83972          ..A....... .......... .......... .......... .......... .......... ..........
S88            ..A....... .......... .......... .......... .......... .......... ..........
E._albertii    ..A....... A......... .....A..T. .......C.. ......AT.. ..A.....C. ..........

            5111
K-12           ACTGGTTAAT GAAATGGGCG GCGATATTTC GTTCCATAGC CAGCCGAATC GCGGTTCAAC TTTCTGGTTC
53638          .......... .......... .......... .......... .......... .......... ..........
ATCC_8739      .......... .......... .......... .......... .......... .......... ..........
HS             .......... .......... .......... .......... .......... .......... ..........
BW2952         .......... .......... .......... .......... .......... .......... ..........
12009          .......... .......... .......... .......... .......... .......... ..........
B171           .......... .......... .......... .......... .......... .......... ..........
E24377A        .......... .......... .......... .......... .......... .......... ..........
SE11           .......... .......... .......... .......... .......... .......... ..........
11128          .......... .......... .......... .......... .......... .......... ..........
11368          .......... .......... .......... .......... .......... .......... ..........
IAI1           .......... .......... .......... .......... .......... .......... ..........
E110019        .......... .......... .......... .......... .......... .......... ..........
B7A            .......... .......... .......... .......... .......... .......... ..........
CB9615         .......... .......... .......... .......... .......... .......... ..........
O157_Sakai     .......... .......... .......... .......... .......... .......... ..........
IAI39          .......... .......... .T........ .......... .......... .T........ ..........
UMN026         .......... .......... .......... .......... .......... .......... ..........
SMS-3-5        .......... .......... .T........ .......... .......... .T........ ..........
536            .......... .......... .......... .......... .......... .......... ..........
APEC_O1        .......... .......... .......... .......... .T........ .T........ ..........
CFT073         .......... .......... .......... .......... .......... .......... ..........
E2348/69       .......... .......... .......... ...T...... .......... .......... ..........
UTI89          .......... .......... .......... .......... .T........ .T........ ..........
F11            .......... .......... .......... .......... .......... .......... ..........
ED1a           .......... .......... .......... .......... .T........ .T........ ..........
83972          .......... .......... .......... .......... .......... .......... ..........
S88            .......... .......... .......... .......... .T........ .T........ ..........
E._albertii    .......... .......... .......C.. .......... .......... .T..C..C.. A.........
```

```
          5181
K-12           CACATTAATC TCGATCTGAA CCCGAACATT ATTATCGAAG GGCCATCCAC CCAGTGCCTC GCAGGTAAGC
53638          .......... .......... .......... .......... .......... .......... ..........
ATCC_8739      .......... .......... .......... .......... .......... .......... ..........
HS             .......... .......... .......... .......... .......... .......... ..........
BW2952         .......... .......... .......... .......... .......... .......... ..........
12009          .......... .......... .......... .......... .......... .......... ..C.......
B171           .......... .......... .......... .......... .......... .......... ..C.......
E24377A        .......... .......... .......... .......... .......... .......... ..C.......
SE11           .......... .......... .......... .......... .......... .......... ..........
11128          .......... .......... .......... .......... .......... .......... ..C.......
11368          .......... .......... .......... .......... .......... .......... ..C.......
IAI1           .......... .......... .......... .......... .......... .......... ..........
E110019        .......... .......... .......... .......... .......... .......... ..C.......
B7A            .......... .......... .......... .......... .......... .......... ..C.......
CB9615         .......... .......... .......... .....T.... .......... .......... ..C.....A.
O157_Sakai     .......... .......... .......... .....T.... .......... .......... ..C.....A.
IAI39          .......... .......... .......... .......... .......... .......... ..C.......
UMN026         .......... .......... T......... .......... .......... ...A...... ..C..C....
SMS-3-5        .......... .......... .......... .......... .......... .......... ..C.......
536            .......... .......... .......... .......... .......... .......... ..T.......
APEC_O1        .......... .......... .......... .......... .......... .......... ..T.......
CFT073         .......... .......... .......... .......... .......... .......... ..T.......
E2348/69       .......... .......... T......... .....T.... .......... .......... ..C.....A.
UTI89          .......... .......... .......... .......... .......... .......... ..T.......
F11            .......... .......... .......... .......... .......... .......... ..T.......
ED1a           .......... .......... .......... .......... .......... .......... ..T.......
83972          .......... .......... .......... .......... .......... .......... ..T.......
S88            .......... .......... .......... .......... .......... .......... ..T.......
E._albertii    .......... .......... ......T... .......... .......... A........T .....C..A.

          5251
K-12           GCCTGGCCTA TGTCGAACCA AACTCCGCAG CAGCGCAATG CACGCTGGAT ATTTTAAGTG AAACGCCGCT
53638          .......... .......... .......... .......... .......... .......... ..........
ATCC_8739      .......... .......... .......... .......... .......... .......... ..........
HS             .......... .......... .......... .......... .......... .......... ..........
BW2952         .......... .......... .......... .......... .......... .......... ..........
12009          .......... .......... .......... .......... .......... .......... ..........
B171           .......... .......... .......... .......... .......... .......... ..........
E24377A        .......... .......... .......... .......... .......... .......... ..........
SE11           .......... .......... .......... .......... .......... .......... ..........
11128          .......... .......... .......... .......... .......... .......... ..........
11368          .......... .......... .......... .......... .......... .......... ..........
IAI1           .......... .......... .......... .......... .......... .......... ..........
E110019        .......... .......... .......... .......... .......... .......... ..........
B7A            .......... .......... .......... .......... .......... .......... ..........
CB9615         .......... .......... .......... .......... .......... .......... ..........
O157_Sakai     .......... .......... .......... .......... .......... .......... ..........
IAI39          .......... .......... .......... .......... .......... .......... ..........
UMN026         .......... .......... .......... .......... .......... .......... ..........
SMS-3-5        .......... .......... .......... .......... .......... .......... ..........
536            .......... .......... .......... ....A..... .......... .......... ..........
APEC_O1        .......... .......... .......... ....A..... .......... .......... ..........
CFT073         .......... .......... .......... ....A..... .......... .......... ..........
E2348/69       .......... .......... .......... .......... .......... .......... ..........
UTI89          .......... .......... .......... ....A..... .......... .......... ..........
F11            .......... .......... .......... ....A..... .......... .......... ..........
ED1a           .......... .......... .......... ....A..... .......... .......... ..........
83972          .......... .......... .......... ....A..... .......... .......... ..........
S88            .......... .......... .......... ....A..... .......... .......... ..........
E._albertii    .T.....T.. ...T.....G ..T.....T. .C.....G.. T..T..C..C ........C. ..........
```

```
           5321
K-12           GGAAGTGGTT TATAGCCCAA CGTTCTCCGC GCTGCCTCCC GCGCATTACG ACATGATGTT GTTAGGCATC
53638          .......... .......... .......... .......... .......... .......... ..........
ATCC_8739      .......... .......... .......... .......... .......... .......... ..........
HS             .......... .......... .......... .......... .......... .......... ..........
BW2952         .......... .......... .......... .......... .......... .......... ..........
12009          .......... .......... .......... .......... .......... .......... ..........
B171           .......... .......... .......... .......... .......... .......... ..........
E24377A        .......... .......... .......... .......... .......... .......... ..........
SE11           .......... .......... .......... .......... .......... .......... ..........
11128          .......... .......... .......... .......... .......... .......... ..........
11368          .......... .......... .......... .......... .......... .......... ..........
IAI1           .......... .......... .......... .......... .......... .......... ..........
E110019        .......... .......... .......... .......... .......... .......... ..........
B7A            .......... .......... .......... .......... .......... .......... ..........
CB9615         .......... .......... .......... .......... .......... .......... ..........
O157_Sakai     .......... .......... .......... .......... .......... .......... ..........
IAI39          .......... .......... .......... .......... .......... .......... A.........
UMN026         .......... .......... .......... .......... .......... .......... A........T
SMS-3-5        .......... .......... .......... .......... .......... .......... ..........
536            .......... .......... .......... .......... .......... .......... A........A
APEC_O1        .......... .......... .......... .......... .......... .......... A........A
CFT073         .......... .......... .......... .......... .......... .......... A........A
E2348/69       ...G...... .......... .......... .......... .......... .......... ..........
UTI89          .......... .......... .......... .......... .......... .......... A........A
F11            .......... .......... .......... .......... .......... .......... A........A
ED1a           .......... .......... .......... .......... .......... .......... A........A
83972          .......... .......... .......... .......... .......... .......... A........A
S88            .......... .......... .......... .......... .......... .......... A........A
E._albertii    ...G...A.. .......... GC.....T.. A..T..G..A .AA..C.... .......... .C....A...

           5391
K-12           GCGGTGACCT TCCGCGAGCC GCTAACAATG CAACATGAGC GATTAGCGAA AGCGGTATCG ATGACCGATT
53638          .......... .......... .......... .......... .......... .......... ..........
ATCC_8739      .......... .......... .......... .......... .......... .......... ..........
HS             .......... .......... .......... .......... .......... .......... ..........
BW2952         .......... .......... .......... .......... .......... .......... ..........
12009          .......... .......... .......... .......... .......... .......... ..........
B171           .......... .......... .......... .......... .......... .......... ..........
E24377A        .......... .......... .......... .......... .......... .......... ..........
SE11           .......... .......... .......... .......... .......... .......... ..........
11128          .......... .......... .......... .......... .......... .......... ..........
11368          .......... .......... .......... .......... .......... .......... ..........
IAI1           .......... .......... .......... .......... .......... .......... ..........
E110019        .......... .......... .......... .......... .......... .......... ..........
B7A            .......... .......... .......... .......... .......... .......... ..........
CB9615         .......... .......... .......... .......... .......... .......... ..........
O157_Sakai     .......... .......... .......... .......... .......... .......... ..........
IAI39          .......... .......... .......... .......... .......... ...A...... ..........
UMN026         .......... .......... .......... .......... .......... .......... ..........
SMS-3-5        .......... .......... .......... .......... .......... ...A...... ..........
536            .......... .......... .......... .......... .......... ...A...... ..........
APEC_O1        .......... .......... .......... .......... .......... ...A...... ..........
CFT073         .......... .......... .......... .......... .......... ...A...... ..........
E2348/69       .......... .......... .......... .......... .......... .......... ..........
UTI89          .......... .......... .......... .......... .......... ...A...... ..........
F11            .......... .......... .......... .......... .......... ...A...... ..........
ED1a           .......... .......... .......... .......... .......... ...A...... ..........
83972          .......... .......... .......... .......... .......... ...A...... ..........
S88            .......... .......... .......... .......... .......... ...A...... ..........
E._albertii    ..C.....A. .......A.. ......G... .......CC. .G..GCTA.. ...C.....C ...G.T....
```

```
           5461
K-12          TCCTGATGCT GGCACTTCCT TGCCATGCAC AAGTCAATGC TGAAAAACTC AAGCAAGATG GTATCGGCGC
53638         .......... .......... .......... .......... .......... .......... ..........
ATCC_8739     .......... .......... .......... .......... .......... .......... ..........
HS            .......... .......... .......... .......... .......... .......... ..........
BW2952        .......... .......... .......... .......... .......... .......... ..........
12009         .......... .......... .......... .......... A......... .......... ..........
B171          .......... .......... .......... .......... A......... .......... ..........
E24377A       .......... .......... .......... .......... A......... .......... ..........
SE11          .......... .......... .......... .......... .......... .......... ..........
11128         .......... .......... .......... .......... A......... .......... ..........
11368         .......... .......... .......... .......... A......... .......... ..........
IAI1          .......... .......... .......... .......... .......... .......... ..........
E110019       .......... .......... .......... .......... A......... .......... ..........
B7A           .......... .......... .......... .......... A......... .......... ..........
CB9615        .......... .......... .......... .......... .......... .......... ..........
O157_Sakai    .......... .......... .......... .......... .......... .......... ..........
IAI39         .T........ .......... .......... .......... .........T .......... ..........
UMN026        .T........ .......... .......... .......... .........T .......... ..........
SMS-3-5       .T........ .......... .......... .......... .........T .......... ..........
536           .......... .......... .......... .......... .........T .......... ..........
APEC_O1       .......... .......... .......... .......... .........T .......... ..........
CFT073        .......... .......... .......... .......... .........T .......... ..........
E2348/69      .......... .......... .......... .......... .......... .......... ..........
UTI89         .......... .......... .......... .......... .........T .......... ..........
F11           .......... .......... .......... .......... .........T .......... ..........
ED1a          .......... .......... .......... .......... .........T .......... ..........
83972         .......... .......... .......... .......... .........T .......... ..........
S88           .......... .......... .......... .......... .........T .......... ..........
E._albertii   AT........ ...G.....C .......T.. .G........ C.....G... .......... ....T.....

           5531
K-12          GTGTCTGCTG AAACCATTAA CACCTACACG CCTGTTGCCT GCCCTGACGG AATTTTGTCA TCACAAACAA
53638         .......... .......... .......... .......... .......... .......... ..........
ATCC_8739     .......... .......... .......... .......... .......... .......... ..........
HS            .......... .......... .......... .......... .......... .......... ..........
BW2952        .......... .......... .......... .......... .......... .......... ..........
12009         .......... .......... .......G.. .......... .......... .......... ..........
B171          .......... .......... .......G.. .......... .......... .......... ..........
E24377A       .......... .......... .......G.. .......... .......... .......... ..........
SE11          .......... .......... .......... .......... .......... .......... ..........
11128         .......... .......... .......G.. .......... .......... .......... ..........
11368         .......... .......... .......G.. .......... .......... .......... ..........
IAI1          .......... .......... .......... .......... .......... .......... ..........
E110019       .......... .......... .......G.. .......... .......... .......... ..........
B7A           .......... .......... .......G.. .......... .......... .......... ..........
CB9615        .......... .......... .......G.. .......... .......... .......... ..........
O157_Sakai    .......... .......... .......G.. .......... .......... .......... ..........
IAI39         .......... .......... .......G.. ......A... .......... .......... ..........
UMN026        .......... .......... .......G.. ......A... .......... .......... ..........
SMS-3-5       .......... .......... .......G.. ......A... .......... .......... ..........
536           .......... .......... .......G.. .......... .......... .......... ..........
APEC_O1       .......... .......... .......G.. .......... .......... .......... ..........
CFT073        .......... .......... .......G.. .......... ........A. .......... ..........
E2348/69      .......... .......... .......G.. .......... .......... .......... ..........
UTI89         .......... .......... .......G.. .......... .......... .......... ..........
F11           .......... .......... .......G.. .......... .......... .......... ..........
ED1a          .......... .......... .......G.. .......... .......... .......... ..........
83972         .......... .......... .......G.. .......... ........A. .......... ..........
S88           .......... .......... .......G.. .......... .......... .......... ..........
E._albertii   C..CT..... .......... .......... T......... ..G.....C. .......... ...A......
```

```
          5601
K-12         AACACGCTTT TGCCTGTAAC CGATGAAAGT AAGCTGGCAA TGACAGTCAT GGCGGTTGAT GACAACCCCG
53638        .......... .......... .........C .......... .......... ...A...... ..........
ATCC_8739    .......... .......... .........C .......... .......... ...A...... ..........
HS           .......... .......... .......... .......... .......... .......... ..........
BW2952       .......... .......... .......... .......... .......... .......... ..........
12009        .......... .......... .........C .......... .......... ...A...... ..........
B171         .......... .......... .........C .......... .......... ...A...... ..........
E24377A      .......... .......... .........C .......... .......... ...A...... ..........
SE11         .......... .......... .......... .......... .......... .......... ..........
11128        .......... .......... .........C .......... .......... ...A...... ..........
11368        .......... .......... .........C .......... .......... ...A...... ..........
IAI1         .......... .......... .......... .......... .......... .......... ..........
E110019      .......... .......... .........C .......... .......... ...A...... ..........
B7A          .......... .......... .........C .......... .......... ...A...... ..........
CB9615       .......... .......... .......... .......... .......... .......... ..........
O157_Sakai   .......... .......... .......... .......... .......... .......... ..........
IAI39        .......... .......... .......... .......... .......... .......... ..........
UMN026       .......... .......... .......... .......... .......... .......... ..........
SMS-3-5      .......... .......... .......... .......... .......... .......... ..........
536          .......... .......... .......... .......... .......... .......... ..........
APEC_O1      .......... .......... .......... .......... .......... .......... ..........
CFT073       .......... .......... .......... .......... .......... .......... ..........
E2348/69     .......... .......... .......... .......... .......... .......... ..........
UTI89        .......... .......... .......... .......... .......... .......... ..........
F11          .......... .......... .......... .......... .......... .......... ..........
ED1a         .......... .......... .......... .......... .......... .......... ..........
83972        .......... .......... .......... .......... .......... .......... ..........
S88          .......... .......... .......... .......... .......... .......... ..........
E._albertii  .....A.... .......C.A .......C.. .......... ....G..... ...C...... ..T.......

          5671
K-12         CTAACCTGAA ACTTATCGGC GCATTGCTGG AAGATATGGT GCAACATGTG GAACTTTGCG ATAGCGGGCA
53638        .......... .......... .......... .......... .......... .......... ..........
ATCC_8739    .......... .......... .......... .......... .......... .......... ..........
HS           .......... .......... .......... .......... .......... .......... ..........
BW2952       .......... .......... .......... .......... .......... .......... ..........
12009        .......... .......... .......... .......... .......... .......... ..........
B171         .......... .......... .......... .......... .......... .......... ..........
E24377A      .......... .......... .......... .......... .......... .......... ..........
SE11         .......... .......... .......... .......... .......... .......... ..........
11128        .......... .......... .......... .......... .......... .......... ..........
11368        .......... .......... .......... .......... .......... .......... ..........
IAI1         .......... .......... .......... .......... .......... .......... ..........
E110019      .......... .......... .......... .......... .......... .......... ..........
B7A          .......... .......... .......... .......... .......... .......... ..........
CB9615       .......... .......... .......... .......... .......... .......... ..........
O157_Sakai   .......... .......... .......... .......... .......... .......... ..........
IAI39        .......... .......... .......... .......... .......... .......... ..........
UMN026       .......... .......... .......... .......... .......... .......... ..........
SMS-3-5      .......... .......... .......... .......... .......... .......... ..........
536          .......... .......... .......... .......... .......... .......... ..........
APEC_O1      .......... .......... .......... .......... .......... .......... ..........
CFT073       .......... .......... .......... .......... .......... .......... ..........
E2348/69     .......... .......... .......... .......... .......... .......... ..........
UTI89        .......... .......... .......... .......... .......... .......... ..........
F11          .......... .......... .......... .......... .......... .......... ..........
ED1a         .......... .......... .......... .......... .......... .......... ..........
83972        .......... .......... .......... .......... .......... .......... ..........
S88          .......... .......... .......... .......... .......... .......... ..........
E._albertii  .A........ G......... .......... .......... ...G.....T ..G....... .......A..
```

```
             5741
K-12         TCAGGCGGTT GAACGGGCGA AACAGATGCC GTTCGATTTG ATCTTAATGG ATATTCAAAT GCCGGACATG
53638        .......... .......... .......... .......... .......... .......... ...T......
ATCC_8739    .......... .......... .......... .......... .......... .......... ...T......
HS           .......... .......... .......... .......... .......... .......... ..........
BW2952       .......... .......... .......... .......... .......... .......... ..........
12009        .......... .......... .......... .......... .......... .......... ...T......
B171         .......... .......... .......... .......... .......... .......... ...T......
E24377A      .......... .......... .......... .......... .......... .......... ...T......
SE11         .......... .......... .......... .......... .......... .......... ...T......
11128        .......... .......... .......... .......... .......... .......... ...T......
11368        .......... .......... .......... .......... .......... .......... ...T......
IAI1         .......... .......... .......... .......... .......... .......... ...T......
E110019      .......... .......... .......... .......... .......... .......... ...T......
B7A          .......... .......... .......... .......... .......... .......... ...T......
CB9615       .......... .......... .......... .......... .......... .......... ...T......
O157_Sakai   .......... .......... .......... .......... .......... .......... ...T......
IAI39        .......... .......... .......... .......... .......... .......... ..........
UMN026       .......... .......... .......... .......... .......... .......... ..........
SMS-3-5      .......... .......... .......... .......... .......... .......... ..........
536          .......... .......... .......... .........A .......... .......... ..........
APEC_O1      .......... .......... .......... .........A .......... .......... ..........
CFT073       .......... .......... .......... .........A .......... .......... ..........
E2348/69     .......... .......... .......... .......... .......... .......... ..........
UTI89        .......... .......... .......... .........A .......... .......... ..........
F11          .......... .......... .......... .........A .......... .......... ..........
ED1a         .......... .......... .......... .........A .......... .......... ..........
83972        .......... .......... .......... .........A .......... .......... ..........
S88          .......... .......... .......... .........A .......... .......... ..........
E._albertii  ......A... .......... .......... C......... .......... .......G.. ..........

             5811
K-12         GATGGCATTC GGGCCTGCGA ACTCATCCAC CAACTCCCGC ATCAGCAACA AACGCCGGTT ATCGCGGTAA
53638        .......... .......... G......... ..G.....C. .......... .......... ..........
ATCC_8739    .......... .......... G......... ..G.....C. .......... .......... ..........
HS           .......... .......... .......... .......... .......... .......... ..........
BW2952       .......... .......... .......... .......... .......... .......... ..........
12009        .......... .......... G......... ..G.....C. .......... .......... ..........
B171         .......... .......... G......... ..G.....C. .......... .......... ..........
E24377A      .......... .......... G......... ..G.....C. .......... .......... ..........
SE11         .......... .......... G......... ..G.....C. .......... .......... ..........
11128        .......... .......... G......... ..G.....C. .......... .......... ..........
11368        .......... .......... G......... ..G.....C. .......... .......... ..........
IAI1         .......... .......... G......... ..G.....C. .......... .......... ..........
E110019      .......... .......... G......... ..G.....C. .......... .......... ..........
B7A          .......... .......... G......... ..G.....C. .......... .......... ..........
CB9615       .......... .......... G......... ..G.....C. .......... .......... ..........
O157_Sakai   .......... .......... G......... ..G.....C. .......... .......... ..........
IAI39        ..C....... .......... .......... ..G.....C. .......... .......... ..........
UMN026       ..C....... .......... .......... ..G.....C. .......... ......A... ..........
SMS-3-5      ..C....... .......... .......... ..G.....C. .......... .......... ..........
536          ..C....... ....A..T.. G......... ..G....... .......G.. .......... ..........
APEC_O1      ..C....... ....A..T.. G......... ..G....... .......G.. .......... ..........
CFT073       ..C....... ....A..T.. G......... ..G....... .......G.. .......... ..........
E2348/69     ..C....... .......... .......... ..G.....C. .......... .......... ..........
UTI89        ..C....... ....A..T.. G......... ..G....... .......G.. .......... ..........
F11          ..C....... ....A..T.. G......... ..G....... .......G.. .......... ..........
ED1a         ..C....... ....A..T.. G......... ..G....... .......G.. .......... ..........
83972        ..C....... ....A..T.. G......... ..G....... .......G.. .......... ..........
S88          ..C....... ....A..T.. G......... ..G....... .......G.. .......... ..........
E._albertii  ..C..T.... ....G..... ...T..T..T ..G..T.... ....A..G.. .......... ..T..A....
```

```
            5881
K-12        CGGCGCATGC AATGGCCGGG CAAAAAGAGA AGCTGCTTGG CGCAGGGATG AGCGATTATC TGGCGAAACC
53638       .......... .......... .......... .......... .......... .......... ..........
ATCC_8739   .......... .......... .......... .......... .......... .......... ..........
HS          .......... .......... .......... .......... .......... .......... ..........
BW2952      .......... .......... .......... .......... .......... .......... ..........
12009       .......... .......... .......... .......... .......... .......... ..........
B171        .......... .......... .......... .......... .......... .......... ..........
E24377A     .......... .......... .......... .......... .......... .......... ..........
SE11        .......... .......... .......... .......... .......... .......... ..........
11128       .......... .......... .......... .......... .......... .......... ..........
11368       .......... .......... .......... .......... .......... .......... ..........
IAI1        .......... .......... .......... .......... .......... .......... ..........
E110019     .......... .......... .......... .......... .......... .......... ..........
B7A         .......... .......... .......... .......... .......... .......... ..........
CB9615      .......... .......... .......... .......... .......... .......... ..........
O157_Sakai  .......... .......... .......... .......... .......... .......... ..........
IAI39       .......... .......... .......... .......... .......... .......... ..........
UMN026      .......... .......... .......... .......... .......... .......... ..........
SMS-3-5     .......... .......... .......... .......... .......... .......... ..........
536         .......... G......... .......... .A........ ...G...... ..T....... ....C.....
APEC_O1     .......... G......... .......... .A........ ...G...... ..T....... ....C.....
CFT073      .......... G......... .......... .A........ ...G...... ..T....... ....C.....
E2348/69    .......... .......... .......... .......... .......... .......... ..........
UTI89       .......... G......... .......... .A........ ...G...... ..T....... ....C.....
F11         .......... G......... .......... .A........ ...G...... ..T....... ....C.....
ED1a        .......... G......... .......... .A........ ...G...... ..T....... ....C.....
83972       .......... G......... .......... .A........ ...G...... ..T....... ....C.....
S88         .......... G......... .......... .A........ ...G...... ..T....... ....C.....
E._albertii .C..A..... G......... ........A. ....A..... ...G...... ..T....... ....A.....

            5951
K-12        GATTGAAGAA GAGCGATTGC ATAATTTGTT GTTGCGCTAC AAGCCTGGCA GCGGTATTTC CTCTCGCGTC
53638       .......... .......... .......... .......... .......... .......... G.........
ATCC_8739   .......... .......... .......... .......... .......... .......... G.........
HS          .......... .......... .......... .......... .......... .......... ..........
BW2952      .......... .......... .......... .......... .......... .......... ..........
12009       .......... .......... .......... .......... .......... .......... ..........
B171        .......... .......... .......... .......... .......... .......... ..........
E24377A     .......... .......... .......... .......... .......... .......... ..........
SE11        .......... .......... .......... .......... .......... .......... ..........
11128       .......... .......... .......... .......... .......... .......... ..........
11368       .......... .......... .......... .......... .......... .......... ..........
IAI1        .......... .......... .......... .......... .......... .......... ..........
E110019     .......... .......... .......... .......... .......... .......... ..........
B7A         .......... .......... .......... .......... .......... .......... ..........
CB9615      .......... .......... .......... .......... .......... .......... G.........
O157_Sakai  .......... .......... .......... .......... .......... .......... G.........
IAI39       .......... .......... .......... .........T .......... .......... ..........
UMN026      .......... ..A....... ........C. .......... .......... .......... ..........
SMS-3-5     .......... .......... .......... .........T .......... .......... ..........
536         ...C...... .......... ........C. .......... .......... .......... ..........
APEC_O1     ...C...... .......... ........C. .......... .......... .......... ..........
CFT073      ...C...... .......... ........C. .......... .......... .......... ..........
E2348/69    .......... .......... .......... .......... .......... .......... ..........
UTI89       ...C...... .......... ........C. .......... .......... .......... ..........
F11         ...C...... .......... ........C. .......... .......... .......... ..........
ED1a        ...C...... .......... ........C. .......... .......... .......... ..........
83972       ...C...... .......... ........C. .......... .......... .......... ..........
S88         ...C...... .......... ........C. .......... .......... .......... ..........
E._albertii A......... .......... .......AC. ......T... ..A....... .......... ...A......
```

```
          6021
K-12         GTGACGCCCG AAGTCAACGA AATTGTGGTG AACCCGAATG CGACCCTCGA CTGGCAACTG GCACTACGCC
53638        .......... .......... .......... .......... .......... .......... ..........
ATCC_8739    .......... .......... .......... .......... .......... .......... ..........
HS           .......... .......... .......... .......... .......... .......... ..........
BW2952       .......... .......... .......... .......... .......... .......... ..........
12009        .......... .......... .......... .......... .......... .......... ..........
B171         .......... .......... .......... .......... .......... .......... ..........
E24377A      .......... .......... .......... .......... .......... .......... ..........
SE11         .......... .......... .......... .......... .......... .......... ..........
11128        .......... .......... .......A.. .......... .......... .......... ..........
11368        .......... .......... .......... .......... .......... .......... ..........
IAI1         .......... .......... .......... .......... .......... .......... ..........
E110019      .......... .......... .......... .......... .......... .......... ..........
B7A          .......... .......... .......... .......... .......... .......... ..........
CB9615       .......... .......... .......... .......... .......... .......... ..........
O157_Sakai   .......... .......... .......... .......... .......... .......... ..........
IAI39        .......... .......... .......... .......... .......... .......... ..........
UMN026       .......... .......... .......... .......... .......... .......... ..........
SMS-3-5      .......... .......... .......... .......... .......... .......... ..........
536          .......... .......... .........C .......... .......... .......... ..........
APEC_O1      .......... .......... .........C .......... .......... .......... ..........
CFT073       .......... .......... .........C .......... .......... .......... ..........
E2348/69     .......... .......... .........  .......... .......... .......... ..........
UTI89        .......... .......... .........C .......... .......... .......... ..........
F11          .......... .......... .........C .......... .......... .......... ..........
ED1a         .......... .......... .........C .......... .......... .......... ..........
83972        .......... .......... .........C .......... .......... .......... ..........
S88          .......... .......... .........C .......... .......... .......... ..........
E._albertii  ........A. .T..G..... G......... ....A....A ....T..... .......... ..G.......

          6091
K-12         AGGCAGCAGG AAAAACCGAT TTAGCGCGCG ATATGCTGCA AATGTTACTC GATTTCCTGC CTGAAGTTCG
53638        .......... .......... .......... .......... .......... .......... ..........
ATCC_8739    .......... .......... .......... .......... .......... .......... ..........
HS           .......... .......... .......... .......... .......... .......... ..........
BW2952       .......... .......... .......... .......... .......... .......... ..........
12009        .......... .......... .......... .......... .......... .......... ..........
B171         .......... .......... .......... .......... .......... .......... ..........
E24377A      .......... .......... .......... .......... .......... .......... ..........
SE11         .......... .......... .......... .......... .......... .......... ..........
11128        .......... .......... .......... .......... .......... .......... ..........
11368        .......... .......... .......... .......... .......... .......... ..........
IAI1         .......... .......... .......... .......... .......... .......... ..........
E110019      .......... .......... .......... .......... .......... .......... ..........
B7A          .......... .......... .......... .......... .......... .......... ..........
CB9615       .......... .......... .......... .......... .......... .......... ..........
O157_Sakai   .......... .......... .......... .......... .......... .......... ..........
IAI39        .......... .......... .......... .......... .......... .......... ..........
UMN026       .......... .......... .......... .......... .......... .......... ..........
SMS-3-5      .......... .......... .......... .......... .......... .......... ..........
536          .......... .......... .......... .......... .......... .......... ..........
APEC_O1      .......... .......... .......... .......... .......... .......... ..........
CFT073       .......... .......... .......... .......... .......... .......... ..........
E2348/69     .......... .......... .......... .......... .......... .......... ..........
UTI89        .......... .......... .......... .......... .......... .......... ..........
F11          .......... .......... .......... .......... .......... .......... ..........
ED1a         .......... .......... .......... .......... .......... .......... ..........
83972        .......... .......... .......... .......... .......... .......... ..........
S88          .......... .......... .......... .......... .......... .......... ..........
E._albertii  ....T..C.. C......... C.G....... .......... .......... A......... .A........
```

```
            6161
K-12         CAACAAAGTT GAGGAACAGC TGGTTGGAGA AAACCCGGAA GGCCTGGTTG ATTTGATTCA TAAACTGCAT
53638        .......... .......... .......... ......A... .......... .......... ..........
ATCC_8739    .......... .......... .......... ......A... .......... .......... ..........
HS           .......... .......... .......... .......... .......... .......... ..........
BW2952       .......... .......... .......... .......... .......... .......... ..........
12009        .......... .......... .......... .......... .......... .......... ..........
B171         .......... .......... .......... .......... .......... .......... ..........
E24377A      .......... .......... .......... .......... .......... .......... ..........
SE11         .......... .......... .......... .......... .......... .......... ..........
11128        .......... .......... .......... .......... .......... .......... ..........
11368        .......... .......... .......... .......... .......... .......... ..........
IAI1         .......... .......... .......... .......... .......... .......... ..........
E110019      .......... .......... .......... .......... .......... .......... ..........
B7A          .......... .......... .......... .......... .......... .......... ..........
CB9615       .......... .......... .......... .......... .......... .......... ..........
O157_Sakai   .......... .......... .......... .......... .......... .......... ..........
IAI39        .......... .......... .......... .......... .......... .......... ..........
UMN026       .......... .......... .......... .......... .......... .......... ..........
SMS-3-5      .......... .......... .......... .......... .......... .......... ..........
536          .......... .......... .......... .......... .......... .......... ..........
APEC_O1      .......... .......... .......... .......... .......... .......... ..........
CFT073       .......... .......... .......... .......... .......... .......... ..........
E2348/69     .......... .......... .......... .......... .......... .......... ..........
UTI89        .......... .......... .......... .......... .......... .......... ..........
F11          .......... .......... .......... .......... .......... .......... ..........
ED1a         .......... .......... .......... .......... .......... .......... ..........
83972        .......... .......... .......... .......... .......... .......... ..........
S88          .......... .......... .......... .......... .......... .......... ..........
E._albertii  T.....G..A ..A..G.... .A........ .......... ..T....... .......C.. C........C

            6231
K-12         GGCAGTTGCG GCTATAGCGG TGTGCCACGT ATGAAGAATC TCTGCCAACT GATCGAACAA CAGCTACGTA
53638        .......... .......... ......G... .......... .......... .......... ..........
ATCC_8739    .......... .......... ......G... .......... .......... .......... ..........
HS           .......... .......... .......... .......... .......... .......... ..........
BW2952       .......... .......... .......... .......... .......... .......... ..........
12009        .......... .......... ......G... .......... .......... .......... ..........
B171         .......... .......... ......G... .......... .......... .......... ..........
E24377A      .......... .......... ......G... .......... .......... .......... ..........
SE11         .......... .......... ......G... .......... .......... .......... ..........
11128        .......... .......... ......G... .......... .......... .......... ..........
11368        .......... .......... ......G... .......... .......... .......... ..........
IAI1         .......... .......... ......G... .......... .......... .......... ..........
E110019      .......... .......... ......G... .......... .......... .......... ..........
B7A          .......... .......... ......G... .......... .......... .......... ..........
CB9615       .......... .......... ......G... .......... .......... T......... ..........
O157_Sakai   .......... .......... ......G... .......... .......... T......... ..........
IAI39        .......... .......... ......G... .......... .......... .......... ..........
UMN026       .......... .......... ......G... .......... .......... .......... ..........
SMS-3-5      .......... .......... ......G... .....A.... .......... .......... ..........
536          .......... .......... ...A..G... .......... .......... .......... ..........
APEC_O1      .......... .......... ...A..G... .......... .......... .......... ..........
CFT073       .......... .......... ...A..G... .......... .......... .......... ..........
E2348/69     .......... .......... ......G... .........A .......... .......... ..........
UTI89        .......... .......... ...A..G... .......... .......... .......... ..........
F11          .......... .......... ...A..G... .......... .......... .......... ..........
ED1a         .......... .......... ...A..G... .......... .......... .......... ..........
83972        .......... .......... ...A..G... .......... .......... .......... ..........
S88          .......... .......... ...A..G... .......... .......... .......... ..........
E._albertii  .......... .T........ C..A..G... .......... .......... C......... ..A.......
```

```
           6301
K-12        GCGGTACTAA AGAAGAAGAT TTGGAACCGG AGCTGCTGGA ACTGTTGGAC GAGATGGATA ATGTCGCGCG
53638       .......... .......... .....G.... .......... .......... .......... ..........
ATCC_8739   .......... .......... .....G.... .......... .......... .......... ..........
HS          .......... .......... .......... .......... .......... .......... ..........
BW2952      .......... .......... .......... .......... .......... .......... ..........
12009       .......... .......... .....G.... .......... .......... .......... ....A.....
B171        .......... .......... .....G.... .......... .......... .......... ....A.....
E24377A     .......... .......... .....G.... .......... .......... .......... ....A.....
SE11        .......... .......... .....G.... .......... .......... .......... ....A.....
11128       .......... .......... .....G.... .......... .......... .......... ....A.....
11368       .......... .......... .....G.... .......... .......... .......... ....A.....
IAI1        .......... .......... .....G.... .......... .......... .......... ....A.....
E110019     .......... .......... .....G.... .......... .......... .......... ....A.....
B7A         .......... .......... .....G.... .......... .......... .......... ....A.....
CB9615      .......... .......... .......... .......... .......... .......... ..........
O157_Sakai  .......... .......... .......... .......... .......... .......... ..........
IAI39       .......... .......... .....G.... .......... ......A... .......... ....A.....
UMN026      .......... .......... .....G.... .......... .......... .......... ....A.....
SMS-3-5     .......... .......... .....G.... .......... ......A... .......... ....A.....
536         A......... .......... ..A..G.... .......... .......... .......... ....A.....
APEC_O1     .......... .......... ..A..G.... .......... .......... .......... ....A.....
CFT073      .......... .......... ..A..G.... .......... .......... .......... ....A.....
E2348/69    .......... .......... ..A..G.... .......... .......... .......... ....A.....
UTI89       .......... .......... ..A..G.... .......... .......... .......... ....A.....
F11         A......... .......... ..A..G.... .......... .......... .......... ....A.....
ED1a        .......... .......... ..A..G.... .......... .......... .......... ....A.....
83972       .......... .......... ..A..G.... .......... .......... .......... ....A.....
S88         .......... .......... ..A..G.... .......... .......... .......... ....A.....
E._albertii .......C.. .......... C.......T. .AT....... ....C..... .......... .C..G.....

           6371
K-12        CGAAGCCAGC AAAATTCTCG GGTAATGGAT GTCTTGTCGA GTAGCAATCG GCTTTTTATG ACGCTGGTTT
53638       .......... .......... .....C...C .......... .......... .......... ..........
ATCC_8739   .......... .......... .....C...C .......... .........A .......... ..........
HS          .......... .......... .......... .......T.. .......... .......... ..........
BW2952      .......... .......... .......... .......... .......... .......... ..........
12009       .......... .......... .....C...C .......... .......... .......... ..........
B171        .......... .......... .....C...C .......... .......... .......... ..........
E24377A     .......... .......... .....C...C .......... .......... .......... ..........
SE11        .......... .......... .....C...C .......... .......... .......... ..........
11128       .......... .......... .....C...C .......... .......... .......... ..........
11368       .......... .......... .....C...C .......... .......... .......... ..........
IAI1        .......... .......... .....C...C .......... .......... .......... ..........
E110019     .......... .......... .....C...C .......... .......... .......... ..........
B7A         .......... .......... .....C...C .......... .......... .......... ..........
CB9615      .......... .......... .....C...C .......... .......... .......... ......T...
O157_Sakai  .......... .......... .....C...C .......... .......... .......... ......T...
IAI39       .......... .......... .....C...C .......... .......... .......... .A........
UMN026      .......... .......... .........C .......... .......... .......... ..........
SMS-3-5     .......... .......... .....C...C .......... .......... .......... .A........
536         .......... .......... .........C .......... .......C.. .A........ ..........
APEC_O1     .......... .......... .........C .......... .......C.. .A........ ..........
CFT073      .......... .......... .........C .......... .......C.. .A........ ..........
E2348/69    .......... .......... .........C .......... .......C.. .A........ ..........
UTI89       .......... .......... .........C .......... .......C.. .A........ ..........
F11         .......... .......... .........C .......... .......C.. .A........ ..........
ED1a        .......... .......... .........C .......... .......C.. .A........ ..........
83972       .......... .......... .........C .......... .......C.. .A........ ..........
S88         .......... .......... .........C .......... .......C.. .A........ ..........
E._albertii T......... ........T. .....----- ---------- ---------- ---------- ----------
```

```
           6441
K-12         CAGATTGGTG ACAAAGTGCC TGTGTTTATG CCGGATGCGG CGTTAACGCC TTATCCGGCC TACAAAACCA
53638        .......... .......... .......... .......... .......... .......... ..........
ATCC_8739    .......... .......... .......... .......... .......... .......... ..........
HS           .......... .......... .......... .......... .......... .......... ..........
BW2952       .......... .......... .......... .......... .......... .......... ..........
12009        .......... .......... .......... .......... .......... .......... ..........
B171         .......... .......... .......... .......... .......... .......... ..........
E24377A      .......... .......... .......... .......... .......... .......... ..........
SE11         .......... .......... .......... .......... .......... .......... ..........
11128        .......... .......... .......... .......... .......... .......... ..........
11368        .......... .......... .......... .......... .......... .......... ..........
IAI1         .......... .......... .......... .......... .......... .......... ..........
E110019      .......... .......... .......... .......... .......... .......... ..........
B7A          .......... .......... .......... .......... .......... .......... ..........
CB9615       .......... .......... .......... .......... .......... .......... ..........
O157_Sakai   .......... .......... .......... .......... .......... .......... ..........
IAI39        ....C..... .......... .......... .......... .......... ......T... ..........
UMN026       .......... ....G..... .......... .......... .......... .......... ..........
SMS-3-5      ....C..... .......... .......... .......... .......... ......T... ..........
536          .......... ....-..... .......... .......... .......... .......... .G........
APEC_O1      .......... ....-..... .......... .......... .......... .......... .G........
CFT073       .......... ....-..... .......... .......... .......... .......... .G........
E2348/69     .......... .A..-..... .......... .......... .......... .......... .G........
UTI89        .......... ....-..... .......... .......... .......... .......... .G........
F11          .......... ....-..... .......... .......... .......... .......... .G........
ED1a         ......A... ....-..... .......... .......... .......... .......... .G........
83972        .......... ....-..... .......... .......... .......... .......... .G........
S88          .......... ....-..... .......... .......... .......... .......... .G........
E._albertii  ---------- ---------- ---------- ---------- ---------- ---------- ----------

           6511
K-12         ATTAAATTCA ATGAATTAAA AAATGATGTA GACCTGATA- -CATAGCACA ACAGGCTATT TTGCGTTTAG
53638        .......... ..A....... ..GC-..... .G.......A G.G....G.. T.....A... ...T....G.
ATCC_8739    .......... ..A....... ..GC-..... .G.......A G.G....G.. T.....A... ...T....G.
HS           .......... .......... .......... .......... .......... .......... ..........
BW2952       .......... .......... .......... .......... .......... .......... ..........
12009        .......... .......... .......... .......... .......... .......... ..........
B171         .......... .......... .......... .......... .......... .......... ..........
E24377A      .......... .......... .......... .......... .......... .......... ..........
SE11         .......... .......... .......... .......... .......... .......... ..........
11128        .......... .......... .......... .......... .......... .......... ..........
11368        .......... .......... .......... .......... .......... .......... ..........
IAI1         .......... .......... .......... .......... .......... .......... ..........
E110019      .......... .......... .......... .......... .......... .......... ..........
B7A          .......... .......... .......... .......... .......... .......... ..........
CB9615       .......... ..A....... ..GC-..... .G.......A G.G....G.. T.....A... ...T....G.
O157_Sakai   .......... ..A....... ..GC-..... .G.......A G.G....G.. T.....A... ...T....G.
IAI39        .......... .......... ..G.-....T .G........ ..G....G.. ......A... ....T...G.
UMN026       .......... .......... ..G.-....T .G........ ..G....G.. ......A... ........G.
SMS-3-5      .......... .......... ..G.-....T .G........ ..G....G.. ......A... ........G.
536          .......... .......... .......... .C........ ..G....G.. ......A... ........G.
APEC_O1      .......... .......... .......... .C........ ..G....G.. ......A... ........G.
CFT073       .......... .......... .......... .C........ ..G....G.. ......A... ........G.
E2348/69     .....G.... .......... .......... AC........ ..G....G.. ......A... ........G.
UTI89        .......... .......... .......... .C........ ..G....G.. ......A... ........G.
F11          .......... .......... .......... .C........ ..G....G.. ......A... ........G.
ED1a         .......... .......... .......... .C........ ..G....G.. ......A... ........G.
83972        .......... .......... .......... .C........ ..G....G.. ......A... ........G.
S88          .......... .......... .......... .C........ ..G....G.. ......A... ........G.
E._albertii  ---------- ---------- ---------- ---------. .--------- ---------- ----------
```

```
              6581
K-12         CATCAGTCTC AAACCGGCTC CAGATAGAGC CGGTTTTGGT TTTCTGTCTT AACGCACCAT GCACGGGCGC
53638        .......... .......... .T..C..... .......... .......... .......... ..........
ATCC_8739    .......... .......... .T..C..... .......... .......... .......... ..........
HS           .......... .......... .......... .......... .......... .......... ......A...
BW2952       .......... .......... .......... .......... .......... .......... ..........
12009        .......... ..G....... .......... .......... .......... .......... ..........
B171         .......... ..G....... .......... .......... .......... .......... ..........
E24377A      .......... ..G....... .......... .......... .......... .......... ..........
SE11         .......... ..G....... .......... .......... .......... .......... ..........
11128        .......... ..G....... .......... .......... .......... .......... ..........
11368        .......... ..G....... .......... .......... .......... .......... ..........
IAI1         .......... ..G....... .......... ..A....... .......... .......... ..........
E110019      .......... ..G....... .......... .......... .......... .......... ..........
B7A          .......... ..G....... .......... .......... .......... .......... ..........
CB9615       .......... .......... .......... .......... ...T...... .......... ..........
O157_Sakai   .......... .......... .......... .......... ...T...... .......... ..........
IAI39        .......... .......... .T..C..... .......T.. ....A..... .......... ..........
UMN026       .......... .......... .T..A..... .......... .......... .......... ..........
SMS-3-5      .......... .......... .T..C..... .......T.. ....A..... .......... ..........
536          .......... .......... .......... .......... .......... .......... ......A...
APEC_O1      .......... .......... .......... .......... .......... .......... ......A...
CFT073       .......... .......... .......... .......... .......... .......... ......A...
E2348/69     .......... .......... .......... .......... .......... .......... ......A...
UTI89        .......... .......... .......... .......... .......... .......... ......A...
F11          .......... .......... .......... .......... .......... .......... ......A...
ED1a         .......... .......... .T..C..... .......T.. ....A..... .......... ..........
83972        .......... .......... .......... .......... .......... .......... ......A...
S88          .......... .......... .......... .......... .......... .......... ......A...
E._albertii  ---------- ---------- ---------- ---------- ---------- ---------- ----------

              6651
K-12         TTGTTATCGA ACGTCCAGCC AGGAATCAGA TACTGCATTC CCATCGCATC GTCACGCGCG CCAAGCCCGT
53638        .......... .......... .......... .......... .......... .......... ..........
ATCC_8739    .......... .......... .......... .......... .......... .......... ..........
HS           .......... .......... .......... .......... .......... .......... ..........
BW2952       .......... .......... .......... .......... .......... .......... ..........
12009        .......... .......... .......... .......... .......... .......... ..........
B171         .......... .......... .......... .......... .......... .......... ..........
E24377A      .......... .......... .......... .......... .......... .......... ..........
SE11         .......... .......... .......... .......... .......... .......... ..........
11128        .......... .......... .......... .......... .......... .......... ..........
11368        .......... .......... .......... .......... .......... .......... ..........
IAI1         .......... .......... .......... .......... .......... .......... ..........
E110019      .......... .......... .......... .......... .......... .......... ..........
B7A          .......... .......... .......... .......... .......... .......... ..........
CB9615       .......... .......... .......... .......... .......... .......... ..........
O157_Sakai   .......... .......... .......... .......... .......... .......... ..........
IAI39        ........A. .......... .........G .......... .......... .......... ..........
UMN026       ........A. .......... .........G .......... .......... .........A ........A.
SMS-3-5      ........A. .......... .........G .......... .......... .........A ........A.
536          .......... .......... G..G.....G .......... .......... .......... ..........
APEC_O1      .......... .......... G..G.....G .......... .......... .......... ..........
CFT073       .......... .......... G..G.....G .......... .......... .......... ..........
E2348/69     .......... .......... G..G.....G .......... .......... .......... ..........
UTI89        .......... .......... G..G.....G .......... .......... .......... ..........
F11          .......... .......... G..G.....G .......... .......... .......... ..........
ED1a         ........A. .......... .........G .......... .......... .......... ..........
83972        .......... .......... G..G.....G .......... .......... .......... ..........
S88          .......... .......... G..G.....G .......... .......... .......... ..........
E._albertii  ---------- ---------- ---------- ---------- ---------- ---------- ----------
```

```
          6721
K-12          GTTTCTGATA CAGCTCATGG GCTTTCATCA CTTGATCCAT ATCGATTTCT ACACCCAGCC CCGGTTTTTC
53638         .......... .......... .......... .......... .......... .......... ..........
ATCC_8739     .......... .......... .......... .......... .......... ........T. ..........
HS            .......... .......... .......... .......... .......... .......... ..........
BW2952        .......... .......... .......... .......... .......... .......... ..........
12009         .......... .......... .......... .......... .......... .......... ..........
B171          .......... .......... .......... .......... .......... .......... ..........
E24377A       .......... .......... .......... .......... .......... .......... ..........
SE11          .......... .......... .......... .......... .......... .......... ..........
11128         .......... .......... .......... .......... .......... .......... ..........
11368         .......... .......... .......... .......... .......... .......... ..........
IAI1          .......... .......... .......... .......... .......... .......... ..........
E110019       .......... .........A .......... .......... .......... .......... ..........
B7A           .......... .........A .......... .......... .....C.... .......... ..........
CB9615        .......... .......... .......... .......... .......... .......... ..........
O157_Sakai    .......... .......... .......... .......... .......... .......... ..........
IAI39         .......... ......G... .......... .C........ .......... ..G....AT. .T.......G
UMN026        .......... ......G... .......... .C........ .......... ..G....AT. .T.......G
SMS-3-5       .......... ......G... .......... .C........ .......... ..G....AT. .T.......G
536           .......... ......G..C .......... .......... .......... .......... ..........
APEC_O1       .......... ......G..C .......... .......... .......... .......... ..........
CFT073        .......... ......G..C .......... .......... .......... .......... ..........
E2348/69      .......... ......G..C .......... .......... .......... .......... ..........
UTI89         .......... ......G..C .......... .......... .......... .......... ..........
F11           .......... ......G..C .......... .......... .......... .......... ..........
ED1a          .......... ......G... .......... .C........ .......... ..G....AT. .T.......G
83972         .......... ......G..C .......... .......... .......... .......... ..........
S88           .......... ......G..C .......... .......... .......... .......... ..........
E._albertii   ---------- ---------- ---------- ---------- ---------- ---------- ----------

          6791
K-12          TGGCACCTGT ACCAGCCCGC CTTTGATCTC AAACGGTTCT TTGGTCAGGC GCTGATTGCC TTCCTGCCAA
53638         .......... .......... .......... .......... .......... .......A.. .........G
ATCC_8739     .......... .......... .......... .......... .......... .......A.. .........G
HS            .......... .......... .......... .......... .......... .......... ..........
BW2952        .......... .......... .......... .......... .......... .......... ..........
12009         .......... .......... .......... .......... .......... .......A.. .........G
B171          .......... .......... .......... .......... .......... .......A.. .........G
E24377A       .......... .......... .......... .......... .......... .......A.. .........G
SE11          .......... .......... .......... .......... .......... .......A.. .........G
11128         C........A .....A.... .......... .......... .......... .......A.. .........G
11368         C........A .....A.... .......... .......... .......... .......A.. .........G
IAI1          .......... .......... .......... .......... .......... .......A.. .........G
E110019       C........A .....A.... .......... .......... .......A.. .......... ..........
B7A           C........A .....A.... .......... .......... .......A.. .......... ..........
CB9615        .......... .......... .......... .......... .......... .......A.. .........G
O157_Sakai    .......... .......... .......... .......... .......... .......A.. .........G
IAI39         C......... .......... .......... .......... ........A. .......... .........G
UMN026        C......... .......... .......... .......... ........A. .......... .........G
SMS-3-5       C......... .......... .......... .......... ........A. .......... .........G
536           .........A .....A.... .......... T......... .......... .......A.. .........G
APEC_O1       .........A .....A.... .......... T......... .......... .......A.. .........G
CFT073        .........A .....A.... .......... T......... .......... .......A.. .........G
E2348/69      .........A .....A.... .......... T......... ........A. .......... .........G
UTI89         .........A .....A.... .......... T......... .......... .......A.. .........G
F11           .........A .....A.... .......... T......... .......... .......A.. .........G
ED1a          C......... .......... .......... .......... ........A. .......... .........G
83972         .........A .....A.... .......... T......... .......... .......A.. .........G
S88           .........A .....A.... .......... T......... .......... .......A.. .........G
E._albertii   ---------- ---------- ---------- ---------- ---------- ---------- ----------
```

```
           6861
K-12         ATCCAGTGCG TATCAATAGC AGTAATTTTA CCCGGTGCAG CGGCGGCAAC ATGGGTAAAC ATCGCCAGGG
53638        .......... .......... .......... .......... .......... .......... ..........
ATCC_8739    .......... .......... .......... .......... .......... .......... ..........
HS           .......... .......... .......... .......... .......... .......... ..........
BW2952       .......... .......... .......... .......... .......... .......... ..........
12009        .......... .......... .......... .......... .......... .......... ..........
B171         .......... .......... .......... .......... .......... .......... ..........
E24377A      .......... .......... .......... .......... .......... .......... ..........
SE11         .......... .......... .......... .......... .......... .......... ..........
11128        .......... .......... .......... .......... .......... .......... ..........
11368        .......... .......... .......... .......... .......... .......... ..........
IAI1         .......... .......... .......... .......... .......... .......... ..........
E110019      .......... .......C.. G..G...... .......... .......... .......... ..........
B7A          .......... .......C.. G..G...... .......... .......... .......... ..........
CB9615       .......... .......... .......... .......... .......... .......... ..........
O157_Sakai   .......... .......... .......... .......... .......... .......... ..........
IAI39        .......... .......... .......... .......... .A....T... .......... ..........
UMN026       .......... .......... .......... .......... .A........ G......... ..........
SMS-3-5      .......... .......... .......... .......... .A....T... .......... ........A.
536          .......... .......C.. G..G...... ........G. .A........ .......... ..........
APEC_O1      .......... .......C.. G..G...... ........G. .A........ .......... ..........
CFT073       .......... .......C.. G..G...... ........G. .A........ .......... ..........
E2348/69     .......... .......... .......... .......... .A....T... .......... ..........
UTI89        .......... .......C.. G..G...... ........G. .A........ .......... ..........
F11          .......... .......C.. G..G...... ........G. .A........ .......... ..........
ED1a         .......... .......C.. .......... .......... .A....T... .......... ..........
83972        .......... .......C.. G..G...... ........G. .A........ .......... ..........
S88          .......... .......C.. G..G...... ........G. .A........ .......... ..........
E._albertii  ---------- ---------- ---------- ---------- ---------- ---------- ----------

           6931
K-12         AAATATCGAA GTGGTTGTTA GAGTGTGAAC CCCAGGTCAG GCCAAATTCA TGGCACATTT GCGCCACACG
53638        .......... .........T .......... .......... .......... .......... ..........
ATCC_8739    .......... .........T .......... .......... .......... .......... ..........
HS           .......... .......... .......... .......... .......... .......... ..........
BW2952       .......... .......... .......... .......... .......... .......... ..........
12009        .......... .........T .......... .......... .......... .......... ..........
B171         .......... .........T .......... .......... .......... .......... ..........
E24377A      .......... .........T .......... .......... .......... .......... ..........
SE11         .......... .........T .......... .......... .......... .......... ..........
11128        .......... .........T .......... .......... .......... .......... ..........
11368        .......... .........T .......... .......... .......... .......... ..........
IAI1         .......... .........T .......... .......... .......... .......... ..........
E110019      .......... .........T .......... .......... .......... .......... ..........
B7A          .......... .........T .......... .......... .......... .......... ..........
CB9615       .......... .......... .......... .......... .......... .......... ..........
O157_Sakai   .......... .......... .......... .......... .......... .......... ..........
IAI39        .......... .......... .......... .......... .......... .......... ..........
UMN026       .......... A..A...... .......... .......... .......... .......... ..........
SMS-3-5      .......... .......... .......... .......... .......... .......... ..........
536          .......... .........G .......... .......... .......... ..A....... ..........
APEC_O1      .......... .........G .......... .......... .......... ..A....... ..........
CFT073       .......... .........G .......... .......... .......... ..A....... ..........
E2348/69     .......... .......... .......... .......... .......... .......... ..........
UTI89        .......... .........G .......... .......... .......... ..A....... ..........
F11          .......... .........G .......... .......... .......... ..A....... ..........
ED1a         .......... .......... .......... .......... .......... .......... ..........
83972        .......... .........G .......... .......... .......... ..A....... ..........
S88          .......... .........G .......... .......... .......... ..A....... ..........
E._albertii  ---------- ---------- ---------- ---------- ---------- ---------- ----------
```

```
             7001
K-12         TACCGAACCT TGCATTGTCC AGAAATGCGG ATCCGCCAGC GGGATATCAA CGGATTGCAG GGAGAGCGTA
53638        .......... .....C.... .......... .......... .......... .......T.A C.....T...
ATCC_8739    .......... .....C.... .......... .......... .......... .......T.A C.....T...
HS           .......... .......... .......... .......... .......... .......... ..........
BW2952       .......... .......... .......... .......... .......... .......... ..........
12009        .......... .....C.... .......... .......... .......... .......... ..........
B171         .......... .....C.... .......... .......... .......... .......... ..........
E24377A      .......... .....C.... .......... .......... .......... .......... ..........
SE11         .......... .....C.... .......... .......... .......... .......... ..........
11128        .......... .....C.... .......... .......... .......... .......... ..........
11368        .......... .....C.... .......... .......... .......... .......... ..........
IAI1         .......... .....C.... .......... .......... .......... .......... ..........
E110019      .......... .....C.... .......... .......... .......... .......... ..........
B7A          .......... .....C.... .......... .......... .......... .......... ..........
CB9615       .......... .......... .......... .......... .......... .......T.A C.........
O157_Sakai   .......... .......... .......... .......... .......... .......T.A C.........
IAI39        .......... .....C.... .......... .......... .......... .......... ..........
UMN026       .......... .....C.... .......... .......... .......... .......... ..........
SMS-3-5      .......... .....C.... .......... .......... .......... .......... ..........
536          .......... .....C.... .......... ......A... .......... .........A C.........
APEC_O1      .......... .....C.... .......... ......A... .......... .........A C.........
CFT073       .......... .....C.... .......... ......A... .......... .........A C.........
E2348/69     .......... .....C.... .......... .......... .......... .......T.A C.........
UTI89        .......... .....C.... .......... ......A... .......... .........A C.........
F11          .......... .....C.... .......... ......A... .......... .........A C.........
ED1a         .......... .....C.... .......... .......... .......... .........A C.........
83972        .......... .....C.... .......... ......A... .......... .........A C.........
S88          .......... .....C.... .......... ......A... .......... .........A C.........
E._albertii  ---------- ---------- ---------- ---------- ---------- ---------- ----------

             7071
K-12         TGGCCCATTT GCCGCCAGTC GGTGGCGATC ATATTGGTTG CAGTCGGTAG ACCTGTCGCG CGACGGAACT
53638        ........C. .......... .......... .......... .......C.. G..A...... ..........
ATCC_8739    ........C. .......... .......... .......... .......C.. G..A...... ..........
HS           .......... .......... .......... .......... .......C.. .......... ..........
BW2952       .......... .......... .......... .......... .......... .......... ..........
12009        ........C. .......... .......... .......... .G.....C.. G..A..T... ..........
B171         ........C. .......... .......... .......... .G.....C.. GT.A..T... ..........
E24377A      ........C. .......... .......... .......... .G.....C.. G..A..T... ..........
SE11         ........C. .......... .......... .......... .G.....C.. G..A..T... ..........
11128        ........C. .......... .......... .......... .G.....C.. G..A..T... ..........
11368        ........C. .......... .......... .......... .G.....C.. G..A..T... ..........
IAI1         ........C. .......... .......... .......... .G.....C.. G..A..T... ..........
E110019      ........C. .......... .......... .......... .G.....C.. G..A..T... ..........
B7A          ........C. .......... .......... .......... .G.....C.. G..A..T... ..........
CB9615       ........C. .......... .......... .......... .......C.. G..A...... ..........
O157_Sakai   ........C. .......... .......... .......... .......C.. G..A...... ..........
IAI39        ........C. .......... .......... .......... .G.....C.. G......... ..........
UMN026       ........C. .......... .......... .......... .......C.. .......... ..........
SMS-3-5      ........C. .......... .......... .......... .G.....C.. G..A...... ..........
536          ........C. .......... .......... .......... .G.....C.. G..A..T... ..........
APEC_O1      ........C. .......... .......... .......... .G.....C.. G..A..T... ..........
CFT073       ........C. .......... .......... .......... .G.....C.. G..A..T... ..........
E2348/69     ........C. .......... .......... .......... .G.....C.. G..A...... ..........
UTI89        ........C. .......... .......... .......... .G.....C.. G..A..T... ..........
F11          ........C. .......... .......... .......... .G.....C.. G..A..T... ..........
ED1a         ........C. .......... .......... .......... .G.....C.. G..A...... ..........
83972        ........C. .......... .......... .......... .G.....C.. G..A..T... ..........
S88          ........C. .......... .......... .......... .G.....C.. G..A..T... ..........
E._albertii  ---------- ---------- ---------- ---------- ---------- ---------- ----------
```

```
           7141
K-12         CTGCCATCAC TTCACGCCCG GAGAAACCTT GCTCCGCACC ACACGGATCT TCTGCATAAG CCAGCGAACC
53638        .......... .......... .......... .......... .......... .......... .......C..
ATCC_8739    .......... .......... .......... .......... .......... .......... .......C..
HS           .......... .......... .......... .......... .......... .......... ....T..C..
BW2952       .......... .......... .......... .......... .......... .......... ..........
12009        .......... .......... .......... .......... .......... .......... .......C..
B171         .......... .......... .......... .......... .......... .......... .......C..
E24377A      .......... .......... .......... .......... .......... .......... .......C..
SE11         .......... .......... .......... .......... .......... .......... .......C..
11128        .A........ .......... .......... .......... .......... .......... .......C..
11368        .A........ .......... .......... .......... .......... .......... .......C..
IAI1         .......... .......... .......... .......... .......... .......... .......C..
E110019      .......... .......... .......... .......... .......... .......... .......C..
B7A          .......... .......... .......... .......... .......... .......... .......C..
CB9615       .......... .......... .......... .......... .......... .......... ..........
O157_Sakai   .......... .......... .......... .......... .......... .......... ..........
IAI39        .......... .......... .......... .......... .......... .......... ..........
UMN026       .......... .......... .......... .......... .......... .......... ..........
SMS-3-5      .......... .......... .......... .......... .......... .......... ..........
536          .......... .......... .......... .......... .......... .......... ..........
APEC_O1      .......... .......... .......... .......... .......... .......... ..........
CFT073       .......... .......... .......... .......... .......... .......... ..........
E2348/69     .......... .......... .......... .......... .......... .......... ..........
UTI89        .......... .......... .......... .......... .......... .......... ..........
F11          .......... .......... .......... .......... .......... .......... ..........
ED1a         .......... .......... .......... .......... .......... .......... ..........
83972        .......... .......... .......... .......... .......... .......... ..........
S88          .......... .......... .......... .......... .......... .......... ..........
E._albertii  ---------- ---------- ---------- ---------- ---------- ---------- ----------

           7211
K-12         TTTCAGGTAT TTACCGATTT TAATCGCTTC GTTCAGCGAC CAGGCACCGT TAGGATCGAG CGTAATACGC
53638        .......... .....A.... .......... .......... .......... .......... ..........
ATCC_8739    .......... .....A.... .......... .......... .......... .......... ..........
HS           .......... .....A.... .......... .......... .......... .......... ..........
BW2952       .......... .......... .......... .......... .......... .......... ..........
12009        .......... .....A.... .......... .......... .......... .......... ..........
B171         .......... .....A.... .......... .......... .......... .......... ..........
E24377A      .......... .....A.... .......... .......... .......... .......... ..........
SE11         .......... .....A.... .......... .......... .......... .......... ..........
11128        .......... .....A.... .......... .......... .......... .......... ..........
11368        .......... .....A.... .......... .......... .......... .......... ..........
IAI1         .......... .....A.... .......... .......... .......... .......... ..........
E110019      .......... .....A.... .......... .......... .......... .......... ..........
B7A          .......... .....A.... .......... .......... .......... .......... ..........
CB9615       .......... ..G....... .......... .......... .......... .......... ..........
O157_Sakai   .......... ..G....... .......... .......... .......... .......... ..........
IAI39        .......... G....A.... .......... .......... .......... .......... G.........
UMN026       .......... .....A.... .......C.. .......... .......... .......... G.........
SMS-3-5      .......... G....A.... .......... .......... .......... .......... G.........
536          .......... G......... .......... .......... .......... .......... ..........
APEC_O1      .......... G......... .......... T......... .......... .......... ..........
CFT073       .......... G......... .......... .......... .......... .......... ..........
E2348/69     .......... .....A.... .......... .......... .......... .......... G.........
UTI89        .......... G......... .......... T......... .......... .......... ..........
F11          .......... G......... .......... .......... .......... .......... ..........
ED1a         .......... .....A.... .......... .......... .......... .......... G.........
83972        .......... G......... .......... .......... .......... .......... ..........
S88          .......... G......... .......... T......... .......... .......... ..........
E._albertii  ---------- ---------- ---------- ---------- ---------- ---------- ----------
```

```
             7281
K-12          GCCTGCGGGA AGCGTTGCGC CAGTGCCACA ATAGACTCGG CCTCTTCTTC CCCGGCCAGT ACACCGCCCT
53638         .......... .A....T... .......... .......... .T........ .......... ........T.
ATCC_8739     .......... .A....T... .......... .......... .T........ .......... ........T.
HS            .......... .A....T... .......... .......... .T........ .......... ..........
BW2952        .......... .......... .......... .......... .......... .......... ..........
12009         .......... .......... .......... .......... .......... .......... ........T.
B171          .......... .......... .......... .......... .......... .......... ........T.
E24377A       .......... .......... .......... .......... .......... .......... ........T.
SE11          .......... .......... .......... .......... .......... .......... ........T.
11128         .......... .......... .......... .......... .......... .......... ........T.
11368         .......... .......... .......... .......... .......... .......... ........T.
IAI1          .......... .......... .......... .......... .......... .......... ........T.
E110019       .......... .......... .......... .......... .......... .......... ........T.
B7A           .......... .......... .......... .......... .......... .......... ........T.
CB9615        .......... .......... .......... .......... .......... .......... ........T.
O157_Sakai    .......... .......... .......... .......... .......... .......... ........T.
IAI39         .....T.... .......... .......... .....T..T. .T........ .......... ..........
UMN026        .....T.... .......... .......... .....T..T. .T........ .......... ..........
SMS-3-5       .....T.... .......... .......... ........T. .T........ .......... ..........
536           .......... .A........ .......... .....T.... .T........ .......... ........T.
APEC_O1       .......... .A........ .......... .....T.... .T........ .......... ........T.
CFT073        .......... .A........ .......... .....T.... .T........ .......... ........T.
E2348/69      .....T.... .......... .......... .....T..T. .T........ .......... .....A....
UTI89         .......... .A........ .......... .....T.... .T........ .......... ........T.
F11           .......... .A........ .......... .....T.... .T........ .......... ........T.
ED1a          .....T.... .......... .......... .....T..T. .T........ .......... ..........
83972         .......... .A........ .......... .....T.... .T........ .......... ........T.
S88           .......... .A........ .......... .....T.... .T........ .......... ........T.
E._albertii   ---------- ---------- ---------- ---------- ---------- ---------- ----------

             7351
K-12          TCAGTTTGAA ATCGTTGAAG CCATATTTTT CATATGCCGC TTCCGCCAGG CGCACCACCG CATCCGGCGT
53638         .......... .......... .......... ....C..... .......... .......... .......T..
ATCC_8739     .......... .......... .......... ....C..... .......... .......... .......T..
HS            .......... .......... .......... .......... .......... .......... ..........
BW2952        .......... .......... .......... .......... .......... .......... ..........
12009         .......... .......... .......... ....C..... .......... .......... ..........
B171          .......... .......... .......... ....C..... .......... .......... ..........
E24377A       .......... .......... .......... ....C..... .......... .......... ..........
SE11          .......... .......... .......... ....C..... .......... .......... ..........
11128         .......... .......... .......... ....C..... .......... .......... ..........
11368         .......... .......... .......... ....C..... .......... .......... ..........
IAI1          .......... .......... .......... ....C..... .......... .......... ..........
E110019       .......... .......... .......... ....C..... .......... .......... ..........
B7A           .......... .......... .......... ....C..... .......... .......... ..........
CB9615        .......... .......... .......... ....C..... .......... .......... ..........
O157_Sakai    .......... .......... .......... ....C..... .......... .......... ..........
IAI39         .......... .......... .......... .......... .......... .......... ..........
UMN026        .......... .......... .......... .......... .......... .......... ..........
SMS-3-5       .......... .......... .......... .......... .......... .......... ..........
536           .......... .......... .......... ....C..... .......... .......... ..........
APEC_O1       .......... .......... .......... ....C..... .......... .......... ..........
CFT073        .......... .......... .......... ....C..... .......... .......... ..........
E2348/69      .......... .......... .......... .......... .......... .......... ..........
UTI89         .......... .......... .......... ....C..... .......... .......... ..........
F11           .......... .......... .......... ....C..... .......... .......... ..........
ED1a          .......... .......... .......... .......... .......... .......... ..........
83972         .......... .......... .......... ....C..... .......... .......... ..........
S88           .......... .......... .......... ....C..... .......... .......... ..........
E._albertii   ---------- ---------- ---------- ---------- ---------- ---------- ----------
```

```
          7421
K-12        CATCGCTTCT TCATGACGCA GGCGATACCA GTCGCATGAG TCATCCGGCT GGCTTTGATA CGGCAGCGGC
53638       .......... .......... .......... .........A .......... .......... .........T
ATCC_8739   .......... .......... .......... .........A .......... .......... .........T
HS          ......G... .......... .......... .......... .......... .......... .........T
BW2952      .......... .......... .......... .......... .......... .......... ..........
12009       .......... .......... .......... .......... .......... .......... .........T
B171        .......... .......... .......... .......... .......... .......... .........T
E24377A     .......... .......... .......... .......... .......... .......... .........T
SE11        .......... .......... .......... .......... .......... .......... .........T
11128       .......... .......... .......... .......... .......... .......... .........T
11368       .......... .......... .......... .......... .......... .......... .........T
IAI1        .......... .......... .......... .......... .......... .......... .........T
E110019     .......... .......... .......... .......... .......... .......... .........T
B7A         .......... .......... .......... .......... .......... .......... .........T
CB9615      .......... .......... .......... .......... .......... .......... ......A..T
O157_Sakai  .......... .......... .......... .......... .......... .......... ......A..T
IAI39       .......... ..G....... .......... ...A...TGA .......... .......... .........T
UMN026      .......... ..G....... .......... ...A...TTA .......... .......... .........T
SMS-3-5     .......... ..G....... .......... ...A...TGA .......... .......... .........T
536         .......... .......... .......... .......... .......... .......... .........T
APEC_O1     .......... .......... .......... .......... .......... .......... .........T
CFT073      .......... .......... .......... .......... .......... .......... .........T
E2348/69    .......... ..G....... .......... ...A...TTA .......... .......... .........T
UTI89       .......... .......... .......... .......... .......... .......... .........T
F11         .......... .......... .......... .......... .......... .......... .........T
ED1a        .......... ..G....... .......... ...A...TTA .......... .......... .........T
83972       .......... .......... .......... .......... .......... .......... .........T
S88         .......... .......... .......... .......... .......... .......... .........T
E._albertii ---------- ---------- ---------- ---------- ---------- ---------- ----------

          7491
K-12        GTGGCTTTGC GATTACCGAC GAAGAACAGA TAACCGAGCA TTTCGACTTC GCTACGCTGT TGACCATCGC
53638       .......... .......... .......... .......... .......... .......... ..G.......
ATCC_8739   .......... .......... .......... .......... .......... .......... ..G.......
HS          .......... .......... .......... .......... .......... .......... ..........
BW2952      .......... .......... .......... .......... .......... .......... ..........
12009       .......... ...C...... .......... .......... .......... .......... ..........
B171        .......... ...C...... .......... .......... .......... .......... ..........
E24377A     .......... ...C...... .......... .......... .......... .......... ..........
SE11        .......... ...C...... .......... .......... .......... .......... ..........
11128       .......... ...C...... .......... .......... .......... .......... ..........
11368       .......... ...C...... .......... .......... .......... .......... ..........
IAI1        ....T..... ...C...... .......... .......... .......... .......... ..........
E110019     .......... ...C...... .......... .......... .......... .......... ..........
B7A         .......... ...C...... .......... .......... .......... .......... ..........
CB9615      .......... .......... .......... .......... .......... .......... ..........
O157_Sakai  .......... .......... .......... .......... .......... .......... ..........
IAI39       .......... .......... .......... .......... .......... .......... ..........
UMN026      .......... .......... .......... .......... .......... .......... ..........
SMS-3-5     .......... .......... .......... .......... .......... .......... ..........
536         ..A....... .......... .......... .......... .......... .......... ..........
APEC_O1     ..A....... ....G..... .......... .......... .......... .......... ..........
CFT073      ..A....... .......... .......... .......... .......... .......... ..........
E2348/69    .......... .......... .......... .......... .......... .......... ..........
UTI89       ..A....... ....G..... .......... .......... .......... .......... ..........
F11         ..A....... .......... .......... .......... .......... .......... ..........
ED1a        .......... .......... .......... .......... .......... .......... ..........
83972       ..A....... .......... .......... .......... .......... .......... ..........
S88         ..A....... ....G..... .......... .......... .......... .......... ..........
E._albertii ---------- ---------- ---------- ---------- ---------- ---------- ----------
```

```
           7561
K-12         CCAGCAGCGA TGCCACGTTT ACCCCCAGAT GCTGCCCCAG CAGATCCAGC ATTGCCGCTT CTATCCCGGT
53638        ......A... .......... .......... .........A .......... .......... ..........
ATCC_8739    ......A... .......... .......... .........A .......... .......... ..........
HS           .......... .......... .......... .......... .......... .......... ..........
BW2952       .......... .......... .......... .......... .......... .......... ..........
12009        .......G.. .......... .......... .......... .......... .......... ..........
B171         .......G.. .......... .......... .......... .......... .......... ..........
E24377A      .......G.. .......... .......... .......... .......... .......... ..........
SE11         .......G.. .......... .......... .......... .......... .......... ..........
11128        .......... .......... .......... .......... .......... .......... ..........
11368        .......... .......... .......... .......... .......... .......... ..........
IAI1         .......G.. .......... .......... .......... .......... .......... ..........
E110019      .......... .......... .......... .......... .......... .......... ..........
B7A          .......... .......... .......... .......... .......... .......... ..........
CB9615       .......G.. .........A .......... .......... .......... .......... ..........
O157_Sakai   .......G.. .........A .......... .......... .......... .......... ..........
IAI39        .......... .........A .......... .......... .......... .......... ..........
UMN026       .......... .........A .......... .......... .......... .......... ..........
SMS-3-5      .......... .........A .......... .......... .......... .......... ..........
536          .......... .......... .......... .......... .......... .......... ..........
APEC_O1      .......... .......... .......... .......... .......... .......... ..........
CFT073       .......... .......... .......... .......... .......... .......... ..........
E2348/69     .......... .........A .......... .......... .......... .......... ..........
UTI89        .......... .......... .......... .......... .......... .......... ..........
F11          .......... .......... .......... .......... .......... .......... ..........
ED1a         .......... .........A .......... .......... .......... .......... ..........
83972        .......... .......... .......... .......... .......... .......... ..........
S88          .......... .......... .......... .......... .......... .......... ..........
E._albertii  ---------- ---------- ---------- ---------- ---------- ---------- ----------

           7631
K-12         AACTACATGA ATAGTGGTAC GTAGGTCAAA TGTCTGCAAA CCGCGCCCAC CAGCATCACG ATCGGCAAAA
53638        .......... .......... .C.AA..... .......... ........G. .G........ ..........
ATCC_8739    .......... .......... .C.AA..... .......... ........G. .G........ ..........
HS           .......... .......... .C.AA..... .......... .......... .......... ..........
BW2952       .......... .......... .......... .......... .......... .......... ..........
12009        .......... .......... .C.AA..... .......... .......... .......... ..........
B171         .......... .......... .C.AA..... .......... .......... .......... ..........
E24377A      .......... .......... .C.AA..... .......... .......... .......... ..........
SE11         .......... .......... .C.AA..... .......... .......... .......... ..........
11128        .......... .......... .C.AA..... .......... .......... .......... ..........
11368        .......... .......... .C.AA..... .......... .......... .......... ..........
IAI1         .......... .......... .C.AA..... .......... .......... .......... ..........
E110019      .......... .......... .C.AA..... .......... .......... .......... ..........
B7A          .......... .......... .C.AA..... .......... .......... .......... ..........
CB9615       .......... .......... .C.AA..... .......... ........G. .G........ ..........
O157_Sakai   .......... .......... .C.AA..... .......... ........G. .G........ ..........
IAI39        ...C...... ........C. .C........ .......... ........G. .G........ .........T
UMN026       ...C...... ........C. .C........ .......... ........G. .G........ .........T
SMS-3-5      ...C...... ........C. .C........ .......... ........G. .G........ .........T
536          .......... .......... .C.AA..... .......... .......... .G........ ..........
APEC_O1      .......... .......... .C.AA..... .......... .......... .G........ ..........
CFT073       .......... .......... .C.AA..... .......... .......... .G........ ..........
E2348/69     .......... ........C. .C.AA..... .......... ........G. .G........ .........T
UTI89        .......... .......... .C.AA..... .......... .......... .G........ ..........
F11          .......... .......... .C.AA..... .......... .......... .G........ ..........
ED1a         ...C...... ........C. .C.AA..... .......... ........G. .G........ .........T
83972        .......... .......... .C.AA..... .......... .......... .G........ ..........
S88          .......... .......... .C.AA..... .......... .......... .G........ ..........
E._albertii  ---------- ---------- ---------- ---------- ---------- ---------- ----------
```

```
             7701
K-12         GTATTACGCA CCAGCGTCAG AACGTTTTTG TATTCACCCA GCGTTTTACC TACCACCAGC GGAATCGCAT
53638        .......... .......... .......... .......... .......... .......... ..........
ATCC_8739    .......... .......... .......... .......... .......... .......... ..........
HS           .......... .......... .......... .......... .......... C..T...... ..........
BW2952       .......... .......... .......... .......... .......... .......... ..........
12009        .......... .......... .......... .......... .......... .......... ..........
B171         .......... .......... .......... .......... .......... .......... ..........
E24377A      .......... .......... .......... .......... .......... .......... ..........
SE11         .......... .......... .......... .......... .......... .......... ..........
11128        .......... .......... .......... .......... .......... .......... ..........
11368        .......... .......... .......... ....T..... .......... .......... ..........
IAI1         .......... .......... .......... .......... .......... .......... ..........
E110019      .......... .......... .......... .......... .......... .......... ..........
B7A          .......... .......... .......... .......... .......... .......... ..........
CB9615       .......... .......... .......... .......... .......... C..T...... .....T....
O157_Sakai   .......... .......... .......... .......... .......... C..T...... .....T....
IAI39        .......... .......... .......... .......... .......... C......... ..........
UMN026       .......... .......... .......... .......... .......... C......... ..........
SMS-3-5      .......... .......... .......... .......... .......... C......... ..........
536          .......... .......... .......... .......... .......... C......... ..........
APEC_O1      .......... .......... .......... .......... .......... C......... ..........
CFT073       .......... .......... .......... .......... .......... C......... ..........
E2348/69     .......... .......... .......... .......... .......... C......... ..........
UTI89        .......... .......... .......... .......... .......... C......... ..........
F11          .......... .......... .......... .......... .......... C......... ..........
ED1a         .......... .......... .......... .......... ..A....... C......... ..........
83972        .......... .......... .......... .......... .......... C......... ..........
S88          .......... .......... .......... .......... .......... C......... ..........
E._albertii  ---------- ---------- ---------- ---------- ---------- ---------- ----------

             7771
K-12         CTTCCAGCGT TTTACGGATT TTCTCGCCGC CGGGAATTTC CCCTACGCCA GTGTGACCAG AATTATCTTT
53638        .......... .......... .......... .......... .......... .......... ..........
ATCC_8739    .......... .......... .......... .......... .......... .......... ..........
HS           .......... .......... .......... .......... .......... .......... ..........
BW2952       .......... .......... .......... .......... .......... .......... ..........
12009        .......... .......... .......... .......... .......... .......... ..........
B171         .......... .......... .......... .......... .......... .......... ..........
E24377A      .......... .......... .......... .......... .......... .......... ..........
SE11         .......... .......... .......... .......... .......... .......... ..........
11128        .......... .......... .......... .......... .......... .......... ..........
11368        .......... .......... .......... .......... .......... .......... ..........
IAI1         .......... .......... .......... .......... .......... .......... ..........
E110019      .......... .......... .......... .......... .......... .......... ..........
B7A          .......... .......... .......... .......... .......... .......... ..........
CB9615       .......... .......... .......... .......... .......... .......... ..........
O157_Sakai   .......... .......... .......... .......... .......... .......... ..........
IAI39        .......... .......... .......... .......... ......C... .......... ..........
UMN026       .......... .......... .......... .......... .......... .......... ..........
SMS-3-5      .......... .......... .......... .......... ......C... .......... ..........
536          .......... .......... .......... .......... .......... ........G. ..........
APEC_O1      .......... .......... .......... .......... .......... ........G. ..........
CFT073       .......... .......... .......... .......... .......... ........G. ......T...
E2348/69     .......... .......... .......... .......... ......C... .......... ..........
UTI89        .......... .......... .......... .......... .......... ........G. ..........
F11          .......... .......... .......... .......... .......... ........G. ..........
ED1a         .......... .......... .......... .......... ......C... .......... ..........
83972        .......... .......... .......... .......... .......... ........G. ..........
S88          .......... .......... .......... .......... .......... ........G. ..........
E._albertii  ---------- ---------- ---------- ---------- ---------- ---------- ----------
```

```
            7841
K-12         -GATAATCAC AA-TATTACG CGTAAAGAAC GGTGCGTGTG CACCACTCAG ATTCATCAGC ATACTGTCAT
53638        .......... .......... ...G...... .......... .......... .......... ..........
ATCC_8739    .......... .......... ...G...... .......... .......... .......... ..........
HS           .......... .......... .......... .......... .......... .......... ..........
BW2952       .......... .......... .......... .......... .......... .......... ..........
12009        .......... .......... .......... .......... .......... .......... ..........
B171         .......... .......... .......... .......... .......... .......... ..........
E24377A      .......... .......... .......... .......... .......... .......... ..........
SE11         .......... .......... .......... .......... .......... .......... ..........
11128        .......... .......... .......... .......... .......... .......... ..........
11368        .......... .......... .......... .......... .......... .......... ..........
IAI1         .......... .......... .......... .......... .......... .......... ..........
E110019      .......... .......... .......... .......... .......... .......... ..........
B7A          .......... .......... .......... .......... .......... .......... ..........
CB9615       .......... .......... ...G...... .......... .......... .......... ..........
O157_Sakai   .......... .......... ...G...... .......... .......... .......... ..........
IAI39        .......... .......... ...G...... .......... .......... .......... ..........
UMN026       .......... .......... ...G...... .......... .......... .......... ..........
SMS-3-5      .......... .......... ...G...... .......... .......... .......... ..........
536          .......... .......... ...G...... .....A.... .......... .......... ..........
APEC_O1      .......... .......... ...G...... .....A.... .......... .......... ..........
CFT073       T......... ..A....... ...G...... .....A.... .......... .......... ..........
E2348/69     .......... .......... ...G...... .......... .......... .......... ..........
UTI89        .......... .......... ...G...... .....A.... .......... .......... ..........
F11          .......... .......... ...G...... .....A.... .......... .......... ..........
ED1a         .......... .......... ...G...... .......... .......... .......... ..........
83972        .......... .......... ...G...... .....A.... .......... .......... ..........
S88          .......... .......... ...G...... .....A.... .......... .......... ..........
E._albertii  .--------- --.------- ---------- ---------- ---------- ---------- ----------

            7911
K-12         GACCCGCCAC CGGGATAACC TGCATTTCAG TAACAACAGG CGTCGTAAAT TGAGAACTCA TATTTATGTC
53638        .......... ......G... .......... .......... .......... .......... ..........
ATCC_8739    .......... ......G... .......... .......... .......... .......... ..........
HS           .......... .......... .......... .......... .......... .......... ..........
BW2952       .......... .......... .......... .......... .......... .......... ..........
12009        .......... ......G... .......... .......... .......... .......... ..........
B171         .......... ......G... .......... .......... .......... .......... ..........
E24377A      .......... ......G... .......... .......... .......... .......... ..........
SE11         .......... ......G... .......... .......... .......... .......... ..........
11128        .......... ......G... .......... .......... .......... .......... ..........
11368        .......... ......G... .......... .......... .......... .......... ..........
IAI1         .......... ......G... .......... .......... .......... .......... ..........
E110019      .......... ......G... .......... .......... .......... .......... ..........
B7A          .......... ......G... .......... .......... .......... .......... ..........
CB9615       .G........ ......G... .......... .......... .......... .......... ..........
O157_Sakai   .G........ ......G... .......... .......... .......... .......... ..........
IAI39        .G........ ......G... .......... .......... .....A.... .......... .C........
UMN026       .G........ ......G... .......... .......... .......... .......... ..........
SMS-3-5      .G........ ......G... .......... .......... .....A.... .......... .C........
536          .......... ......G... .......... .......... .......... .......... ..........
APEC_O1      .......... ......G... .......... .......... .......... .......... ..........
CFT073       .......... ......G... .......... .......... .......... .......... ..........
E2348/69     .G........ ......G... .......... .......... .......... .......... ..........
UTI89        .......... ......G... .......... .......... .......... .......... ..........
F11          .......... ......G... .......... .......... .......... .......... ..........
ED1a         .G........ ......G... .......... .......... .......... .......... .C........
83972        .......... ......G... .......... .......... .......... .......... ..........
S88          .......... ......G... .......... .......... .......... .......... ..........
E._albertii  ---------- ---------- ---------- ---------- ---------- ---------- ----------
```

```
          7981
K-12        CTTTTTCAGA ATCAATGACG GCCGAAAACG GGACGTTTAC GGTCAAAGGT CCAGCCGGGG ATCAGGTACT
53638       .......... .......... .......... .......... .......... .......... ..........
ATCC_8739   .......... .......... .......... .......... .......... .......... ..........
HS          .......... .......... .......... .......... .......... .......... ..........
BW2952      .......... .......... .......... .......... .......... .......... ..........
12009       .......... .......... .......... .......... .......... .......... ..........
B171        .......... .......... .......... .......... .......... .......... ..........
E24377A     .......... .......... .......... .......... .......... .......... ..........
SE11        .......... .......... .......... .......... .......... .......... ..........
11128       .......... .......... .......... .......... .......... .......... ..........
11368       .......... .......... .......... .......... .......... .......... ..........
IAI1        .......... .......... .......... .......... .......... .......... ..........
E110019     .......... .......... .......... .......... .......... .......... ..........
B7A         .......... .......... .......... .......... .......... .......... ..........
CB9615      .......... .......... .......... .......... .......... .......... ..........
O157_Sakai  .......... .......... .......... .......... .......... .......... ..........
IAI39       ........A. .......... .......... .......... .......... .......... ..........
UMN026      ........A. .......... .......... .......... .......... .......... ..........
SMS-3-5     ........A. .......... .......... .......... .......... .......... ..........
536         .......... .......... .......... .......... .......... .......... ..........
APEC_O1     .......... .......... .......... .......... .......... .......... ..........
CFT073      .......... .......... .......... .......... .......... .......... ..........
E2348/69    ........A. .......... .......... .......... .......... .......... ..........
UTI89       .......... .......... .......... .......... .......... .......... ..........
F11         .......... .......... .......... .......... .......... .......... ..........
ED1a        ........A. .......... .......... .......... .......... .......... ..........
83972       .......... .......... .......... .......... .......... .......... ..........
S88         .......... .......... .......... .......... .......... .......... ..........
E._albertii ---------- ---------- ---------- ---------- ---------- ---------- ----------

          8051
K-12        GCATCGGACC TGCGTCGTTA CGCGCACCGC CAGGCAGACG TTTATAGGCC TCATGTGCCT TTTGTACCTG
53638       .......... .......... .......... .......... .......... .......... ..........
ATCC_8739   .......... .......... .......... .......... .......... .......... ..........
HS          .......... .......... .......... .......... .......... .......... ..........
BW2952      .......... .......... .......... .......... .......... .......... ..........
12009       .......... .......... .........A .......... .......... .......... ..........
B171        .......... .......... .........A .......... .......... .......... ..........
E24377A     .......... .......... .........A .......... .......... .......... ..........
SE11        .......... .......... .........A .......... .......... .......... ..........
11128       .......... .......... .......... .......... .......... .......... ..........
11368       .......... .......... .......... .......... .......... .......... ..........
IAI1        .......... .......... .........A .......... .......... .......... ..........
E110019     .......... .......... .......... .......... .......... .......... ..........
B7A         .......... .......... .......... .......... .......... .......... ..........
CB9615      .......G.. .......... .......... .......... .......... .......... ..........
O157_Sakai  .......G.. .......... .......... .......... .......... .......... ..........
IAI39       .......G.. C......... .......... .......... .......... .....C.... ..........
UMN026      .......G.. C......... .......... .......... .......... .....C.... ..........
SMS-3-5     .......G.. C......... .......... .......... .......... .....C.... ..........
536         .......G.. .......... .......... .......... .......... .......... ..........
APEC_O1     .......G.. .......... .......... .......... .......... .......... ..........
CFT073      .......G.. .......... .......... .......... .......... .......... ..........
E2348/69    .......G.. C......... .......... .......... .......... .....C.... ..........
UTI89       .......G.. .......... .......... .......... .......... .......... ..........
F11         .......G.. .......... .......... .......... .......... .......... ..........
ED1a        .......G.. C..A...... .......... .......... .......... .....C.... ..........
83972       .......G.. .......... .......... .......... .......... .......... ..........
S88         .......G.. .......... .......... .......... .......... .......... ..........
E._albertii ---------- ---------- ---------- ---------- ---------- ---------- ----------
```

```
            8121
K-12         TTCCCAGTCC AGTTCCACGC CCAGACCGGG CGCATCAGGA ACGGCAATTT TTCCGTTTTT AATCTCCAGC
53638        .......... .......... .......... .......... .......... .......... ..........
ATCC_8739    .......... .......... .......... .......... .......... .......... ..........
HS           .......... .......... .......... .......... .......... .......... ..........
BW2952       .......... .......... .......... .......... .......... .......... ..........
12009        .......... .......... .......... .......... .......... .......... ..........
B171         .......... .......... .......... .......... .......... .......... ..........
E24377A      .......... .......... .......... .......... .......... .......... ..........
SE11         .......... .......... .......... .......... .......... .......... ..........
11128        .......... .......... .......... .......... .......... .......... ..........
11368        .......... .......... .......... .......... .......... .......... ..........
IAI1         .......... .......... .......... .......... .......... .......... ..........
E110019      .......... .......... .......... .......... .......... .......... ..........
B7A          .......... .......... .......... .......... .......... .......... ..........
CB9615       .......... .......... .......... .......... .......... .......... ..........
O157_Sakai   .......... .......... .......... .......... .......... .......... ..........
IAI39        C......... .......... .......... .......... .......... ....A..... ..........
UMN026       C......... .......... .......... .......... .......... .......... ..........
SMS-3-5      C.......T. .......... .......... .......... .......... ....A..... ..........
536          .......... .......... .......... .......... .......... .......... ..........
APEC_O1      .......... .......... .......... .......... .......... .......... ..........
CFT073       .......... .......... .......... .......... .......... .......... ..........
E2348/69     C......... .......... .......... .......... .......... .......... ..........
UTI89        .......... .......... .......... .......... .......... .......... ..........
F11          .......... .......... .......... .......... .......... .......... ..........
ED1a         C......... .......... .......... .......... .......... .......... ..........
83972        .......... .......... .......... .......... .......... .......... ..........
S88          .......... .......... .......... .......... .......... .......... ..........
E._albertii  ---------- ---------- ---------- ---------- ---------- ---------- ----------

            8191
K-12         GGATTTTGGG TCAGGCGACA ATCGCCCTCC TGCCAAATCC AGTGGGTATC GATAGCGGTA GGATTACCCG
53638        .......... .......... .......... .......... .......... .......... ..........
ATCC_8739    .......... .......... .......... .......... .......... .......... ..........
HS           .......... .......... .......... .......... .......... .......... ..........
BW2952       .......... .......... .......... .......... .......... .......... ..........
12009        .......T.. .......... .......... .......... .......... .......... ..........
B171         .......T.. .......... .......... .......... .......... .......... ..........
E24377A      .......T.. .......... .......... .......... .......... .......... ..........
SE11         .......T.. .......... .......... .......... .......... .......... ..........
11128        .......T.. .......... .......... .......... .......... .......... ..........
11368        .......T.. .......... .......... .......... .......... .......... ..........
IAI1         .......... .......... .......... .......... .......... .......... ..........
E110019      .......T.. .......... .......... .......... .......... .......... ..........
B7A          .......T.. .......... .......... .......... .......... .......... ..........
CB9615       .......... .......... .......... .......... .......... .......... ..........
O157_Sakai   .......... .......... .......... .......... .......... .......... ..........
IAI39        .......T.. .......... .......... .......... .......... .......... ..........
UMN026       .......T.. .......... .......... .......... .......... .......... ..........
SMS-3-5      .......T.. .......... .......... .......... .......... .......... ..........
536          .......T.. ....A..... .......... .......... .......... .......... ..........
APEC_O1      .......T.. ....A..... .......... .......... .......... .......... ..........
CFT073       .......T.. ....A..... .......... .......... .......... .......... ..........
E2348/69     .......... .......... .......... .......... .......... .......... ..........
UTI89        .......T.. ....A..... .......... .......... .......... .......... ..........
F11          .......T.. ....A..... .......... .......... .......... .......... ..........
ED1a         .......T.. .......... ...T...... .......... .......... .......... ..........
83972        .......T.. ....A..... .......... .......... .......... .......... ..........
S88          .......T.. ....A..... .......... .......... .......... .......... ..........
E._albertii  ---------- ---------- ---------- ---------- ---------- ---------- ----------
```

```
           8261
K-12        GTGCCGCCGC GCCCACATGG GTAAACATCG CCAGAGAGAT ATCGAAATGG TTATTAGAAT GGCAGCCCCA
53638       .......... .......... .......... .......... .......... .......... ..........
ATCC_8739   .......... .......... .......... .......... .......... .......... ..........
HS          .......... .......... .......... .......... .......... .......... ..........
BW2952      .......... .......... .......... .......... .......... .......... ..........
12009       .......... .......... .......... .......... .......... .......... ..........
B171        .......... .......... .......... .......... .......... .......... ..........
E24377A     .......... .......... .......... .......... .......... .......... ..........
SE11        .......... .......... .......... .......... .......... .......... ..........
11128       .......... .......... .......... .......... .......... .......... ..........
11368       .......... .......... .......... .......... .......... .......... ..........
IAI1        .......... .......... .......... .......... .......... .......... ..........
E110019     .......... .......... .......... .......... .......... .......... ..........
B7A         .......... .......... .......... .......... .......... .......... ..........
CB9615      .......... .......... .......... .......... .......... ..G..G.... ..........
O157_Sakai  .......... .......... .......... .......... .......... ..G..G.... ..........
IAI39       .......... .......... .......... .......... ...A..G... .....G.... ..........
UMN026      .......... A......... .......... .......... ...A..G... .....G.... ..........
SMS-3-5     .......... .......... .......... .......... ...A..G... .....G.... ..........
536         .......... .......... .......... .......... ...A..G... .......... ..........
APEC_O1     .......... .......... .......... .......... ...A..G... .......... ..........
CFT073      .......... .......... .......... .......... ...A..G... .......... ..........
E2348/69    .......... A......... .......... .......... ...A..G... .....G.... ..........
UTI89       .......... .......... .......... .......... ...A..G... .......... ..........
F11         .......... .......... .......... .......... ...A..G... .......... ..........
ED1a        .......... .......... .......... .......... ...A..G... .......... ..........
83972       .......... .......... .......... .......... ...A..G... .......... ..........
S88         .......... .......... .......... .......... ...A..G... .......... ..........
E._albertii ---------- ---------- ---------- ---------- ---------- ---------- ----------

           8331
K-12        GGTCAGCCCC CAGTCGTCGC AAAGCTGCGC CACACGGACT GCACCGGAAA GCGTCCAGAA GTGCGGATCG
53638       .......... .......... .......... .......... .......... .......... ..........
ATCC_8739   .......... .......... .......... .......... .......... .......... ..........
HS          .......... .......... .......... .......... .......... .......... ..........
BW2952      .......... .......... .......... .......... .......... .......... ..........
12009       .......... .......... .......... .......... .......... .......... ..........
B171        .......... .......... .......... .......... .......... .......... ..........
E24377A     .......... .......... .......... .......... .......... .A........ ..........
SE11        .......... .......... .......... .......... .......... .......... ..........
11128       .......... .......... .......... .......... .......... .......... ..........
11368       .......... .......... .......... .......... .......... .......... ..........
IAI1        .......... .......... .......... .......... .......... .......... ..........
E110019     .......... .......... .......... .......... .......... .......... ..........
B7A         .......... .......... .......... .......... .......... .......... ..........
CB9615      .......... .......... .......... ...T...... .......... .......... ..........
O157_Sakai  .......... .......... .......... ...T...... .......... .......... ..........
IAI39       .......... .......... .......... T..G...... .......... .......... ......G...
UMN026      .......... .......... .......... T..G...... .......... .......... A.........
SMS-3-5     .......... .......... .......... T..G...... .......... .......... ......G...
536         .......... .......... ....T..... ...G...... .......... .......... ..........
APEC_O1     .......... .......... ....T..... ...G...... .......... .......... ..........
CFT073      .......... .......... ....T..... ...G...... .......... .......... ..........
E2348/69    .......... .......... .......... T..G...... .......... .......... ..........
UTI89       .......... .......... ....T..... ...G...... .......... .......... ..........
F11         .......... .......... ....T..... ...G...... .......... .......... ..........
ED1a        .......... .......... ....T..... ...G...... .......... .......... A.........
83972       .......... .......... ....T..... ...G...... .......... .......... ..........
S88         .......... .......... ....T..... ...G...... .......... .......... ..........
E._albertii ---------- ---------- ---------- ---------- ---------- ---------- ----------
```

```
           8401
K-12         GCAAGTGGAA TATCTACCGC ATTGAGCATC ACCGCATGAC CCATTTCGCG CCAGTTGGTG GCGATCATGT
53638        .......... .......... .......... .......... .......... .......... ..........
ATCC_8739    .......... .......... .......... .......... .......... .......... ..........
HS           .......... .......... .......... .......... .......... .......... ..........
BW2952       .......... .......... .......... .......... .......... .......... ..........
12009        .......... .......... .......... .......... .......... .......... ..........
B171         .......... .......... .......... .......... .......... .......... ..........
E24377A      .......... .......... .......... .......... .......... .......... ..........
SE11         .......... .......... .......... .......... .......... .......... ..........
11128        .......... .......... .......... .......... .......... .......... ..........
11368        .......... .......... .......... .......... .......... .......... ..........
IAI1         .......... .......... .......... .......... .......... .......... ..........
E110019      .......... .......... .......... .......... .......... .......... ..........
B7A          .......... .......... .......... .......... .......... .......... ..........
CB9615       .......... .......... .......... .......... .......... .......... ........A.
O157_Sakai   .......... .......... .......... .......... .......... .......... ........A.
IAI39        .......... .......... .......... ........G. .......... .......... ........A.
UMN026       .......... ....C..... ...A.....T ........G. .......... .......... ..A.......
SMS-3-5      .......... .......... .......... ........G. .......... .......... ........A.
536          .......... .......... .......... .......... .......... .......... ..........
APEC_O1      .......... .......... .......... .......... .......... .......... ..........
CFT073       .......... .......... .......... .......... .......... .......... ..........
E2348/69     .......... .......... .......... .......... .......... .......... ..........
UTI89        .......... .......... .......... .......... .......... .......... ..........
F11          .......... .......... .......... .......... .......... .......... ..........
ED1a         .......... ....C..... .........T ........G. .......... .......... ........A.
83972        .......... .......... .......... .......... .......... .......... ..........
S88          .......... .......... .......... .......... .......... .......... ..........
E._albertii  ---------- ---------- ---------- ---------- ---------- ---------- ----------

           8471
K-12         TAGTCGCGAC GGGCAAGCCG GTCGCCCGTC GAAATTCCGC CATCACTTCA CGTCCGGAGA AGCCCTGTTC
53638        .......... .......... ........C. ....C..... .......... .......... ..........
ATCC_8739    .......... .......... ........C. ....C..... .......... .......... ..........
HS           .......... .......... .......... .......... .......... .......... ..........
BW2952       .......... .......... .......... .......... .......... .......... ..........
12009        .......... .......... ........C. ....C..... ...A...... ..A....... .A........
B171         .......... .......... ........C. ....C..... ...A...... ..A....... .A........
E24377A      .......... .......... ........C. ....C..... ...A...... ..A....... .A........
SE11         .......... .......... ........C. ....C..... ...A...... ..A....... .A........
11128        .......... .......... ........C. ....C..... .......... ..A....... .A........
11368        .......... .......... ........C. ....C..... .......... ..A....... .A........
IAI1         .......... .......... ........C. ....C..... ...A...... ..A....... .A........
E110019      .......... .......... ........C. ....C..... ...A...... ..A....... .A........
B7A          .......... .......... ........C. ....C..... ...A...... ..A....... .A........
CB9615       .......... .......... ........C. ....C..... .......... .......... ..........
O157_Sakai   .......... .......... ........C. ....C..... .......... .......... ..........
IAI39        .......... .......... ........C. ....C..... .......... ..A....... .C........
UMN026       .G........ .....G.... .......... ....C..A.. .......... ..A....... .A.....C..
SMS-3-5      .......... .......... ........C. .......... .......... .......... .A........
536          .G........ .......... .......... ....C..... .......... ..A....... .A.....C..
APEC_O1      .G........ .......... .......... ....C..... .......... ..A....... .A.....C..
CFT073       .G........ .......... .......... ....C..... .......... ..A....... .A.....C..
E2348/69     .G........ .......... .......... ....C..... .......... ..A....... .A.....C..
UTI89        .G........ .......... .......... ....C..... .......... ..A....... .A.....C..
F11          .G........ .......... .......... ....C..... .......... ..A....... .A.....C..
ED1a         .......... .......... .......... ....C..... .......... .......... .A........
83972        .G........ .......... .......... ....C..... .......... ..A....... .A.....C..
S88          .G........ .......... .......... ....C..... .......... ..A....... .A.....C..
E._albertii  ---------- ---------- ---------- ---------- ---------- ---------- ----------
```

```
          8541
K-12          TGCGCCGCAT GGATCTTCGG CATAGGTAAG AACATCATTC AGCCCTTTGC ATAAAGAAAT GGCTTCATCA
53638         .......... .......... .......... .......... .......... .C........ ..........
ATCC_8739     .......... .......... .......... .......... .......... .C........ ..........
HS            .......... .......... .......... .......... .......... .......... ..........
BW2952        .......... .......... .......... .......... .......... .......... ..........
12009         .......... .......... .......... .......... .......... ...G...... ..........
B171          .......... .......... .......... .......... .......... ...G...... ..........
E24377A       .......... .......... .......... .......... .......... ...G...... ..........
SE11          .......... .......... .......... .......... .......... ...G...... ..........
11128         .......... .......... .......... .......... .......... ...G...... ..........
11368         .......... .......... .......... .......... .......... ...G...... ..........
IAI1          .......... .......... .......... .......... .......... ...G...... ..........
E110019       .......... .......... .......... .......... .......... ...G...... ..........
B7A           .......... .......... .......... .......... .......... ...G...... ..........
CB9615        .......... .......... .......... .......... .......... .C.G...... ..........
O157_Sakai    .......... .......... .......... .......... .......... .C.G...... ..........
IAI39         .........C ..G.....C. .......... .......... .......... .C.G...... ..........
UMN026        A......... ........A. .......G.. .......... .......... .C.G...... T.........
SMS-3-5       ......A..C .......... .......G.. .......... .......... .C.G...... A.........
536           A........C ..G.....C. .......... G......... .......... .C.G...... ..........
APEC_O1       A........C ..G.....C. .......... G......... .......... .C.G...... ..........
CFT073        A........C ..G.....C. .......... .........T .......... .C.G...... ..........
E2348/69      A........C ..G.....C. .......... .......... .......... .C.G...... ..........
UTI89         A........C ..G.....C. .......... G......... .......... .C.G...... ..........
F11           A........C ..G.....C. .......... G......... .......... .C.G...... ..........
ED1a          ......A..C .......... .......G.. .......... .......... .C.G...... A.........
83972         A........C ..G.....C. .......... .........T .......... .C.G...... ..........
S88           A........C ..G.....C. .......... G......... .......... .C.G...... ..........
E._albertii   ---------- ---------- ---------- ---------- ---------- ---------- ----------

          8611
K-12          AGCAGCCATG CACCGTTGGG ATCAACGGTA ATCCGCGCAT CCGGGAAGCG TTTCTTCAAT GCACGAACAG
53638         ........G. .......... .......... .......... .........T .......... ..........
ATCC_8739     ........G. .......... .......... .......... .........T .......... ..........
HS            .......... .......... .......... .......... .......... .......... ..........
BW2952        .......... .......... .......... .......... .......... .......... ..........
12009         ..T.....G. .......... .......... .......... .A........ .......... ..........
B171          ..T.....G. .......... .......... .......... .A........ .......... ..........
E24377A       ..T.....G. .......... .......... .......... .A........ .......... ..........
SE11          ..T.....G. .......... .......... .......... .A........ .......... ..........
11128         ..T.....G. .......... .......... .......... .A........ .......... ..C.......
11368         ..T.....G. .......... .......... .......... .A........ .......... ..C.......
IAI1          ..T.....G. .......... .......... .......... .A........ .......... ..........
E110019       ..T.....G. .......... .......... .......... .A........ .......... ..........
B7A           ..T.....G. .......... .......... .......... .A........ .......... ..........
CB9615        .......... .......... .......... .......T.. .......... .......... ..........
O157_Sakai    .......... .......... .......... .......... .......... .......... ..........
IAI39         ........G. ....A..... .......... .......... .A........ .......... ..........
UMN026        .......... .......... .......... ..T....... .......... .......... ..........
SMS-3-5       .......... .T........ .......... .......... .......... .......... ..........
536           ........G. ....A..... .......... .......... .A........ .......... ..........
APEC_O1       ........G. ....A..... .......... .......... .A........ .......... ..........
CFT073        ........G. ....A..... .......... .......... .A........ .......... .T...G....
E2348/69      ........G. ....A..... .......... .......... .A........ .......... ..........
UTI89         ........G. ....A..... .......... .......... .A........ .......... ..........
F11           ........G. ....A..... .......... .......... .A........ .......... ..........
ED1a          .......... .T........ .......... .......... .A........ .......... ..........
83972         ........G. ....A..... .......... .......... .A........ .......... .T...G....
S88           ........G. ....A..... .......... .......... .A........ .......... ..........
E._albertii   ---------- ---------- ---------- ---------- ---------- ---------- ----------
```

```
            8681
K-12        TGTCGATTTC TTGCTCGCCA GGTAACACGC CGCCCTTAAG TTTGAAATCT TTAAAGCCGT AGCGATCCTG
53638       .......... .......... ........C. .......... .......... .......... ..........
ATCC_8739   .......... .......... ........C. .......... .......... .......... ..........
HS          .......... .......... .......... .......... .......... .......... ..........
BW2952      .......... .......... .......... .......... .......... .......... ..........
12009       .......... .......... ........C. .......... .......... ........A. ..........
B171        .......... .......... ........C. .......... .......... ........A. ..........
E24377A     .......... .......... ........C. .......... .......... ........A. ..........
SE11        .......... .......... ........C. .......... .......... ........A. ..........
11128       .......... .......... ........C. .......... .......... ........A. ..........
11368       .......... .......... ........C. .......... .......... ........A. ..........
IAI1        .......... .......... ........C. .......... .......... ........A. ..........
E110019     .......... .......... ........C. .......... .......... ........A. ..........
B7A         .......... .......... ........C. .......... .......... ........A. ..........
CB9615      .......... .......... .......... .......... .......... .......... ..........
O157_Sakai  .......... .......... .......... .......... .......... .......... ..........
IAI39       .......... .......... ........T. .......... .......... ........A. ..........
UMN026      .......... .......... ........C. .......... .......... ........A. ..........
SMS-3-5     .......... .......... ........T. .......... .......... ........A. ..........
536         .......... .......... .......... .A........ .......... ........A. ..........
APEC_O1     .......... .......... .......... .A........ .......... ........A. ..........
CFT073      .......... .......... .......... .A........ .......... ........A. ..........
E2348/69    .......... .......... .......... .A........ .......... ........A. ..........
UTI89       .......... .......... .......... .A........ .......... ........A. ..........
F11         .......... .......... .......... .A........ .......... ........A. ..........
ED1a        .......... .......... .......... .A........ .......... ........A. ..........
83972       .......... .......... .......... .A........ .......... ........A. ..........
S88         .......... .......... .......... .A........ .......... ........A. ..........
E._albertii ---------- ---------- ---------- ---------- ---------- ---------- ----------

            8751
K-12        TGAGGCTTCC GCCAGACGCA CAACGGCTTC GCTGTTCATT GCTTTCTGAT GCCGCAACTG ATACCACTCA
53638       .......... .......... .......... .........C .......... .G........ ..........
ATCC_8739   .......... .......... .......... .........C .......... .G........ ..........
HS          .......... .......... .......... .........C .......... .G........ ..........
BW2952      .......... .......... .......... .......... .......... .......... ..........
12009       .......... .......... .......... .........C .......... .G........ ..........
B171        .......... .......... .......... .........C .......... .G........ ..........
E24377A     .......... .......... .......... .........C .......... .G........ ..........
SE11        .......... .......... .......... .........C .......... .G........ ..........
11128       .......... .......... .......... .........C .......... .G........ ..........
11368       .......... .......... .......... .........C .......... .G........ ..........
IAI1        .......... .......... .......... .........C .......... .G........ ..........
E110019     .......... .......... .......... .........C .......... .G........ ..........
B7A         .......... .......... .......... .........C .......... .G........ ..........
CB9615      .......... .......... .......... .........C .......... .G........ ..........
O157_Sakai  .......... .......... .......... .........C .......... .G........ ..........
IAI39       ...A...... .......... .......... .........C .......... .......T.. ..........
UMN026      ...A...... .......... .......... .........C .......... .......T.. ..........
SMS-3-5     ......G... .......... .......... .........C .......... .......T.. ..........
536         .......... .......... .......... .........C .......... .......... ..........
APEC_O1     .......... .......... .......... .........C .......... .......... ..........
CFT073      .......... .......... .......... .........C .......... .......... ..........
E2348/69    .......... .......... .......... .........C .......... .......... ..........
UTI89       .......... .......... .......... .........C .......... .......... ..........
F11         .......... .......... .......... .........C .......... .......... ..........
ED1a        .......... .......... .......... .........C .......... .......... ..........
83972       .......... .......... .......... .........C .......... .......... ..........
S88         .......... .......... .......... .........C .......... .......... ..........
E._albertii ---------- ---------- ---------- ---------- ---------- ---------- ----------
```

```
            8821
K-12        TGGTTGCCCG GCGTATTTTC CACATAAGGA AGATCGGTTT TGGTCCGATC ACCGATATAA AACAGATAAC
53638       .......... .......... ..G....... .......... .......... .......... ..........
ATCC_8739   .......... .......... ..G....... .......... .......... .......... ..........
HS          .......... .......... ..G....... .......... .......... .......... ..........
BW2952      .......... .......... .......... .......... .......... .......... ..........
12009       .......... .......... ..G....... .......... .......... .......... ..........
B171        .......... .......... ..G....... .......... .......... .......... ..........
E24377A     .......... .......... ..G....... .......... .......... .......... ..........
SE11        .......... .......... ..G....... .......... .......... .......... ..........
11128       .......... .......... ..G....... .......... .......... .......... ..........
11368       .......... .......... ..G....... .......... .......... .......... ..........
IAI1        .......... .......... .......... .......... .......... .......... ..........
E110019     .......... .......... ..G....... .......... .......... .......... ..........
B7A         .......... .......... ..G....... .......... .......... .......... ..........
CB9615      .......... .......... ..G....... .......... .......... .......... ..........
O157_Sakai  .......... .......... ..G....... .......... .......... .......... ..........
IAI39       .......... .......... ..G....... .......... .......... .......... ..........
UMN026      .......... .......... ..G....... .......... .......... .......... ..........
SMS-3-5     .......... .A........ ..G....... .......... .......... .......... ..........
536         .......... .......... ..G....... .......... .......... .......... ..........
APEC_O1     .......... .......... ..G....... .......... .......... .......... ..........
CFT073      .......... .......... ..G....... .......... .......... .......... ..........
E2348/69    .......... .......... ..G....... .......... .......... .......... ..........
UTI89       .......... .......... ..G....... .......... .......... .......... ..........
F11         .......... .......... ..G....... .......... .......... .......... ..........
ED1a        .......... .......... ..G....... .......... .......... .......... ..........
83972       .......... .......... ..G....... .......... .......... .......... ..........
S88         .......... .......... ..G....... .......... .......... .......... ..........
E._albertii ---------- ---------- ---------- ---------- ---------- ---------- ----------

            8891
K-12        CGAGGACGGT AATAGCCTCG CGTTGCTTGC CTGGCCCTAA CAGTTCGCAG ACCGGAACAT TCAGCGCCTT
53638       .......... .....T.... .......... .......C.. .......... .......... ..........
ATCC_8739   .......... .....T.... .......... .......C.. .......... .......... ..........
HS          .......... .....T.... .......... .......C.. .......... .......... ..........
BW2952      .......... .......... .......... .......... .......... .......... ..........
12009       .......... .....T.... .......... .......C.. .......... .......... ..........
B171        .......... .....T.... .......... .......C.. .......... .......... ..........
E24377A     .......... .....T.... .......... .......C.. .......... .......... ..........
SE11        .......... .....T.... .......... .......C.. .......... .......... ..........
11128       .......... .....T.... .......... .......C.. .......... .......... ..........
11368       .......... .....T.... .......... .......C.. .......... .......... ..........
IAI1        .......... .....T.... .......... .......C.. .......... .......... ..........
E110019     .......... .....T.... .......... .......C.. .......... .......... ..........
B7A         .......... .....T.... .......... .......C.. .......... .......... ..........
CB9615      .......... ......A... .......... .......... .......... .......... ..........
O157_Sakai  .......... ......A... .......... .......... .......... .......... ..........
IAI39       .......... ......A... .......... .......... .......... .......... ..........
UMN026      .......... .......... .......... .......C.. .......... .......... ..........
SMS-3-5     ....A..... ......A..A .......... .......... .......... .......... ..........
536         ....A..... ......A..A .......... .......... .......... .......... .........G
APEC_O1     ....A..... ......A..A .......... .......... .......... .......... .........G
CFT073      ....A..... ......A..A .......... .......... .......... .......... .........G
E2348/69    ....A..... ......A..A .......... .......... .......... .......... .........G
UTI89       ....A..... ......A..A .......... .......... .......... .......... .........G
F11         ....A..... ......A..A .......... .......... .......... .......... .........G
ED1a        ....A..... ......A..A .......... .......... .......... .......... .........G
83972       ....A..... ......A..A .......... .......... .......... .......... .........G
S88         ....A..... ......A..A .......... .......... .......... .......... .........G
E._albertii ---------- ---------- ---------- ---------- ---------- ---------- ----------
```

```
          8961
K-12         ACCTAGCAGG TCAAGCAAAG CGGCTTCCAG CGCCGCCACG GCGTTAACGC GTAATTCAAA GGTCCAGGCA
53638        .......... ........G. .......... .......... ..A....... .C........ A.........
ATCC_8739    .......... ........G. .......... .......... ..A....... .C........ A.........
HS           .......... ........G. .......... .......... ..A....... .C........ A.........
BW2952       .......... .......... .......... .......... .......... .......... ..........
12009        .......... ........G. .......... .......... ..A....... .C........ A.........
B171         .......... ........G. .......... .......... ..A....... .C........ A.........
E24377A      .......... ........G. .......... .......... ..A....... .C........ A.........
SE11         .......... ........G. .......... .......... ..A....... .C........ A.........
11128        .......... ........G. .......... .......... ..A....... .C........ A.........
11368        .......... ........G. .......... .......... ..A....... .C........ A.........
IAI1         .......... ........G. .......... .......... ..A....... .C........ A.........
E110019      .......... ........G. .......... .......... ..A....... .C........ A.....A...
B7A          .......... ........G. .......... .......... ..A....... .C........ A.........
CB9615       .......... ........G. .......... .......... .......... .C........ .........C
O157_Sakai   .......... ........G. .......... .......... .......... .C........ .........C
IAI39        .......... .......... .......... .......... ..A....... .C........ A.........
UMN026       .......... ........G. .......... .......... ..A....... .C........ A.........
SMS-3-5      .......... ........G. .......... .......... ..A....... .C........ A.........
536          .....A.... ........G. .......... .......... ..A....... .C........ A.........
APEC_O1      .....A.... ........G. .......... .......... ..A....... .C........ A.........
CFT073       .....A.... ........G. .......... .......... ..A....... .C........ A.........
E2348/69     .....A.... ........G. .......... .......... ..A....... .C........ A.........
UTI89        .....A.... ........G. .......... .......... ..A....... .C........ A.........
F11          .....A.... ........G. .......... .......... ..A....... .C........ A.........
ED1a         .....A.... ........G. .......... .......... ..A....... .C........ A.........
83972        .....A.... ........G. .......... .......... ..A....... .C........ A.........
S88          .....A.... ........G. .......... .......... ..A....... .C........ A.........
E._albertii  ---------- ---------- ---------- ---------- ---------- ---------- ----------

          9031
K-12         CCTTTACCGA AGGTATCAAA ATCGGCGGCC TGATTACCTT TATGCACCTG CTGGACCACT TTATTCAGTC
53638        .....G.... .......... ......T... .......... .......... ...A...... ........G.
ATCC_8739    .....G.... .......... ......T... .......... .......... ...A...... ........G.
HS           .....G.... .......... ......T... .......... .......... ...A...... ........G.
BW2952       .......... .......... .......... .......... .......... .......... ..........
12009        .....G.... .......... ......T... .......... .......... ...A...... ........G.
B171         .....G.... .......... ......T... .......... .......... ...A...... ........G.
E24377A      .....G.... .......... ......T... .......... .......... ...A...... ........G.
SE11         .....G.... .......... ......T... .......... .......... ...A...... ........G.
11128        .T...G.... .......... ......TA.. .......... .......... ...A...... ........G.
11368        .....G.... .......... ......T... .......... .......... ...A...... ........G.
IAI1         .....G.... .......... ......T... .......... .......... ...A...... ........G.
E110019      .....G.... .......... ......T... .......... .......... ...A...... ........G.
B7A          .....G.... .......... ......T... .......... .......... ...A...... ........G.
CB9615       .......... .......... ......T..T .......... .......... ...A...... ........G.
O157_Sakai   .......... .......... ......T..T .......... .......... ...A...... ........G.
IAI39        .....G.... .......... ......T... .......... .......... ...A...... ..........
UMN026       .....G.... .......... ......T... .......... .......... ...A...... ........G.
SMS-3-5      .....G.... .......... ......T... .......... .......... ...A...... ........G.
536          .....G.... .......... ......T... .......... .....G.... ...A...... ........G.
APEC_O1      .....G.... .......... ......T... .......... .......... ...A...... ........G.
CFT073       .....G.... .......... ......T... .......... .......... ...A...... ........G.
E2348/69     .....G.... ...C...... ......T... .......... .......... ...A...... ........G.
UTI89        .....G.... .......... ......T... .......... .......... ...A...... ........G.
F11          .....G.... .......... ......T... .......... .....G.... ...A...... ........G.
ED1a         .....G.... .......... ......T... .......... .......... ...A...... ........G.
83972        .....G.... .......... ......T... .......... .......... ...A...... ........G.
S88          .....G.... .......... ......T... .......... .......... ...A...... ........G.
E._albertii  ---------- ---------- ---------- ---------- ---------- ---------- ----------
```

```
          9101
K-12         GCGCAACTTC CTGGCCCAGA ACCATCGGAA TAGCATCGAC AAGCGTCTGA TAAATCACAT CTCCGCCCGG
53638        .......... .......... .......... .......... C..T..T... ........C. ..........
ATCC_8739    .......... .......... .......... .......... C..T..T... ........C. ..........
HS           .......... .......... .......... .......... C..T..T... ........C. ..........
BW2952       .......... .......... .......... .......... .......... .......... ..........
12009        .......... .......... .......... .......... C..T..T... ........C. ..........
B171         .......... .......... .......... .......... C..T..T... ........C. ..........
E24377A      .......... .......... .......... .......... C..T..T... ........C. ..........
SE11         .......... .......... .......... .......... C..T..T... ........C. ..........
11128        .......... .......... .......... .......... C..T..T... ........C. ..........
11368        .......... .......... .......... .......... C..T..T... ........C. ..........
IAI1         .......... .......... .......... .......... C..T..T... ........C. ..........
E110019      .......... ...A...... .......... .......... C..T..T... ........C. ..........
B7A          .......... .......... .......... .......... C..T..T... ........C. ..........
CB9615       .......... .........C .......... .......... C.AT...... ........C. .......T..
O157_Sakai   .......... ..A......C .......... .......... C.AT...... ........C. .......T..
IAI39        .T..G.TC.. ........AC .......... .......... C..T..T... ........C. ..........
UMN026       .......... .........C .......... .......... C.AT...... ........C. .......T..
SMS-3-5      .......... .........C .......... ....C..... C..T...... ........C. ..........
536          .......... .........C .......... ....C.T... C..T..T... ........C. ..........
APEC_O1      .......... .........C .......... ....C.T... C..T..T... ........C. ..........
CFT073       .......... .........C .......... ....C.T... C..T..T... ........C. ..........
E2348/69     .......... .........C .......... ....C..... C..T..T... ........C. ..........
UTI89        .......... .........C .......... ....C.T... C..T..T... ........C. ..........
F11          .......... .........C .......... ....C.T... C..T..T... ........C. ..........
ED1a         .......... .........C .......... ....C.T... C..T..T... ........C. ..........
83972        .......... .........C .......... ....C.T... C..T..T... ........C. ..........
S88          .......... .........C .......... ....C.T... C..T..T... ........C. ..........
E._albertii  ---------- ---------- ---------- ---------- ---------- ---------- ----------

          9171
K-12         CGCTTCACCA ATGCCGGTAT GCCCGGCGTT ATCGGTGAGT ACCACAATAT TGCGGGTGAA ATATGCGTTA
53638        .......... .......... .......... .......... .......... .......... ..........
ATCC_8739    .......... .......... .......... .......... .......... .......... ..........
HS           .......... .......... .......... .......... .......... .......... ..........
BW2952       .......... .......... .......... .......... .......... .......... ..........
12009        .......... .......... .......... .......... .......... .......... ..........
B171         .......... .......... .......... .......... .......... .......... ..........
E24377A      .......... .......... .......... .......... .......... .......... ..........
SE11         .......... .......... .......... .......... .......... .......... ..........
11128        .......... .......... .......... .......... .......... .......... ..........
11368        .......... .......... .......... .......... .......... .......... ..........
IAI1         .......... .......... .......... .......... .......... .......... ..........
E110019      .......... .......... .......... .......... .......... .......... ..........
B7A          .......... .......... .......... .......... .......... .......... ..........
CB9615       .......... .......... .......... .......... .......... ....A..... ..........
O157_Sakai   .......... .......... .......... .......... .......... ....A..... ..........
IAI39        .......... ..A....... .......... .......... .......... .......... ..........
UMN026       .......... .......... .......... .......... .......... ....A..... ..........
SMS-3-5      .......... .......... .......... .......... .......... .......... ..........
536          .......... .......... .......A.. .......... .......... .......... ..........
APEC_O1      .......... .......... .......A.. .......... .......... .......... ..........
CFT073       .......... .......... A......A.. .......... .......... .......... ..........
E2348/69     .......... .......... .......A.. .......... .......... .......... ..........
UTI89        .......... .......... .......A.. .......... .......... .......... ..........
F11          .......... .......... .......A.. .......... .......... .......... ..........
ED1a         .......... .......... .......A.. .......... .......... .......... ..........
83972        .......... .......... A......A.. .......... .......... .......... ..........
S88          .......... .......... .......A.. .......... .......... .......... ..........
E._albertii  ---------- ---------- ---------- ---------- ---------- ---------- ----------
```

```
         9241
K-12        TGTGCCCCAC CAATATTAAG CAACATGCTG TCATGCCCGG CCACCGGAAT GACTTTCATA TCAGTAATAA
53638       .......... .......... .........A ..G....... .G........ .......... ..........
ATCC_8739   .......... .......... .........A ..G....... .G........ .......... ..........
HS          .......... .......... .........A ..G....... .G........ .......... ..........
BW2952      .......... .......... .......... .......... .......... .......... ..........
12009       .......... .......... .........A ..G....... .G........ .......... ..........
B171        .......... .......... .........A ..G....... .G........ .......... ..........
E24377A     .......... .......... .........A ..G....... .G........ .......... ..........
SE11        .......... .......... .........A ..G....... .G........ .......... ..........
11128       .......... .......... .........A ..G....... .G........ .......... ..........
11368       .......... .......... .........A ..G....... .G........ .......... ..........
IAI1        .......... .......... .........A ..G....... .G........ .......... ..........
E110019     .......... .......... .........A ..G....... .G........ .......... ..........
B7A         .....A.... .......... .........A ..G....... .G........ .......... ..........
CB9615      ..C....... .......... .......... ..G.A..... .G........ .......... ..........
O157_Sakai  ..C....... .......... .......... ..G.A..... .G........ .......... ..........
IAI39       .....A.... .......... .......... .......... .......... .......... ..........
UMN026      ..C....... .......... .........A ..G....... .G........ .......... ..........
SMS-3-5     .....A.... .......... .......... .......... .......... .......... ..........
536         .....A.... .......... .......... .....T.... .G........ .......... ..........
APEC_O1     .....A.... .......... .......... .....T.... .G........ .......... ..........
CFT073      .....A.... .......... .......... .....T.... .G........ .......... ..........
E2348/69    .....A.... .......... .......... .....T.... .G........ .......... ..........
UTI89       .....A.... .......... .......... .....T.... .G........ .......... ..........
F11         .....A.... .......... .......... .....T.... .G........ .......... ..........
ED1a        .....A.... .......... .......... .....T.... .G........ .......... ..........
83972       .....A.... .......... .......... .....T.... .G........ .......... ..........
S88         .....A.... .......... .......... .....T.... .G........ .......... ..........
E._albertii ---------- ---------- ---------- ---------- ---------- ---------- ----------

         9311
K-12        CAGGACTGGA TTGTGTCGCC ATCTTATTGC CCCGCAACAG GTTTCAACTC GATACGCTTG ATATCGCCCA
53638       .......... ........T. .......... .....G.... ......G... .......... ..........
ATCC_8739   .......... ........T. .......... .....G.... ......G... .......... ..........
HS          .......... ........T. .......... .....G.... ......G... .......... ..........
BW2952      .......... .......... .......... .......... .......... .......... ..........
12009       .......... ........T. .......... .....G.... ......G... .......... ..........
B171        .......... ........T. .......... .....G.... ......G... .......... ..........
E24377A     .......... ........T. .......... .....G.... ......G... .......... ..........
SE11        .......... ........T. .......... .....G.... ......G... .......... ..........
11128       .......... ........T. .......... .....G.... ......G... .......... ..........
11368       .......... ........T. .......... .....G.... ......G... .......... ..........
IAI1        .......... ........T. .......... .....G.... ......G... .......... ..........
E110019     .......... ........T. .......... .....G.... ......G... .......... ..........
B7A         .......... ........T. .......... .....G.... ......G... .......... ..........
CB9615      .......... ........T. .......... .....G.... ......G... .......... ..........
O157_Sakai  .......... ........T. .......... ...A.G.... ......G... .......... ..........
IAI39       .......... .......... .......... .......... .......... .......... ..........
UMN026      .......... ........T. .......... .....G.... ......G... .......... ..........
SMS-3-5     .......... .......... .......... .......... .......... .......... ..........
536         .......... ........T. .......... .....G.... ......G... A......... ..........
APEC_O1     .......... ........T. .......... .....G.... ......G... A......... ..........
CFT073      .......... ........T. .......... .....G.... ......G... A......... ..........
E2348/69    .......... ........T. .......... .....G.... ......G... A......... ..........
UTI89       .......... ........T. .......... .....G.... ......G... A......... ..........
F11         .......... ........T. .......... .....G.... ......G... A......... ..........
ED1a        .......... ........T. .......... .....G.... ......G... A......... ..........
83972       .......... ........T. .......... .....G.... ......G... A......... ..........
S88         .......... ........T. .......... .....G.... ......G... A......... ..........
E._albertii ---------- ---------- ---------- ---------- ---------- ---------- ----------
```

```
             9381
K-12         CCAGCACCAG GTAGCTCAGT ACCGCGATTA AGGCATGAAC ACCAACATAA ATCAGCGCCC CATTAAACGA
53638        .......... .......... .......... .......... .......... .......... .......G..
ATCC_8739    .......... .......... .......... .......... .......... .......... .......G..
HS           .......... .......... .......... .......... .......... .......... .......G..
BW2952       .......... .......... .......... .......... .......... .......... ..........
12009        .......... .......... .......... .......... .......... .......... .......G..
B171         .......... .......... .......... .......... .......... .......... .......G..
E24377A      .......... .......... .......... .......... .......... .......... .......G..
SE11         .......... .......... .......... .......... .......... .......... .......G..
11128        .......... .......... .......... .......... .......... .......... .......G..
11368        .......... .......... .......... .......... .......... .......... .......G..
IAI1         .......... .......... .......... .......... .......... .......... .......G..
E110019      .......... .......... .......... .......... .......... .......... .......G..
B7A          .......... .......... .......... .......... .......... .......... .......G..
CB9615       .......... .......... .......... .......... .......... .......... ..........
O157_Sakai   .......... .......... .......... .......... .......... .......... ..........
IAI39        ...A...... .......... .......... .......... .......... .......... .......G..
UMN026       .......... .......... .......... .......... .......... .......... .......G..
SMS-3-5      ...A...... .......... .......... .......... .......... .......... .......G..
536          .......... ......G... .......... .......... .......... .......... .......G..
APEC_O1      .......... ......G... .......... .......... .......... .......... .......G..
CFT073       .......... ......G... .......... .......... .......... .......... .......G..
E2348/69     .......... ......G... .......... .......... .......... .......... .......G..
UTI89        .......... ......G... .......... .......... .......... .......... .......G..
F11          .......... ......G... .......... .......... .......... .......... .......G..
ED1a         .......... ......G... .......... .......... .......... .......... .......G..
83972        .......... ......G... .......... .......... .......... .......... .......G..
S88          .......... ......G... .......... .......... .......... .......... .......G..
E._albertii  ---------- ---------- ---------- ---------- ---------- ---------- ----------

             9451
K-12         GCCAGTCGTG CCAACGATAT AACCAATTGC GATTGGCGTG ACGATGCCAG AAATGTTACC GAACATGTTG
53638        .......... .......... .......... .......... .......... .......... ..........
ATCC_8739    .......... .......... .......... .......... .......... .......... ..........
HS           .......... .......... .......... .......... .......... .......... ..........
BW2952       .......... .......... .......... .......... .......... .......... ..........
12009        .......... .......... .......... .......... .......... .......... ..........
B171         .......... .......... .......... .......... .......... .......... ..........
E24377A      .......... .......... .......... .......... .......... .......... ..........
SE11         .......... .......... .......... .......... .......... .......... ..........
11128        .......... .......... .......... .......... .......... .......... ..........
11368        .......... .......... .......... .......... .......... .......... ..........
IAI1         .......... .......... .......... .......... .......... .......... ..........
E110019      .......... .......... .......... .......... .......... .......... ..........
B7A          .......... .......... .......... .......... .......... .......... ..........
CB9615       .......... .......... .......... .......... .......... .......... ..........
O157_Sakai   .......... .......... .......... .......... .......... .......... ..........
IAI39        .......... .......... .......... .......... .......... .......... ..........
UMN026       .......... .......... .......... .......... .......... .......... ..........
SMS-3-5      .......... .......... .......... .......... .......... .......... ..........
536          .......... .......... .......... .......... .......... .......... A........A
APEC_O1      .......... .......... .......... .......... .......... .......... A........A
CFT073       .......... .......... .......... .......... .......... .......... A........A
E2348/69     .......... .......... .......... .......... .......... .......... A........A
UTI89        .......... .......... .......... .......... .......... .......... A........A
F11          .......... .......... .......... .......... .......... .......... A........A
ED1a         .......... .......... .......... .......... .......... .......... A........A
83972        .......... .......... .......... .......... .......... .......... A........A
S88          .......... .......... .......... .......... .......... .......... A........A
E._albertii  ---------- ---------- ---------- ---------- ---------- ---------- ----------
```

```
            9521
K-12        AACAGGCCAC CGGAAAGACC GCTGATCTCT TTTGGCGCGG TATCTGCCAT TACTGCCCAA CCCAGCGCCC
53638       .......... .......... .......... .......... .......... .......... ..........
ATCC_8739   .......... .......... .......... .......... .......... .......... ..........
HS          .......... .......... .......... .......... .......... .......... ..........
BW2952      .......... .......... .......... .......... .......... .......... ..........
12009       .......... .......... .......... .......... .......... .......... ..........
B171        .......... .......... .......... .......... .......... .......... ..........
E24377A     .......... .......... .......... .......... .......... .......... ..........
SE11        .......... .......... .......... .......... .......... .......... ..........
11128       .......... .......... .......... .......... .......... .......... ..........
11368       .......... .......... .......... .......... .......... .......... ..........
IAI1        .......... .......... .......... .......... .......... .......... ..........
E110019     .......... .......... .......... .......... .......... .......... ..........
B7A         .......... .......... .......... .......... .......... .......... ..........
CB9615      .......... .......... .......... .......... .......... .......... ..........
O157_Sakai  .......... .......... .......... .......... .......... .......... ..........
IAI39       .......... .......... .......... .......... .......... .......... ..........
UMN026      .......... .......... .......... .......... .......... .......... ..........
SMS-3-5     .......... .......... .......... .......... .......... .......... ..........
536         .......... .......... .......... ........A. ....A..... G..A...... ........A.
APEC_O1     .......... .......... .......... ........A. ....A..... ...A...... ........A.
CFT073      .......... .......... .......... ........A. ....A..... ...A...... ........A.
E2348/69    .......... .......... .......... ........A. ....A..... ...A...... ........A.
UTI89       .......... .......... .......... ........A. ....A..... ...A...... ........A.
F11         .......... .......... .......... ........A. ....A..... ...A...... ........A.
ED1a        .......... .......... .......... ........A. ....A..... ...A...... ........A.
83972       .......... .......... .......... ........A. ....A..... ...A...... ........A.
S88         .......... .......... .......... ........A. ....A..... ...A...... ........A.
E._albertii ---------- ---------- ---------- ---------- ---------- ---------- ----------

            9591
K-12        CGATGCCCTT ACCGAAGAAG GCCAGCGCCA TAAAGCCGAT GATCATCCAC TCAACGTTGA CGTAGTTGCA
53638       .......... .......... .......... .......... .......... .......... ..........
ATCC_8739   .......... .......... .......... .......... .......... .......... ..........
HS          .......... .......... .......... .......... .......... .......... ..........
BW2952      .......... .......... .......... .......... .......... .......... ..........
12009       .......... .......... .......... .......... .......... .......... ..........
B171        .......... .......... .......... .......... .......... .......... ..........
E24377A     .......... .......... .......... .......... .......... .......... ..........
SE11        .......... .......... .......... .......... .......... .......... ..........
11128       .......... .......... .......... .......... .......... .......... ..........
11368       .......... .......... .......... .......... .......... .......... ..........
IAI1        .......... .......... .......... .......... .......... .......... ..........
E110019     .......... .......... .......... .......... .......... .......... ..........
B7A         .......... .......... .......... .......... .......... .......... ..........
CB9615      .A........ .......... .......... .......... .......... .......... ..........
O157_Sakai  .A........ .......... .......... .......... .......... .......... ..........
IAI39       .......... .......... .......... .......... .......... .......... ..........
UMN026      .......... .......... .......... .......... .......... .......... ..........
SMS-3-5     .......... .......... .......... .......... .......... .......... ..........
536         ....C..... .......... .......... .......... .......... .......... ..........
APEC_O1     ....C..... .......... .......... .......... .......... .......... ..........
CFT073      ....C..... .......... .......... .......... .......... .......... ..........
E2348/69    ....C..... .......... .......... .......... .......... .......... ..........
UTI89       ....C..... .......... .......... .......... .......... .......... ..........
F11         ....C..... .......... .......... .......... .......... .......... ..........
ED1a        ....C..... .......... .......... .......... .......... .......... ..........
83972       ....C..... .......... .......... .......... .......... .......... ..........
S88         ....C..... .......... .......... .......... .......... .......... ..........
E._albertii ---------- ---------- ---------- ---------- ---------- ---------- ----------
```

```
            9661
K-12         GAACACCATC ACCATCGACA GCAACATGCC CATTACGATC GGTGTTTTAC GCGCAATGTT CAGCGATCCC
53638        .......... .......... .......... .......... .......... .......... ..........
ATCC_8739    .......... .......... .......... .......... .......... .......... ..........
HS           .......... .......... .......... .......... .......... .......... ..........
BW2952       .......... .......... .......... .......... .......... .......... ..........
12009        .......... .......... .......... .......... .......... .......... ..........
B171         .......... .......... .......... .......... .......... .......... ..........
E24377A      .......... .......... .......... .......... .......... .......... ..........
SE11         .......... .......... .......... .......... .......... .......... ..........
11128        .......... .......... .......... .......... .......... .......... ..........
11368        .......... .......... .......... .......... .......... .......... ..........
IAI1         .......... .......... .......... .......... .......... .......... ..........
E110019      .......... .......... .......... .......... .......... .......... ..........
B7A          .......... .......... .......... .......... .......... .......... ..........
CB9615       .......... .......... .......... .......... .......... .......... ..........
O157_Sakai   .......... .......... .......... .......... .......... .......... ..........
IAI39        .......... .......... .......... .......... .......... .......... ..........
UMN026       .......... .......... .......... .......... .......... .......... ..........
SMS-3-5      .......... .......... .......... .......... .......... .......... ..........
536          .......... .......... .......... ...C...... .......... .......... ...T..A...
APEC_O1      .......... .......... A......... ...C...... .......... .......... ...T..A...
CFT073       .......... .......... .......... ...C...... .......... .......... ...T..A...
E2348/69     .......... .......... A......... ...C...... .......... .......... ...T..A...
UTI89        .......... .......... A......... ...C...... .......... .......... ...T..A...
F11          .......... .......... .......... ...C...... .......... .......... ...T..A...
ED1a         .......... .......... A......... ...C...... .......... .......... ...T..A...
83972        .......... .......... .......... ...C...... .......... .......... ...T..A...
S88          .......... .......... A......... ...C...... .......... .......... ...T..A...
E._albertii  ---------- ---------- ---------- ---------- ---------- ---------- ----------

            9731
K-12         GTGCGGCGCA TCAGCCAGTC GGAAATAATC CCACCCAGCA CACCGCCGAT AAAACCGCAA ACCGCCGGAA
53638        .......... .......... .......... .......... .......... .......... ......T...
ATCC_8739    .......... .......... .......... .......... .......... .......... ..........
HS           .......... .......... .......... .......... .......... .......... ..........
BW2952       .......... .......... .......... .......... .......... .......... ..........
12009        .......... .......... .......... .......... .......... .......... ..........
B171         .......... .......... .......... .......... .......... .......... ..........
E24377A      .......... .......... .......... .......... .......... .......... ..........
SE11         .......... .......... .......... .......... .......... .......... ..........
11128        .......... .......... .......... .......... .......... .......... ..........
11368        .......... .......... .......... .......... .......... .......... ..........
IAI1         .......... .......... .......... .......... .......... .......... ..........
E110019      .......... .......... .......... .......... .......... .......... ..........
B7A          .......... .......... .......... .......... .......... .......... ..........
CB9615       .......... .......... .......... .......... ..T.A..... ......A... ..........
O157_Sakai   .......... .......... .......... .......... ....A..... ......A... ..........
IAI39        .......... .......... .......... .......... .......... .......... ..........
UMN026       .......... .......... .......... .......... .......... .......... ..........
SMS-3-5      .......... .......... .......... .......... .......... .......... ..........
536          .....A.... .......... .......... .......... .......... .......... ..........
APEC_O1      .....A.... .......... .......... .......... .......... .......... ..........
CFT073       .....A.... .......... .......... ..C....A.. .......... .......... ..........
E2348/69     .....A.... .......... .......... .......... .......... .......... ..........
UTI89        .....A.... .......... .......... .......... .......... .......... ..........
F11          .....A.... .......... .......... .......... .......... .......... ..........
ED1a         .....A.... .......... .......... .......... .......... .......... ..........
83972        .....A.... .......... .......... ..C....A.. .......... .......... ..........
S88          .....A.... .......... .......... .......... .......... .......... ..........
E._albertii  ---------- ---------- ---------- ---------- ---------- ---------- ----------
```

```
          9801
K-12        CGGAAGCCAC AAAGCCCGCT TTCAGAATCG ACATCCCGCG TGCCTGCACC AGATAAACCG GGAACCAGGT
53638       .......... .......... .......... .......... .......... .......... ..........
ATCC_8739   .......... .......... .......... .......... .......... .......... ..........
HS          .......... .......... .......... .......... .......... .......... ..........
BW2952      .......... .......... .......... .......... .......... .......... ..........
12009       .......... .......... .......... .......A.. C......... .......... .A........
B171        .......... .......... .......... .......A.. C......... .......... .A........
E24377A     .......... .......... .......... .......A.. C......... .......... .A........
SE11        .......... .......... .......... .......A.. C......... .......... .A........
11128       .......... .......... .......... .......A.. C......... .......... .A........
11368       .......... .......... .......... .......A.. C......... .......... .A........
IAI1        .......... .......... .......... .......A.. C......... .......... .A........
E110019     .......... .......... .......... .......... .......... .......... ..........
B7A         .......... .......... .......... .......... .......... .......... .A........
CB9615      .......... .......... .......... .......A.. C......... .......... .A........
O157_Sakai  .......... .......... .......... .......A.. C......... .......... .A........
IAI39       .......... .......... .......... .......A.. C......... .......... ..........
UMN026      .......... .......... .......... .......A.. C......... .......... ..........
SMS-3-5     .......... .......... .......... .......A.. C......... .......... ..........
536         .......... .......... .......... .......... C......... .......... ..........
APEC_O1     .......... .......... .......... .......... C......... .......... ..........
CFT073      .A........ ......T... .......... .......... C......... .......... ..........
E2348/69    .......... .......... .......... .......... C......... .......... ..........
UTI89       .......... .......... .......... .......... C......... .......... ..........
F11         .......... .......... .......... .......... C......... .......... ..........
ED1a        .......... .......... .......... .......... C......... .......... ..........
83972       .A........ ......T... .......... .......... C......... .......... ..........
S88         .......... .......... .......... .......... C......... .......... ..........
E._albertii ---------- ---------- ---------- ---------- ---------- ---------- ----------

          9871
K-12        AATAAAGAAG TAAGTCAGGG CGTTGATACA GTACTGACCG ATATAAACGC CGATCATCAT CCGTGACCCT
53638       .......... .......... .......... .......... .......... .......... .........G
ATCC_8739   .......... .......... .......... .......... .......... .......... .........G
HS          .......... .......... .......... .......... .......... .......... .........G
BW2952      .......... .......... .......... .......... .......... .......... ..........
12009       ...G..A... .......... .......... .......... .......... .......... .........G
B171        ...G..A... .......... .......... .......... .......... .......... .........G
E24377A     ...G..A... .......... .......... .......... .......... .......... .........G
SE11        ...G..A... .......... .......... .......... .......... .......... .........G
11128       ...G..A... .......... .......... .......... .......... .......... .........G
11368       ...G..A... .......... .......... .......... .......... .......... .........G
IAI1        ...G..A... .......... .......... .......... .......... .......... .........G
E110019     .......... .......... .......... .......... .......... .......... .........G
B7A         ...G..A... .......... .......... .......... .......... .......... .........G
CB9615      ...G..A... .......... .......... .......... .......... .......... .........G
O157_Sakai  ...G..A... .......... .......... .......... .......... .......... .........G
IAI39       .......... .......... .......... .......... .......... .......... .........G
UMN026      .......... .......... .......... .......... .......... .......... .........G
SMS-3-5     .......... .......... .......... .......... .......... .......... .........G
536         ......A... .......... .......... .......... .......... .......... .........G
APEC_O1     ......A... .......... .......... .......... .......... .......... .........G
CFT073      ......A... .......... .......... .......... .......... .......... .........G
E2348/69    ...G..A... .......... .......... .......... .......... .......... .........G
UTI89       ......A... .......... .......... .......... .......... .......... .........G
F11         ......A... .......... .......... .......... .......... .......... .........G
ED1a        ......A... .......... .......... .......... .......... .......... .........G
83972       ......A... .......... .......... .......... .......... .......... .........G
S88         ......A... .......... .......... .......... .......... .......... .........G
E._albertii ---------- ---------- ---------- ---------- ---------- ---------- ----------
```

```
            9941
K-12         AGCAGCTGTT TGATCTGCCC CCACTTCACG CTGAACGGCA CTTTAACTTT GGTGTTTTGC TGATCCATAT
53638        .......... .......... .......... .......... .......... A......... ..........
ATCC_8739    .......... .......... .......... .......... .......... A......... ..........
HS           .......... .......... .......... .......... .......... A......... ..........
BW2952       .......... .......... .......... .......... .......... .......... ..........
12009        .....T.... .......... .......... .......... .......... .......... ..........
B171         .....T.... .......... .......... .......... .......... .......... ..........
E24377A      .....T.... .......... .......... .......... .......... .......... ..........
SE11         .....T.... .......... .......... .......... .......... .......... ..........
11128        .....T.... .......... .......... .......... .......... .......... ..........
11368        .....T.... .......... .......... .......... .......... .......... ..........
IAI1         .....T.... .......... .......... .......... .......... .......... ..........
E110019      .......... .......... .......... ...G...... .......... A......... ..........
B7A          .....T.... .......... .......... .......... .......... .......... ..........
CB9615       .....T.... .......... .......... .......... .......... .......... ..........
O157_Sakai   .....T.... .......... .......... .......... .......... .......... ..........
IAI39        .......... .......... .......... ........T. .......... .......... ..........
UMN026       .....T.... .......... .......... .......... .......... .......... ..........
SMS-3-5      .......... .......... .......... .......... .......... .......... ..........
536          .......... .......... .......... .......... .......... .......... ..........
APEC_O1      .......... .......... .......... ..A....... .......... .......... ..........
CFT073       .......... .......... .......... .......... .......... .......... ..........
E2348/69     .......... .......... .......... ..A....... .......... .......... ..........
UTI89        .......... .......... .......... ..A....... .......... .......... ..........
F11          .......... .......... .......... .......... .......... .......... ..........
ED1a         .......... .......... .......... ..A....... .......... .......... ..........
83972        .......... .......... .......... .......... .......... .......... ..........
S88          .......... .......... .......... ..A....... .......... .......... ..........
E._albertii  ---------- ---------- ---------- ---------- ---------- ---------- ----------

           10011
K-12         TGATCAGCGC ACCACCCGCG GCGATGTACT CCAGCTCTTT CTTATTTACC CCCGGATGTT GATTTGGCTC
53638        .......... G......... .....A.... .......... ..G......G .......... ..........
ATCC_8739    .......... G......... .....A.... .......... ..G......G .......... ..........
HS           .......... G......... .....A.... .......... ..G......G .......... ..........
BW2952       .......... .......... .......... .......... .......... .......... ..........
12009        .......... G......... .......... .......... ..G....... .......... ..........
B171         .......... G......... .......... .......... ..G....... .......... ......A...
E24377A      .......... G......... .....A.... .......... ..G....... .......... ..........
SE11         .......... G......... .....A.... .......... ..G....... .......... ..........
11128        .......... G......... .......... .......... ..G....... .......... ..........
11368        .......... G......... .......... .......... ..G....... .......... ..........
IAI1         .......... G......... .....A.... .......... ..G....... .......... ..........
E110019      .......... G......... .....A.... .......... ..G....... .......... ..........
B7A          .......... G......... .......... .......... ..G....... .......... ..........
CB9615       .......... G......... .......... .......... ..G....... .......... ..........
O157_Sakai   .......... G......... .......... .......... ..G....... .......... ..........
IAI39        .......... G........C .......... .......... ..G......A .......... ..........
UMN026       .......... G......... .......... .......... ..G....... .......... ..........
SMS-3-5      .......... G......... .......... .......... ..G......A .......... ..........
536          .......... G......... .......... .......... ..G......G .......... ..........
APEC_O1      .......... G......... .......... .......... ..G......G .......... ..........
CFT073       .......... G......... .......... .......... ..G......G .......... ..........
E2348/69     .......... G......... .......... .......... ..G......G A......... ..........
UTI89        .......... G......... .......... .......... ..G......G .......... ..........
F11          .......... G......... .......... .......... ..G......G .......... ..........
ED1a         .......... G......... .......... .......... ..G......G .......... ..........
83972        .......... G......... .......... .......... ..G......G .......... ..........
S88          .......... G......... .......... .......... ..G......G .......... ..........
E._albertii  ---------- ---------- ---------- ---------- ---------- ---------- ----------
```

```
         10081
K-12         GTGGATGACT TTCAACCAGA TAAAGCTGAT GACAATCCCC AGACCGCCCA TAAAGAAGAA GACGTGTGAC
53638        .......... .......... .......... ......G... .......... .......... ..........
ATCC_8739    .......... .......... .......... ......G... .......... .......... ..........
HS           .......... .......... .......... ......G... .......... .......... ..........
BW2952       .......... .......... .......... .......... .......... .......... ..........
12009        .......... .......... .......... .......... .......... .......... ..........
B171         .......... .......... .......... .......... .......... .......... ..........
E24377A      .......... .......... .......... .......... .......... .......... ..........
SE11         .......... .......... .......... .......... .......... .......... ..........
11128        .......... .......... .......... .......... .......... .......... ..........
11368        .......... .......... .......... .......... .......... .......... ..........
IAI1         .......... .......... .......... .......... .......... .......... ..........
E110019      .......... .......... .......... .......... .......... .......... ..........
B7A          .......... .......... .......... .......... .......... .......... ..........
CB9615       .......... .......... .......... .G....G... .......... .......... ..........
O157_Sakai   .......... .......... .......... .G....G... .......... .......... ..........
IAI39        .......... .......... .......... ......G... .......... .......... ..........
UMN026       .......... .......... .......... .......... .......... .......... ..........
SMS-3-5      .......... .......... .......... ......G... .......... .......A.. ..........
536          .......... .......... .......... ......G... .......... .......... ..........
APEC_O1      .......... .......... .......... ......G... .......... .......A.. ..........
CFT073       .......... .......... .......... ......G... .......... .......... ..........
E2348/69     .......... .......... .......... ......G... .......... .......A.. ..........
UTI89        .......... .......... .......... ......G... .......... .......A.. ..........
F11          .......... .......... .......... ......G... .......... .......... ..........
ED1a         .......... .......... .......... ......G... .......... .......A.. ..........
83972        .......... .......... .......... ......G... .......... .......... ..........
S88          .......... .......... .......... ......G... .......... .......A.. ..........
E._albertii  ---------- ---------- ---------- ---------- ---------- ---------- ----------

         10151
K-12         CAGCCCACTT CATGCGTCAG CCAGCCCATA ATCGGCGCGA AGATCACCGT TGCGAAGTAT TGAGCGGAGT
53638        .......... .......... .......... .......... ....A..... .......... ..G.......
ATCC_8739    .......... .......... .......... .......... ....A..... .......... ..G.......
HS           .......... .......... .......... .......... ....A..... .......... ..G.......
BW2952       .......... .......... .......... .......... .......... .......... ..........
12009        .......... .......... .......... .......... .......... .......... ..........
B171         .......... .......... .......... .......... .......... .......... ..........
E24377A      .......... .......... .......... .......... .......... .......... ..........
SE11         .......... .......... .......... .......... .......... .......... ..........
11128        .......... .......... .......... .......... .......... .......... ..........
11368        .......... .......... .......... .......... .......... .......... ..........
IAI1         .......... .......... .......... .......... .......... .......... ..........
E110019      .......... .......... .......... .......... .......... .......... ..........
B7A          .......... .......... .......... .......... .......... .......... ..........
CB9615       .......... .......... .......... .......... .......... .......... ..........
O157_Sakai   .......... .......... .......... .......... .......... .......... ..........
IAI39        .......... .......... .......... .......... .......... .......... ..........
UMN026       .......... .......... .......... .......... .......... .......... ..........
SMS-3-5      .......... .......... .......... .......... .......... .......... ..........
536          .......... ....A....A .......... .......... ....A..... .......... ..G.......
APEC_O1      .......... .......... .......... .......... ....A..... .......... ..........
CFT073       .......... ....A....A .......... .......... ....A..... .......... ..G.......
E2348/69     .......... .......... .......... .......... ....A..... .......... ..........
UTI89        .......... .......... .......... .......... ....A..... .......... ..........
F11          .......... ....A....A .......... .......... ....A..... .......... ..G.......
ED1a         .......... .......... .......... .......... ....A..... .......... ..........
83972        .......... ....A....A .......... .......... ....A..... .......... ..G.......
S88          .......... .......... .......... .......... ....A..... .......... ..........
E._albertii  ---------- ---------- ---------- ---------- ---------- ---------- ----------
```

```
           10221
K-12          TAAAAATCGA CACCGCCGTT CCCCTTTCCT GCGCCGGAAA CCAGGCCGCA ACAATGCGAC TGTTGCCGGG
53638         .......... .......... .......... .......... .......... .......... ..........
ATCC_8739     .......... .......... .......... .......... .......... .......... ..........
HS            .......... .......... .......... .......... .......... .......... ..........
BW2952        .......... .......... .......... .......... .......... .......... ..........
12009         .......... .......... .......... .......... .......... .......... ..........
B171          .......... .......... .......... .......... .......... .......... ..........
E24377A       .......... .......... .......... .......... .......... .......... ..........
SE11          .......... .......... .......... .......... .......... .......... ..........
11128         .......... .......... .......... .......... .......... .......... ..........
11368         .......... .......... .......... .......... .......... .......... ..........
IAI1          .......... .......... .......... .......... .......... .......... ..........
E110019       .......... .......... .......... .......... .......... .......... ..........
B7A           .......... .......... .......... .......... .......... .......... ..........
CB9615        .......... .......... .......... .......... .......... .......... ..........
O157_Sakai    .......... .......... .......... .......... .......... .......... ..........
IAI39         .......... .......... .......... .......... .........T .......... ....A.....
UMN026        .......... .......... .......... .......... .......... .......... ..........
SMS-3-5       .......... .......... .......... .......... .......... .......... ..........
536           .......... .......... .......... .......... .......... ........G. .......A..
APEC_O1       .......... .......... .......... .......... .......... .......... ..........
CFT073        .......... .......... .......... .......... .......... ........G. .......A..
E2348/69      .......... .......... .......... .......... .......... .......... ..........
UTI89         .......... .......... .......... .......... .......... .......... ..........
F11           .......... .......... .......... .......... .......... ........G. .......A..
ED1a          .......... .......... .......... .......... .......... .......... ..........
83972         .......... .......... .......... .......... .......... ........G. .......A..
S88           .......... .......... .......... .......... .......... .......... ..........
E._albertii   ---------- ---------- ---------- ---------- ---------- ---------- ----------

           10291
K-12          GAAAGATGGC GCTTCAGCAA GCCCGACCAG GAAGCGCAGC GTAAACAGGG CAACGATAAT GCCGAATCCA
53638         .......... .......... .......... .......... .......... .......... ..........
ATCC_8739     .......... .......... .......... .......... .......... .......... ..........
HS            .......... .......... .......... .......... .......... .......... ..........
BW2952        .......... .......... .......... .......... .......... .......... ..........
12009         .......... .......... .......... .........A .......... .......... ..........
B171          .......... .......... .......... .........A .......... .......... ..........
E24377A       .......... .......... .......... .........A .......... .......... ..........
SE11          .......... .......... .......... .........A .......... .......... ..........
11128         .......... .......... .......... .......... .......... .......... ..........
11368         .......... .......... .......... .......... .......... .......... ..........
IAI1          .......... .......... .......... .........A .......... .......... ..........
E110019       .......... .......... .......... .........A .......... .......... ..........
B7A           .......... .......... .......... .........A .......... .......... ..........
CB9615        .......... .......... .......... .........A .......... .......... ..........
O157_Sakai    .......... .......... .......... .........A .......... .......... ..........
IAI39         .......... .......... .......... .......... .......... .......... ..........
UMN026        .......... .......... .......... .......... .......... .......... ..........
SMS-3-5       ......G... .......... .......... .......... .......... .......... ..........
536           ......G... .......... .......... .........T .......... .......... ..........
APEC_O1       ......G... .......... .......... .......... .......... .......... ..........
CFT073        ......G... .......... .......... .........T .......... .......... ..........
E2348/69      ......G... .......... .......... .......... .......... .......... ..........
UTI89         ......G... .......... .......... .......... .......... .......... ..........
F11           ......G... .......... .......... .........T .......... .......... ..........
ED1a          ......G... .......... .......... .......... .......... .......... ..........
83972         ......G... .......... .......... .........T .......... .......... ..........
S88           ......G... .......... .......... .......... .......... .......... ..........
E._albertii   ---------- ---------- ---------- ---------- ---------- ---------- ----------
```

```
         10361
K-12         CTAAAGATAT CGACGAAGCC TTGCAGCAAG GTAAACATCG ACCAGATAAA GATCGACCAG AAGTAGACGC
53638        .......... .......... .......... .......... .......... .......... ..........
ATCC_8739    .......... .......... .......... .......... .......... .......... ..........
HS           .......... .......... .......... .......... .......... .......... ..........
BW2952       .......... .......... .......... .......... .......... .......... ..........
12009        .......... .......... .......... .......... .......... .......... ..........
B171         .......... .......... .......... .......... .......... .......... ..........
E24377A      .......... .......... .......... .......... .......... .......... ..........
SE11         .......... .......... .......... .......... .......... .......... ..........
11128        .......... .......... .......... .......... .......... .......... ..........
11368        .......... .......... .......... .......... .......... .......... ..........
IAI1         .......... .......... .......... .......... .......... .......... ..........
E110019      .......... .......... .......... .......... .......... .......... ..........
B7A          .......... .......... .......... .......... .......... .......... ..........
CB9615       .......... .......... .......... .......... .......... .......... ..........
O157_Sakai   .......... .......... .......... .......... .......... .......... ..........
IAI39        .....A.... .......... .......... .......... .......... .......... ..........
UMN026       .......... .......... .......... .......... .......... .......... ..........
SMS-3-5      .......... .......... .......... .......... .......... .......... ..........
536          .......... .......... .......... .......... .......... .......... ..........
APEC_O1      .......... .......... .......... .......... .......... .......... ..........
CFT073       .......... .......... .......... .......... .......... .......... ..........
E2348/69     .......... .......... .......... .......... .......... .......... ..........
UTI89        .......... .......... .......... .......... .......... .......... ..........
F11          .......... .......... .......... .......... .......... .......... ..........
ED1a         .......... .......... .......... .......... .......... .......... ..........
83972        .......... .......... .......... .......... .......... .......... ..........
S88          .......... .......... .......... .......... .......... .......... ..........
E._albertii  ---------- ---------- ---------- ---------- ---------- ---------- ----------

         10431
K-12         GTTTTGAACC AAAACGGTCC AGCAACCAGC CACCAGGGAT CTGCCCGATA ACATAAGCCC ATGAGAAAGC
53638        .......... .......... .......... .......... .......... .......... ..........
ATCC_8739    .......... .......... .......... .......... .......... .......... ..........
HS           .......... .......... .......... .......... .......... .......... ..........
BW2952       .......... .......... .......... .......... .......... .......... ..........
12009        .......... ......A... ........A. .......... .......... .......... .......G..
B171         .......... ......A... ........A. .......... .......... .......... .......G..
E24377A      .......... .......... .......... .......... .......... .......... .......G..
SE11         .......... .......... .......... .......... .......... .......... .......G..
11128        .......... ......A... ........A. .......... .......... .......... .......G..
11368        .......... ......A... ........A. .......... .......... .......... .......G..
IAI1         .......... .......... .......... .......... .......... .......... .......G..
E110019      .......... .......... .......... .......... .......... .......... .......G..
B7A          .......... ......A... ........A. .......... .......... .......... .......G..
CB9615       .......... ......A... ........A. .......... .......... .......... .......G..
O157_Sakai   .......... ......A... ........A. .......... .......... .......... .......G..
IAI39        .......... ......A... ........A. .......... .......... .......... ..........
UMN026       .......... ......A... ........A. .......... .......... .......... .......G..
SMS-3-5      .......... ......A... ........A. .......... .......... .......... ..........
536          .......... ......A... .......... .......... .......... .....C.... .......G..
APEC_O1      .......... ......A... .......... .......... .......... .....C.... .......G..
CFT073       .......... ......A... .......... .......... .......... .....C.... .......G..
E2348/69     .......... ......A... .......... .......... .......... .....C.... .......G..
UTI89        .......... ......A... .......... .......... .......... .....C.... .......G..
F11          .......... ......A... .......... .......... .......... .....C.... .......G..
ED1a         .......... ......A... .......... ....T..... .......... .....C.... .......G..
83972        .......... ......A... .......... .......... .......... .....C.... .......G..
S88          .......... ......A... .......... .......... .......... .....C.... .......G..
E._albertii  ---------- ---------- ---------- ---------- ---------- ---------- ----------
```

```
          10501
K-12         AGAGAACACA TAGCCCATTC CCACGGGATC AAGGCCGATA TCTTTGGCCA TTTCCGAACC GGCGATAGAG
53638        .......... .......... ....A..... .......... .......... .......... ..........
ATCC_8739    .......... .......... ....A..... .......... .......... .......... ..........
HS           .......... .......... ....A..... .......... .......... .......... ..........
BW2952       .......... .......... .......... .......... .......... .......... ..........
12009        .......... .......... .......... .......... .......... .......... ...A.....A
B171         .......... .......... .......... .......... .......... .......... ...A.....A
E24377A      .......... .......... .......... .......... .......... .......... ...A.....A
SE11         .......... .......... .......... .......... .......... .......... ...A.....A
11128        .......... .......... .......... .......... .......... .......... A..A.....A
11368        .......... .......... .......... .......... .......... .......... A..A.....A
IAI1         .......... .......... .......... .......... .......... .......... ...A.....A
E110019      .......... .......... .......... .......... .......... .......... ...A.....A
B7A          .......... .......... .......... .......... .......... .......... ...A.....A
CB9615       .......... .......... .......... .......... .......... .......... ...A.....A
O157_Sakai   .......... .......... .......... .......... .......... .......... ...A.....A
IAI39        .......... .......... ....A..... .......... .......... .......... ..........
UMN026       .......... .......... .......... .......... .......... .......... ...A.....A
SMS-3-5      .......... .......... ....A..... .......... .......... .......... ..........
536          .......... .......... .......... .......... .......... .......... ...A.....A
APEC_O1      .......... .......... .......... .......... .......... .......... ...A.....A
CFT073       .......... .......... .......... .......... .......... .......... ...A.....A
E2348/69     .......... .......... .......... .......... .......... .......... ...A.....A
UTI89        .......... .......... .......... .......... .......... .......... ...A.....A
F11          .......... .......... .......... .......... .......... .......... ...A.....A
ED1a         .......... .......... .......... .......... .......... .......... ...A.....A
83972        .......... .......... .......... .......... .......... .......... ...A.....A
S88          .......... .......... .......... .......... .......... .......... ...A.....A
E._albertii  ---------- ---------- ---------- ---------- ---------- ---------- ----------

          10571
K-12         AGCGTAGCGC GGTCGCCGTA GTTGAAGGAT GTGACGATAA ACAACATCAC CACTATCCAG TAACGAGCAT
53638        .......... .......... .......... .......... .......... .......... ..........
ATCC_8739    .......... .......... .......... .......... .......... .......... ..........
HS           .......... .......... .......... .......... .......... .......... ..........
BW2952       .......... .......... .......... .......... .......... .......... ..........
12009        .......... .......... .......... .......... .......... .......... ..........
B171         .......... .......... .......... .......... .......... .......... ..........
E24377A      .......... .......... .......... .......... .......... .......... ..........
SE11         .......... .......... .......... .......... .......... .......... ..........
11128        .......... .......... .......... .......... .......... .......... ..........
11368        .......... .......... .......... .......... .......... .......... ..........
IAI1         .......... .......... .......... .......... .......... .......... ..........
E110019      .......... .......... .......... .......... .......... .......... ..........
B7A          .......... .......... .......... .......... .......... .......... ..........
CB9615       .......... .......... .......... .......... .......... .......... ..........
O157_Sakai   .......... .......... .......... .......... .......... .......... ..........
IAI39        .......... .......... .......... .......... .......... .......... ..........
UMN026       .......... .......... .......... .......... .......... .......... ..........
SMS-3-5      .......... .......... .......... .......... .......... .......... ..........
536          .......... .......... .......... .......... .......... .......... ..........
APEC_O1      .......... .......... .......... .......... .......... .......... ..........
CFT073       .......... .......... .......... .......... .......... .......... ..........
E2348/69     .......... .......... .......... .......... .......... .......... ..........
UTI89        .......... .......... .......... .......... .......... .......... ..........
F11          .......... .......... .......... .......... .......... .......... ..........
ED1a         .......... .......... .......... .......... .......... .......... ..........
83972        .......... .......... .......... .......... .......... .......... ..........
S88          .......... .......... .......... .......... .......... .......... ..........
E._albertii  ---------- ---------- ---------- ---------- ---------- ---------- ----------
```

```
        10641
K-12          TTGTGCGTTT TTCCACACTG CTCGCAGCCT GACTTAAAGA ACTCATTGTT GCACTCCTGA AAATTCGCGT
53638         .......... .......... .......... .......... .......... .......... ..........
ATCC_8739     .......... .......... .......... .......... .......... .......... ..........
HS            .......... .......... .......... .......... .......... .......... ..........
BW2952        .......... .......... .......... .......... .......... .......... ..........
12009         .C........ .......... .......... .......... .......... .......... ..........
B171          .C........ .......... .......... .......... .......... .......... ..........
E24377A       .C........ .......... .......... .......... .......... .......... ..........
SE11          .C........ .......... .......... .......... .......... .......... ..........
11128         .C........ .......... .......... .......... .......... .......... ..........
11368         .C........ .......... .......... .......... .......... .......... ..........
IAI1          .C........ .......... .......... .......... .......... .......... ..........
E110019       .C........ .......... .......... .......... .......... .......... ..........
B7A           .C........ .......... .......... .......... .......... .......... ..........
CB9615        .C........ .......... .......... .......... .......... .......... ..........
O157_Sakai    .C........ .......... .......... .......... .......... .......... ..........
IAI39         .C........ .......... .......... .......... .......... .......... ..........
UMN026        .C........ .......... .......... .......... .......... .......... ..........
SMS-3-5       .C........ .......... .......... .......... .......... .......... ..........
536           .C........ .......... .......... .......... .......... .......... ..........
APEC_O1       .C........ .......... .......... .......... .......... .......... ..........
CFT073        .C........ .......... .......... .......... .......... .......... ..........
E2348/69      .C........ .......... .......... .......... .......... .......... ..........
UTI89         .C........ .......... .......... .......... .......... .......... ..........
F11           .C........ .......... .......... .......... .......... .......... ..........
ED1a          .C........ .......... .......... .......... .......... .......... ..........
83972         .C........ .......... .......... .......... .......... .......... ..........
S88           .C........ .......... .......... .......... .......... .......... ..........
E._albertii   ---------- ---------- ---------- ---------- ---------- ---------- ----------

        10711
K-12          TAGCCACGCT CACTCTGGAC TGCGACATCG CCAGGAAATC AGAGGTGACG TAGGGTGTTT TTTGCCGTTT
53638         .......... .......... .......... .......... .......... .......... ..........
ATCC_8739     .......... .......... .......... .......... .......... .......... ..........
HS            .......... .......... .......... .......... .......... .......... ..........
BW2952        .......... .......... .......... .......... .......... .......... ..........
12009         .......... .......... ........A. .......... .......... .......... ..........
B171          .......... .......... ........A. .......... .......... .......... ..........
E24377A       .......... .......... ........A. .......... .......... .......... ..........
SE11          .......... .......... ........A. .......... .......... .......... ..........
11128         .......... .......... .......... .......... .......... .......... ..........
11368         .......... .......... .......... .......... .......... .......... ..........
IAI1          .......... .......... .......... .......... .......... .......... ..........
E110019       .......... .......... ........A. .......... .......... .......... ..........
B7A           .......... .......... ........A. .......... .......... .......... ..........
CB9615        .......... .......... .......... .......... .......... .......... ..........
O157_Sakai    .......... .......... .......... .......... .......... .......... ..........
IAI39         .......... .......... .......... .......... .......... .......... ..........
UMN026        .......... .......... .......... .......... .......... .......... ..........
SMS-3-5       .......... .......... .......... .......... .......... .......... ..........
536           .......... .......... .......... .......... .......... .......... ..-.......
APEC_O1       .......... .......... .......... .......... .......... .......... ..-.......
CFT073        .......... .......... .......... .-........ .......... .......... ..-.......
E2348/69      .......... .......... .......... .......... .......... .......... ..-.......
UTI89         .......... .......... .......... .......... .......... .......... ..-.......
F11           .......... .......... .......... .......... .......... .......... ..-.......
ED1a          .......... .......... .......... .......... .......... .......... ..-.......
83972         .......... .......... .......... .-........ .......... .......... ..-.......
S88           .......... .......... .......... .......... .......... .......... ..-.......
E._albertii   ---------- ---------- ---------- ---------- ---------- ---------- ----------
```

```
        10781
K-12         TTATAGGTCG TTCGCCGAAT ACGGCGCGTG TTTATATCTG GCAATAGCAG TATAAAAAGC GCGCCATAGC
53638        .......... .......... .......... .......... .......... .......... ..........
ATCC_8739    .......... .......... .......... .......... .......... .......... ..........
HS           .......... .......... .......... .......... .......... .......... ..........
BW2952       .......... .......... .......... .......... .......... .......... ..........
12009        .......... .......... .......... .......... .......... .......... ..........
B171         .......... .......... .......... .......... .......... .......... ..........
E24377A      .......... .......... .......... .......... .......... .......... ..........
SE11         .......... .......... .......... .......... .......... .......... ..........
11128        .......... .......... .......... .......... .......... .......... ..........
11368        .......... .......... .......... .......... .......... .......... ..........
IAI1         .......... .......... .......... .......... .......... .......... ..........
E110019      .......... .......... .......... .......... .......... .......... ..........
B7A          .......... .......... .......... .......... .......... .......... ..........
CB9615       .......... .......... .......... .......... .......... .......... ..........
O157_Sakai   .......... .......... .......... .......... .......... .......... ..........
IAI39        .......... .......... G......... .......... .......... .......... ..........
UMN026       .......... .......... .......... .......... .......... .......... ..........
SMS-3-5      .......... .......... .......... .......... .......... .......... ..........
536          .......... .......... .......... .......... .......... .......... ..........
APEC_O1      .......... ...A...... .......... .......... .......... .......... ..........
CFT073       .......... .......... .......... .......... .......... .......... ..........
E2348/69     .......... .......... .......... .......... .......... .......... ..........
UTI89        .......... .......... .......... .......... .......... .......... ..........
F11          .......... .......... .......... .......... .......... .......... ..........
ED1a         .......... .......... .......... .......... .......... .......... ..........
83972        .......... .......... .......... .......... .......... .......... ..........
S88          .......... ...A...... .......... .......... .......... .......... ..........
E._albertii  ---------- ---------- ---------- ---------- ---------- ---------- ----------

        10851
K-12         GGCTCACCGT GCAACAACAC AACATTAATG CGTTCAATGA GGCCCGATTT TGGCATTAGC CCTGGACGGT
53638        .......... .......... .......... .......... .......... .......... ..........
ATCC_8739    .......... .......... .......... .......... .......... .......... ..........
HS           .......... .......... .......... .......... .......... .......... ..........
BW2952       .......... .......... .......... .......... .......... .......... ..........
12009        .......... .......... .......... .......... .......... .......... ..........
B171         .......... .......... .......... .......... .......... .......... ..........
E24377A      .......... .......... .......... .......... .......... .......... ..........
SE11         .......... .......... .......... .......... .......... .......... ..........
11128        .......... .......... .......... .......... .......... .......... ..........
11368        .......... .......... .......... .......... .......... .......... ..........
IAI1         .......... .......... .......... .......... .......... .......... ..........
E110019      .......... .......... .......... .......... .......... .......... ..........
B7A          .......... .......... .......... .......... .......... .......... ..........
CB9615       .......... .......... .......... .......... .......... .......... ..........
O157_Sakai   .......... .......... .......... .......... .......... .......... ..........
IAI39        .......... .......... .......... .......... .......... .......... ..........
UMN026       .......... .......... .......... .......... .....A.... .......... ..........
SMS-3-5      .......... .......... .......... .......... .......... .......... ..........
536          .......... .......... .......... .......... .......... .......... ..........
APEC_O1      .......... .......... .......... .......... .......... .......... ..........
CFT073       .......... .......... .......... .......... .......... .......... ..........
E2348/69     .......... .......... .......... .......... .......... .......... ..........
UTI89        .......... .......... .......... .......... .......... .......... ..........
F11          .......... .......... .......... .......... .......... .......... ..........
ED1a         .......... .......... .......... .......... .......... .......... ..........
83972        .......... .......... .......... .......... .......... .......... ..........
S88          .......... .......... .......... .......... .......... .......... ..........
E._albertii  ---------- ---------- ---------- ---------- ---------- ---------- ----------
```

```
         10921
K-12          GGAATCCACT TCACGGAAAT GAAAACAAGA ACAAGAAAGG AAGGGTTAAA ACGAAGAAAT AAAGAAGAGT
53638         .......... .......... .......... .......... .......... .......... ..........
ATCC_8739     .......... .......... .......... .......... .......... .......... ..........
HS            .......... .......... .......... .......... .......... .......... ..........
BW2952        .......... .......... .......... .......... .......... .......... ..........
12009         .......... .......... .......... .......... .......... .......... ..........
B171          .......... .......... .......... .......... .......... .......... ..........
E24377A       .......... .......... .......... .......... .......... .......... ..........
SE11          .......... .......... .......... .......... .......... .......... ..........
11128         .......... .......... .......... .......... ......A... .......... ..........
11368         .......... .......... .......... .......... ......A... .......... ..........
IAI1          .......... .......... .......... .......... ......A... .......... ..........
E110019       .......... .......... .......... .......... .......... .......... ..........
B7A           .......... .......... .......... .......... .......... .......... ..........
CB9615        .......... .......... .......... .......... .......... .......... ..........
O157_Sakai    .......... .......... .......... .......... .......... .......... ..........
IAI39         .......... .......... .......... .......... .......... .......... ..........
UMN026        .......... .......... .......... .......... .......... .......... ..........
SMS-3-5       .......... .......... ........A. .......... .......... T......... ..........
536           .......... .......... .......... .......... .......... .......... ..........
APEC_O1       .......... .......... .......... .......... .......... .......... ..........
CFT073        .......... .......... .......... .......... .......... .......... ..........
E2348/69      .......... .......... .......... .......... ....A..... .......... ..........
UTI89         .......... .......... .......... .......... .......... .......... ..........
F11           .......... .......... .......... .......... .......... .......... ..........
ED1a          .......... .......... .......... .......... .......... .......... ..........
83972         .......... .......... .......... .......... .......... .......... ..........
S88           .......... .......... .......... .......... .......... .......... ..........
E._albertii   ---------- ---------- ---------- ---------- ---------- ---------- ----------

         10991
K-12          ATGAAATGGA TCGCTTGACT CCAGGCAAAC GCCAGTAAAA ATCCGCGCTA TGAAGCAGTT TTTACTGGCA
53638         .......... .......... .......... .......... ......A... .......... ..........
ATCC_8739     .......... .......... .......... .......... ......A... .......... ..........
HS            .......... .......... .......... .......... ......A... .......... ..........
BW2952        .......... .......... .......... .......... .......... .......... ..........
12009         .......... .......... .......... .......... .......... .......... ..........
B171          .......... .......... .......... .......... .......... .......... ..........
E24377A       .......... .......... .......... .......... .......... .......... ..........
SE11          .......... .......... .......... .......... .......... .......... ..........
11128         .......... .......... .......... .......... .......... .......... ..........
11368         .......... .......... .......... .......... .......... .......... ..........
IAI1          .......... .......... .......... .......... .......... .......... ..........
E110019       .......... .......... .......... .......... .......... .......... ..........
B7A           .......... .......... .......... .......... .......... .......... ..........
CB9615        .......... .......... .......... .......... .......... .......... ..........
O157_Sakai    .......... .......... .......... .......... .......... .......... ..........
IAI39         .......... .......... .......... .......... .......... .......... ..........
UMN026        .......... .......... .......... .......... .......... .......... ..........
SMS-3-5       .......... .......... .......... .......... .......... .......... ..........
536           .......... .......... .......... .......... .......... .......... ..........
APEC_O1       .......... .......... .......... .......... .......... .......... ..........
CFT073        .......... .......... .......... .......... .......... .......... ..........
E2348/69      .......... .......... .......... .......... .......... .......... ..........
UTI89         .......... .......... .......... .......... .......... .......... ..........
F11           .......... .......... .......... .......... .......... .......... ..........
ED1a          .......... .......... .......... .......... .......... .......... ..........
83972         .......... .......... .......... .......... .......... .......... ..........
S88           .......... .......... .......... .......... .......... .......... ..........
E._albertii   ---------- ---------- ---------- ---------- ---------- ---------- ----------
```

```
            11061
K-12         TTTGCCTGAA AAGATTCGAT TCAGCACCGC TAAAACGACA TTTACCGCTC GCTGAACATA TCAGGACAAC
53638        .......... .......... .......T.. .......G.. .......... .......... ..........
ATCC_8739    .......... .......... .......T.. .......G.. .......... .......... ..........
HS           .......... .......... .......T.. .......G.. .......... .......... ..........
BW2952       .......... .......... .......... .......... .......... .......... ..........
12009        .......... ..T....... .......... .......... .......... .......... ..........
B171         .......... ..T....... .......... .......... .......... .......... ..........
E24377A      .......... .......... .......... .......... .......... .......... ..........
SE11         .......... .......... .......... .......... .......... .......... ..........
11128        .......... .......... .......... .......... .......... .......... ..........
11368        .......... .......... .......... .......... .......... .......... ..........
IAI1         .......... .......... .......... .......... .......... .......... ..........
E110019      .......... .......... .......... .......... .......... .......... ..........
B7A          .......... ..T....... .......... .......... .......... .......... ..........
CB9615       .......... .......... .......... .......... .......... .......... ..........
O157_Sakai   .......... .......... .......... .......... .......... .......... ..........
IAI39        .......... .......... .......... .......G.. .......... .......G.. ..........
UMN026       .......... .......... .......... .......G.. .......... .......G.. ..........
SMS-3-5      .......... .......... .......T.. .......G.. .......... .......G.. ..........
536          .......... .......... .......... .......G.. .......... .......... ..........
APEC_O1      .......... .......... .......... .......G.. .......... .......... ..........
CFT073       .......... .......... .......... .......G.. .......... .......... ..........
E2348/69     .......... .......... .......... .......G.. .......... .......... ..........
UTI89        .......... .......... .......... .......G.. .......... .......... ..........
F11          .......... .......... .......... .......G.. .......... .......... ..........
ED1a         .......... .......... .......... .......G.. .......... .......... ..........
83972        .......... .......... .......... .......G.. .......... .......... ..........
S88          .......... .......... .......... .......G.. .......... .......... ..........
E._albertii  ---------- ---------- ---------- ---------- ---------- ---------- ----------

            11131
K-12         AGCGTGCCCC ACTGTTCGAC CCACGGATTT GATTCCGTTT CCGGTTCCGG GTTCTCACTG GCGTCAATCA
53638        .......... .A........ .......... .......... .......... .......... ..........
ATCC_8739    .......... .A........ .......... .......... .......... .......... ..........
HS           .......... .A........ .......... .......... .......... .......... ..........
BW2952       .......... .......... .......... .......... .......... .......... ..........
12009        .......... .......... .......... .......... .......... .......... ..........
B171         .......... .......... .......... .......... .......... .......... ..........
E24377A      .......... .......... .......... .......... .......... .......... ..........
SE11         .......... .......... .......... .......... .......... .......... ..........
11128        .......... .......... .......... .......... .......... .......... ..........
11368        .......... .......... .......... .......... .......... .......... ..........
IAI1         .......... .......... .......... .......... .......... .......... ..........
E110019      .......... .......... .......... .......... .......... .......... ..........
B7A          .......... .......... .......... .......... .......... .......... ..........
CB9615       .......... .......... .......... .......... .......... .......... ..........
O157_Sakai   .......... .......... .......... .......... .......... .......... ..........
IAI39        .......... .A.....A.. .......... .......... .......... .......... ..........
UMN026       .......... .A........ .......... .......... .......... .......... ..........
SMS-3-5      .......... .A........ .......... .......... .......... .......... ..........
536          .T........ .A.....A.. .......... .......... .......... .......... ..........
APEC_O1      .T........ .A.....A.. .......... .......... .......... .......... ..........
CFT073       .T........ .A.....A.. .......... .......... .......... .......... ..........
E2348/69     .T........ .A.....A.. .......... .......... .......... .......... ..........
UTI89        .T........ .A.....A.. .......... .......... .......... .......... ..........
F11          .T........ .A.....A.. .......... .......... .......... .......... ..........
ED1a         .T........ .A.....A.. .......... .......... .......... .......... ..........
83972        .T........ .A.....A.. .......... .......... .......... .......... ..........
S88          .T........ .A.....A.. .......... .......... .......... .......... ..........
E._albertii  ------.... .T.....A.. ...G.....A ..AA...... ....C..... .G........ ..A....C..
```

```
             11201
K-12         ACAGCATTTC GCCAACCCGC TGAGCGCTCT GTTCCTGCAA CAAGGCATCG AACTGTTTGC CGCCATTGCA
53638        .......... .......... .......... .......... .......... .......... ..........
ATCC_8739    .......... .......... .......... .......... .......... .......... ..........
HS           .......... .......... .......... .......... .......... .......... ..........
BW2952       .......... .......... .......... .......... .......... .......... ..........
12009        .......... .......... .......... .......... .......... .......... ..........
B171         .......... .......... .......... .......... .......... .......... ..........
E24377A      ....T..... .......... .......... .......... .......... .......... ..........
SE11         ....T..... .......... .......... .......... .......... .......... ..........
11128        ....T..... .......... .......... .......... .......... .......... ..........
11368        ....T..... .......... .......... .......... .......... .......... ..........
IAI1         ....T..... .......... .......... .......... .......... .......... ..........
E110019      ....T..... .......... .......... .......... .......... .......... ..........
B7A          ....T..... .......... .......... .......... .......... .......... ..........
CB9615       .......... .......... .......... .........G .......... .......... ..........
O157_Sakai   .......... .......... .......... .........G .......... .......... ..........
IAI39        .......... T..C...... .......... .......... .......... .......... ..........
UMN026       .......... T..C..T... .......... .......... .......... .......... ..........
SMS-3-5      .......... T..C..T... .......... .......... .....T.... .......... ..........
536          .......... .......... .......... .......... .......... .......... ..........
APEC_O1      .......... .......... .......... .......... .......... .......... ..........
CFT073       .......... .......... .......... .......... .......... .......... ..........
E2348/69     .......... T..C...... .......... .......... .......... .......... ..........
UTI89        .......... .......... .......... .......... .......... .......... ..........
F11          .......... .......... .......... .......... .......... .......... ..........
ED1a         .......... .......... .......... .......... .......... .......... ..........
83972        .......... .......... .......... .......... .......... .......... ..........
S88          .......... .......... .......... .......... .......... .......... ..........
E._albertii  ....T..... T..C...... ..C..A.... .......A.G ...C...... .....C.... ....G.....

             11271
K-12         GAAATTCACA TAACTACTGT CGCCGAGCGC AATCACGCCA TAACGCAGAT TCGGCTGGAA GCCCAGACTA
53638        .......... .......... .......... .......... .......... .......... ..........
ATCC_8739    .......... .......... .......... .......... .......... .......... ..........
HS           .......... .......... .......... .......... .......... .......... ..........
BW2952       .......... .......... .......... .......... .......... .......... ..........
12009        ......T... .....G.... .......... .......... .......... .......... ..........
B171         ......T... .....G.... .......... .......... .......... .......... ..........
E24377A      ......T... .....G.... .......... .......... .......... .......... ..........
SE11         ......T... .....G.... .......... .......... .......... .......... ..........
11128        ......T... .....G.... .......... .......... .......... .......... ..........
11368        ......T... .....G.... .......... .......... .......... .......... ..........
IAI1         ......T... .....G.... .......... .......... .......... .......... ..........
E110019      ......T... .....G.... .......... .......... .......... .......... ..........
B7A          ......T... .....G.... .......... .......... .......... .......... ..........
CB9615       .......... .......... .......... .......... .......... .......... ..........
O157_Sakai   .......... .......... .......... .......... .......... .......... ..........
IAI39        .......... .......... .......... .......... .......... .......... ......GG..
UMN026       .......... .......... .......... .......... .......... .......... ......GG..
SMS-3-5      .......... .......... .......... .......... .......... .......... ......GG..
536          .......... .......... .......... .......... .......... .......... ..........
APEC_O1      .......... .......... .......... .......... .......... .......... ..........
CFT073       .......... .......... .......... .......... .......... .......... ..........
E2348/69     .......... .......... .......... .......... .......... .......... ..........
UTI89        .......... .......... .......... .......... .......... .......... ..........
F11          .......... .......... .......... .......... .......... .......... ..........
ED1a         .......... .......... .......... .......... .......... .......... ..........
83972        .......... .......... .......... .......... .......... .......... ..........
S88          .......... .......... .......... .......... .......... .......... ..........
E._albertii  .......... ..G..G.... ....A..... .......... .......... .......... C.....G...
```

```
         11341
K-12          TCTTTGATTC CCTGAAAGAG TGGCACAATG CTATCAGGAA GGTCGCCCTG CCCGGTCGTG GACGTAACCA
53638         .......... .......... .......... .......... .......... .......... ..........
ATCC_8739     .......... .......... .......... .......... .......... .......... ..........
HS            .......... .......... .......... .......... .......... .......... ..........
BW2952        .......... .......... .......... .......... .......... .......... ..........
12009         .......... .......... C......... .......... .......... .......... ..........
B171          .......... .......... C......... .......... .......... .......... ..........
E24377A       .......... .......... C......... .......... .......... .......... ..........
SE11          .......... .......... C......... .......... .......... .......... ..........
11128         .......... .......... C......... .......... .......... .......... ..........
11368         .......... .......... C......... .......... .......... .......... ..........
IAI1          .......... .......... C......... .......... .......... .......... ..........
E110019       .......... .......... C......... .......... .......... .......... ..........
B7A           .......... .......... C......... .......... .......... .......... ..........
CB9615        .......... .......... C......... .......... .......... .......... ..........
O157_Sakai    .......... .......... C......... .......... .......... .......... ..........
IAI39         .....A.... .......... C......... .A........ .......... .......... ..........
UMN026        .......... .......... C......... .......... .......... .......... ..........
SMS-3-5       .....A.... .......... C......... .A........ .......... .......... ..........
536           .......... .......... .........A .......... .......... .......... ..........
APEC_O1       .......... .......... .......... .......... .......... .......... ..........
CFT073        .......... .......... .......... .......... .......... .......... ..........
E2348/69      .......... .......... .......... .......... .......... .......... ..........
UTI89         .......... .......... .......... .......... .......... .......... ..........
F11           .......... .......... .........A .......... .......... .......... ..........
ED1a          .......... .......... .......... .......... .......... .......... ..........
83972         .......... .......... .......... .......... .......... .......... ..........
S88           .......... .......... .......... .......... .......... .......... ..........
E._albertii   ..C..T.... .......... C......... ..G..G.... .A........ G......... ..G..T....

         11411
K-12          CCAGAACATA CTTATCCTGA TAGGGCAGCC AGTCGCTTAA TTCAGGATCT TCAAATACCG TTGCTTTGTG
53638         .......... .......... .......... .......... .......... .......... ..........
ATCC_8739     .......... .......... .......... .......... .......... .......... ..........
HS            .......... .......... .......... .......... .......... .......... ..........
BW2952        .......... .......... .......... .......... .......... .......... ..........
12009         .......... .......... .......... .......... .......... .......... ..........
B171          .......... .......... .......... .......... .......... .......... ..........
E24377A       .......... .......... ..A....... .......... .......... .......... ..........
SE11          .......... .......... ..A....... .......... .......... .......... ..........
11128         .......... .......... .......... .......... .......... .......... ..........
11368         .......... .......... .......... .......... .......... .......... ..........
IAI1          .......... .......... ..A....... .......... .......... .......... ..........
E110019       .......... .......... ..A....... .......... .......... .......... ..........
B7A           .......... .......... ..A....... .......... .......... .......... ..........
CB9615        .......... .......... .......... .......... .......... .......... ..........
O157_Sakai    .......... .......... .......... .......... .......... .......... ..........
IAI39         .......... .......... .......... .......... .......... .......... ..........
UMN026        .......... .......... .......... .......... .......... .......... ..........
SMS-3-5       .......... .......... .......... .......... .......... .......... ..........
536           .......... .......... .......... .......... .......... .......... ..........
APEC_O1       .......... .......... .......... .......... .......... .......... ..........
CFT073        .......... .......... .......... .......... .......... .......... ..........
E2348/69      .......... .......... .......... .......... ......C... .......... ..........
UTI89         .......... .......... .......... .......... .......... .......... ..........
F11           .......... .......... .......... .......... .......... .......... ..........
ED1a          .......... .......... .......... .......... .......... .......... ..........
83972         .......... .......... .......... .......... .......... .......... ..........
S88           .......... .......... .......... .......... .......... .......... ..........
E._albertii   ....T..... ...G...... ..T....A.. .....T.... .......... ........A. .C........
```

```
         11481
K-12         GCCCTGCGCG GTCAGAATCG CTTCCGCTTC TTCGGCCACT AACAGTGAAT TCCCGTACAT GGTGCCGACA
53638        .......... .......... .......... .......... .......... .......... ..........
ATCC_8739    .......... .......... .......... .......... .......... .......... ..........
HS           .......... .......... .......... .......... .......... .......... ..........
BW2952       .......... .......... .......... .......... .......... .......... ..........
12009        ......T... ........T. ....T..... ...C...... .....C.... .T........ ..........
B171         ......T... ........T. ....T..... ...C...... .....C.... .T........ ..........
E24377A      ......T... ........T. ....T..... ...C...... .....C.... .T........ ..........
SE11         ......T... ........T. ....T..... ...C...... .....C.... .T........ ..........
11128        ......T... ........T. ....T..... ...C...... .....C.... .T........ ..........
11368        ......T... ........T. ....T..... ...C...... .....C.... .T........ ..........
IAI1         ......T... ........T. ....T..... ...C...... .....C.... .T........ ..........
E110019      ......T... ........T. ....T..... ...C...... .....C.... .T........ ..........
B7A          ......T... ........T. ....T..... ...C...... .....C.... .T........ ..........
CB9615       ......T... ........T. ....T..... ...C...... .....C.... .T........ ..........
O157_Sakai   ......T... ........T. ....T..... ...C...... .....C.... .T........ ..........
IAI39        .......... ........T. .......... ...C...... .....C.... .T........ A.........
UMN026       ......T... ........T. ....T..... ...C...... .....C.... .T........ ..........
SMS-3-5      .......... ........T. .......... ...C...... .....C.... .T........ A.........
536          .......... .......... .......... .......... .......... .......... ..........
APEC_O1      .......... .......... .......... .......... .......... .......... ..........
CFT073       .......... .......... .......... .......... .......... .......... ..........
E2348/69     .......... .......... .......... .......... .......... .......... ..........
UTI89        .......... .......... .......... .......... .......... .......... ..........
F11          .......... .......... .......... .......... .......... .......... ..........
ED1a         .......... .......... .......... .......... .......... .......... ..........
83972        .......... .......... .......... .......... .......... .......... ..........
S88          .......... .......... .......... .......... .......... .......... ..........
E._albertii  C..T..TCT. .A..A..... .......... ......A... .G..A..CG. .G........ ......C...

         11551
K-12         AAAATACCAA TTTCCGCCAT ACCGTTTTCT CCCTGGATTA GGAACTTATC TCTCCATCCT GACCCGATGG
53638        .......... .......... .......... .......... .......... .......... ..........
ATCC_8739    .......... .......... .......... .......... .......... .......... ..........
HS           .......... .......... .......... .......... .......... .......... ..........
BW2952       .......... .......... .......... .......... .......... .......... ..........
12009        .......... .......... .......... .......... .......... .......... ..........
B171         .......... .......... .......... .......... .......... .......... ..........
E24377A      .......... .......... .......... .......... .......... .......... ..........
SE11         .......... .......... .......... .......... .......... .......... ..........
11128        .......... .......... .......... .......... .......... .......... ..........
11368        .......... .......... .......... .......... .......... .......... ..........
IAI1         .......... .......... .......... .......... .......... .......... ..........
E110019      .......... .......... .......... .......... .......... .......... ..........
B7A          .......... .......... .......... .......... .......... .......... ..........
CB9615       .......... .......... .......... .......... .......... .......... ..........
O157_Sakai   .......... .......... .......... .......... .......C.. .......... ..........
IAI39        .......... .....A.... .......... .......... .......... .......... ..........
UMN026       .......... .......... .......... .......... .......... .......... ..........
SMS-3-5      .......... .......... .......... .......... .......... .......... ..........
536          .......... .......... .......... .......... .......... .......... ..........
APEC_O1      .......... .......... .......... .......... .......... .......... ..........
CFT073       .......... .......... .......... .......... .......... .......... ..........
E2348/69     .......... .......... .......... .......... .......... .......... ..........
UTI89        .......... .......... .......... .......... .......... .......... ..........
F11          .......... .......... .......... .......... .......... .......... ..........
ED1a         .......... .......... .......... .......... .......... .......... ..........
83972        .......... .......... .......... .......... .......... .......... ..........
S88          .......... .......... .......... .......... .......... .......... ..........
E._albertii  .......... .......... .......... .......... .........G ..A....... .........A
```

```
         11621
K-12          CGCACTGAAC TCAACCCTTT CATTTTCAGG AAGCAGACCG CGCCAGCCAA ATTGTGATAA CGCCTGCATC
53638         .......... .......... .......... .......... .......... .......... ..........
ATCC_8739     .......... .......... .......... .......... .......... .......... ..........
HS            .......... .......... .......... .......... .......... .......... ..........
BW2952        .......... .......... .......... .......... .......... .......... ..........
12009         .......... .......... .......... .......... .......... .......... ..........
B171          .......... .......... .......... .......... .......... .......... ..........
E24377A       .......... .......... .......... .......... .......... .......... ..........
SE11          .......... .......... .......... .......... .......... .......... ..........
11128         .......... .......... .......... .......... .......... .......... ..........
11368         .......... .......... .......... .......... .......... .......... ..........
IAI1          .......... .......... .......... .......... .......... .......... ..........
E110019       .......... .......... .......... .......... .......... .......... ..........
B7A           .......... .......... .......... .......... .......... .......... ..........
CB9615        .......... .......... .......... .......... .......... .......... ..........
O157_Sakai    .......... .......... .......... .......... .......... .......... ..........
IAI39         .......... .......... .......... .........C .......... .......... ..........
UMN026        .......... .......... .......... .......... .......... .......... ..........
SMS-3-5       .......... .......... .......... .........C .......... .......... ..........
536           .......... .......... .......... .......... .......... .......... T.........
APEC_O1       .......... .......... .......... .........C .......... .......... ..........
CFT073        .......... .......... .......... .......... .......... .......... T.........
E2348/69      .......... .......... .......... .......... .......... .......... ..........
UTI89         .......... .......... .......... .........C .......... .......... ..........
F11           .......... .......... .......... .......... .......... .......... T.........
ED1a          .......... .......... .......... .......... .......... .......... ..........
83972         .......... .......... .......... .......... .......... .......... T.........
S88           ......A... .......... .......... .........C .......... .......... ..........
E._albertii   .T.G...... .......... .......... .......... .......... .........G ..........

         11691
K-12          CAGGTGTCGT CCAGGCCCGC GTGAATAGTC AGCGGCTCGC CAGTAAAAGG ATGCGTCAAT GACAACTGAC
53638         .......... .......... .......... .......... .......... .......... ..........
ATCC_8739     .......... .......... .......... .......... .......... .......... ..........
HS            .......... .......... .......... .......... .......... .......... ..........
BW2952        .......... .......... .......... .......... .......... .......... ..........
12009         .......... .......... .......... .......... .......... .......... ..........
B171          .......... .......... .......... .......... .......... .......... ..........
E24377A       .......... .......... .......... .......... .......... .......... ..........
SE11          .......... .......... .......... .......... .......... .......... ..........
11128         .......... .......... .......... .......... .......... .......... ..........
11368         .......... .......... .......... .......... .......... .......... ..........
IAI1          .......... .......... .......... .......... .......... .......... ..........
E110019       .......... .......... .......... .......... .......... .......... ..........
B7A           .......... .......... .......... .......... .......... .......... ..........
CB9615        .......... .......... .......... ........A. .......... .......... ..........
O157_Sakai    .......... .......... .......... ........A. .......... .......... ..........
IAI39         .......... .......... .......... .......... .......... .......... ..........
UMN026        .......... .......... .......... .......... .......... .......... ..........
SMS-3-5       .......... .......... .......... .......... .......... .......... ..........
536           .......... .......... ........C. .......... .......... .......... ..........
APEC_O1       .......... .......... .......... .......... .......... .......... ..........
CFT073        .......... .......... ........C. .......... .......... .......... ..........
E2348/69      .......... .......... .......... .......... .......... .......... ..........
UTI89         .......... .......... .......... .......... .......... .......... ..........
F11           .......... .......... ........C. .......... .......... .......... ..........
ED1a          .......... .......... .......... .......... .......... .......... ..........
83972         .......... .......... ........C. .......... .......... .......... ..........
S88           .......... .......... .......... .......... .......... .......... ..........
E._albertii   .....A.T.. ....A..... ...G..T..G ........C. .C........ ......T... .......TG.
```

```
        11761
K-12         TGGCATGTAG CATTAACCGC TGGAGGCCAA AATGCTCAGC ACCGCTGCGA TTCTGGCGTA AATCGCCATG
53638        .......... .......... .......... .......... .......... .......... ..........
ATCC_8739    .......... .......... .......... .......... .......... .......... ..........
HS           .......... .......... .......... .......... .......... .......... ..........
BW2952       .......... .......... .......... .......... .......... .......... ..........
12009        .......... .......... .......... .......... .......... .......... ..........
B171         .......... .......... .......... .......... .......... .......... ..........
E24377A      .......... .......... .......... .......... .......... .......... ..........
SE11         .......... .......... .......... .......... .......... .......... ..........
11128        .......... .......... .......... .......... .......... .......... ..........
11368        .......... .......... .......... .......... .......... .......... ..........
IAI1         .......... .......... .......... .......... .......... .......... ..........
E110019      .......... .......... ..T....... .......... .......... .......... ..........
B7A          .......... .......... .......... .......... .......... .......... ..........
CB9615       ......C... .......... ..A..A.... .......... .......... .......... ..........
O157_Sakai   ......C... .......... ..A..A.... .......... .......... .......... ..........
IAI39        ......C... .......... ..A....... ....T..... .......... .......... ....A.....
UMN026       ......C.A. .......... ..A....... ....T..... .......... .......... ....A.....
SMS-3-5      ......C... .......... ..A....... ....T..... .......... .......... ....A.....
536          ......C... .......... ..A....... .......... .......... .......... ....A.....
APEC_O1      .A....C... .......... ..A....... ....T..... .......... .......... ....A.....
CFT073       ......C... .......... ..A....... .......... G......... .......... ....A.....
E2348/69     ......C... .......... ..A....... .......... .......... .......... ....A.....
UTI89        .A....C... .......... ..A....... ....T..... .......... .......... ....A.....
F11          ......C... .......... ..A....... .......... .......... .......... ....A.....
ED1a         ......C... .......... ..A....... .......... .......... .......... ....A.....
83972        ......C... .......... ..A....... .......... G......... .......... ....A.....
S88          .A....C... .......... ..A....... ....T..... .......... .......... ....A.....
E._albertii  ......C... .........T ..A....... .G..T..... CG.A...... .......... ..........

        11831
K-12         TTTGCTATCG CCAATAATCG GATGACGCAA ATGGGCAAGA TGTCGGCGAA GCTGATGTTT GCGTCCGGTT
53638        .......... .......... .......... .......... .......... .......... ..........
ATCC_8739    .......... .......... .......... .......... .......... .......... ..........
HS           .......... .......... .......... .......... .......... .......... ..........
BW2952       .......... .......... .......... .......... .......... .......... ..........
12009        .......... .......... .......... .......... .......... .......... ..........
B171         .......... .......... .......... .......... .......... .......... ..........
E24377A      .......... .......... .......... .......... .......... .......... ..........
SE11         .......... .......... .......... .......... .......... .......... ..........
11128        .......... .......... .......... .......... .......... .......... ..........
11368        .......... .......... .......... .......... .......... .......... ..........
IAI1         .......... .......... .......... .......... .......... .......... ..........
E110019      .......... .......... .......... .......... .......... .......... ..........
B7A          .......... .......... .......... .......... .......... .......... ..........
CB9615       .......... .......... .......... .......... .......... .......... ..........
O157_Sakai   .......... .......... .......... .......... .......... .......... ..........
IAI39        .....G.... .......... .G..G..... .......... ..G..A..G. ....G..C.. A.........
UMN026       .....G.... .......... .G..G..... .......... ..G..A..G. ....G..C.. ..........
SMS-3-5      .....G.... .......... .G..G..... .......... ..G..A..G. ....G..C.. A.........
536          .....G.... .......... .G..C..... .......... ..G..A.... ....G..C.. ......A...
APEC_O1      .....G.... .......... .G..G..... .......... ..G..A..G. ....G..C.. A.........
CFT073       .....G.... .......... .G..C..... .......... ..G..A.... ....G..C.. ......A...
E2348/69     .....G.... .......... .G..C..... .......... ..G..A.... ....G..C.. ......A...
UTI89        .....G.... .......... .G..G..... .......... ..G..A..G. ....G..C.. A.........
F11          .....G.... .......... .G..C..... .......... ..G..A.... ....G..C.. ......A...
ED1a         .....G.... .......... .G..C..... .......... ..G..A.... ....G..C.. ......A...
83972        .....G.... .......... .G..C..... .......... ..G..A.... ....G..C.. ......A...
S88          .....G.... .......... .G..G..... .......... ..G..A..G. ....G..C.. A.........
E._albertii  C....G..A. ..G.....G. .......... ...A..G... .....A..T. ....G..C.. ......T...
```

```
        11901
K-12         TTCGGCTCCA GTTCCACCAG GCCGTAGCGC GTGGTCGGGT AACGTCCGGT CGCTACCGGC ATTTCTACGG
53638        .......... .......... .......... .......... .......... .......... ..........
ATCC_8739    .......... .......... .......... .......... .......... .......... ..........
HS           .......... .......... .......... .......... .......... .......... ..........
BW2952       .......... .......... .......... .......... .......... .......... ..........
12009        .......... .......... .......... .......... .......... .......... ..........
B171         .......... .......... .......... .......... .......... .......... ..........
E24377A      .......... .......... .......... .......... .......... .......... ..........
SE11         .......... .......... .......... .......... .......... .......... ..........
11128        .......... .......... .......... .......... .......... .......... ..........
11368        .......... .......... .......... .......... .......... .......... ..........
IAI1         .......... .......... .......... .......... .......... .......... ..........
E110019      .......... .......... .......... .......... .......... .......... ..........
B7A          .......... .......... .......... .......... .......... .......... ..........
CB9615       .......... .......... .......... .......... .......... .......... ..........
O157_Sakai   .......... .......... .......... .......... .......... .......... ..........
IAI39        .......... .......... .......... .......... .......... .......... ..........
UMN026       .......... .......... .......... .......... .......... .......... ..........
SMS-3-5      .......... .......... .......... .......... .......... .......... ..........
536          .......... .......... .......... .......... .......A.. .......... ..........
APEC_O1      .......... ....T..... .......... .......... .......... .......... ..........
CFT073       .......... .......... .......... .......... .......... .......... ..........
E2348/69     .......... .......... .......... .......... .......... .......... ..........
UTI89        .......... ....T..... .......... .......... .......... .......... ..........
F11          .......... .......... .......... .......... .......A.. .......... ..........
ED1a         .......... .......... .......... .......... .......... .......... ..........
83972        .......... .......... .......... .......... .......... .......... ..........
S88          .......... ....T..... .......... .......... .......... .......... ..........
E._albertii  ..T..T.... .C........ .........G ..C....... ....C..... ...C...... ........C.

        11971
K-12         TCGCCAGACC GCGATAATGC GTCACTGCTG GCTGCGGGCC TTTATCTTCG CGGGCAAATT TATCAGCGAT
53638        .......... .......... .......... .......... .......... .......... ..........
ATCC_8739    .......... .......... .......... .......... .......... .......... ..........
HS           .......... .......... .......... .......... .......... .......... ..........
BW2952       .......... .......... .......... .......... .......... .......... ..........
12009        .......... .......... .......... ......A... .......... .......... ..........
B171         .......... .......... .......... ......A... .......... .......... ..........
E24377A      .......... .......... .......... ......A... .......... .......... ..........
SE11         .......... .......... .......... ......A... .......... .......... ..........
11128        .......... .......... .......... ......A... .......... .......... ..........
11368        .......... .......... .......... ......A... .......... .......... ..........
IAI1         .......... .......... .......... ......A... .......... .......... ..........
E110019      .......... .......... .......... ......A... .......... .......... ..........
B7A          .......... .......... .......... ......A... .......... .......... ..........
CB9615       .......... .......... .......... .......... .......... .......... ..........
O157_Sakai   .......... .......... .......... .......... .......... .......... ..........
IAI39        .......... .......... .......... ....T..... .......... .......... ..........
UMN026       .......... .......... .......... ....T..... .......... .......... ..........
SMS-3-5      .......... .......... .......... .......... .......... .......... ..........
536          .......... .......... .......... .......... .......... .......... ..........
APEC_O1      .......... .......... .......... .......... .......... .......... ..........
CFT073       .......... .......... .......... .......... .......... .......... ..........
E2348/69     .......... .......... .......... .......... .......... .......... ..........
UTI89        .......... .......... .......... .......... .......... .......... ..........
F11          .......... .......... .......... .......... .......... .......... ..........
ED1a         .......... .......... .......... .......... .......... .......... ..........
83972        .......... .......... .......... .......... .......... .......... ..........
S88          .......... .......... .......... .......... .......... .......... ..........
E._albertii  .T........ A......... .....C.... ....T..A.. .........A .....G.... ..........
```

```
          12041
K-12         TTTGTCCAGT TCTTCCACCA GTGGATAATC CAGCACCGCT TCTTCCATCA ACCAGCCGCG CACAATCGCA
53638        .......... .......... .......... .......... .......... .......... ..........
ATCC_8739    .......... .......... .......... .......... .......... .......... ..........
HS           .......... .......... .......... .......... .......... .......... ..........
BW2952       .......... .......... .......... .......... .......... .......... ..........
12009        .......... .......... .......... ...A..T... .......... .......... ..........
B171         .......... .......... .......... ...A..T... .......... .......... ..........
E24377A      .......... .......... .......... ...A..T... .......... .......... ..........
SE11         .......... .......... .......... ...A..T... .......... .......... ..........
11128        .......... .......... .......... ...A..T... .......... .......... ..........
11368        .......... .......... .......... ...A..T... .......... .......... ..........
IAI1         .......... .......... .......... ...A..T... .......... .......... ..........
E110019      .......... .......... .......... ...A..T... .......... .......... ..........
B7A          .......... .......... .......... ...A..T... .......... .......... ..........
CB9615       .......... .......... .......... .......... .......... .......... ..........
O157_Sakai   .......... .......... .......... .......... .......... .......... ..........
IAI39        .......... .......... .......... ...A..T... .......... .......... ..........
UMN026       .......... .......... .......... ...A..T... .......... .......... ..........
SMS-3-5      .......... .......... .......... .......... .......... .......... ..........
536          .......... .......... .......... .......... .......... .......A.. ..........
APEC_O1      .......... .......... .......... .......... .......... .......... ..........
CFT073       .......... ......G... .......... .......... .......... .......A.. ..........
E2348/69     .......... .......... .......... .......... .......... .......A.. ..........
UTI89        .......... .......... .......... .......... .......... .......... ..........
F11          .......... .......... .......... .......... .......... .......A.. ..........
ED1a         .......... .......... .......... .......... .......... .......A.. ..........
83972        .......... .......... .......... .......... .......... .......A.. ..........
S88          .......... .......... .......... .......... .......... .......... ..........
E._albertii  ...A.....C .......... ....G..... ...TT....C .......... ....A..... .........G
```

```
          12111
K-12         TGGTAACGTT TCTGGATTTG GTGCTGTTCA AACTGTTGTG CCAGCAGCCG TCCGGCCTCG CTGGATAATC
53638        .......... .......... .......... .......... .......... .......... ..........
ATCC_8739    .......... .......... .......... .......... .......... .......... ..........
HS           .......... .......... .......... .......... .......... .......... ..........
BW2952       .......... .......... .......... .......... .......... .......... ..........
12009        .......... .......... .......... .......... .......... .......... ..........
B171         .......... .......... .......... .......... .......... .......... ..........
E24377A      .......... .......... .......... .......... .......... .......... ..........
SE11         .......... .......... .......... .......... .......... .......... ..........
11128        .......... .......... .......... .......... .......... .......... ..........
11368        .......... .......... .......... .......... .......... .......... ..........
IAI1         .......... .......... .......... .......... .......... .......... ..........
E110019      .......... .......... .......... .......... .......... .......... ..........
B7A          .......... .......... .......... .......... .......... .......... ..........
CB9615       .......... .......... .......... .......... .......... .......... ..........
O157_Sakai   .......... .......... .......... .......... .......... .......... ..........
IAI39        .......... .......... .......... .......... .......... .......... ..........
UMN026       .......... .......... .......... .......... .......... .......... ..........
SMS-3-5      .......... .......... .......... .......... .......... .......... ..........
536          .......... .......... .......... .......... .......... ......T... ..........
APEC_O1      .......... .......... .......... .......... .......... .......... ..........
CFT073       .......... .......... .......... .......... .......... ......T... ..........
E2348/69     .......... .......... .......... .......... .......... ......T... ..........
UTI89        .......... .......... .......... .......... .......... .......... ..........
F11          .......... .......... .......... .......... .......... ......T... ..........
ED1a         .......... .......... .......... .......... .......... ......T... ..........
83972        .......... .......... .......... .......... .......... ......T... ..........
S88          .......... .......... .......... .......... .......... .......... ..........
E._albertii  .......A.. ....A..... .......... ........C. ......A... C......... .....C....
```

```
        12181
K-12         CCATCAACAA CACACCAGAA GTGGGTCGGT CCAGACGATG AGCAGTAAAA ACATGCTGGC CTATCTGGTC
53638        .......... .......... .......... .......... .......... .......... ..........
ATCC_8739    .......... .......... .......... .......... .......... .......... ..........
HS           .......... .......... .......... .......... .......... .......... ..........
BW2952       .......... .......... .......... .......... .......... .......... ..........
12009        .......... .......... .......... .......... ...G...... ..G....... .A.....A..
B171         .......... .......... .......... .......... ...G...... ..G....... .A.....A..
E24377A      .......... .......... .......... .......... ...G...... ..G....... .A.....A..
SE11         .......... .......... .......... .......... ...G...... ..G....... .A.....A..
11128        .......... .......... .......... .......... ...G...... ..G....... .A.....A..
11368        .......... .......... .......... .......... ...G...... ..G....... .A.....A..
IAI1         .......... .......... .......... .......... ...G...... ..G....... .A.....A..
E110019      .......... .......... .......... .......... ...G...... ..G....... .A.....A..
B7A          .......... .......... .......... .......... ...G...... ..G....... .A.....A..
CB9615       .......... .......... .......... .......... .......... .......... ..........
O157_Sakai   .......... .......... .......... .......... .......... .......... ..........
IAI39        .......... .......... ..T....... .......... .........C .........T ..........
UMN026       .......... .......... ..T....... .......... .........C .......... ..........
SMS-3-5      .......... .......... .......... .......... .........C .......... ..........
536          .......... .......... ..T....... .......... .......... .......... ..........
APEC_O1      .......... .......... .......... .......... .........C .......... .....A....
CFT073       .......... .......... ..T....... .......... .......... .......... ..........
E2348/69     .......... .......... ..T....... .......... .......... .......... ..........
UTI89        .......... .......... .......... .......... .........C .......... .....A....
F11          .......... .......... ..T....... .......... .......... .......... ..........
ED1a         .......... .......... ..T....... .......... .......... .......... ..........
83972        .......... .......... ..T....... .......... .......... .......... ..........
S88          .......... .......... .......... .......... .........C .......... .....A....
E._albertii  ......GT.. ...G..G... ..C....... ....G..... T..G...... ........A. .A........

        12251
K-12         ACGCACGGTT TGCATGACCA CTACTTTCTC GTCGCGATCC AGCCAGCTGC GGTGAACCAG CCAGCCGGAG
53638        .......... .......... .......... .......... .......... .......... ..........
ATCC_8739    .......... .......... .......... .......... .......... .......... ..........
HS           .......... .......... .......... .......... .......... .......... ..........
BW2952       .......... .......... .......... .......... .......... .......... ..........
12009        .......... .......... .......... .......... .......... .......... ..........
B171         .......... .......... .......... .......... .......... .......... ..........
E24377A      .......... .......... .......... .......... .......... .......... ..........
SE11         .......... .......... .......... .......... .......... .......... ..........
11128        .......... .......... .......... .......... .......... .......... ..........
11368        .......... .......... .......... .......... .......... .......... ..........
IAI1         .......... .......... .......... .......... .......... .......... ..........
E110019      .......... .......... .......... .......... .......... .......... ..........
B7A          .......... .......... .......... .......... .......... .......... ..........
CB9615       .......... .......... .......... .......... ........A. .......... ..........
O157_Sakai   .......... .......... .......... .......... ........A. .......... ..........
IAI39        .......... .......... .......... .......... .......... .......... ..........
UMN026       .......... .......... .......... .......... .......... .......... ..........
SMS-3-5      .......... .......... .......... .......... .......... .......... ..........
536          .......... .......... .......... .......... .......... .......... ..........
APEC_O1      .......... .......... .......... .......... .......... .......... ..........
CFT073       .......... .......... .......... .......... .......... .......... ..........
E2348/69     .......... .......... .......... .......... .......... .........A ..........
UTI89        .......... .......... .......... .......... .......... .......... ..........
F11          .......... .......... .......... .......... .......... .......... ..........
ED1a         .......... .......... .......... .......... .......... .........A ..........
83972        .......... .......... .......... .......... .......... .......... ..........
S88          .......... .......... .......... .......... .......... .......... ..........
E._albertii  .......... .....C..G. .C........ ...A...... .....A..A. .A.......A .........C
```

```
         12321
K-12          GGTTTATTTA CCGCAACCAG CCATTCATCC TGATAGAGTA TTTCCAGCAT TAGCTCGCAT CATCCGCAAA
53638         .......... .......... .......... .......... .......... .......... ..........
ATCC_8739     .......... .......... .......... .......... .......... .......... ..........
HS            .......... .......... .......... .......... .......... .......... ..........
BW2952        .......... .......... .......... .......... .......... .......... ..........
12009         .......... .......... .......... .......... .......... .......... ..........
B171          .......... .......... .......... .......... .......... .......... ..........
E24377A       .......... .......... .......... .......... .......... .......... ..........
SE11          .......... .......... .......... .......... .......... .......... ..........
11128         .......... .......... .......... .......... .......... .......... ..........
11368         .......... .......... .......... .......... .......... .......... ..........
IAI1          .......... .......... .......... .......... .......... .......... ..........
E110019       .......... .......... .......... .......... .......... .......... ..........
B7A           .......... .......... .......... .......... .......... .......... ..........
CB9615        .......... .......... .......... .......... .......... .......... ..........
O157_Sakai    .......... .......... .......... .......... .......... .......... ..........
IAI39         .......... .......... .......... .......... .......... .......... ..........
UMN026        .......... .......... .......... .......... .......... .......... ..........
SMS-3-5       .......... .......... .......... .......... .......... .......... ..........
536           .......... .......... .......... .......... .......... .......... ..........
APEC_O1       .......... .......... .......... .......... .......... .......... ..........
CFT073        .......... .......... .......... .......... .......... .......... ..........
E2348/69      .......... .......... .......... .......... .......... .......... ..........
UTI89         .......... .......... .......... .......... .......... .......... ..........
F11           .......... .......... .......... .......... .......... .......... ..........
ED1a          .......... .......... .......... .......... .......... .......... ..........
83972         .......... .......... .......... .......... .......... .......... ..........
S88           .......... .......... .......... .......... .......... .......... ..........
E._albertii   ........C. .......... .......... ..G....... .......... C..TGA.T.. ..........

         12391
K-12          AAGAGCATCC AGTTTTTCCA GCTCAGCCAG AATAAGCGCG CGTTGCGGAT GGTCCGTCGC CAGCGCCATT
53638         .......... .......... .......... .......... .......... .......... ..........
ATCC_8739     .......... .......... .......... .......... .......... .......... ..........
HS            .......... .......... .......... .......... .......... .......... ..........
BW2952        .......... .......... .......... .......... .......... .......... ..........
12009         .......... .......... .......... .......... .......... .......... ..........
B171          .......... .......... .......... .......... .......... .......... ..........
E24377A       .......... .......... .......... .......... .......... .......... ..........
SE11          .......... .......... .......... .......... .......... .......... ..........
11128         .......... .......... .......... .......... .......... .......... ..........
11368         .......... .......... .......... .......... .......... .......... ..........
IAI1          .......... .......... A......... .......... .......... .......... ..........
E110019       .......... .......... A......... .......... .......... .......... ..........
B7A           .......... .......... .......... .......... .......... .......... ..........
CB9615        .......... .......... A......... .......... .......... .......... ..........
O157_Sakai    .......... .......... A......... .......... .......... .......... ..........
IAI39         .......... .......... .......... .......... ........G. .......... ...A......
UMN026        .......... .......... .......... .......... ........G. .......... ..........
SMS-3-5       .......... .......... .......... .......... ........G. .......... ..........
536           .......... .......... ...T...... .......... ........G. A......... ..........
APEC_O1       .......... .......... .......... .......... .......... .A..A..... ..........
CFT073        .......... .....C.... .........A .......... .......... .A..A..... ..........
E2348/69      .......... .......... ...T...... .......... ........G. .......... ..........
UTI89         .......... .......... .......... .......... .......... .A..A..... ..........
F11           .......... .......... ...T...... .......... ........G. A......... ..........
ED1a          .......... .......... ...T...... .......... ........G. .......... ..........
83972         .......... .....C.... .........A .......... .......... .A..A..... ..........
S88           .......... .......... .......... .......... .......... .A..A..... ..........
E._albertii   .......... ..C....... .T..T..... ...T.A.... .....G..G. .A........ ..........
```

```
            12461
K-12          TCATAATAGG GTGCAACGGC AAAAGCGCCC GGTAACGGCT GTTTGTTATC TAACAAATCG TGCATTCGCG
53638         .......... .......... .......... .......... .......... .......... ..........
ATCC_8739     .......... .......... .......... .......... .......... .......... ..........
HS            .......... .......... .......... .......... .......... .......... ..........
BW2952        .......... .......... .......... .......... .......... .......... ..........
12009         .......... .......... .......... .......... .......... .......... ..........
B171          .......... .......... .......... .......... .......... .......... ..........
E24377A       .......... .......... .......... .......... .......... .......... ..........
SE11          .......... .......... .......... .......... .......... .......... ..........
11128         .......... .......... .......... .......... .......... .......... ..........
11368         .......... .......... .......... .......... .......... .......... ..........
IAI1          .......... .......A.. ......A... .......... .G.......T .......... ..........
E110019       .......... .......A.. .......... .......... .G.......T .......... ..........
B7A           .......... .......... .......... .......... .......... .......... ..........
CB9615        .......... .......... .......... .......... .G.......T .......... ..........
O157_Sakai    .......... .......... .......... .......... .G.......T .......... ..........
IAI39         .......... .A........ .......... .......... .G........ .......... ..........
UMN026        .......... .A........ .......... .......... .G.....T.. .......... ..........
SMS-3-5       .......... .A........ .......... .......... .G.....T.. .......... ..........
536           ........A. .C.....A.. .......... .......... .G........ .......... ..........
APEC_O1       .......... .C.....A.. .......... .......... .G........ .......... .....C....
CFT073        .......... .C.....A.. .......... .......... .G........ .......... ..........
E2348/69      ........A. .C.....A.. .......... .......... .G........ .......... ..........
UTI89         .......... .C.....A.. .......... .......... .G........ .......... .....C....
F11           ........A. .C.....A.. .......... .......... .G........ .......... ..........
ED1a          .......... .C.....A.. .......... .......... .G........ .......... ..........
83972         .......... .C.....A.. .......... .......... .G........ .......... ..........
S88           .......... .C.....A.. .......... .......... .G........ .......... .....C....
E._albertii   ........C. .C..C..... ...T..CG.. .....A.... .G.G...... ...T...... .....C....

            12531
K-12          GGATCAGCAC CCACTGCAAC CACTCCAGTG GTTCCATGGT GTCCATAAAG AACGGTTGGG TACTATTAAA
53638         .......... .......... .......... .......... .......... .......... ..........
ATCC_8739     .......... .......... .......... .......... .......... .......... ..........
HS            .......... .......... .......... .......... .......... .......... ..........
BW2952        .......... .......... .......... .......... .......... .......... ..........
12009         .......... .......... .......... .......... .......... .......... ..........
B171          .......... .......... .......... .......... .......... .......... ..........
E24377A       .......... .......... .......... .......... .......... .......... ..........
SE11          .......... .......... .......... .......... .......... .......... ..........
11128         .......... .......... .......... .......... .......... .......... ..........
11368         .......... .......... .......... .......... .......... .......... ..........
IAI1          .......... .......... .......... .......... .......... .......... ..........
E110019       .......... .......... .......... .......... .......... .......... ..........
B7A           .......... .......... .......... .......... .......... .......... ..........
CB9615        .......... .......... .......... .......... .......... .......... ..........
O157_Sakai    .......... .......... .......... .......... .......... .......... ..........
IAI39         .......... .......... .......... .......... .......... .......... ..........
UMN026        .......... .......... .......... .......... .......... .......... ..........
SMS-3-5       .......... .......... .......... .......... .......... .......... ..........
536           .......... .......... .......... .......... .......... ........A. ....G.....
APEC_O1       .A.....T.. ...T...... .......... .......... .......... .....C.... ....CGC...
CFT073        .......... .......... .......... .......... .......... ........A. ....G.....
E2348/69      .......... .......... .......... .......... .......... ........A. ....G.....
UTI89         .A.....T.. ...T...... .......... .......... .......... .....C.... ....CGC...
F11           .......... .......... .......... .......... .......... ........A. ....G.....
ED1a          .......... .......... .......... .......... .......... ........A. ....G.....
83972         .......... .......... .......... .......... .......... ........A. ....G.....
S88           .A.....T.. ...T...... .......... .......... .......... .....C.... ....CGC...
E._albertii   .A.....T.. ...T...... .......... .C.....C.. A......... ..T..C.... ....CGC...
```

```
         12601
K-12          TTGATGCGGC TGGGGTTCAT CGTTTCGCCA GTGCTGATGT TCACGCAGTA ACGCTTCAAG CGCCTGCAAC
53638         .......... .......... .......... .......... .......... .......... ..........
ATCC_8739     .......... .......... .......... .......... .......... .......... ..T.......
HS            .......... .......... .......... .......... .......... .......... ..T.......
BW2952        .......... .......... .......... .......... .......... .......... ..........
12009         .......... .......... .......... .......... .......... .......... ..........
B171          .......... .......... .......... .......... .......... .......... ..........
E24377A       .......... .......... .......... .......... .......... .......... ..........
SE11          .......... .......... .......... .......... .......... .......... ..........
11128         .......... .......... .......... .......... .......... .......... ..........
11368         .......... .......... .......... .......... .......... .......... ..........
IAI1          .......... .......... .......... .......... .......... .......... ..........
E110019       .......... .......... .......... .......... .......... .......... ..........
B7A           .......... .......... .......... .......... .......... .......... ..........
CB9615        .......... .......... .......... .......... .......... .......... ..........
O157_Sakai    .......... .......... .......... .......... .......... .......... ..........
IAI39         .......... .......... .......... .......... .......... .......... ..........
UMN026        .......... .......... .......... .......... .......... .......... ..........
SMS-3-5       .......... .......... .......... .......... .......... .......... ..........
536           .......... A......... .......... .......... .......... .......... ..........
APEC_O1       .......... A......... .......... .......... .......... .......... ..........
CFT073        .......... A......... .......... .......... .......... .......... ..........
E2348/69      .......... A......... .......... .......... .......... .......... ..........
UTI89         .......... A......... .......... .......... .......... .......... ..........
F11           .......... A......... .......... .......... .......... .......... ..........
ED1a          .......... A......... .......... .......... .......... .......... ..........
83972         .......... A......... .......... .......... .......... .......... ..........
S88           .......... A......... .......... .......... .......... .......... ..........
E._albertii   .......... .......... ..C.CT.... .......... .....T.A.. G......... ..........

         12671
K-12          TGGAGACGAA CGCGGTCATG AGTGGTCATA AAGCAACCTC AATAAGAAAA A-CTGCCGCG AAGGATAGCA
53638         .......... .......... .......... .......... .......... .......... ..........
ATCC_8739     .......... .......... .......... .......... .......... .......... ..........
HS            .......... .......... .......... .......... .......... .......... ..........
BW2952        .......... .......... .......... .......... .......... .......... ..........
12009         .......... .......... .......... .......... .......... .......... ..........
B171          .......... .......... .......... .......... .......... .......... ..........
E24377A       .......... .......... .......... .......... .......... .......... ..........
SE11          .......... .......... .......... .......... .......... .......... ..........
11128         .......... .......... .......... .......... .......... .......... ..........
11368         .......... .......... .......... .......... .......... .......... ..........
IAI1          .......... .......... .......... .......... .......... .......... ..........
E110019       .......... .......... .......... .......... .......... .......... ..........
B7A           .......... .......... .......... .......... .......... .......... ..........
CB9615        .......... .......... .......... .......... .......... .......... ..........
O157_Sakai    .......... .......... .......... .......... .......... .......... ..........
IAI39         .......... .......... .......... .......... .......... .......... ..........
UMN026        .......... .......... .......... .......... .......... .......... ...A......
SMS-3-5       ....A..... .......... .......... .......... .......... .......... ..........
536           .......... .......... .......... .......... .......... .......... ...A......
APEC_O1       .......... .......... .......... .......... .......... .......... ...A......
CFT073        .......... .......... .......... .......... .......... .......... ...A......
E2348/69      .......... .......... .......... .......... .......... .......... ...A......
UTI89         .......... .......... .......... .......... .......... .......... ...A......
F11           .......... .......... .......... .......... .......... .......... ...A......
ED1a          .......... .......... .......... .......... .......... .......... ...A......
83972         .......... .......... .......... .......... .......... .......... ...A......
S88           .......... .......... .......... .......... .......... .......... ...A......
E._albertii   .......... .......... .......... .......... ..G....... .A........ ..........
```

```
          12741
K-12          GGAATAAAAA AAGGGAGCAC TGTATTCACA GCGCTCCCGG TTCGTTTCGC AGCATTCCAG CTACTTTTGT
53638         .......... .......... .......... .......... .......... .......... ..........
ATCC_8739     .......... .......... .......... .......... .......... .......... ..........
HS            .......... .......... .......... .......... .......... .......... ..........
BW2952        .......... .......... .......... .......... .......... .......... ..........
12009         .......... .......... .......... .......... .......... .......... ..........
B171          .......... ....T..... .......... .......... .......... .......... ..........
E24377A       .......... .......... .......... .......... .......... .......... ..........
SE11          .......... .......... .......... .......... .......... .......... ..........
11128         .......... .......... .......... .......... .......... .......... ..........
11368         .......... .......... .......... .......... .......... .......... ..........
IAI1          .......... .......... .......... .......... .......... .......... ..........
E110019       .......... .......... .......... .......... .......... .......... ..........
B7A           .......... .......... .......... .......... .......... .......... ..........
CB9615        .......... .......... .......... .T........ .......... .......... ..........
O157_Sakai    .......... .......... .......... .T........ .......... .......... ..........
IAI39         .......... .......... .......... .T........ .......... .......... ..........
UMN026        .......... .......... .......... .T........ .......... .......... ..........
SMS-3-5       .......... .......... .......... .T........ .......... .......... ..........
536           .......... .......... .......... .T........ .......... ........G. ........A.
APEC_O1       .......... .......... .......... .T........ .......... ........G. ..........
CFT073        .......... .......... .......... .T........ .......... ........G. ..........
E2348/69      .......... .......... .......... .T........ .......... ........G. ........A.
UTI89         .......... .......... .......... .T........ .......... ........G. ..........
F11           .......... .......... .......... .T........ .......... ........G. ........A.
ED1a          .......... .......... .......... .T........ .......... ........G. ..........
83972         .......... .......... .......... .T........ .......... ........G. ..........
S88           .......... .......... .......... .T........ .......... ........G. ..........
E._albertii   .......... .......... .......... .T........ .......... .......... ..........

          12811
K-12          TGCTCCCTGC TCATCCTTGA CAACTTTTCC TCTGGCCTTG CGGCCAATCG TTCATCCTGA ACTATTGCTT
53638         .......... .......... .......... .......... .......... .......... ..........
ATCC_8739     .......... .......... .......... .......... .......... .......... ..........
HS            .......... .......... .......... .......... .......... .......... ..........
BW2952        .......... .......... .......... .......... .......... .......... ..........
12009         .......... .......... .......... .......... .......... .......... ..........
B171          .......... .......... .......... .......... .......... .......... ..........
E24377A       .......... .......... .......... .......... .......... .......... ..........
SE11          .......... .......... .......... .......... .......... .......... ..........
11128         .......... .......... .......... .......... .......... .......... ..........
11368         .......... .......... .......... .......... .......... .......... ..........
IAI1          .......... .......... .......... .......... .......... .......... ..........
E110019       .......... .......... .......... .......... .......... .......... ..........
B7A           .......... .......... .......... .......... .......... .......... ..........
CB9615        .......... .......... .......... .......A.. .......... .......... ..........
O157_Sakai    .......... .......... .......... .......A.. .......... .......... ..........
IAI39         .......... .......... .......... .......... .......... .......... ..........
UMN026        .......... .......... .......... .......... .......... .......... ..........
SMS-3-5       .......... .......... .......... .......... ..A....... .......... ..........
536           .......... .......... .......... .......... .......... .......... ..........
APEC_O1       .......... .......... .......... .......... .......... .......... ..........
CFT073        .......... .......... .......... .......... .......... .......... ..........
E2348/69      .......... .......... .......... .........A .......... .......... ..........
UTI89         .......... .......... .......... .......... .......... .......... ..........
F11           .......... .......... .......... .......... .......... .......... ..........
ED1a          .......... .......... .......... .......... .......... .......... ..........
83972         .......... .......... .......... .......... .......... .......... ..........
S88           .......... .......... .......... .......... .......... .......... ..........
E._albertii   .......... .......... .......... .......... .......... ...T...... ..........
```

```
            12881
K-12         CCTGCTCACA CCACCCCGAT GTGAT-ACTT CATCCTGAAG TGTCCCTGGC CTTCCTGACC CACCGAATCA
53638        .......... .......... .......... .......... .......... .......... ..........
ATCC_8739    .......... .......... .......... .......... .......... .......... ..........
HS           .......... .......... .......... .......... .......... .......... ..........
BW2952       .......... .......... .......... .......... .......... .......... ..........
12009        .......... .......... .......... .......... .......... .......... ..........
B171         .......... .......... .......... .......... .......... .......... ..........
E24377A      .......... .......... .......... .......... .......... .......... ..........
SE11         .......... .......... .......... .......... .......... .......... ..........
11128        .......... .......... .......... .......... .......... .......... ..........
11368        .......... .......... .......... .......... .......... .......... ..........
IAI1         .......... .......... .......... .......... .......... .......... ..........
E110019      .......... .......... .......... .......... .......... .......... ..........
B7A          .......... .......... .......... .......... .......... .......... ..........
CB9615       .......... .......... .......... .......... ......A... .......... ..........
O157_Sakai   .......... .......... .......... .......... ......A... .......... ..........
IAI39        .......... .......... .......... .......... .......... .......... ..........
UMN026       .......... .......... .......... .......... .......... .......... ..........
SMS-3-5      .......... .......... .......... .......... ...T...... .......... ..........
536          .......... .......... .......... .......... ...T...... .......... ..........
APEC_O1      .......... .......... .......... .......... ...T...... .......... ..........
CFT073       .......... .......... .......... .......... ...T...... .......... ..........
E2348/69     .......... .......... .......... .......... ...T...... .......... ..........
UTI89        .......... .......... .......... .......... ...T...... .......... ..........
F11          .......... .......... .......... .......... ...T...... .......... ..........
ED1a         ..C....... .......... .......... .......... ...T...... .......... ..........
83972        .......... .......... .......... .......... ...T...... .......... ..........
S88          .......... .......... .......... .......... ...T...... .......... ..........
E._albertii  .....A.... ........GC .....T.... .......... C......... .......... .G..A.....

            12951
K-12         TCCTGACCGG TTCTCATTCT CCATCCTGGA GGTGTCCTTT AACGCGTCCT GCGTCATCCT CTTCGCTTCA
53638        .......... .......... .......... .......... .......... .......... ..........
ATCC_8739    .......... .......... .......... .......... .......... .......... ..........
HS           .......... .......... .......... .......... .......... .......... ..........
BW2952       .......... .......... .......... .......... .......... .......... ..........
12009        .......... .......... .......... .......... .......... .......... ..........
B171         .......... .......... .......... .......... .......... .......... ..........
E24377A      .......... .......... .......... .......... .......... .......... ..........
SE11         .......... .......... .......... .......... .......... .......... ..........
11128        .......... .......... .......... .......... .......... .......... ..........
11368        .......... .......... .......... .......... .......... .......... ..........
IAI1         .......... .......... .......... .......... .......... .......... ..........
E110019      .......... .......... .......... .......... .......... .......... ..........
B7A          .......... .......... .......... .......... .......... .......... ..........
CB9615       .......... .......... .......... .......... .......... .......... ..........
O157_Sakai   .......... .......... .......... .......... .......... .......... ..........
IAI39        .......... .......... .......... .......... .......... .......... ..........
UMN026       .......... .......... .......... .......... .......... .......... ..........
SMS-3-5      .......... .......... .......... .......... .......... .......... ..........
536          .......... .......... .......... .......... .......... .......... ..........
APEC_O1      .......... .......... .......... .......... .......... .......... ..........
CFT073       .......... .......... .......... .......... .......... .......... ..........
E2348/69     .......... .......... .......... .......... .......... .......... ..........
UTI89        .......... .......... .......... .......... .......... .......... ..........
F11          .......... .......... .......... .......... .......... .......... ..........
ED1a         .......... .......... .......... .......... ....T..... .......... ..........
83972        .......... .......... .......... .......... .......... .......... ..........
S88          .......... .......... .......... .......... .......... .......... ..........
E._albertii  .......T.. C......... .......... .......... .....A...A ....T..... ..........
```

```
         13021
K-12         TCCAGAAGCC TTTCCCTGAA ACACCATCCT GGTGTGTCCT GCAGAAGTGT CATCATCCTG ATGTTCACTT
53638        .......... .......... .......... .......... .......... .......... ..........
ATCC_8739    .......... .......... .......... ....C..... .......... .......... ..........
HS           .......... .......... .......... ....C..... .......... .......... ..........
BW2952       .......... .......... .......... .......... .......... .......... ..........
12009        .......... .......... .......... .......... .......... .......... ..........
B171         .......... .......... .......... .......... .......... .......... ..........
E24377A      .......... .......... .......... .......... .......... .......... ..........
SE11         .......... .......... .......... .......... .......... .......... ..........
11128        .......... .......... .......... .......... .......... .......... ..........
11368        .......... .......... .......... .......... .......... .......... ..........
IAI1         .......... .......... .......... .......... .......... .......... ..........
E110019      .......... .......... .......... .......... .......... .......... ..........
B7A          .......... .......... .......... .......... .......... .......... ..........
CB9615       .......... .......... .......... .......... T......... .......... ..........
O157_Sakai   .......... .......... .......... .......... T......... .......... ..........
IAI39        .......... .......... .......... .......... T......... .......... ..........
UMN026       .......... .......... .......... .......... T......... .......... ..........
SMS-3-5      .......... .......... .......... .......... T......... .......... ..........
536          .......... .......... .......... .......... T......... .......... ..........
APEC_O1      .......... .......... .......... .......... T......... .......... ..........
CFT073       .......... .......... .......... .......... T......... .......... ..........
E2348/69     .......... .......... .......... .......... T......... .......... ..........
UTI89        .......... .......... .......... .......... T......... .......... ..........
F11          .......... .......... .......... .......... T......... .......... ..........
ED1a         .......... .......... .......... .......... T......... .......... ..........
83972        .......... .......... .......... .......... T......... .......... ..........
S88          .......... .......... .......... .......... T......... .......... ..........
E._albertii  .......... .......... .......... ..C....... T......... .......... ..........

         13091
K-12         CGTTGTCTGA CTCCCTGTCG ACGAAGATAG AATCGTCTTT TTCGCGAAGT CTTACAAGGC GCTTACAGAC
53638        .......... .......... .......... .......... .......... .......... ..........
ATCC_8739    .......... .......... .......... .......... .......... .......... ..........
HS           .......... .......... .......... .......... .......... .......... ..........
BW2952       .......... .......... .......... .......... .......... .......... ..........
12009        .......... .......... .......... .......... .......... .......... ..........
B171         .......... .......... .........A .......... .......... .......... ..........
E24377A      .......... .......... .......... .......... .......... .......... ..........
SE11         .......... .......... .......... .......... .......... .......... ..........
11128        .......... .......... .......... .......... .......... .......... ..........
11368        .......... .......... .......... .......... .......... .......... ..........
IAI1         .......... .......... .......... .......... .......... .......... ..........
E110019      .......... .......... .......... .......... .......... .......... ..........
B7A          .......... .......... .......... .......... .......... .......... ..........
CB9615       .......... .......... .......... .......... ........C. .......... ..........
O157_Sakai   .......... .......... .......... .......... ........C. .......... ..........
IAI39        .......... .......... .......... .......... .......... .......... ..........
UMN026       .......... .......... .......... .......... .......... .......... ..........
SMS-3-5      .......... .......... .......... .......... .......... .......... ..........
536          .......... .......... .......... .......... ...T...... .......... ..........
APEC_O1      .......... .......... .......... .......... ...T...... .......... ..........
CFT073       .......... .......... .......... .......... ...T...... .......... ..........
E2348/69     .......... .......... .......... .......... ...T...... .......... ..........
UTI89        .......... .......... .......... .......... ...T...... .......... ..........
F11          .......... .......... .......... .......... ...T...... .......... ..........
ED1a         .......... .......... .......... .......... ...T...... .......... ..........
83972        .......... .......... .......... .......... ...T...... .......... ..........
S88          .......... .......... .......... .......... ...T...... .......... ..........
E._albertii  ..C....... .......... .......... .......... ..A..CG... .......... ..........
```

```
         13161
K-12          AATGCCTTAA GGAAAATTTC ATATGAAAGT TAAGAAT-TA ACAATCAACA TTTTGATTTT AAATGAAAAT
53638         .......... .......... .......... .......... ....GT.... .G...T.... ..........
ATCC_8739     .......... .......... .......... .......... ....GT.... .G...T.... ..........
HS            .......... .......... .......... .......... ....GT.... .G...T.... ..........
BW2952        .......... .......... .......... .......... .......... .......... ..........
12009         .......... .......... .......... .......... .......... .......... ..........
B171          .......... .......... .......... .......... .......... .......... ..........
E24377A       .......... .......... .......... .......... .......... .......... ..........
SE11          .......... .......... .......... .......... .......... .......... ..........
11128         .......... .......... .......... .......... .......... .......... ..........
11368         .......... .......... .......... .......... .......... .......... ..........
IAI1          .......... .......... .......... .......... .......... .......... ..........
E110019       .......... .......... .......... .......... .......... .......... ..........
B7A           .......... .......... .......... .......... .......... .......... ..........
CB9615        .......... .......... .......... ..G....... ....G..... .G...T.... ..........
O157_Sakai    .......... .......... .......... ..G....... ....G..... .G...T.... ..........
IAI39         .......... .......... .......... ........G  ....A..T.  .A........ ..........
UMN026        .......... .......... .......... ....G....  ....G..T.  .A........ ..........
SMS-3-5       .......... .......... .......... ........G  ....G..T.  .A........ ..........
536           .......... .......... .......... .......... ....G..... .G...T.... ......G...
APEC_O1       .......... ...T...... .......... .......... ....G..... .....T.... ..........
CFT073        .......... .......... .......... .......... ....G..... .G...T.... ......G...
E2348/69      .......... .......... .......... .......... ....G..... .G...T.... ......G...
UTI89         .......... ...T...... .......... .......... ....G..... .....T.... ..........
F11           .......... .......... .......... .......... ....G..... .G...T.... ......G...
ED1a          .......... .......... .......... .......... ....G..... .G...T.... ......G...
83972         .......... .......... .......... .......... ....G..... .G...T.... ......G...
S88           .......... ...T...... .......... .......... ....G..... .....T.... ..........
E._albertii   ...ATG.... .....GC... ....A...A. ...A..AA.. G..--T..T. AA.......G ....A.....

         13231
K-12          AACGATTCTT ATCTTTTTAA GTATTCGCTT ATTTCCTGGC GATCTCCTAC AGAGTCTGTA AGAGATCTCT
53638         .......... .......... .......... .......... .......... ..G....... ..........
ATCC_8739     .......... .......... .......... .......... .......... ..G....... ..........
HS            .......... .......... .......... .......... .......... ..G....... ..........
BW2952        .......... .......... .......... .......... .......... .......... ..........
12009         .......... .......... .......... .......... .......... .......... ..........
B171          .......... .......... .......... .......... .......... .......... ..........
E24377A       .......... .......... .......... .......... .......... .......... ..........
SE11          .......... .......... .......... .......... .......... .......... ..........
11128         .......... .......... .......... .......... .......... .......... ..........
11368         .......... .......... .......... .......... .......... .......... ..........
IAI1          .......... .......... .......... .......... .......... .......... ..........
E110019       .......... .......... .......... .......... .......... .......... ..........
B7A           .......... .......... .......... .......... .......... .......... ..........
CB9615        .......... .......... .......... .......... .......... .......... ..........
O157_Sakai    .......... .......... .......... .......... .......... .......... ..........
IAI39         .......... .......... .......... .......... .......... .......... ..........
UMN026        .......... .......... .......... .......... .......... .......... ..........
SMS-3-5       .......... .......... .......... .......... .......... .......... ..........
536           .......... .......... .......... .......... .......... .......... ..........
APEC_O1       .......... .......... .......... .......... .......... .......... ..........
CFT073        .......... .......... .......... .......... .......... .......... ..........
E2348/69      .......... .......... .......... .......... .......... .......... ..........
UTI89         .......... .......... .......... .......... .......... .......... ..........
F11           .......... .......... .......... .......... .......... .......... ..........
ED1a          .......... .......... .......... .......... .......... .......... ..........
83972         .......... .......... .......... .......... .......... .......... ..........
S88           .......... .......... .......... .......... .......... .......... ..........
E._albertii   .T.A...... .......... .....TA... .......... .......... ..G....... ..........
```

```
         13301
K-12          CACAACCAGA TGGGGTATTT GGATTACAGA AGAGGCTTAA GTTGATTAAG GAATTCCGCA AGATTTGAGG
53638         .......... .......... .......... .......... .......... .......... ..........
ATCC_8739     .......... .......... .......... .......... .......... .......... ..........
HS            .......... .......... .......... .......... .......... .......... ..........
BW2952        .......... .......... .......... .......... .......... .......... ..........
12009         .......... .......... .......... .......... .......... .......... ..........
B171          .......... .......... .......... .......... .......... .......... ..........
E24377A       .......... .......... .......... .......... .......... .......... ..........
SE11          .......... .......... ......A... .......... .......... .......... ..........
11128         .......... .......... .......... .......... .......... .......... ..........
11368         .......... .......... .......... .......... .......... .......... ..........
IAI1          .......... .......... .......... .......... .......... .......... ..........
E110019       .......... .......... .......... .......... .......... .......... ..........
B7A           .......... .......... .......... .......... .......... .......... ..........
CB9615        .......... .......... .......... .......... .......... .......... .......G..
O157_Sakai    .......... .......... .......... .......... .......... .......... .......G..
IAI39         ........A. .......... .......... .......... .......... .......... ..........
UMN026        ........A. .......... .......... .......... .......... .......... ..........
SMS-3-5       .......... .......... .......... .......... .......... .......... ..........
536           .......... .......... .......... .......... .......... .......... ..........
APEC_O1       .......... .......... .......... .......... .......... .......... ..........
CFT073        .......... .......... .......... .......... .......... .......... ..........
E2348/69      .......... .......... .......... .......... .......... .......... ..........
UTI89         .......... .......... .......... .......... .......... .......... ..........
F11           .......... .......... .......... .......... .......... .......... ..........
ED1a          .......... .......... .......... .......... .......... .......... ..........
83972         .......... .......... .......... .......... .......... .......... ..........
S88           .......... .......... .......... .......... .......... .......... ..........
E._albertii   .......... C......... .......... .......... ....G..... .......... ..........

         13371
K-12          CTAAATGCGT CCGTTTACGT GTTCCCAGCG TCTCTTTGCA CACTTCACCG GAGAGATTAC ACACCGAGAT
53638         .......... .......... .......... .......... .......... .......... ..........
ATCC_8739     .......... .......... .......... .......... .......... .......... ..........
HS            .......... .......... .......... .......... .......... .......... ..........
BW2952        .......... .......... .......... .......... .......... .......... ..........
12009         .......... .......... .......... .......... .......... .......... ..........
B171          .......... .......... .......... .......... .......... .......... ..........
E24377A       .......... .......... .......... .......... .......... .......... ..........
SE11          .......... .......... .......... .......... .......... .......... ..........
11128         .......... .......... .......... .......... .......... .......... ..........
11368         .......... .......... .......... .......... .......... .......... ..........
IAI1          .......... .......... .......... .......... .......... .......... ..........
E110019       .......... .......... .......... .......... .......... .......... ..........
B7A           .......... .......... .......... .......... .......... .......... ..........
CB9615        .......... .......... .......... .......A.. .......... .......... ..........
O157_Sakai    .......... .......... .......... .......A.. .......... .......... ..........
IAI39         .......... .......... .......... .......... .......... .......... ..........
UMN026        .........C .......... .......... .......A.. .......... .......... ..........
SMS-3-5       .......... .......... .......... .......... .......... .......... ..........
536           .......... .......... .......... .......... .......... .......... ..........
APEC_O1       .......... .......... .......... .......... .......... .......... ..........
CFT073        .......... .......... .....T.... .......... .......... .......... ..........
E2348/69      .......... .......... .......... .......... .......... .......... ..........
UTI89         .......... .......... .......A.. .......... .......... .......... ..........
F11           .......... .......... .......... .......... .......... .......... ..........
ED1a          .......... .......... .......... .......... .......... .......... ..........
83972         .......... .......... .....T.... .......... .......... .......... ..........
S88           .......... .......... .......... .......... .......... .......... ..........
E._albertii   .C.G.G.... ......C..C .......A.. .......... ...A..G..C .T......G. ....G.....
```

```
         13441
K-12         GACCTCTAAC TCTTCTTCCA GCGTCGCGAT AAATAGTGTG GGCGGCAGTT TTAGCCTTTT CTGAGTCACC
53638        .......... .......... .......... ......C... .......... .......... ..........
ATCC_8739    .......... .......... .......... ......C... .......... .......... ..........
HS           .......... .......... .......... ......C... .......... .......... ..........
BW2952       .......... .......... .......... .......... .......... .......... ..........
12009        .......... .......... .......... ......C... .......... .......... ..........
B171         .......... .......... .......... ......C... .......... .......... ..........
E24377A      .......... .......... .......... ......C... .......... .......... ..........
SE11         .......... .......... .......... ......C... .......... .......... ..........
11128        .......... .......... .......... ......C... .......... .......... ..........
11368        .......... .......... .......... ......C... .......... .......... ..........
IAI1         .......... .......... .......... ......C... .......... .......... ..........
E110019      .......... .......... .......... ......C... .......... .......... ..........
B7A          .......... .......... .......... ......C... .......... .......... ..........
CB9615       .......... .......... .......... ......C... ..T....... .......... ..........
O157_Sakai   .......... .......... .......... ......C... ..T....... .......... ..........
IAI39        .......... .......... .......... ......C... .......... .......... ..........
UMN026       C......... .......... .......... ......C... .......A.. .......... ..........
SMS-3-5      C......... .......... .......... ......C... .......... .......... ..........
536          .......... .......... .......... ......C... .......... .......... ..........
APEC_O1      .......... .......... .......... ......C... .......... .......... ..........
CFT073       .......... .......... .......... ......C... .......... .......... ..........
E2348/69     .......... .......... .......... ......C... .......... .......... ..........
UTI89        .......... .......... .......... ......C... .......... .......... ..........
F11          .......... .......... .......... ......C... .......... .......... ..........
ED1a         .......... .......... .......... ......C... .......... .......... ..........
83972        .......... .......... .......... ......C... .......... .......... ..........
S88          .......... .......... .......... ......C... .......... .......... ..........
E._albertii  C.....C.GT .......... ....G..A.. ...G.....C ..T.A...C. .A..G.G... ......G...

         13511
K-12         AGATGACCAA TCAGATTTTC CTGCACCCGA CGGAAGTCGT CTTCACTCCA GGTTTGCAGC AATGTCAGTT
53638        .......... .......... .......... .......... .......... .......... ..........
ATCC_8739    .......... .......... .......... .......... .......... .......... ..........
HS           .......... .......... .......... .......... .......... .......... ..........
BW2952       .......... .......... .......... .......... .......... .......... ..........
12009        .......... .......... .......... .......... .......... .......... ..........
B171         .......... .......... .......... .......... .......... .......... ..........
E24377A      .......... .......... .......... .......... .......... .......... ..........
SE11         .......... .......... .......... .......... .......... .......... ..........
11128        .......... .......... .......... .......... .......... .......... ..........
11368        .......... .......... .......... .......... .......... .......... ..........
IAI1         .......... .......... .......... .......... .......... .......... ..........
E110019      .......... .......... .......... .......... .......... .......... ..........
B7A          .......... .......... .......... .......... .......... .......... ..........
CB9615       .......... .......... .......... .......... .......... .......... ..........
O157_Sakai   .......... .......... .......... .......... .......... .......... ..........
IAI39        .......... .......... .......... .....A.... .......... .......... ..........
UMN026       .......... .......... .......... .......... .......... .......... ..........
SMS-3-5      .......... .......... .......... .......... .......... .......... ..........
536          .......... .......... .......... .......... .......... .......... ..........
APEC_O1      .......... .......... .......... .......... .......... .......... ..........
CFT073       .......... .......... .......... .......... .......... .......... ..........
E2348/69     .......... .......... .......... .......... .......... .......... ..........
UTI89        .......... .......... .......... .......... .......... .......... ..........
F11          .......... .......... .......... .......... .......... .......... ..........
ED1a         .......... .......... .......... .......... .......... .......... ..........
83972        .......... .......... .......... .......... .......... .......... ..........
S88          .......... .......... .......... .......... .......... .......... ..........
E._albertii  .....T.... .......C.. .........G .....A.... .C..G..... .......... ........AG
```

```
            13581
K-12         TGATATCGCC AAACTGCGCG TGCATATCCC CGGCAAACTG AGTCGTATAA AAGGTATGAA TTGTGGGTTG
53638        .......... .......... .......... .......... .......... .......... ..........
ATCC_8739    .......... .......... .......... .......... .......... .......... ..........
HS           .......... .......... .......... .......... .......... .......... ..........
BW2952       .......... .......... .......... .......... .......... .......... ..........
12009        .......... .......... .......... .......... .......... .......... ..........
B171         .......... .......... .......... .......... .......... .......... ..........
E24377A      .......... .......... .......... .......... .......... .......... ..........
SE11         .......... .......... .......... .......... .......... .......... ..........
11128        .......... .......... .......... .......... .......... .......... ..........
11368        .......... .......... .......... .......... .......... .......... ..........
IAI1         .......... .......... .......... .......... .......... .......... ..........
E110019      .......... .......... .......... .......... .......... .......... ..........
B7A          .......... .......... .......... .......... .......... .......... ..........
CB9615       .......... .......... .......... .......... .......... .......... ..........
O157_Sakai   .......... .......... .......... .......... .......... .......... ..........
IAI39        .......... .......... .......... .......... .......... .......... .C........
UMN026       .A........ .......... .......... .......... .......... .......... ..........
SMS-3-5      .......... .......... .......... .......... .......... .......... ..........
536          .......... .......... .......... .......... .......... .......... .C........
APEC_O1      .......... .......... .......... .......... .......... .......... .C........
CFT073       .......... .......... .......... .......... .......... .......... .C........
E2348/69     .......... .......... .......... .......... .......... .......... .C........
UTI89        .......... .......... .......... .......... .......... .......... .C........
F11          .......... .......... .......... .......... .......... .......... .C........
ED1a         .......... .......... .......... .......... .......... .......... .C........
83972        .......... .......... .......... .......... .......... .......... .C........
S88          .......... .......... .......... .......... .......... .......... .C........
E._albertii  G......A.. .........A .......... .......... G......... ....A..... ....A..C..

            13651
K-12         TATCACAATA TCAAAAGCGC GTTCAACCGC GTTTACATTT TGTTCCCCGG TAAACGGCTG GGGTTGCCAG
53638        .......... .......... .......... .......... .......... .......... ..........
ATCC_8739    .......... .......... .......... .......... .......... .......... ..........
HS           .......... .......... .......... .......... .......... .......... ..........
BW2952       .......... .......... .......... .......... .......... .......... ..........
12009        .......... .......... .......... .......... .......... .......... ..........
B171         .......... .......... .......... .......... .......... .......... ..........
E24377A      .......... .......... .......... .......... .......... .......... ..........
SE11         .......... .......... .......... .......... .......... .......... ..........
11128        .......... .......... .......... .......... .......... .......... ..........
11368        .......... .......... .......... .......... .......... .......... ..........
IAI1         .......... .......... .......... .......... .......... .......... ..........
E110019      .......... .......... .......... .......... .......... .......... ..........
B7A          .......... .......... .......... .......... .......... .......... ..........
CB9615       .......... .......... .......... .......... .......... .......... ..........
O157_Sakai   .......... .......... .......... .......... .......... .......... ..........
IAI39        .......... .......... .......... .......... .......... .......... ..........
UMN026       .......... .......... .......... .......... .......... .......... ..........
SMS-3-5      .......... ........A. .......... .......... .......... .......... ..........
536          .......... .......... .......... .......... .......... .......... ..........
APEC_O1      .......... .......... .......... .......... .......... .......... ..........
CFT073       .......... .......... .......... .......... .......... .......... ..........
E2348/69     .......... .......... .......... .......... .......... .......... ..........
UTI89        .......... .......... .......... .......... .......... .......... ..........
F11          .......... .......... .......... .......... .......... .......... ..........
ED1a         .......... .......... .......... .......... .......... .......... ..........
83972        .......... .......... .......... .......... .......... .......... ..........
S88          .......... .......... .......... .......... .......... .......... ..........
E._albertii  .......... ........T. .......... ...C...... ..C..T.... .......... ..........
```

```
         13721
K-12          TATACGGCAT CTTCGGTAGT TGAAATAATG CACGGTGAAG GAACACCGTA CAGTTCCTCA CTTAACGGCC
53638         .......... .......... .......... .......... .......... .......... ..........
ATCC_8739     .......... .......... .......... .......... .......... .......... ..........
HS            .......... .......... .......... .......... .......... .......... ..........
BW2952        .......... .......... .......... .......... .......... .......... ..........
12009         .......... .......... .......... .......... .......... .......... ..........
B171          .......... .......... .......... .......... .......... .......... ..........
E24377A       .......... .......... .......... .......... .......... .......... ..........
SE11          .......... .......... .......... .......... .......... .......... ..........
11128         .......... .......... .......... .......... .......... .......... ..........
11368         .......... .......... .......... .......... .......... .......... ..........
IAI1          .......... .......... .......... .......... .......... .......... ..........
E110019       .......... .......... .......... .......... .......... .......... ..........
B7A           .......... .......... .......... .......... .......... .......... ..........
CB9615        .......... ....A..... .......... .......... ....G..... .......... ..........
O157_Sakai    .......... ....A..... .......... .......... ....G..... .......... ..........
IAI39         ........G. .......G.. .......... .......... ....G..... .......... ..C.......
UMN026        ........G. .......G.. .......... .......... ....G..... .......... ..C.......
SMS-3-5       .......... .......... .......... ..........            .......... ..C.......
536           .......... .......... .......... .......... ....G..... .......... ..........
APEC_O1       .......... .......... .......... .......... ....G..... .......... ..........
CFT073        .......... .......... .......... .......... ....G..... .......... ..........
E2348/69      .......... .......... .......... .......... ....G..... .......... ..........
UTI89         .......... .......... .......... .......... ....G..... .......... ..........
F11           .......... .......... .......... .......... ....G..... .......... ..........
ED1a          ....T..... .......... .......... .......... ....G..... .......... ..........
83972         .......... .......... .......... .......... ....G..... .......... ..........
S88           .......... .......... .......... .......... ....G..... .......... ..........
E._albertii   .......... .G.T...G.. .......... ..T..C..G. ....G..A.. A..C.....G ..C.......

         13791
K-12          AGCTTTTATG CTCTTCATGC CACGCATCGC AGTAGCGTGC AGTAAAATCT TTCAGGGCTT GTGCGGTCAA
53638         .......... .......... .......... .......... .......... .......... ..........
ATCC_8739     .......... .......... .......... .......... .......... .......... ..........
HS            .......... .......... .......... .......... .......... .......... ..........
BW2952        .......... .......... .......... .......... .......... .......... ..........
12009         .......... .......... .......... .......... .......... .......... ..........
B171          .......... .......... .......... .......... .......... .......... ..........
E24377A       .......... .......... .......... .......... .......... .......... ..........
SE11          .......... .......... .......... .......... .......... .......... ..........
11128         .......... .......... .......... .......... .......... .......... ..........
11368         .......... .......... .......... .......... .......... .......... ..........
IAI1          .......... .......... .......... .......... .......... .......... ..........
E110019       .......... .......... .......... .......... .......... .......... ..........
B7A           .......... .......... .......... .......... .......... .......... ..........
CB9615        .......... .......... .......... .......... .......... .......... ..........
O157_Sakai    .......... .......... .......... .......... .......... .......... ..........
IAI39         .......... .......... .......... .......... .......... .......... ..........
UMN026        .......... .......... .......... .......... .......... .......... ..........
SMS-3-5       .......... .......... .......... .......... .......... .......... ..........
536           .......... .......... .......... .......... .......... .......... ..........
APEC_O1       .......... .......... .......... .......... .......... .......... ..........
CFT073        .......... .......... .......... .......... .......... .......... ..........
E2348/69      .......... .......... .......... .......... .......... .......... ..........
UTI89         .......... .......... .......... .......... .......... .......... ..........
F11           .......... .......... .......... .......... .......... .......... ..........
ED1a          .......... .......... .......... .......... .......... .......... ..........
83972         .......... .......... .......... .......... .......... .......... ..........
S88           .......... .......... .......... .......... .......... .......... ..........
E._albertii   .A..G..G.. ..G....... ..G....... ....A..... G......... ....A..... ..........
```

```
         13861
K-12         ATCGTCCACT AATTTCTCTC TTCACTTAAA CCAGATACAC TTGTCGTTTA GTTTATCTGG TTTATGACGG
53638        .......... .......... .......... .......... .......... .......... ..........
ATCC_8739    .......... .......... .......... .......... .......... .......... ..........
HS           .......... .......... .......... .......... .......... .......... ..........
BW2952       .......... .......... .......... .......... .......... .......... ..........
12009        .......... .......... .......... .......... .......... .......... ..........
B171         .......... .......... .......... .......... .......... .......... ..........
E24377A      .......... .......... .......... .......... .......... .......... ..........
SE11         .......... .......... .......... .......... .......... .......... ..........
11128        .......... .......... .......... .......... .......... .......... ..........
11368        .......... .......... .......... .......... .......... .......... ..........
IAI1         .......... .......... .......... .......... .......... .......... ..........
E110019      .......... .......... .......... .......... .......... .......... ..........
B7A          .......... .......... .......... .......... .......... .......... ..........
CB9615       .......... .......... .......... .......... .......... .......... ..........
O157_Sakai   .......... .......... .......... .......... .......... .......... ..........
IAI39        .......... .......... .......... .......... .......... .......... ..........
UMN026       .......... .......... .......... .......... .......... .......... ..........
SMS-3-5      .......... .......... .......... .......... .......... .......... ..........
536          .......... .......... .......... .......... .......... .......... ..........
APEC_O1      .......... .......... .......... .......... .......... .......... ..........
CFT073       .......... .......... .......... .......... .......... .......... ..........
E2348/69     .......... .......... .......... .......... .......... .......... ..........
UTI89        .......... .......... .......... .......... .......... .......... ..........
F11          .......... .......... .......... .......... .......... .......... ..........
ED1a         .......... .......... .......... .......... .......... .......... ..........
83972        .......... .......... .......... .......... .......... .......... ..........
S88          .......... .......... .......... .......... .......... .......... ..........
E._albertii  T......... .......... .......... ..G....... ...C...... .......... ..........

         13931
K-12         TGAAACATGT CTTCTTATGC AAACCATCAG GCACTTGCGG GCCTGACTCT TGGAAAATCA ACCGATTACC
53638        .......... .......... .......... .......... .......... .......... ..........
ATCC_8739    .......... .......... .......... .......... .......... .......... ..........
HS           .......... .......... .......... .......... .......... .......... ..........
BW2952       .......... .......... .......... .......... .......... .......... ..........
12009        .......... .......... .......... .......... .......... .......... ..........
B171         .......... .......... .......... .......... .......... .......... ..........
E24377A      .......... .......... .......... .......... .......... .......... ..........
SE11         .......... .......... .......... .......... .......... .......... ..........
11128        .......... .......... .......... .......... .......... .......... ..........
11368        .......... .......... .......... .......... .......... .......... ..........
IAI1         .......... .......... .......... .......... .......... .......... ..........
E110019      .......... .......... .......... .......... .......... .......... ..........
B7A          .......... .......... .......... .......... .......... .......... ..........
CB9615       .......... .......... .......... .......... .......... .......... ..........
O157_Sakai   .......... .......... .......... .......... .......... .......... ..........
IAI39        .......... .......... .......... .......... .......... .......... ..........
UMN026       .......... .......... .......... .T........ .......... .......... ..........
SMS-3-5      .......... .......... .......... .......... .......... .......... ..........
536          .......... .......... .......... .......... .......A.. .......... ..........
APEC_O1      .......... .......... .......... .......... .......A.. .......... ..........
CFT073       .......... .......... .......... .......... .......... .......... ..........
E2348/69     .......... .......... .......... .......... .......A.. .......... ........T.
UTI89        .......... .......... .......... .......... .......A.. .......... ..........
F11          .......... .......... .......... .......... .......A.. .......... ..........
ED1a         .......... .......... .......... .......... .......A.. .......... ..........
83972        .......... .......... .......... .......... .......... .......... ..........
S88          .......... .......... .......... .......... .......A.. .......... ..........
E._albertii  .......... ........A. .......... .......... .......... C......... ..........
```

```
         14001
K-12         GGGATACCTA TGACGCCAGC CTACTGCAAG GCGTTCCACG CAGCCTGAAT CGCGACCCGC TGGGTCTGAA
53638        .......... .......... .......... .......... .......... .......... ..........
ATCC_8739    .......... .......... .......... .......... .......... .......... ..........
HS           .......... .......... .......... .......... .......... .......... ..........
BW2952       .......... .......... .......... .......... .......... .......... ..........
12009        .......... .......... .......... .......... .......... .......... ..........
B171         .......... .......... .......... .......... .......... .......... ..........
E24377A      .......... .......... .......... .......... .......... .......... ..........
SE11         .......... .......... .......... .......... .......... .......... ..........
11128        .......... ........A. .......... .......... .......... .......... ..........
11368        .......... ........A. .......... .......... .......... .......... ..........
IAI1         .......... .......... .......... .......... .......... .......... ..........
E110019      .......... ........A. .......... .......... .......... .......... ..........
B7A          .......... .......... .......... .......... .......... .......... ..........
CB9615       ....G..... .......... .......... .......... .......... .......... ..........
O157_Sakai   .......... .......... .......... .......... .......... .......... ..........
IAI39        .......... .......... .......... .......G.. T..T...... .....T.... ..........
UMN026       .......... .......... .......... .......G.. T..T...... .....T.... ..........
SMS-3-5      .......... .......... .......... .......G.. T..T...... .....T.... ..........
536          .......... .......... .......... ....A..... .......... .......... ..........
APEC_O1      .......... .......... .......... .......... .......... .......... ..........
CFT073       .......... .......... .......... .......... .......... .......... ..........
E2348/69     .......... .......... .......... ....A..... .......... .......... ..........
UTI89        .......... .......... .......... .......... .......... .......... G.........
F11          .......... .......... .......... ....A..... .......... .......... ..........
ED1a         .......... .......... .......... .......... .......... .......... ..........
83972        .......... .......... .......... .......... .......... .......... ..........
S88          .......... .......... .......... .......... .......... .......... ..........
E._albertii  .......T.. .......... ..G....... .......... ...TT..... .....T.... .A..CT....

         14071
K-12         AGCGGATAAC CTGCCTTTTC ACGGTACGGA TATCTGGACG CTGTATGAAC TTTCCTGGCT GAATGCGAAA
53638        .......... .......... .......... .......... .......... .......... ..........
ATCC_8739    .......... .......... .......... .......... .......... .......... ..........
HS           .......... .......... .......... .......... .......... .......... ..........
BW2952       .......... .......... .......... .......... .......... .......... ..........
12009        .......... .......... .......... .......... .......... .......... ..........
B171         .......... .......... .......... .......... .......... .......... ..........
E24377A      .......... .......... .......... .......... .......... .......... ..........
SE11         .......... .......... .......... .......... .......... .......... ..........
11128        .......... .......... .......... .......... .......... .......... ..........
11368        .......... .......... .......... .......... .......... .......... ..........
IAI1         .......... .......... .......... .......... .......... .......... ..........
E110019      .......... .......... .......... .......... .......... .......... ..........
B7A          .......... .......... .......... .......... .......... .......... ..........
CB9615       .......... .......... .A........ .......... .......... .......... ..........
O157_Sakai   .......... .......... .A........ .......... .......... .......... ..........
IAI39        .......... .......... .......... .......... ..A....... .......... ..........
UMN026       .......... .......... .......... .......... ..A....... .......... ..........
SMS-3-5      .......... .......... .......... .......... ..A....... .......... ..........
536          .......... .......... .......... .......... .......... .......... ..........
APEC_O1      .......... .......... .......... .......... .......... .......... ..........
CFT073       .......... .......... .......... .......... .......... .......... ..........
E2348/69     .......... .......... .......... .......... .......... .......... ..........
UTI89        .......... .......... .......... .......... .......... .......... ..........
F11          .......... .......... .......... .......... .......... .......... ..........
ED1a         .......... .......... .......... .......... .......... .......... ..........
83972        .......... .......... .......... .......... .......... .......... ..........
S88          .......... .......... .......... .......... .......... .......... ..........
E._albertii  .........T .....A.... .....G.... .......... ..A....... .......... ...C......
```

```
         14141
K-12         GGTTTGCCGC AGGTCGCTGT CGGTCATGTT GAACTTGATT ACACCAGCGT AAATCTGATT GAGTCGAAGA
53638        .......... .......... .......... .......... .......... .......... ..........
ATCC_8739    .......... .......... .......... .......... .......... .......... ..........
HS           .......... .......... .......... .......... .......... .......... ..........
BW2952       .......... .......... .......... .......... .......... .......... ..........
12009        .......... .......... .......... .......... .......... .......... ..........
B171         .......... .......... .......... .......... .......... .......... ..........
E24377A      .......... .......... .......... .......... .......... .......... ..........
SE11         .......... .......... .......... .......... .......... .......... ..........
11128        .......... ....T..C.. ......C... .......... .........C .......... ..........
11368        .......... ....T..C.. ......C... .......... .........C .......... ..........
IAI1         .......... .......... .......... .......... .......... .......... ..........
E110019      .......... ....T..C.. ......C... .......... .........C .......... ..........
B7A          .......... .......... .......... .......... .......... .......... ..........
CB9615       .......... .......... .......... .......... .......... .......... ..........
O157_Sakai   .......... .......... .......... .......... .......... .......... ..........
IAI39        .......... .......C.. .......... .......... .........C .......... ..........
UMN026       .......... .......C.. .......... .......... .........C .......... ..........
SMS-3-5      .......... .......C.. .......... .......... .........C .......... ..........
536          .......... .......... .......... .......... .......... .......... ..........
APEC_O1      .......... .......... .......... .......... .......... .......... ..........
CFT073       .......... .......... .......... .......... .......... .......... ..........
E2348/69     .......... .......... .......... .......... .......... .......... ..........
UTI89        .......... .......... .......... .......... .......... .......... ..........
F11          .......... .......... .......... .......... .......... .......... ..........
ED1a         .......... .......... .......... .......... .......... .......... ..........
83972        .......... .......... .......... .......... .......... .......... ..........
S88          .......... .......... .......... ........G. .......... .......... ..........
E._albertii  .......... .......C.. .........C .......... .......... G.....C... ..A.....A.

         14211
K-12         GTTTTAAGCT CTATCTCAAC AGTTTTAACC AGACGCGTTT TAATAACTGG GATGAGGTGC GCCAGACGCT
53638        .......... .......... .......... .......... .......... .......... ..........
ATCC_8739    .......... .......... .......... .......... .......... .......... ..........
HS           .......... .......... .......... .......... .......... .......... ..........
BW2952       .......... .......... .......... .......... .......... .......... ..........
12009        .......A.. .......... .......... .......... .......... .......... ..........
B171         .......... .......... .......... .......... .......... .......... ..........
E24377A      .......... .......... .......... .......... .......... .......... ..........
SE11         .......... .......... .......... .......... .......... .......... ..........
11128        .......... .......... .......... .......... .......... .......... ..........
11368        .......... .......... .......... .......... .......... .......... ..........
IAI1         .......... .......... .......... .......... .......... .......... ..........
E110019      .......... .......... .......... .......... .......... .......... ..........
B7A          .......... .......... .......... .......... .......... .......... ..........
CB9615       .......... .......... .......... .......... .......... .......... ..........
O157_Sakai   .......... .......... .......... .......... .......... .......... ..........
IAI39        .......... .......... .......... .......... .......... .......... ..........
UMN026       .......... .......... .......... .......... .......... .......... ..........
SMS-3-5      .......... .......... .......... .......... .......... .......... ..........
536          .......... .......... .......... .......... .......... .......... ..........
APEC_O1      .......... .......... .......... .......... .......... .......... ..........
CFT073       .......... .......... .......... .......... .......... .......... ..........
E2348/69     .......... .......... .......... .......... .......... .......... ..........
UTI89        .......... .......... .......... .......... .......... .......... ..........
F11          .......... .......... .......... .......... .......... .......... ..........
ED1a         .......... .......... .......... .......... .......... .......... ..........
83972        .......... .......... .......... .......... .......... .......... ..........
S88          .......... .......... .......... .......... .......... .......... ..........
E._albertii  .C..C..A.. G........T ..C....... .......C.. ...C...... .......... ..........
```

```
        14281
K-12         GGAGCGCGAC TTAAGCACTT GCGCTCAGGG TAAGATTAGC GTGGCGTTAT ATCGTCTTGA TGAACTGGAA
53638        .......... .......... .......... .......... .......... .......... ..........
ATCC_8739    .......... .......... .......... .......... .......... .......... ..........
HS           .......... .......... .......... .......... .......... .......... ..........
BW2952       .......... .......... .......... .......... .......... .......... ..........
12009        .......... .......... ....C..... ....G..... .......... .......... ..........
B171         .......... .......... ....C..... ....G..... .......... .......... ..........
E24377A      .......... .......... ....C..... ....G..... .......... .......... ..........
SE11         .......... .......... ....C..... ....G..... .......... .......... ..........
11128        .......... .......... ....C..... ....G..... .......... .......... ..........
11368        .......... .......... ....C..... ....G..... .......... .......... ..........
IAI1         .......... .......... ....C..... ....G..... .......... .......... ..........
E110019      .......... .......... ....C..... ....G..... .......... .......... ..........
B7A          .......... .......... ....C..... ....G..... .......... .......... ..........
CB9615       .......... .......... .......... .G..G..... .......... .......... ..........
O157_Sakai   .......... .......... .......... .G..G..... .......... .......... ..........
IAI39        .......... .......... .......... ....G..... .......... .......... ..........
UMN026       .......... .......... .......... ....G..... .......... .......... ..........
SMS-3-5      .......... .......... .......... ....G..... .......... .......... ..........
536          .......... .......... ....C..A.. ....G..... .......... .......... ..........
APEC_O1      .......... .......... ....C..... ....G..... .......... .......... ..........
CFT073       .......... .......... ....C..... ....G..... .......... .......... ..........
E2348/69     .......... .......... ....C..... ....G..... .......... .......... ..........
UTI89        .......... .......... ....C..... ....G..... .......... .......... ..........
F11          .......... .......... ....C..A.. ....G..... .......... .......... ..........
ED1a         .......... .......... ....C..... ....G..... .......... .......... ..........
83972        .......... .......... ....C..... ....G..... .......... .......... ..........
S88          .......... .......... ....C..... ....G..... .......... .......... ..........
E._albertii  ...A..T..T ......G.C. .T..C..... .GC.G.C..T ..A....G.. ....C..... ..........

        14351
K-12         GGCCAGCCGA TAGGTCATTT TAATGGCACT TGCATTGATG ACCAGGATAT CACTATCGAT AACTATGAAT
53638        .......... .......... .......... .......... .......... .......... ..........
ATCC_8739    .......... .......... .......... .......... .......... .......... ..........
HS           .......... .......... .......... .......... .......... .......... ..........
BW2952       .......... .......... .......... .......... .......... .......... ..........
12009        .......... .......... C........C .......... .......... .......... ..........
B171         .......... .......... C........C .......... .......... .......... ..........
E24377A      .......... .......... C........C .......... .......... .......... ..........
SE11         .......... .......... C........C .......... .......... .......... ..........
11128        .......... .......... C........C .......... .......... .......... ..........
11368        .......... .......... C........C .......... .......... .......... ..........
IAI1         .......... .......... C........C .......... .......... .......... ..........
E110019      .......... .......... C........C .......... .......... .......... ..........
B7A          .......... .......... C........C .......... .......... .......... ..........
CB9615       .......... .......... C......... .......... .......... .......... ..........
O157_Sakai   .......... .......... C......... .......... .......... .......... ..........
IAI39        .....A.... .......... C......... .......... .......... .......... ..........
UMN026       .....A.... .......... C......... .......... .......... .......... ..........
SMS-3-5      .....A.... .......... C......... .......... .......... .......... ..........
536          .......... .......... C........C .......... .......... .......... ..........
APEC_O1      .......... .......... C........C .......... .......... .......... ..........
CFT073       .......... .......... C........C .......... .......... .......... ..........
E2348/69     .......... .......... C........C .......... .......... .......... ...G......
UTI89        .......... .......... C........C .......... .......... .......... ..........
F11          .......... .......... C........C .......... .......... .......... ..........
ED1a         .......... .......... C........C .......... .......... .......... ..........
83972        .......... .......... C........C .......... .......... .......... ..........
S88          .......... .......... C........C .......... .......... .......... ..........
E._albertii  ........A. ....C..... ........TC .......... .......... .G.C..T... ........G.
```

```
         14421
K-12         TCACTACTGA CTATCTGGAG AATGCCACCT GTGGTGAAAA AGTAGTGGAA GAGACGCTTG TCAGCCACCT
53638        .......... .......... .......... .......... .......... .......... ..........
ATCC_8739    .......... .......... .......... .......... .......... .......... ..........
HS           .......... .......... .......... .......... .......... .......... ..........
BW2952       .......... .......... .......... .......... .......... .......... ..........
12009        .......... .......... .........A .C........ .......... .......... ..........
B171         .......... .......... .........A .C........ .......... .......... ..........
E24377A      .......... .......... .........A .C........ .......... .......... ..........
SE11         .......... .......... .........A .C........ .......... .......... ..........
11128        .......... .......... .........A .C........ .......... .......... ..........
11368        .......... .......... .........A .C........ .......... .......... ..........
IAI1         .......... .......... .........A .C........ .......... .......... ..........
E110019      .......... .......... .........A .C........ .......... .......... ..........
B7A          .......... .......... .........A .C........ .......... .......... ..........
CB9615       .......... .......... .........A .......... .......... ........C. ..........
O157_Sakai   .......... .......... .........A .......... .......... ........C. ..........
IAI39        .......... .......... .........A .......... ...G...... .......... ..........
UMN026       .......... .......... .........A ....A..... ......A... ........C. ..........
SMS-3-5      .......... .......... .........A ....C..... ......A... ........C. ..........
536          .......... .......... .........A .C........ .......... .......... ..........
APEC_O1      .......... .......... .........A .C........ .......... .......... ..........
CFT073       .......... .......... .........A .C........ .......... .......... ..........
E2348/69     .......... .......... .........A .......... .......... .......... ..........
UTI89        .......... .......... .........A .C........ .......... .......... ..........
F11          .......... .......... .........A .C........ .......... .......... ..........
ED1a         .......... .......... .........A .C........ .......... .......... ..........
83972        .......... .......... .........A .C........ .......... .......... ..........
S88          .......... .......... .........A .C........ .......... .......... ..........
E._albertii  ....C..C.. ...C...A.A G.....G..A .C..C..G.. C..G...... ........G. .T......T.

         14491
K-12         GCTGAAATCA AACTGCCTGA TCACCCATCA ACCAGATTGG GGTTCGCTCC AAATTCAGTA TCGTGGACGC
53638        .......... .......... .......... .......... ......A... .......... ..........
ATCC_8739    .......... .......... .......... .......... ......A... .......... ..........
HS           .......... .......... .......... .......... ......A... .......... ..........
BW2952       .......... .......... .......... .......... .......... .......... ..........
12009        .......... .......... .......... .......... ......A... .......... ..........
B171         .......... .......... .......... .......... ......A... .......... ..........
E24377A      .......... .......... .......... .......... ......A... .......... ..........
SE11         .......... .......... .......... .......... ......A... .......... ..........
11128        .......... .......... .......... .......... ......A... .......... ..........
11368        .......... .......... .......... .......... ......A... .......... ..........
IAI1         .......... .......... .......... .......... ......A... .......... ..........
E110019      .......... .......... .......... .......... ......A... .......... ..........
B7A          .......... .......... .......... .......... ......A... .......... ..........
CB9615       .......... .......... .......... .......... ......A... .......... ..........
O157_Sakai   .......... .......... .......... .......... ......A... .......... ..........
IAI39        .......... .......... .......... .......... ..A...A... .......... ..........
UMN026       .......... ..T....... .......... .......... ..A...A... .......... ..........
SMS-3-5      .......... .......... .......... .......... ..A...A... .......... ..........
536          .......... ..T....... .......... .......... ..A...A... .......... ..........
APEC_O1      .......... ..T....... .......... .......... ..A...A... .......... ..........
CFT073       .......... .......... .......... .......... ......A... .......... ..........
E2348/69     .......... .......... .......... .......... ......A... .......... ..........
UTI89        .......... ..T....... .......... .......... ..A...A... .......... ..........
F11          .......... ..T....... .......... .......... ..A...A... .......... ..........
ED1a         .......... .......... .......... .......... ......A... .......... ..........
83972        .......... .......... .......... .......... ......A... .......... ..........
S88          .......... ..T....... .......... .......... ..A...A... .......... ..........
E._albertii  .........C .......... .A..T..C.. G.....C... ..C..AA.T. .......... C..A..T...
```

```
           14561
K-12         CAAATTGACA GAGAAAAACT GCTGCGTTAC CTGGTCTCAT TCCGTCATCA CAACGAGTTC CACGAACAGT
53638        .......... .......... .......... .......... .......... .......... ..........
ATCC_8739    .......... .......... .......... .......... .......... .......... ..........
HS           .......... .......... .......... .......... .......... .......... ..........
BW2952       .......... .......... .......... .......... .......... .......... ..........
12009        .......... .......... .......... .......... .......... .......... ..........
B171         .......... .......... .......... .......... .......... .......... ..........
E24377A      .......... .......... .......... .......... .......... .......... ..........
SE11         .......... .......... .......... .......... .......... .......... ..........
11128        .......... .......... .......... .......... .......... .......... ..........
11368        .......... .......... .......... .......... .......... .......... ..........
IAI1         .......... .......... .......... .......... .......... .......... ..........
E110019      .......... .......... .......... .......... .......... .......... ..........
B7A          .......... .......... .......... .......... .......... .......... ..........
CB9615       .......... .......... .......... .......... .......... .......... ..........
O157_Sakai   .......... .......... .......... .......... .......... .......... ..........
IAI39        .......... .......... .......... ........T. ....C..... .......... ..........
UMN026       .......... .......... .......... .....T..T. .......... .......... ..........
SMS-3-5      .......... .......... .......... ........T. ....C..... .......... ..........
536          .......... .......... .......... .....T..T. .......... .......... ..........
APEC_O1      .......... .......... .......... .....T..T. .......... .......... ..........
CFT073       .......... .......... .......... ........T. .......... .......... ..........
E2348/69     .......... .......... .......... ........T. .......... .......... ..........
UTI89        .......... .......... .......... .....T..T. .......... .......... ..........
F11          .......... .......... .......... .....T..T. .......... .......... ..........
ED1a         .......... .......... .......... ........T. .......... .......... ..........
83972        .......... .......... .......... ........T. .......... .......... ..........
S88          .......... .......... .......... .....T..T. .......... .......... ..........
E._albertii  A.G....... .G.....GT. .........T ........T. .......... .......... ..T..G..A.

           14631
K-12         GCGTGGAACG CATCTTTAAT GACCTGTTAC GCTTCTGCCA GCCAGAAAAA TTGAGCGTTT ACGCACGTTA
53638        .......... .......... .......... .......... .......... .......... ..........
ATCC_8739    .......... .......... .......... .......... .......... .......... ..........
HS           .......... .......... .......... .......... .......... .......... ..........
BW2952       .......... .......... .......... .......... .......... .......... ..........
12009        .......... .......... .......... .......... .......... .......... ..........
B171         .......... .......... .......... .......... .......... .......... ..........
E24377A      .......... .......... .......... .......... ......G... ..A....... ..........
SE11         .......... .......... .......... .......... ......G... ..A....... ..........
11128        .......... .......... .......... .......... ......G... ..A....... ..........
11368        .......... .......... .......... .......... ......G... ..A....... ..........
IAI1         .......... .......... .......... .......... ......G... ..A....... ..........
E110019      .......... .......... .......... .......... ......G... ..A....... ..........
B7A          .......... .......... .......... .......... ......G... ..A....... ..........
CB9615       .......... .......... .......... .......... ......G... .......... ..........
O157_Sakai   .......... .......... .......... .......... ......G... .......... ..........
IAI39        .......... .......... .......... .......... ......G... .......... ..........
UMN026       .......... .......... .......... .......... ......G... .......... ..........
SMS-3-5      .......... .......... .......... .......... ......G... .......... ..........
536          .......... .......... .......... .......... ......G... .......... ..........
APEC_O1      .......... .......... .......... .......... ......G... .......... ..........
CFT073       .......... .......... .......... .......... .......... .......... ..........
E2348/69     .......... .......... .......... .......... .......... .......... ..........
UTI89        .......... .......... .......... .......... ......G... .......... ..........
F11          .......... .......... .......... .......... ......G... .......... ..........
ED1a         .......... .......... .......... .......... .......... .......... ..........
83972        .......... .......... .......... .......... .......... .......... ..........
S88          .......... .......... .......... .......... ......G... .......... ..........
E._albertii  ....A..G.. T..T...... .......... .......T.. .......... ..A....... ....T.....
```

```
        14701
K-12          TACCCGTCGT GGCGGTCTGG ACATTAACCC GTGGCGCAGT AATAGCGATT TTGTCCCATC GACCACAAGA
53638         .......... .......... .......... .......... .......... .......... ..........
ATCC_8739     .......... .......... .......... .......... .......... .......... ..........
HS            .......... .......... .......... .......... .......... .......... ..........
BW2952        .......... .......... .......... .......... .......... .......... ..........
12009         .......... .......... .......... .......... .......... .......... ..........
B171          .......... .......... .......... .......... .......... .......... ..........
E24377A       .......... .......... .......... .......... .......... .......... ..........
SE11          .......... .......... .......... .......... .......... .......... ..........
11128         .......... .......... .......... .......... .......... .......... ..........
11368         .......... .......... .......... .......... .......... .......... ..........
IAI1          .......... .......... .......... .......... .......... .......... ..........
E110019       .......... .......... .......... .......... .......... .......... ..........
B7A           .......... .......... .......... .......... .......... .......... ..........
CB9615        .......... .......... .......... .......... .......... .......... ..........
O157_Sakai    .......... .......... .......... .......... .......... .......... ..........
IAI39         .......... .......... .......... .......... .......... .......... ..........
UMN026        .......... .......... .......... .......... .......... .......... ..........
SMS-3-5       .......... .......... .......... .......... .......... .......... ..........
536           .......... .......... .......... .......... ....A..... .......... ..........
APEC_O1       .......... .......... .......... .......... .......... .......... ..........
CFT073        .......... .......... .......... .......... ....A..... .......... ..........
E2348/69      .......... .......... .......... .......... ....A..... .......... ..........
UTI89         .......... .......... .......... .......... .......... .......... ..........
F11           .......... .......... .......... .......... ....A..... .......... ..........
ED1a          .......... .......... .......... .......... ....A..... .......... ..........
83972         .......... .......... .......... .......... ....A..... .......... ..........
S88           .......... .......... .......... .......... .......... .......... ..........
E._albertii   ...T..C..C ..T..G.... .......T.. C........C ....C...C. .......... .GTG..CC.G

        14771
K-12          CTGGTTCGGC AATAAATTTT TTCTCAATTT TGCGTGCTGG ATTCACGCAG AAGGTTGTGA AAGGTCATCA
53638         .......... .......... .......... .......... .......... .......... ..........
ATCC_8739     .......... .......... .......... .......... .......... .......... ..........
HS            .......... .......... .......... .......... .......... .......... ..........
BW2952        .......... .......... .......... .......... .......... .......... ..........
12009         .......... .......... .......... .......... .......... .......... ..........
B171          .......... .......... .......... .......... .......... .......... ..........
E24377A       .......... .......... .......... .......... .......... .......... ..........
SE11          .......... .......... .......... .......... .......... .......... ..........
11128         .......... .......... .......... .......... .......... .......... ..........
11368         .......... .......... .......... .......... .......... .......... ..........
IAI1          .......... .......... .......... .......... .......... .......... ..........
E110019       .......... .......... .......... .......... .......... .......... ..........
B7A           .......... .......... .......... .......... .......... .......... ..........
CB9615        .......... .......... .......... .......... .......... .......... ..........
O157_Sakai    .......... .......... .......... .......... .......... .......... ..........
IAI39         .......... .......... .......... .......... .......... .......... ..........
UMN026        .......... .......... .......... .......... .......... .......... ..........
SMS-3-5       .......... .......... .......... .......... .......... .......... ..........
536           .......... .......... .......... .......... .......... .......... .......G..
APEC_O1       .......... .......... .......... .......... .......... .......... .......G..
CFT073        .......... .......... .......... .......... .......... .......... .......G..
E2348/69      .......... .......... .......... .......... .......... .......... .......G..
UTI89         .......... .......... .......... .......... .......... .......... .......G..
F11           .......... .......... .......... .......... .......... .......... .......G..
ED1a          .......... .......... .......... .......... .......... .......... .......G..
83972         .......... .......... .......... .......... .......... .......... .......G..
S88           .......... .......... .......... .......... .......... .......... .......G..
E._albertii   .....A..A. .......... .......... .....A.... G........T .......... ...A......
```

```
         14841
K-12         GGCAGGGCTA TTGTAATCAA AGGGAATGAC GATATTCGTC CCATAAGGAG TTTTTCTTGA TTACACATAT
53638        .......... .......... .......... .......... .......... .......... ..........
ATCC_8739    .......... .......... .......... .......... .......... .......... ..........
HS           .......... .......... .......... .......... .......... .......... ..........
BW2952       .......... .......... .......... .......... T......... .......... ..........
12009        .......... .......... .......... .......... .......... .......... ..........
B171         .......... .......... .......... .......... .......... .......... ..........
E24377A      .......... .......... .......... .......... .......... .......... ..........
SE11         .......... .......... .......... .......... .......... .......... ..........
11128        .......... .......... .......... .......... .......... .......... ..........
11368        .......... .......... .......... .......... .......... .......... ..........
IAI1         .......... .......... .......... .......... .......... .......... ..........
E110019      .......... .......... .......... .......... .......... .......... ..........
B7A          .......... .......... .......... .......... .......... .......... ..........
CB9615       .......... .......... .......... .......... .......... .......... ..........
O157_Sakai   .......... .......... .......... .......... .......... .......... ..........
IAI39        .......... .......... .......... .......... .......... .......... ..........
UMN026       .......... .......... .......... .......... .......... .......... ..........
SMS-3-5      .......... .......... .......... .......... .......... .......... ..........
536          .......... .......... .......... .......... .......... .......... ..........
APEC_O1      .......... .......... .......... .......... .......... .......... ..........
CFT073       .......... .......... .......... .......... .......... .......... ..........
E2348/69     .......... .......... .......... .......... .......... .......... ..........
UTI89        .......... .......... .......... .......... .......... .......... ..........
F11          .......... .......... .......... .......... .......... .......... ..........
ED1a         .......... .......... .......... .......... .......... .......... ..........
83972        .......... .......... .......... .......... .......... .......... ..........
S88          .......... .......... .......... .......... .......... .......... ..........
E._albertii  .......... .......... .......... .......... .......... .......... ..........

         14911
K-12         TAGCCCGCTT GGCTCCATGG ATATGTTGTC GCAGCTGGAA GTGGATATGC TTAAACGCAC CGCCAGCAGC
53638        .......... .......... .......... .......... .......... .......... ..........
ATCC_8739    .......... .......... .......... .......... .......... .......... ..........
HS           .......... .......... .......... .......... .......... .......... ..........
BW2952       .......... .......... .......... .......... .......... .......... ..........
12009        .......... .......... .......... .......... .......... .......... ..........
B171         .......... .......... .......... .......... .......... .......... ..........
E24377A      .......... .......... .......... .......... .......... .......... ..........
SE11         .......... .......... .......... .......... .......... .......... ..........
11128        .......... .......... .......... .......... .......... .......... ..........
11368        .......... .......... .......... .......... .......... .......... ..........
IAI1         .......... .......... .......... .......... .......... .......... ..........
E110019      .......... .......... .......... .......... .......... .......... ..........
B7A          .......... .......... .......... .......... .......... .......... ..........
CB9615       .......... .......... .......... .......... .......... .......... ..........
O157_Sakai   .......... .......... .......... .......... .......... .......... ..........
IAI39        .......... .......... .......... .......... .......... .......... ..........
UMN026       .......... .......... .......... .......... .......... .......... ..........
SMS-3-5      .......... .......... .......... .......... .......... .......... ..........
536          .......... .......... .......... .......... .......... .......... ..........
APEC_O1      .......... .......... .......... .......... .......... .......... ..........
CFT073       .......... .......... .......... .......... .......... .......... ..........
E2348/69     .......... .......... .......... .......... .......... .......... ..........
UTI89        .......... .......... .......... .......... .......... .......... ..........
F11          .......... .......... .......... .......... .......... .......... ..........
ED1a         .......... .......... .......... .......... .......... .......... ..........
83972        .......... .......... .......... .......... .......... .......... ..........
S88          .......... .......... .......... .......... .......... .......... ..........
E._albertii  .......... .......... .......... .......... .......... .......... ..........
```

```
          14981
K-12           GACCTCTATC AACTGTTTCG CAACTGTTCA CTTGCCGTAC TGAACTCCGG TAGTTTGACC GATAACAGCA
53638          .......... .......... .......... .......... .......... .......... ..........
ATCC_8739      .......... .......... .......... .......... .......... .......... ..........
HS             .......... .......... .......... .......... .......... .......... ..........
BW2952         .......... .......... .......... .......... .......... .......... ..........
12009          .......... .......... .......... .......... .......... .......... ..........
B171           .......... .......... .......... .......... .......... .......... ..........
E24377A        .......... .......... .......... .......... .......... .......... ..........
SE11           .......... .......... .......... .......... .......... .......... ..........
11128          .......... .......... .......... .......... .......... .......... ..........
11368          .......... .......... .......... .......... .......... .......... ..........
IAI1           .......... .......... .......... .......... .......... .......... ..........
E110019        .......... .......... .......... .......... .......... .......... ..........
B7A            .......... .......... .......... .......... .......... .......... ..........
CB9615         .......... .......... .......... .......... .......... .......... ..........
O157_Sakai     .......... .......... .......... .......... .......... .......... ..........
IAI39          .......... .......... .......... .......... .......... .......... ..........
UMN026         .......... .......... .......... .......... .......... .......... ..........
SMS-3-5        .......... .......... .......... .......... .......... .......... ..........
536            .......... .......... .......... .......... .......... .......... ..........
APEC_O1        .......... .......... .......... .......... .......... .......... ..........
CFT073         .......... .......... .......... .......... .......... .......... ..........
E2348/69       .......... .......... .......... .......... .......... .......... ..........
UTI89          .......... .......... .......... .......... .......... .......... ..........
F11            .......... .......... .......... .......... .......... .......... ..........
ED1a           .......... .......... .......... .......... .......... .......... ..........
83972          .......... .......... .......... .......... .......... .......... ..........
S88            .......... .......... .......... .......... .......... .......... ..........
E._albertii    .......... .......... .........G ........T. .......... ...CC..... ..........

          15051
K-12           AAGAATTGCT GTCTCGTTTT GAAAATTTCG ATATTAACGT CTTGCGCCGT GAACGCGGCG TAAAGCTGGA
53638          .......... .......... .......... .......... .......... .......... ..........
ATCC_8739      .......... .......... .......... .......... .......... .......... ..........
HS             .......... .......... .......... .......... .......... .......... ..........
BW2952         .......... .......... .......... .......... .......... .......... ..........
12009          .......... .......... .......... .......... .......... .......... ..........
B171           .......... .......... .......... .......... .......... .......... ..........
E24377A        .......... .......... .......... .......... .C........ .......... ..........
SE11           .......... .......... .......... .......... .C........ .......... ..........
11128          .......... .......... .......... .......... .C........ .......... ..........
11368          .......... .......... .......... .......... .C........ .......... ..........
IAI1           .......... .......... .......... .......... .C........ .......... ..........
E110019        .......... .......... .......... .......... .C........ .......... ..........
B7A            .......... .......... .......... .......... .C........ .......... ..........
CB9615         .......... .......... .......... .......... .......... .......... ..........
O157_Sakai     .......... .......... .......... .......... .......... .......... ..........
IAI39          .......... .......... .......... .......... .C........ .......... ..........
UMN026         .......... .......... .......... .......... .C........ .......... ..........
SMS-3-5        .......... .......... .......... .......... .C........ .......... ..........
536            .......... .......... .......... .......... .C........ .......... ..........
APEC_O1        .......... .......... .......... .......... .C........ .......... ..........
CFT073         .......... .......... .......... .......... .C........ .......... ..........
E2348/69       .......... .......... .......... .......... .C........ .......... ..........
UTI89          .......... .......... .......... .......... .C........ .......... ..........
F11            .......... .......... .......... .......... .C........ .......... ..........
ED1a           .......... .......... .......... .......... .C........ .......... ..........
83972          .......... .......... .......... .......... .C........ .......... ..........
S88            .......... .......... .......... .......... .C........ .......... ..........
E._albertii    .......... .......... ..G.....T. .C........ .C........ .......... .T........
```

```
            15121
K-12         ACTGATTAAT CCCCCGGAAG AGGCTTTTGT CGATGGGCGA ATTATTCGCG CTTTGCAGGC CAACTTGTTC
53638        .......... .......... .......... .......... .......... .......... ..........
ATCC_8739    .......... .......... .......... .......... .......... .......... ..........
HS           .......... .......... .......... .......... .......... .......... ..........
BW2952       .......... .......... .......... .......... .......... .......... ..........
12009        .......... .......... .......... .......... .......... .......... ..........
B171         .......... .......... .......... .......... .......... .......... ..........
E24377A      .......... .......... .......... .......... .......... .......... ..........
SE11         .......... .......... .......... .......... .......... .......... ..........
11128        .......... .......... .......... .......... .......... .......... ..........
11368        .......... .......... .......... .......... .......... .......... ..........
IAI1         .......... .......... .......... .......... .......... .......... ..........
E110019      .......... .......... .......... .......... .......... .......... ..........
B7A          .......... .......... .......... .......... .......... .......... ..........
CB9615       .......... .......... .......... .......... .......... .......... ..........
O157_Sakai   .......... .......... .......... .......... .......... .......... ..........
IAI39        .......... .......... .......... .......... .......... .......... ..........
UMN026       .......... .......... .......... .......... .......... .......... ..........
SMS-3-5      .......... .......... .......... .......... .......... .......... ..........
536          ......A... .......... .......... .......... .......... .......... ..........
APEC_O1      ......A... .......... .......... .......... .......... .......... ..........
CFT073       ......A... .......... .......... .......... .......... .......... ..........
E2348/69     ......A... .......... .......... .......... .......... .......... ..........
UTI89        ......A... .......... .......... .......... .......... .......... ..........
F11          ......A... .......... .......... .......... .......... .......... ..........
ED1a         ......A... .......... .......... .......... .......... .......... ..........
83972        ......A... .......... .......... .......... .......... .......... ..........
S88          ......A... .......... .......... .......... .......... .......... ..........
E._albertii  ......C... ..A....... .......C.. .........T .....C.... .C........ T........T

            15191
K-12         GCGGTCCTGC GTGACATTCT CTTCGTTTAC GGGCAAATCC ATAACACCGT TCGTTTTCCC AACCTGAATC
53638        .......... .......... .......... .......... .......... .......... ..........
ATCC_8739    .......... .......... .......... .......... .......... .......... ..........
HS           .......... .......... .......... .......... .......... .......... ..........
BW2952       .......... .......... .......... .......... .......... .......... ..........
12009        .......... .......... .......... .......... .......... .......... ..........
B171         .......... .......... .......... .......... .......... .......... ..........
E24377A      .....T.... .A........ .......... .......... ....T..... .......... ..........
SE11         .....T.... .A........ .......... .......... ....T..... .......... ..........
11128        .....T.... .A........ .......... .......... ....T..... .......... ..........
11368        .....T.... .A........ .......... .......... ....T..... .......... ..........
IAI1         .....T.... .A........ .......... .......... ....T..... .......... ..........
E110019      .....T.... .A........ .......... .......... ....T..... .......... ..........
B7A          .....T.... .A........ .......... .......... ....T..... .......... ..........
CB9615       .....T.... .A........ .......... .......... ....T..... .......... ..........
O157_Sakai   .....T.... .A........ .......... .......... ....T..... .......... ..........
IAI39        .....T.... .......... .......... .......... .......... .......... ..........
UMN026       .....T.... .......... .......... .......... .......... .......... ..........
SMS-3-5      .....T.... .C........ .......... .......... .......... .......... ..........
536          .....T.... .......... .........T ........T. .......... .......... ..........
APEC_O1      .....T.... .......... .........T ........T. .......... .......... ..........
CFT073       .....T.... .......... .........T ........T. .......... .......... ..........
E2348/69     .....T.... .......... .........T ........T. .......... .......... ..........
UTI89        .....T.... .......... .........T ........T. .......... .......... ..........
F11          .....T.... .......... .........T ........T. .......... .......... ..........
ED1a         .....T.... .......... .........T ........T. .......... .......... ..........
83972        .....T.... .......... .........T ........T. .......... .......... ..........
S88          .....T.... .......... .........T ........T. .......... .......... ..........
E._albertii  .......... .C........ .......... ........T. .......... .........A ........C.
```

```
         15261
K-12         TCGACAACTC CGTCCACATC ACTAACCTGG TCTTTTCCAT CTTGCGTAAC GCTCGCGCGC TGCATGTGGG
53638        .......... .......... .......... .......... .......... .......... ..........
ATCC_8739    .......... .......... .......... .......... .......... .......... ..........
HS           .......... .......... .......... .......... .......... .......... ..........
BW2952       .......... .......... .......... .......... .......... .......... ..........
12009        .......... .......... .......... .......... .......... .......... ..........
B171         .......... .......... .......... .......... .......... .......... ..........
E24377A      .......... .......... .......... .......... .......... .......... ..........
SE11         .......... .......... .......... .......... .......... .......... ..........
11128        .......... .......... .......... .......... .......... .......... ..........
11368        .......... .......... .......... .......... .......... .......... ..........
IAI1         .......... .......... .......... .......... .......... .......... ..........
E110019      .......... .......... .......... .......... .......... .......... ..........
B7A          .......... .......... .......... .......... .......... .......... ..........
CB9615       .......... .......... .......... .......... .......... ........A. ..........
O157_Sakai   .......... .......... .......... .......... .......... .......... ..........
IAI39        .......... .......... .......... .......... .......... .......... .......T..
UMN026       .......... .......... .......... .......... .......... .......... .......T..
SMS-3-5      .......... .......... .......... .......... .......... .......... .......T..
536          .......... .......... .......... .......... .......... .......... .......C..
APEC_O1      .......... .......... .......... .......... .......... .......... .......C..
CFT073       .......... .......... .......... .......... .......... .......... .......C..
E2348/69     .......... .......... .......... .......... .......... .......... .......C..
UTI89        .......... .......... ......T... .......... .......... .......... .......C..
F11          .......... .......... .......... .......... .......... .......... .......C..
ED1a         .......... .......... .......... .......... .......... .......... .......C..
83972        .......... .......... .......... .......... .......... .......... .......C..
S88          .......... .......... .......... .......... .......... .......... .......C..
E._albertii  .......... ......T..T ..C....... .......T.. A.....C... ..C....... .......T..

         15331
K-12         TGAAGCGCCA AATATGGTGG TCTGCTGGGG CGGTCACTCA ATTAACGAAA ACGAGTATTT GTATGCCCGT
53638        .......... .......... .......... .......... .......... .......... ..........
ATCC_8739    .......... .......... .......... .......... .......... .......... ..........
HS           .......... .......... .......... .......... .......... .......... ..........
BW2952       .......... .......... .......... .......... .......... .......... ..........
12009        .......... .......... .......... .......... .......... .T........ ..........
B171         .......... .......... .......... .......... .......... .T........ ..........
E24377A      .......... .......... .......... .......... .......... .......... ..........
SE11         .......... .......... .......... .......... .......... .......... ..........
11128        .......... .......... .......... .......... .......... .......... ..........
11368        .......... .......... .......... .......... .......... .......... ..........
IAI1         .......... .......... .......... .......... .......... .......... ..........
E110019      .......... .......... .......... .......... .......... .T........ ..........
B7A          .......... .......... .......... .......... .......... .......... ..........
CB9615       .......... .......... .......... .......... .......... .T........ ..........
O157_Sakai   .......... .......... .......... .......... .......... .T........ ..........
IAI39        .......... .......... .......... ...G...... .......... .......... ..........
UMN026       .......... .......... .......... ...G...... .......... .......... ..........
SMS-3-5      .......... .......... .......... ...G...... .......... .......... ..........
536          .......... .......... .......... .......... .......... .......... ..........
APEC_O1      .......... .......... .......... .......... .......... .......... ..........
CFT073       .......... .......... .......... .......... .......... .......... ..........
E2348/69     .......... .......... .......... .......... .......... .......... ..........
UTI89        .......... .......... .......... .......... .......... .......... ..........
F11          .......... .......... .......... .......... .......... .......... ..........
ED1a         .......... .......... .......... .......... .......... .......... ..........
83972        .......... .......... .......... .......... .......... .......... ..........
S88          .......... .......... .......... .......... .......... .......... ..........
E._albertii  C........G .......... .G........ .......... .......... ........C. ...C..T...
```

```
         15401
K-12         CGCGTCGGAA ACCAGCTGGG CCTGCGTGAG CTGAATATCT GCACCGGCTG TGGTCCGGGA GCGATGGAAG
53638        .......... .......... .......... .......... .......... .......... ..........
ATCC_8739    .......... .......... .......... .......... .......... .......... ..........
HS           .......... .......... .......... .......... .......... .......... ..........
BW2952       .......... .......... .......... .......... .......... .......... ..........
12009        .......... .......... .......... .......... .......... .......... ..........
B171         .......... .......... .......... .......... .......... .......... ..........
E24377A      .......... .......... .......... .......... .......... .......... ..........
SE11         .......... .......... .......... .......... .......... .......... ..........
11128        .......... .......... .......... .......... .......... .......... ..........
11368        .......... .......... .......... .......... .......... .......... ..........
IAI1         .......... .......... .......... .......... .......... .......... ..........
E110019      .......... .......... .......... .......... .......... .......... ..........
B7A          .......... .......... .......... .......... .......... .......... ..........
CB9615       .......... .......... .......... .......... .......... .......... ..........
O157_Sakai   .......... .......... .......... .......... .......... .......... ..........
IAI39        ........G. .T........ .......... .......... .......... .......... ..........
UMN026       .....A..G. .T........ .......... .......... .......... .......... ..........
SMS-3-5      ........G. .T........ .......... .......... .......... .......... ..........
536          ........G. .......... .......... .......... .......... .......... ..........
APEC_O1      ........G. .T........ .......... .......... .......... .......... ..........
CFT073       ........G. .......... .......... .......... .......... .......... ..........
E2348/69     ........G. .......... .......... .......... .......... .......... ..........
UTI89        ........G. .T........ .......... .......... .......... .......... ..........
F11          ........G. .......... .......... .......... .......... .......... ..........
ED1a         .....A..G. .......... .......... .......... .......... .......... ..........
83972        ........G. .......... .......... .......... .......... .......... ..........
S88          ........G. .T........ .......... .......... .......... .......... ..........
E._albertii  ........T. .T.....C.. T.....C... ..A....... ....T..... .......... ..........

         15471
K-12         CGCCGATGAA AGGTGCTGCG GTCGGACACG CGCAGCAGCG TTACAAAGAC AGTCGTTTTA TTGGTATGAC
53638        .......... .......... .......... .......... .......... .......... ..........
ATCC_8739    .......... .......... .......... .......... .......... .......... ..........
HS           .......... .......... .......... .......... .......... .......... ..........
BW2952       .......... .......... .......... .......... .......... .......... ..........
12009        .......... .......... .......... .......... .......... .......... ..........
B171         .......... .......... .......... .......... .......... .......... ..........
E24377A      .......... .......... .......... .......... .......... .......... ..........
SE11         .......... .......... .......... .......... .......... .......... ..........
11128        .......... .......... .......... .......... .......... .......... ..........
11368        .......... .......... .......... .......... .......... .......... ..........
IAI1         .......... .......... .......... .......... .......... .......... ..........
E110019      .......... .......... .......... .......... .......... .......... ..........
B7A          .......... .......... .......... .......... .......... .......... ..........
CB9615       .......... .......... .......... .......... .......... .......... ..........
O157_Sakai   .......... .......... .......... .......... .......... .......... ..........
IAI39        ....A..... .......... .......... .A........ .......... .......... ..........
UMN026       .......... ...C...... .......... .A........ .......... .......... ..........
SMS-3-5      ....A..... .......... .......... .A........ .......... .......... ..........
536          .......... .......... .......... .......... .......... .....C.... ..........
APEC_O1      .......... .......... .......... .......... .......... .....C.... ..........
CFT073       .T........ .......... .......... .......... .......... .....C.... ..........
E2348/69     .......... .......... .......... .......... .......... .....C.... ..........
UTI89        .......... .......... .......... .......... .......... .....C.... ..........
F11          .......... .......... .......... .......... .......... .....C.... ..........
ED1a         .......... .......... .......... .......... .......... .....C.... ..........
83972        .......... .......... .......... .......... .......... .....C.... ..........
S88          .......... .......... .......... .......... .......... .....C.... ..........
E._albertii  .......... ...C..G... .....T.... .T.....A.. .......... ..C....... ....C.....
```

```
             15541
K-12         AGAGCCGTCG ATTATCGCCG CTGAACCGCC TAACCCGCTG GTCAACGAAT TGATCATCAT GCCAGATATC
53638        .......... .......... .......... .......... .......... .......... ...G......
ATCC_8739    .......... .......... .......... .......... .......... .......... ...G......
HS           .......... .......... .......... .......... .......... .......... ...G......
BW2952       .......... .......... .......... .......... .......... .......... ..........
12009        .......... .......... .......... .......... .......... .......... ...G......
B171         .......... .......... .......... .......... .......... .......... ...G......
E24377A      .......... .......... .......... .......... .......... .......... ...G......
SE11         .......... .......... .......... .......... .......... .......... ...G......
11128        C......... .......... .......... .......... .......... .......... ..........
11368        C......... .......... .......... .......... .......... .......... ..........
IAI1         .......... .......... .......... .......... .......... .......... ..........
E110019      .......... .......... .......... .......... .......... .......... ...G......
B7A          .......... .......... .......... .......... .......... .......... ...G......
CB9615       .......... .......... .......... .......... .......... .......... ...G......
O157_Sakai   .......... .......... .......... .......... .......... .......... ...G......
IAI39        ......A... .......... .......... .......... .......... .......... ...G......
UMN026       .......... .......... .......... .......... .......... .......... ...G......
SMS-3-5      ......A... .......... .......... .......... .......... .......... ...G......
536          .......... .......... .......... .......... .......... .......... ..........
APEC_O1      .......... .......... .......... .......... .......... .......... ..........
CFT073       .......... .......... .......... .......... .......... .......... ..........
E2348/69     .......... .......... .......... .......... .......... .......... ..........
UTI89        .......... .......... .......... .......... .......... .......... ..........
F11          .......... .......... .......... .......... .......... .......... ..........
ED1a         .......... .......... .......... .......... .......... .......... ..........
83972        .......... .......... .......... .......... .......... .......... ..........
S88          .......... .......... .......... .......... .......... .......... ..........
E._albertii  G.....A... ..C....... .......A.. .......... ..T......C .......... ...G.....T

             15611
K-12         GAAAAACGTC TGGAAGCGTT TGTCCGTATC GCTCACGGTA TCATTATCTT CCCTGGCGGT GTGGGTACGG
53638        .......... .......... .......... ........C. .......... .......... ..........
ATCC_8739    .......... .......... .......... ........C. .......... .......... ..........
HS           .......... .......... .......... ........C. .......... .......... ..........
BW2952       .......... .......... .......... .......... .......... .......... ..........
12009        .......... .......... .......... ........C. .......... .......... ..........
B171         .......... .......... .......... ........C. .......... .......... ..........
E24377A      .......... .......... .......... ........C. .......... ......A... ..........
SE11         .......... .......... .......... ........C. .......... ......A... ..........
11128        .......... .......... .......... .......... .......... .......... ..........
11368        .......... .......... .......... .......... .......... .......... ..........
IAI1         .......... .......... .......... .......... .......... .......... ..........
E110019      .......... .......... .......... ........C. .......... .......... ..........
B7A          .......... .......... .......... ........C. .......... ......A... ..........
CB9615       .......... .......... .......... ........C. .......... .......... ..........
O157_Sakai   .......... .......... .......... ........C. .......... .......... ..........
IAI39        .......... .......... .......... .......... .......... .......... ..........
UMN026       .......... .......... .......... .......... .......... .......... ..........
SMS-3-5      .......... .......... .......... .......... .......... .......... ..........
536          .......... .......... .......... .......... .......... .......... ........A.
APEC_O1      .......... .......... .......... .......... .......... .......... ........A.
CFT073       .......... .......... .......... .......... .......... .......... ........A.
E2348/69     .......... .......... .......... .......... .......... .......... ........A.
UTI89        .......... .......... .......... .......... .......... .......... ........A.
F11          .......... .......... .......... .......... .......... .......... ........A.
ED1a         .......... .......... .......... .......... .......... .......... ........A.
83972        .......... .......... .......... .......... .......... .......... ........A.
S88          .......... .......... .......... .......... .......... .......... ........A.
E._albertii  ........C. .......... C..G...... ..C.....C. .......... .......... ..........
```

```
          15681
K-12         CAGAAGAGTT GCTCTATTTG CTGGGAATTT TAATGAACCC GGCCAACAAA GATCAGGTTT TACCATTGAT
53638        .......... ...G...... .......... .......... .......... .......... ..........
ATCC_8739    .......... ...G...... .......... .......... .......... .......... ..........
HS           .......... ...G...... .......... .......... .......... .......... ..........
BW2952       .......... .......... .......... .......... .......... .......... ..........
12009        .......... ...G...... .......... .......... .......... .......... ..........
B171         .......... ...G...... .......... .......... .......... .......... ..........
E24377A      .......... ...G...... .......... .......... .......... .......... ..........
SE11         .......... ...G...... .......... .......... .......... .......... ..........
11128        .......... ...G...... .......... .......... .......... .......... ..........
11368        .......... ...G...... .......... .......... .......... .......... ..........
IAI1         .......... ...G...... .......... .......... .......... .......... ..........
E110019      .......... ...G...... .......... .......... .......... .......... ..........
B7A          .......... ...G...... .......... .......... .......... .......... ..........
CB9615       .......... ...G...... .......... .......... .......... .......... ..........
O157_Sakai   .......... ...G...... .......... .......... .......... .......... ..........
IAI39        .......... ...G...... .......... .......... .......... .......... ..........
UMN026       .......... ...G...... .......... .......... .......... .......... ..........
SMS-3-5      .......... ...G...... .......... .......... .......... .......... ........C.
536          .......... ...G...... .......... .......... .......... .......... ..........
APEC_O1      .......... ...G...... .......... .......... .......... .......... ..........
CFT073       .......... ...G...... .......... .......... .......... .......... ..........
E2348/69     .......... ...G...... .......... .......... .......... .......... ..........
UTI89        .......... ...G...... .......... .......... .......... .......... ..........
F11          .......... ...G...... .......... .......... .......... .......... ..........
ED1a         .......... ...G...... .......... .......... .......... .......... ..........
83972        .......... ...G...... .......... .......... .......... .......... ..........
S88          .......... ...G...... .......... .......... .......... .......... ..........
E._albertii  .T........ ...G.....A T.A..G.... .G.....T.. ...G...... A.......A. .G.....A..

          15751
K-12         CCTCACCGGC CCGAAAGAGA GCGCCGACTA CTTCCGCGTA CTGGACGAGT TTGTCGTGCA TACGCTGGGT
53638        .......... .......... .......... .......... .......... .......... ..........
ATCC_8739    .......... .......... .......... .......... .......... .......... ..........
HS           .......... .......... .......... .......... .......... .......... ..........
BW2952       .......... .......... .......... .......... .......... .......... ..........
12009        .......... .......... .......... .......... .......... .......A.. .........C
B171         .......... .......... .......... .......... .......... .......A.. .........C
E24377A      .......... .......... .......... .......... .......... .......A.. .........C
SE11         .......... .......... .......... .......... .......... .......A.. .........C
11128        .......... .......... .......... .......... .......... .......A.. .........C
11368        .......... .......... .......... .......... .......... .......A.. .........C
IAI1         .......... .......... .......... .......... .......... .......A.. ..........
E110019      .......... .......... .......... .......... .......... .......A.. .........C
B7A          .......... .......... .......... .......... .......... .......A.. .........C
CB9615       .......... .......... .......... .......... .......... .......A.. .........C
O157_Sakai   .......... .......... .......... .......... .......... .......A.. .........C
IAI39        .......... .......... .......... .......... .......... .......... ....T.....
UMN026       .......... .......... .......... .......... .......... .......... ....T.....
SMS-3-5      .......... .......... .......... .......... .......... .......... ....T.....
536          .......... .......... .......... .......... .......... .......... ..........
APEC_O1      .......... .......... .......... .......... .......... .......... ..........
CFT073       .......... .......... .......... .......... .......... .......... ..........
E2348/69     .......... .......... .......... .......... .......... .......... ..........
UTI89        .......... .......... .......... .......... .......... .......... ..........
F11          .......... .......... .......... .......... .......... .......... ..........
ED1a         .......... .......... .......... .......... .......... .......... ..........
83972        .......... .......... .......... .......... .......... .......... ..........
S88          .......... .......... .......... .......... .......... .......... ..........
E._albertii  .........A .......... .......T.. .......... .......... ....T..... .........A
```

```
        15821
K-12         GAAAACGCGC GCCGCCATTA CCGCATCATC ATTGATGACG CCGCTGAAGT CGCTCGTCAG ATGAAAAAAT
53638        .......... .......... .......... .......... .......... .......... ..........
ATCC_8739    .......... .......... .......... .......... .......... .......... ..........
HS           .......... .......... .......... .......... .......... .......... ..........
BW2952       .......... .......... .......... .......... .......... .......... ..........
12009        .......... .......... .......... .......... .......... .......... ..........
B171         .......... .......... .......... .......... .......... .......... ..........
E24377A      .......... .......... .......... .......... .......... .......... ..........
SE11         .......... .......... .......... .......... .......... .......... ..........
11128        .......... .......... .......... .......... .......... .......... ..........
11368        .......... .......... .......... .......... .......... .......... ..........
IAI1         .......... .......... .......... .......... .......... .......... ..........
E110019      .....T.... .......... .......... .......... .......... .......... ..........
B7A          .......... .......... .......... .......... .......... .......... ..........
CB9615       .......... .......... ......A... .......... .......... ...C...... ..........
O157_Sakai   .......... .......... ......A... .......... .......... ...C...... ..........
IAI39        .......... .......... .......... .......... .......... .......... ..........
UMN026       .......... .......... .......... .......... .......... .......... ..........
SMS-3-5      .......... .......... .......... .......... .......... .......... ..........
536          .......... .......... .......... .......... .......... .......... ..........
APEC_O1      .......... .......... .......... .......... .......... .......... ..........
CFT073       .......... .......... .......... .......... .......... .......... ..........
E2348/69     .......... .......... .......... .......... .......... .......... ..........
UTI89        .......... .......... .......... .......... .......... .......... ..........
F11          .......... .......... .......... .......... .......... .......... ..........
ED1a         .......... .......... .......... .......... .......... .......... ..........
83972        .......... .......... .......... .......... .......... .......... ..........
S88          .......... .......... .......... .......... .......... .......... ..........
E._albertii  ........A. .T........ T......... .......G.. .G........ G..C..G... ........GG

        15891
K-12         CGATGCCGCT GGTGAAAGAA AATCGCCGTG ATACAGGCGA TGCCTACAGC TTTAACTGGT CAATGCGCAT
53638        .......... .......... .......... .......... .......... .......... ..........
ATCC_8739    .......... .......... .......... .......... .......... .......... ..........
HS           .......... .......... .......... .......... .......... .......... ..........
BW2952       .......... .......... .......... .......... .......... .......... ..........
12009        .......... .......... ........C. .C..T..T.. C..T...... .......... ..........
B171         .......... .......... ........C. .C..T..T.. C..T...... .......... ..........
E24377A      .......... .......... ........C. .C..T..T.. C..T...... .......... ..........
SE11         .......... .......... ........C. .C..T..T.. C..T...... .......... ..........
11128        .......... .......... ........C. .C..T..T.. C..T...... .......... ..........
11368        .......... .......... ........C. .C..T..T.. C..T...... .......... ..........
IAI1         .......... .......... .......... .......... .......... .......... ..........
E110019      .......... .......... ........C. .C..T..T.. C..T...... .......... ..........
B7A          .......... .......... ........C. .C..T..T.. C..T...... .......... ..........
CB9615       .......... .......... .......... .......... .......... .......... ..........
O157_Sakai   .......... .......... .......... .......... .......... .......... ..........
IAI39        .......... .......... ........C. .C........ .......... .......... ..........
UMN026       .......... .......... ........C. .C........ .......... .......... ..........
SMS-3-5      .......... .......... ........C. .C........ .......... .......... ..........
536          .......... .......... ........C. .C..T..T.. C..T...... .......... ..........
APEC_O1      .......... .......... ........C. .C..T..T.. C..T...... .......... ..........
CFT073       .......... .......... ........C. .C..T..T.. C..T...... .......... ..........
E2348/69     .......... .......... ........C. .C..T..T.. C..T...... .......... ..........
UTI89        .......... .......... ........C. .C..T..T.. C..T...... .......... ..........
F11          .......... .......... ........C. .C..T..T.. C..T...... .......... ..........
ED1a         .......... .......... ........C. .C..T..T.. C..T...... .......... ..........
83972        .......... .......... ........C. .C..T..T.. C..T...... .......... ..........
S88          .......... .......... ........C. .C..T..T.. C..T...... .......... ..........
E._albertii  ........T. .......... ........C. ....C..A.. ...T..T... .......... ..........
```

```
         15961
K-12         TGCGCCAGAT TTGCAAATGC CGTTTGAGCC GTCTCACGAG AATATGGCTA ATCTGAAGCT TTACCCGGAT
53638        .......... .......... .......... .......... .......... .......... ..........
ATCC_8739    .......... .......... .......... .......... .......... .......... ..........
HS           .......... .......... .......... .......... .......... .......... ..........
BW2952       .......... .......... .......... .......... .......... .......... ..........
12009        ...A...... .......... .......... .......... .......... .......... ..........
B171         ...A...... .......... .......... .......... .......... .......... ..........
E24377A      ...A...... .......... .......... .......... .......... .......... ..........
SE11         ...A...... .......... .......... .......... .......... .......... ..........
11128        ...A...... .......... .......... .......... .......... .......... ..........
11368        ...A...... .......... .......... .......... .......... .......... ..........
IAI1         .......... .......... .......... .......... .......... .......... ..........
E110019      ...A...... .......... .......... .......... .......... .......... ..........
B7A          ...A...... .......... .......... .......... .......... .......... ..........
CB9615       .......... .......... .A........ .......... .......... .......... ..........
O157_Sakai   .......... .......... .A........ .......... .......... .......... ..........
IAI39        .......... .......... .......... .......... .......... .......... ..........
UMN026       .......... .......... .......... .......... .......... .......... ..........
SMS-3-5      .......... .......... .......... .......... .......... .......... ..........
536          .......... .......... .......... .......... .......... .......... ..........
APEC_O1      .......... .......... .......... .......... .......... .......... ..........
CFT073       .......... .......... .......... .......... .......... .......... ..........
E2348/69     .......... .......... .......... .......... .......... .......... ..........
UTI89        .......... .......... .......... .......... .......... .......... ..........
F11          .......... .......... .......... .......... .......... .......... ..........
ED1a         .......... .......... .......... .......... .......... .......... ..........
83972        .......... .......... .......... .......... .......... .......... ..........
S88          .......... .......... .......... .......... .......... .......... ..........
E._albertii  .A.A..G... C......... .......A.. .A.C..T..A ..C.....C. .......... G.........

         16031
K-12         CAACCTGTTG AAGTGCTGGC TGCCGACCTG CGCCGTGCGT TCTCCGGTAT TGTGGCGGGT AACGTAAAAG
53638        .......... .......... .......... .......... .......... .......... ..........
ATCC_8739    .......... .......... .......... .......... .......... .......... ..........
HS           .......... .......... .......... .....C.... .......... .......... ..........
BW2952       .......... .......... .......... .......... .......... .......... ..........
12009        .......... .......... .......... .......... .......... .......... ..........
B171         .......... .......... .......... .......... .......... .......... ..........
E24377A      .......... .......... .......... .......... .......... .......... ..........
SE11         .......... .......... .......... .......... .......... .......... ..........
11128        .......... .......... .......... .......... .......... .......... ..........
11368        .......... .......... .......... .......... .......... .......... ..........
IAI1         .......... .......... .......... .......... .......... .......... ..........
E110019      .......... .......... .......... .......... .......... .......... ..........
B7A          .......... .......... .......... .......... .......... .......... ..........
CB9615       .......... .......... ......T... .......... .......... .......... ..........
O157_Sakai   .......... .......... ......T... .......... .......... .......... ..........
IAI39        .....C.... .......... .......... .......... .......... .......... ..........
UMN026       .....C.... .......... .......... .......... .......... .......... ..........
SMS-3-5      .....C.... .......... .......... .......... .......... .......... ..........
536          .....C.... .......... .......... .......... .......... .......... ..T.......
APEC_O1      .....C.... .......... .......... .......... .......... .......... ..T.......
CFT073       .....C.... .......... .......... .......... .......... .......... ..T.......
E2348/69     .....C.... .......... .......... .......... .......... .......... ..T.......
UTI89        .....C.... .......... .......... .......... .......... .......... ..T.......
F11          .....C.... .......... .......... .......... .......... .......... ..T.......
ED1a         .....C.... .......... .......... .......... .......... .......... ..T.......
83972        .....C.... .......... .......... .......... .......... .......... ..T.......
S88          .....C.... .......... .......... .......... .......... .......... ..T.......
E._albertii  ..G....... ....A..... G..G..T... ..T..C.... .......... .......... .....T....
```

```
             16101
K-12         AAGTCGGTAT TCGCGCCATT GAAGAGTTTG GTCCTTACAA AATCAACGGC GATAAAGAGA TTATGCGTCG
53638        .......... .......... .......... .......... .......... .......... ..........
ATCC_8739    .......... .......... .......... .......... .......... .......... ..........
HS           .......... .......... .......... .......... .......... .......... ..........
BW2952       .......... .......... .......... .......... .......... .......... ..........
12009        .......... .......... .......... .......... .......... .......... ..........
B171         .......... .......... .......... .......... .......... .......... ..........
E24377A      .......... .......... .......... .......... .......... .......... ..........
SE11         .......... .......... .......... .......... .......... .......... ..........
11128        .......... .......... .......... .......... .......... .......... ..........
11368        .......... .......... .......... .......... .......... .......... ..........
IAI1         .......... .......... .......... .......... .......... .......... ..........
E110019      .......... .......... .......... .......... .......... .......... ..........
B7A          .......... .......... .......... .......... .......... .......... ..........
CB9615       .......... .......... .......... .......... .......... .......... ..........
O157_Sakai   .......... .......... .......... .......... .......... .......... ..........
IAI39        .......... .......... .......... .......T.. .......... .......... ..........
UMN026       .......... .......... .......... .......T.. .......... .......... ..........
SMS-3-5      .......... .......... .......... .......T.. .......... .......... ..........
536          .......... .......... ...A...... .......... .......... .......... ..........
APEC_O1      .......... .......... .......... .......... .......... .......... ..........
CFT073       .......... .......... .......... .......... .......... ........A. ..........
E2348/69     .......... .......... .......... .......... .......... ........A. ..........
UTI89        .......... .......... .......... .......... .......... .......... ..........
F11          .......... .......... ...A...... .......... .......... .......... ..........
ED1a         .......... .......... ...A...... .......... .......... .......... ..........
83972        .......... .......... .......... .......... .......... ........A. ..........
S88          .......... .......... .......... .......... .......... .......... ..........
E._albertii  .......C.. .......... ........C. .G........ .......... ........A. ..........

             16171
K-12         TATGGACGAC CTGCTACAGG GTTTTGTTGC CCAGCATCGT ATGAAGTTGC CAGGCTCAGC CTACATCCCT
53638        .......... .......... .......... .......... ......C... .......... ..........
ATCC_8739    .......... .......... .......... .......... ......C... .......... ..........
HS           .......... .......... .......... .......... ......C... .......... ..........
BW2952       .......... .......... .......... .......... .......... .......... ..........
12009        .......... .......... .......... .......... .......... .......... ..........
B171         .......... .......... .......... .......... .......... .......... ..........
E24377A      .......... .......... .......... .......... .......... .......... ..........
SE11         .......... .......... .......... .......... .......... .......... ..........
11128        .......... .......... .......... .......... .......... .......... ..........
11368        .......... .......... .......... .......... .......... .......... ..........
IAI1         .......... .......... .......... .......... .......... .......... ..........
E110019      .......... .......... .......... .......... .......... .......... ..........
B7A          .......... .......... .......... .......... .......... .......... ..........
CB9615       .......... .......... .......... .......... .......... .......... ..........
O157_Sakai   .......... .......... .......... .......... .......... .......... ..........
IAI39        ......T... .......... .......... .......... .......... .......... ..........
UMN026       ......T... .......... .......... .......... .......... .......... ..........
SMS-3-5      ......T... .......... .......... .......... .......... .......... ..........
536          .......... .......... .......... .......... .......... .......... ..........
APEC_O1      .......... .......... .......... .......... .......... .......... ..........
CFT073       .......... .......... .......... .......... .......... .......... ..........
E2348/69     .......... .......... .......... .......... .......... .......... ..........
UTI89        .......... .......... .......... .......... .......... .......... ..........
F11          .......... .......... .......... .......... .......... .......... ..........
ED1a         .......... .......... .......... .......... .......... .......... ..........
83972        .......... .......... .......... .......... .......... .......... ..........
S88          .......... .......... .......... .......... .......... .......... ..........
E._albertii  ......T... .......... .C.....C.. .......... .......... .T.....T.. ......T...
```

```
         16241
K-12         TGCTACGAAA TCTGCACGTA ATCTCCGCTC CCCCGGTGAC GTTTGTTGCC GGGTTTTCTC GTTTTTGGTC
53638        .......... .......... .......... .......... .......... .......... ..........
ATCC_8739    .......... .......... .......... .......... .......... .......... ..........
HS           .......... .......... .......... .......... .......... .......... ..........
BW2952       .......... .......... .......... .......... .......... .......... ..........
12009        .......... .......... .......... .......... .......... .......... ..........
B171         .......... .......... .......... .......... .......... .......... ..........
E24377A      .......... .......... .......... .......... .......... .......... ..........
SE11         .......... .......... .......... .......... .......... .......... ..........
11128        .......... .......... ......C... .......... .......... .......... ..........
11368        .......... .......... ......C... .......... .......... .......... ..........
IAI1         .......... .......... ......C... .......... .......... .......... ..........
E110019      .......... .......... ......C... .......... .......... .......... ..........
B7A          .......... .......... .......... .......... .......... .......... ..........
CB9615       .......... .......... ......C... .......... .......... .......... ..........
O157_Sakai   .......... .......... ......C... .......... .......... .......... ..........
IAI39        .......... .......... ......C-.. .......... .......... .......... ..........
UMN026       .......... .......... ......C... .......... .......... .......... ..........
SMS-3-5      .......... .......... ......C-.. .......... .......... .......... ..........
536          .......... .......... ......C... .......... .......... .......... A.........
APEC_O1      .......... .......A.. ......C... .......... .......... .......... ..........
CFT073       .......... .......... ......C... .......... .......... .......... ..........
E2348/69     .......... .......... .G....C... .......... .......... .......... ..........
UTI89        .......... .......A.. ......C... .......... .......... .......... ..........
F11          .......... .......... ......C... .......... .......... .......... A.........
ED1a         .......... .......... ......C... .......... .......... .......... A.........
83972        .......... .......... ......C... .......... .......... .......... ..........
S88          .......... .......A.. ......C... .......... .......... .......... ..........
E._albertii  .......... .....G.T.. ...-..CT.. T......A.. A......... ....C..... -.........

         16311
K-12         ACTTACTCAT CAACTCATTT CATTTGTTAT ATGAATGTTT CTTACCACCC TCACGCGACA AATATCATCA
53638        .......... .......... .......... .......... .......... .......... ..........
ATCC_8739    .......... .......... .......... .......... .......... .......... ..........
HS           .......... .......... .......... .......... .......... .......... ..........
BW2952       .......... .......... .......... .......... .......... .......... ..........
12009        .......... .......... .......... .......... .......... .......... ..........
B171         .......... .......... .......... .......... .......... .......... ..........
E24377A      .......... .......... .......... .......... .......... .......... ..........
SE11         .......... .......... .......... .......... .......... .......... ..........
11128        .......... .......... .......... .......... .......... .......... ..........
11368        .......... .......... .......... .......... .......... .......... ..........
IAI1         .......... .......... .......... .......... .......... .......... ..........
E110019      .......... .......... .......... .......... .......... .......... ..........
B7A          .......... .......... .......... .......... .......... .......... ..........
CB9615       .......... .......... .......... .......... .......... .......... ..........
O157_Sakai   .......... .......... .......... .......... .......... .......... ..........
IAI39        ..A...C... ...T.T.... .......... .......... .......... ........T. ........T.
UMN026       .......... .......... .......... .......... .......... ......A... ..........
SMS-3-5      ..A...C... ...T.T.... .......... .......... .......... ........T. ........T.
536          .......... .......... .......... .......... .....T.... .......... .......C..
APEC_O1      .......... .......... .......... .......... .......... .......... .......C..
CFT073       .......... .......... .......... .......... .C........ .......... .......C..
E2348/69     .......... .......... .......... .......... .......T.. ........T. .......C..
UTI89        .......... .......... .......... .......... .......... .......... .......C..
F11          .......... .......... .......... .......... .....T.... .......... .......C..
ED1a         .......... .......... .......... .......... .......... .......... .......C..
83972        .......... .......... .......... .......... .C........ .......... .......C..
S88          .......... .......... .......... .......... .......... .......... .......C..
E._albertii  .AAA.TCAGG TCGT.T.... .......... C......C.. .AGCGTGT.A .G.GCT..TT ......CAT.
```

```
        16381
K-12         CAGTTAATAT GTCATACAAT TTATGTTGCA ACGCAAACGT TTCCCTATTT TCATAAACCG TATTTTATCG
53638        .......... .......... .......... .......... .......... .......... ..........
ATCC_8739    .......... .......... .......... .......... .......... .......... ..........
HS           .......... .......... .......... .......... .......... .......... ..........
BW2952       .......... .......... .......... .......... .......... .......... ..........
12009        .......... .......... .......... .......... .......... .......... ..........
B171         .......... .......... .......... .......... .......... .......... ..........
E24377A      .......... .......... .......... .......... .......... .......... ..........
SE11         .......... .......... .......... .......... .......... .......... ..........
11128        .......... .......... ....A..... .......... ..A....... .......... ..........
11368        .......... .......... ....A..... .......... .......... .......... ..........
IAI1         .......... .......... ....A..... .......... .......... .......... ..........
E110019      .......... A......... ....A..... .......... .......... .......... ..........
B7A          .......... .......... .......... .......... .......... .......... ..........
CB9615       .......... .......... ....A..... .......... .......... .......... ..........
O157_Sakai   .......... .......... ....A..... .......... .......... .......... ..........
IAI39        .......... .......... ........T. .......... ..T..C.... .........A C.........
UMN026       .......... .......... ....A..... .......... .......... .........A C.........
SMS-3-5      T......... .......... ........T. .......... .......... .........A C.........
536          .......... .......... ........A. .......... .......... .........A ........T.
APEC_O1      .......... .......... ........G. .......... .......... .........A C.........
CFT073       .......... .......... ........A. .......... .......... .........A C.........
E2348/69     .......... .......... ........A. .......... .......... .........A C.........
UTI89        .......... .......... ........G. .......... .......... .........A C.........
F11          .......... .......... ........A. .......... .......... .........A ........T.
ED1a         .......... .......... ........A. .......... .......... .........A ........T.
83972        .......... .......... ........A. .......... .......... .........A C.........
S88          .......... .......... ........G. .......... .......... .........A C.........
E._albertii  T.A.C..... .......... ...CACC... .......... ..A...T.C. .A.A.G.A.. C.........

        16451
K-12         CTATGAAAAA GAAATTATCG CCATGATTAA CTAAAAGTAT TGATTTTTTC AGTTCAACCT ACATATATTG
53638        .......... .......... .......... .......... .......... .......... ..........
ATCC_8739    .......... .......... .......... .......... .......... .......... ..........
HS           .......... .......... .......... .......... .......... .......... ..........
BW2952       .......... .......... .......... .......... .......... .......... ..........
12009        .......... .......... .......... .......... .......... .......... ..........
B171         .......... .......... .......... .......... .......... .......... ..........
E24377A      .......... .......... .......... .......... .......... .......... ..........
SE11         .......... .......... .......... .......... .......... .......... ..........
11128        .......... .......... .......... .......... .......... .......... ..........
11368        .......... .......... .......... .......... .......... .......... ..........
IAI1         .......... .......... .......... .......... .......... .......... ..........
E110019      .......... .......... .......... .......... .......... .......... ..........
B7A          .......... .......... .......... .......... .......... .......... ..........
CB9615       .......... .......... .......... .......... .......... .......... ..........
O157_Sakai   .......... .......... .......... .......... .......... .......... ..........
IAI39        .......... .......... .......... .......... .......... .......... ..........
UMN026       .......... .......... .......... .......... .......... .......... ..........
SMS-3-5      .......... .......... .......... .......... .......... .......... ..........
536          .C........ .......... ........G. .......... .......... .......... ..........
APEC_O1      .C........ .......... .......... .......... .......... .......... ..........
CFT073       .C........ .......... .......... .......... .......... .......... ..........
E2348/69     .C..A..... .......... .......... .......... .......... .......... ..........
UTI89        .C........ .......... .......... .......... .......... .......... ..........
F11          .C........ .......... ........G. .......... .......... .......... ..........
ED1a         .C........ .......... ........G. .......... .......... .......... ..........
83972        .C........ .......... .......... .......... .......... .......... ..........
S88          .C........ .......... .......... .......... .......... .......... ..........
E._albertii  ..T.A..... ...T..C... TA..T..... .......... ..T....... .......... ..........
```

```
         16521
K-12          CGCGCCCCGG AAGAAGTCAG ATGTCGTTTA ATGGGCAAAT ATTGCCCTTA AATTCTCTTT TACTTTTGAT
53638         .......... .......... .......... .......... .......... .......... ..........
ATCC_8739     .......... .......... .......... .......... .......... .......... ..........
HS            .......... .......... .......... .......... .......... .......... ..........
BW2952        .......... .......... .......... .......... .......... .......... ..........
12009         .......... .......... .......... .......... .......... .......... ..........
B171          .......... .......... .......... .......... .......... .......... ..........
E24377A       .......... .......... .......... .......... .......... .......... ..........
SE11          .......... .......... .......... .......... .......... .......... ..........
11128         .......... .......... .......... .......... .......... .......... ..........
11368         .......... .......... .......... .......... .......... .......... ..........
IAI1          .......... .......... .......... .......... .......... .......... ..........
E110019       .......... .......... .......... .......... .......... .......... ..........
B7A           .......... .......... .......... .......... .......... .......... ..........
CB9615        .......... .......... .......... .......... .......... .......... ..........
O157_Sakai    .......... .......... .......... .......... .......... .......... ..........
IAI39         .......... .......... .......... .......... ....A..... .....A.... ..........
UMN026        .......... .......... .......... .......... .......... .......... ..........
SMS-3-5       .......... .......... .......... .......... .......... .......... ..........
536           .........T .......... .......... .....T.... .......... .......... ..........
APEC_O1       .........T .......... .......... .....T.... .......... .......... ..........
CFT073        .........T .......... .......... .....T.... .......... .......... ..........
E2348/69      .......... .......... .......... .......... .......... .......... ..........
UTI89         .........T .......... .......... .....T.... .......... .......... ..........
F11           .........T .......... .......... .....T.... .......... .......... ..........
ED1a          .........T .......... .......... .....T.... .......... .......... ..........
83972         .........T .......... .......... .....T.... .......... .......... ..........
S88           .........T .......... .......... .....T.... .......... .......... ..........
E._albertii   .......... ....G..... .......... T....T.... .......... .......... ..........

         16591
K-12          TTACAGAGTA AAGCGTTGGG ATAATCTATC TTCCAAGTAG ATTATTGTAT TTGAGATCAA GATCACTGAT
53638         .......... .......... .......... .......... .......... ...G...... ..........
ATCC_8739     .......... .......... .......... .......... .......... ...G...... ..........
HS            .......... .......... .......... .......... .......... ...G...... ..........
BW2952        .......... .......... .......... .......... .......... .......... ..........
12009         .......... .......... .......... .......... .......... .......... ..........
B171          .......... .......... .......... .......... .......... .......... ..........
E24377A       .......... .......... .......... .......... .......... .......... ..........
SE11          .......... .......... .......... .......... .......... .......... ..........
11128         .......... .......... .......... .......... .......... ...G...... ..........
11368         .......... .......... .......... .......... .......... ...G...... ..........
IAI1          .......... .......... .......... .......... .......... .......... ..........
E110019       .......... .......... .......... .......... .......... ...G...... ..........
B7A           .......... .......... .......... .......... .......... .......... ..........
CB9615        .......... .......... .......... .......... .......... ...G...... ..........
O157_Sakai    .......... .......... .......... .......... .......... ...G...... ..........
IAI39         .......... .......... .......... .......... .......... ...G...... ..........
UMN026        .......... .......... .......... .......... .......... ...G...... ..........
SMS-3-5       .......... .......... .......... .......... .......... ...G...... ..........
536           .......... ........T. ..T....... .......... .......... ...G...... ..........
APEC_O1       .......... ........T. ..T....... .......... .......... ...G...... ..........
CFT073        .......... .......... ..T....... .......... .......... ...G...... ..........
E2348/69      .......... .......... ..T....... .......... .......... ...G...... ..........
UTI89         .......... ........T. ..T....... .......... .......... ...G...... ..........
F11           .......... ........T. ..T....... .......... .......... ...G...... ..........
ED1a          .......... ........T. ..T....... .......... .......... ...G...... ..........
83972         .......... .......... ..T....... .......... .......... ...G...... ..........
S88           .......... ........T. ..T....... .......... .......... ...G...... ..........
E._albertii   .......... ...A.CC.A. ..T....... ..AT...... .......... .......... ..........
```

```
         16661
K-12          AGATACATAA CTTGTGTGTA TCTTTCCGCC CTCAAATTAT TACGGCGGTA AATGATTAAG CCATCGCCGA
53638         .......... .......... .......... .......... .......... .......... ..........
ATCC_8739     .......... .......... .......... .......... .......... .......... ..........
HS            .......... .......... .......... .......... .......... .......... ..........
BW2952        .......... .......... .......... .......... .......... .......... ..........
12009         .......... .......... .......... .......... .......... .......... ..........
B171          .......... .......... .......... .......... .......... .......... ..........
E24377A       .......... .......... .......... .......... .......... .......... ..........
SE11          .......... .......... .......... .......... .......... .......... ..........
11128         .......... .......... .......... .......... .......... .......... ..........
11368         .......... .......... .......... .......... .......... .......... ..........
IAI1          .......... .......... .......... .......... .......... .......... ..........
E110019       .......... .......... .......... .......... .......... .......... ..........
B7A           .......... .......... .......... .......... .......... .......... ..........
CB9615        .......... .......... .......... .......... .......... .......... ..........
O157_Sakai    .......... .......... .......... .......... .......... .......... ..........
IAI39         .......... .......... .......... .......... .......... .......... ..........
UMN026        .......... .......... .......... .......... .......... .......... ..........
SMS-3-5       .......... .......... .......... .......... .......... .......... ..........
536           .......... .......... .......... .......... .....T..A. .......... ..........
APEC_O1       .......... .......... .......... .......... .....T..A. .......... ..........
CFT073        .......... .......... .......... .......... .....T..A. .......... ..........
E2348/69      .......... .......... .......... .......... .....T..A. .......... ..........
UTI89         .......... .......... .......... .......... .....T..A. .......... ..........
F11           .......... .......... .......... .......... .....T..A. .......... ..........
ED1a          .......... .......... .......... .......... .....T..A. .......... ..........
83972         .......... .......... .......... .......... .....T..A. .......... ..........
S88           .......... .......... .......... .......... .....T..A. .......... ..........
E._albertii   .......... G......... .......... .......... ........A. ......A... .........G

         16731
K-12          TAGACAGATT TCATTTTTAC GGTCAGGCAC CTTCCCGGGC TGAACTGGCT AAAAGCTGAA TTATTTGCAT
53638         .......... .......... .......... .......... .......... .......... ..........
ATCC_8739     .......... .......... .......... .......... .......... .......... ..........
HS            .......... .......... .......... .......... .......... .......... ..........
BW2952        .......... .......... .......... .......... .......... .......... ..........
12009         .......... .......... .......... .......... .......... .......... ..........
B171          .......... .......... .......... .......... .......... .......... ..........
E24377A       .......... .......... .......... .......... .......... .......... ..........
SE11          .......... .......... .......... .......... .......... .......... ..........
11128         .......... .......... .......... .......... .......... .......... ..........
11368         .......... .......... .......... .......... .......... .......... ..........
IAI1          .......... .......... .......... .......... .......... .......... ..........
E110019       .......... .......... .......... .......... .......... .......... ..........
B7A           .......... .......... .......... .......... .......... .......... ..........
CB9615        .......... .......... .......... .......... .......... .......... ..........
O157_Sakai    .......... .......... .......... .......... .......... .......... ..........
IAI39         .......... .......... .......... .......... .......... .......... ..........
UMN026        .......... .......... .......... .......... .......... .......... ..........
SMS-3-5       .......... .......... .......... .......... .......... .......... ..........
536           .......... .......... .......... .......... .......... .......... ..........
APEC_O1       .......... .......... .......... .......... .......... .......... ..........
CFT073        .......... .......... .......... .......... .......... .......... ..........
E2348/69      .......... .......... .......... .......... .......... .......... ..........
UTI89         .......... .......... .......... .......... .......... .......... ..........
F11           .......... .......... .......... .......... .......... .......... ..........
ED1a          .......... .......... .......... .......... .......... .......... ..........
83972         .......... .......... .......... .......... .......... .......... ..........
S88           .......... .......... .......... .......... .......... .......... ..........
E._albertii   .......... .......... ..C...A... .......... ...G...... .......... ..T.......
```

```
            16801
K-12         TCCTCCAGGA GAAATAGATG GAAACGACTC AAACCAGCAC GATTGCGTCG AAAGACTCTC GTAGTGCCTG
53638        .......... .......... .......... .......... .......... .......... ..........
ATCC_8739    .......... .......... .......... .......... .......... .......... ..........
HS           .......... .......... .......... .......... .......... .......... ..........
BW2952       .......... .......... .......... .......... .......... .......... ..........
12009        .......... .......... .......... .......... .......... .......... ..........
B171         .......... .......... .......... .......... .......... .......... ..........
E24377A      .......... .......... .......... .......... .......... .......... ..........
SE11         .......... .......... .......... .......... .......... .......... ..........
11128        .......... .......... .......... .......... .......... .......... ..........
11368        .......... .......... .......... .......... .......... .......... ..........
IAI1         .......... .......... .......... .......... .......... .......... ..........
E110019      .......... .......... .......... .......... .......... .......... ..........
B7A          .......... .......... .......... .......... .......... .......... ..........
CB9615       .......... .......... .......... .......... .......... .......... ..........
O157_Sakai   .......... .......... .......... .......... .......... .......... ..........
IAI39        .......... .......... .....A.... .......... .......... .......... ..........
UMN026       .......... .......... .......... .......... .......... .......... ..........
SMS-3-5      .......... .......... .......... .......... .......... .......... ..........
536          .......... .......... .......... .......... .......... .......... ..........
APEC_O1      .......... .......... .......... .......... .......... .......... ..........
CFT073       .......... .......... .......... .......... .......... .......... ..........
E2348/69     .......... .......... .......... .......... .......... .......... ..........
UTI89        .......... .......... .......... .......... .......... .......... ..........
F11          .......... .......... .......... .......... .......... .......... ..........
ED1a         .......... .......... .......... .......... .......... .......... ..........
83972        .......... .......... .......... .......... .......... .......... ..........
S88          .......... .......... .......... .......... .......... .......... ..........
E._albertii  .......... .......... .......... .......... .......... .......... .C........

            16871
K-12         GCGCAAGACA GACACCATGT GGATGCTGGG CCTTTACGGC ACGGCAATCG GCGCGGGCGT GCTGTTCCTG
53638        .......... .......... .......... .......... .......... .......... ..........
ATCC_8739    .......... .......... .......... .......... .......... .......... ..........
HS           .......... .......... .......... .......... .......... .......... ..........
BW2952       .......... .......... .......... .......... .......... .......... ..........
12009        .......... .......... .......... .......... .......... .......... ..........
B171         .......... .......... .......... .......... .......... .......... ..........
E24377A      .......... .......... .......... .......... .......... .......... ..........
SE11         .......... .......... .......... .......... .......... .......... ..........
11128        .......... .......... .......... .......... .......... .......... ..........
11368        .......... .......... .......... .......... .......... .......... ..........
IAI1         .......... .......... .......... .......... .......... .......... ..........
E110019      .......... .......... .......... .......... .......... .......... ..........
B7A          .......... .......... .......... .......... .......... .......T.. ..........
CB9615       .......... .......... .......... .......... .......... .......... ..........
O157_Sakai   .......... .......... .......... .......... .......... .......... ..........
IAI39        .......... .......... .......... .......... .......... .......... ..........
UMN026       .......... .......... .......... .......... .......... .......... ..........
SMS-3-5      .......... .......... .......... .......... .......... .......... ..........
536          .......... .......... .......... .......... .......... .......... ..........
APEC_O1      .......... .......... .......... .......... .......... .......... ..........
CFT073       .......... .......... .......... .......... .......... .......... ..........
E2348/69     .......... .......... .......... .......... .......... .......... ..........
UTI89        .......... .......... .......... .......... .......... .......... ..........
F11          .......... .......... .......... .......... .......... .......... ..........
ED1a         .......... .......... .......... .......... .......... .......... ..........
83972        .......... .......... .......... .......... .......... .......... ..........
S88          .......... .......... .......... .......... .......... .......... ..........
E._albertii  .......... ..T....... .......... ...G...... .....T.... .......T.. ..........
```

```
         16941
K-12         CCAATCAACG CCGGTGTTGG CGGTATGATC CCGCTGATCA TCATGGCTAT CCTTGCGTTC CCGATGACGT
53638        .......... .......... .......... .......... .......... .......... ..........
ATCC_8739    .......... .......... .......... .......... .......... .......... ..........
HS           .......... .......... .......... .......... .......... .......... ..........
BW2952       .......... .......... .......... .......... .......... .......... ..........
12009        .......... .......... .......... .......... .......... .......... ..........
B171         .......... .......... .......... .......... .......... .......... ..........
E24377A      .......... .......... .......... .......... .......... .......... ..........
SE11         .......... .......... .......... .......... .......... .......... ..........
11128        .......... .......... .......... .......... .......... .......... ..........
11368        .......... .......... .......... .......... .......... .......... ..........
IAI1         .......... .......... .......... .......... .......... .......... ..........
E110019      .......... .......... .......... .......... .......... .......... ..........
B7A          .......... .......... .......... .......... .......... .......... ..........
CB9615       .......... .......... .......... .......... .......... .......... ..........
O157_Sakai   .......... .......... .......... .......... .......... .......... ..........
IAI39        .......... .......... .......... .......... .......... .......... ........A.
UMN026       .......... .......... .......... .......... .......... .......... ........A.
SMS-3-5      .......... .......... .......... .......... .......... .......... ........A.
536          .......... .T........ .......... .......... .......... .......... ..........
APEC_O1      .......... .T........ .......... .......... .......... .......... ..........
CFT073       .......... .T........ .......... .......... .......... .......... ..........
E2348/69     .......... .T........ .......... .......... .......... .......... ..........
UTI89        .......... .T........ .......... .......... .......... .......... ..........
F11          .......... .T........ .......... .......... .......... .......... ..........
ED1a         .......... .T........ .......... .......... .......... .......... ..........
83972        .......... .T........ .......... .......... .......... .......... ..........
S88          .......... .T........ .......... .......... .......... .......... ..........
E._albertii  ..G....... .......C.. .......... ..A..A.... .T........ ...C...... ..........

         17011
K-12         TTTTTGCTCA CCGCGGCCTG ACTCGCTTCG TACTGTCTGG TAAAAACCCG GGCGAAGACA TCACCGAGGT
53638        .......... .......... .......... .......... .......... .......... ..........
ATCC_8739    .......... .......... .......... .......... .......... .......... ..........
HS           .......... .......... .......... .......... .......... .......... ..........
BW2952       .......... .......... .......... .......... .......... .......... ..........
12009        .......... .......... .......... .......... .......... .......... ..........
B171         .......... .......... .......... .......... .......... .......... ..........
E24377A      .......... .......... .......... .......... .......... .......... ..........
SE11         .......... .......... .......... .......... .......... .......... ..........
11128        .......... .......... .......... .......... .......... .......... ..........
11368        .......... .......... .......... .......... .......... .......... ..........
IAI1         .......... .......... .......... .......... .......... .......... ..........
E110019      .......... .......... .......... .......... .......... .......... ..........
B7A          .......... .......... .......... .......... .......... .......... ..........
CB9615       .......... .......... .......... .......... .......... .......... .T........
O157_Sakai   .......... .......... .......... .......... .......... .......... .T........
IAI39        .......... .......... .......... .......... .........A .......... ..........
UMN026       .......... .......... .......... .......... .........A .......... ..........
SMS-3-5      .......... .......... .......... .......... .........A .......... ..........
536          .......... .......... .......... .......... .......... .......... ..........
APEC_O1      .......... .......... .......... .......... .......... .......... ..........
CFT073       .......... .......... .......... .......... .......... .......... ..........
E2348/69     .......... .......... .......... .......... .......... .......... ..........
UTI89        .......... .......... .......... .G........ .......... .......... ..........
F11          .......... .......... .......... .......... .......... .......... ..........
ED1a         .......... .......... .......... .......... .......... .......... ..........
83972        .......... .......... .......... .......... .......... .......... ..........
S88          .......... .......... .......... .......... .......... .......... ..........
E._albertii  ....C..... .......... .......... .G.....C.. ......T... ..T....... ..........
```

```
             17081
K-12         TGTAGAAGAA CACTTTGGTA TTGGCGCAGG TAAACTGATT ACCCTGCTCT ACTTCTTCGC TATCTACCCG
53638        .......... .......... .......... .......... .......... .T........ ..........
ATCC_8739    .......... .......... .......... .......... .......... .T........ ..........
HS           .......... .......... .......... .......... ...T...... .T........ ..........
BW2952       .......... .......... .......... .......... .......... .......... ..........
12009        .......... .......... .......... .......... .......... .......... ..........
B171         .......... .......... .......... .......... .......... .......... ..........
E24377A      .......... .......... .......... .......... .......... .......... ..........
SE11         .......... .......... .......... .......... .......... .......... ..........
11128        .......... .......... .......... .......... .......... .......... ..........
11368        .......... .......... .......... .......... .......... .......... ..........
IAI1         .......... .......... .......... .......... .......... .......... ..........
E110019      .......... .......... .......... .......... .......... .......... ..........
B7A          .......... .......... .......... .......... .......... .......... ..........
CB9615       .......... .......... .......... .......... .......... .......... ..........
O157_Sakai   .......... .......... .......... .......... .......... .......... ..........
IAI39        .......... .......... .......... .......... .......... .......... ..........
UMN026       .......... .......... .......... .......... .......... .......... ..........
SMS-3-5      .......... .......... .......... .......... .......... .......... ..........
536          .......... .......... .......... .......... ..T....... .......... ..........
APEC_O1      .......... .......... .......... .......... ..T....... .......... ..........
CFT073       .......... .......... .......... .......... .......... .......... ..........
E2348/69     .......... .......... .......... .......... .......... .......... ..........
UTI89        .......... .......... .......... .......... ..T....... .......... ..........
F11          .......... .......... .......... .......... ..T....... .......... ..........
ED1a         .......... .......... .......... .......... ..T....... .......... ..........
83972        .......... .......... .......... .......... .......... .......... ..........
S88          .......... .......... .......... .......... ..T....... .......... ..........
E._albertii  A..G...... .......... .......... .......... .......... .T........ .........A

             17151
K-12         ATCCTGCTGG TTTATAGCGT GGCAATCACC AATACCGTTG AAAGCTTCAT GTCTCACCAG CTGGGTATGA
53638        .......... .......... A......... .....A.... .......... .......... ..........
ATCC_8739    .......... .......... A......... .....A.... .......... .......... ..........
HS           .......... .......... A......... .....A.... .......... .......... ..........
BW2952       .......... .......... .......... .......... .......... .......... ..........
12009        .......... .......... .......... .....A.... .......... .......... ..........
B171         .......... .......... .......... .....A.... .......... .......... ..........
E24377A      .......... .......... .......... .....A.... .......... .......... ..........
SE11         .......... .......... .......... .....A.... .......... .......... ..........
11128        .......... .......... .......... .....A.... .......... .......... ..........
11368        .......... .......... .......... .....A.... .......... .......... ..........
IAI1         .......... .......... .......... .......... .......... .......... ..........
E110019      .......... .......... .......... .....A.... .......... .......... ..........
B7A          .......... .......... .......... .....A.... .......... .......... ..........
CB9615       .......... .......... .......... .....A.... .......... .......... ..........
O157_Sakai   .......... .......... .......... .....A.... .......... .......... ..........
IAI39        .......... .......... .......... .....A.... .......... .......... ..........
UMN026       .......... .......... .......... .....A.... .......... .......... ..........
SMS-3-5      .......... .......... .......... .....A.... .......... .......... ..........
536          .......... .......... .......... .....A.... .......... .......... ..........
APEC_O1      .......... .......... .......... .....A.... .......... .......... ..........
CFT073       .......... .......... .......... .....A.... .......... .......... ..........
E2348/69     .......... .......... .......... .....A.... .......... .......... ..........
UTI89        .......... .......... .......... .....A.... .......... .......... ..........
F11          .......... .......... .......... .....A.... .......... .......... ..........
ED1a         .......... .......... .......... .....A.... .......... .......... ..........
83972        .......... .......... .......... .....A.... .......... .......... ..........
S88          .......... .......... .......... .....A.... .......... .......... ..........
E._albertii  .......... .A..C..... .......... ..C..G..A. .......... ...C...... T.........
```

```
          17221
K-12         CGCCACCGCC GCGTGCGATT CTGTCGCTGA TCCTGATCGT GGGTATGATG ACCATCGTTC GCTTCGGTGA
53638        .A........ .......... .......... .......... .......... .......... ..........
ATCC_8739    .A........ .......... .......... .......... .......... .......... ..........
HS           .A........ .......... .......... .......... .......... .......... ..........
BW2952       .......... .......... .......... .......... .......... .......... ..........
12009        .......... .......... .......... .......... .......... .......... ....T.....
B171         .......... .......... .......... .......... .......... .......... ....T.....
E24377A      .......... .......... .......... .......... .......... .......... ....T.....
SE11         .......... .......... .......... .......... .......... .......... ....T.....
11128        .......... .......... .......... .......... .......... .......... ....T.....
11368        .......... .......... .......... .......... .......... .......... ....T.....
IAI1         .......... .......... .......... .......... .......... .......... ..........
E110019      .......... .......... .......... .......... .......... .......... ..........
B7A          .......... .......... .......... .......... .......... .......... ....T.....
CB9615       .A........ .......... .......... .......... .......... .......... ....T.....
O157_Sakai   .A........ .......... .......... .......... .......... .......... ....T.....
IAI39        .A........ .......... .......... .......... .......... .......... ..........
UMN026       .A........ .......... .......... .......... .......... .......... ..........
SMS-3-5      .A........ .......... .......... .......... .......... .......... ..........
536          .......... .......... .......... .......... .......... .......... ..........
APEC_O1      .......... .......... .......... .......... .......... .......... ..........
CFT073       .......... .......... .......... .......... .......... .......... ..........
E2348/69     .......... .......... .......... .......... .......... .......... ..........
UTI89        .......... .......... .......... .......... .......... .......... ..........
F11          .......... .......... .......... .......... .......... .......... ..........
ED1a         .......... .......... .......... .......... .......... .......... ..........
83972        .......... .......... .......... .......... .......... .......... ..........
S88          .......... .......... .......... .......... .......... .......... ..........
E._albertii  .A..G..A.. .......... ....C..... .T........ C......... .......... ..........

          17291
K-12         GCAGATGATC GTTAAAGCGA TGAGTATTCT GGTATTCCCG TTTGTTGGCG TACTGATGCT GCTGGCTCTG
53638        .......... .......... .......... .......... .......... .......... ..........
ATCC_8739    .......... .......... .......... .......... .......... .......... ..........
HS           .......... .......... .......... .......... .......... .......... ..........
BW2952       .......... .......... .......... .......... .......... .......... ..........
12009        .......... .......... .......... .......... .......... .......... ..........
B171         .......... .......... .......... .......... .......... .......... ..........
E24377A      .......... .......... .......... .......... .......... .......... ..........
SE11         .......... .......... .......... .......... .......... .......... ..........
11128        .......... .......... .......... .......... .......... .......... ..........
11368        .......... .......... .......... .......... .......... .......... ..........
IAI1         .......... .......... .......... .......... .......... .......... ..........
E110019      .......... .......... .......... .......... .......... .......... ..........
B7A          .......... .......... .......... .......... .......... .......... ..........
CB9615       A......... .......... .......... .......... .......... .......... ..........
O157_Sakai   A......... .......... .......... .......... .......... .......... ..........
IAI39        ...A...... .......... .......... .......... .......... .......... ..........
UMN026       .......... .......... .......... .......... .......... .......... ..........
SMS-3-5      .......... .......... ....C..... .......... .......... .......... ..........
536          .......... .......... .......... .......... .......... .......... ..........
APEC_O1      .......... .......... .......... .......... .......... .......... ..........
CFT073       ...A...... ....G..... .......... .......... .......... .......... ..........
E2348/69     ...A...... ....G..... .......... .......... .......... .......... ..........
UTI89        .......... .......... .......... .......... .......... .......... ..........
F11          .......... .......... .......... .......... .......... .......... ..........
ED1a         .......... .......... .......... .......... .......... .......... ..........
83972        ...A...... ....G..... .......... .......... .......... .......... ..........
S88          .......... .......... .......... .......... .......... .......... ..........
E._albertii  .......... .......... ....C..C.. .......... ..C....CA. .T........ ..........
```

```
         17361
K-12          TACCTGATCC CGCAGTGGAA CGGCGCTGCA CTGGAAACGC TGTCTCTGGA CACTGCATCT GCAACCGGAA
53638         .......... .......... ......A... .......... .......... .......... ........G.
ATCC_8739     .......... .......... ......A... .......... .......... .......... ........G.
HS            .......... .......... ......A... .......... .......... .......... ........G.
BW2952        .......... .......... .......... .......... .......... .......... ..........
12009         .......... .......... ......A... .......... .......... .......... ........G.
B171          .......... .......... ......A... .......... .......... .......... ........G.
E24377A       .......... .......... ......A... .......... .......... .......... ..........
SE11          .......... .......... ......A... .......... .......... .......... ..........
11128         .......... .......... ......A... .......... .......... .......... ..........
11368         .......... .......... ......A... .......... .......... .......... ..........
IAI1          .....C.... .......... ......A... .......... .......... .......... ..........
E110019       .......... .......... ......A... .......... .......... .......... ..........
B7A           .......... .......... ......A... .......... .......... .......... ..........
CB9615        .......... .......... ......A... .......... .......... .......... ..........
O157_Sakai    .......... .......... ......A... .......... .......... .......... ..........
IAI39         .......... .......... ......A... .......... .......... .......... ..........
UMN026        .......... .......... ......A... .......... .......... .........G ..........
SMS-3-5       .......... .......... ......A... ........A. .......... .......... ..........
536           .......... .......... ......A... .......... .......... .......... ..........
APEC_O1       .......... .......... ......A... .......... .......... .......... ..........
CFT073        ...T...... .......... ......A... .......... .......... .......... ..........
E2348/69      ...T...... .......... ......A... .......... .......... .......... ..........
UTI89         .......... .......... ......A... .......... .......... .......... ..........
F11           .......... .......... ......A... .......... .......... .......... ..........
ED1a          .......... .......... ......A... .......... .......... .......... ..........
83972         ...T...... .......... ......A... .......... .......... .......... ..........
S88           .......... .......... ......A... .......... .......... .......... ..........
E._albertii   .......... .......... T.....G..G .........T .......... T..C..GA.. T.T..T..T.

         17431
K-12          ACGGTCTGTG GATGACCCTG TGGCTGGCAA TTCCGGTAAT GGTGTTCTCG TTCAACCACT CTCCGATCAT
53638         .......... .......... .......... .......... .......... .......... ..........
ATCC_8739     .......... .......... ....A..... .......... .......... .......... ..........
HS            .......... .......... .......... .......... .......... .......... ..........
BW2952        .......... .......... .......... .......... .......... .......... ..........
12009         .......... .......... .......... .......... .......... .......... ..........
B171          .......... .......... .......... .......... .......... .......... ..........
E24377A       .......... .......... .......... .......... .......... .......... ..........
SE11          .......... .......... .......... .......... .......... .......... ..........
11128         .......... .......... .......... .......... .......... .......... ..........
11368         .......... .......... .......... .......... .......... .......... ..........
IAI1          .......... .......... .......... .......... .......... .......... ..........
E110019       .......... .......... .......... .......... .........T .......... ..........
B7A           .......... .......... .......... .......... .......... .......... ..........
CB9615        .......... .......... .......... .......... .......... .......... ..........
O157_Sakai    .......... .......... .......... .......... .......... .......... ..........
IAI39         .......... .......... .......... .......... .......... .......... ..........
UMN026        .......... .......... .......... ....A..... .......... .......... ..........
SMS-3-5       .......... .......... .......... .......... .......... .......... ..........
536           .......... ......A... .......... .......... .......... .......... ..........
APEC_O1       .......... ......A... .......... .......... .......... .......... ..........
CFT073        .......... ......A... .......... .......... .......... .......... ..........
E2348/69      .......... .......... .......... .......... .......... .......... ..........
UTI89         .......... ......A... .......... .......... .......... .......... ..........
F11           .......... ......A... .......... .......... .......... .......... ..........
ED1a          .......... ......A... .......... .......... .......... .......... ..........
83972         .......... ......A... .......... .......... .......... .......... ..........
S88           .......... ......A... .......... .......... .......... .......... ..........
E._albertii   .......... .......... ........G. ....A..... .......... .......... ..........
```

```
         17501
K-12            CTCTTCTTTC GCCGTTGCGA AGCGTGAAGA GTACGGCGAT ATGGCAGAAC AGAAATGCTC GAAGATCCTG
53638           .......... .......... .......... .......... .......... .......... .........T
ATCC_8739       .......... .......... .......... .......... .......... .......... .........T
HS              .......... .......... .......... .......... .......... .......... .........T
```
[truncated: 1,113,059 more chars]
